# Supplementary figures and images for: Plasma biomarkers of the amyloid pathway are associated with geographic atrophy secondary to age-related macular degeneration
Source: PLoS One. 2020 Aug 7;15(8):e0236283. doi: 10.1371/journal.pone.0236283 (PMC7413518; doi:10.1371/journal.pone.0236283)

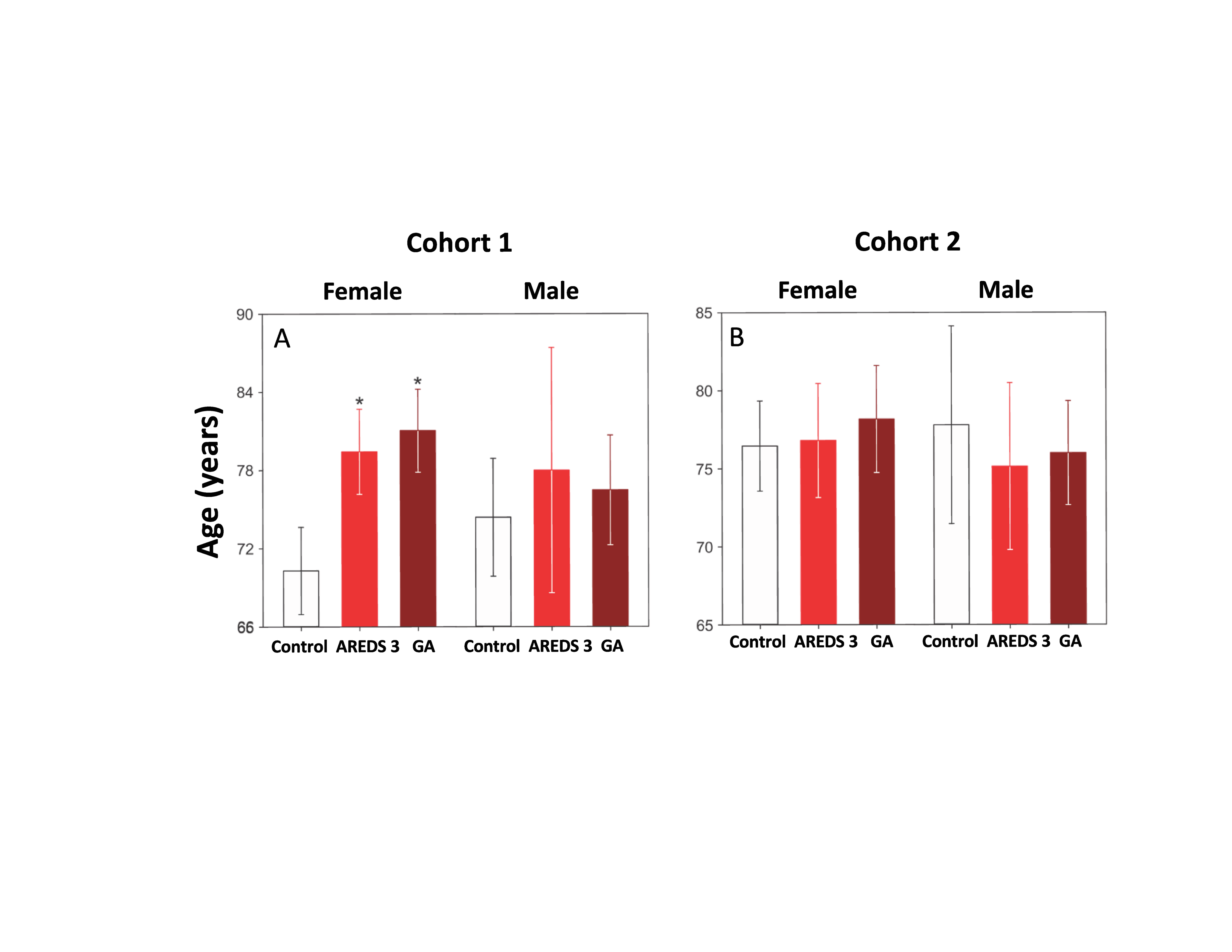

Supplement: S1 Fig — Data are presented as the least squares means ± 95% confidence limits. Analysis was conducted by a two-way analysis of variance followed by the Tukey test on the raw data. Asterisks (*) indicate statistically significant differences (P < 0.05) versus women control group in Figure A. (Cohort 1). The Y axes are different in each graph. (TIF) [file pone.0236283.s001.tif]

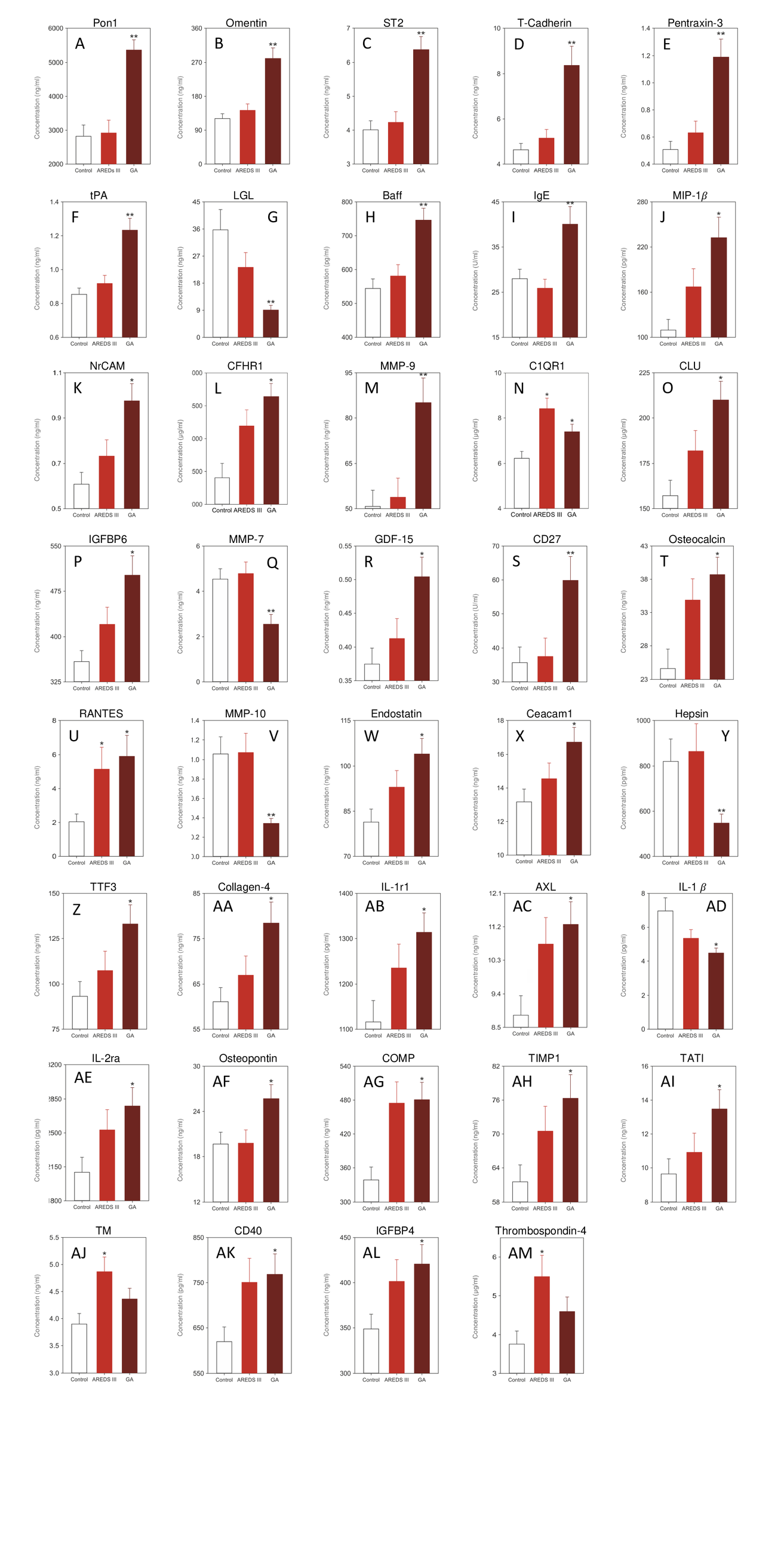

Supplement: S2 Fig — Asterisk (*), statistically significant differences (P < 0.05) versus the control group. Double asterisk (**), statistically significant differences (P<0.05) versus control and AREDS III groups. The Y axes in these graphs have different scales. Please refer to Fig 3 in the manuscript for Aβ(1–40), Aβ(1–42) and sAPP. Key: Control, non-AMD control; AREDS 3, intermediate dry AMD; GA, geographic atrophy. (A) Pon1. Data represent the fitted means ± SE. Analysis of covariance using age as the covariate followed by the Tukey test on the raw data. (B) Omentin. Data represent the fitted geometric means ± SE estimated by the Taylor series expansion. Analysis of covariance using age as the covariate followed by the Tukey test on the base 10 logarithm of the data. (C) ST2. Data represent the fitted geometric means ± SE estimated by the Taylor series expansion. Analysis of covariance using age as the covariate followed by the Tukey test on the base 10 logarithm of the data. (D) T-Cadherin. Data represent the fitted means ± SE estimated by the Taylor series expansion. Analysis of covariance using age as the covariate followed by the Tukey test on the inverse of the data. (E) Pentraxin-3. Data represent the fitted geometric means ± SE estimated by the Taylor series expansion. Analysis of covariance using age as the covariate followed by the Tukey test on the base 10 logarithm of the data. (F) tPA. Data represent the fitted means ± SE estimated by the Taylor series expansion. Analysis of covariance using age as the covariate followed by the Tukey test on the inverse of the data. (G) LGL. Data represent the fitted geometric means ± SE estimated by the Taylor series expansion. Analysis of covariance using age as the covariate followed by the Tukey test on the base 10 logarithm of the data. (H) Baff. Data represent the fitted geometric means ± SE estimated by the Taylor series expansion. Analysis of covariance using age as the covariate followed by the Tukey test on the base 10 logari [file pone.0236283.s002.tif]
