# Supplementary material for: Plasma biomarkers of the amyloid pathway are associated with geographic atrophy secondary to age-related macular degeneration
Source: PLoS One. 2020 Aug 7;15(8):e0236283. doi: 10.1371/journal.pone.0236283 (PMC7413518; doi:10.1371/journal.pone.0236283)
Supplement: S1 Table — (DOC) [file pone.0236283.s003.doc]

S1 Table: Cohort 2: Sources and glossary of ELISA and multiplex assays used for measurement of plasma samples.

Analyte	ELISA Source	Catalogue #	
tPA	eBioscience	BMS258/2	
Ab (1-40)	IBL International	RE59781	
Ab (1-42)	IBL International	JP27719	
AXL	R&D Systems	DY154	
Baff	R&D Systems	DY124-05	
CD27	R&D Systems	DY382-05	
CD40	R&D Systems	DCCD40	
C1QR1	Ray Biotech	ELH-C1qR1-5	
Ceacam1	R&D Systems	DY2244	
CFHR1	R&D systems	DY4779	
COMP	R&D Systems	DY3134	
GDF-15	R&D Systems	DY957	
Endostatin	R&D Systems	DY1098	
Hepsin	Enzo Life Sciences	ADI-900-220-0001	
IL-2ra	R&D Systems	DY223	
LGL	Antibodies-online.com	MBS2885377	
NrCAM	R&D systems	DY2034	
Osteocalcin	Ray Biotech	ELH-Osteocalcin-1	
sAPP	R&D Systems	DY850	
T-cadherin	RayBiotech	ELH-CDH13-5	
TATI (SPINK1)	R&D Systems	DY7496-05	
TFF3	R&D Systems	DY4407	
TM  	R&D Systems	DY3947	
TSP-4	Antibodies-online.com	ABIN824785	
			
Analyte	Multiplexing Source	Catalogue #	
CLU	BIO-RAD	171ATR1CK	
Collagen-4	R&D Systems	LSAHM-06	
IgE	BIO-RAD	171A3102M	
IGFBP4	BIO-RAD	171AGR1CK	
IGFBP6	BIO-RAD	171AGR1CK	
IL-1b	BIO-RAD	M50-0KCAF0Y	
IL-1r1	R&D Systems	LSAHM-06	
MIP-1b	BIO-RAD	M50-0KCAF0Y	
MMP-9	BIO-RAD	171-BM004M	
MMP-9	BIO-RAD	171-BM004M	
MMP-10	BIO-RAD	171-BM004M	
Omentin	BIO-RAD	171AMR2CK	
Osteopontin set	BIO-RAD	171-BL032M	
Pon1	BIO-RAD	171AMR2CK	
PTX-3	BIO-RAD	171AMR2CK	
RANTES	BIO-RAD	M50-0KCAF0Y	
ST2	R&D Systems	LSAHM-06	
TIMP1	BIO-RAD	171AM002M	


____________________________________________________________________________________
Glossary
 Ab, Amyloid-beta
(s)APP, serum amyloid precursor protein 
AXL, AXL receptor tyrosine kinase
Baff, member of TNF ligand family 
Ceacam1, carcinoembryonic antigen related adhesion molecule 1 
CFHR1, complement factor H related 1 
CLU, clusterin 
COMP, cartilage oligomeric matric protein
C1QR1, complement 1 q receptor 1 
IgE, immunoglobulin-g E
IGFBP4, insulin like growth factor binding protein 4 
IGFBP6, insulin like growth factor binding protein 6
LGL, lactogluthathione lyase/ glyoxalase 1
IL-1b, interleukin-1beta 
IL1r1, interleukin-1 receptor 1 
IL2ra, interleukin-2 receptor subunit alpha 
MIP-1b, macrophage inflammatory protein-1 beta (CCL4) 
MMP-7, matrix metalloproteinase-7
MMP-9, matrix metalloproteinase-9 
NrCAM, neuronal adhesion molecule
Pon1, paroxonase-1 
PTX-3, pentraxin-3
RANTES, regulated on activation, normal T cell expressed and secreted 
ST2, suppression of tumorigenicity
TATI, tumor-associated trypsinogen inhibitor 
TIMP1 tissue inhibit of metalloproteinase-1
tPA, tissue plasminogen activator 
TFF3, trefoil factor 3 
TM, thrombomodulin 
TSP-4, thrombospondin-4
