# Supplementary material for: Plasma biomarkers of the amyloid pathway are associated with geographic atrophy secondary to age-related macular degeneration
Source: PLoS One. 2020 Aug 7;15(8):e0236283. doi: 10.1371/journal.pone.0236283 (PMC7413518; doi:10.1371/journal.pone.0236283)
Supplement: S2 Table — YELLOW. Original 56 statistically significant analytes (refer to Fig 1H in the manuscript). BLUE. Aβ(1–42) was not statistically significant. GREEN. sAPP was statistically significant. Key: Control, non-AMD control; AREDS 3, intermediate dry AMD; GA, geographic atrophy. (DOCX) [file pone.0236283.s004.docx]

**S2 Table: Cohort 1: Summary Statistics (N, mean, standard error of the mean [SE], 25 and 75% percentiles**

**[25% and 75%] and interquartile range [IQR]) of Analyte Concentrations by group.**

| **ANALYTE** | **GROUP** | **N** | **MEAN** | **SE** | **MEDIAN** | **25%** | **75%** | **IQR** |
| --- | --- | --- | --- | --- | --- | --- | --- | --- |
| 6Ckine (pg/ml) | control | 33 | 351.55 | 14.2 | 338 | 309 | 372 | 63 |
|  | AREDS 3 | 24 | 327.88 | 14.61 | 336 | 276 | 375.25 | 98.75 |
|  | GA | 37 | 370.43 | 14.1 | 379 | 290 | 422 | 132 |
| Adiponectin (ug/ml) | control | 33 | 4.23 | 0.36 | 3.9 | 2.4 | 5.9 | 3.5 |
|  | AREDS 3 | 24 | 6.88 | 1.37 | 5.05 | 3.4 | 6.43 | 3.03 |
|  | GA | 37 | 5.49 | 0.6 | 4.3 | 3.5 | 6.8 | 3.3 |
| Adrenomedulin | control | 33 | 2.4 | 0.13 | 2.4 | 2 | 2.8 | 0.8 |
| (ADM; ng/ml) | AREDS 3 | 24 | 2.9 | 0.2 | 2.65 | 2.27 | 3.4 | 1.12 |
|  | GA | 37 | 3.13 | 0.23 | 2.9 | 2.6 | 3.4 | 0.8 |
| Agouti-related | control | 33 | 167 | 0 | 167 | 167 | 167 | 0 |
| Protein (AGRP; | AREDS 3 | 24 | 167.58 | 0.58 | 167 | 167 | 167 | 0 |
| pg/ml) | GA | 37 | 170.24 | 2.88 | 167 | 167 | 167 | 0 |
| Aldose Reductase | control | 33 | 8.56 | 0.46 | 8.4 | 6.9 | 10 | 3.1 |
| (Aldose.red; ng/ml) | AREDS 3 | 24 | 10.56 | 0.78 | 9.55 | 7.4 | 12.25 | 4.85 |
|  | GA | 37 | 10.51 | 0.84 | 10 | 7.4 | 12 | 4.6 |
| Alpha-1-acid | control | 33 | 549.97 | 21.33 | 540 | 470 | 605 | 135 |
| Glycoprotein-1 | AREDS 3 | 24 | 514.71 | 29.07 | 505.5 | 419.25 | 562.5 | 143.25 |
| (AGP1; ug/ml) | GA | 37 | 588.73 | 33.56 | 546 | 465 | 646 | 181 |
| Alpha-1-anti- | control | 33 | 1.00 | 0.07 | 0.89 | 0.76 | 1.11 | 0.35 |
| chymotrypsin | AREDS 3 | 24 | 1.14 | 0.07 | 1.11 | 0.98 | 1.21 | 0.23 |
| (AACT; mg/ml) | GA | 36 | 1.10 | 0.10 | 0.95 | 0.81 | 1.03 | 0.23 |
| Alpha-1-anti | control | 33 | 1.57 | 0.07 | 1.6 | 1.4 | 1.7 | 0.3 |
| trypsin | AREDS 3 | 24 | 1.64 | 0.07 | 1.6 | 1.4 | 1.7 | 0.3 |
| (AAT; mg/ml) | GA | 37 | 1.83 | 0.08 | 1.8 | 1.5 | 2.1 | 0.6 |
| Alpha-1-micro- | control | 33 | 15.91 | 0.67 | 15 | 13 | 18 | 5 |
| globulin | AREDS 3 | 24 | 17.58 | 1.45 | 15.5 | 12.75 | 18.75 | 6 |
| (A1micro; ug/ml) | GA | 37 | 16.65 | 0.79 | 16 | 14 | 18 | 4 |
| Alpha-2-macro- | control | 33 | 1.4 | 0.07 | 1.3 | 1.1 | 1.6 | 0.5 |
| globulin | AREDS 3 | 24 | 1.54 | 0.07 | 1.55 | 1.3 | 1.7 | 0.4 |
| (A2macro; mg/ml) | GA | 37 | 1.74 | 0.11 | 1.7 | 1.3 | 2.1 | 0.8 |
| Alpha-fetoprotein | control | 33 | 1.17 | 0.11 | 1.2 | 0.54 | 1.5 | 0.96 |
| (AFP; ng/ml) | AREDS 3 | 24 | 1.63 | 0.27 | 1.1 | 0.79 | 1.92 | 1.13 |
|  | GA | 37 | 1.39 | 0.18 | 1 | 0.79 | 1.7 | 0.91 |
| Amphiregulin | control | 33 | 393 | 0 | 393 | 393 | 393 | 0 |
| (AR; pg/ml) | AREDS 3 | 24 | 393 | 0 | 393 | 393 | 393 | 0 |
|  | GA | 37 | 393 | 0 | 393 | 393 | 393 | 0 |
| Angiogenin (ng/ml) | control | 33 | 314.12 | 19.08 | 292 | 251 | 361 | 110 |
|  | AREDS 3 | 24 | 330.17 | 23.88 | 298 | 244 | 374.75 | 130.75 |
|  | GA | 37 | 356.27 | 22.06 | 332 | 279 | 410 | 131 |
| Angiopoietin-1 | control | 33 | 10.37 | 2.32 | 6.6 | 5.4 | 8.9 | 3.5 |
| (ANG-1; ng/ml) | AREDS 3 | 24 | 13.05 | 2.51 | 8.3 | 6.88 | 11.75 | 4.87 |
|  | GA | 37 | 12.29 | 2.11 | 7.7 | 5.7 | 12 | 6.3 |
| Angiopoietin-1 | control | 33 | 3.77 | 0.31 | 3.4 | 2.5 | 4.7 | 2.2 |
| (ANG-1; ng/ml) | AREDS 3 | 24 | 3.99 | 0.38 | 2.95 | 2.77 | 4.95 | 2.18 |
|  | GA | 37 | 4.73 | 0.33 | 4.5 | 3.6 | 5.2 | 1.6 |
| Angiopoietin- | control | 33 | 112.36 | 6.33 | 110 | 89 | 131 | 42 |
| Related protein-3 | AREDS 3 | 24 | 118.5 | 6.42 | 110 | 93.75 | 140.5 | 46.75 |
| (ANGPTL3; ng/ml) | GA | 37 | 121.86 | 5.53 | 119 | 100 | 136 | 36 |
| Angiotensin | control | 33 | 71.91 | 6 | 61 | 54 | 82 | 28 |
| Converting Enzyme | AREDS 3 | 24 | 72.29 | 5.3 | 65 | 57 | 91 | 34 |
| (ACE; ng/ml) | GA | 37 | 82.49 | 5.99 | 70 | 56 | 98 | 42 |
| Anti-leukoprotein | control | 33 | 25.48 | 1.04 | 26 | 21 | 27 | 6 |
| (A) | AREDS 3 | 24 | 28.04 | 1.29 | 27.5 | 25.75 | 29.25 | 3.5 |
| ALP; ng/ml) | GA | 37 | 31.59 | 2.25 | 28 | 25 | 33 | 8 |
| Anti-thrombin-III | control | 33 | 285.52 | 8.91 | 279 | 248 | 319 | 71 |
| (AT-III; ug/ml) | AREDS 3 | 24 | 274.29 | 9.86 | 269.5 | 238.5 | 311 | 72.5 |
|  | GA | 37 | 305.73 | 12.87 | 284 | 242 | 371 | 129 |
| Apolipoprotein(a) | control | 33 | 256.68 | 65.87 | 92 | 30 | 341 | 311 |
| (LP(a); ug/ml) | AREDS 3 | 24 | 119.71 | 23 | 76 | 27.75 | 168.75 | 141 |
|  | GA | 37 | 211.31 | 44.61 | 128 | 22 | 262 | 240 |
| Apolipoprotein-A1 | control | 33 | 1.55 | 0.07 | 1.4 | 1.3 | 1.9 | 0.6 |
| (Apo A-1; mg/ml) | AREDS 3 | 24 | 1.72 | 0.11 | 1.5 | 1.28 | 2.05 | 0.77 |
|  | GA | 37 | 1.77 | 0.09 | 1.7 | 1.3 | 2.1 | 0.8 |
| Apolipoprotein-A2 | control | 33 | 305.67 | 12.2 | 293 | 258 | 322 | 64 |
| (Apo A-II; ng/ml) | AREDS 3 | 24 | 314.08 | 16.08 | 300.5 | 264.75 | 328.25 | 63.5 |
|  | GA | 37 | 311.76 | 11.17 | 308 | 274 | 349 | 75 |
| Apolipoprotein-A4 | control | 33 | 14.84 | 2.24 | 12 | 10 | 15 | 5 |
| (Apo A-IV; ug/ml) | AREDS 3 | 24 | 16.9 | 1.65 | 15 | 13 | 16.75 | 3.75 |
|  | GA | 37 | 14.6 | 1.3 | 13 | 11 | 16 | 5 |
| Apolipoprotein-B | control | 33 | 1.05 | 0.06 | 0.93 | 0.82 | 1.28 | 0.46 |
| (Apo B; mg/ml) | AREDS 3 | 24 | 1.13 | 0.08 | 1.08 | 0.80 | 1.46 | 0.65 |
|  | GA | 37 | 1.12 | 0.07 | 1.06 | 0.84 | 1.29 | 0.45 |
| Apolipoprotein-C1 | control | 33 | 253.21 | 9.46 | 249 | 227 | 280 | 53 |
| (Apo C-1; ng/ml) | AREDS 3 | 24 | 265.54 | 14.63 | 259.5 | 216 | 299.75 | 83.75 |
|  | GA | 37 | 267.73 | 10.71 | 270 | 216 | 310 | 94 |
| Apolipoprotein-CIII | control | 33 | 188.30 | 8.35 | 194 | 146 | 219 | 73 |
| (Apo C-III; ug/ml) | AREDS 3 | 24 | 182.79 | 9.26 | 194 | 154.75 | 204.5 | 49.75 |
|  | GA | 37 | 199.22 | 10.87 | 198 | 171 | 229 | 58 |
| Apolipoprotein-D | control | 33 | 97.94 | 5.03 | 91 | 80 | 114 | 34 |
| (Apo D; ug/ml) | AREDS 3 | 24 | 125.25 | 11.84 | 119 | 85.5 | 135.25 | 49.75 |
|  | GA | 37 | 108.57 | 6.39 | 104 | 84 | 134 | 50 |
| Apolipoprotein-E | control | 33 | 37.12 | 1.89 | 35 | 29 | 47 | 18 |
| (Apo E; ug/ml) | AREDS 3 | 24 | 38.5 | 2.94 | 35.5 | 30.75 | 43.75 | 13 |
|  | GA | 37 | 41.24 | 2.64 | 37 | 31 | 50 | 19 |
| Apolipoprotein-H | control | 33 | 255.36 | 13.91 | 246 | 218 | 282 | 64 |
| (Apo H; ug/ml) | AREDS 3 | 24 | 274.33 | 13.59 | 256 | 236.5 | 320.5 | 84 |
|  | GA | 37 | 279.38 | 11.79 | 275 | 232 | 319 | 87 |
| AXL Receptor | control | 33 | 9.27 | 0.61 | 8.5 | 7.1 | 11 | 3.9 |
| Tyrosine Kinase | AREDS 3 | 24 | 11.19 | 0.62 | 12 | 9 | 13 | 4 |
| (AXL; ng/ml) | GA | 37 | 11.89 | 0.59 | 12 | 9.7 | 15 | 5.3 |
| B Cell-Activating | control | 33 | 559.82 | 26.8 | 527 | 465 | 631 | 166 |
| Factor | AREDS 3 | 24 | 603.46 | 33.25 | 615 | 486.25 | 692.75 | 206.5 |
| (Baff; pg/ml) | GA | 37 | 778.81 | 35.09 | 783 | 671 | 884 | 213 |
| B Lymphocyte | control | 33 | 46.67 | 0.36 | 46 | 46 | 46 | 0 |
| Chemoattractant | AREDS 3 | 24 | 68.33 | 17.89 | 46 | 46 | 46 | 0 |
| (BLC; pg/ml) | GA | 37 | 46.16 | 0.16 | 46 | 46 | 46 | 0 |
| Amyloid-β (1-40) | control | 33 | 0.28 | 0.02 | 0.26 | 0.18 | 0.39 | 0.21 |
| (Aβ(1-40); ng/ml) | AREDS 3 | 24 | 0.32 | 0.02 | 0.34 | 0.25 | 0.42 | 0.17 |
|  | GA | 37 | 0.38 | 0.02 | 0.36 | 0.31 | 0.45 | 0.14 |
| Amyloid-β (1-42) | control | 33 | 0.32 | 0.05 | 0.2 | 0.15 | 0.31 | 0.16 |
| (Aβ(1-42); ng/ml) | AREDS 3 | 24 | 0.26 | 0.03 | 0.22 | 0.18 | 0.27 | 0.09 |
|  | GA | 37 | 0.34 | 0.08 | 0.24 | 0.15 | 0.31 | 0.16 |
| Beta 2-Micro- | control | 33 | 1.92 | 0.11 | 1.8 | 1.5 | 2.1 | 0.60 |
| globulin | AREDS 3 | 24 | 2.37 | 0.16 | 2.1 | 1.9 | 2.55 | 0.65 |
| (B2M; ug/ml) | GA | 37 | 2.38 | 0.13 | 2.2 | 1.9 | 2.6 | 0.70 |
| Beta-micro- | control | 33 | 13.39 | 2.10 | 13 | 5.3 | 17 | 11.70 |
| seminoprotein | AREDS 3 | 24 | 20.32 | 5.15 | 10 | 4.4 | 25.25 | 20.85 |
| PSP94; ng/ml) | GA | 37 | 25.14 | 4.81 | 18 | 11 | 33 | 22.00 |
| Betacellulin | control | 33 | 83.36 | 0.36 | 83 | 83 | 83 | 0 |
| (BTC; pg/ml) | AREDS 3 | 24 | 87.58 | 4.58 | 83 | 83 | 83 | 0 |
|  | GA | 37 | 84.49 | 1.49 | 83 | 83 | 83 | 0 |
| Brain-Derived | control | 33 | 3.90 | 1.44 | 1.5 | 0.86 | 2.60 | 1.74 |
| Neurotrophic Factor | AREDS 3 | 24 | 6.01 | 1.69 | 2.7 | 1.500 | 4.55 | 3.05 |
| (BDNF; ng/ml) | GA | 37 | 5.98 | 1.56 | 2.5 | 0.88 | 5.10 | 4.22 |
| C-Peptide | control | 33 | 1.82 | 0.27 | 1.4 | 0.95 | 2.00 | 1.05 |
| (ng/ml) | AREDS 3 | 24 | 1.45 | 0.25 | 1.1 | 0.65 | 1.45 | 0.80 |
|  | GA | 37 | 1.63 | 0.17 | 1.4 | 0.94 | 2.30 | 1.36 |
| C-Reactive Protein | control | 33 | 4.83 | 1.01 | 2.9 | 1.4 | 4.9 | 3.5 |
| (CRP; ug/ml) | AREDS 3 | 24 | 2.74 | 0.65 | 1.5 | 0.63 | 3.42 | 2.8 |
|  | GA | 37 | 6.58 | 3.02 | 2.5 | 1.2 | 4.6 | 3.4 |
| Cadherin-1 | control | 33 | 2.47 | 0.19 | 2.29 | 1.87 | 2.91 | 1.04 |
| (E-Cad; ug/ml) | AREDS 3 | 24 | 2.94 | 0.29 | 2.49 | 2.18 | 3.29 | 1.12 |
|  | GA | 37 | 3.12 | 0.19 | 3.11 | 2.36 | 3.80 | 1.44 |
| Cadherin-1 | control | 33 | 4.96 | 0.22 | 4.9 | 4.1 | 5.7 | 1.6 |
| (T-Cad; ng/ml) | AREDS 3 | 24 | 5.52 | 0.29 | 5.6 | 4.55 | 6.4 | 1.85 |
|  | GA | 37 | 13.96 | 1.52 | 14 | 5.5 | 21 | 15.5 |
| Calbindin (ng/ml) | control | 33 | 14 | 0 | 14 | 14 | 14 | 0 |
|  | AREDS 3 | 24 | 14.08 | 0.08 | 14 | 14 | 14 | 0 |
|  | GA | 37 | 14.19 | 0.10 | 14 | 14 | 14 | 0 |
| Cancer Antigen 125 | control | 33 | 5.79 | 0.16 | 5.4 | 5.4 | 5.8 | 0.4 |
| (CA-125; U/ml) | AREDS 3 | 24 | 6.06 | 0.3 | 5.4 | 5.4 | 5.8 | 0.4 |
|  | GA | 37 | 6.54 | 0.29 | 5.4 | 5.4 | 7.7 | 2.3 |
| Cancer Antigen 15-3 | control | 33 | 3.44 | 0.28 | 3.3 | 2.3 | 3.9 | 1.6 |
| (CA-15-3; U/ml) | AREDS 3 | 24 | 4.29 | 0.4 | 3.7 | 3 | 5.15 | 2.15 |
|  | GA | 37 | 3.95 | 0.41 | 3.5 | 2 | 4.8 | 2.8 |
| Cancer Antigen 19-9 | control | 33 | 6.47 | 0.78 | 5.3 | 1.8 | 9.8 | 8 |
| (CA-19-9; U/ml) | AREDS 3 | 24 | 13.02 | 3.83 | 7.55 | 2.9 | 14.5 | 11.6 |
|  | GA | 37 | 14.28 | 1.91 | 12 | 5 | 19 | 14 |
| Carbonic Anydrase 9 | control | 33 | 0.28 | 0.03 | 0.22 | 0.22 | 0.24 | 0.02 |
| (CA9; ng/ml) | AREDS 3 | 24 | 0.4 | 0.06 | 0.26 | 0.22 | 0.45 | 0.23 |
|  | GA | 37 | 0.35 | 0.05 | 0.24 | 0.22 | 0.32 | 0.1 |
| Carcinoembryonic | control | 33 | 1.39 | 0.23 | 1 | 0.58 | 1.6 | 1.02 |
| Antigen | AREDS 3 | 24 | 1.13 | 0.23 | 0.91 | 0.62 | 1.1 | 0.48 |
| (CEA; ng/ml) | GA | 37 | 1.65 | 0.22 | 1.1 | 0.75 | 2 | 1.25 |
| Carcinoembryonic | control | 33 | 13.95 | 1.000 | 13 | 11 | 15 | 4 |
| Antigen-Related Cell | AREDS 3 | 24 | 15.59 | 1.32 | 13 | 11.75 | 18 | 6.25 |
| Adhesion Molecule 1 | GA | 37 | 17.25 | 0.74 | 16 | 14 | 19 | 5 |
| (cecam1; ng/ml)  Carcinoembryonic | control | 33 | 63.55 | 3.72 | 65 | 51 | 71 | 20 |
| Antigen-Related Cell | AREDS 3 | 24 | 68.38 | 5.26 | 63.5 | 54 | 82.25 | 28.25 |
| Adhesion Molecule 6 | GA | 37 | 75.19 | 6.8 | 64 | 55 | 78 | 23 |
| (ceacam6; ng/ml)  Cartilage Oligomeric | control | 33 | 359.76 | 20.99 | 338 | 299 | 443 | 144 |
| Matrix Protein | AREDS 3 | 24 | 512.54 | 40.67 | 510 | 372.75 | 636 | 263.25 |
| (COMP; ng/ml) | GA | 37 | 517.35 | 33.25 | 504 | 367 | 653 | 286 |
| Cathespsin D | control | 33 | 42.12 | 4.68 | 35 | 27 | 39 | 12 |
| (ng/ml) | AREDS 3 | 24 | 55.33 | 8.27 | 39.5 | 33 | 65 | 32 |
|  | GA | 37 | 53.81 | 4.14 | 44 | 37 | 64 | 27 |
| Cathepsin D | control | 33 | 336.15 | 29.66 | 273 | 229 | 349 | 120 |
| (ng/ml) | AREDS 3 | 24 | 338.46 | 27.93 | 313 | 243 | 347.25 | 104.25 |
|  | GA | 37 | 425.62 | 26.61 | 396 | 306 | 499 | 193 |
| CD5 Antigen-like | control | 33 | 5.02 | 0.52 | 4.11 | 3.43 | 5.65 | 2.22 |
| (CD5L; ug/ml) | AREDS 3 | 24 | 4.94 | 0.40 | 4.77 | 3.96 | 6.33 | 2.37 |
|  | GA | 37 | 5.41 | 0.53 | 4.96 | 3.69 | 6.18 | 2.49 |
| CD27 (U/ml) | control | 33 | 42.27 | 8.29 | 26 | 19 | 41 | 22 |
|  | AREDS 3 | 24 | 58.21 | 15.06 | 35.5 | 24 | 51.25 | 27.25 |
|  | GA | 37 | 80.78 | 8.55 | 65 | 42 | 107 | 65 |
| CD40 (ng/ml) | control | 33 | 0.67 | 0.05 | 0.63 | 0.51 | 0.70 | 0.19 |
|  | AREDS 3 | 24 | 0.90 | 0.08 | 0.74 | 0.64 | 1.02 | 0.38 |
|  | GA | 37 | 0.90 | 0.06 | 0.76 | 0.65 | 1.00 | 0.35 |
| CD40 Ligand | control | 33 | 0.38 | 0.13 | 0.17 | 0.1 | 0.27 | 0.17 |
| (CD40L; ng/ml) | AREDS 3 | 24 | 0.66 | 0.21 | 0.26 | 0.15 | 0.42 | 0.28 |
|  | GA | 37 | 0.51 | 0.14 | 0.16 | 0.07 | 0.38 | 0.31 |
| CD163 (ng/ml) | control | 33 | 235.21 | 17.86 | 222 | 170 | 253 | 83 |
|  | AREDS 3 | 24 | 273.04 | 25.18 | 234 | 196.25 | 312 | 115.75 |
|  | GA | 37 | 270.78 | 20.49 | 247 | 200 | 299 | 99 |
| Cellular Fibronectin | control | 33 | 11.94 | 1.88 | 8.9 | 4.5 | 14 | 9.5 |
| (cFib; ug/ml) | AREDS 3 | 24 | 10.1 | 1.44 | 8.3 | 5.92 | 12 | 6.08 |
|  | GA | 37 | 8.47 | 1.26 | 5.1 | 3.4 | 12 | 8.6 |
| Ceruloplasmin | control | 33 | 155.52 | 5.68 | 158 | 138 | 169 | 31 |
| (ug/ml) | AREDS 3 | 24 | 172.04 | 8.43 | 166.5 | 150.75 | 183.25 | 32.5 |
|  | GA | 37 | 185.14 | 9.36 | 172 | 139 | 217 | 78 |
| Chemerin (ng/m) | control | 33 | 88.42 | 4.2 | 83 | 71 | 98 | 27 |
|  | AREDS 3 | 24 | 85.75 | 3.99 | 81 | 73.75 | 95.5 | 21.75 |
|  | GA | 37 | 97.7 | 4.71 | 89 | 77 | 108 | 31 |
| Chemokine CC-4 | control | 33 | 5.13 | 0.32 | 4.7 | 4.1 | 5.9 | 1.8 |
| (HCC-4; ng/ml) | AREDS 3 | 24 | 4.24 | 0.31 | 4.25 | 3.15 | 4.78 | 1.62 |
|  | GA | 37 | 5.55 | 0.4 | 4.9 | 4.3 | 6.5 | 2.2 |
| Chromogranin-A | control | 33 | 548.52 | 87.48 | 362 | 270 | 657 | 387 |
| C(gA; ng/ml) | AREDS 3 | 24 | 663.71 | 116.19 | 450.5 | 305.5 | 909.5 | 604 |
|  | GA | 37 | 668.22 | 94.87 | 447 | 324 | 788 | 464 |
| Ciliary Neurotrophic | control | 33 | 19.18 | 0.18 | 19 | 19 | 19 | 0 |
| Factor | AREDS 3 | 24 | 19 | 0 | 19 | 19 | 19 | 0 |
| (CNFT; pg/ml) | GA | 37 | 19 | 0 | 19 | 19 | 19 | 0 |
| Clusterin | control | 33 | 166.33 | 8.46 | 157 | 144 | 174 | 30 |
| (CLU; ug/ml) | AREDS 3 | 24 | 186.79 | 10.94 | 167 | 156 | 213 | 57 |
|  | GA | 37 | 218.59 | 11.93 | 206 | 165 | 261 | 96 |
| Collagen IV | control | 33 | 61.73 | 3.97 | 54 | 45 | 73 | 28 |
| (Collagen4; ng/ml) | AREDS 3 | 24 | 81.62 | 7.89 | 72.5 | 57.75 | 83 | 25.25 |
|  | GA | 37 | 100.84 | 7.15 | 91 | 71 | 125 | 54 |
| Complement C3 | control | 33 | 1.1 | 0.05 | 1.1 | 0.88 | 1.2 | 0.32 |
| (C3; mg/ml) | AREDS 3 | 24 | 0.98 | 0.04 | 0.93 | 0.84 | 1.1 | 0.26 |
|  | GA | 37 | 1.15 | 0.07 | 1.1 | 0.96 | 1.3 | 0.34 |
| Complement | control | 33 | 6.3 | 0.32 | 6.1 | 5 | 7.5 | 2.5 |
| Component C1q | AREDS 3 | 24 | 8.87 | 0.56 | 8.05 | 7.55 | 9.55 | 2 |
| Receptor | GA | 37 | 7.75 | 0.32 | 7.5 | 6.7 | 9 | 2.3 |
| (c1qr1; ug/ml)  Complement Factor | control | 33 | 585.24 | 17.8 | 570 | 526 | 637 | 111 |
| H | AREDS 3 | 24 | 551.33 | 16.18 | 552 | 521.25 | 614 | 92.75 |
| (CFH; ug/ml) | GA | 37 | 613.35 | 20.59 | 616 | 549 | 694 | 145 |
| Complement Factor | control | 33 | 2.40 | 0.24 | 2.99 | 1.50 | 3.50 | 2.00 |
| H-Related Protein 1 | AREDS 3 | 24 | 3.19 | 0.24 | 3.36 | 2.33 | 4.09 | 1.76 |
| (CFHR1; mg/ml) | GA | 37 | 3.64 | 0.16 | 3.91 | 3.03 | 4.30 | 1.27 |
| Cortisol (ng/ml) | control | 33 | 181.70 | 13.15 | 171 | 139 | 198 | 59 |
|  | AREDS 3 | 24 | 181.54 | 16.14 | 175 | 136 | 188 | 52 |
|  | GA | 37 | 180.27 | 10.95 | 173 | 137 | 233 | 96 |
| Creatin Kinase-MB | control | 33 | 1.27 | 0.13 | 1.0 | 0.79 | 1.5 | 0.71 |
| (CK-MB; ng/ml) | AREDS 3 | 24 | 1.43 | 0.11 | 1.45 | 1.08 | 1.7 | 0.62 |
|  | GA | 37 | 1.28 | 0.15 | 1.1 | 0.8 | 1.6 | 0.80 |
| Cystatin-A (ng/ml) | control | 33 | 1.51 | 0.17 | 1.3 | 0.86 | 1.9 | 1.04 |
|  | AREDS 3 | 24 | 3.66 | 1.57 | 1.6 | 1.1 | 2.52 | 1.42 |
|  | GA | 37 | 3.01 | 0.93 | 1.2 | 0.86 | 1.8 | 0.94 |
| Cystanin-B (ng/ml) | control | 33 | 10.58 | 0.89 | 9.5 | 7.1 | 12 | 4.9 |
|  | AREDS 3 | 24 | 12.71 | 2.13 | 8.85 | 7.75 | 13.25 | 5.5 |
|  | GA | 37 | 14.93 | 1.54 | 13 | 8.6 | 16 | 7.4 |
| Cystatin-C (ug/ml) | control | 33 | 0.89 | 0.04 | 0.89 | 0.77 | 0.94 | 0.17 |
|  | AREDS 3 | 24 | 1.09 | 0.06 | 1.03 | 0.90 | 1.16 | 0.25 |
|  | GA | 37 | 1.07 | 0.05 | 1.02 | 0.89 | 1.22 | 0.33 |
| Decorin (ng/ml) | control | 33 | 1.78 | 0.04 | 1.8 | 1.7 | 1.9 | 0.2 |
|  | AREDS 3 | 24 | 1.78 | 0.06 | 1.8 | 1.5 | 1.9 | 0.4 |
|  | GA | 37 | 2.11 | 0.13 | 2.0 | 1.6 | 2.4 | 0.8 |
| Dickkopt-related | control | 33 | 0.28 | 0.07 | 0.19 | 0.19 | 0.19 | 0 |
| Protein-1 | AREDS 3 | 24 | 0.36 | 0.1 | 0.19 | 0.19 | 0.19 | 0 |
| (DKK-1; ng/ml) | GA | 37 | 0.33 | 0.08 | 0.19 | 0.19 | 0.20 | 0.01 |
| Dipeptidyl Peptidase | control | 33 | 235.03 | 11.98 | 233 | 187 | 273 | 86 |
| IV | AREDS 3 | 24 | 242.83 | 9.54 | 231 | 203 | 286 | 83 |
| (DPPIV; ng/ml) | GA | 37 | 236.76 | 9.01 | 227 | 193 | 276 | 83 |
| Dopamine Beta- | control | 33 | 6.98 | 1.12 | 5.8 | 2.6 | 8.1 | 5.5 |
| Hydroxylase | AREDS 3 | 24 | 6.82 | 1.32 | 4.45 | 2.45 | 8.3 | 5.85 |
| (DBH; ng/ml) | GA | 37 | 6.55 | 0.84 | 5.4 | 3.3 | 7.8 | 4.5 |
| E-Selectin (ng/ml) | control | 33 | 9.07 | 0.57 | 9.1 | 7.4 | 10 | 2.6 |
|  | AREDS 3 | 24 | 8.32 | 0.63 | 7.95 | 6.25 | 9.88 | 3.62 |
|  | GA | 37 | 7.8 | 0.65 | 7.0 | 5.1 | 10 | 4.9 |
| EN-RAGE (ng/ml) | control | 33 | 29.47 | 6.24 | 18 | 14 | 37 | 23 |
|  | AREDS 3 | 24 | 132.6 | 87.30 | 27.5 | 8.25 | 58 | 49.75 |
|  | GA | 37 | 114.83 | 46.17 | 22 | 11 | 68 | 57 |
| Endoglin (ng/ml) | control | 33 | 3.05 | 0.12 | 3.1 | 2.6 | 3.6 | 1 |
|  | AREDS 3 | 24 | 3.33 | 0.17 | 3.15 | 2.7 | 4.03 | 1.33 |
|  | GA | 37 | 3.45 | 0.14 | 3.2 | 2.7 | 4.1 | 1.4 |
| Endostatin (ng/ml) | control | 33 | 82.39 | 4.91 | 82 | 62 | 90 | 28 |
|  | AREDS 3 | 24 | 98.92 | 6.44 | 86.5 | 83 | 108 | 25 |
|  | GA | 37 | 109.97 | 5.00 | 113 | 85 | 126 | 41 |
| Eotaxin-1 (pg/ml) | control | 33 | 132.42 | 9.19 | 133 | 88 | 150 | 62 |
|  | AREDS 3 | 24 | 179.79 | 27.01 | 150.5 | 99.25 | 189.75 | 90.5 |
|  | GA | 37 | 129.22 | 11.84 | 88 | 62 | 172 | 110 |
| Eotaxin-2 (pg/ml) | control | 33 | 653.09 | 79.7 | 485 | 380 | 747 | 367 |
|  | AREDS 3 | 24 | 1,041.04 | 190.62 | 647.5 | 325.75 | 1445 | 1119.25 |
|  | GA | 37 | 979.22 | 112 | 833 | 484 | 1210 | 726 |
| Eotaxin-3 (pg/ml) | control | 33 | 100.79 | 0.55 | 100 | 100 | 100 | 0 |
|  | AREDS 3 | 24 | 101.62 | 0.9 | 100 | 100 | 100 | 0 |
|  | GA | 37 | 100.35 | 0.35 | 100 | 100 | 100 | 0 |
| Epidermal Growth | control | 33 | 53.74 | 25.43 | 12 | 7.1 | 27 | 19.9 |
| Factor | AREDS 3 | 24 | 122 | 46.48 | 24 | 12.5 | 56.5 | 44 |
| (EGF; pg/ml) | GA | 37 | 74.17 | 23.88 | 16 | 6 | 36 | 30 |
| Epidermal Growth | control | 33 | 3.43 | 0.12 | 3.4 | 3.1 | 3.7 | 0.6 |
| Factor Receptor | AREDS 3 | 24 | 3.26 | 0.13 | 3.2 | 2.9 | 3.52 | 0.62 |
| (EGFR; ng/ml) | GA | 37 | 3.24 | 0.12 | 3.4 | 2.7 | 3.6 | 0.9 |
| Epiregulin | control | 33 | 15 | 0 | 15 | 15 | 15 | 0 |
| (EPR; pg/ml) | AREDS 3 | 24 | 15.96 | 0.96 | 15 | 15 | 15 | 0 |
|  | GA | 37 | 15.24 | 0.24 | 15 | 15 | 15 | 0 |
| Epithelial Cell | control | 33 | 128.24 | 4.41 | 119 | 119 | 119 | 0 |
| Adhesion Molecule | AREDS 3 | 24 | 161.04 | 12.95 | 138 | 119 | 180.5 | 61.5 |
| (EpCam; pg/ml) | GA | 37 | 139.62 | 13.23 | 119 | 119 | 119 | 0 |
| Epithelial-Derived | control | 33 | 0.4 | 0.11 | 0.12 | 0.06 | 0.53 | 0.47 |
| Neutrophilic Activa- | AREDS 3 | 24 | 0.65 | 0.12 | 0.52 | 0.11 | 0.9 | 0.78 |
| ting Protein 78 | GA | 37 | 0.99 | 0.26 | 0.45 | 0.12 | 1.2 | 1.08 |
| (ENA-78; ng/ml)  Factor VII (ng/ml) | control | 33 | 548.58 | 25.61 | 540 | 476 | 600 | 124 |
|  | AREDS 3 | 24 | 529.29 | 29.8 | 509.5 | 439 | 631.75 | 192.75 |
|  | GA | 37 | 528.46 | 25.98 | 505 | 439 | 620 | 181 |
| Fas Ligand | control | 33 | 52.97 | 0.71 | 52 | 52 | 52 | 0 |
| (FasL; pg/ml) | AREDS 3 | 24 | 52.38 | 0.29 | 52 | 52 | 52 | 0 |
|  | GA | 37 | 55.92 | 1.44 | 52 | 52 | 52 | 0 |
| FASLG Receptor | control | 33 | 15.73 | 1.57 | 14 | 11 | 17 | 6 |
| (FAS; ng/ml) | AREDS 3 | 24 | 16.62 | 1.89 | 15 | 9.15 | 19.25 | 10.1 |
|  | GA | 37 | 21.82 | 3.02 | 19 | 12 | 25 | 13 |
| Fatty Acid Binding | control | 33 | 12.38 | 0.84 | 11 | 9 | 15 | 6 |
| Protein Adipocyte | AREDS 3 | 24 | 13.72 | 1.51 | 12 | 9.12 | 15.25 | 6.12 |
| (FABP.adyp; ng/ml) | GA | 37 | 19.64 | 2.08 | 20 | 11 | 24 | 13 |
| Fatty Acid Binding | control | 33 | 7.85 | 0.44 | 6.9 | 6.9 | 7.7 | 0.8 |
| Protein Heart | AREDS 3 | 24 | 8.7 | 0.53 | 7.8 | 6.9 | 8.43 | 1.53 |
| (FABP.heart; ng/ml) | GA | 37 | 9.31 | 0.81 | 6.9 | 6.9 | 10 | 3.1 |
| Fatty Acid Binding | control | 33 | 21.24 | 0.24 | 21 | 21 | 21 | 0 |
| Protein Liver | AREDS 3 | 24 | 23.67 | 1.47 | 21 | 21 | 21 | 0 |
| (FABP.liver; ng/ml) | GA | 37 | 22.7 | 1.17 | 21 | 21 | 21 | 0 |
| Ferritin (ng/ml) | control | 33 | 112.18 | 12.78 | 87 | 65 | 129 | 64 |
|  | AREDS 3 | 24 | 96.25 | 12.57 | 85 | 59 | 117.5 | 58.5 |
|  | GA | 37 | 149.43 | 20.09 | 103 | 51 | 241 | 190 |
| Fetulin-A (ug/ml) | control | 33 | 613.73 | 25.71 | 594 | 547 | 728 | 181 |
|  | AREDS 3 | 24 | 618.46 | 22.04 | 622.5 | 559.5 | 658.25 | 98.75 |
|  | GA | 37 | 687.51 | 29.96 | 659 | 562 | 832 | 270 |
| Fibrinogen (mg/ml) | control | 33 | 3.74 | 0.22 | 3.8 | 3.5 | 4.2 | 0.7 |
|  | AREDS 3 | 24 | 3.36 | 0.31 | 3.7 | 3.15 | 4.12 | 0.97 |
|  | GA | 37 | 4.14 | 0.29 | 4.3 | 3.2 | 5.4 | 2.2 |
| FGF-4 (pg/ml) | control | 33 | 243 | 0 | 243 | 243 | 243 | 0 |
|  | AREDS 3 | 24 | 243 | 0 | 243 | 243 | 243 | 0 |
|  | GA | 37 | 243 | 0 | 243 | 243 | 243 | 0 |
| FGF-21 (pg/ml) | control | 33 | 0.19 | 0.05 | 0.08 | 0.04 | 0.14 | 0.1 |
|  | AREDS 3 | 24 | 0.15 | 0.02 | 0.12 | 0.08 | 0.22 | 0.14 |
|  | GA | 37 | 0.3 | 0.1 | 0.13 | 0.08 | 0.19 | 0.11 |
| FGF-23 (pg/ml) | control | 33 | 0.12 | 0.01 | 0.11 | 0.06 | 0.18 | 0.12 |
|  | AREDS 3 | 24 | 0.15 | 0.01 | 0.14 | 0.1 | 0.2 | 0.1 |
|  | GA | 37 | 0.17 | 0.03 | 0.12 | 0.08 | 0.18 | 0.1 |
| FGF-basic (pg/ml) | control | 33 | 27 | 0 | 27 | 27 | 27 | 0 |
|  | AREDS 3 | 24 | 27 | 0 | 27 | 27 | 27 | 0 |
|  | GA | 37 | 27 | 0 | 27 | 27 | 27 | 0 |
| Fibulin-1C (ug/ml) | control | 33 | 25.91 | 1.14 | 24 | 20 | 30 | 10 |
|  | AREDS 3 | 24 | 28.42 | 2.66 | 26 | 23 | 29 | 6 |
|  | GA | 37 | 28.14 | 1.72 | 29 | 22 | 34 | 12 |
| Ficolin-3 (ug/ml) | control | 33 | 19.21 | 1.42 | 17 | 14 | 23 | 9 |
| ficolin3 | AREDS 3 | 24 | 15.69 | 0.98 | 15 | 12.75 | 18.25 | 5.5 |
|  | GA | 37 | 19.7 | 0.91 | 19 | 17 | 24 | 7 |
| Follicle Stimulating | control | 33 | 38.15 | 5.18 | 34 | 11 | 53 | 42 |
| Hormone | AREDS 3 | 24 | 41.72 | 4.94 | 39.5 | 32 | 55 | 23 |
| (FSH; mU/ml) | GA | 37 | 29.73 | 3.39 | 29 | 9 | 49 | 40 |
| Galectin-3 (ng/ml) | control | 33 | 18.88 | 0.56 | 19 | 17 | 21 | 4 |
|  | AREDS 3 | 24 | 19.25 | 1.05 | 18 | 15.75 | 24 | 8.25 |
|  | GA | 37 | 19.98 | 0.73 | 21 | 18 | 23 | 5 |
| Gastric Inhibitory | control | 33 | 0.49 | 0.09 | 0.33 | 0.23 | 0.55 | 0.32 |
| Polypeptide | AREDS 3 | 24 | 0.51 | 0.13 | 0.27 | 0.14 | 0.62 | 0.48 |
| (GIP; ng/ml) | GA | 37 | 0.41 | 0.06 | 0.33 | 0.11 | 0.64 | 0.53 |
| Gelsolin (ug/ml) | control | 33 | 33.91 | 1.19 | 32 | 30 | 38 | 8 |
|  | AREDS 3 | 24 | 39.33 | 1.75 | 36.5 | 32.75 | 44.25 | 11.5 |
|  | GA | 37 | 34.65 | 1.38 | 34 | 29 | 41 | 12 |
| Glucagon (pg/ml) | control | 33 | 335 | 1 | 334 | 334 | 334 | 0 |
|  | AREDS 3 | 24 | 334 | 0 | 334 | 334 | 334 | 0 |
|  | GA | 37 | 334 | 0 | 334 | 334 | 334 | 0 |
| Glucagon-Like | control | 33 | 24 | 0 | 24 | 24 | 24 | 0 |
| Peptide 1, active | AREDS 3 | 24 | 24 | 0 | 24 | 24 | 24 | 0 |
| (GLP-1 active; pg/ml) | GA | 37 | 24 | 0 | 24 | 24 | 24 | 0 |
| Glucagon-Like | control | 33 | 8.34 | 0.35 | 7.3 | 7.3 | 7.9 | 0.6 |
| Peptide 1, total | AREDS 3 | 24 | 8.18 | 0.37 | 7.3 | 7.3 | 7.45 | 0.15 |
| (GLP-1 total; pg/ml) | GA | 37 | 9.15 | 0.62 | 7.3 | 7.3 | 9 | 1.7 |
| Glucose-6-phosphate | control | 33 | 44.21 | 4.21 | 35 | 28 | 50 | 22 |
| Isomerase | AREDS 3 | 24 | 61.92 | 11.95 | 45 | 32 | 66.75 | 34.75 |
| (G6PI; ng/ml) | GA | 37 | 64.51 | 11.55 | 41 | 31 | 59 | 28 |
| Glutathione S- | control | 33 | 9.01 | 0.95 | 5.7 | 5.7 | 11 | 5.3 |
| Transferase Alpha | AREDS 3 | 24 | 10.56 | 2.89 | 6 | 5.7 | 10 | 4.3 |
| (GST-Alpha; ng/ml) | GA | 37 | 11.7 | 1.52 | 7.3 | 5.7 | 15 | 9.3 |
| Glutathione S- | control | 33 | 3.9 | 0.09 | 3.8 | 3.8 | 3.8 | 0 |
| Transferase Mu | AREDS 3 | 24 | 3.81 | 0.01 | 3.8 | 3.8 | 3.8 | 0 |
| (GST-Mu; ng/ml) | GA | 37 | 3.89 | 0.09 | 3.8 | 3.8 | 3.8 | 0 |
| Glucagon Phospho- | control | 33 | 57.64 | 11.19 | 37 | 37 | 37 | 0 |
| rylase isoenzyme | AREDS 3 | 24 | 83.17 | 18.86 | 37 | 37 | 117 | 80 |
| (GPBB; ng/ml) | GA | 37 | 79.46 | 19.73 | 37 | 37 | 61 | 24 |
| Granulocyte Colony | control | 33 | 6.83 | 0.39 | 6.3 | 6.3 | 6.3 | 0 |
| Stimulating Protein | AREDS 3 | 24 | 6.42 | 0.09 | 6.3 | 6.3 | 6.3 | 0 |
| (G-CSF; pg/ml) | GA | 37 | 6.75 | 0.19 | 6.3 | 6.3 | 6.3 | 0 |
| Granulocyte- | control | 33 | 53 | 0 | 53 | 53 | 53 | 0 |
| Macrophage Colony | AREDS 3 | 24 | 56.58 | 3.58 | 53 | 53 | 53 | 0 |
| Stimulating Protein | GA | 37 | 53 | 0 | 53 | 53 | 53 | 0 |
| (GM-CSF; pg/ml)  Growth/Differentia- | control | 33 | 0.38 | 0.05 | 0.32 | 0.23 | 0.43 | 0.20 |
| tion-15 | AREDS 3 | 24 | 0.47 | 0.04 | 0.40 | 0.36 | 0.48 | 0.12 |
| (GDF-15; ng/ml) | GA | 37 | 0.58 | 0.04 | 0.56 | 0.46 | 0.64 | 0.18 |
| Growth Hormone | control | 33 | 0.54 | 0.15 | 0.24 | 0.14 | 0.51 | 0.37 |
| (GH; ng/ml) | AREDS 3 | 24 | 0.44 | 0.12 | 0.24 | 0.17 | 0.49 | 0.32 |
|  | GA | 37 | 0.4 | 0.11 | 0.18 | 0.11 | 0.39 | 0.28 |
| Growth-Related | control | 33 | 46.55 | 8.88 | 27 | 18 | 45 | 27 |
| Alpha Protein | AREDS 3 | 24 | 63.71 | 12.16 | 50 | 22.75 | 68.75 | 46 |
| (GRO-alpha; pg/ml) | GA | 37 | 73 | 10.24 | 60 | 28 | 93 | 65 |
| Haptoglobin (mg/ml) | control | 33 | 1.35 | 0.18 | 1.2 | 0.42 | 2.3 | 1.88 |
|  | AREDS 3 | 24 | 1.08 | 0.16 | 0.84 | 0.61 | 1.6 | 0.99 |
|  | GA | 37 | 1.39 | 0.2 | 1.1 | 0.71 | 1.5 | 0.79 |
| HE4 (pMolar) | control | 33 | 578.48 | 64.56 | 497 | 339 | 668 | 329 |
|  | AREDS 3 | 24 | 696.96 | 77.19 | 552 | 453 | 737 | 284 |
|  | GA | 37 | 698.35 | 64.09 | 575 | 474 | 746 | 272 |
| Heat Shock Protein- | control | 33 | 40 | 0 | 40 | 40 | 40 | 0 |
| 60 | AREDS 3 | 24 | 40 | 0 | 40 | 40 | 40 | 0 |
| (HSP-60; ng/ml) | GA | 37 | 42.54 | 2.54 | 40 | 40 | 40 | 0 |
| Heat Shock Protein- | control | 33 | 7.34 | 0.69 | 5.7 | 4.9 | 8.5 | 3.6 |
| 70 | AREDS 3 | 24 | 11.99 | 3.51 | 7.45 | 4.68 | 11.5 | 6.82 |
| (HSP-60; ng/ml) | GA | 37 | 11.82 | 2.25 | 6.3 | 4.7 | 13 | 8.3 |
| Hemopexin (mg/ml) | control | 33 | 1.37 | 0.05 | 1,30 | 1.20 | 1.47 | 0.27 |
|  | AREDS 3 | 24 | 1.37 | 0.60 | 1.32 | 1.23 | 1.39 | 0.15 |
|  | GA | 37 | 1.51 | 0.56 | 1.57 | 1.26 | 1.71 | 0.45 |
| Heparin Binding EGF- | control | 33 | 27.06 | 3.12 | 23 | 23 | 23 | 0 |
| Like Growth Factor | AREDS 3 | 24 | 27.00 | 2.13 | 23 | 23 | 23 | 0.25 |
| (HB-EGF; pg/ml) | GA | 37 | 27.35 | 2.37 | 23 | 23 | 23 | 0 |
| Hepatocyte Growth | control | 33 | 5.58 | 0.54 | 4.9 | 4.2 | 5.3 | 1.1 |
| Factor | AREDS 3 | 24 | 6.26 | 0.66 | 5.35 | 4 | 6.72 | 2.72 |
| (HGF; ng/ml) | GA | 37 | 6.73 | 0.49 | 6.2 | 4.9 | 7.3 | 2.4 |
| HGF Receptor | control | 33 | 53.03 | 3.08 | 49 | 43 | 61 | 18 |
| (ng/ml) | AREDS 3 | 24 | 57.46 | 4.07 | 59 | 43 | 65 | 22 |
|  | GA | 37 | 61.32 | 3.00 | 57 | 52 | 78 | 26 |
| Hepsin (pg/ml) | control | 33 | 852.70 | 42.15 | 773 | 674 | 1030 | 356 |
|  | AREDS 3 | 24 | 921.96 | 43.91 | 870 | 788 | 1033 | 245 |
|  | GA | 37 | 695.46 | 49.36 | 640 | 486 | 843 | 357 |
| Human Chorionic | control | 33 | 1.9 | 0.18 | 1.3 | 1.3 | 2.2 | 0.9 |
| Gonadotropin beta | AREDS 3 | 24 | 2.2 | 0.38 | 1.4 | 1.3 | 2.6 | 1.3 |
| (HCG; mU/ml) | GA | 37 | 1.66 | 0.11 | 1.3 | 1.3 | 1.6 | 0.3 |
| Human Epidermal | control | 33 | 0.44 | 0.02 | 0.42 | 0.36 | 0.48 | 0.12 |
| Growth Factor-2 | AREDS 3 | 24 | 0.45 | 0.03 | 0.41 | 0.36 | 0.52 | 0.16 |
| (HER-2; ng/ml) | GA | 37 | 0.48 | 0.02 | 0.51 | 0.36 | 0.59 | 0.23 |
| IgA (mg/ml) | control | 33 | 2.79 | 0.3 | 2.4 | 1.6 | 4 | 2.4 |
|  | AREDS 3 | 24 | 2.56 | 0.4 | 2.15 | 1.37 | 3.1 | 1.73 |
|  | GA | 37 | 2.74 | 0.24 | 2 | 1.7 | 3.4 | 1.7 |
| IgE (mg/ml) | control | 33 | 50.73 | 10.36 | 23 | 22 | 40 | 18 |
|  | AREDS 3 | 24 | 59.12 | 32.92 | 22 | 22 | 25.25 | 3.25 |
|  | GA | 37 | 106.08 | 28.99 | 35 | 26 | 101 | 75 |
| IgM (mg/ml) | control | 33 | 1.46 | 0.15 | 1.3 | 0.79 | 1.8 | 1.01 |
|  | AREDS 3 | 24 | 1.97 | 0.33 | 1.45 | 0.7 | 2.67 | 1.97 |
|  | GA | 37 | 1.7 | 0.15 | 1.3 | 1.1 | 2 | 0.9 |
| Insulin (ulU/ml) | control | 33 | 2.31 | 0.45 | 1.4 | 0.96 | 2.1 | 1.14 |
|  | AREDS 3 | 24 | 1.76 | 0.29 | 1.3 | 0.99 | 1.92 | 0.93 |
|  | GA | 37 | 2.23 | 0.3 | 1.9 | 1.4 | 2.5 | 1.1 |
| Insulin-Like Growth | control | 33 | 11.96 | 2.96 | 4.2 | 4.2 | 6.2 | 2 |
| Factor Binding | AREDS 3 | 24 | 16.3 | 3.62 | 6.65 | 4.2 | 18.75 | 14.55 |
| Protein-1 | GA | 37 | 16.19 | 3.17 | 6.3 | 4.2 | 19 | 14.8 |
| (IGFBP1; ng/ml)  IGFBP2 (ng/ml) | control | 33 | 115.61 | 9.35 | 104 | 77 | 152 | 75 |
|  | AREDS 3 | 24 | 175.83 | 19.72 | 160 | 110.75 | 201 | 90.25 |
|  | GA | 37 | 159.49 | 11.69 | 151 | 99 | 218 | 119 |
| IGFBP3 (ug/ml) | control | 33 | 1.63 | 0.10 | 1.52 | 1.27 | 1.76 | 0.49 |
|  | AREDS 3 | 24 | 1.89 | 0.15 | 1.73 | 1.42 | 1.98 | 0.56 |
|  | GA | 37 | 1.61 | 0.10 | 1.53 | 1.29 | 1.82 | 0.53 |
| IGFBP4 (ng/ml) | control | 33 | 361.33 | 26.8 | 311 | 287 | 388 | 101 |
|  | AREDS 3 | 24 | 481.54 | 46.27 | 401 | 356 | 510.25 | 154.25 |
|  | GA | 37 | 517.95 | 45.59 | 416 | 380 | 577 | 197 |
| IGFBP5 (ng/ml) | control | 33 | 204.12 | 4.5 | 206 | 181 | 222 | 41 |
|  | AREDS 3 | 24 | 214.75 | 6.11 | 216.5 | 199.75 | 234.25 | 34.5 |
|  | GA | 37 | 188.81 | 4.62 | 192 | 168 | 207 | 39 |
| IGFBP6 (ng/ml) | control | 33 | 397.48 | 38.8 | 368 | 277 | 416 | 139 |
|  | AREDS 3 | 24 | 488.58 | 43.16 | 430.5 | 387 | 496.75 | 109.75 |
|  | GA | 37 | 589.89 | 37.74 | 554 | 441 | 655 | 214 |
| IGFBP7 (ng/ml) | control | 33 | 39.61 | 1.36 | 39 | 35 | 43 | 8 |
|  | AREDS 3 | 24 | 44.54 | 1.66 | 45 | 41.75 | 49 | 7.25 |
|  | GA | 37 | 46.38 | 1.73 | 45 | 40 | 50 | 10 |
| Intercellular | control | 33 | 120.58 | 5.27 | 118 | 101 | 135 | 34 |
| Adhesion Molecule 1 | AREDS 3 | 24 | 115.75 | 5.3 | 115 | 101.75 | 132.5 | 30.75 |
| (ICAM-1; ng/ml) | GA | 37 | 120.81 | 5.92 | 115 | 97 | 147 | 50 |
| Interferon Alpha | control | 33 | 0.01 | 0 | 0.01 | 0.01 | 0.01 | 0 |
| (IFN-alpha; pg/ml) | AREDS 3 | 24 | 0.01 | 0 | 0.01 | 0.01 | 0.01 | 0 |
|  | GA | 37 | 0.01 | 0 | 0.01 | 0.01 | 0.01 | 0 |
| Interferon Gamma | control | 33 | 4.1 | 0 | 4.1 | 4.1 | 4.1 | 0 |
| (IFN-gamma; pg/ml) | AREDS 3 | 24 | 4.1 | 0 | 4.1 | 4.1 | 4.1 | 0 |
|  | GA | 37 | 4.11 | 0.01 | 4.1 | 4.1 | 4.1 | 0 |
| Interferon Gamma | control | 33 | 288.67 | 23.23 | 252 | 201 | 315 | 114 |
| Induced Protein 10 | AREDS 3 | 24 | 349.42 | 65.33 | 295 | 204.25 | 346 | 141.75 |
| (IP-10; pg/ml) | GA | 37 | 343.95 | 21.56 | 328 | 258 | 419 | 161 |
| Interferon Gamma | control | 33 | 45.73 | 1.44 | 42 | 42 | 42 | 0 |
| Induced Alpha | AREDS 3 | 24 | 53.46 | 5.78 | 42 | 42 | 44 | 2 |
| Chemoattractant | GA | 37 | 48.95 | 2.17 | 42 | 42 | 48 | 6 |
| (ITAC; pg/ml)  IL-1 alpha (ng/ml) | control | 33 | 0 | 0 | 0 | 0 | 0 | 0 |
|  | AREDS 3 | 24 | 0 | 0 | 0 | 0 | 0 | 0 |
|  | GA | 37 | 0 | 0 | 0 | 0 | 0 | 0 |
| IL-1 beta (pg/ml) | control | 33 | 51.03 | 15.62 | 5.8 | 4.3 | 59 | 54.7 |
|  | AREDS 3 | 24 | 33.68 | 18.74 | 5.4 | 4.53 | 5.7 | 1.17 |
|  | GA | 37 | 4.89 | 0.25 | 4.8 | 3.4 | 5.8 | 2.4 |
| IL-1 Receptor | control | 33 | 882.94 | 51.14 | 898 | 694 | 1000 | 306 |
| Antagonist | AREDS 3 | 24 | 848.83 | 59.56 | 797 | 694 | 1110 | 416 |
| (IL-1ra; pg/ml) | GA | 37 | 836.08 | 46 | 848 | 592 | 1110 | 518 |
| IL-1 Receptor Type 1 | control | 33 | 1,105 | 42.89 | 1,080 | 950 | 1,210 | 260 |
| (IL-1R1; pg/ml) | AREDS 3 | 24 | 1,240 | 43.29 | 1,190 | 1,087 | 1,412 | 325 |
|  | GA | 37 | 1,320 | 46.86 | 1,400 | 1,190 | 1,550 | 360 |
| IL-1 Receptor Type 2 | control | 33 | 10.07 | 0.48 | 9.8 | 8.1 | 12 | 3.9 |
| (IL-1RII; ng/ml) | AREDS 3 | 24 | 9.67 | 0.47 | 8.9 | 7.6 | 12 | 4.4 |
|  | GA | 37 | 9.33 | 0.42 | 9.4 | 7.1 | 11 | 3.9 |
| IL-2 (pg/ml) | control | 33 | 28 | 0 | 28 | 28 | 28 | 0 |
|  | AREDS 3 | 24 | 30.62 | 2.63 | 28 | 28 | 28 | 0 |
|  | GA | 37 | 28 | 0 | 28 | 28 | 28 | 0 |
| IL-2 Receptor Alpha | control | 33 | 2.13 | 0.15 | 1.92 | 1.60 | 2.34 | 0.74 |
| (IL-2ra; ng/ml) | AREDS 3 | 24 | 2.97 | 0.37 | 2.40 | 1.74 | 3.45 | 1.72 |
|  | GA | 37 | 3.05 | 0.18 | 2.94 | 2.31 | 3.71 | 1.40 |
| IL-3 (ng/ml) | control | 33 | 0.01 | 0 | 0.01 | 0.01 | 0.01 | 0 |
|  | AREDS 3 | 24 | 0.01 | 0 | 0.01 | 0.01 | 0.01 | 0 |
|  | GA | 37 | 0.01 | 0 | 0.01 | 0.01 | 0.01 | 0 |
| IL-4 (pg/ml) | control | 33 | 35 | 0 | 35 | 35 | 35 | 0 |
|  | AREDS 3 | 24 | 40.38 | 5.38 | 35 | 35 | 35 | 0 |
|  | GA | 37 | 35 | 0 | 35 | 35 | 35 | 0 |
| IL-5 (pg/ml) | control | 33 | 9.1 | 0 | 9.1 | 9.1 | 9.1 | 0 |
|  | AREDS 3 | 24 | 9.1 | 0 | 9.1 | 9.1 | 9.1 | 0 |
|  | GA | 37 | 9.1 | 0 | 9.1 | 9.1 | 9.1 | 0 |
| IL-6 (pg/ml) | control | 33 | 4.82 | 0.33 | 4.4 | 4.4 | 4.4 | 0 |
|  | AREDS 3 | 24 | 4.8 | 0.4 | 4.4 | 4.4 | 4.4 | 0 |
|  | GA | 37 | 5.14 | 0.54 | 4.4 | 4.4 | 4.4 | 0 |
| IL-6 Receptor | control | 33 | 20.52 | 1.1 | 19 | 16 | 23 | 7 |
| (IL-6r; ng/ml) | AREDS 3 | 24 | 22.35 | 1.59 | 22 | 19 | 25.5 | 6.5 |
|  | GA | 37 | 21.97 | 1.21 | 21 | 17 | 26 | 9 |
| IL-6 Receptor | control | 33 | 161.79 | 6.79 | 152 | 143 | 172 | 29 |
| Subunit Beta | AREDS 3 | 24 | 165.46 | 8.25 | 156.5 | 146.25 | 185 | 38.75 |
| (IL-6rbeta; ng/ml) | GA | 37 | 178.08 | 6.39 | 177 | 150 | 207 | 57 |
| IL-7 (pg/ml) | control | 33 | 17 | 0 | 17 | 17 | 17 | 0 |
|  | AREDS 3 | 24 | 17 | 0 | 17 | 17 | 17 | 0 |
|  | GA | 37 | 17.03 | 0.03 | 17 | 17 | 17 | 0 |
| IL-8 (pg/ml) | control | 33 | 7.93 | 1.63 | 4.1 | 4.1 | 8.1 | 4 |
|  | AREDS 3 | 24 | 10 | 3.8 | 4.3 | 4.1 | 6.7 | 2.6 |
|  | GA | 37 | 26.42 | 17.62 | 6.2 | 4.5 | 9.5 | 5 |
| IL-10 (pg/ml) | control | 33 | 4.93 | 0.17 | 4.7 | 4.7 | 4.7 | 0 |
|  | AREDS 3 | 24 | 8.97 | 3.97 | 4.7 | 4.7 | 4.7 | 0 |
|  | GA | 37 | 4.81 | 0.07 | 4.7 | 4.7 | 4.7 | 0 |
| IL-12 Subunit p40 | control | 33 | 0.43 | 0.03 | 0.39 | 0.33 | 0.51 | 0.18 |
| (IL-12p40; ng/ml) | AREDS 3 | 24 | 0.47 | 0.03 | 0.45 | 0.36 | 0.55 | 0.18 |
|  | GA | 37 | 0.41 | 0.02 | 0.41 | 0.29 | 0.49 | 0.2 |
| IL-12 Subunit p70 | control | 33 | 44 | 0 | 44 | 44 | 44 | 0 |
| (IL-12p70; pg/ml) | AREDS 3 | 24 | 44 | 0 | 44 | 44 | 44 | 0 |
|  | GA | 37 | 44 | 0 | 44 | 44 | 44 | 0 |
| IL-13 (pg/ml) | control | 33 | 9.1 | 0 | 9.1 | 9.1 | 9.1 | 0 |
|  | AREDS 3 | 24 | 9.18 | 0.08 | 9.1 | 9.1 | 9.1 | 0 |
|  | GA | 37 | 9.1 | 0 | 9.1 | 9.1 | 9.1 | 0 |
| IL-15 (ng/ml) | control | 33 | 0.71 | 0.03 | 0.67 | 0.55 | 0.81 | 0.26 |
|  | AREDS 3 | 24 | 0.76 | 0.04 | 0.75 | 0.55 | 0.87 | 0.32 |
|  | GA | 37 | 0.69 | 0.03 | 0.67 | 0.55 | 0.79 | 0.24 |
| IL-16 (pg/ml) | control | 33 | 375.88 | 21.12 | 367 | 305 | 407 | 102 |
|  | AREDS 3 | 24 | 565.50 | 117.56 | 383.5 | 327.25 | 574.75 | 247.5 |
|  | GA | 37 | 568.35 | 101.67 | 391 | 323 | 500 | 177 |
| IL-17 (pg/ml) | control | 33 | 3.1 | 0 | 3.1 | 3.1 | 3.1 | 0 |
|  | AREDS 3 | 24 | 3.1 | 0 | 3.1 | 3.1 | 3.1 | 0 |
|  | GA | 37 | 3.1 | 0 | 3.1 | 3.1 | 3.1 | 0 |
| IL-18 (pg/ml) | control | 33 | 195.00 | 16.56 | 184 | 131 | 239 | 108 |
|  | AREDS 3 | 24 | 189.04 | 18.67 | 169.5 | 123 | 243 | 120 |
|  | GA | 37 | 222.41 | 21.2 | 199 | 131 | 264 | 133 |
| IL-18 Binding Protein | control | 33 | 12.51 | 0.98 | 11.0 | 9.0 | 15.0 | 6.0 |
| (IL-18bp; ng/ml) | AREDS 3 | 24 | 14.52 | 1.13 | 14.5 | 9.78 | 16.0 | 6.2 |
|  | GA | 37 | 15.47 | 0.84 | 15.0 | 11.0 | 18.0. | 7.0 |
| IL-22 (ng/ml) | control | 33 | 0.16 | 0.01 | 0.17 | 0.14 | 0.20 | 0.06 |
|  | AREDS 3 | 24 | 0.20 | 0.02 | 0.18 | 0.16 | 0.23 | 0.07 |
|  | GA | 37 | 0.21 | 0.01 | 0.20 | 0.17 | 0.26 | 0.09 |
| IL-23 (ng/ml) | control | 33 | 1.88 | 0.09 | 1.6 | 1.6 | 2.1 | 0.5 |
|  | AREDS 3 | 24 | 1.85 | 0.07 | 1.6 | 1.6 | 2.12 | 0.52 |
|  | GA | 37 | 1.75 | 0.07 | 1.6 | 1.6 | 1.7 | 0.1 |
| IL-31 (ng/ml) | control | 33 | 0.42 | 0 | 0.42 | 0.42 | 0.42 | 0 |
|  | AREDS 3 | 24 | 0.42 | 0 | 0.42 | 0.42 | 0.42 | 0 |
|  | GA | 37 | 0.42 | 0 | 0.42 | 0.42 | 0.42 | 0 |
| Kallikrein-5 (ng/ml) | control | 33 | 2.26 | 0.14 | 2.4 | 1.6 | 2.8 | 1.2 |
|  | AREDS 3 | 24 | 2.27 | 0.29 | 1.75 | 1.4 | 2.8 | 1.4 |
|  | GA | 37 | 2.53 | 0.22 | 2.2 | 1.9 | 3 | 1.1 |
| Kallikrein-5 (pg/ml) | control | 33 | 683 | 0 | 683 | 683 | 683 | 0 |
|  | AREDS 3 | 24 | 683 | 0 | 683 | 683 | 683 | 0 |
|  | GA | 37 | 683 | 0 | 683 | 683 | 683 | 0 |
| Kidney Injury | control | 33 | 0.04 | 0 | 0.03 | 0.03 | 0.04 | 0 |
| Molecule-1 | AREDS 3 | 24 | 0.04 | 0 | 0.03 | 0.03 | 0.04 | 0 |
| (KIM-1; ng/ml) | GA | 37 | 0.05 | 0 | 0.04 | 0.03 | 0.06 | 0.02 |
| Lactoferrin | control | 33 | 35.26 | 4.68 | 24 | 17 | 50 | 33 |
| (LTF; ng/ml) | AREDS 3 | 24 | 39.71 | 5.79 | 29.5 | 23 | 45 | 22 |
|  | GA | 37 | 38.05 | 5.91 | 23 | 18 | 39 | 21 |
| Lactoylglutathione | control | 33 | 45.91 | 7.8 | 34 | 16 | 47 | 31 |
| Lyase | AREDS 3 | 24 | 30.52 | 4.72 | 24 | 13 | 39.75 | 26.75 |
| (LGL; ng/ml) | GA | 37 | 21.88 | 5.01 | 7.6 | 2.7 | 34 | 31.3 |
| Latency Associated | control | 33 | 3.64 | 0.51 | 2.6 | 1.7 | 4.3 | 2.6 |
| Peptide of Trans- | AREDS 3 | 24 | 5.25 | 0.7 | 3.95 | 2.98 | 6.7 | 3.72 |
| forming Growth | GA | 37 | 5.36 | 0.67 | 3.9 | 2 | 6.6 | 4.6 |
| Factor Beta 1  (LPA TGF-b1; ng/ml)  Lectin-Like Oxidized | control | 33 | 1.24 | 0.13 | 0.85 | 0.75 | 1.5 | 0.75 |
| LDL Receptor-1 | AREDS 3 | 24 | 1.35 | 0.23 | 0.8 | 0.75 | 1.35 | 0.6 |
| (LOX-1; ng/ml) | GA | 37 | 1.15 | 0.22 | 0.75 | 0.75 | 0.75 | 0 |
| Leptin (ng/ml) | control | 33 | 19.52 | 2.98 | 13 | 5.9 | 30 | 24.1 |
|  | AREDS 3 | 24 | 14.57 | 1.74 | 13.5 | 8.15 | 19.75 | 11.6 |
|  | GA | 37 | 19.17 | 3.52 | 13 | 4.4 | 28 | 23.6 |
| Leptin Receptor | control | 33 | 17.06 | 0.84 | 16 | 14 | 19 | 5 |
| (Leptin-R; ng/ml) | AREDS 3 | 23 | 20.13 | 1.86 | 16 | 15 | 24 | 9 |
|  | GA | 37 | 17.86 | 0.9 | 17 | 14 | 19 | 5 |
| Leucine-Rich Alpha- | control | 33 | 61.58 | 3.23 | 59 | 45 | 73 | 28 |
| 2 Glycoprotein | AREDS 3 | 24 | 65.62 | 5.19 | 59.5 | 46.75 | 75.5 | 28.75 |
| (LRG1; ug/ml) | GA | 37 | 73.27 | 5.68 | 69 | 55 | 84 | 29 |
| Lumican (ug/ml) | control | 33 | 5.71 | 0.22 | 5.7 | 5.1 | 6.5 | 1.4 |
|  | AREDS 3 | 24 | 6.5 | 0.31 | 6.7 | 5.92 | 7.25 | 1.33 |
|  | GA | 37 | 6.67 | 0.25 | 6.4 | 5.9 | 7.3 | 1.4 |
| Luteinizing Hormone | control | 33 | 8.35 | 0.83 | 6.9 | 4.7 | 10 | 5.3 |
| (LH; mlU/ml) | AREDS 3 | 24 | 9.06 | 0.88 | 7.5 | 6.33 | 11.25 | 4.92 |
|  | GA | 37 | 6.31 | 0.56 | 5.1 | 3.5 | 8.6 | 5.1 |
| Macrophage Colony | control | 33 | 0.6 | 0.04 | 0.63 | 0.38 | 0.77 | 0.39 |
| Stimulating Factor-1 | AREDS 3 | 24 | 0.8 | 0.1 | 0.61 | 0.46 | 1.02 | 0.56 |
| (M-CSF; ng/ml) | GA | 37 | 0.71 | 0.05 | 0.65 | 0.48 | 0.91 | 0.43 |
| Macrophage- | control | 33 | 426.12 | 23.15 | 414.0 | 357 | 508 | 151 |
| Derived Chemokine | AREDS 3 | 24 | 464.54 | 22.07 | 450.5 | 397 | 517.75 | 120.75 |
| (MDC; pg/ml) | GA | 37 | 413.03 | 25.16 | 365 | 347 | 472 | 125 |
| Macrophage In- | control | 33 | 36.06 | 2.22 | 31 | 31 | 33 | 2 |
| flammatory Protein-1 | AREDS 3 | 24 | 39.62 | 4.75 | 31 | 31 | 36.25 | 5.25 |
| (MIP-1alpha; pg/ml) | GA | 37 | 60.78 | 18.61 | 31 | 31 | 35 | 4 |
| MIP-1β (pg/ml) | control | 33 | 136.73 | 14.97 | 112 | 84 | 154 | 70 |
|  | AREDS 3 | 24 | 197.62 | 34.72 | 139.5 | 127.25 | 183.5 | 56.25 |
|  | GA | 37 | 402.05 | 120.63 | 175 | 148 | 276 | 128 |
| MIP-3alpha (pg/ml) | control | 33 | 36.76 | 2.63 | 32 | 32 | 32 | 0 |
|  | AREDS 3 | 24 | 52.58 | 13.92 | 32 | 32 | 32 | 0 |
|  | GA | 37 | 62.08 | 19.84 | 32 | 32 | 32 | 0 |
| MIP-3beta (pg/ml) | control | 33 | 310.36 | 23.24 | 272 | 210 | 355 | 145 |
|  | AREDS 3 | 24 | 259.58 | 23.92 | 238.5 | 180 | 297.75 | 117.75 |
|  | GA | 37 | 373.86 | 71.74 | 272 | 217 | 386 | 169 |
| Macrophage | control | 33 | 0.4 | 0.08 | 0.22 | 0.16 | 0.45 | 0.29 |
| Migration Inhibitory | AREDS 3 | 24 | 0.42 | 0.1 | 0.18 | 0.13 | 0.58 | 0.46 |
| Factor | GA | 37 | 0.34 | 0.05 | 0.24 | 0.12 | 0.45 | 0.33 |
| (MIF; ng/ml)  Macrophage | control | 33 | 190.12 | 13.96 | 177 | 142 | 223 | 81 |
| Stimulating Protein | AREDS 3 | 24 | 152.83 | 16.39 | 140 | 107.75 | 187.75 | 80 |
| (MSP; ng/ml) | GA | 37 | 199.49 | 24.31 | 167 | 126 | 217 | 91 |
| Maspin (ug/ml) | control | 33 | 3.79 | 0 | 3.79 | 3.79 | 3.79 | 0 |
|  | AREDS 3 | 24 | 3.79 | 0 | 3.79 | 3.79 | 3.79 | 0 |
|  | GA | 37 | 3.79 | 0 | 3.79 | 3.79 | 3.79 | 0 |
| Mast/Stem Cell | control | 33 | 7.01 | 0.27 | 6.8 | 6 | 7.7 | 1.7 |
| Growth Factor | AREDS 3 | 24 | 7.83 | 0.48 | 7.1 | 6.27 | 8.3 | 2.03 |
| Receptor | GA | 37 | 7.8 | 0.3 | 7.5 | 6.5 | 9.3 | 2.8 |
| (SCFR; ng/ml)  Matrix Metallo- | control | 33 | 3.19 | 0.84 | 1.8 | 1.7 | 2.6 | 0.9 |
| proteinase-1 | AREDS 3 | 24 | 5.26 | 1.4 | 1.8 | 1.7 | 3.8 | 2.1 |
| (MMP-1; ng/ml) | GA | 37 | 4.43 | 1.2 | 1.7 | 1.7 | 3.5 | 1.8 |
| MMP-2 (ug/ml) | control | 33 | 1.32 | 0.034 | 0.13 | 1.15 | 1.44 | 0.29 |
|  | AREDS 3 | 24 | 1.41 | 0.067 | 1.34 | 1.20 | 1.57 | 0.37 |
|  | GA | 37 | 1.35 | 0.061 | 1.34 | 1.14 | 1.56 | 0.42 |
| MMP-3 (ng/ml) | control | 33 | 7.52 | 0.5 | 7.3 | 5.8 | 9 | 3.2 |
|  | AREDS 3 | 24 | 8.77 | 0.84 | 7.35 | 6.25 | 11 | 4.75 |
|  | GA | 37 | 9.2 | 1.46 | 6.4 | 4.1 | 10 | 5.9 |
| MMP-7 (ng/ml) | control | 33 | 4.22 | 0.36 | 3.7 | 2.8 | 4.6 | 1.8 |
|  | AREDS 3 | 24 | 4.94 | 0.42 | 4.35 | 3.8 | 5.73 | 1.93 |
|  | GA | 37 | 2.73 | 0.51 | 0.55 | 0.22 | 4.8 | 4.58 |
| MMP-9 (ng/ml) | control | 33 | 58.64 | 4.20 | 56 | 39 | 73 | 34 |
|  | AREDS 3 | 24 | 58.00 | 5.31 | 57 | 40 | 74.5 | 34.5 |
|  | GA | 37 | 108.35 | 16.02 | 79 | 54 | 100 | 46 |
| MMP-9 Total (ng/ml) | control | 33 | 747.79 | 65.33 | 662 | 499 | 1060 | 561 |
|  | AREDS 3 | 24 | 723.96 | 81.12 | 640 | 411.25 | 1072.5 | 661.25 |
|  | GA | 37 | 627.05 | 72.35 | 427 | 324 | 852 | 528 |
| MMP-10 (ng/ml) | control | 33 | 1.12 | 0.09 | 0.96 | 0.83 | 1.2 | 0.37 |
|  | AREDS 3 | 24 | 1.33 | 0.26 | 0.98 | 0.79 | 1.33 | 0.54 |
|  | GA | 37 | 0.72 | 0.13 | 0.16 | 0.11 | 1.2 | 1.09 |
| Mesothelin | control | 33 | 51.52 | 5.03 | 43 | 36 | 53 | 17 |
| (MSLN; nM) | AREDS 3 | 24 | 59.08 | 5.36 | 53 | 40.5 | 74.5 | 34 |
|  | GA | 37 | 66.76 | 5.77 | 65 | 41 | 81 | 40 |
| MHC Class I Chain- | control | 33 | 81.79 | 1.79 | 80 | 80 | 80 | 0 |
| Related Protein A | AREDS 3 | 24 | 86.46 | 4.25 | 80 | 80 | 80 | 0 |
| (MICA; pg/ml) | GA | 37 | 104.65 | 9.22 | 80 | 80 | 98 | 18 |
| Monocyte Chemo- | control | 33 | 196.94 | 17.15 | 179 | 130 | 224 | 94 |
| tactic Protein-1 | AREDS 3 | 24 | 212.12 | 31.93 | 144 | 99 | 293.5 | 194.5 |
| (MCP-1; pg/ml) | GA | 37 | 197.16 | 18.68 | 170 | 123 | 224 | 101 |
| MCP-2 (pg/ml) | control | 33 | 28.64 | 1.66 | 27 | 22 | 35 | 13 |
|  | AREDS 3 | 24 | 39.42 | 10.94 | 26 | 21 | 34.75 | 13.75 |
|  | GA | 37 | 41.97 | 8.62 | 32 | 25 | 41 | 16 |
| MCP-3 (pg/ml) | control | 33 | 3.3 | 0 | 3.3 | 3.3 | 3.3 | 0 |
|  | AREDS 3 | 24 | 3.3 | 0 | 3.3 | 3.3 | 3.3 | 0 |
|  | GA | 37 | 3.3 | 0 | 3.3 | 3.3 | 3.3 | 0 |
| MCP-4 (ug/ml) | control | 33 | 2.18 | 0.21 | 1.92 | 1.45 | 2.36 | 0.91 |
|  | AREDS 3 | 24 | 3.10 | 0.65 | 1.69 | 1.55 | 3.13 | 1.59 |
|  | GA | 37 | 3.12 | 0.36 | 2.32 | 1.83 | 3.74 | 1.91 |
| Monokine Induced | control | 33 | 0.78 | 0.10 | 0.65 | 0.30 | 0.96 | 0.66 |
| by Gamma Interferon | AREDS 3 | 24 | 1.42 | 0.36 | 0.82 | 0.54 | 1.49 | 0.95 |
| (MIG; ug/ml) | GA | 37 | 1.54 | 0.20 | 1.26 | 0.76 | 2.01 | 1.25 |
| Myeloid Progenitor | control | 33 | 1.1 | 0.06 | 1 | 0.83 | 1.3 | 0.47 |
| Inhibitory Factor 1 | AREDS 3 | 24 | 1.32 | 0.11 | 1.25 | 0.99 | 1.4 | 0.41 |
| (MPIF-1; ng/ml) | GA | 37 | 1.46 | 0.14 | 1.3 | 1.1 | 1.7 | 0.6 |
| Myeloperoxidase | control | 33 | 425.09 | 57.38 | 372 | 238 | 485 | 247 |
| (MPO; ng/ml) | AREDS 3 | 24 | 690.38 | 201.93 | 340 | 238 | 557 | 319 |
|  | GA | 37 | 690.35 | 186.58 | 306 | 204 | 622 | 418 |
| Myoglobin (ng/ml) | control | 33 | 30.37 | 4.63 | 25 | 20 | 30 | 10 |
|  | AREDS 3 | 24 | 34.29 | 3.18 | 30.5 | 24.5 | 40 | 15.5 |
|  | GA | 37 | 34.07 | 4.34 | 28 | 19 | 42 | 23 |
| N-terminal pro- | control | 33 | 0.44 | 0.08 | 0.33 | 0.17 | 0.45 | 0.28 |
| Hormone of brain | AREDS 3 | 24 | 1.08 | 0.18 | 0.83 | 0.46 | 1.24 | 0.78 |
| Natriuretic Peptide | GA | 37 | 1.24 | 0.23 | 0.61 | 0.26 | 1.84 | 1.58 |
| (NTproBNP; ug/ml)  Nerve Growth | control | 33 | 0.05 | 0 | 0.04 | 0.04 | 0.04 | 0 |
| Factor-beta | AREDS 3 | 24 | 0.05 | 0 | 0.04 | 0.04 | 0.04 | 0 |
| (NGF-beta; ng/ml) | GA | 37 | 0.05 | 0 | 0.04 | 0.04 | 0.04 | 0 |
| Neurofilament | control | 33 | 118.18 | 6.05 | 109 | 109 | 109 | 0 |
| Heavy Polypeptide | AREDS 3 | 24 | 174.67 | 36.22 | 110 | 109 | 170 | 61 |
| (NF-H; pg/ml) | GA | 37 | 155.46 | 21.75 | 109 | 109 | 122 | 13 |
| Neuron-Specific | control | 33 | 4.41 | 0.48 | 3.1 | 2.4 | 6.1 | 3.7 |
| Enolase | AREDS 3 | 24 | 4.66 | 0.51 | 4.15 | 2.6 | 5.93 | 3.33 |
| (NSE; ng/ml) | GA | 37 | 5.28 | 0.59 | 4.4 | 3.2 | 6 | 2.8 |
| Neuronal Cell | control | 33 | 0.65 | 0.05 | 0.58 | 0.46 | 0.7 | 0.24 |
| Adhesion Molecule | AREDS 3 | 24 | 0.79 | 0.06 | 0.72 | 0.56 | 0.99 | 0.43 |
| (Nr-CAM; ng/ml) | GA | 37 | 1.11 | 0.09 | 1.1 | 0.76 | 1.4 | 0.64 |
| Neuropilin-1 (ng/ml) | control | 33 | 151.24 | 4.49 | 152 | 131 | 167 | 36 |
|  | AREDS 3 | 24 | 164.29 | 9.15 | 157.5 | 138.5 | 179.5 | 41 |
|  | GA | 37 | 168.89 | 5.54 | 174 | 148 | 189 | 41 |
| Neutrophil Activating | control | 33 | 1.08 | 0.36 | 0.43 | 0.29 | 0.84 | 0.56 |
| Peptide 2 | AREDS 3 | 24 | 1.83 | 0.53 | 0.79 | 0.51 | 1.34 | 0.83 |
| (NAP-2; ug/ml) | GA | 37 | 1.70 | 0.37 | 0.81 | 0.36 | 1.82 | 1.47 |
| Neutrophil Gelanti- | control | 33 | 224.73 | 13.26 | 202 | 169 | 263 | 94 |
| nase Associated | AREDS 3 | 24 | 302.42 | 42.27 | 235 | 158.75 | 369.25 | 210.5 |
| Lipocalin | GA | 37 | 254.32 | 38.89 | 177 | 146 | 239 | 93 |
| (NGAL; ng/ml)  Omentin (ng/ml) | control | 33 | 129.18 | 9.15 | 118 | 99 | 151 | 52 |
|  | AREDS 3 | 23 | 152.78 | 11.04 | 142 | 116.5 | 191.5 | 75 |
|  | GA | 37 | 364.65 | 40.74 | 288 | 157 | 531 | 374 |
| Osteocalcin (ng/ml) | control | 33 | 23.23 | 1.5 | 23 | 18 | 28 | 10 |
|  | AREDS 3 | 24 | 35.58 | 4.11 | 33 | 20.75 | 43 | 22.25 |
|  | GA | 37 | 39.49 | 2.79 | 41 | 27 | 50 | 23 |
| Osteopontin (ng/ml) | control | 33 | 19.27 | 1.3 | 17 | 14 | 26 | 12 |
|  | AREDS 3 | 24 | 23.34 | 2.42 | 22 | 15.75 | 27.75 | 12 |
|  | GA | 37 | 29.92 | 2.05 | 28 | 23 | 38 | 15 |
| Osteoprotegerin | control | 33 | 4.72 | 0.32 | 4.5 | 3.4 | 5.7 | 2.3 |
| (OPG; ng/ml) | AREDS 3 | 24 | 6.53 | 0.62 | 5.4 | 4.8 | 7.42 | 2.62 |
|  | GA | 37 | 6.92 | 0.42 | 6.8 | 5.2 | 8.8 | 3.6 |
| P-Selectin (ng/ml) | control | 33 | 69.82 | 5.99 | 58 | 44 | 79 | 35 |
|  | AREDS 3 | 24 | 89.21 | 12.02 | 74.5 | 57.75 | 90.25 | 32.5 |
|  | GA | 37 | 83.73 | 8.99 | 66 | 57 | 89 | 32 |
| Pancreatic Poly- | control | 33 | 181.85 | 40.99 | 108 | 67 | 221 | 154 |
| peptide | AREDS 3 | 24 | 185.79 | 27.02 | 147.5 | 92 | 217 | 125 |
| (PPP; pg/ml) | GA | 37 | 258.97 | 44.86 | 146 | 78 | 354 | 276 |
| Pancreatic Secretory | control | 33 | 9.97 | 0.85 | 9 | 7 | 11 | 4 |
| Trypsin Inhibitor | AREDS 3 | 24 | 12.6 | 1.44 | 9.75 | 7.68 | 15 | 7.32 |
| (TATI; ng/ml) | GA | 37 | 17.5 | 2.82 | 12 | 8.8 | 18 | 9.2 |
| Paraoxonase-1 | control | 33 | 3.09 | 0.12 | 3.09 | 2.59 | 3.55 | 0.96 |
| (Pon1; ug/ml) | AREDS 3 | 23 | 2.81 | 0.206 | 2.96 | 2.07 | 3.60 | 1.53 |
|  | GA | 37 | 5.21 | 0.45 | 4.60 | 3.09 | 7.34 | 4.25 |
| Pentraxin-3 (ng/ml) | control | 33 | 0.61 | 0.05 | 0.62 | 0.32 | 0.81 | 0.49 |
|  | AREDS 3 | 23 | 0.73 | 0.1 | 0.7 | 0.39 | 0.94 | 0.56 |
|  | GA | 37 | 1.55 | 0.23 | 1.1 | 0.75 | 1.8 | 1.05 |
| Pepsinogen I | control | 33 | 108.82 | 14.99 | 81 | 45 | 134 | 89 |
| (PGI; ng/ml) | AREDS 3 | 24 | 141.12 | 20.58 | 111.5 | 80.25 | 179.75 | 99.5 |
|  | GA | 37 | 125.19 | 15.92 | 96 | 64 | 169 | 105 |
| Pepdidase D | control | 33 | 15.55 | 0.61 | 15 | 13 | 17 | 4 |
| (PEPD; ug/ml) | AREDS 3 | 24 | 17.42 | 1.1 | 16 | 14 | 19.5 | 5.5 |
|  | GA | 37 | 16.62 | 0.7 | 15 | 13 | 19 | 6 |
| Peptide YY | control | 33 | 315.58 | 34.73 | 217 | 210 | 302 | 92 |
| (PYY; pg/ml) | AREDS 3 | 24 | 277.88 | 21.54 | 217 | 210 | 309.25 | 99.25 |
|  | GA | 37 | 358.32 | 48.06 | 245 | 217 | 355 | 138 |
| Periostin (ng/ml) | control | 33 | 99.21 | 5.21 | 104 | 77 | 116 | 39 |
|  | AREDS 3 | 24 | 125.25 | 18.85 | 100.5 | 78.75 | 142.5 | 63.75 |
|  | GA | 37 | 147.49 | 14.58 | 108 | 92 | 182 | 90 |
| Phosphserine | control | 33 | 2.17 | 0.14 | 2.1 | 1.5 | 2.3 | 0.8 |
| Aminotransferase | AREDS 3 | 24 | 2.55 | 0.39 | 2.1 | 1.4 | 2.62 | 1.23 |
| (PSAT; ng/ml) | GA | 37 | 2.95 | 0.29 | 2.4 | 1.7 | 3.5 | 1.8 |
| Pigment Epithelial | control | 33 | 4.68 | 0.23 | 4.50 | 3.67 | 5.50 | 1.83 |
| Derived Factor | AREDS 3 | 24 | 4.84 | 0.23 | 4.76 | 4.24 | 5.22 | 0.98 |
| (PEDF; ug/ml) | GA | 37 | 5.34 | 0.26 | 5.09 | 4.55 | 5.94 | 1.39 |
| Placenta Growth | control | 33 | 29 | 0 | 29 | 29 | 29 | 0 |
| Factor | AREDS 3 | 24 | 29.29 | 0.29 | 29 | 29 | 29 | 0 |
| (PLGF; pg/ml) | GA | 37 | 29.43 | 0.26 | 29 | 29 | 29 | 0 |
| Plasminogen | control | 33 | 33.91 | 7.08 | 22 | 14 | 28 | 14 |
| Activator Inhibitor | AREDS 3 | 24 | 51.59 | 11.54 | 31.5 | 18.75 | 53.25 | 34.5 |
| (PAI-1; ng/ml) | GA | 37 | 53.37 | 10.15 | 41 | 16 | 55 | 39 |
| Platelet Endothelial | control | 33 | 57.12 | 2.19 | 56 | 48 | 63 | 15 |
| Cell Adhesion | AREDS 3 | 24 | 57.17 | 3.36 | 54.5 | 46.25 | 64.25 | 18 |
| Molecule 1 | GA | 37 | 58.81 | 2.57 | 58 | 47 | 71 | 24 |
| (PECAM-1; ng/ml)  Platelet-Derived | control | 33 | 1.90 | 0.88 | 0.43 | 0.26 | 1.05 | 0.79 |
| Growth Factor BB | AREDS 3 | 24 | 2.86 | 0.92 | 0.91 | 0.47 | 2.11 | 1.64 |
| (PDGF-BB; ng/ml) | GA | 37 | 2.59 | 0.73 | 0.65 | 0.15 | 2.60 | 2.45 |
| Progesterone (ng/ml) | control | 33 | 6.84 | 0.16 | 6.5 | 6.5 | 6.5 | 0 |
|  | AREDS 3 | 24 | 7.22 | 0.43 | 6.5 | 6.5 | 6.7 | 0.2 |
|  | GA | 37 | 7.56 | 0.47 | 6.5 | 6.5 | 7.2 | 0.7 |
| Progranulin (ng/ml) | control | 33 | 11.25 | 1 | 10 | 7.7 | 13 | 5.3 |
|  | AREDS 3 | 24 | 13.18 | 1.05 | 13 | 8.7 | 16.25 | 7.55 |
|  | GA | 37 | 10.58 | 0.63 | 11 | 7.2 | 13 | 5.8 |
| Proinsulin Intact | control | 33 | 11.12 | 0.12 | 11 | 11 | 11 | 0 |
| (pM) | AREDS 3 | 24 | 11.88 | 0.88 | 11 | 11 | 11 | 0 |
|  | GA | 37 | 11.7 | 0.65 | 11 | 11 | 11 | 0 |
| Proinsulin Total | control | 33 | 42.36 | 0.36 | 42 | 42 | 42 | 0 |
| (pM) | AREDS 3 | 24 | 45.96 | 3.96 | 42 | 42 | 42 | 0 |
|  | GA | 37 | 45.03 | 3.03 | 42 | 42 | 42 | 0 |
| Prolactin (ng/ml) | control | 33 | 4.18 | 0.31 | 3.9 | 2.9 | 4.9 | 2 |
|  | AREDS 3 | 24 | 5.00 | 0.48 | 4.2 | 3.48 | 6.1 | 2.6 |
|  | GA | 37 | 4.58 | 0.31 | 4.1 | 3.3 | 5.5 | 2.2 |
| Prostasin (ng/ml) | control | 33 | 197.82 | 12.81 | 176 | 151 | 234 | 83 |
|  | AREDS 3 | 24 | 197.54 | 13.53 | 187.5 | 154 | 218.75 | 64.75 |
|  | GA | 37 | 242.84 | 16.69 | 233 | 165 | 302 | 137 |
| Prostate-Specific | control | 33 | 0.1 | 0.02 | 0.02 | 0.02 | 0.14 | 0.12 |
| Antigen, Free | AREDS 3 | 24 | 0.12 | 0.05 | 0.02 | 0.02 | 0.02 | 0 |
| (PSA-f; ng/ml) | GA | 37 | 0.24 | 0.09 | 0.02 | 0.02 | 0.11 | 0.09 |
| Prostate-Specific | control | 33 | 0.72 | 0.19 | 0.1 | 0.1 | 1.1 | 1 |
| Antigen, Total | AREDS 3 | 24 | 0.94 | 0.41 | 0.1 | 0.1 | 0.1 | 0 |
| (tPSA; ng/ml) | GA | 37 | 2.86 | 1.4 | 0.1 | 0.1 | 0.88 | 0.78 |
| Protein DJ-1 | control | 33 | 139.27 | 22.40 | 100 | 62 | 152 | 90 |
| (DJ-1; ng/ml) | AREDS 3 | 24 | 135.00 | 24.24 | 109 | 70.75 | 152.25 | 81.5 |
|  | GA | 37 | 162.22 | 30.19 | 97 | 47 | 163 | 116 |
| Protein S100 A4 | control | 33 | 58.03 | 12.16 | 29 | 16 | 69 | 53 |
| (ng/ml) | AREDS 3 | 24 | 53.17 | 16.22 | 18.5 | 16 | 38.25 | 22.25 |
|  | GA | 37 | 41.78 | 10.68 | 16 | 16 | 23 | 7 |
| Pulmonary and | control | 33 | 107.06 | 6.73 | 103 | 83 | 131 | 48 |
| Activation-Regulated | AREDS 3 | 24 | 118.96 | 16.73 | 104 | 73.75 | 126.5 | 52.75 |
| Chemokine | GA | 37 | 128.95 | 9.38 | 133 | 73 | 166 | 93 |
| (PARC; ng/ml)  Pulmonayr Surfac- | control | 33 | 10.88 | 0.98 | 9.9 | 6.7 | 13 | 6.3 |
| tant-Associated | AREDS 3 | 24 | 10.28 | 1.01 | 10 | 6.73 | 13 | 6.27 |
| Protein D | GA | 37 | 11.89 | 2.15 | 6.7 | 4.6 | 12 | 7.4 |
| (SP-D; ng/ml)  Receptor for | control | 33 | 3.13 | 0.43 | 2.4 | 1.8 | 3.4 | 1.6 |
| Advanced Glycosyl- | AREDS 3 | 24 | 3.1 | 0.34 | 2.85 | 1.67 | 4.1 | 2.42 |
| ation End Products | GA | 37 | 3.3 | 0.3 | 3 | 1.8 | 4.3 | 2.5 |
| (RAGE; ng/ml)  Receptor Tyrosine- | control | 33 | 0.29 | 0.04 | 0.23 | 0.14 | 0.39 | 0.25 |
| Protein Kinase | AREDS 3 | 24 | 0.26 | 0.03 | 0.22 | 0.18 | 0.29 | 0.11 |
| (ErbB3; ng/ml) | GA | 37 | 0.3 | 0.05 | 0.21 | 0.16 | 0.32 | 0.16 |
| Resistin (ng/ml) | control | 33 | 2.04 | 0.12 | 1.9 | 1.6 | 2.4 | 0.8 |
|  | AREDS 3 | 24 | 2.54 | 0.2 | 2.4 | 1.85 | 3.12 | 1.28 |
|  | GA | 37 | 2.82 | 0.21 | 2.5 | 1.9 | 3.5 | 1.6 |
| Retinol-Binding | control | 33 | 31.91 | 1.67 | 30 | 25 | 37 | 12 |
| Protein-4 | AREDS 3 | 24 | 35.04 | 2.35 | 35.5 | 26.75 | 40 | 13.25 |
| (RBP-4; ug/ml) | GA | 37 | 37.27 | 1.96 | 38 | 29 | 42 | 13 |
| S100 Calcium- | control | 33 | 0.33 | 0.02 | 0.28 | 0.23 | 0.37 | 0.14 |
| Binding Protein B | AREDS 3 | 24 | 0.36 | 0.03 | 0.34 | 0.23 | 0.45 | 0.22 |
| (S100-B; ng/ml) | GA | 37 | 0.26 | 0.01 | 0.23 | 0.23 | 0.23 | 0 |
| Selenoprotein P | control | 33 | 5.25 | 0.17 | 5.01 | 4.48 | 5.64 | 1.16 |
| (ug/ml) | AREDS 3 | 24 | 5.91 | 0.26 | 5.86 | 5.27 | 6.14 | 0.87 |
|  | GA | 37 | 5.80 | 0.19 | 5.65 | 5.09 | 6.46 | 1.37 |
| Serotransferrin | control | 33 | 207.12 | 6.76 | 210 | 181 | 228 | 47 |
| (Transferrin; mg/dl) | AREDS 3 | 24 | 207.17 | 9.29 | 201.5 | 167.5 | 238 | 70.5 |
|  | GA | 37 | 229.92 | 8.98 | 225 | 182 | 272 | 90 |
| Serum Amyloid A | control | 32 | 13.72 | 5.31 | 4.90 | 2.55 | 10.45 | 7.90 |
| (SAA; ug/ml) | AREDS 3 | 24 | 7.77 | 1.45 | 5.61 | 2.99 | 9.86 | 6.88 |
|  | GA | 36 | 8.52 | 3.86 | 3.52 | 2.17 | 5.99 | 3.82 |
| Serum Amyloid P | control | 33 | 14.18 | 0.73 | 13 | 11 | 17 | 6 |
| (SAP; ug/ml) | AREDS 3 | 24 | 11.88 | 0.78 | 11 | 9.23 | 14 | 4.77 |
|  | GA | 37 | 13.16 | 0.71 | 13 | 10 | 16 | 6 |
| Sex Hormone | control | 33 | 42.42 | 2.7 | 39 | 31 | 56 | 25 |
| Binding Globulin | AREDS 3 | 24 | 59.21 | 4.64 | 58 | 37.75 | 71.75 | 34 |
| (SHBG; nM) | GA | 37 | 50.51 | 4.02 | 48 | 38 | 57 | 19 |
| Sortilin (ng/ml) | control | 33 | 3.87 | 0.49 | 3.1 | 2.6 | 3.7 | 1.1 |
|  | AREDS 3 | 24 | 4.85 | 0.73 | 3.8 | 3.1 | 4.4 | 1.3 |
|  | GA | 37 | 4.65 | 0.38 | 4.3 | 3.2 | 5 | 1.8 |
| Squamous Cell | control | 33 | 0.60 | 0.03 | 0.52 | 0.52 | 0.60 | 0.08 |
| Carcinoma Antigen-1 | AREDS 3 | 24 | 0.65 | 0.06 | 0.52 | 0.52 | 0.60 | 0.08 |
| (SCCA-1; ng/ml) | GA | 37 | 0.68 | 0.03 | 0.58 | 0.52 | 0.78 | 0.26 |
| ST2 (ng/ml) | control | 33 | 4.05 | 0.24 | 3.5 | 3.1 | 4.8 | 1.7 |
|  | AREDS 3 | 24 | 4.55 | 0.32 | 4.2 | 3.65 | 5.33 | 1.68 |
|  | GA | 37 | 7.07 | 0.46 | 7.0 | 5.0 | 8.5 | 3.5 |
| Stem Cell Factor | control | 33 | 390.0 | 19.48 | 375 | 337 | 467 | 130 |
| (SCF; pg/ml) | AREDS 3 | 24 | 424.5 | 21.47 | 412 | 370 | 472 | 102 |
|  | GA | 37 | 453.0 | 23.48 | 405 | 344 | 533 | 189 |
| Stromal Cell-Derived | control | 33 | 2.57 | 0.096 | 2.44 | 2.31 | 2.80 | 0.49 |
| Factor-1 | AREDS 3 | 24 | 3.08 | 9.12 | 3.07 | 2.61 | 3.33 | 0.72 |
| (SDF-1; ug/ml) | GA | 37 | 2.85 | 0.12 | 2.84 | 2.50 | 3.08 | 0.58 |
| Superoxide Dismu- | control | 33 | 45.79 | 5.18 | 38 | 23 | 54 | 31 |
| Tase 1, soluble | AREDS 3 | 24 | 38.79 | 4.68 | 31 | 20.25 | 58.75 | 38.5 |
| (SOD-1; ng/ml) | GA | 37 | 40.89 | 3.91 | 38 | 25 | 56 | 31 |
| T-Cell Specific | control | 33 | 4.03 | 0.91 | 1.8 | 0.92 | 4 | 3.08 |
| Protein, RANTES | AREDS 3 | 24 | 7.38 | 1.18 | 6.4 | 2.32 | 11.25 | 8.93 |
| (RANTES; ng/ml) | GA | 37 | 11.41 | 1.78 | 7.4 | 2 | 15 | 13 |
| T Lymphocyte- | control | 33 | 256.24 | 114.93 | 99 | 99 | 148 | 49 |
| Secreted Protein | AREDS 3 | 24 | 567.54 | 196.04 | 103 | 99 | 517 | 418 |
| (I-309; pg/ml) | GA | 37 | 196.59 | 40.8 | 121 | 99 | 149 | 50 |
| Tamm-Horsfall | control | 33 | 0.04 | 0 | 0.04 | 0.03 | 0.05 | 0.02 |
| Urinary Glycoprotein | AREDS 3 | 24 | 0.05 | 0 | 0.05 | 0.03 | 0.06 | 0.02 |
| (THP; ug/ml) | GA | 37 | 0.03 | 0 | 0.03 | 0.02 | 0.05 | 0.02 |
| Tenascin-C | control | 33 | 342.85 | 32.53 | 303 | 235 | 414 | 179 |
| (TN-C; ng/ml) | AREDS 3 | 24 | 566.71 | 99.99 | 482 | 327 | 636.5 | 309.5 |
|  | GA | 37 | 466.57 | 58.96 | 375 | 249 | 585 | 336 |
| Tenascin-X | control | 33 | 83.03 | 7.73 | 72 | 60 | 101 | 41 |
| (TN-X; ng/ml) | AREDS 3 | 24 | 83.75 | 8.27 | 76 | 58.25 | 100.75 | 42.5 |
|  | GA | 37 | 101.86 | 9.52 | 96 | 49 | 150 | 101 |
| Testosterone Total | control | 33 | 1.69 | 0.16 | 1 | 1 | 2.4 | 1.4 |
| (testosterone.total; | AREDS 3 | 24 | 1.57 | 0.25 | 1 | 1 | 1.57 | 0.57 |
| ng/ml) | GA | 37 | 1.89 | 0.17 | 1.4 | 1 | 2.7 | 1.7 |
| Tetranectin (ug/ml) | control | 33 | 13.14 | 0.53 | 13 | 11 | 15 | 4 |
|  | AREDS 3 | 24 | 14.65 | 0.64 | 14 | 13.75 | 17 | 3.25 |
|  | GA | 37 | 13.57 | 0.59 | 12 | 11 | 17 | 6 |
| Thrombin Activatable | control | 33 | 8.42 | 0.3 | 8.1 | 7 | 9.5 | 2.5 |
| Fibrinolysis-1 | AREDS 3 | 24 | 8.43 | 0.29 | 8.3 | 7.45 | 9 | 1.55 |
| (TAF-1; ug/ml) | GA | 37 | 9.07 | 0.29 | 9.5 | 8.2 | 10 | 1.8 |
| Thrombomodulin | control | 33 | 3.95 | 0.19 | 3.9 | 3.2 | 4.4 | 1.2 |
| (TM; ng/ml) | AREDS 3 | 24 | 5.1 | 0.3 | 4.95 | 4.3 | 5.43 | 1.13 |
|  | GA | 37 | 4.59 | 0.21 | 4.4 | 3.6 | 5.3 | 1.7 |
| Thrombospondin-1 | control | 33 | 2.66 | 0.59 | 1.46 | 1.00 | 3.05 | 2.02 |
| (TSP-1; mg/ml) | AREDS 3 | 24 | 4.00 | 0.74 | 2.64 | 2.12 | 3.39 | 1.27 |
|  | GA | 37 | 3.99 | 0.72 | 2.65 | 1.05 | 4.58 | 3.53 |
| Thrombospondin-4 | control | 33 | 3.8 | 0.28 | 3.5 | 2.9 | 4.5 | 1.6 |
| (TSP-4; ug/ml) | AREDS 3 | 24 | 6.15 | 0.52 | 6.25 | 4.62 | 7.4 | 2.78 |
|  | GA | 37 | 5.6 | 0.51 | 4.8 | 3.6 | 7 | 3.4 |
| Thymus & Activation | control | 33 | 0.10 | 0.03 | 0.06 | 0.04 | 0.08 | 0.04 |
| Related Chemokine | AREDS 3 | 24 | 0.21 | 0.08 | 0.07 | 0.06 | 0.13 | 0.07 |
| (TARC; ng/ml) | GA | 37 | 0.16 | 0.03 | 0.10 | 0.06 | 0.17 | 0.11 |
| Thymus Expressed | control | 33 | 237.00 | 30.86 | 171 | 99 | 404 | 305 |
| Chemokine | AREDS 3 | 24 | 269.17 | 82.14 | 169.5 | 99 | 250.75 | 151.75 |
| (TECK; pg/ml) | GA | 37 | 253.86 | 27.09 | 211 | 125 | 358 | 233 |
| Thyroglobulin | control | 33 | 17 | 0 | 17 | 17 | 17 | 0 |
| (TG; ng/ml) | AREDS 3 | 24 | 17 | 0 | 17 | 17 | 17 | 0 |
|  | GA | 37 | 35.76 | 5.15 | 17 | 17 | 38 | 21 |
| Thyroid Stimulating | control | 33 | 1.61 | 0.15 | 1.6 | 0.98 | 2 | 1.02 |
| Hormone | AREDS 3 | 24 | 2.6 | 0.94 | 1.55 | 1.2 | 2.08 | 0.88 |
| (TSH; ulU/ml) | GA | 37 | 2.19 | 0.16 | 2.1 | 1.5 | 2.8 | 1.3 |
| Thryoxine-Binding | control | 33 | 29.12 | 1.23 | 30 | 23 | 33 | 10 |
| Globulin | AREDS 3 | 24 | 30.58 | 1.63 | 30 | 24.75 | 36.25 | 11.5 |
| (TBG; ug/ml) | GA | 37 | 34.68 | 1.77 | 35 | 26 | 42 | 16 |
| Tissue Inhibitor of | control | 33 | 66.85 | 4.97 | 59 | 53 | 65 | 12 |
| Metalloproteinases-1 | AREDS 3 | 24 | 82.71 | 7.86 | 72 | 59 | 88 | 29 |
| (TIMP1; ng/ml) | GA | 37 | 87.24 | 6.18 | 79 | 64 | 95 | 31 |
| TIMP2 (ng/ml) | control | 33 | 61.94 | 1.51 | 59 | 57 | 65 | 8 |
|  | AREDS 3 | 24 | 67.62 | 3.04 | 63.5 | 57.75 | 76.25 | 18.5 |
|  | GA | 37 | 69.19 | 2.06 | 71 | 62 | 76 | 14 |
| TIMP3 (ng/ml) | control | 33 | 7.71 | 1.5 | 5.5 | 3.5 | 7.5 | 4 |
|  | AREDS 3 | 24 | 10.58 | 2.1 | 7.95 | 5.8 | 9.2 | 3.4 |
|  | GA | 37 | 9.57 | 1.31 | 6.8 | 4 | 12 | 8 |
| Tissue Plasminogen | control | 33 | 0.95 | 0.08 | 0.84 | 0.76 | 0.88 | 0.12 |
| Activator | AREDS 3 | 24 | 0.97 | 0.06 | 0.88 | 0.8 | 0.95 | 0.15 |
| (tPA; ng/ml) | GA | 37 | 1.48 | 0.12 | 1.3 | 0.9 | 1.9 | 1 |
| TNF-Related | control | 33 | 13.77 | 0.93 | 13 | 11 | 15 | 4 |
| Apoptosis Inducing | AREDS 3 | 24 | 16.41 | 1.64 | 14.5 | 10.75 | 20 | 9.25 |
| Ligand Receptor-3 | GA | 37 | 15.91 | 1.55 | 14 | 11 | 20 | 9 |
| (TRAIL-R3; ng/ml)  Transferrin | control | 33 | 2.36 | 0.1 | 2.4 | 1.90 | 2.7 | 0.80 |
| Receptor Protein 1 | AREDS 3 | 24 | 2.46 | 0.12 | 2.4 | 2.08 | 2.6 | 0.52 |
| (TFR1; ug/ml) | GA | 37 | 2.64 | 0.12 | 2.7 | 2.30 | 3 | 0.70 |
| Transforming | control | 33 | 19 | 0 | 19 | 19 | 19 | 0 |
| Growth Factor alpha | AREDS 3 | 24 | 19 | 0 | 19 | 19 | 19 | 0 |
| (TGF-alpha; pg/ml) | GA | 37 | 19.05 | 0.05 | 19 | 19 | 19 | 0 |
| TGF-beta-3 (pg/ml) | control | 33 | 38.27 | 1.27 | 37 | 37 | 37 | 0 |
|  | AREDS 3 | 24 | 37 | 0 | 37 | 37 | 37 | 0 |
|  | GA | 37 | 40.81 | 3.81 | 37 | 37 | 37 | 0 |
| Transthyretin | control | 33 | 22.79 | 0.92 | 23 | 20 | 24 | 4 |
| (TTR; mg/dl) | AREDS 3 | 24 | 21.96 | 1.18 | 20 | 18.75 | 26.25 | 7.5 |
|  | GA | 37 | 24.46 | 1.21 | 24 | 19 | 29 | 10 |
| Trefoid Factor 3 | control | 33 | 0.10 | 0.01 | 0.08 | 0.07 | 0.11 | 0.04 |
| (TFF3; ug/ml) | AREDS 3 | 24 | 0.12 | 0.01 | 0.10 | 0.09 | 0.12 | 0.04 |
|  | GA | 37 | 0.16 | 0.02 | 0.12 | 0.1 | 0.16 | 0.06 |
| Tumor Necrosis | control | 33 | 43.12 | 0.12 | 43 | 43 | 43 | 0 |
| Factor alpha | AREDS 3 | 24 | 53.62 | 10.63 | 43 | 43 | 43 | 0 |
| (TNF-alpha; pg/ml) | GA | 37 | 43 | 0 | 43 | 43 | 43 | 0 |
| TNF-beta (pg/ml) | control | 33 | 11 | 0 | 11 | 11 | 11 | 0 |
|  | AREDS 3 | 24 | 13.12 | 2.12 | 11 | 11 | 11 | 0 |
|  | GA | 37 | 11 | 0 | 11 | 11 | 11 | 0 |
| Tumor Necrosis | control | 33 | 0.36 | 0.03 | 0.35 | 0.28 | 0.41 | 0.13 |
| Factor Ligand Super- | AREDS 3 | 24 | 0.4 | 0.03 | 0.37 | 0.29 | 0.42 | 0.13 |
| Family member 12 | GA | 37 | 0.34 | 0.02 | 0.31 | 0.26 | 0.37 | 0.11 |
| (Tweak; ng/ml)  Tumor Necrosis | control | 33 | 3.02 | 0.14 | 2.9 | 2.5 | 3.7 | 1.2 |
| Factor Ligand Super- | AREDS 3 | 24 | 3.76 | 0.24 | 3.5 | 3.18 | 4.35 | 1.17 |
| Family member 12 | GA | 37 | 4.08 | 0.24 | 3.8 | 3 | 4.7 | 1.7 |
| (APRIL; ng/ml)  Tumor Necrosis | control | 33 | 1.56 | 0.11 | 1.45 | 1.11 | 1.77 | 0.66 |
| Factor Receptor 1 | AREDS 3 | 24 | 1.79 | 0.15 | 1.62 | 1.38 | 1.81 | 0.43 |
| (TNFR1; ug/ml) | GA | 37 | 2.02 | 0.14 | 1.81 | 1.44 | 2.16 | 0.72 |
| TNFR2 (pg/ml) | control | 33 | 6.19 | 0.57 | 5.5 | 3.9 | 7.3 | 3.4 |
|  | AREDS 3 | 24 | 8.02 | 0.79 | 6.85 | 5.5 | 9.8 | 4.3 |
|  | GA | 37 | 7.92 | 0.44 | 7.2 | 5.8 | 9 | 3.2 |
| TIE-2 (ng/ml) | control | 33 | 8.75 | 0.53 | 8.3 | 7 | 9.6 | 2.6 |
|  | AREDS 3 | 24 | 8.86 | 0.7 | 8.65 | 6.05 | 11 | 4.95 |
|  | GA | 37 | 9.19 | 0.64 | 8.1 | 6.9 | 12 | 5.1 |
| Urokinas-type Plas- | control | 33 | 565.67 | 29.2 | 541 | 454 | 644 | 190 |
| minogen Activator | AREDS 3 | 24 | 585.04 | 45.69 | 571 | 439.25 | 653.5 | 214.25 |
| (uPA; pg/ml) | GA | 37 | 637.16 | 39.95 | 612 | 494 | 715 | 221 |
| Urokinas-type Plas- | control | 33 | 1.93 | 0.15 | 1.8 | 1.5 | 2.2 | 0.7 |
| minogen Activator | AREDS 3 | 24 | 2.13 | 0.22 | 1.85 | 1.48 | 2.58 | 1.1 |
| Receptor | GA | 37 | 2.5 | 0.17 | 2.5 | 1.7 | 3.1 | 1.4 |
| (uPAR; ng/ml)  Vascular Cell | control | 33 | 456.61 | 22.24 | 432 | 374 | 530 | 156 |
| Adhesion Molecule 1 | AREDS 3 | 24 | 571.38 | 40.32 | 537 | 466 | 630 | 164 |
| (VCAM-1; ng/ml) | GA | 37 | 591.89 | 28.18 | 572 | 487 | 670 | 183 |
| VEGF-A (pg/ml) | control | 33 | 81.39 | 9.53 | 70 | 55 | 84 | 29 |
|  | AREDS 3 | 24 | 100.54 | 14.9 | 82.5 | 61 | 100.25 | 39.25 |
|  | GA | 37 | 112.03 | 13.31 | 89 | 63 | 127 | 64 |
| VEGF-B (ng/ml) | control | 33 | 5.3 | 0 | 5.3 | 5.3 | 5.3 | 0 |
|  | AREDS 3 | 24 | 5.3 | 0 | 5.3 | 5.3 | 5.3 | 0 |
|  | GA | 37 | 5.3 | 0 | 5.3 | 5.3 | 5.3 | 0 |
| VEGF-C (ng/ml) | control | 33 | 33.33 | 8.28 | 20 | 18 | 26 | 8 |
|  | AREDS 3 | 24 | 45.42 | 9.82 | 25.5 | 23.5 | 34.5 | 11 |
|  | GA | 37 | 44.65 | 8.11 | 28 | 20 | 41 | 21 |
| VEGF-D (pg/ml) | control | 33 | 276.33 | 10.32 | 250 | 250 | 250 | 0 |
|  | AREDS 3 | 24 | 285.62 | 11.25 | 250 | 250 | 308.75 | 58.75 |
|  | GA | 37 | 347.92 | 24.23 | 280 | 250 | 372 | 122 |
| VEGF Receptor 1 | control | 33 | 630 | 0 | 630 | 630 | 630 | 0 |
| (VEGFR-1; pg/ml) | AREDS 3 | 24 | 630 | 0 | 630 | 630 | 630 | 0 |
|  | GA | 37 | 630 | 0 | 630 | 630 | 630 | 0 |
| VEGF Receptor 2 | control | 33 | 5.16 | 0.22 | 5.2 | 4.2 | 6 | 1.8 |
| (VEGFR-2; ng/ml) | AREDS 3 | 24 | 4.5 | 0.2 | 4.45 | 3.77 | 4.93 | 1.15 |
|  | GA | 37 | 5.11 | 0.26 | 5 | 4 | 6.5 | 2.5 |
| VEGF Receptor 3 | control | 33 | 36.03 | 3.1 | 37 | 24 | 42 | 18 |
| (VEGFR-3; ng/ml) | AREDS 3 | 24 | 36.29 | 3.27 | 38.5 | 22 | 47.5 | 25.5 |
|  | GA | 37 | 42.78 | 3.65 | 40 | 32 | 48 | 16 |
| Visceral Adipose | control | 33 | 114.94 | 14.03 | 101 | 67 | 128 | 61 |
| Tissue-Derived | AREDS 3 | 23 | 184.17 | 43.38 | 132 | 80.5 | 177 | 96.5 |
| Serpin A12 | GA | 37 | 281.16 | 134.34 | 109 | 80 | 183 | 103 |
| (Vaspin; pg/ml)  Visfatin (ng/ml) | control | 33 | 1.29 | 0.15 | 0.97 | 0.74 | 1.7 | 0.96 |
|  | AREDS 3 | 24 | 5.52 | 3.54 | 1.05 | 0.71 | 2.3 | 1.59 |
|  | GA | 37 | 4.8 | 2.12 | 0.96 | 0.54 | 2.2 | 1.66 |
| Vitamin D-Binding | control | 33 | 221.06 | 17.3 | 213 | 149 | 279 | 130 |
| Protein | AREDS 3 | 24 | 248.46 | 16.73 | 231 | 211.75 | 284.25 | 72.5 |
| (VDBP; ug/ml) | GA | 37 | 245.73 | 17.91 | 249 | 164 | 300 | 136 |
| Vitamin K- | control | 33 | 13.39 | 0.69 | 13 | 11 | 14 | 3 |
| Dependent Protein S | AREDS 3 | 24 | 13.03 | 0.78 | 12 | 10.75 | 14 | 3.25 |
| (VKDPS; ug/ml) | GA | 37 | 13.26 | 0.68 | 13 | 11 | 15 | 4 |
| Vitronectin (ug/ml) | control | 33 | 735.64 | 98.82 | 504 | 425 | 871 | 446 |
|  | AREDS 3 | 24 | 696.29 | 109.34 | 530 | 407.25 | 615.5 | 208.25 |
|  | GA | 37 | 688.32 | 67.9 | 582 | 454 | 800 | 346 |
| von Willebrand | control | 33 | 52.42 | 3.57 | 56 | 39 | 61 | 22 |
| Factor | AREDS 3 | 24 | 78.67 | 11.01 | 72.5 | 50.75 | 94.5 | 43.75 |
| (vWF; ug/ml) | GA | 37 | 74.43 | 9.28 | 60 | 36 | 92 | 56 |
| Chitase-3-like-1 | control | 33 | 53.55 | 15.61 | 30 | 18 | 46 | 28 |
| Human Cartilage | AREDS 3 | 24 | 45.79 | 6.33 | 37 | 21.75 | 58.5 | 36.75 |
| Protein | GA | 37 | 62.14 | 6.76 | 58 | 25 | 82 | 57 |

Analyzed by Separate ELISA:

| Soluble Amyloid | control | 14 | 7.93 | 2.56 | 5.27 | 2.54 | 7.12 | 4.58 |
| --- | --- | --- | --- | --- | --- | --- | --- | --- |
| Precursor Protein | AREDS 3 | 20 | 8.00 | 1.51 | 6.68 | 2.54 | 10.57 | 8.03 |
| (sAPP; pg/ml) | GA | 29 | 14.93 | 2.14 | 11.66 | 8.47 | 16.79 | 8.32 |
