# Supplementary material for: Plasma biomarkers of the amyloid pathway are associated with geographic atrophy secondary to age-related macular degeneration
Source: PLoS One. 2020 Aug 7;15(8):e0236283. doi: 10.1371/journal.pone.0236283 (PMC7413518; doi:10.1371/journal.pone.0236283)
Supplement: S3 Table — Atrophy and their glossary in order of their P values. The 40 markers were detected by Myriad Luminex xMAP Technology. * sAPP was detected by ELISA and analyzed separately; Aβ(1–42) was not statistically significant but was included for comparison with Aβ(1–40). (DOCX) [file pone.0236283.s005.docx]

| **S3 Table. Cohort 1: 40 Statistically significant biomarkers for Geographic**  **Atrophy and their glossary in order of their P values** | | | | | | | |
| --- | --- | --- | --- | --- | --- | --- | --- |
| 1. Pon1 | 15. CLU |  | 29. AXL |  |  |  |  |
| 1. Omentin | 16. IGFBP6 |  | 30. IL-1β |  |  |  |  |
| 1. ST2 | 17. MMP-7 |  | 31. IL-2ra |  |  |  |  |
| 1. T-cadherin | 18. GDF-15 |  | 32. Osteopontin |  |  |  |  |
| 1. Pentraxin-3 | 19. CD27 |  | 33. COMP |  |  |  |  |
| 1. tPA | 20. Osteocalcin |  | 34. TIMP1 |  |  |  |  |
| 1. LGL | 21. RANTES |  | 35. TATI |  |  |  |  |
| 1. Baff | 22. MMP-10 |  | 36. TM |  |  |  |  |
| 1. IgE | 23. Endostatin |  | 37. CD40 |  |  |  |  |
| 1. MIP-1β | 24. Ceacam1 |  | 38. IGFBP4 |  |  |  |  |
| 1. NrCAM | 25. Hepsin |  | 39. Thrombospondin-4 |  |  |  |  |
| 1. CFHR1 | 26. TFF3 |  | 40. Aβ(1-40)* |  |  |  |  |
| 1. MMP-9 | 27. Collagen-4 |  |  |  |  |  |  |
| 1. C1QR1 | 28. IL-1r1 |  |  |  |  |  |  |
|  |  |  |  |  |  |  |  |

**Glossary of Biomarkers:**

Aβ, Amyloid-beta

sAPP, soluble amyloid precursor protein

AXL, AXL receptor tyrosine kinase

Baff, member of TNF ligand family

CD27, cluster of differentiation 27

CD40, cluster of differentiation 40

Ceacam1, carcinoembryonic antigen related adhesion molecule 1

CFHR-1, complement factor H related-1

CLU, clusterin

COMP, cartilage oligomeric matric protein

C1QR1, complement 1 q receptor 1

GDF-15, growth differentiation factor 15

IgE, immunoglobulin g E

IGFBP4, insulin like growth factor binding protein 4

IGFBP6, insulin like growth factor binding protein 6

LGL, lactogluthathione lyase/ glyoxalase 1

IL-1β, interleukin-1beta

IL-1r1, interleukin-1 receptor 1

IL-2ra, interleukin-2 receptor subunit alpha

MIP-1β, macrophage inflammatory protein-1 beta (CCL4)

MMP-7, matrix metalloproteinase-7

MMP-9, matrix metalloproteinase-9

MMP-10, matrix metalloproteinase

NrCAM, Neuronal cell adhesion molecule

Pon1, paroxonase-1

RANTES, regulated on activation, normal T cell expressed and secreted

ST2, suppression of tumorigenicity

TATI, tumor-associated trypsinogen inhibitor

TIMP1, tissue inhibitor of metalloproteinase-1

TFF3, trefoil factor 3

TM, thrombomodulin

tPA, tissue plasminogen activator
