# Supplementary material for: Plasma biomarkers of the amyloid pathway are associated with geographic atrophy secondary to age-related macular degeneration
Source: PLoS One. 2020 Aug 7;15(8):e0236283. doi: 10.1371/journal.pone.0236283 (PMC7413518; doi:10.1371/journal.pone.0236283)
Supplement: S4 Table — Key: Control, non-AMD control; AREDS 3, intermediate dry AMD; GA, geographic atrophy. (DOCX) [file pone.0236283.s006.docx]

**S4 Table. Cohort 2: Summary Statistics (N, mean, standard error of the mean [SE], median [MEDIAN],**

**25 and 75% percentiles [25% and 75%] and interquartile range [IQR]) of analyte concentrations by group**

| **ANALYTE** | **GROUP** | **N** | **MEAN** | **SE** | **MEDIAN** | **25%** | **75%** | **IQR** |
| --- | --- | --- | --- | --- | --- | --- | --- | --- |
| Paraoxonase-1 | control | 27 | 8.77 | 0.73 | 8.34 | 6.99 | 10.16 | 3.17 |
| Pon1 (ug/ml) | AREDS 3 | 22 | 7.43 | 0.66 | 6.80 | 5.39 | 8.75 | 3.36 |
|  | GA | 29 | 7.12 | 0.63 | 5.92 | 4.05 | 9.71 | 5.66 |
| Omentin (ng/ml) | control | 26 | 166.29 | 14.90 | 158.53 | 137.56 | 188.62 | 51.06 |
|  | AREDS 3 | 22 | 197.09 | 23.29 | 193.47 | 99.12 | 259.75 | 160.63 |
|  | GA | 29 | 740.63 | 82.50 | 681.03 | 372.95 | 103.00 | 657.05 |
| ST2 (ng/ml) | control | 28 | 6.54 | 0.76 | 6.40 | 3.90 | 8.62 | 4.71 |
|  | AREDS 3 | 22 | 9.50 | 1.51 | 9.34 | 2.96 | 13.62 | 10.67 |
|  | GA | 15 | 10.24 | 1.63 | 8.56 | 6.52 | 12.19 | 5.67 |
| Cadherin-1 | control | 27 | 91.07 | 4.21 | 88.59 | 78.02 | 106.87 | 28.85 |
| (T-cad; pg/ml) | AREDS 3 | 21 | 92.05 | 7.42 | 79.18 | 66.04 | 111.33 | 45.29 |
|  | GA | 30 | 113.32 | 7.03 | 112.56 | 85.32 | 143.17 | 57.85 |
| Pentraxin-3 (pg/ml) | control | 27 | 617.42 | 64.43 | 573.47 | 407.86 | 711.41 | 303.56 |
|  | AREDS 3 | 22 | 788.80 | 142.23 | 657.37 | 494.85 | 746.58 | 251.73 |
|  | GA | 29 | 1,779.67 | 179.09 | 1,460.97 | 1,129.65 | 2,237.07 | 1,107.42 |
| Tissue Plasminogen | control | 29 | 136.08 | 8.21 | 126.27 | 104.36 | 155.76 | 51.39 |
| Activator | AREDS 3 | 22 | 154.17 | 15.62 | 140.02 | 108.93 | 161.26 | 52.33 |
| (tPA; pg/ml) | GA | 19 | 267.21 | 27.86 | 265.21 | 176.00 | 323.25 | 147.26 |
| Lactoylglutathione | control | 28 | 14.96 | 1.18 | 14.19 | 10.18 | 18.57 | 8.40 |
| Lyase | AREDS 3 | 22 | 22.51 | 2.94 | 17.19 | 13.78 | 28.31 | 14.52 |
| (LGL; ug/ml) | GA | 30 | 18.067 | 1.37 | 17.98 | 13.04 | 21.73 | 8.68 |
| B Cell-Activating | control | 28 | 28.45 | 1.58 | 27.31 | 24.80 | 32.04 | 7.24 |
| Factor | AREDS 3 | 22 | 36.73 | 5.27 | 29.49 | 24.70 | 34.32 | 9.61 |
| (Baff; pg/ml) | GA | 30 | 59.33 | 25.12 | 33.74 | 24.49 | 40.45 | 15.96 |
| IgE (pg/ml) | control | 27 | 471.65 | 179.37 | 102.61 | 17.98 | 288.68 | 270.69 |
|  | AREDS 3 | 22 | 351.47 | 165.03 | 43.68 | 25.89 | 201.22 | 175.33 |
|  | GA | 29 | 349.39 | 83.42 | 167.13 | 43.07 | 504.20 | 461.13 |
| MIP-1beta (pg/ml) | control | 28 | 275.46 | 48.93 | 141.93 | 62.85 | 429.32 | 366.48 |
|  | AREDS 3 | 22 | 283.16 | 53.03 | 268.13 | 65.01 | 446.68 | 381.66 |
|  | GA | 12 | 205.41 | 43.25 | 134.00 | 98.25 | 283.55 | 185.30 |
| MMP-9 (ng/ml) | control | 28 | 30.92 | 9.72 | 12.39 | 8.65 | 24.61 | 15.97 |
|  | AREDS 3 | 22 | 26.40 | 5.81 | 17.49 | 6.80 | 37.51 | 307.11 |
|  | GA | 15 | 52.02 | 13.09 | 36.51 | 23.81 | 56.25 | 324.45 |
| Complement | control | 28 | 366.95 | 19.06 | 382.77 | 333.39 | 412.94 | 79.55 |
| Component C1q | AREDS 3 | 22 | 498.84 | 47.82 | 469.67 | 345.95 | 626.00 | 280.06 |
| Receptor (pg/ml) | GA | 30 | 438.73 | 30.14 | 405.69 | 311.71 | 498.42 | 186.71 |
| Clusterin | control | 27 | 134.21 | 54.90 | 54.92 | 28.19 | 107.21 | 79.02 |
| (CLU; ng/ml) | AREDS 3 | 22 | 154.67 | 32.95 | 111.52 | 61.78 | 148.71 | 86.93 |
|  | GA | 24 | 89.70 | 29.63 | 52.53 | 30.50 | 88.55 | 58.05 |
| IGFBP6 (pg/ml) | control | 27 | 230.98 | 18.91 | 197.15 | 159.72 | 307.46 | 147.74 |
|  | AREDS 3 | 22 | 306.21 | 32.80 | 238.76 | 202.31 | 388.66 | 186.35 |
|  | GA | 29 | 440.63 | 32.06 | 423.18 | 300.49 | 549.02 | 218.53 |
| MMP-7 (ng/ml) | control | 28 | 3.80 | 0.27 | 3.34 | 2.87 | 4.35 | 1.49 |
|  | AREDS 3 | 22 | 5.15 | 0.73 | 4.26 | 3.60 | 4.82 | 1.22 |
|  | GA | 15 | 2.50 | 0.57 | 1.53 | 0.76 | 4.48 | 3.72 |
| Growth/ | control | 28 | 659.13 | 65.74 | 599.43 | 429.37 | 880.12 | 450.75 |
| Differentiation | AREDS 3 | 22 | 989.83 | 139.06 | 777.32 | 517.47 | 1313.64 | 796.17 |
| Factor-15 (pg/ml) | GA | 15 | 854.55 | 97.42 | 715.85 | 606.20 | 963.35 | 5357.15 |
| T-Cell Specific | control | 28 | 13.45 | 0.79 | 14.21 | 9.93 | 16.60 | 6.67 |
| Protein, RANTES | AREDS 3 | 22 | 12.55 | 1.10 | 13.81 | 9.42 | 15.57 | 6.16 |
| (RANTES; ng/ml) | GA | 12 | 15.25 | 1.54 | 17.46 | 9.68 | 19.65 | 9.97 |
| MMP-10 (ng/ml) | control | 28 | 1.51 | 0.18 | 1.36 | 0.97 | 1.83 | 0.85 |
|  | AREDS 3 | 22 | 1.47 | 0.19 | 1.13 | 0.82 | 2.01 | 1.19 |
|  | GA | 15 | 2.60 | 0.82 | 1.52 | 0.96 | 3.02 | 2.06 |
| Endostatin (ng/ml) | control | 28 | 1.21 | 0.20 | 0.72 | 0.42 | 1.70 | 1.27 |
|  | AREDS 3 | 22 | 1.09 | 0.17 | 0.78 | 0.52 | 1.60 | 1.08 |
|  | GA | 30 | 1.71 | 0.39 | 1.07 | 0.88 | 1.54 | 0.66 |
| sAPP * (pg/ml) | control | 26 | 1.70 | 0.18 | 1.56 | 1.07 | 2.21 | 1.14 |
|  | AREDS 3 | 21 | 1.68 | 0.18 | 1.63 | 0.96 | 2.52 | 1.56 |
|  | GA | 29 | 2.42 | 0.18 | 2.41 | 1.62 | 2.92 | 1.30 |
| Carcinoembryonic | control | 28 | 284.61 | 40.90 | 215.89 | 135.95 | 422.16 | 286.21 |
| Antigen-Related Cell | AREDS 3 | 22 | 404.95 | 98.01 | 229.81 | 128.83 | 414.53 | 285.70 |
| Adhesion Molecule 1 | GA | 30 | 470.54 | 133.78 | 327.23 | 224.34 | 424.22 | 199.87 |
| (ceacam1; pg/ml)  Hepsin (U/ml) | control | 28 | 11.49 | 5.63 | 4.76 | 4.14 | 6.98 | 2.83 |
|  | AREDS 3 | 22 | 95.96 | 83.91 | 4.83 | 4.51 | 5.72 | 1.21 |
|  | GA | 28 | 16.99 | 4.12 | 11.23 | 8.82 | 14.13 | 5.32 |
| Trefoid Factor 3 | control | 28 | 293.48 | 26.47 | 255.54 | 211.16 | 294.85 | 83.69 |
| (TTF3; pg/ml) | AREDS 3 | 22 | 339.40 | 28.83 | 306.80 | 239.30 | 396.27 | 156.97 |
|  | GA | 26 | 273.57 | 28.70 | 248.59 | 205.19 | 314.54 | 109.34 |
| Collagen IV | control | 27 | 411.37 | 64.21 | 416.95 | 129 | 561.01 | 432.01 |
| (Collagen 4; pg/ml) | AREDS 3 | 22 | 374.22 | 70.85 | 267.01 | 129 | 525.00 | 396.00 |
|  | GA | 15 | 283.80 | 50.81 | 267.01 | 129 | 343.81 | 214.81 |
| IL-1 Receptor Type 1 | control | 28 | 958.85 | 93.34 | 1,099.20 | 695.28 | 1,290.09 | 594.82 |
| (IL-1R1; pg/ml) | AREDS 3 | 22 | 1,087.02 | 126.56 | 1,168.16 | 863.87 | 1,535.22 | 671.35 |
|  | GA | 15 | 1,052.42 | 133.71 | 1,145.20 | 744.12 | 1,427.01 | 682.89 |
| AXL Receptor | control | 28 | 806.58 | 40.36 | 807.66 | 683.95 | 955.18 | 271.23 |
| Tyrosine Kinase | AREDS 3 | 22 | 882.16 | 74.09 | 869.32 | 631.43 | 1,023.90 | 392.47 |
| (AXL; pg/ml) | GA | 30 | 1,413.50 | 112.20 | 1,363.85 | 1,023.95 | 1,683.31 | 659.36 |
| IL-1beta (pg/ml) | control | 28 | 57.05 | 22.34 | 20.34 | 4.35 | 40.92 | 36.57 |
|  | AREDS 3 | 22 | 29.13 | 5.62 | 36.30 | 3.82 | 45.25 | 41.44 |
|  | GA | 12 | 19.93 | 5.84 | 5.20 | 4.04 | 39.33 | 35.29 |
| Osteopontin (ng/ml) | control | 28 | 74.045 | 4.43 | 77.37 | 61.66 | 92.41 | 30.75 |
|  | AREDS 3 | 22 | 85.57 | 6.41 | 91.39 | 64.41 | 106.57 | 42.15 |
|  | GA | 13 | 109.03 | 12.33 | 93.18 | 72.22 | 136.68 | 64.46 |
| Tissue Inhibitor of | control | 27 | 61.92 | 4.025 | 59.21 | 44.33 | 78.83 | 34.50 |
| Metalloproteinases-1 | AREDS 3 | 22 | 73.78 | 5.24 | 72.30 | 54.87 | 85.07 | 30.20 |
| TIMP1 (ng/ml) | GA | 27 | 66.64 | 4.250 | 65.98 | 52.27 | 76.26 | 23.99 |
| Pancreatic Secretory | control | 28 | 2.01 | 0.10 | 1.90 | 1.62 | 2.36 | 0.75 |
| Trypsin Inhibitor | AREDS 3 | 22 | 2.75 | 0.19 | 2.76 | 2.01 | 3.54 | 1.53 |
| (TATI; ng/ml) | GA | 30 | 2.64 | 0.15 | 2.59 | 2.06 | 3.14 | 1.08 |
| Thrombomodulin | control | 29 | 335.58 | 13.88 | 332.15 | 285.41 | 373.89 | 88.48 |
| (TM; pg/ml) | AREDS 3 | 22 | 407.30 | 32.42 | 363.75 | 302.66 | 454.42 | 151.76 |
|  | GA | 28 | 384.14 | 19.26 | 380.66 | 321.72 | 459.60 | 137.88 |
| CD40 (pg/ml) | control | 28 | 239.46 | 29.39 | 246.12 | 61.62 | 366.24 | 304.62 |
|  | AREDS 3 | 22 | 357.83 | 66.12 | 328.58 | 92.83 | 478.51 | 385.68 |
|  | GA | 15 | 359.49 | 173.22 | 186.48 | 66.02 | 293.87 | 227.85 |
| IGFBP4 (pg/ml) | control | 27 | 438.04 | 19.97 | 440.46 | 355.55 | 489.40 | 133.85 |
|  | AREDS 3 | 22 | 564.73 | 44.99 | 489.10 | 403.43 | 656.35 | 252.92 |
|  | GA | 29 | 483.77 | 32.16 | 443.72 | 367.12 | 570.83 | 203.71 |
| Thrombospondin-4 | control | 28 | 183.15 | 7.082 | 177.95 | 156.41 | 199.42 | 43.01 |
| (TSP-4; ug/ml) | AREDS 3 | 22 | 199.11 | 12.04 | 194.01 | 161.46 | 240.26 | 78.80 |
|  | GA | 30 | 197.18 | 8.55 | 186.65 | 173.71 | 231.55 | 57.83 |
| Amyloid-β (1-40) | control | 33 | 336.15 | 29.66 | 273.00 | 229.00 | 349.00 | 120.00 |
| (Aβ(1-40); pg/ml) | AREDS 3 | 24 | 338.46 | 27.93 | 313.00 | 243.00 | 347.25 | 104.25 |
|  | GA | 35 | 425.62 | 26.61 | 396.00 | 306.00 | 499.00 | 193.00 |
| Amyloid-β (1-42) | control | 27 | 68.22 | 19.56 | 26.20 | 21.52 | 44.22 | 22.69 |
| (Aβ(1-42); pg/ml) | AREDS 3 | 21 | 40.36 | 10.39 | 25.14 | 23.41 | 28.11 | 4.70 |
|  | GA | 16 | 45.45 | 7.77 | 40.51 | 27.63 | 50.24 | 22.61 |
|  |  |  |  |  |  |  |  |  |
