# Supplementary material for: Plasma biomarkers of the amyloid pathway are associated with geographic atrophy secondary to age-related macular degeneration
Source: PLoS One. 2020 Aug 7;15(8):e0236283. doi: 10.1371/journal.pone.0236283 (PMC7413518; doi:10.1371/journal.pone.0236283)
Supplement: S5 Table — (DOCX) [file pone.0236283.s007.docx]

**S5 Table: Cohort 1: Nomenclature of significant proteins and genes identified using bioinformatics**

| **Assay** | **Gene** | **Entrez Gene ID** | **Description** |
| --- | --- | --- | --- |
| APP | APP | 351 | Amyloid beta (A4) precursor protein |
| AXL | AXL | 558 | AXL receptor tyrosine kinase |
| Baff | TNFSF13B | 10673 | Tumor necrosis factor (ligand) superfamily, member 13b |
| C1QR1 | CD93 | 22918 | CD93 molecule |
| CD27 | CD27 | 939 | CD27 molecule |
| CD40 | CD40 | 958 | CD40 molecule, TNF receptor superfamily member 5 |
| Ceacam1 | CECAM1 | 634 | Carcinoembryonic antigen-related cell adhesion molecule-1 |
| CFHR1 | CFHR1 | 3078 | Complement factor H-related 1 |
| CLU | CLU | 1191 | Clusterin |
| Collagen-4 | COL4A1 | 1282 | Collagen, type IV, alpha 1 |
| Collagen-4 | COL4A2 | 1284 | Collagen, type IV, alpha 2 |
| Collagen-4 | COL4A3 | 1285 | Collagen, type IV, alpha 3 |
| Collagen-4 | COL4A4 | 1286 | Collagen, type IV, alpha 4 |
| Collagen-4 | COL4A5 | 1287 | Collagen, type IV, alpha 5 |
| Collagen-4 | COL4A6 | 1288 | Collagen, type IV, alpha 6 |
| COMP | COMP | 1311 | Cartilage oligometric matrix protein |
| Endostatin | COL18A1 | 80781 | Collagen, type XVIII, alpha 1 |
| GDF-15 | GDF-15 | 9518 | Growth differentiation factor-15 |
| Hepsin | HPN | 3249 | hepsin |
| IgE | IGHE | 3497 | Immunoglobulin heavy constant epsilon |
| IGFBP4 | IGFBP4 | 3487 | Insulin-like growth factor binding protein-4 |
| IGFBP6 | IBFPB6 | 3489 | Insulin-like growth factor binding protein-6 |
| IL-1β | IL1B | 3553 | Interleukin-1 𝛽 |
| IL-1ra | IL1R1 | 3554 | Interleukin-1 receptor, type 1 |
| IL-2ra | IL2RA | 3559 | Interleukin-2 receptor, alpha |
| LGL | GLO1 | 2739 | Lactogluthathione lyase / glyoxalase I |
| MIP-1𝛽 | CCL4 | 6351 | Chemokine (C-C motif) ligand 4 |
| MMP-10 | MMP10 | 4319 | Matrix metallopeptidase-10 (stromelysin 2) |
| MMP-7 | MMP7 | 4316 | Matrix metallopeptidase-7 (matrilysin, uterine) |
| MMP-9 | MMP9 | 4318 | Matrix metallopeptidase-9 (gelatinase B, type IV collagenase) |
| NrCAM | NRCAM | 4897 | Neuronal cell adhesion molecule |
| Omentin | ITLN1 | 55600 | Intelectin-1 (galactofuranoase binding) |
| Osteoclastin | BGLAP | 632 | Bone gamma-carboxyglutamate (gla) protein |
| Osteopontin | SPP1 | 6696 | Secreted phosphoprotein-1 |
| Pentraxin-3 | PTX3 | 5806 | Pentraxin-3, long |
| Pon1 | PON1 | 5444 | Paraoxonase 1 |
| RANTES | CCL5 | 6352 | Chemokine (C-C motif) ligand 5 |
| ST2 | ST2 | 6761 | Suppression of tumorigenecity |
| T-cadherin | CDH13 | 1012 | Cadherin 13 |
| TATI | SPINK1 | 6690 | Serine peptidase inhibitor, Kazal type 1 |
| TFF3 | TFF3 | 7033 | Trefoil factor-3 (intestinal) |
| TIMP1 | TIMP1 | 7076 | TIMP metallopeptidase inhibitor 1 |
| TM | THBD | 7056 | Thrombomodulin |
| tPA | PLAT | 5327 | Tissue plasminogen activator |
| TSP-4 | THBS4 | 7060 | Thrombospondin-4 |
