# Supplementary material for: Plasma biomarkers of the amyloid pathway are associated with geographic atrophy secondary to age-related macular degeneration
Source: PLoS One. 2020 Aug 7;15(8):e0236283. doi: 10.1371/journal.pone.0236283 (PMC7413518; doi:10.1371/journal.pone.0236283)
Supplement: S1 File — (ZIP) [file Boxplots for Reviewers Cohorts 1, 2, Ab ratios.pdf › Boxplots for Reviewers Cohorts 1, 2, Ab ratios.pdf]

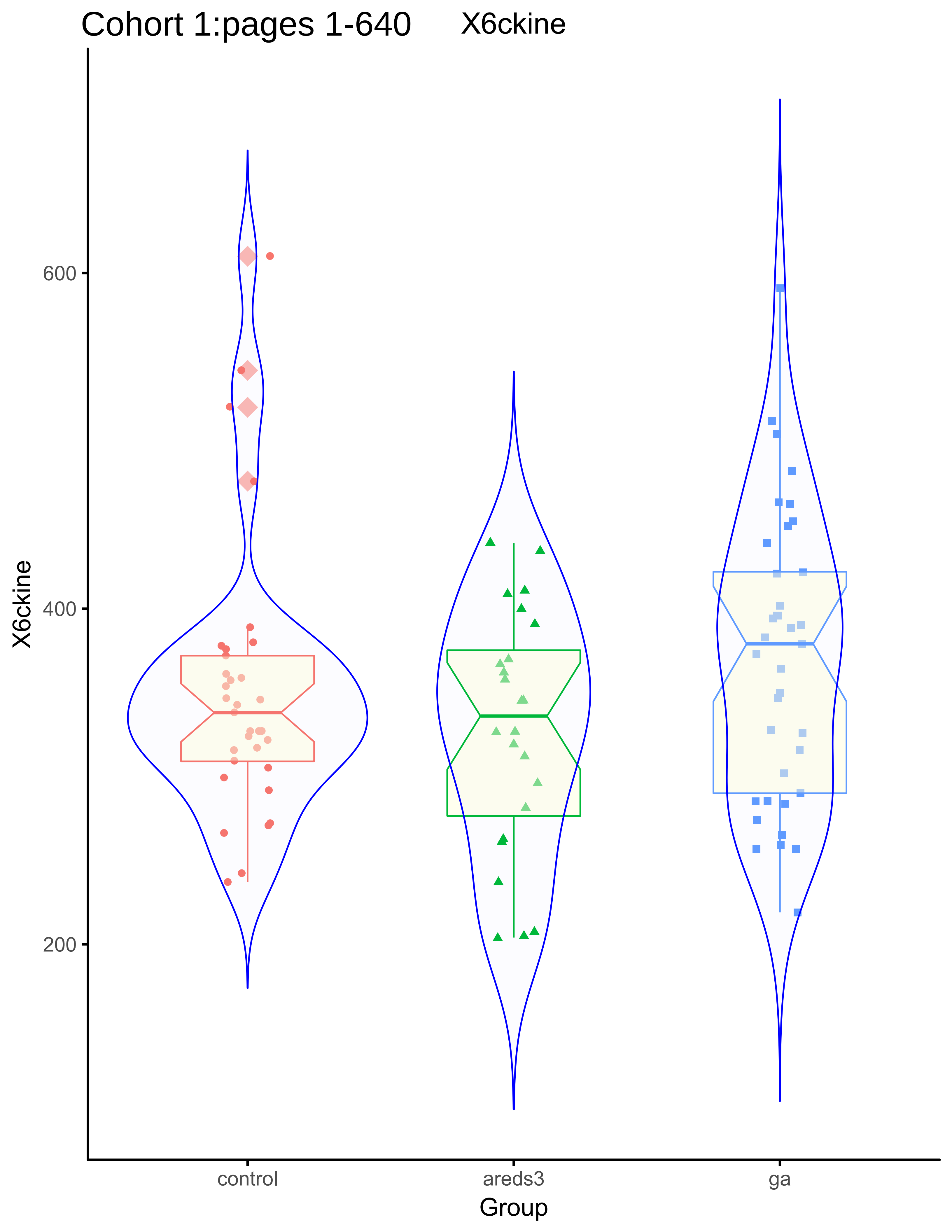
Cohort 1:pages 1-640
X6ckine
X6ckine
600
400
200
control
areds3
ga
Group

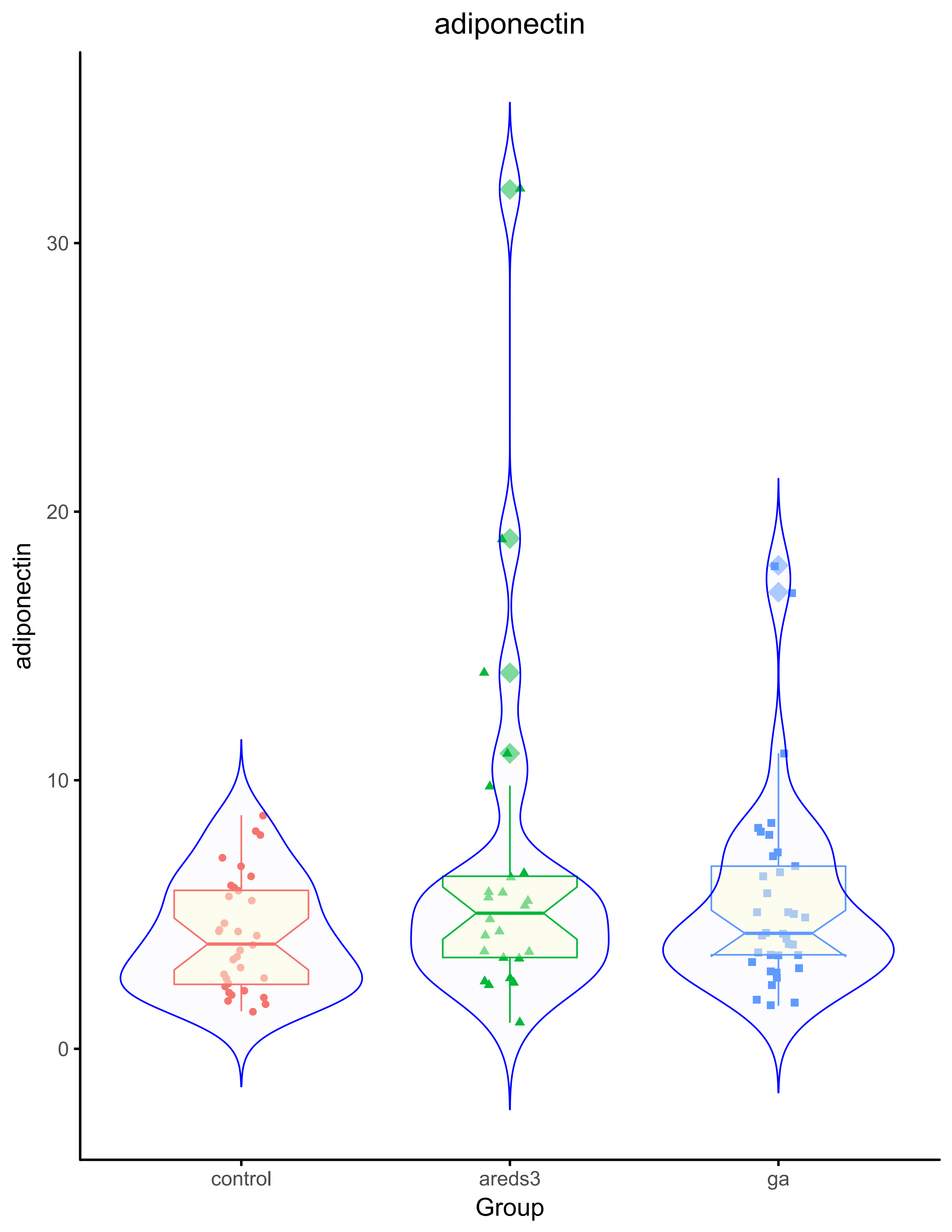
adiponectin
30
20
10
0
adiponectin
control
areds3
ga
Group

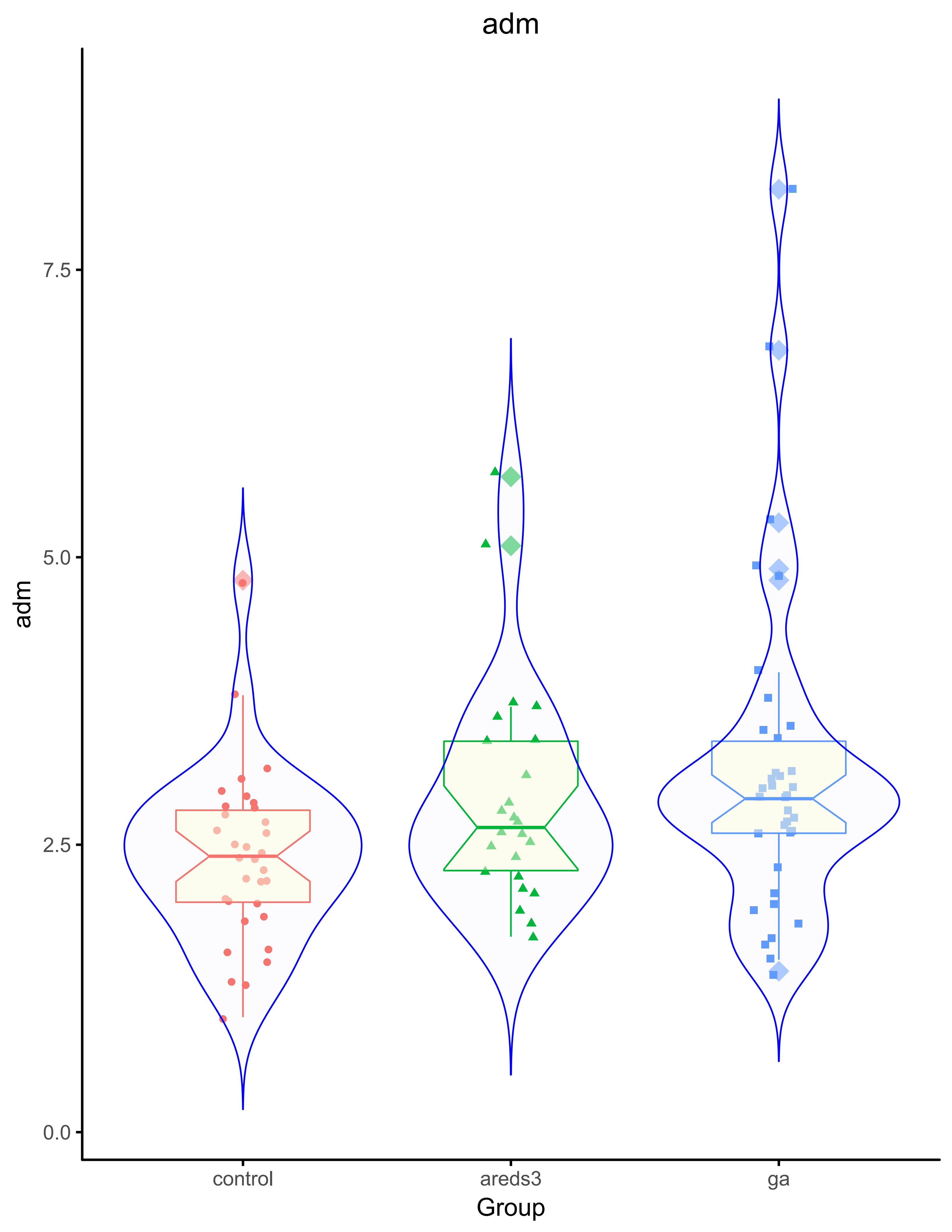

adm
7.5
5.0
2.5
0.0
adm
control
areds3
ga
Group

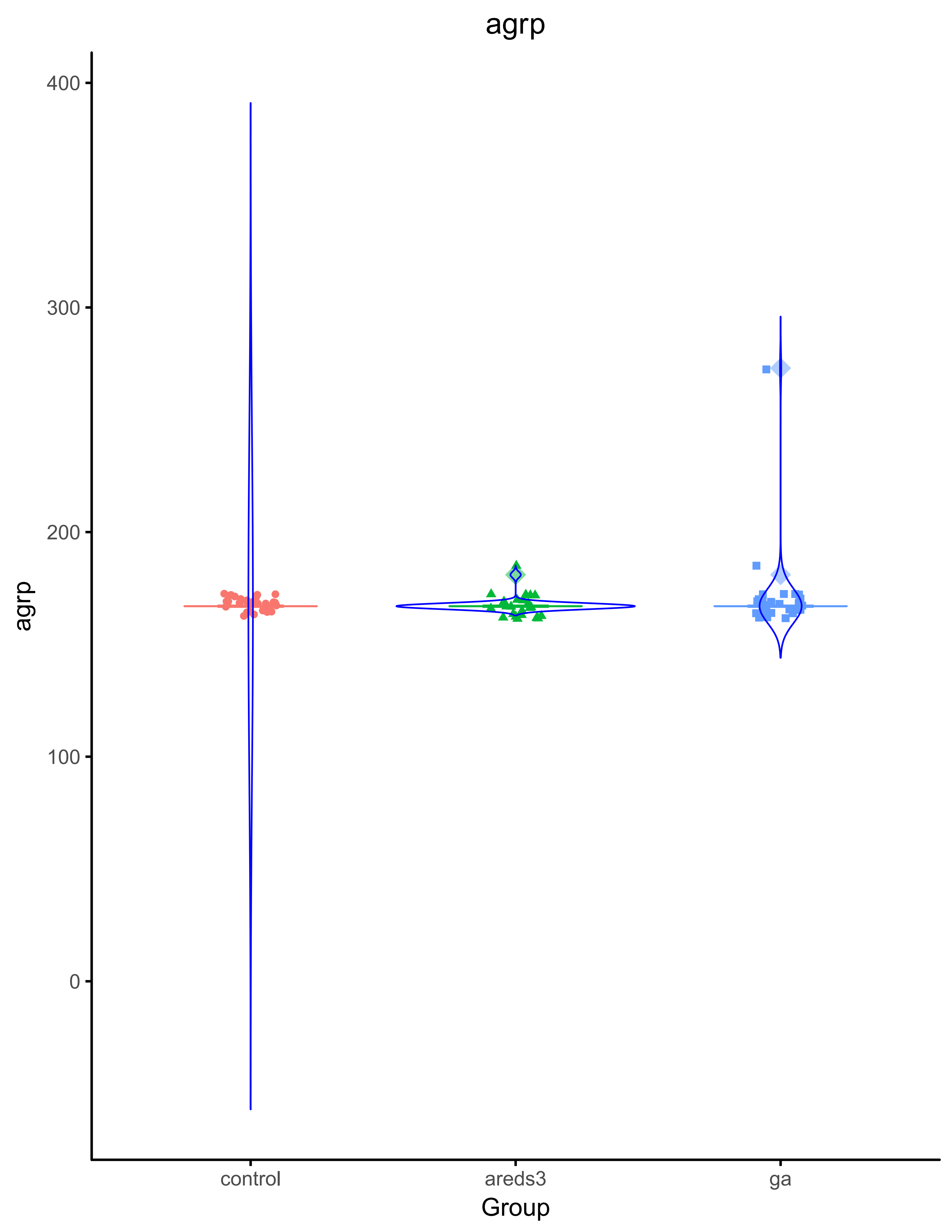

agrp
400
300
200
100
0
agrp
control
areds3
ga
Group

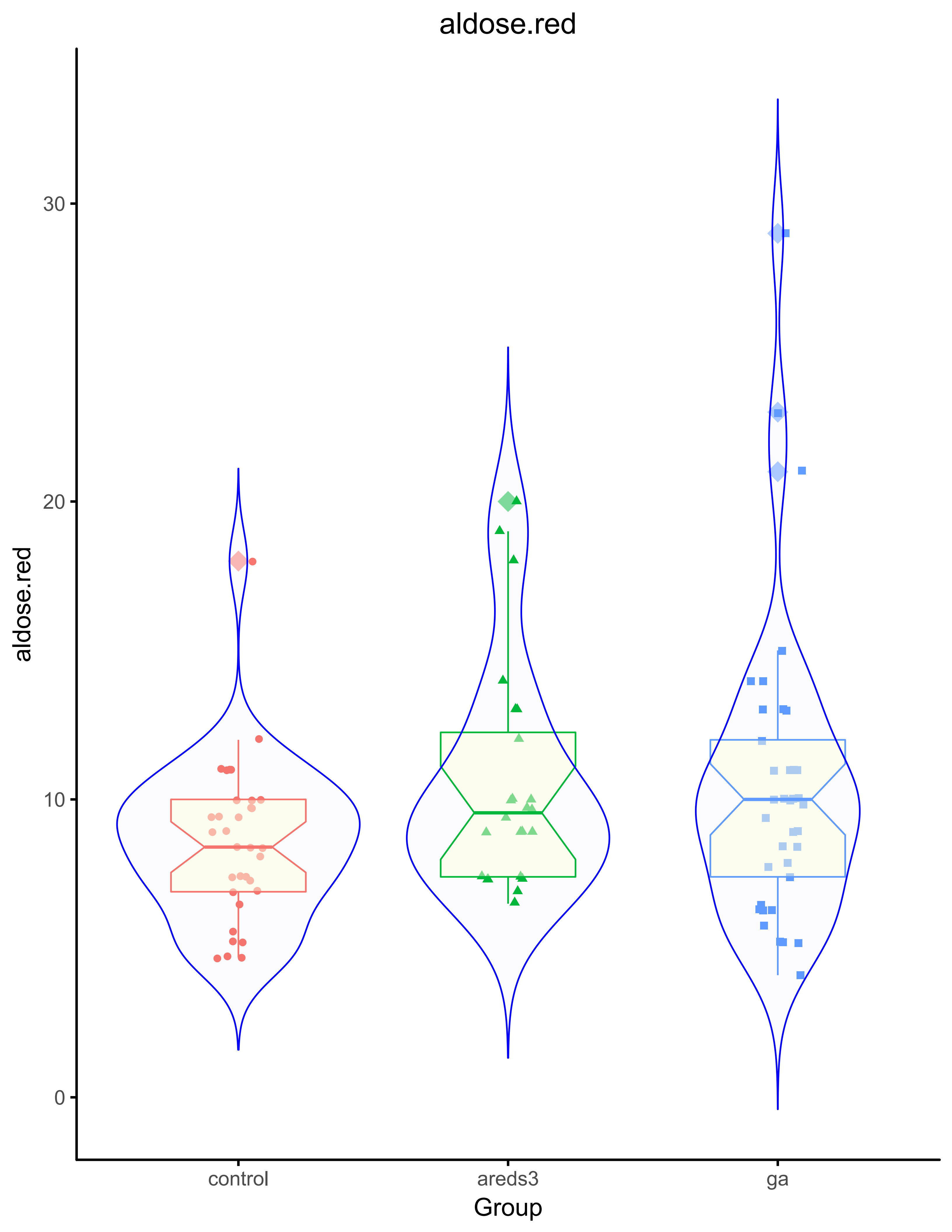

aldose.red
aldose.red
30
20
10
0
control
areds3
ga
Group

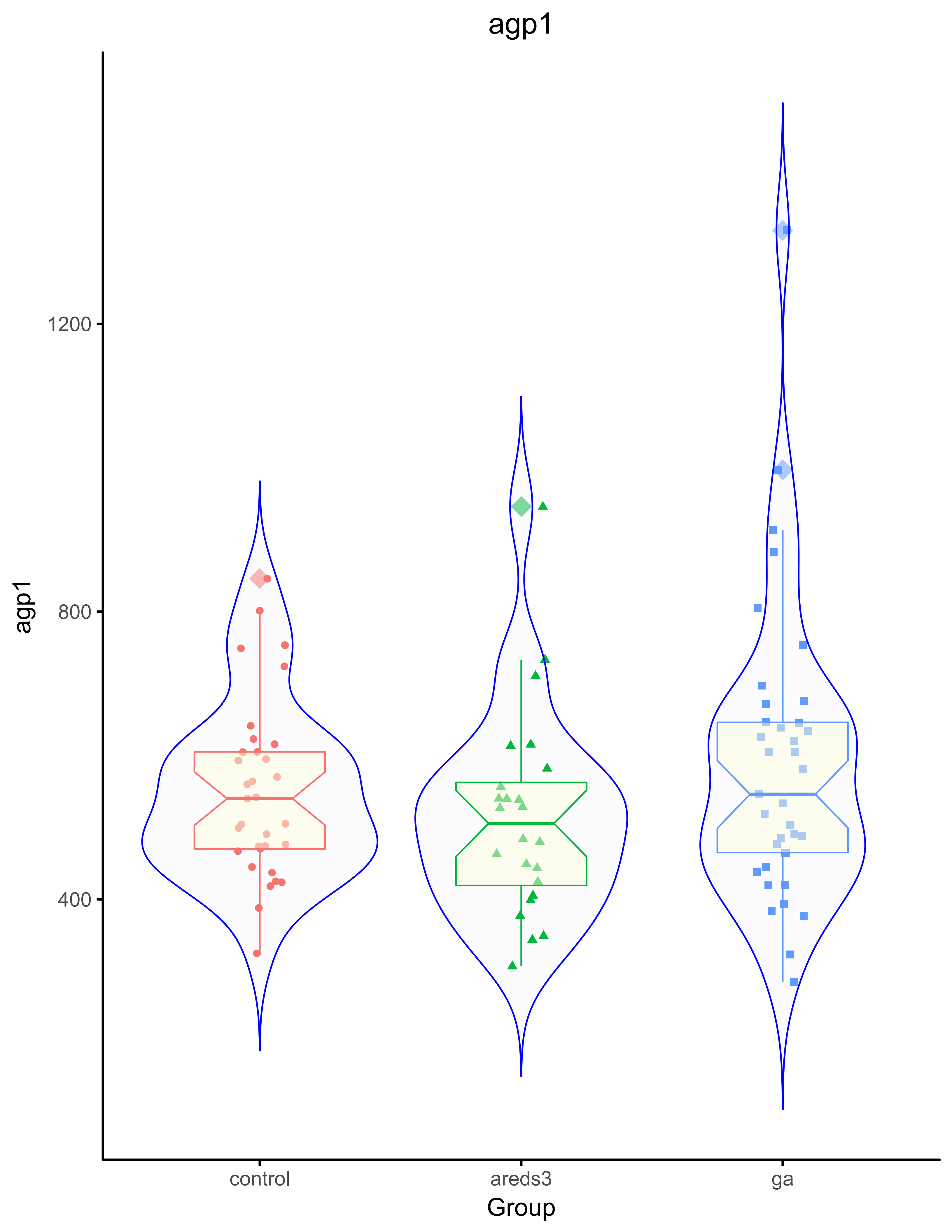
agp1
1200
800
400
agp1
control
areds3
ga
Group

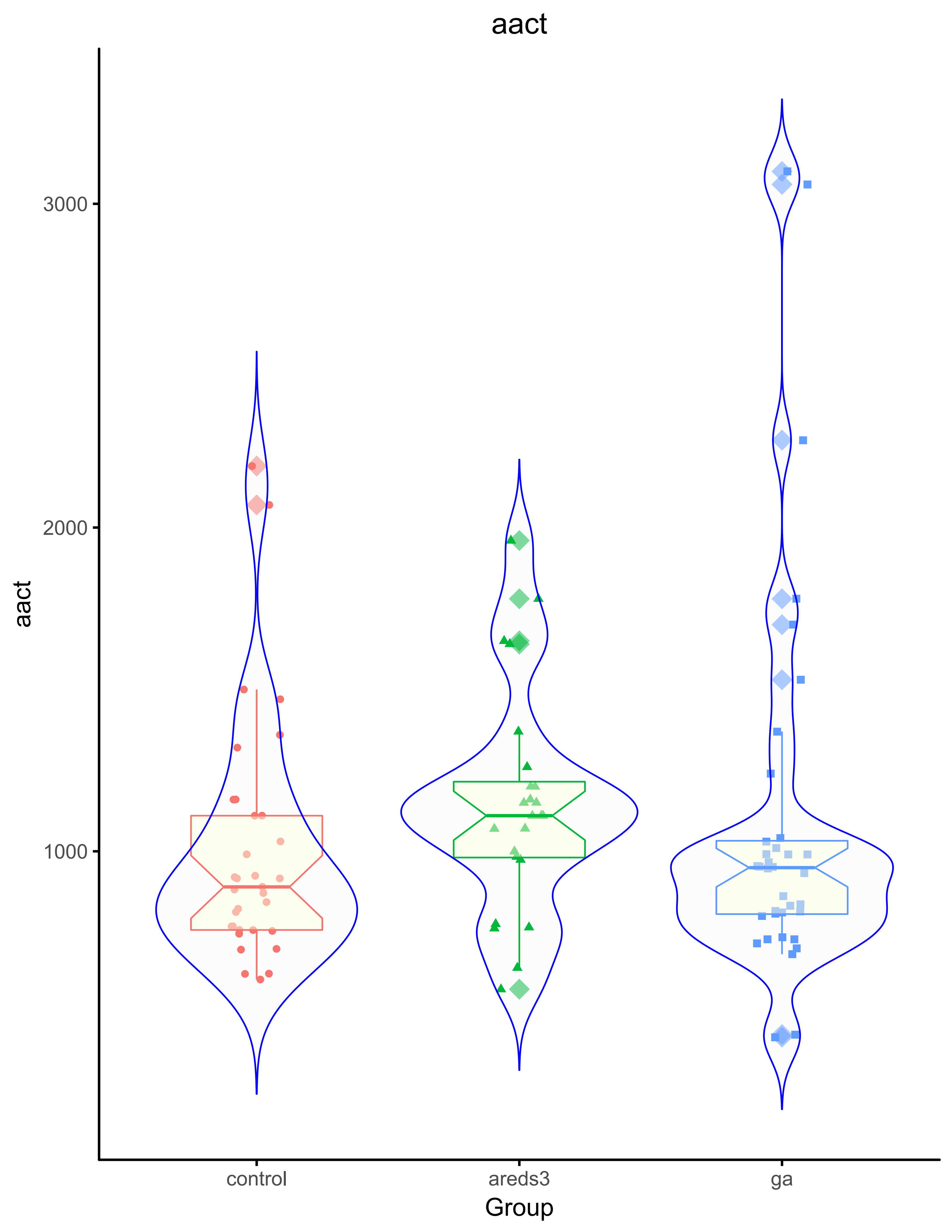

aact
3000
2000
1000
aact
control
areds3
ga
Group

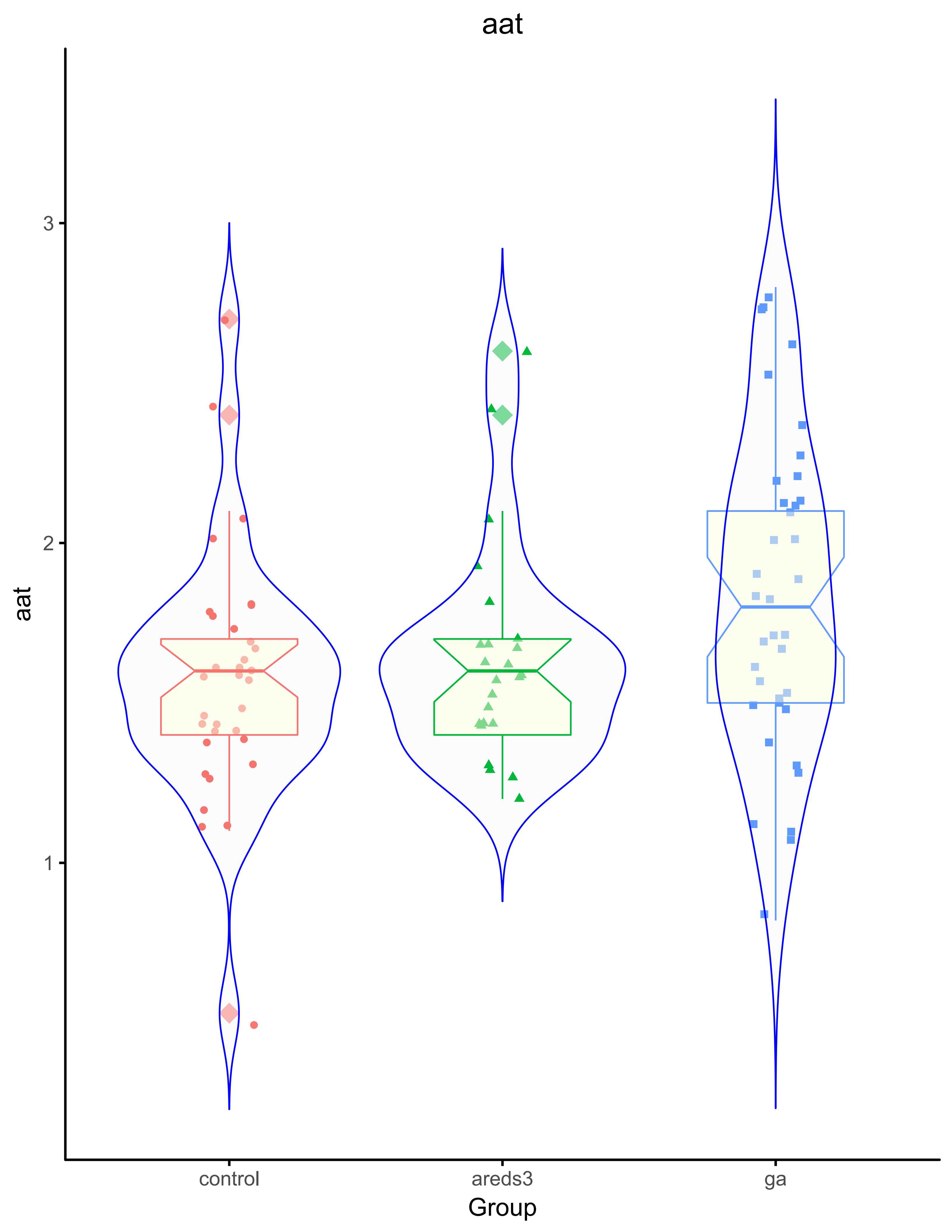
aat
3
2
1
aat
control
areds3
ga
Group

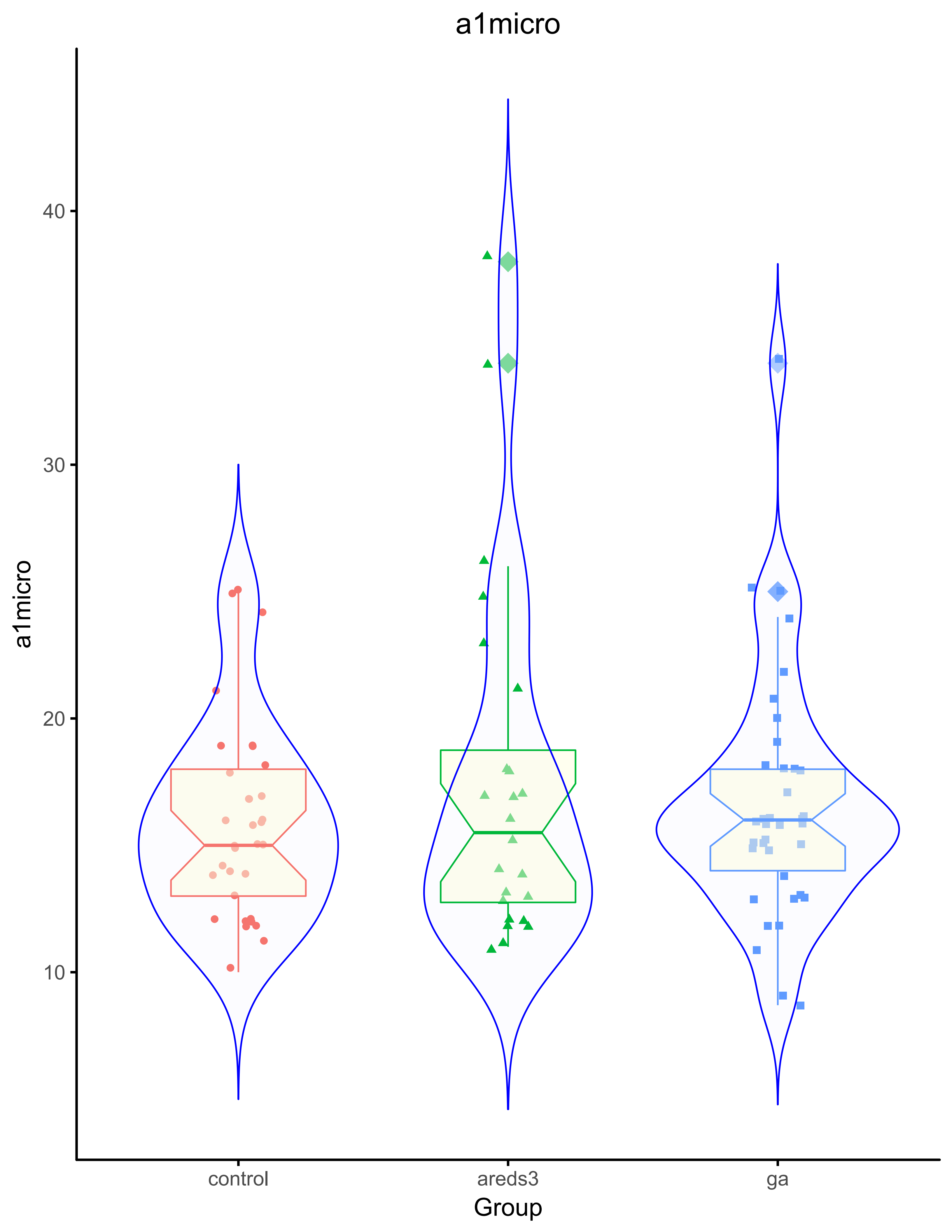
a1micro
a1micro
40
30
20
10
control
areds3
ga
Group

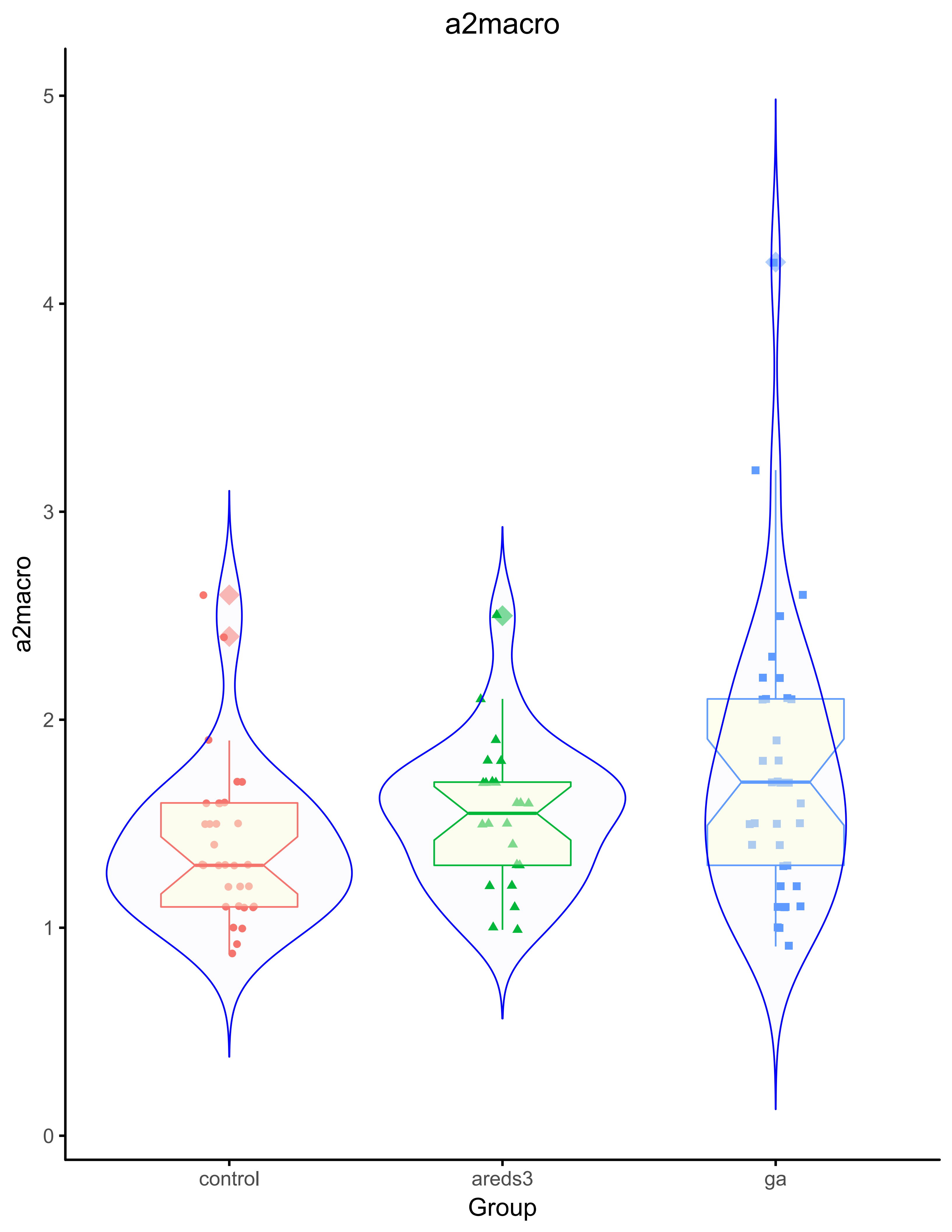

a2macro
5
4
3
2
1
0
a2macro
control
areds3
ga
Group

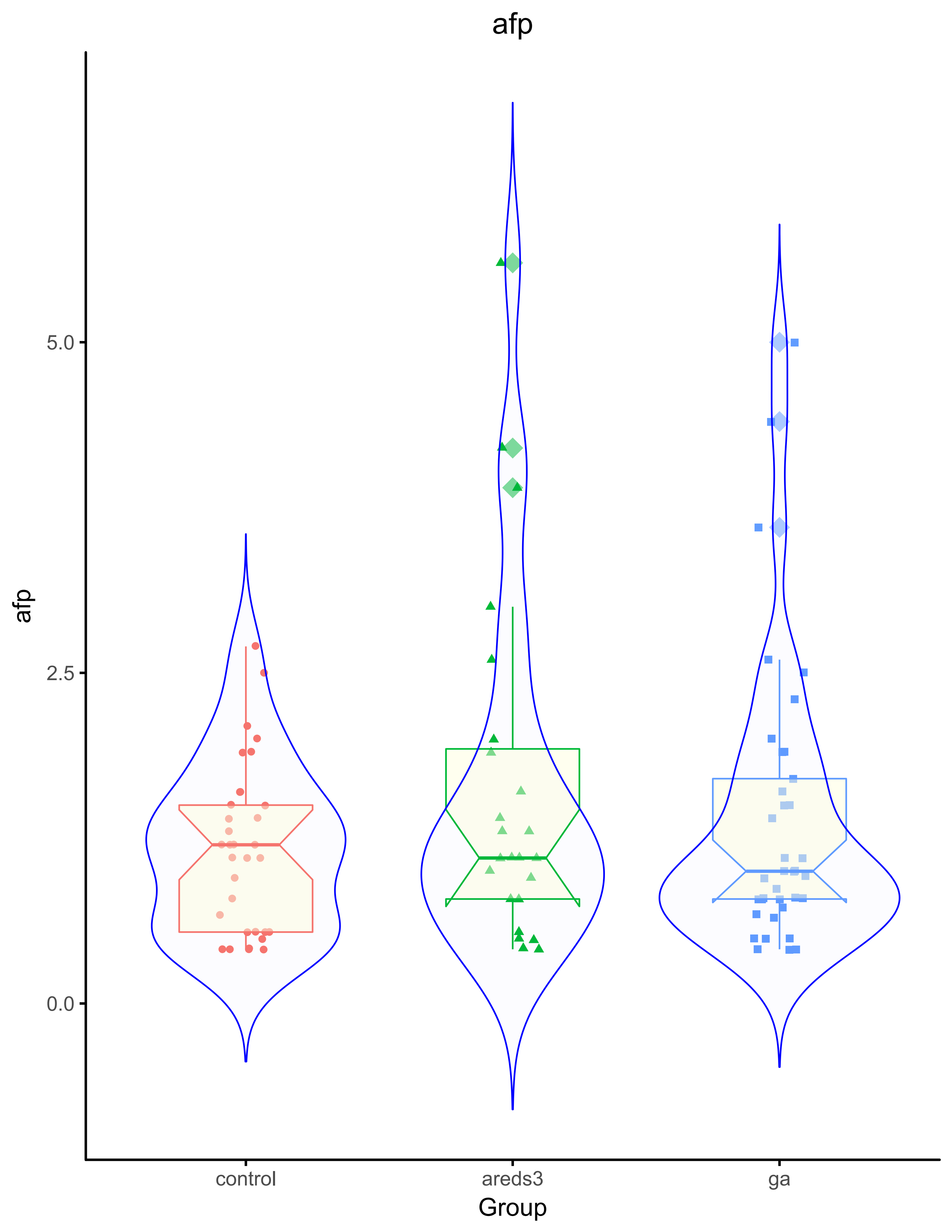
afp
afp
0.0
2.5
5.0
control
areds3
ga
Group

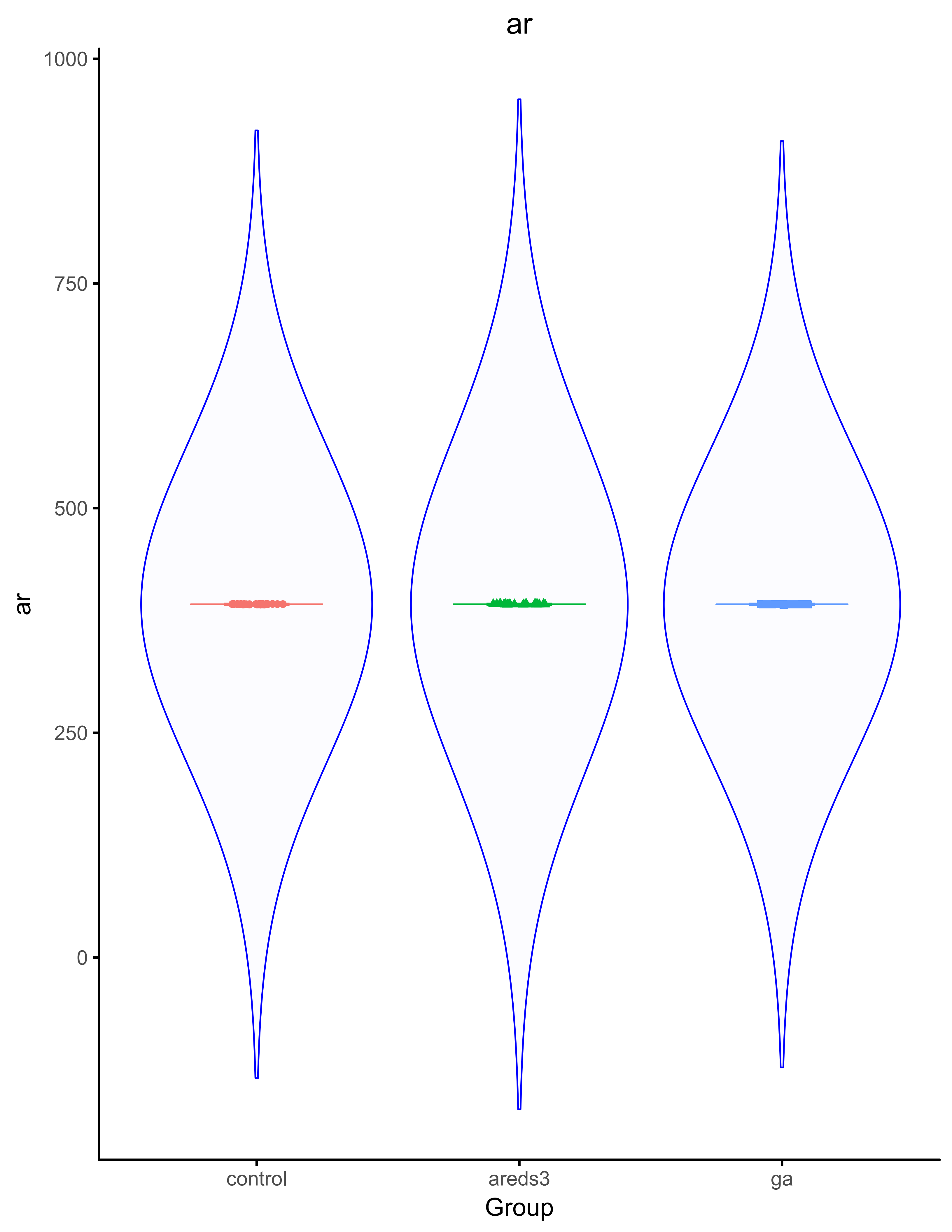

ar
1000
750
500
250
0
ar
control
areds3
ga
Group

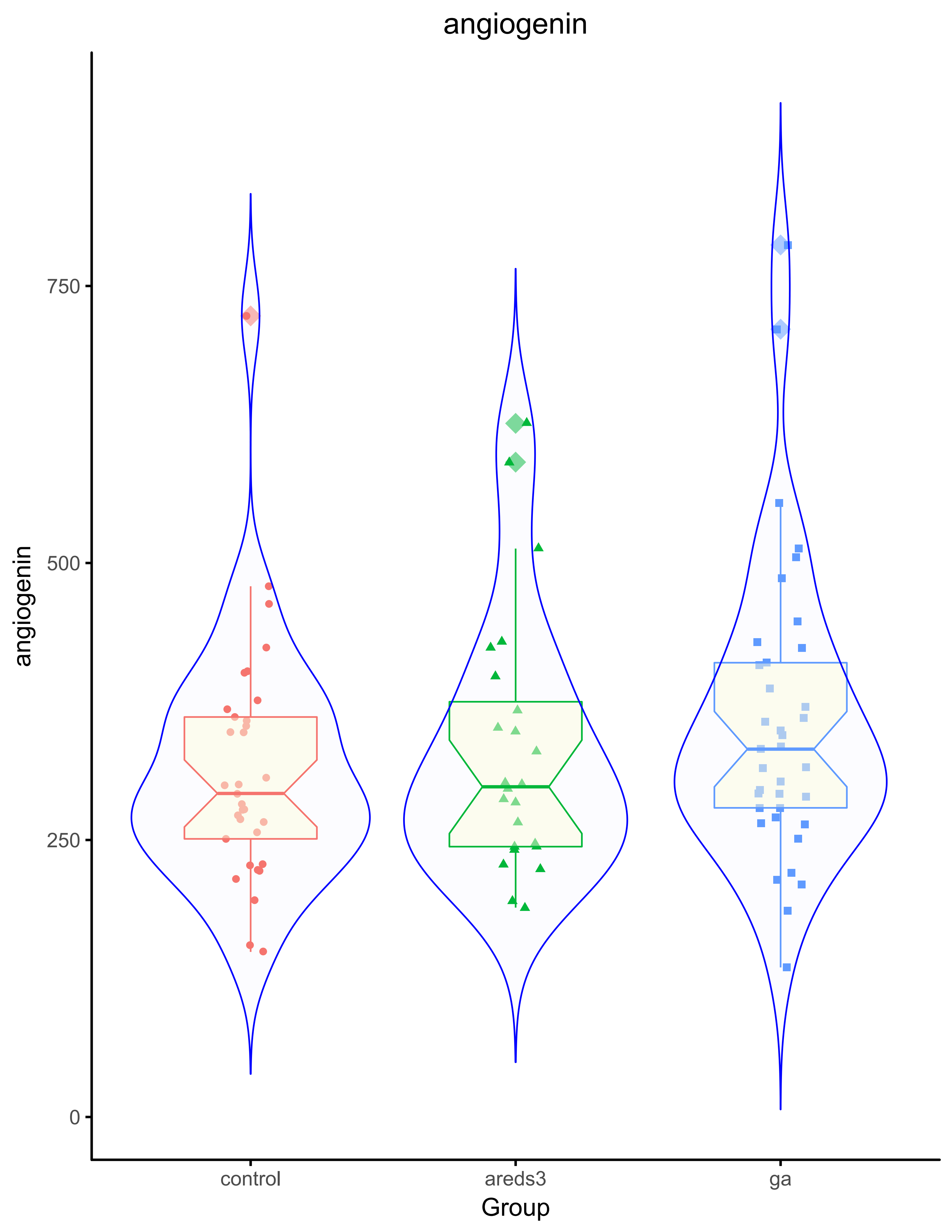

angiogenin
angiogenin
750
500
250
0
control
areds3
ga
Group

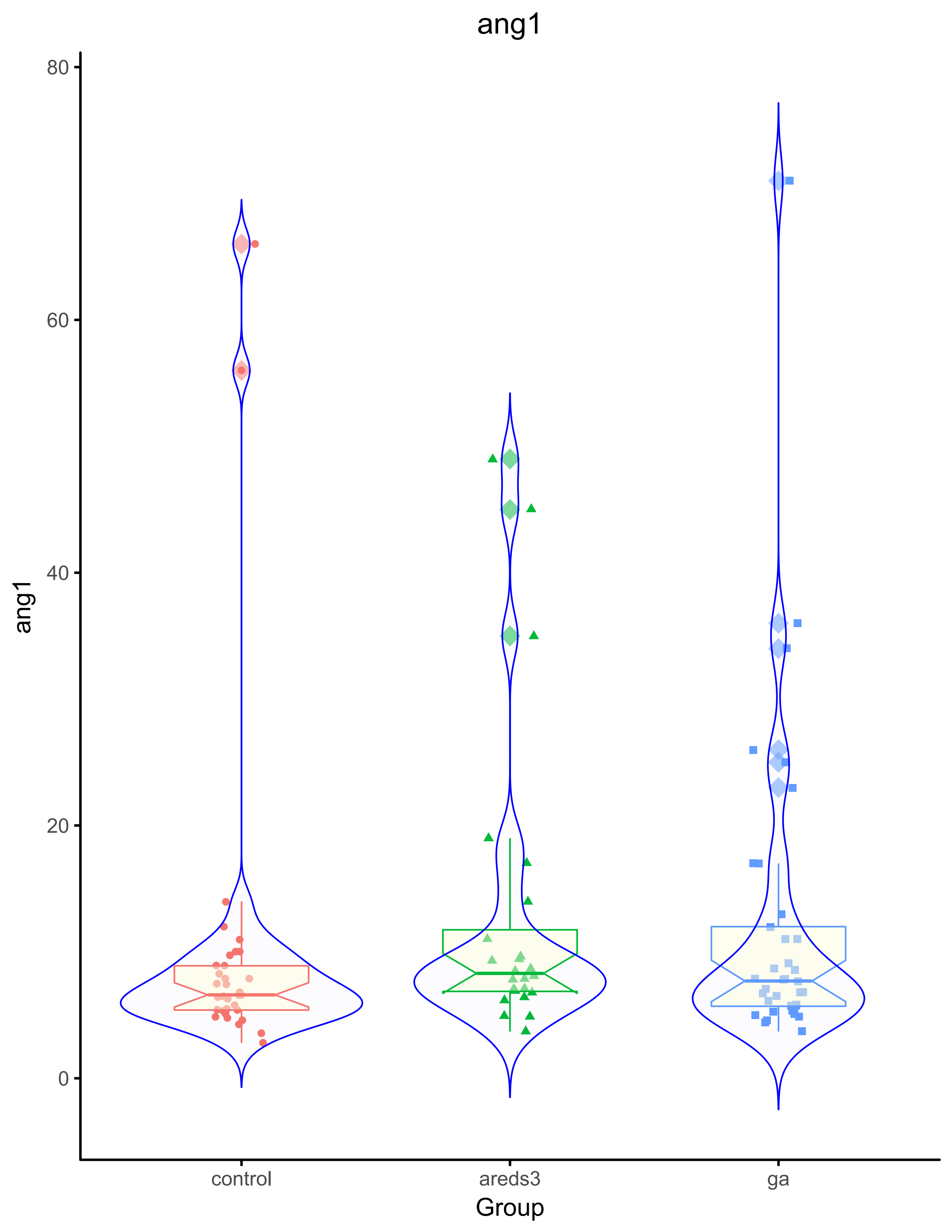
ang1
80
60
40
20
0
ang1
control
areds3
ga
Group

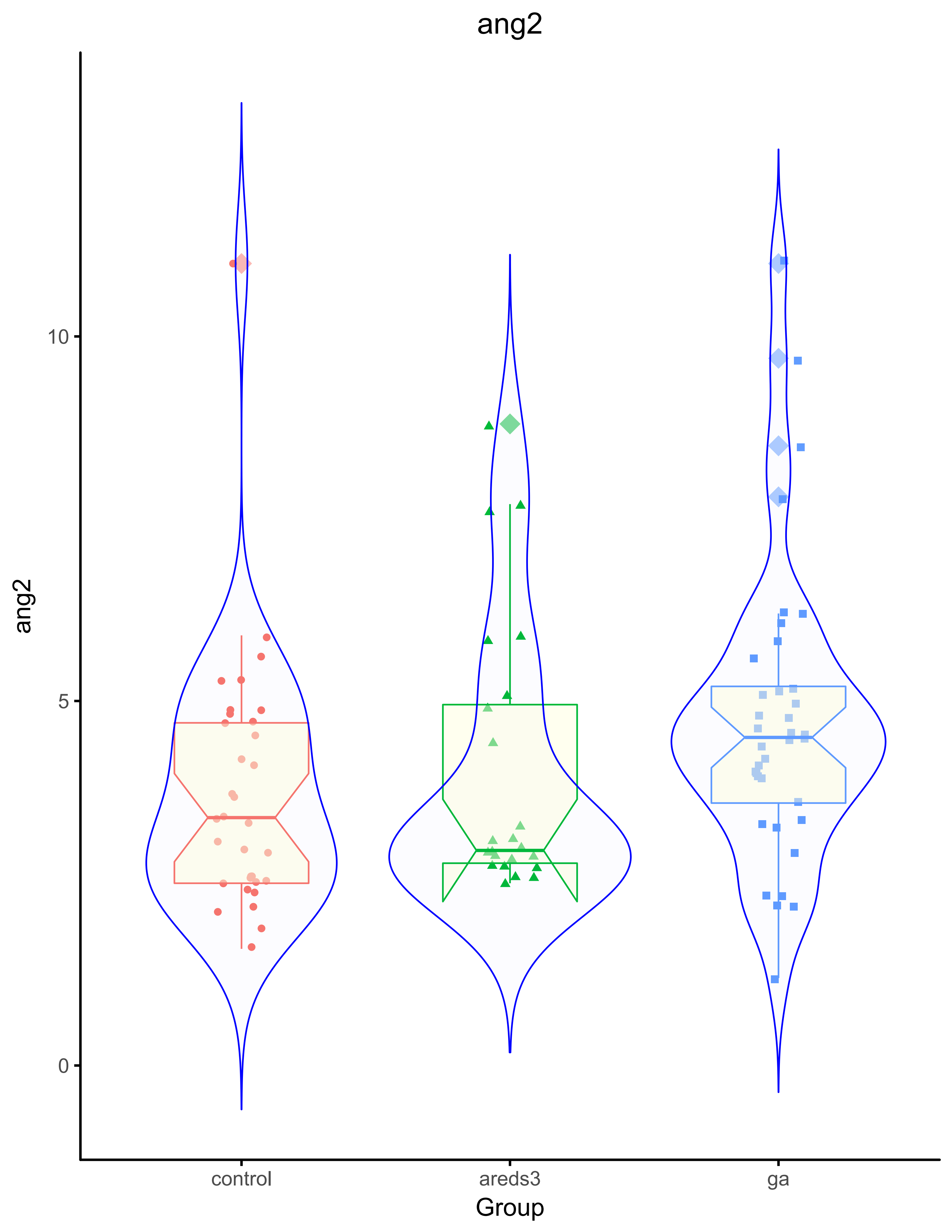

ang2
ang2
10
5
0
control
areds3
ga
Group

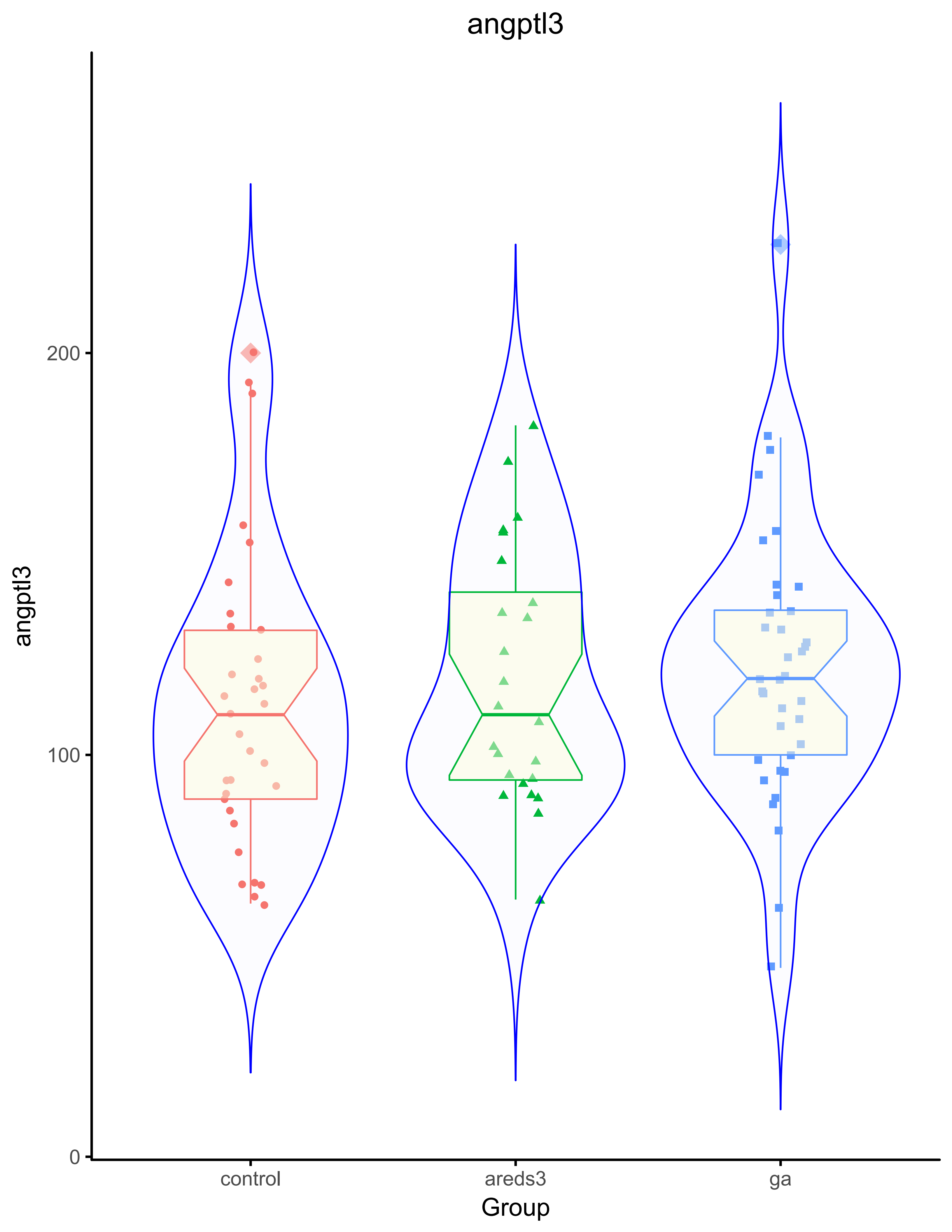
angptl3
angptl3
200
100
0
control
areds3
ga
Group

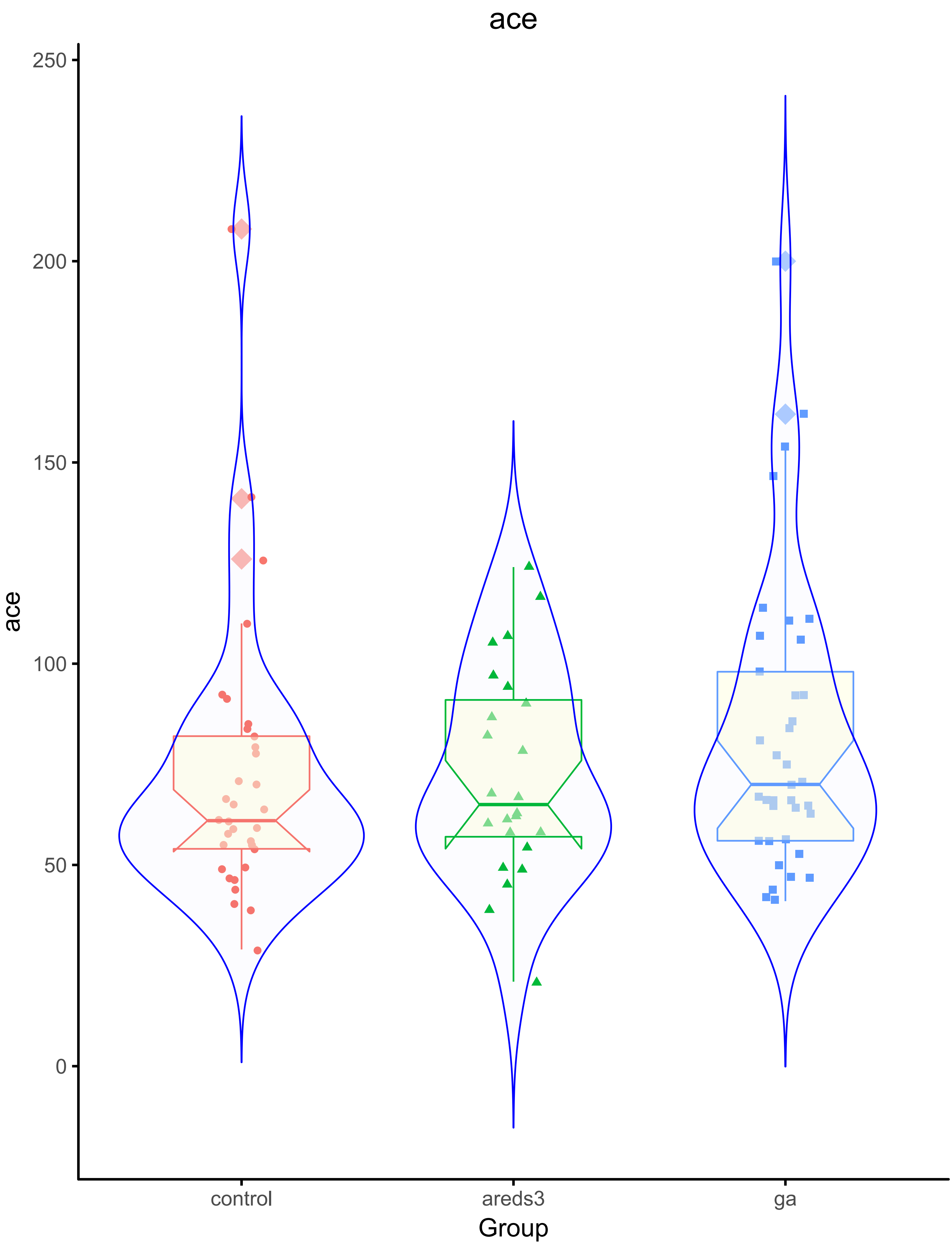
ace
250
200
150
100
50
0
ace
control
areds3
ga
Group

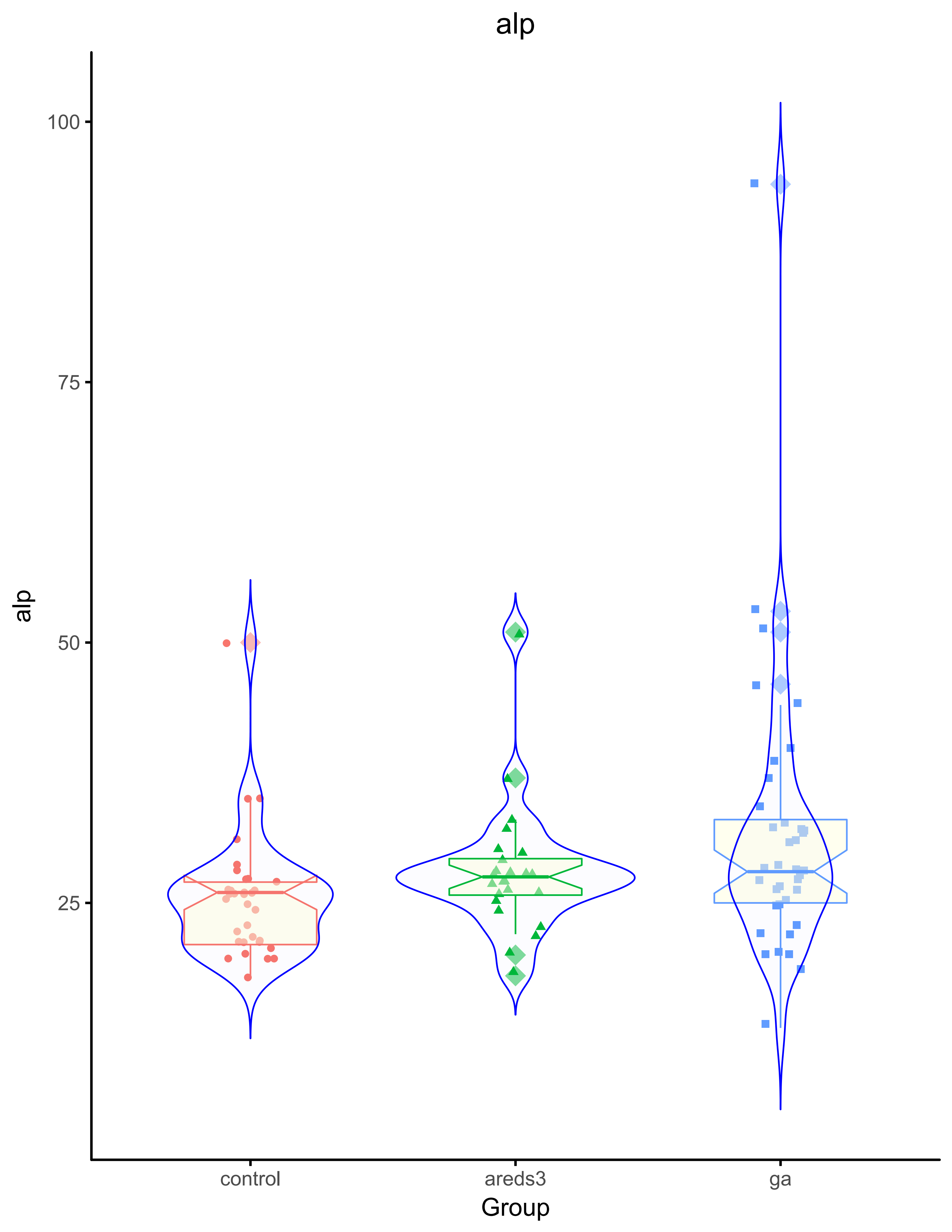

alp
100
75
50
25
alp
control
areds3
ga
Group

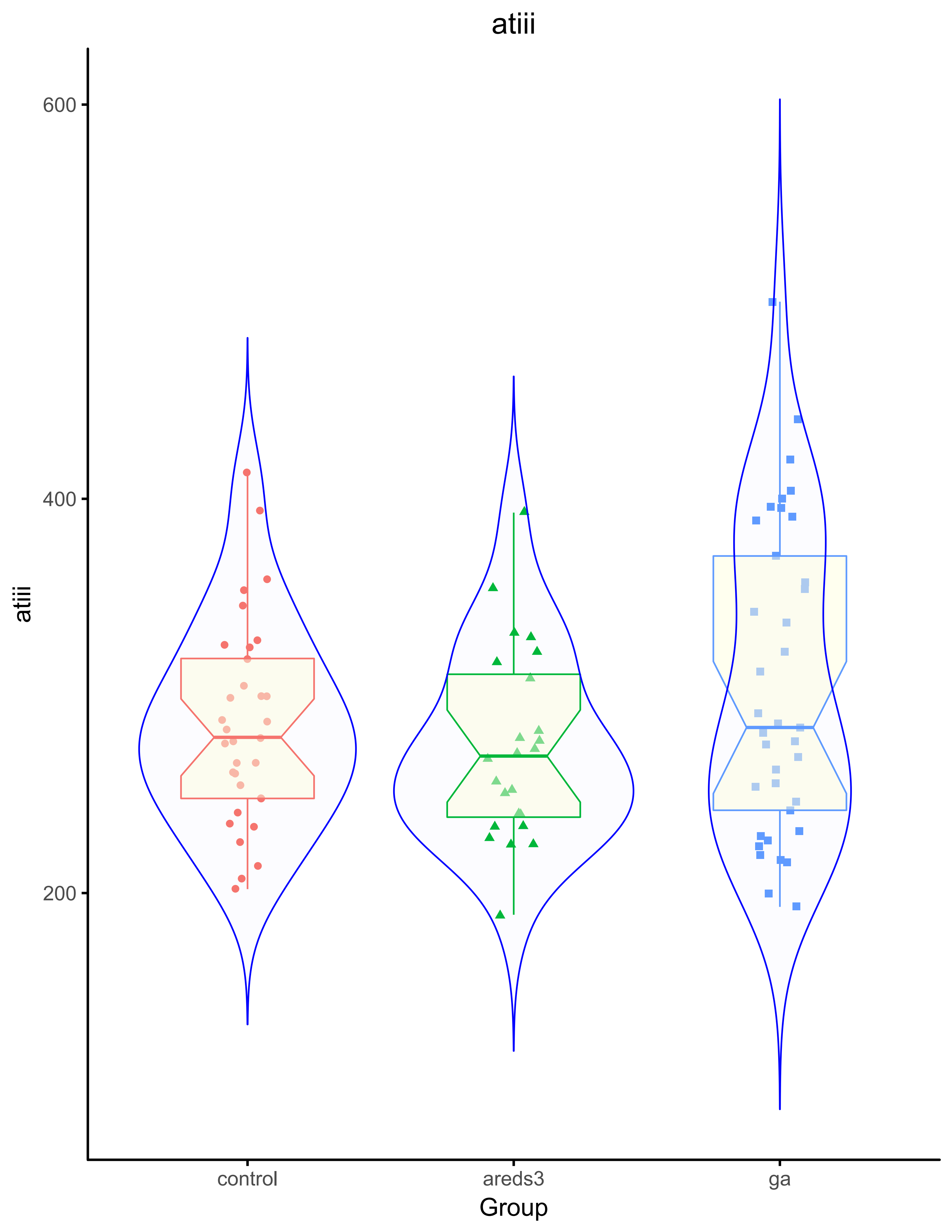
atiii
600
400
200
atiii
control
areds3
ga
Group

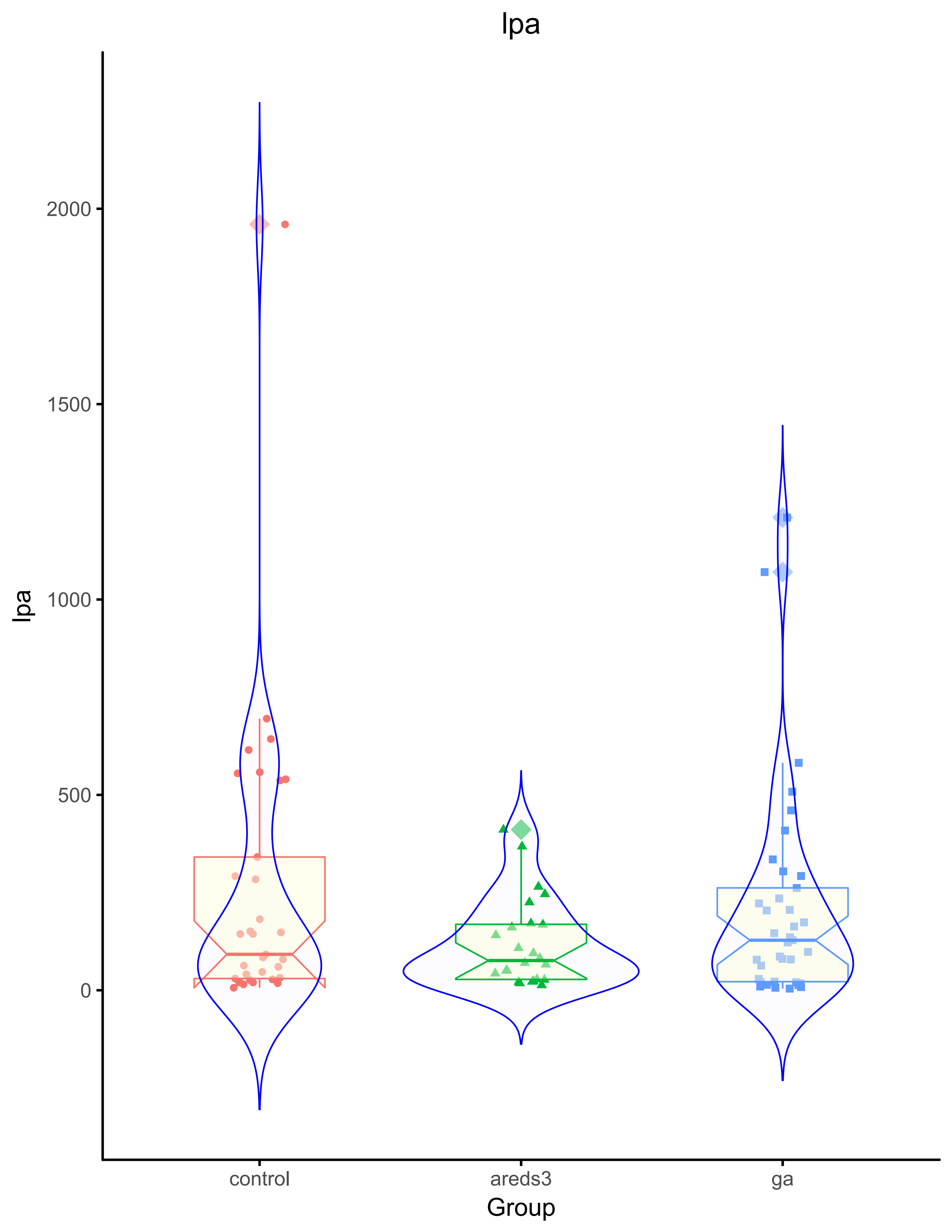

lpa
2000
1500
1000
500
0
lpa
control
areds3
ga
Group

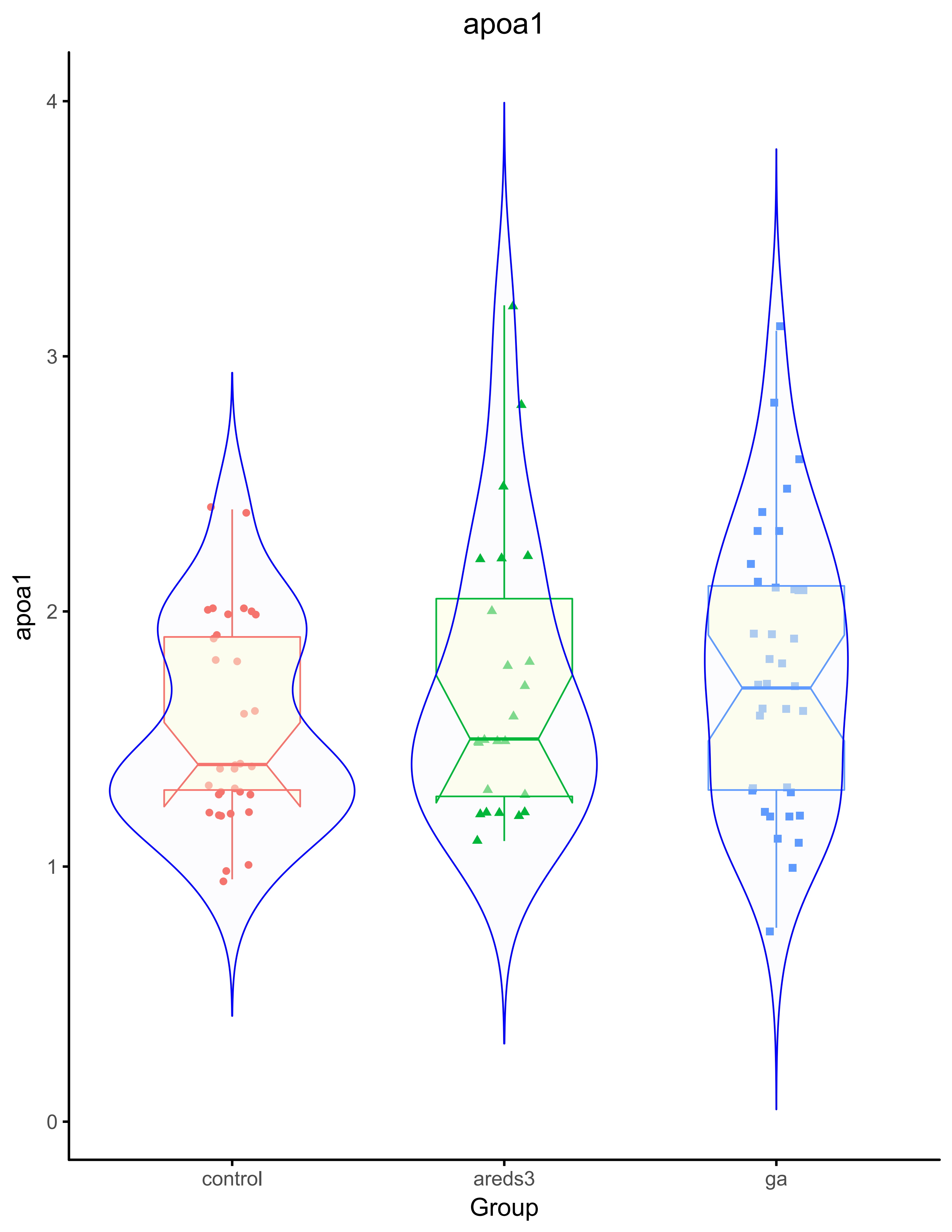
apoa1
4
3
2
1
0
apoa1
control
areds3
ga
Group

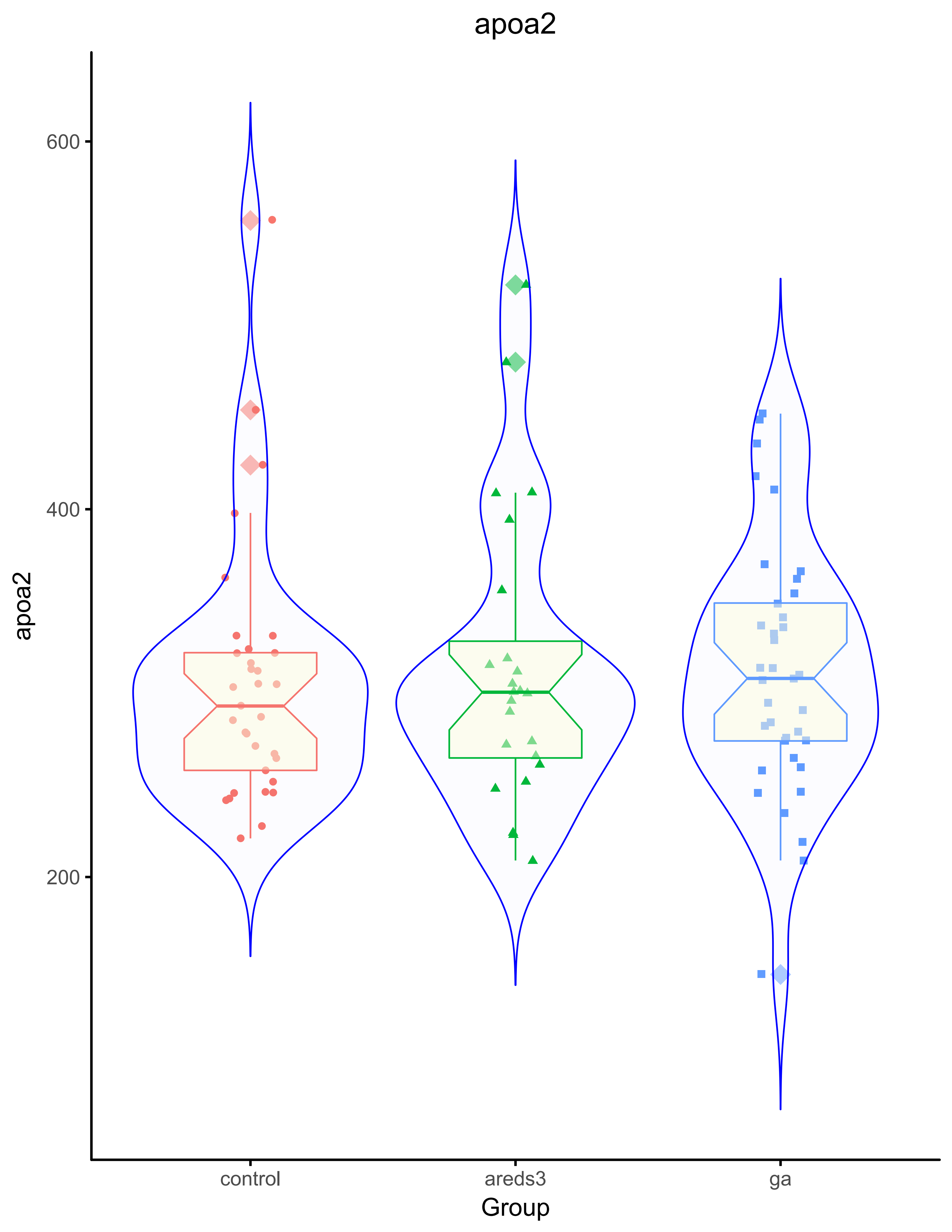

apoa2
600
400
200
apoa2
control
areds3
ga
Group

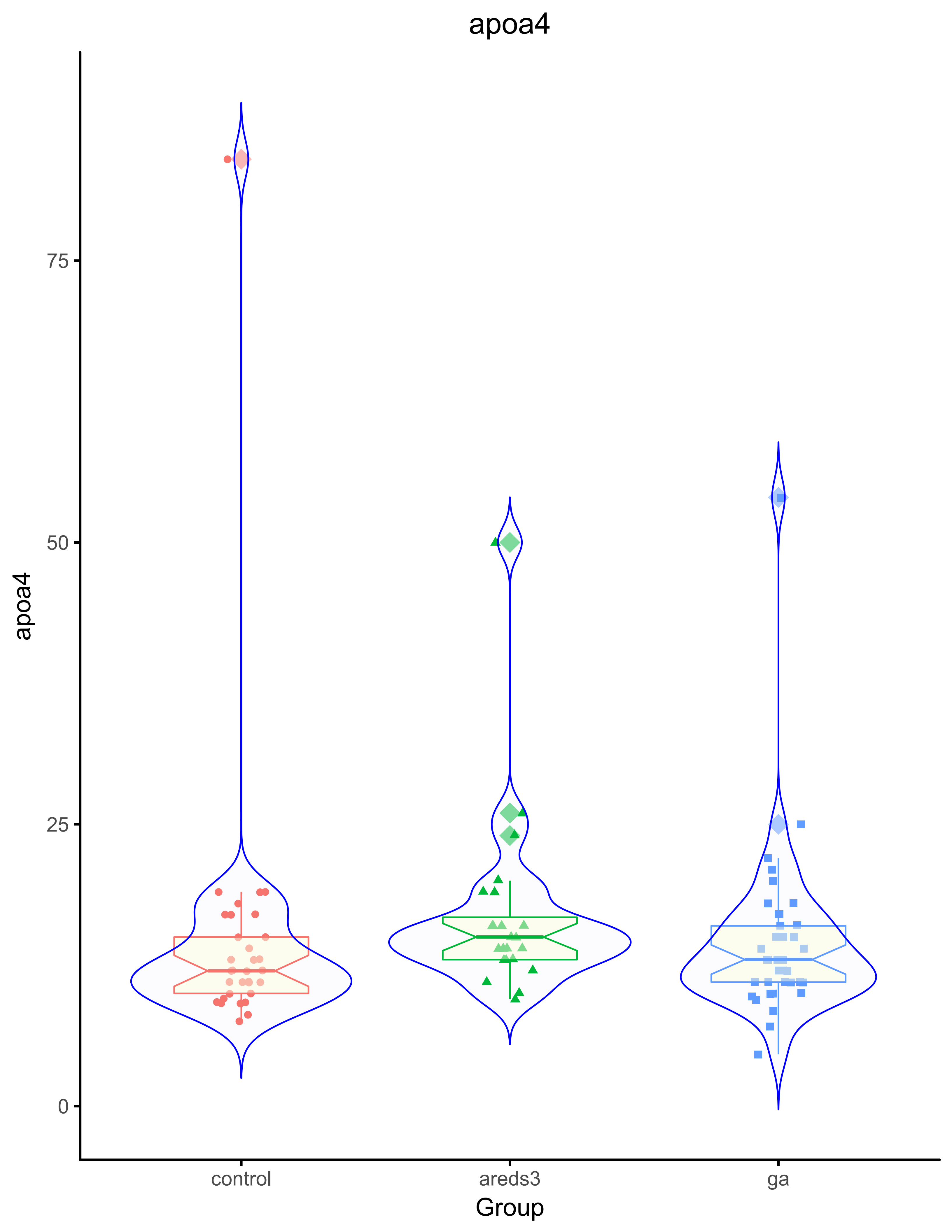
apoa4
75
50
25
0
apoa4
control
areds3
ga
Group

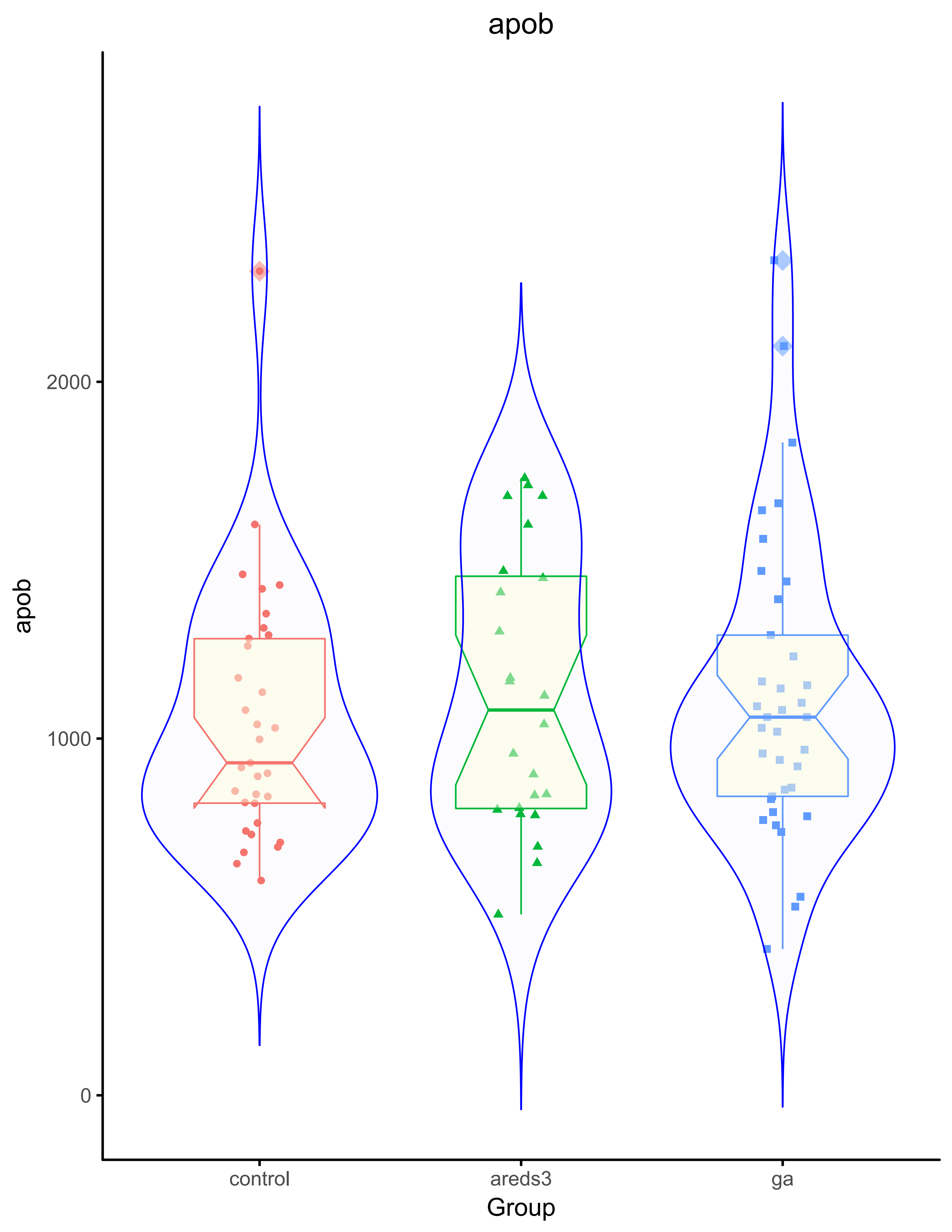
apob
2000
1000
0
apob
control
areds3
ga
Group

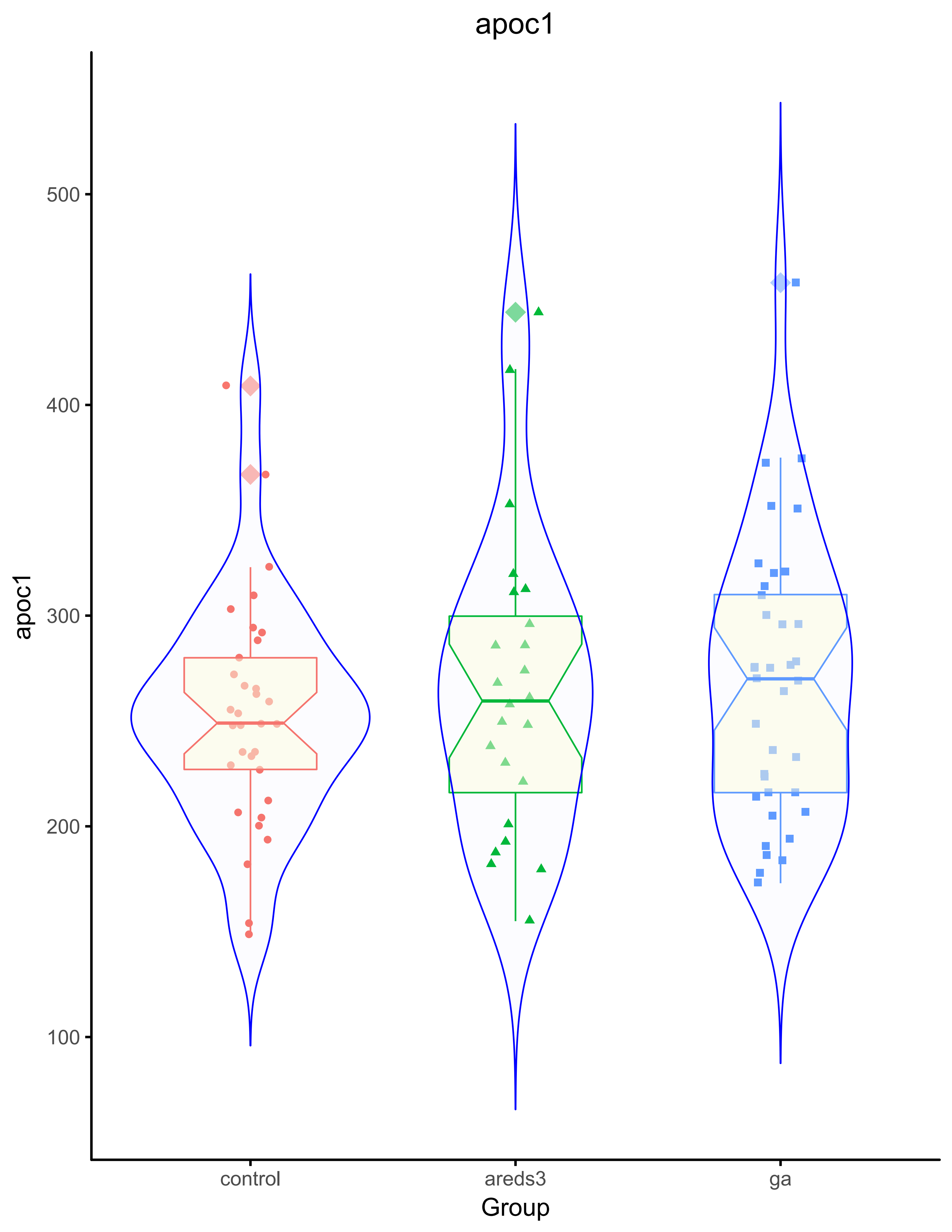
apoc1
500
400
300
200
100
apoc1
control
areds3
ga
Group

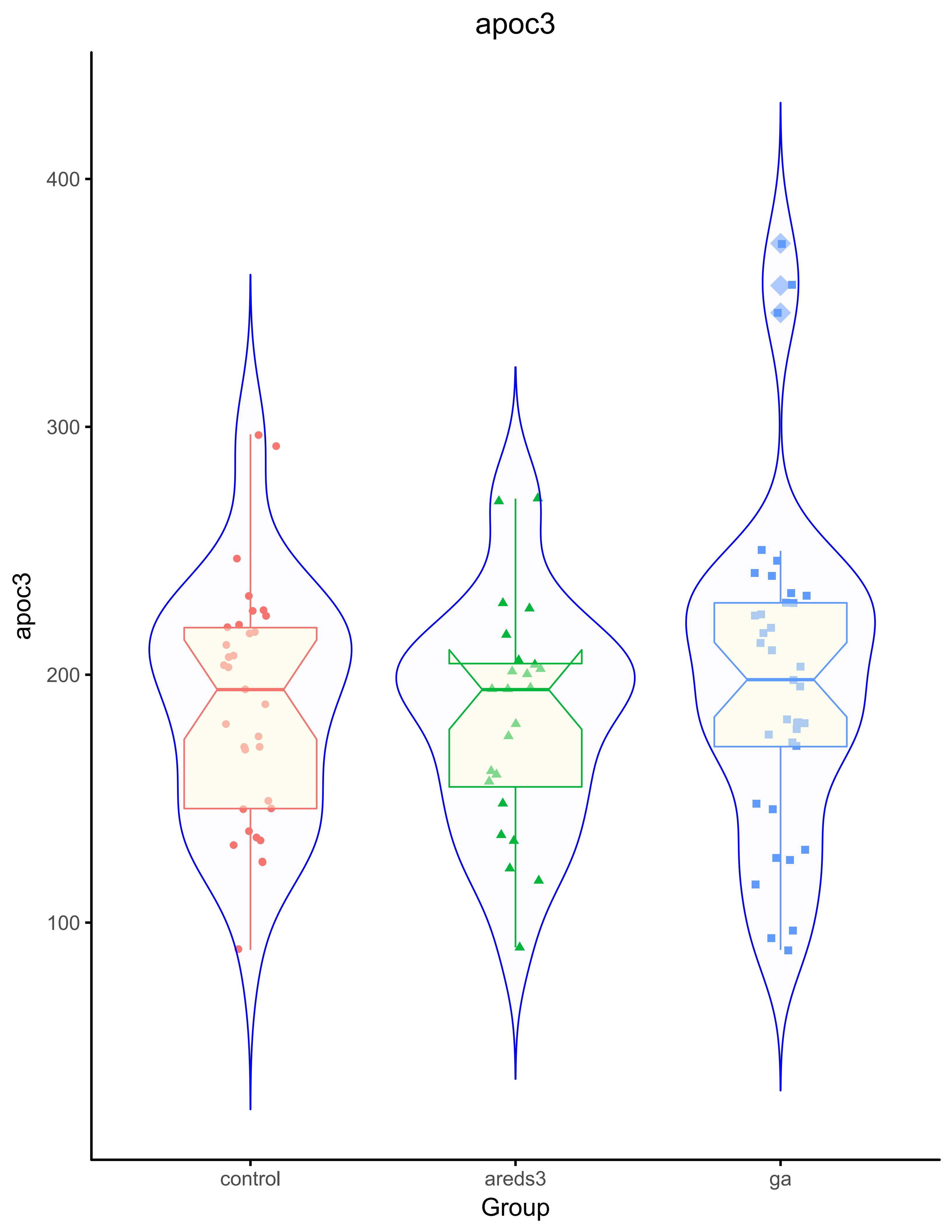

apoc3
400
300
200
100
apoc3
control
areds3
ga
Group

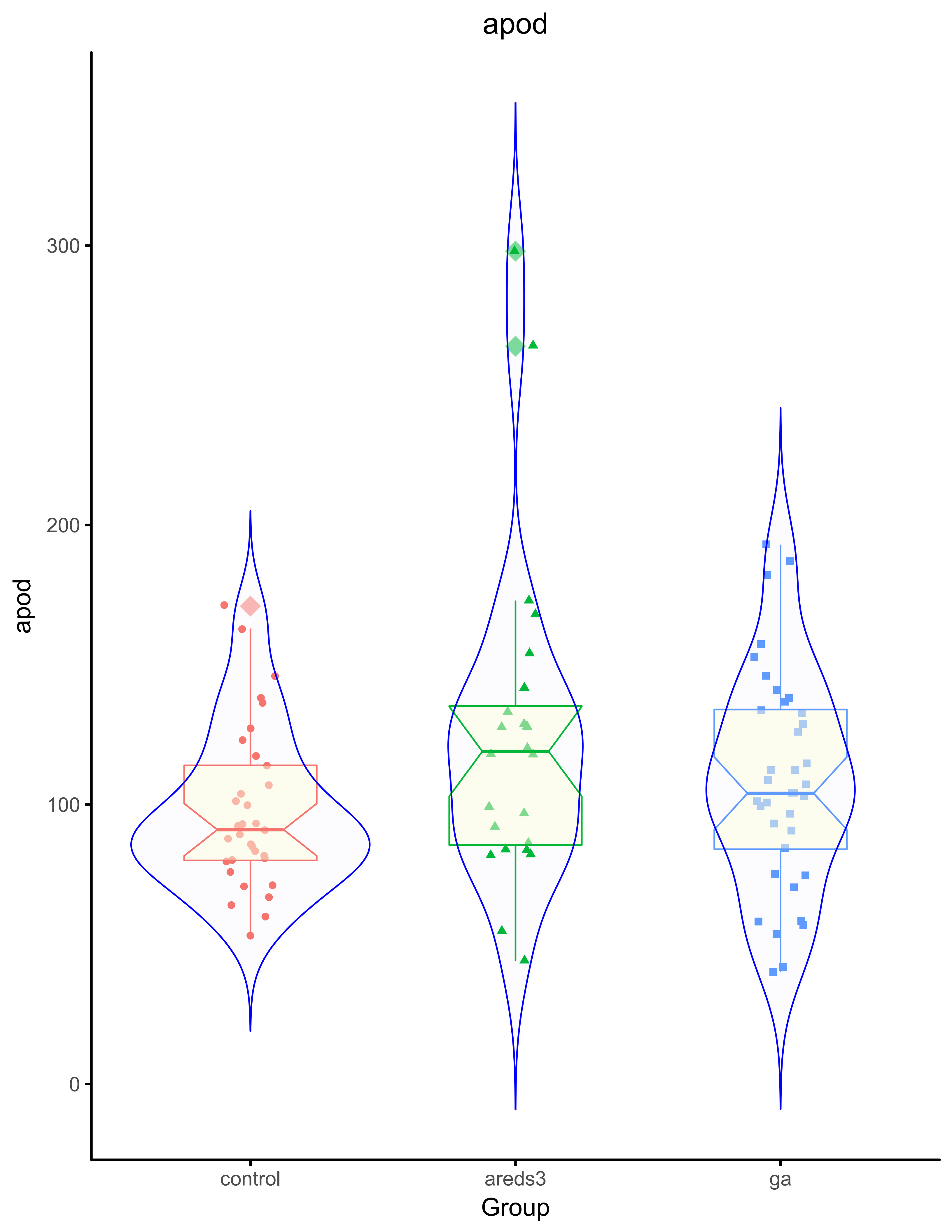
apod
apod
300
200
100
0
control
areds3
ga
Group

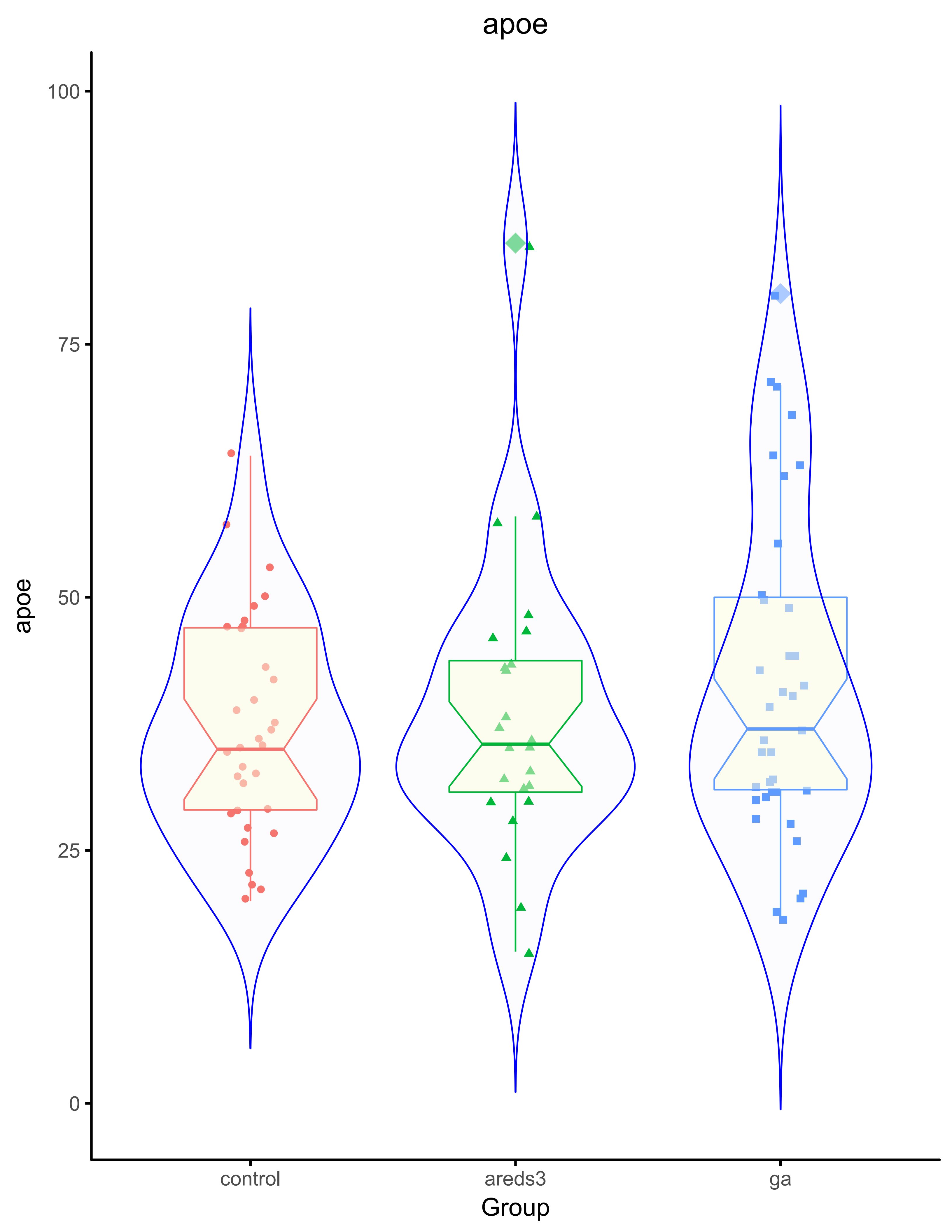

apoe
100
75
50
25
0
apoe
control
areds3
ga
Group

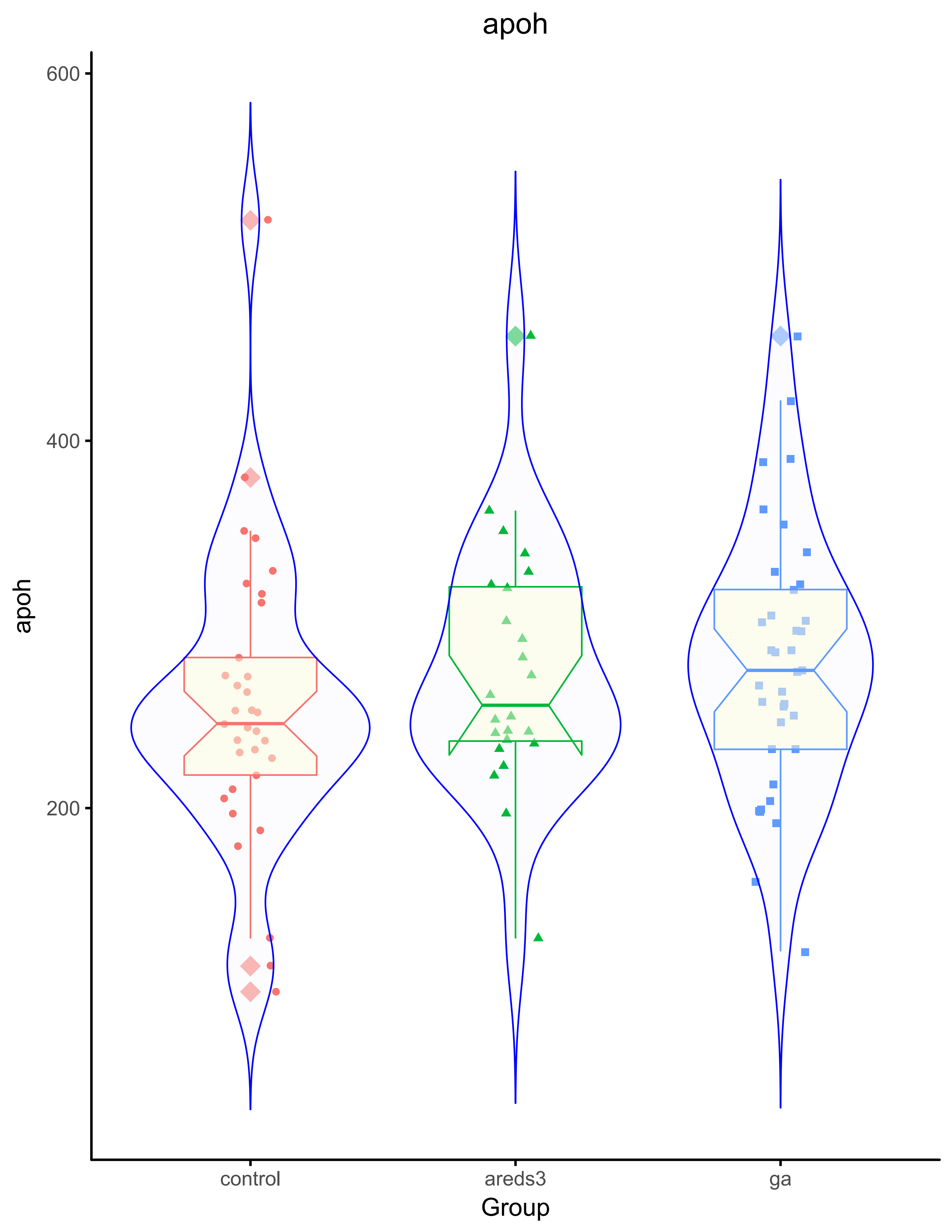

apoh
600
400
200
apoh
control
areds3
ga
Group

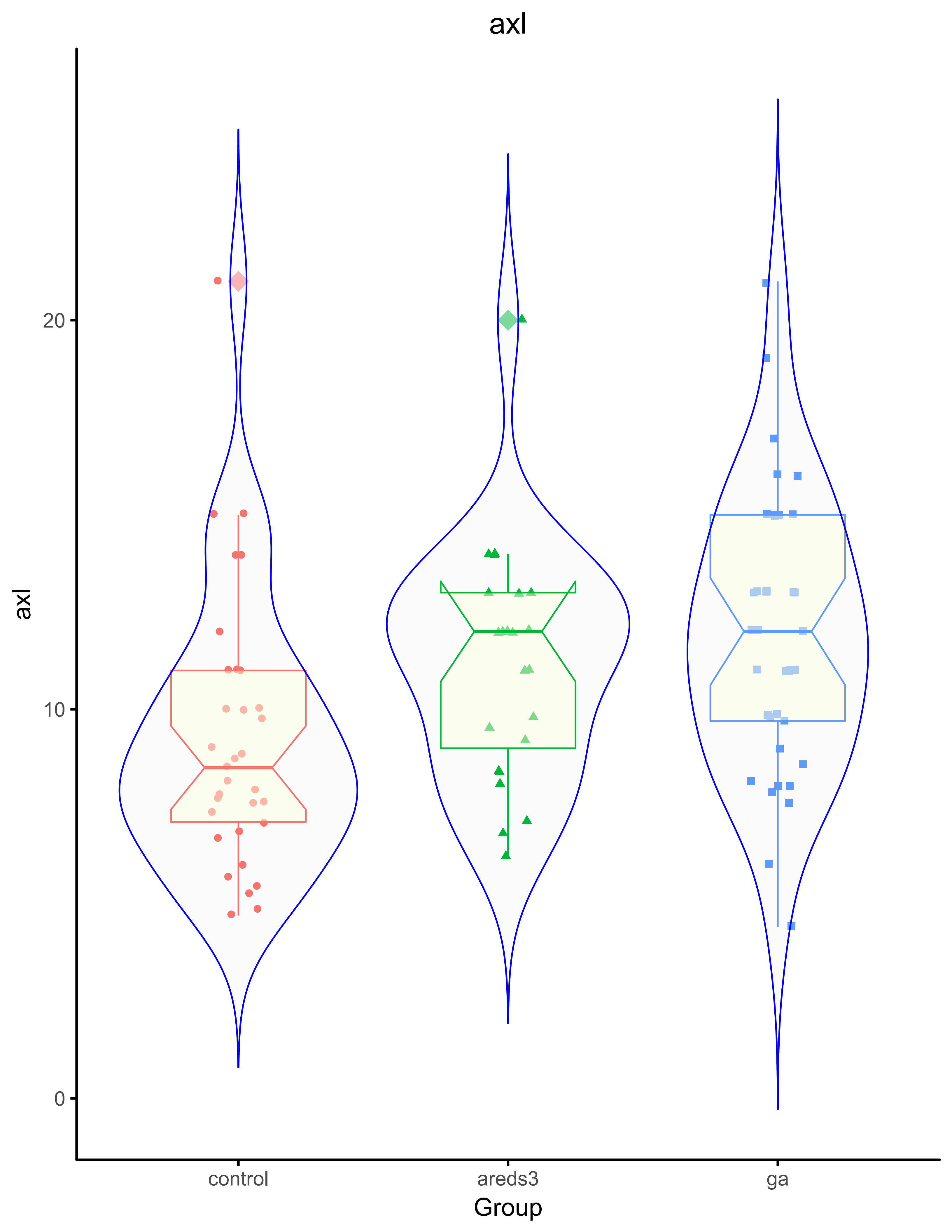
axl
20
10
0
axl
control
areds3
ga
Group

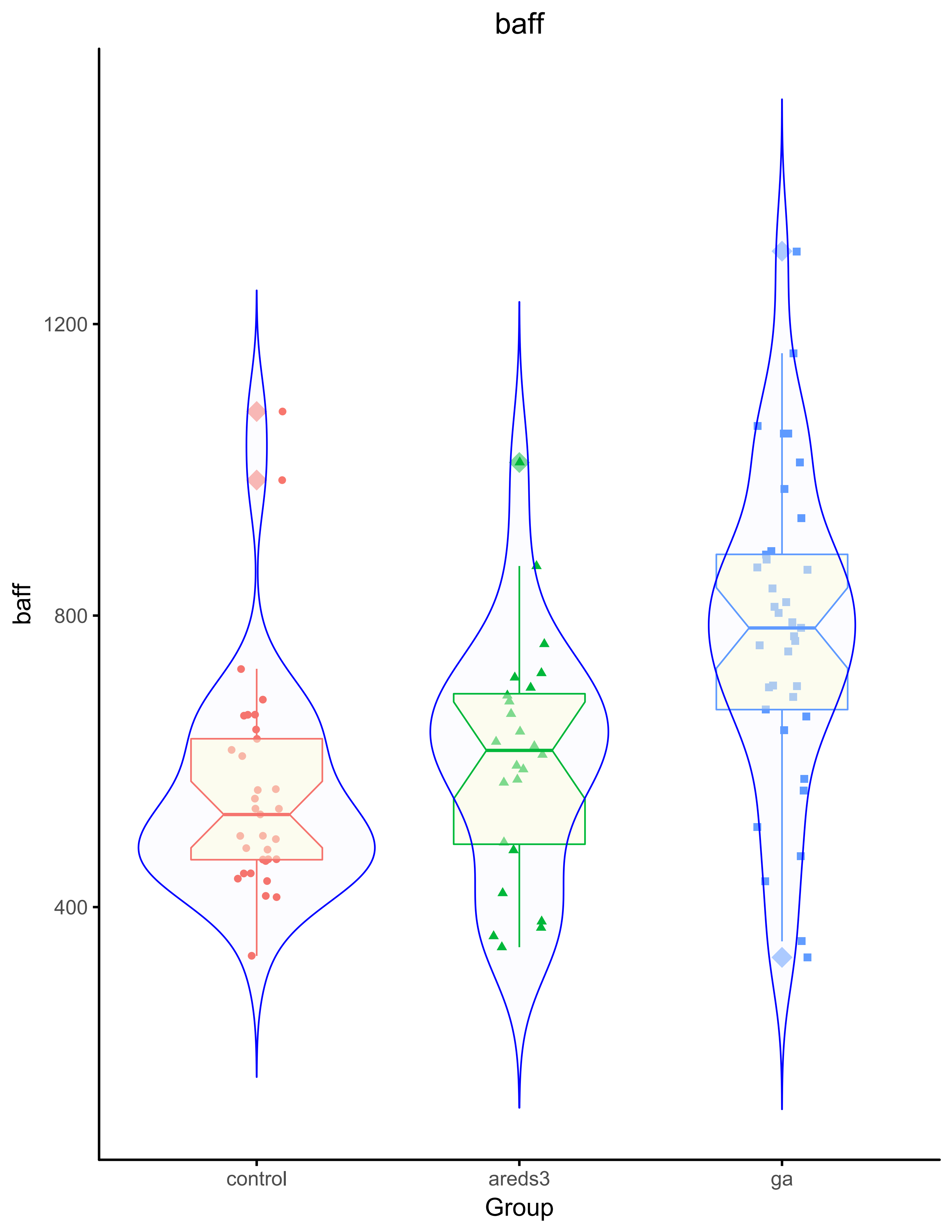

baff
1200
800
400
baff
control
areds3
ga
Group

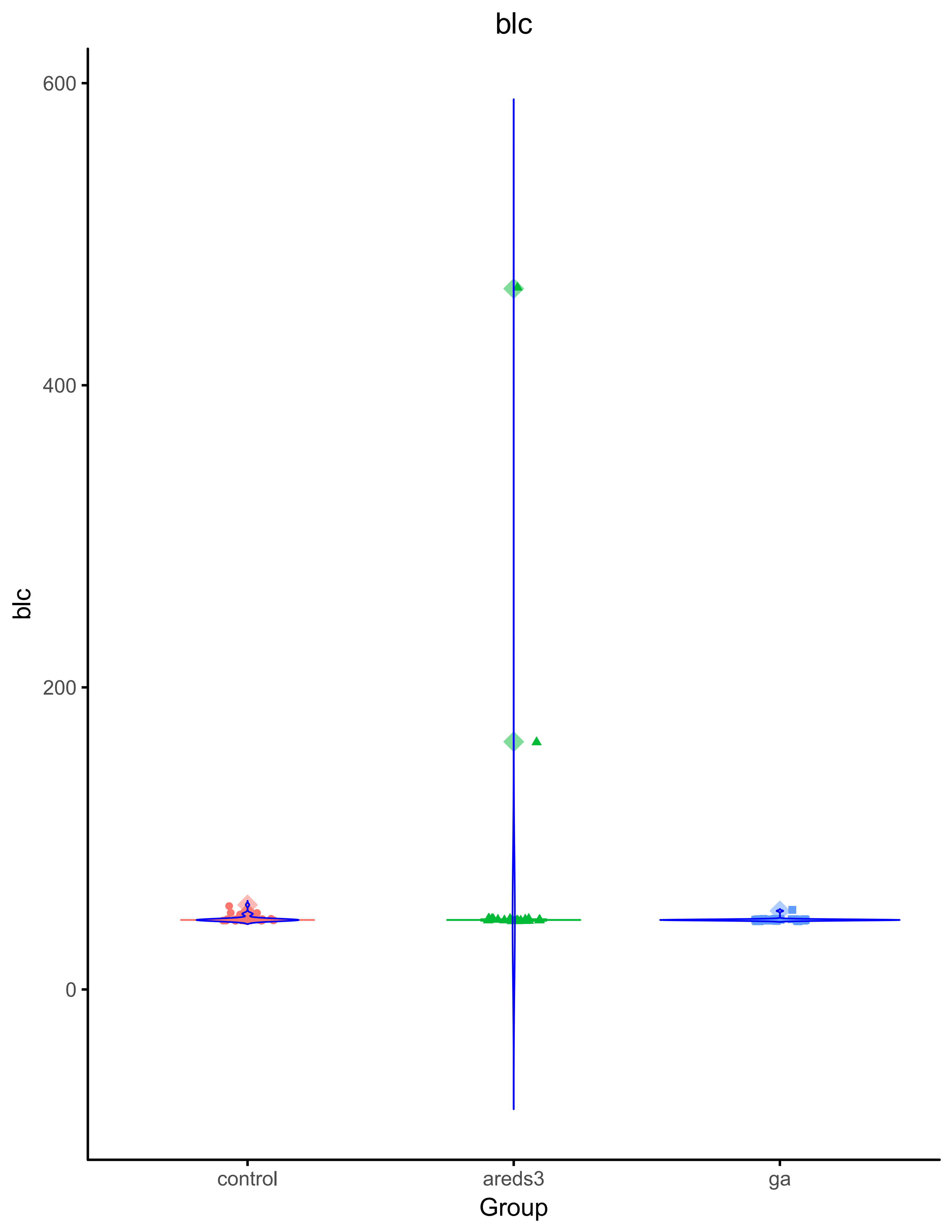
blc
600
400
200
0
blc
control
areds3
ga
Group

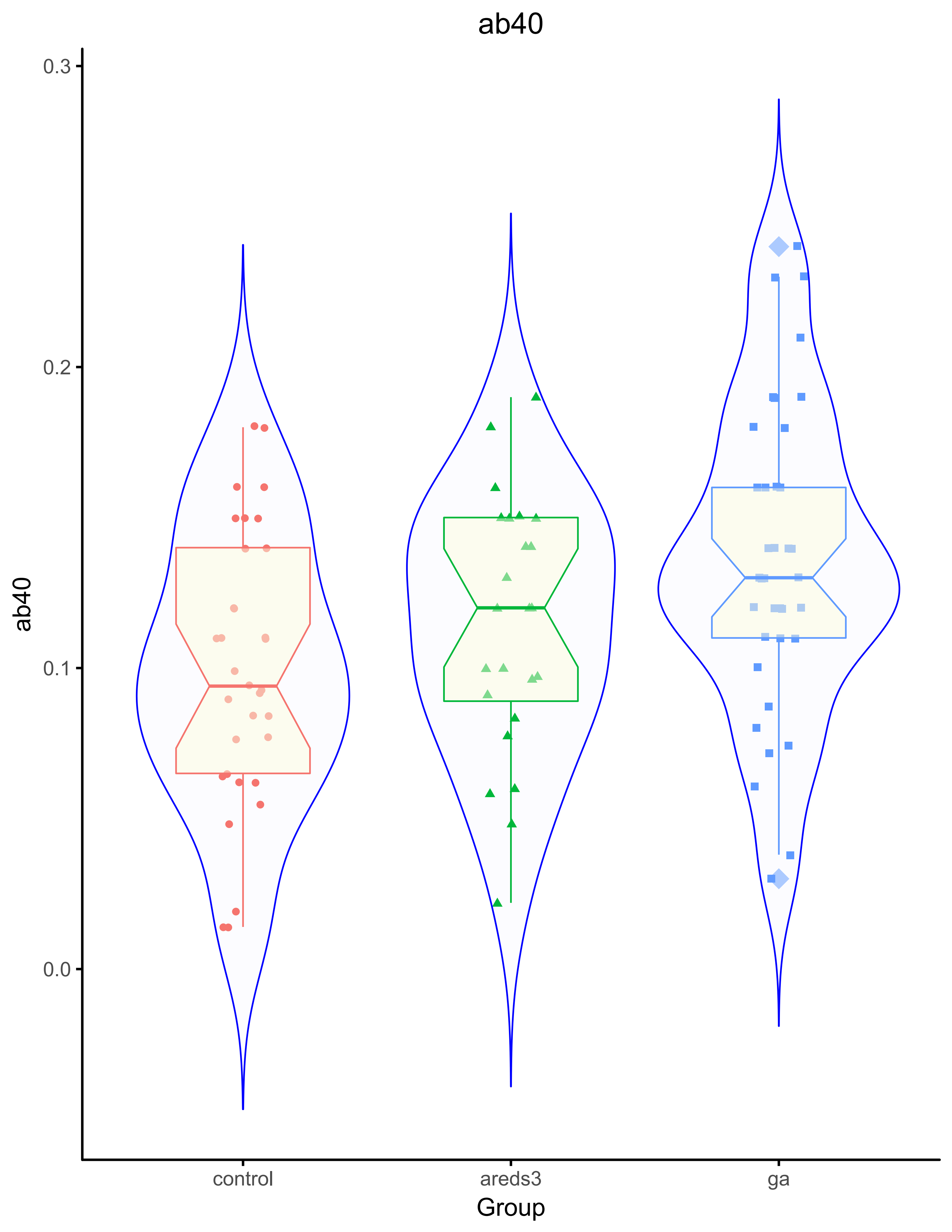
ab40
0.3
0.2
0.1
0.0
ab40
control
areds3
ga
Group

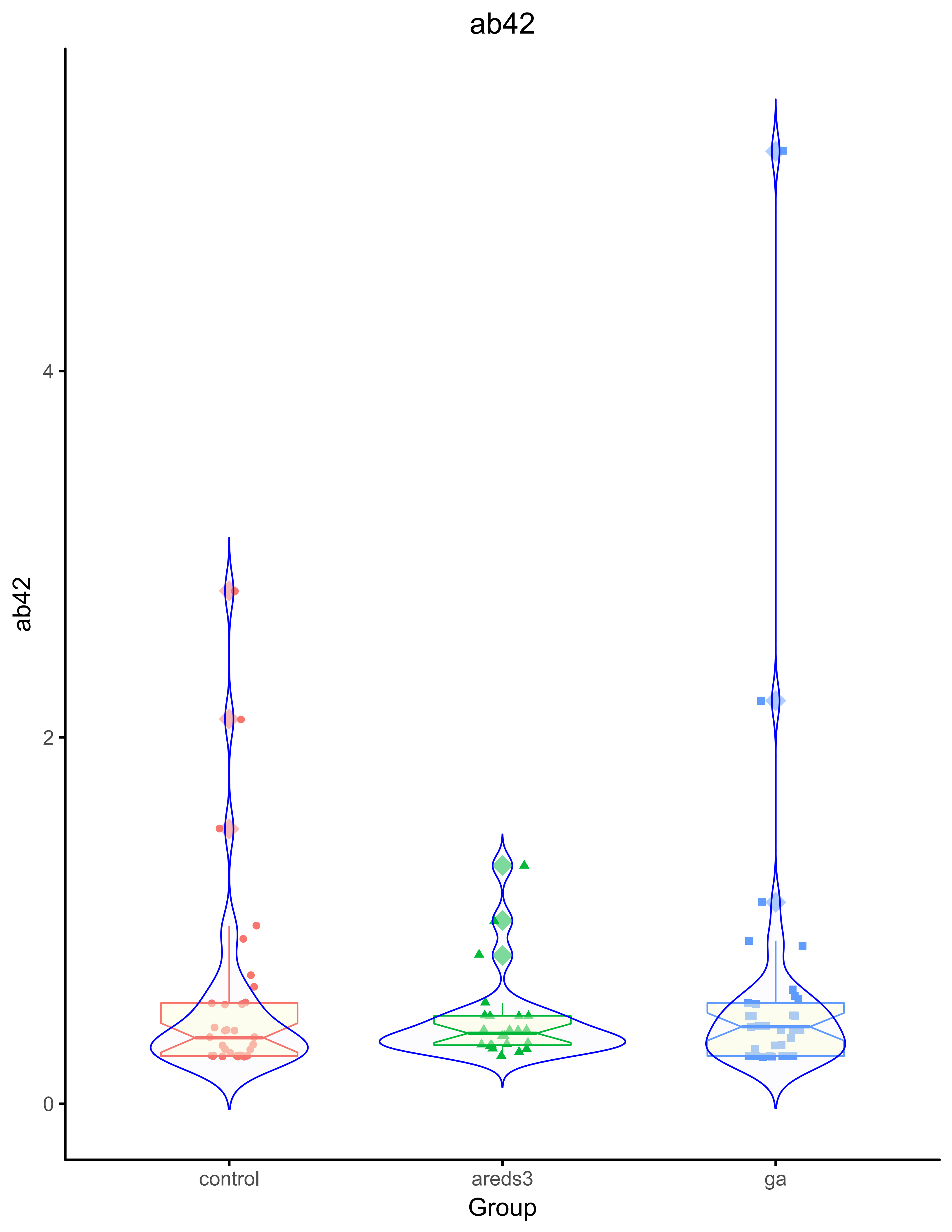
ab42
ab42
0
2
4
control
areds3
ga
Group

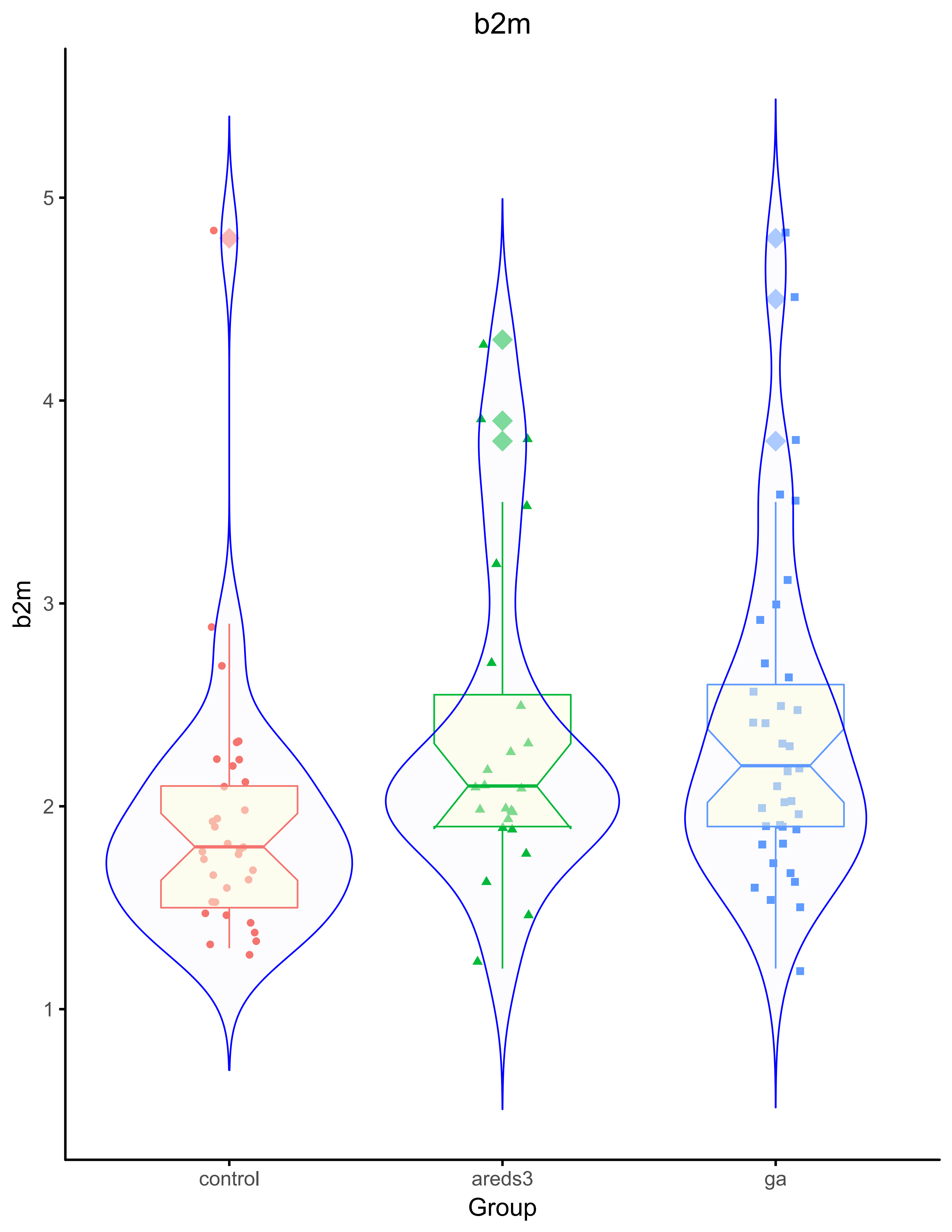

b2m
5
4
3
2
1
b2m
control
areds3
ga
Group

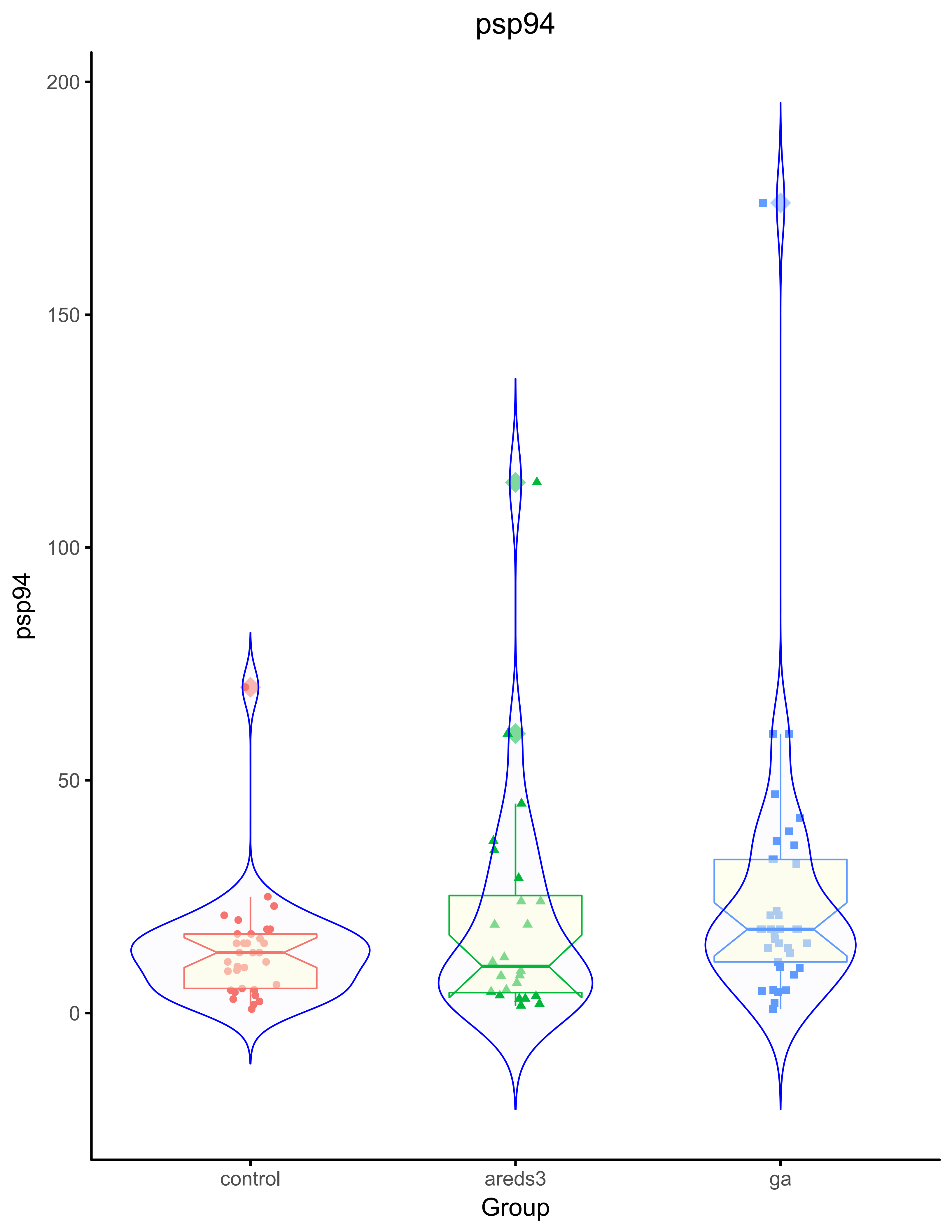
psp94
200
150
100
50
0
psp94
control
areds3
ga
Group

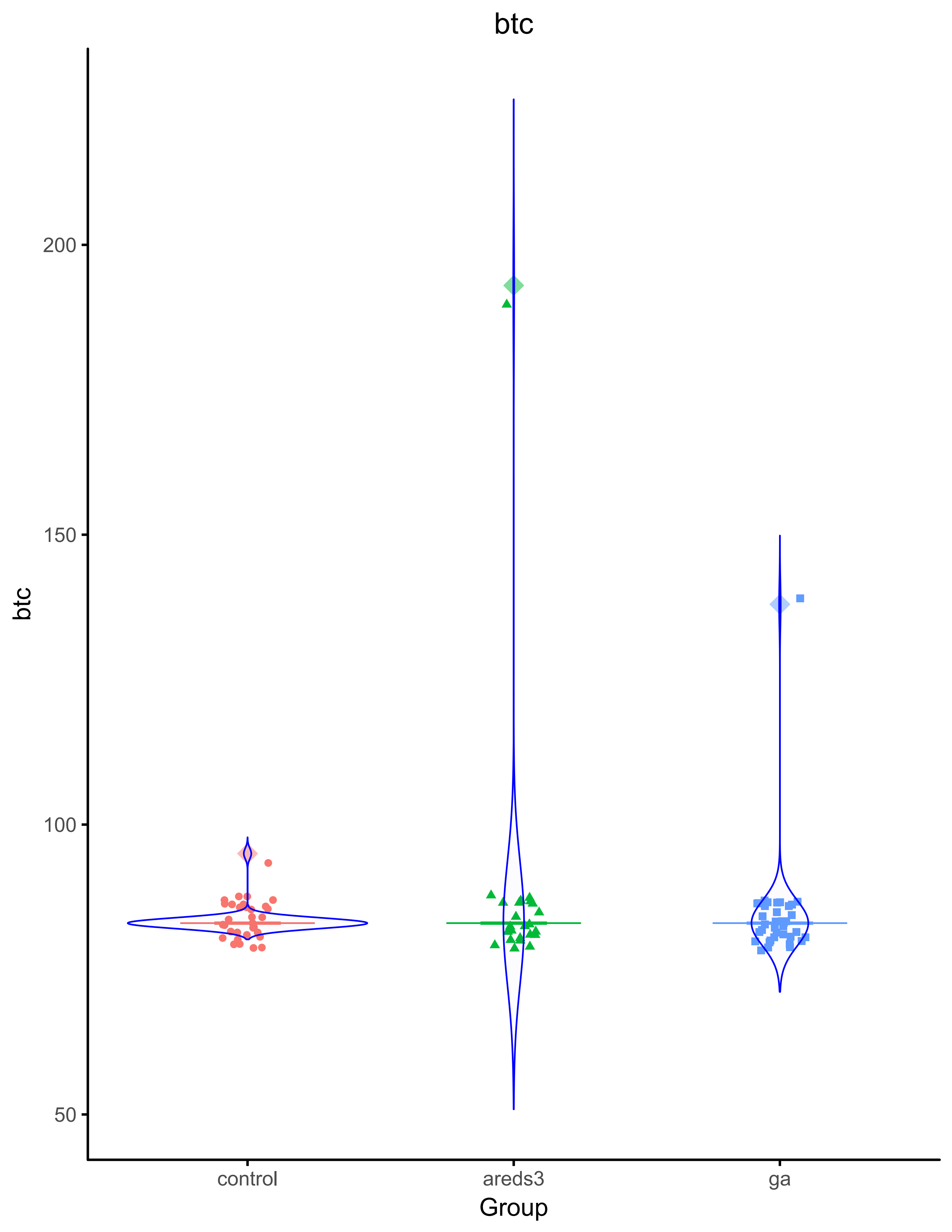

btc
btc
200
150
100
50
control
areds3
ga
Group

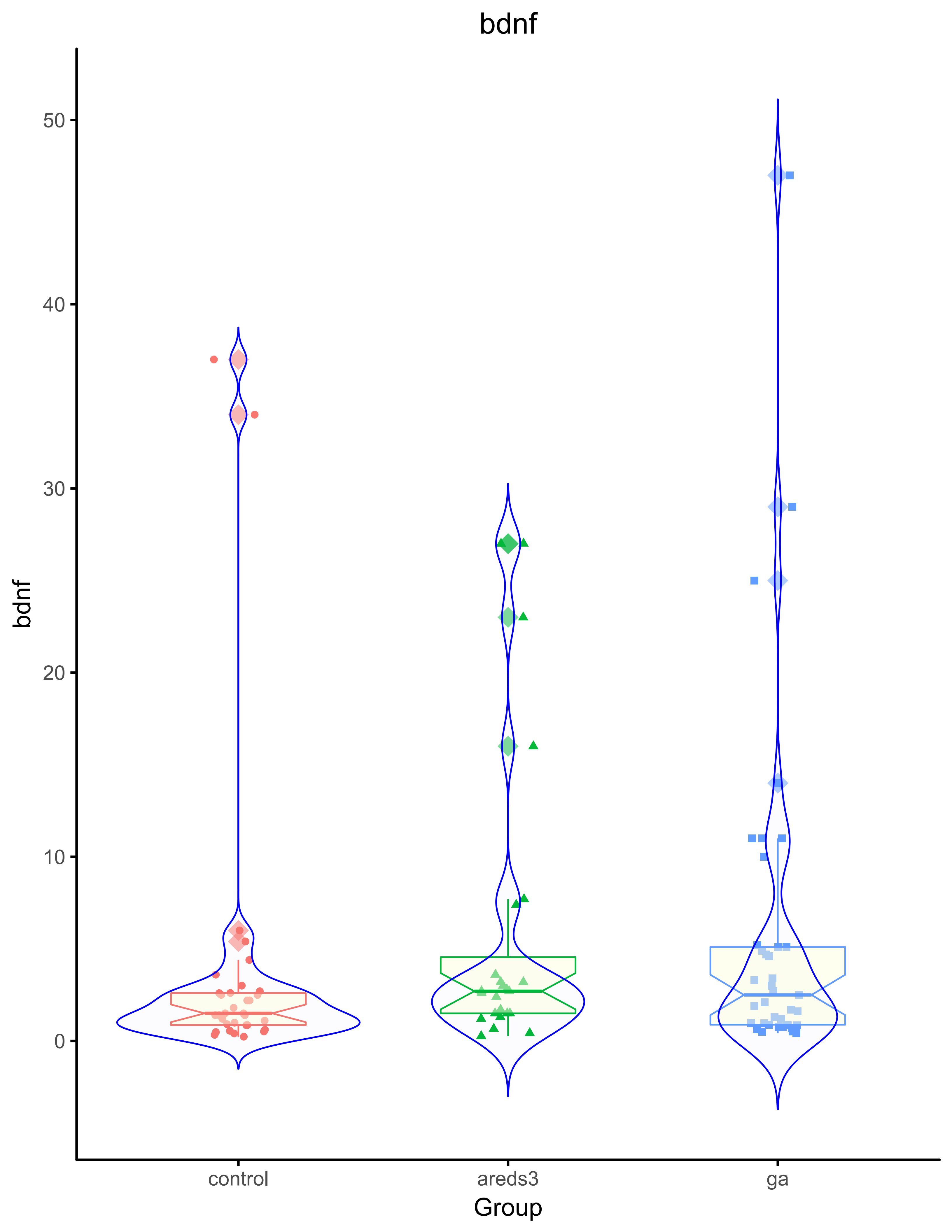
bdnf
bdnf
50
40
30
20
10
0
control
areds3
ga
Group

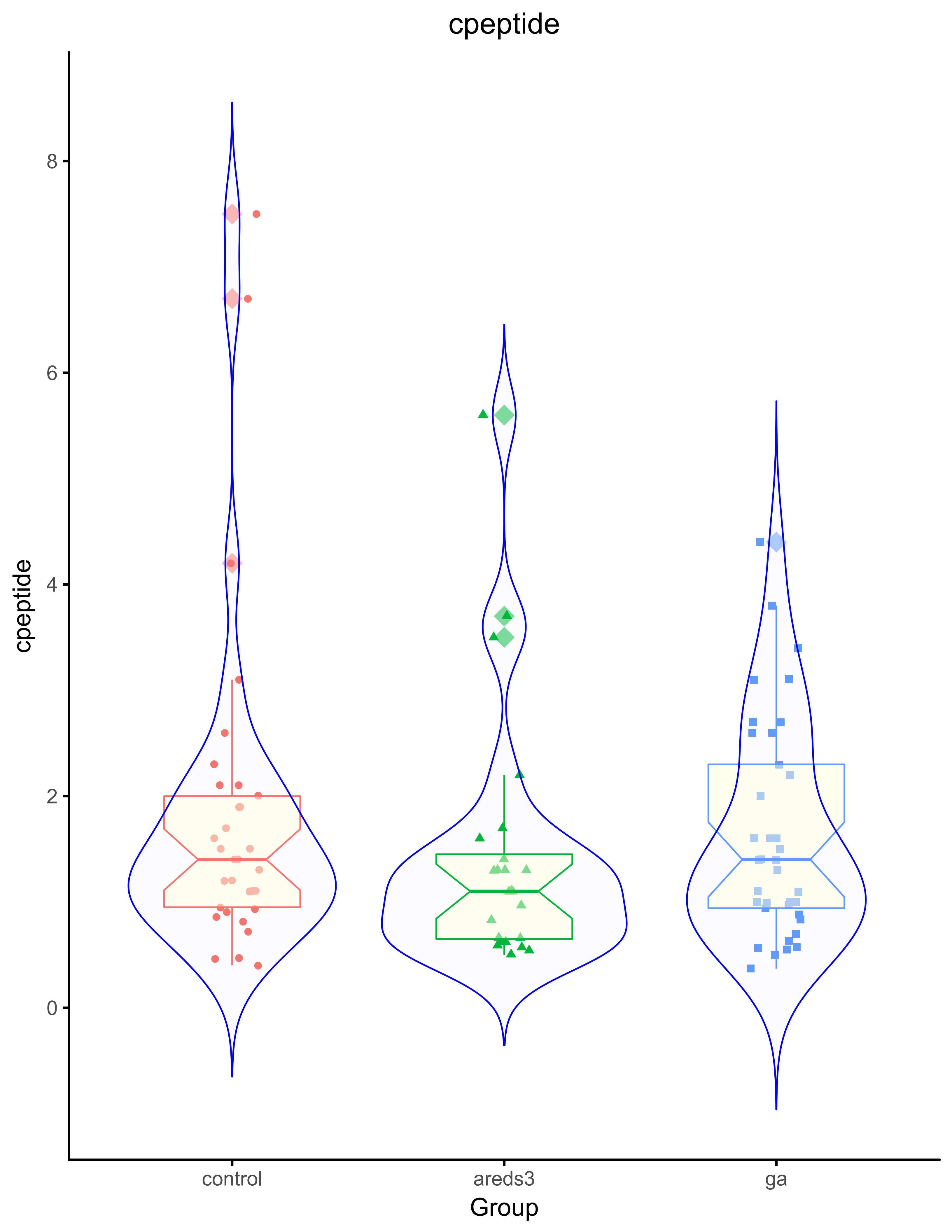
cpeptide
cpeptide
0
2
4
6
8
control
areds3
ga
Group

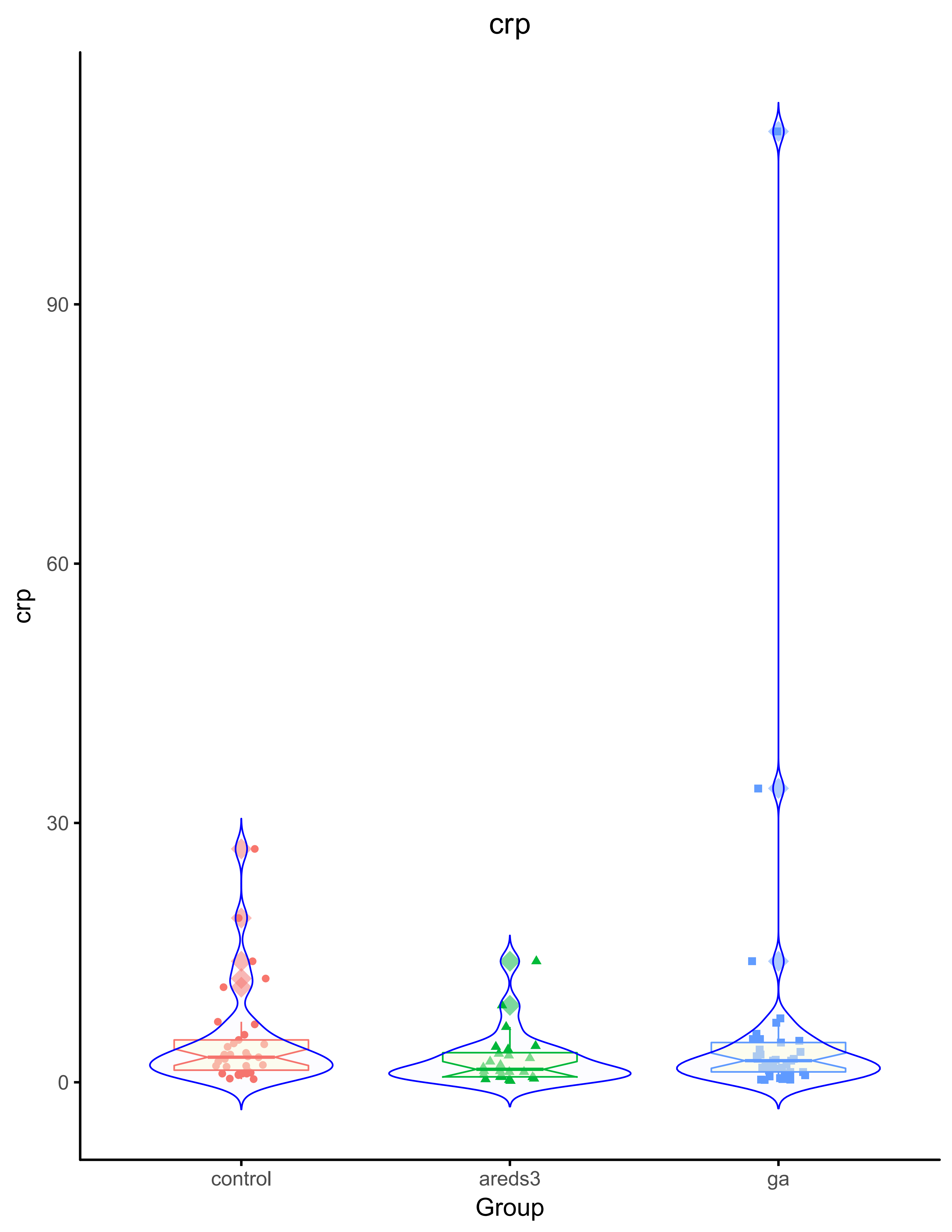
crp
90
60
30
0
crp
control
areds3
ga
Group

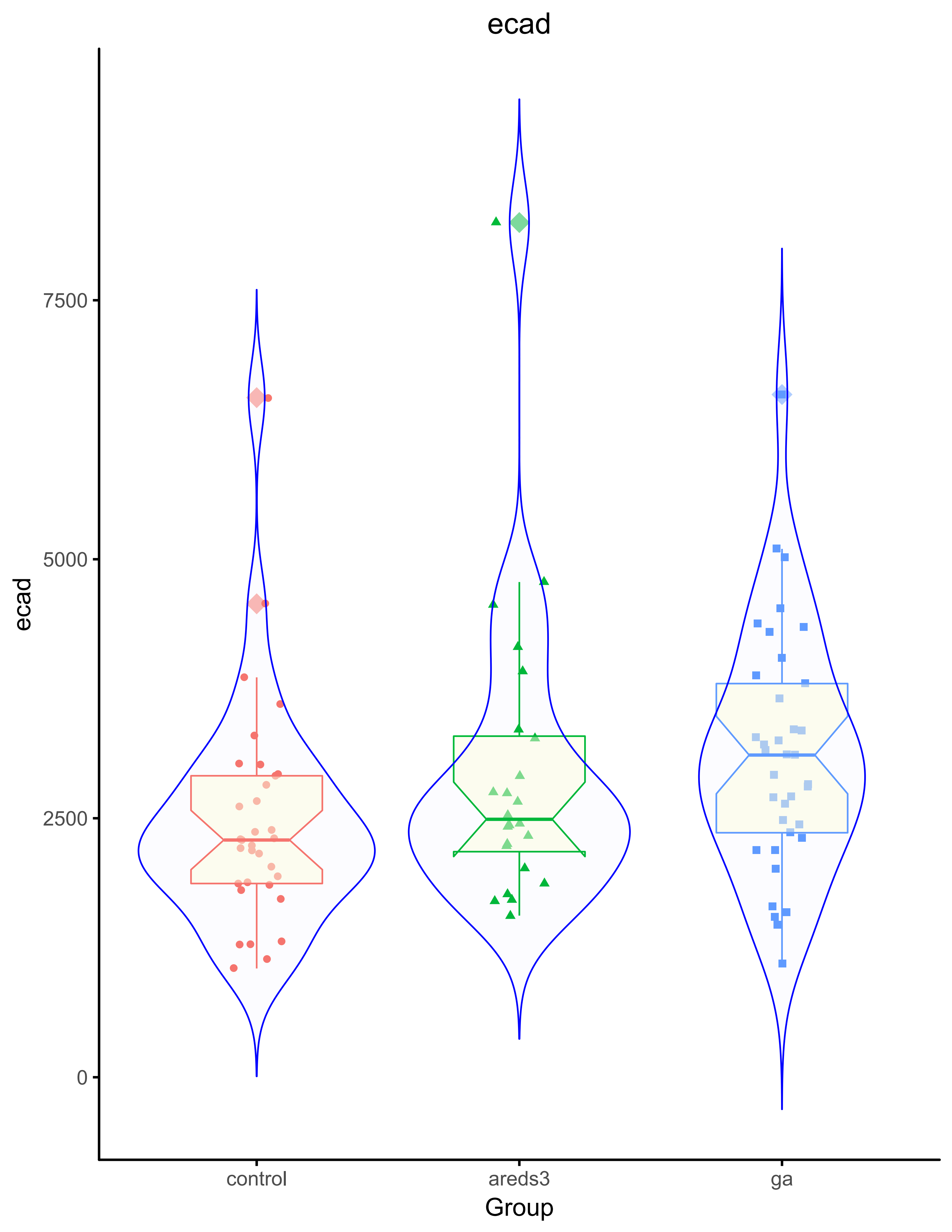

ecad
7500
5000
2500
0
ecad
control
areds3
ga
Group

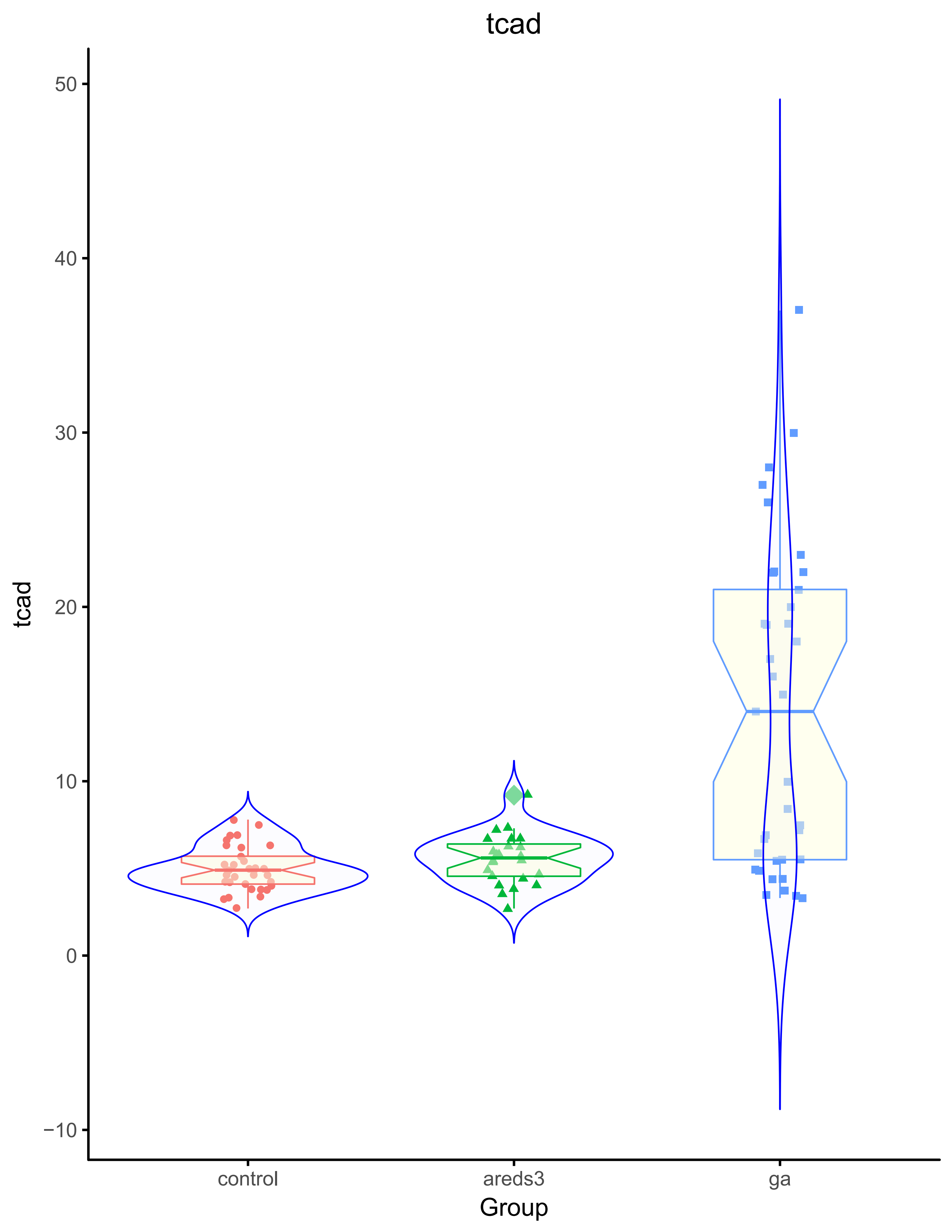

tcad
50
40
30
20
10
0
−10
tcad
control
areds3
ga
Group

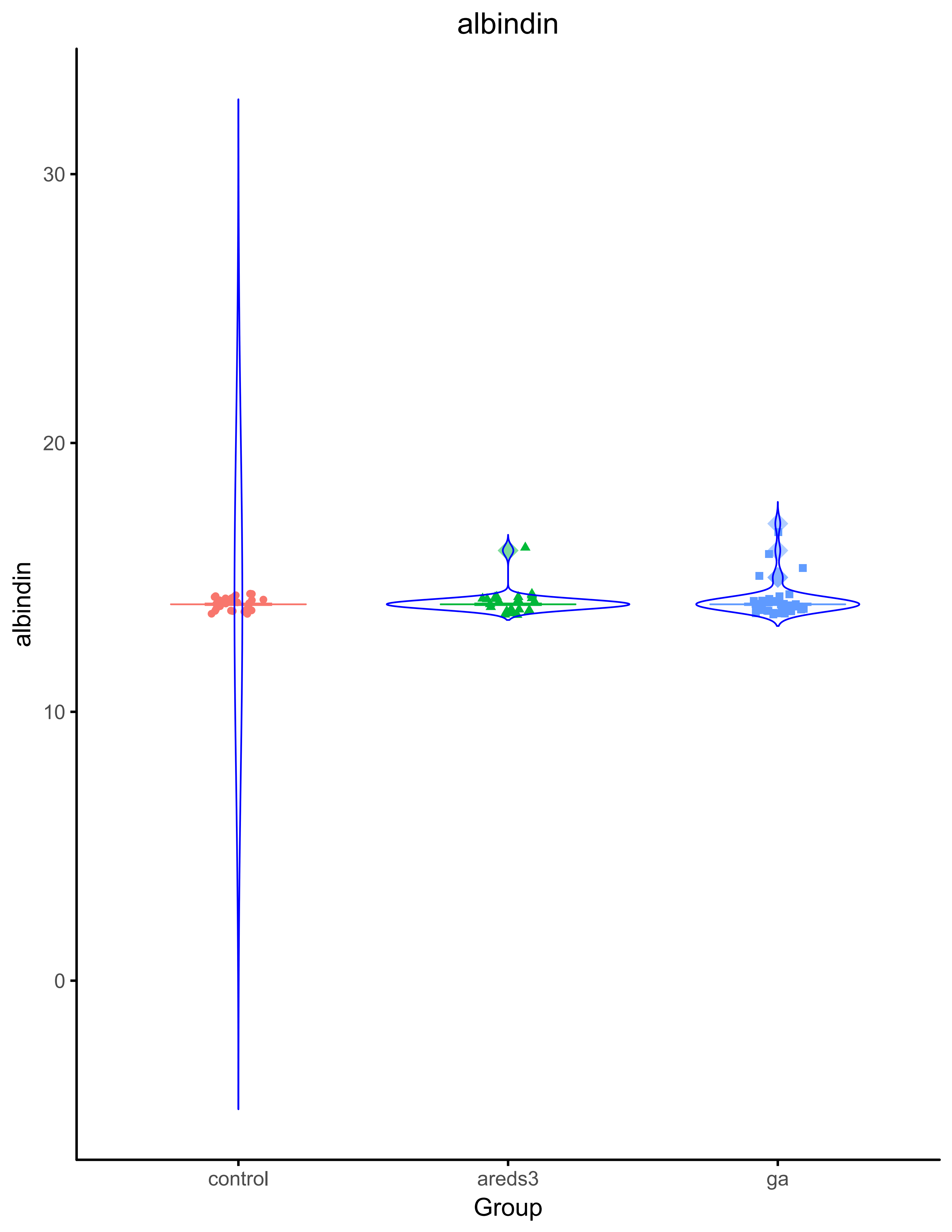
albindin
30
20
10
0
albindin
control
areds3
ga
Group

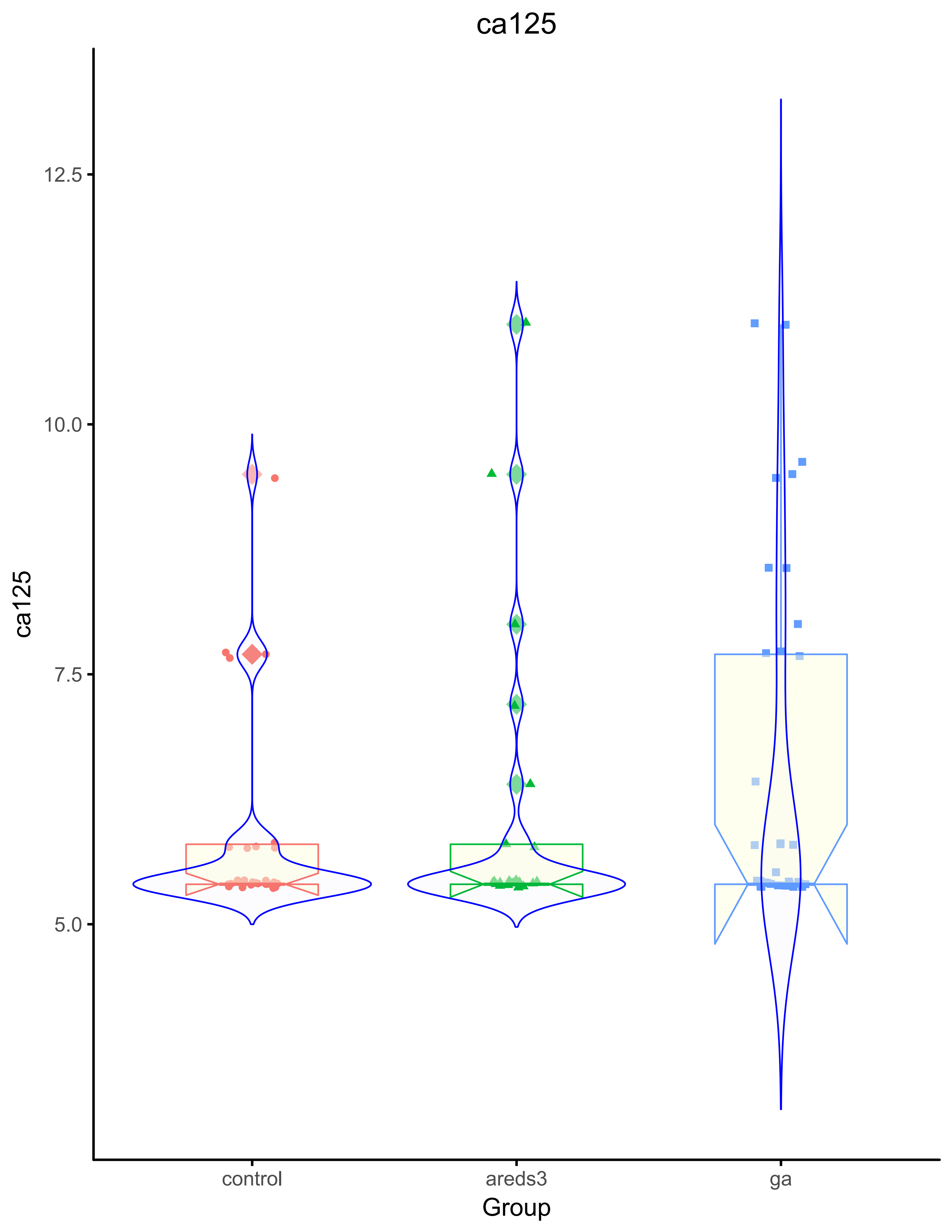
ca125
12.5
10.0
7.5
5.0
ca125
control
areds3
ga
Group

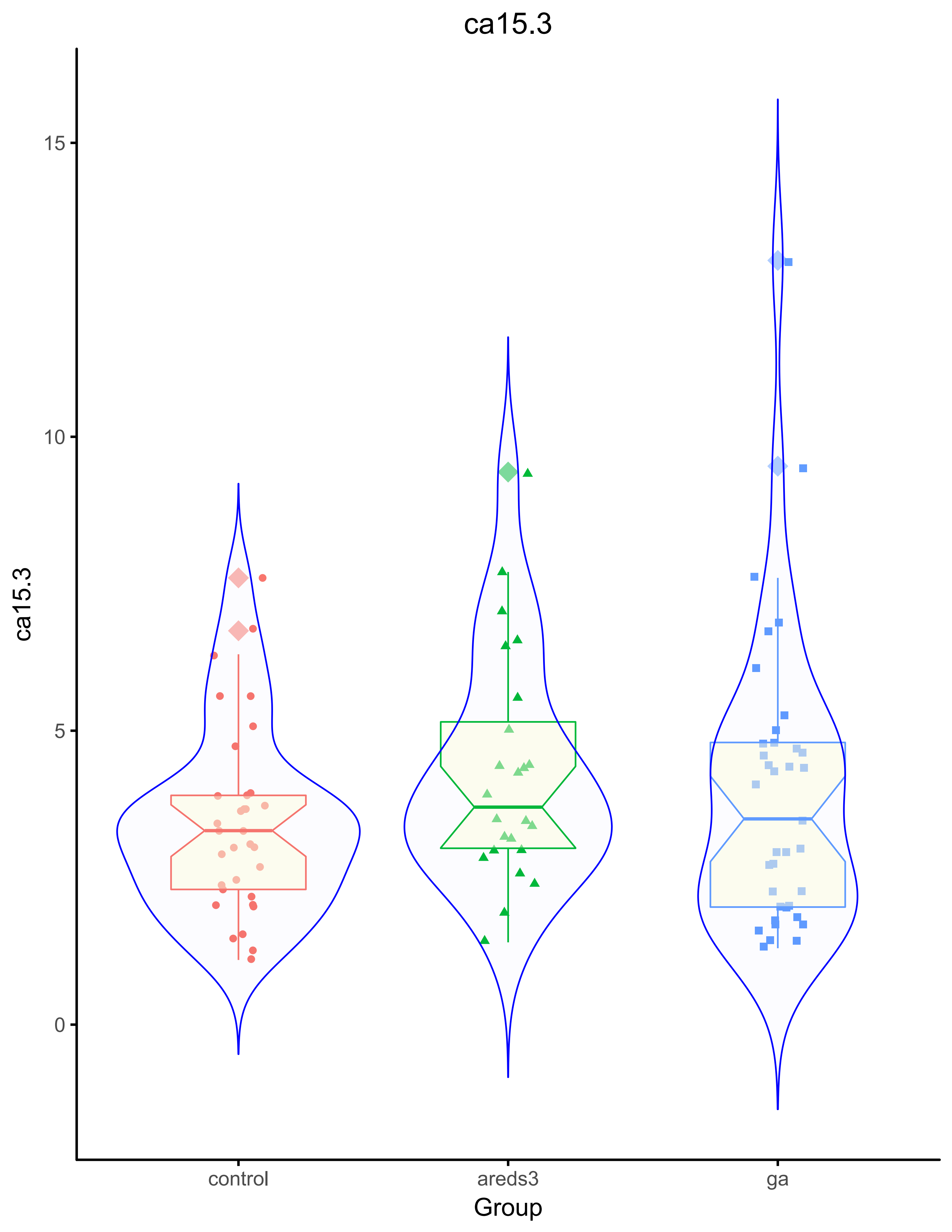

ca15.3
15
10
5
0
ca15.3
control
areds3
ga
Group

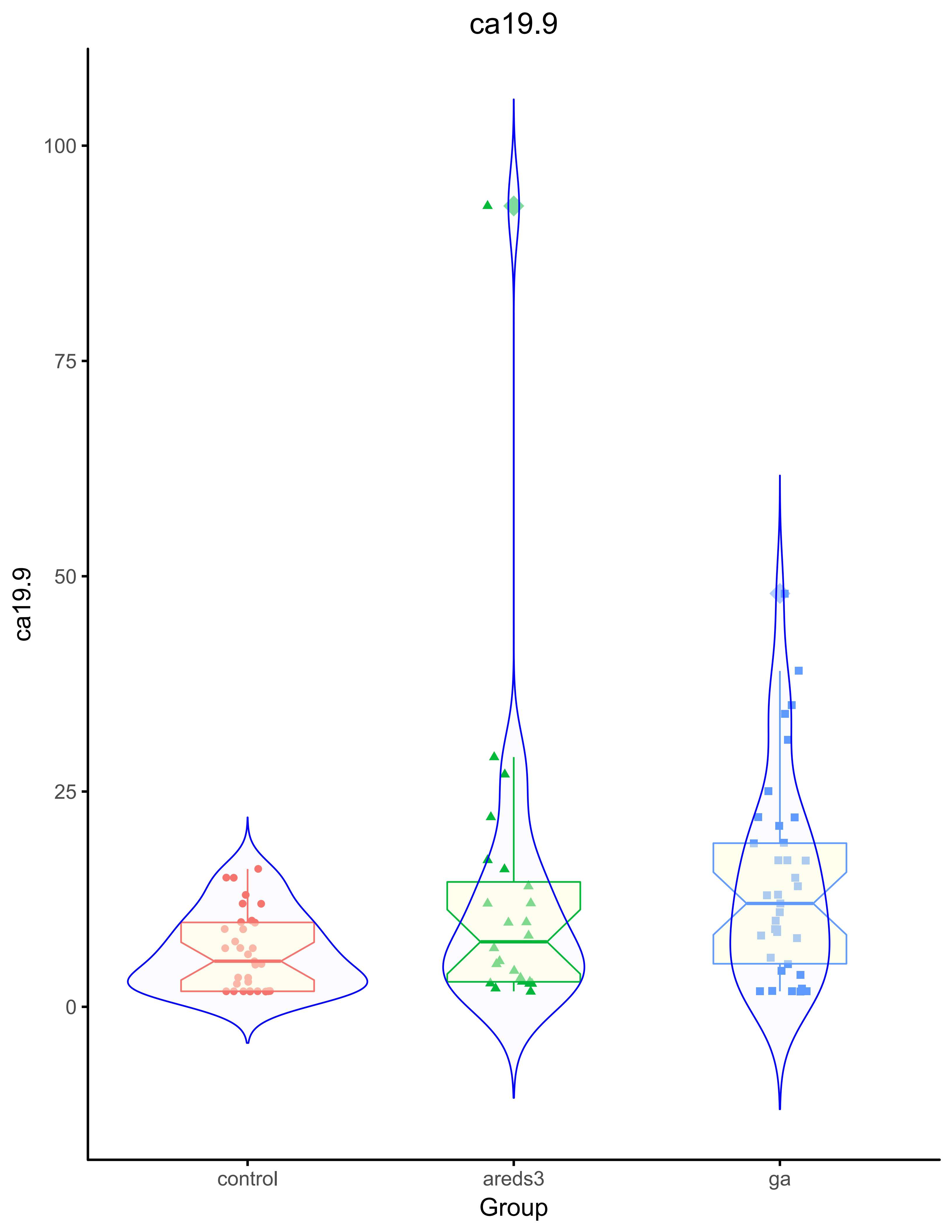

ca19.9
ca19.9
100
75
50
25
0
control
areds3
ga
Group

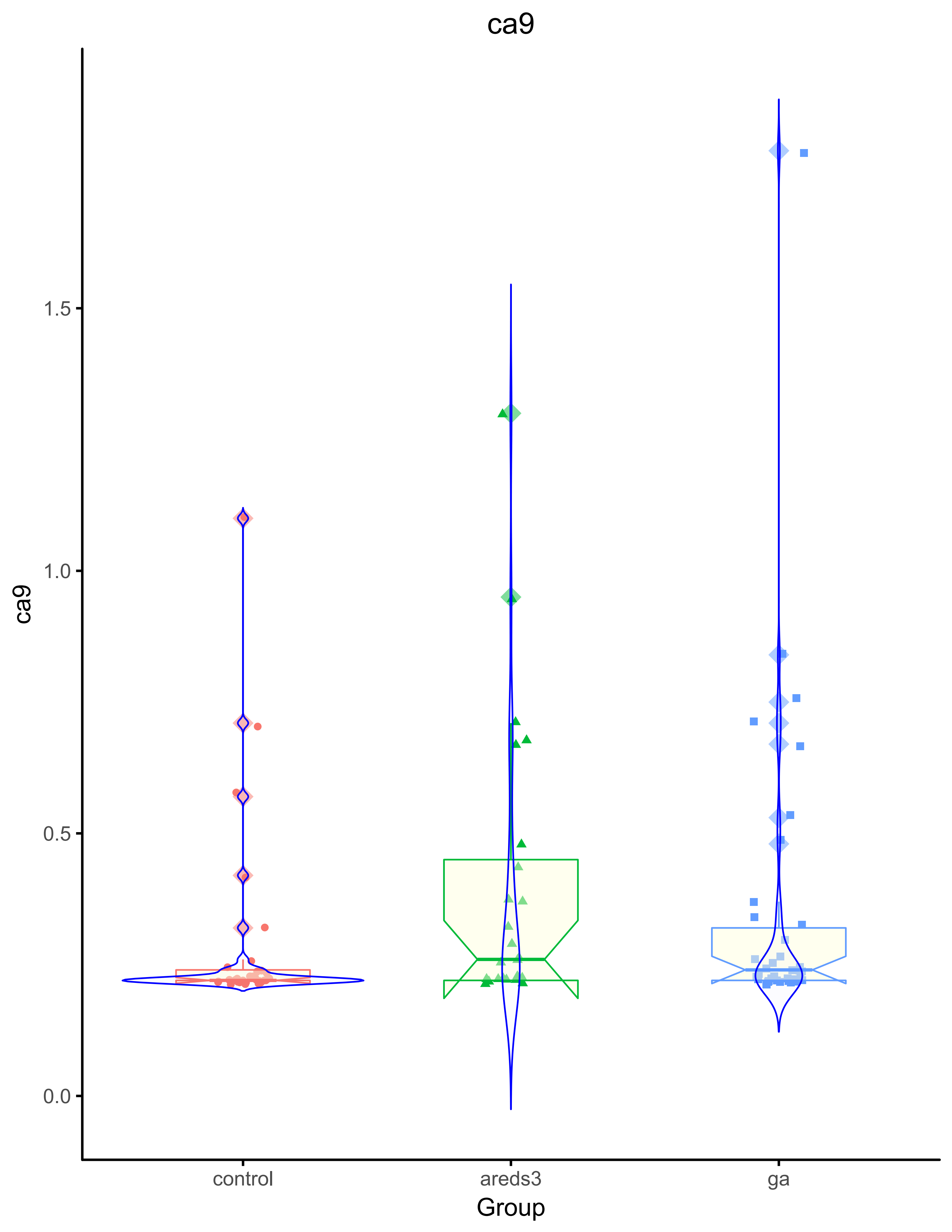

ca9
1.5
1.0
0.5
0.0
ca9
control
areds3
ga
Group

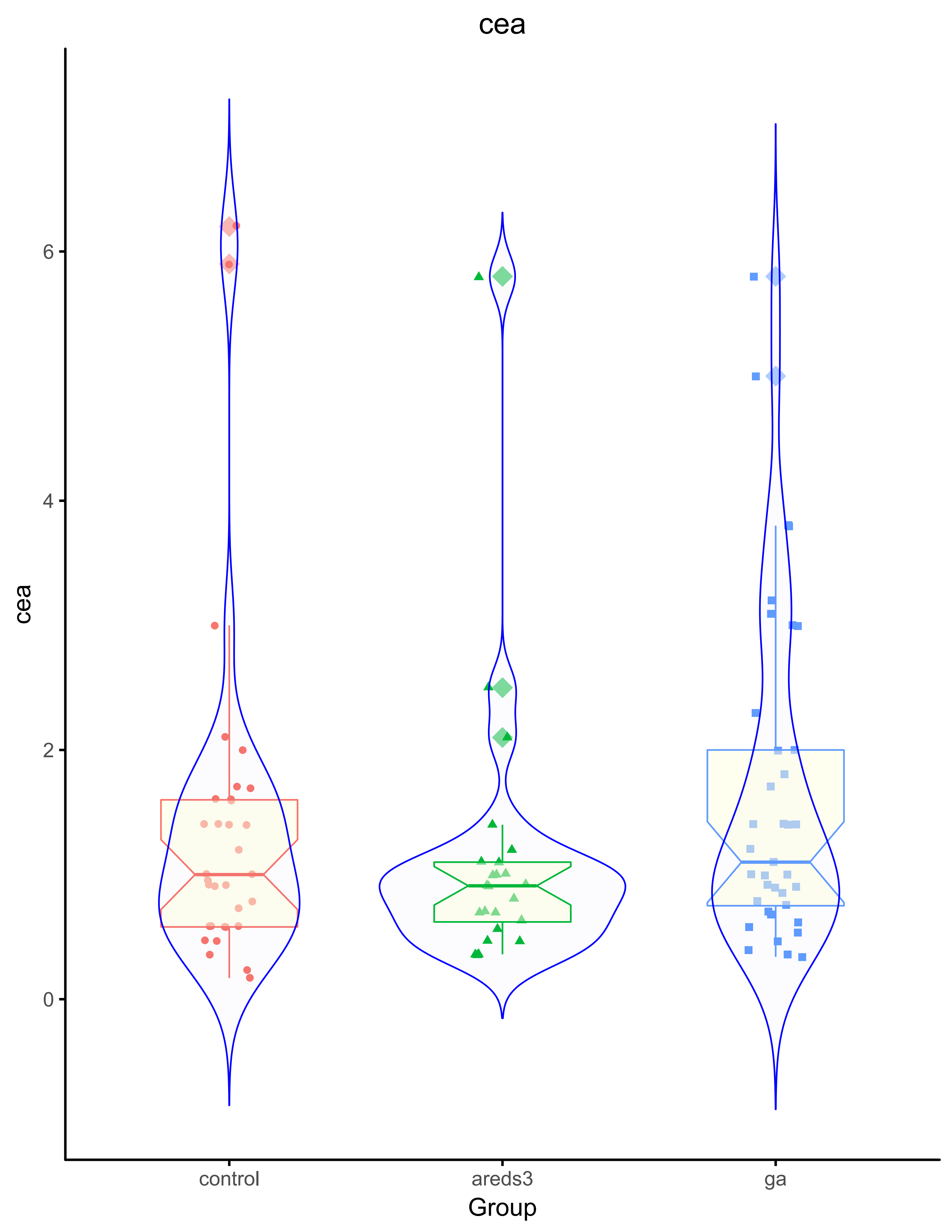

cea
cea
6
4
2
0
control
areds3
ga
Group

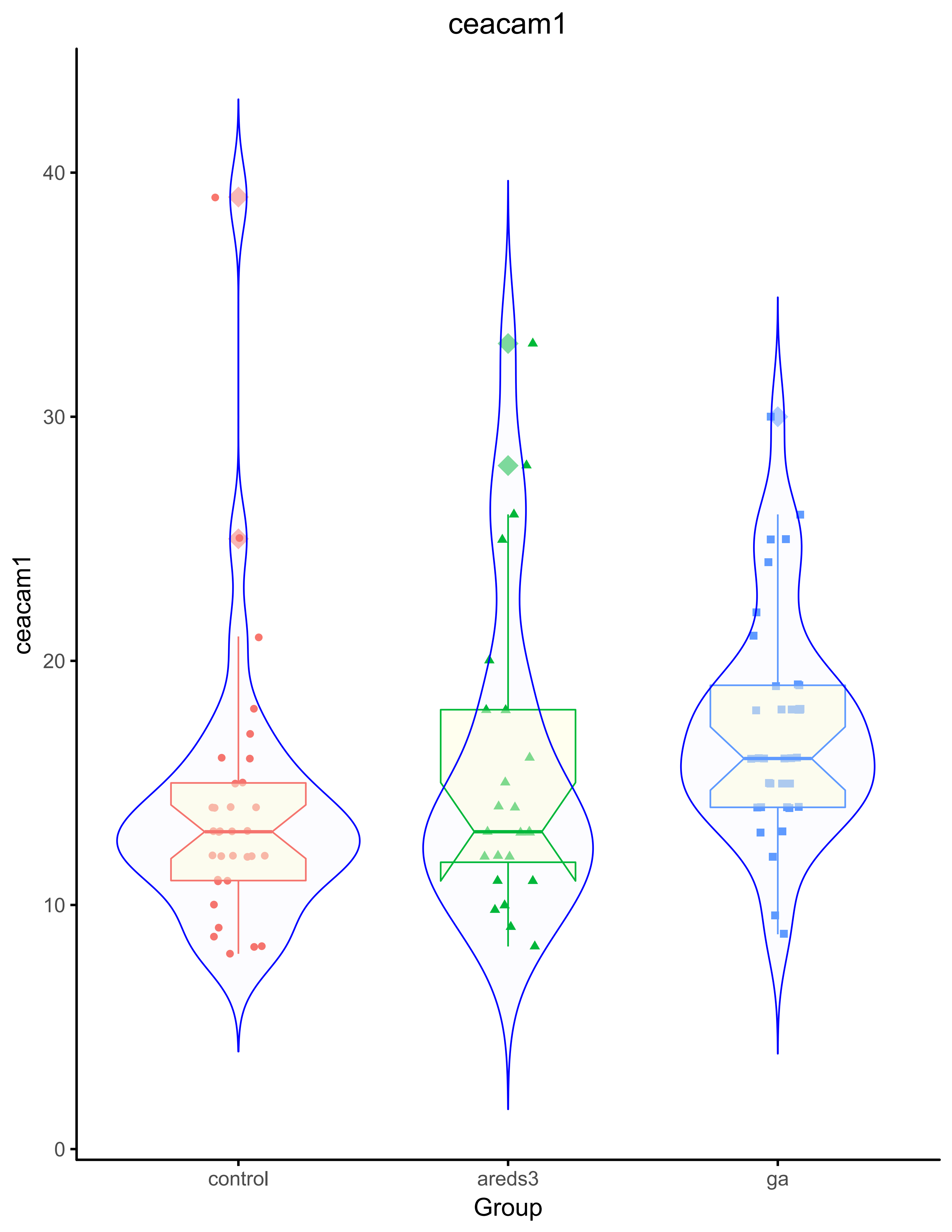

ceacam1
40
30
20
10
0
ceacam1
control
areds3
ga
Group

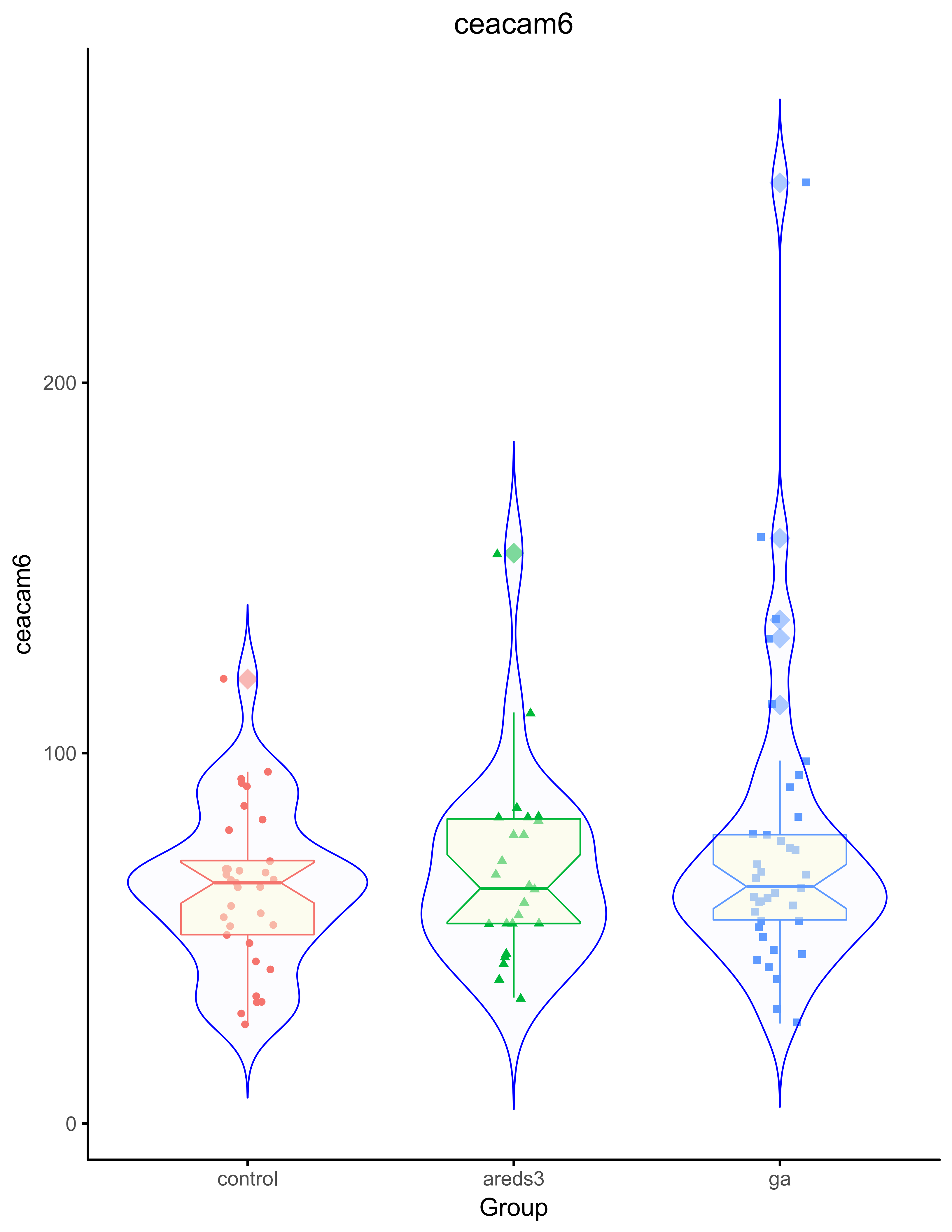

ceacam6
ceacam6
200
100
0
control
areds3
ga
Group

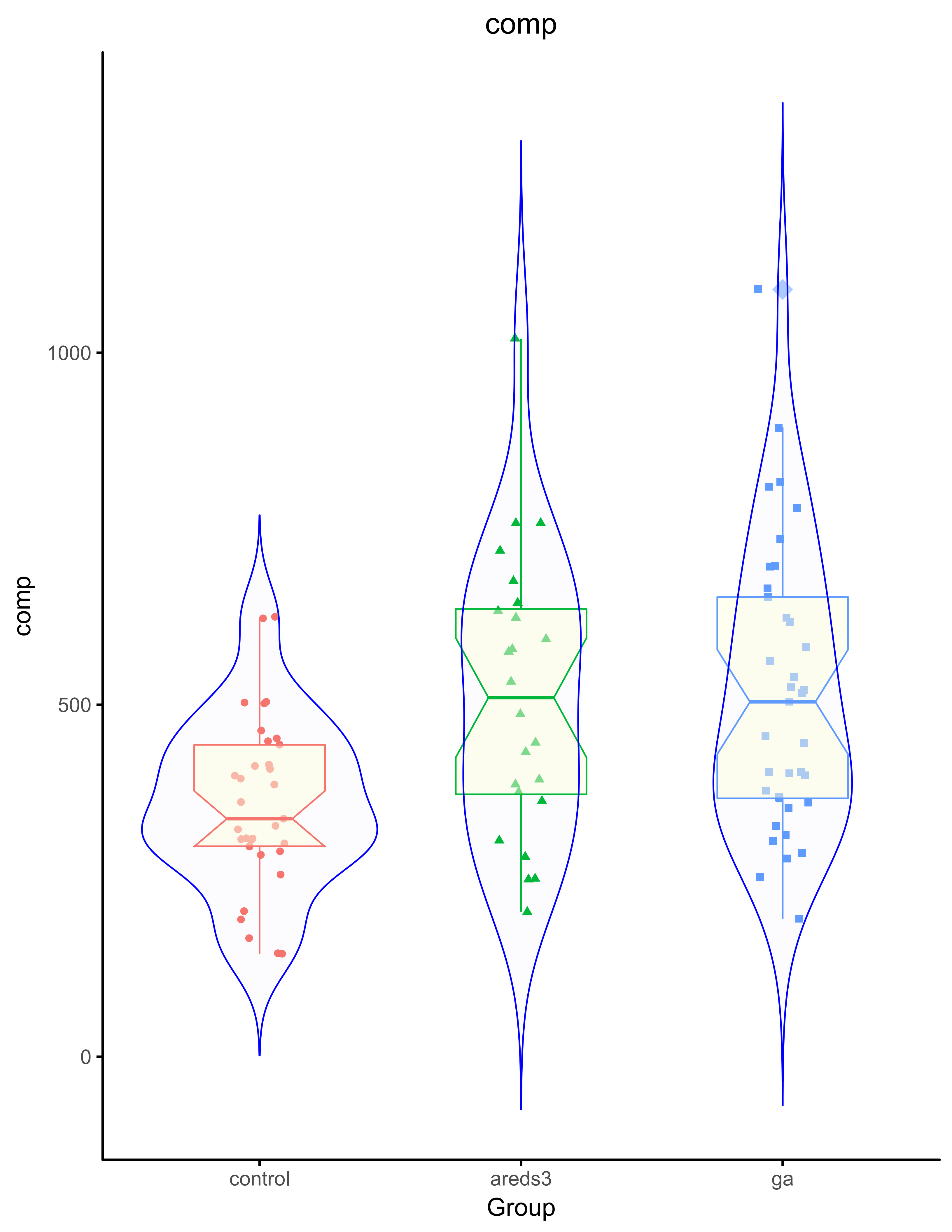
comp
comp
1000
500
0
control
areds3
ga
Group

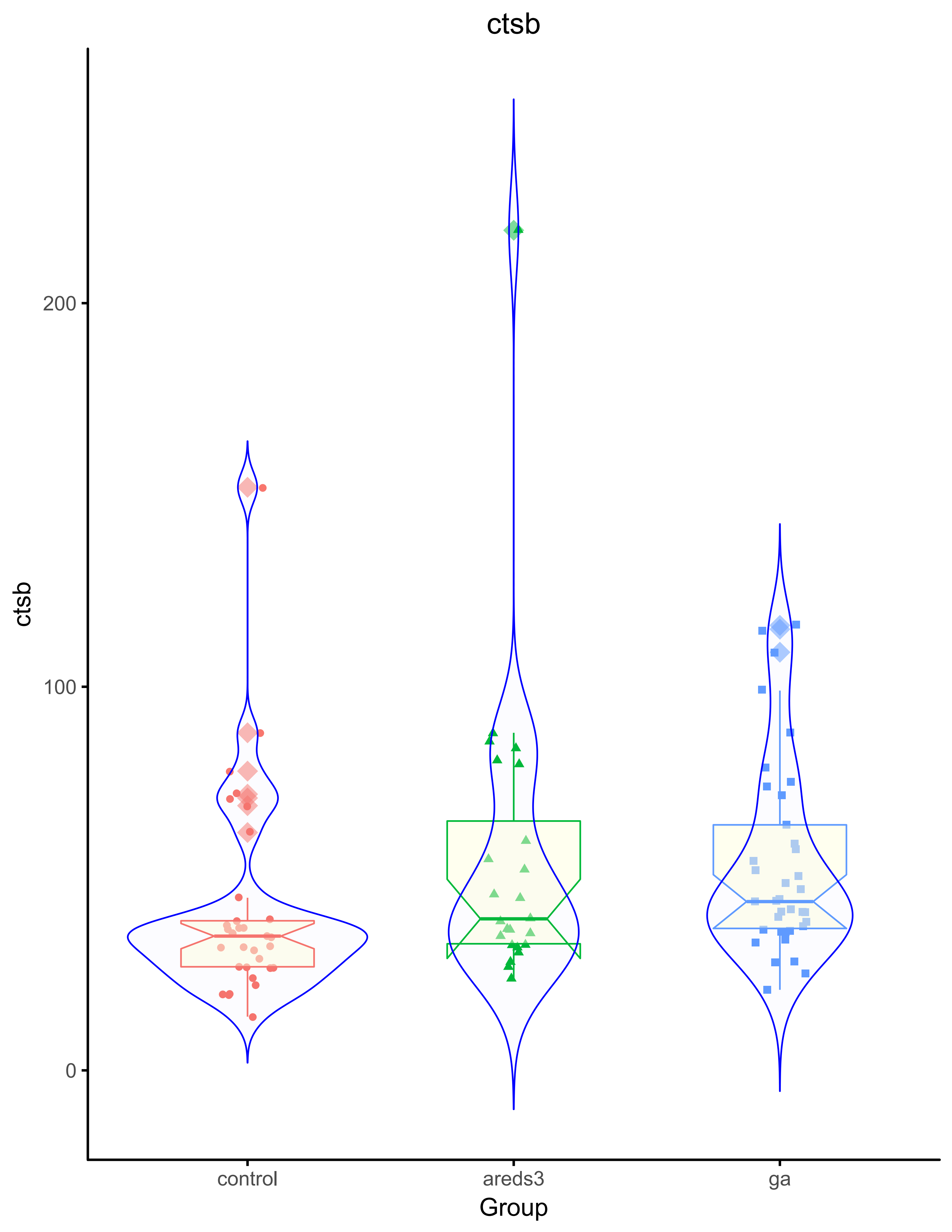
ctsb
200
100
0
ctsb
control
areds3
ga
Group

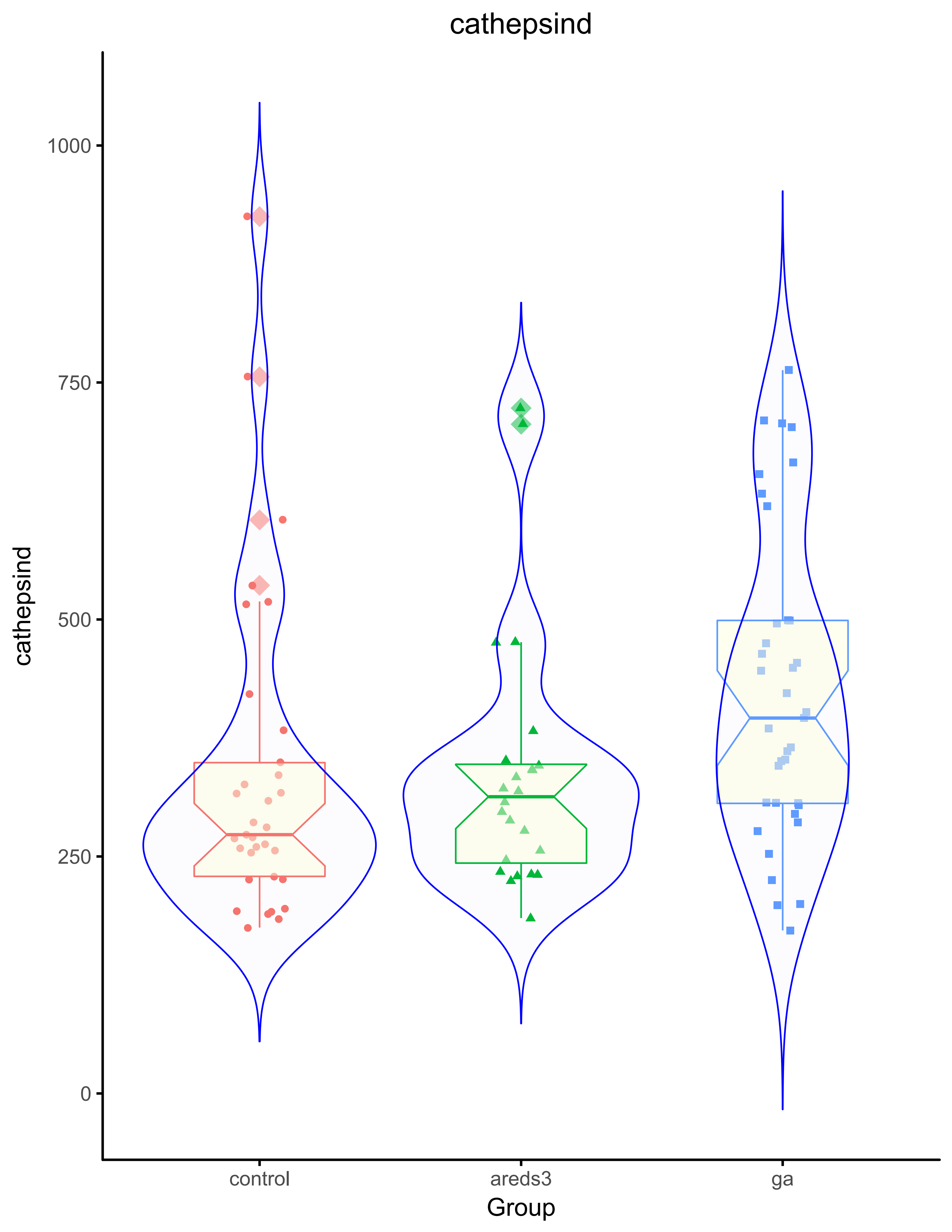
cathepsind
1000
750
500
250
0
cathepsind
control
areds3
ga
Group

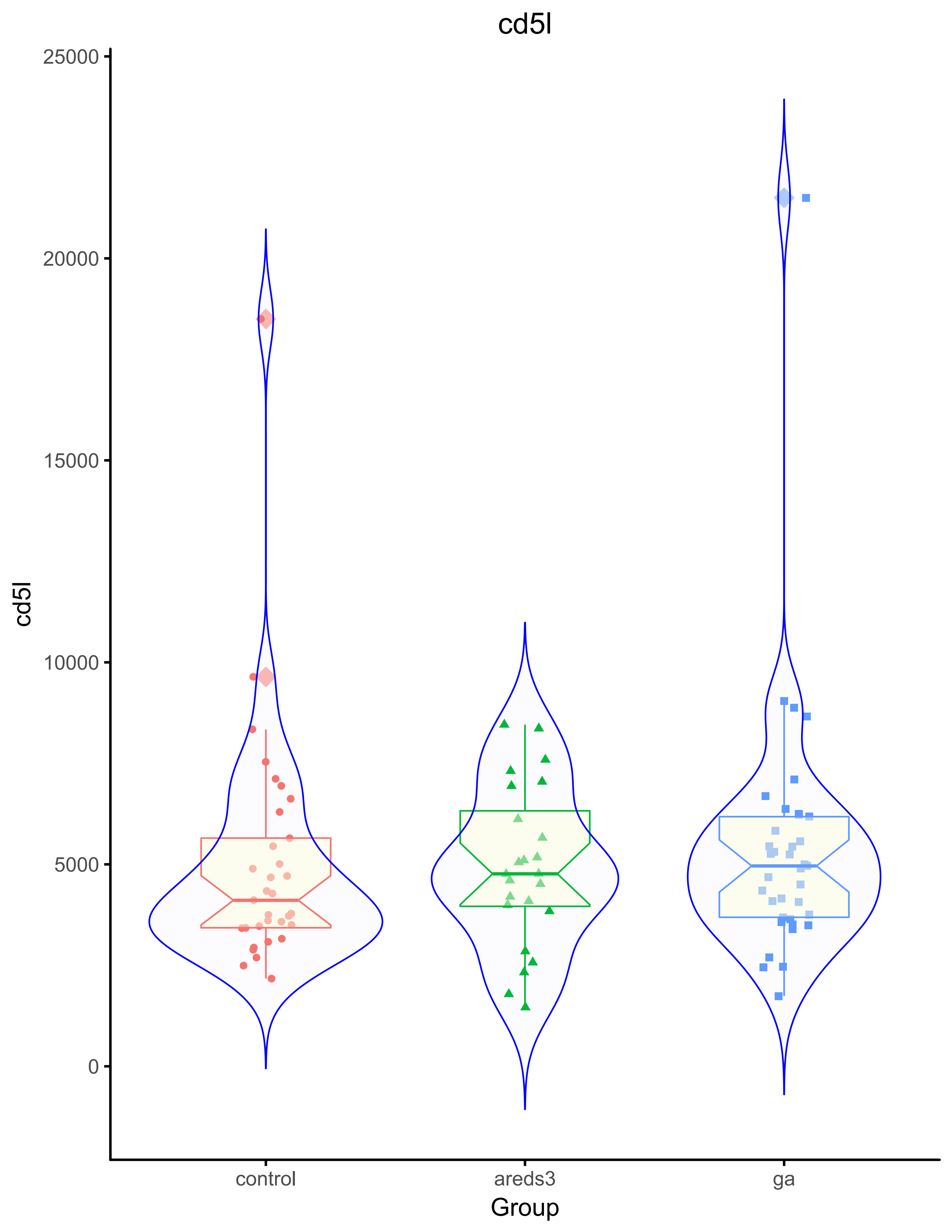
cd5l
25000
20000
15000
10000
5000
0
cd5l
control
areds3
ga
Group

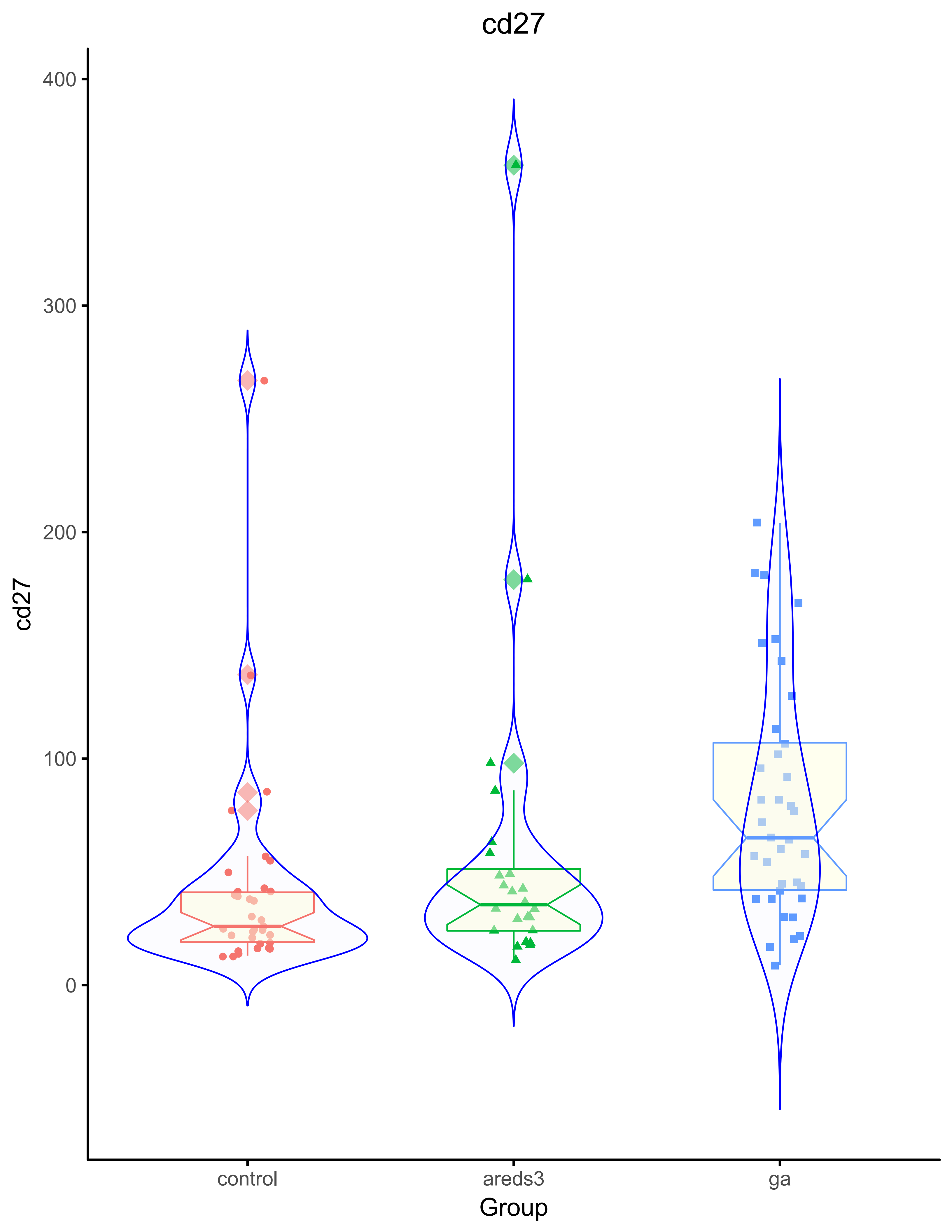

cd27
400
300
200
100
0
cd27
control
areds3
ga
Group

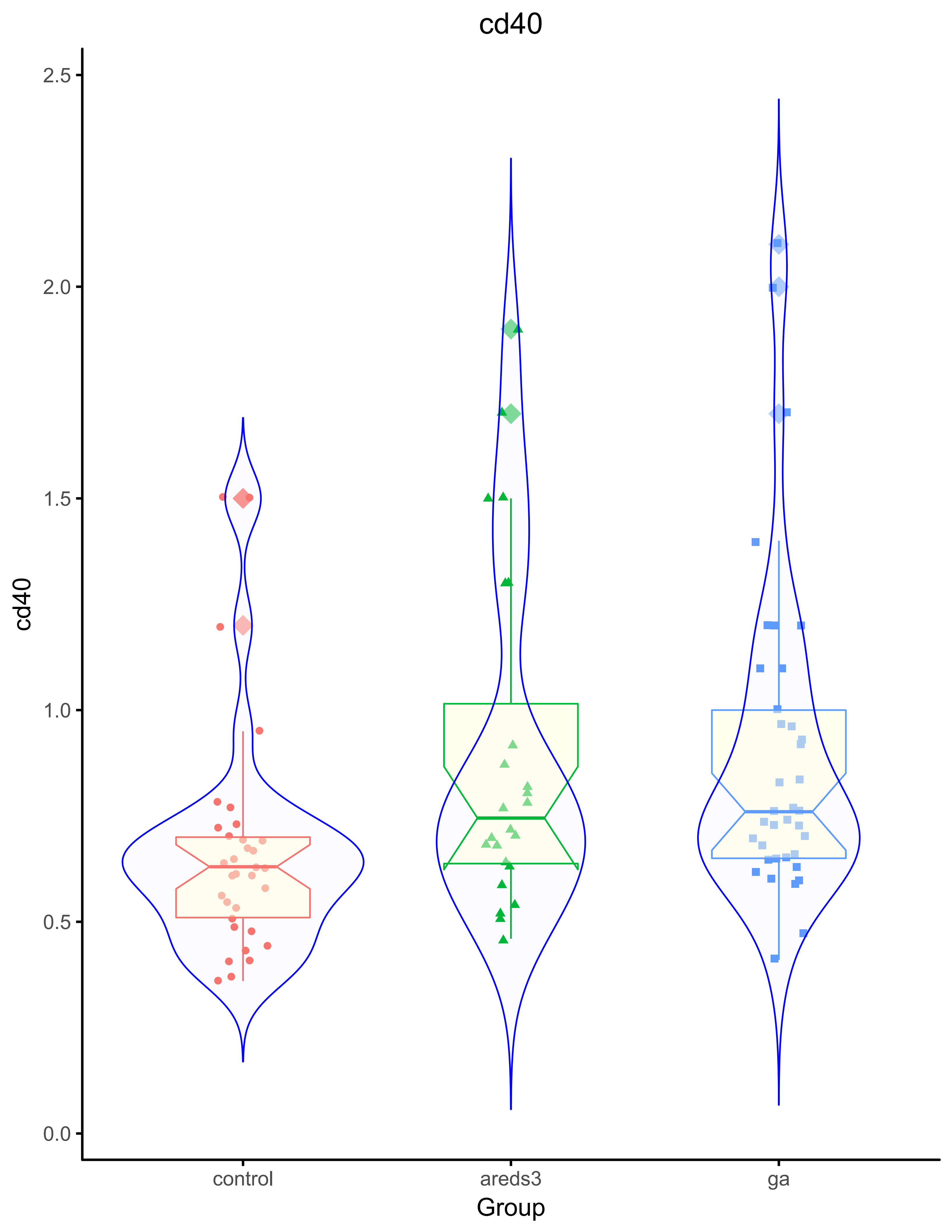
cd40
cd40
2.5
2.0
1.5
1.0
0.5
0.0
control
areds3
ga
Group

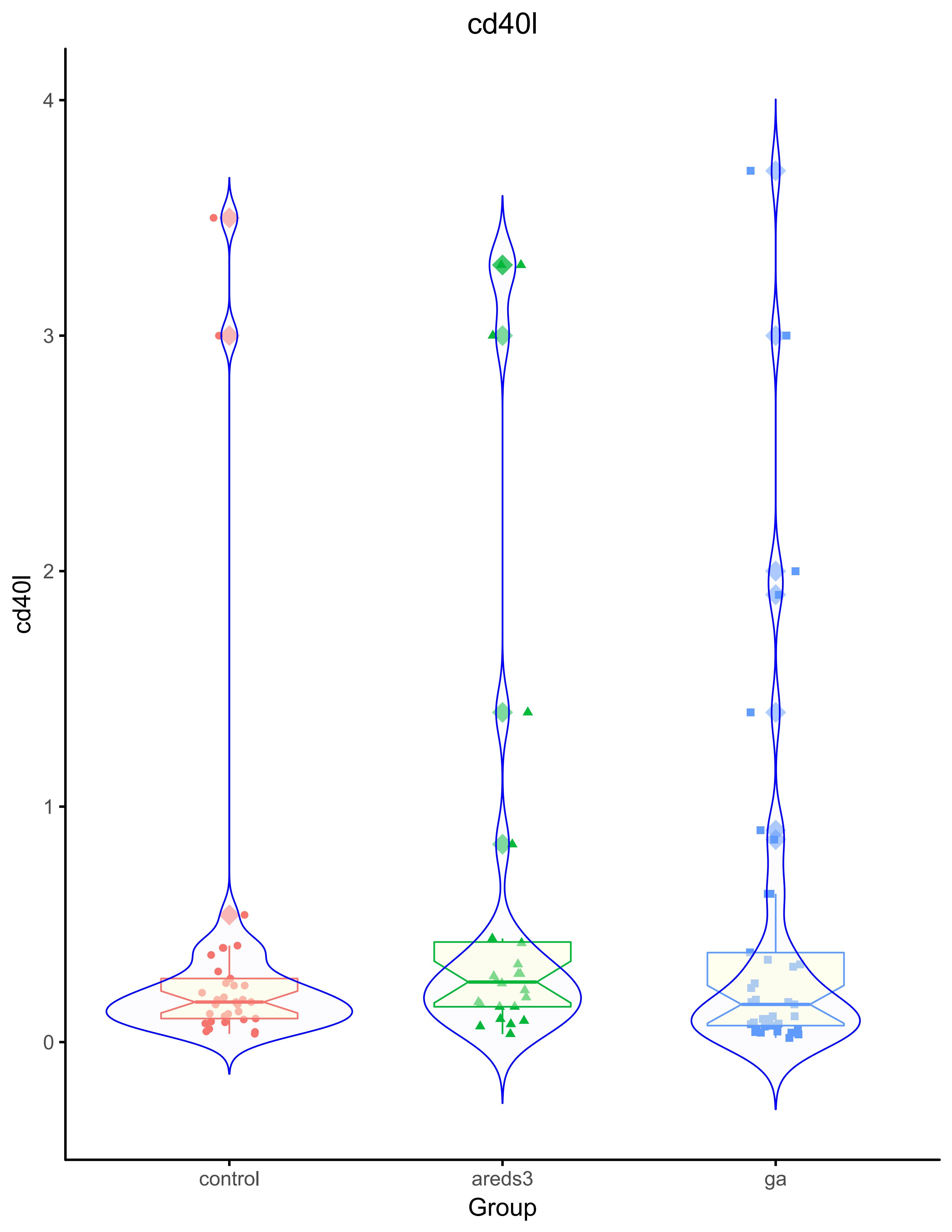
cd40l
4
3
2
1
0
cd40l
control
areds3
ga
Group

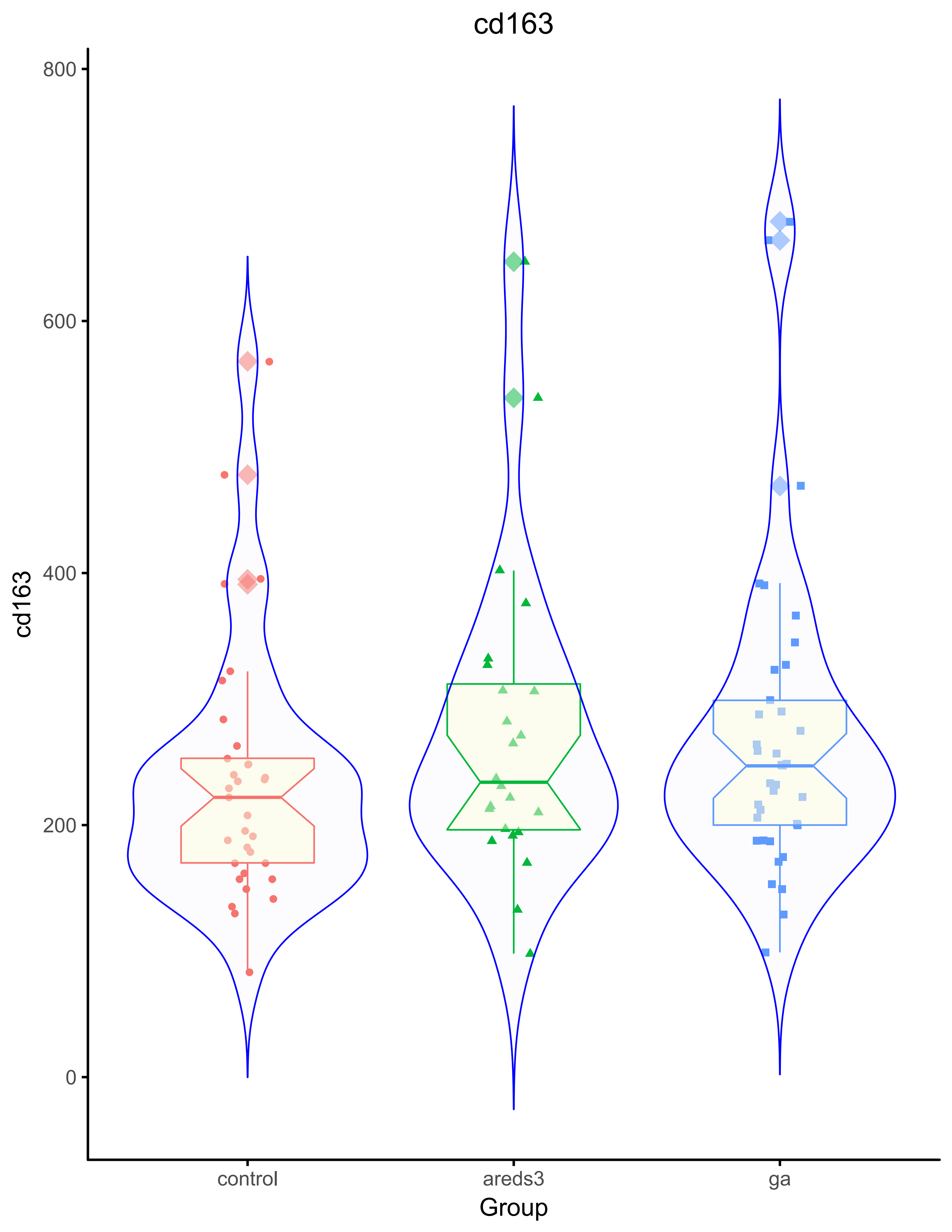

cd163
800
600
400
200
0
cd163
control
areds3
ga
Group

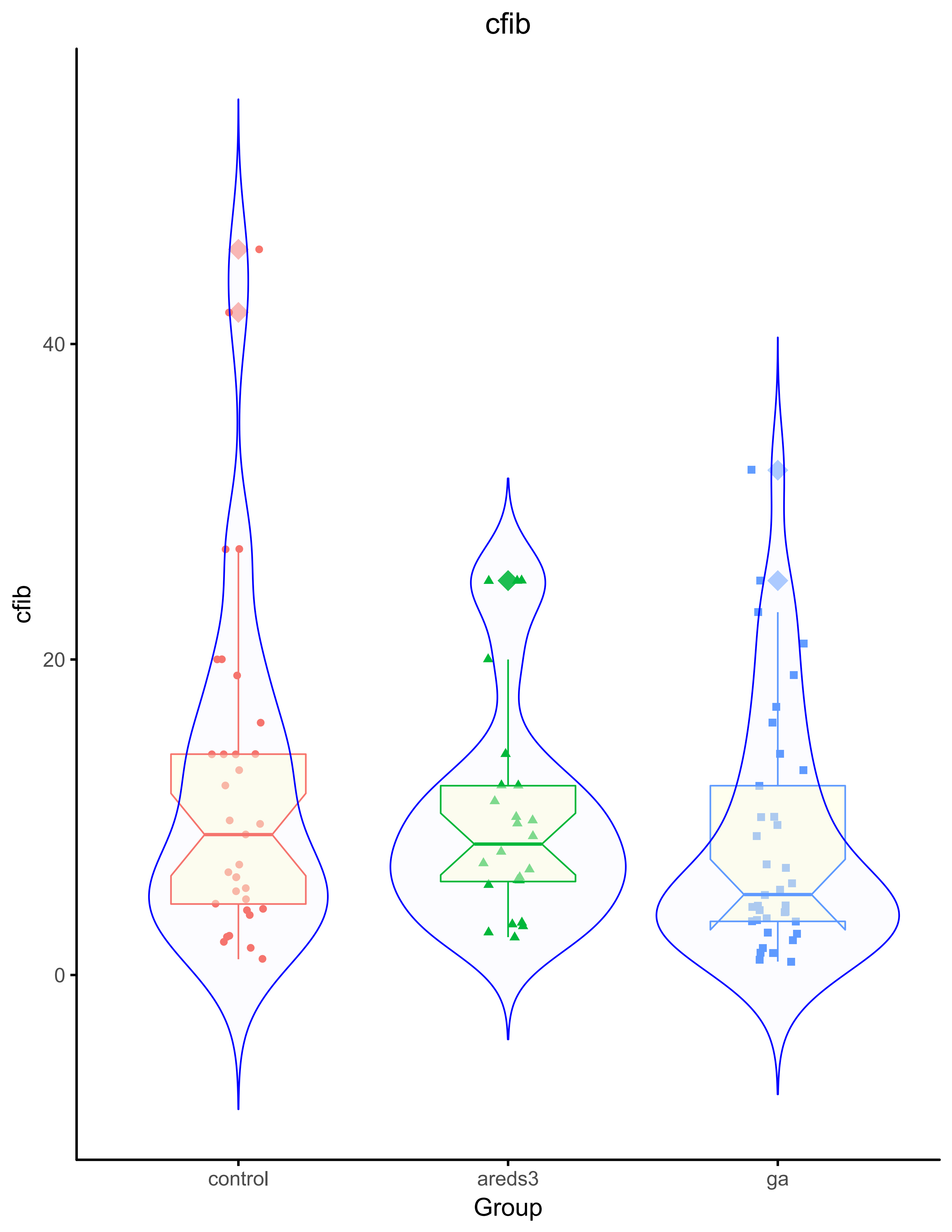
cfib
40
20
0
cfib
control
areds3
ga
Group

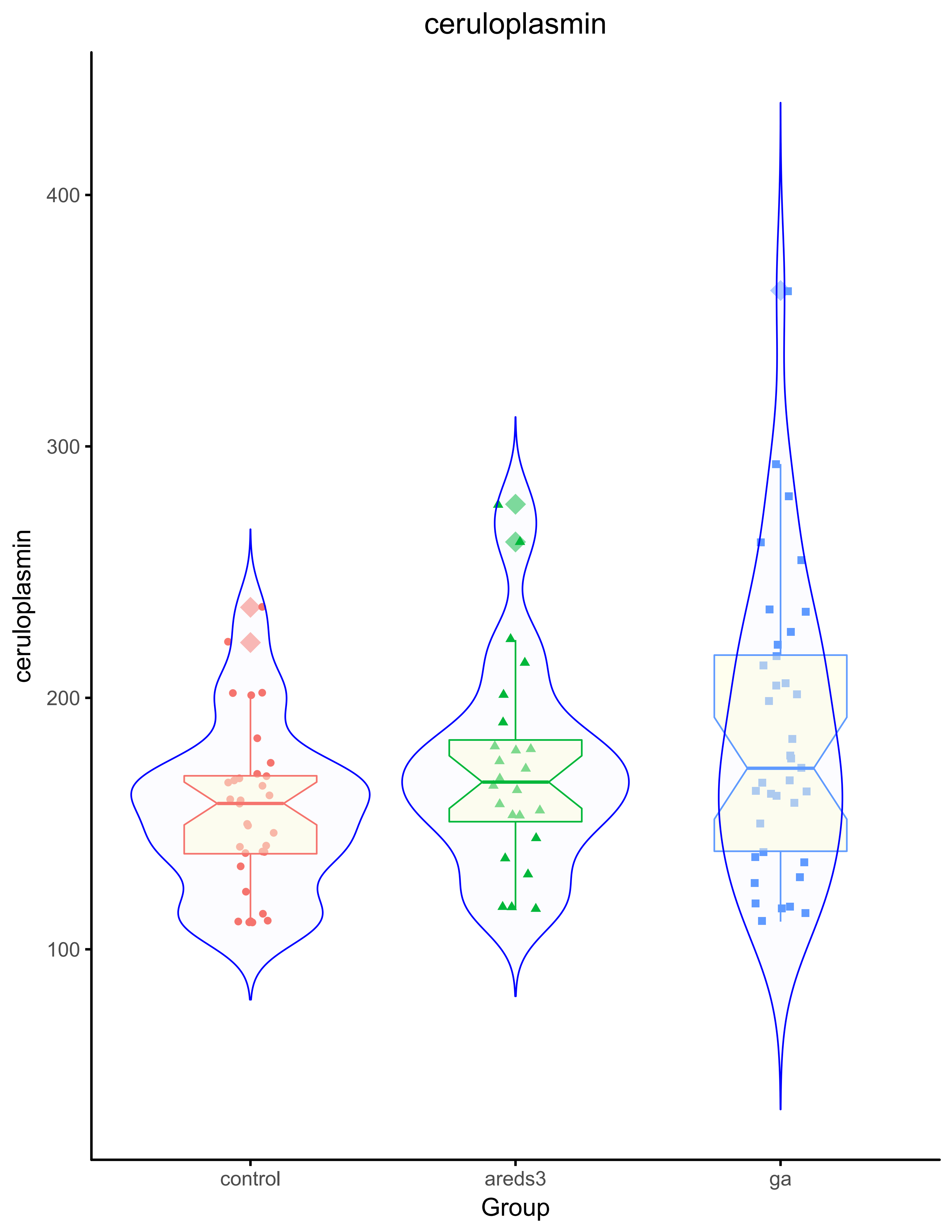

ceruloplasmin
400
300
200
100
ceruloplasmin
control
areds3
ga
Group

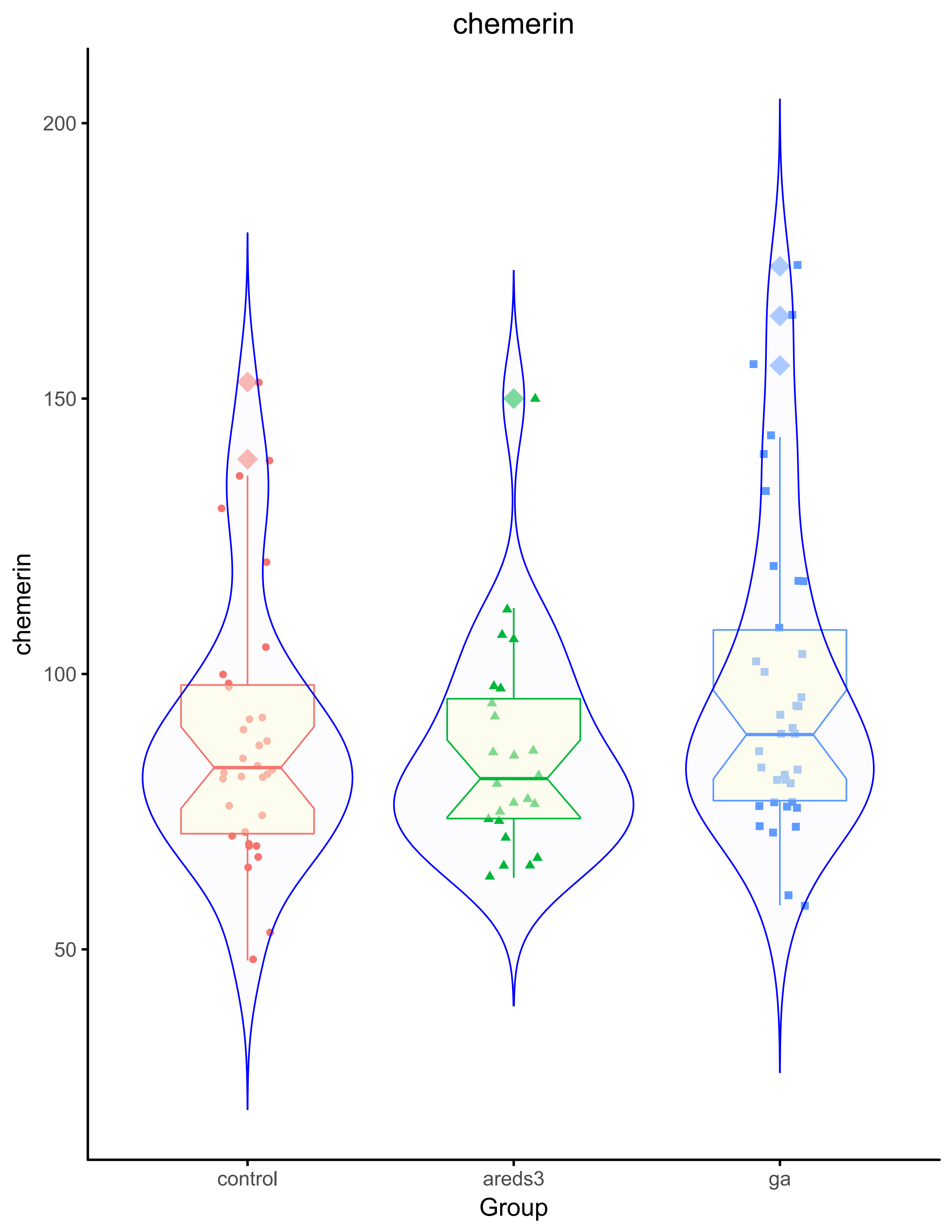
chemerin
200
150
100
50
chemerin
control
areds3
ga
Group

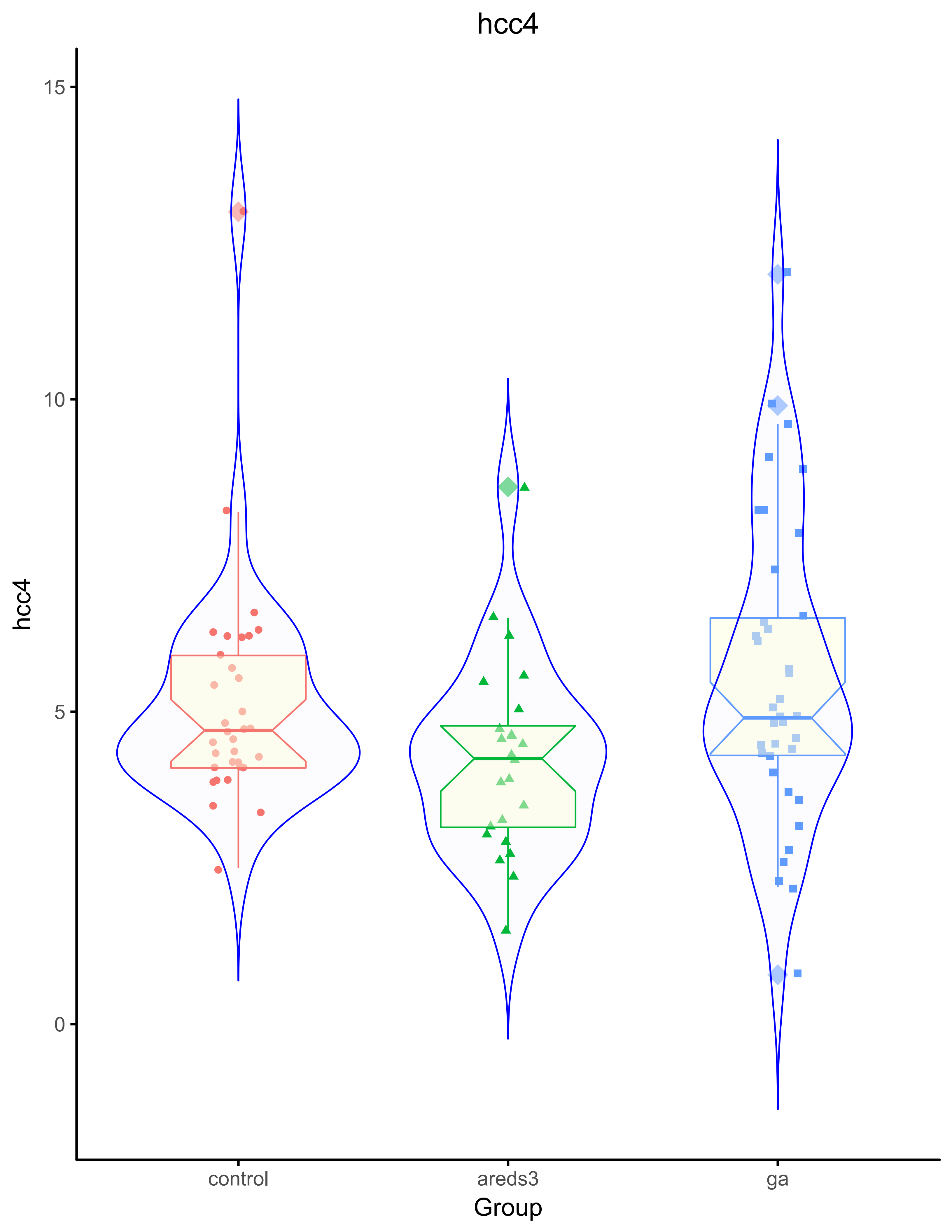

hcc4
15
10
5
0
hcc4
control
areds3
ga
Group

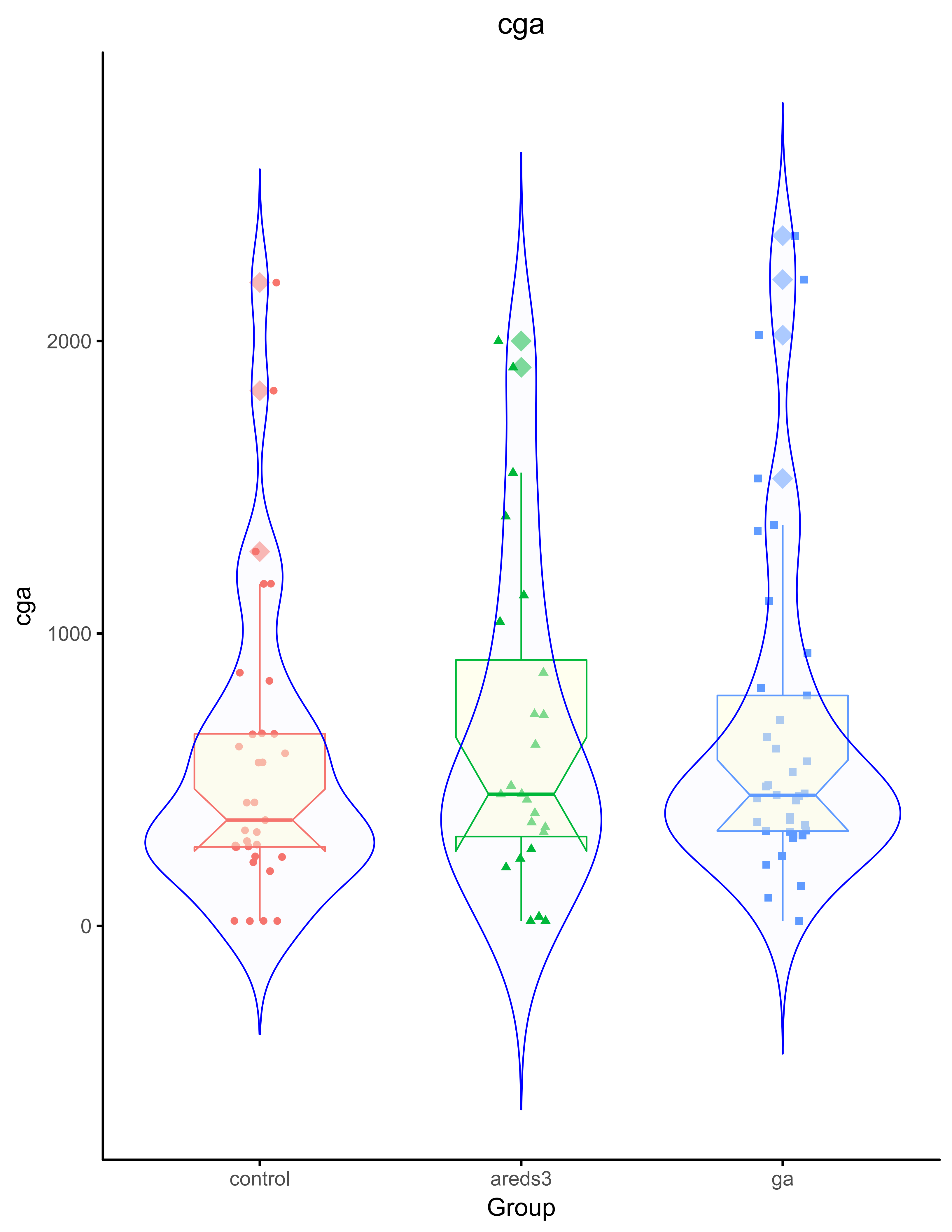
cga
2000
1000
0
cga
control
areds3
ga
Group

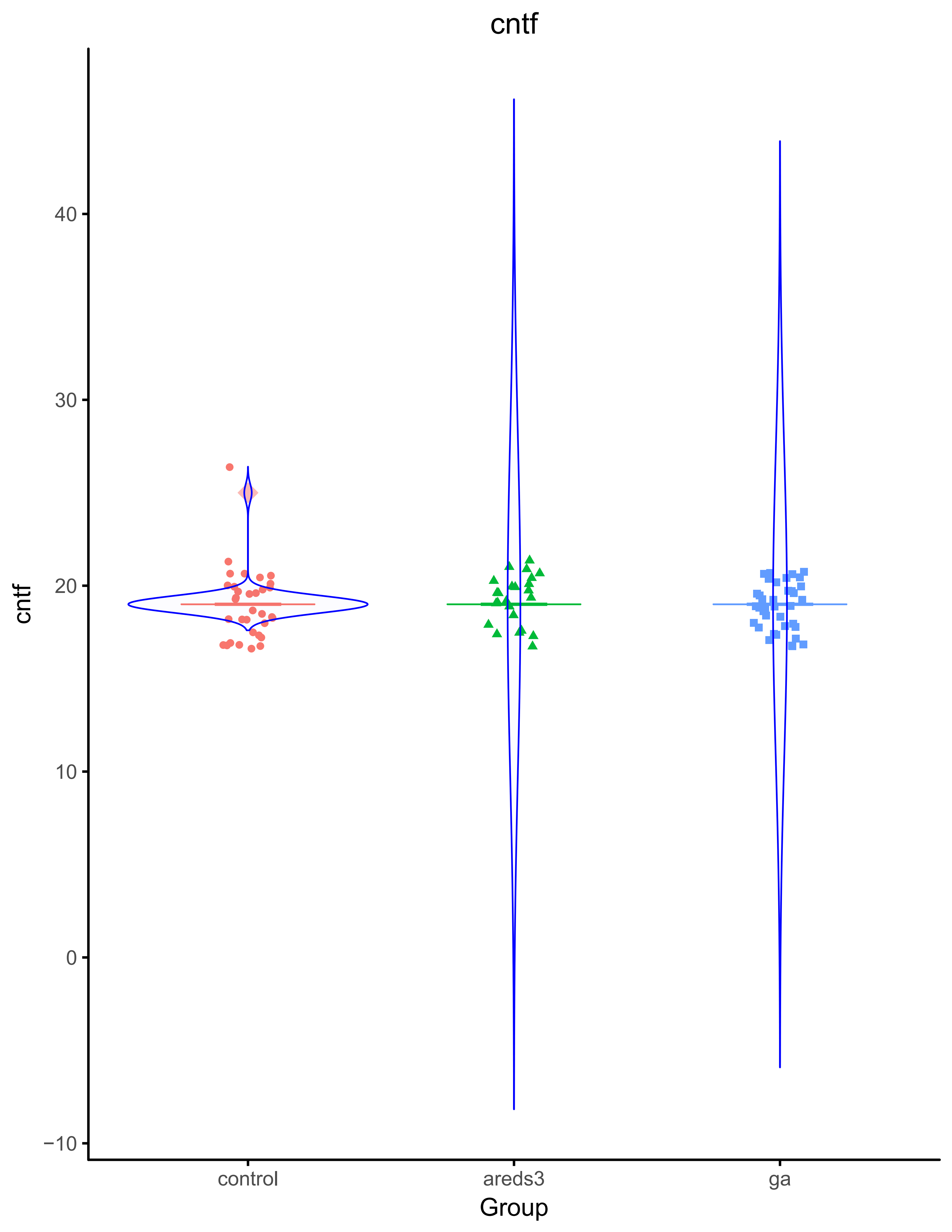

cntf
40
30
20
10
0
−10
cntf
control
areds3
ga
Group

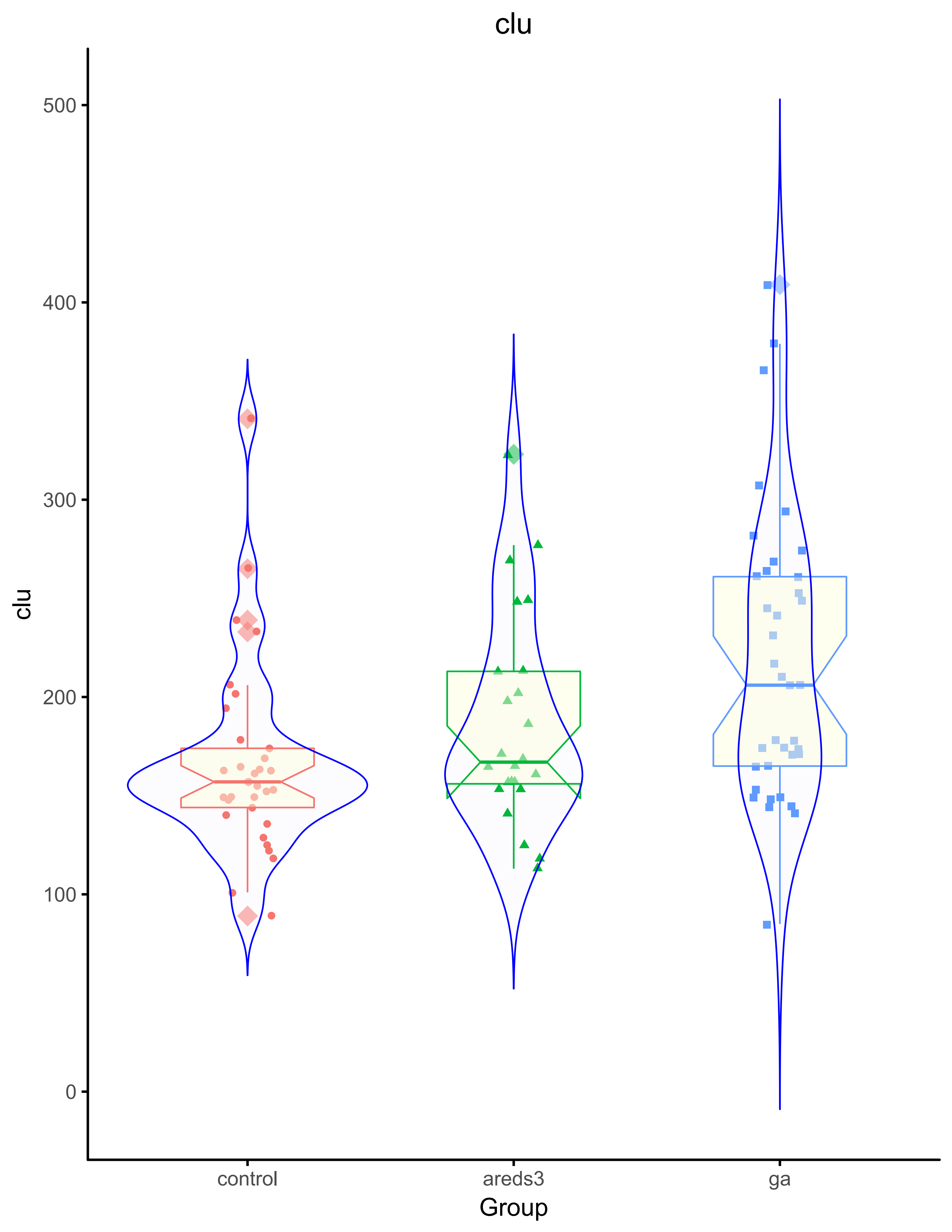

clu
500
400
300
200
100
0
clu
control
areds3
ga
Group

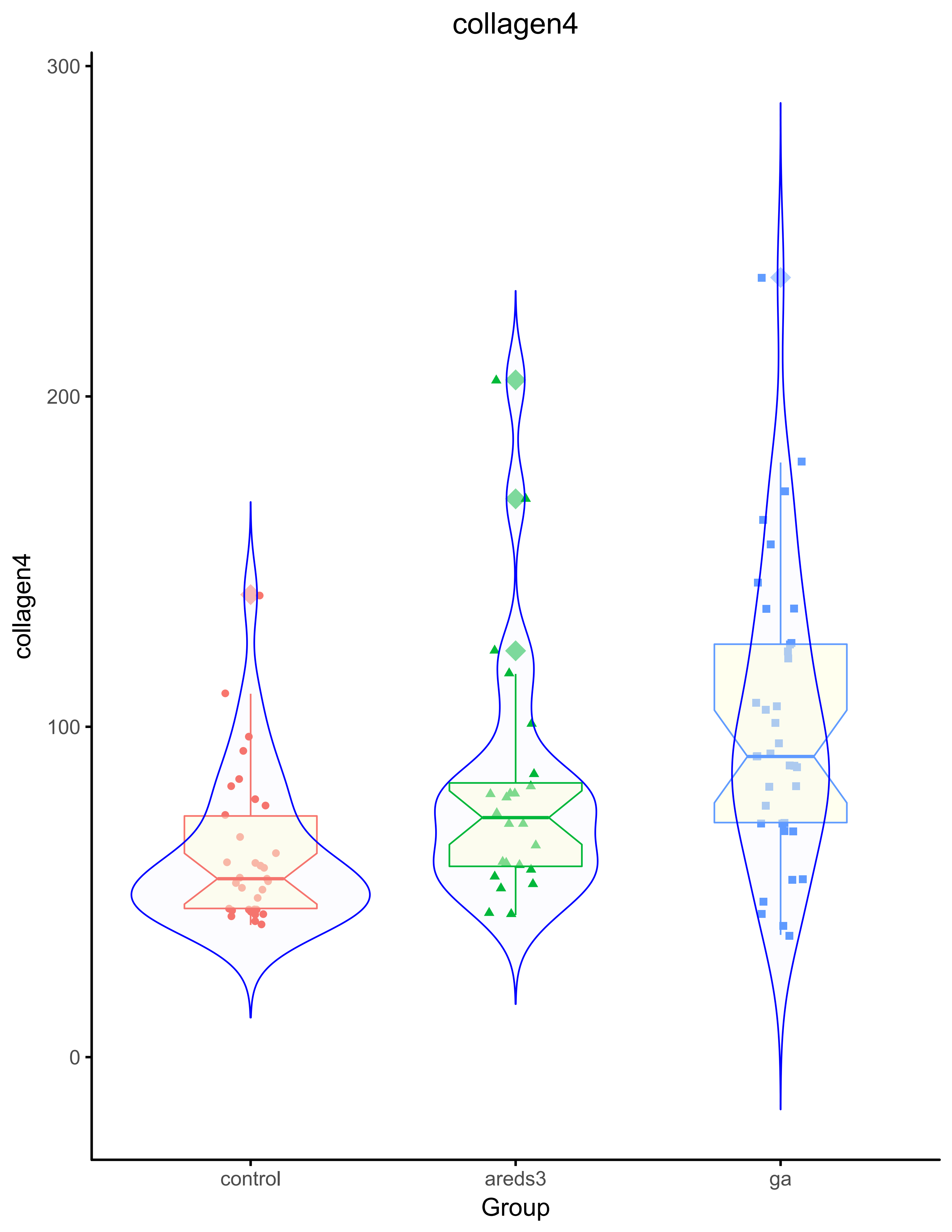

collagen4
300
200
100
0
collagen4
control
areds3
ga
Group

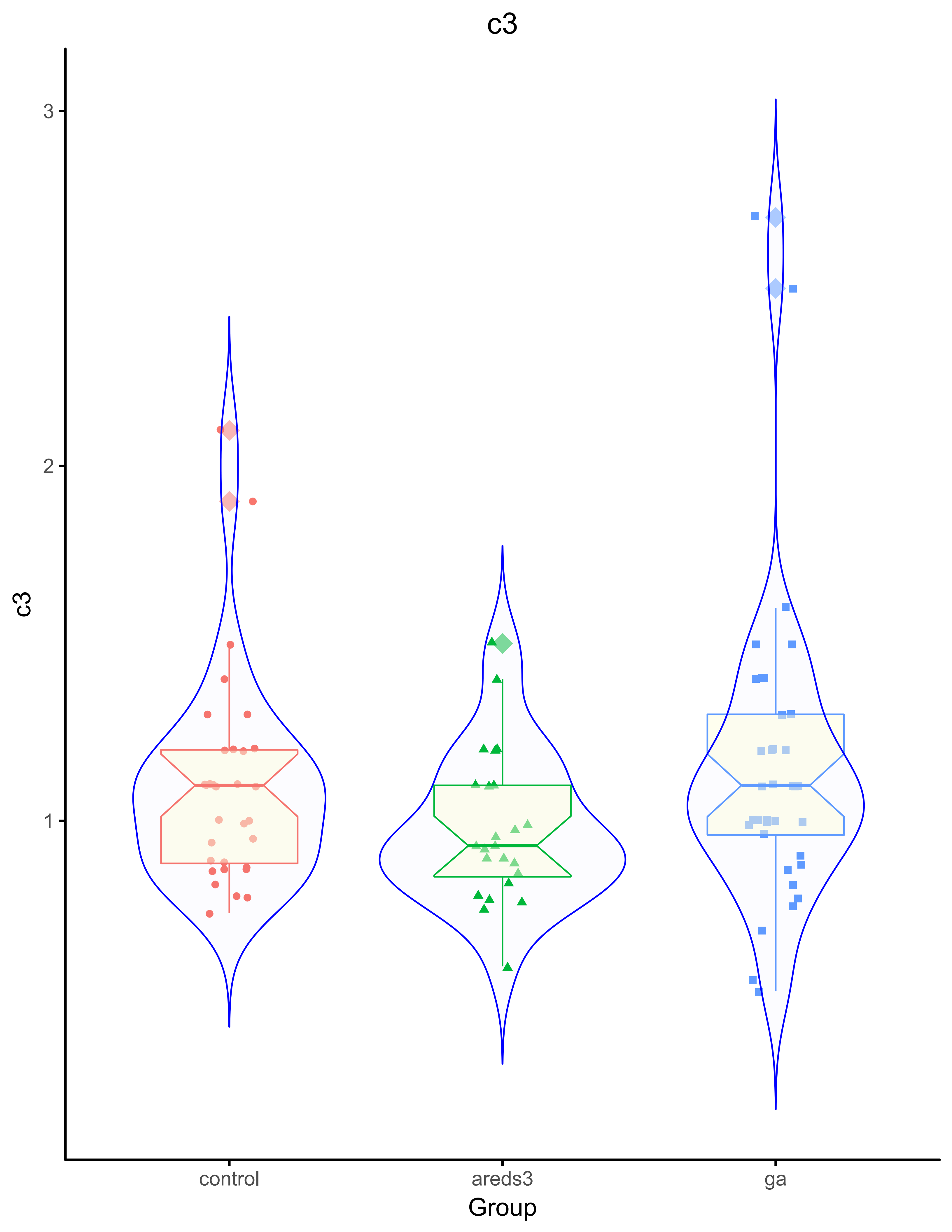
c3
3
2
1
c3
control
areds3
ga
Group

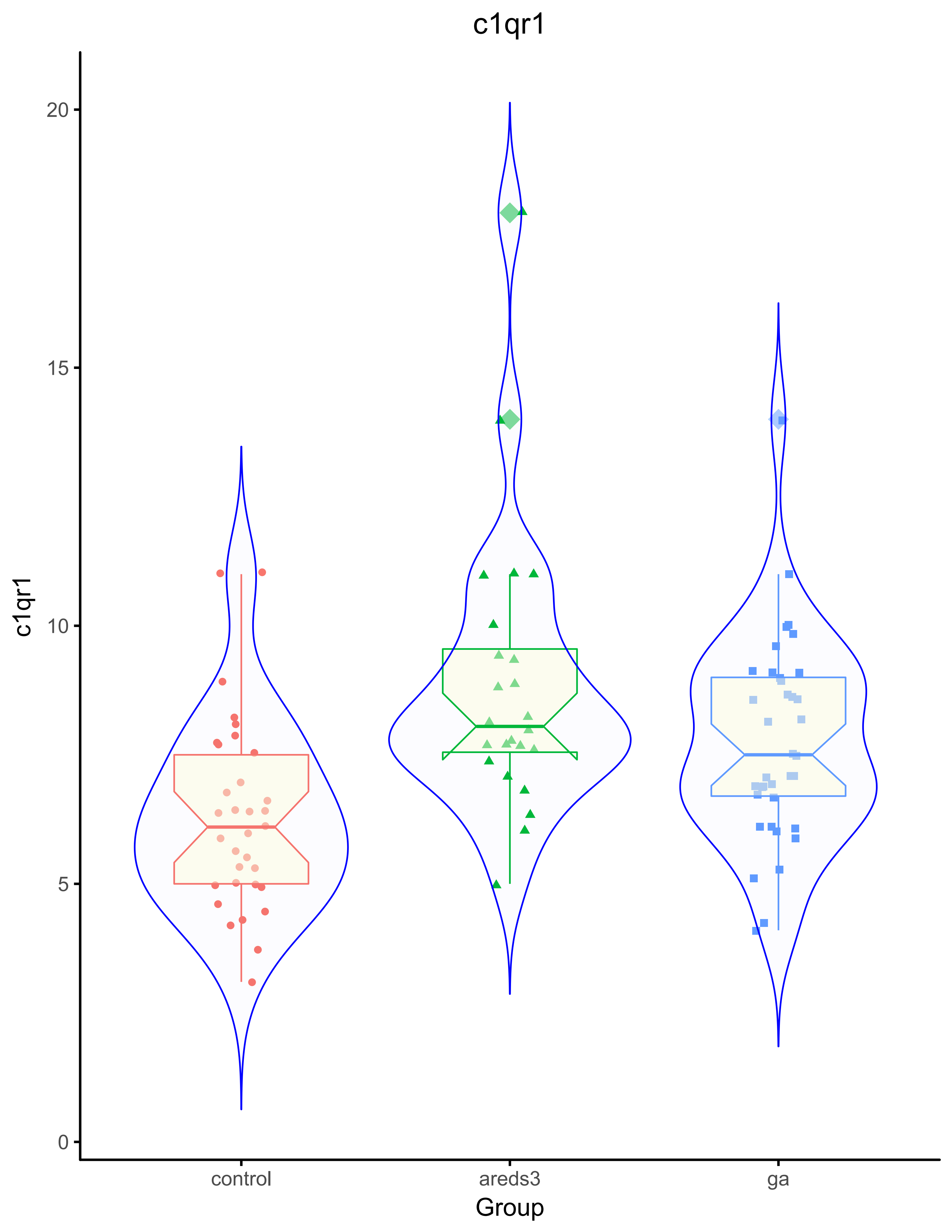

c1qr1
20
15
10
5
0
c1qr1
control
areds3
ga
Group

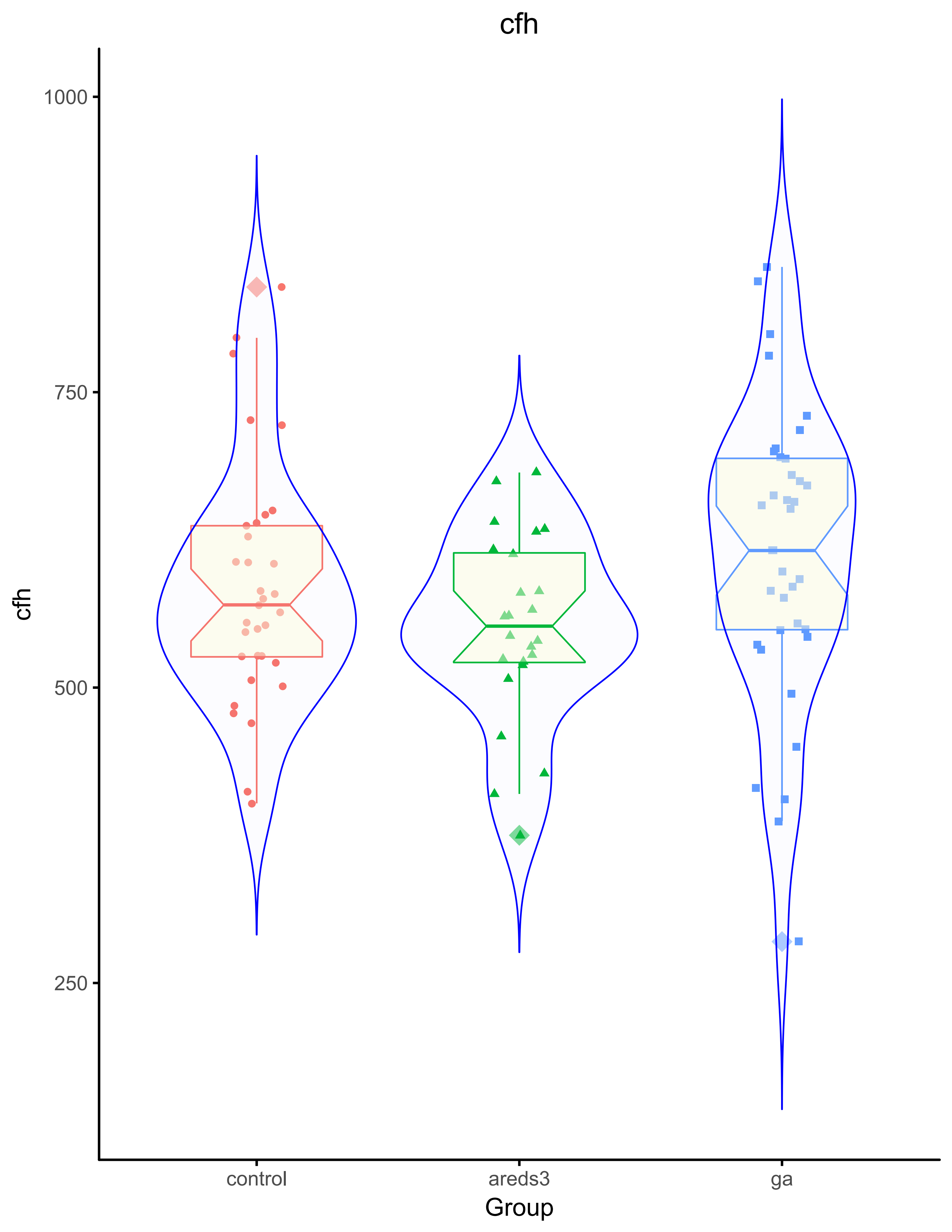

cfh
1000
750
500
250
cfh
control
areds3
ga
Group

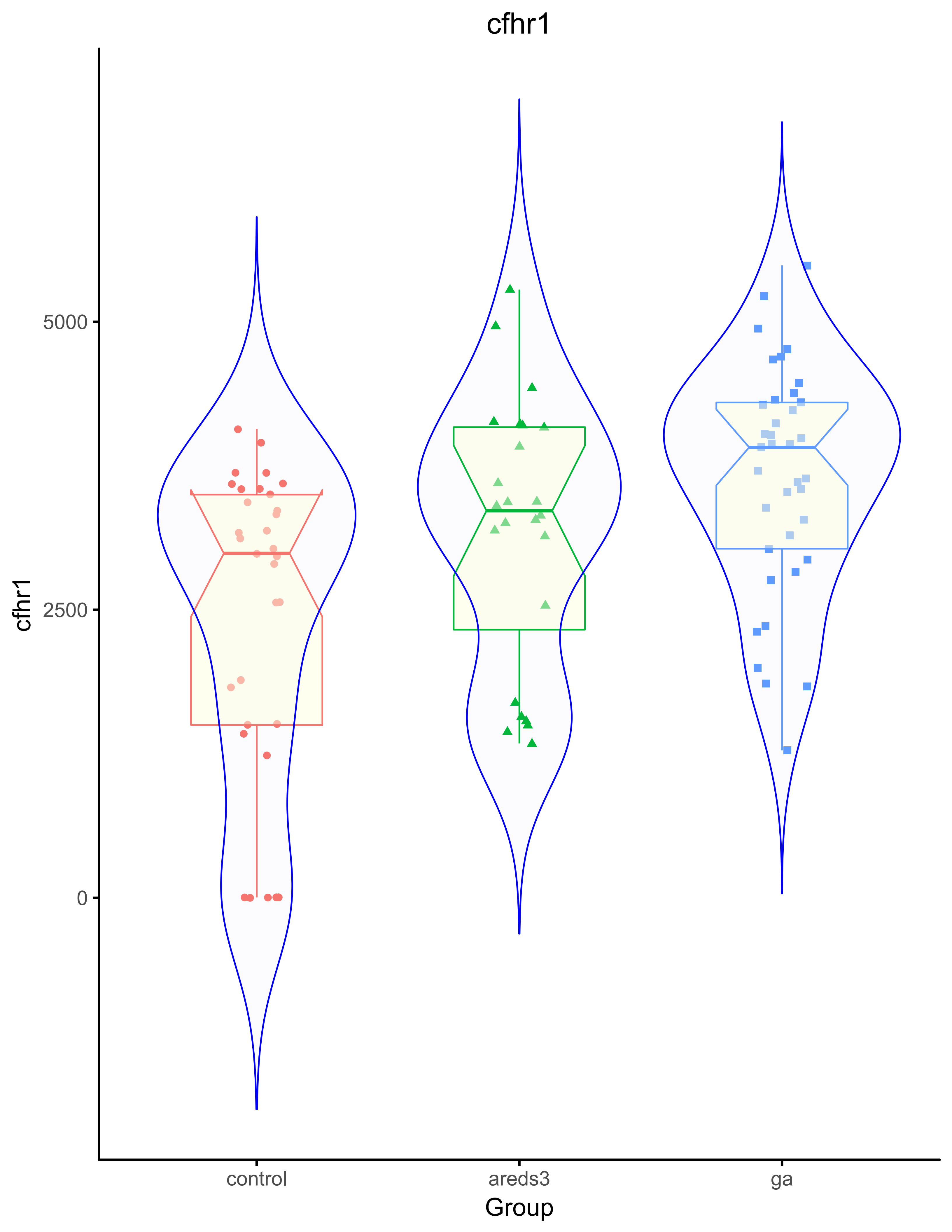
cfhr1
cfhr1
5000
2500
0
control
areds3
ga
Group

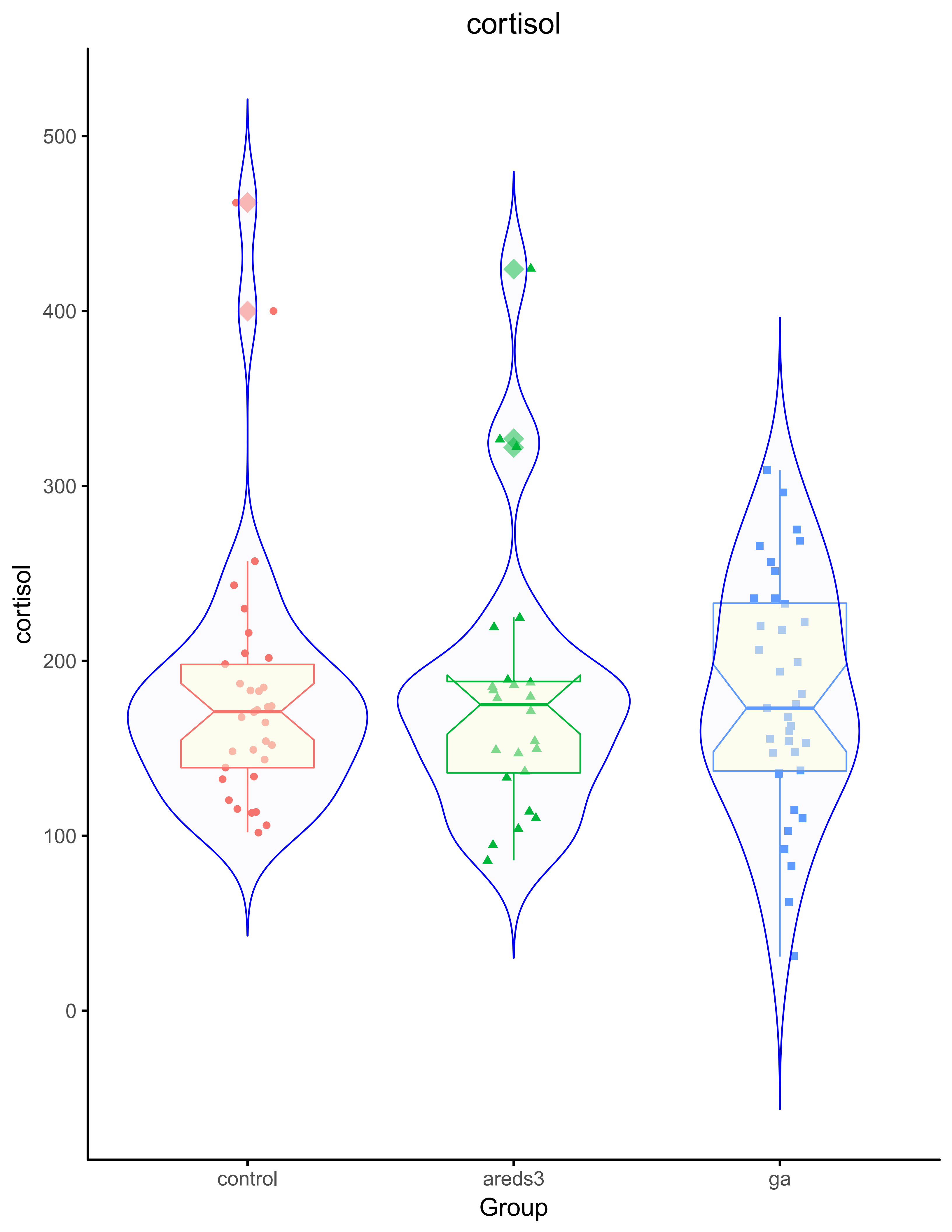
cortisol
500
400
300
200
100
0
cortisol
control
areds3
ga
Group

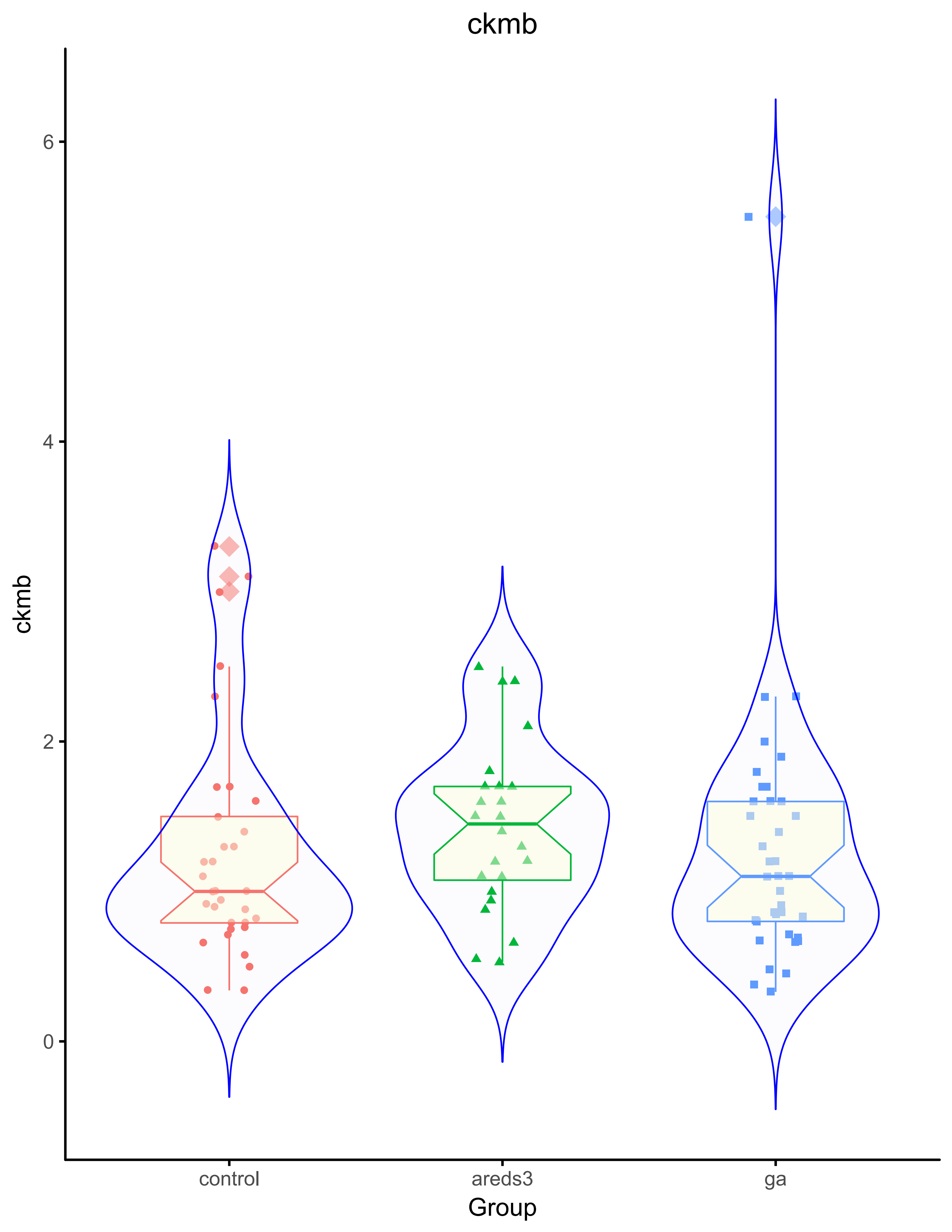
ckmb
6
4
2
0
ckmb
control
areds3
ga
Group

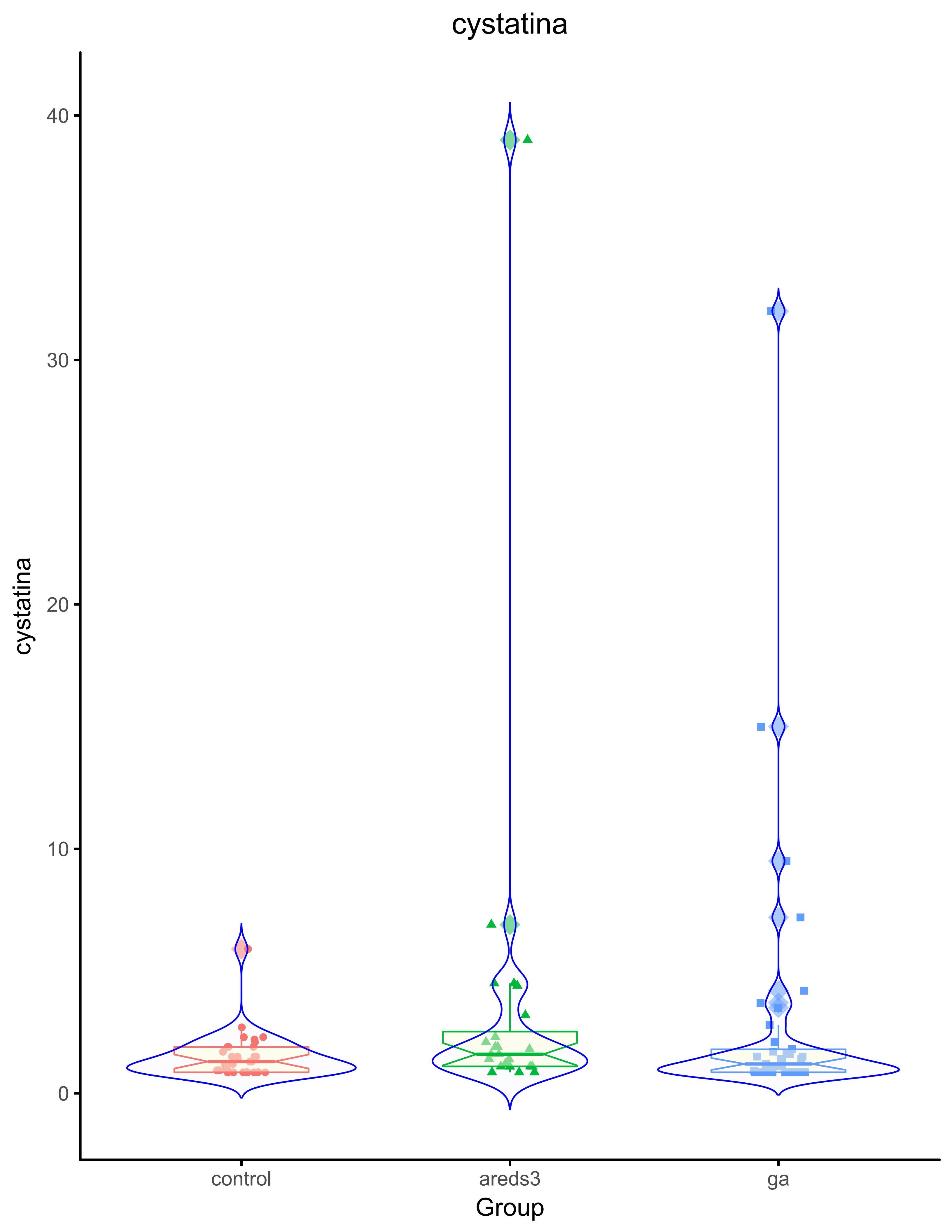
cystatina
40
30
20
10
0
cystatina
control
areds3
ga
Group

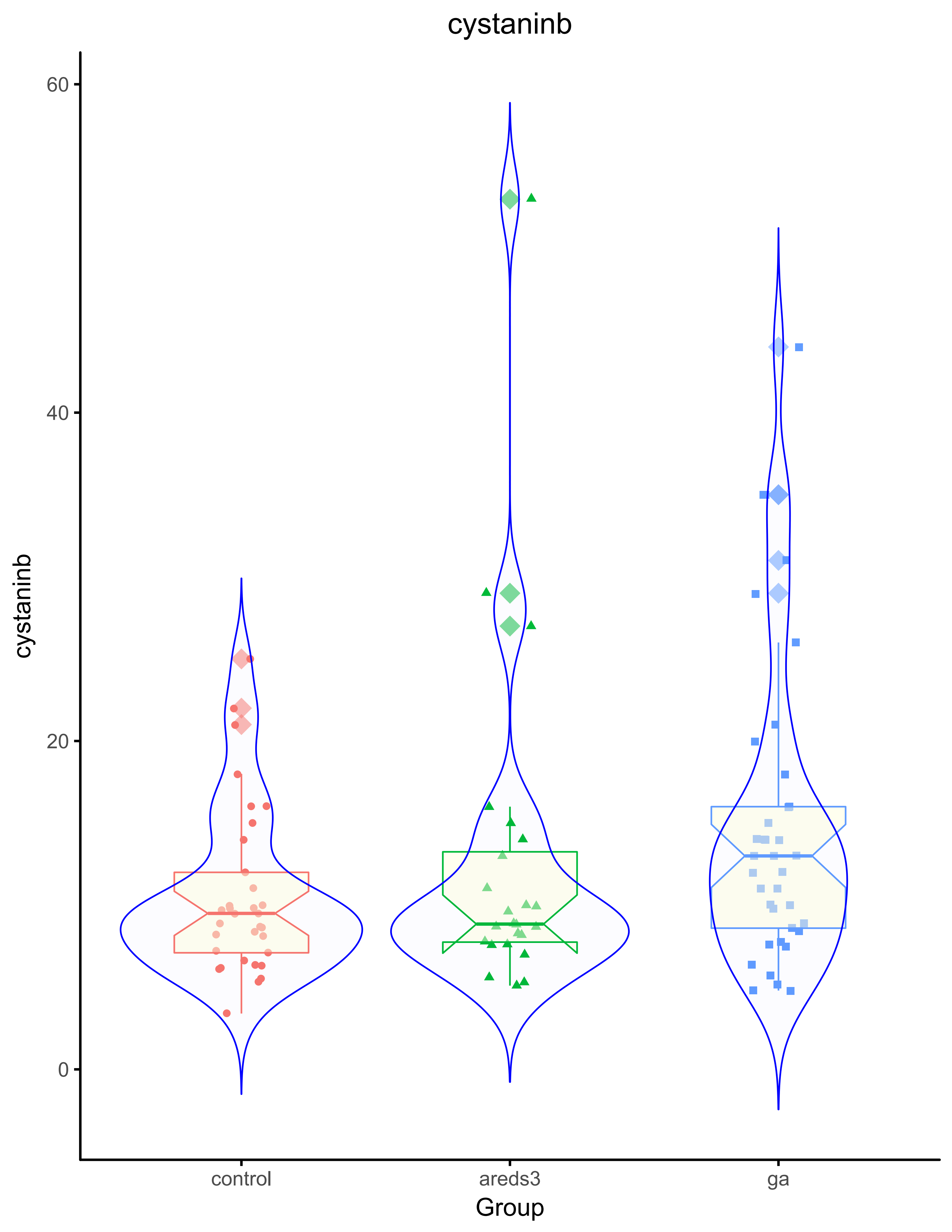
cystaninb
60
40
20
0
cystaninb
control
areds3
ga
Group

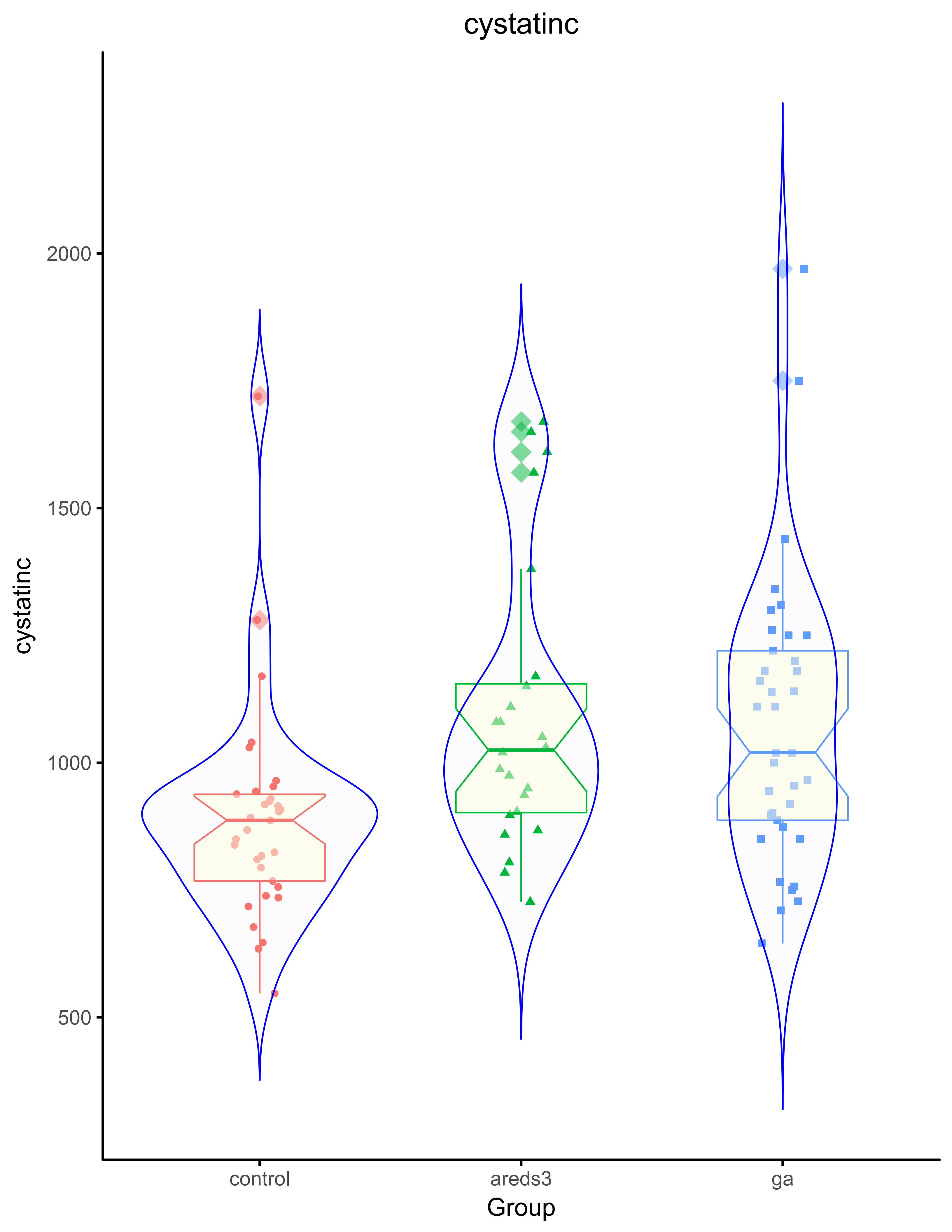
cystatinc
2000
1500
1000
500
cystatinc
control
areds3
ga
Group

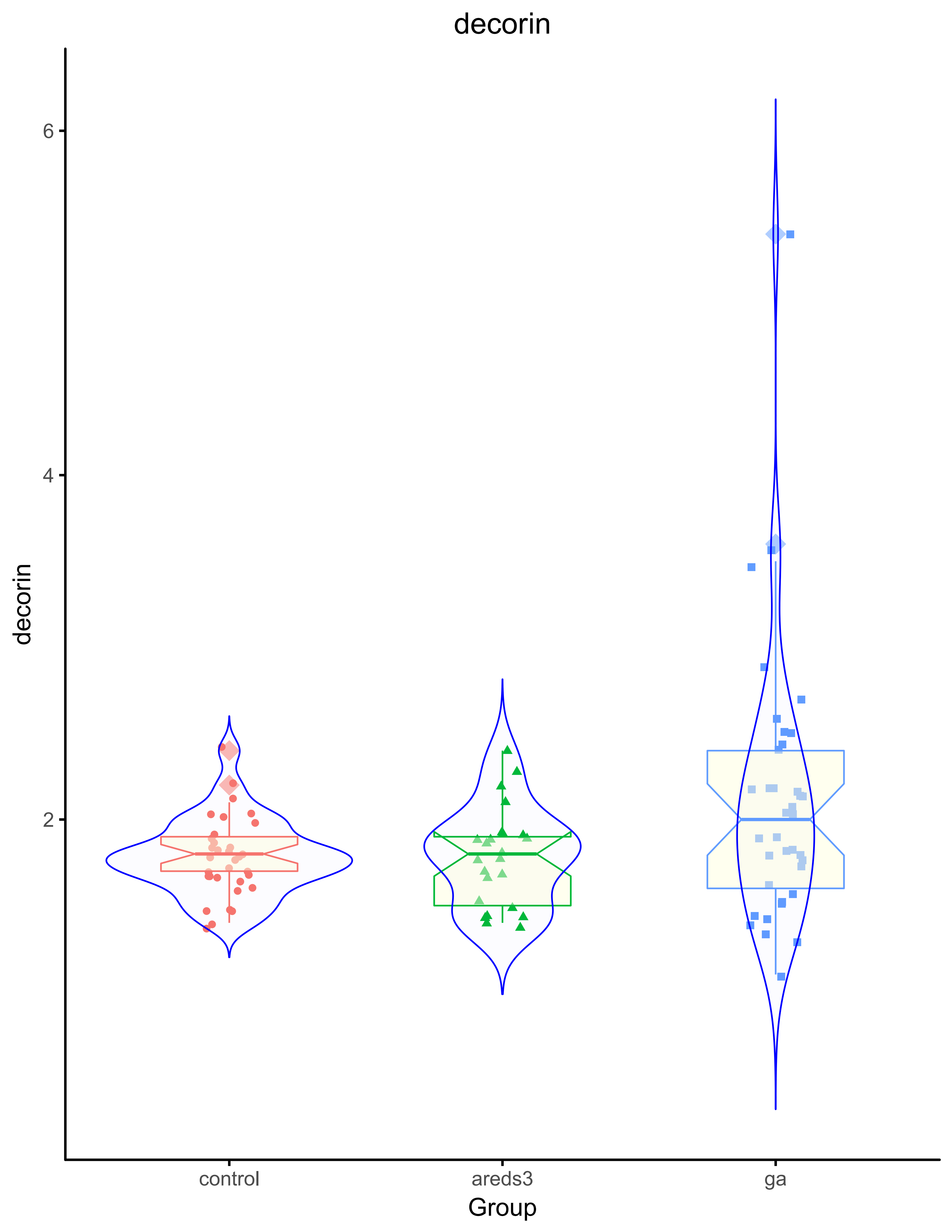

decorin
6
4
2
decorin
control
areds3
ga
Group

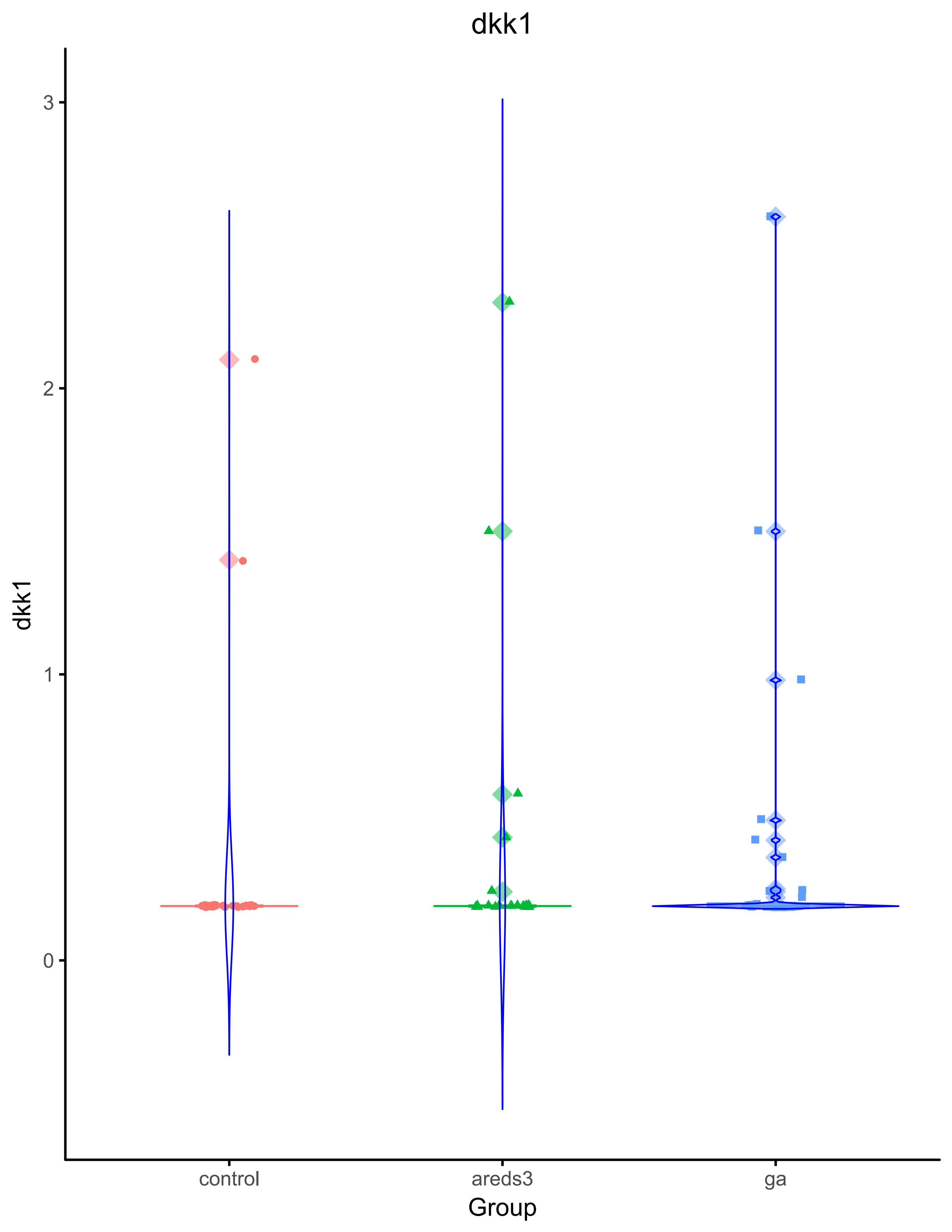
dkk1
3
2
1
0
dkk1
control
areds3
ga
Group

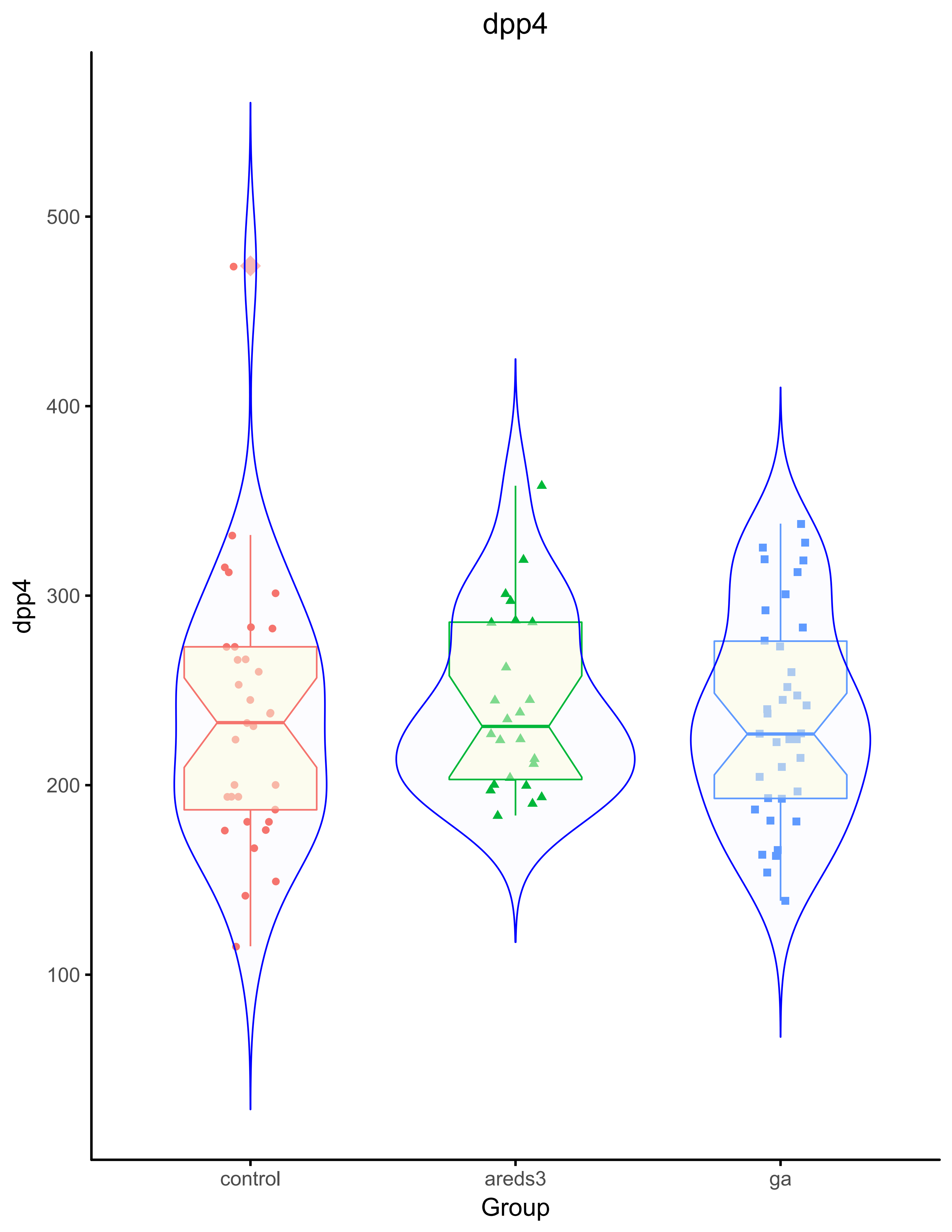
dpp4
500
400
300
200
100
dpp4
control
areds3
ga
Group

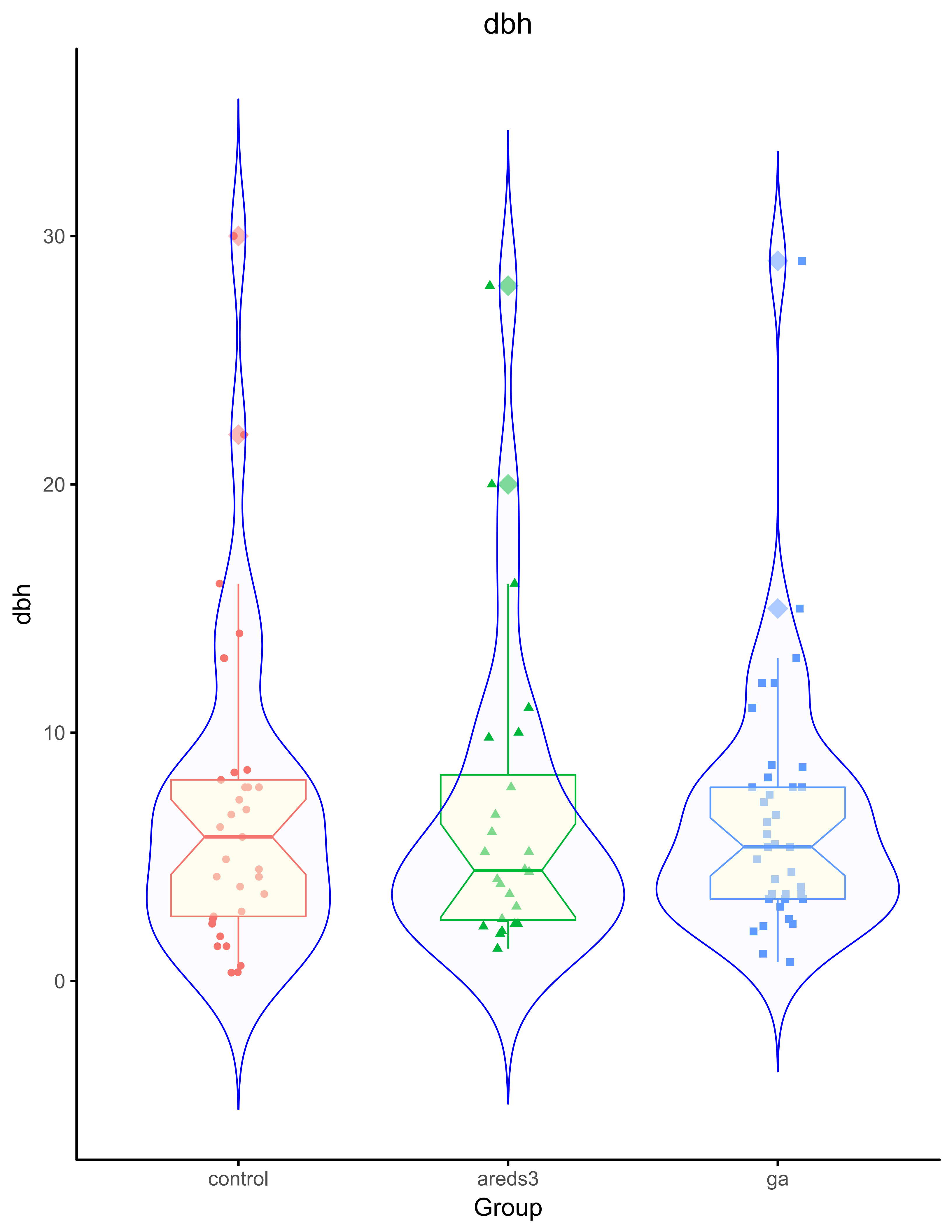
dbh
dbh
30
20
10
0
control
areds3
ga
Group

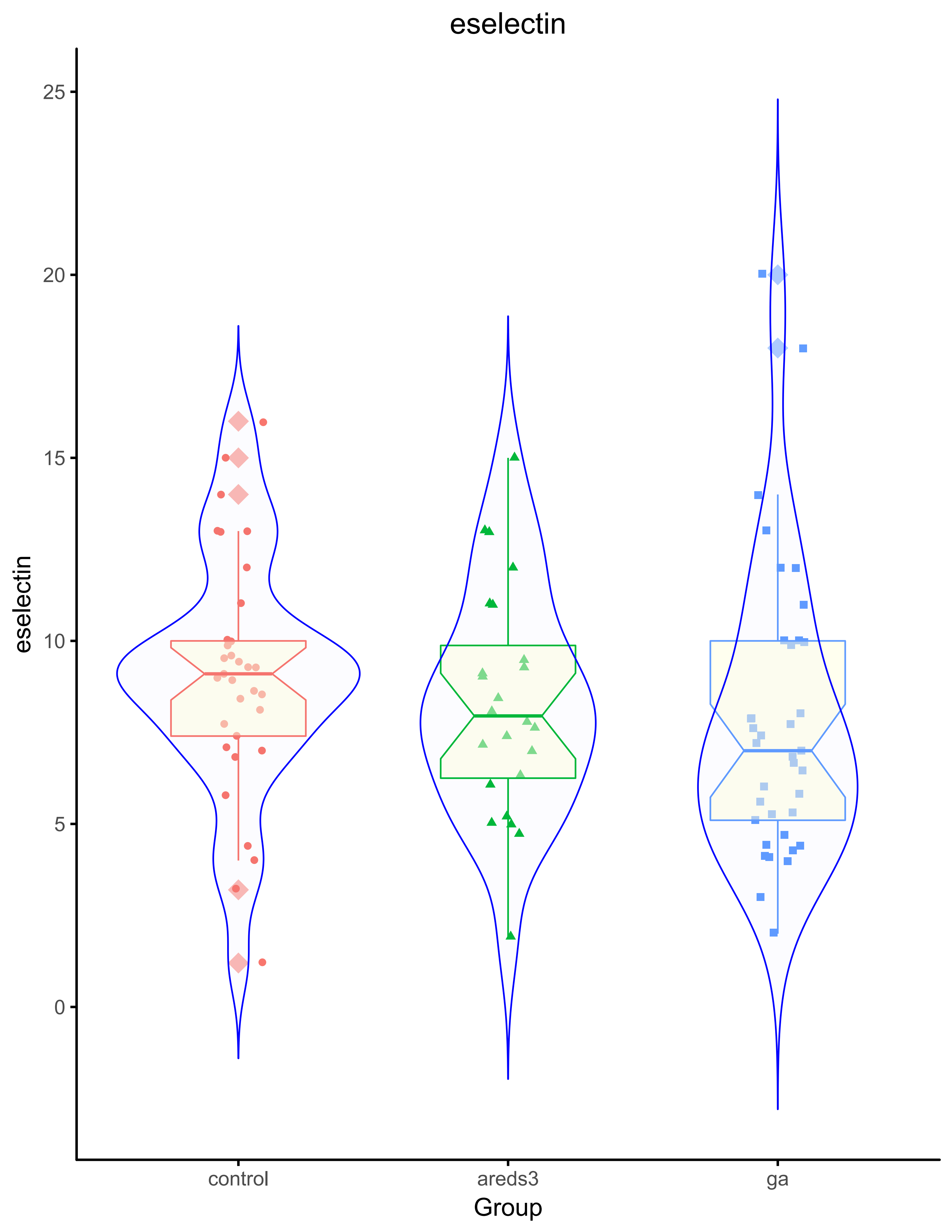
eselectin
25
20
15
10
5
0
eselectin
control
areds3
ga
Group

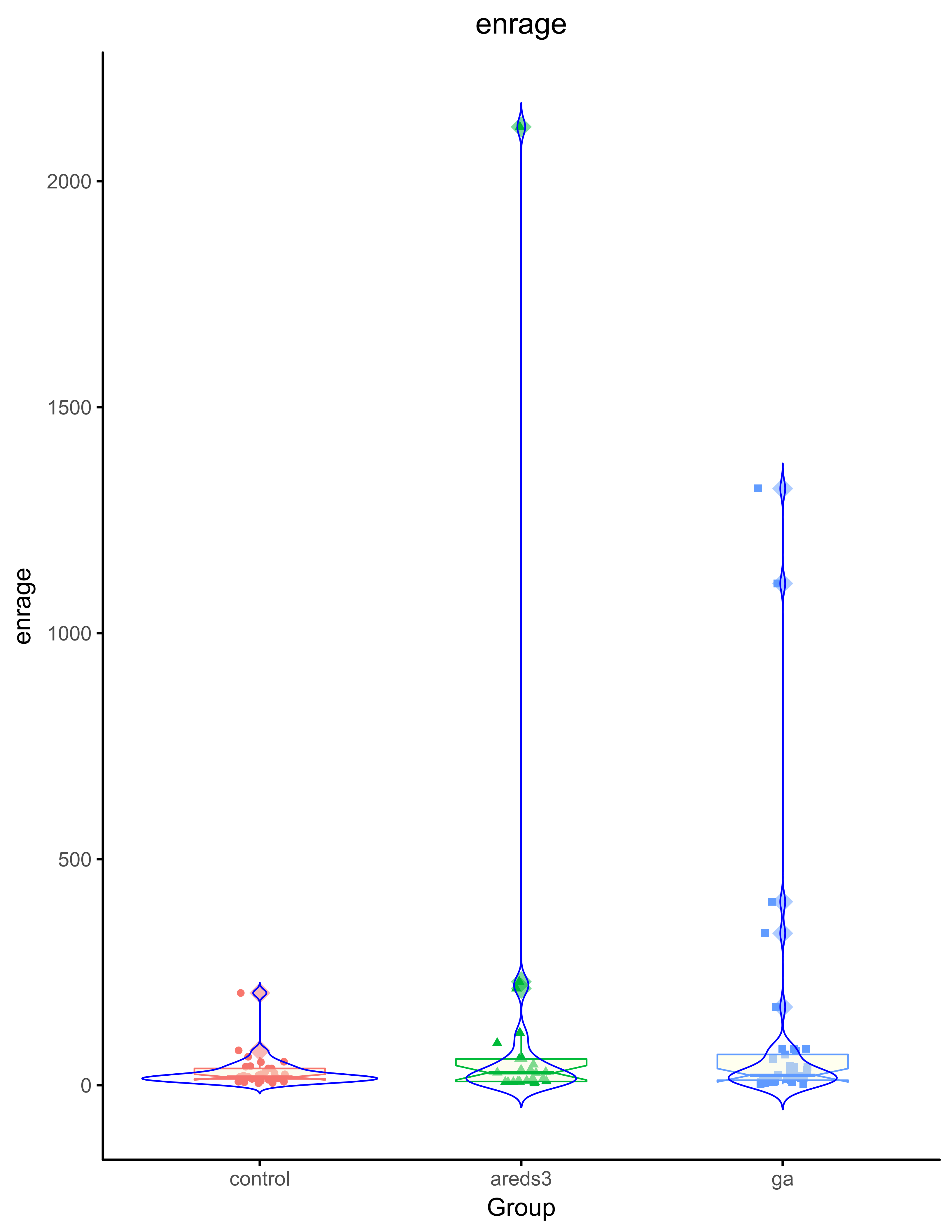
enrage
2000
1500
1000
500
0
enrage
control
areds3
ga
Group

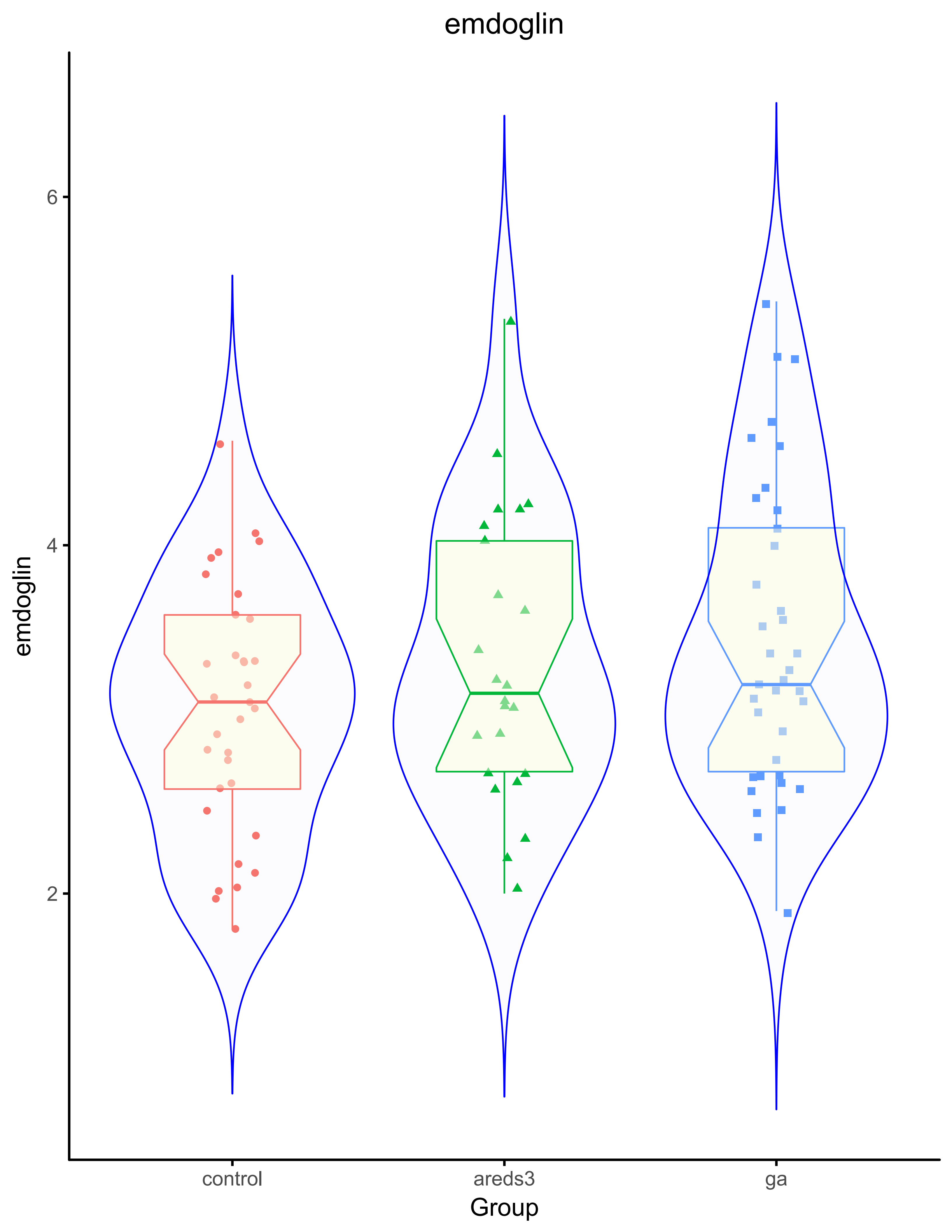

emdoglin
emdoglin
6
4
2
control
areds3
ga
Group

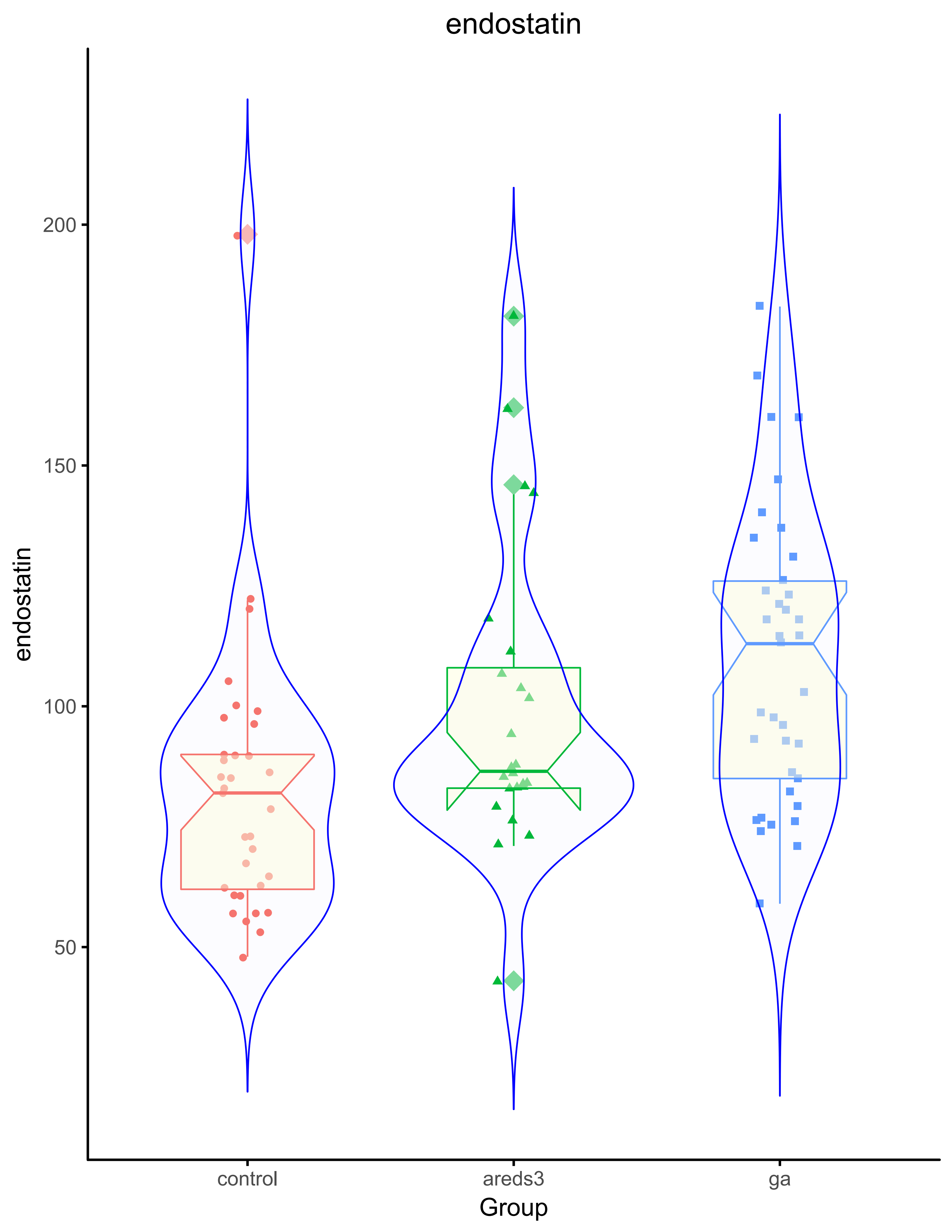
endostatin
endostatin
200
150
100
50
control
areds3
ga
Group

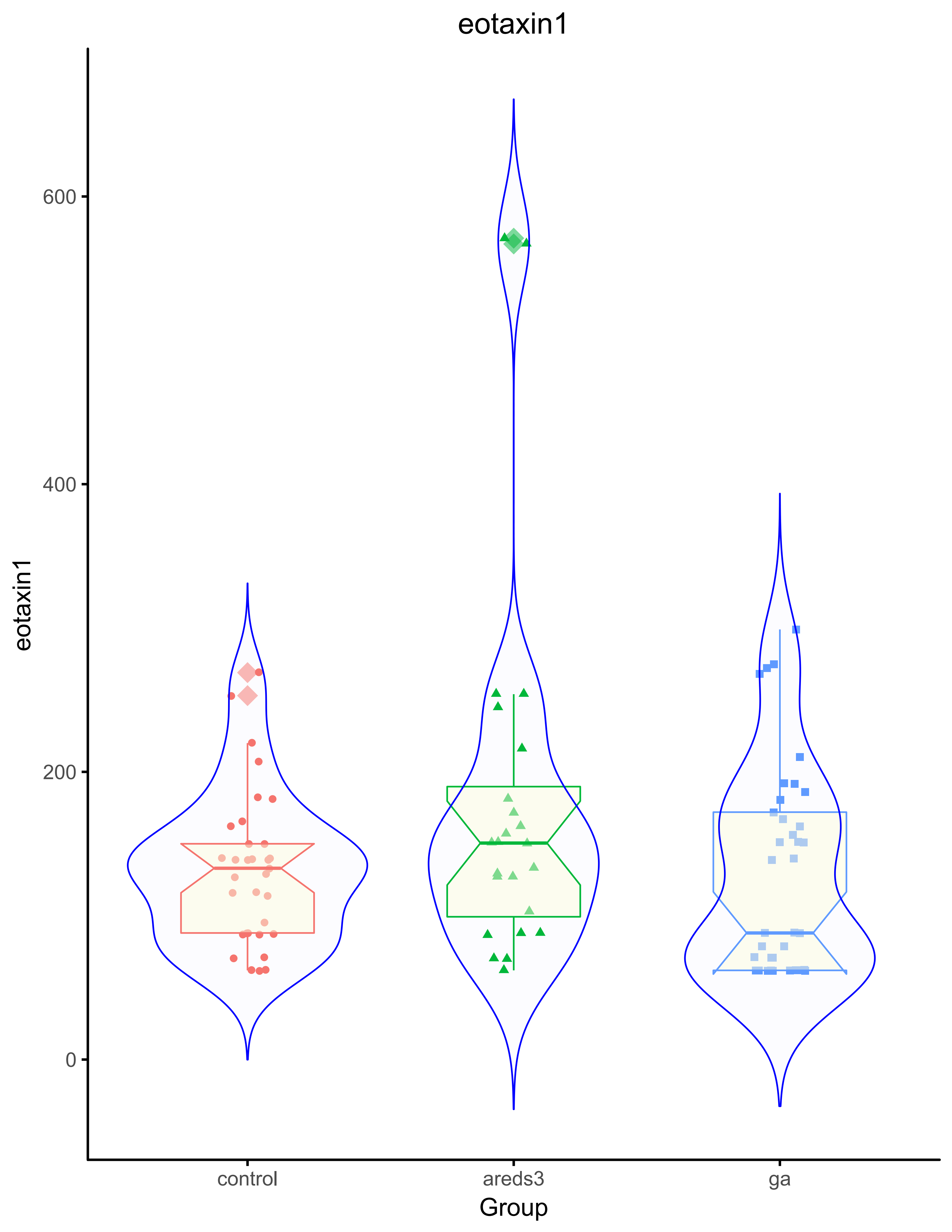

eotaxin1
600
400
200
0
eotaxin1
control
areds3
ga
Group

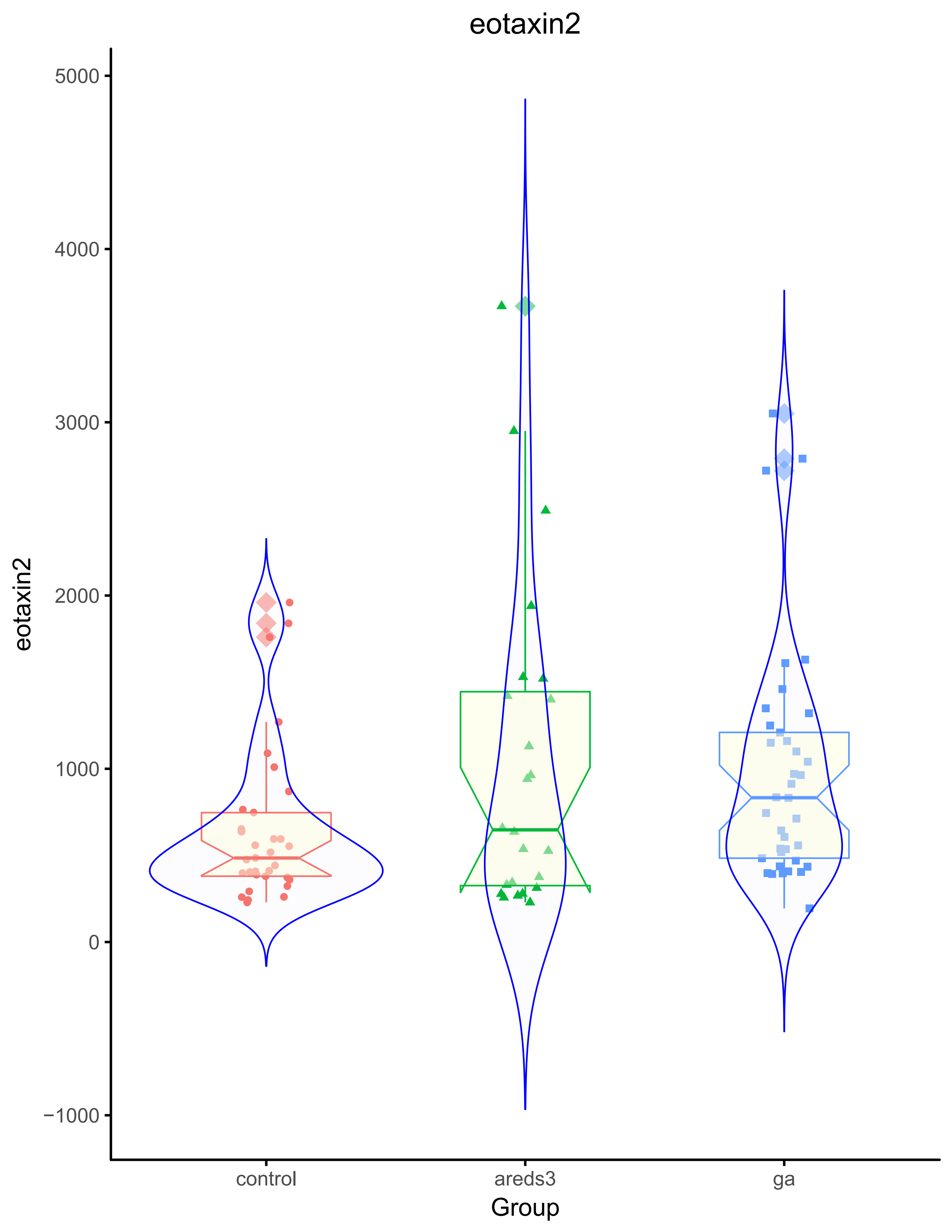

eotaxin2
5000
4000
3000
2000
1000
0
−1000
eotaxin2
control
areds3
ga
Group

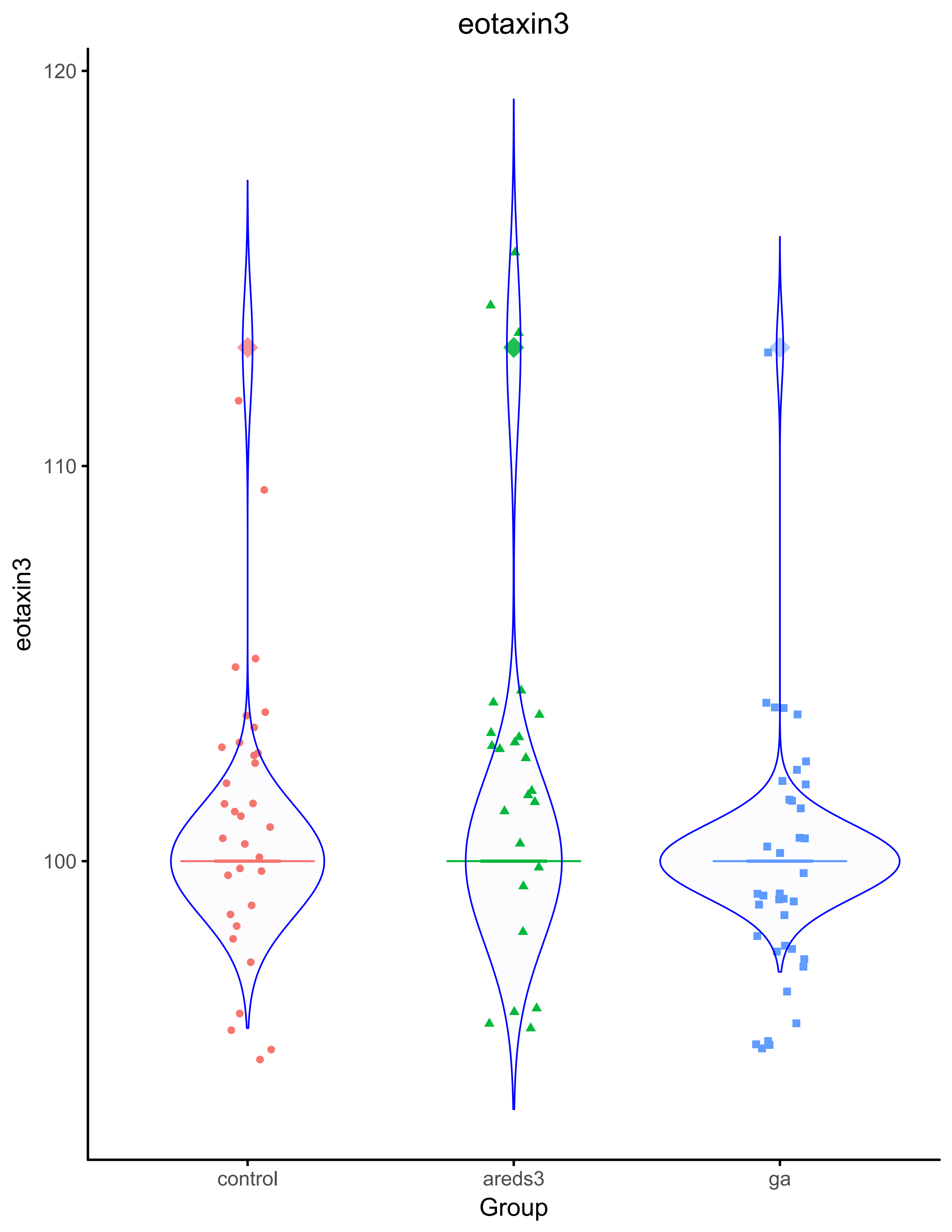

eotaxin3
120
110
100
eotaxin3
control
areds3
ga
Group

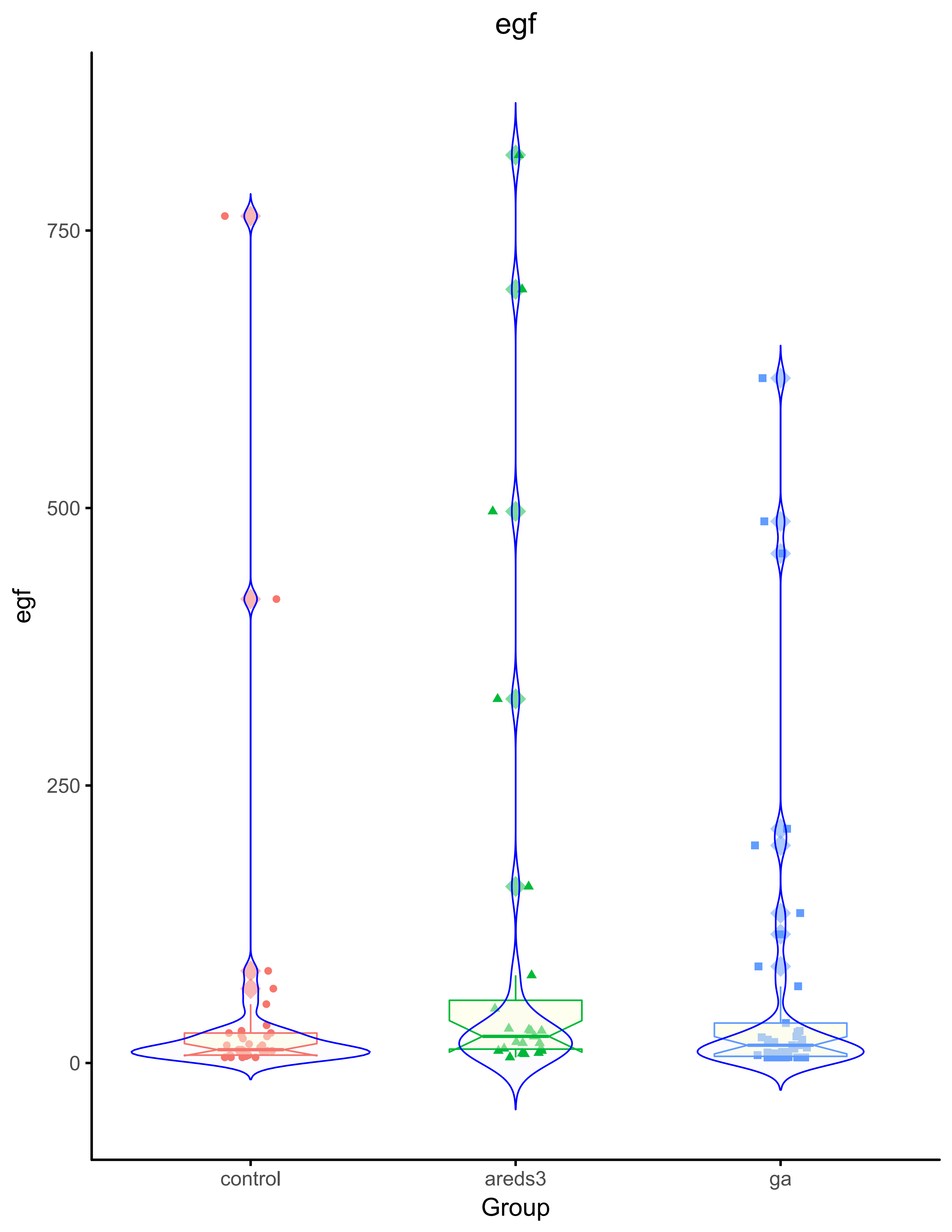
egf
750
500
250
0
egf
control
areds3
ga
Group

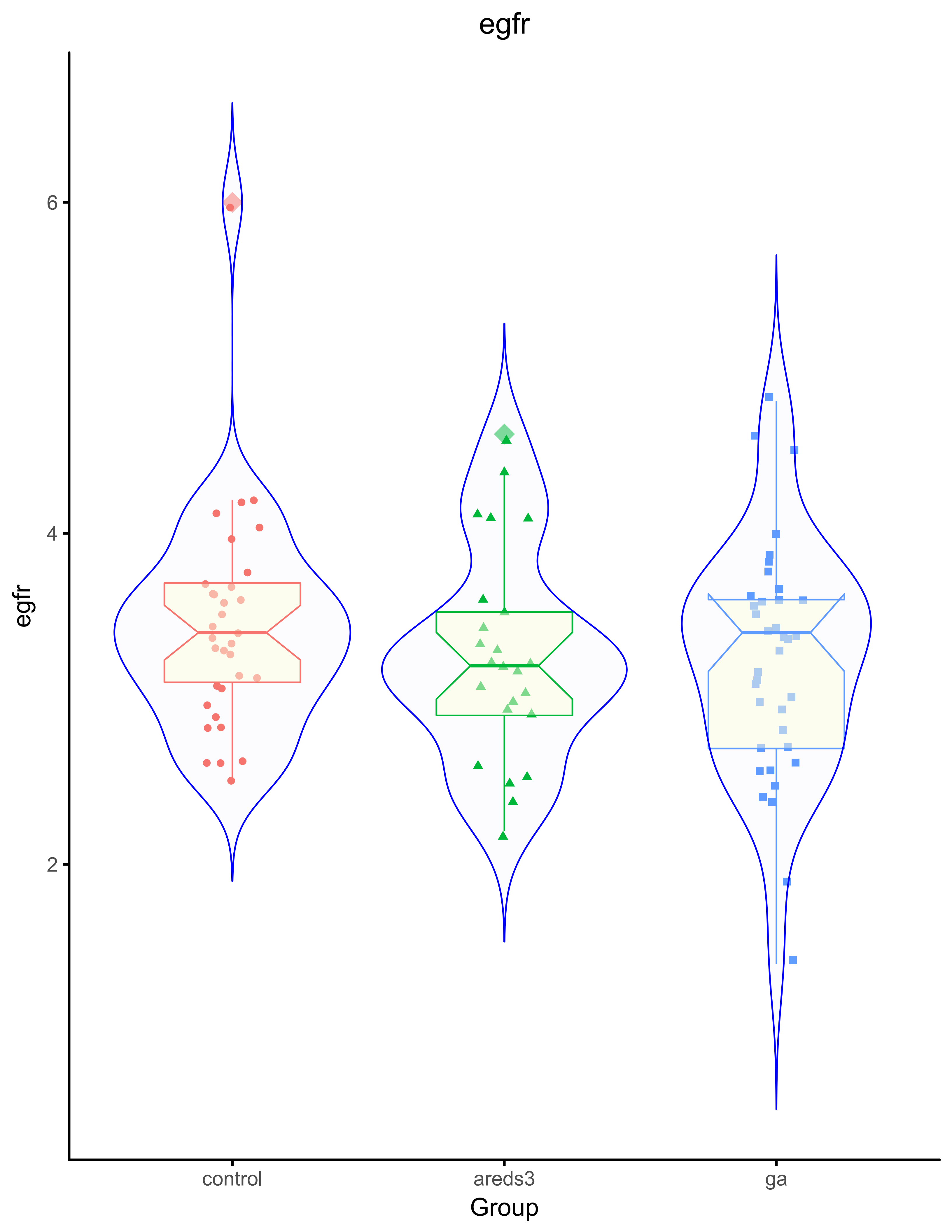

egfr
6
4
2
egfr
control
areds3
ga
Group

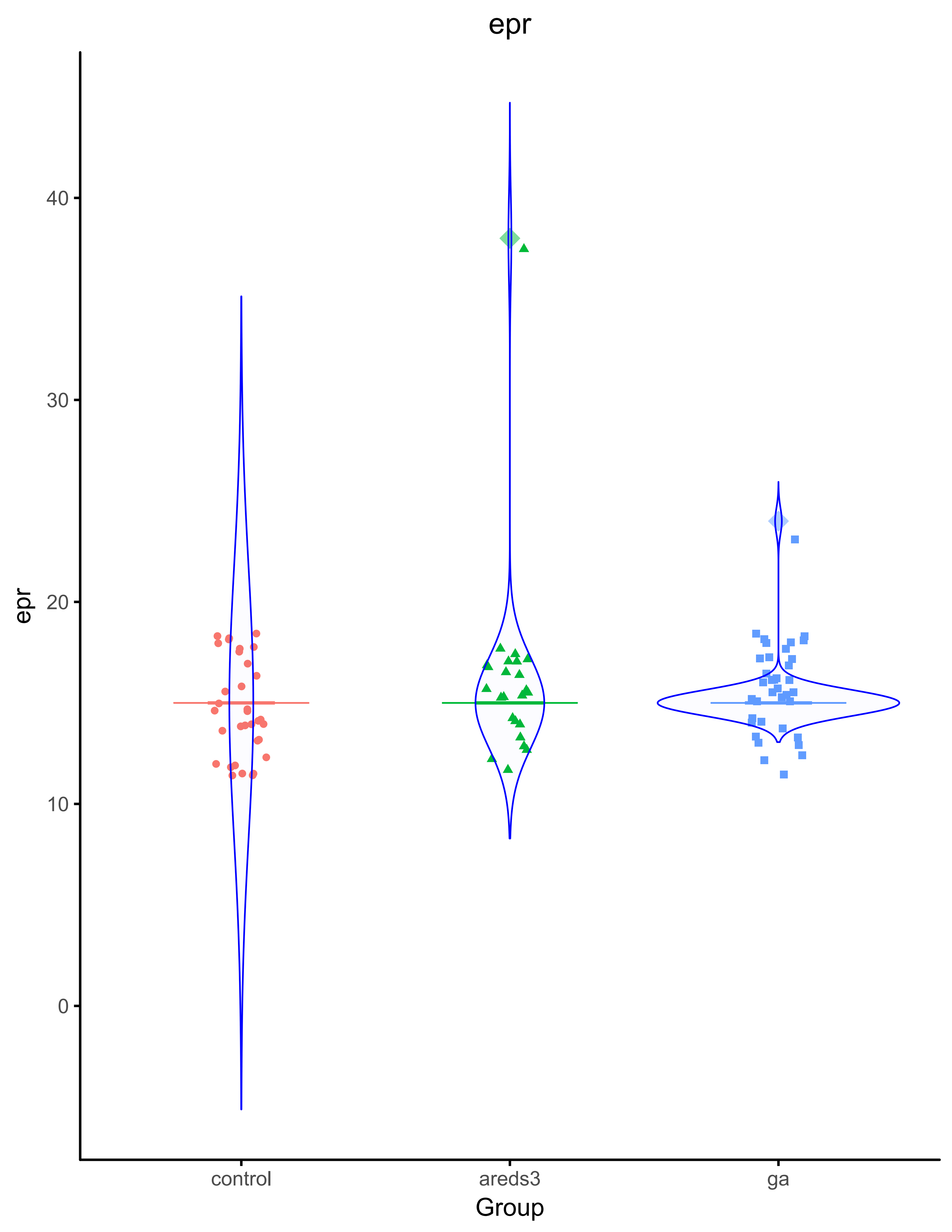

epr
40
30
20
10
0
epr
control
areds3
ga
Group

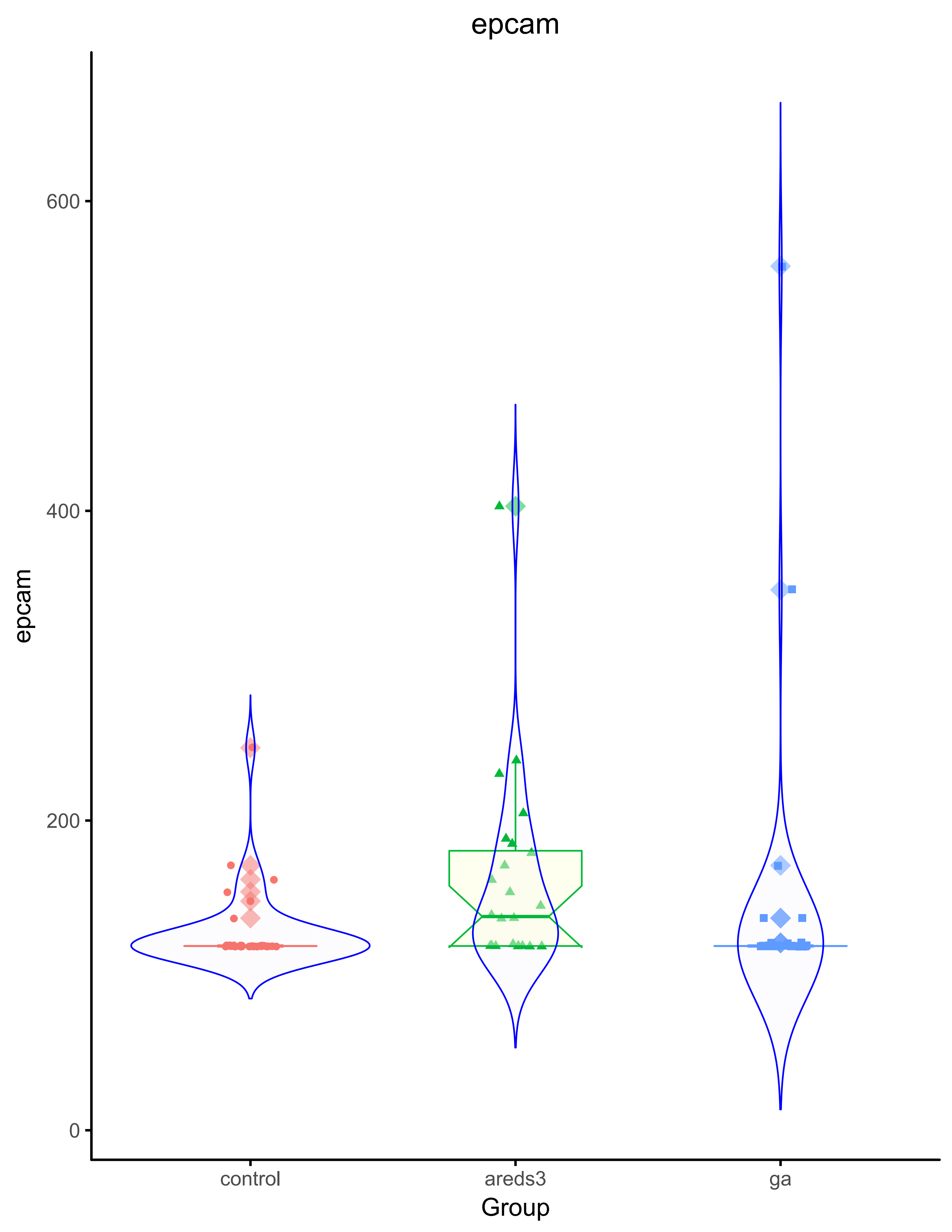
epcam
epcam
600
400
200
0
control
areds3
ga
Group

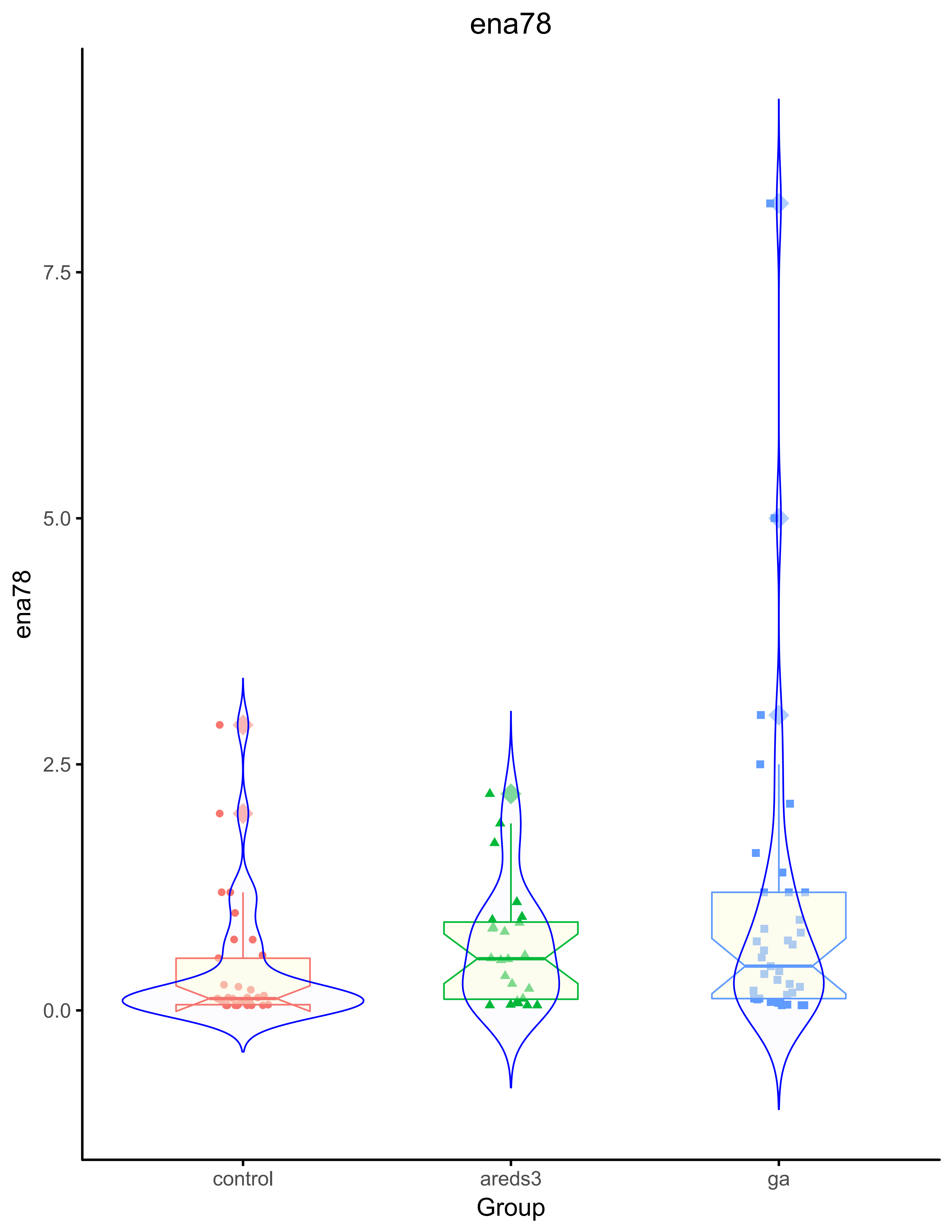

ena78
ena78
7.5
5.0
2.5
0.0
control
areds3
ga
Group

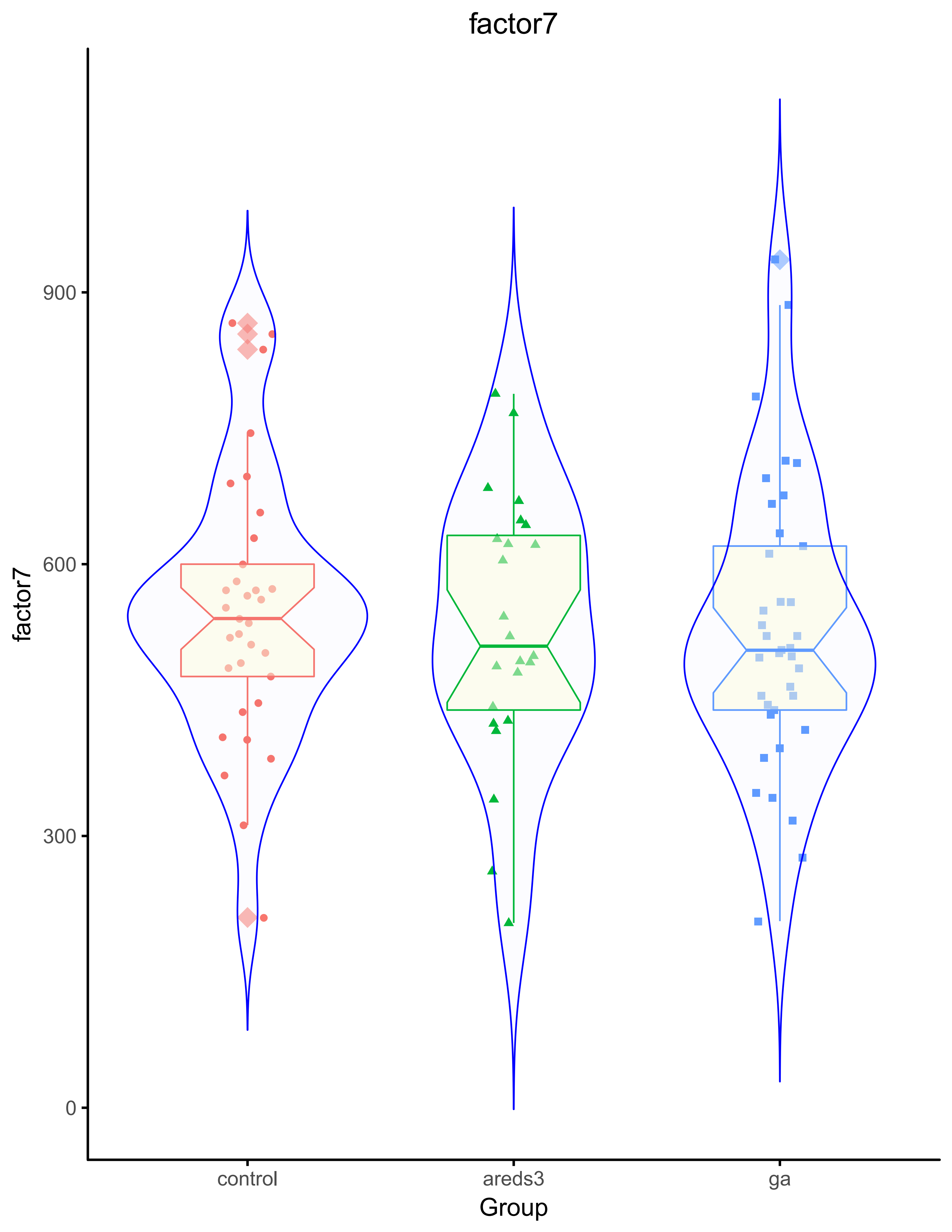
factor7
factor7
900
600
300
0
control
areds3
ga
Group

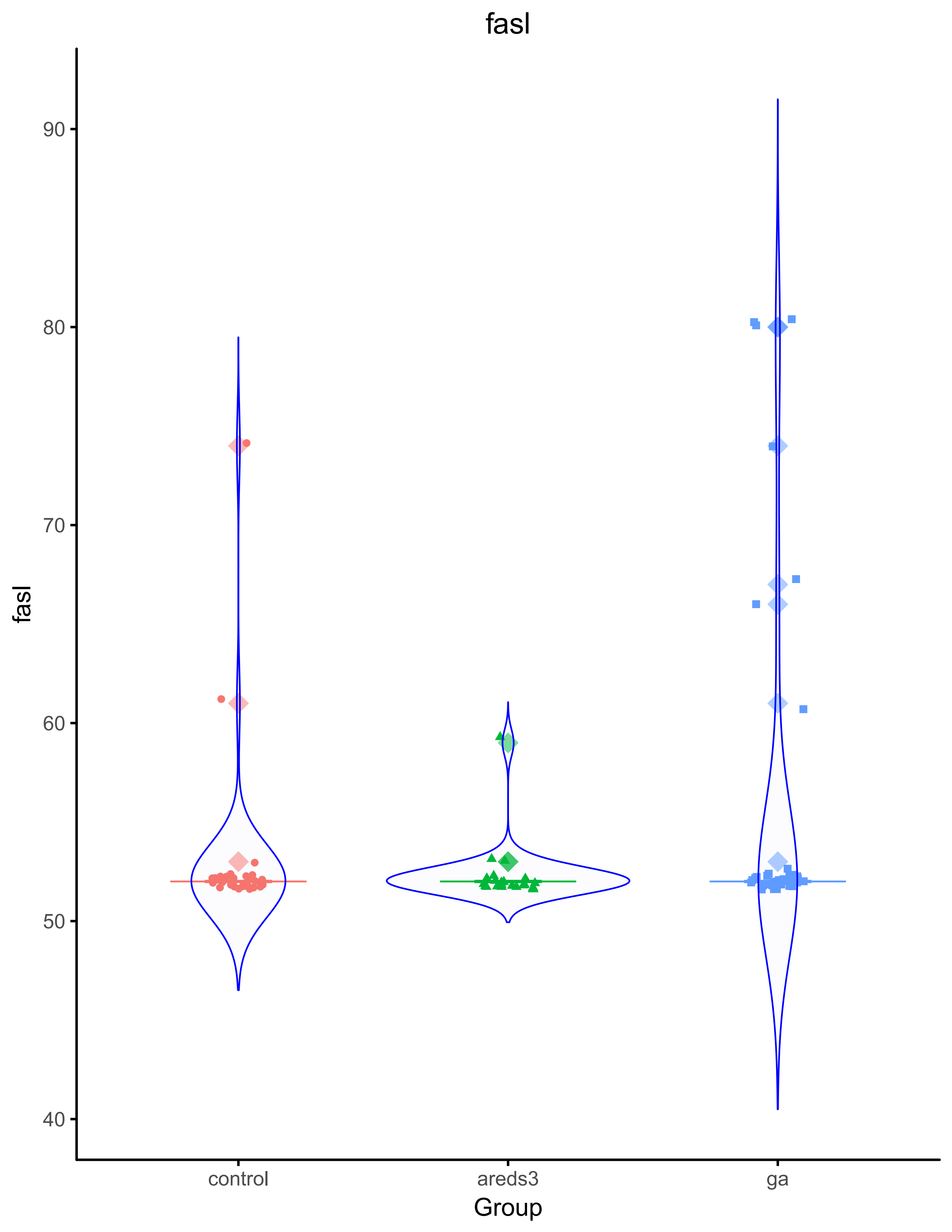

fasl
90
80
70
60
50
40
fasl
control
areds3
ga
Group

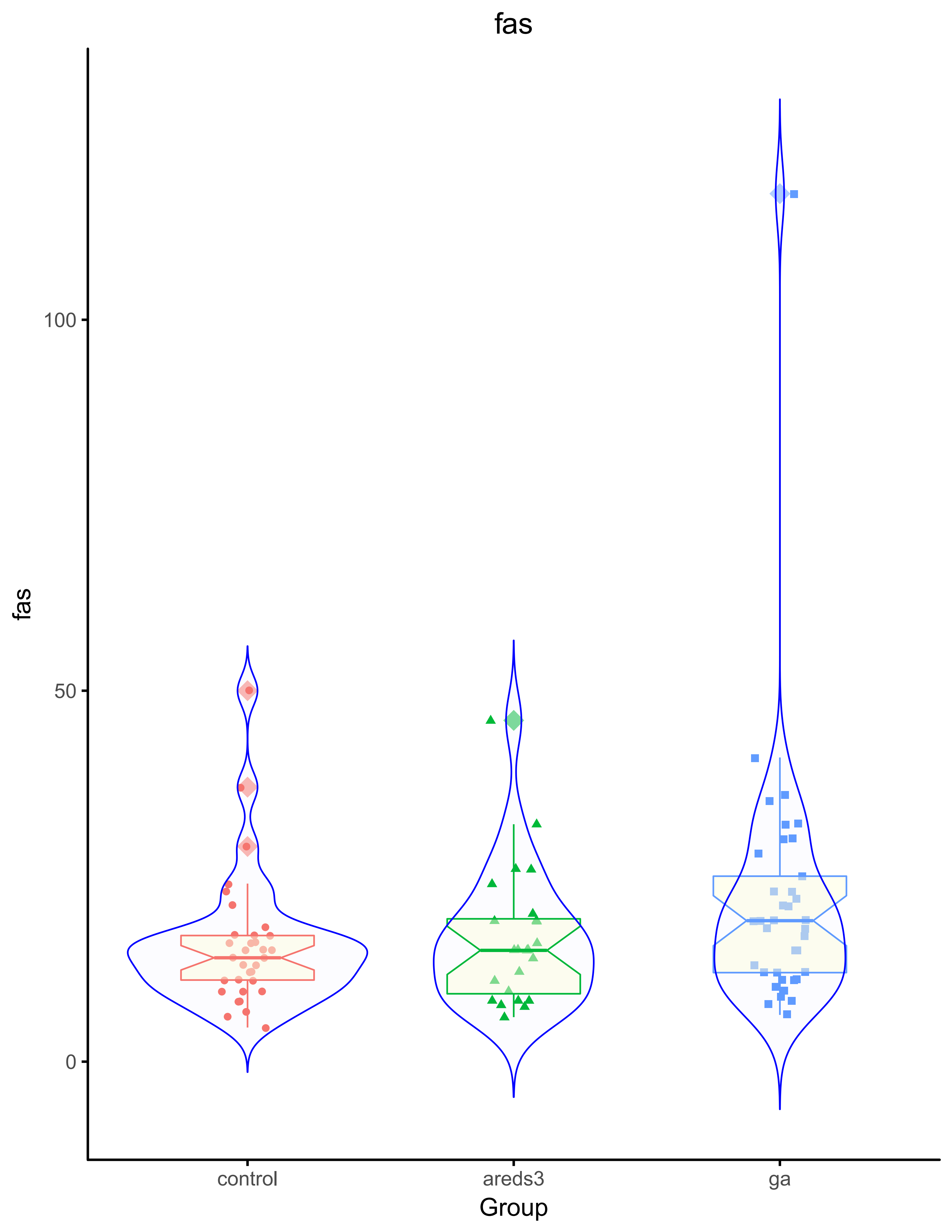
fas
100
50
0
fas
control
areds3
ga
Group

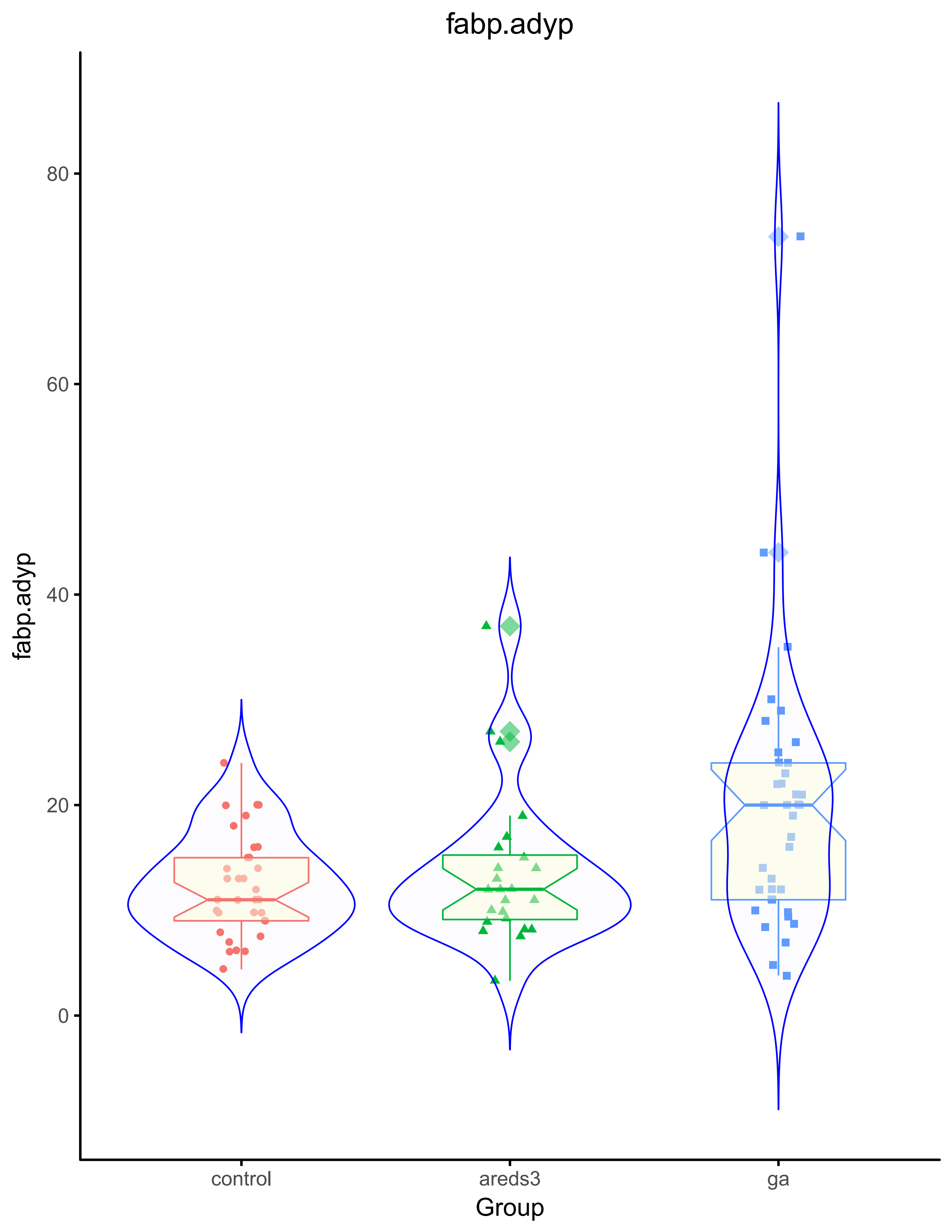
fabp.adyp
fabp.adyp
0
20
40
60
80
control
areds3
ga
Group

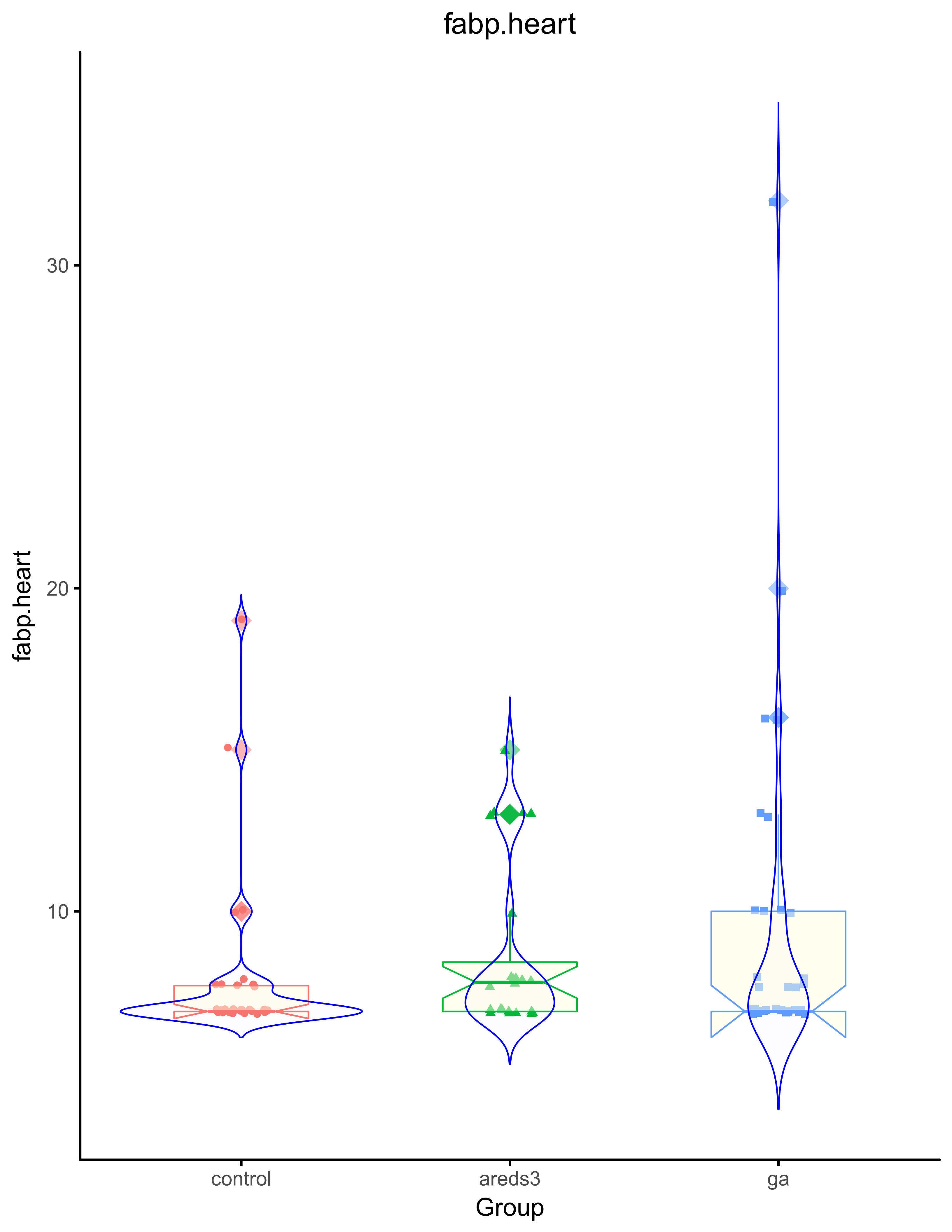
fabp.heart
fabp.heart
30
20
10
control
areds3
ga
Group

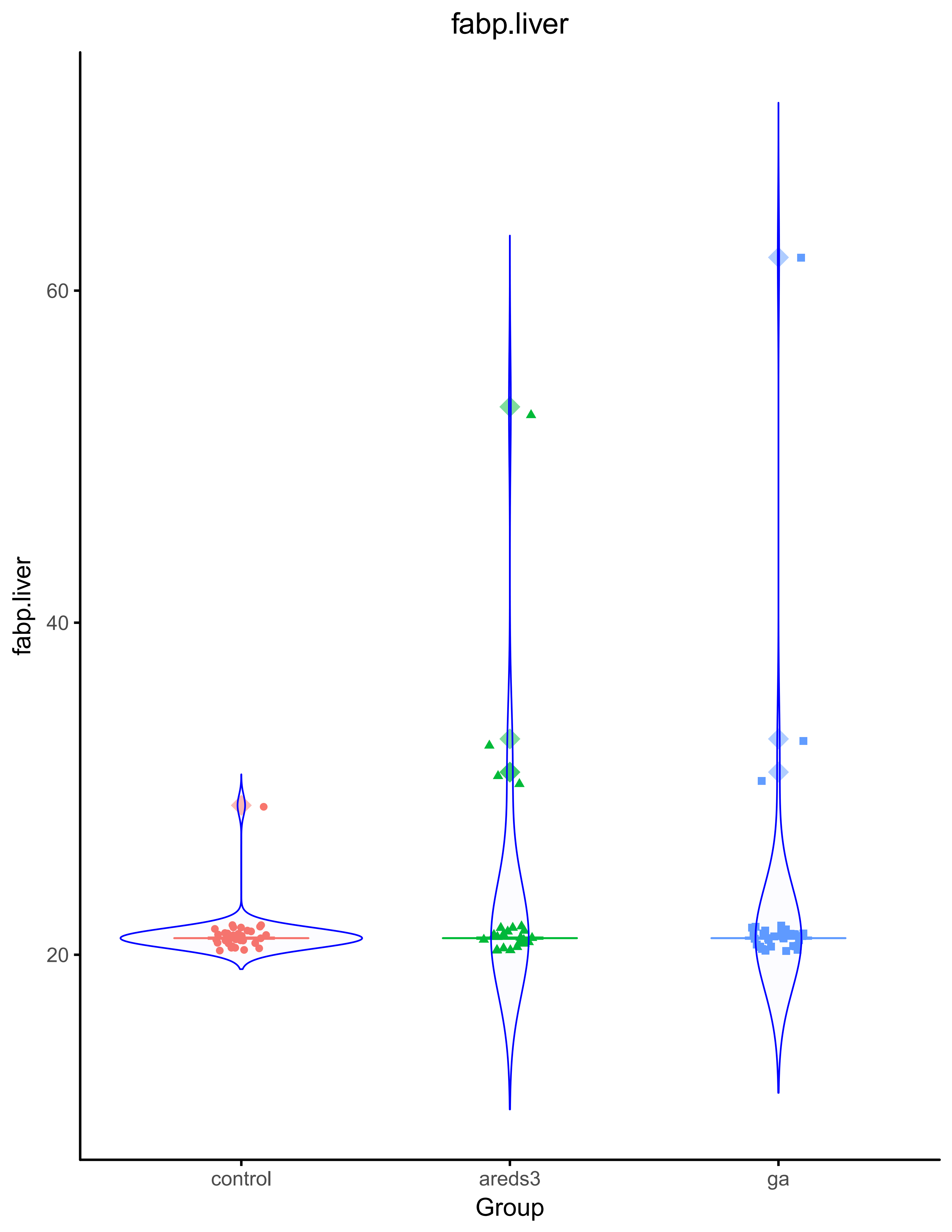
fabp.liver
fabp.liver
60
40
20
control
areds3
ga
Group

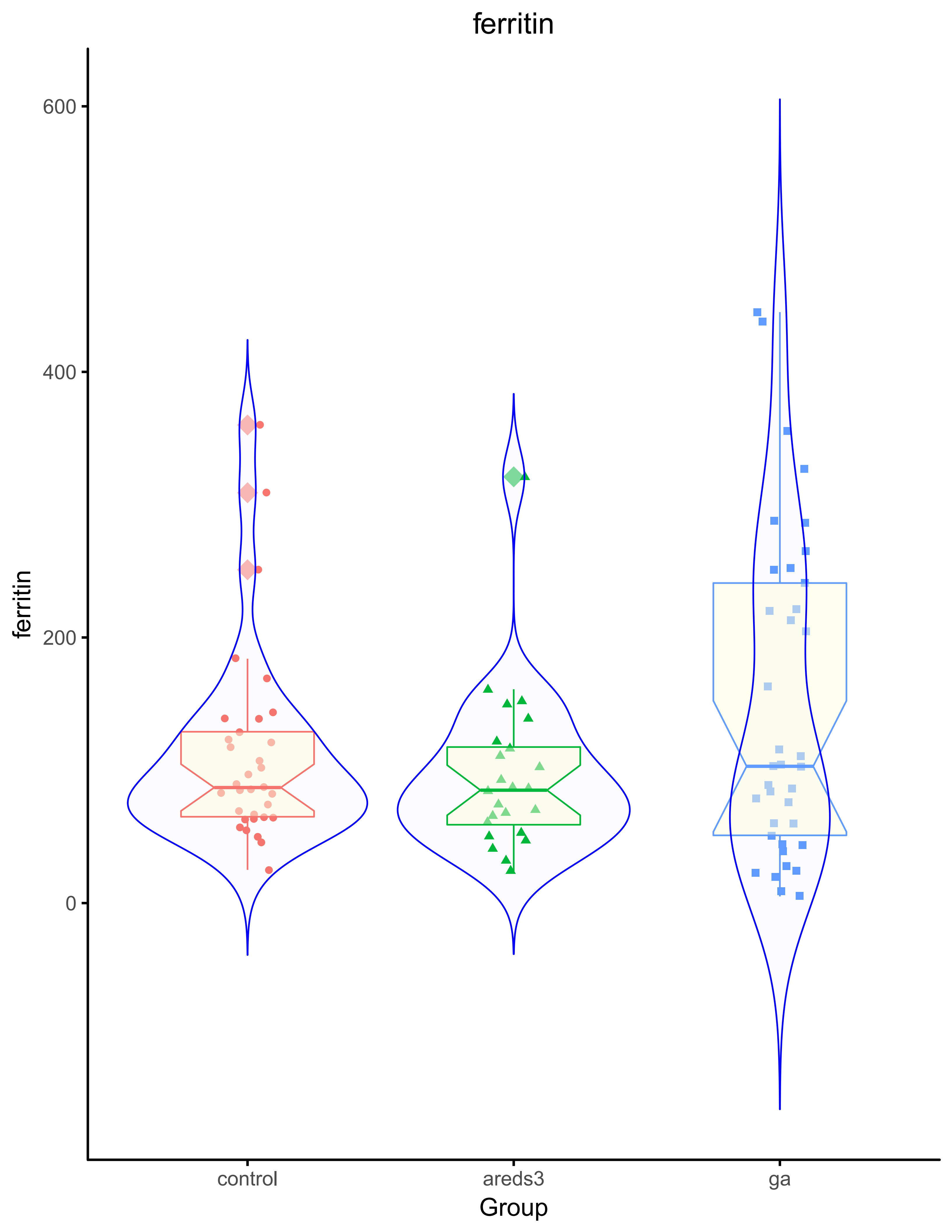

ferritin
600
400
200
0
ferritin
control
areds3
ga
Group

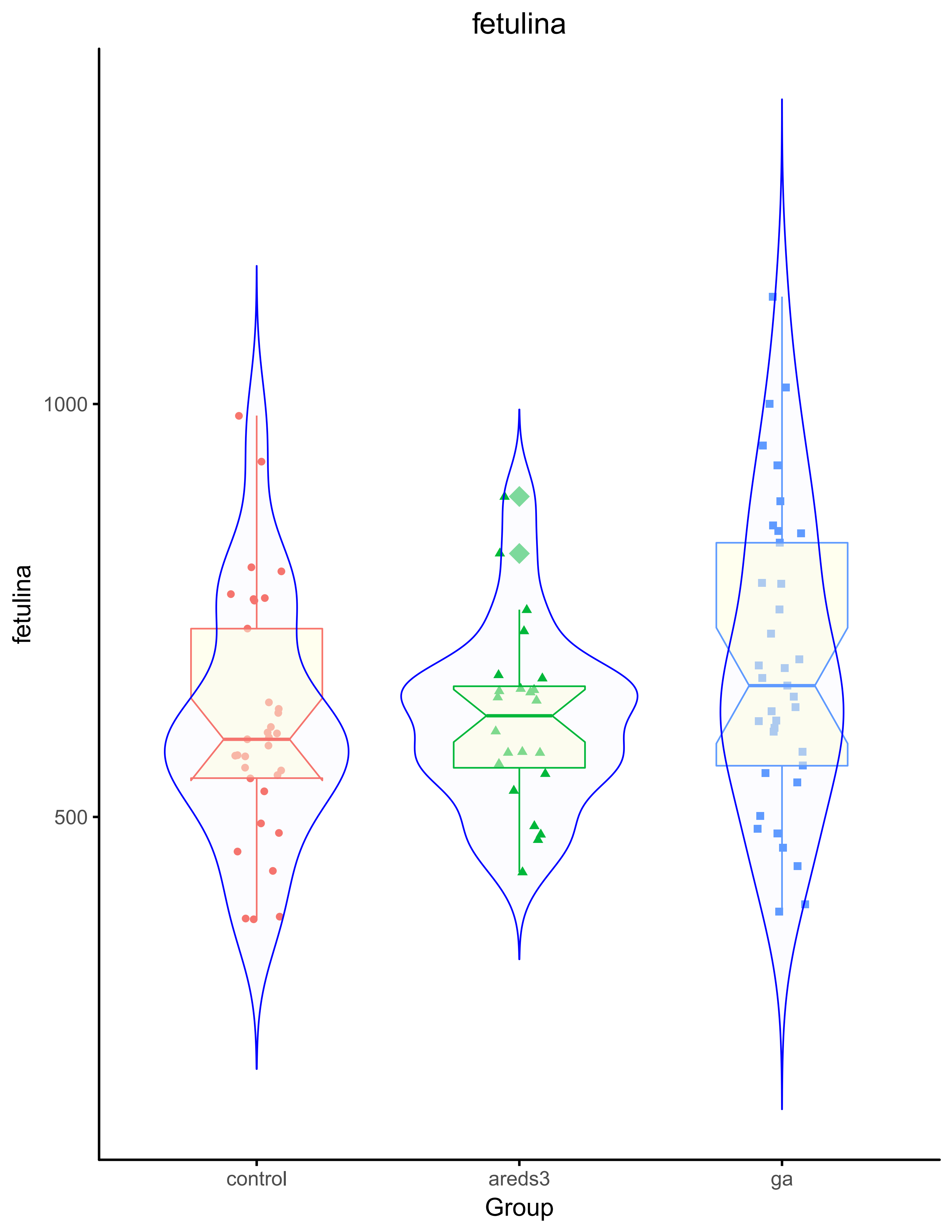

fetulina
fetulina
1000
500
control
areds3
ga
Group

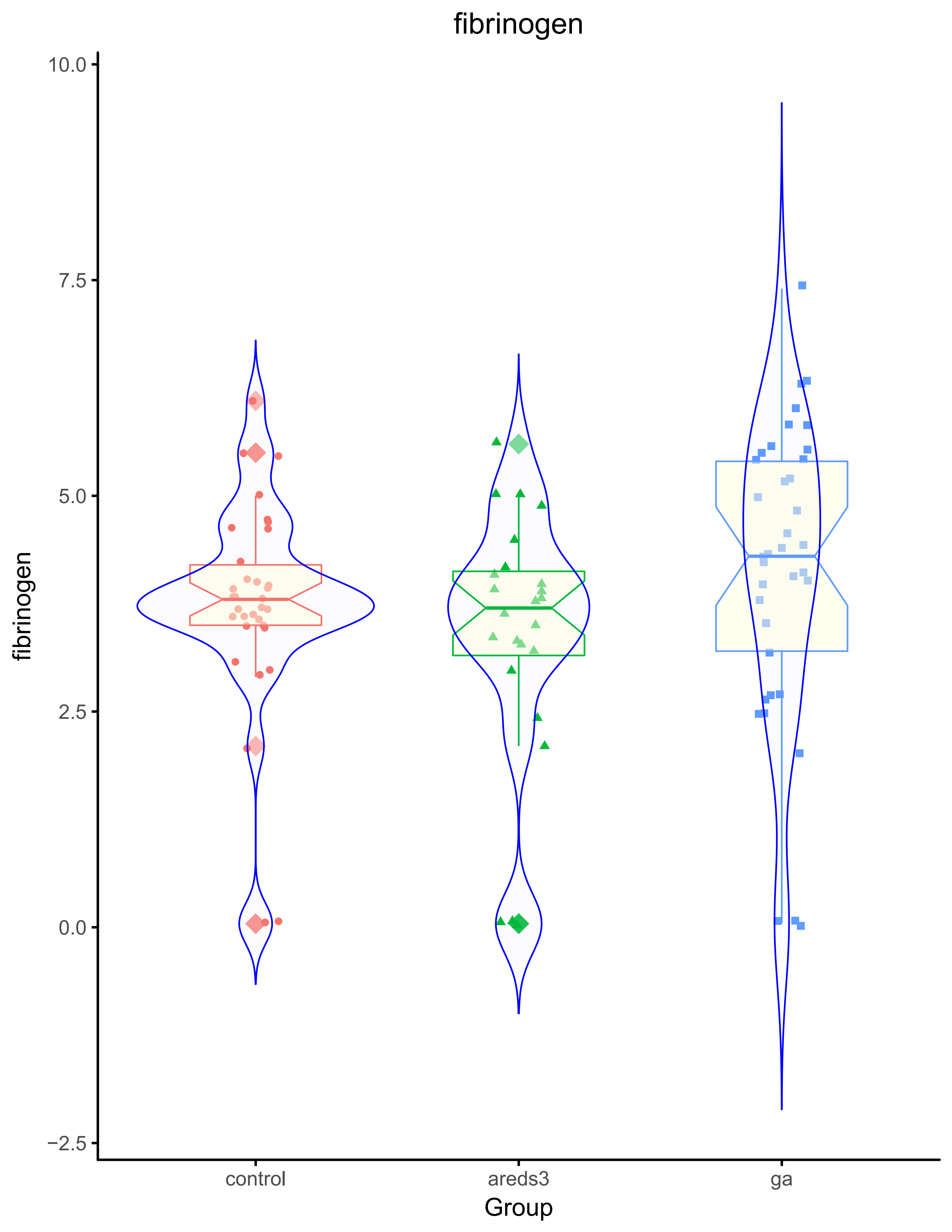

fibrinogen
10.0
7.5
5.0
2.5
0.0
−2.5
fibrinogen
control
areds3
ga
Group

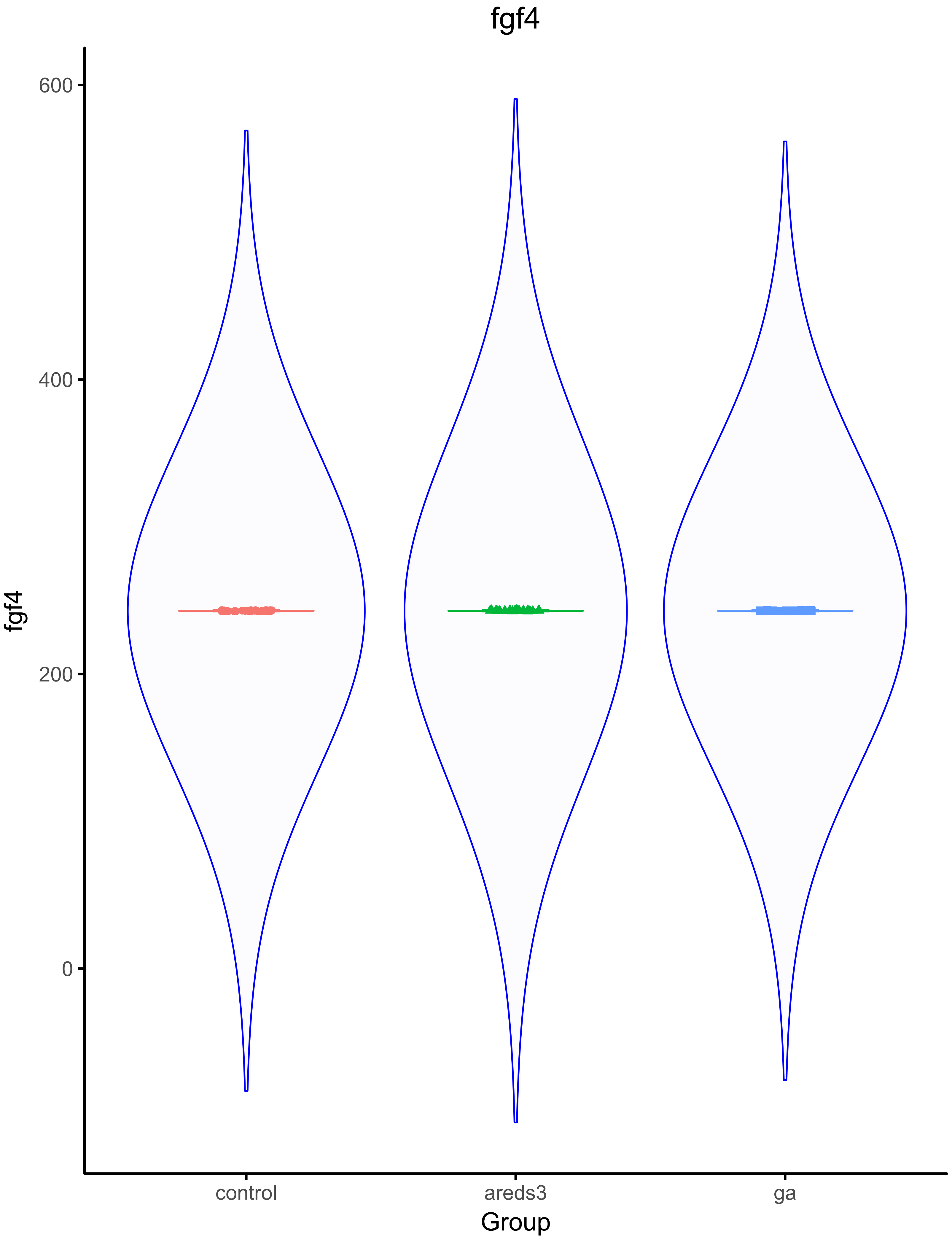
fgf4
600
400
200
0
fgf4
control
areds3
ga
Group

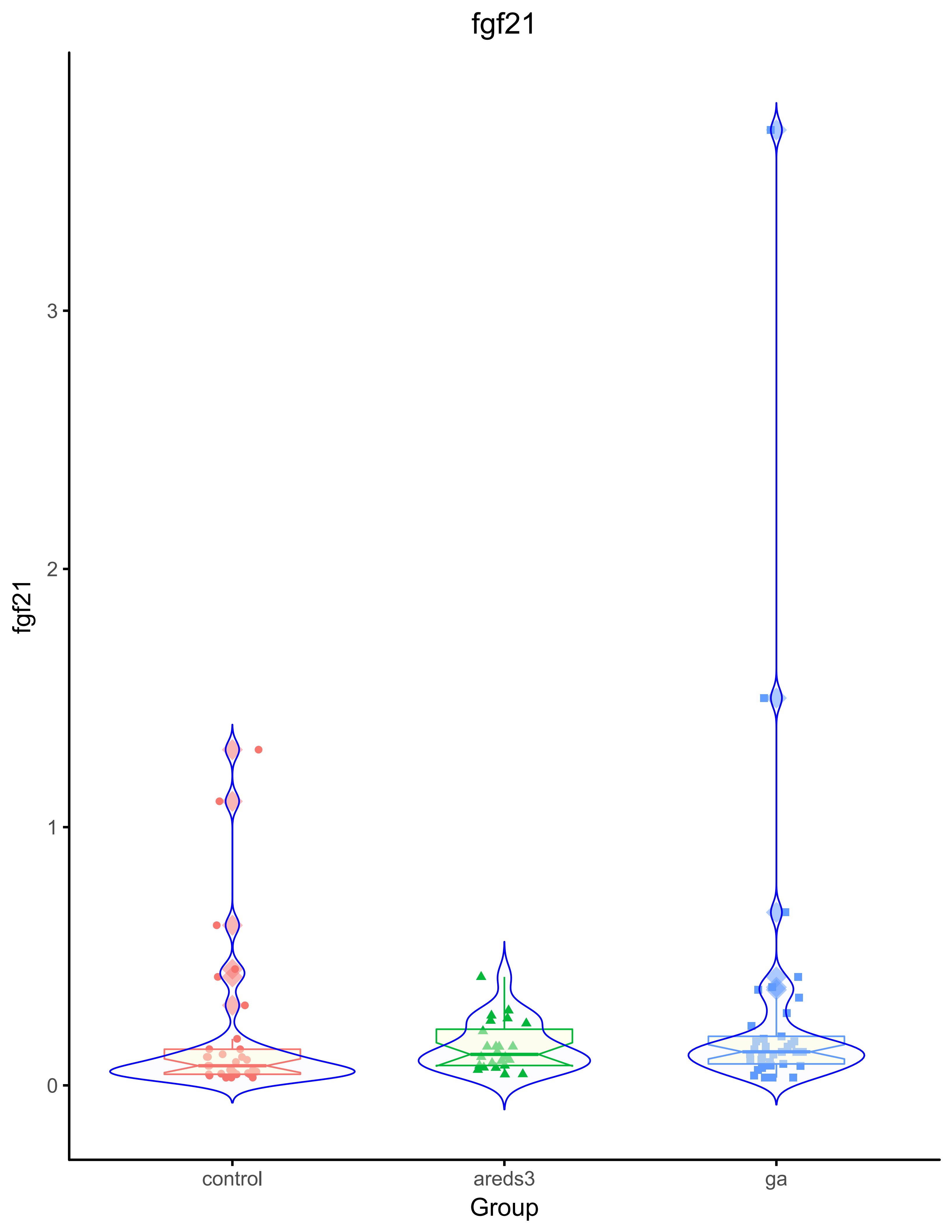

fgf21
fgf21
0
1
2
3
control
areds3
ga
Group

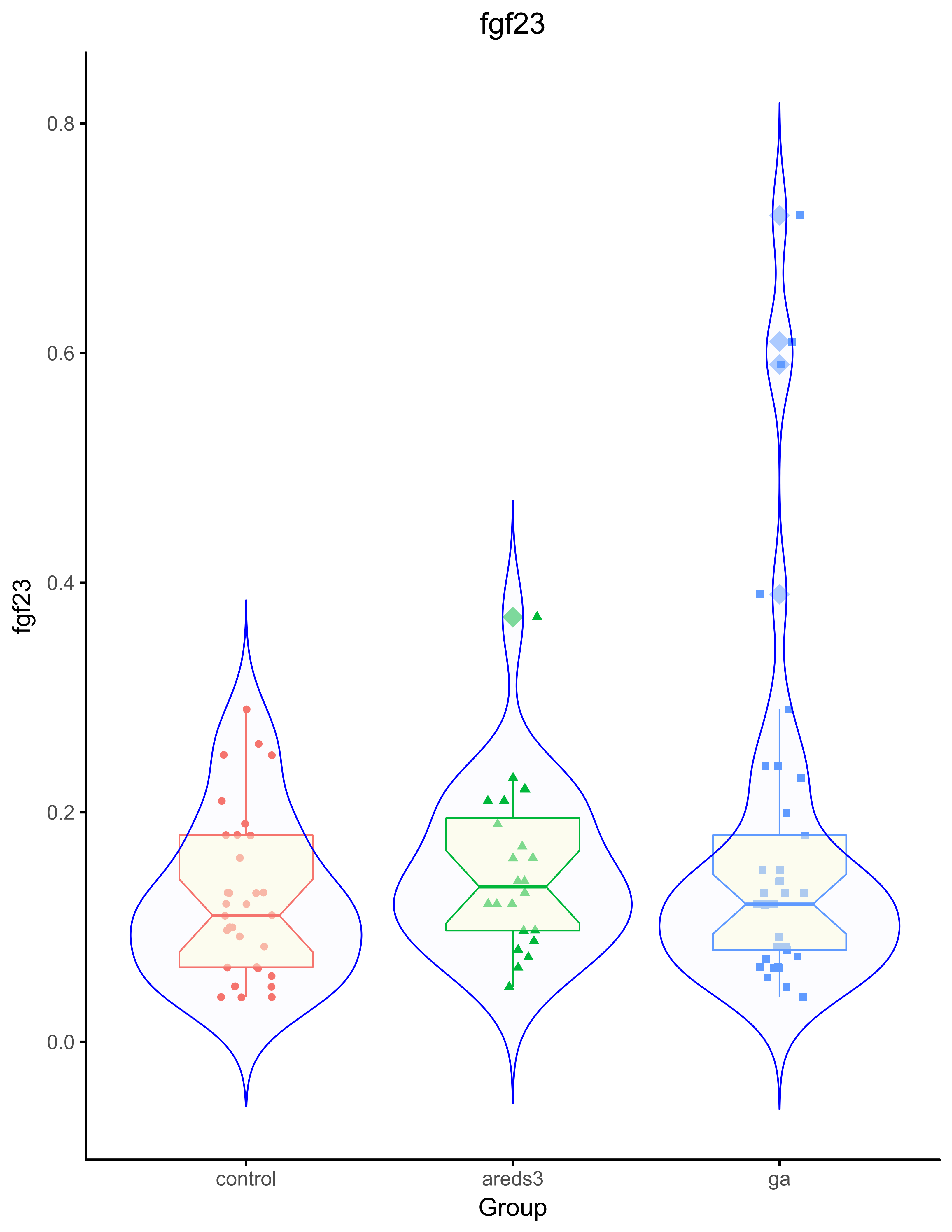

fgf23
0.8
0.6
0.4
0.2
0.0
fgf23
control
areds3
ga
Group

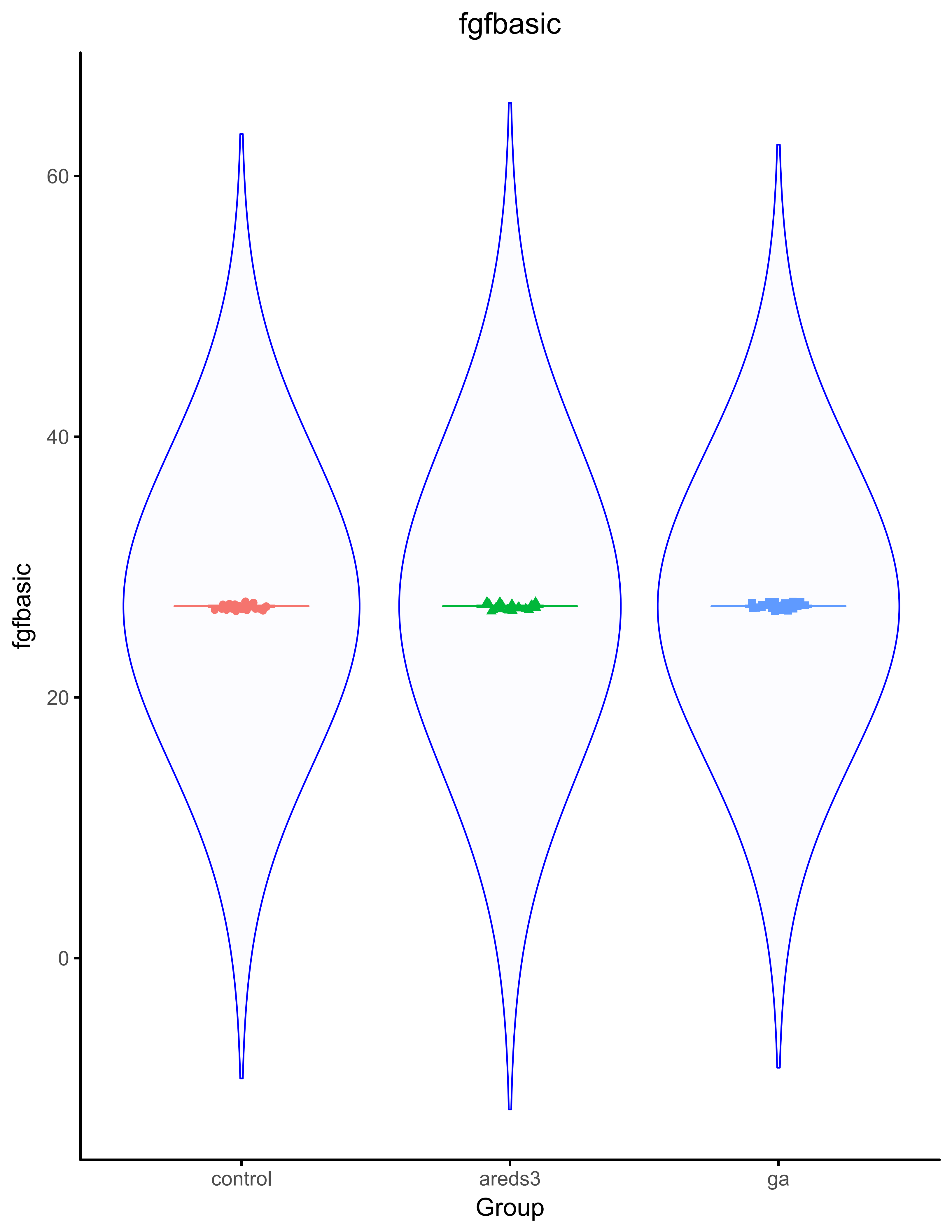

fgfbasic
60
40
20
0
fgfbasic
control
areds3
ga
Group

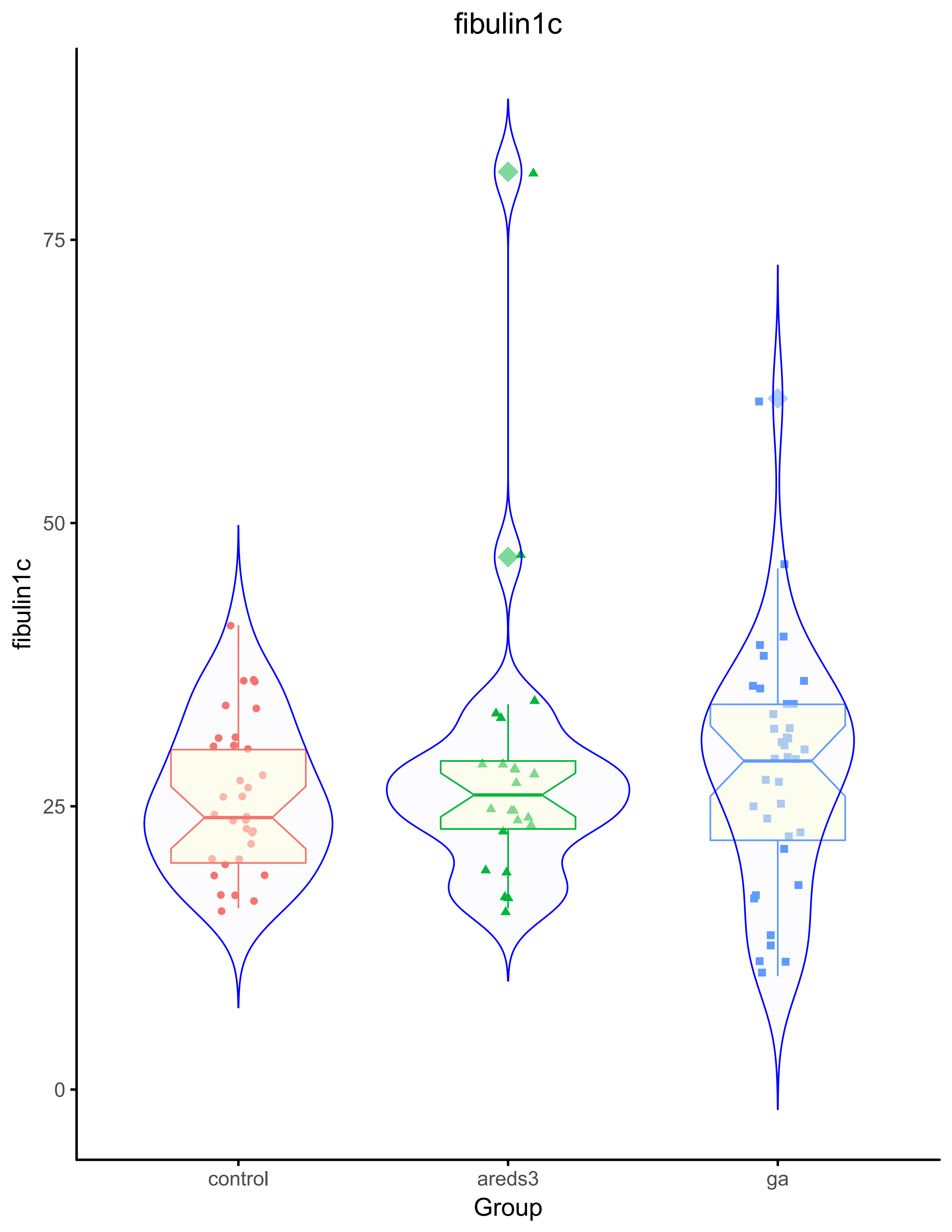

fibulin1c
fibulin1c
75
50
25
0
control
areds3
ga
Group

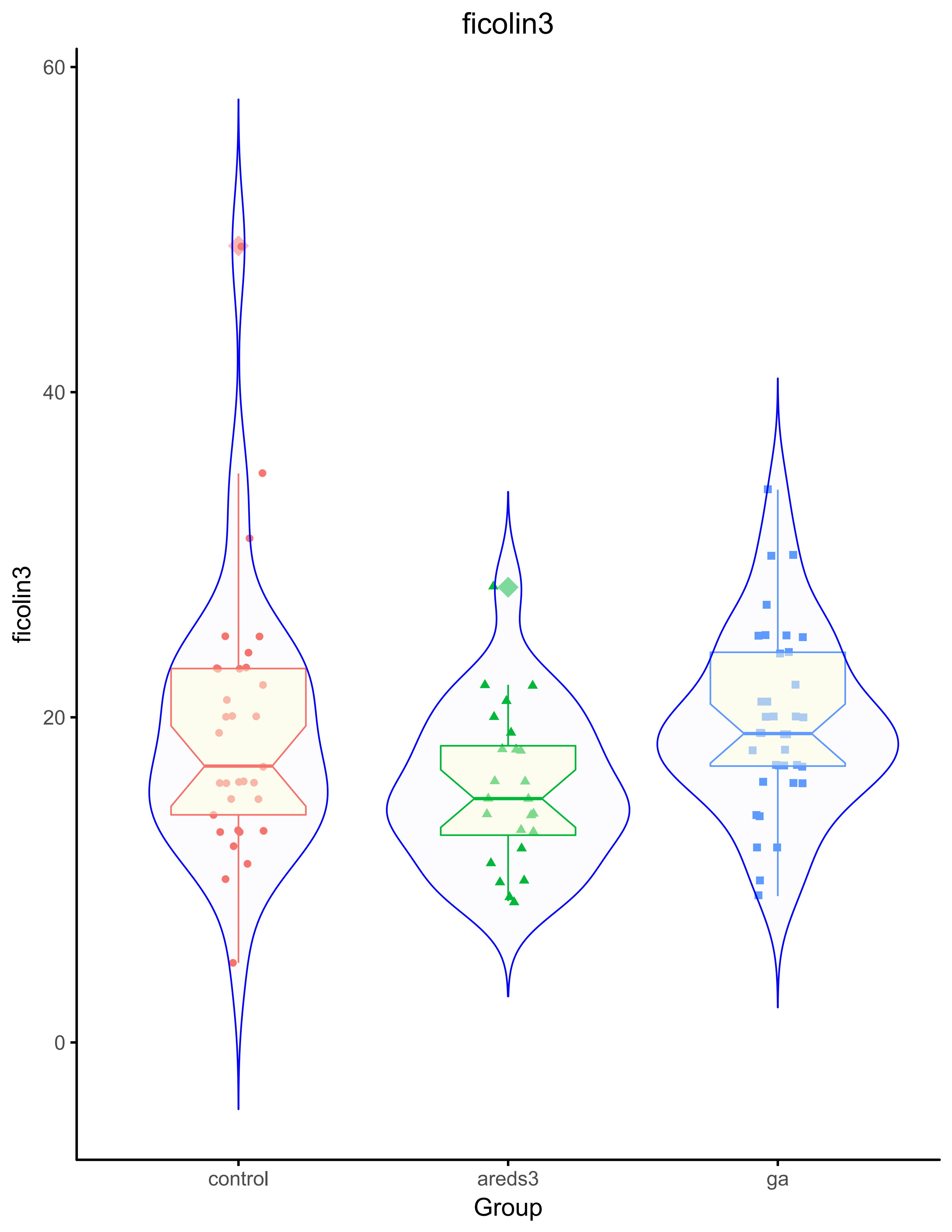
ficolin3
60
40
20
0
ficolin3
control
areds3
ga
Group

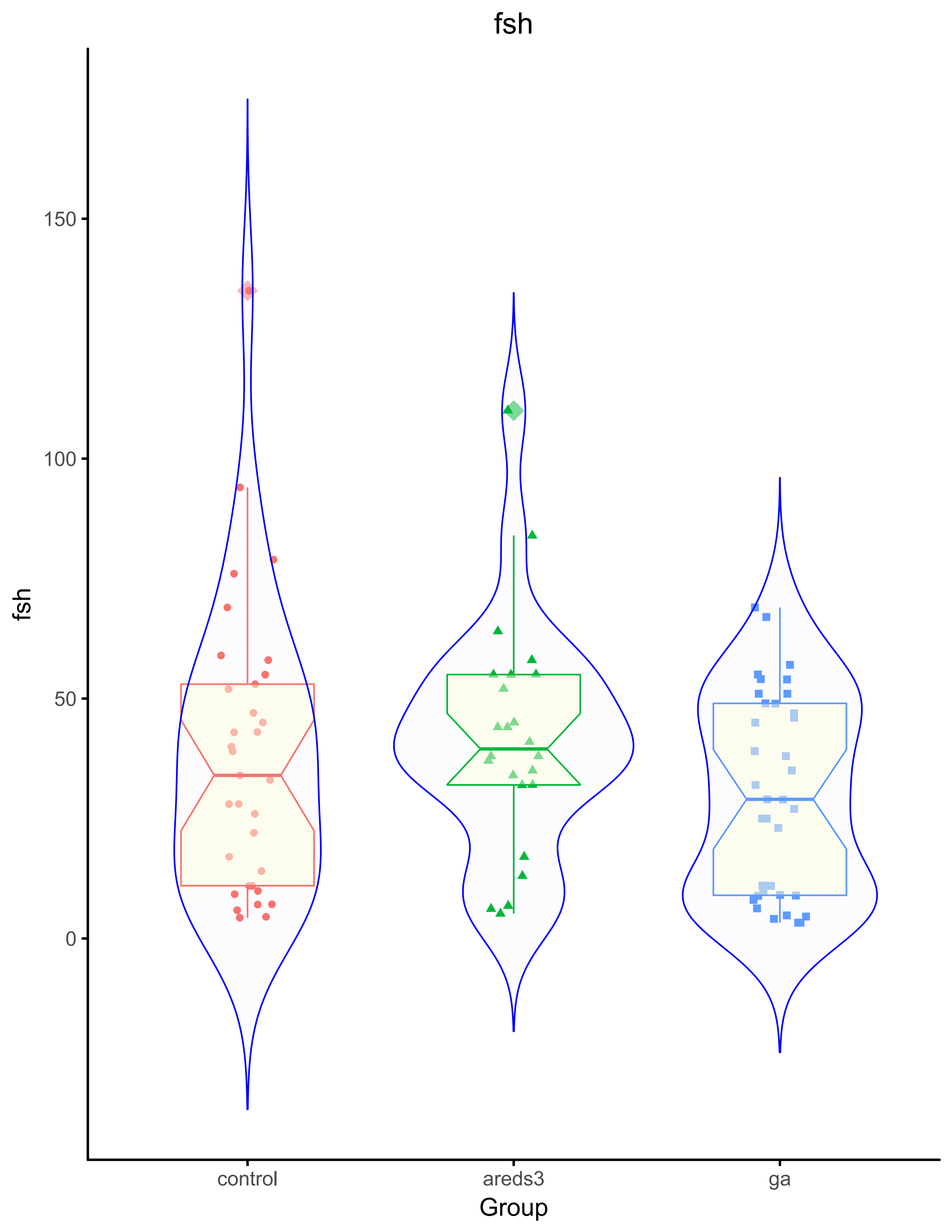

fsh
150
100
50
0
fsh
control
areds3
ga
Group

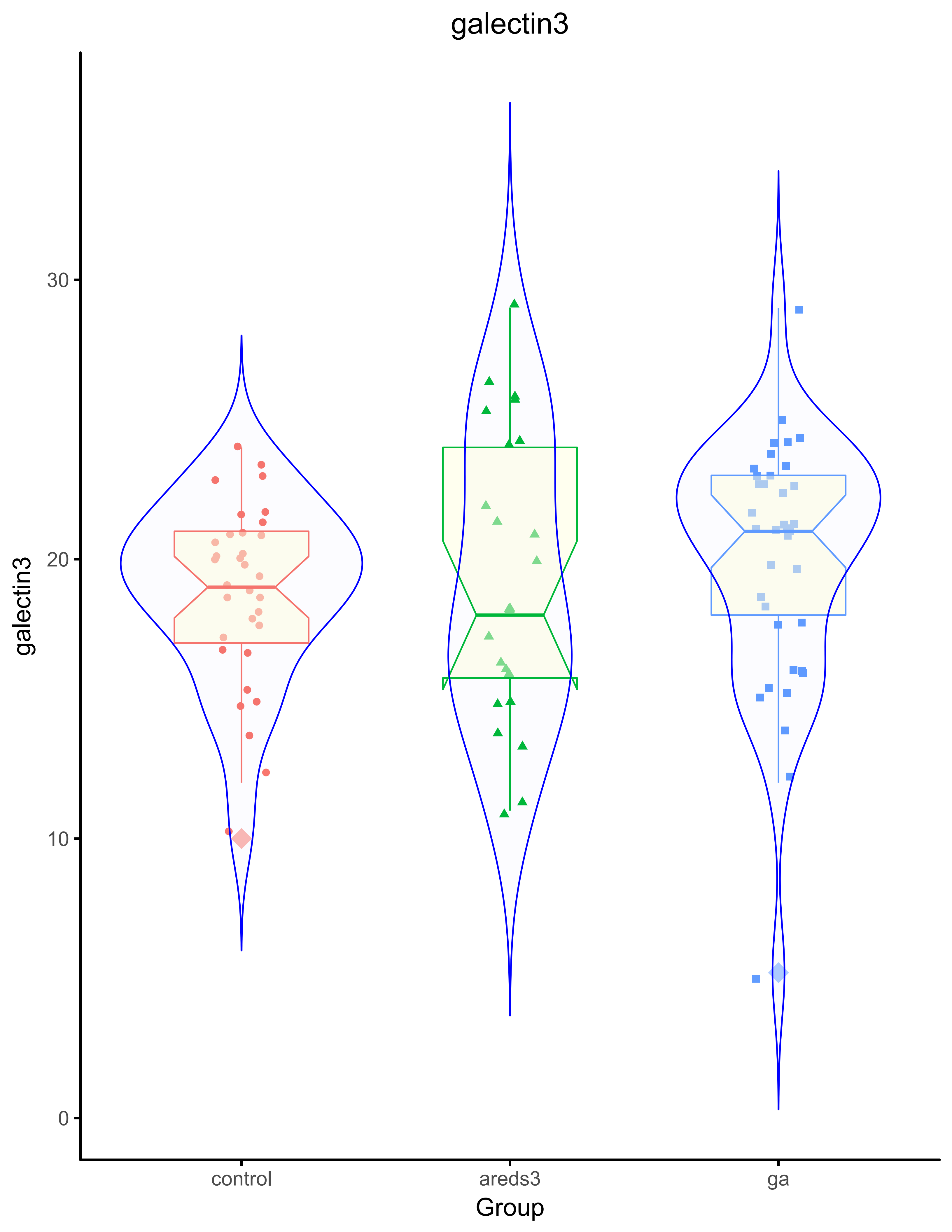
galectin3
30
20
10
0
galectin3
control
areds3
ga
Group

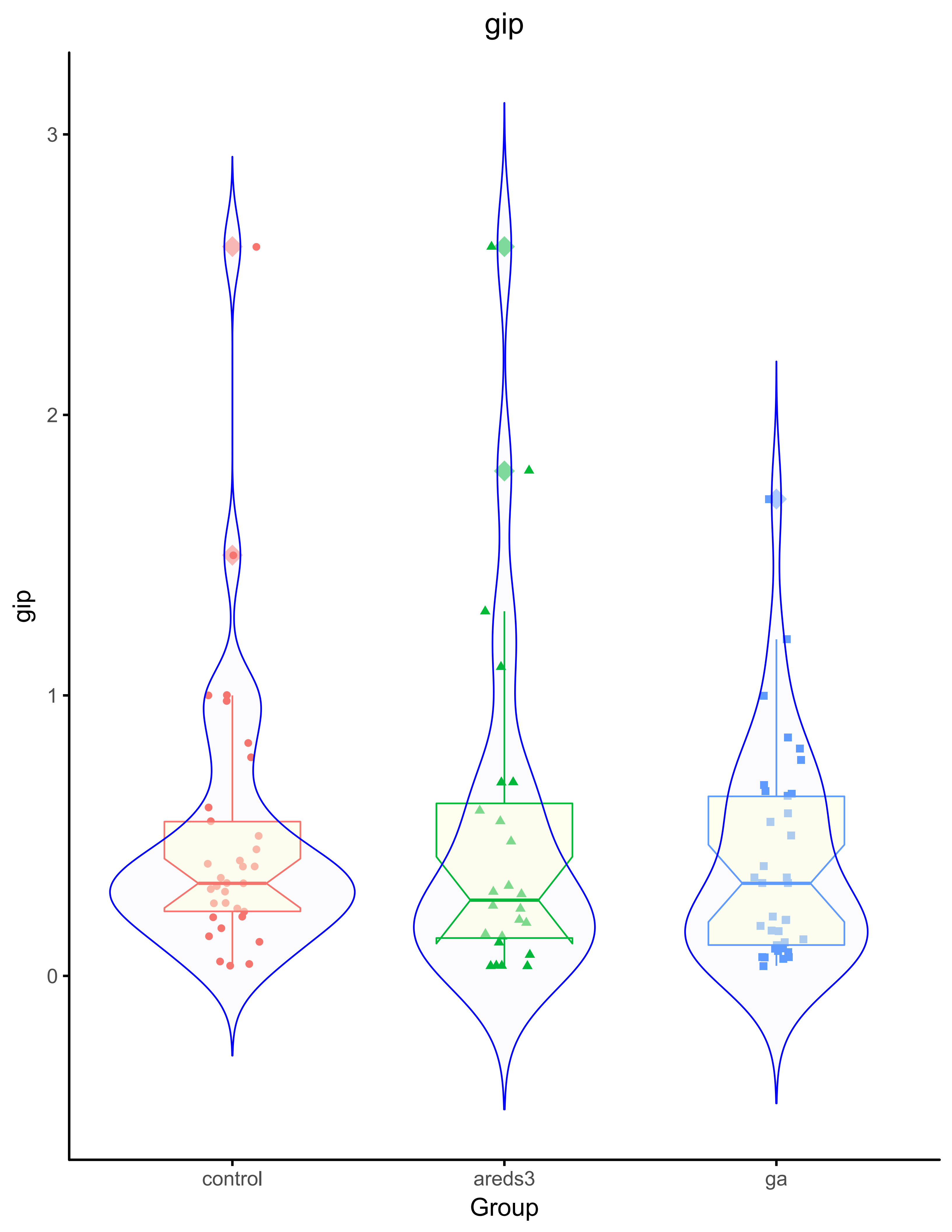
gip
3
2
1
0
gip
control
areds3
ga
Group

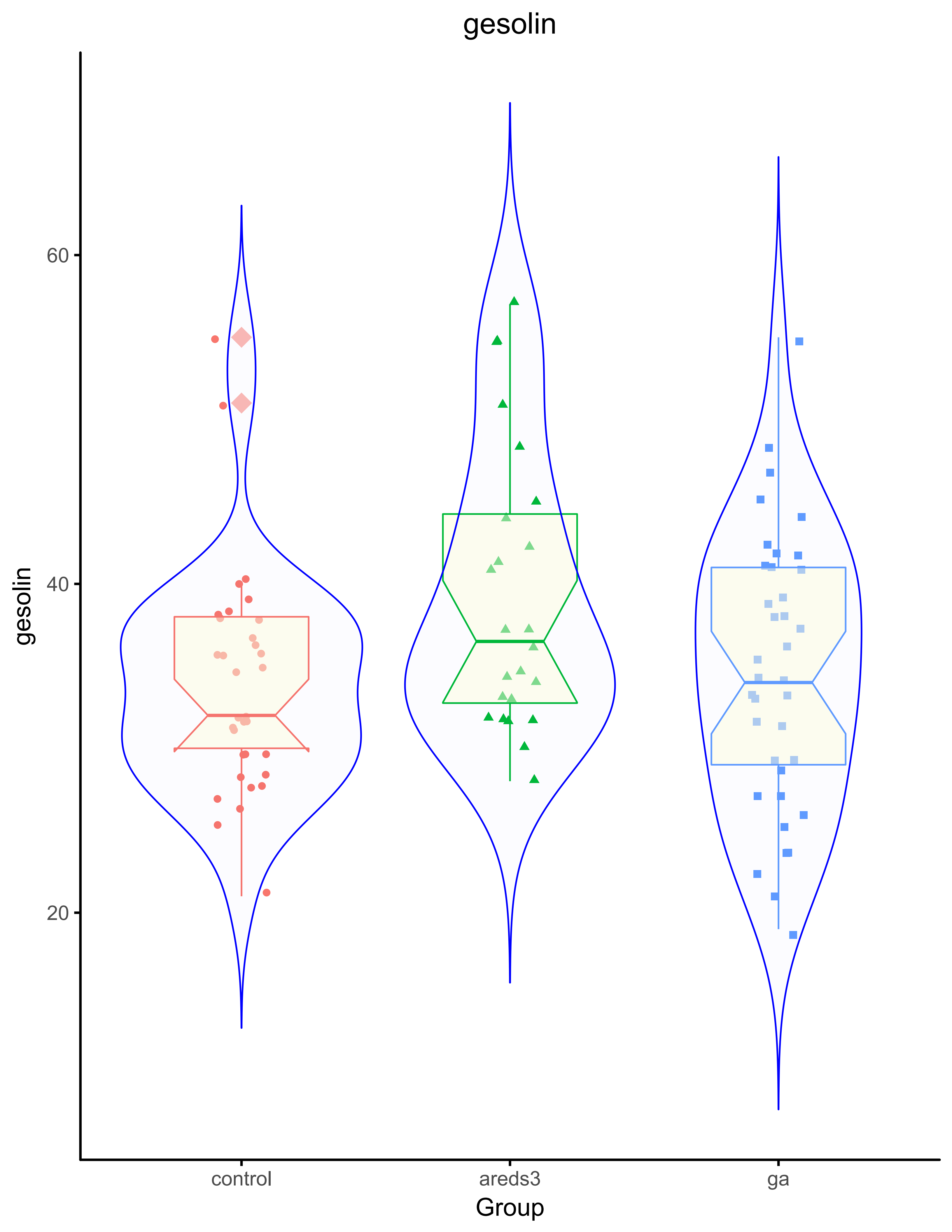
gesolin
gesolin
60
40
20
control
areds3
ga
Group

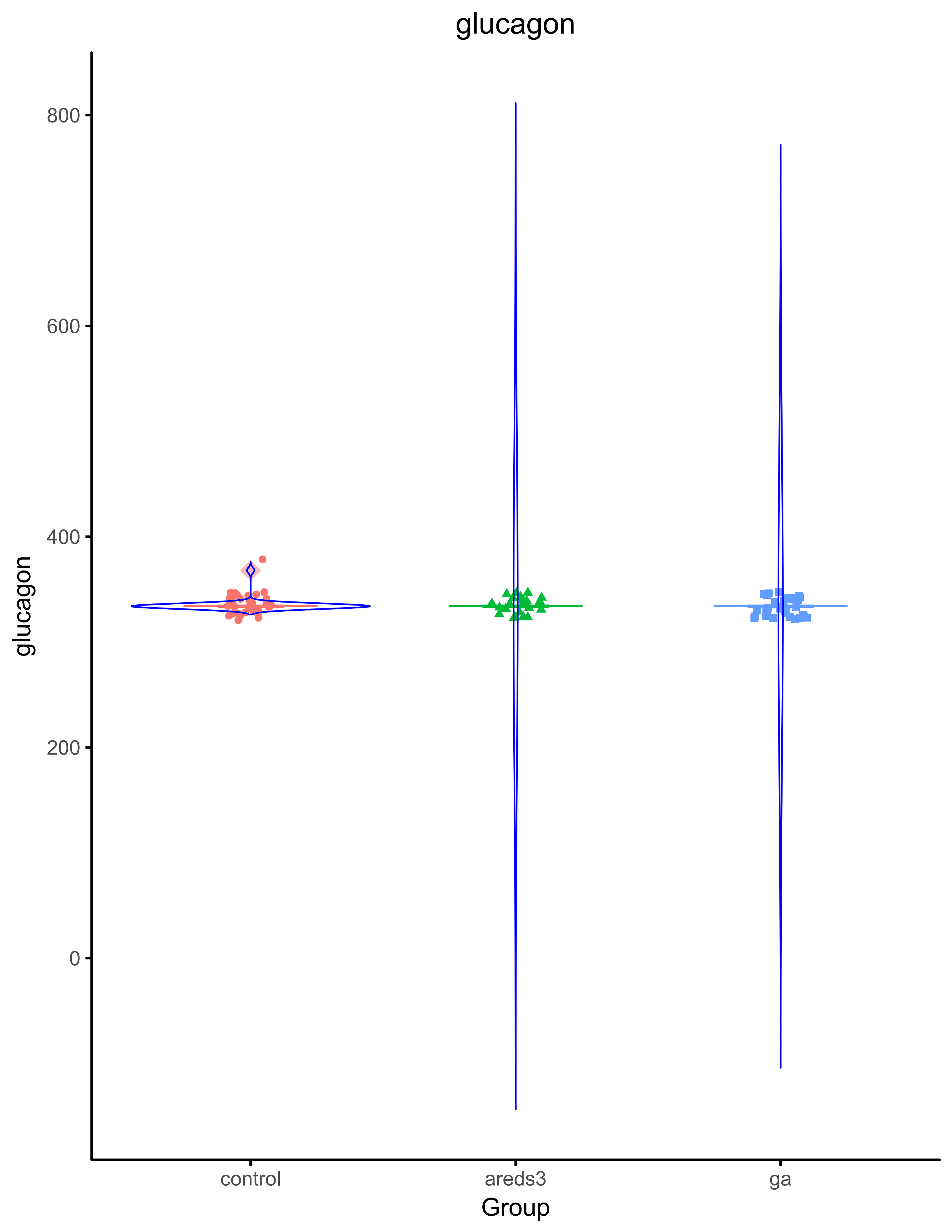
glucagon
800
600
400
200
0
glucagon
control
areds3
ga
Group

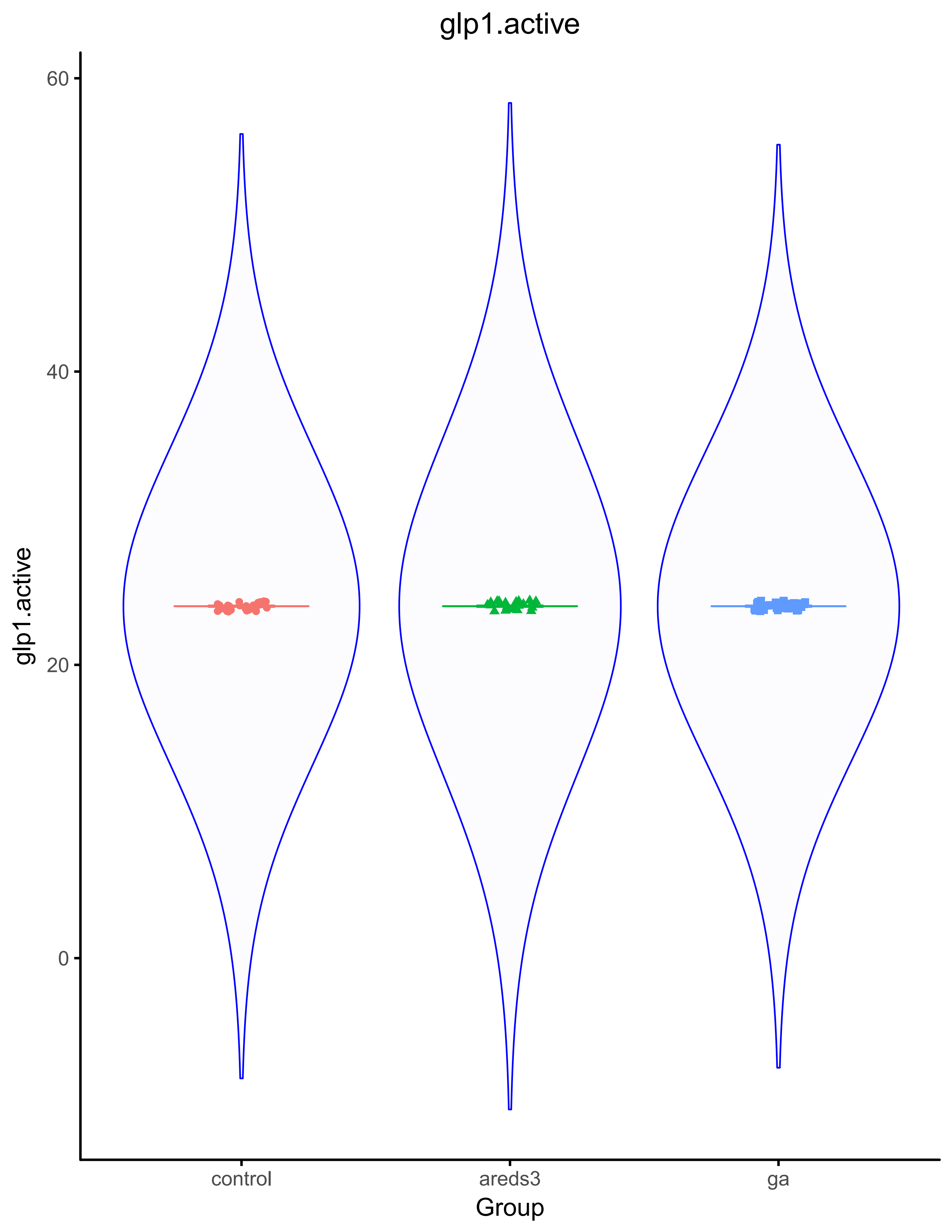
glp1.active
60
40
20
0
glp1.active
control
areds3
ga
Group

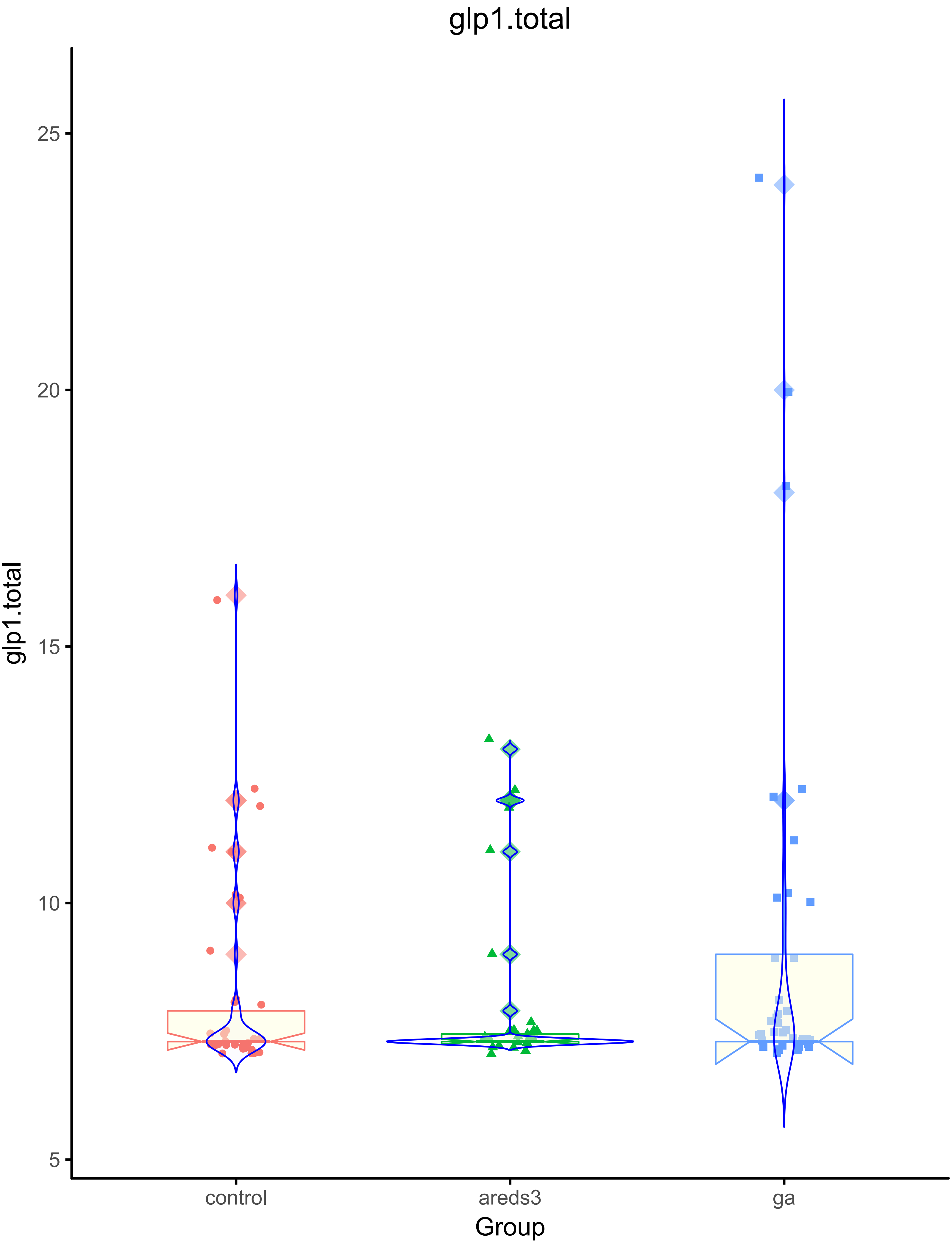

glp1.total
25
20
15
10
5
glp1.total
control
areds3
ga
Group

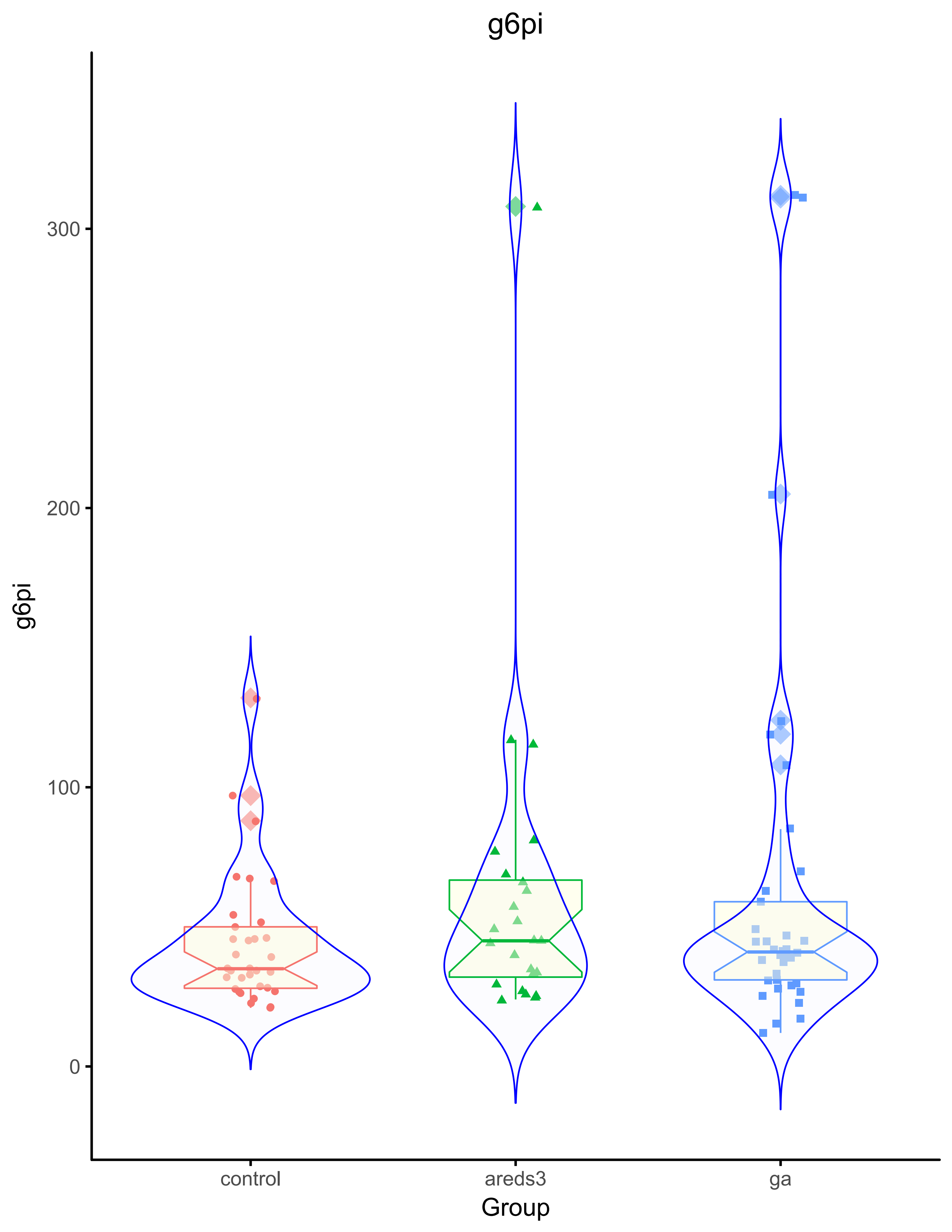

g6pi
300
200
100
0
g6pi
control
areds3
ga
Group

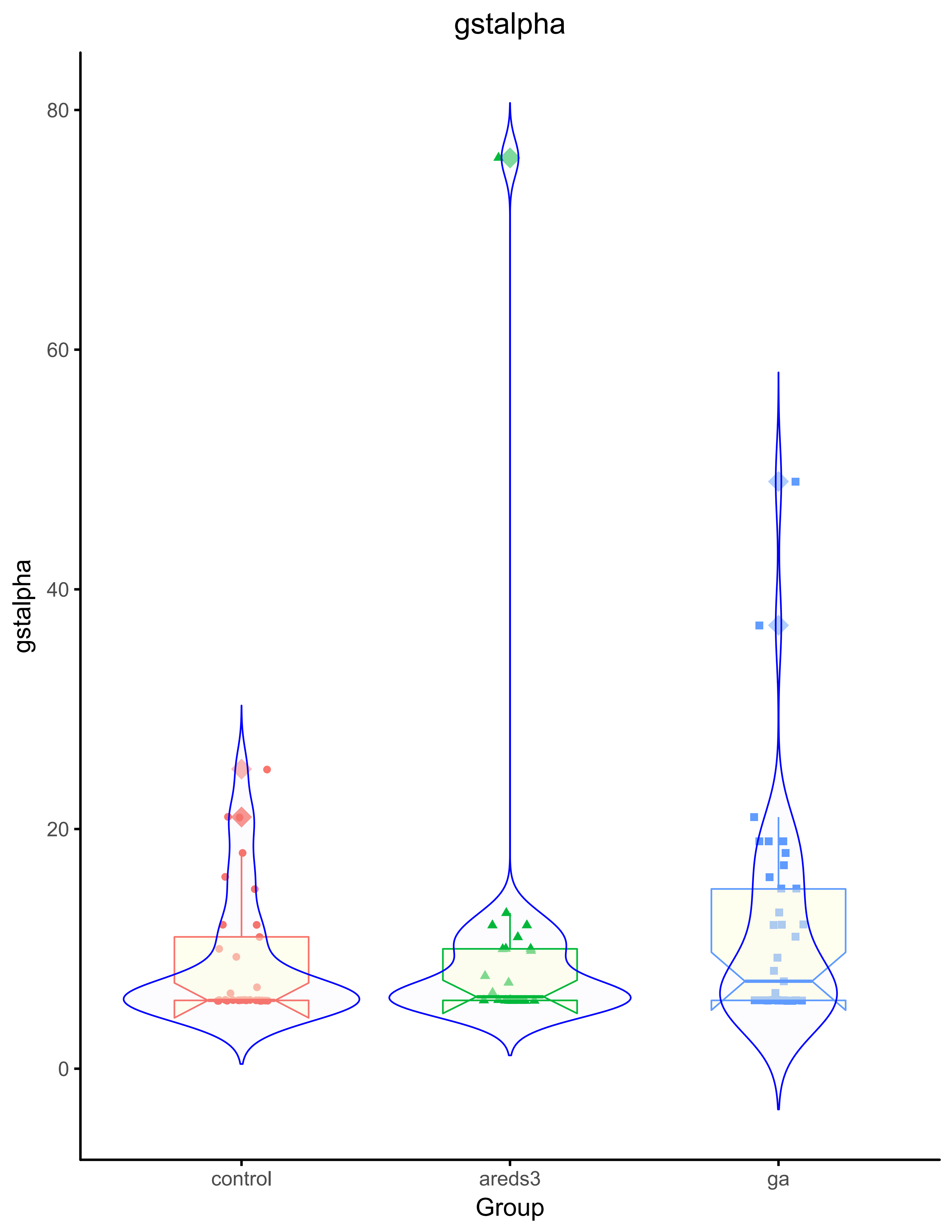

gstalpha
80
60
40
20
0
gstalpha
control
areds3
ga
Group

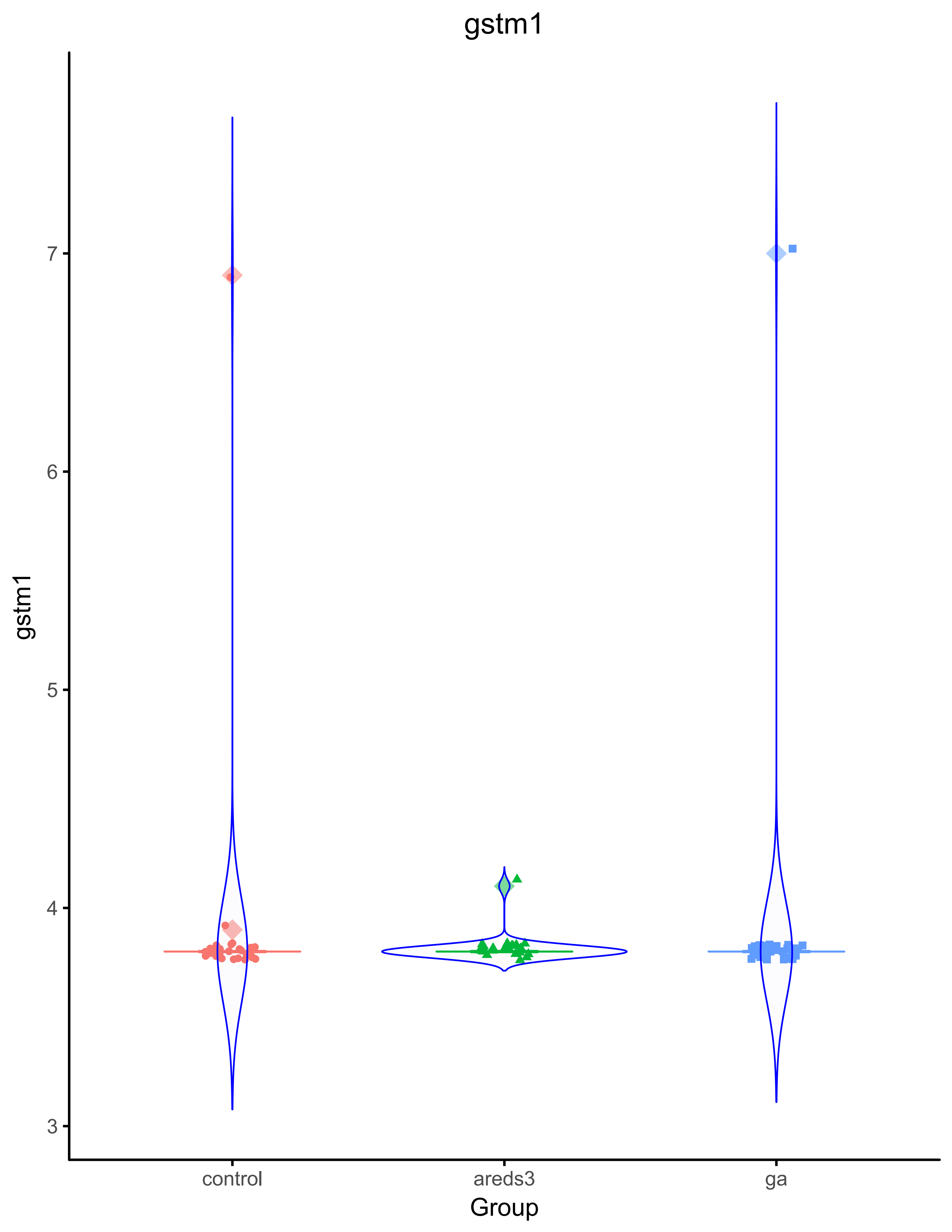

gstm1
gstm1
7
6
5
4
3
control
areds3
ga
Group

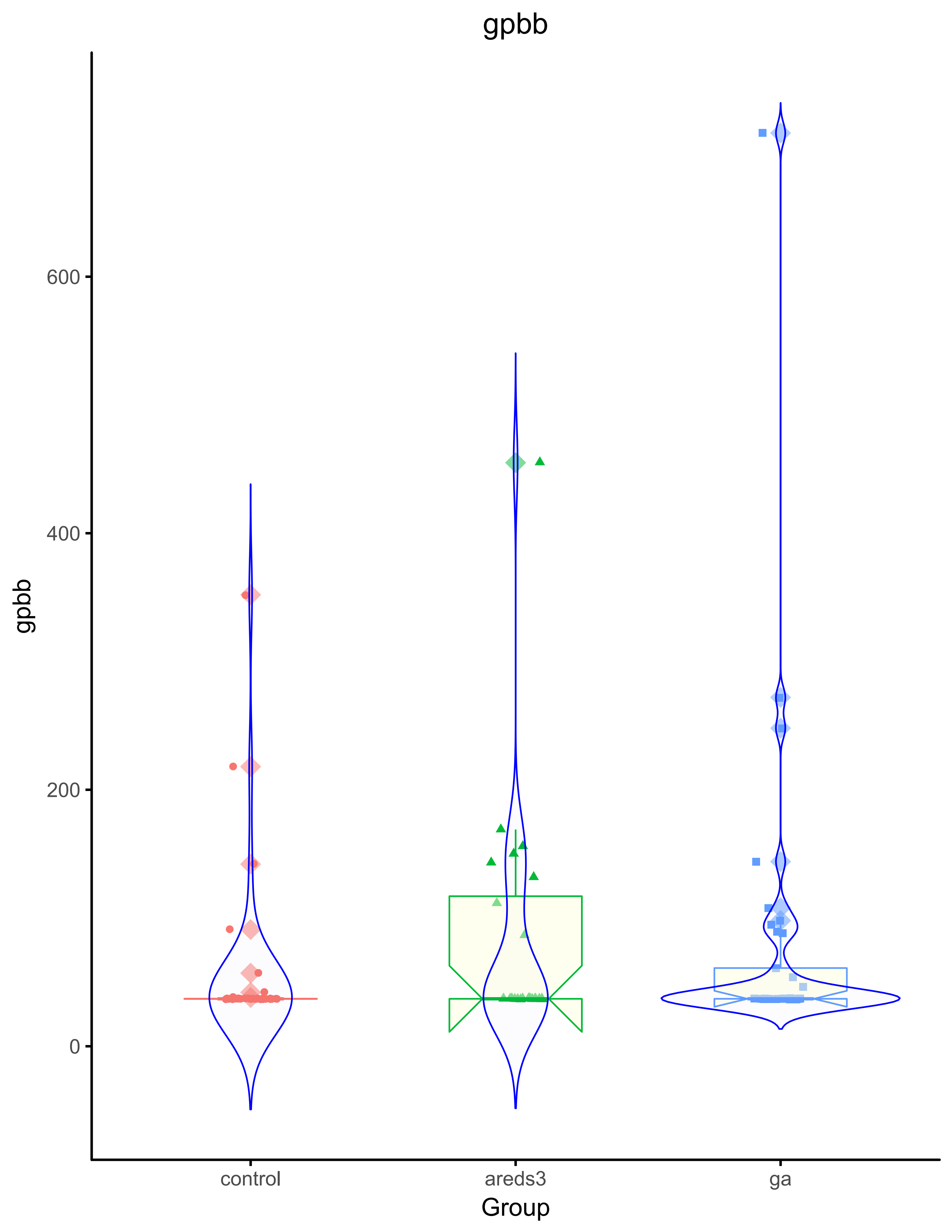
gpbb
gpbb
600
400
200
0
control
areds3
ga
Group

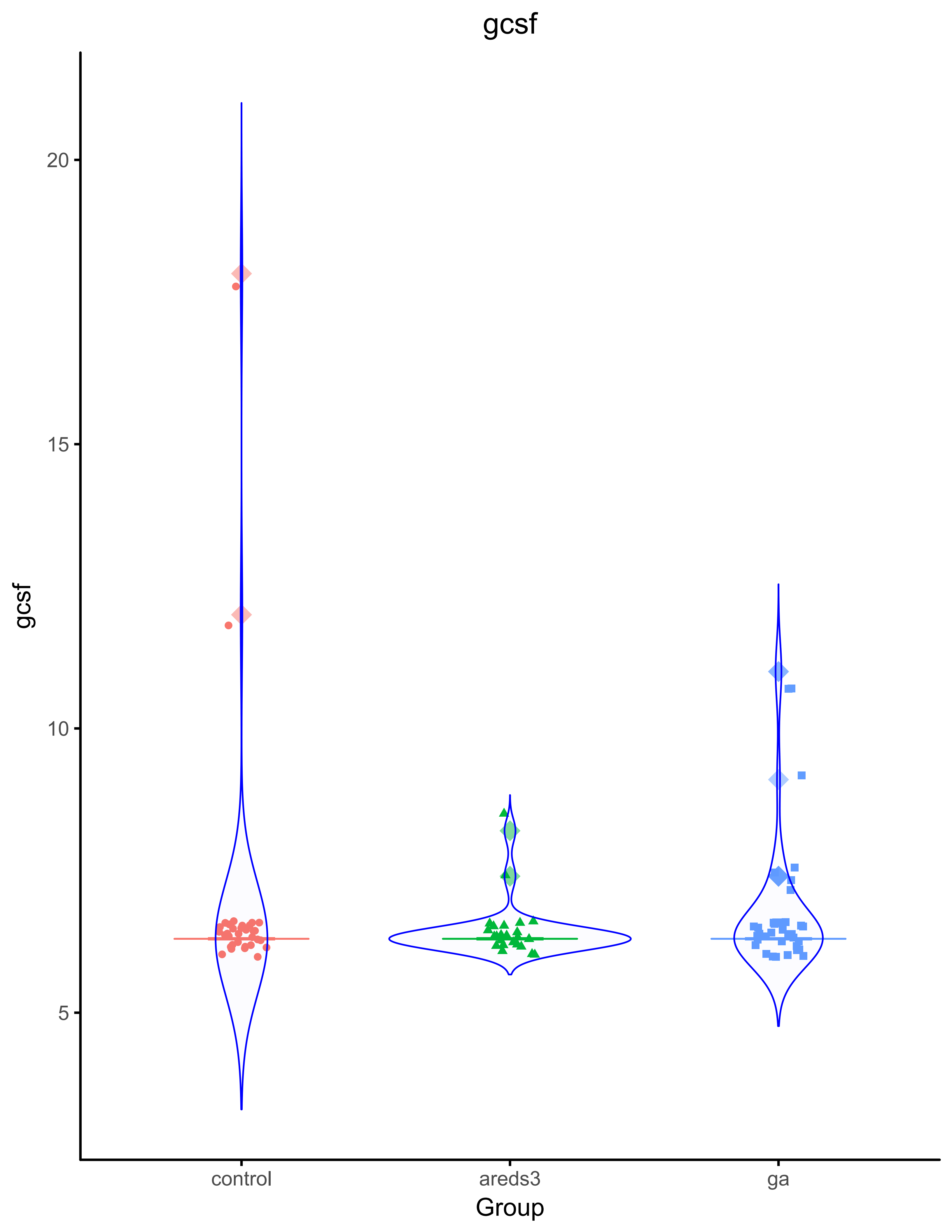

gcsf
20
15
10
5
gcsf
control
areds3
ga
Group

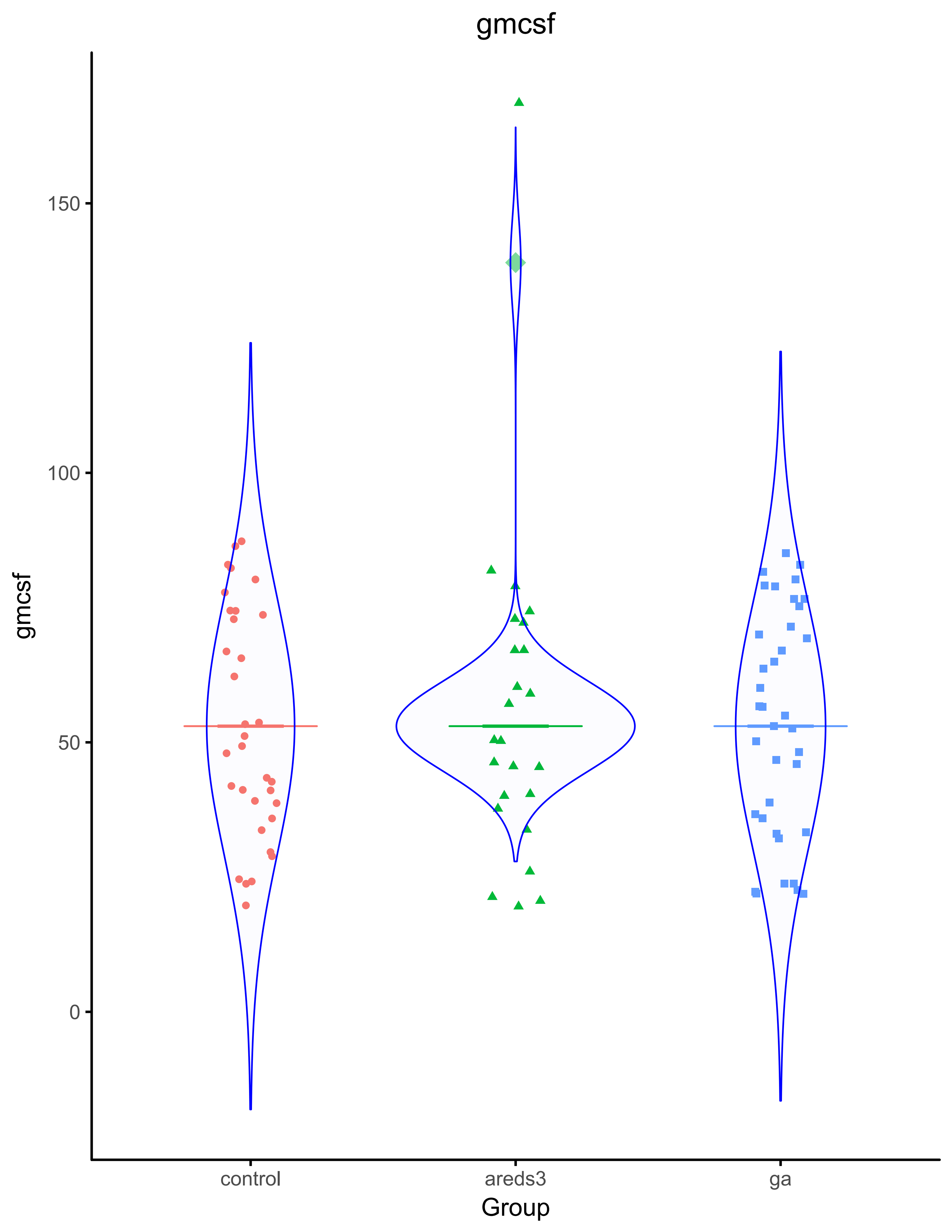

gmcsf
150
100
50
0
gmcsf
control
areds3
ga
Group

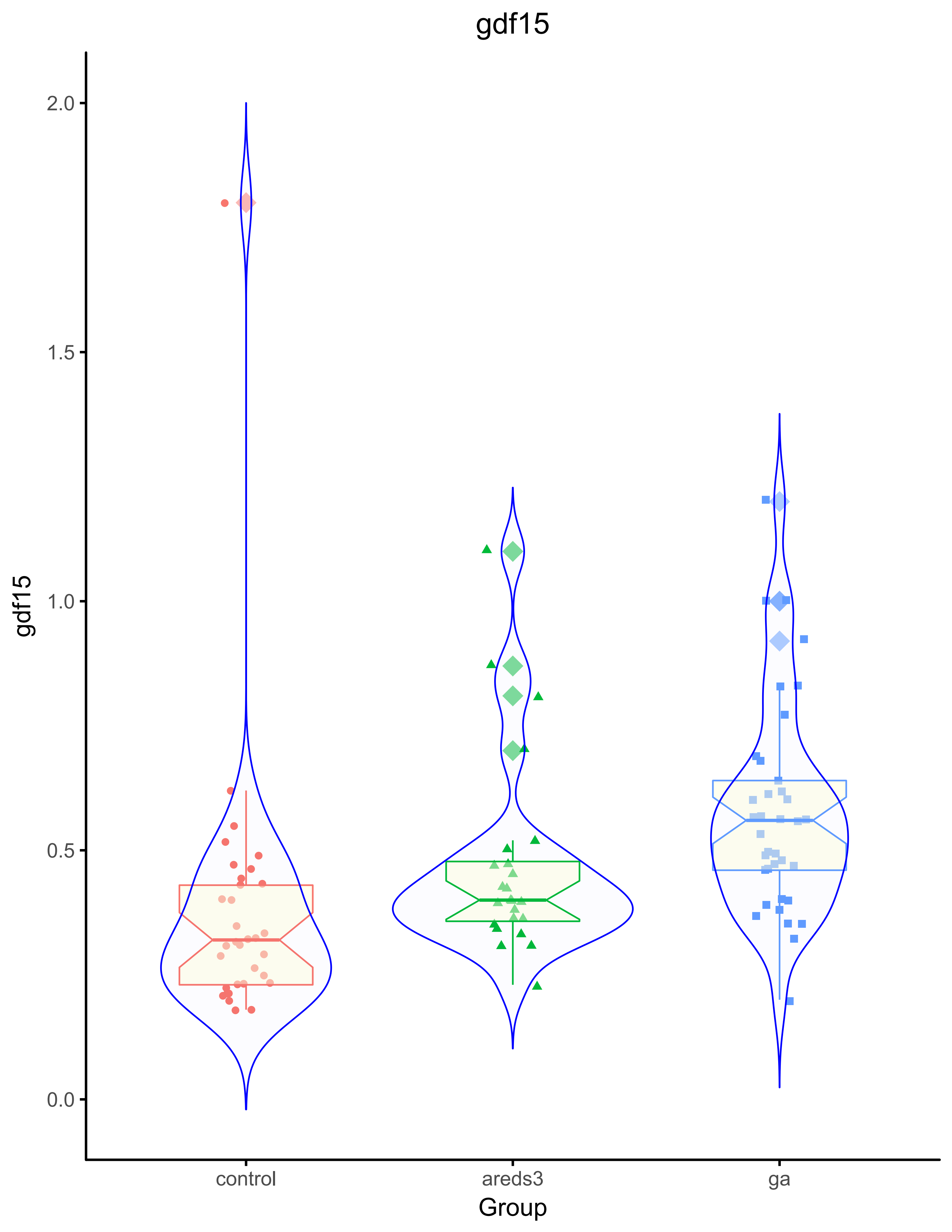
gdf15
2.0
1.5
1.0
0.5
0.0
gdf15
control
areds3
ga
Group

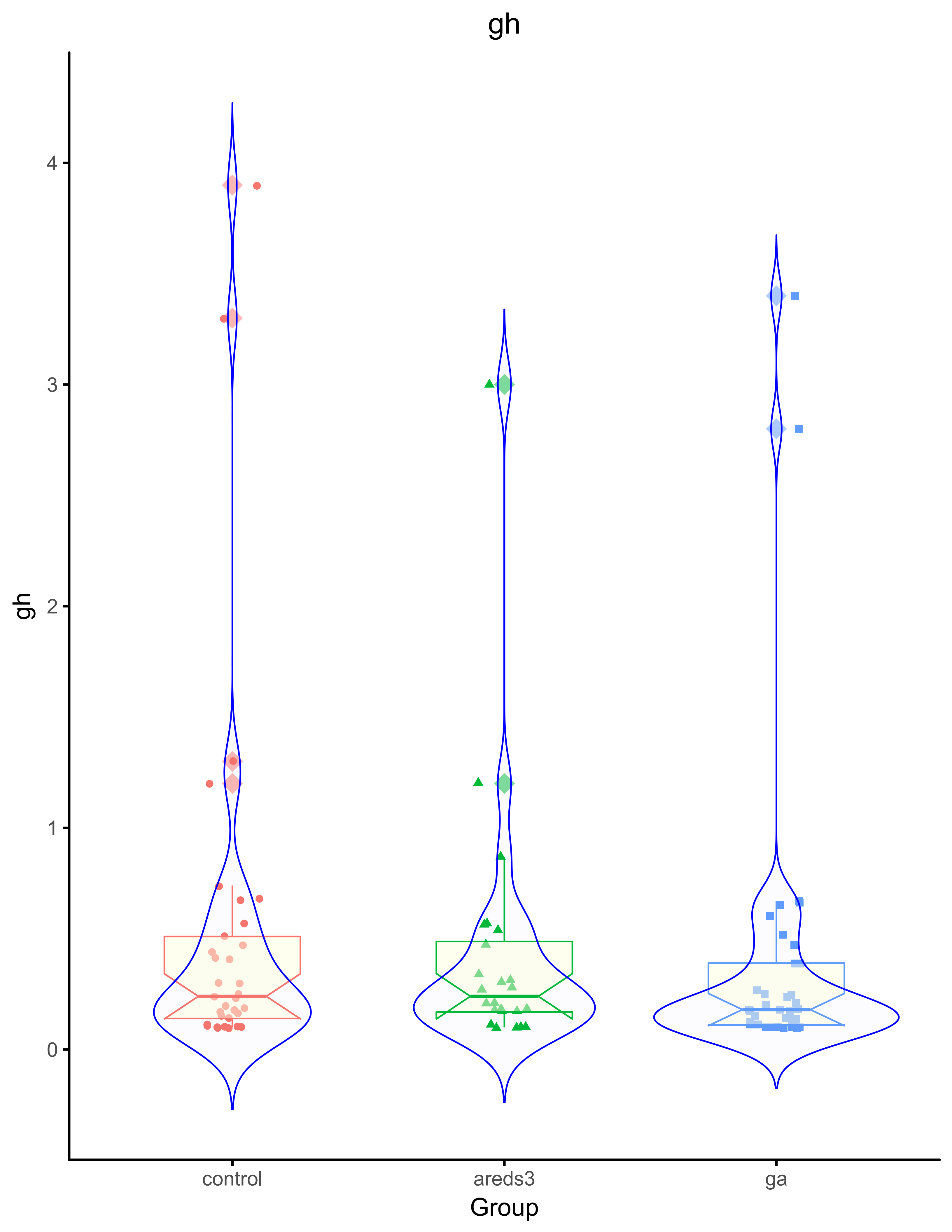

gh
4
3
2
1
0
gh
control
areds3
ga
Group

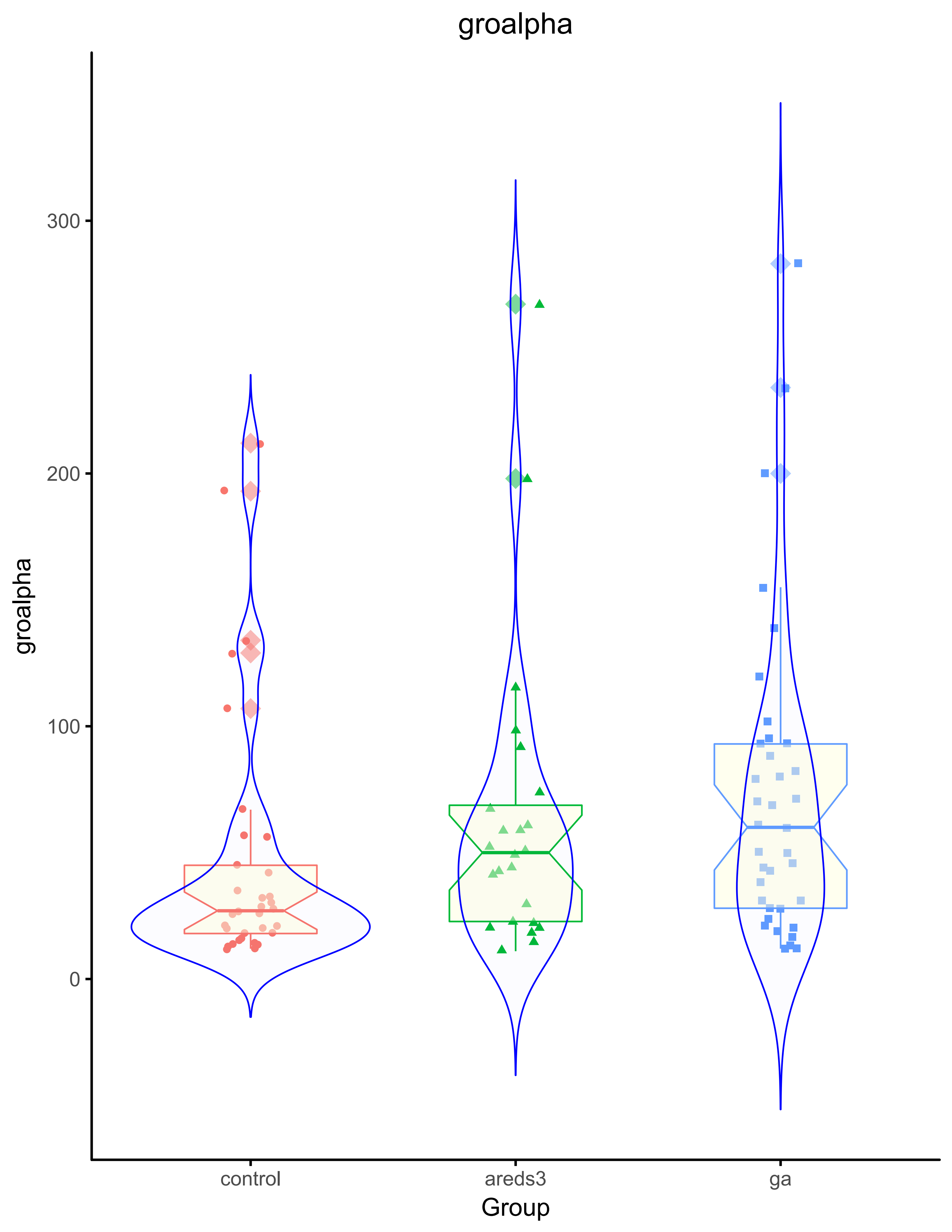
groalpha
300
200
100
0
groalpha
control
areds3
ga
Group

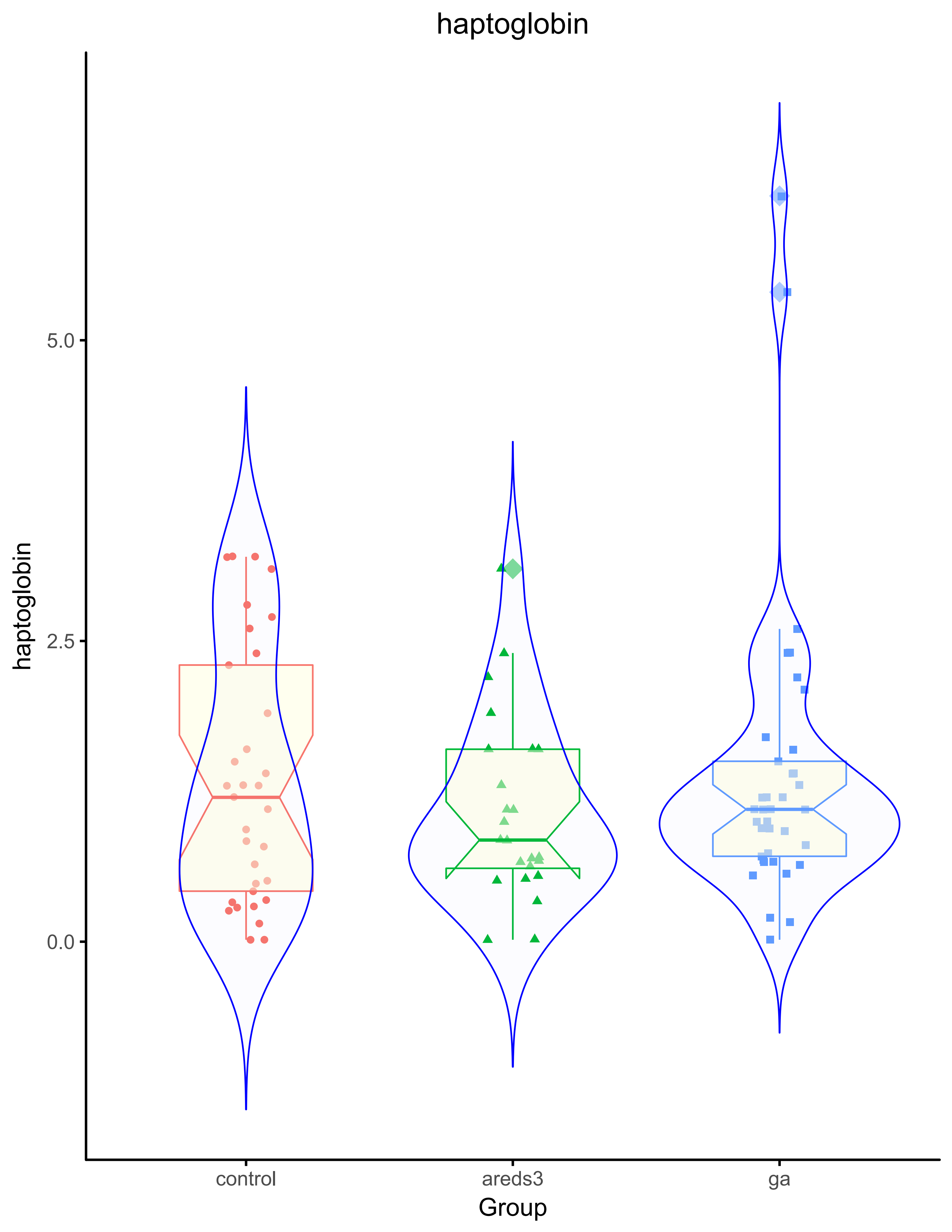
haptoglobin
haptoglobin
5.0
2.5
0.0
control
areds3
ga
Group

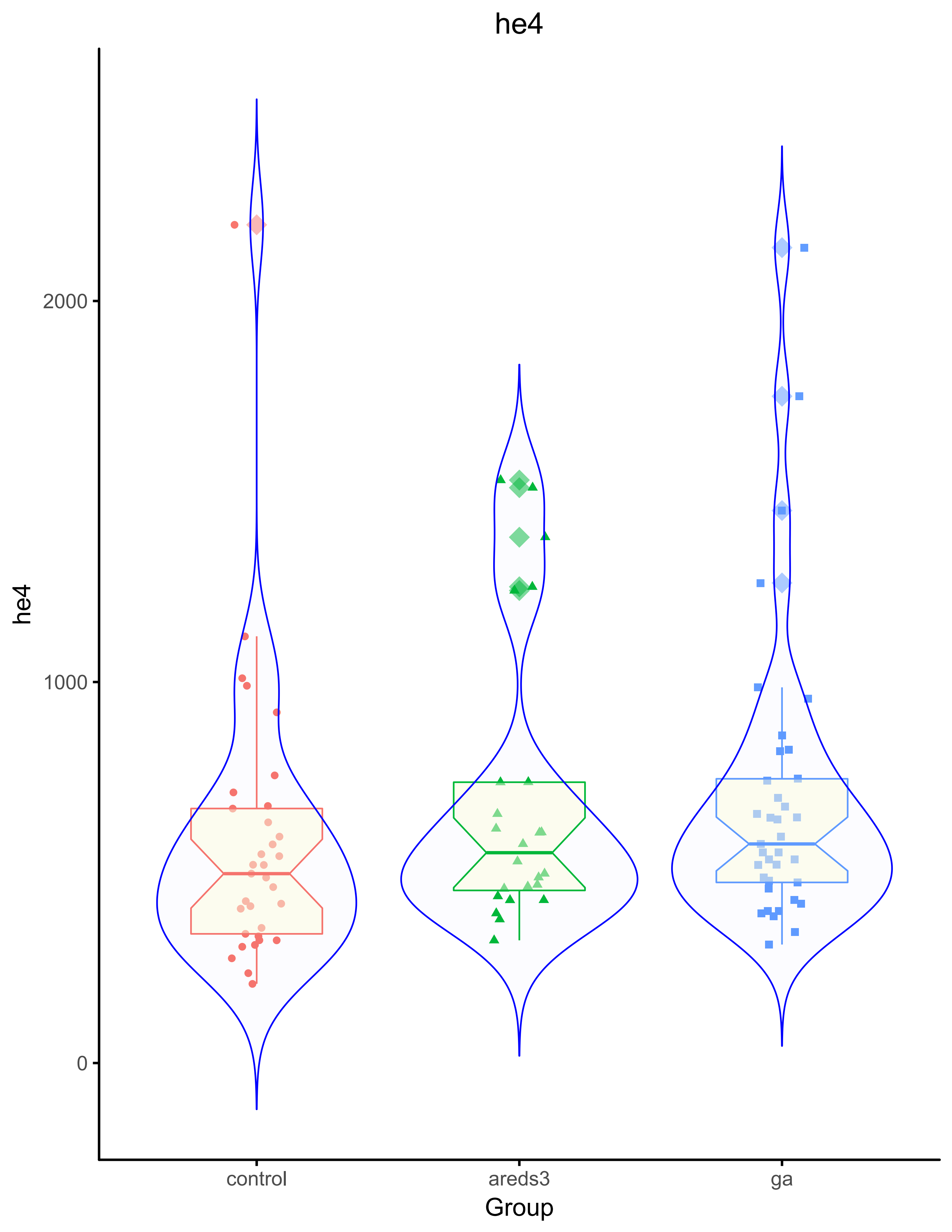
he4
2000
1000
0
he4
control
areds3
ga
Group

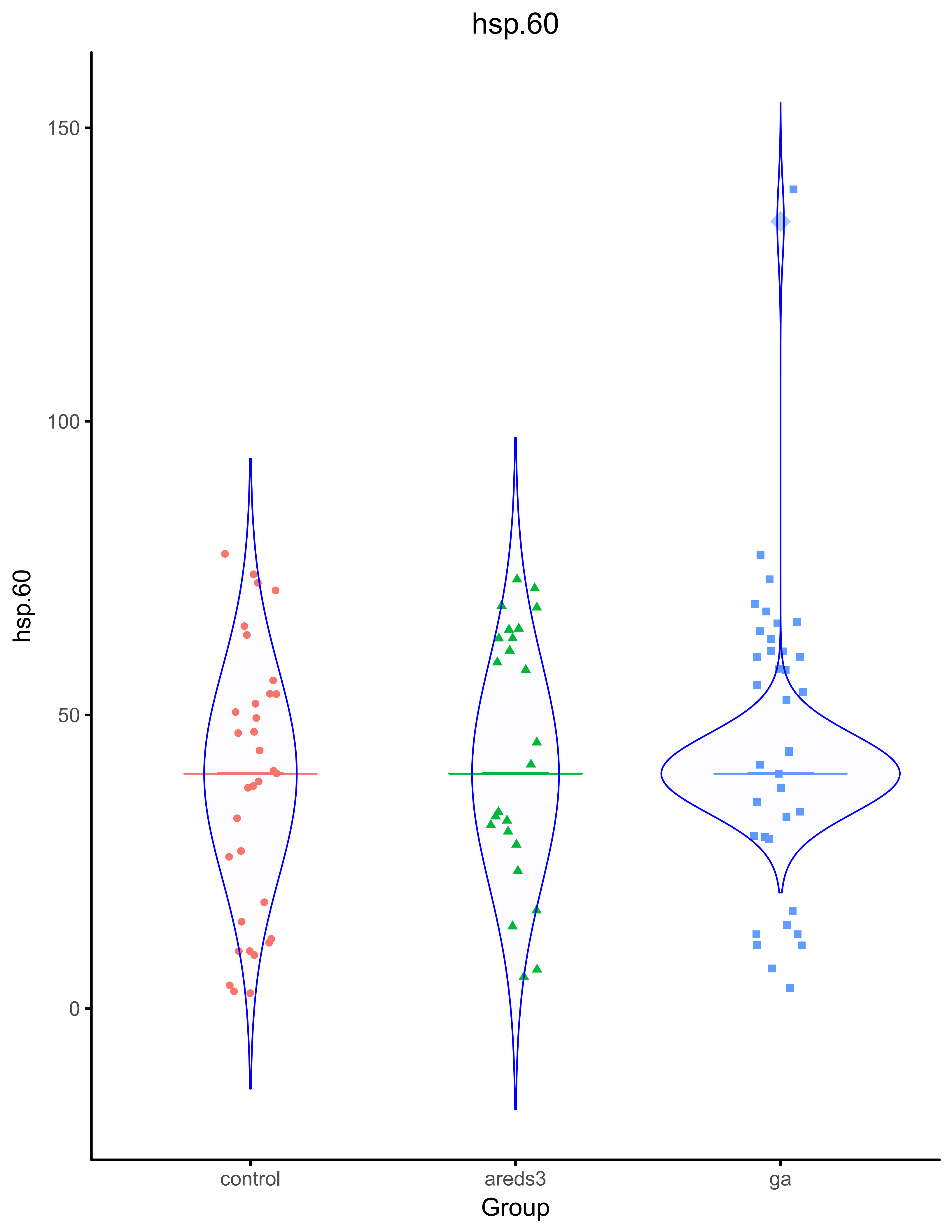
hsp.60
150
100
50
0
hsp.60
control
areds3
ga
Group

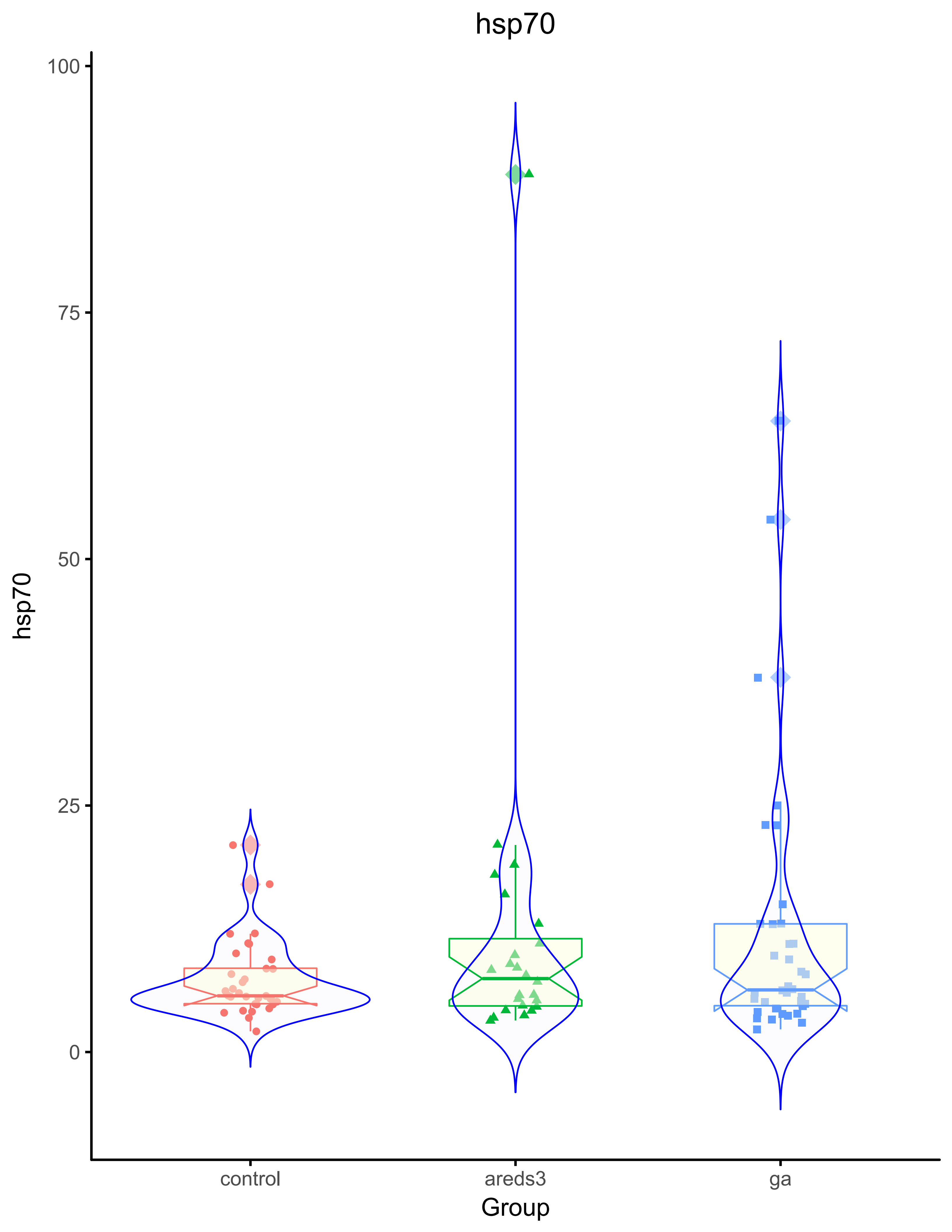

hsp70
100
75
50
25
0
hsp70
control
areds3
ga
Group

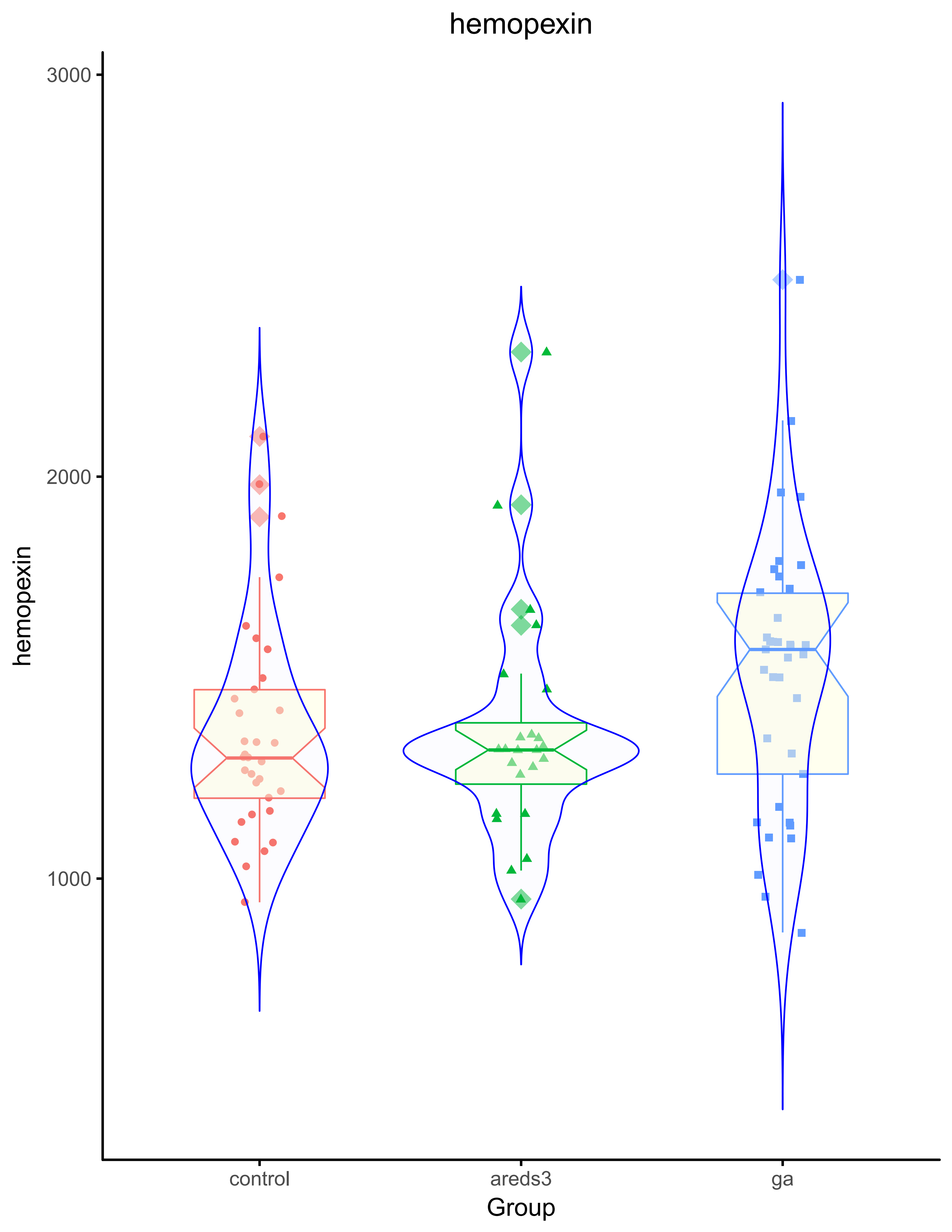
hemopexin
3000
2000
1000
hemopexin
control
areds3
ga
Group

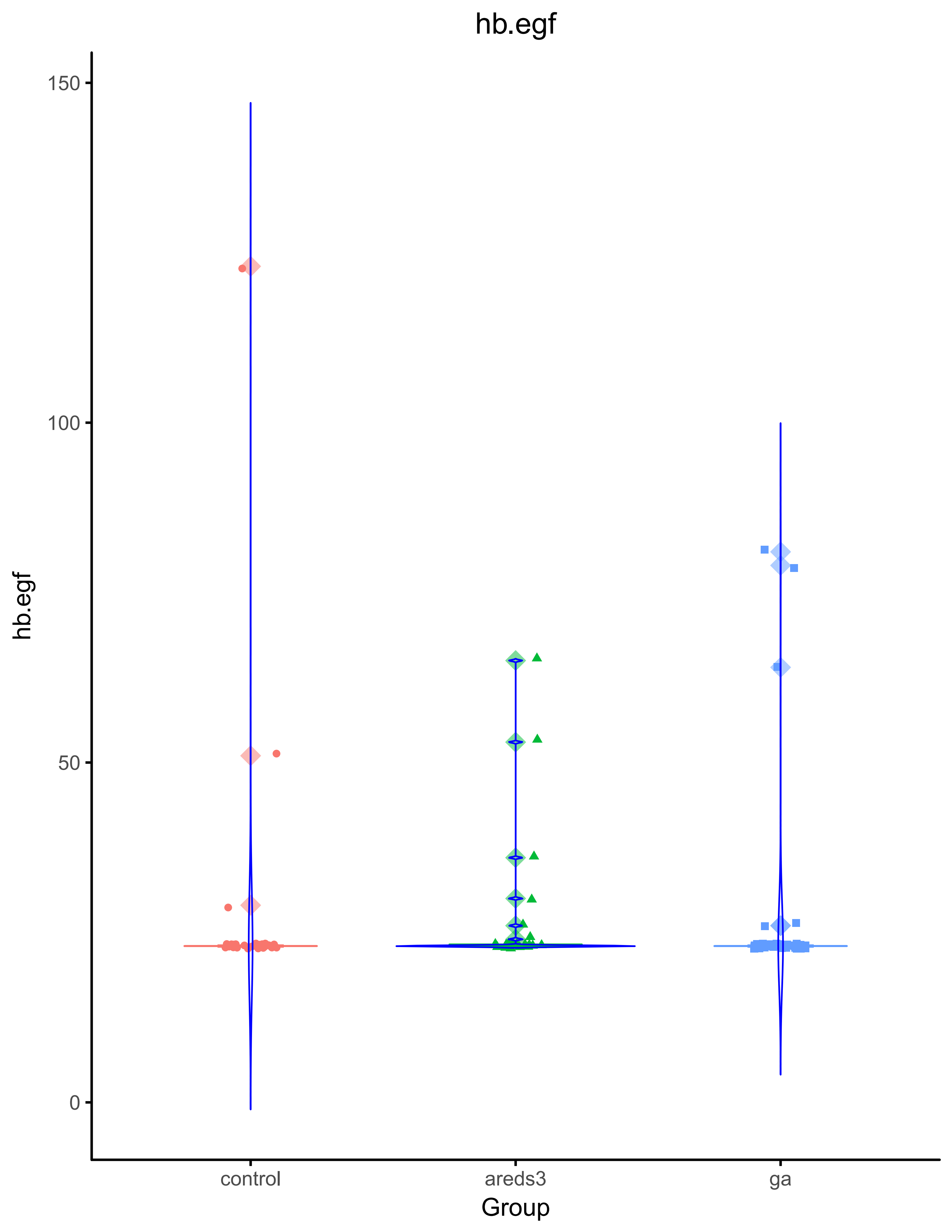
hb.egf
150
100
50
0
hb.egf
control
areds3
ga
Group

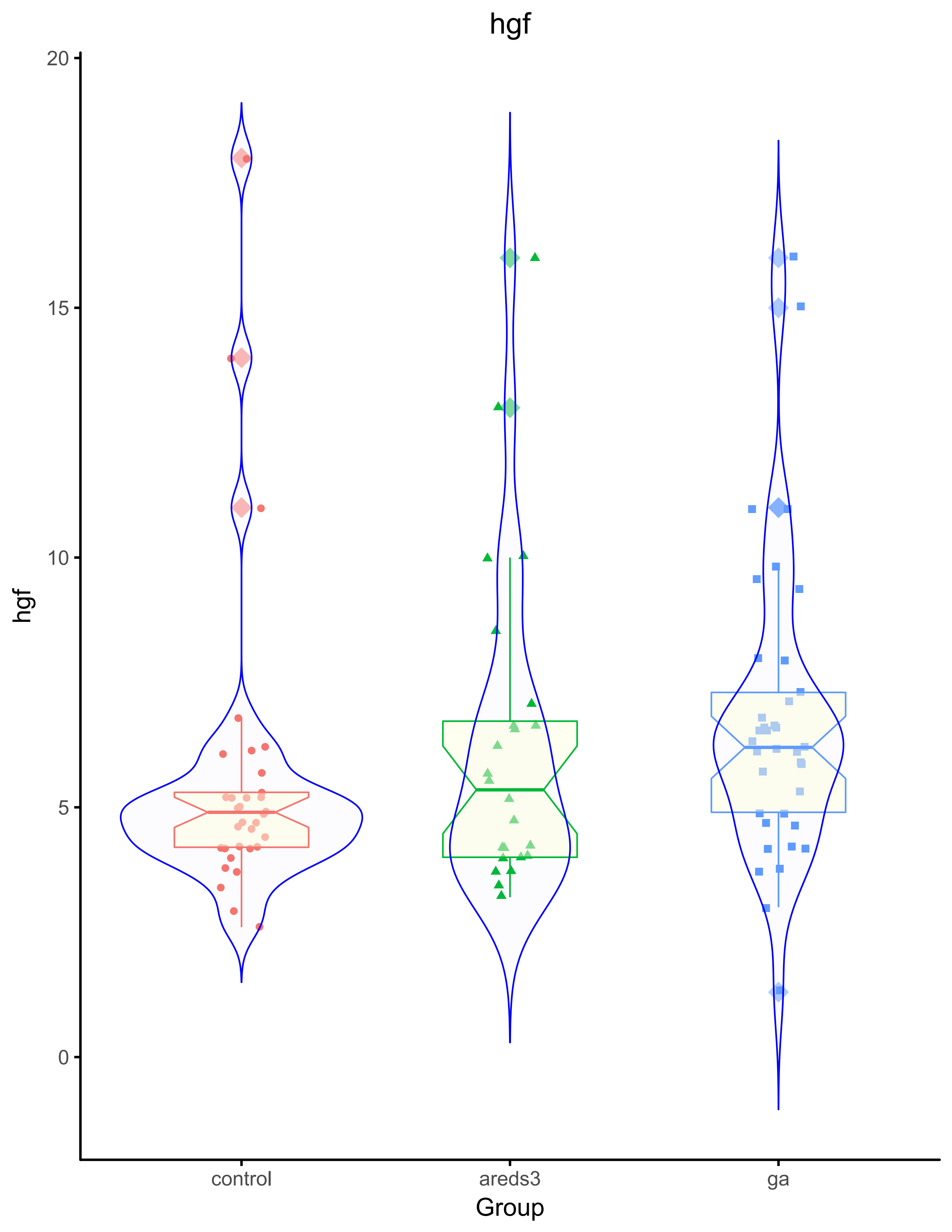
hgf
20
15
10
5
0
hgf
control
areds3
ga
Group

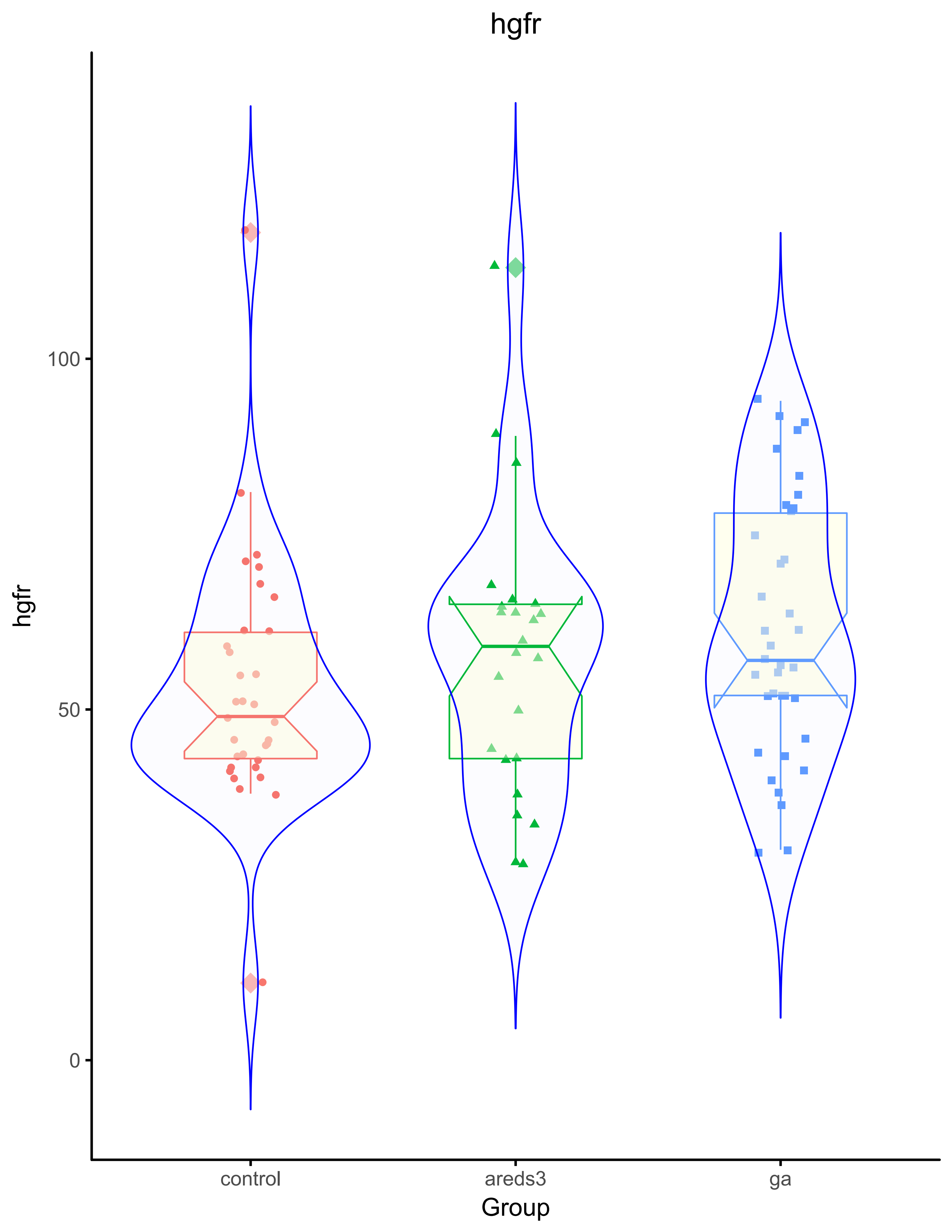

hgfr
100
50
0
hgfr
control
areds3
ga
Group

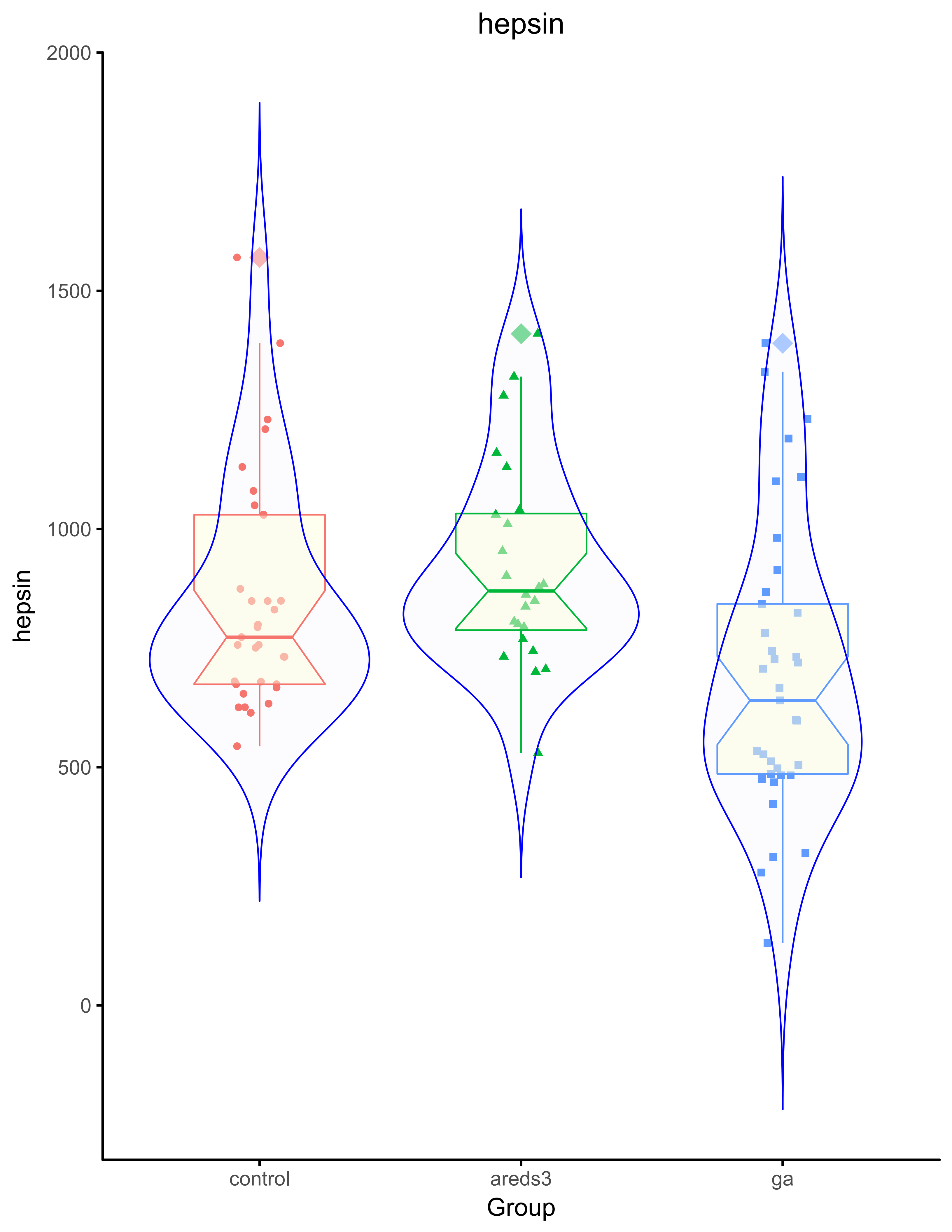
hepsin
2000
1500
1000
500
0
hepsin
control
areds3
ga
Group

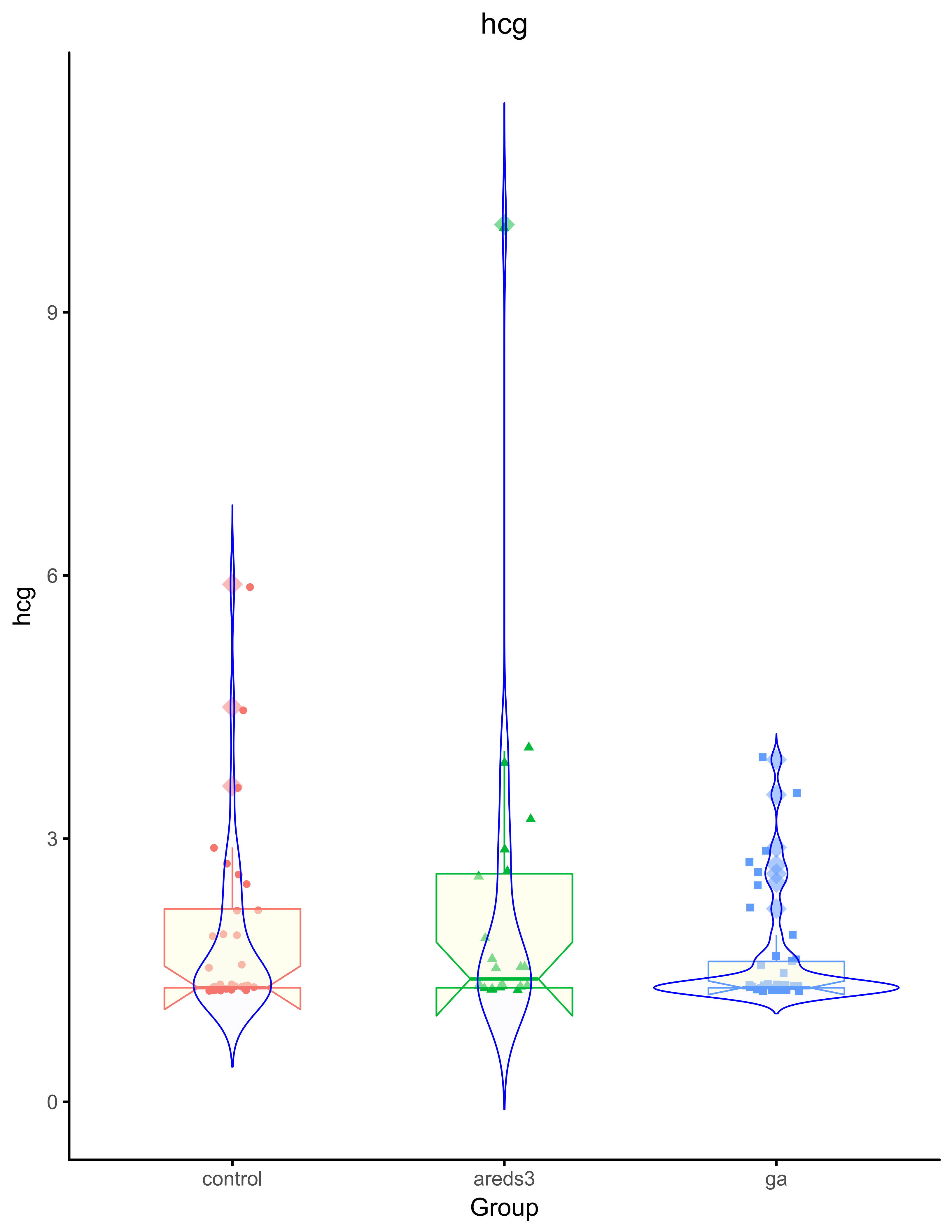
hcg
hcg
0
3
6
9
control
areds3
ga
Group

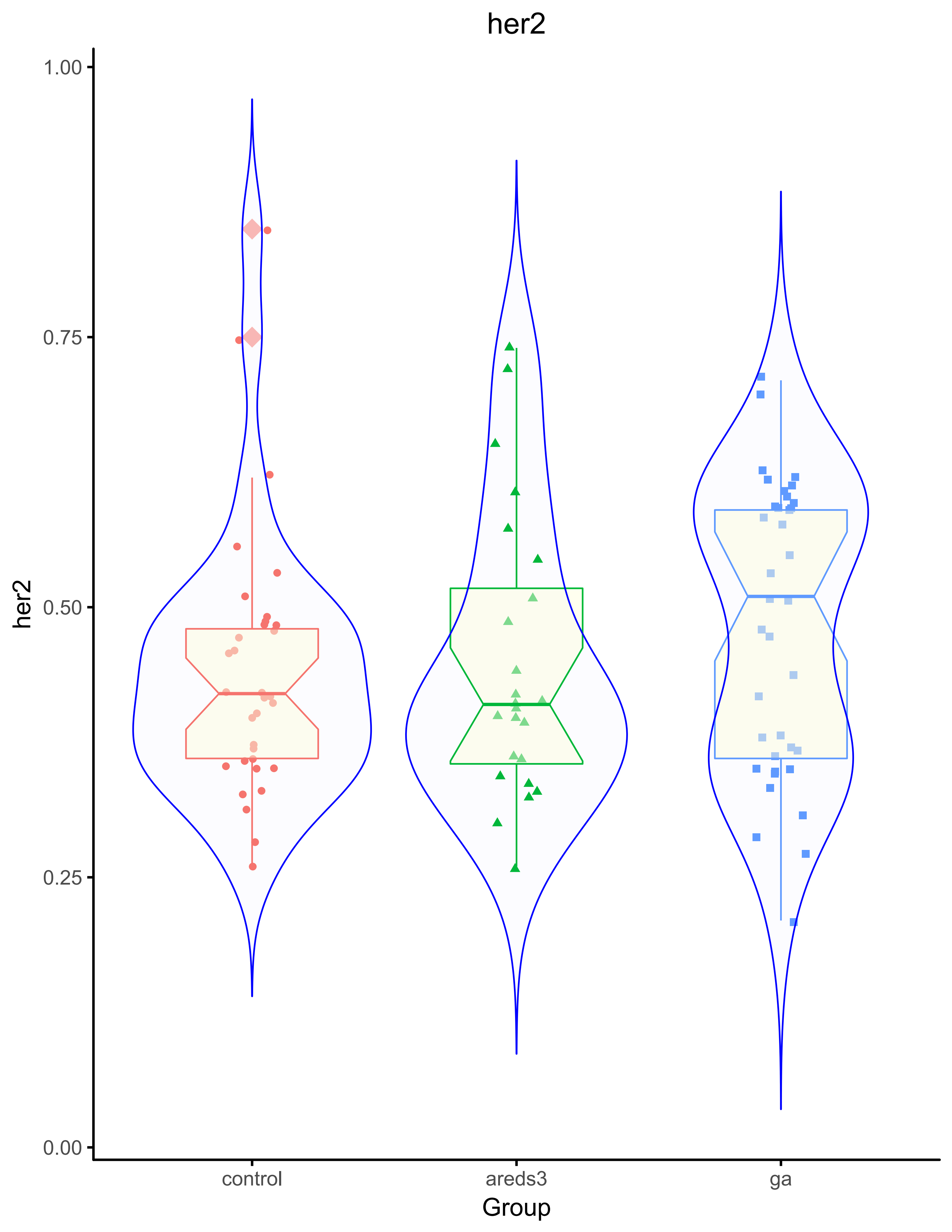
her2
1.00
0.75
0.50
0.25
0.00
her2
control
areds3
ga
Group

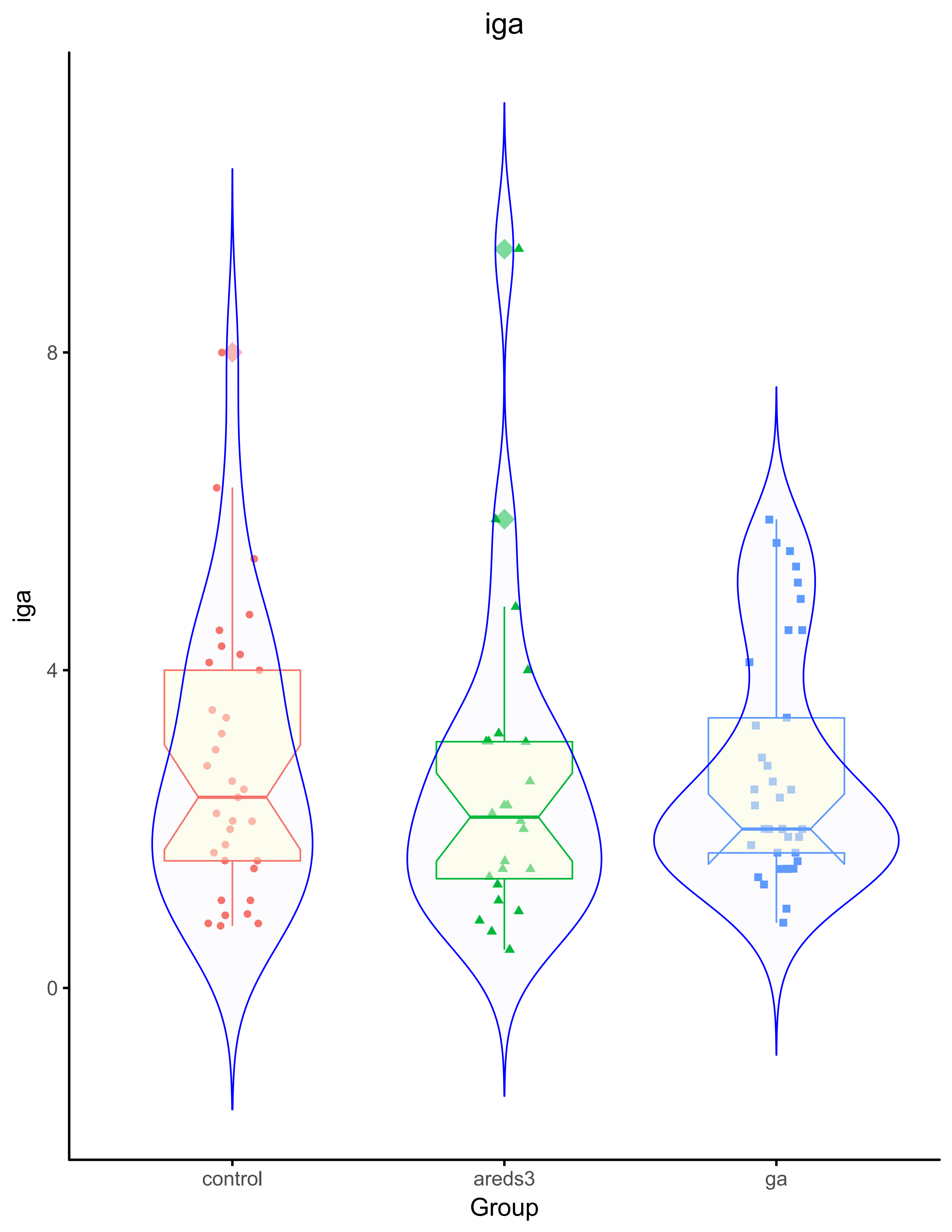

iga
8
4
0
iga
control
areds3
ga
Group

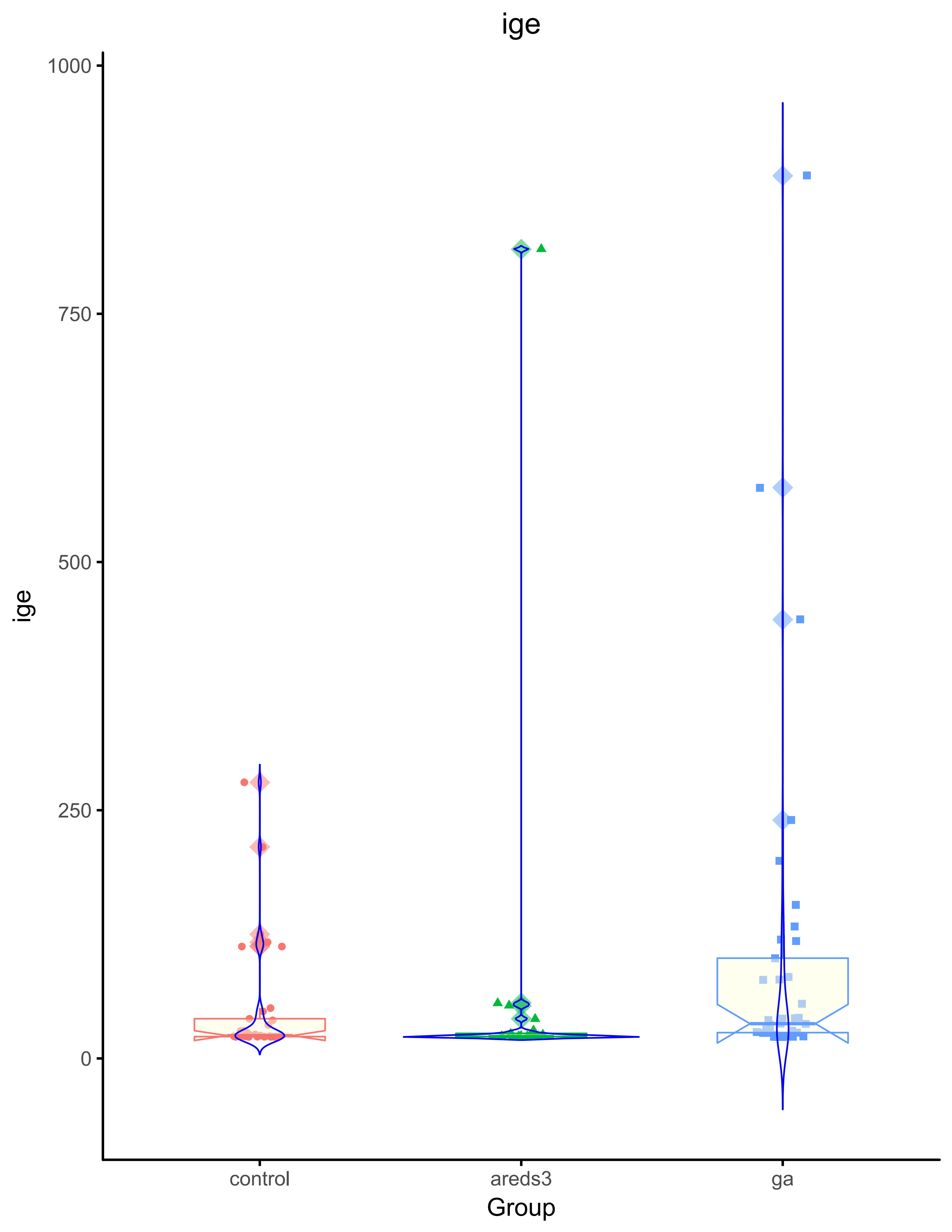
ige
1000
750
500
250
0
ige
control
areds3
ga
Group

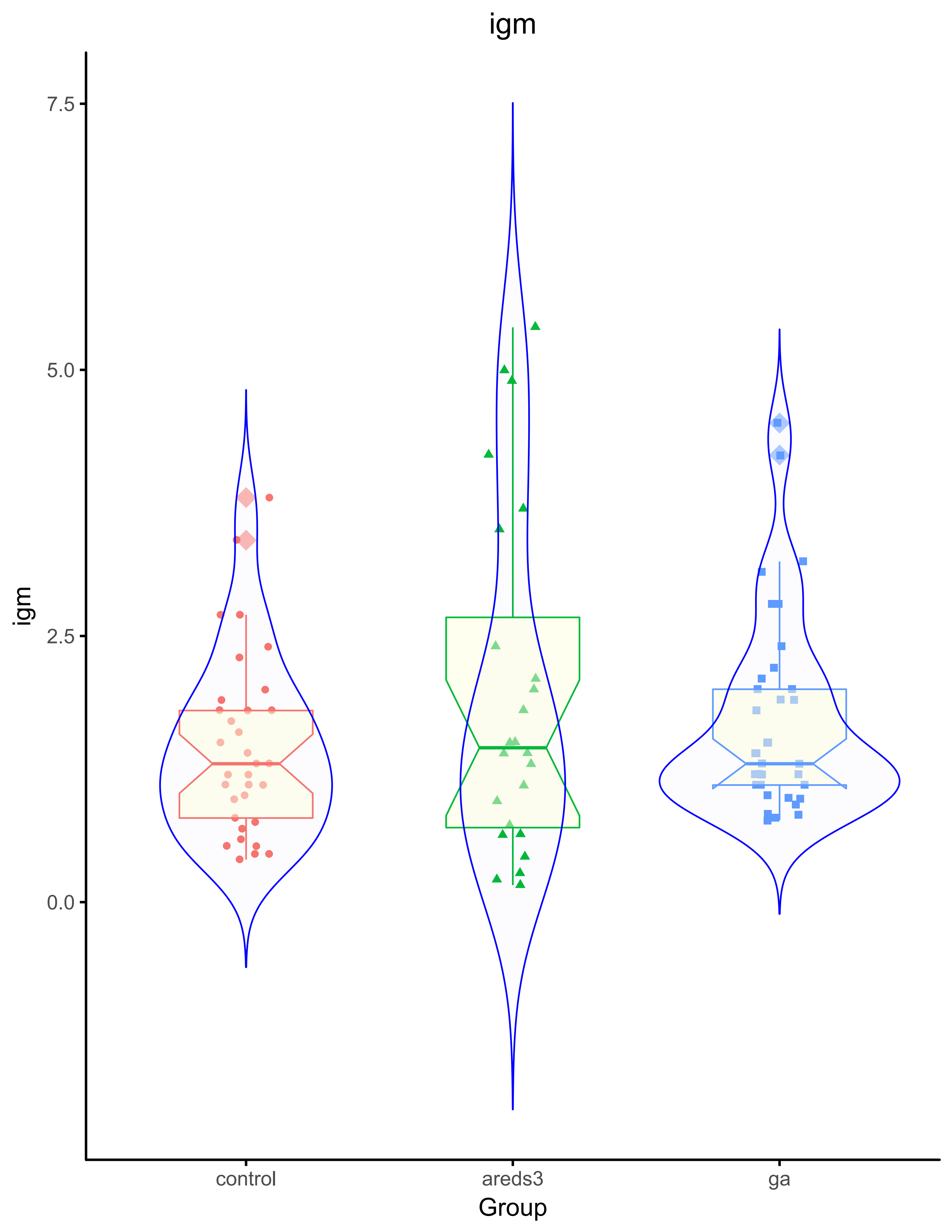

igm
7.5
5.0
2.5
0.0
igm
control
areds3
ga
Group

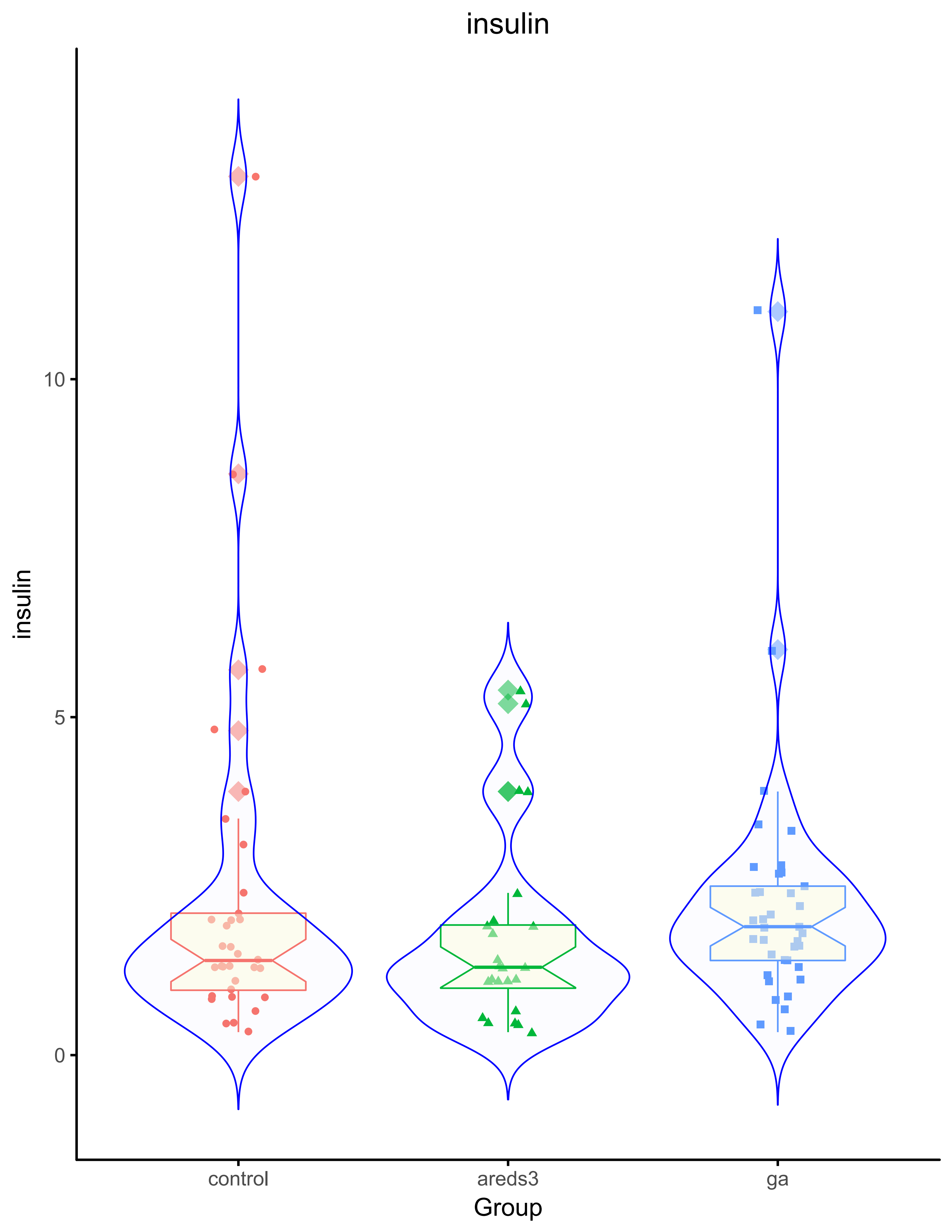

insulin
10
5
0
insulin
control
areds3
ga
Group

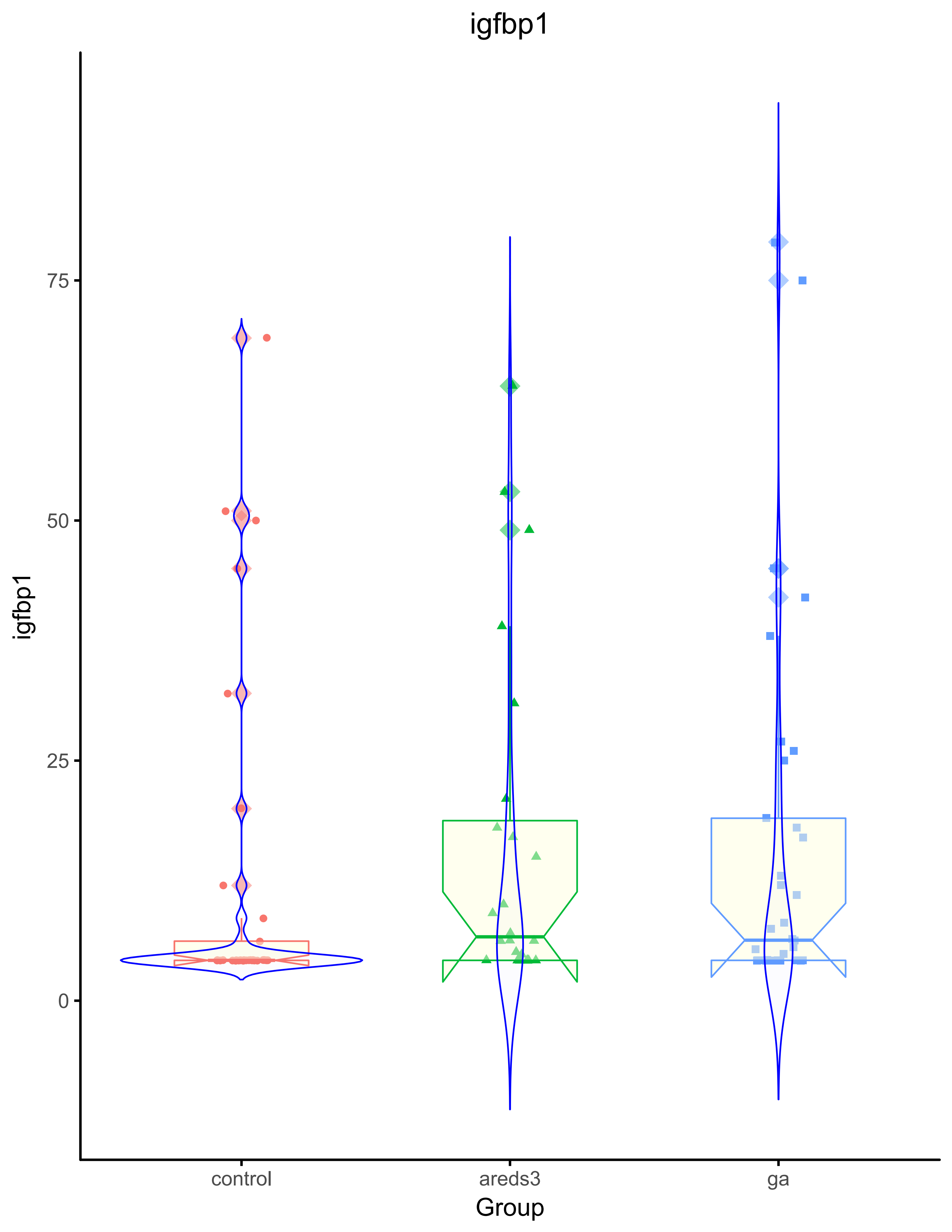
igfbp1
igfbp1
75
50
25
0
control
areds3
ga
Group

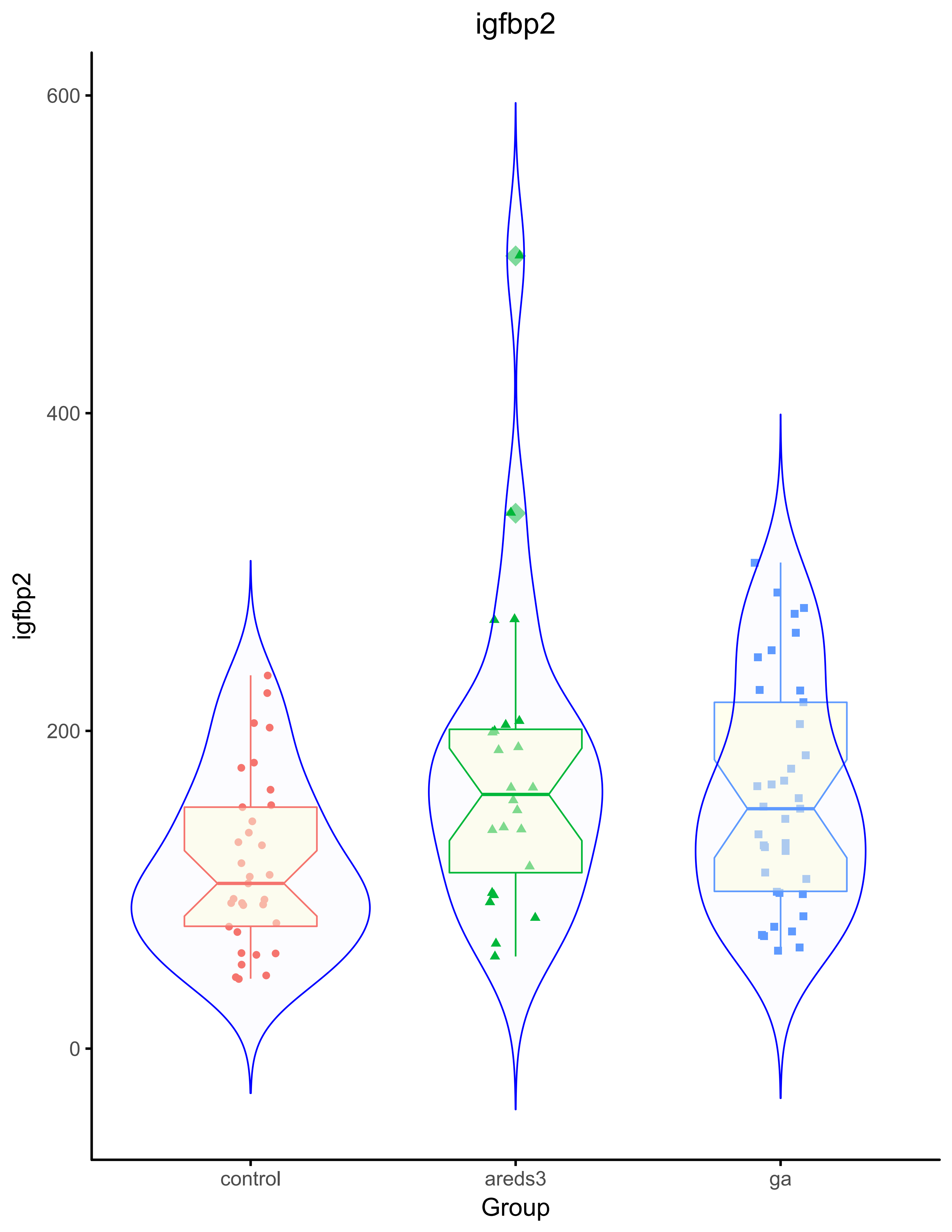
igfbp2
600
400
200
0
igfbp2
control
areds3
ga
Group

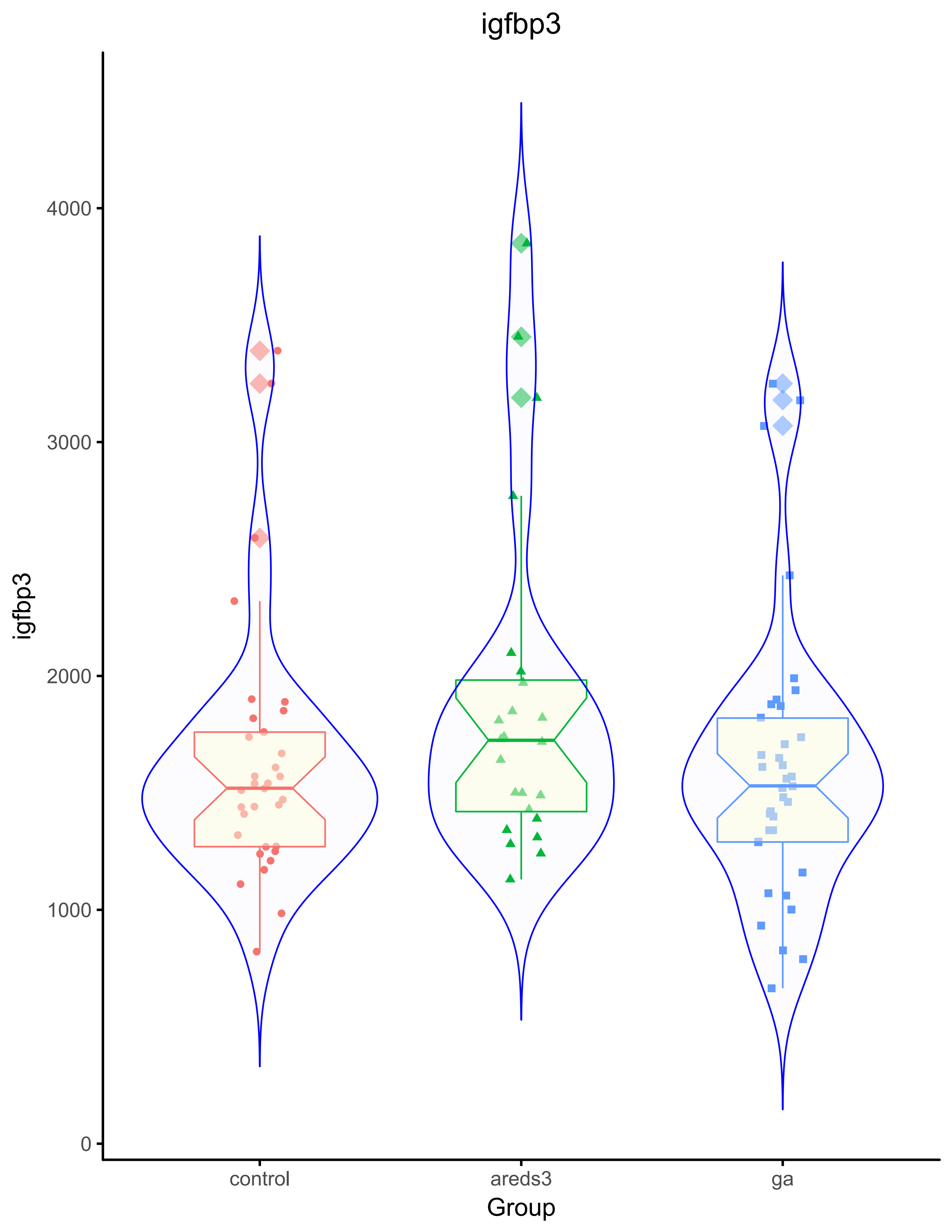

igfbp3
4000
3000
2000
1000
0
igfbp3
control
areds3
ga
Group

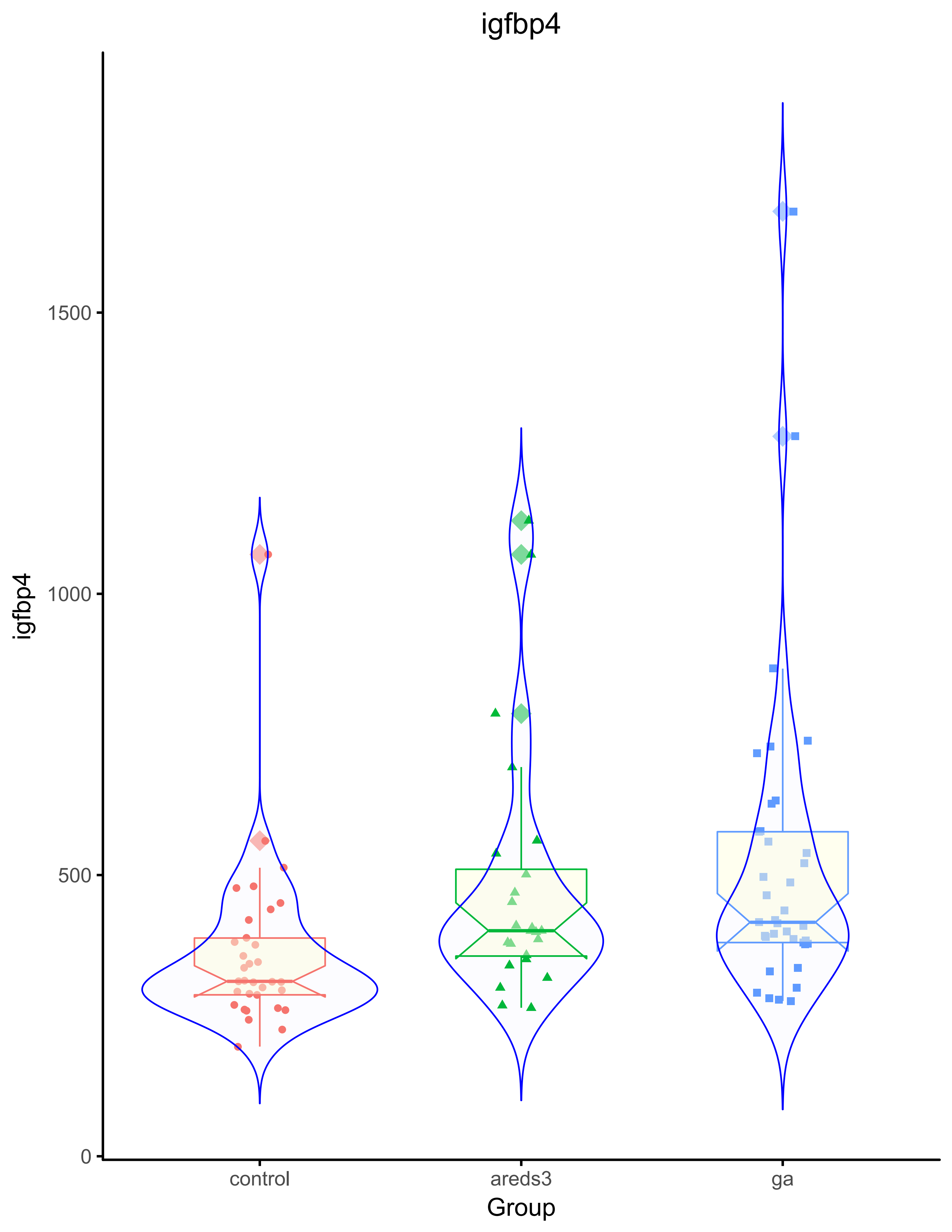

igfbp4
1500
1000
500
0
igfbp4
control
areds3
ga
Group

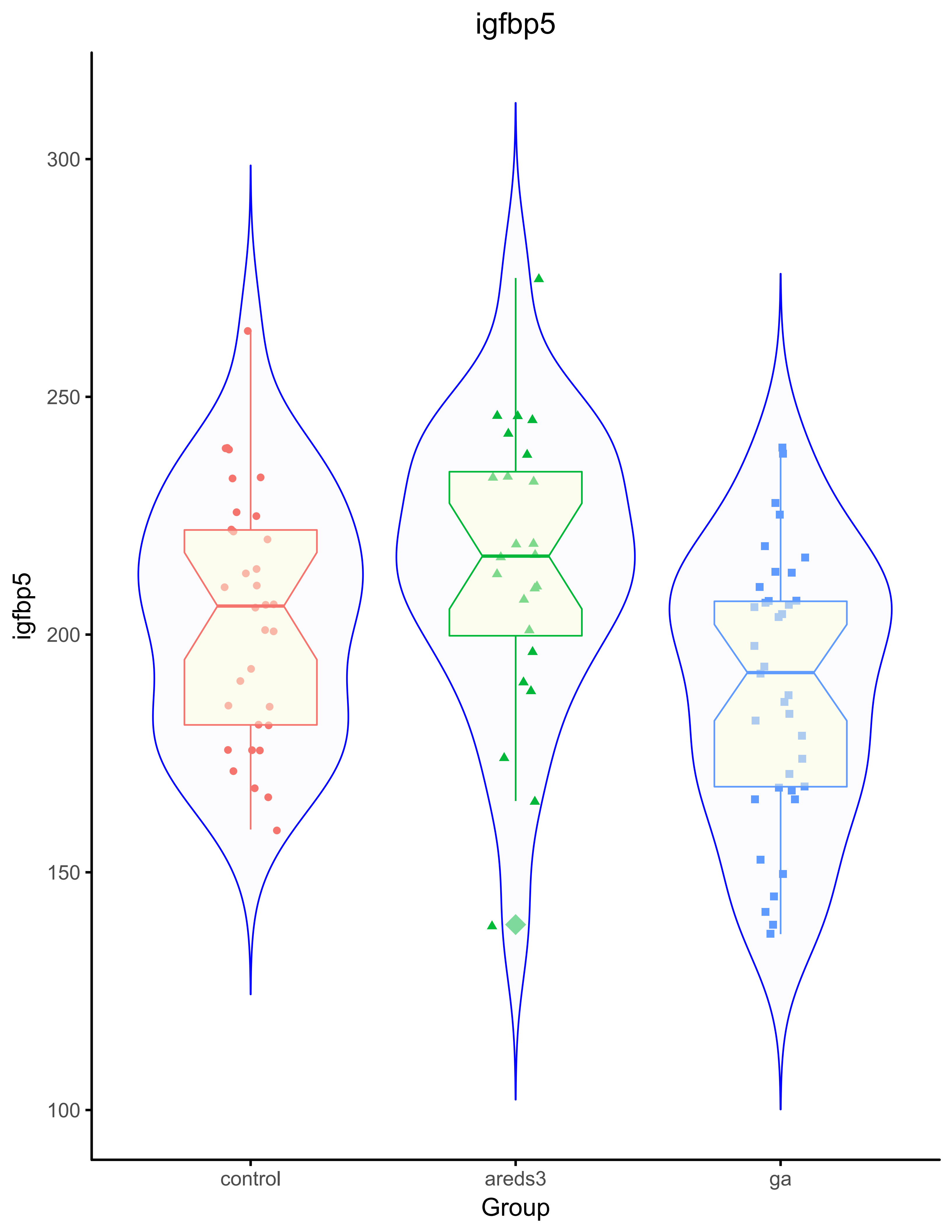
igfbp5
300
250
200
150
100
igfbp5
control
areds3
ga
Group

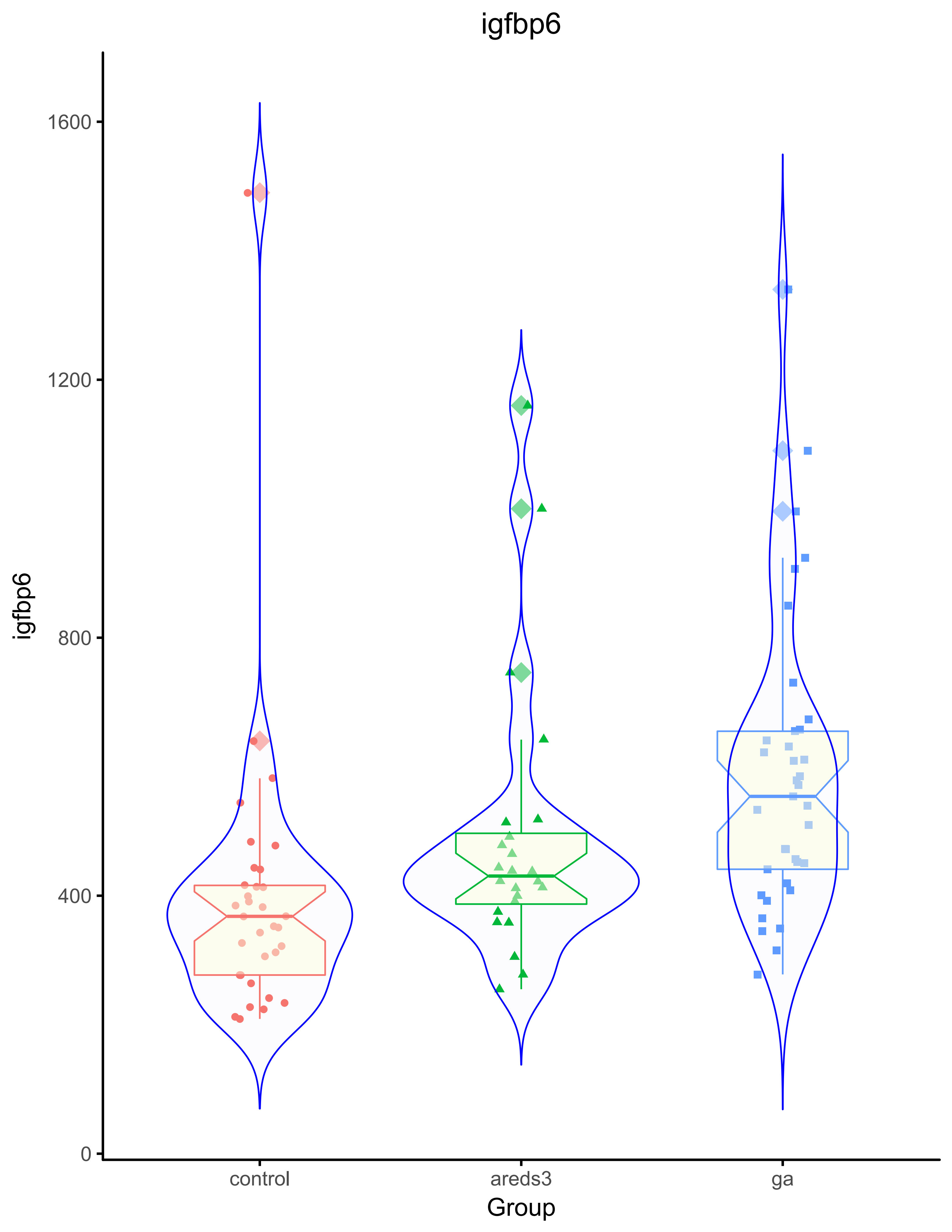
igfbp6
igfbp6
1600
1200
800
400
0
control
areds3
ga
Group

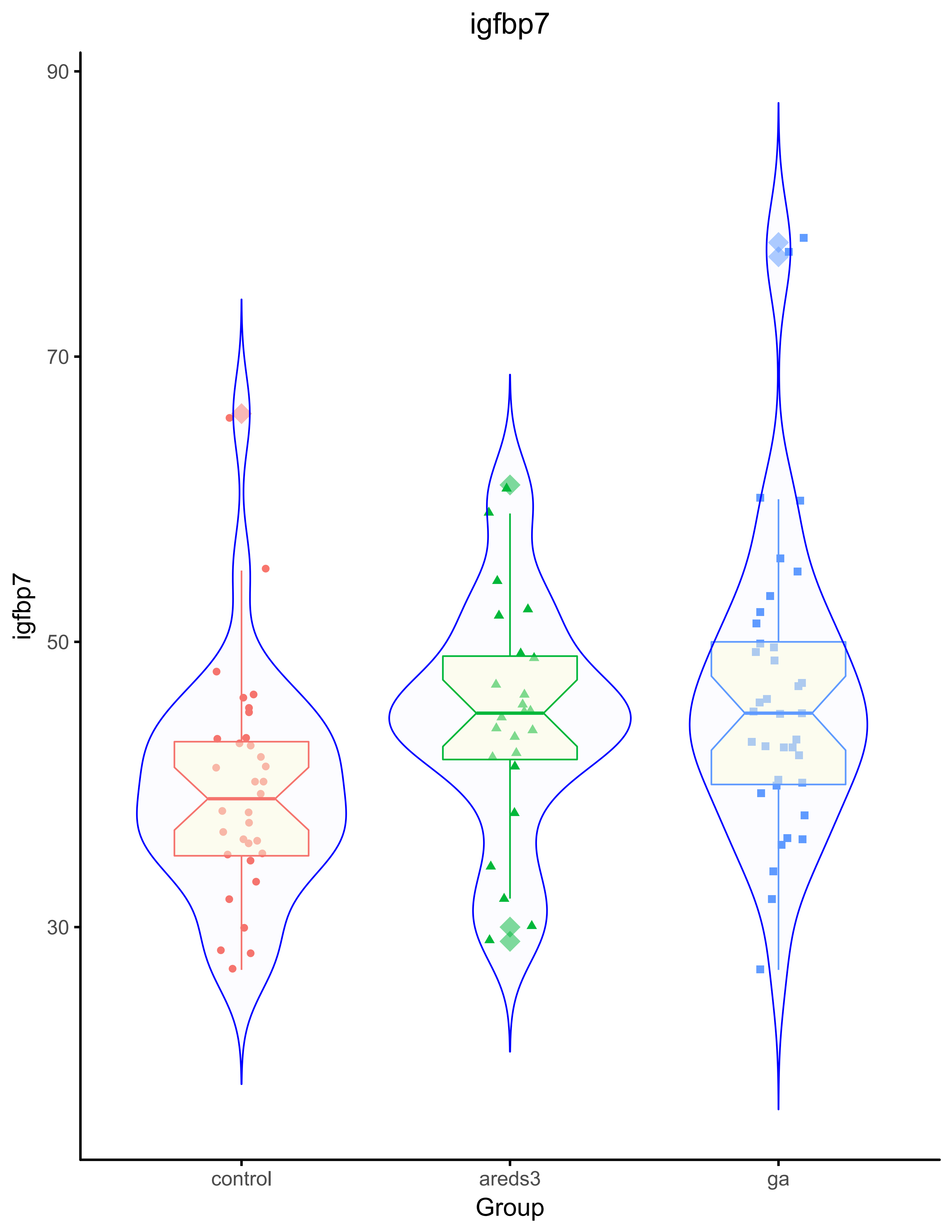
igfbp7
90
70
50
30
igfbp7
control
areds3
ga
Group

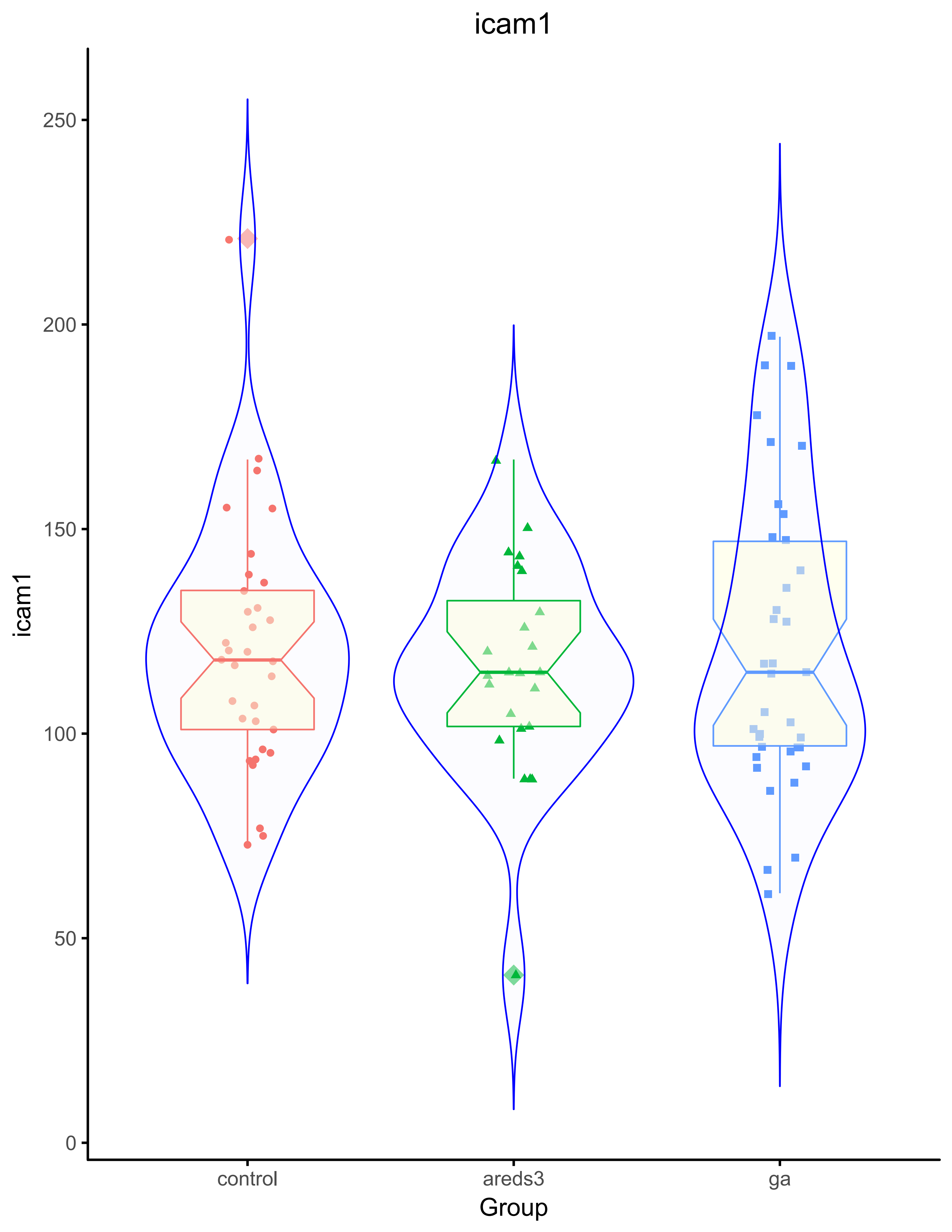
icam1
250
200
150
100
50
0
icam1
control
areds3
ga
Group

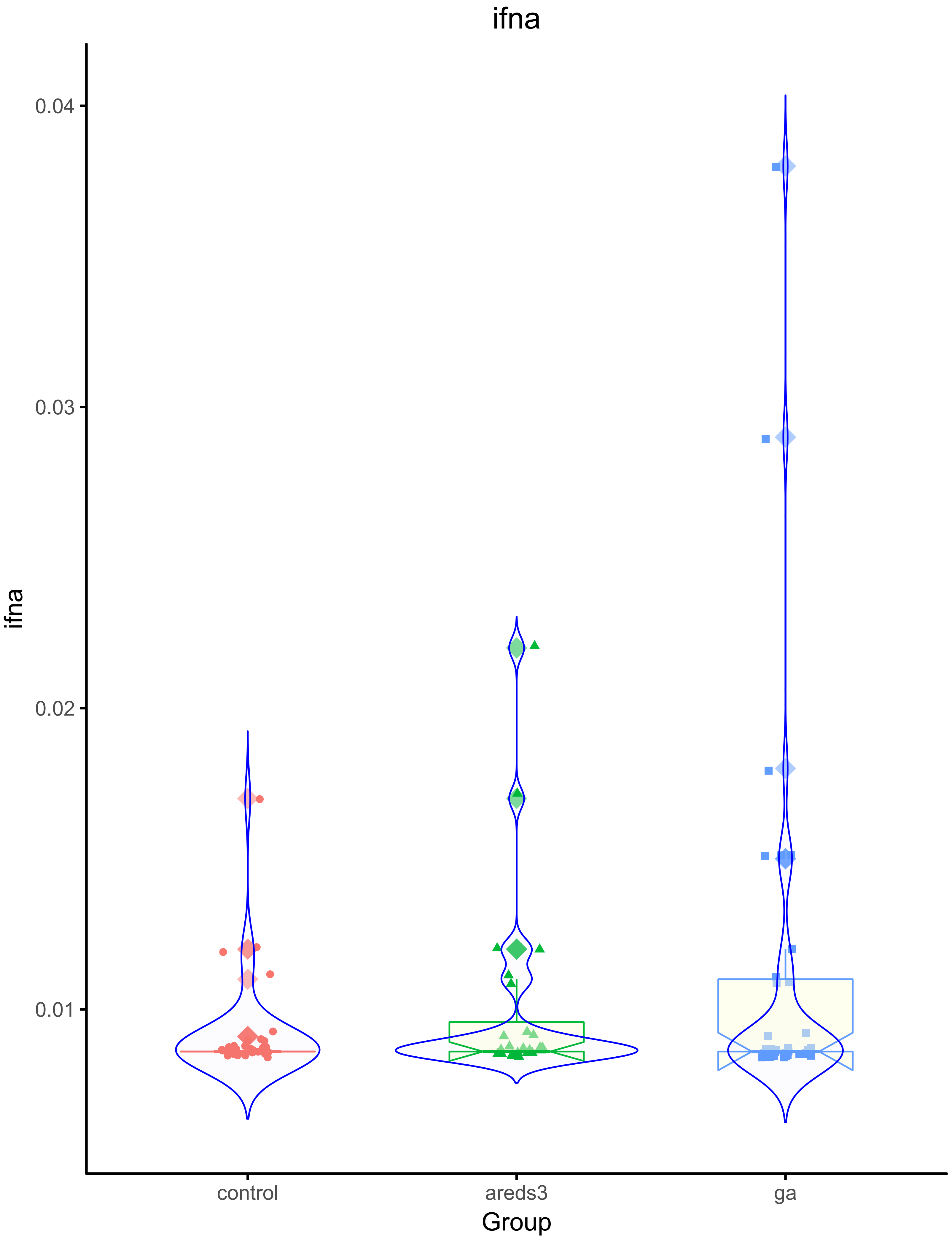

ifna
0.04
0.03
0.02
0.01
ifna
control
areds3
ga
Group

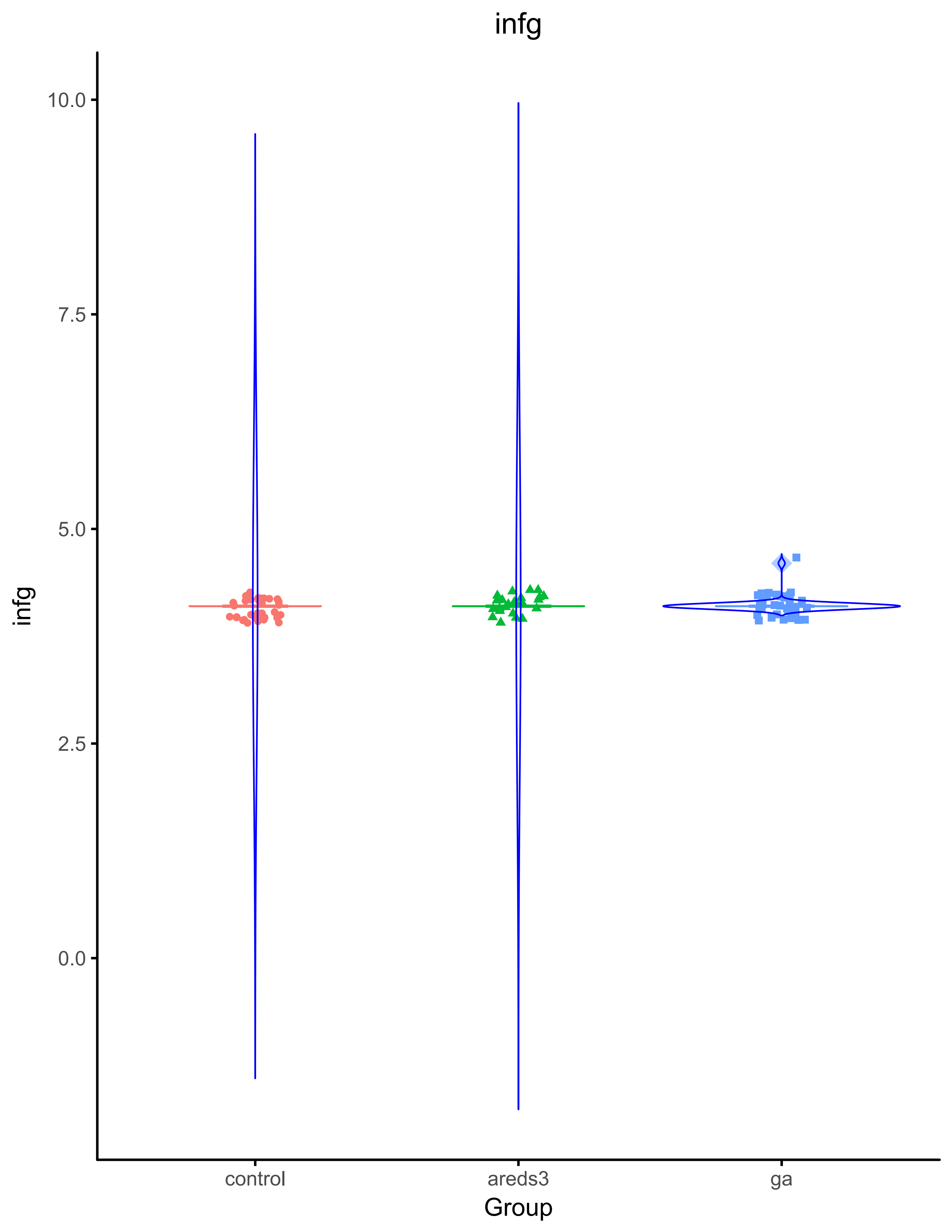

infg
10.0
7.5
5.0
2.5
0.0
infg
control
areds3
ga
Group

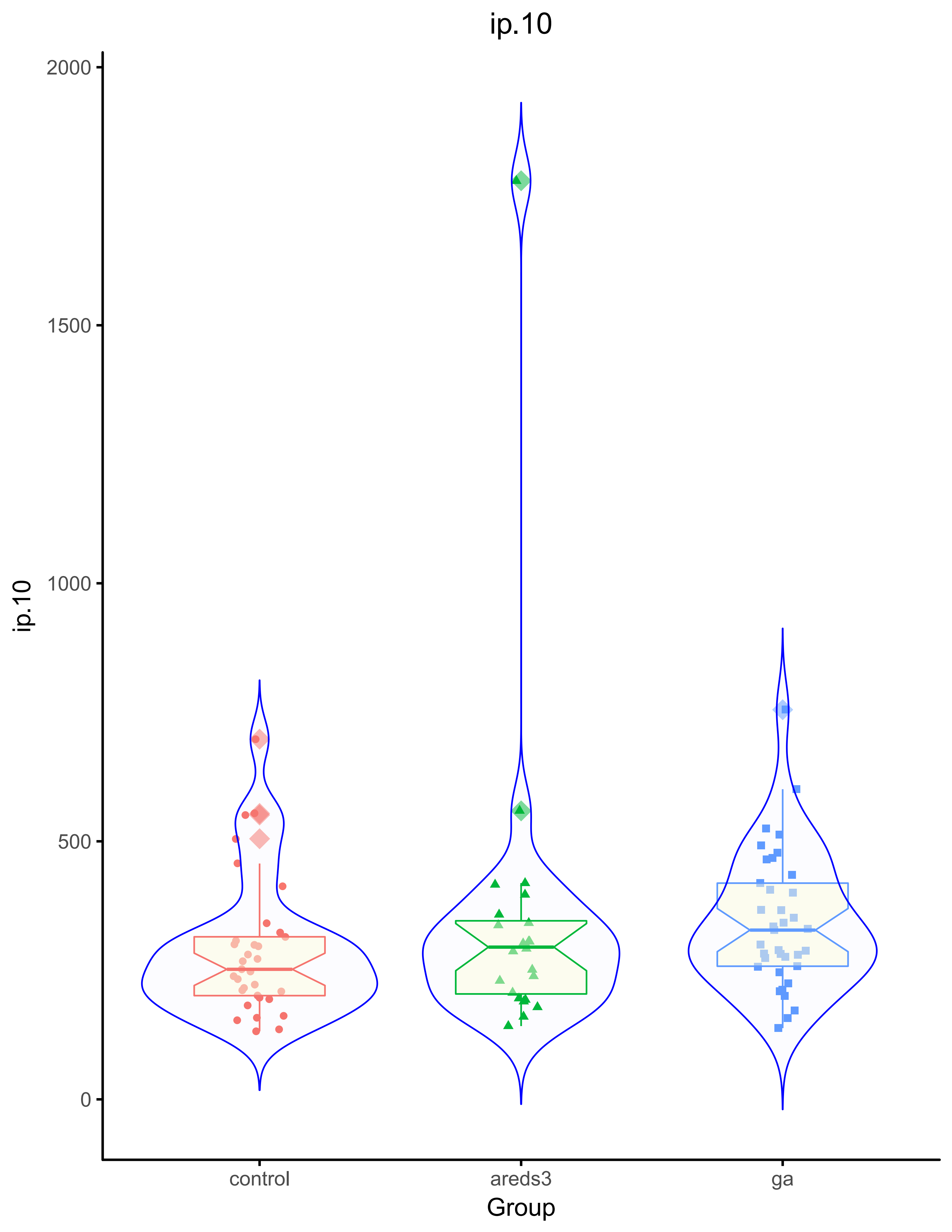

ip.10
2000
1500
1000
500
0
ip.10
control
areds3
ga
Group

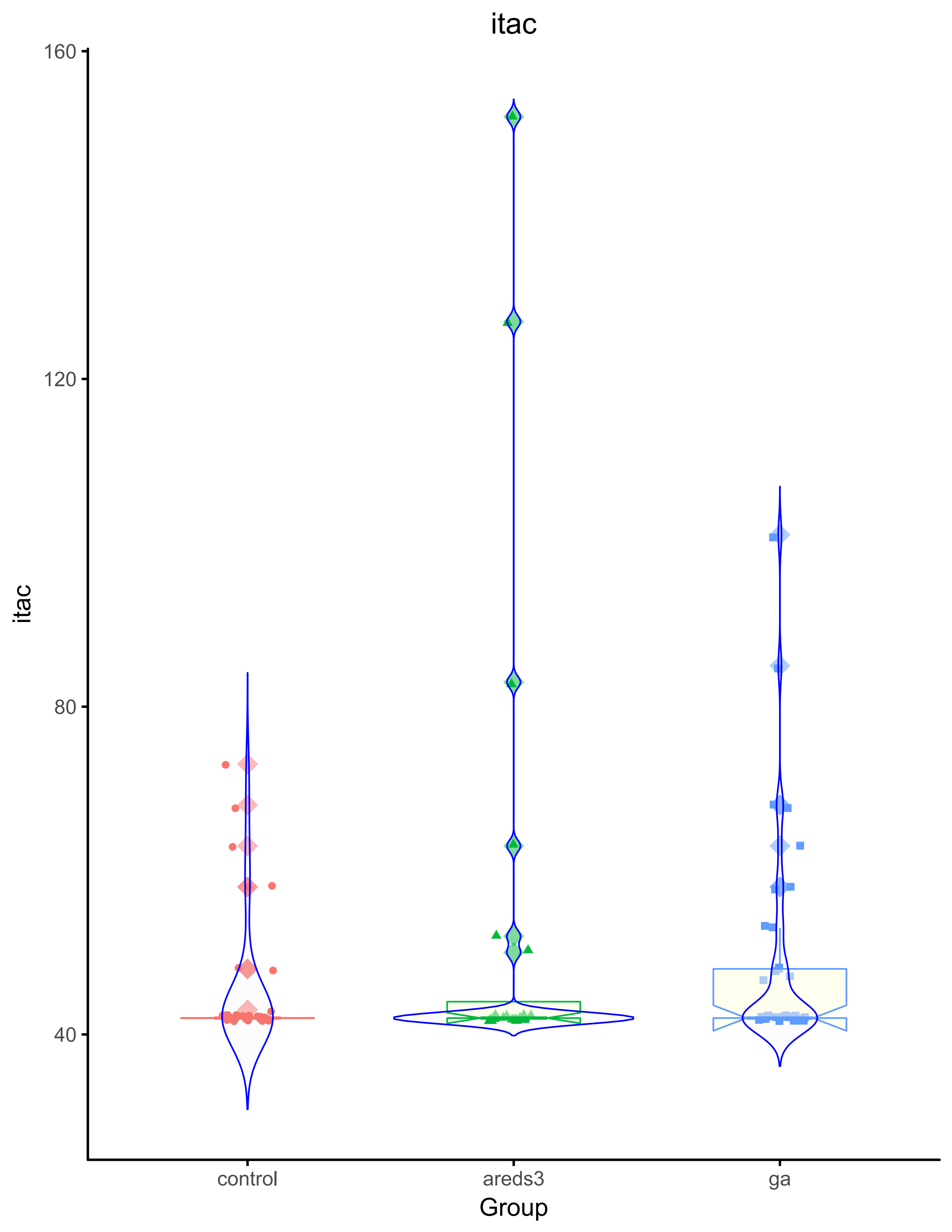

itac
160
120
80
40
itac
control
areds3
ga
Group

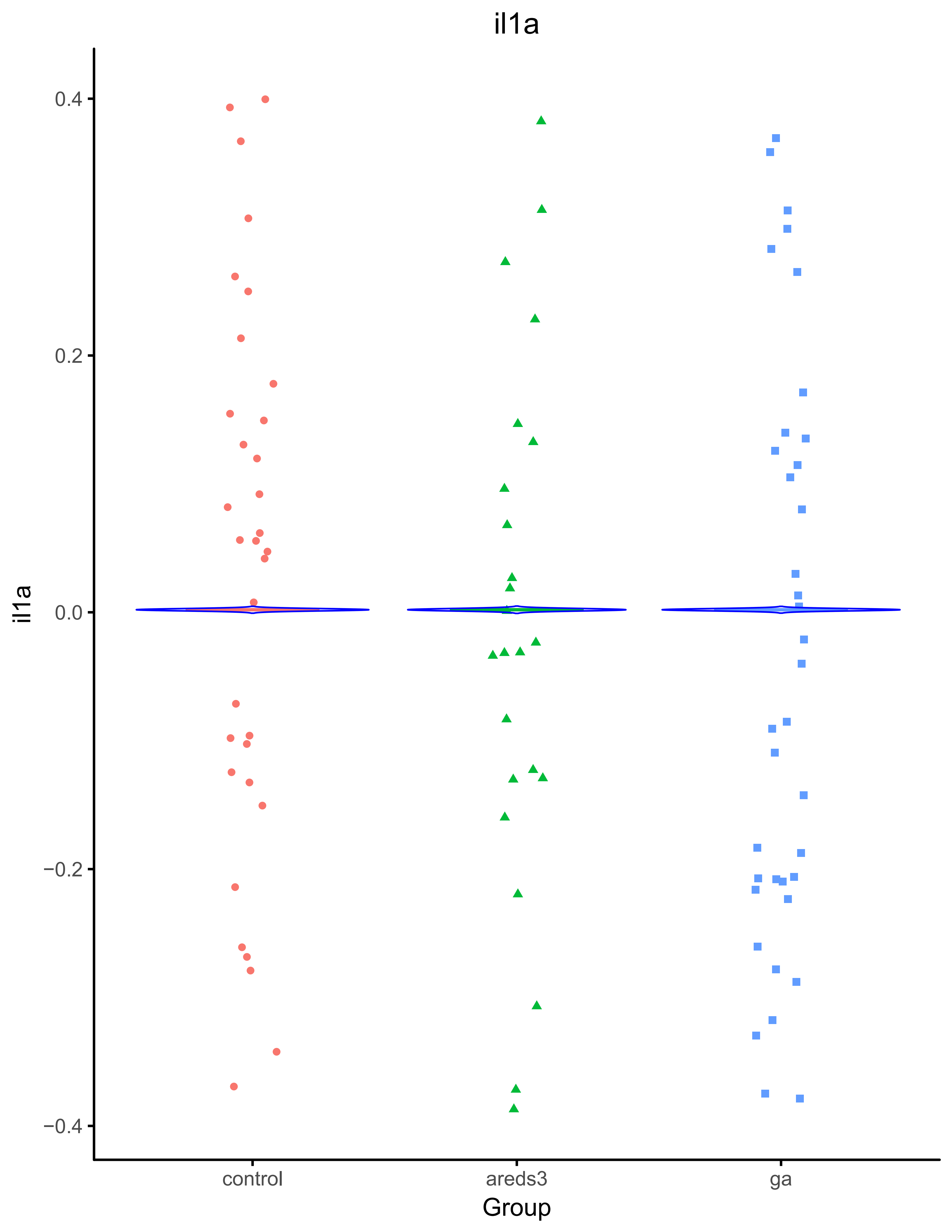

il1a
0.4
0.2
0.0
−0.2
−0.4
il1a
control
areds3
ga
Group

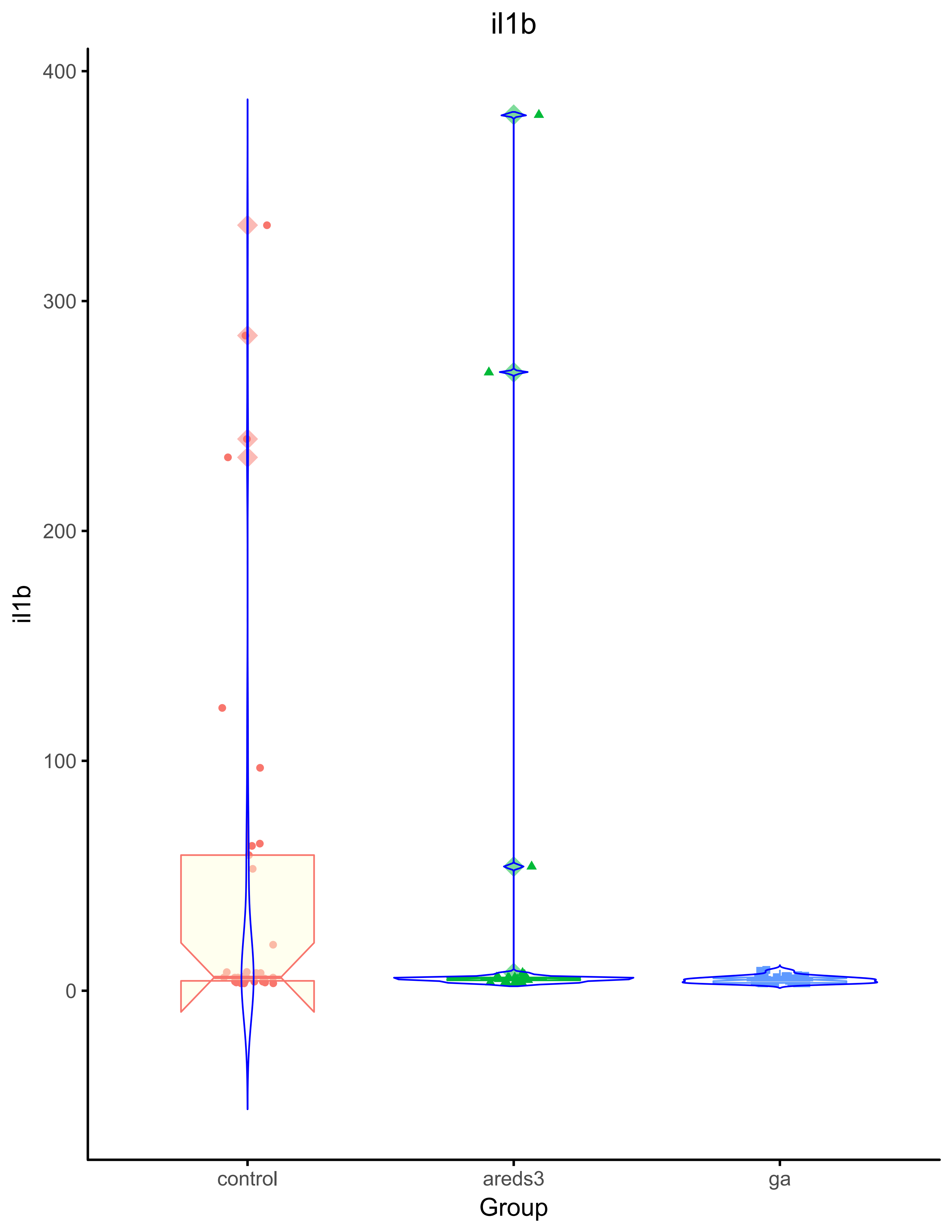
il1b
400
300
200
100
0
il1b
control
areds3
ga
Group

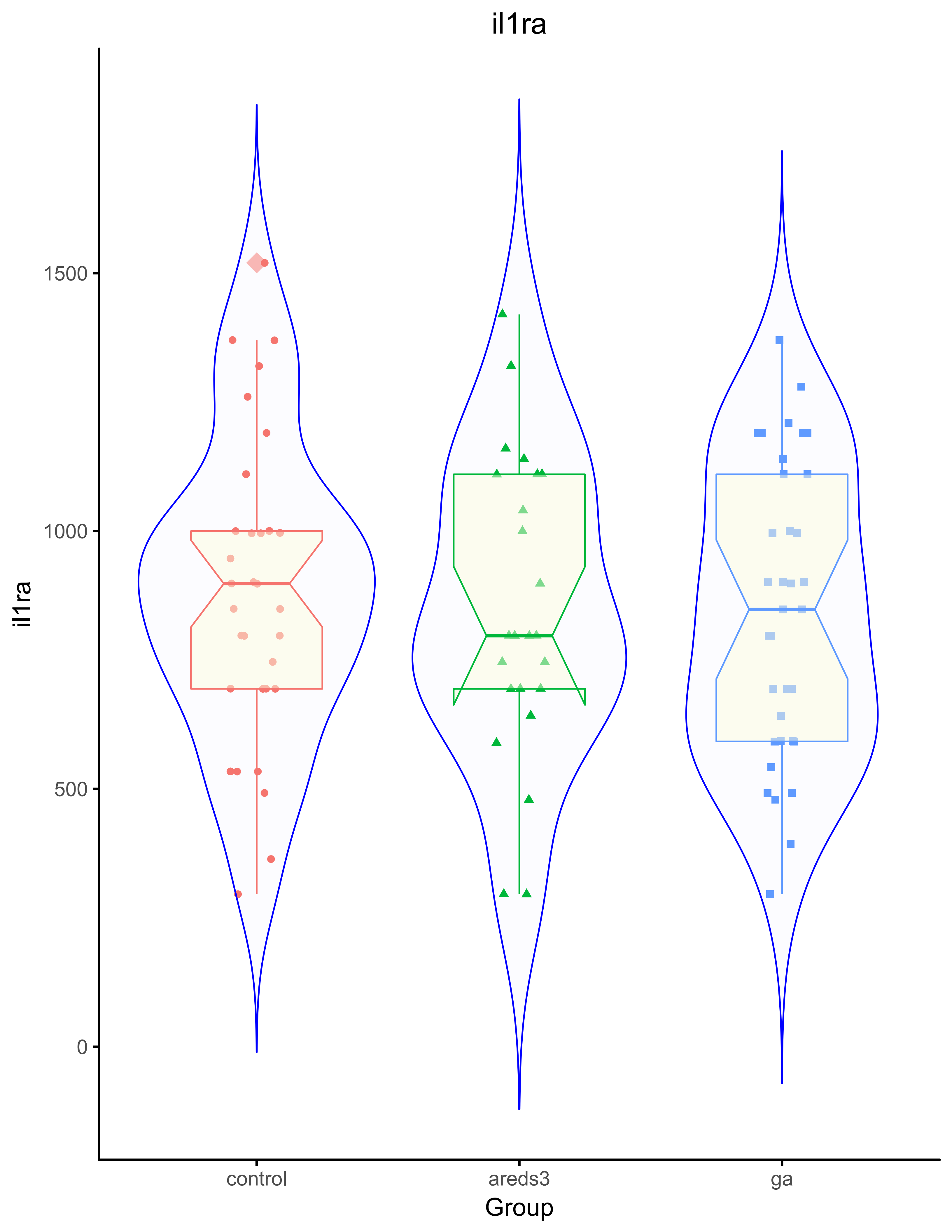

il1ra
1500
1000
500
0
il1ra
control
areds3
ga
Group

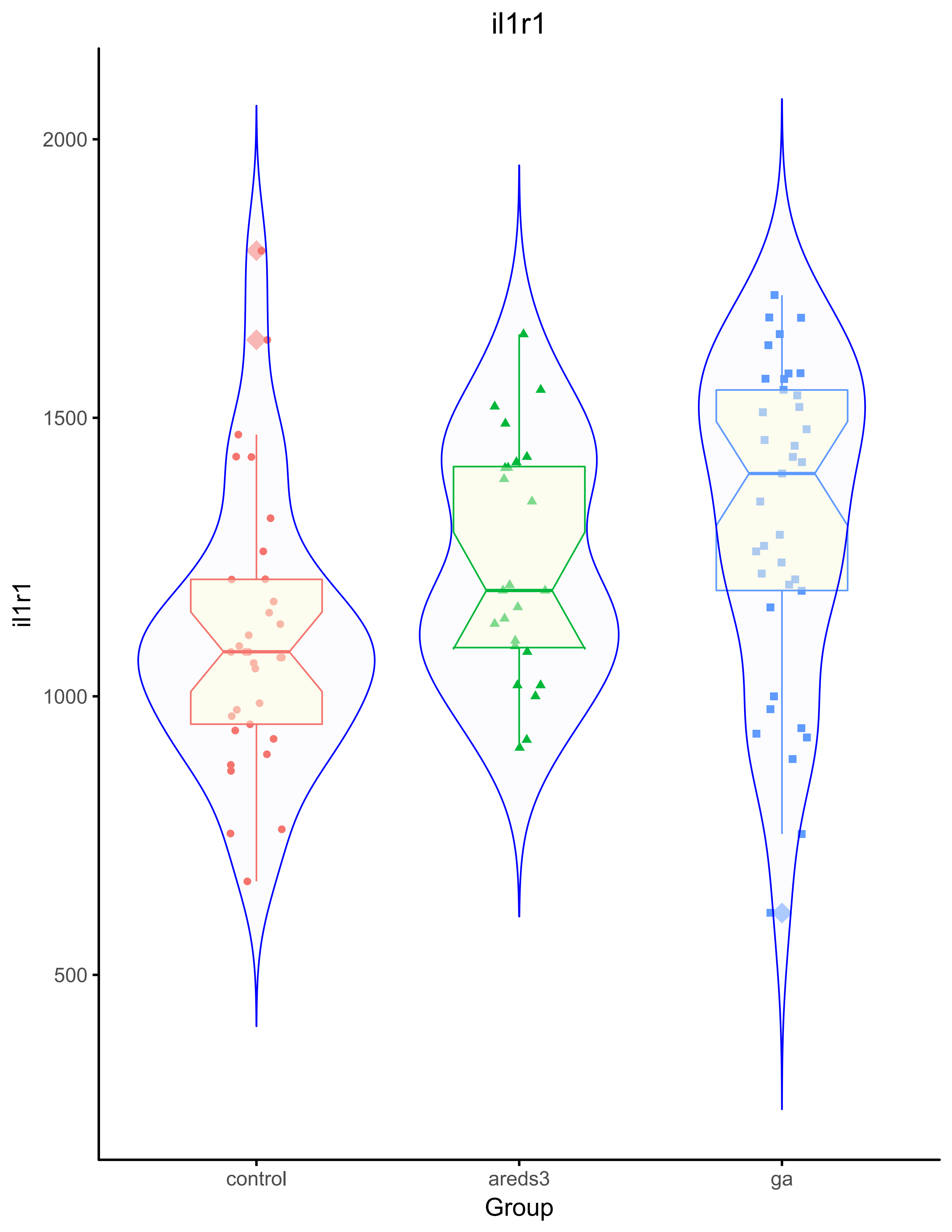

il1r1
2000
1500
1000
500
il1r1
control
areds3
ga
Group

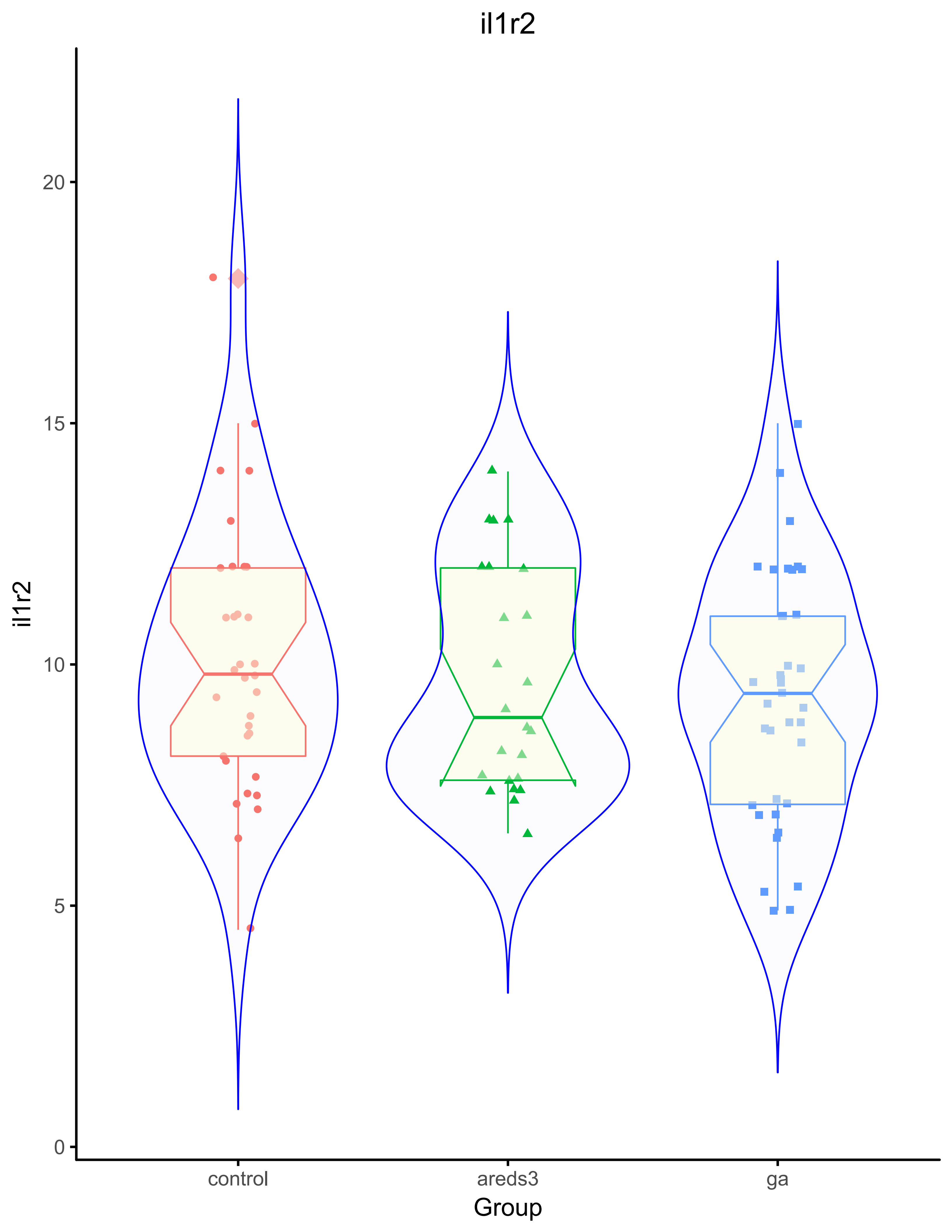
il1r2
il1r2
20
15
10
5
0
control
areds3
ga
Group

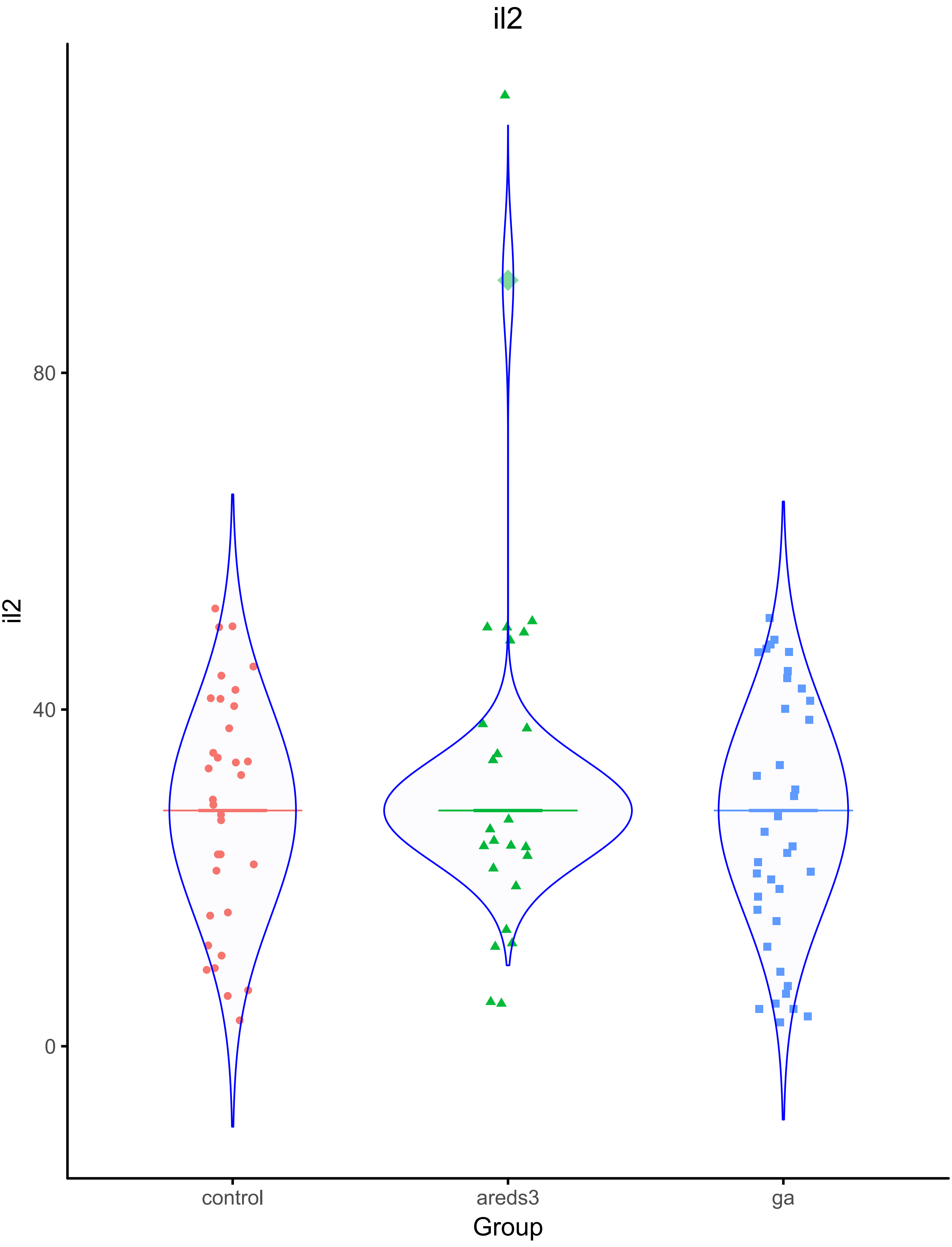
il2
80
40
0
il2
control
areds3
ga
Group

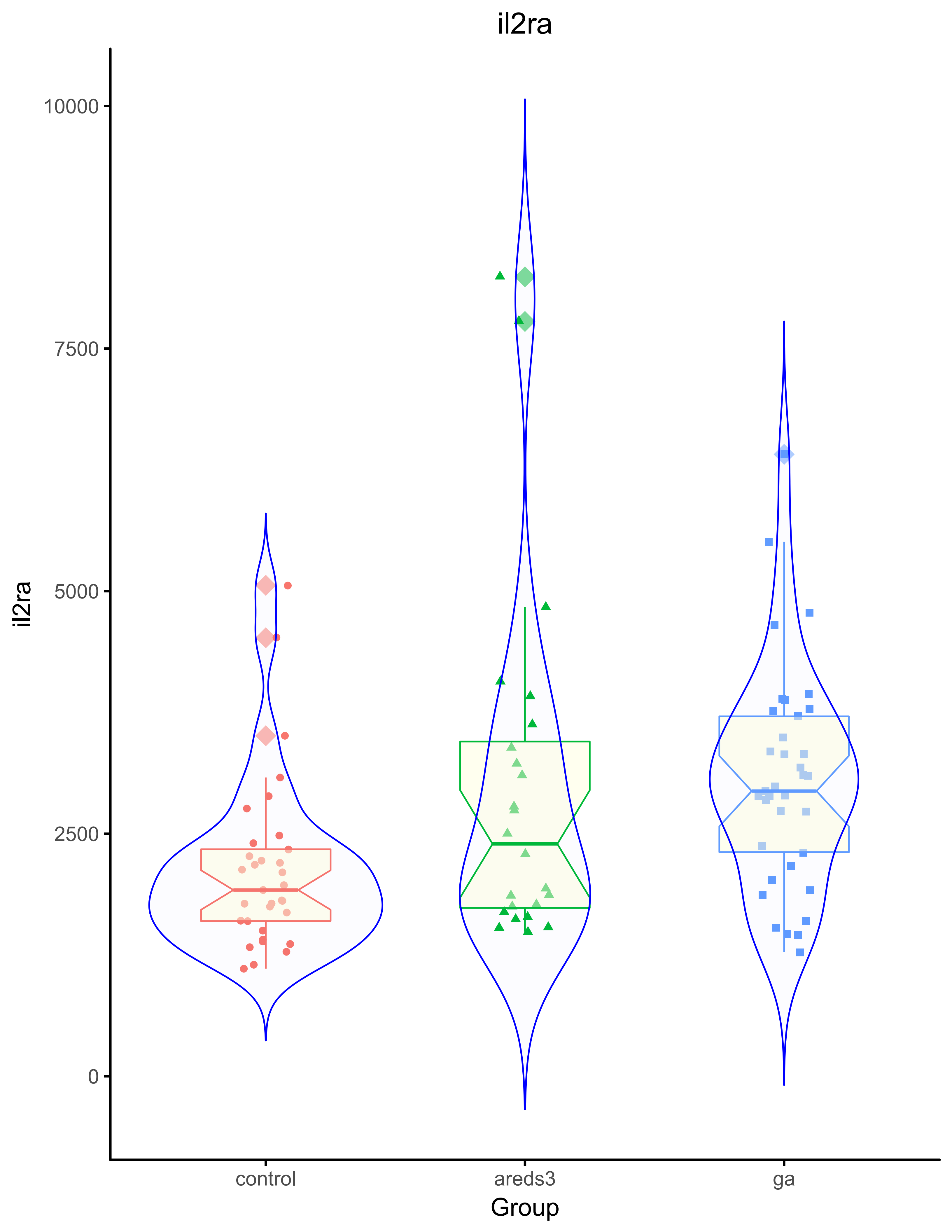
il2ra
10000
7500
5000
2500
0
il2ra
control
areds3
ga
Group

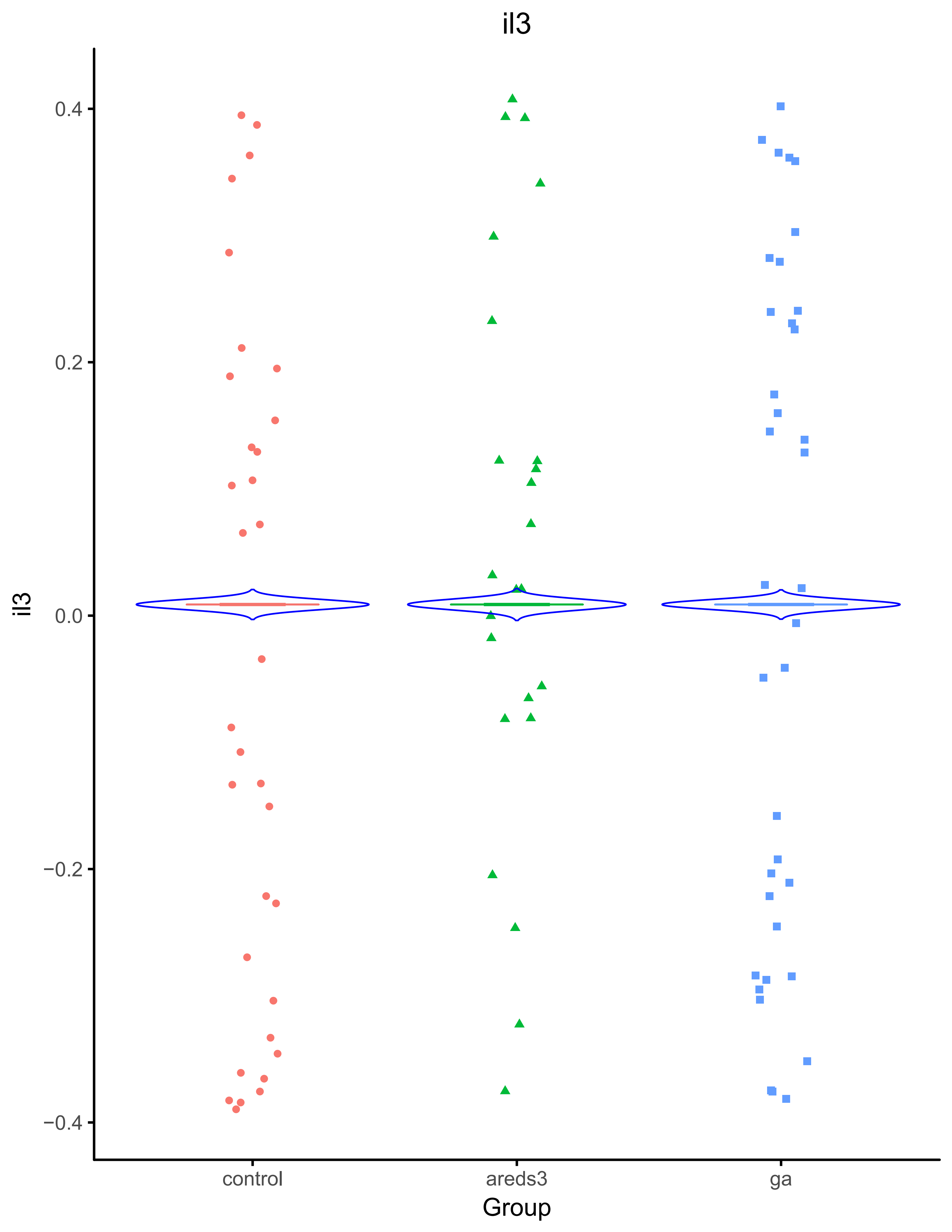

il3
0.4
0.2
0.0
−0.2
−0.4
il3
control
areds3
ga
Group

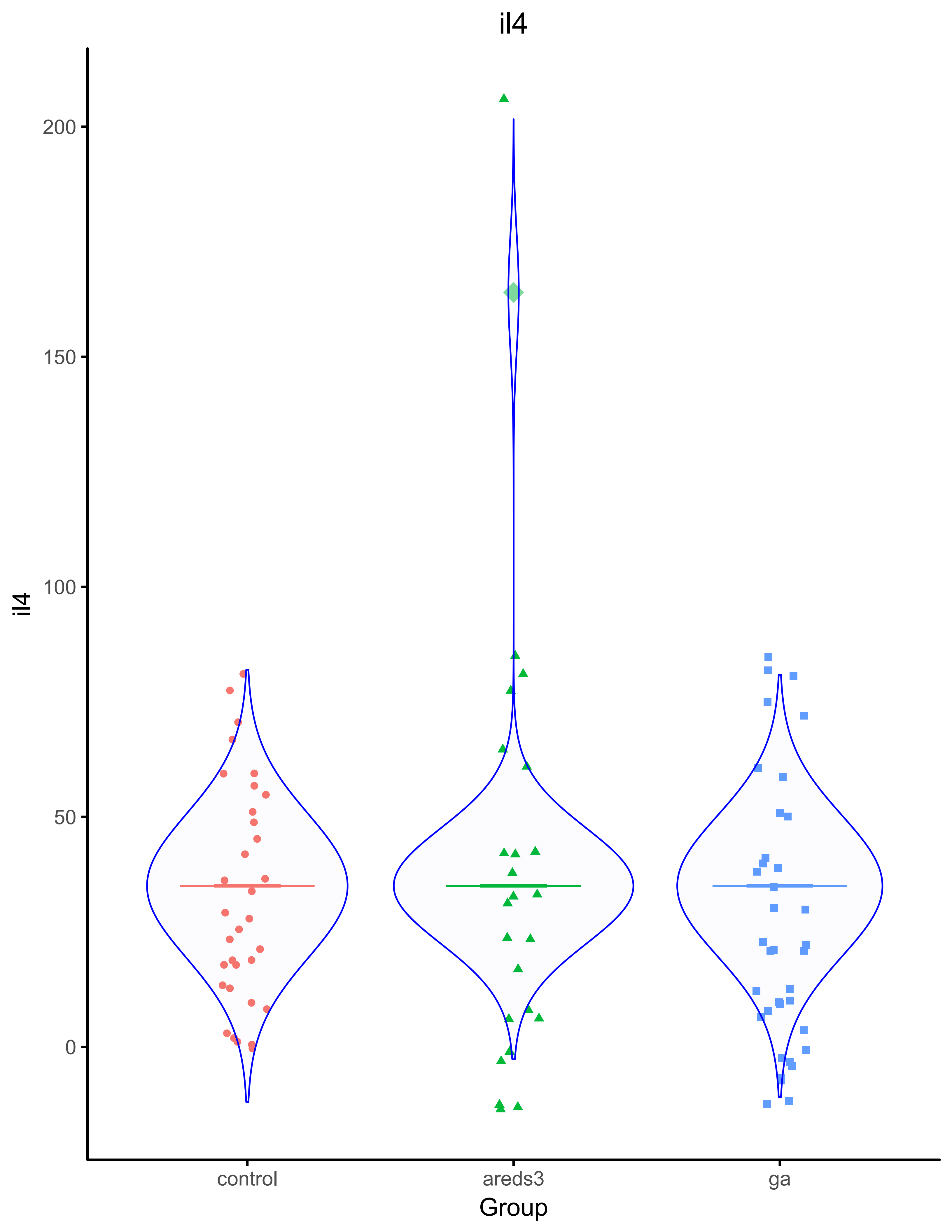

il4
200
150
100
50
0
il4
control
areds3
ga
Group

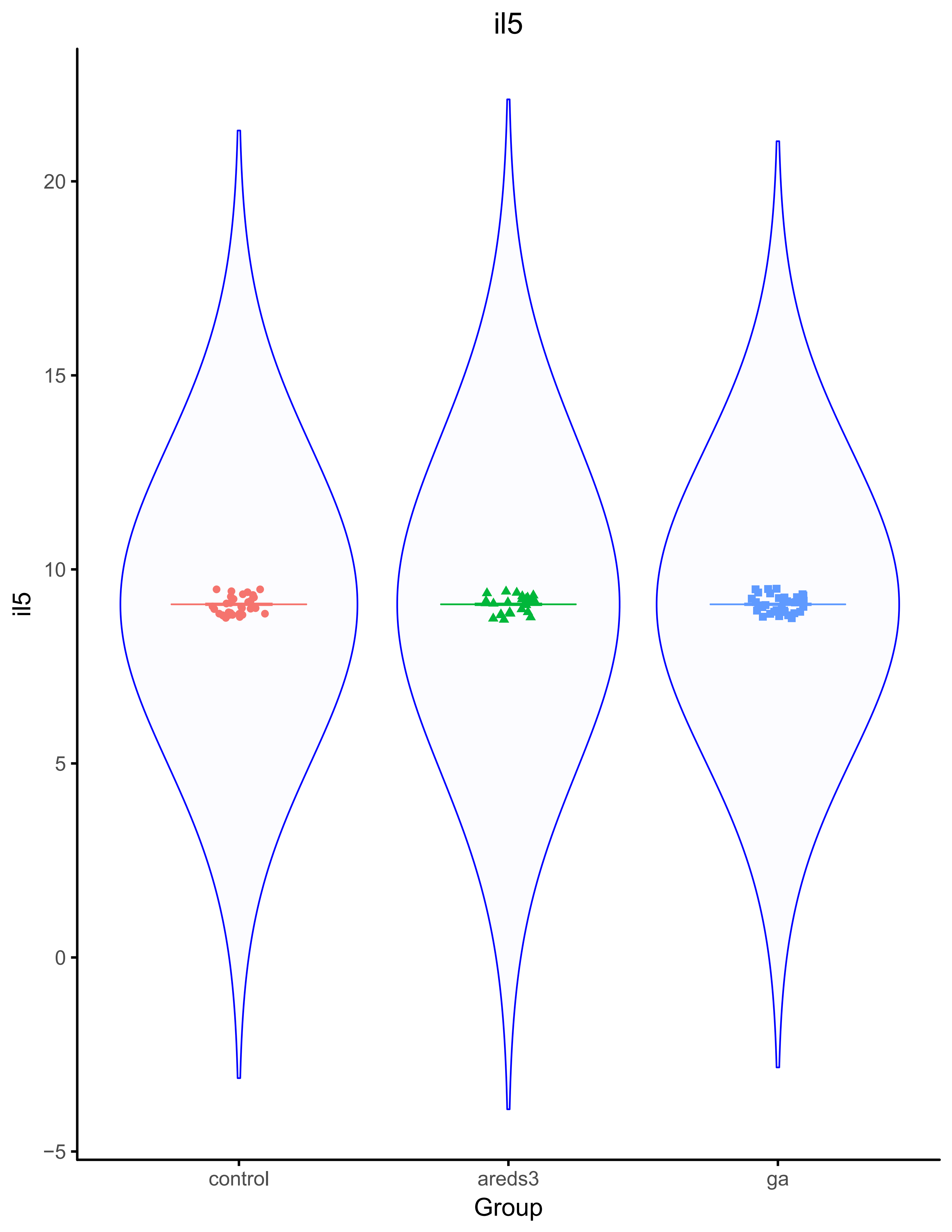

il5
20
15
10
5
0
−5
il5
control
areds3
ga
Group

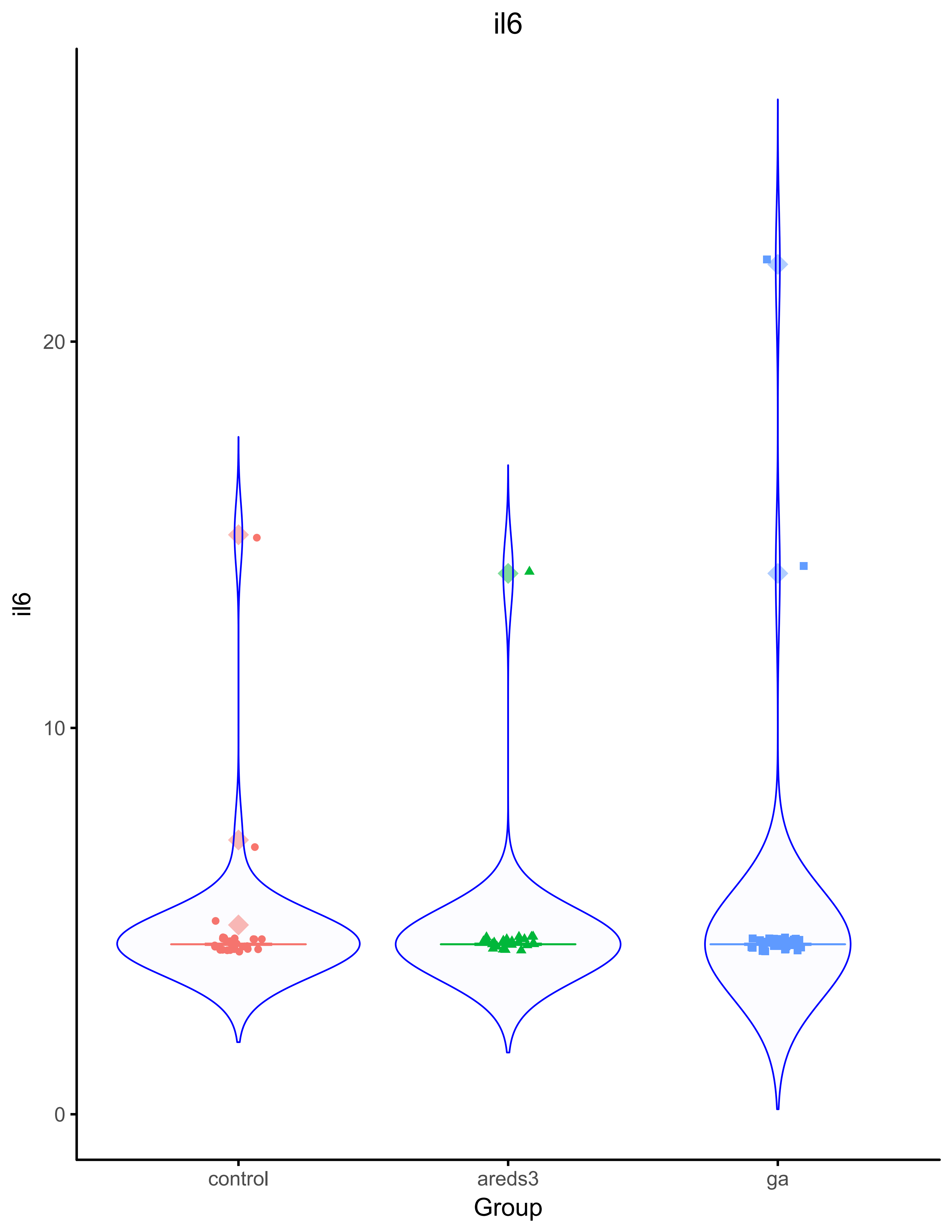
il6
il6
20
10
0
control
areds3
ga
Group

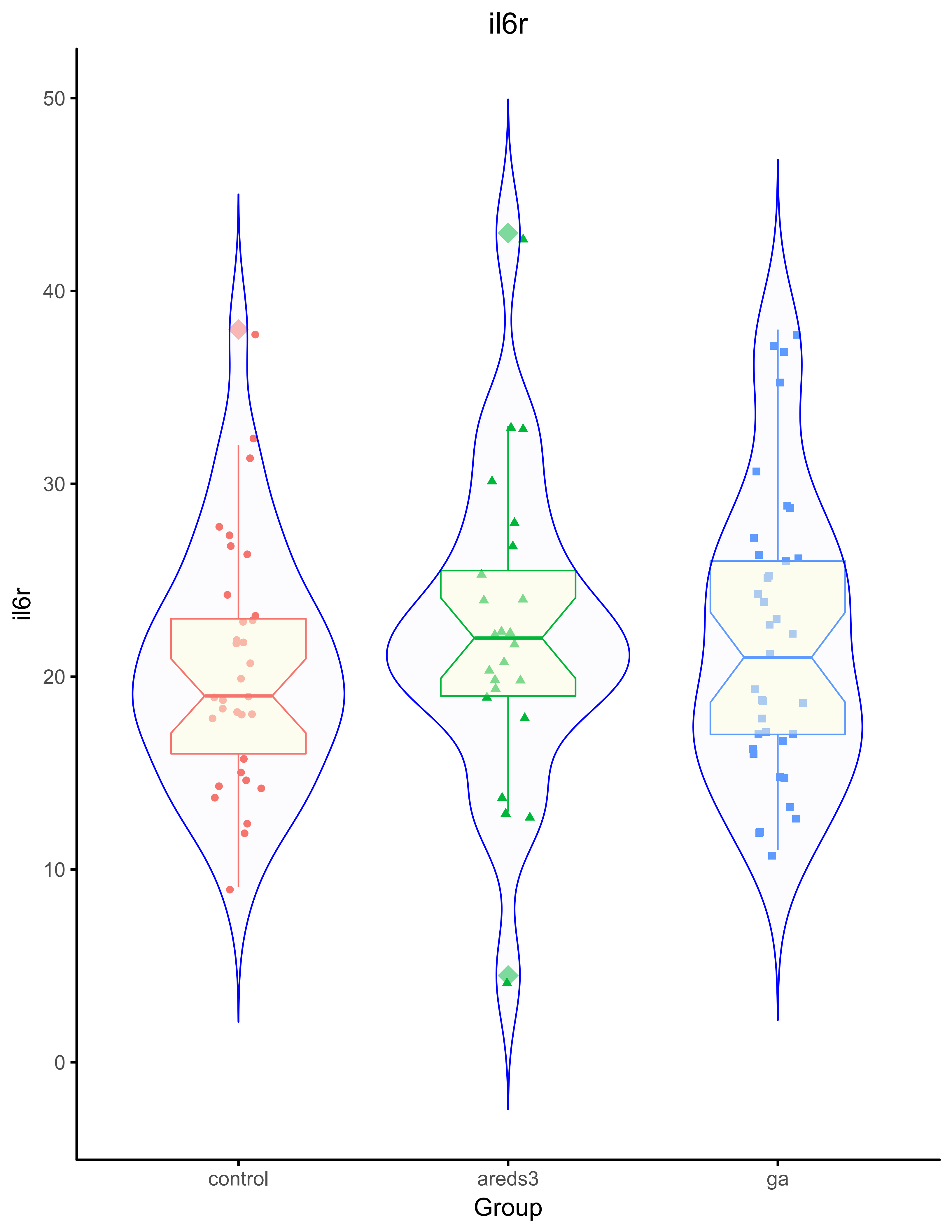
il6r
50
40
30
20
10
0
il6r
control
areds3
ga
Group

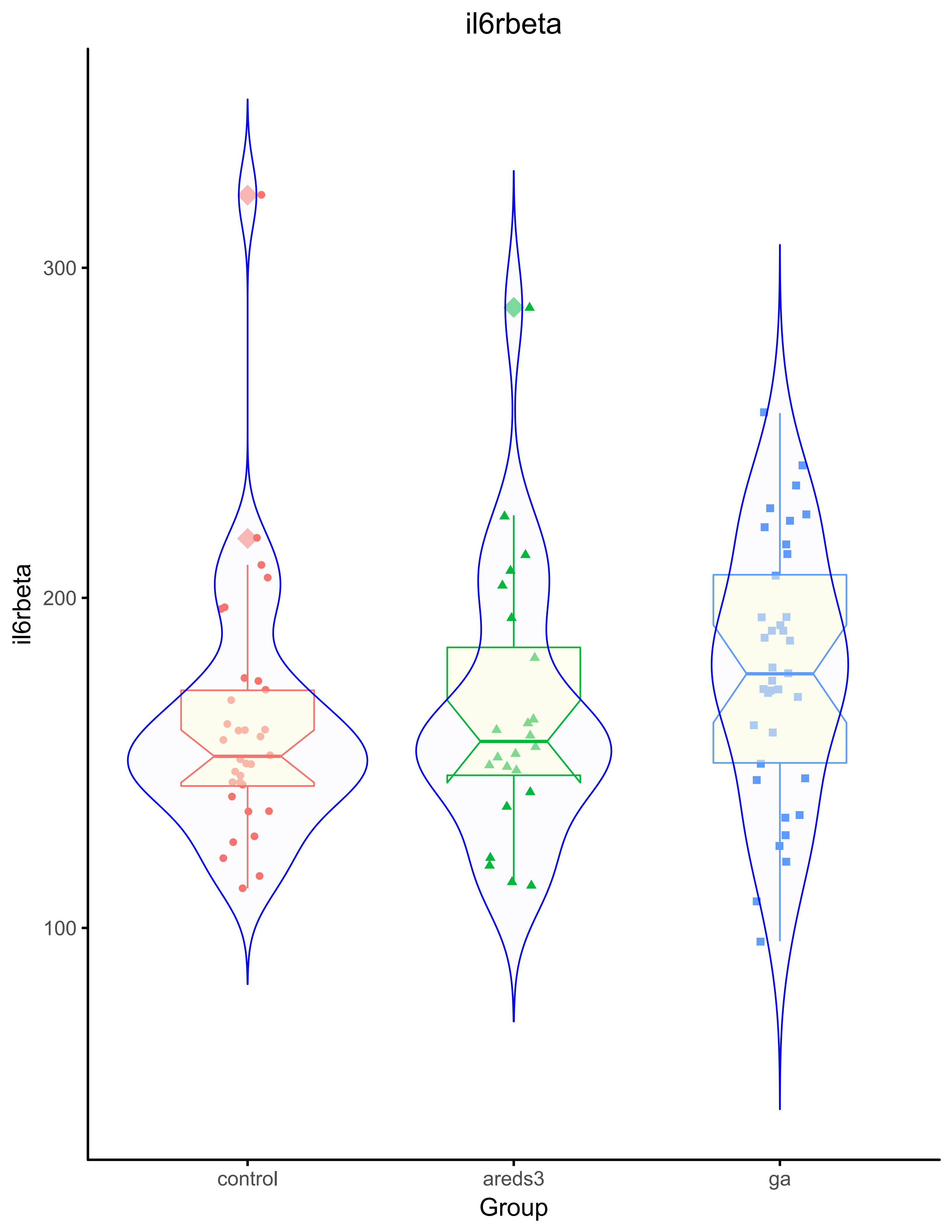
il6rbeta
300
200
100
il6rbeta
control
areds3
ga
Group

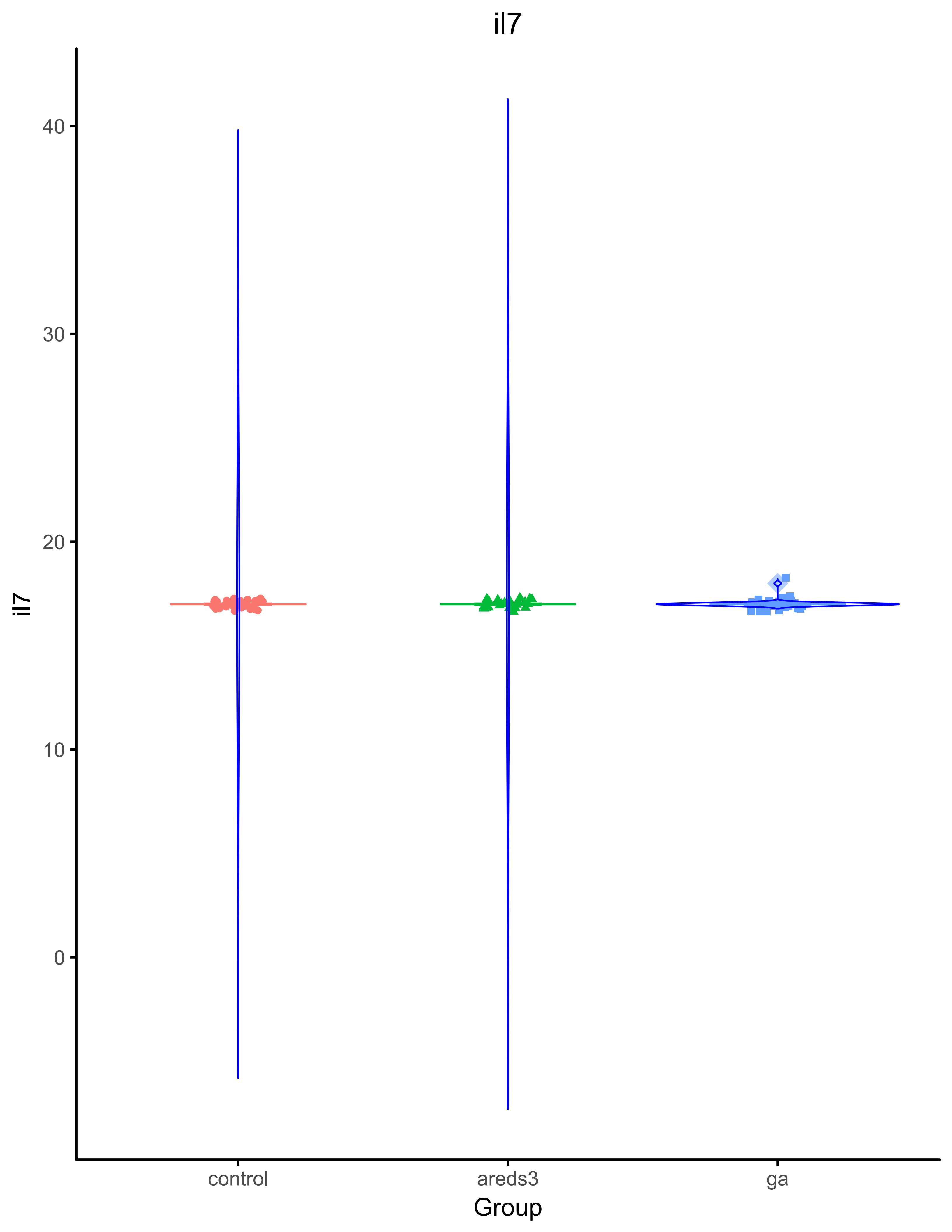

il7
il7
40
30
20
10
0
control
areds3
ga
Group

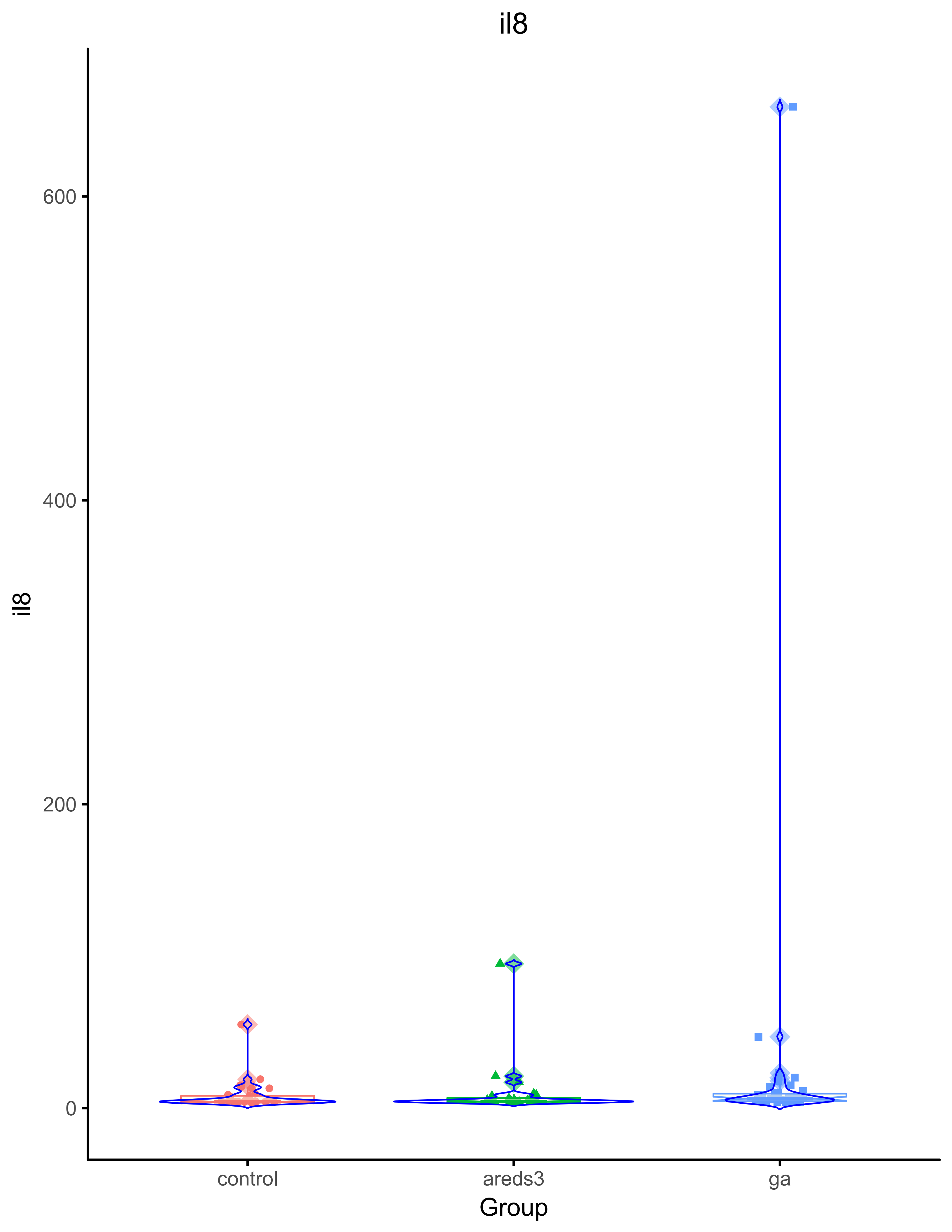

il8
600
400
200
0
il8
control
areds3
ga
Group

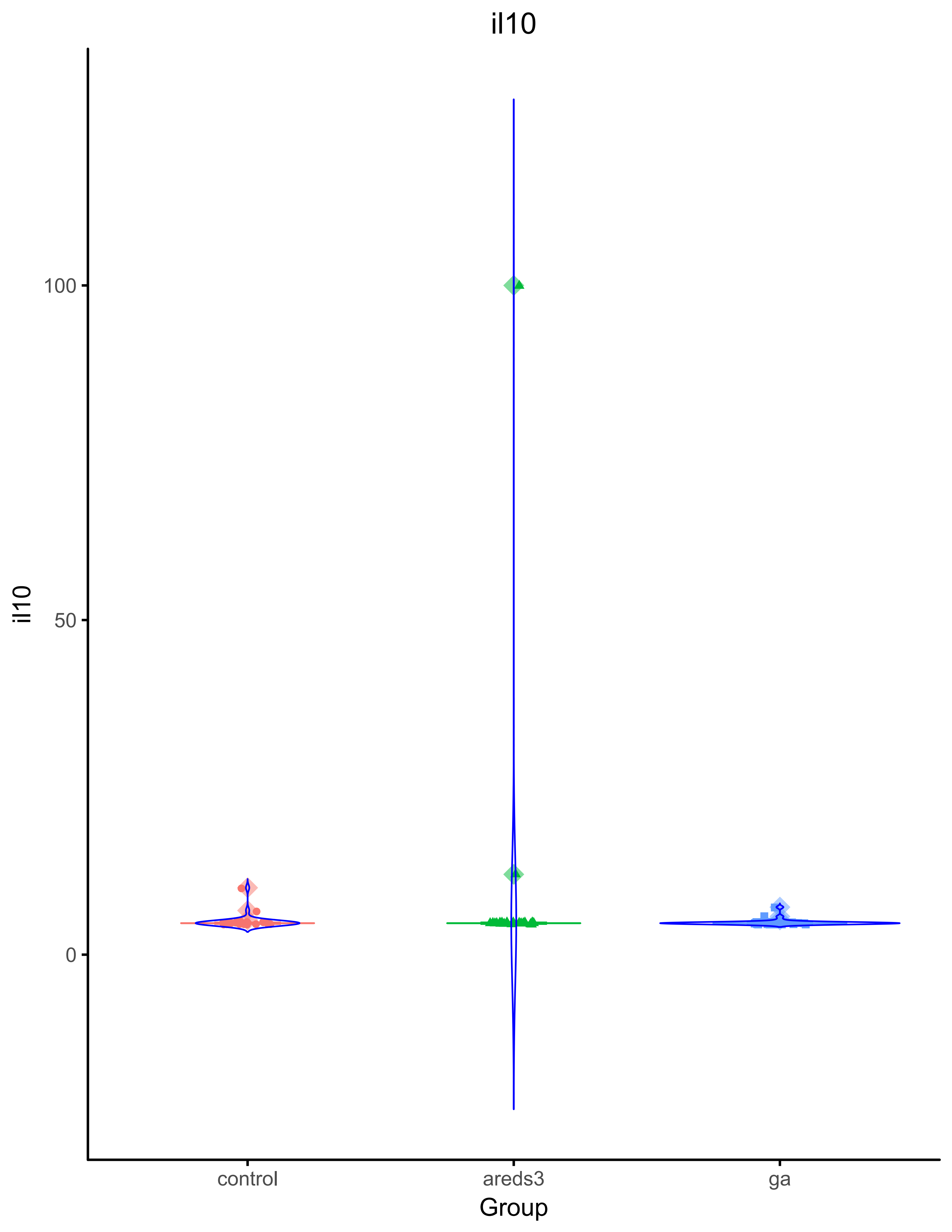
il10
100
50
0
il10
control
areds3
ga
Group

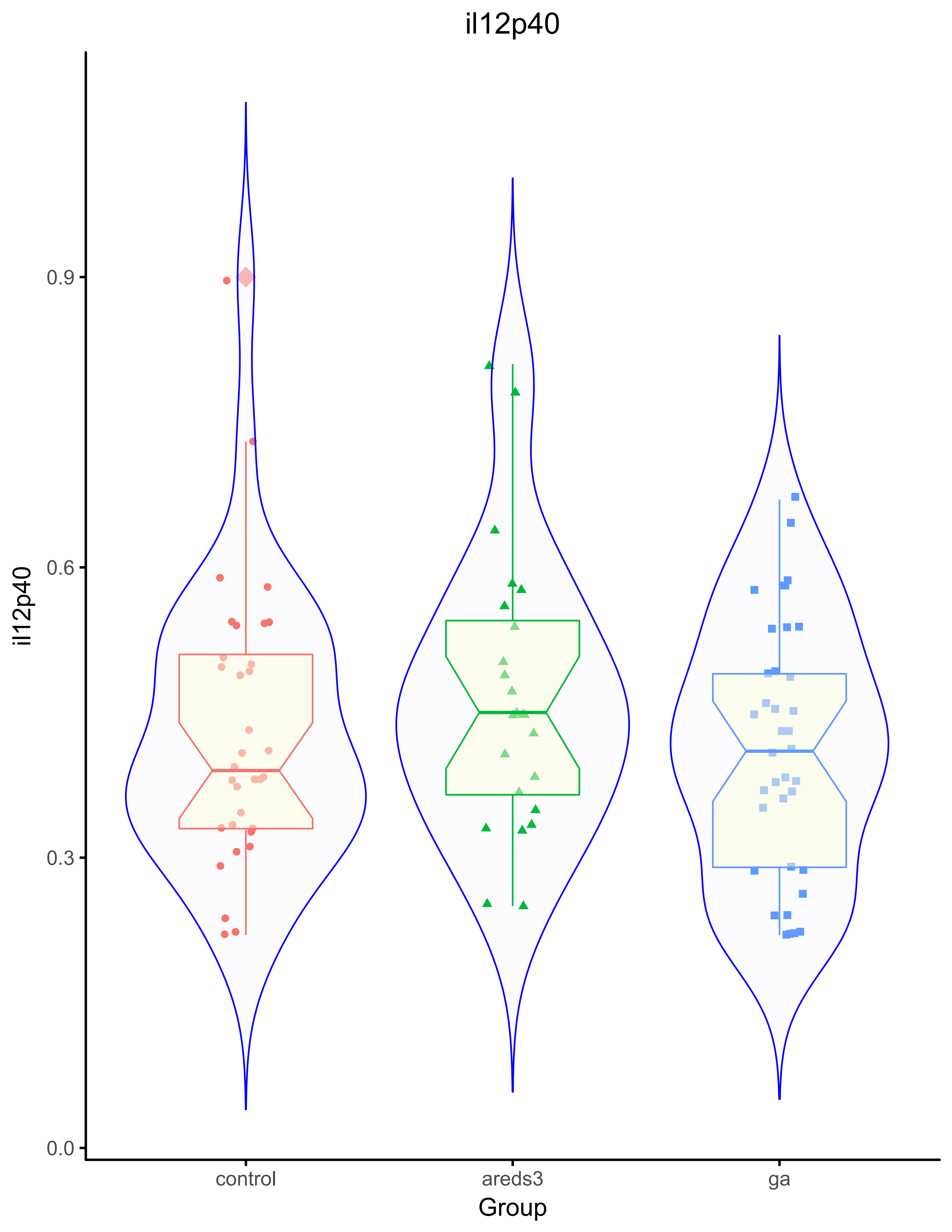

il12p40
il12p40
0.9
0.6
0.3
0.0
control
areds3
ga
Group

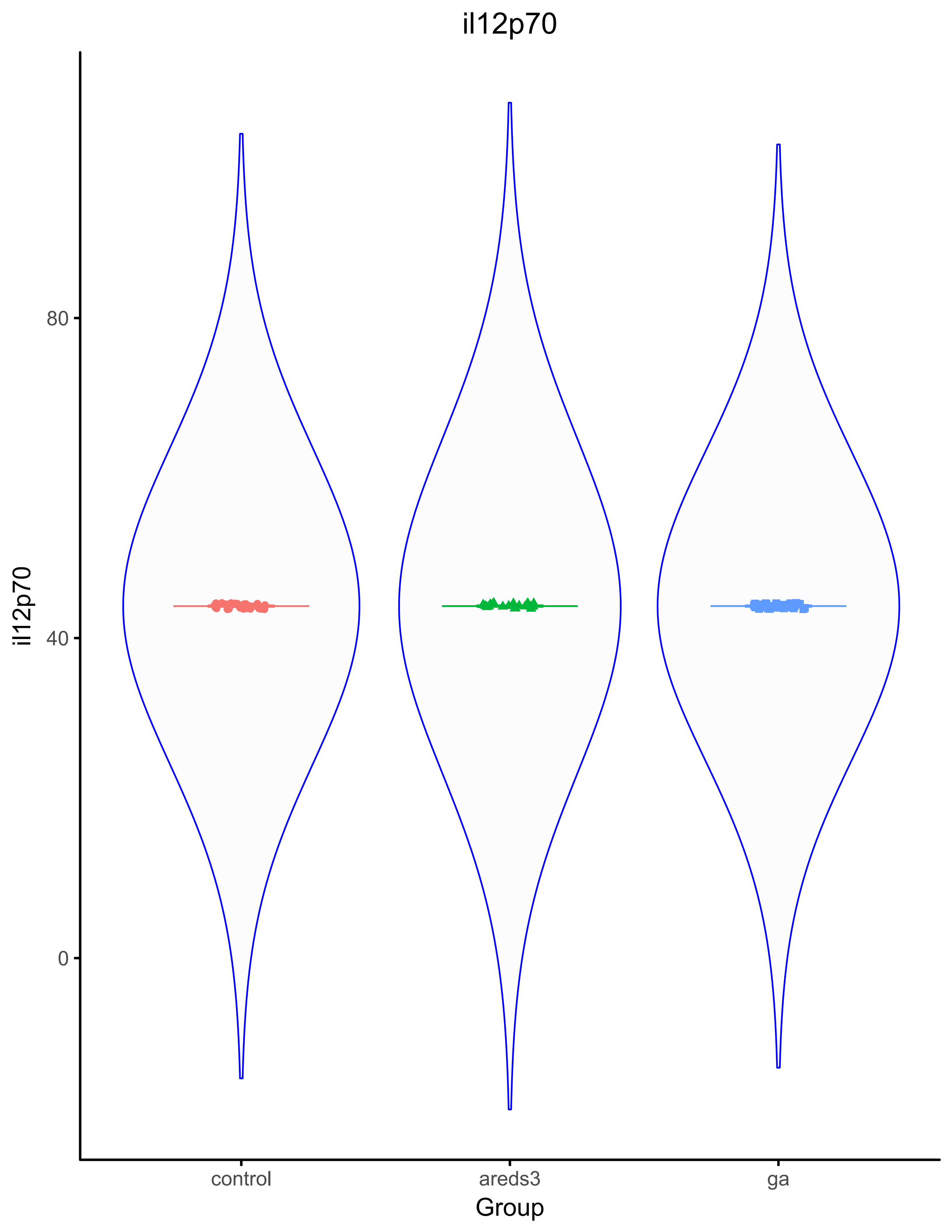

il12p70
80
40
0
il12p70
control
areds3
ga
Group

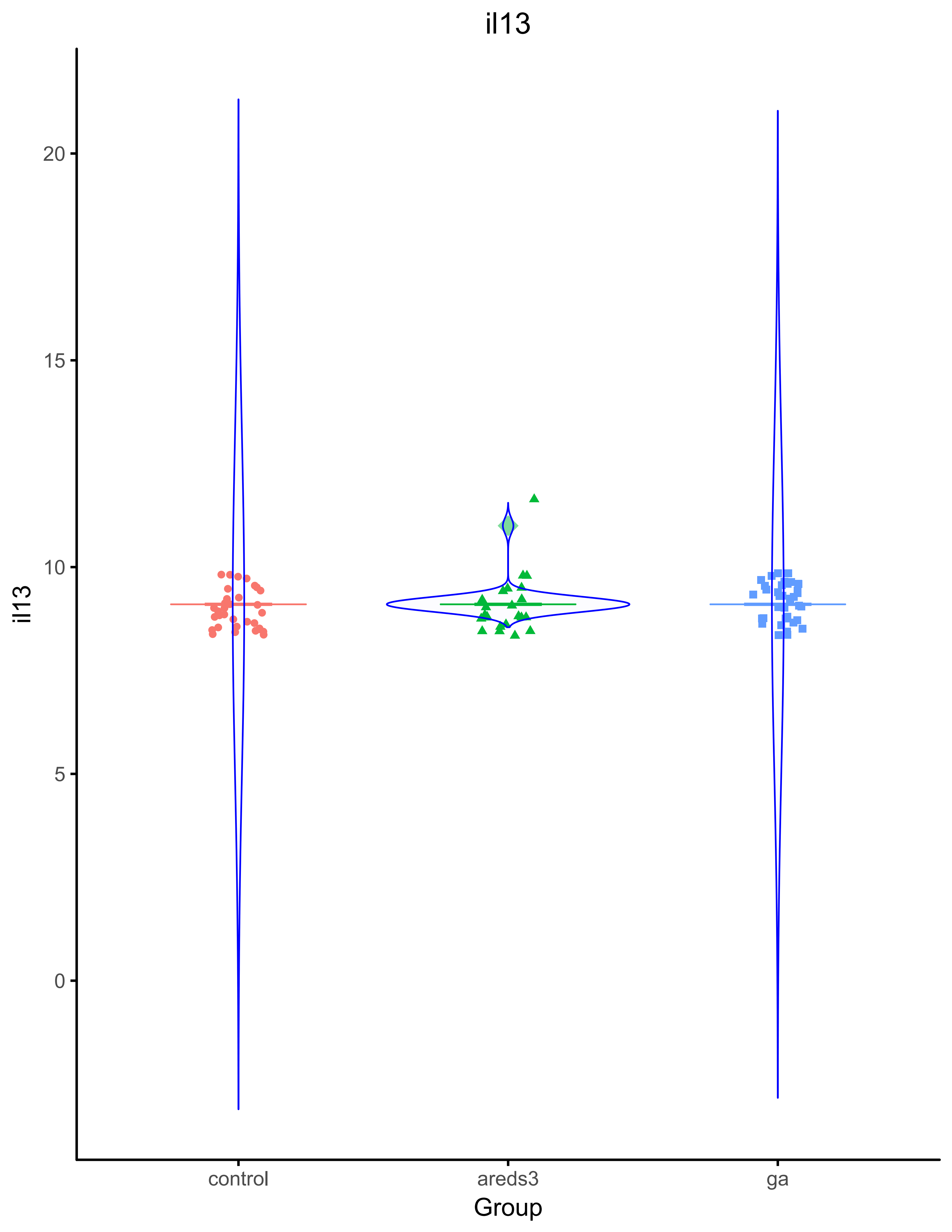
il13
il13
20
15
10
5
0
control
areds3
ga
Group

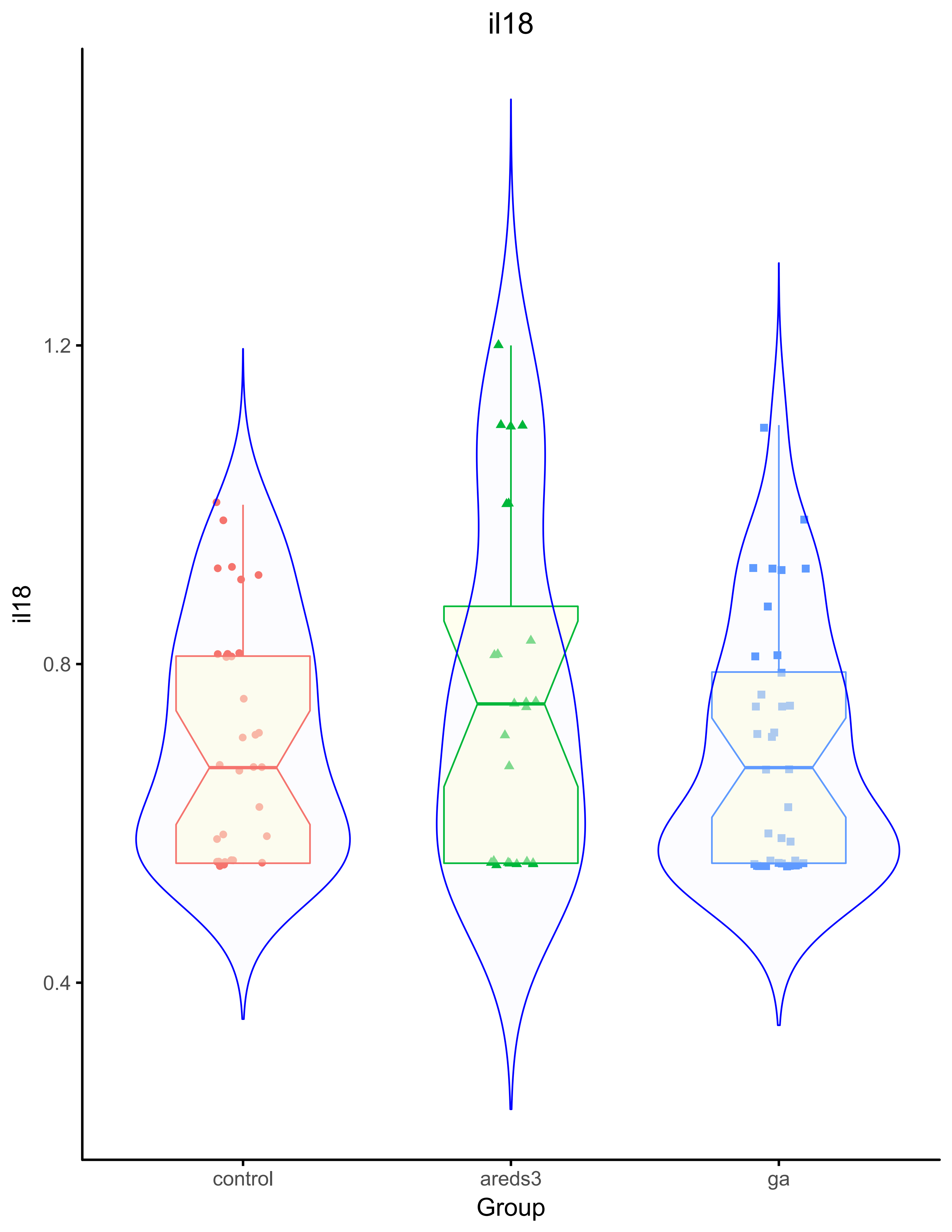

il18
1.2
0.8
0.4
il18
control
areds3
ga
Group

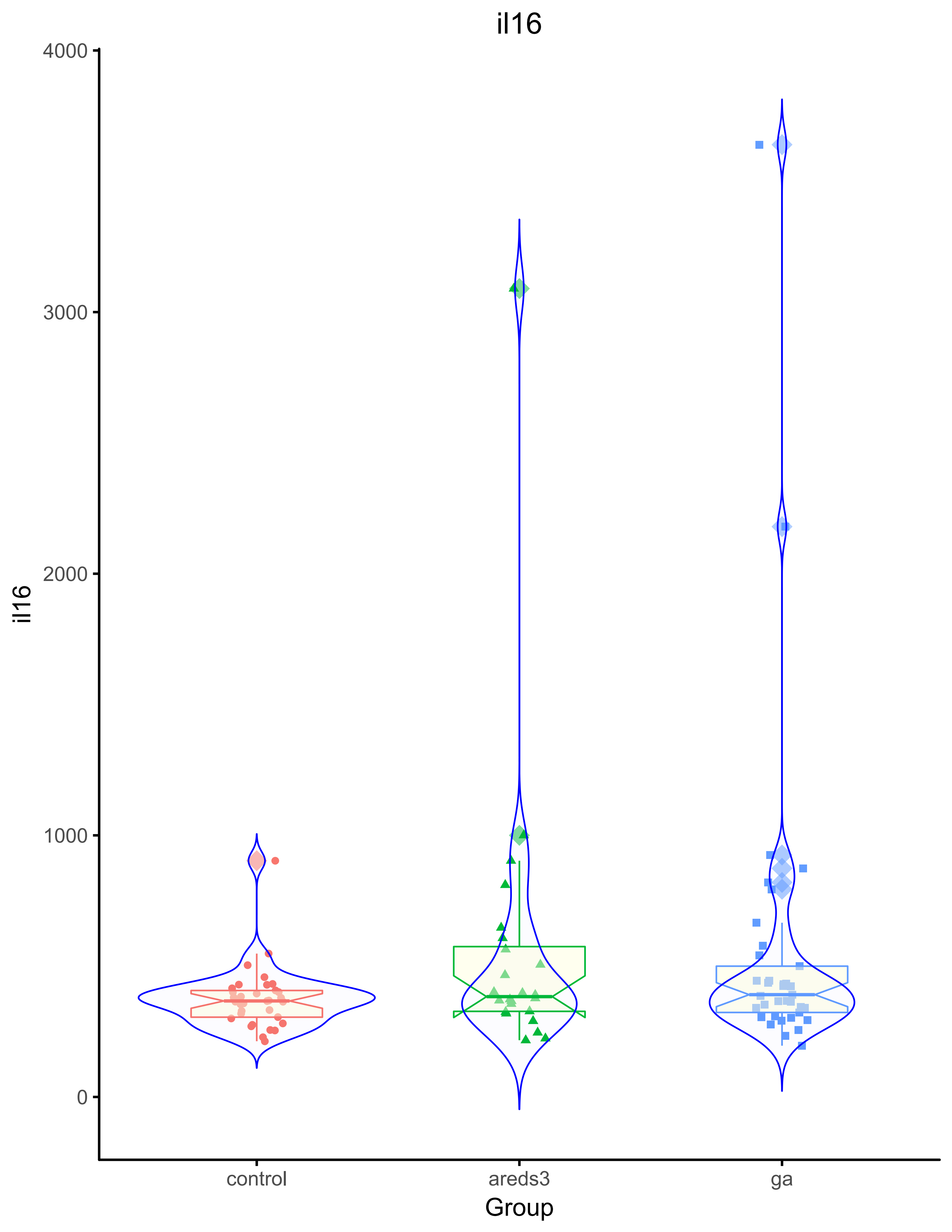

il16
4000
3000
2000
1000
0
il16
control
areds3
ga
Group

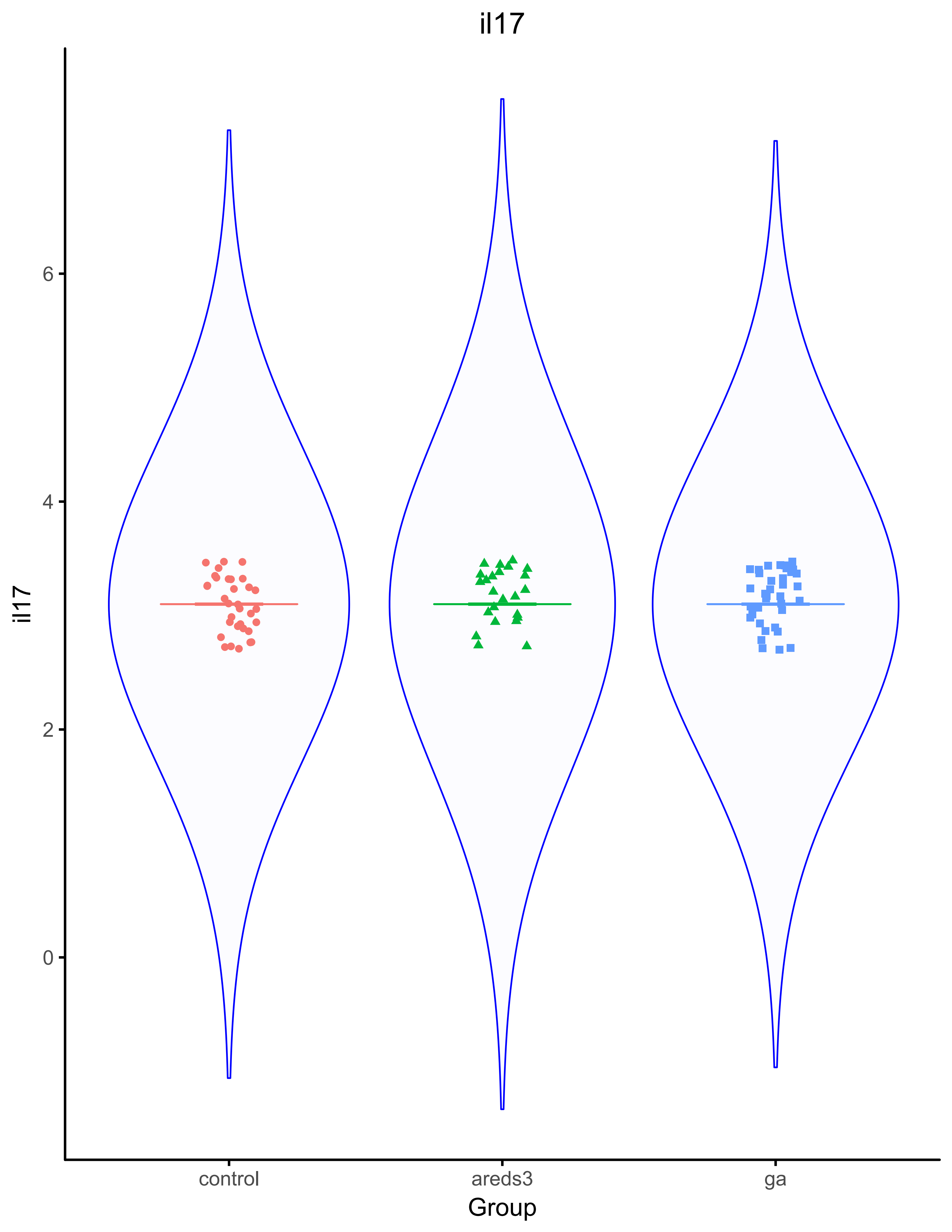

il17
il17
6
4
2
0
control
areds3
ga
Group

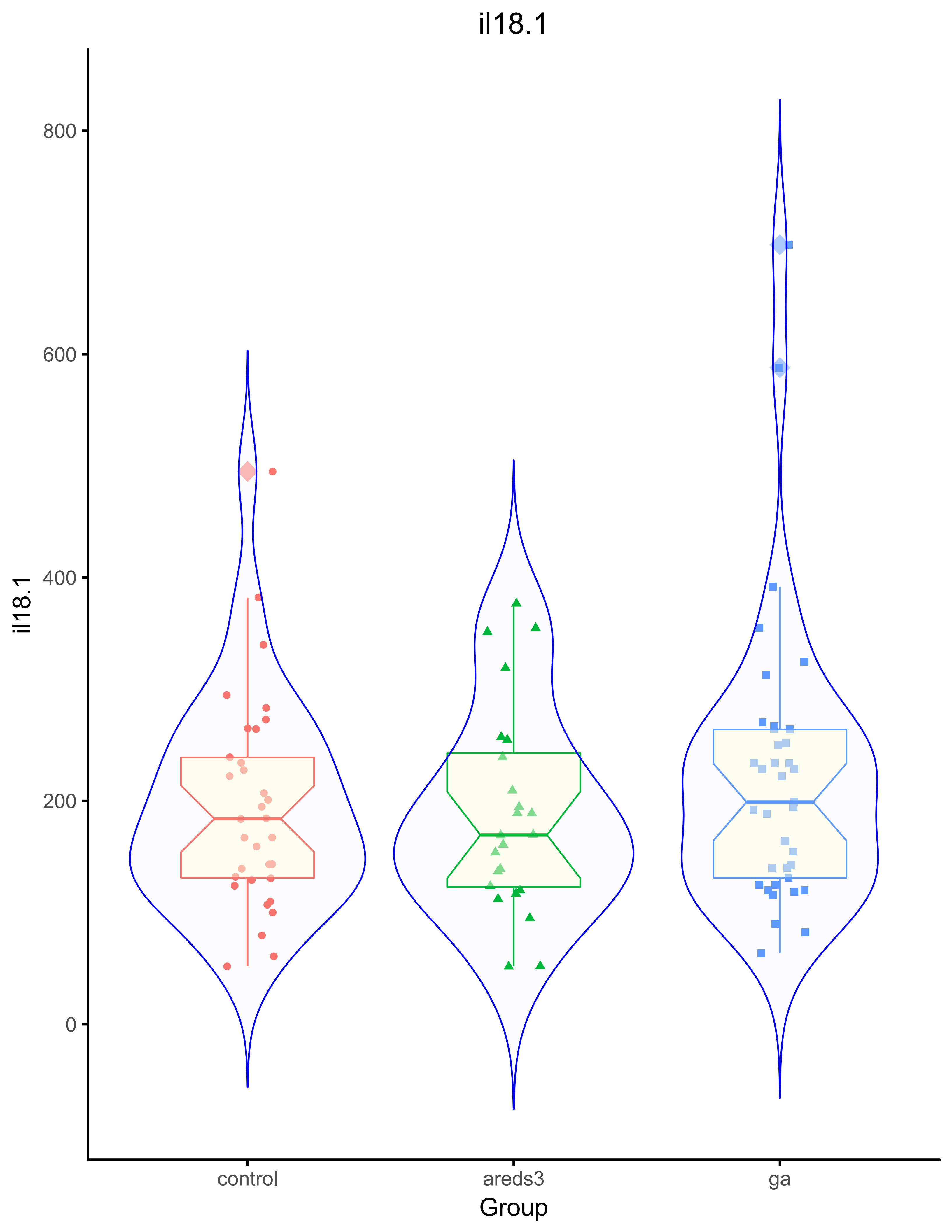
il18.1
800
600
400
200
0
il18.1
control
areds3
ga
Group

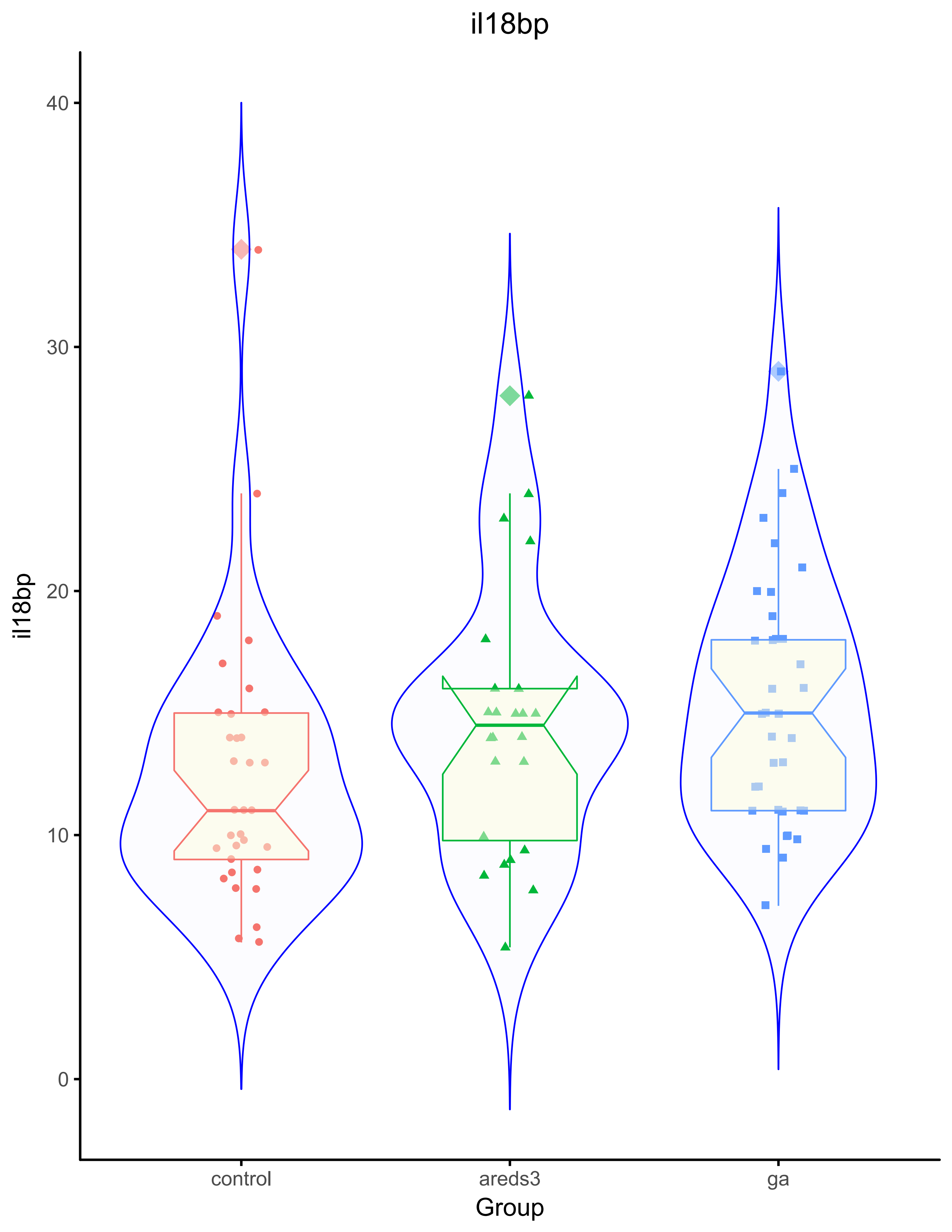
il18bp
il18bp
40
30
20
10
0
control
areds3
ga
Group

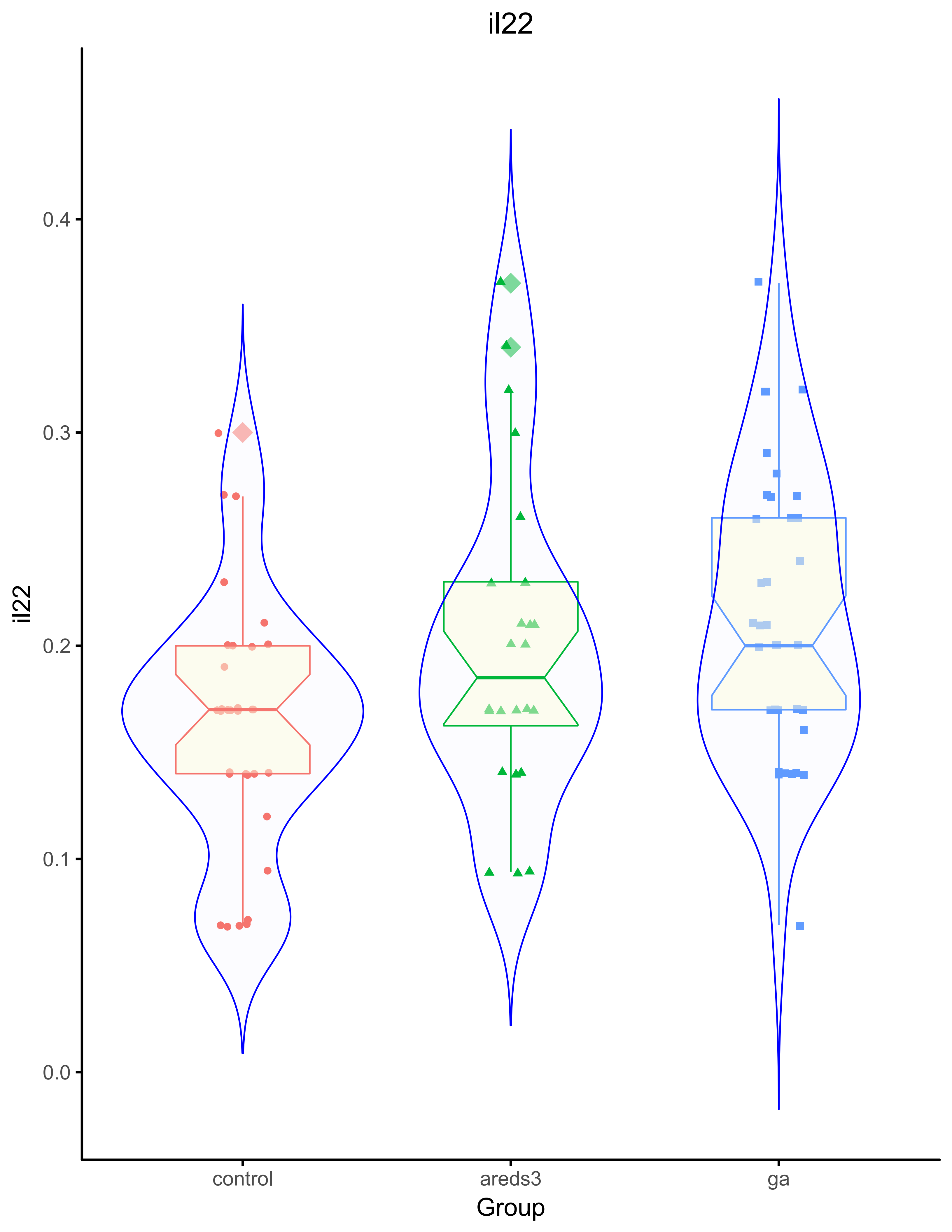

il22
0.0
0.1
0.2
0.3
0.4
il22
control
areds3
ga
Group

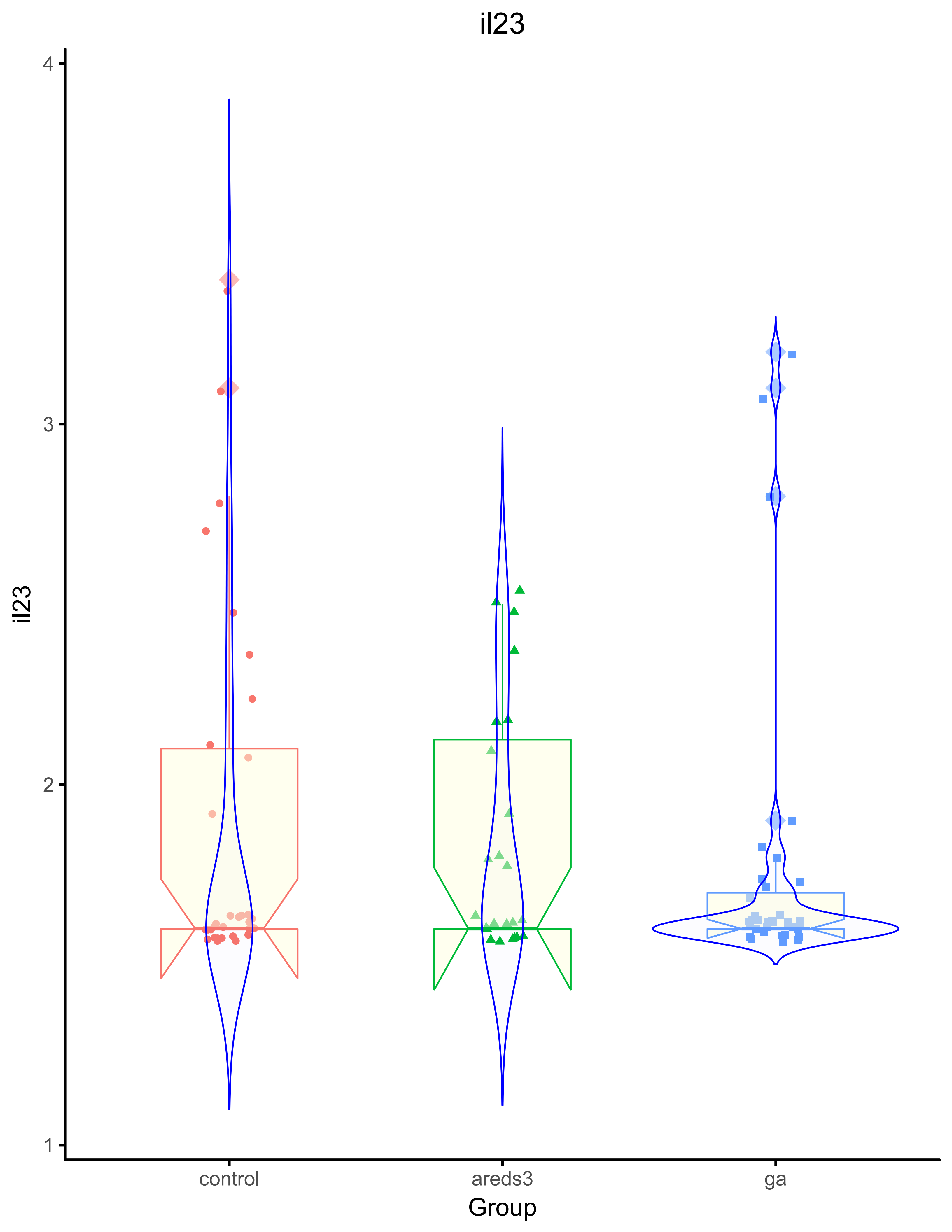

il23
4
3
2
1
il23
control
areds3
ga
Group

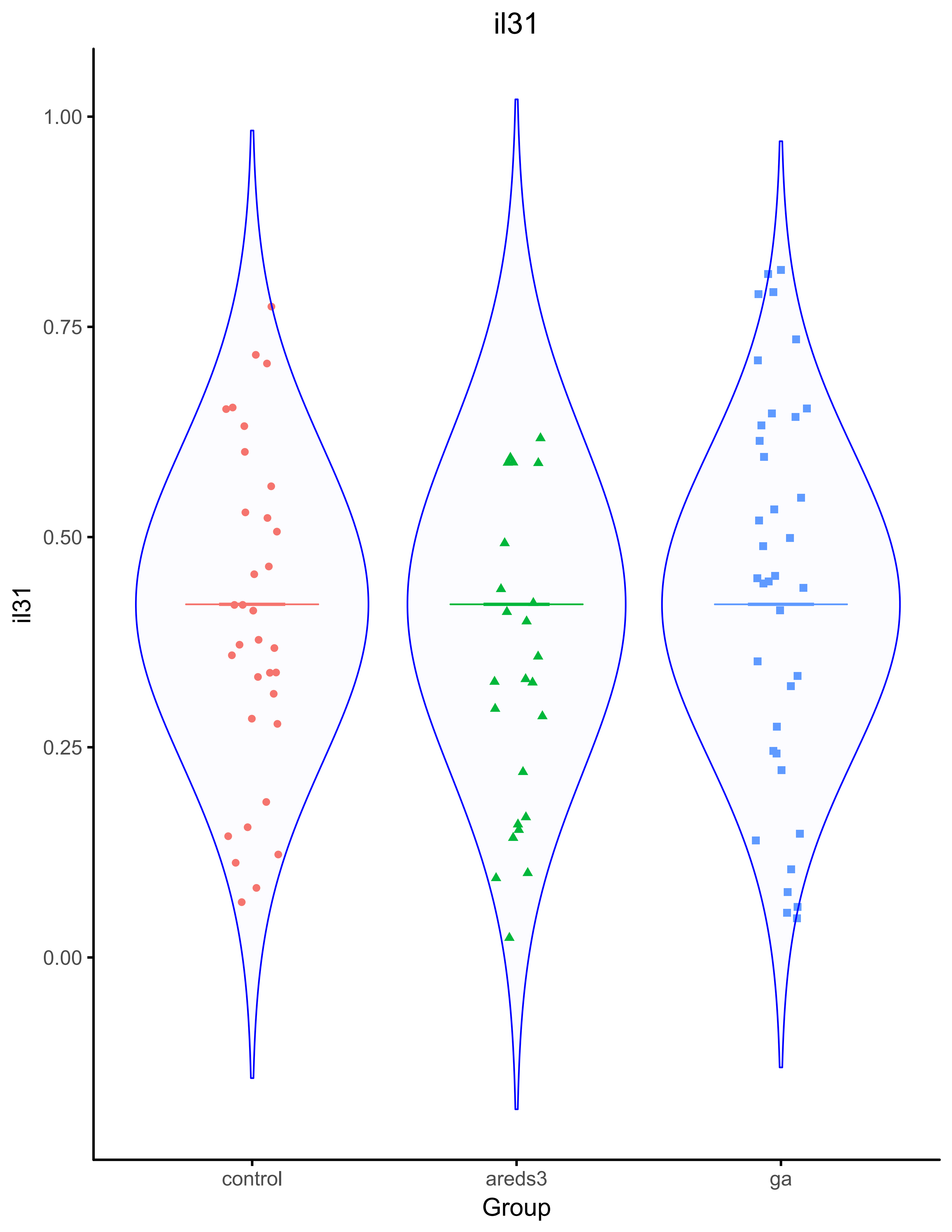
il31
1.00
0.75
0.50
0.25
0.00
il31
control
areds3
ga
Group

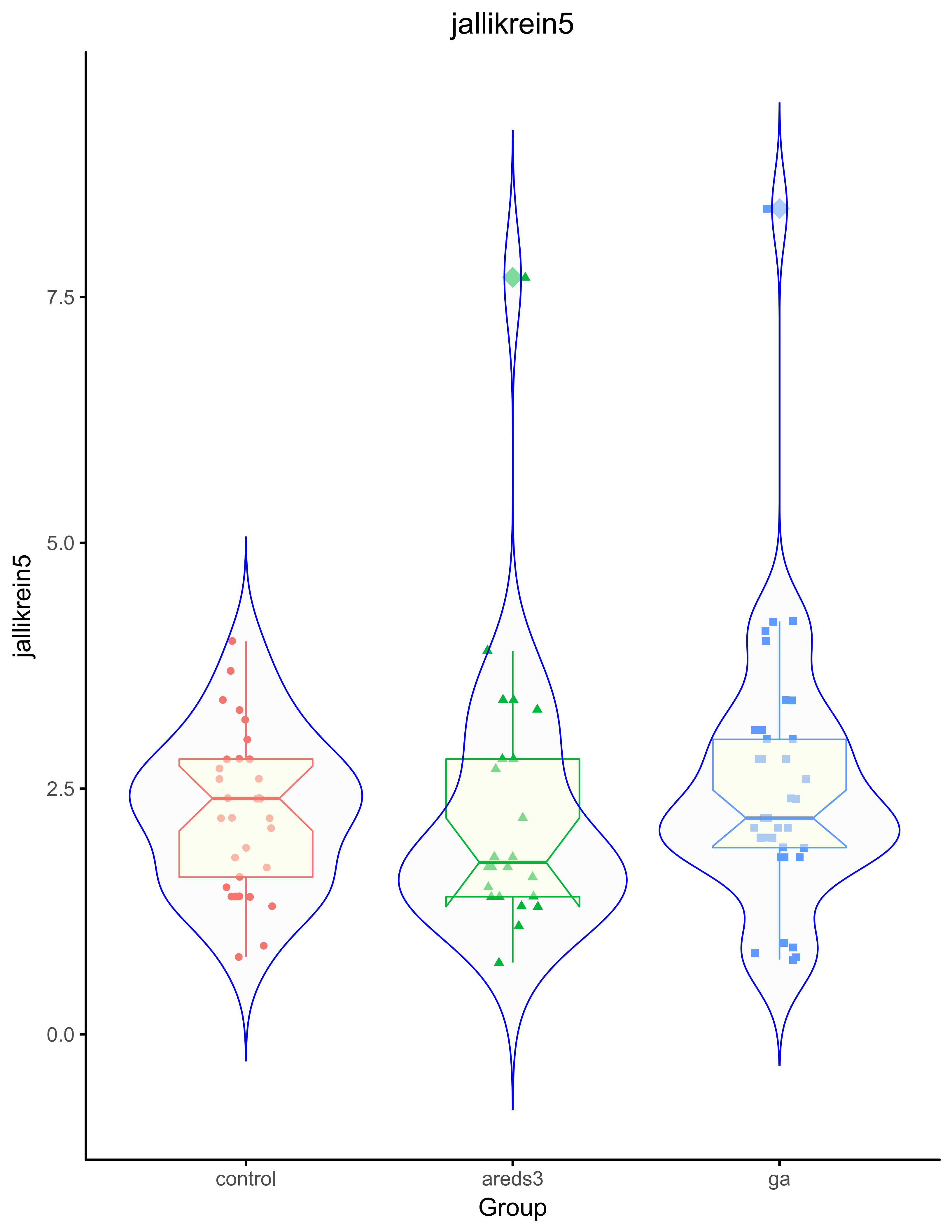

jallikrein5
0.0
2.5
5.0
7.5
jallikrein5
control
areds3
ga
Group

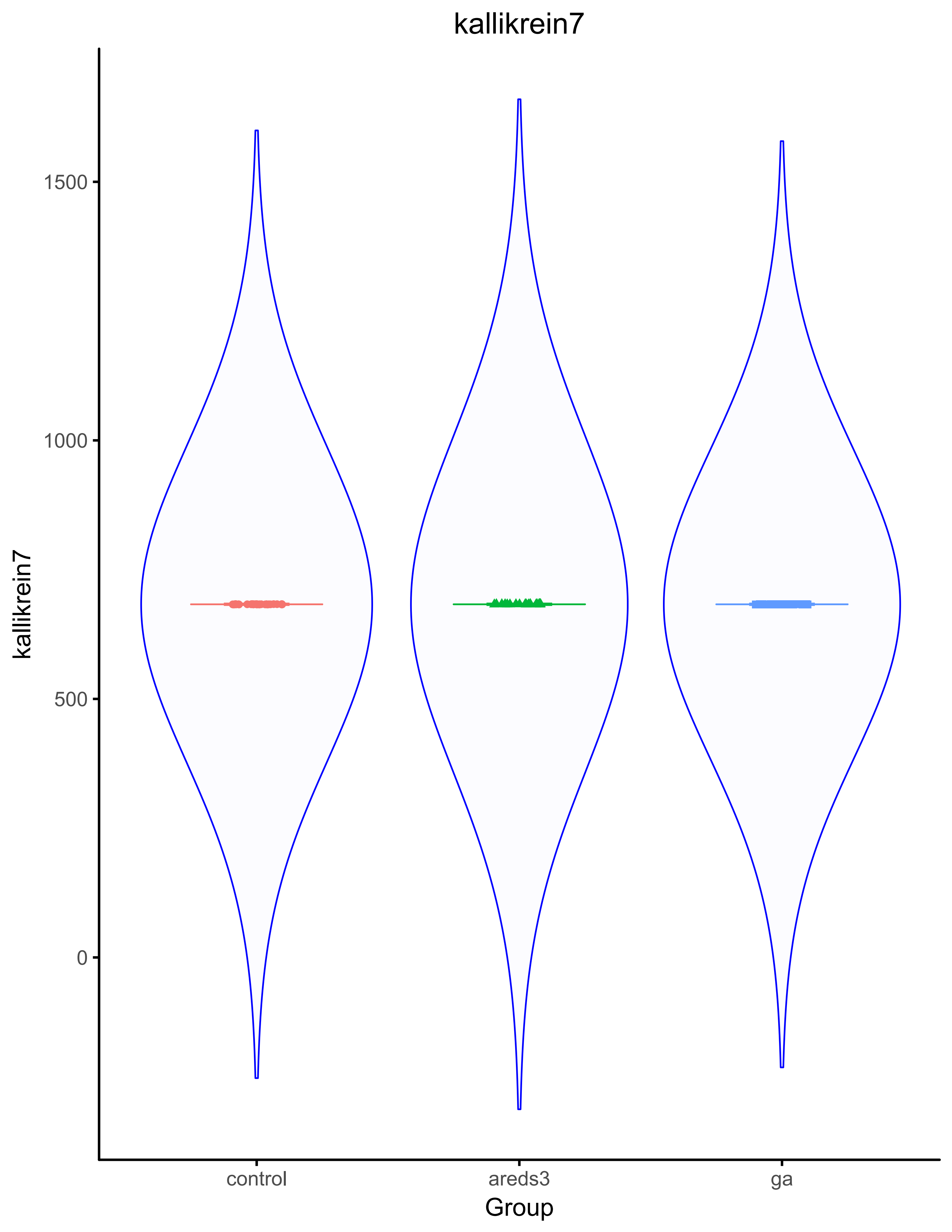
kallikrein7
1500
1000
500
0
kallikrein7
control
areds3
ga
Group

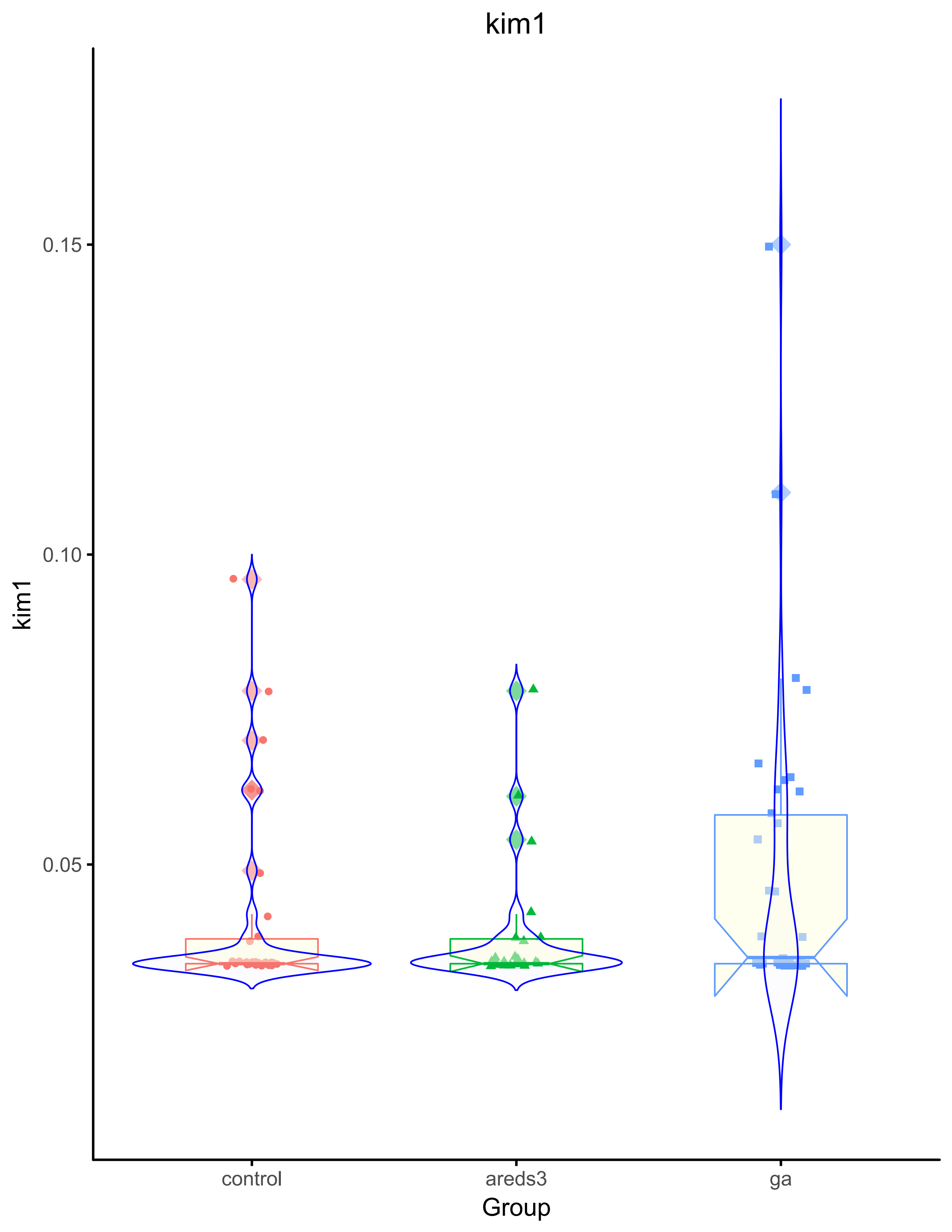

kim1
0.15
0.10
0.05
kim1
control
areds3
ga
Group

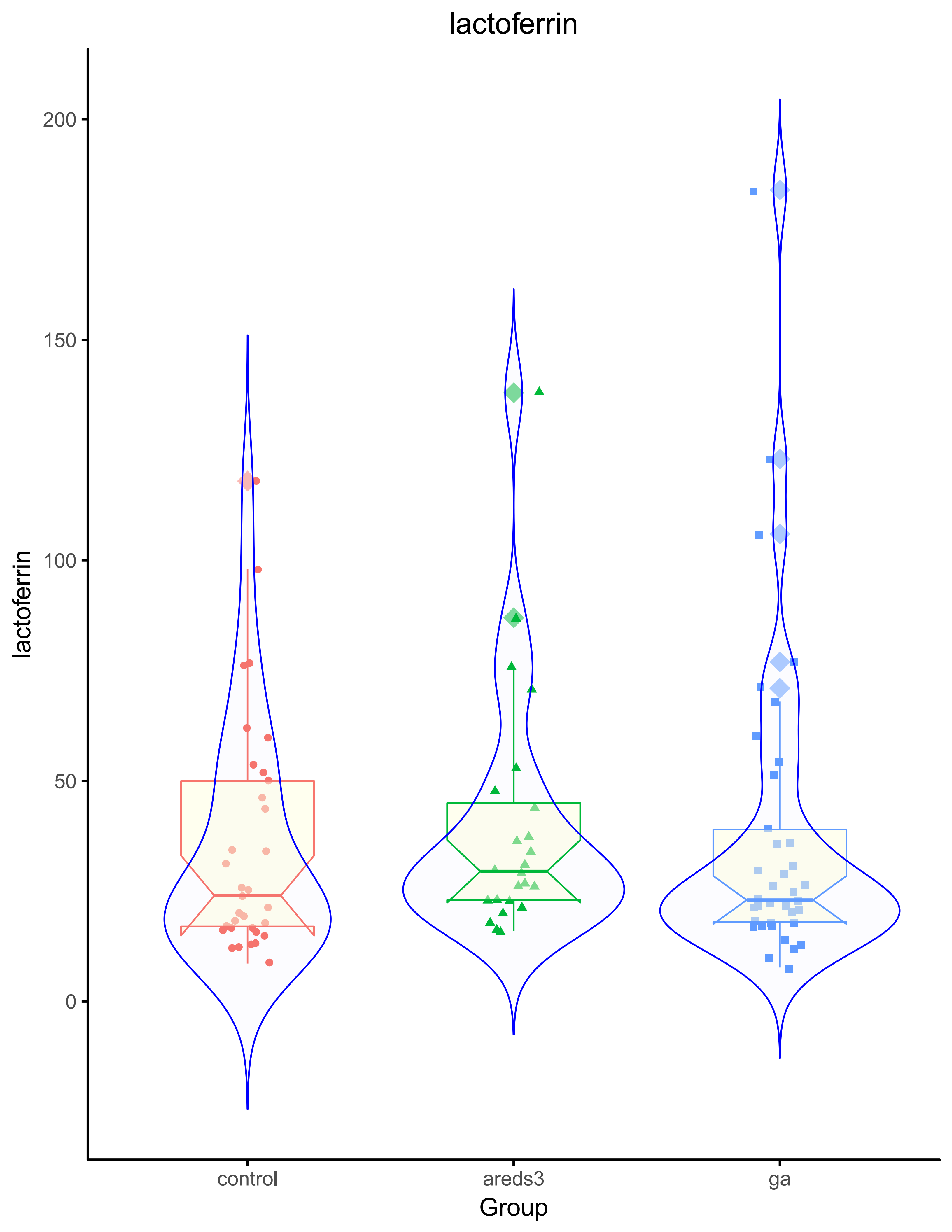

lactoferrin
200
150
100
50
0
lactoferrin
control
areds3
ga
Group

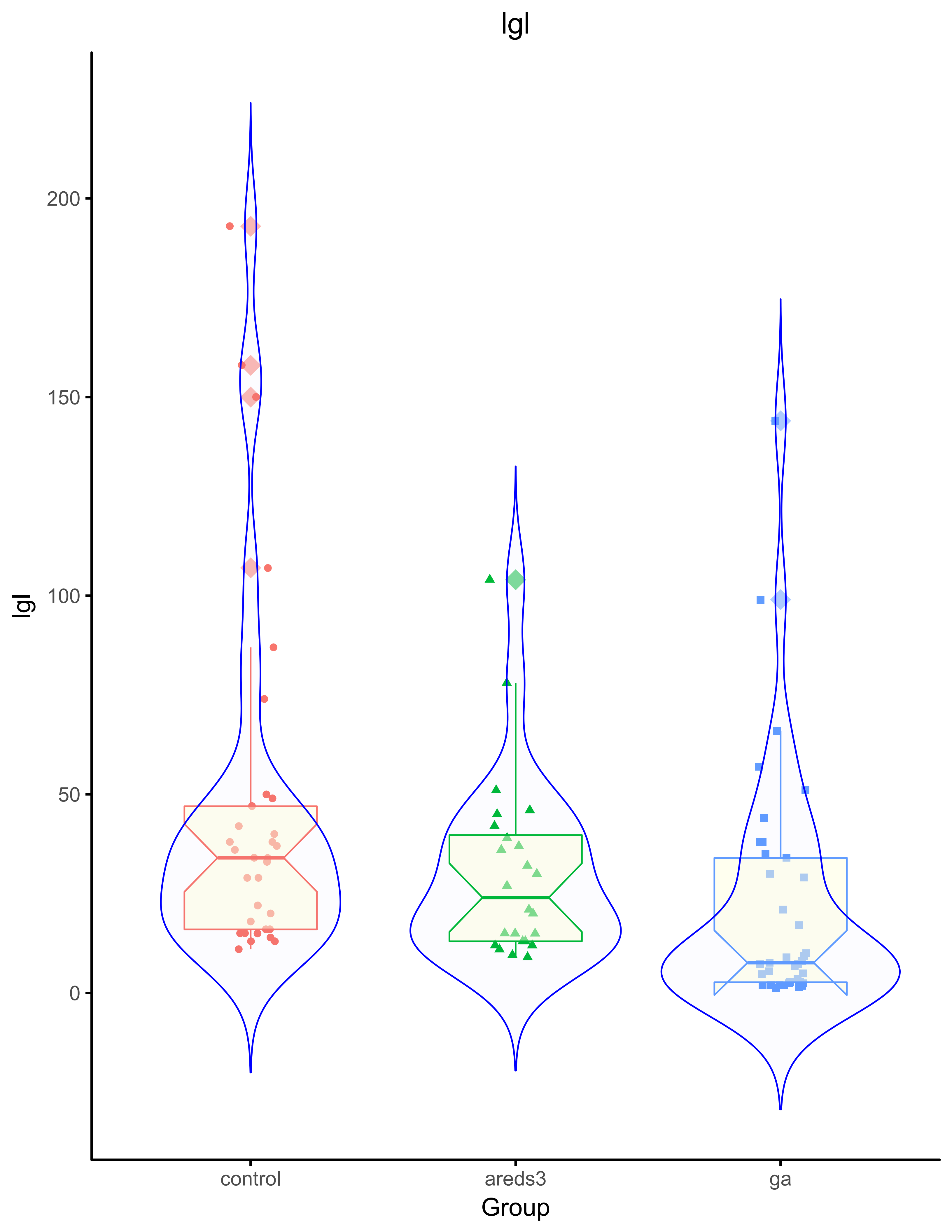

lgl
200
150
100
50
0
lgl
control
areds3
ga
Group

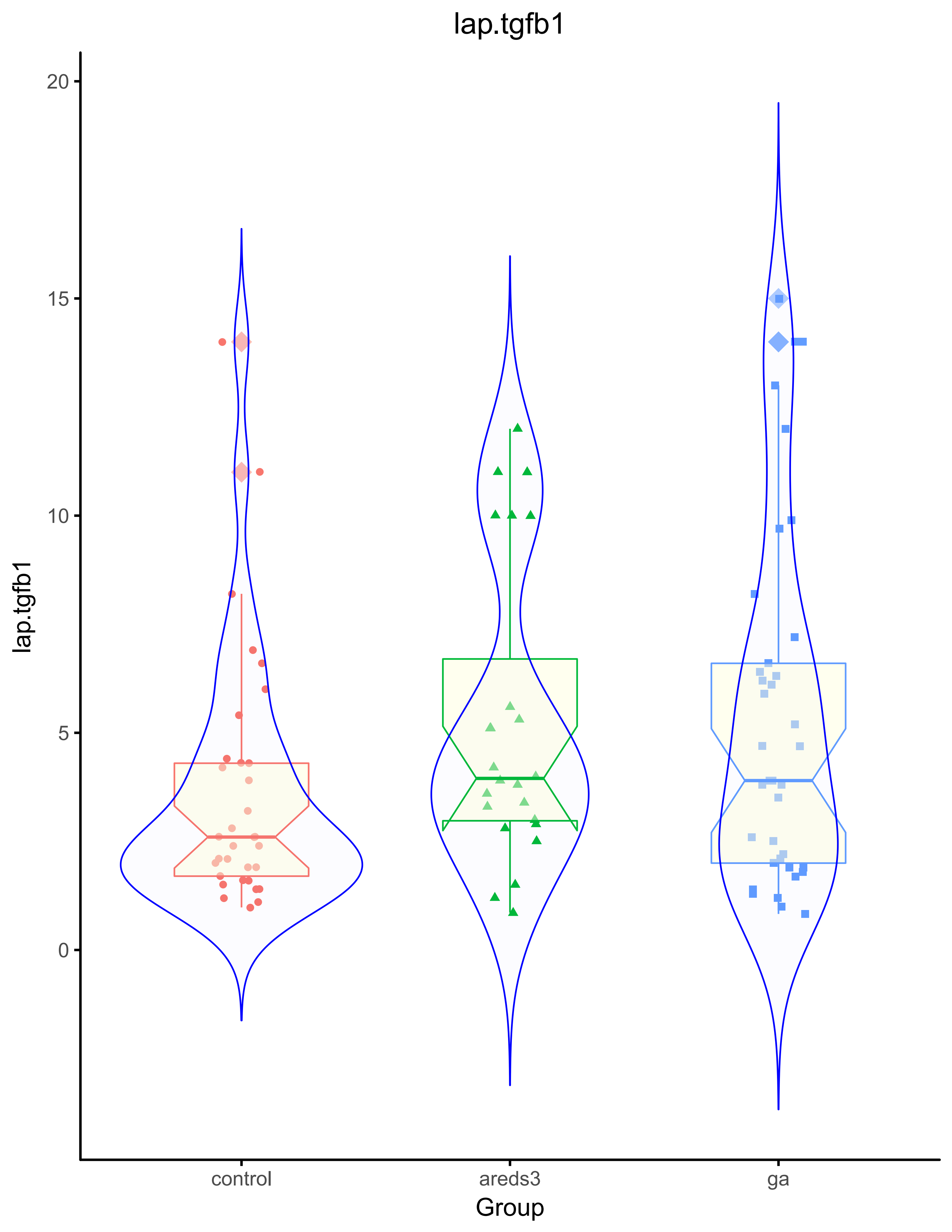

lap.tgfb1
20
15
10
5
0
lap.tgfb1
control
areds3
ga
Group

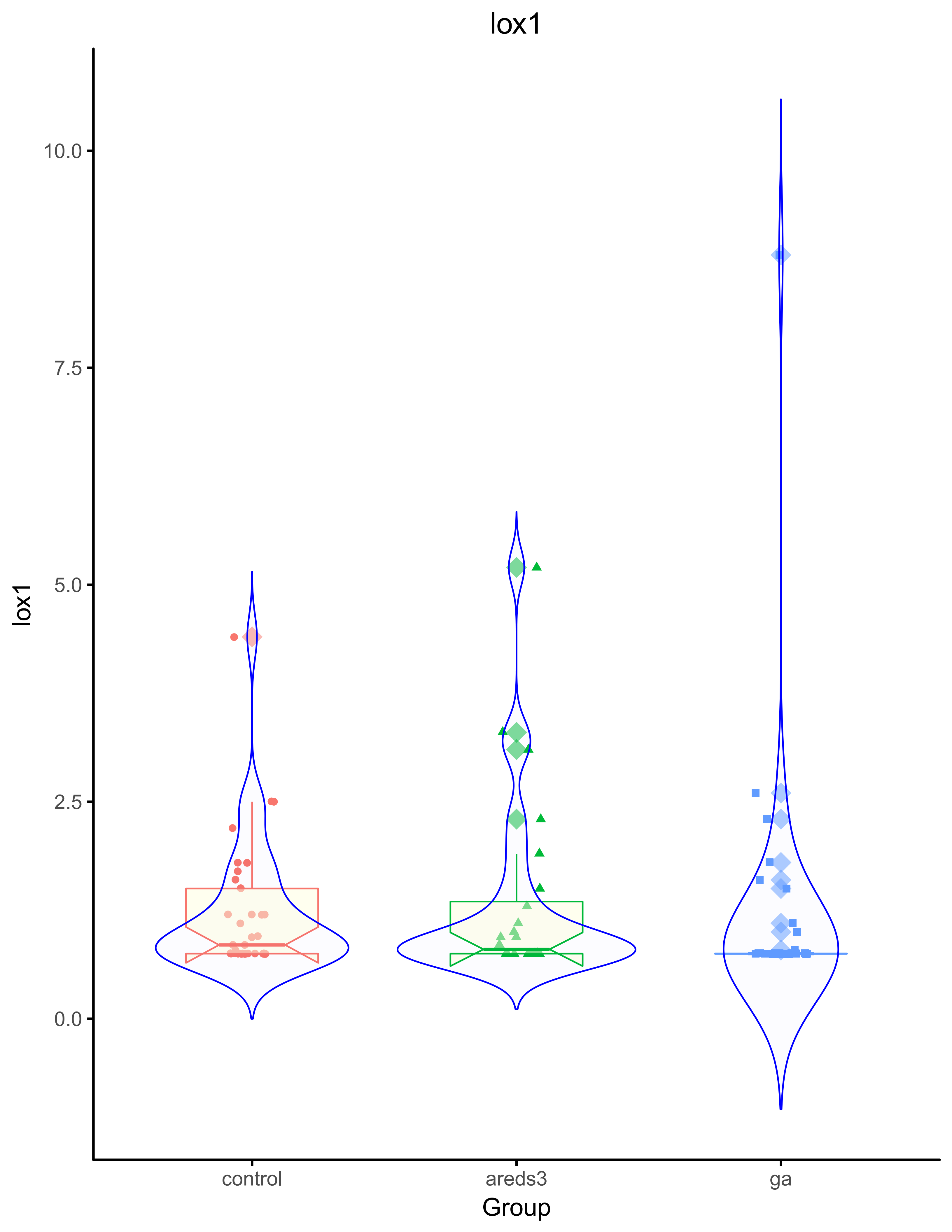

lox1
10.0
7.5
5.0
2.5
0.0
lox1
control
areds3
ga
Group

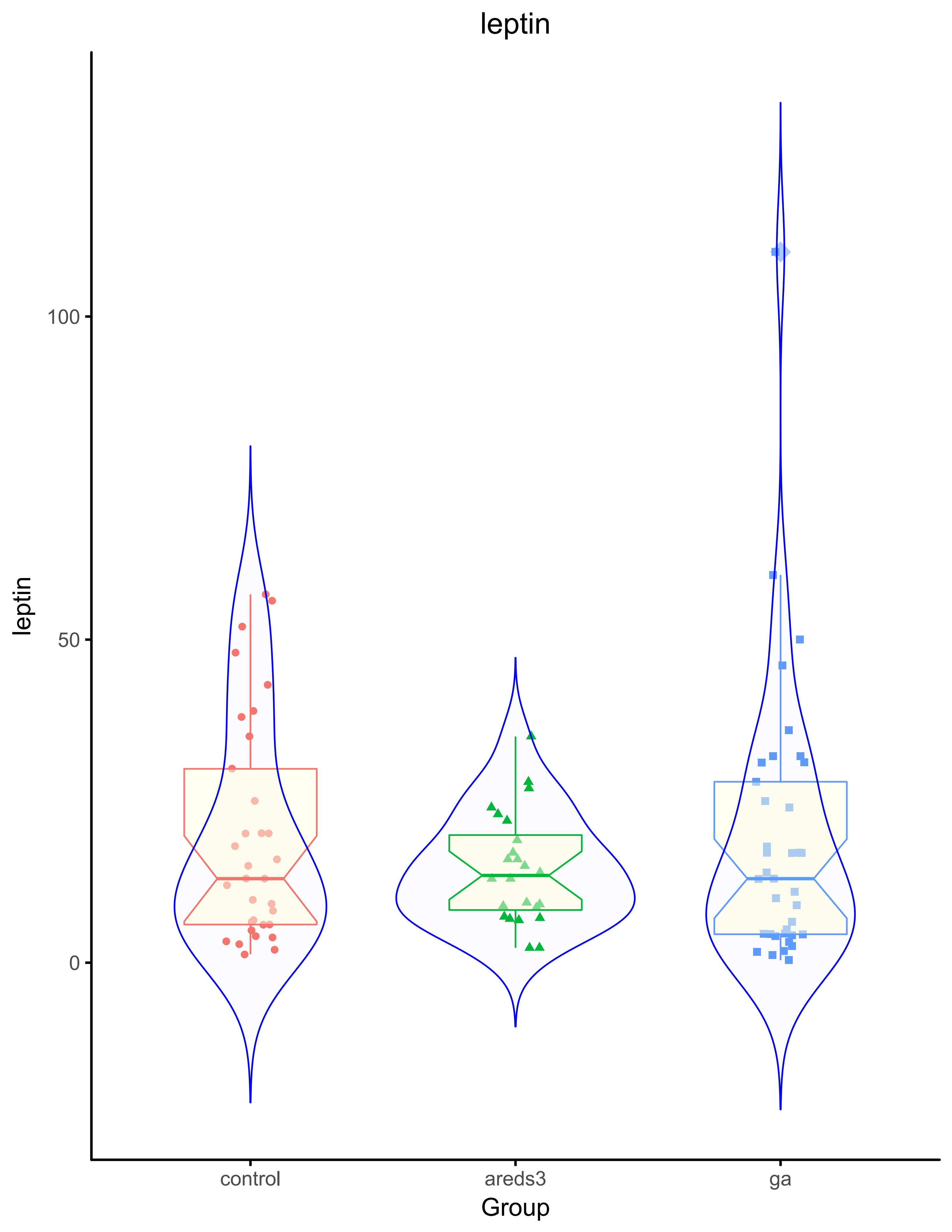

leptin
100
50
0
leptin
control
areds3
ga
Group

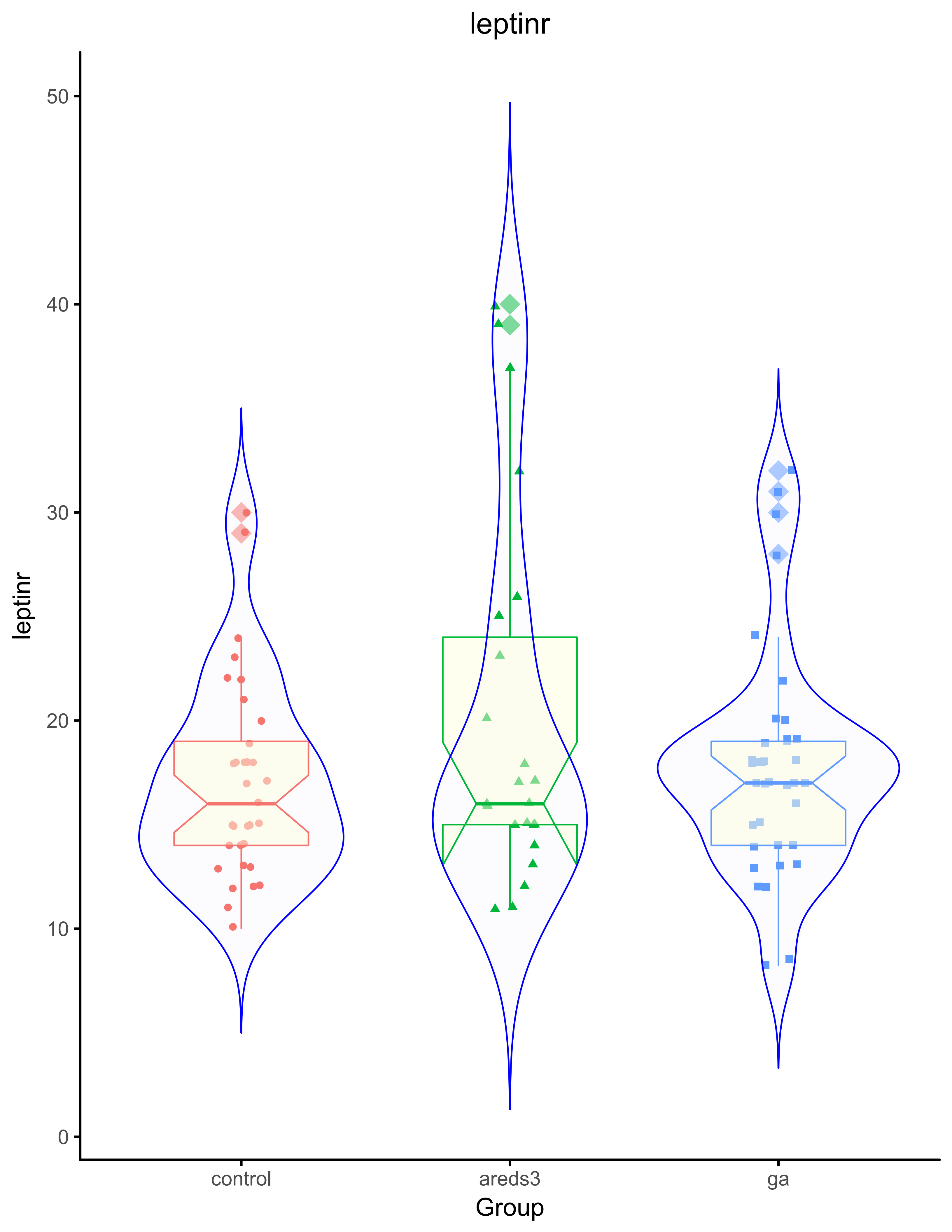

leptinr
50
40
30
20
10
0
leptinr
control
areds3
ga
Group

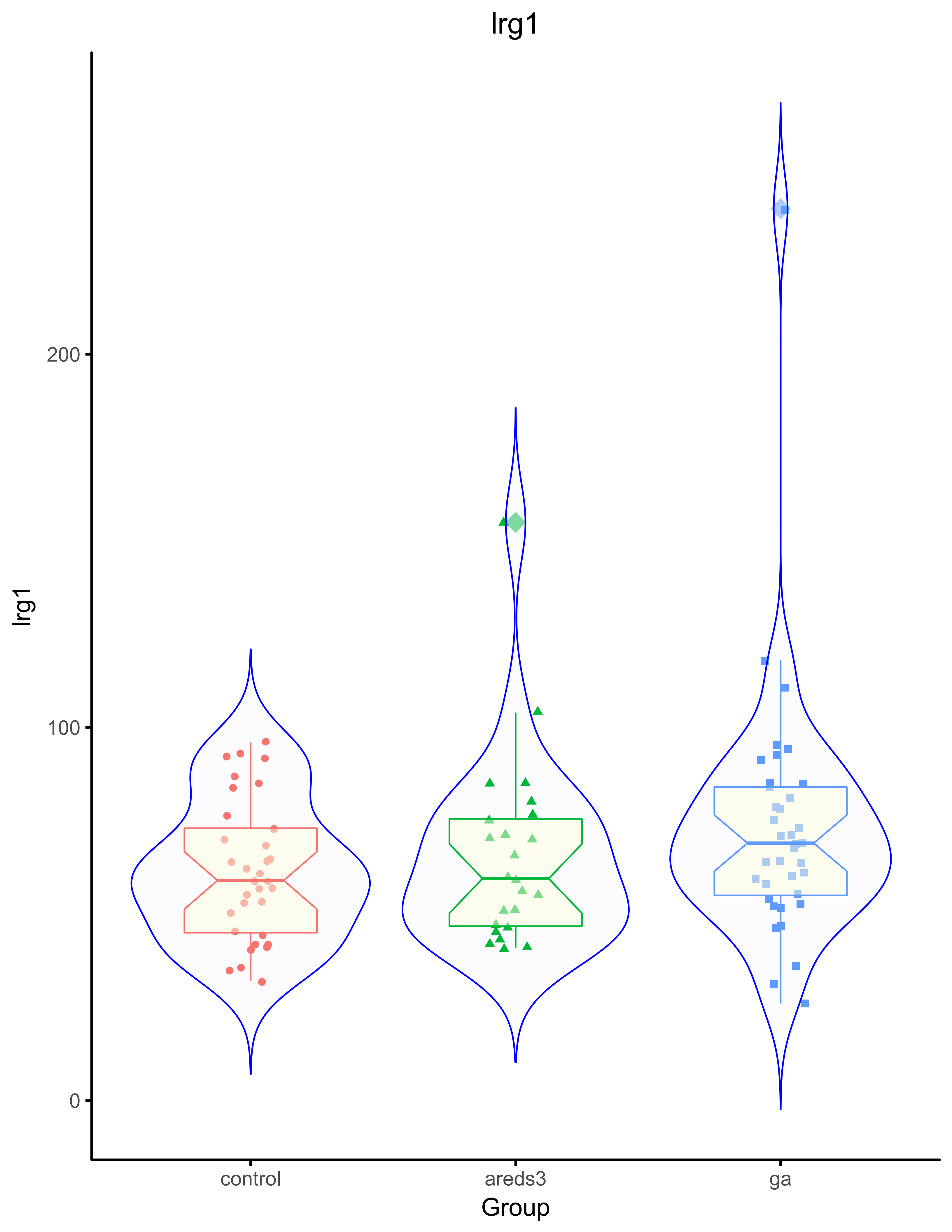

lrg1
lrg1
200
100
0
control
areds3
ga
Group

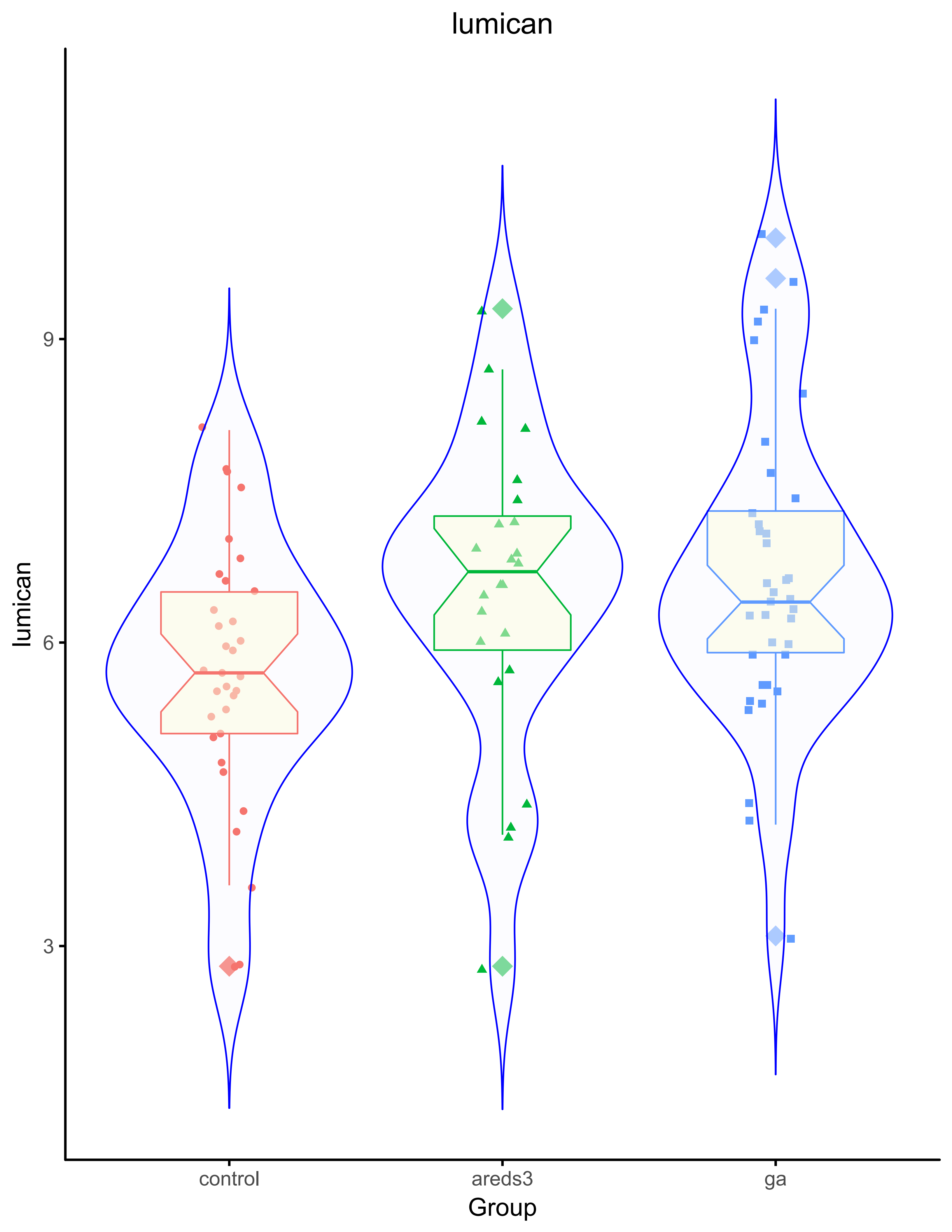

lumican
lumican
9
6
3
control
areds3
ga
Group

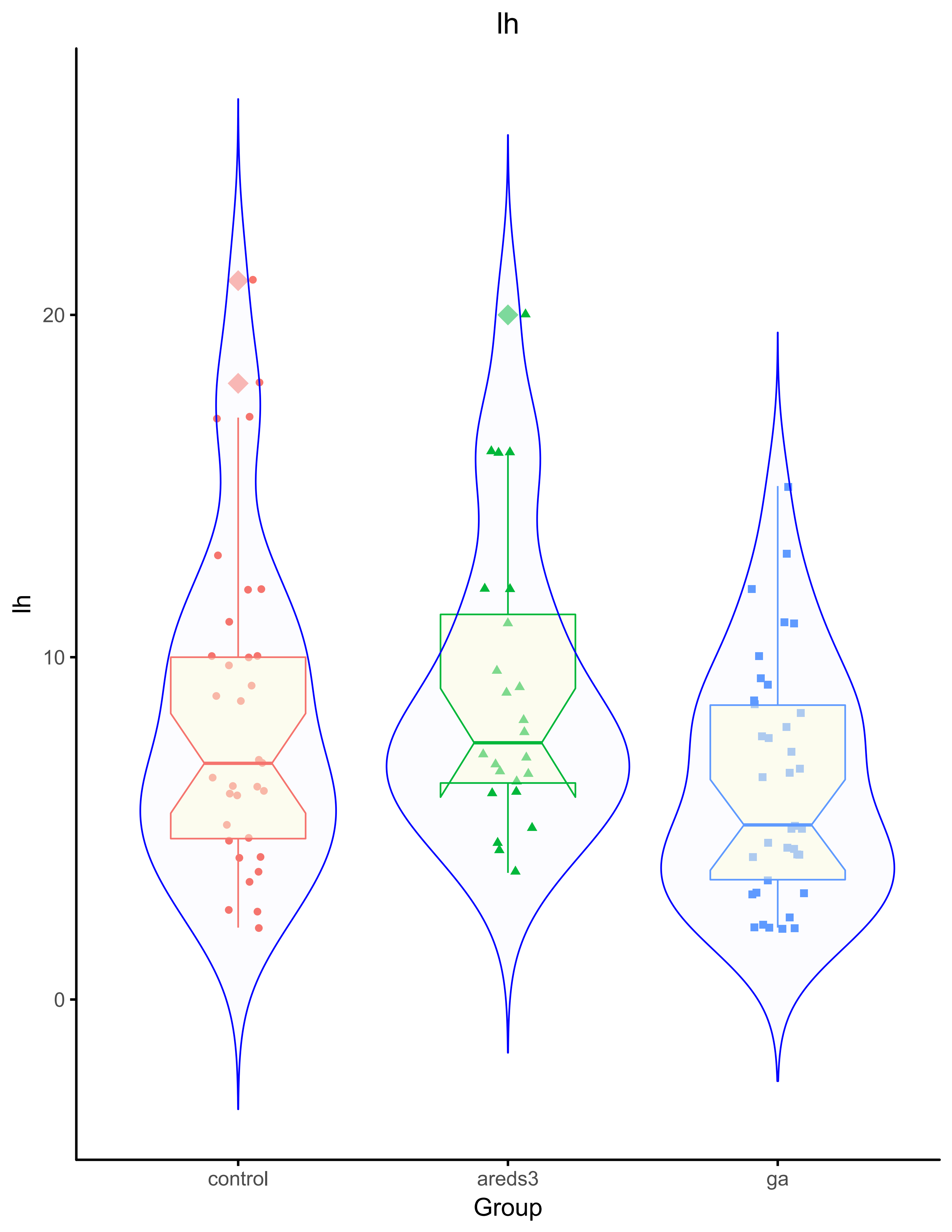

lh
20
10
0
lh
control
areds3
ga
Group

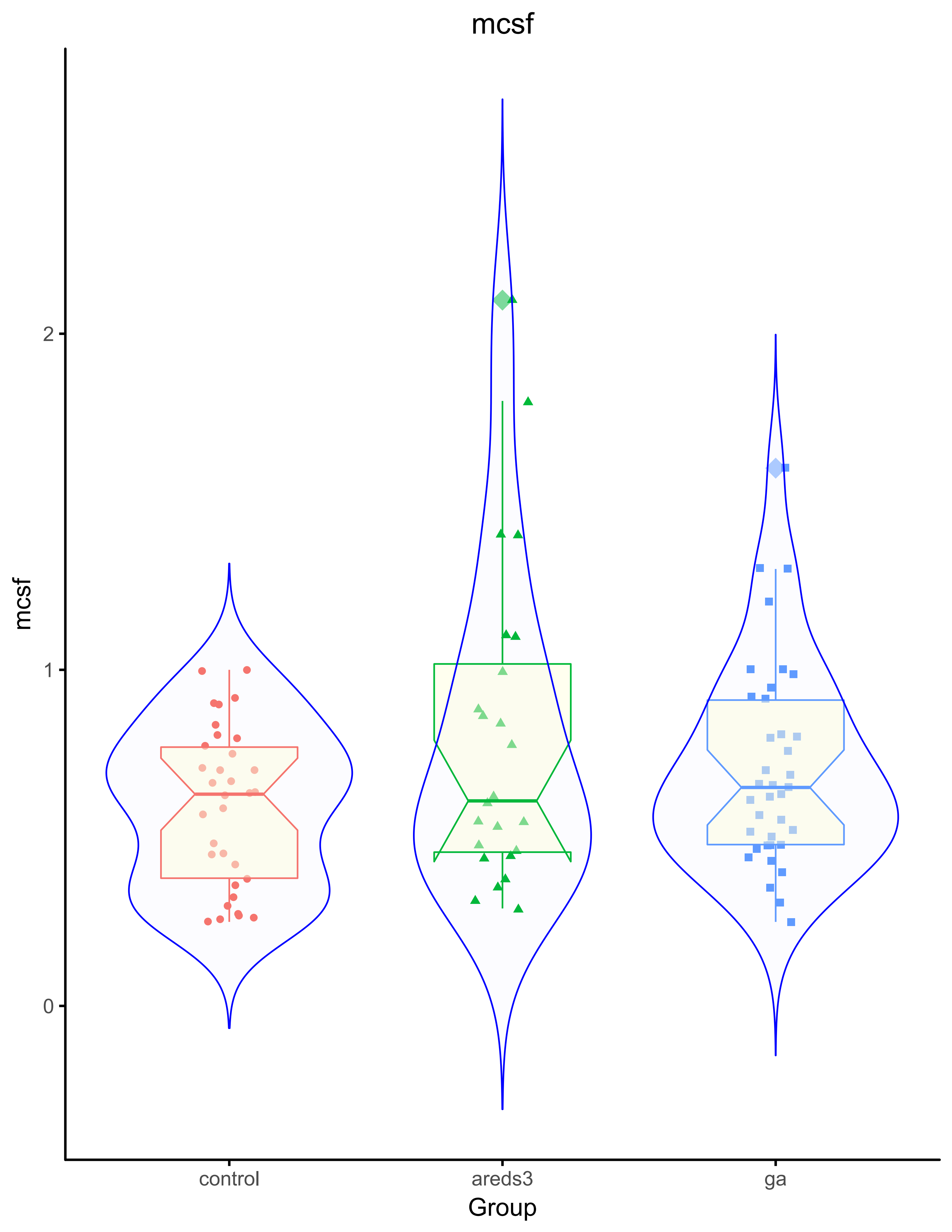
mcsf
mcsf
2
1
0
control
areds3
ga
Group

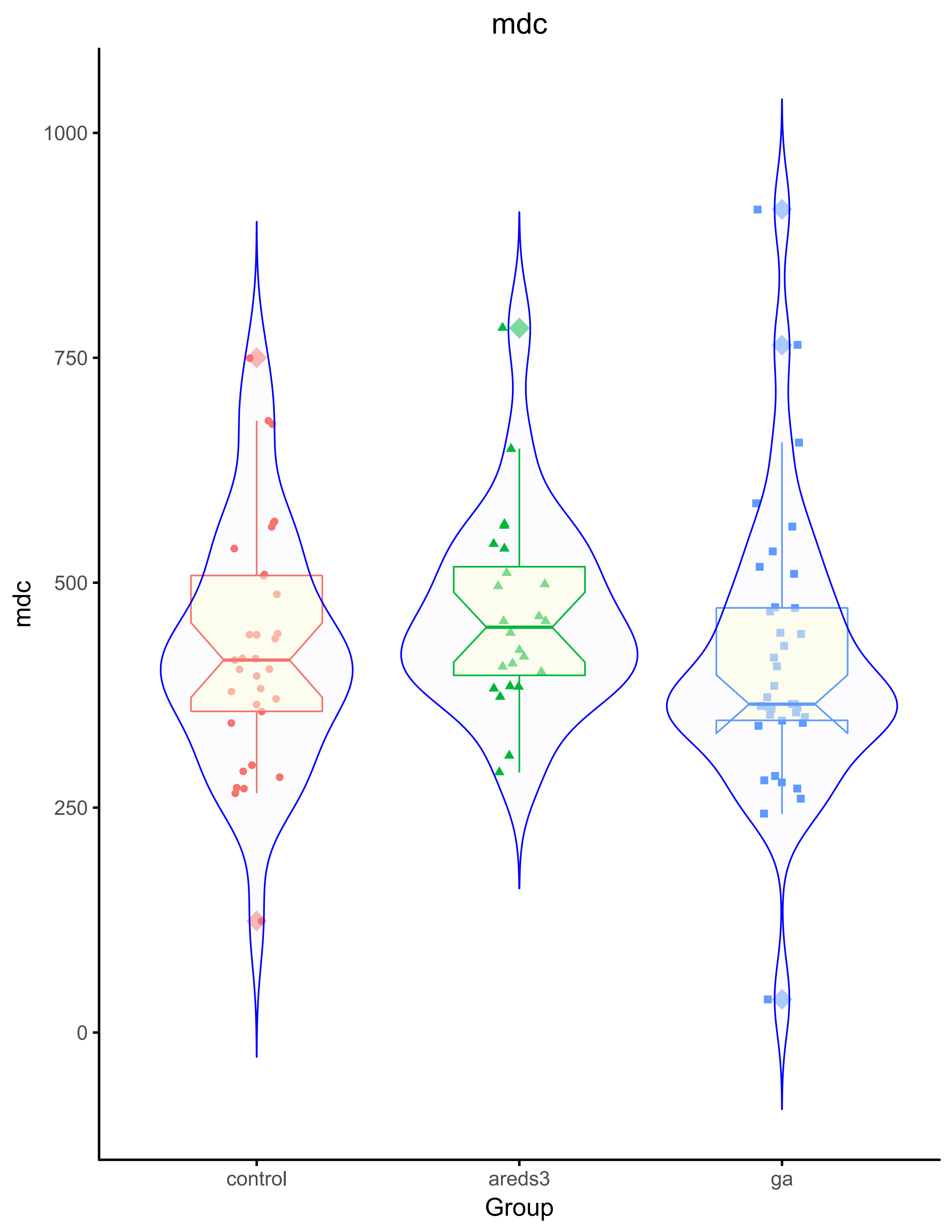
mdc
1000
750
500
250
0
mdc
control
areds3
ga
Group

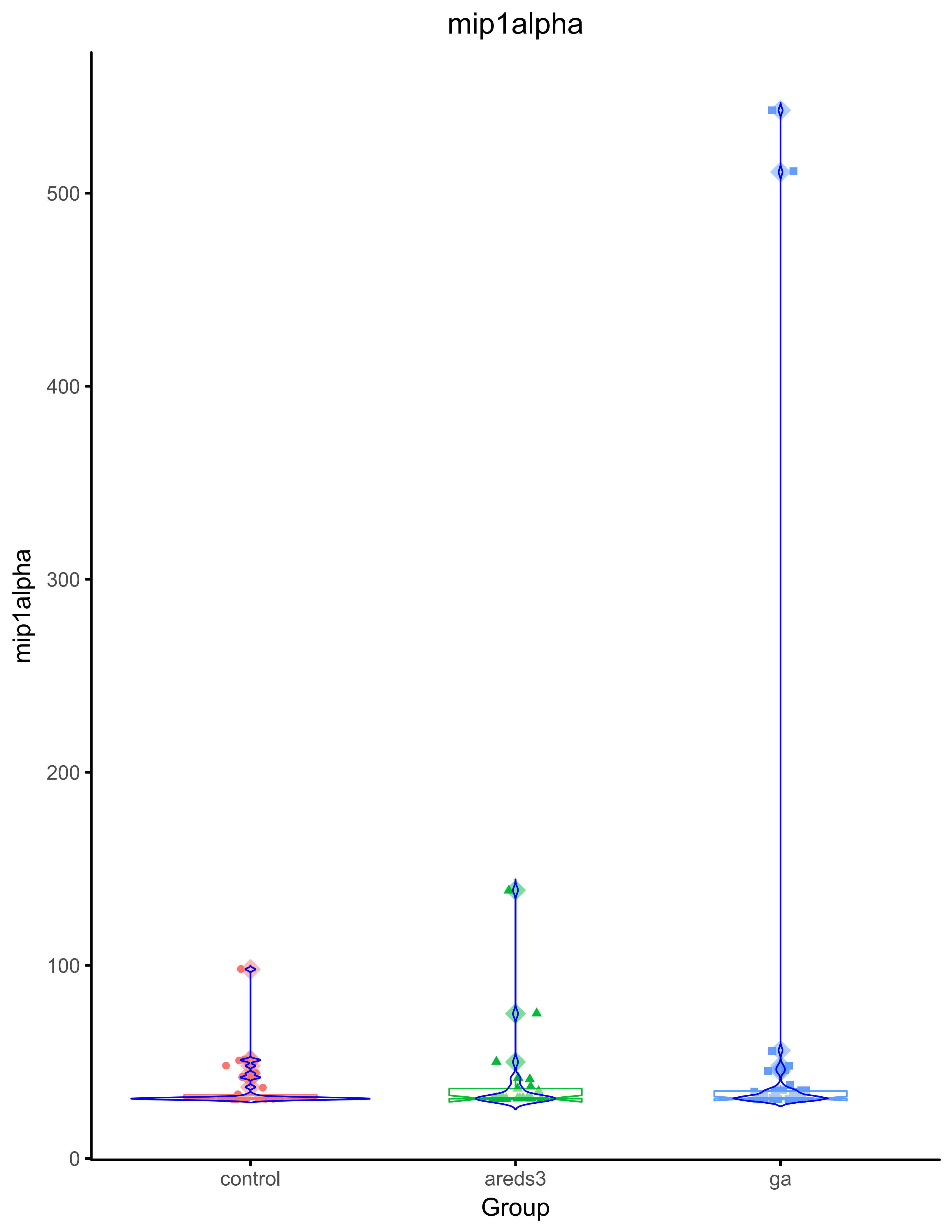
mip1alpha
500
400
300
200
100
0
mip1alpha
control
areds3
ga
Group

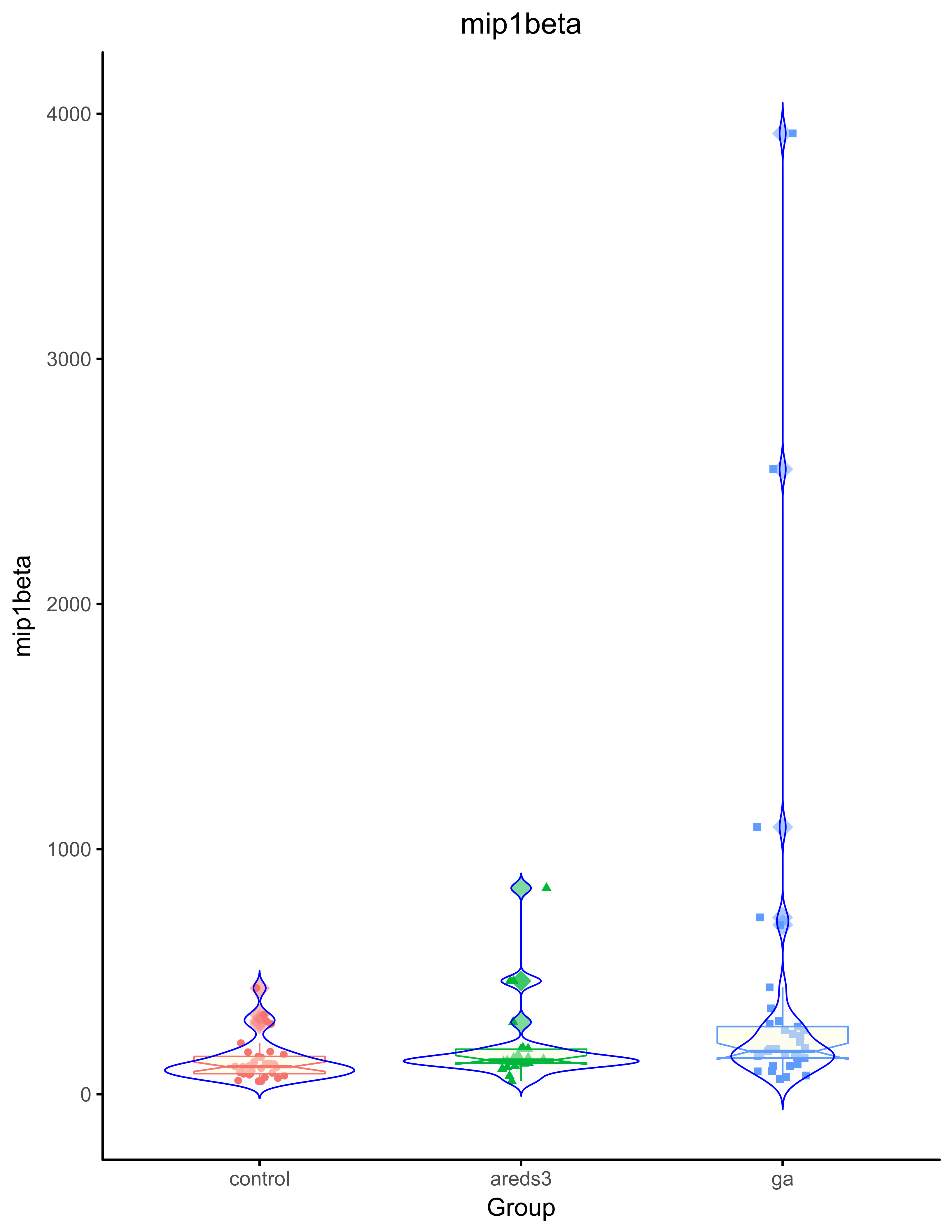
mip1beta
4000
3000
2000
1000
0
mip1beta
control
areds3
ga
Group

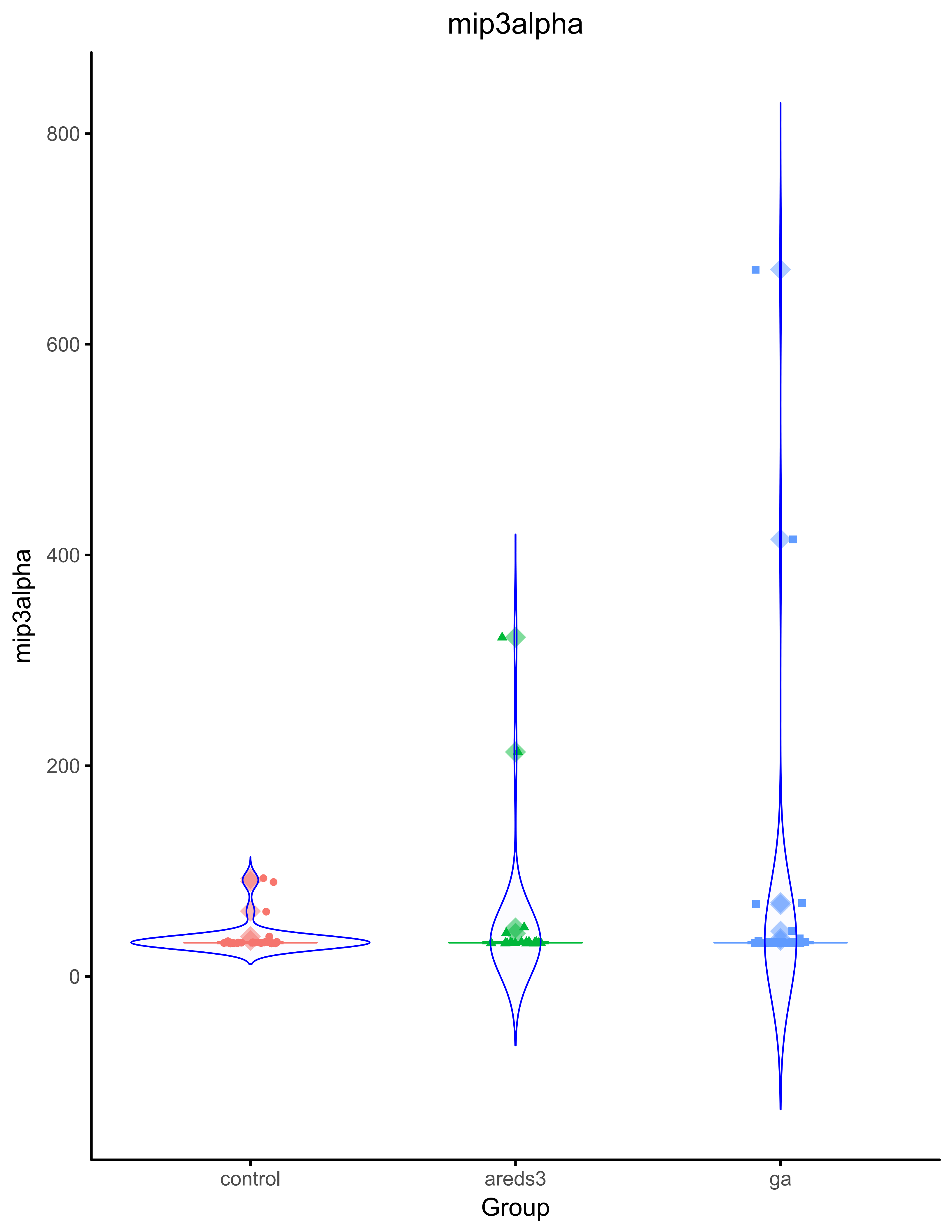
mip3alpha
800
600
400
200
0
mip3alpha
control
areds3
ga
Group

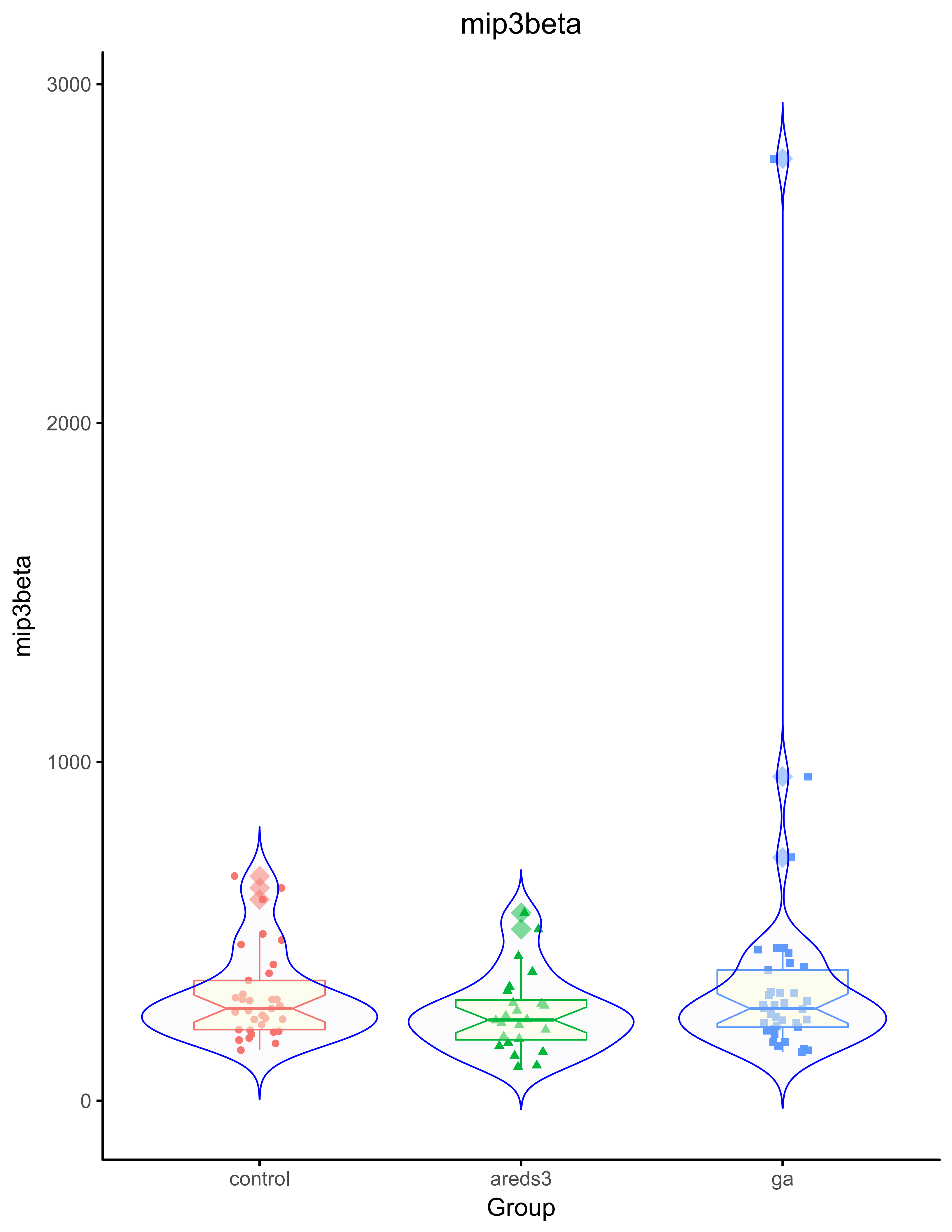
mip3beta
3000
2000
1000
0
mip3beta
control
areds3
ga
Group

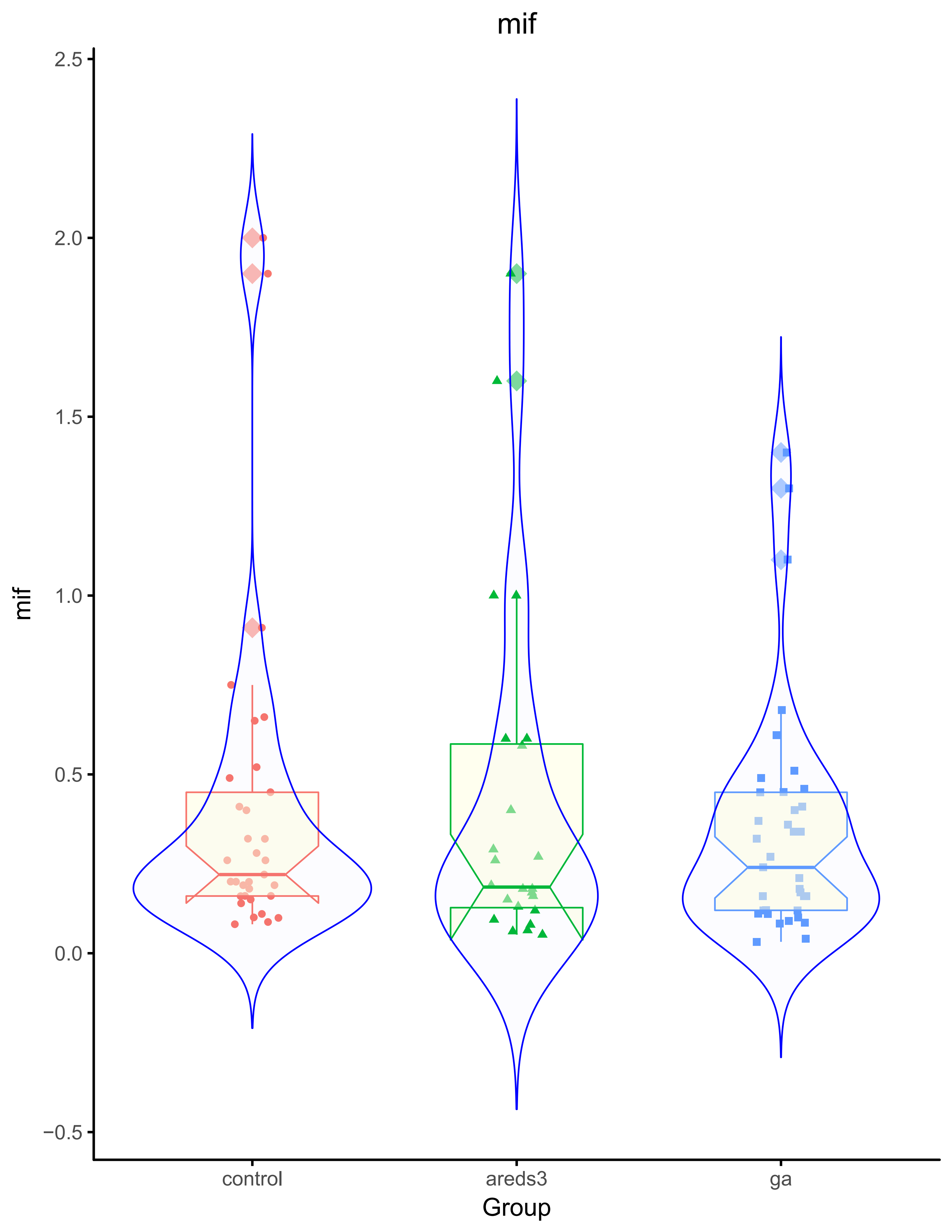
mif
2.5
2.0
1.5
1.0
0.5
0.0
−0.5
mif
control
areds3
ga
Group

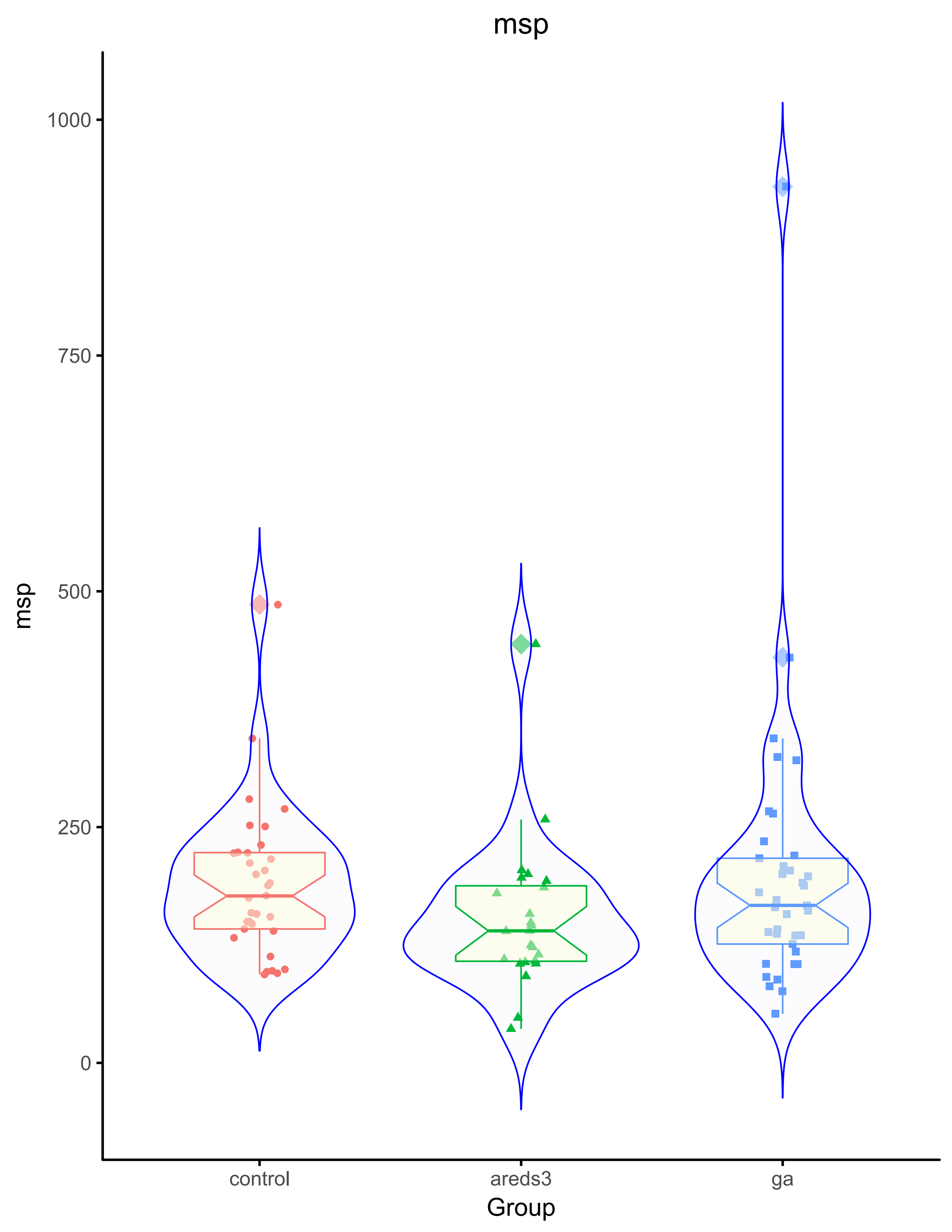

msp
1000
750
500
250
0
msp
control
areds3
ga
Group

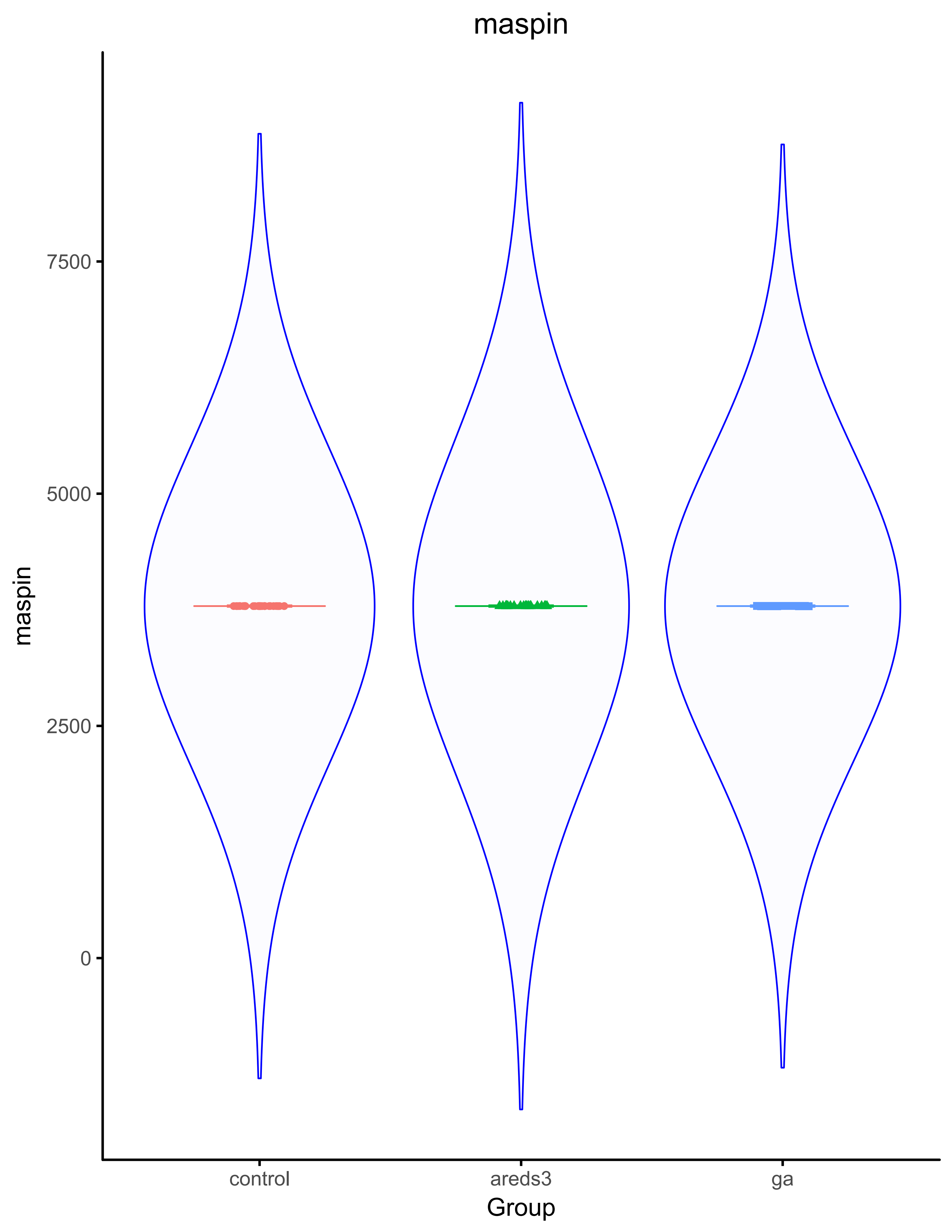
maspin
7500
5000
2500
0
maspin
control
areds3
ga
Group

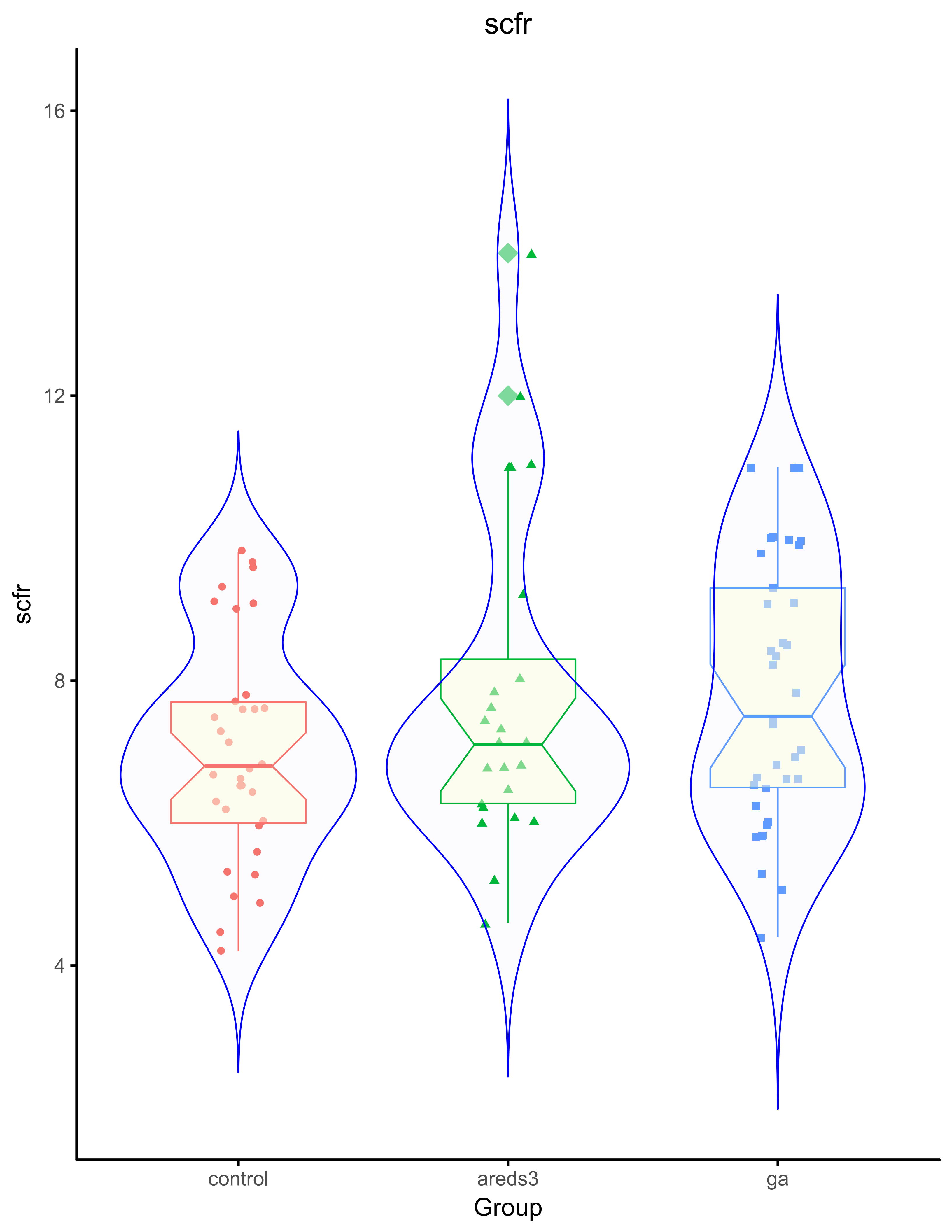
scfr
16
12
8
4
scfr
control
areds3
ga
Group

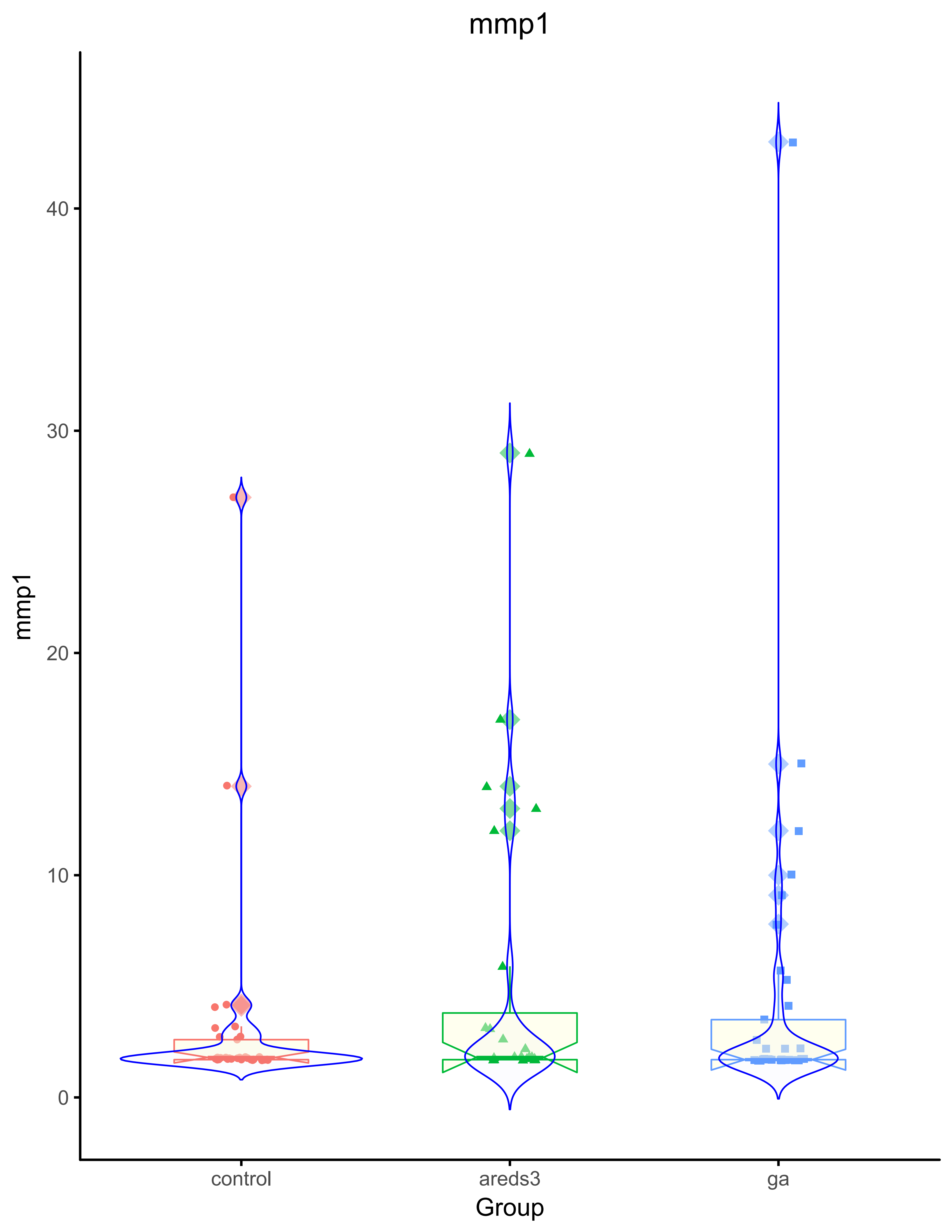

mmp1
40
30
20
10
0
mmp1
control
areds3
ga
Group

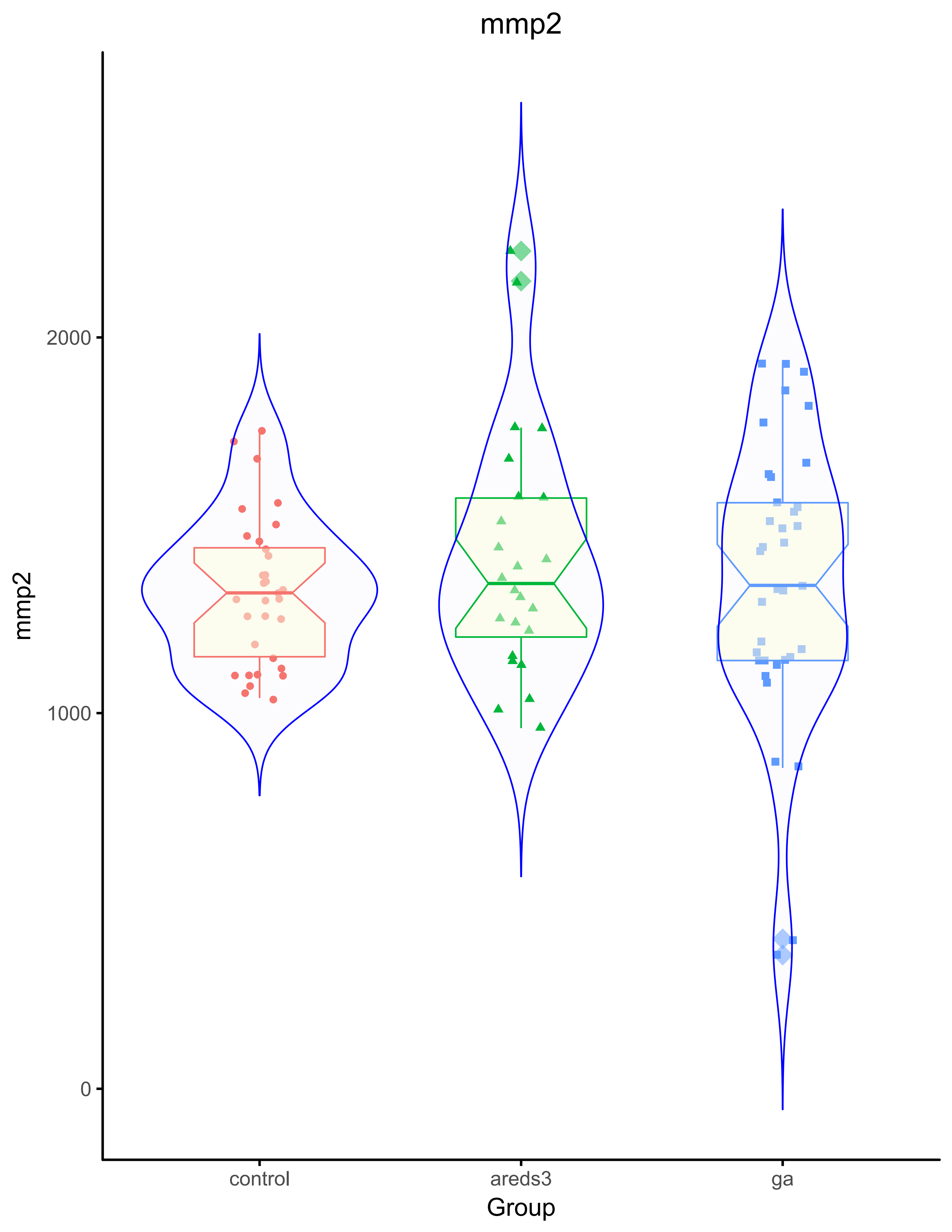
mmp2
2000
1000
0
mmp2
control
areds3
ga
Group

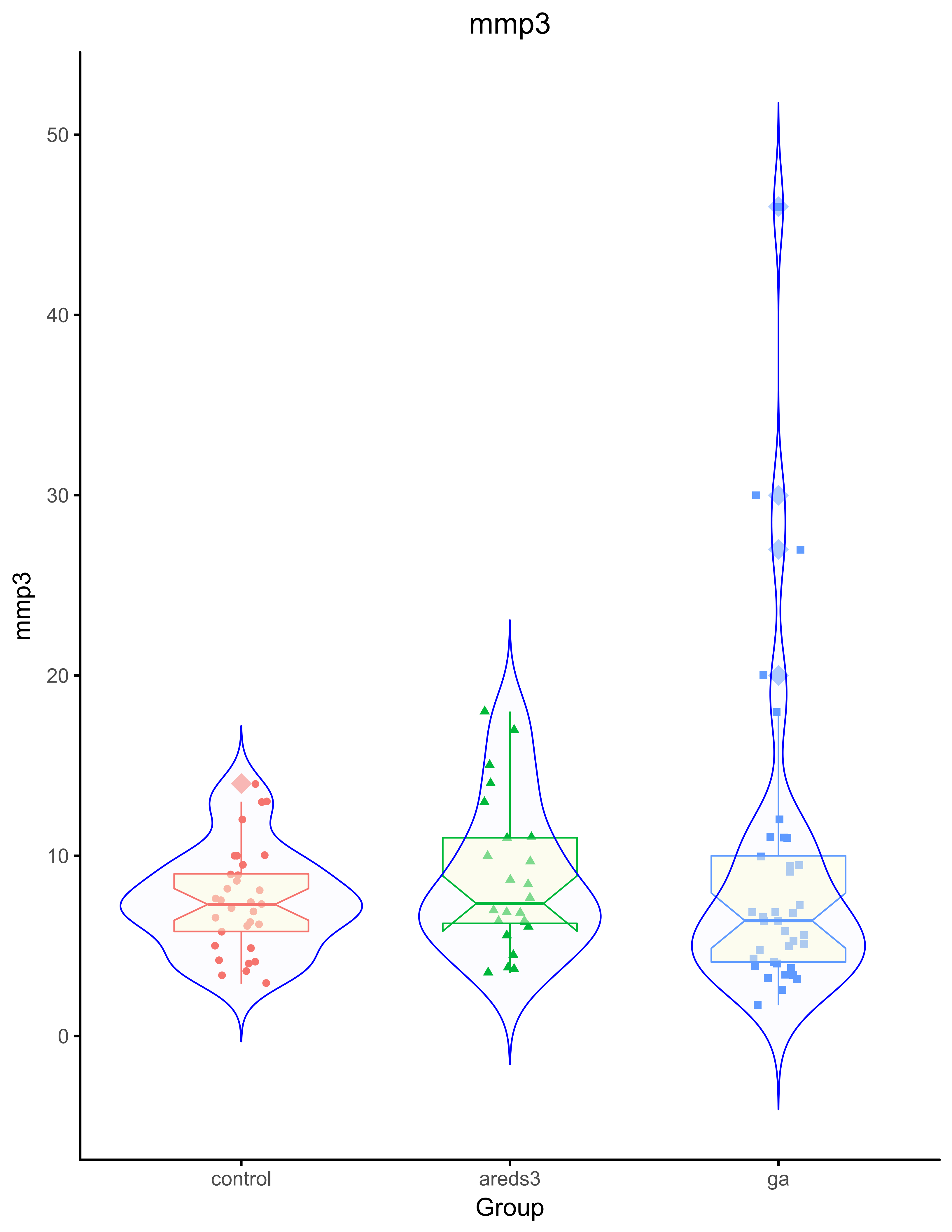

mmp3
50
40
30
20
10
0
mmp3
control
areds3
ga
Group

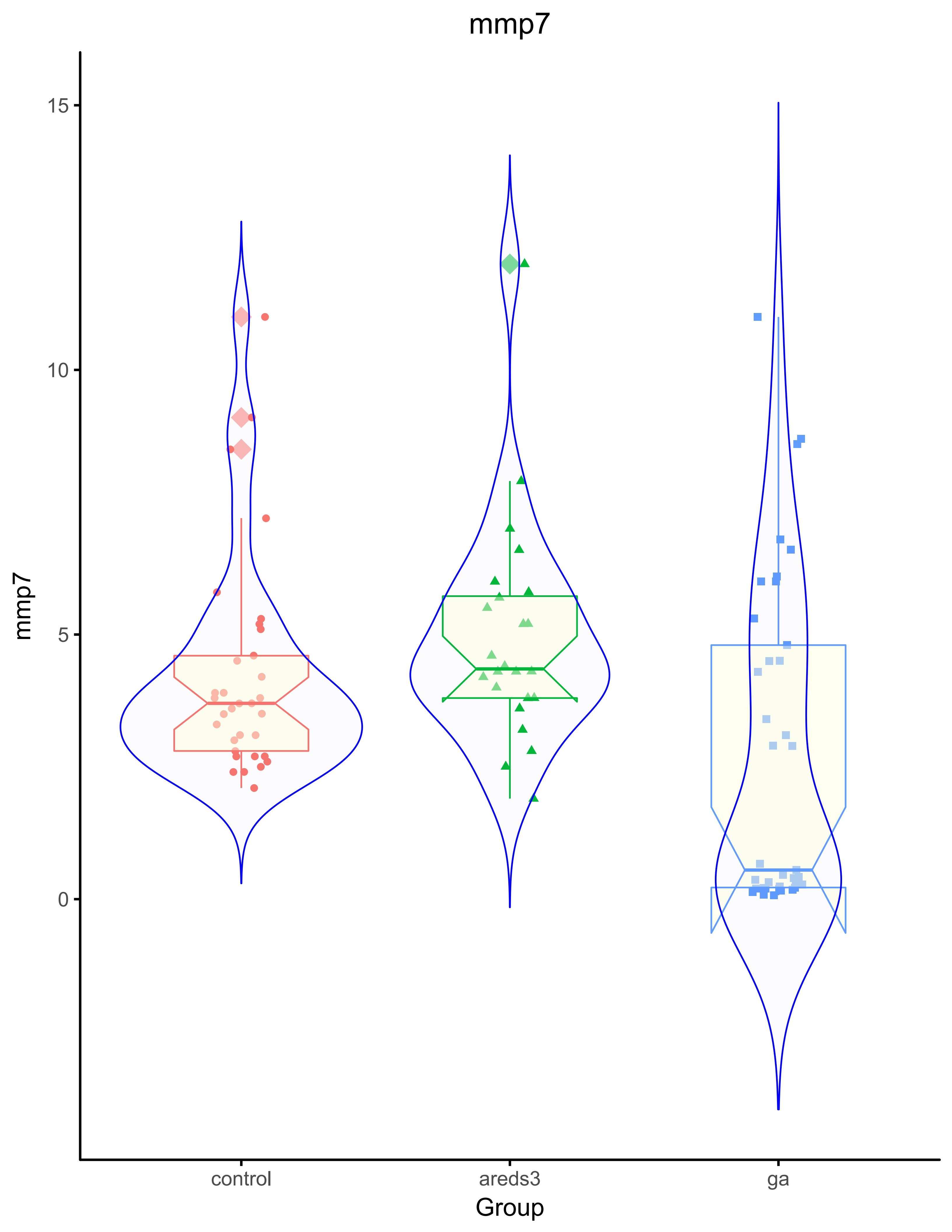

mmp7
15
10
5
0
mmp7
control
areds3
ga
Group

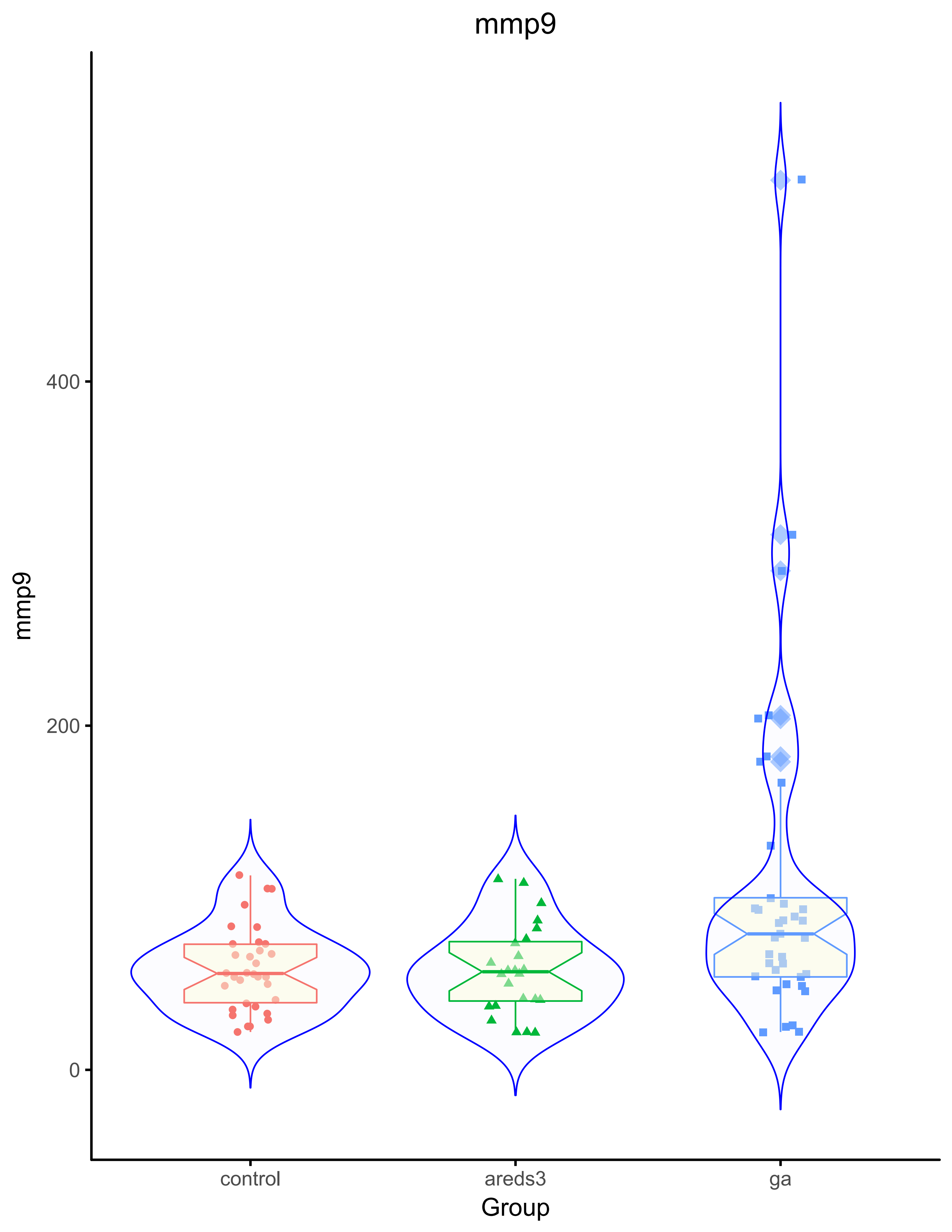

mmp9
400
200
0
mmp9
control
areds3
ga
Group

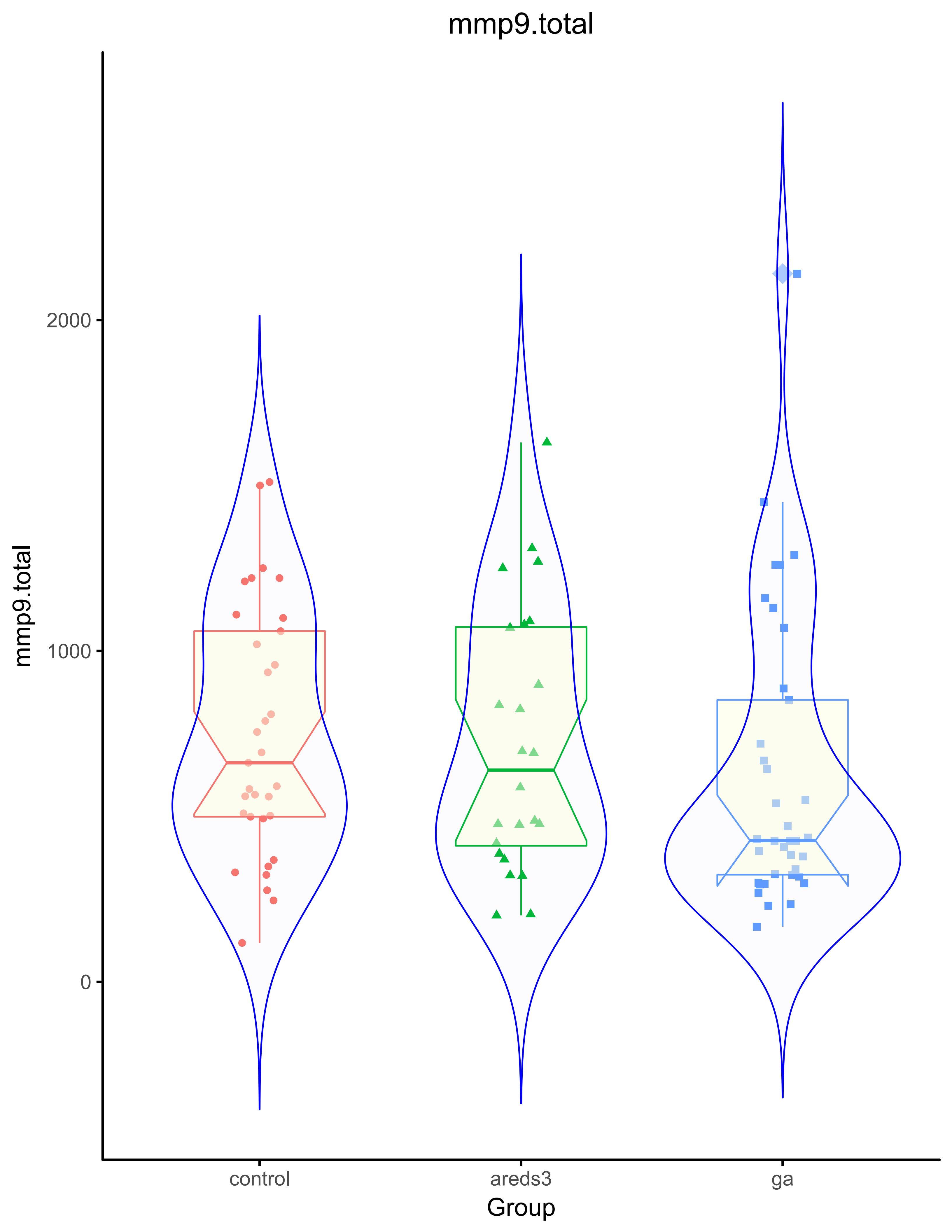

mmp9.total
2000
1000
0
mmp9.total
control
areds3
ga
Group

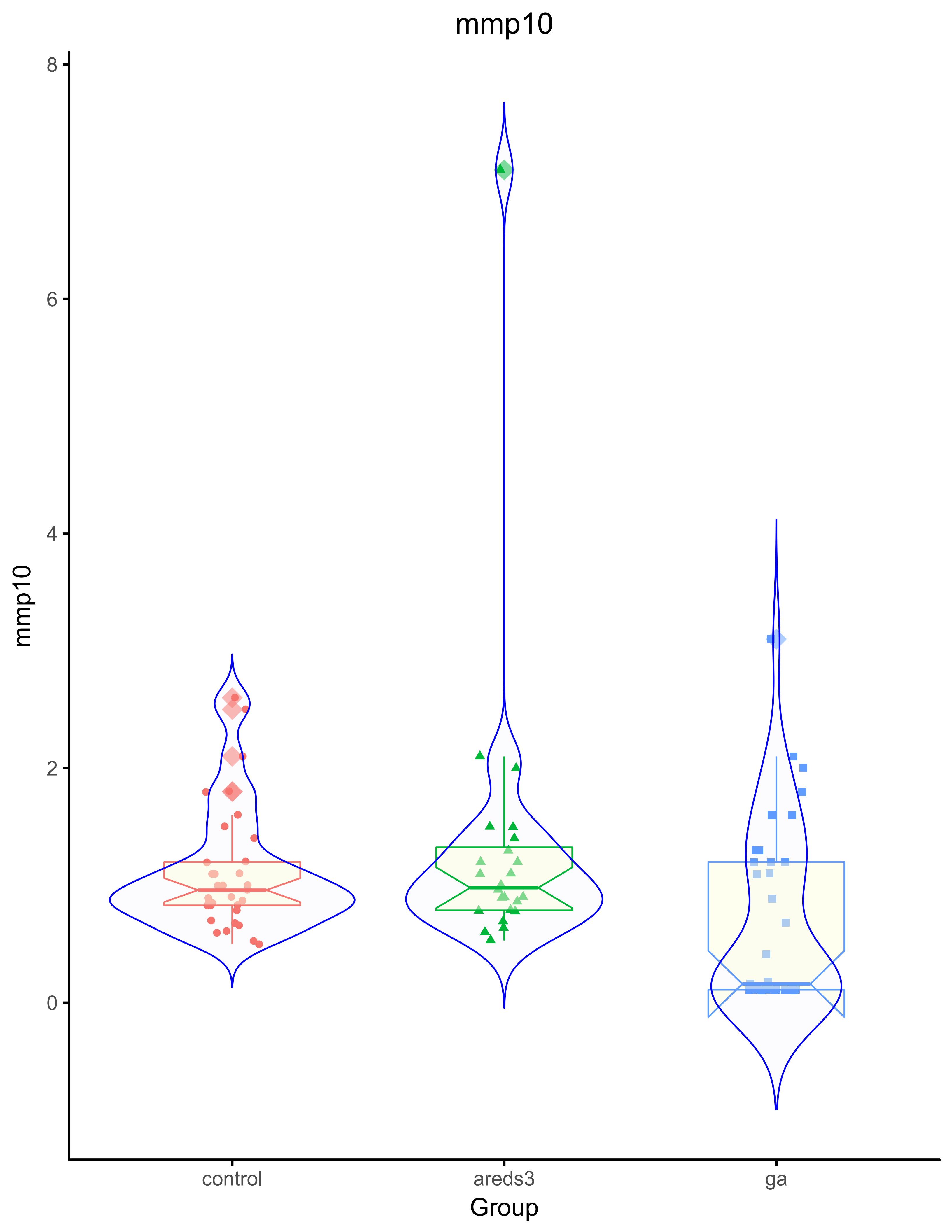

mmp10
8
6
4
2
0
mmp10
control
areds3
ga
Group

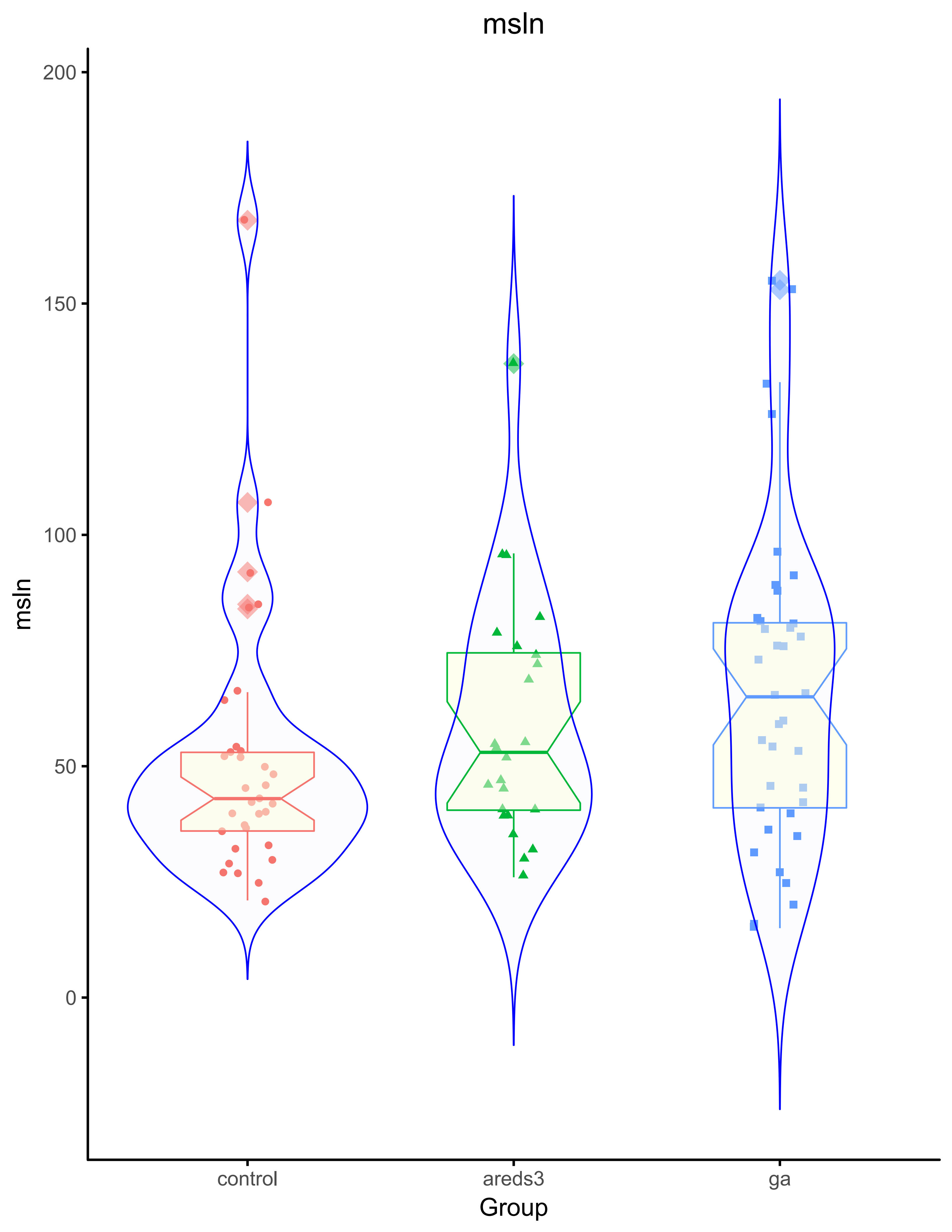

msln
200
150
100
50
0
msln
control
areds3
ga
Group

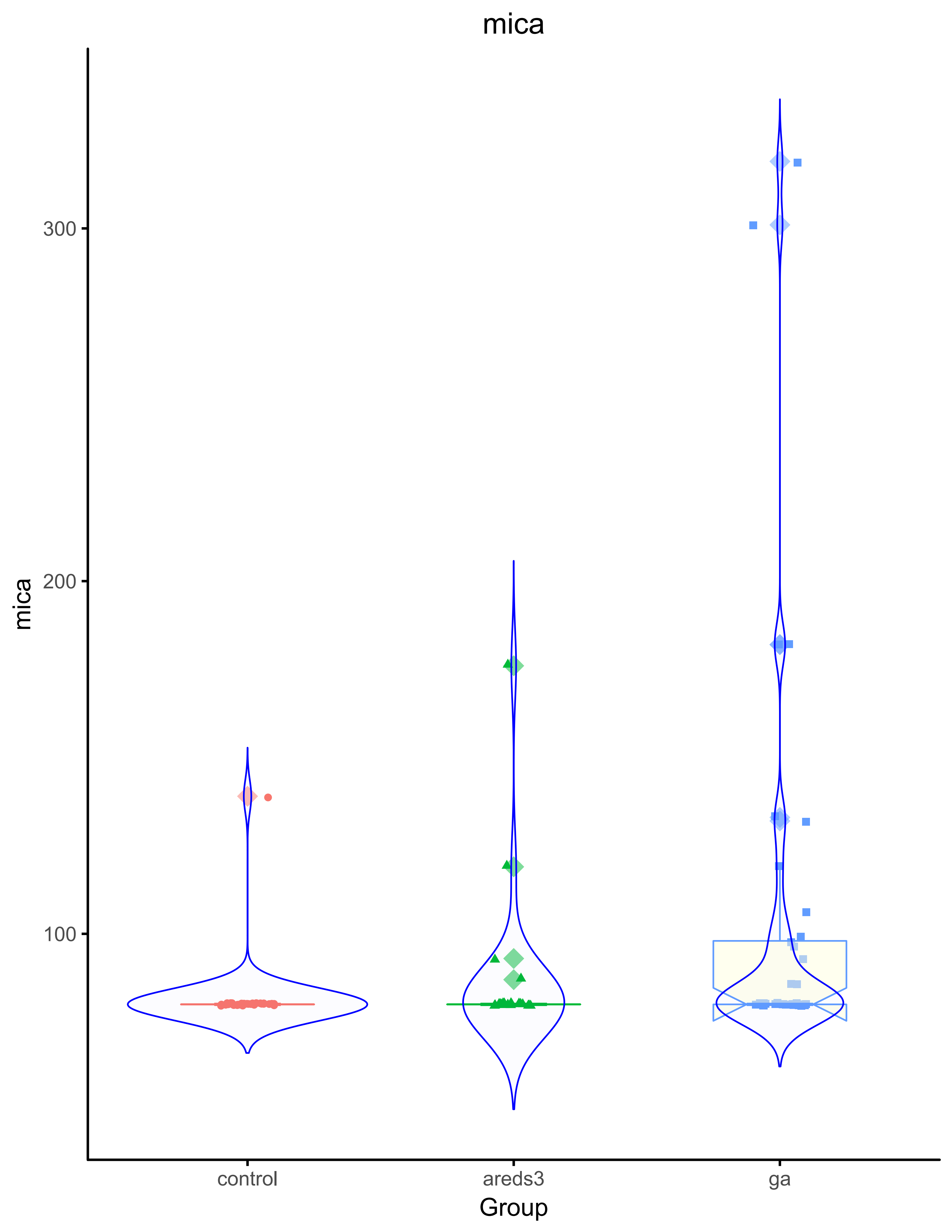
mica
300
200
100
mica
control
areds3
ga
Group

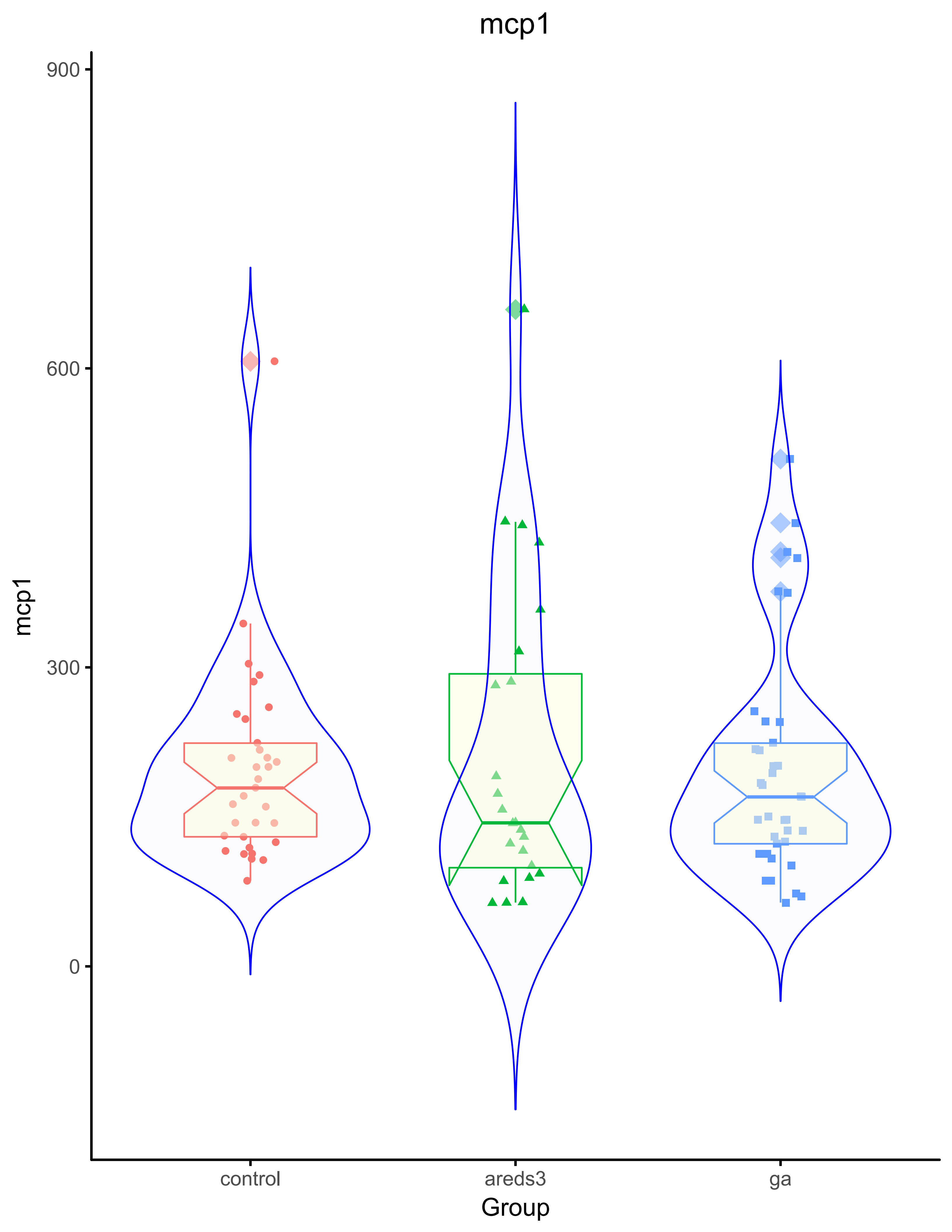

mcp1
900
600
300
0
mcp1
control
areds3
ga
Group

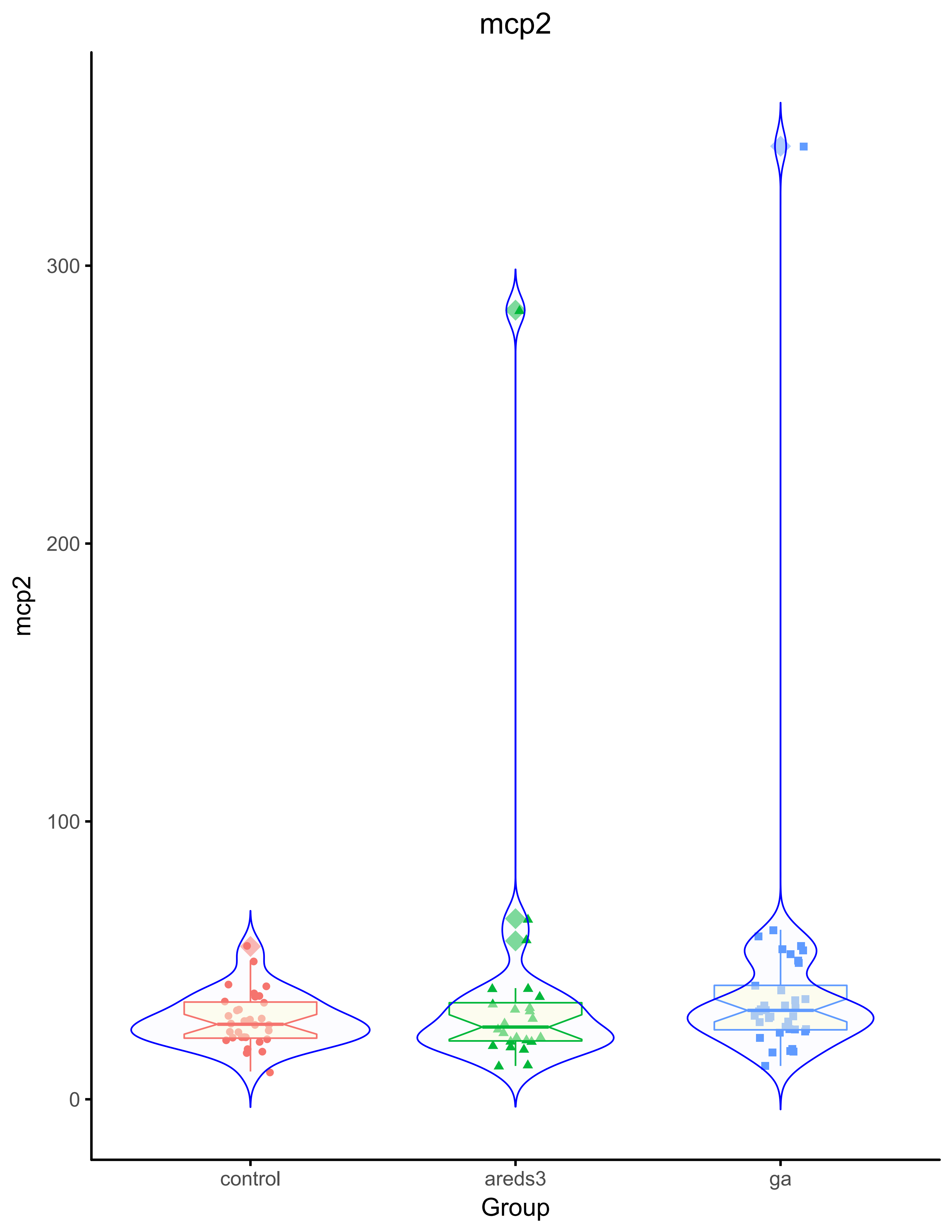

mcp2
300
200
100
0
mcp2
control
areds3
ga
Group

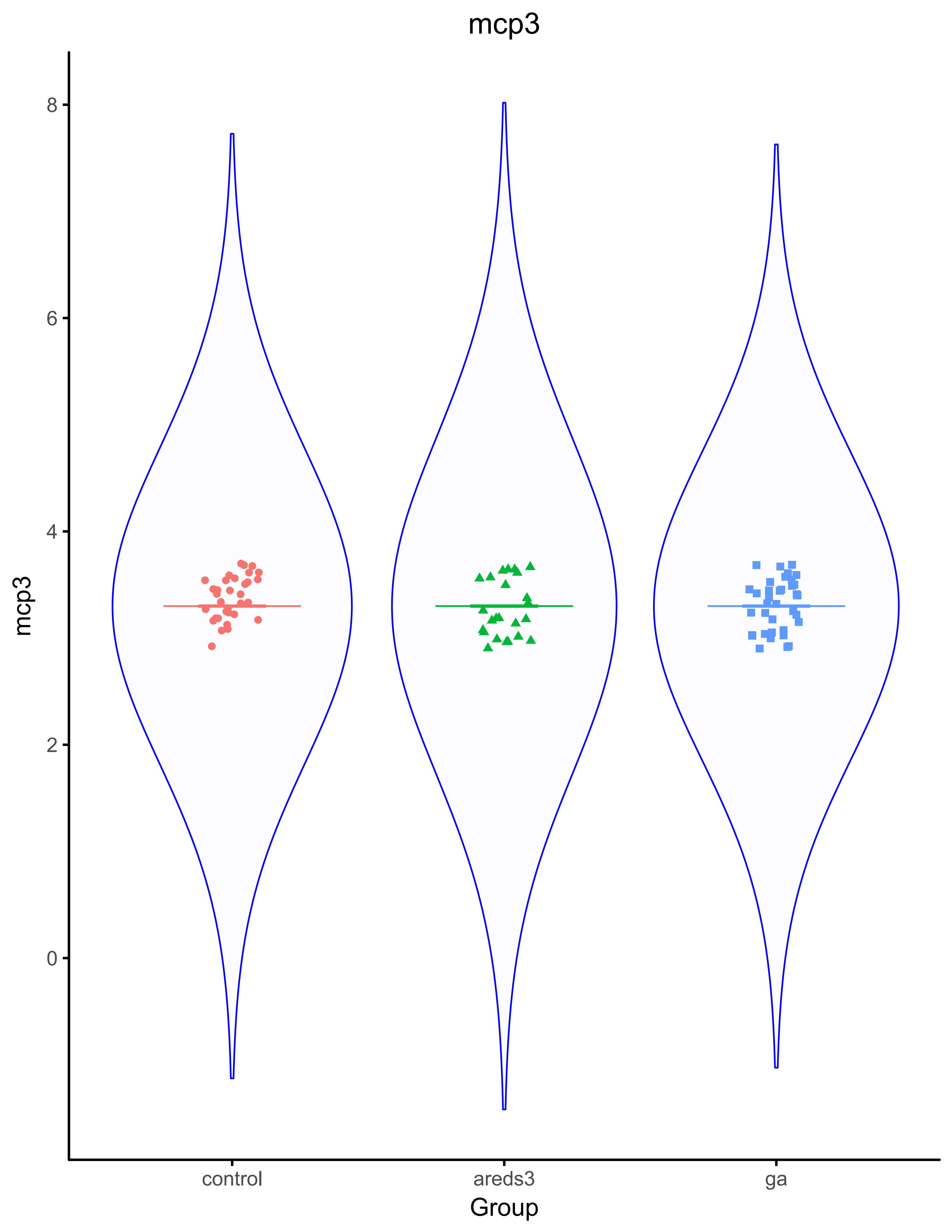

mcp3
8
6
4
2
0
mcp3
control
areds3
ga
Group

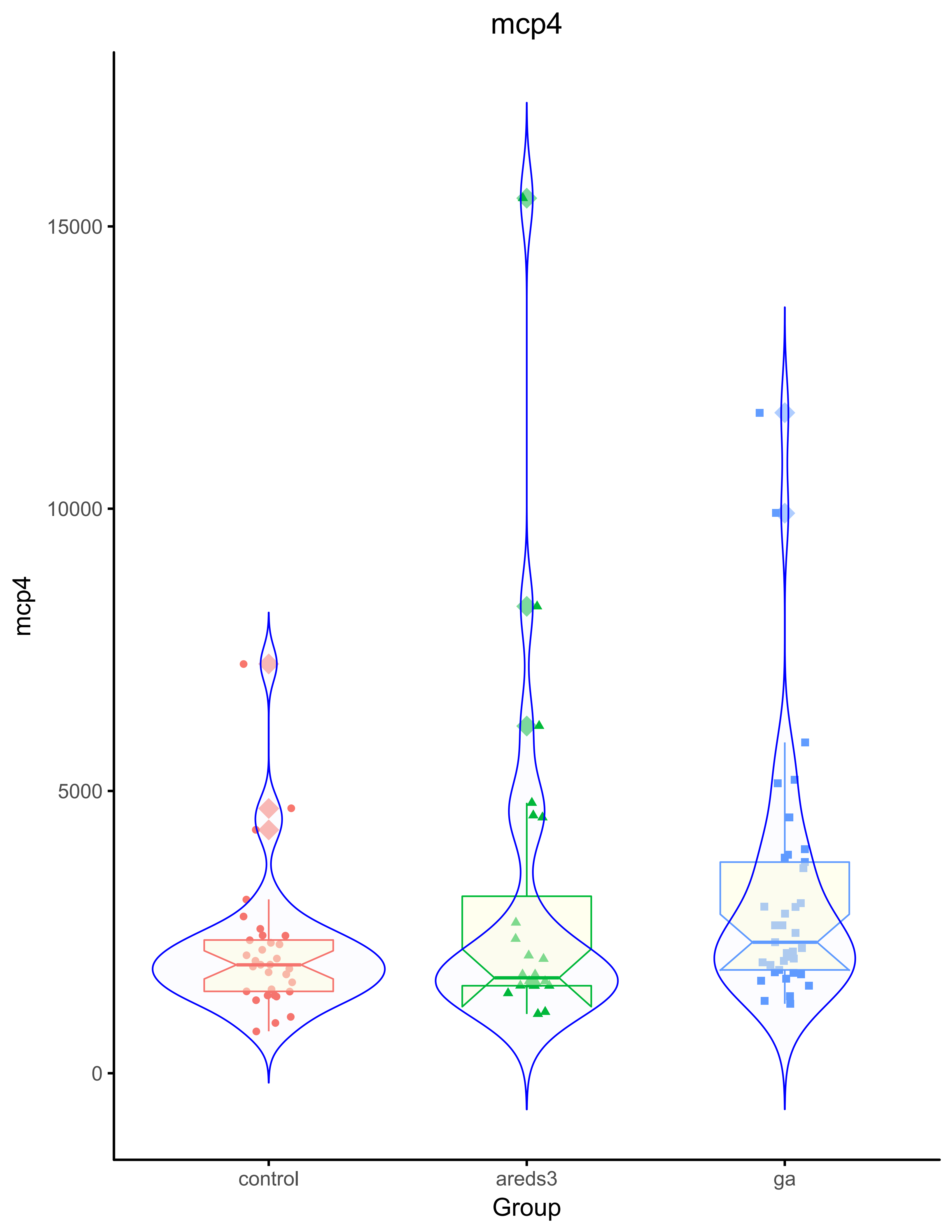

mcp4
15000
10000
5000
0
mcp4
control
areds3
ga
Group

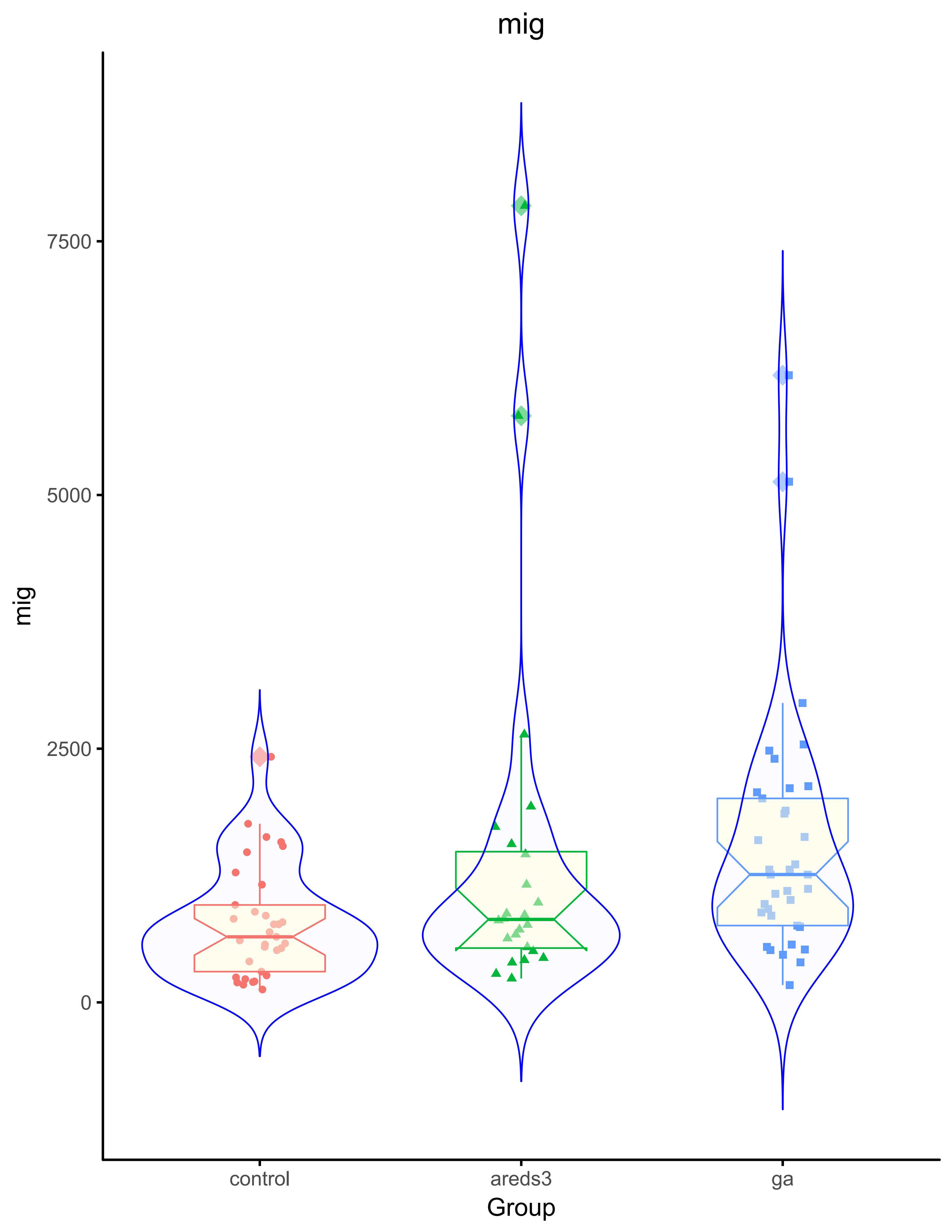
mig
7500
5000
2500
0
mig
control
areds3
ga
Group

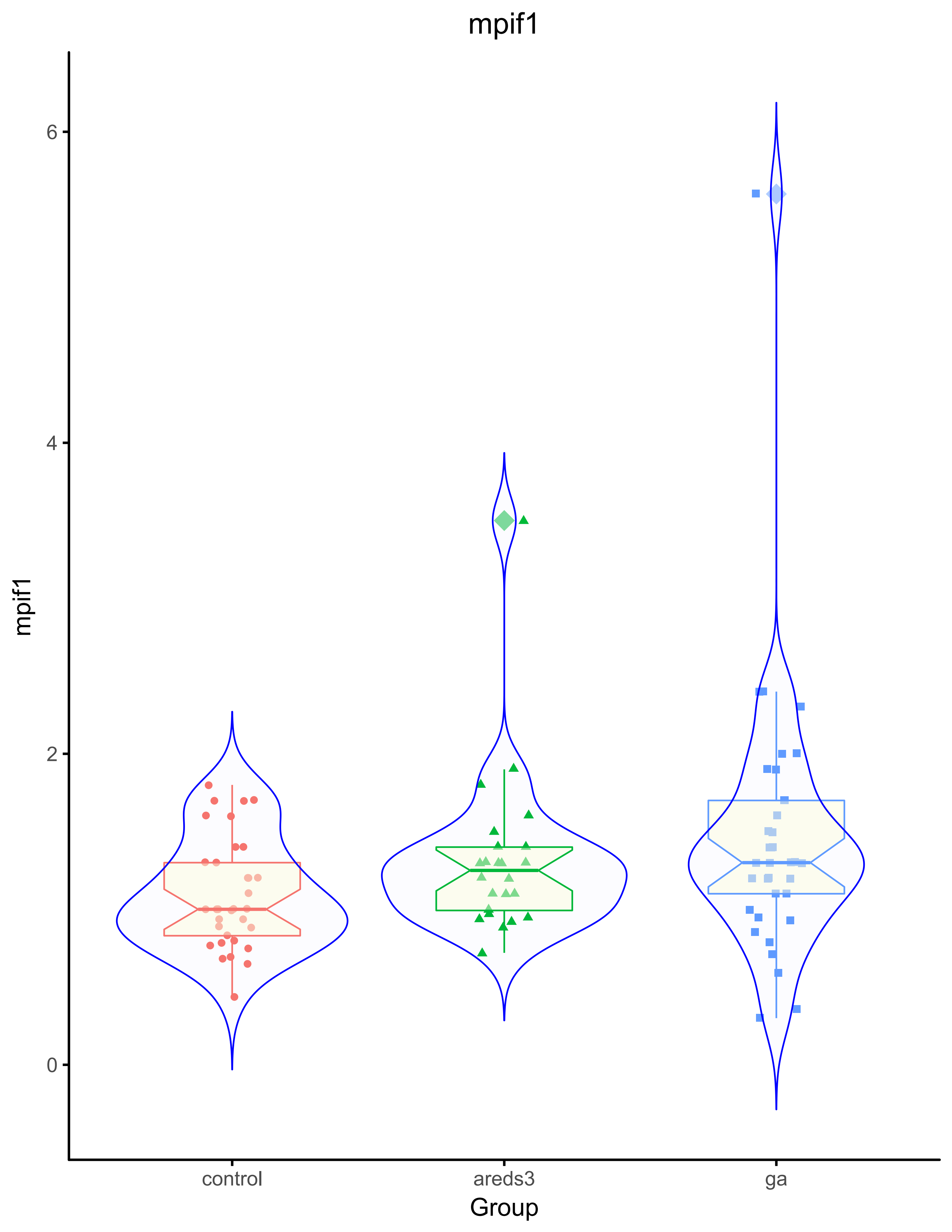

mpif1
mpif1
6
4
2
0
control
areds3
ga
Group

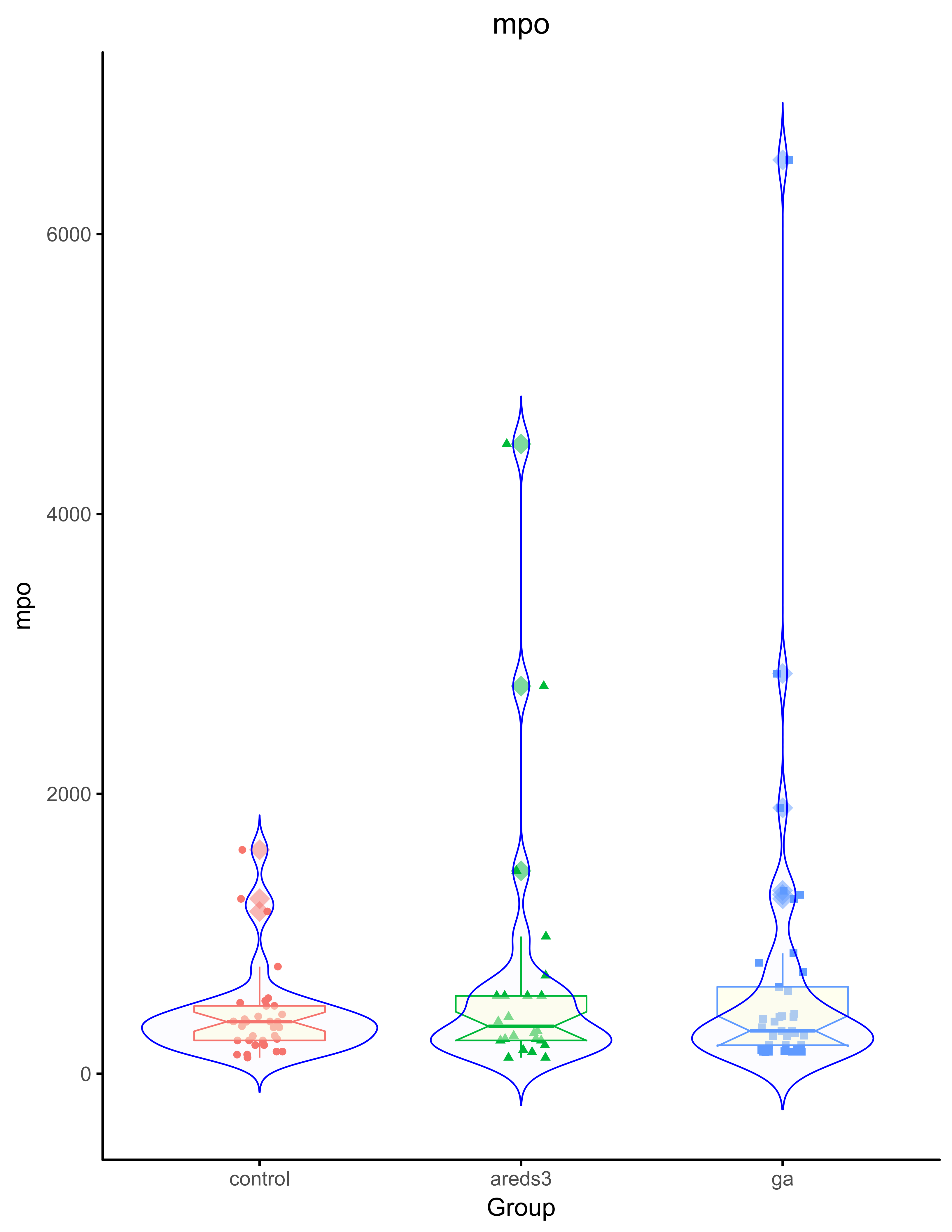

mpo
6000
4000
2000
0
mpo
control
areds3
ga
Group

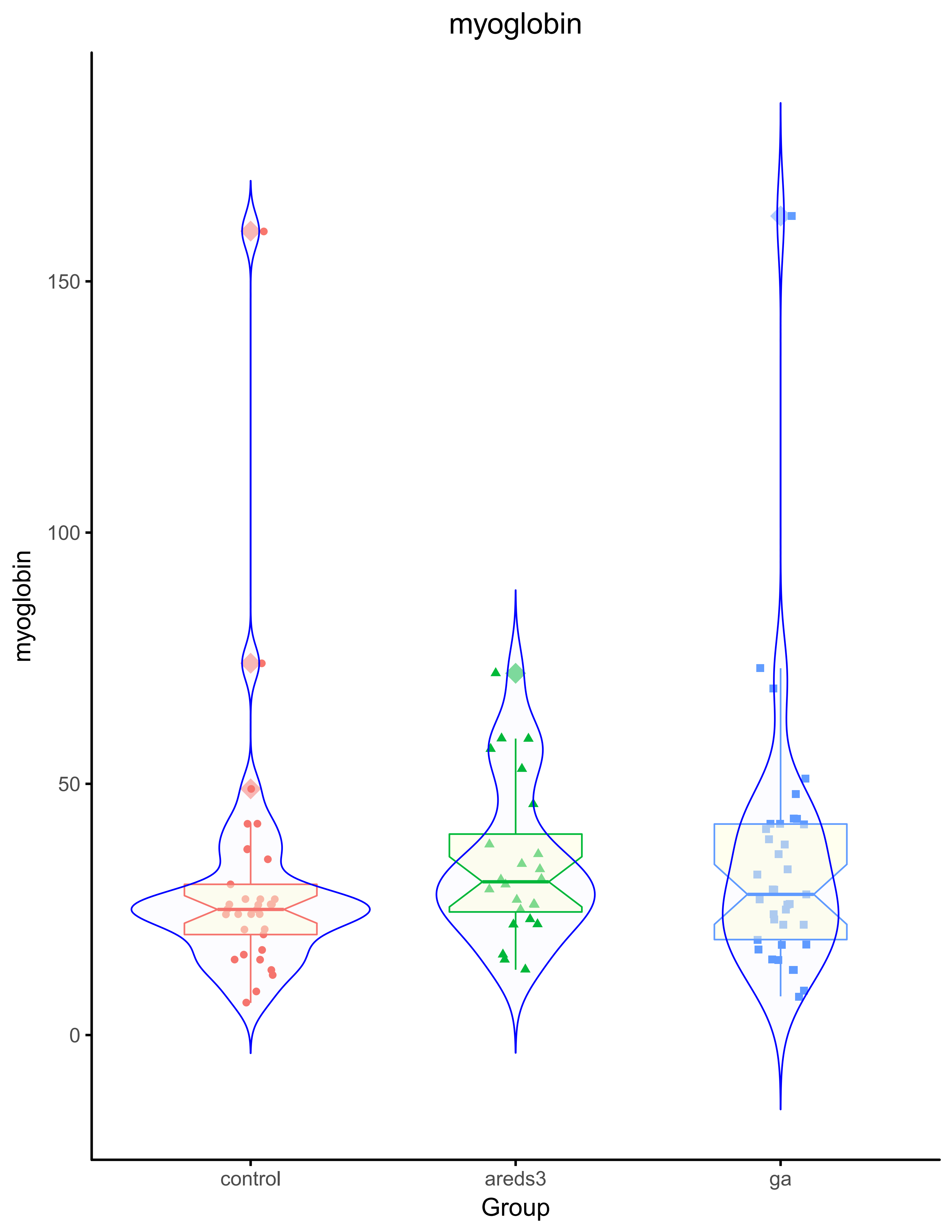

myoglobin
150
100
50
0
myoglobin
control
areds3
ga
Group

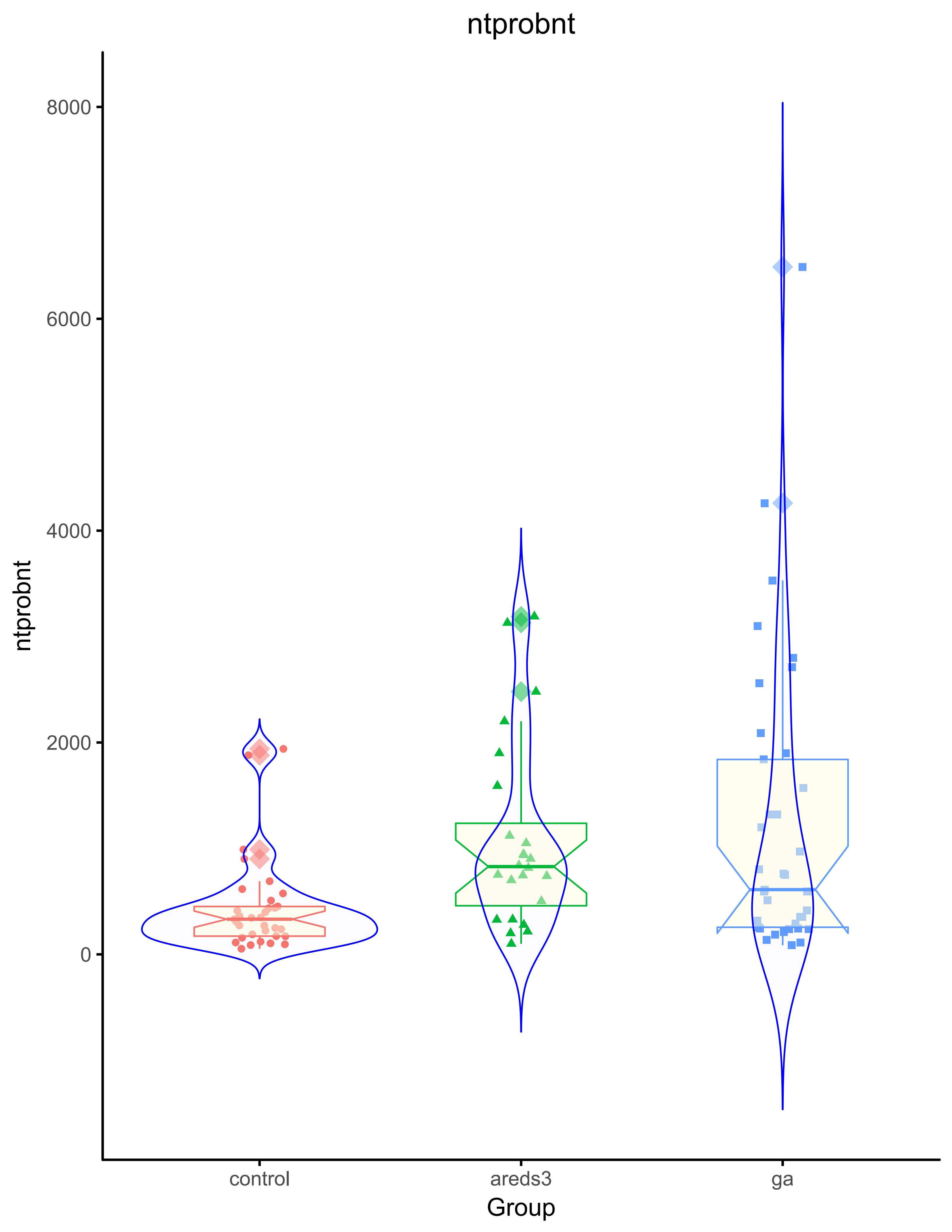

ntprobnt
8000
6000
4000
2000
0
ntprobnt
control
areds3
ga
Group

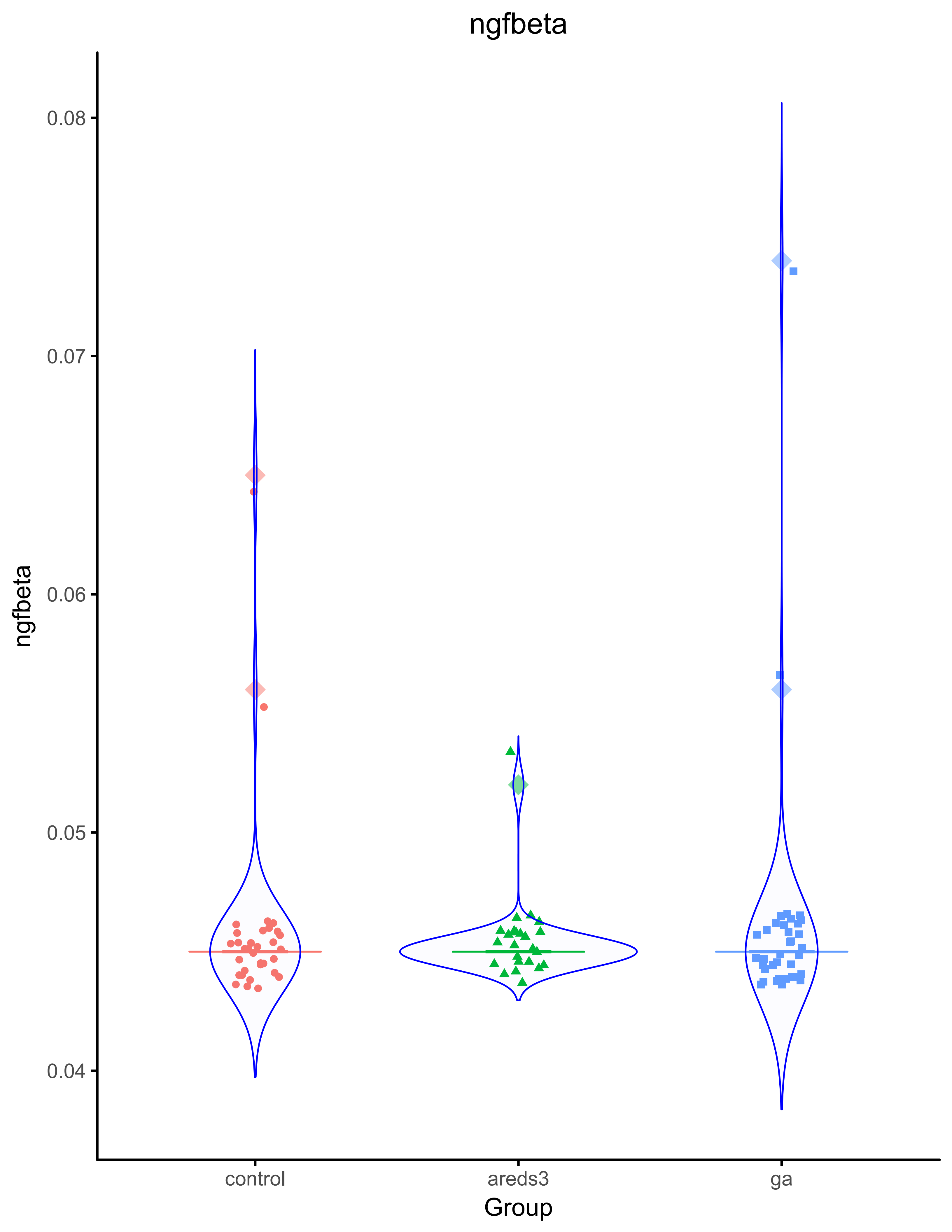

ngfbeta
0.08
0.07
0.06
0.05
0.04
ngfbeta
control
areds3
ga
Group

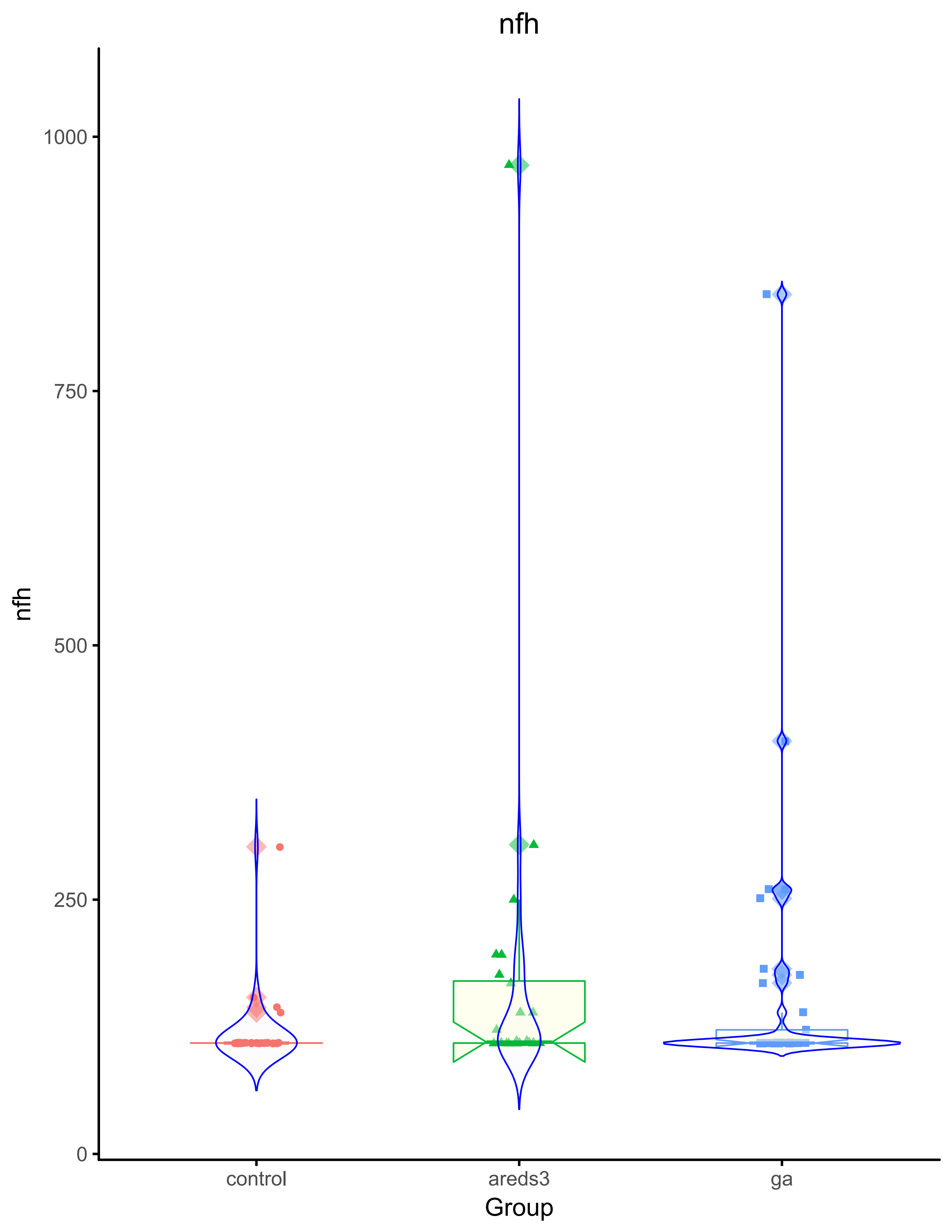
nfh
nfh
1000
750
500
250
0
control
areds3
ga
Group

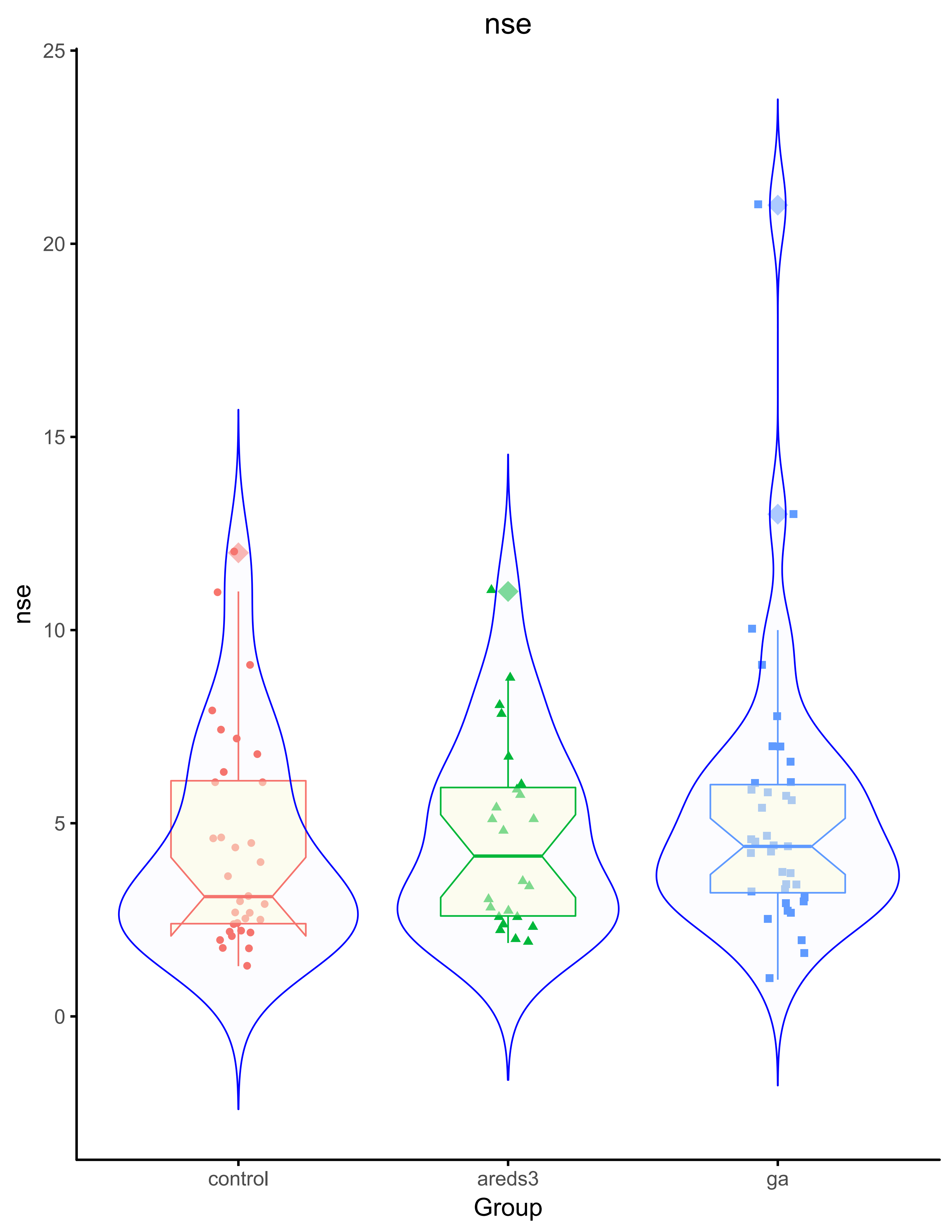

nse
25
20
15
10
5
0
nse
control
areds3
ga
Group

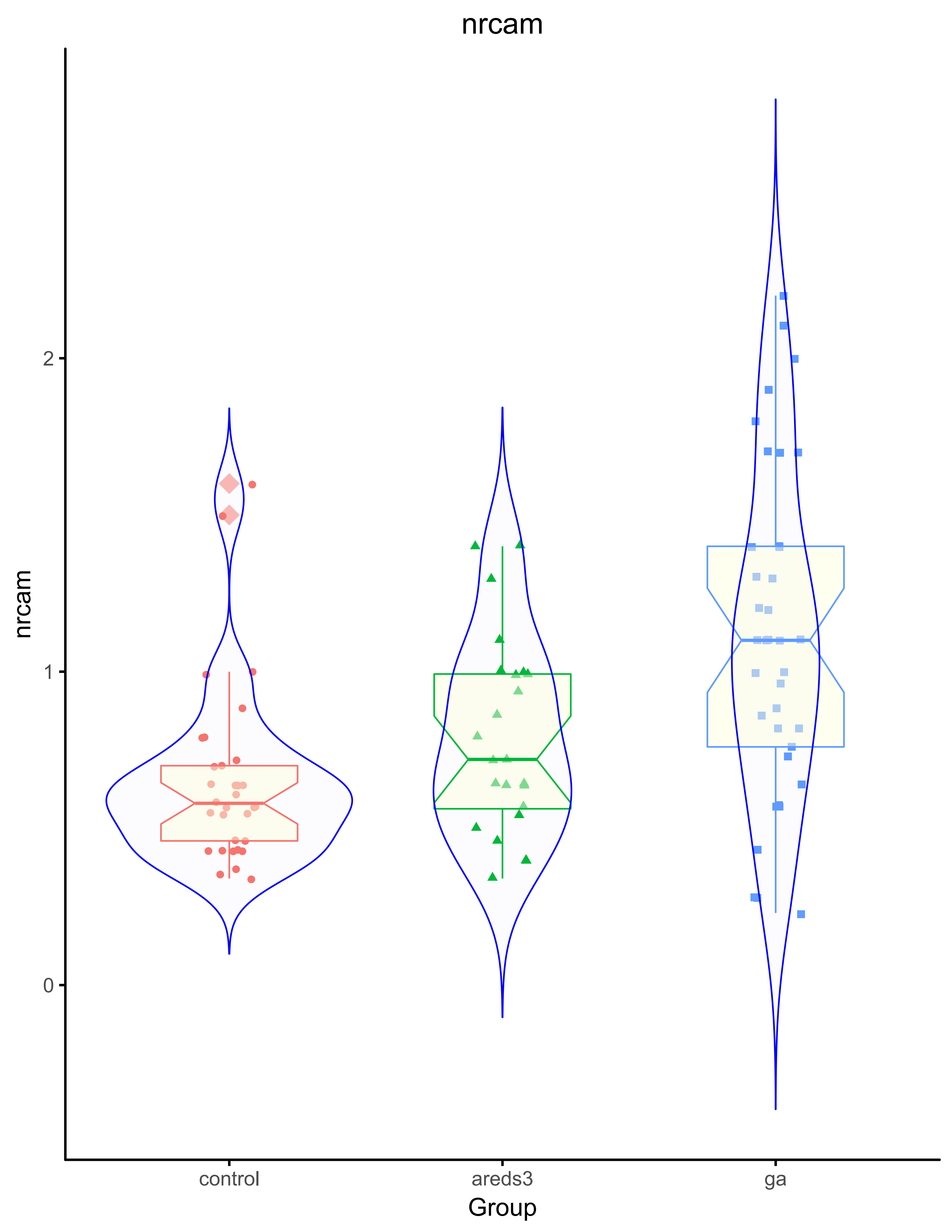

nrcam
2
1
0
nrcam
control
areds3
ga
Group

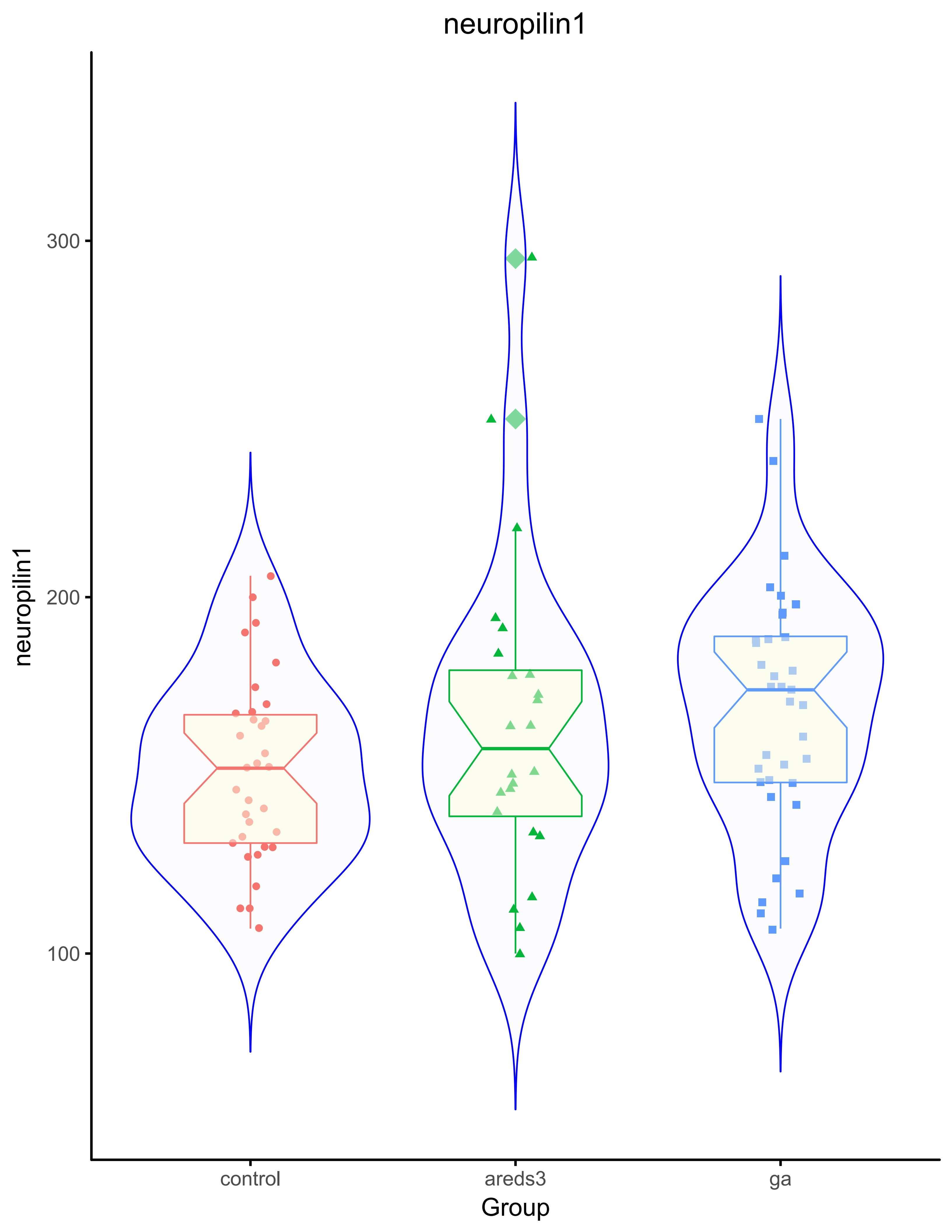

neuropilin1
300
200
100
neuropilin1
control
areds3
ga
Group

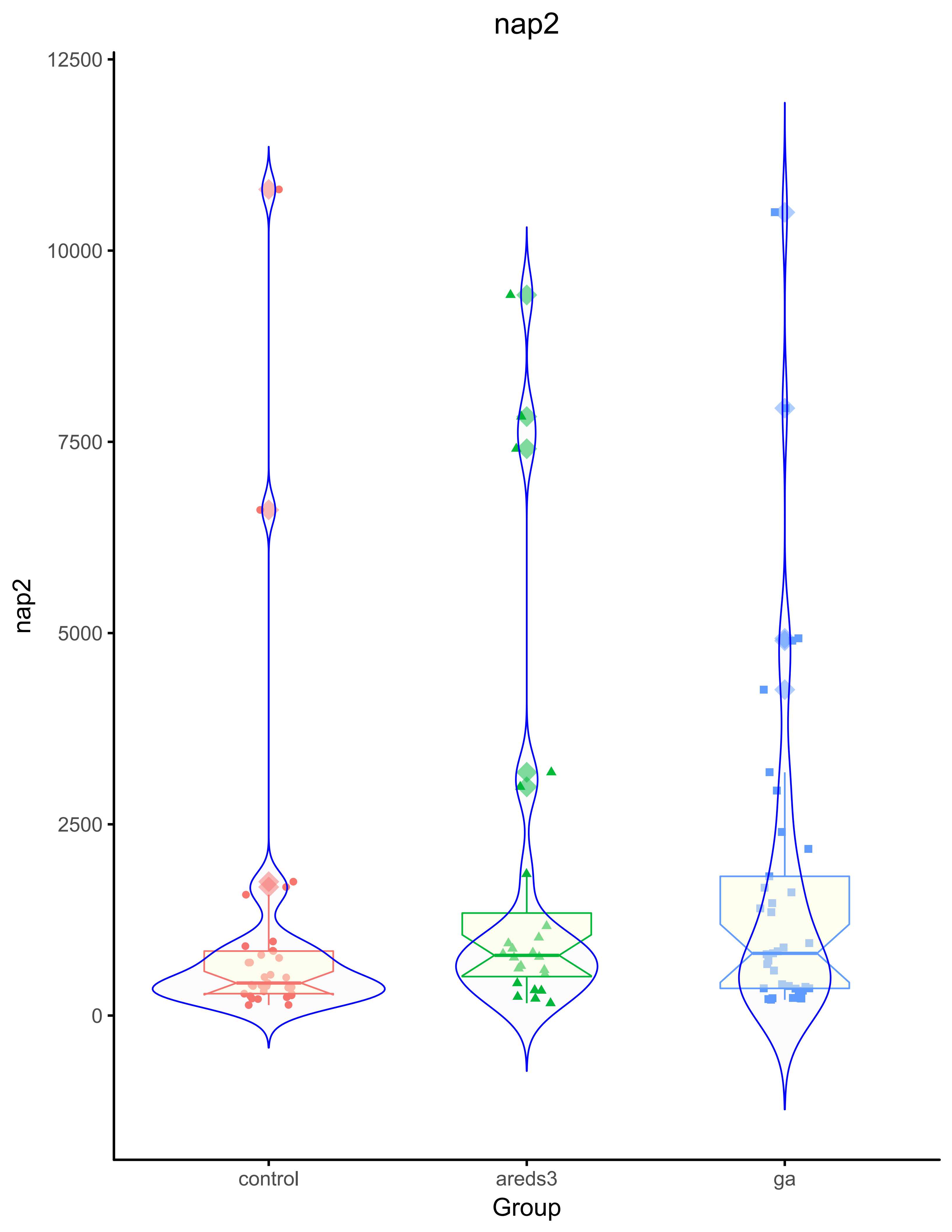
nap2
12500
10000
7500
5000
2500
0
nap2
control
areds3
ga
Group

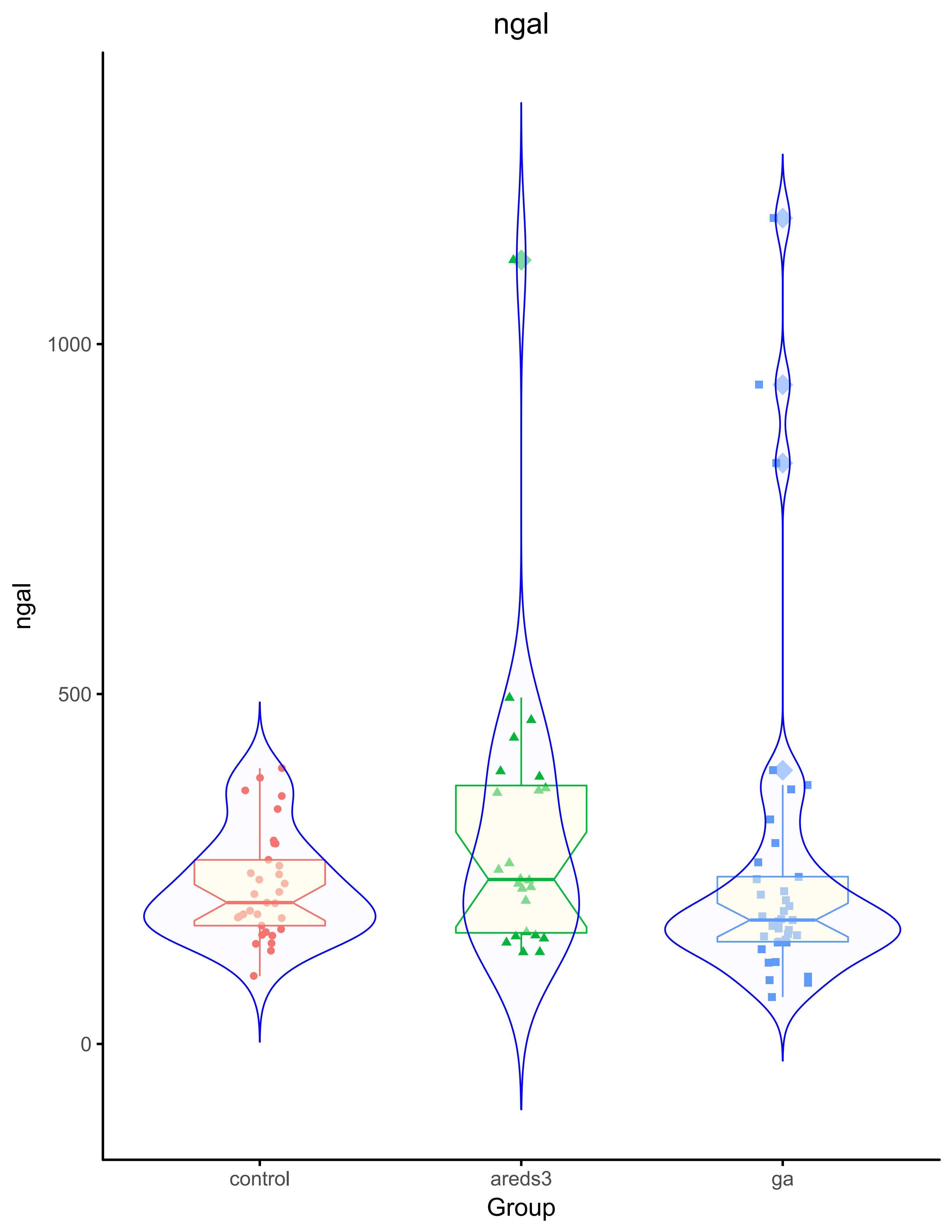
ngal
1000
500
0
ngal
control
areds3
ga
Group

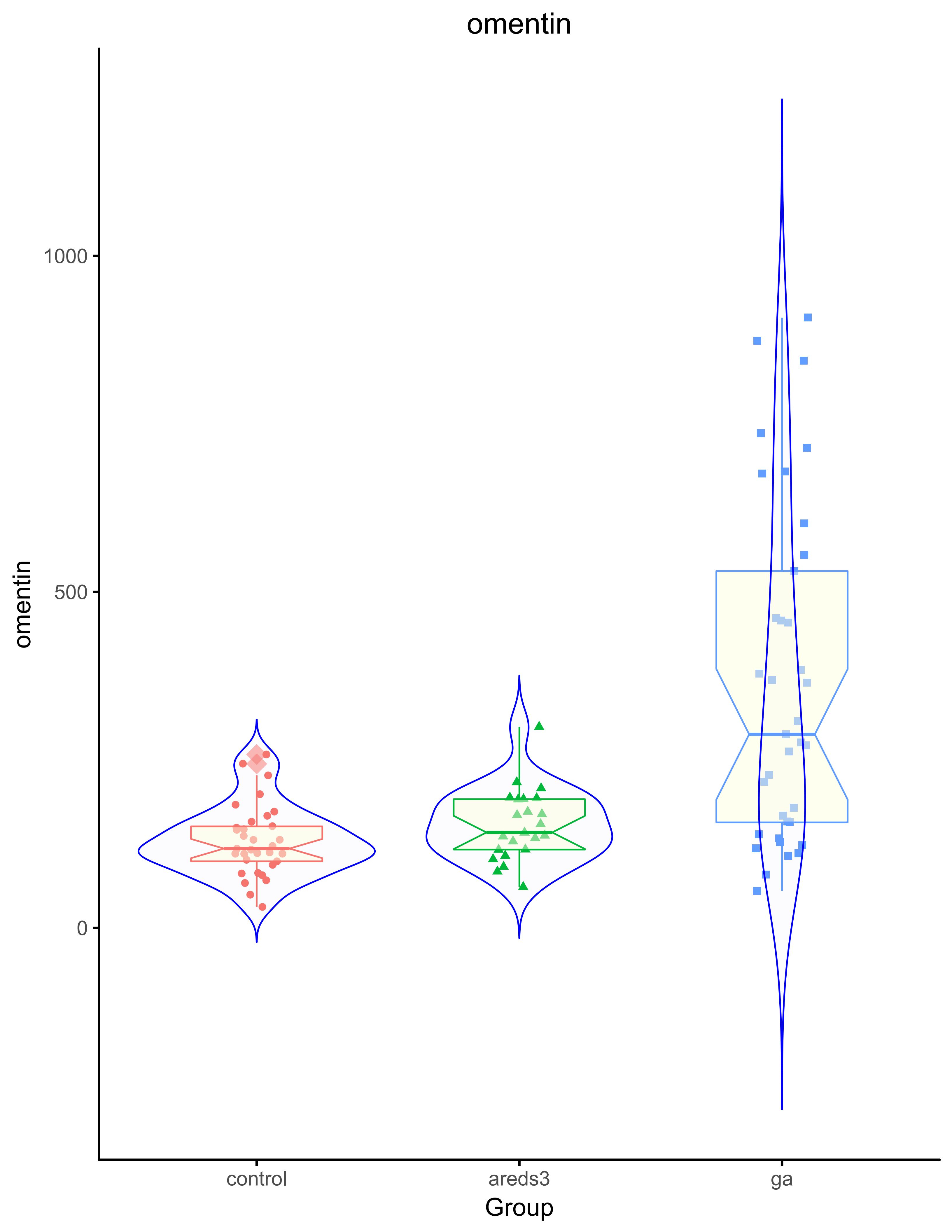
omentin
omentin
1000
500
0
control
areds3
ga
Group

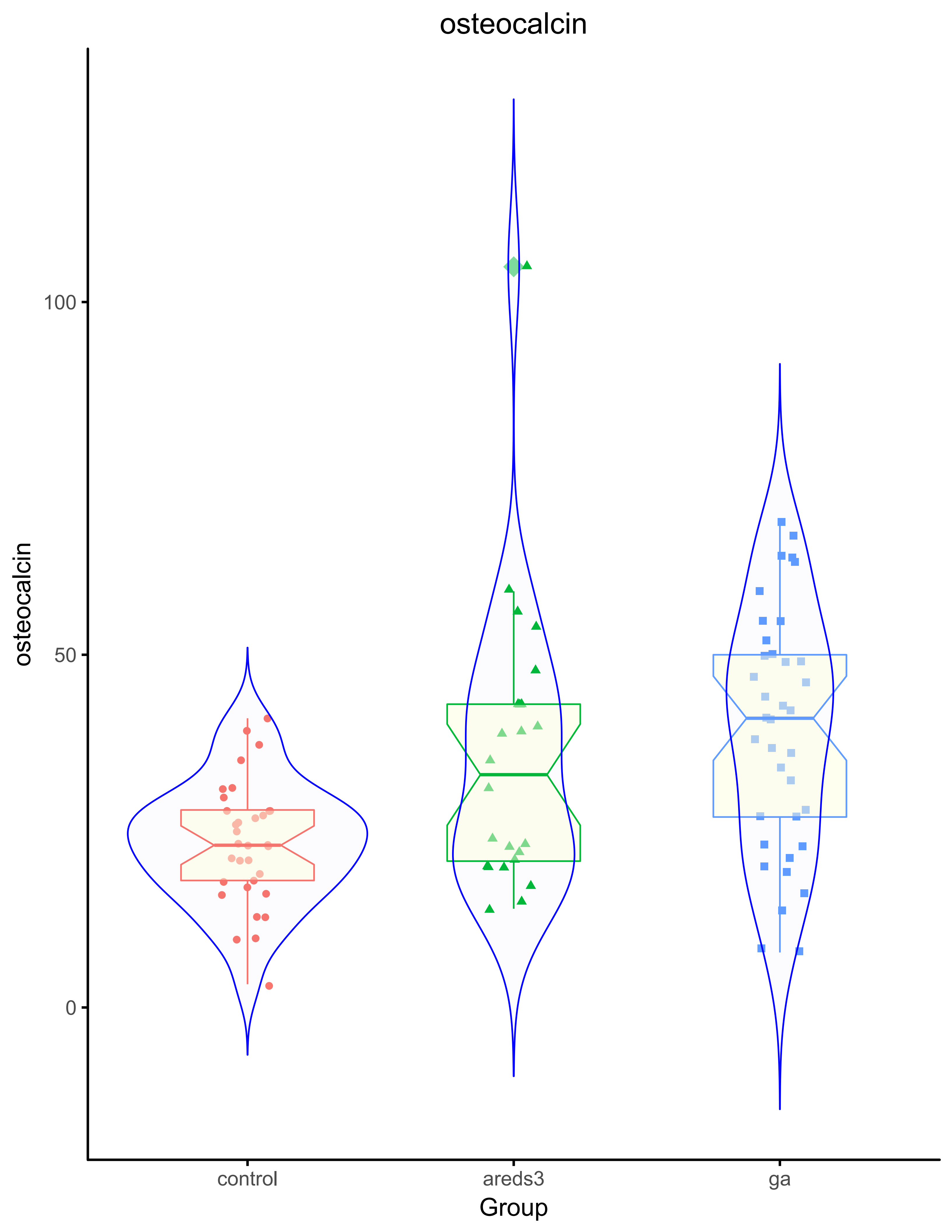
osteocalcin
osteocalcin
100
50
0
control
areds3
ga
Group

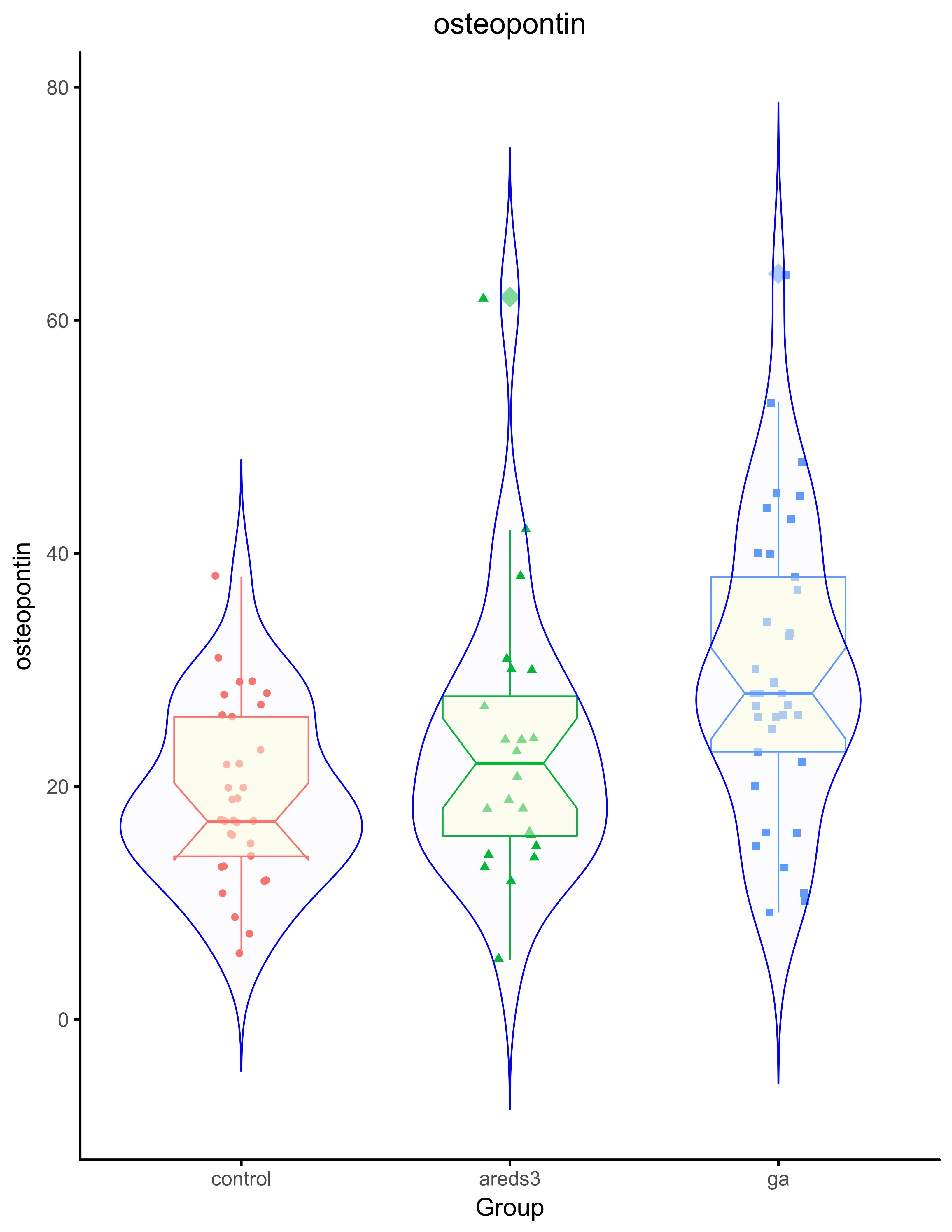

osteopontin
80
60
40
20
0
osteopontin
control
areds3
ga
Group

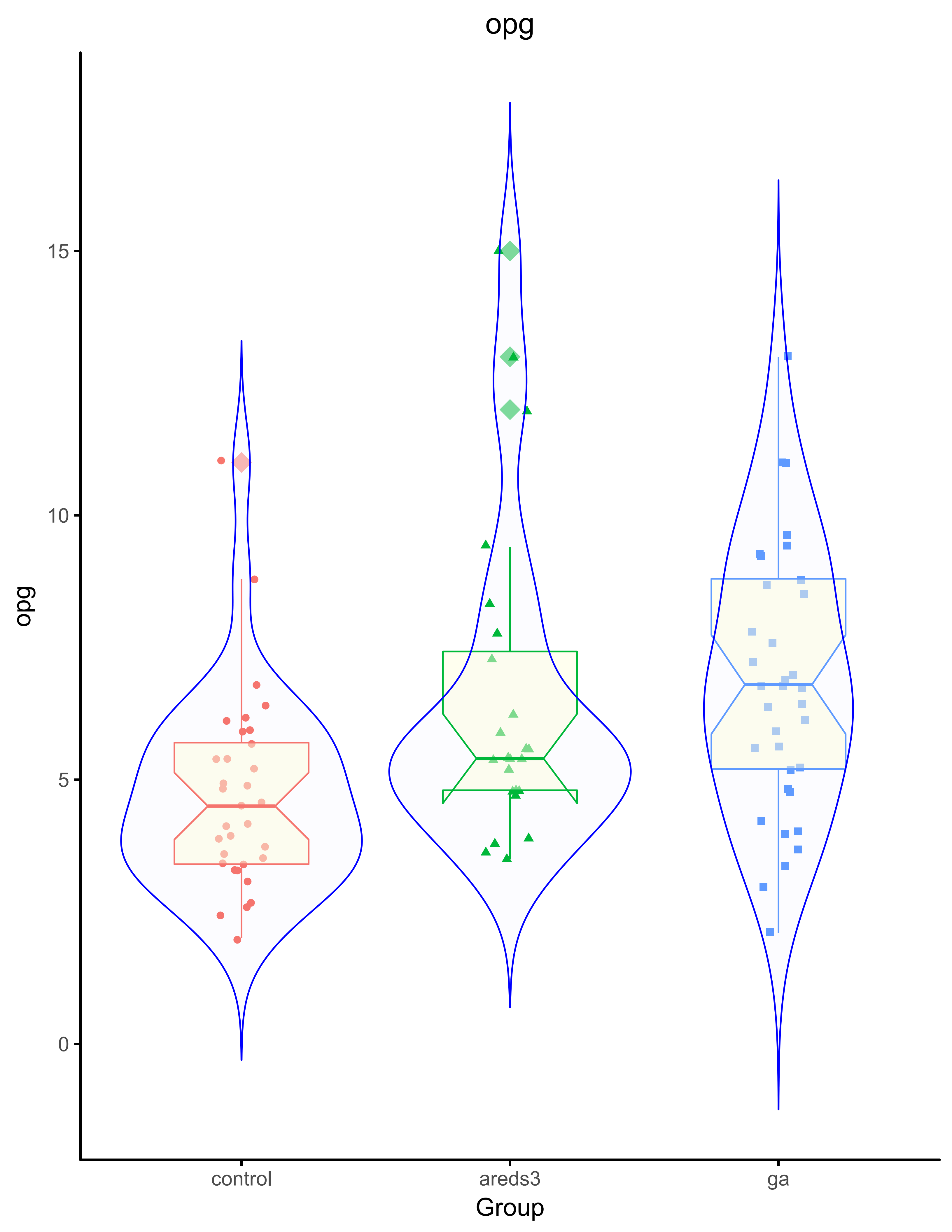

opg
15
10
5
0
opg
control
areds3
ga
Group

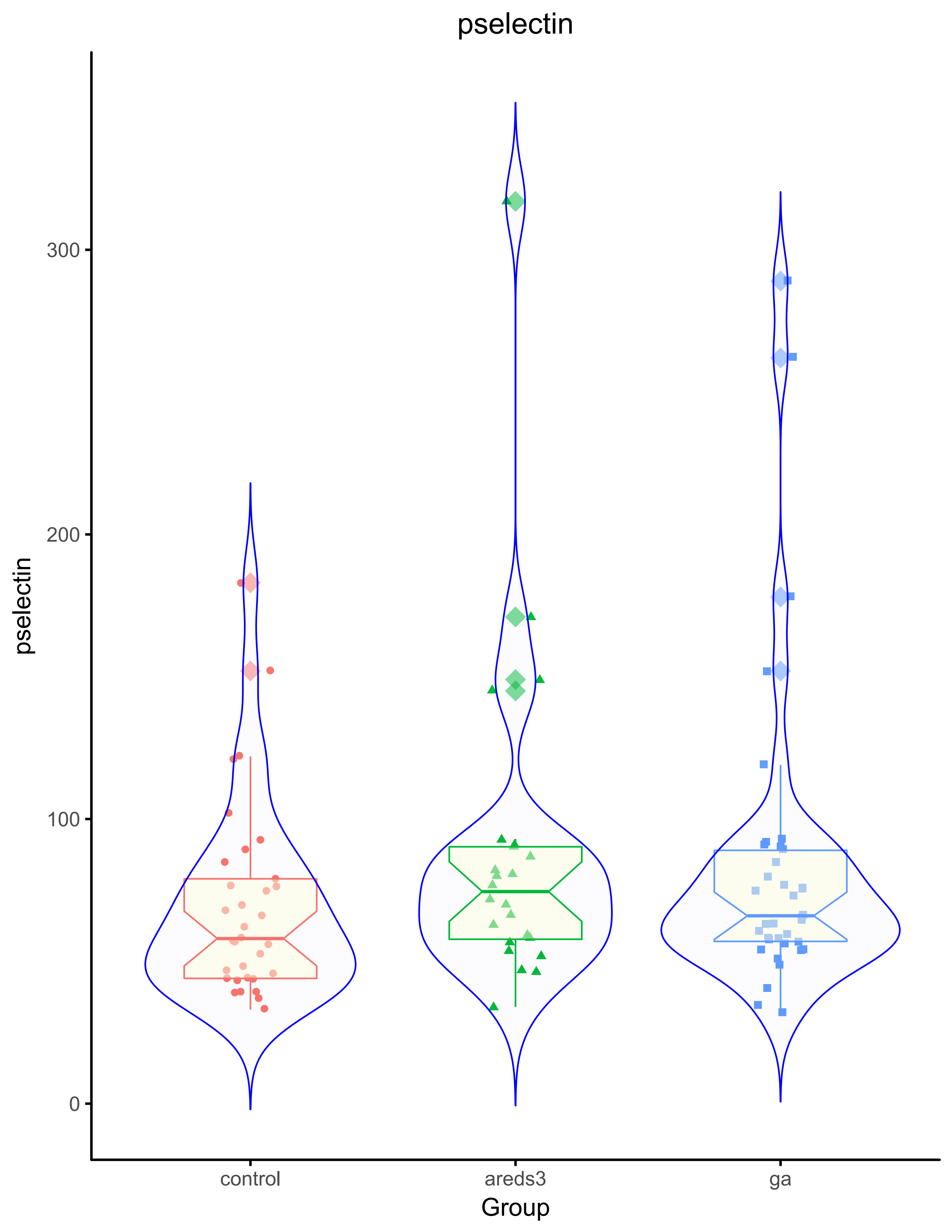

pselectin
300
200
100
0
pselectin
control
areds3
ga
Group

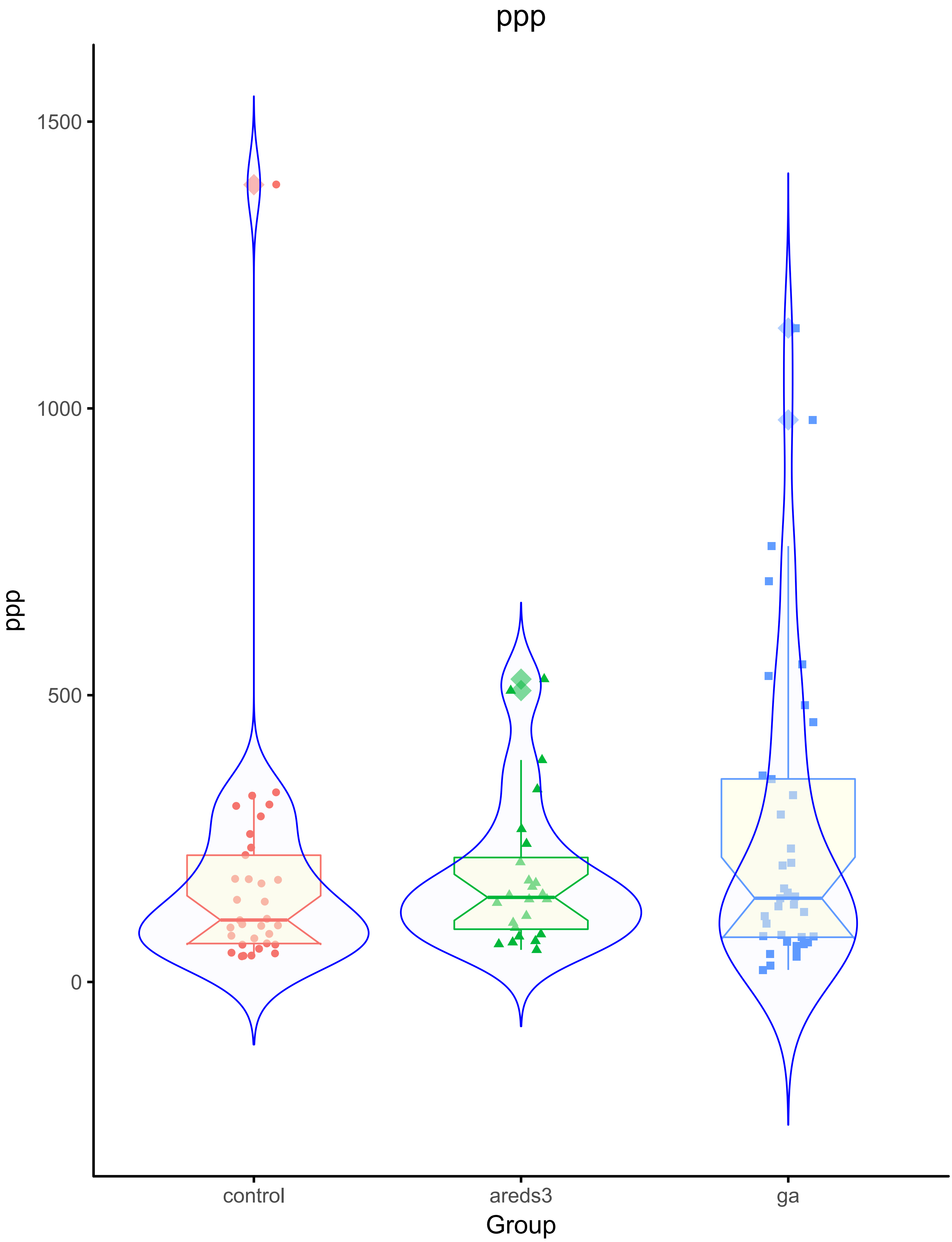

ppp
1500
1000
500
0
ppp
control
areds3
ga
Group

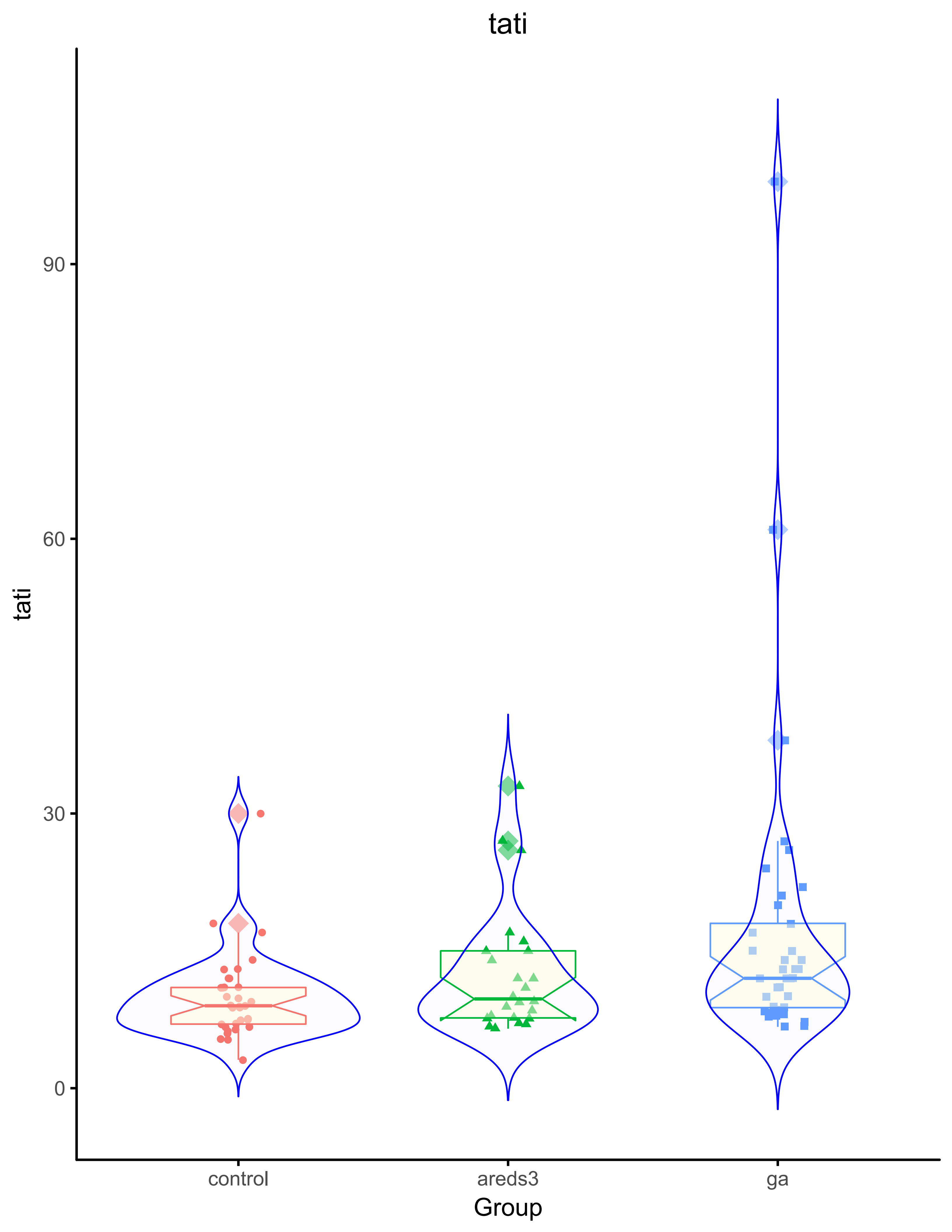
tati
90
60
30
0
tati
control
areds3
ga
Group

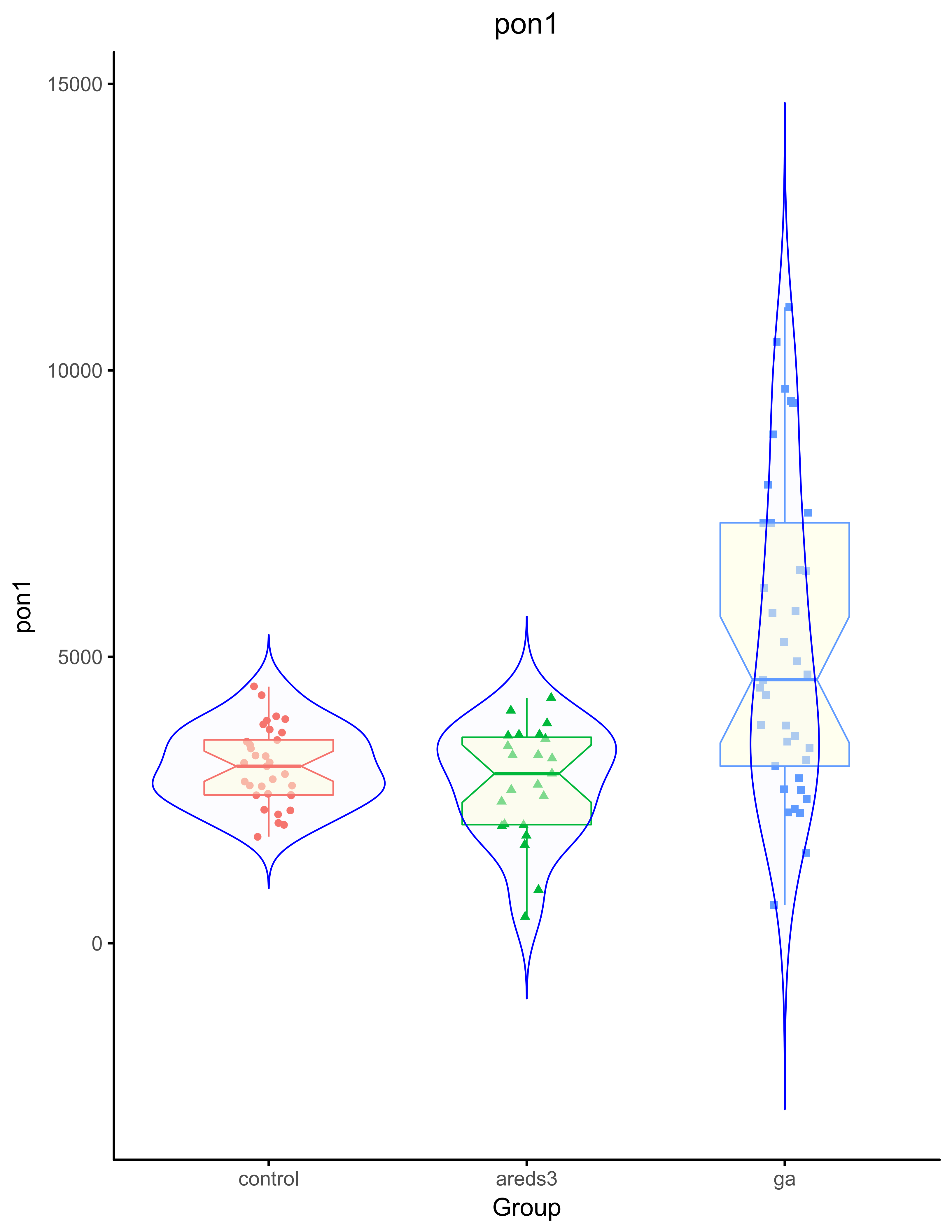
pon1
15000
10000
5000
0
pon1
control
areds3
ga
Group

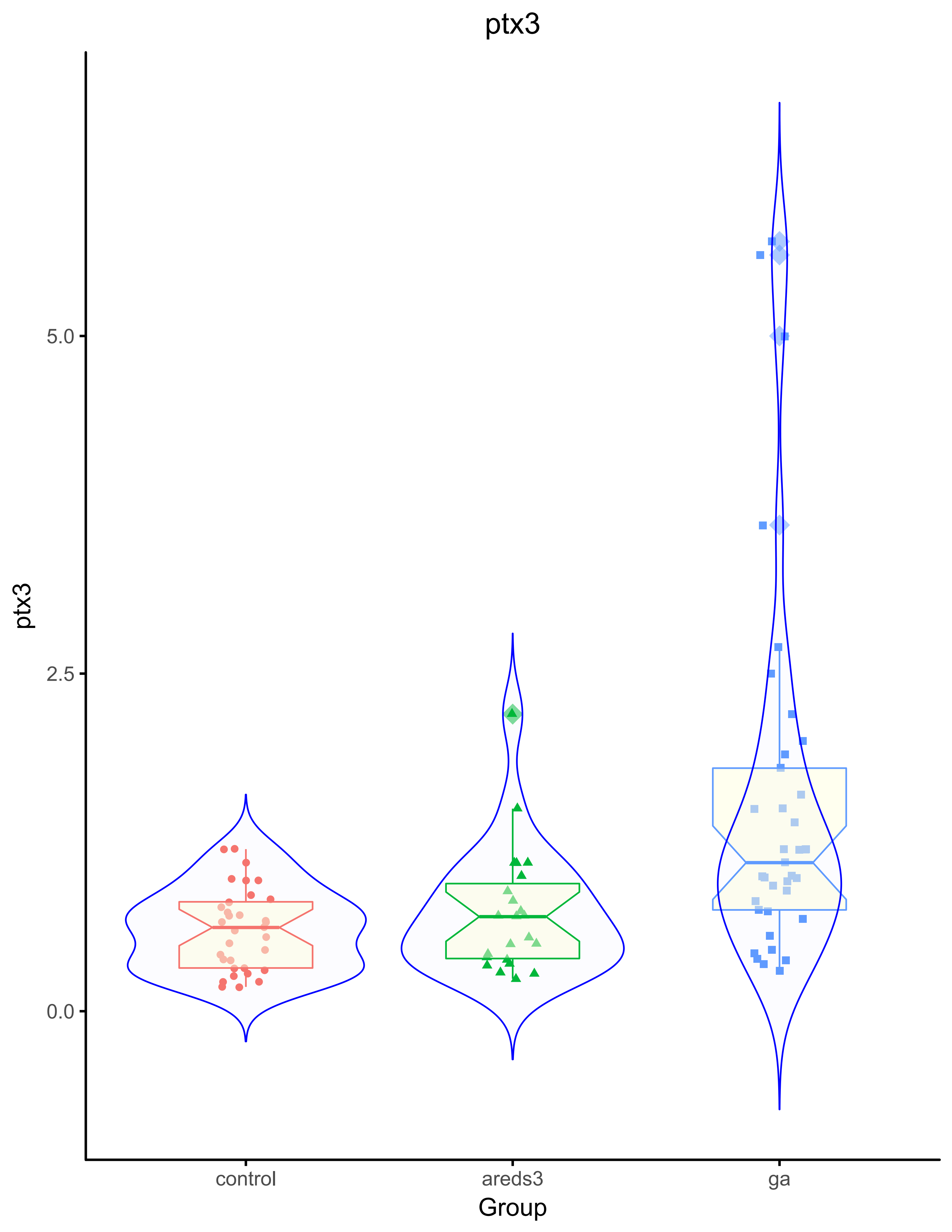
ptx3
ptx3
5.0
2.5
0.0
control
areds3
ga
Group

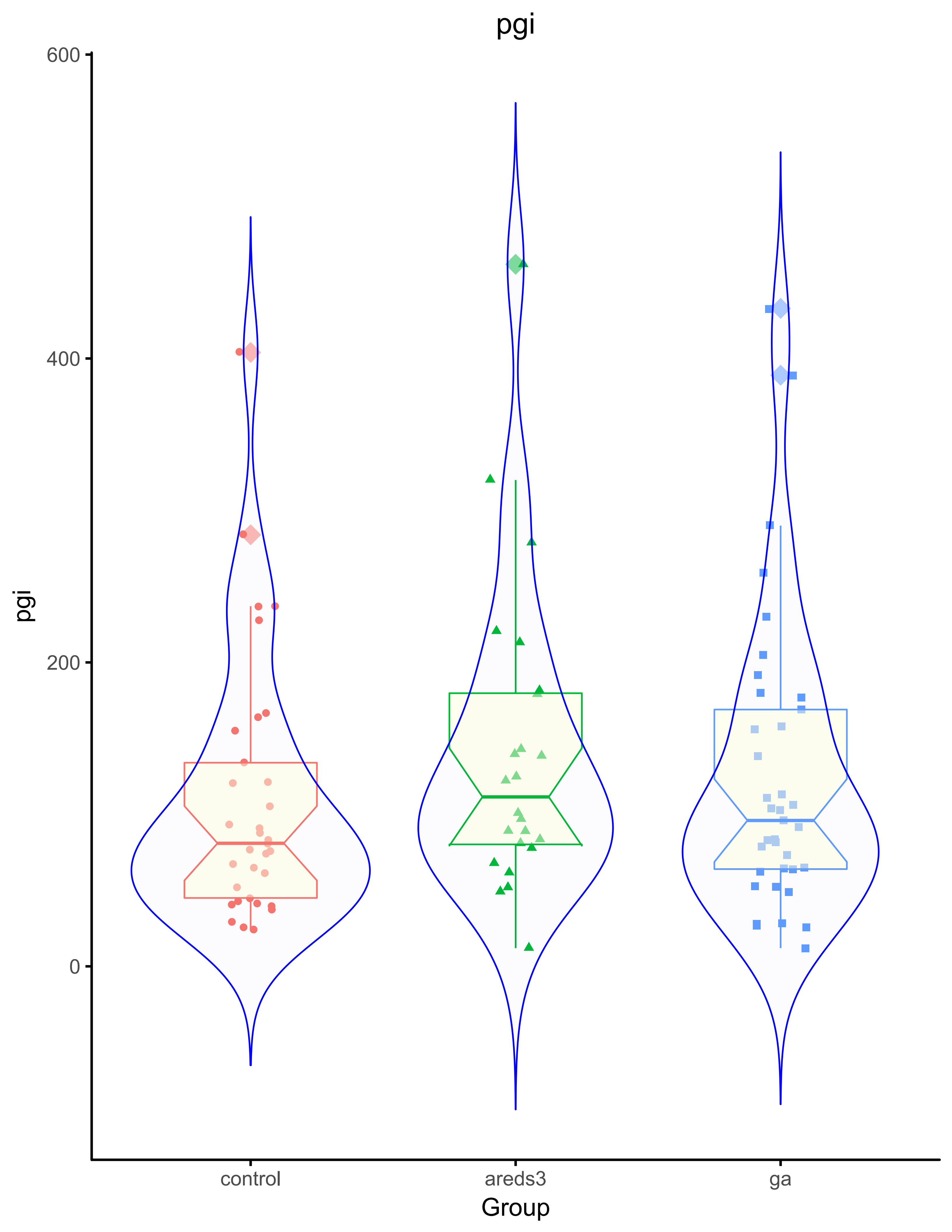

pgi
600
400
200
0
pgi
control
areds3
ga
Group

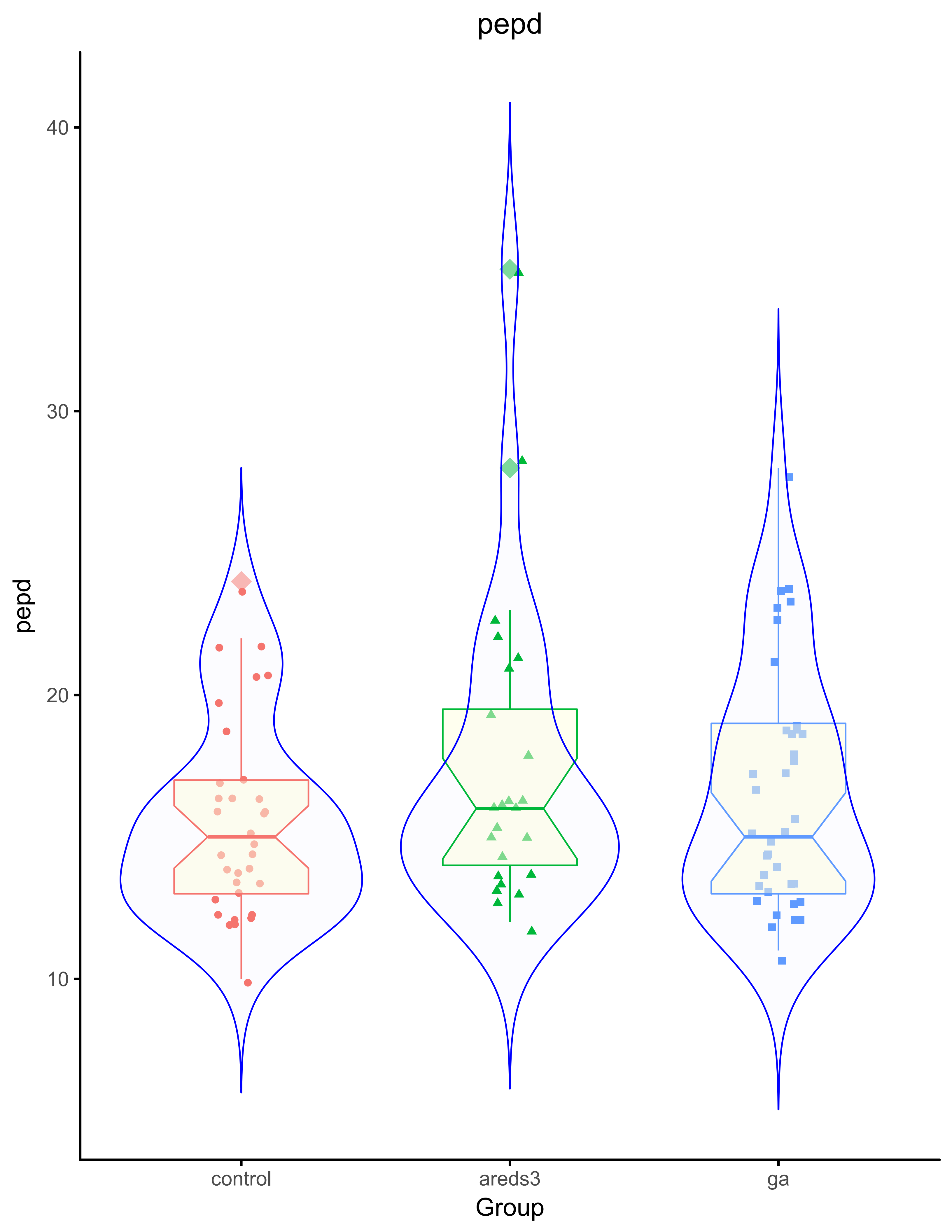
pepd
40
30
20
10
pepd
control
areds3
ga
Group

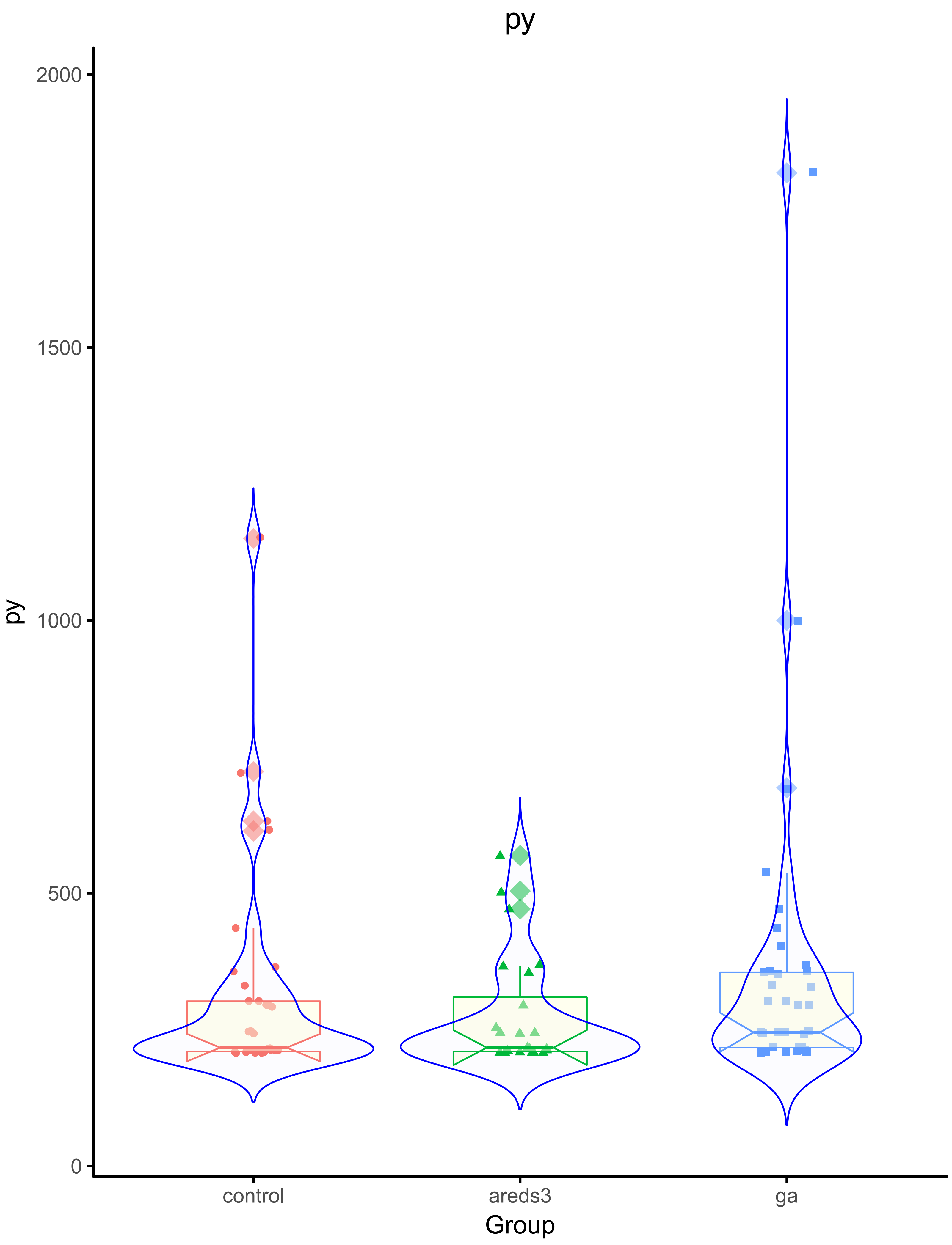
py
2000
1500
1000
500
0
py
control
areds3
ga
Group

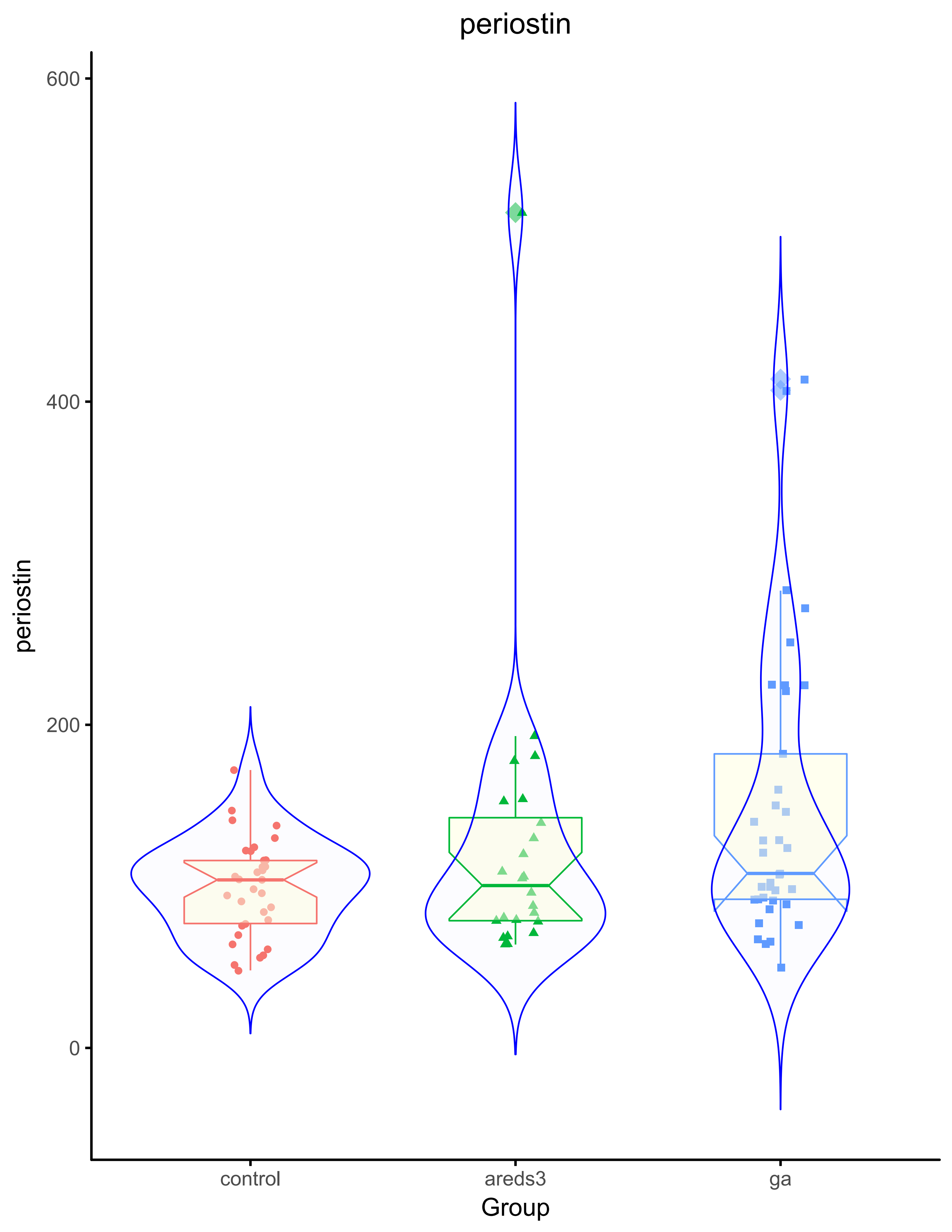
periostin
600
400
200
0
periostin
control
areds3
ga
Group

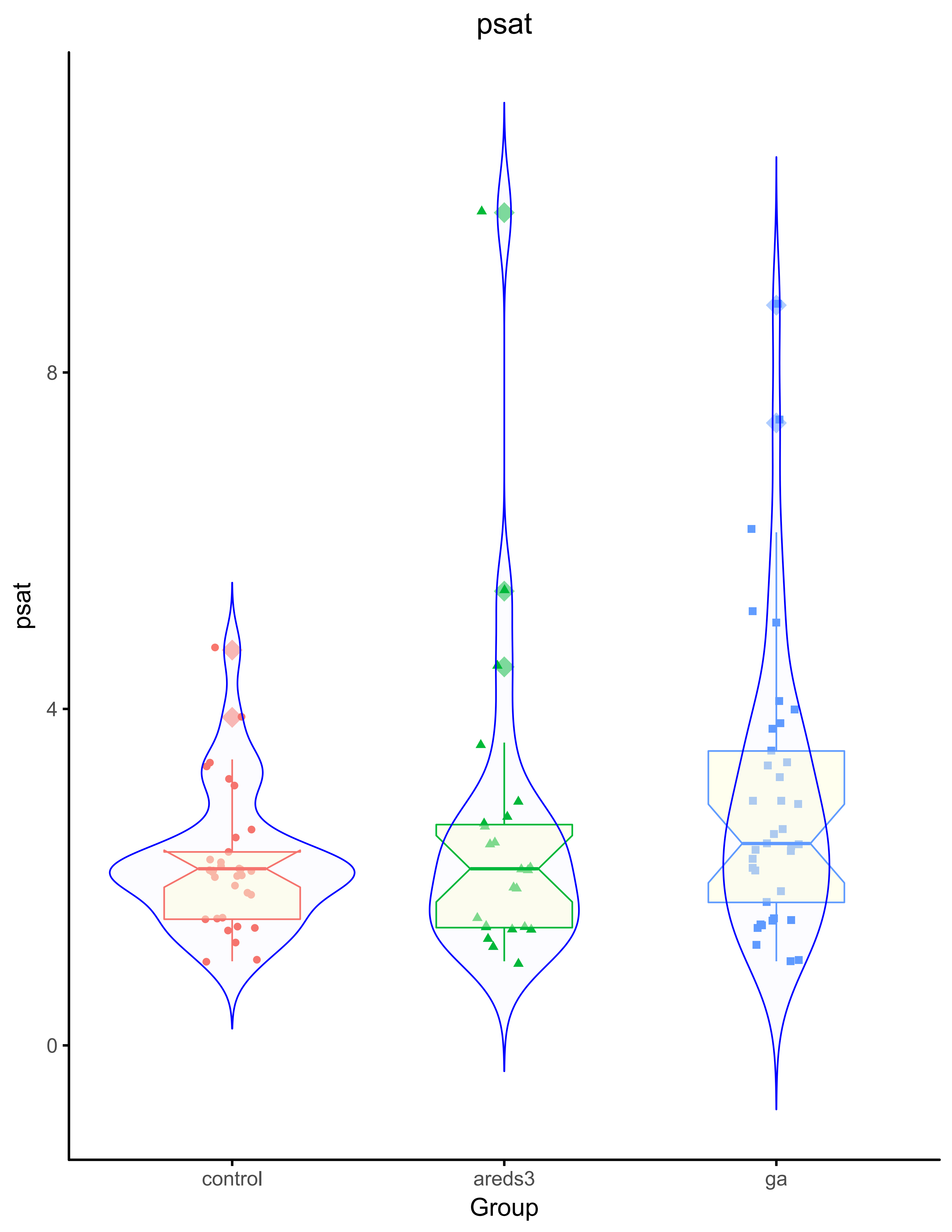

psat
8
4
0
psat
control
areds3
ga
Group

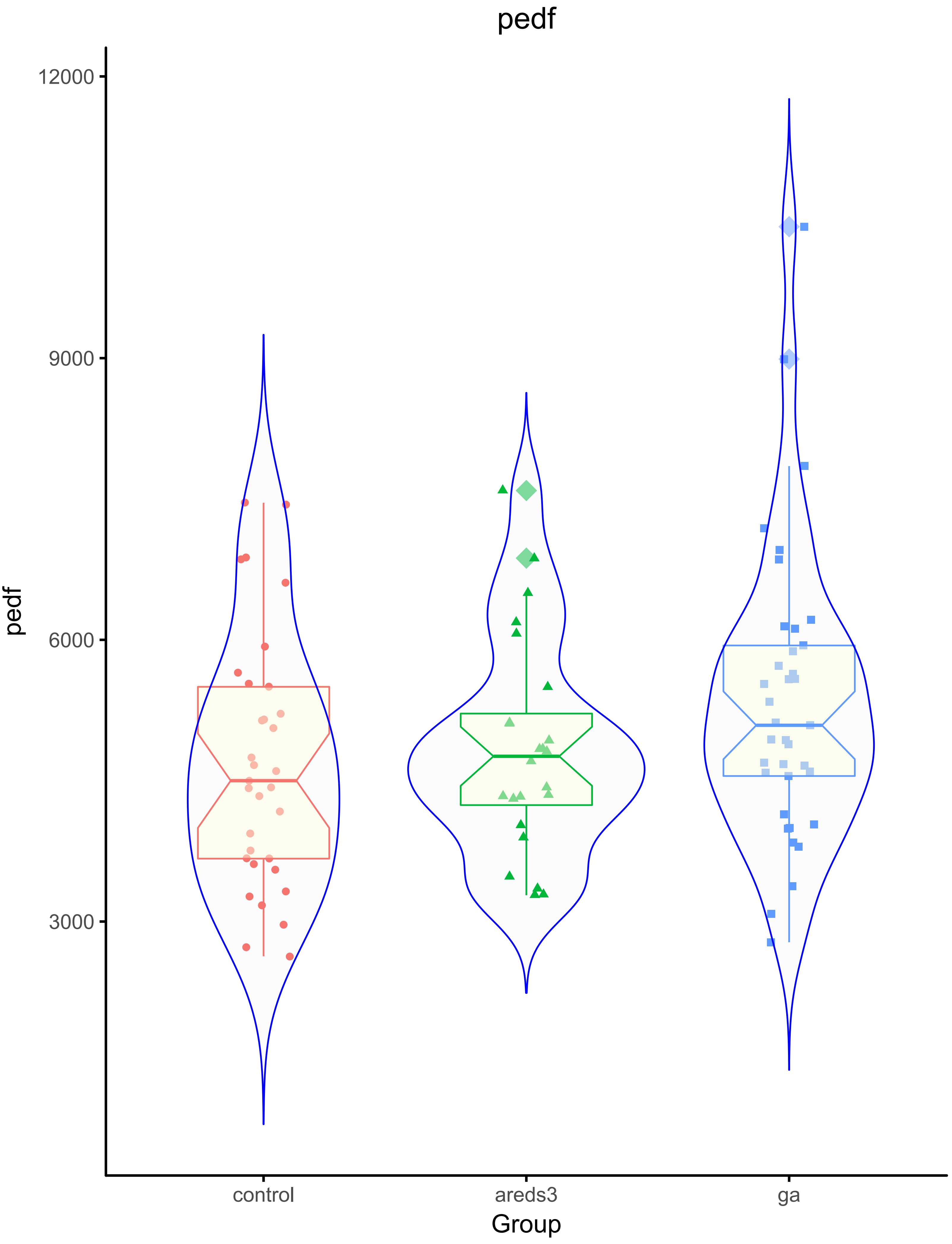

pedf
12000
9000
6000
3000
pedf
control
areds3
ga
Group

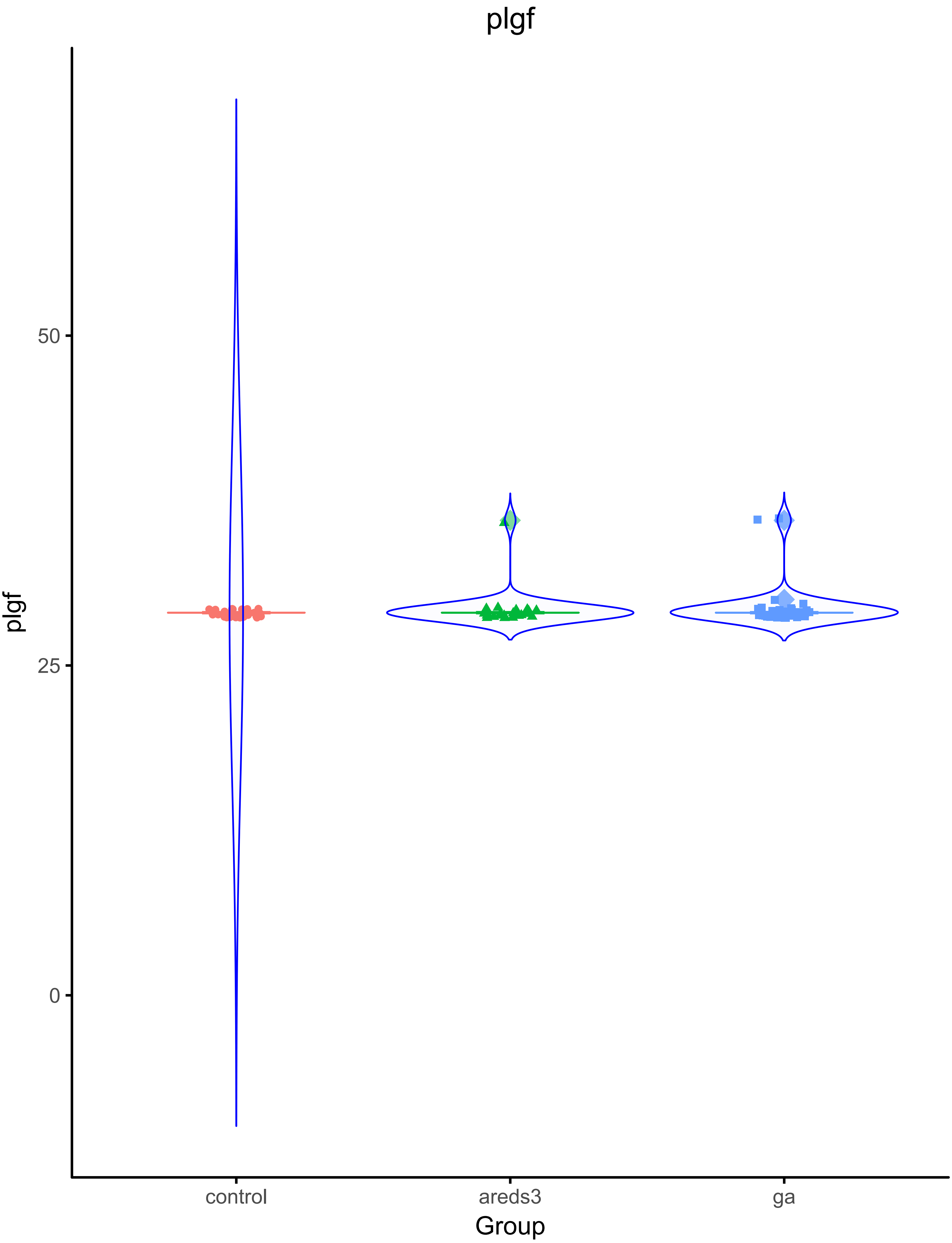
plgf
plgf
50
25
0
control
areds3
ga
Group

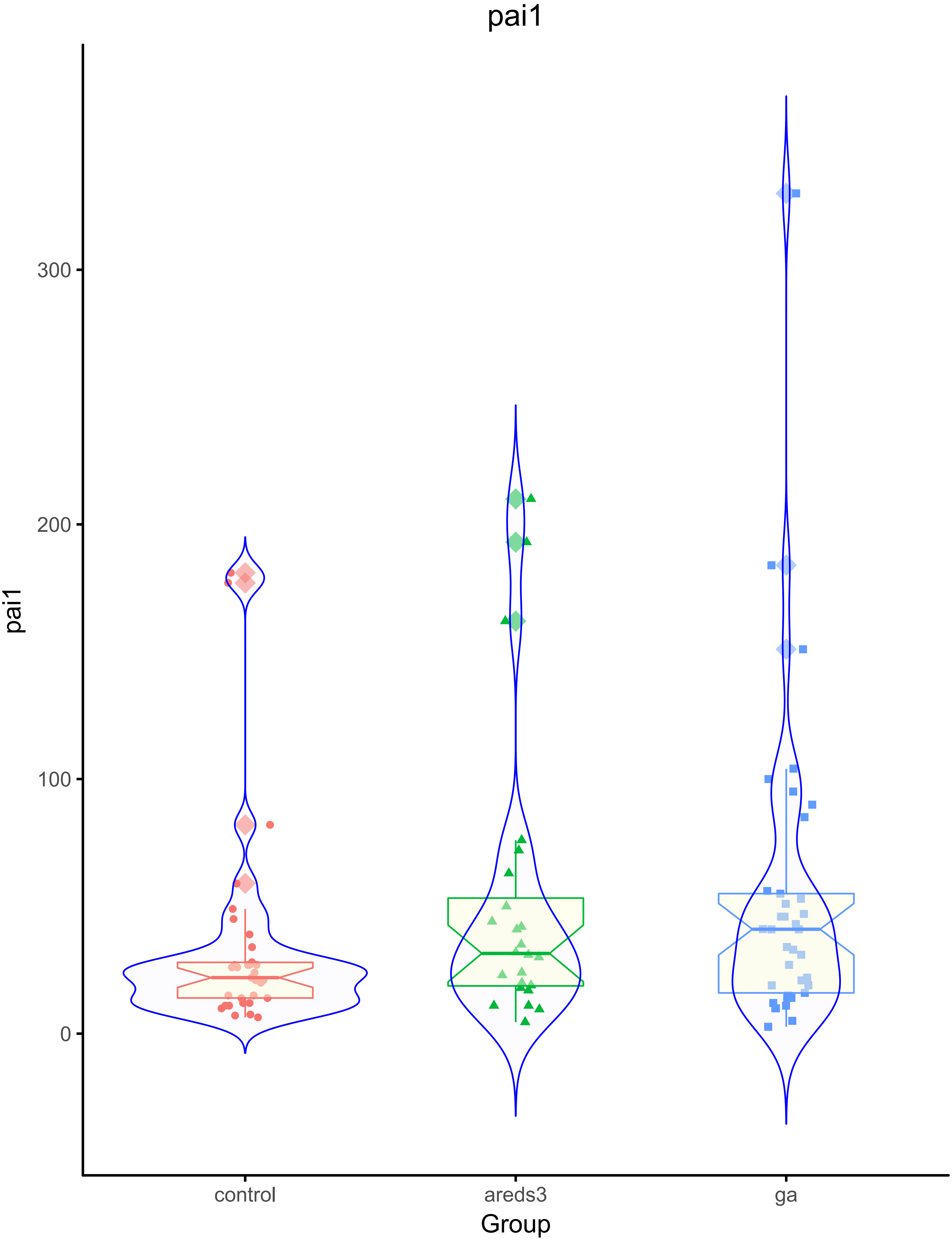
pai1
300
200
100
0
pai1
control
areds3
ga
Group

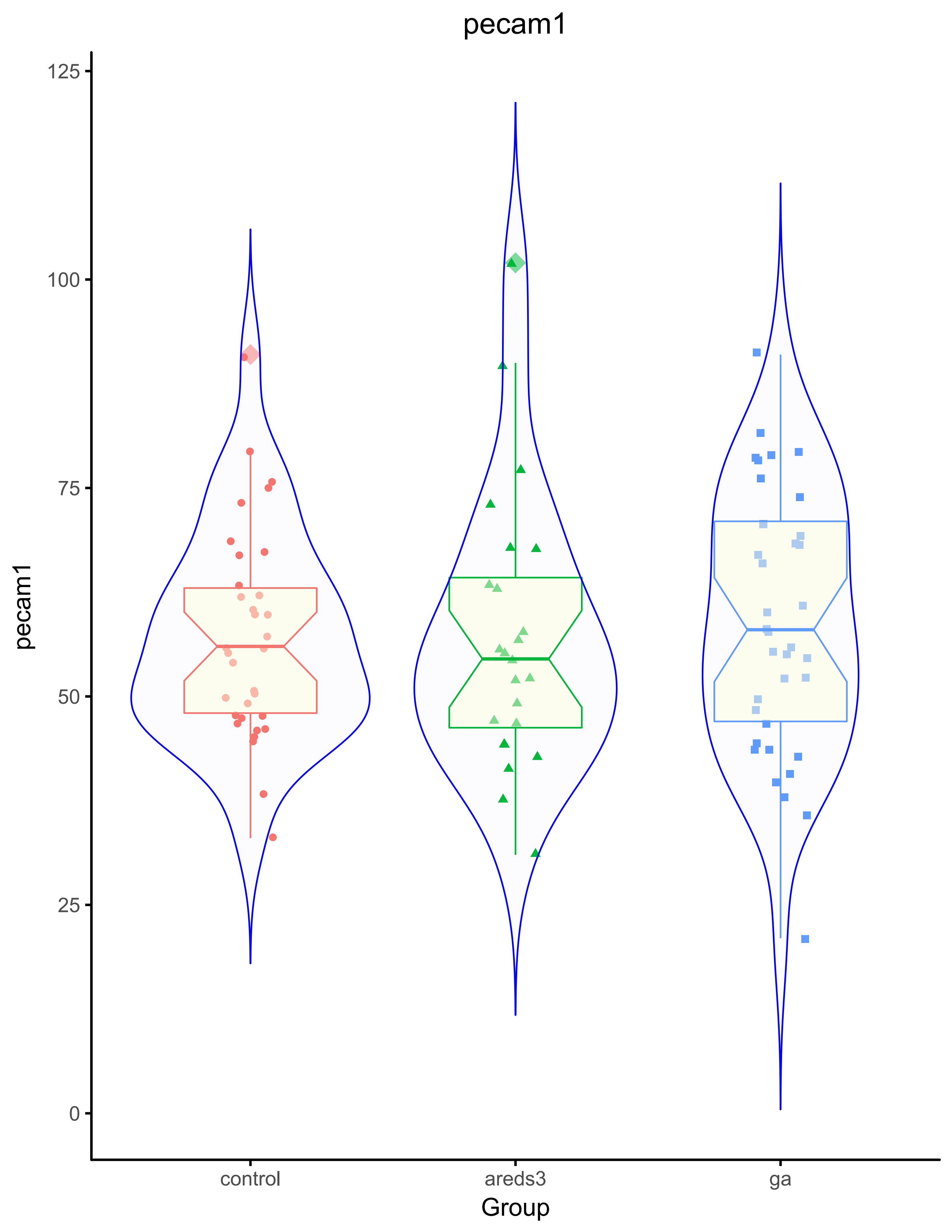

pecam1
125
100
75
50
25
0
pecam1
control
areds3
ga
Group

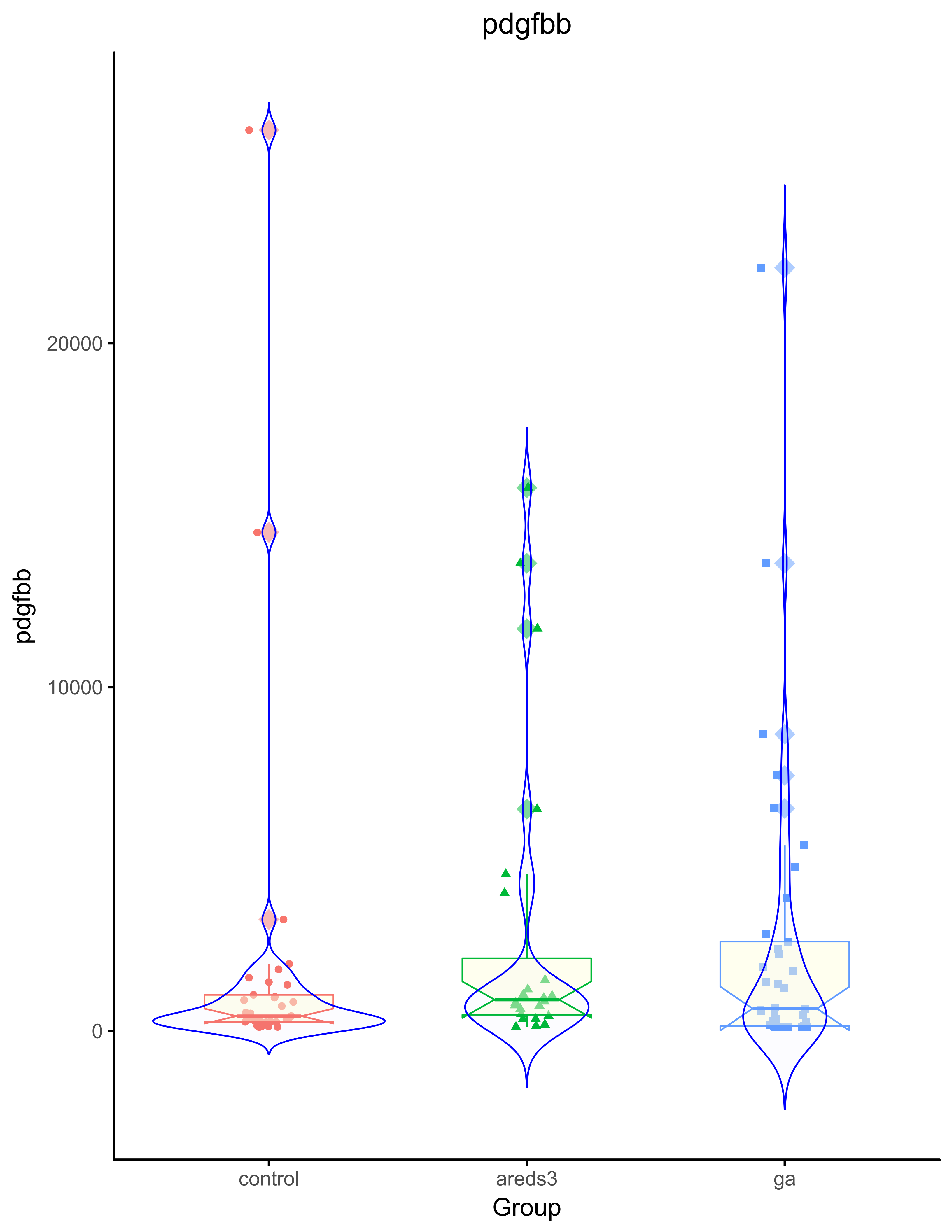

pdgfbb
20000
10000
0
pdgfbb
control
areds3
ga
Group

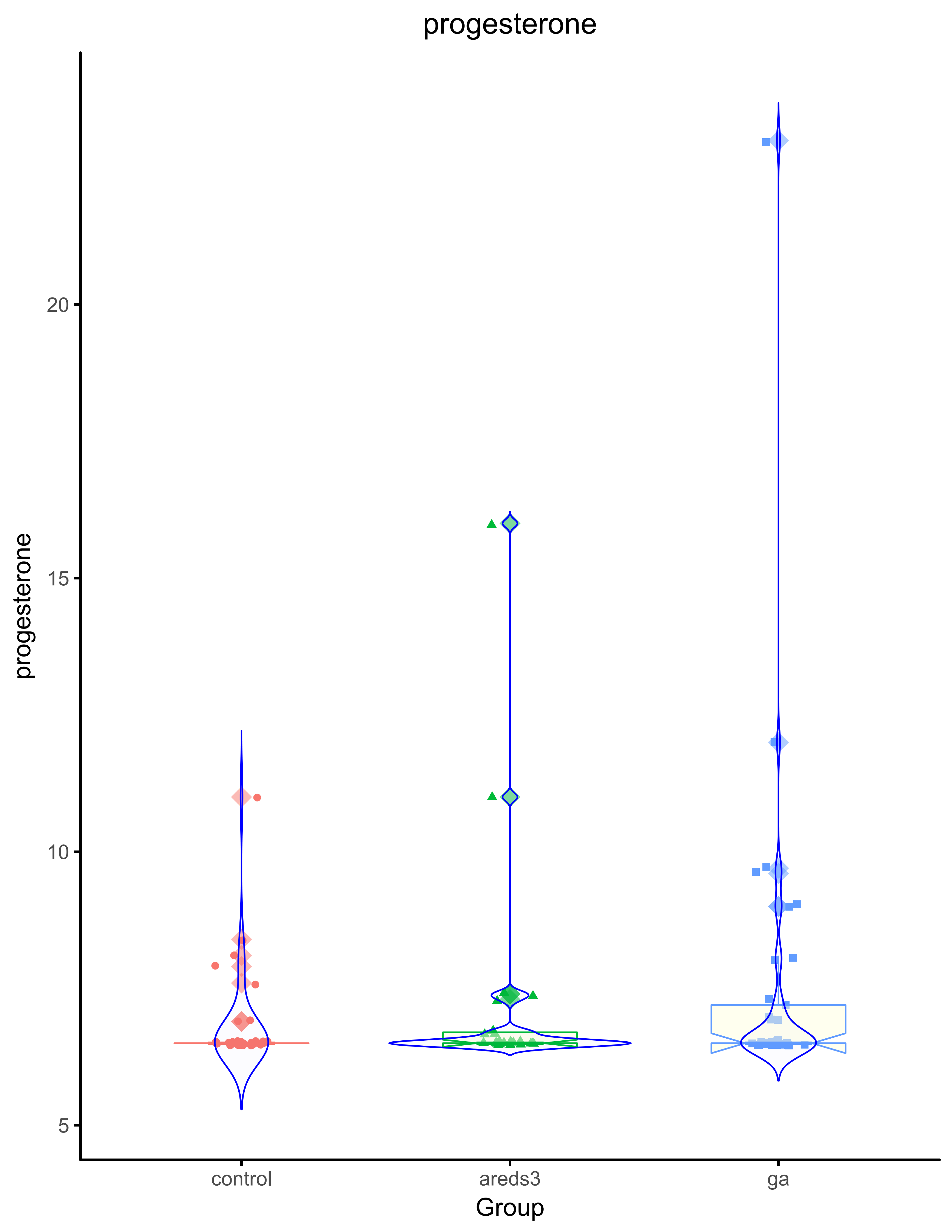

progesterone
progesterone
20
15
10
5
control
areds3
ga
Group

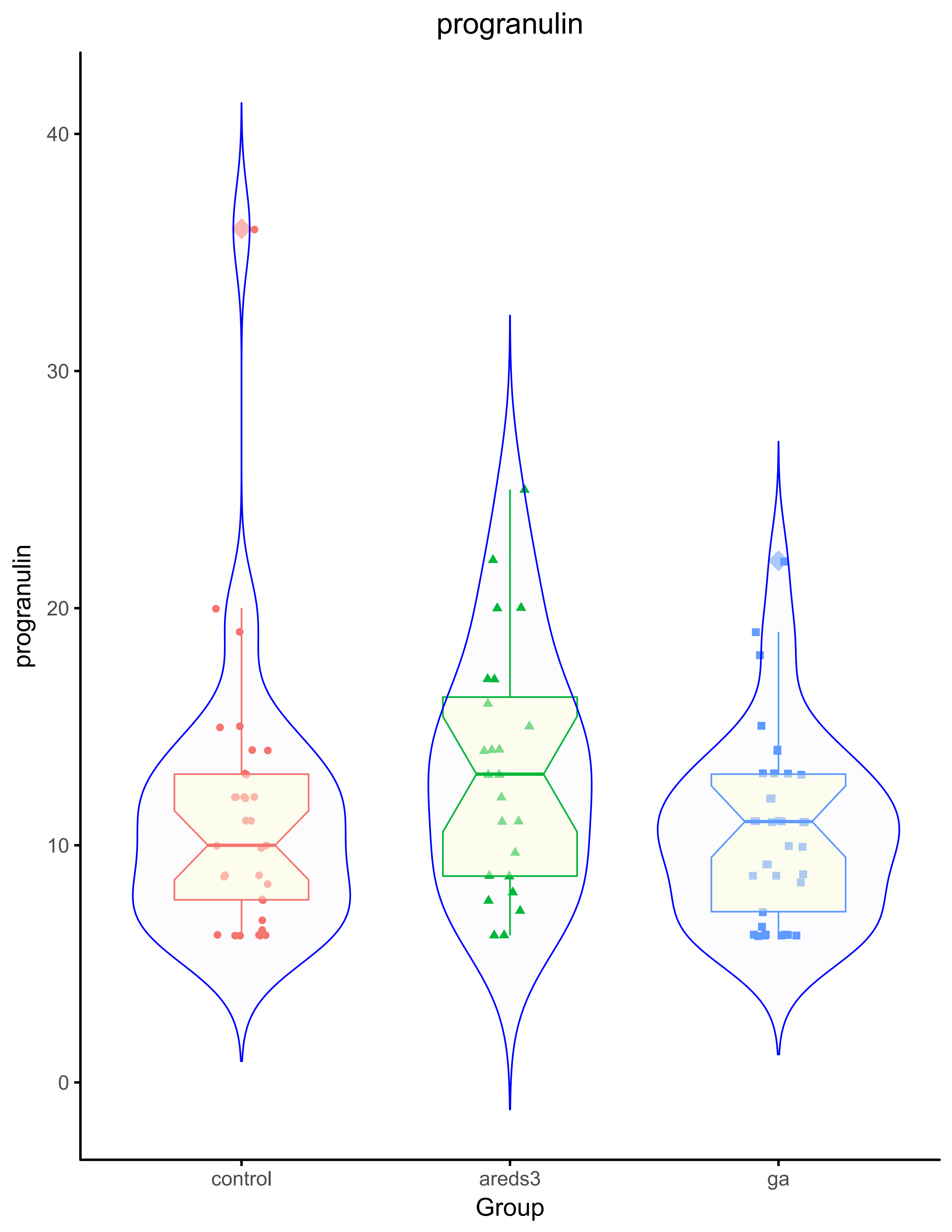
progranulin
progranulin
40
30
20
10
0
control
areds3
ga
Group

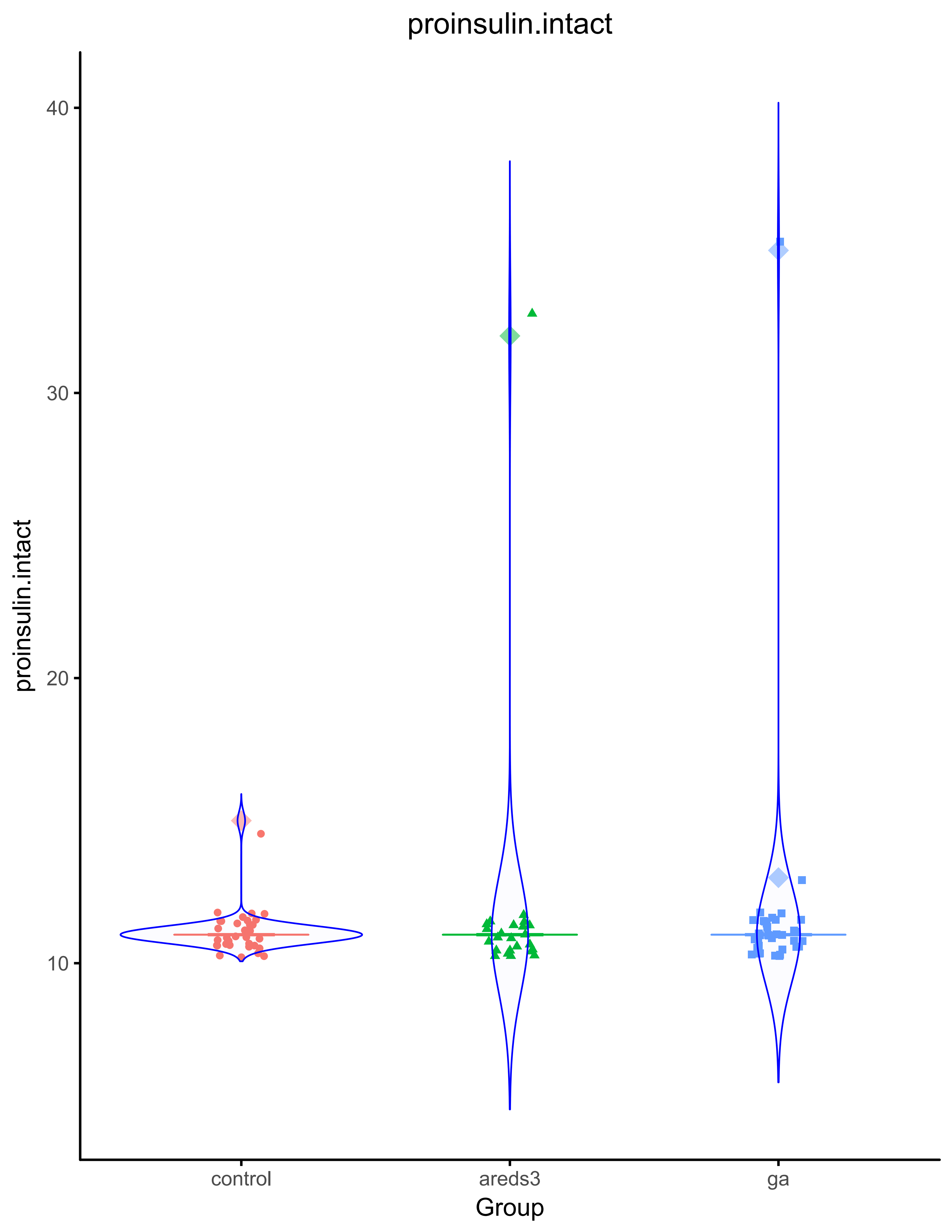

proinsulin.intact
40
30
20
10
proinsulin.intact
control
areds3
ga
Group

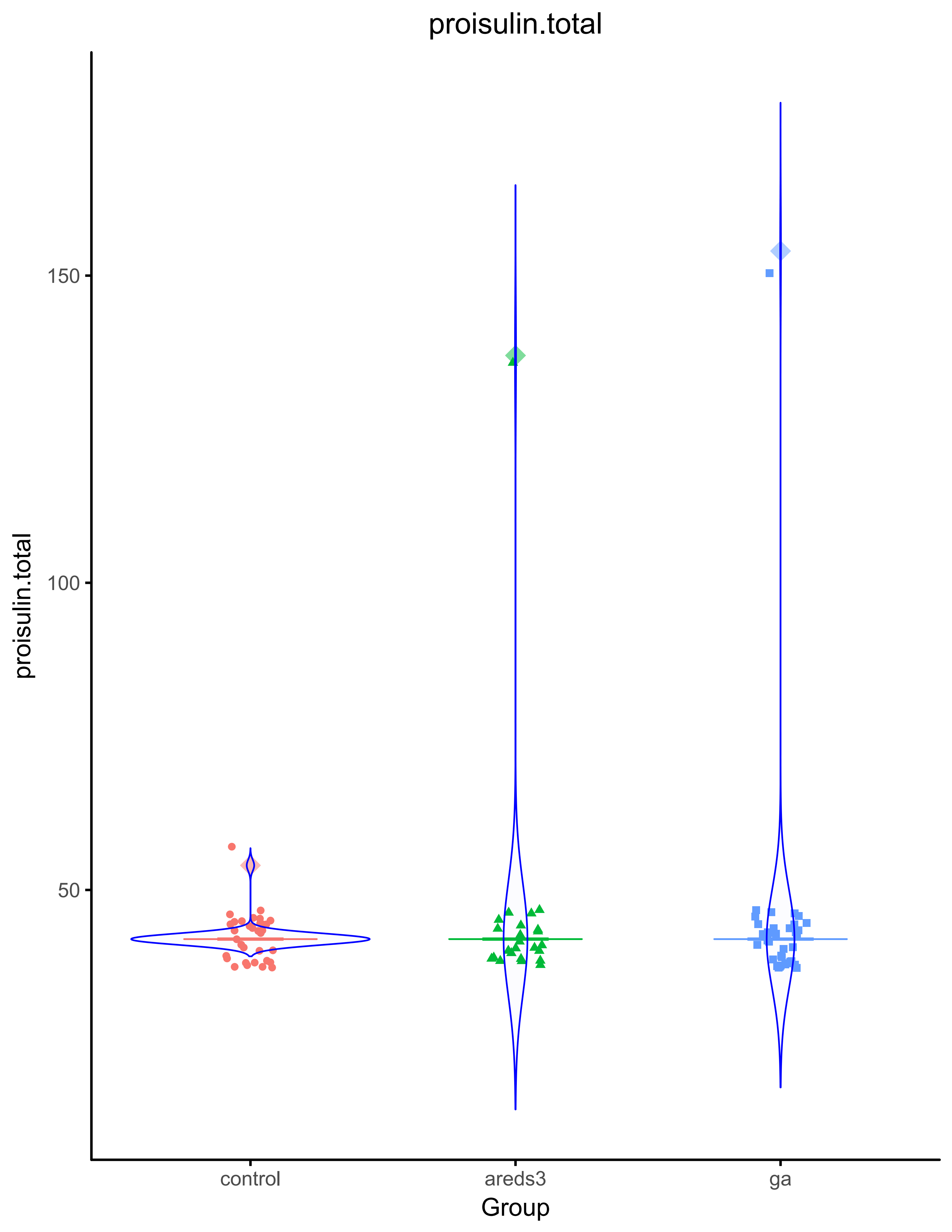

proisulin.total
150
100
50
proisulin.total
control
areds3
ga
Group

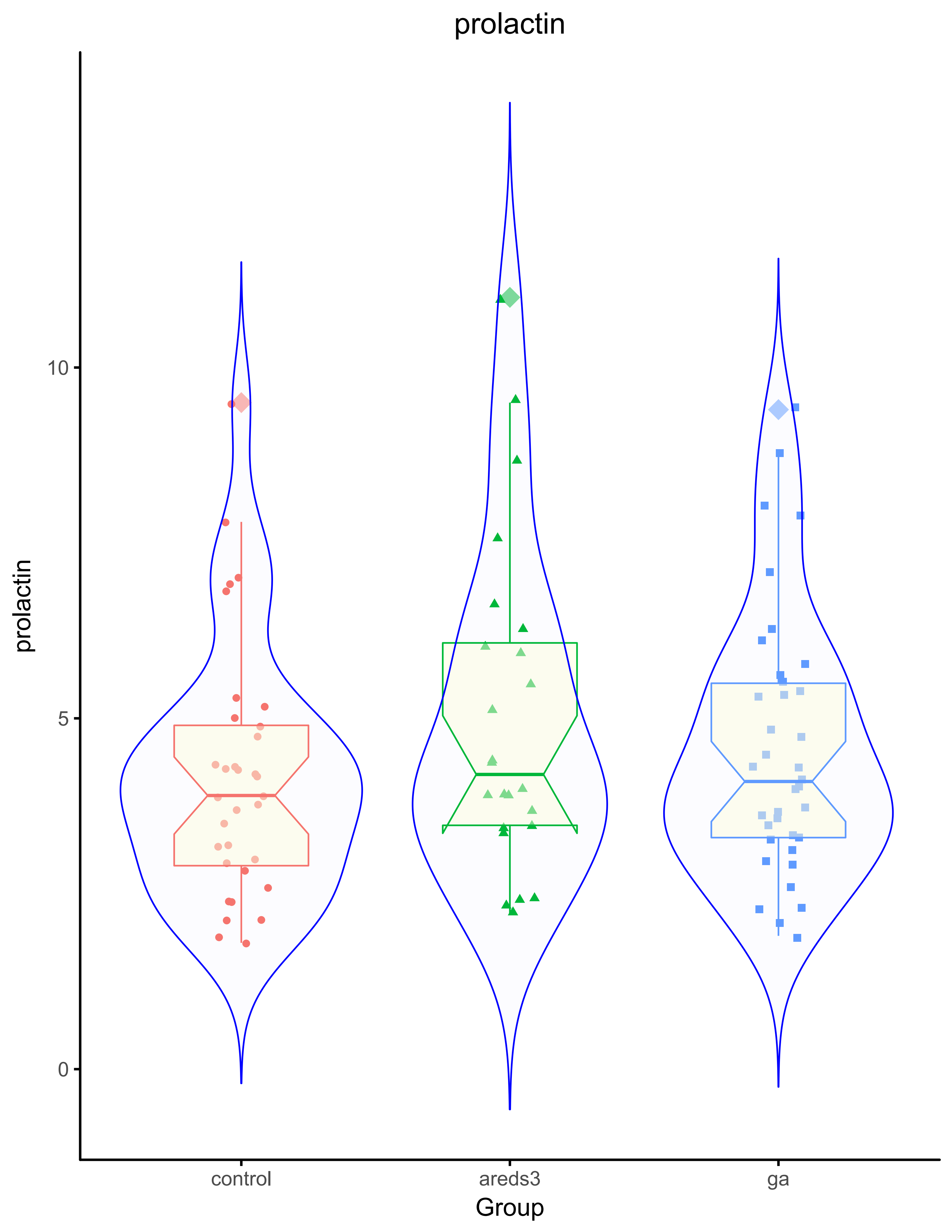

prolactin
prolactin
10
5
0
control
areds3
ga
Group

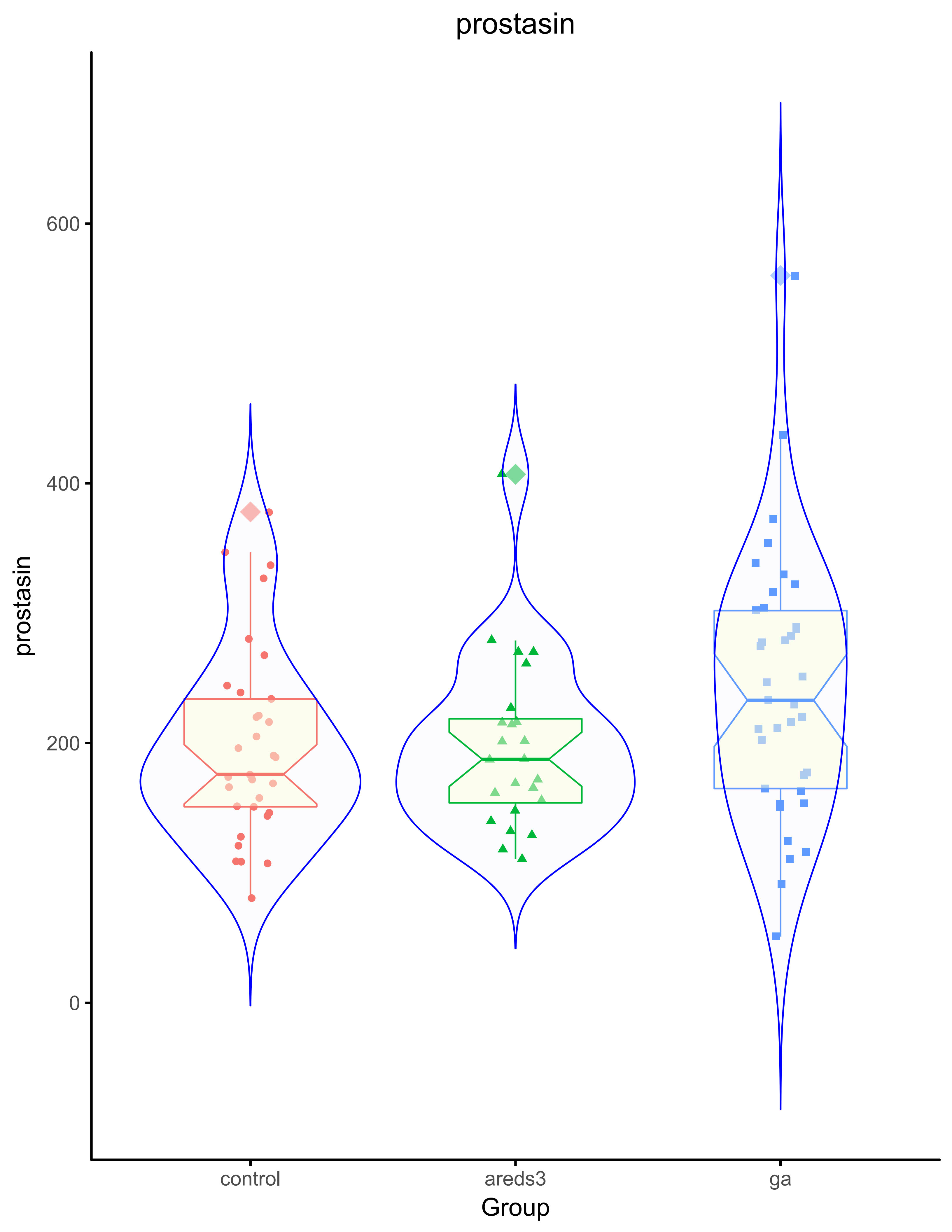

prostasin
600
400
200
0
prostasin
control
areds3
ga
Group

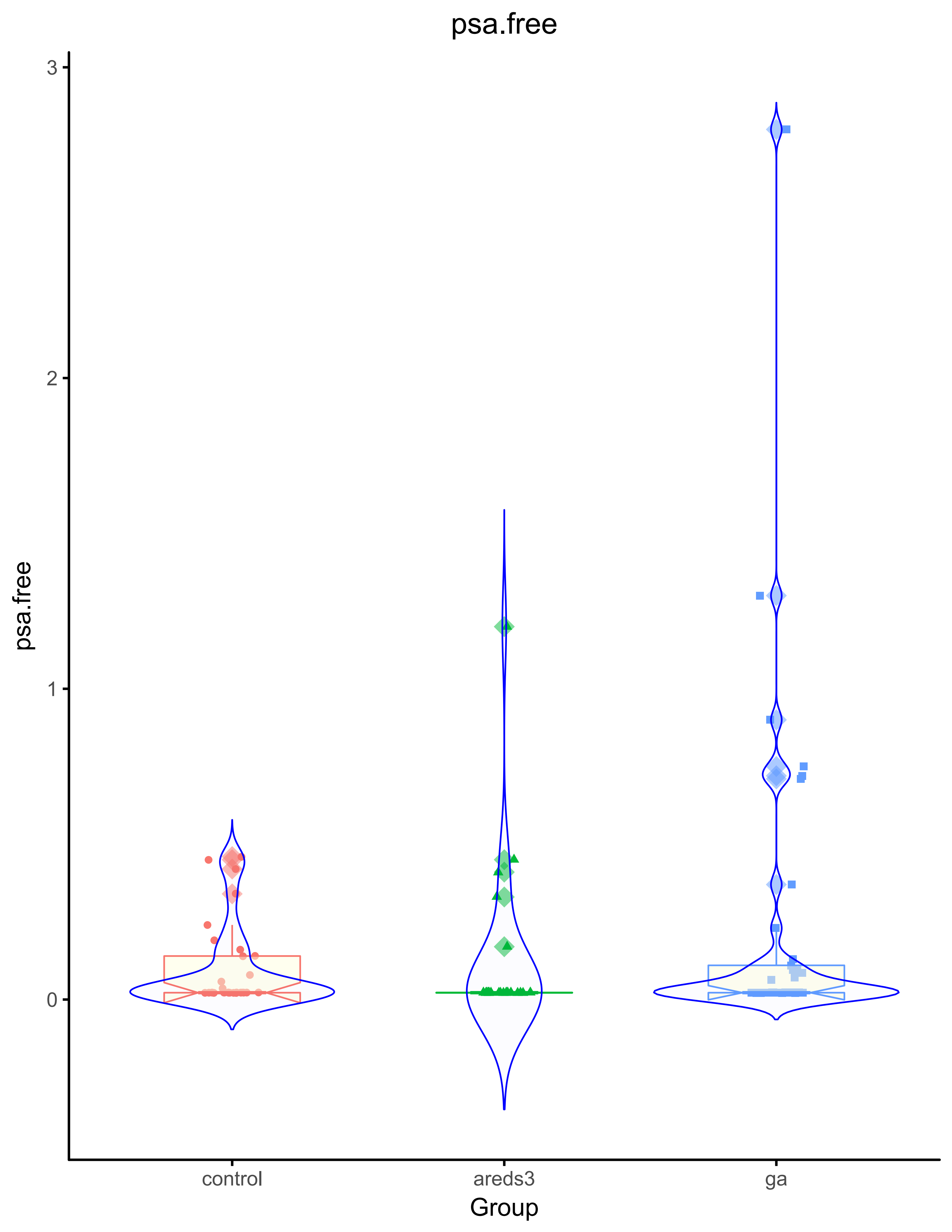

psa.free
3
2
1
0
psa.free
control
areds3
ga
Group

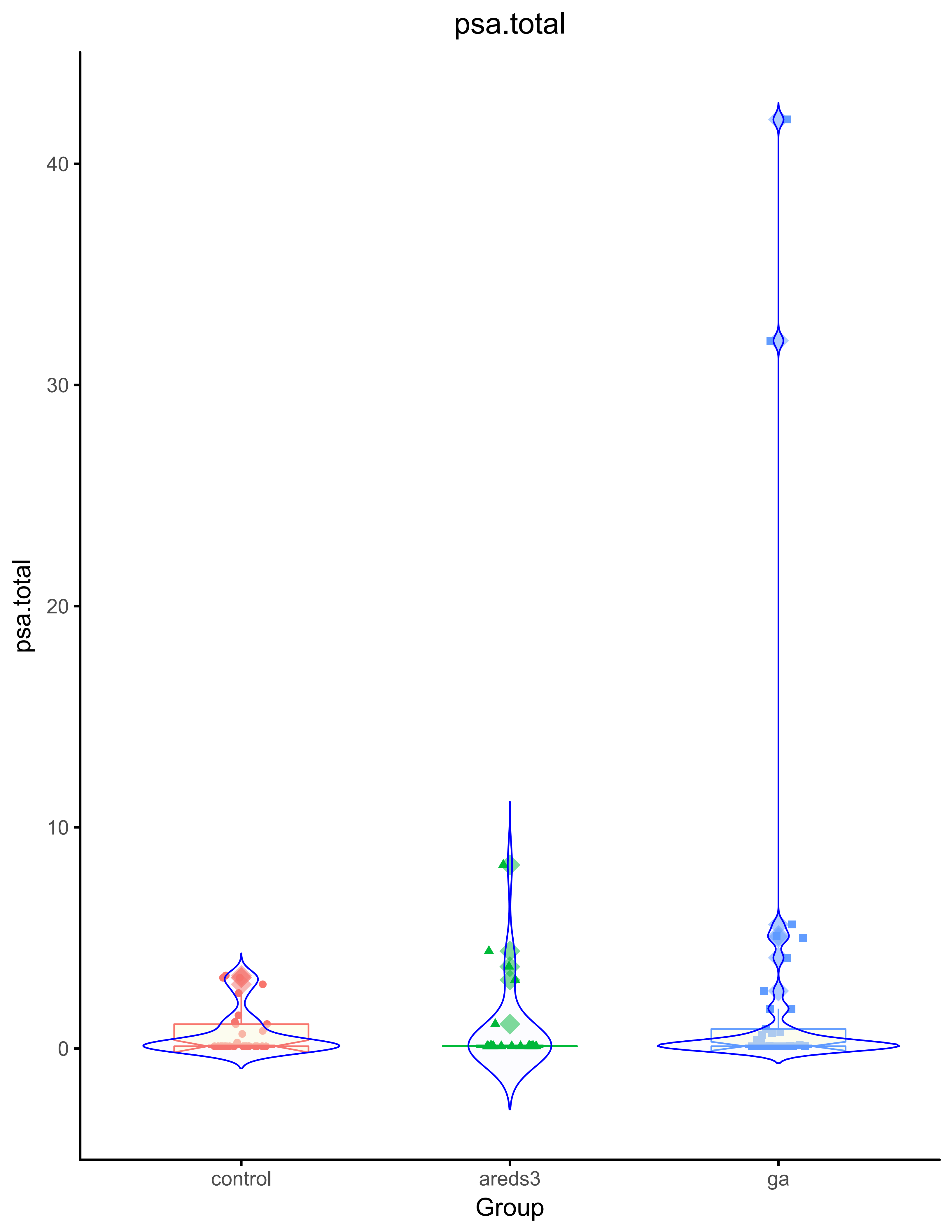
psa.total
40
30
20
10
0
psa.total
control
areds3
ga
Group

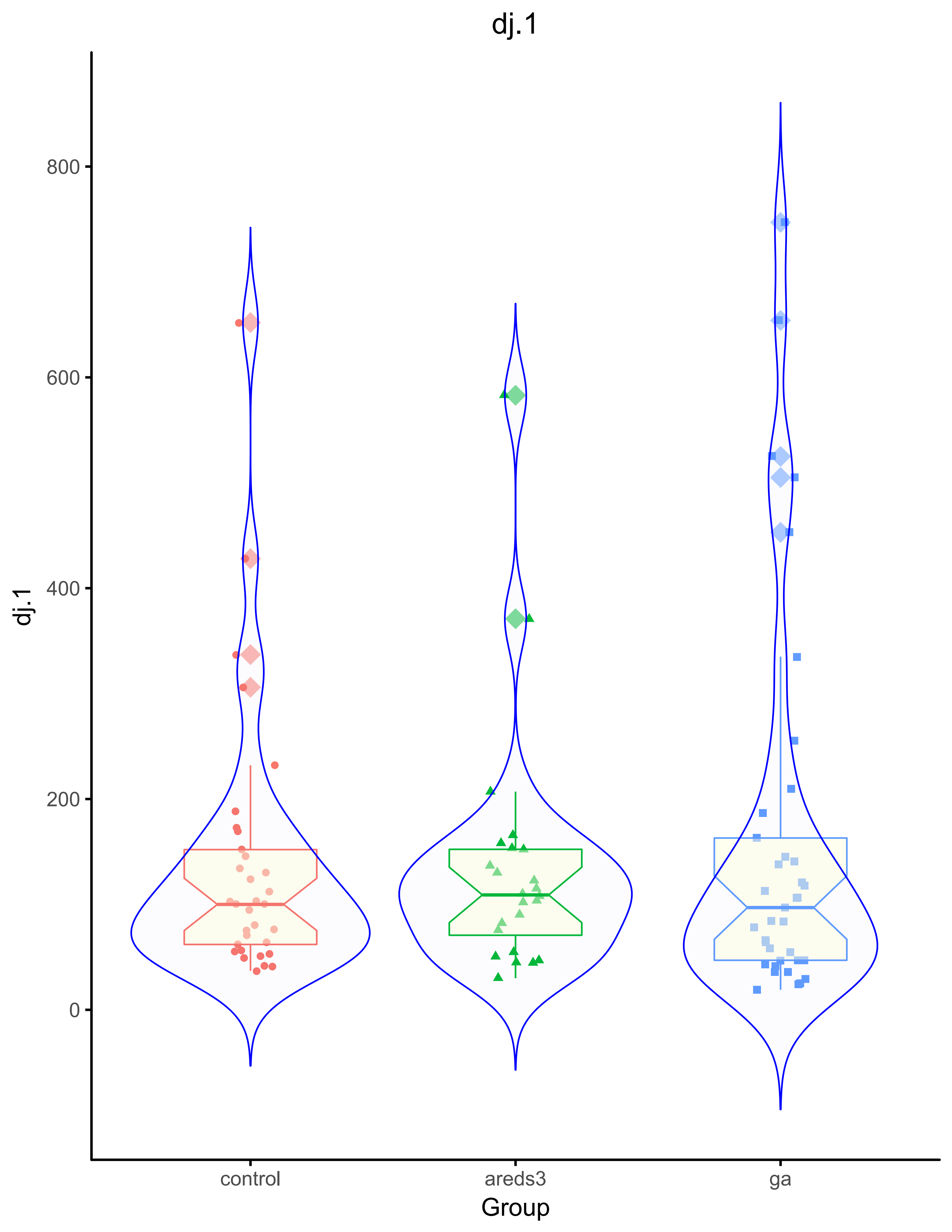
dj.1
800
600
400
200
0
dj.1
control
areds3
ga
Group

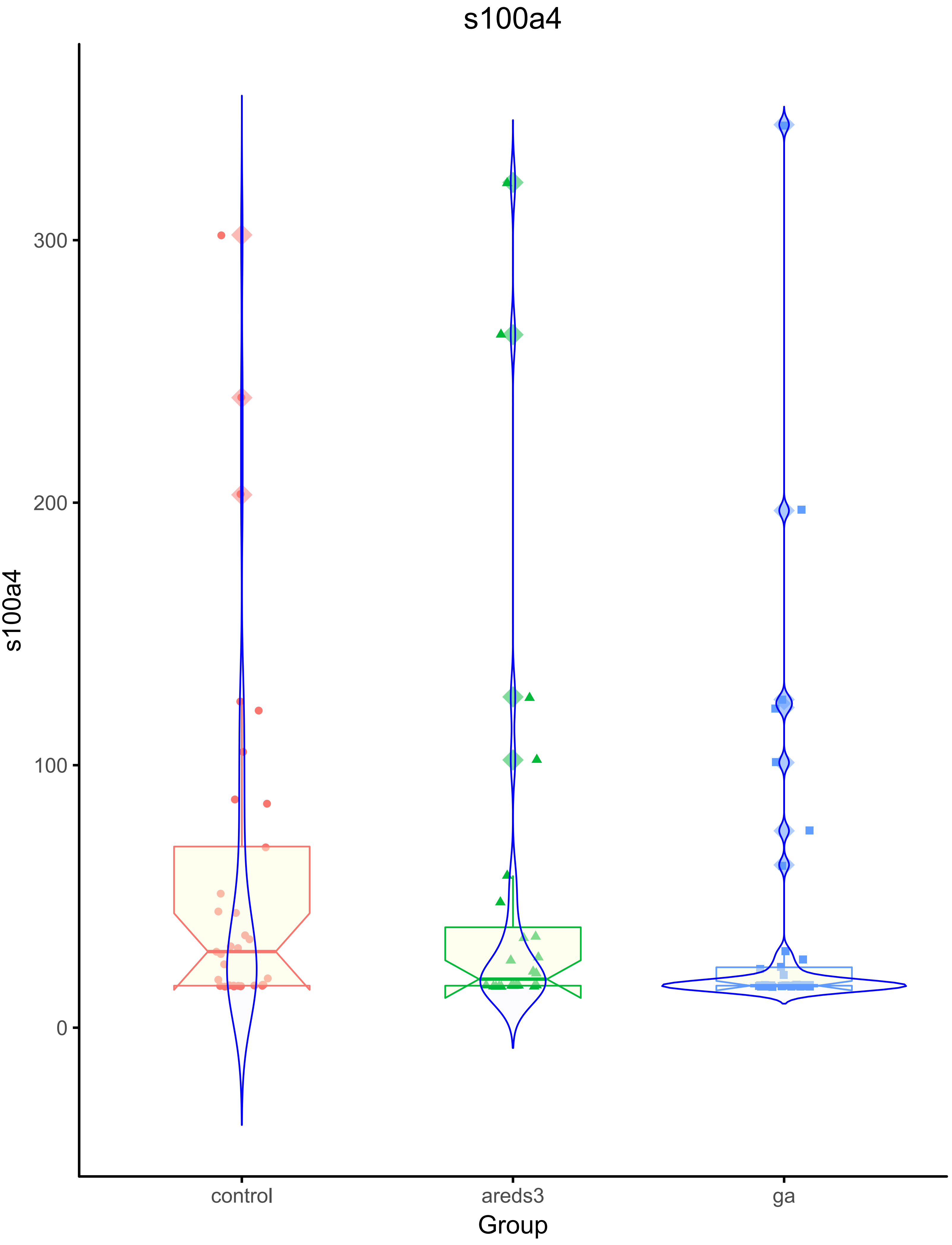

s100a4
300
200
100
0
s100a4
control
areds3
ga
Group

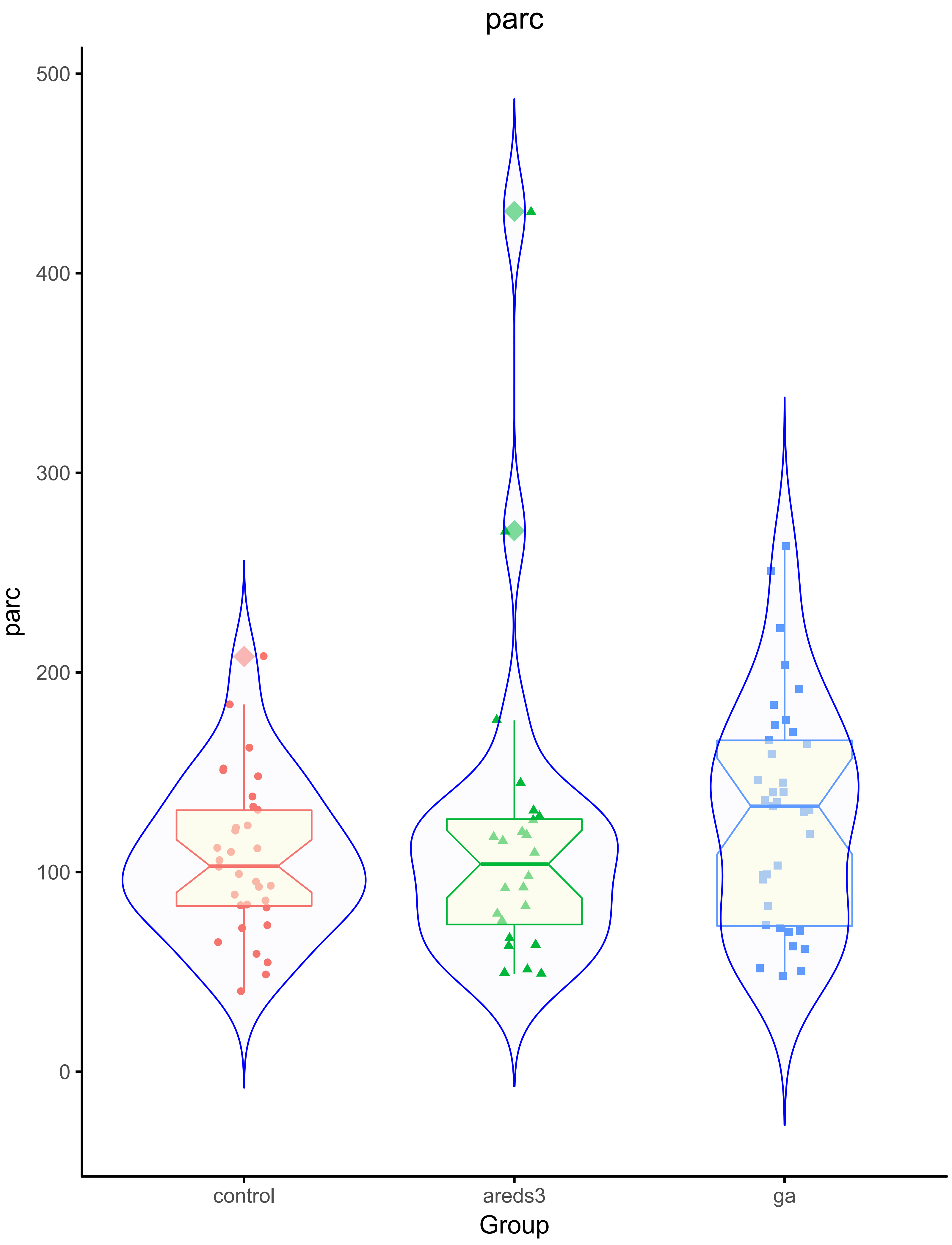

parc
500
400
300
200
100
0
parc
control
areds3
ga
Group

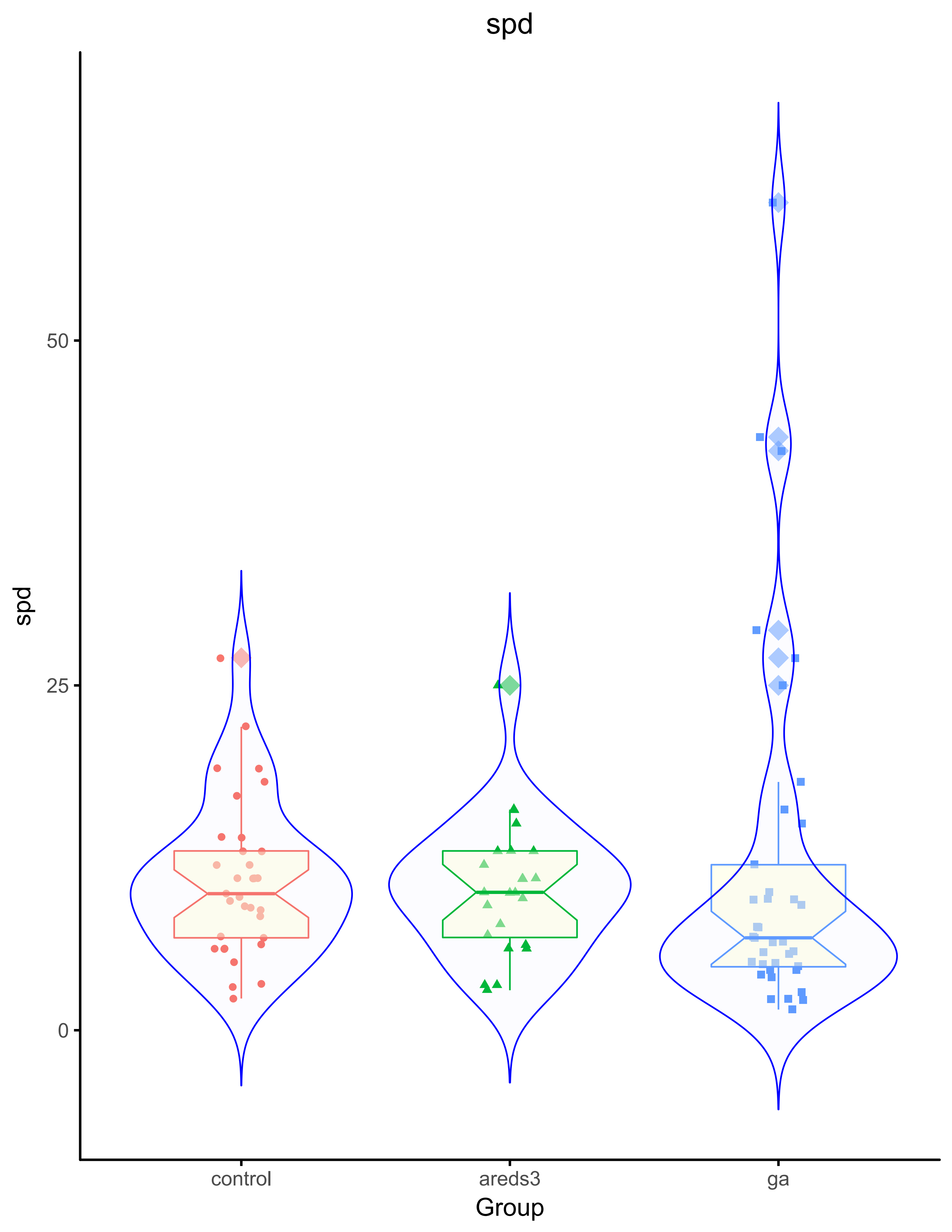
spd
spd
50
25
0
control
areds3
ga
Group

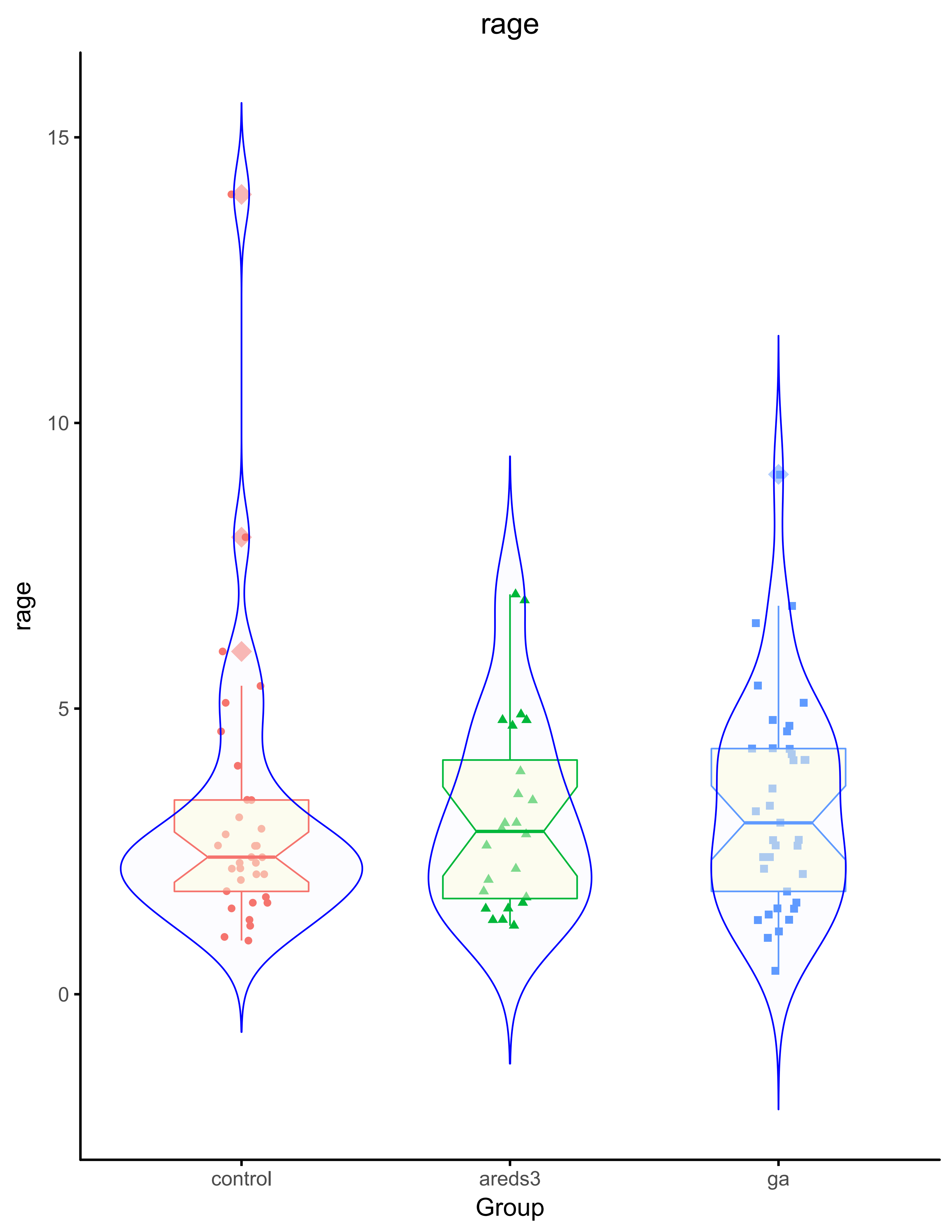

rage
rage
15
10
5
0
control
areds3
ga
Group

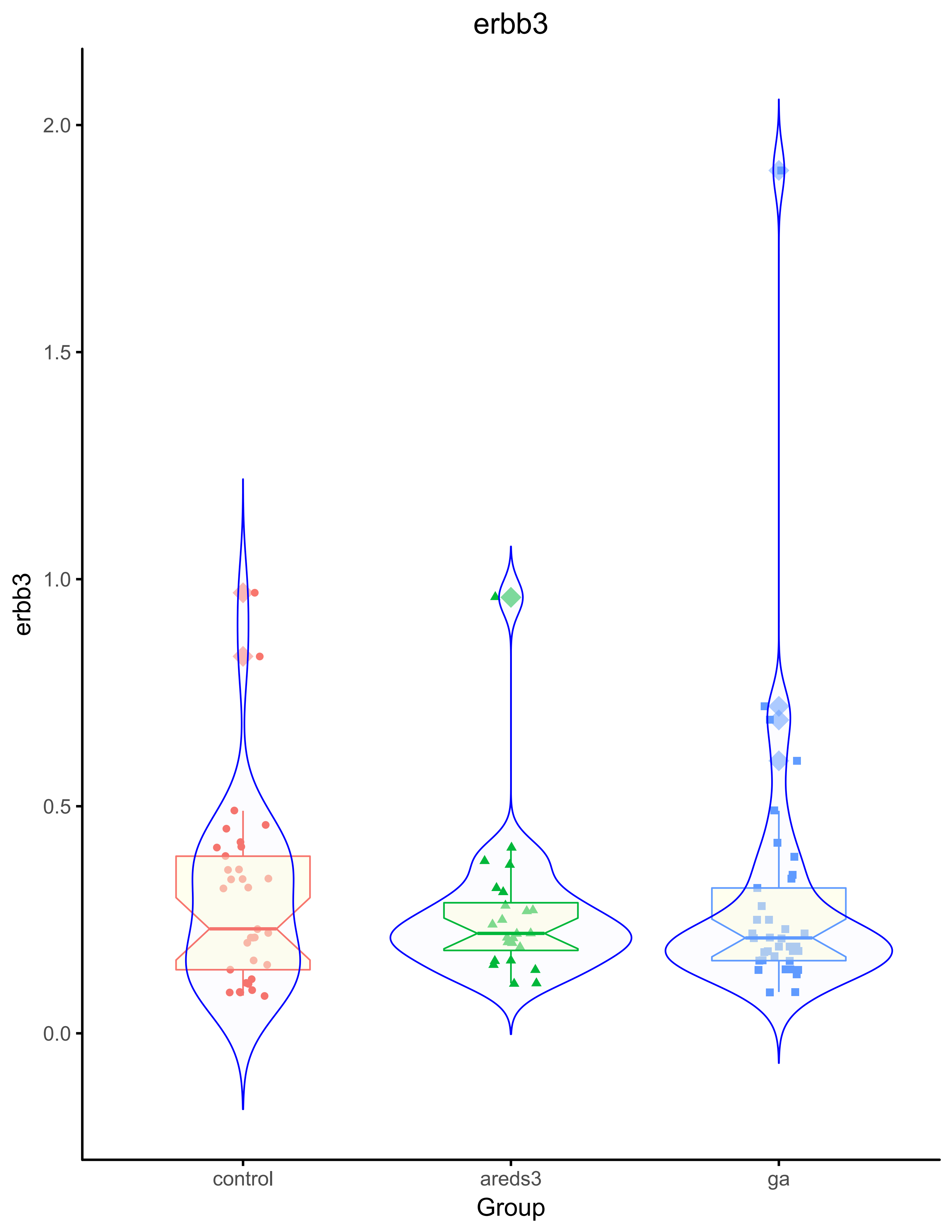

erbb3
erbb3
2.0
1.5
1.0
0.5
0.0
control
areds3
ga
Group

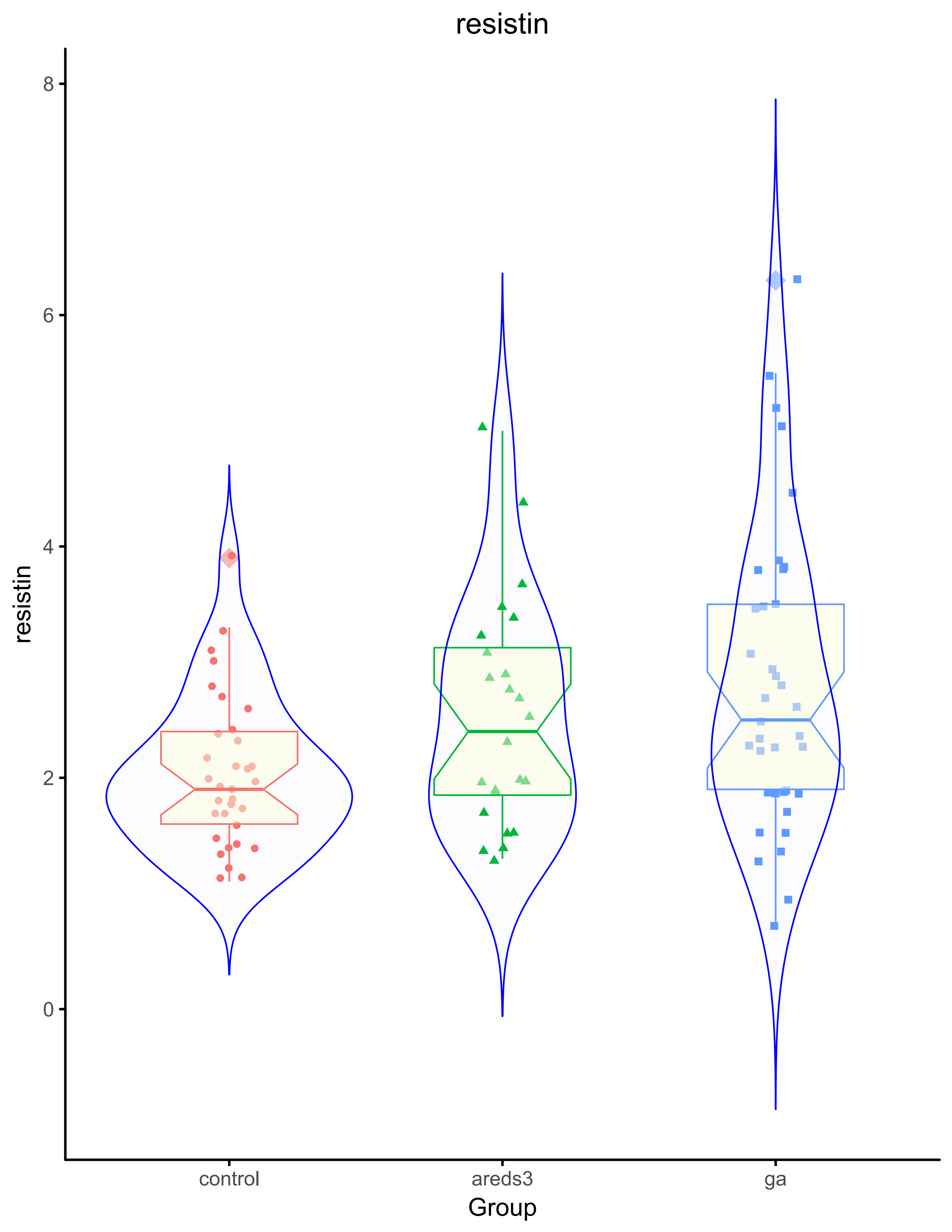
resistin
8
6
4
2
0
resistin
control
areds3
ga
Group

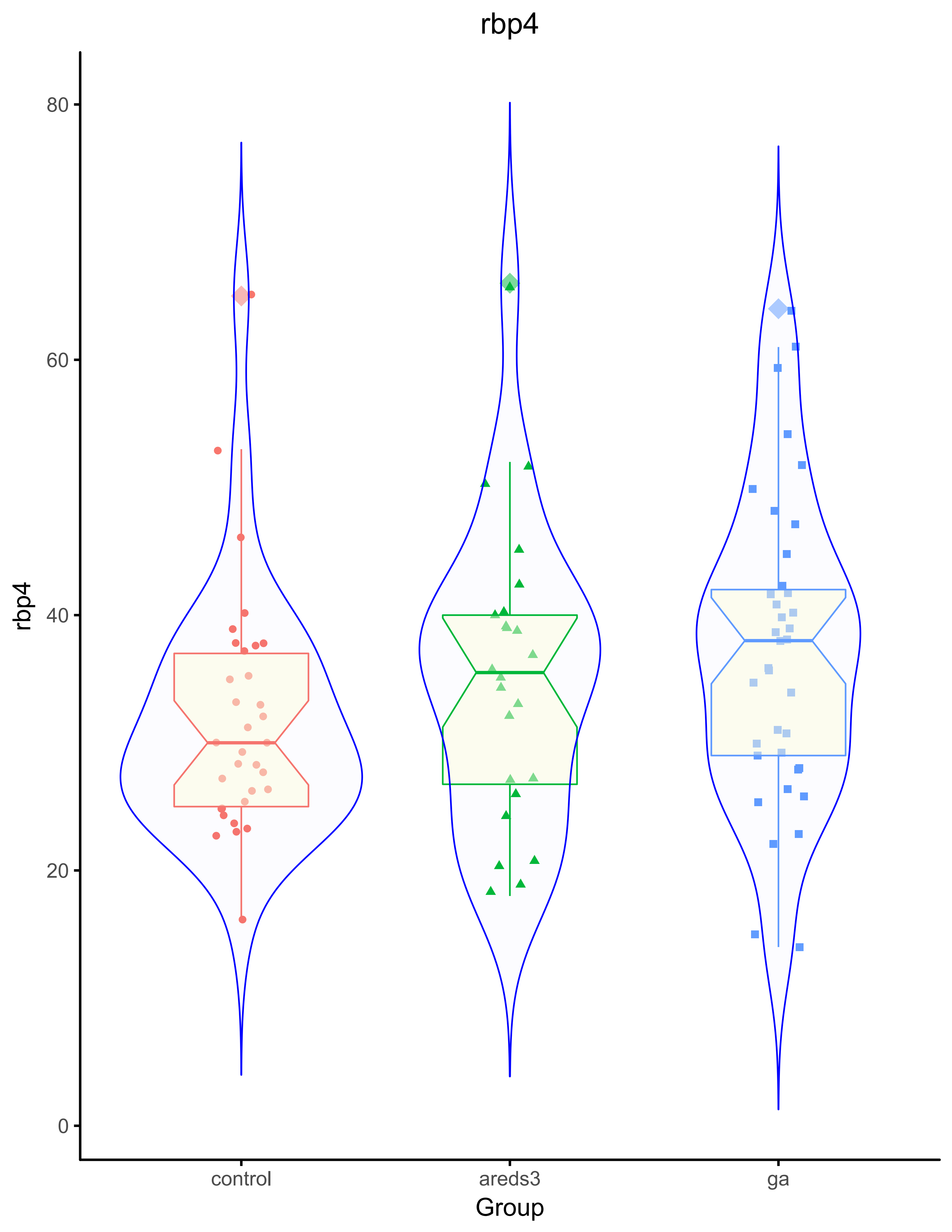
rbp4
80
60
40
20
0
rbp4
control
areds3
ga
Group

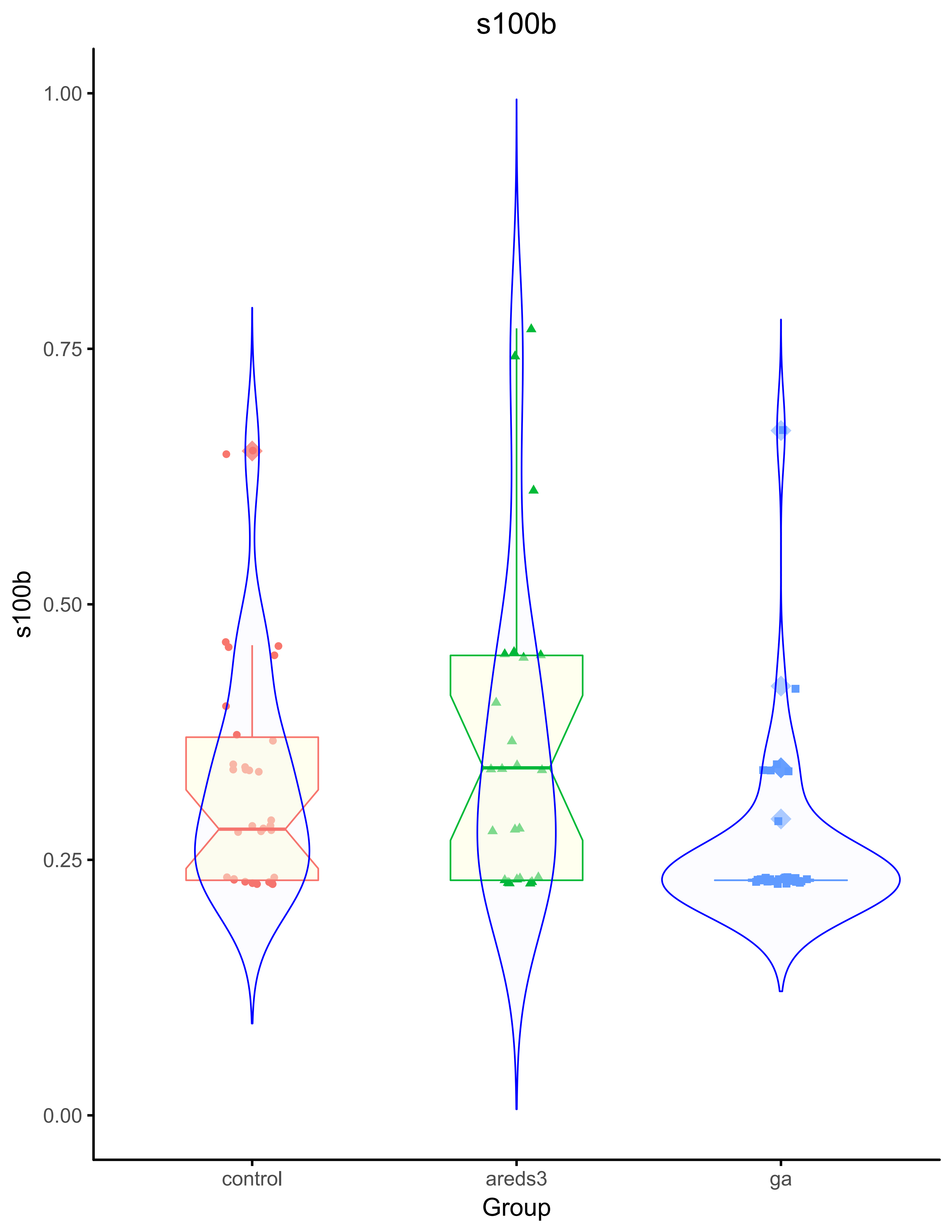
s100b
1.00
0.75
0.50
0.25
0.00
s100b
control
areds3
ga
Group

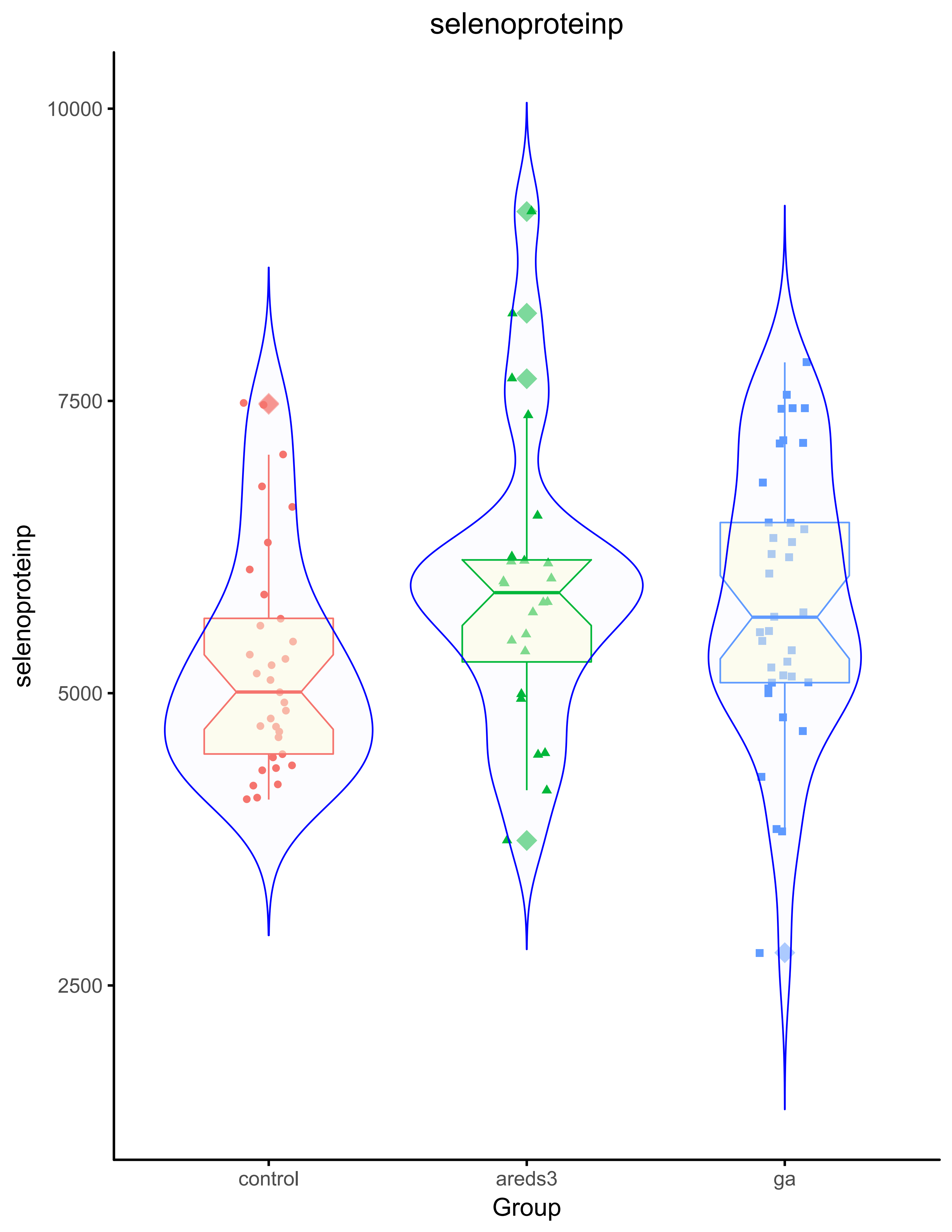
selenoproteinp
10000
7500
5000
2500
selenoproteinp
control
areds3
ga
Group

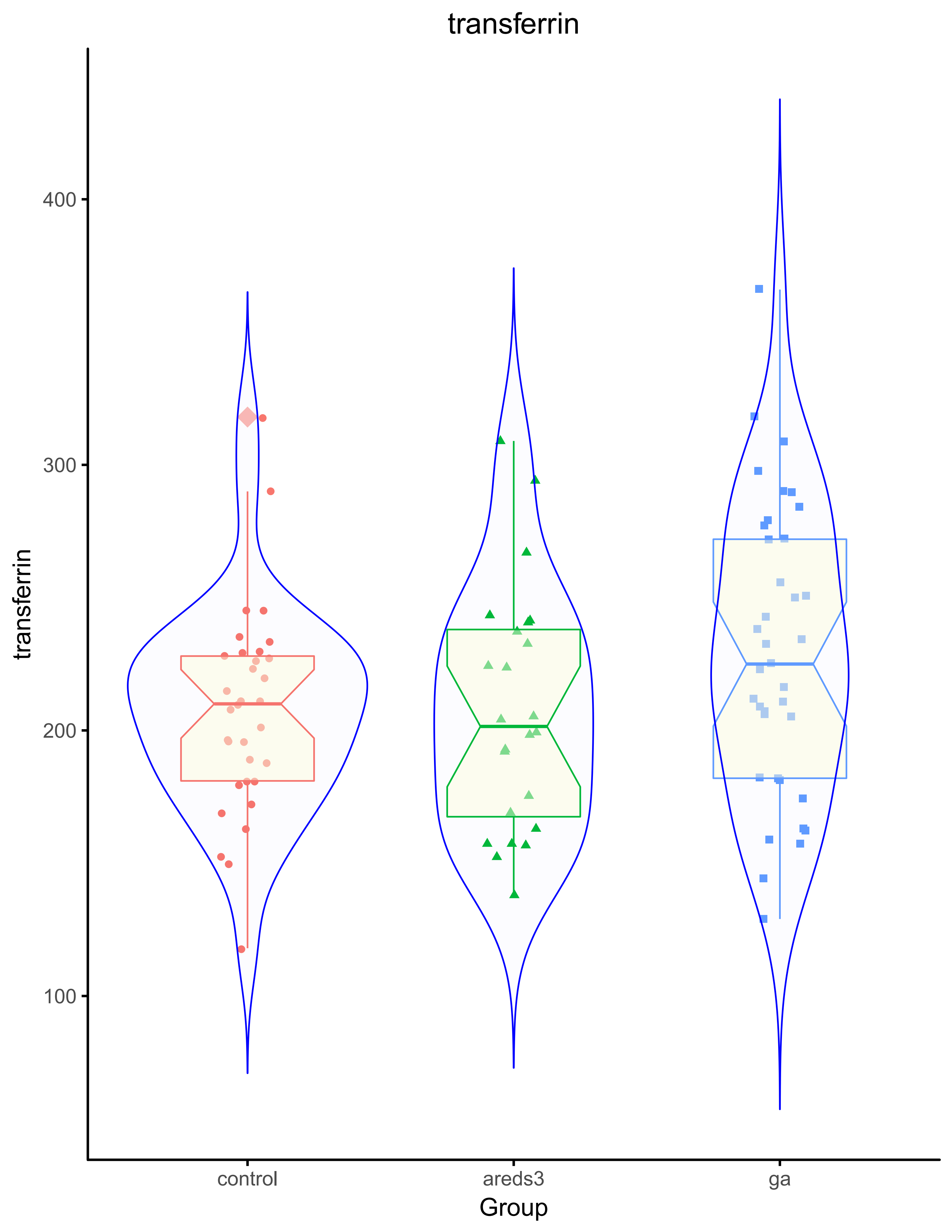

transferrin
400
300
200
100
transferrin
control
areds3
ga
Group

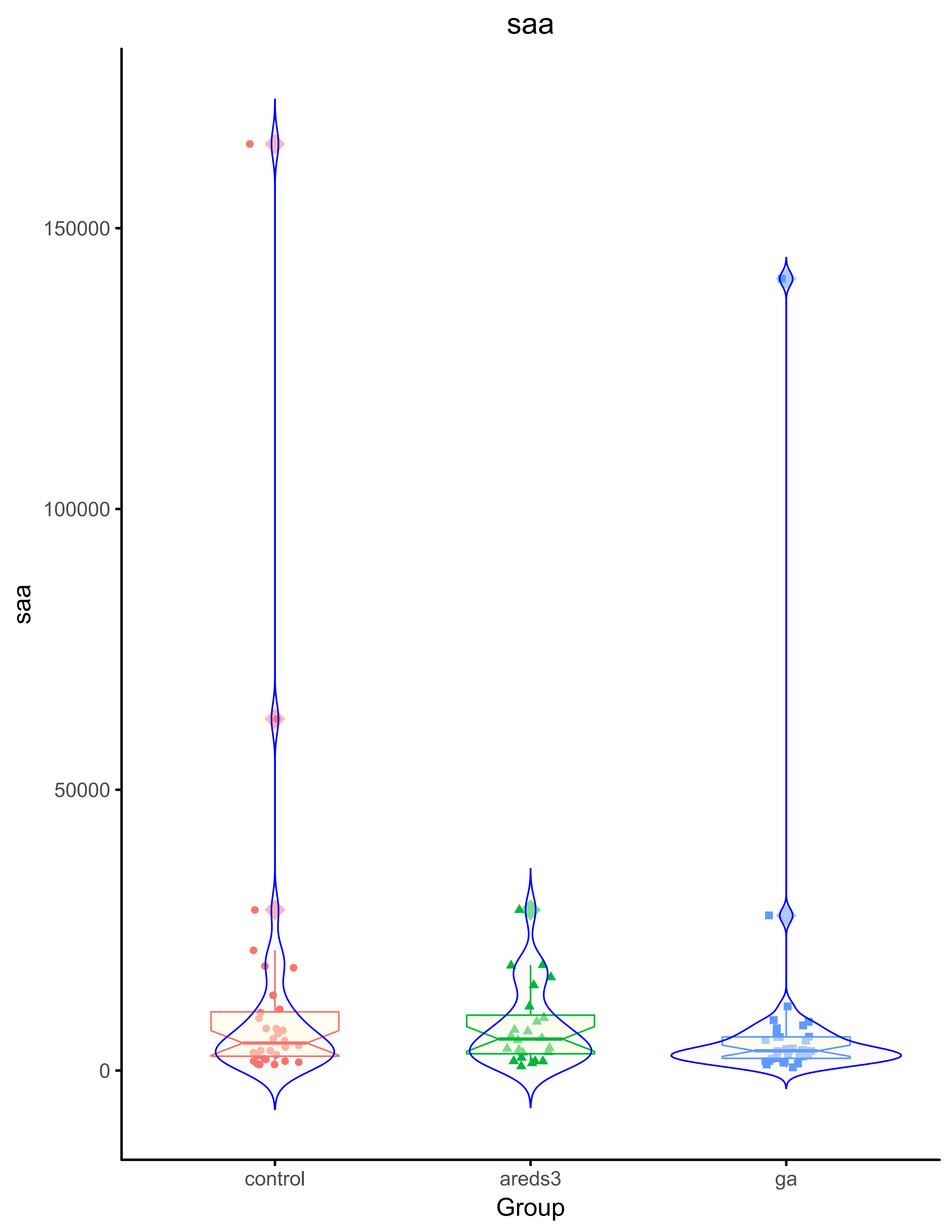
saa
150000
100000
50000
0
saa
control
areds3
ga
Group

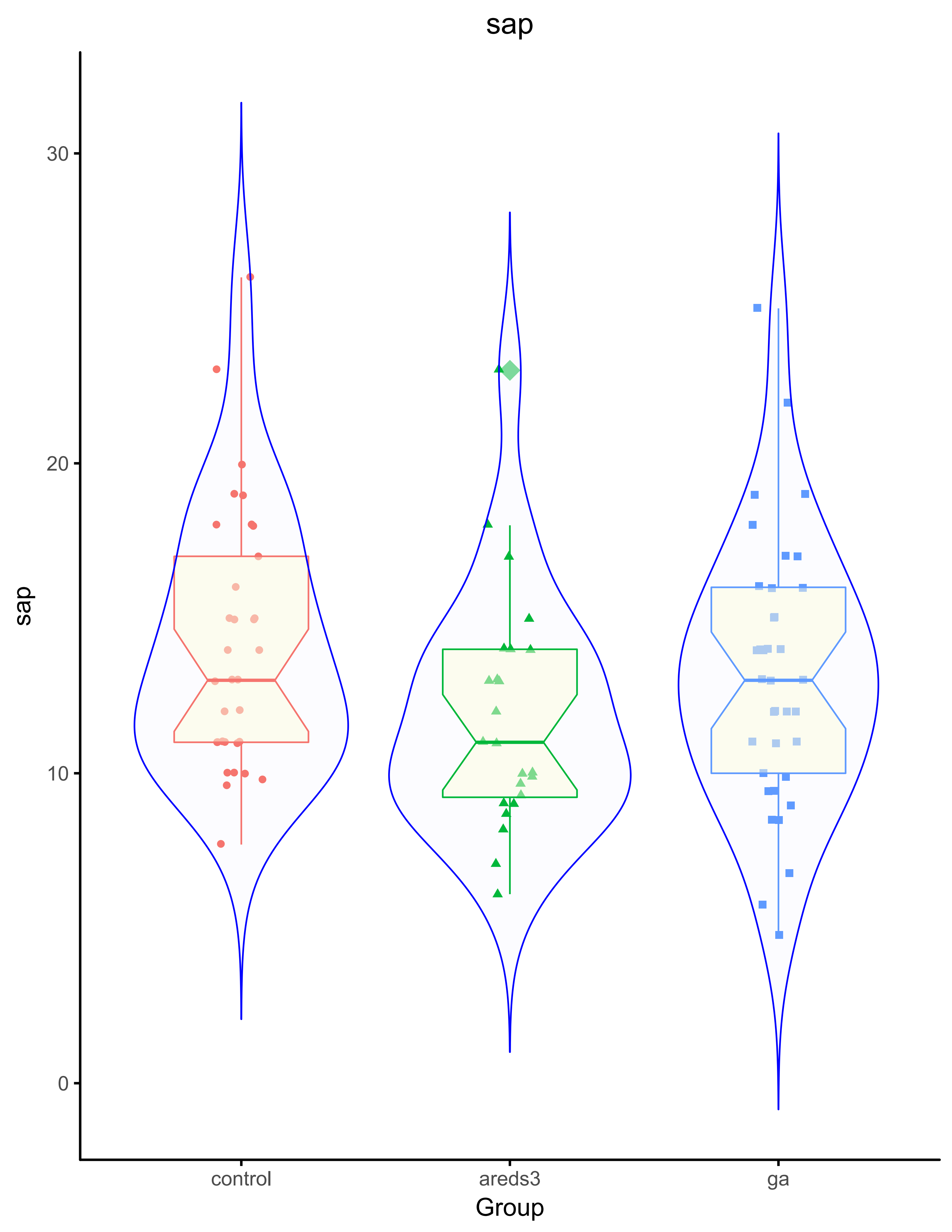

sap
30
20
10
0
sap
control
areds3
ga
Group

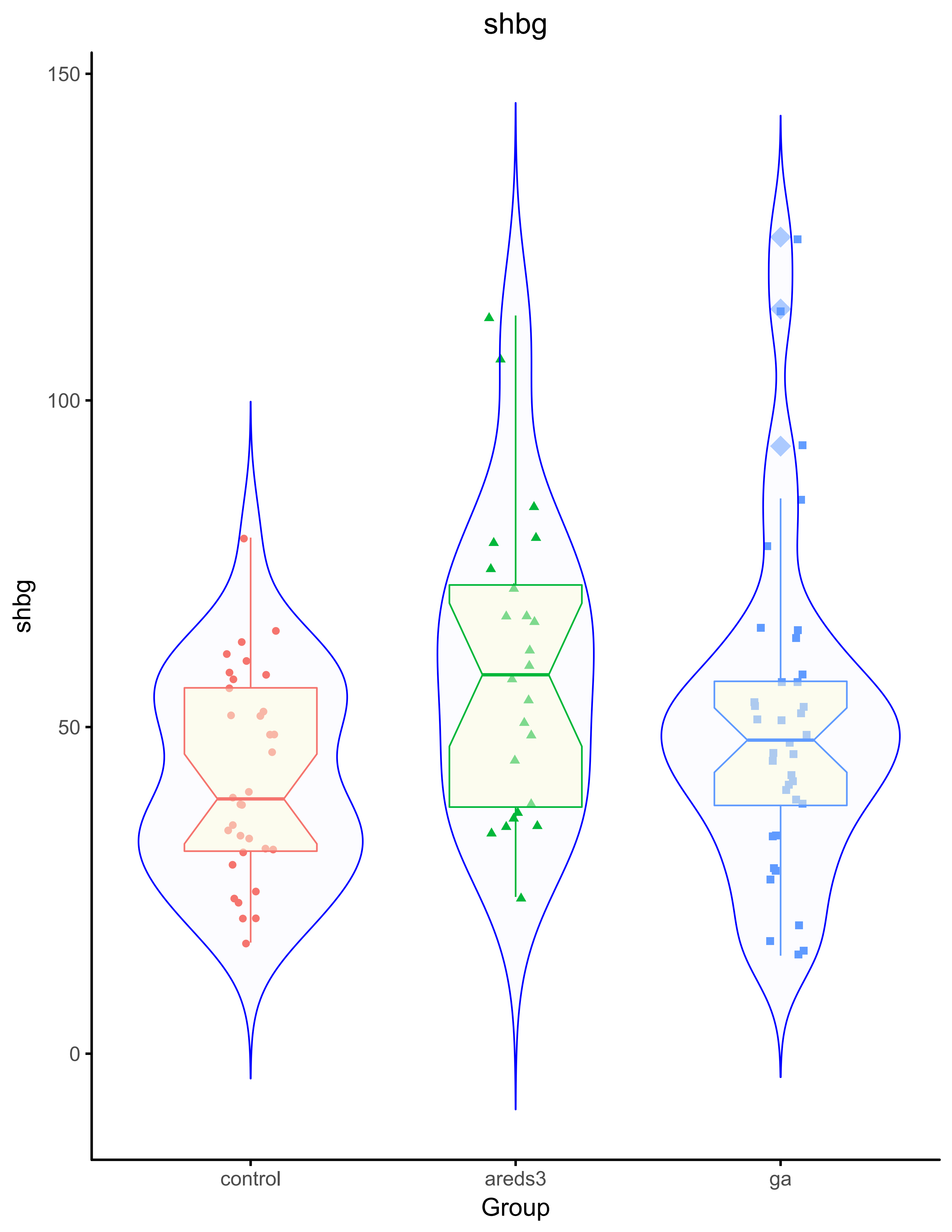
shbg
150
100
50
0
shbg
control
areds3
ga
Group

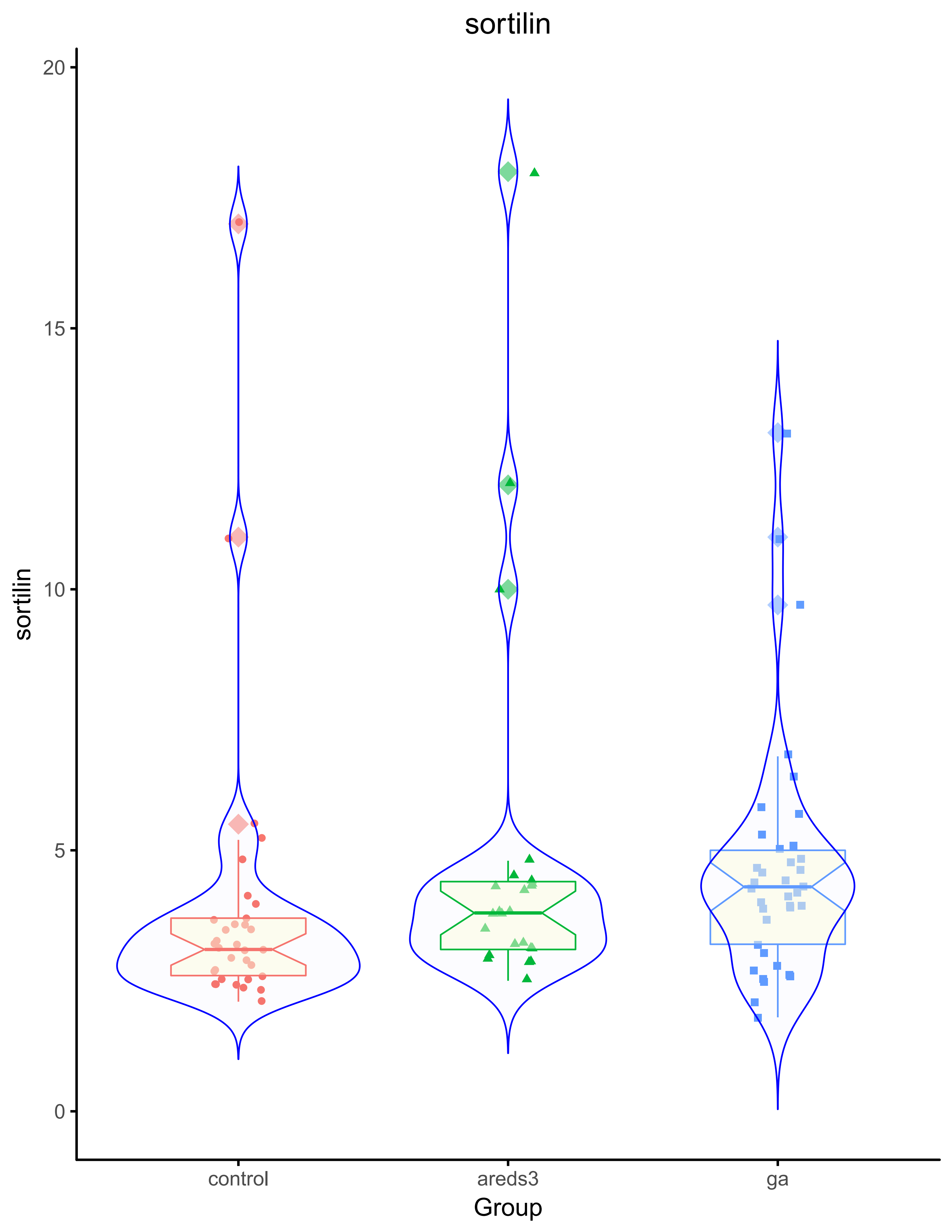
sortilin
20
15
10
5
0
sortilin
control
areds3
ga
Group

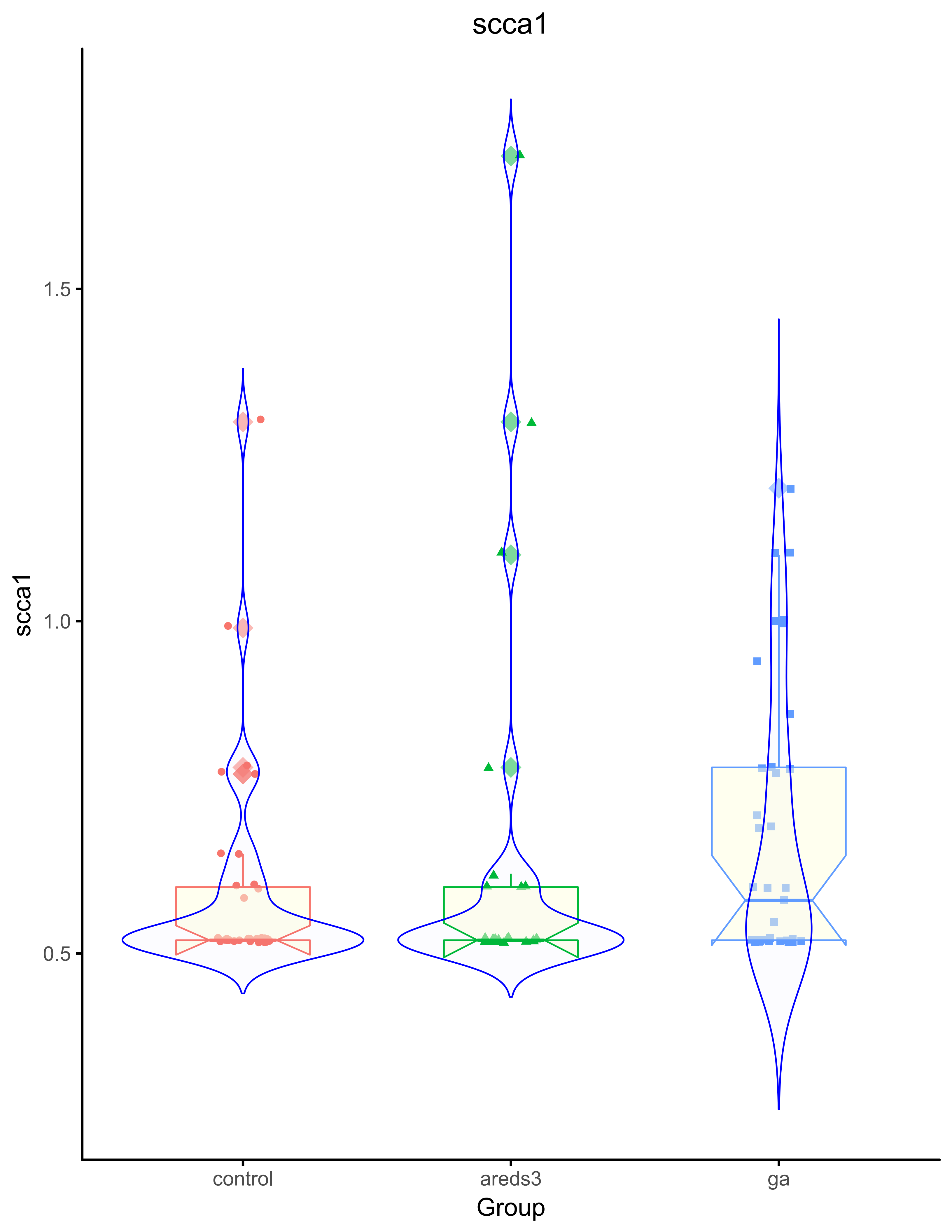
scca1
1.5
1.0
0.5
scca1
control
areds3
ga
Group

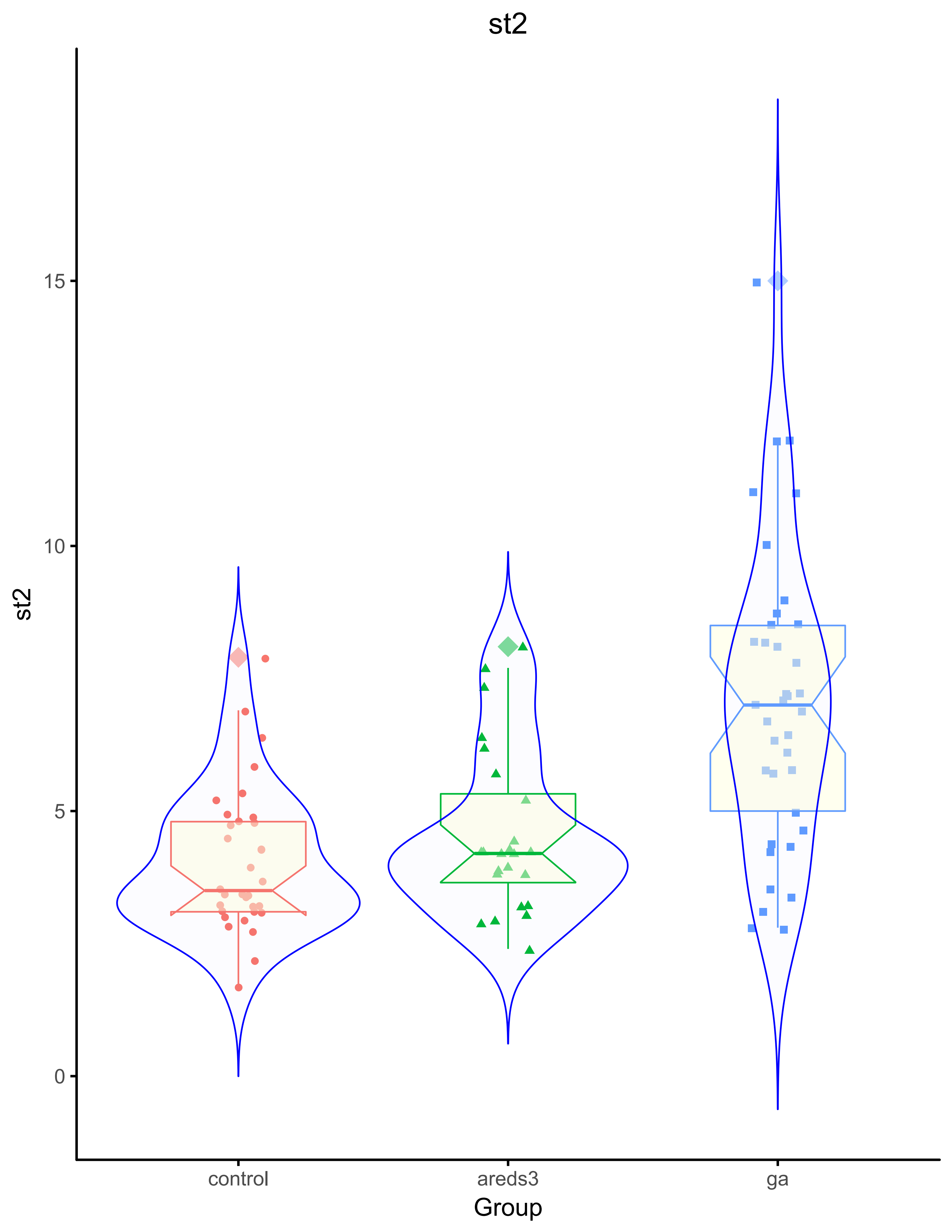
st2
st2
15
10
5
0
control
areds3
ga
Group

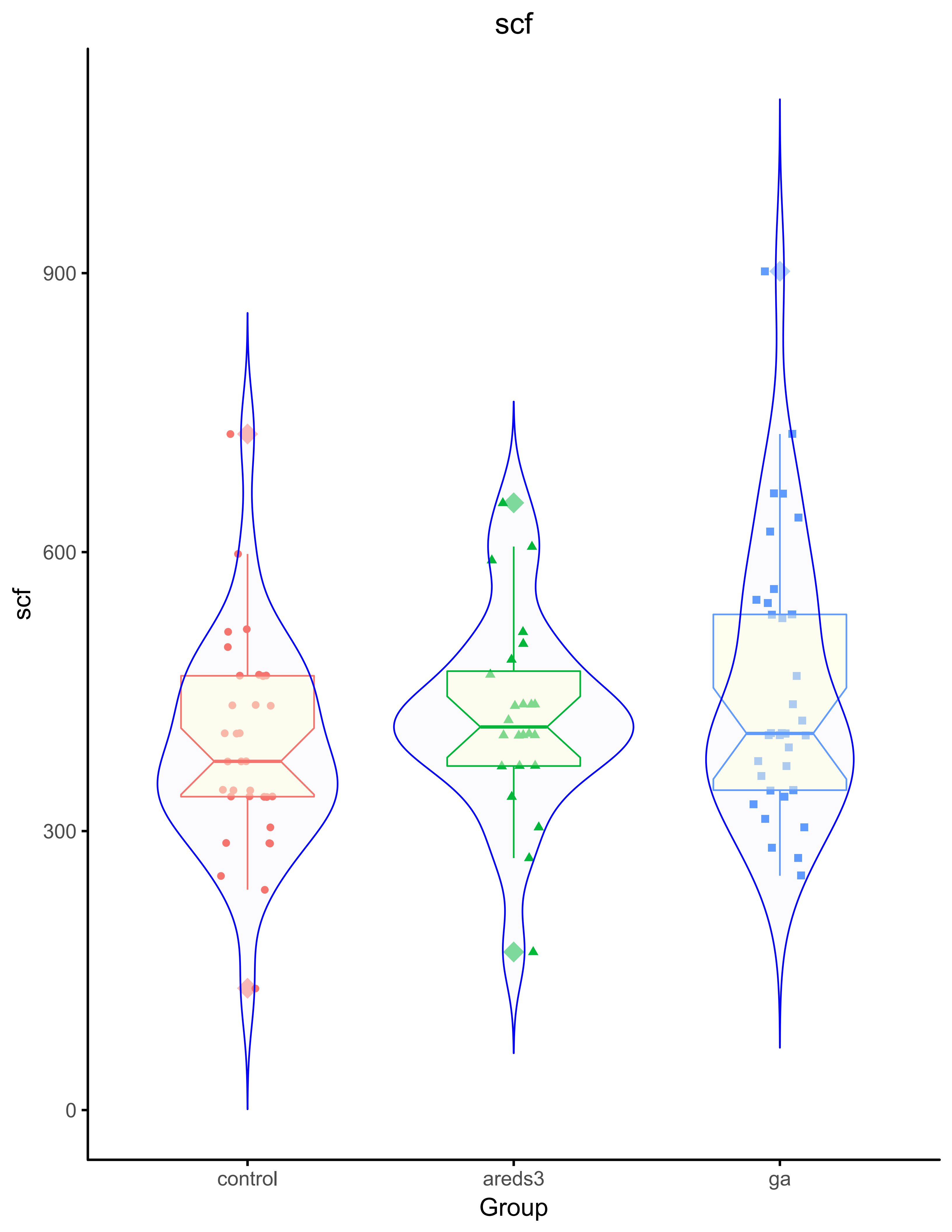
scf
900
600
300
0
scf
control
areds3
ga
Group

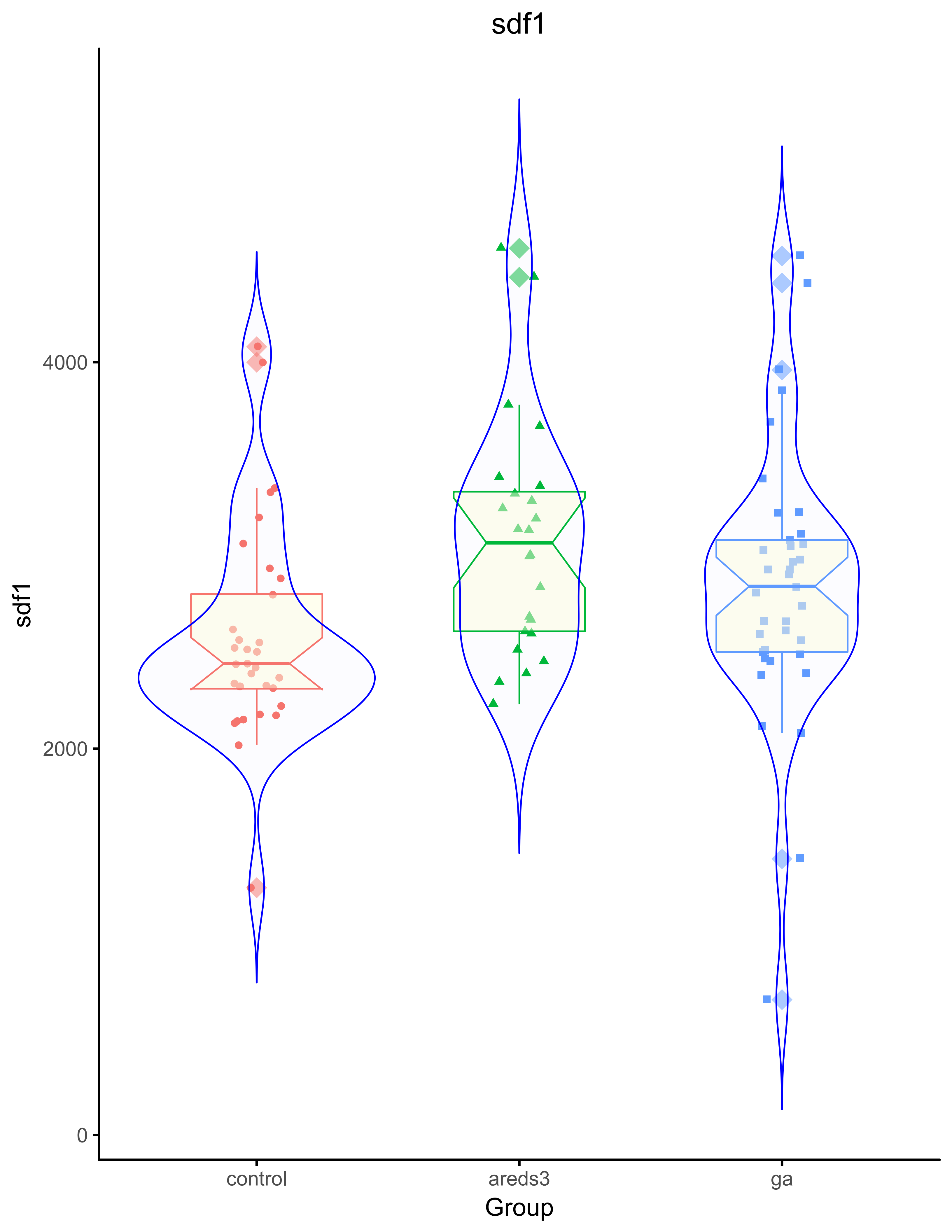
sdf1
sdf1
4000
2000
0
control
areds3
ga
Group

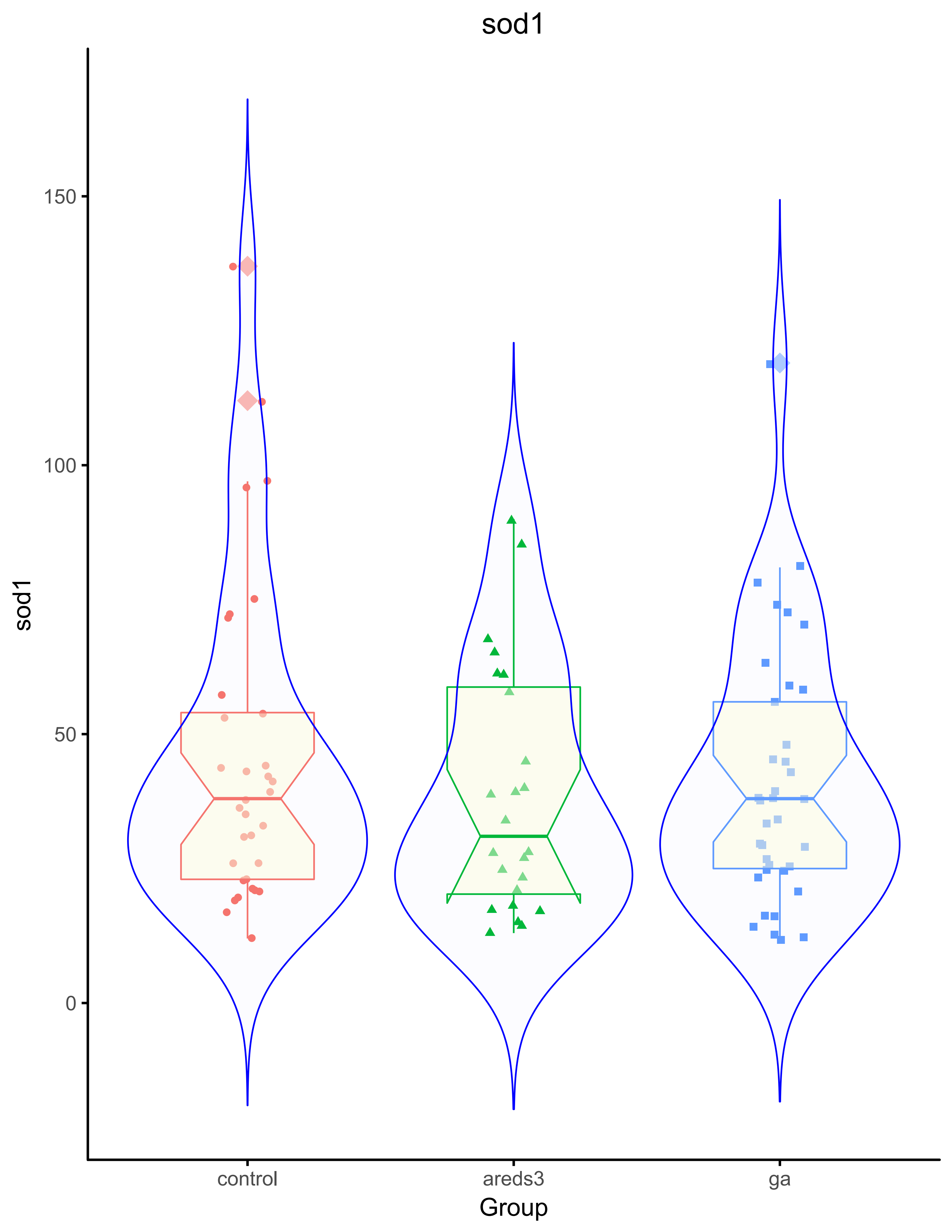
sod1
150
100
50
0
sod1
control
areds3
ga
Group

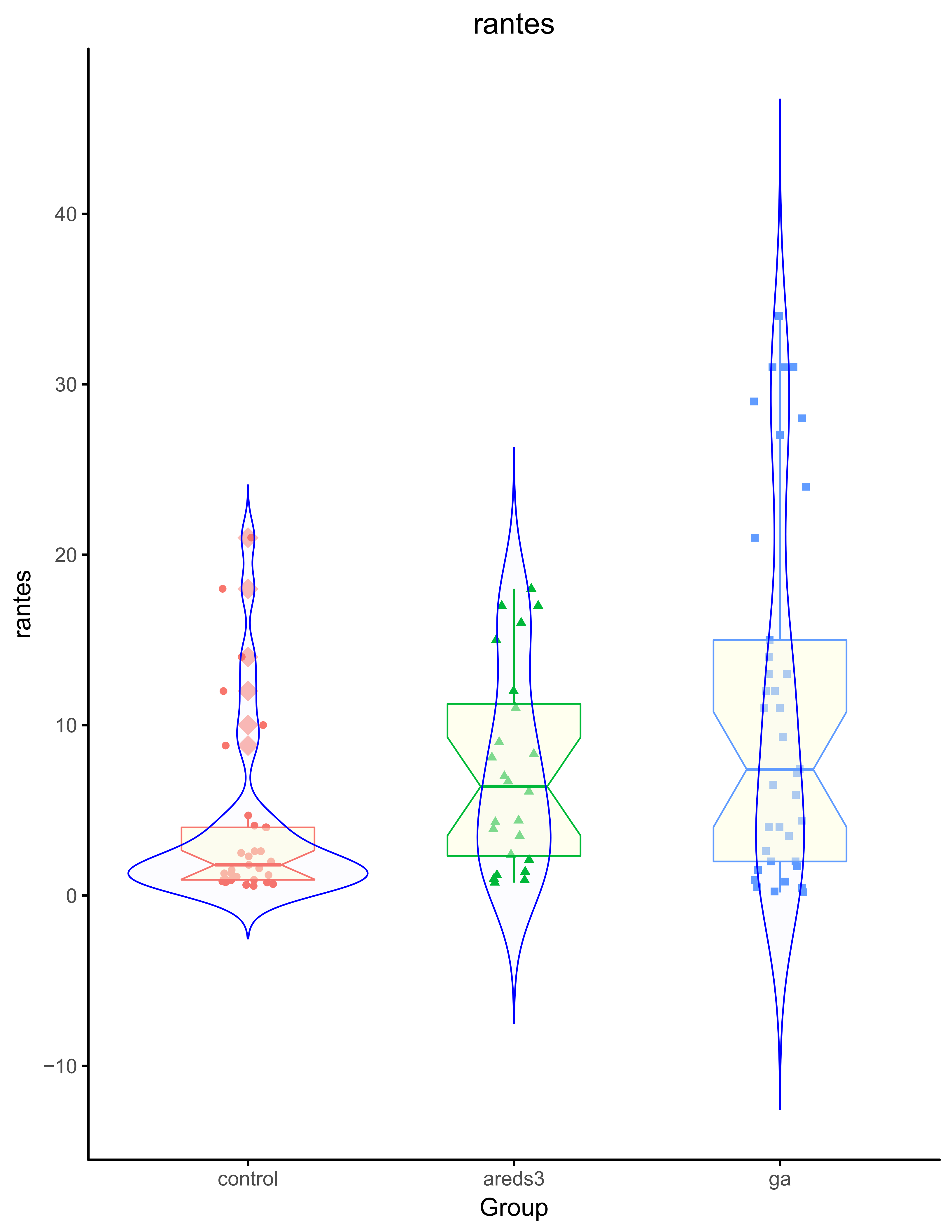
rantes
40
30
20
10
0
−10
rantes
control
areds3
ga
Group

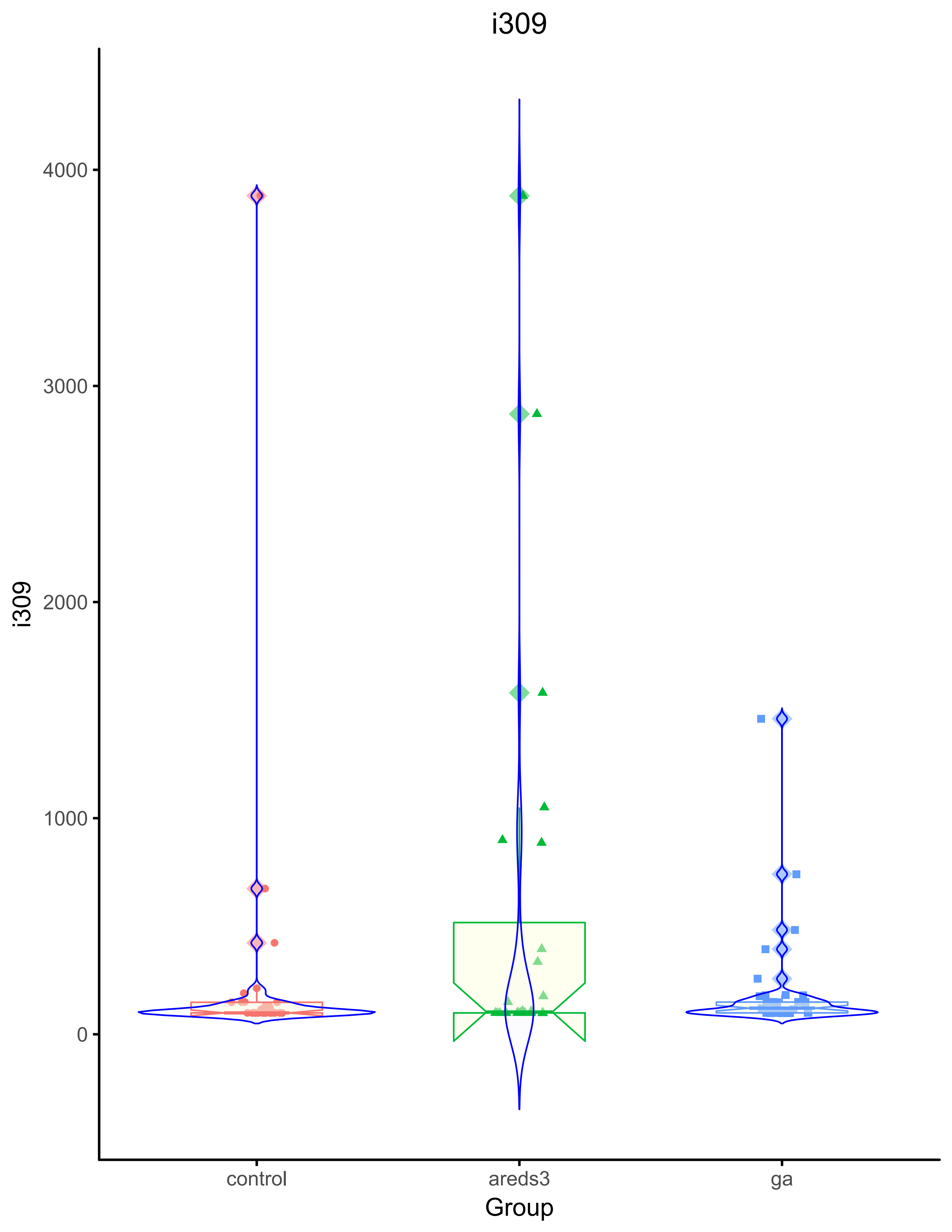
i309
4000
3000
2000
1000
0
i309
control
areds3
ga
Group

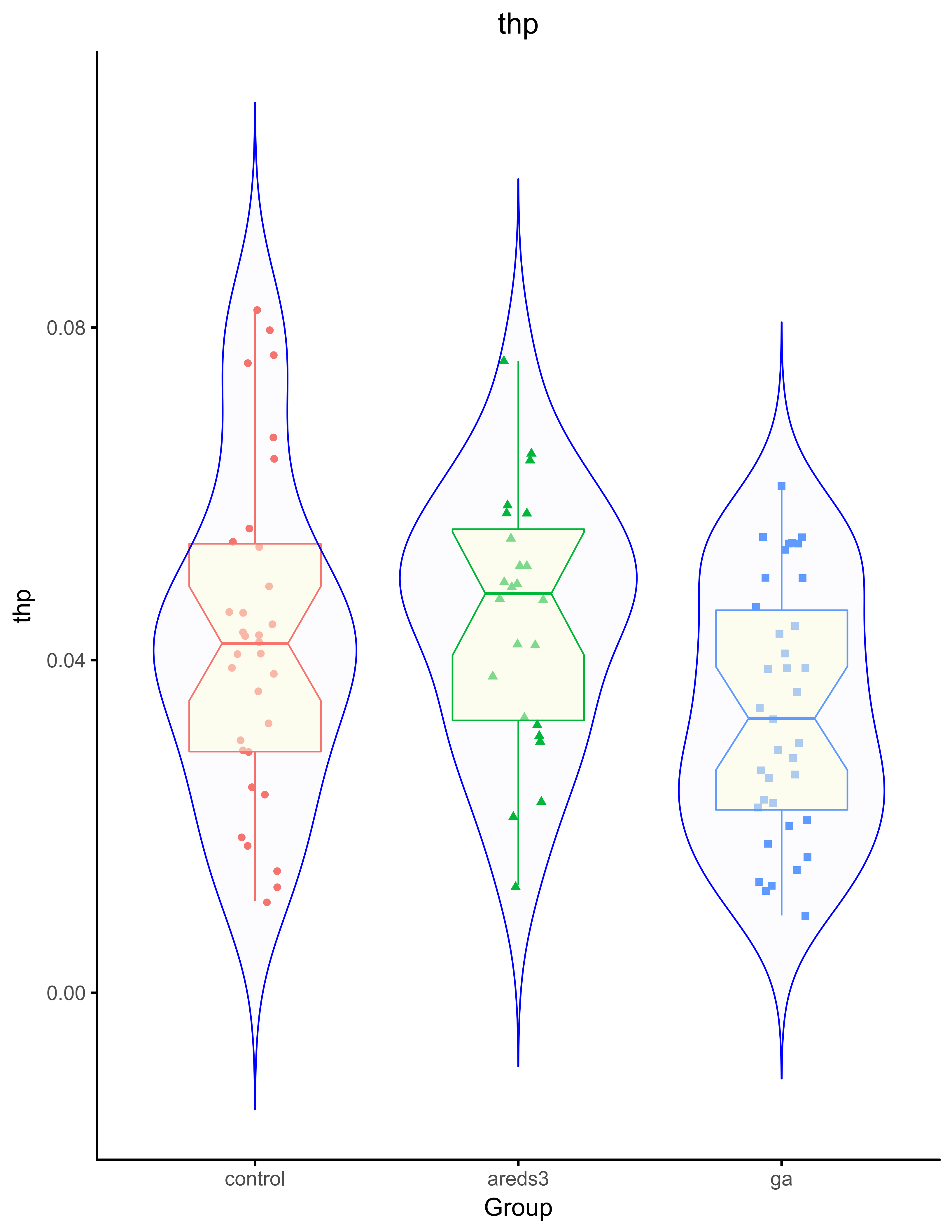
thp
thp
0.08
0.04
0.00
control
areds3
ga
Group

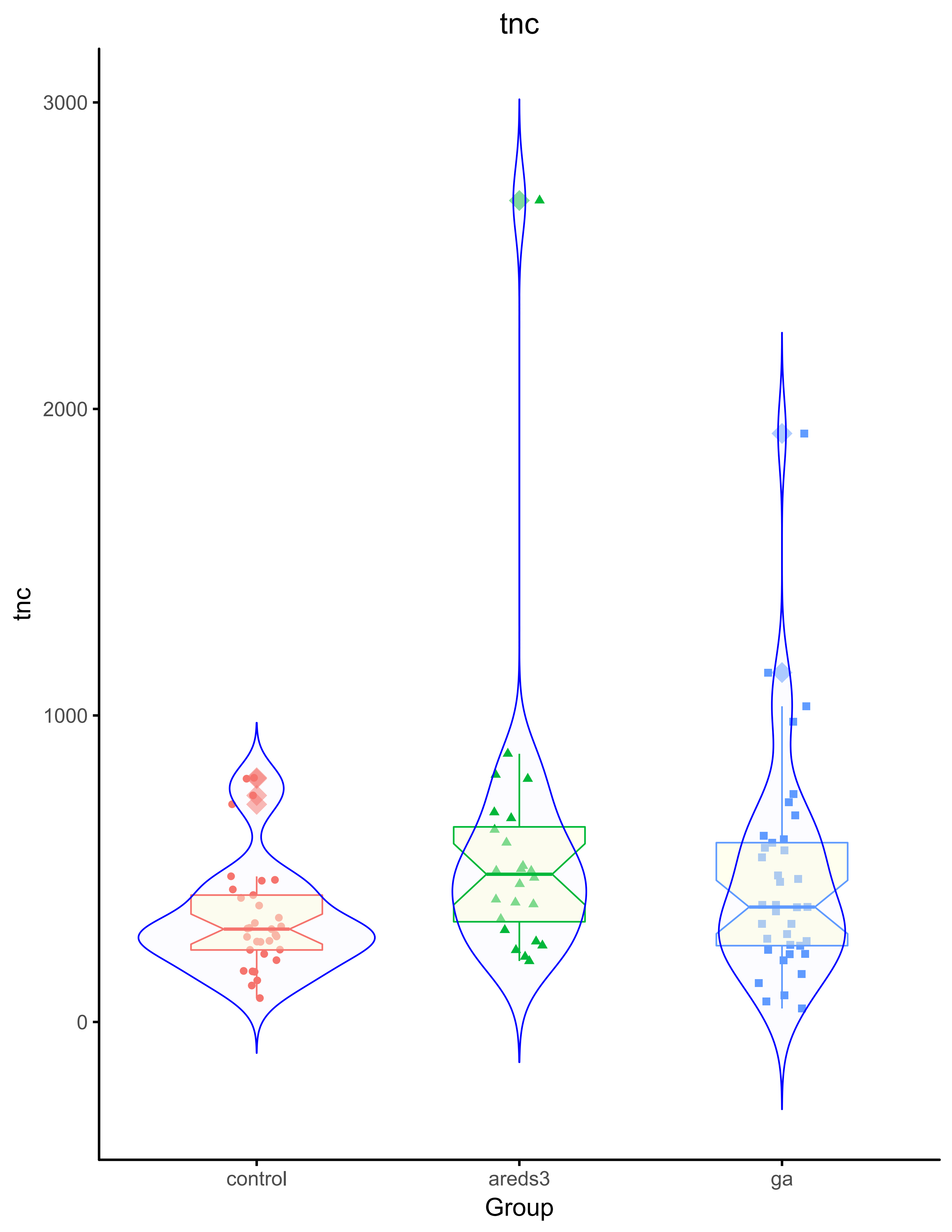
tnc
3000
2000
1000
0
tnc
control
areds3
ga
Group

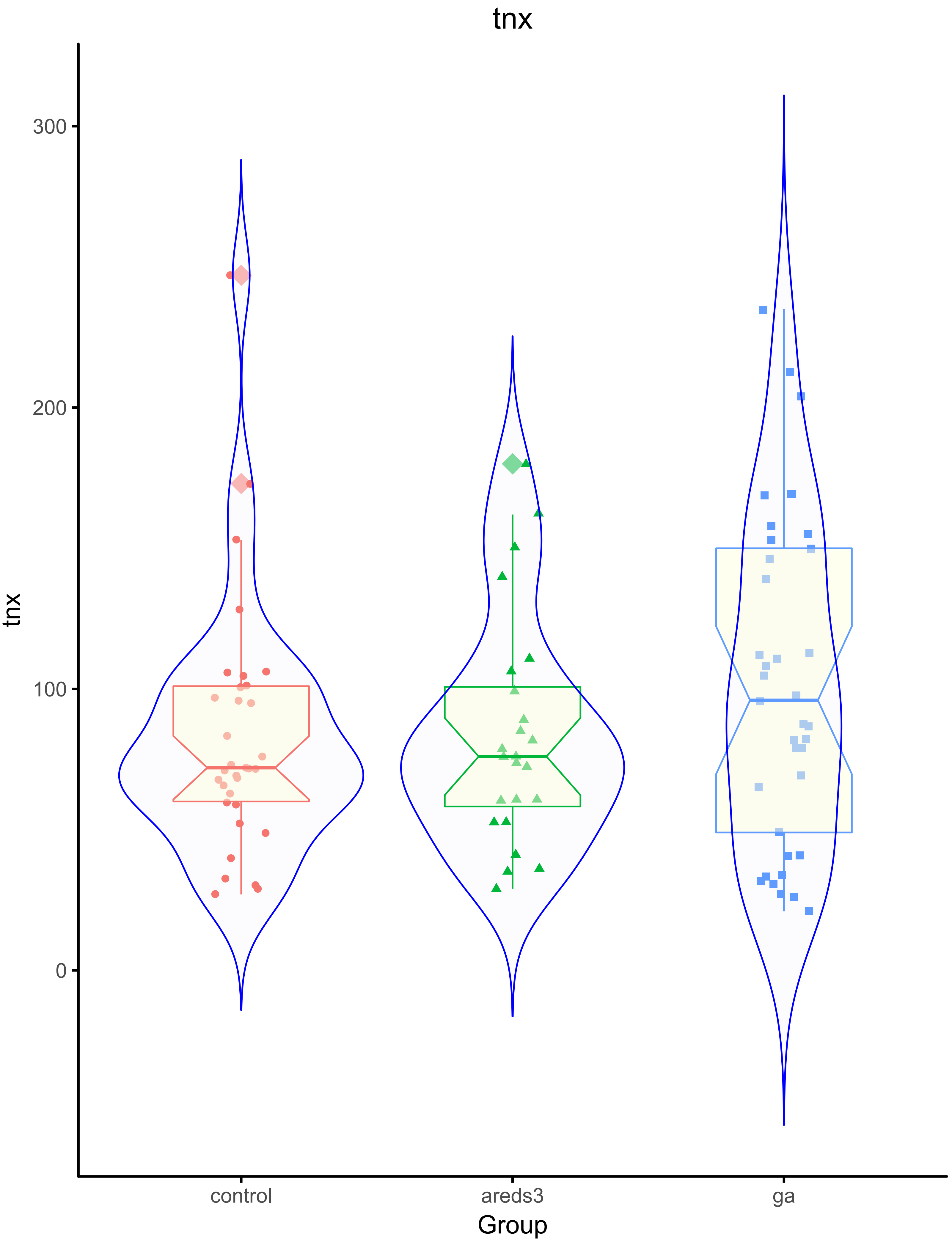

tnx
300
200
100
0
tnx
control
areds3
ga
Group

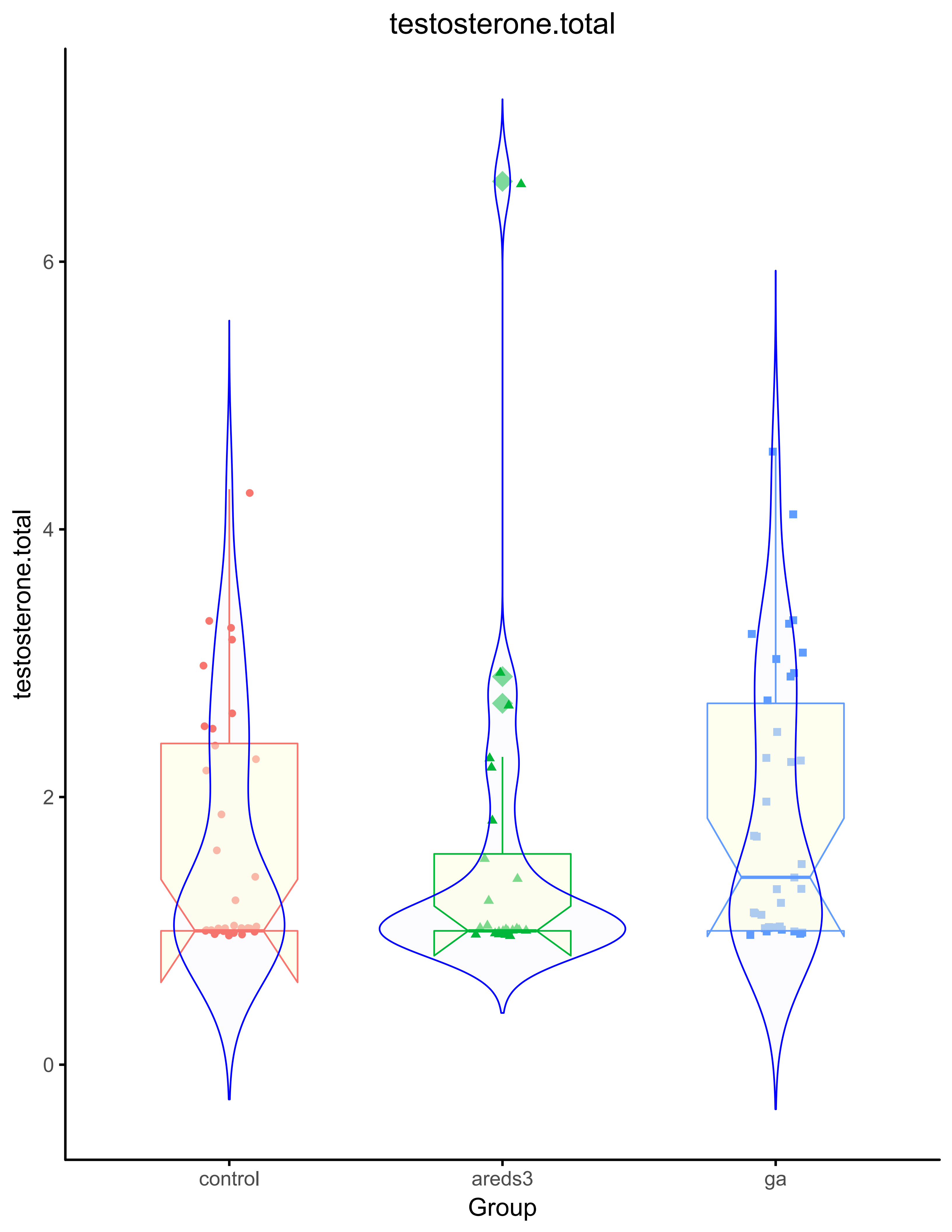
testosterone.total
testosterone.total
0
2
4
6
control
areds3
ga
Group

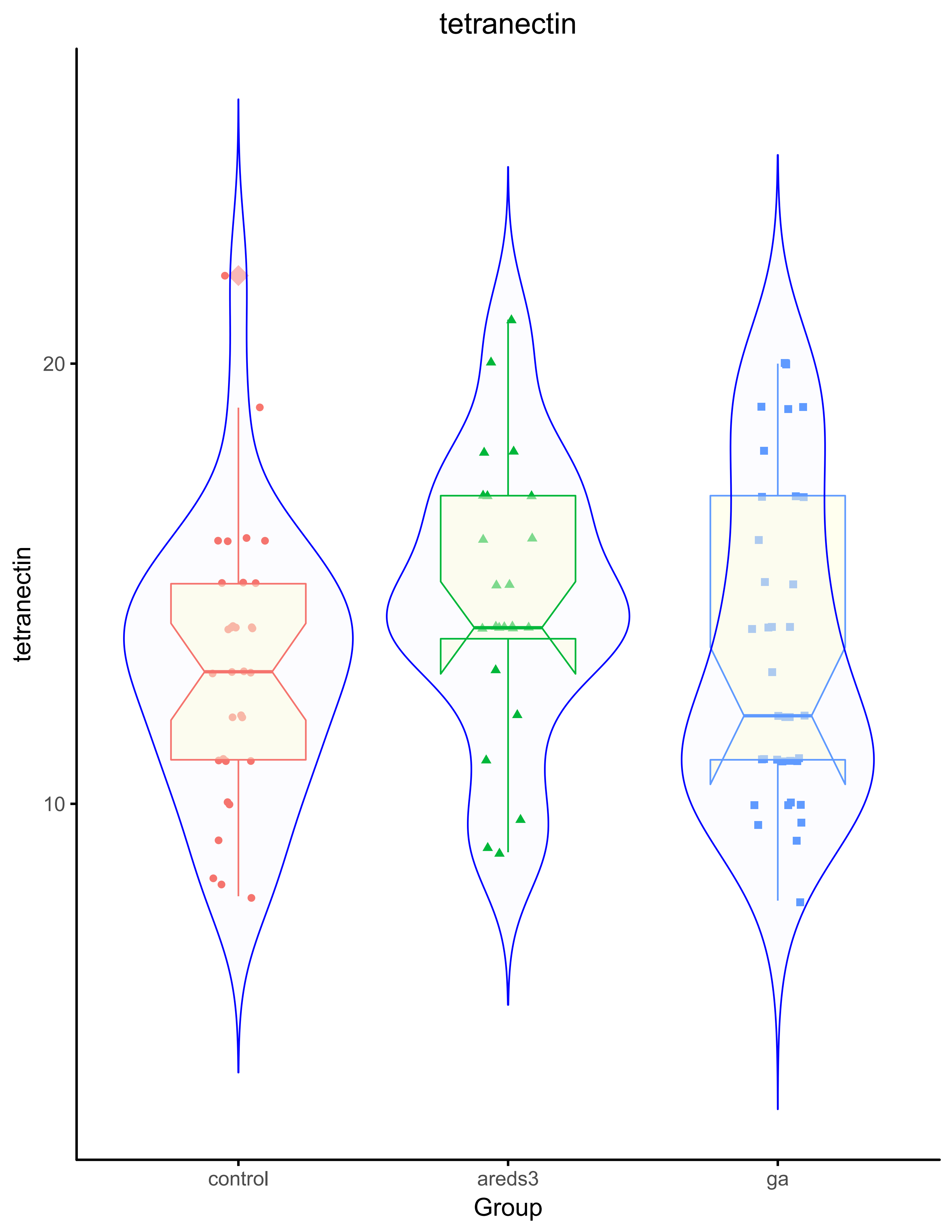
tetranectin
20
10
tetranectin
control
areds3
ga
Group

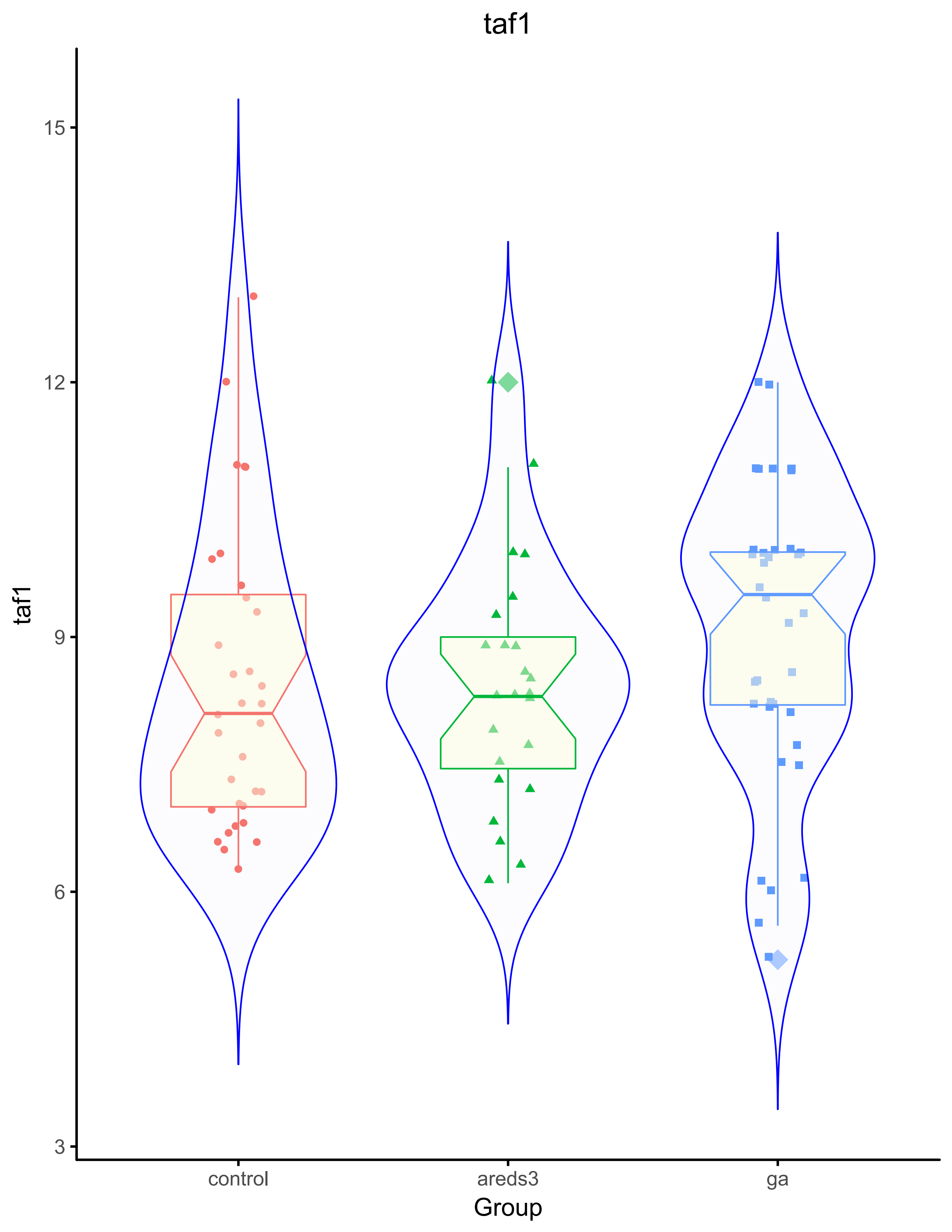
taf1
15
12
9
6
3
taf1
control
areds3
ga
Group

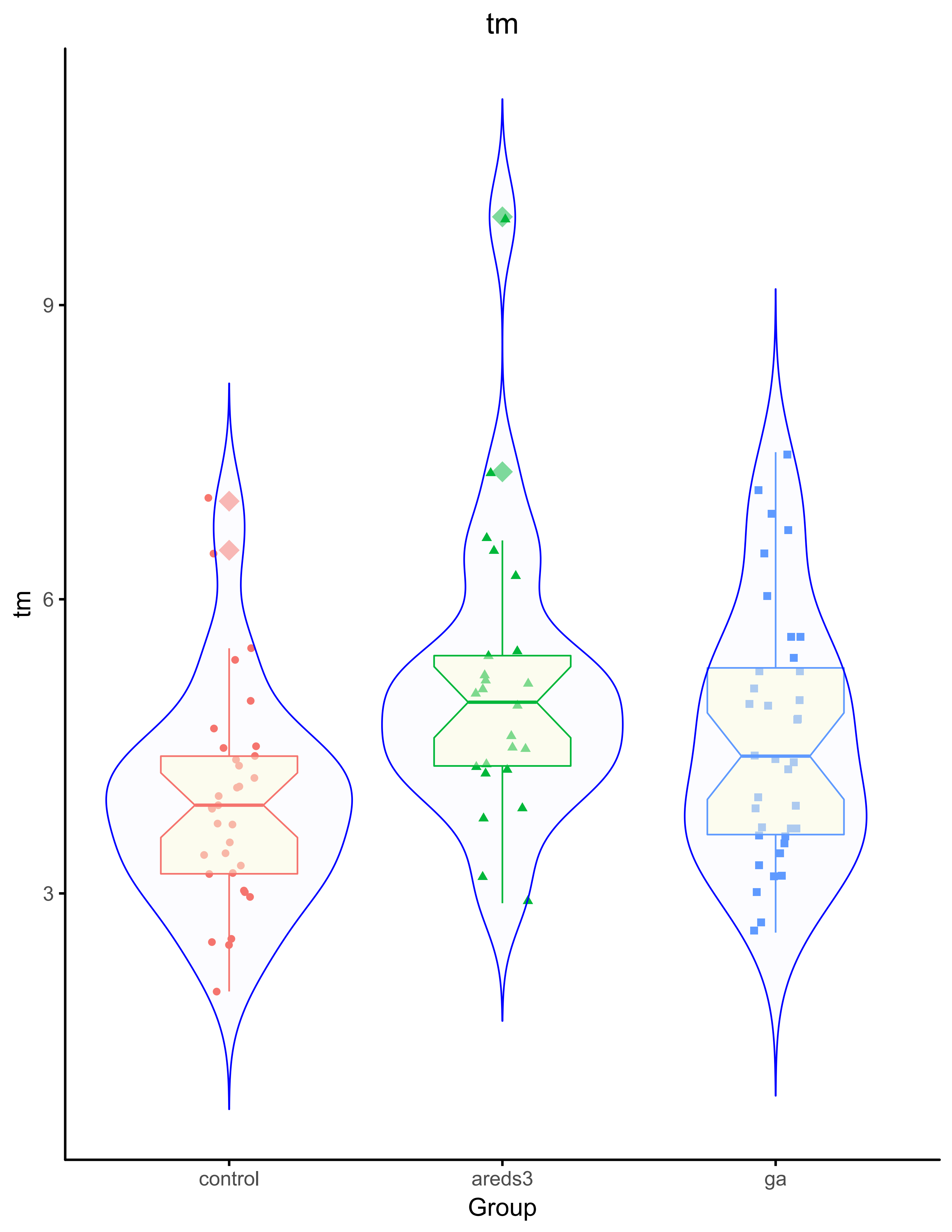
tm
9
6
3
tm
control
areds3
ga
Group

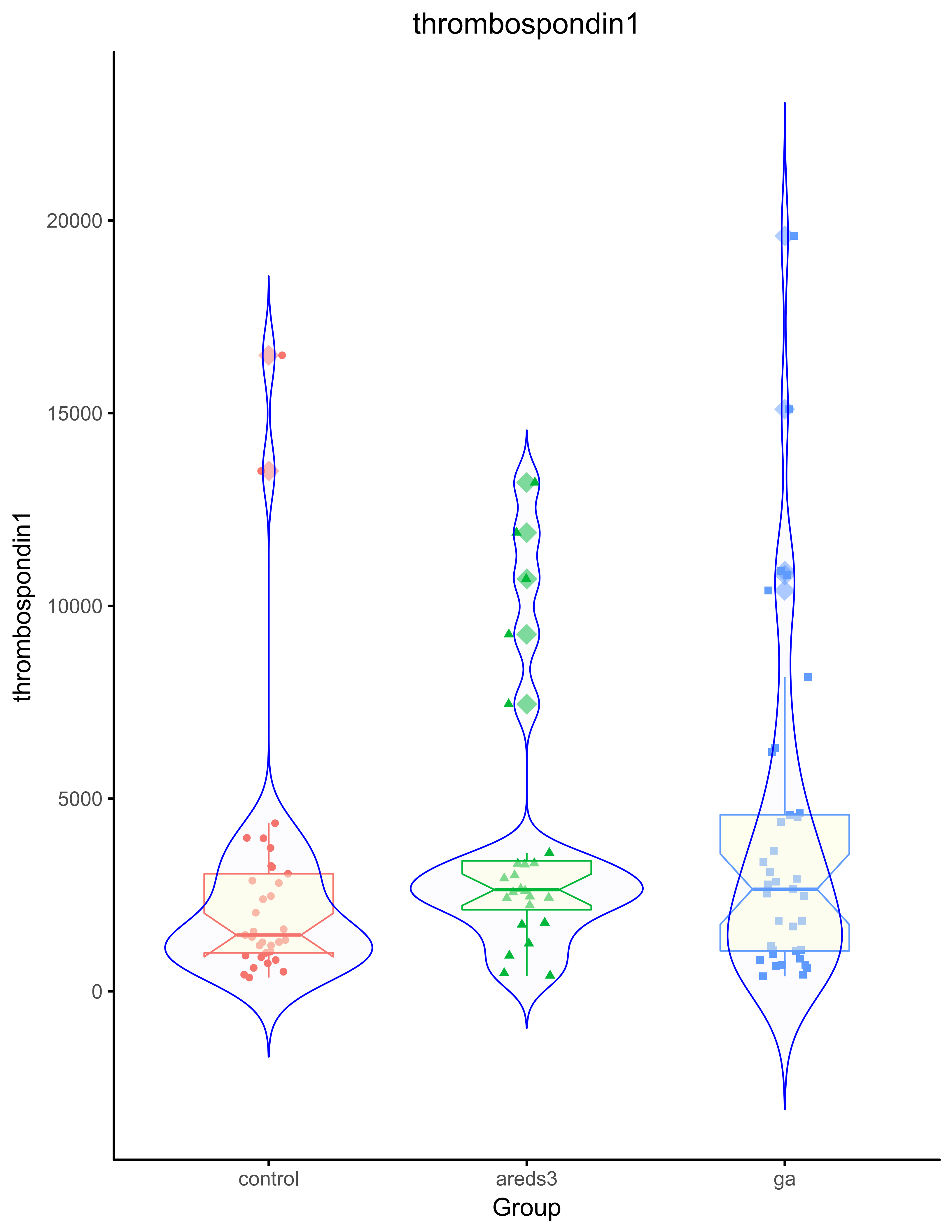
thrombospondin1
20000
15000
10000
5000
0
thrombospondin1
control
areds3
ga
Group

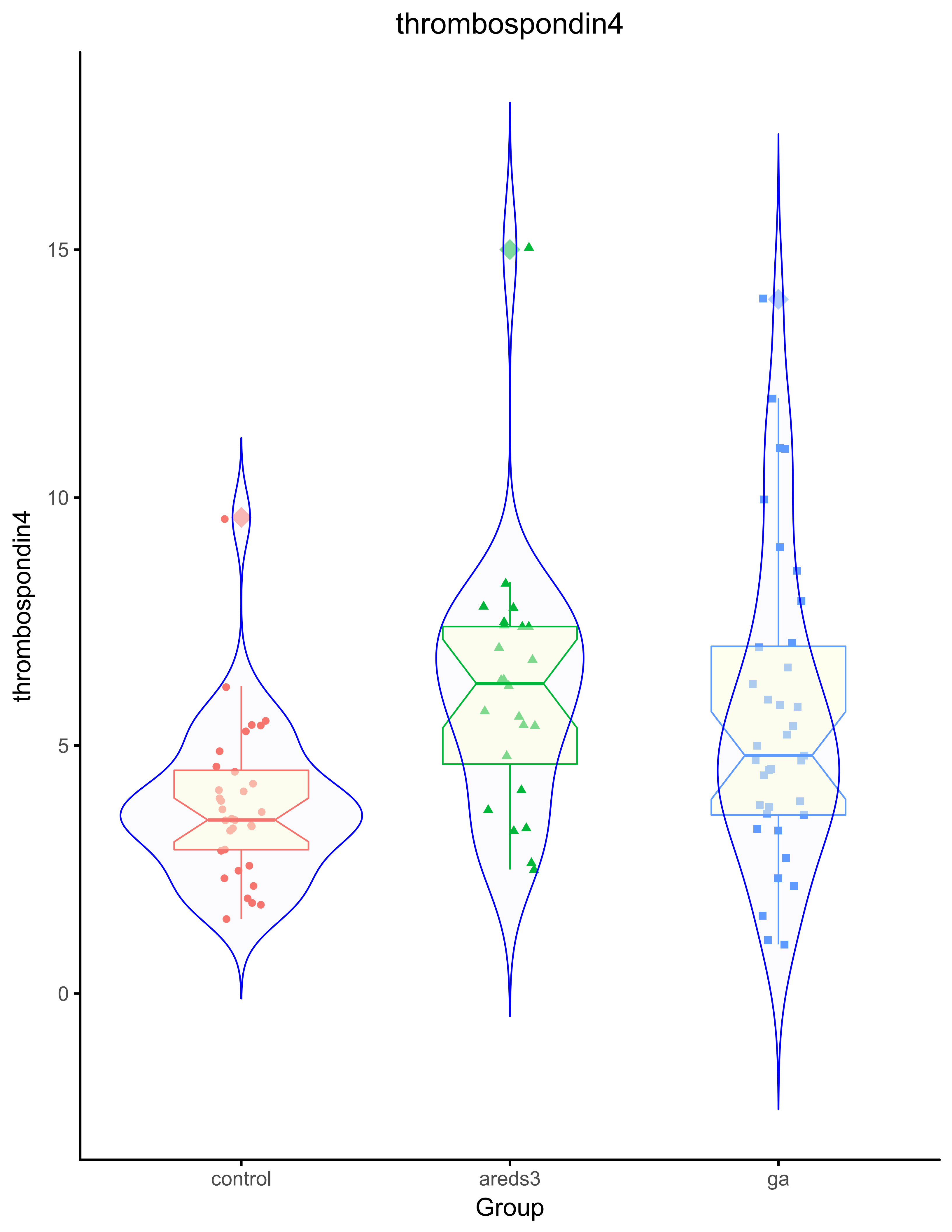
thrombospondin4
thrombospondin4
15
10
5
0
control
areds3
ga
Group

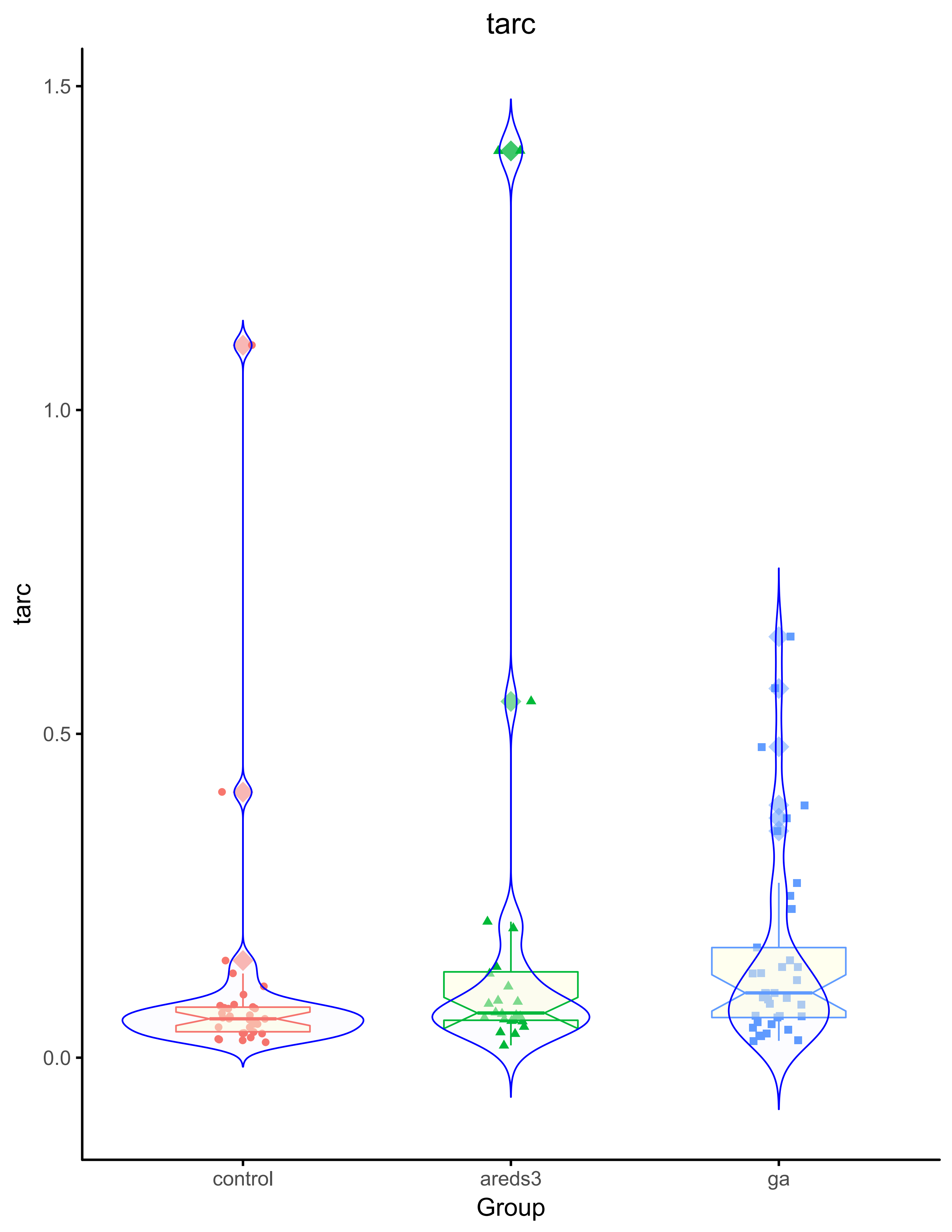

tarc
1.5
1.0
0.5
0.0
tarc
control
areds3
ga
Group

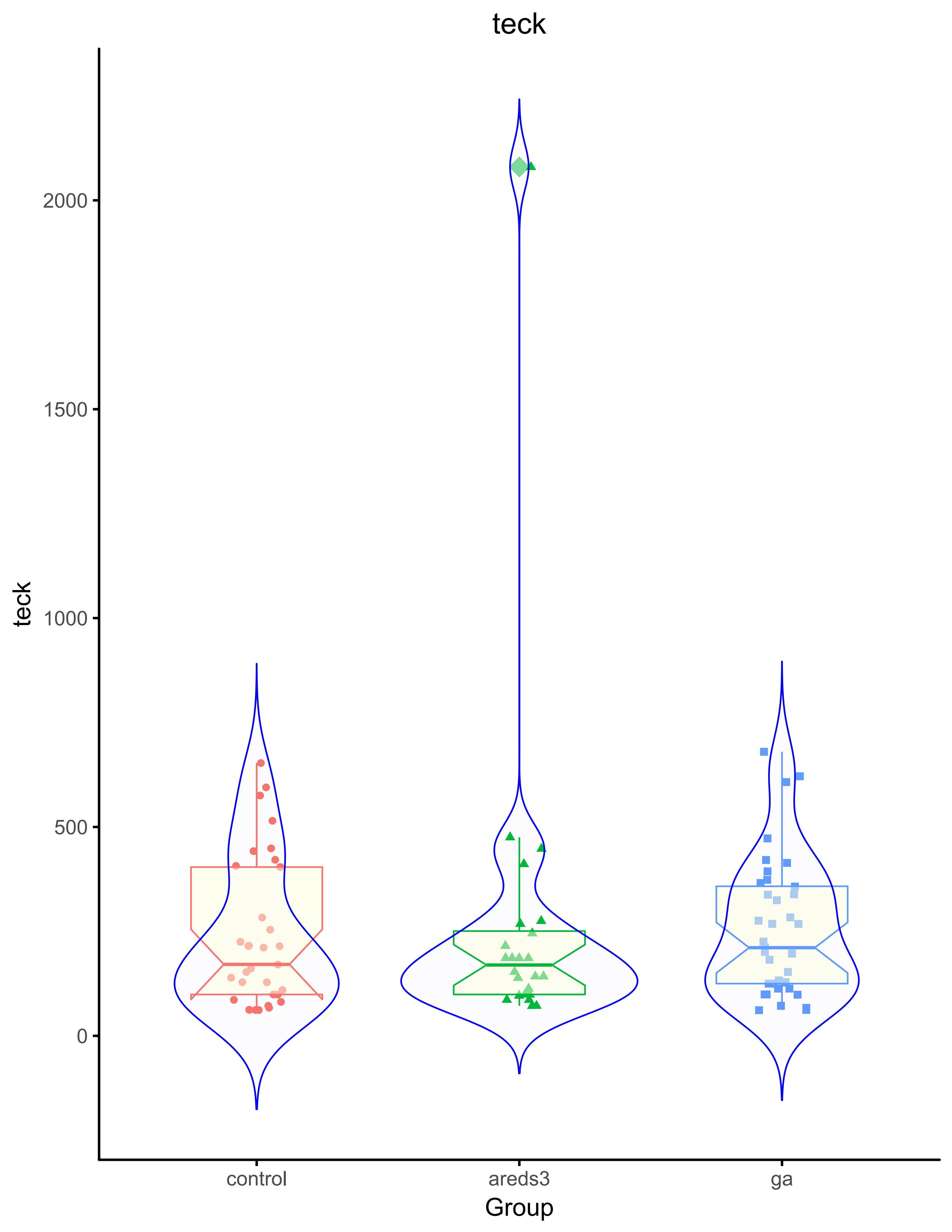
teck
teck
2000
1500
1000
500
0
control
areds3
ga
Group

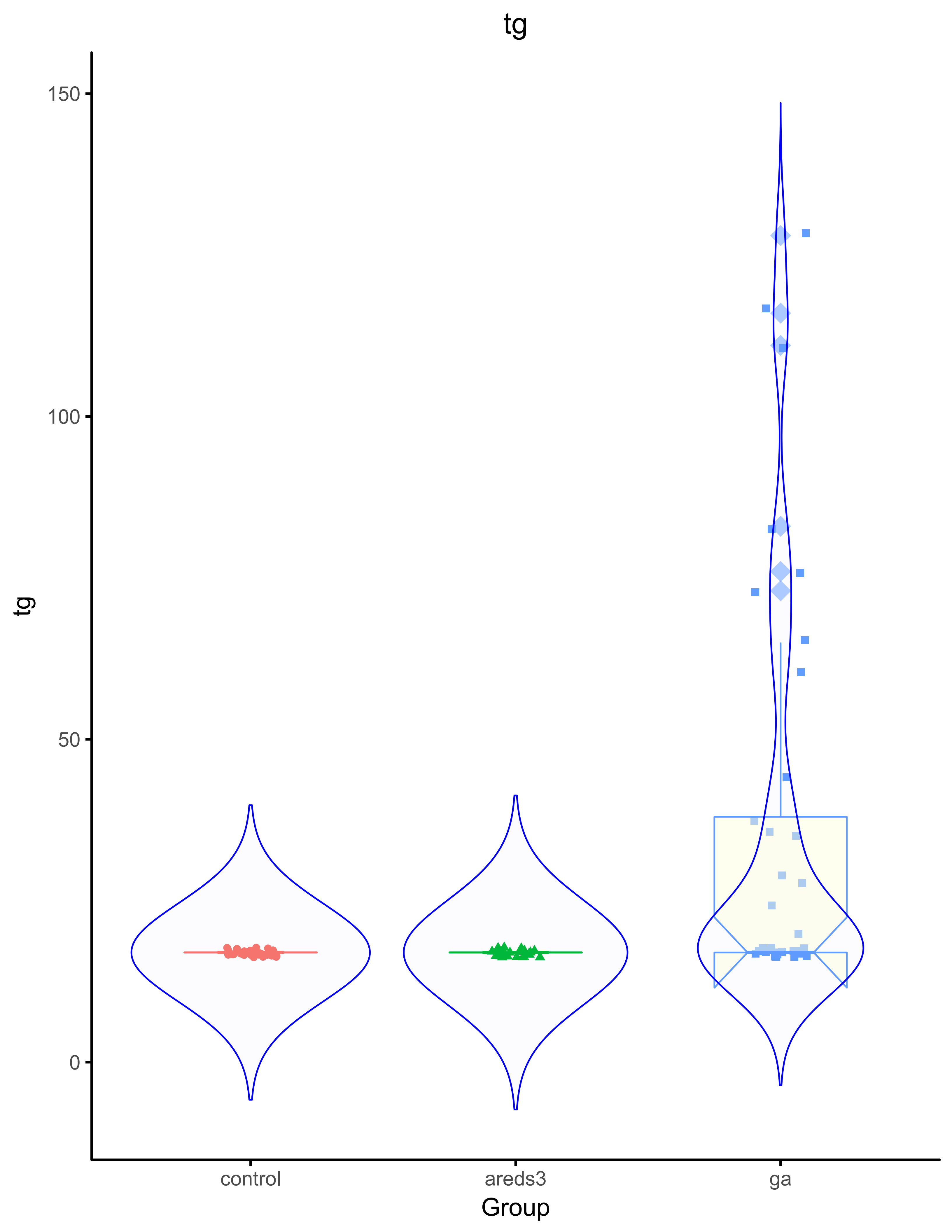

tg
150
100
50
0
tg
control
areds3
ga
Group

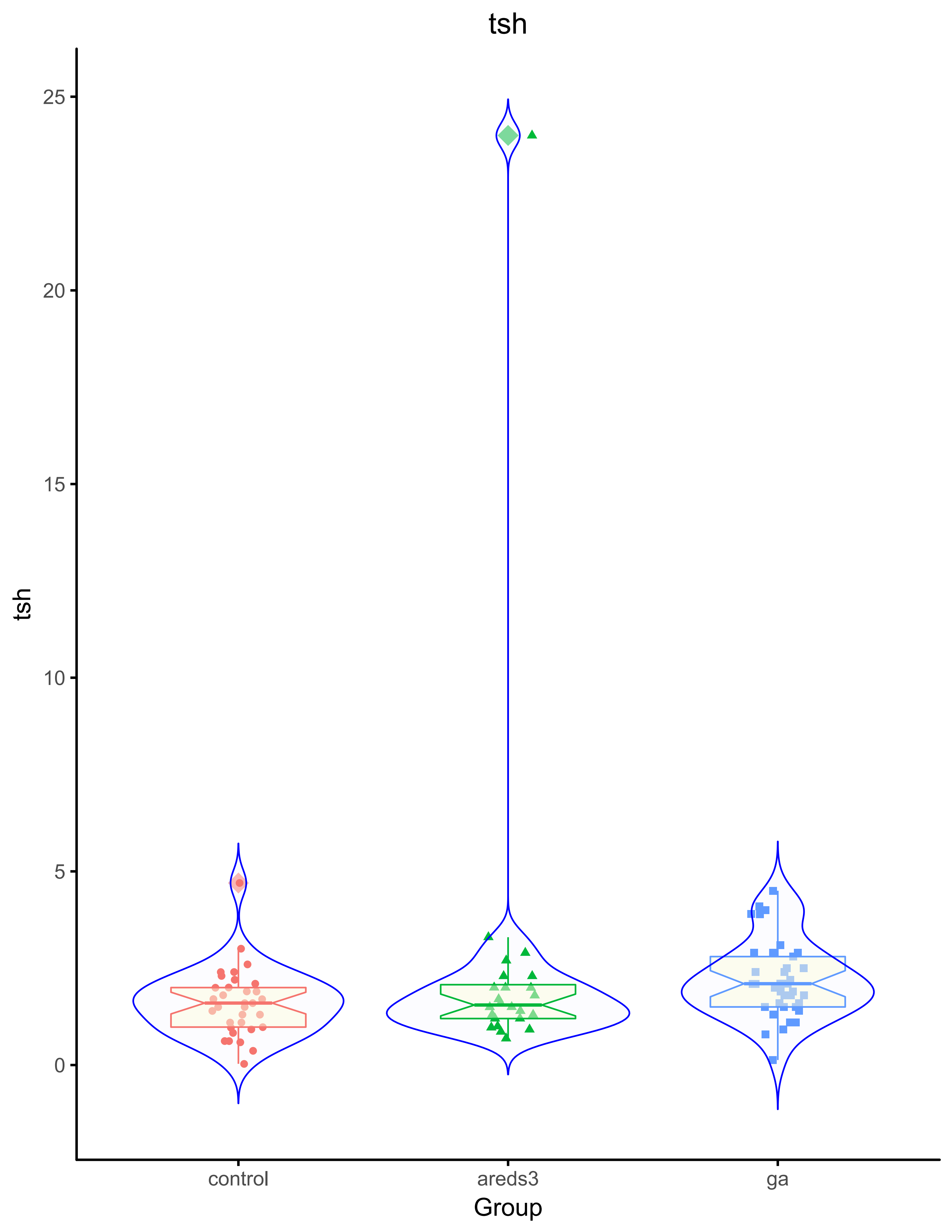

tsh
25
20
15
10
5
0
tsh
control
areds3
ga
Group

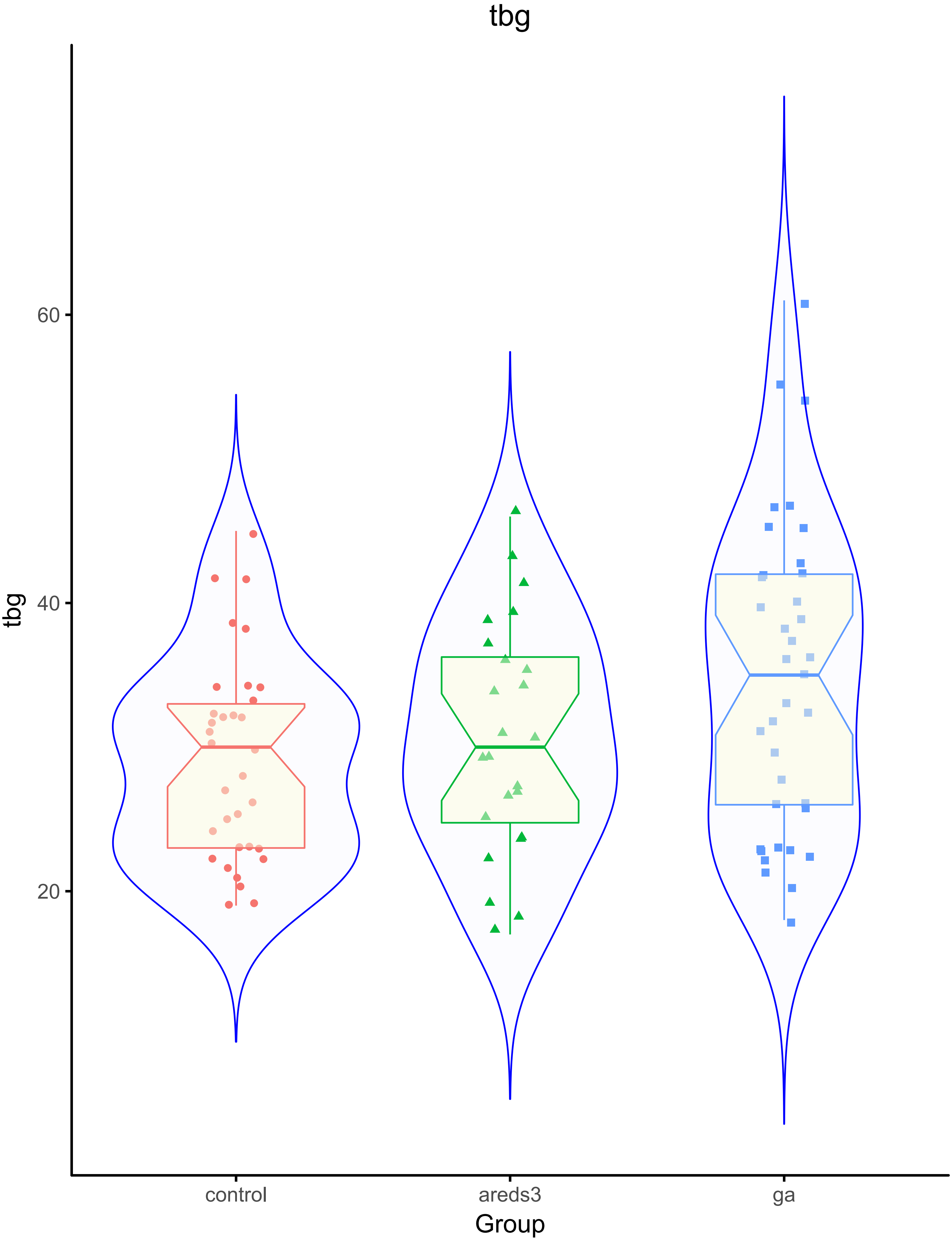
tbg
60
40
20
tbg
control
areds3
ga
Group

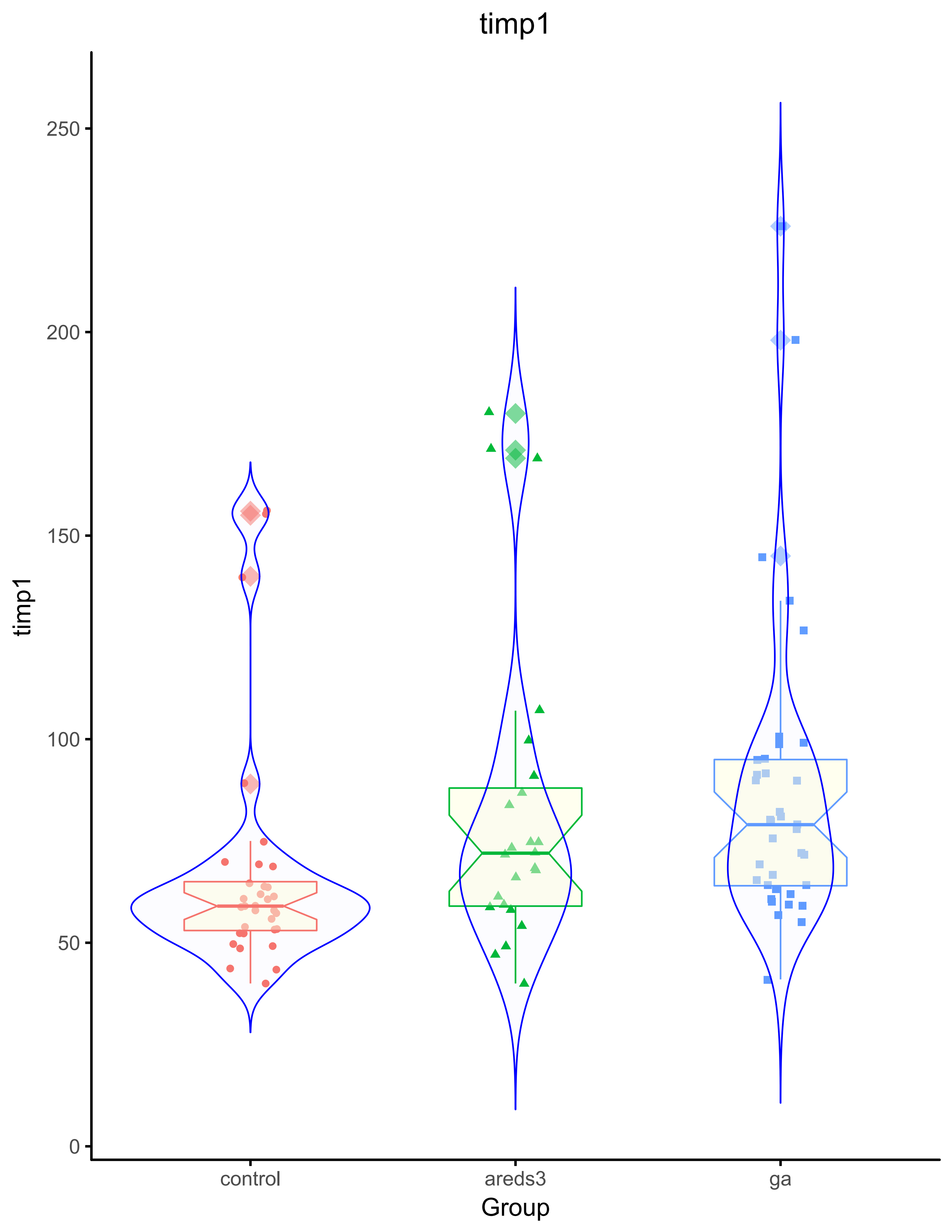
timp1
250
200
150
100
50
0
timp1
control
areds3
ga
Group

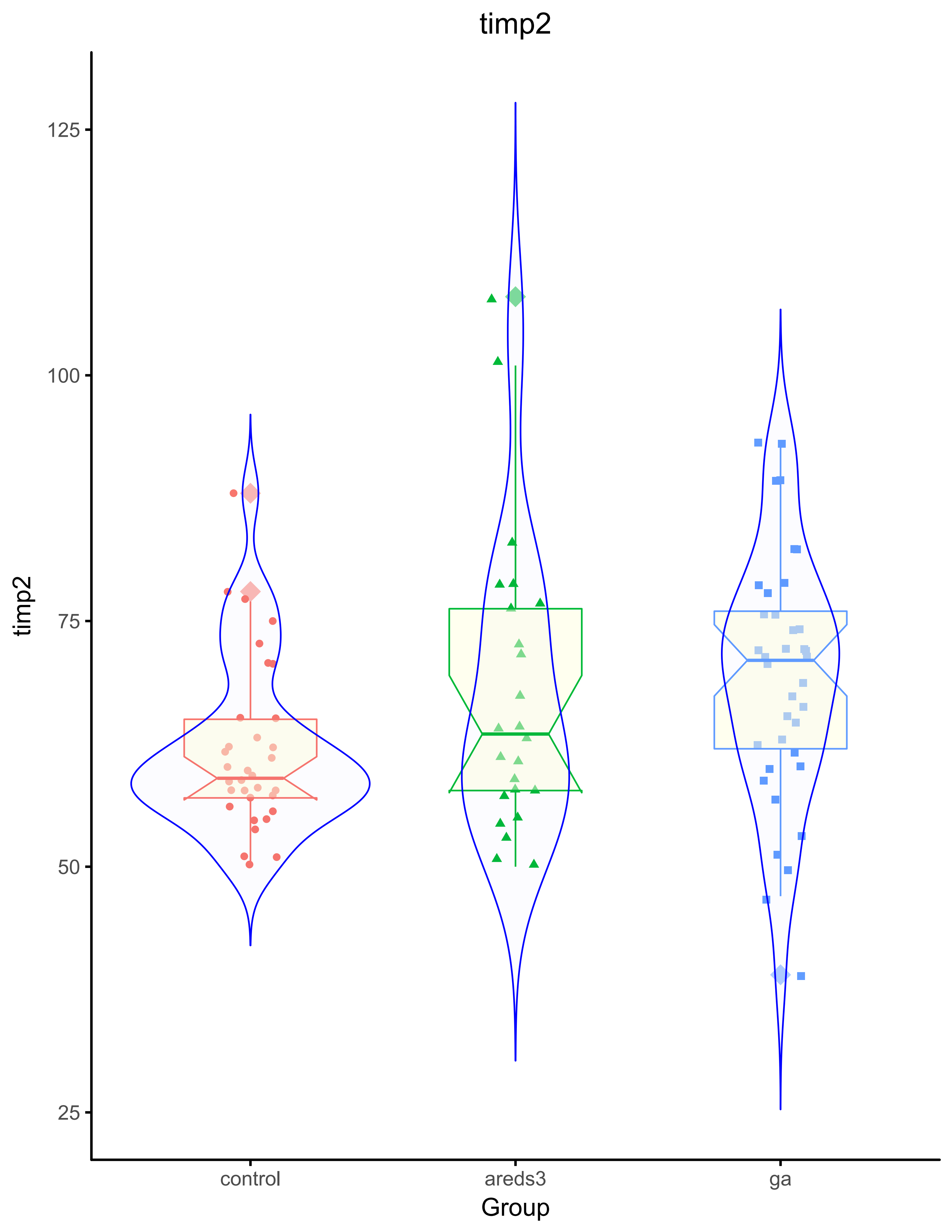
timp2
125
100
75
50
25
timp2
control
areds3
ga
Group

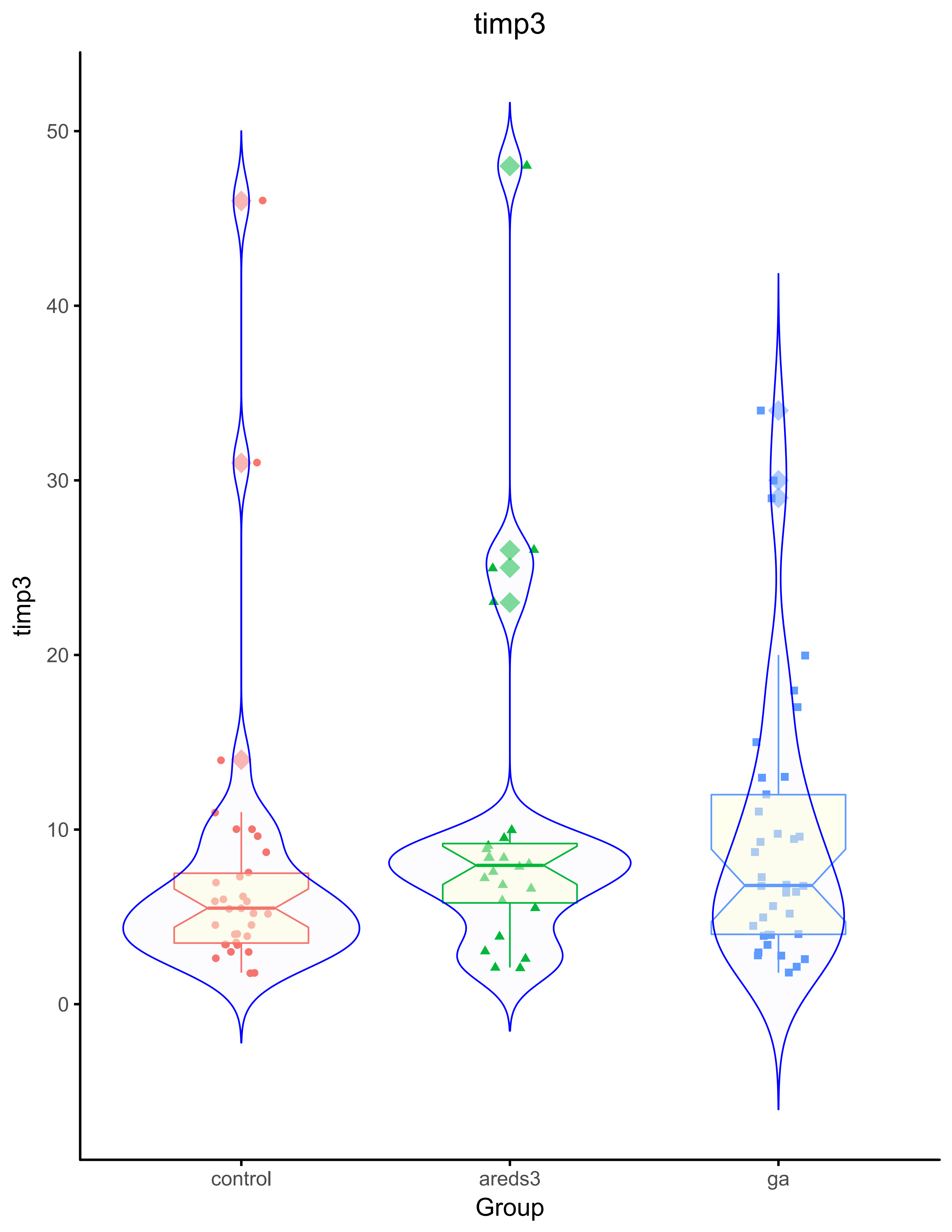
timp3
50
40
30
20
10
0
timp3
control
areds3
ga
Group

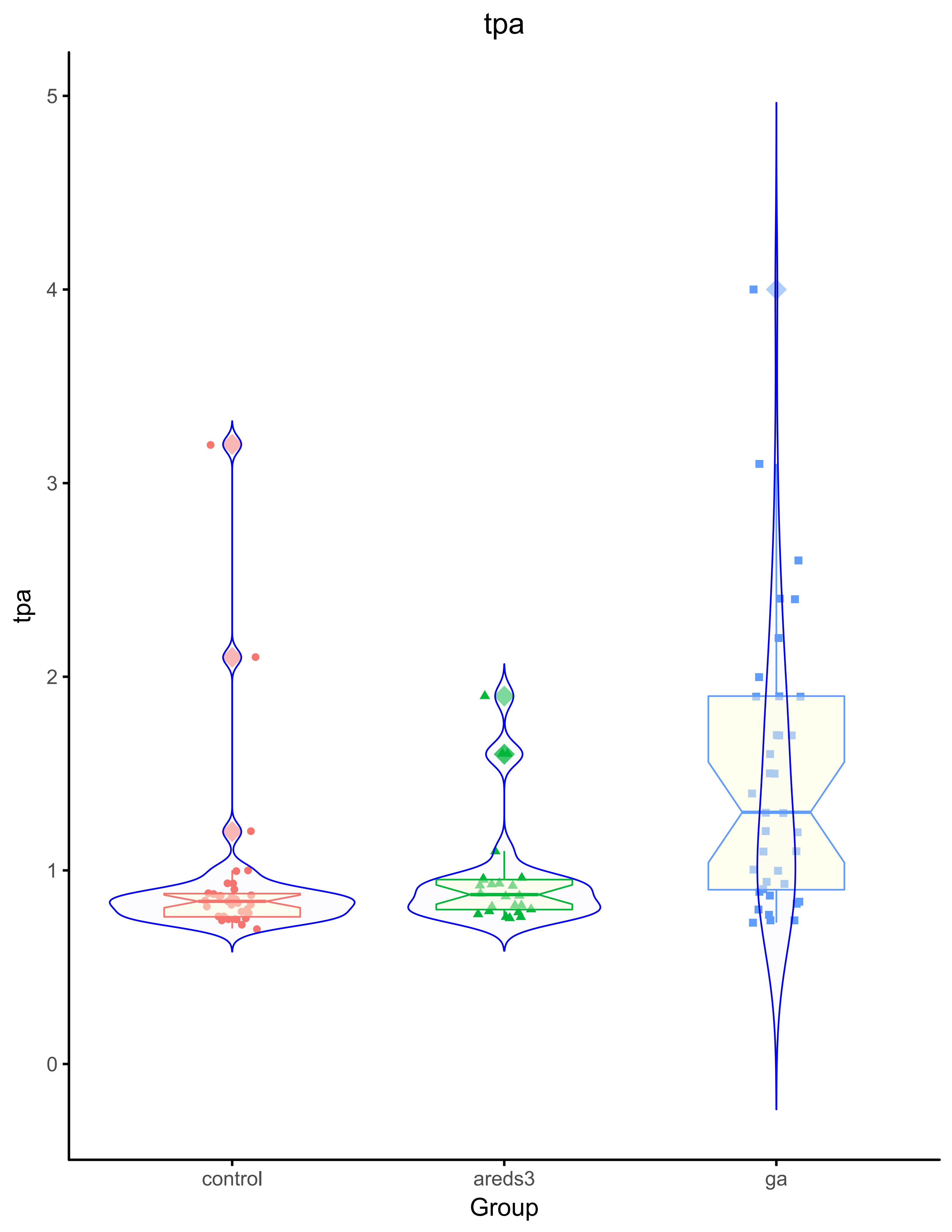
tpa
tpa
5
4
3
2
1
0
control
areds3
ga
Group

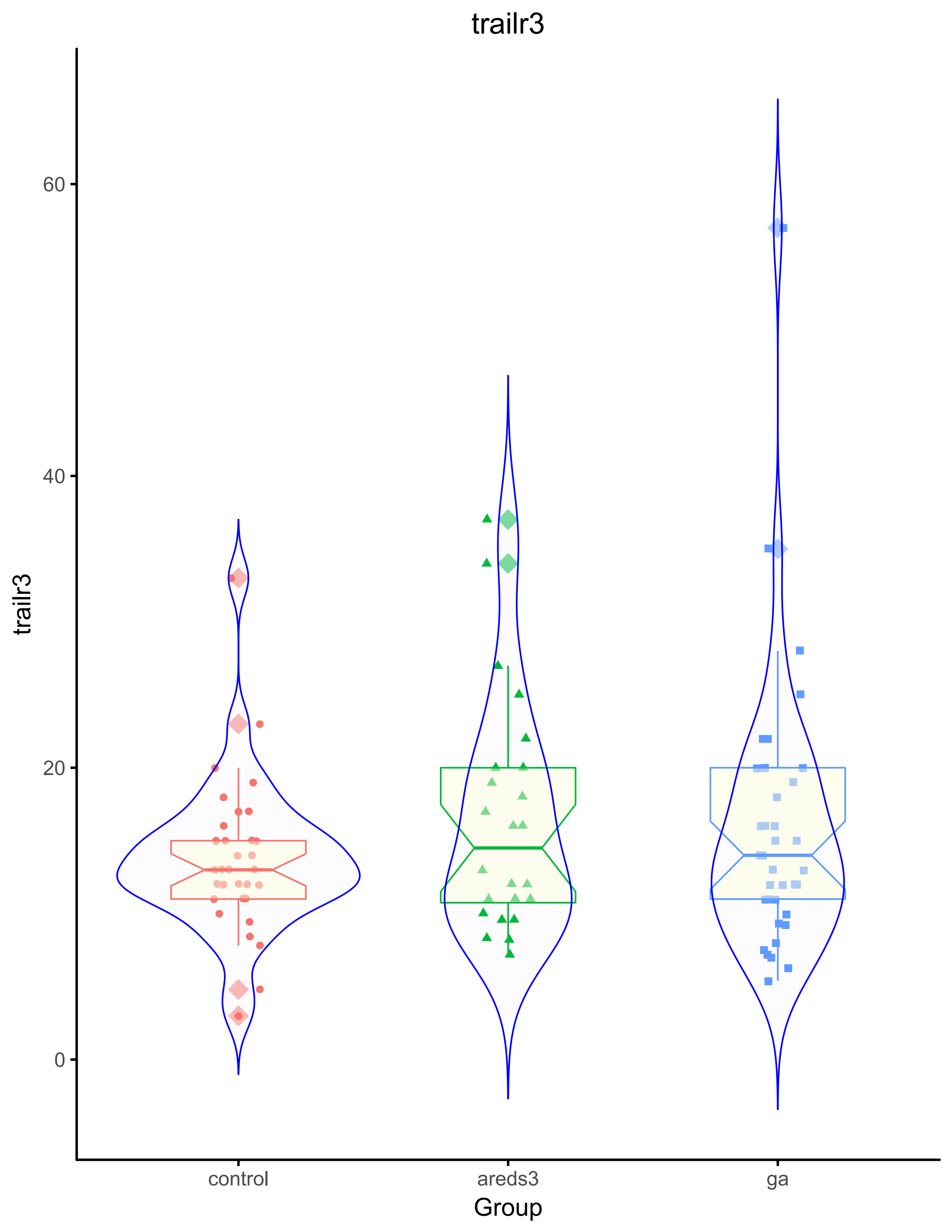

trailr3
trailr3
60
40
20
0
control
areds3
ga
Group

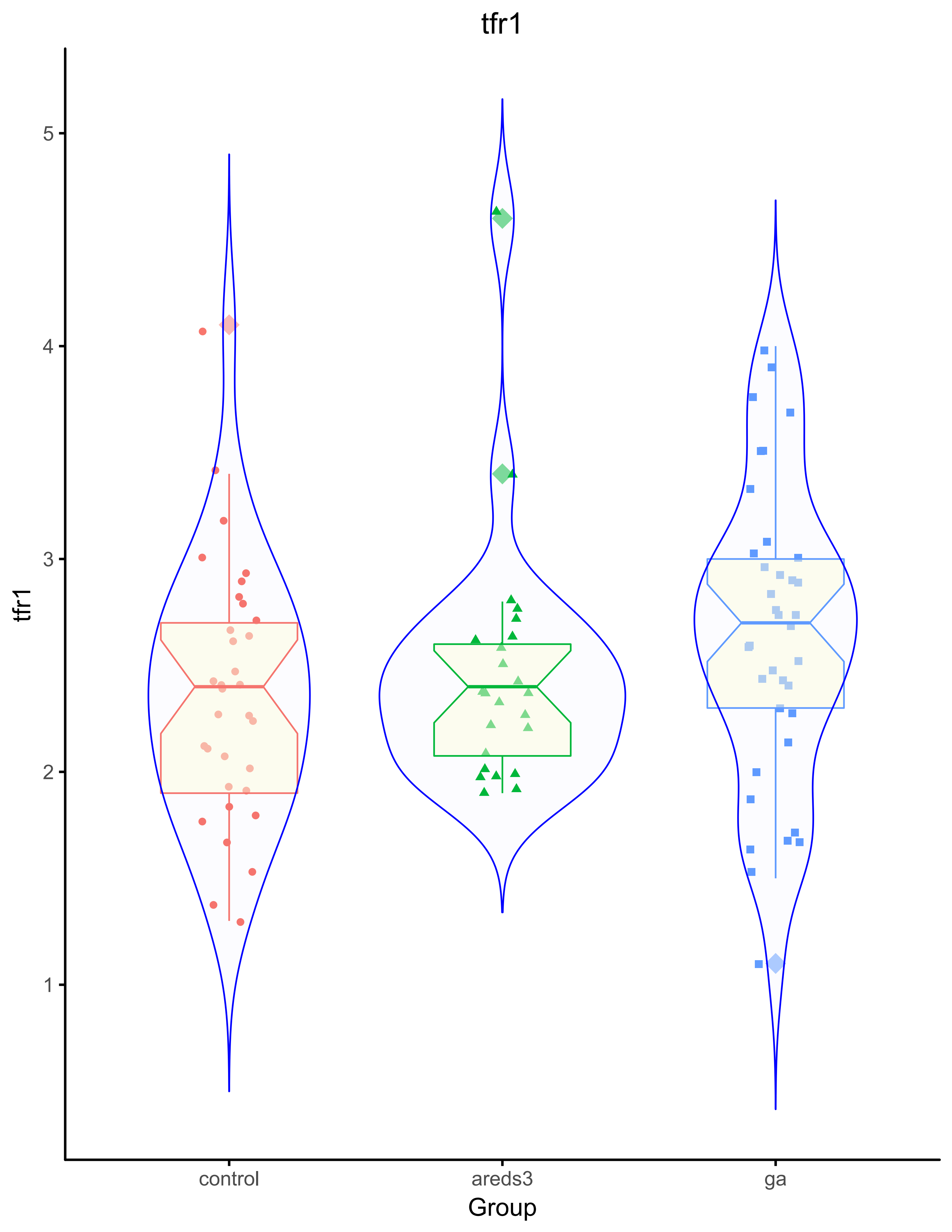

tfr1
tfr1
5
4
3
2
1
control
areds3
ga
Group

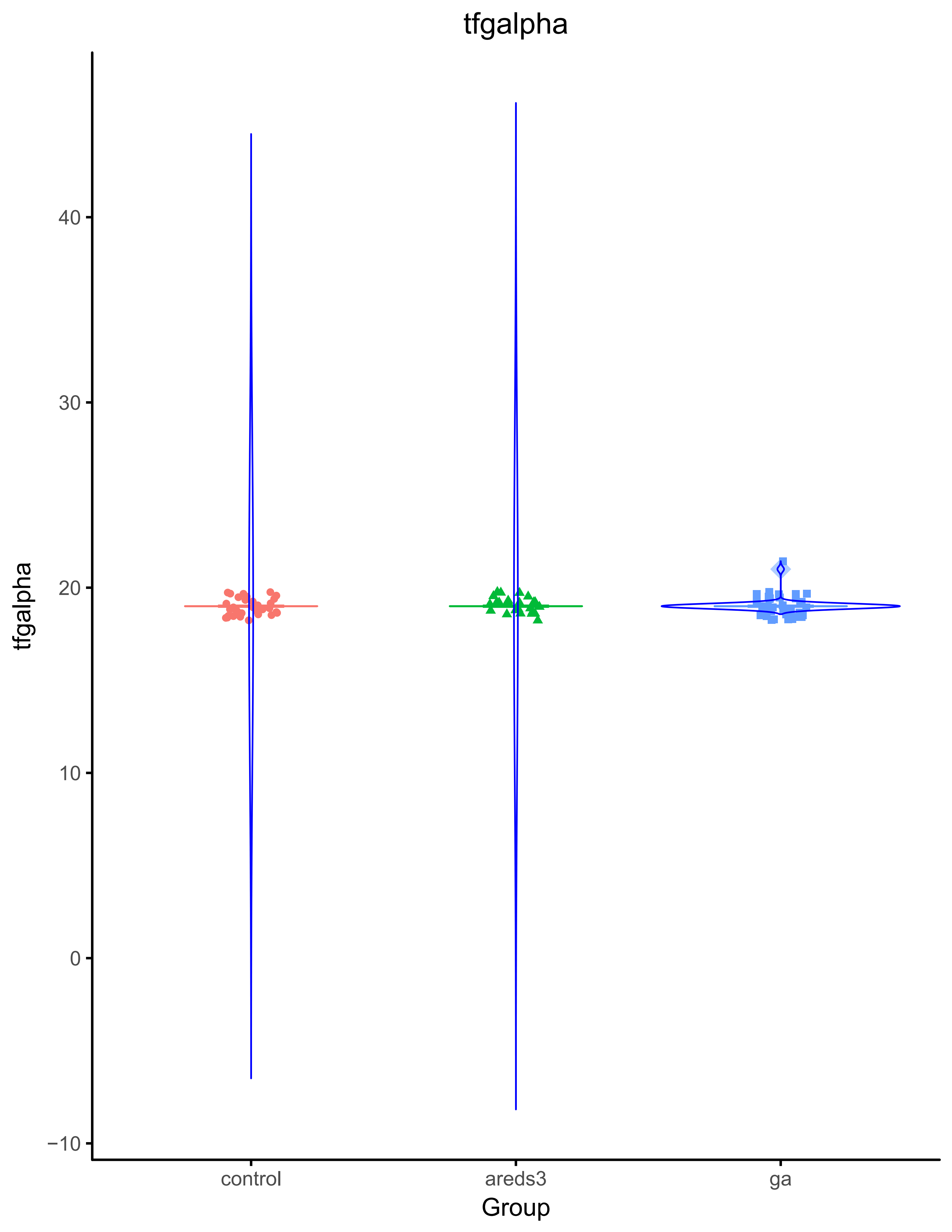
tfgalpha
tfgalpha
40
30
20
10
0
−10
control
areds3
ga
Group

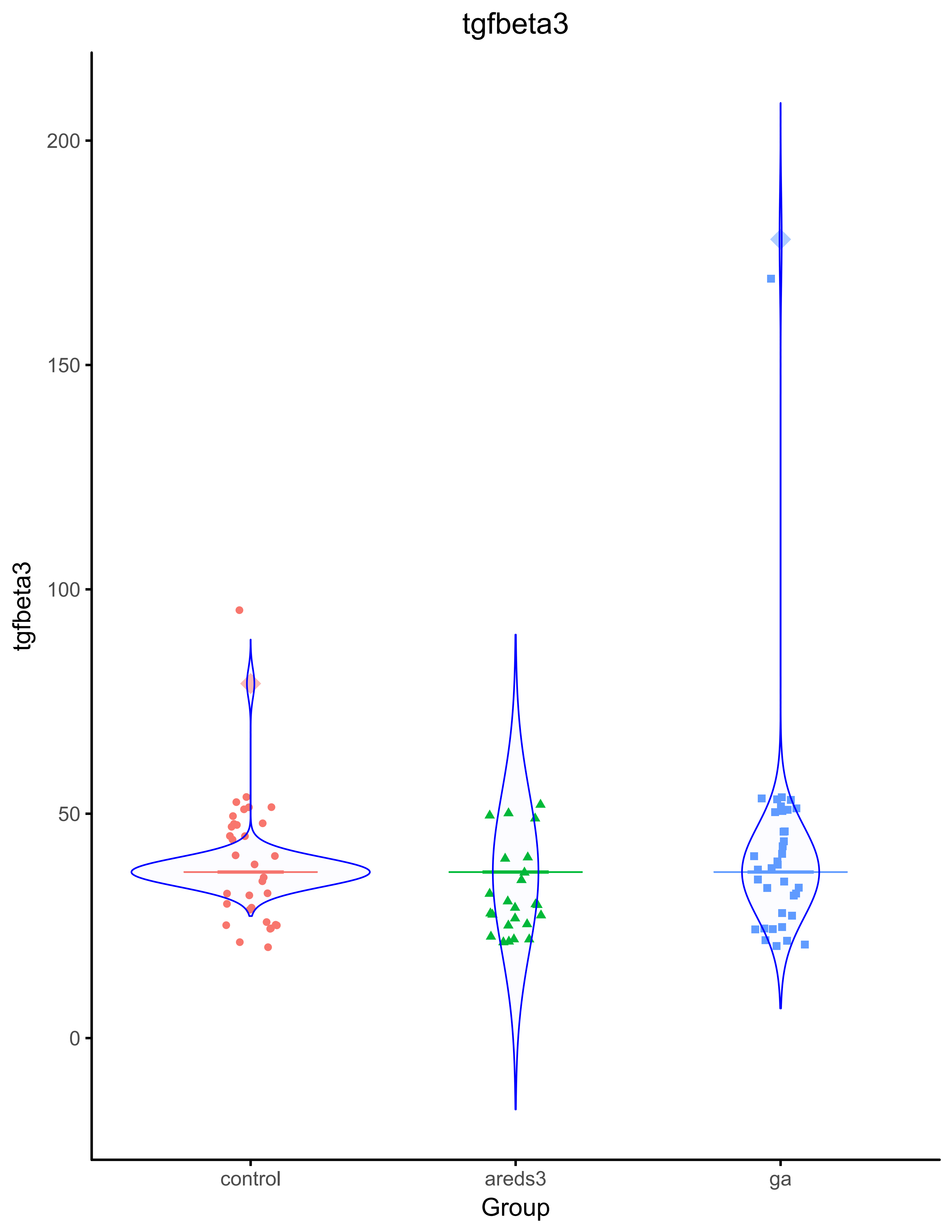

tgfbeta3
200
150
100
50
0
tgfbeta3
control
areds3
ga
Group

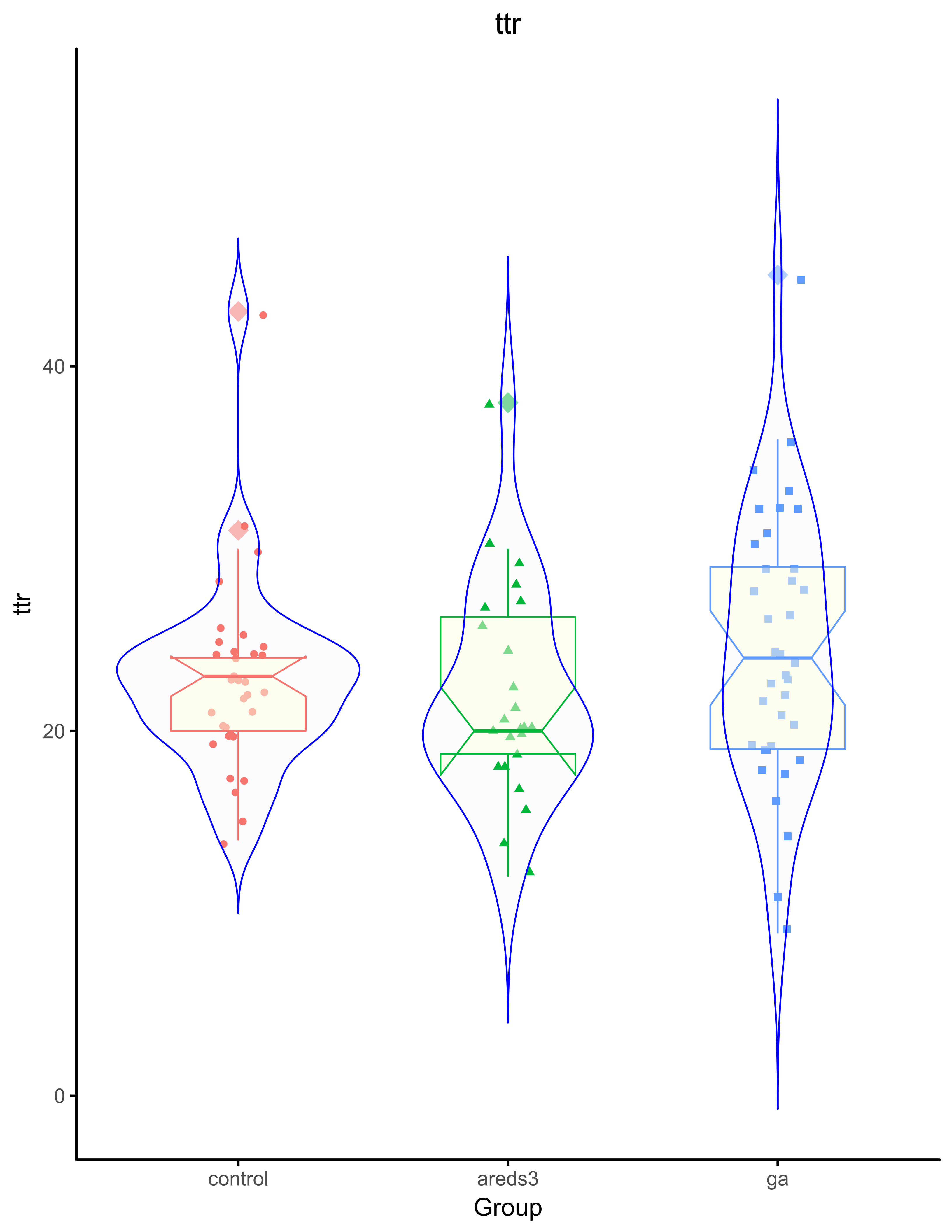

ttr
40
20
0
ttr
control
areds3
ga
Group

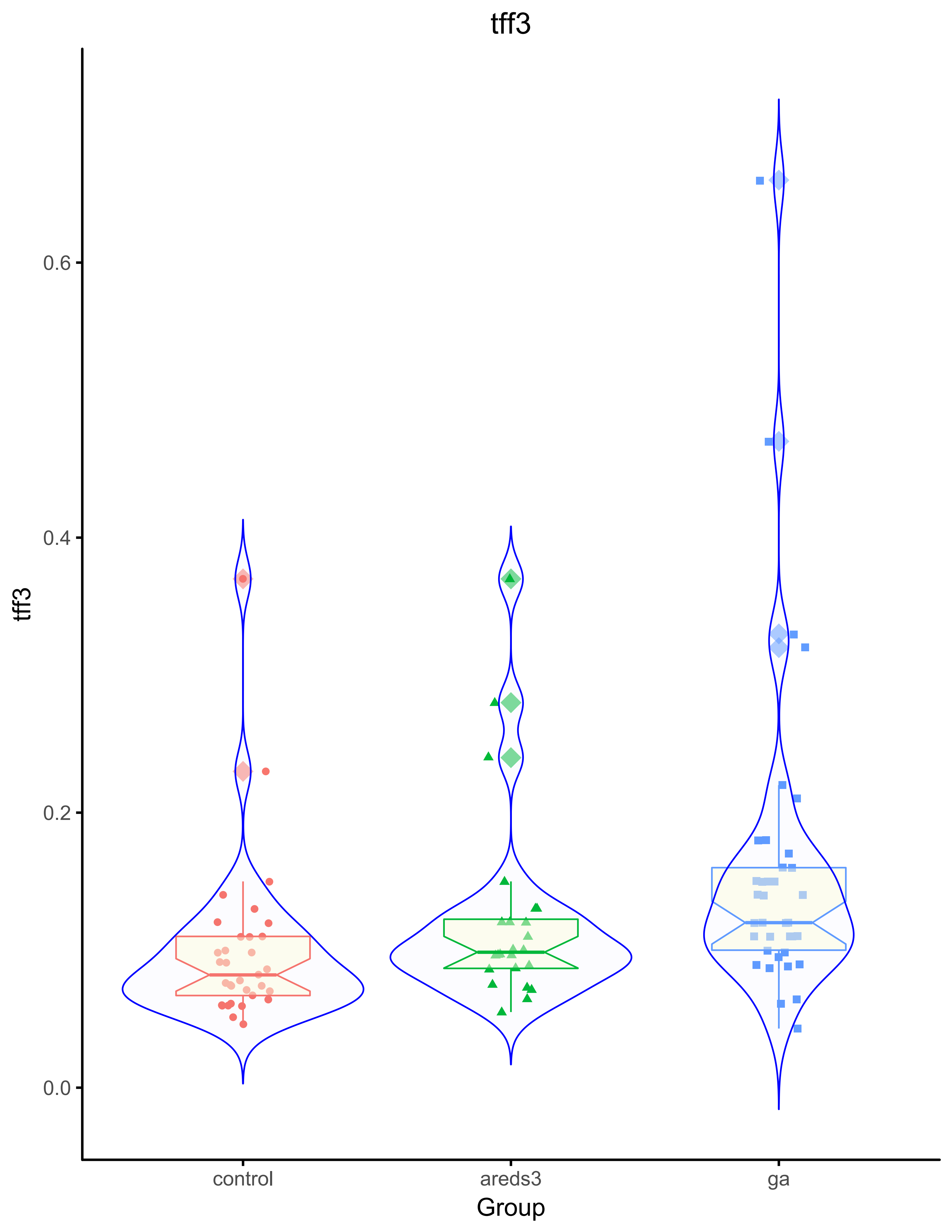
tff3
tff3
0.6
0.4
0.2
0.0
control
areds3
ga
Group

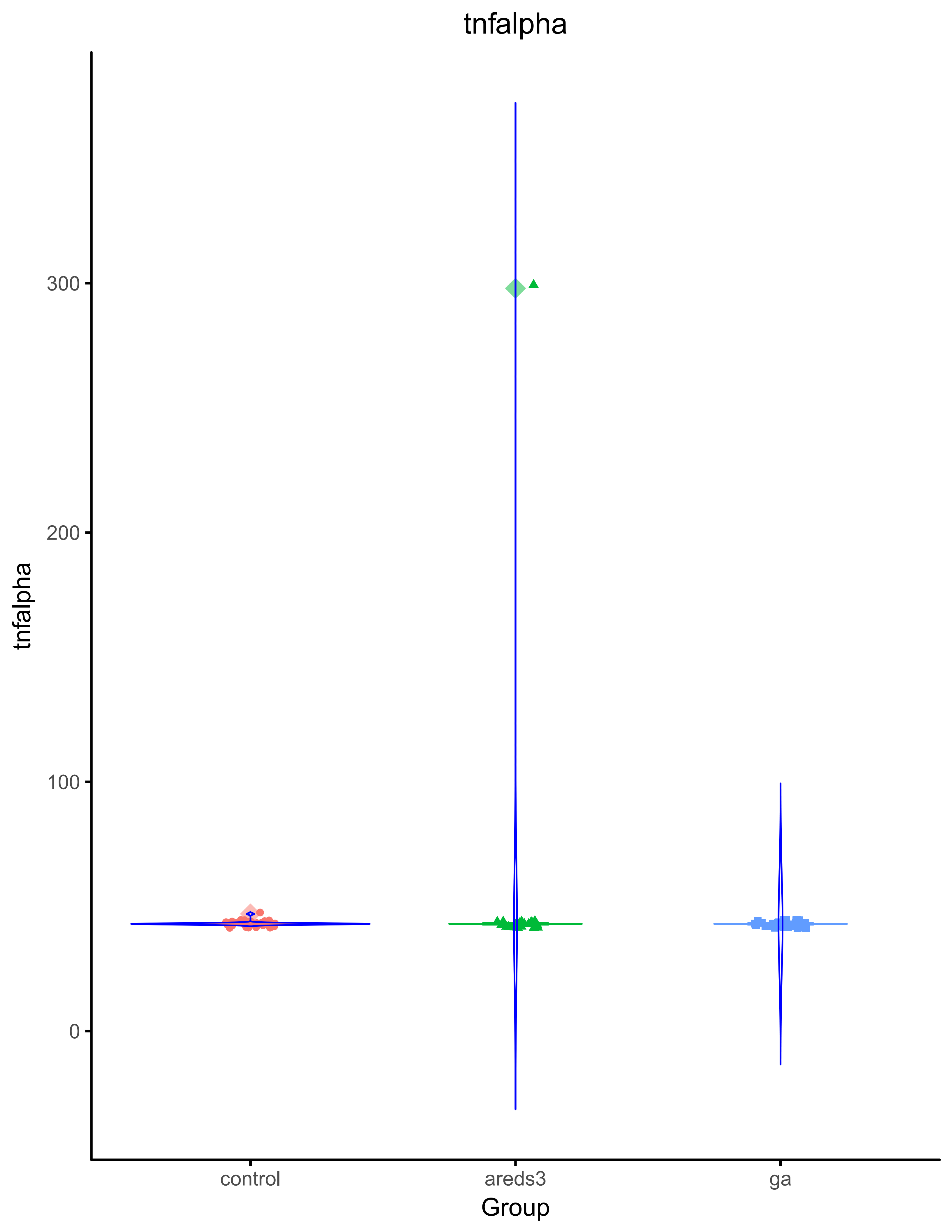

tnfalpha
300
200
100
0
tnfalpha
control
areds3
ga
Group

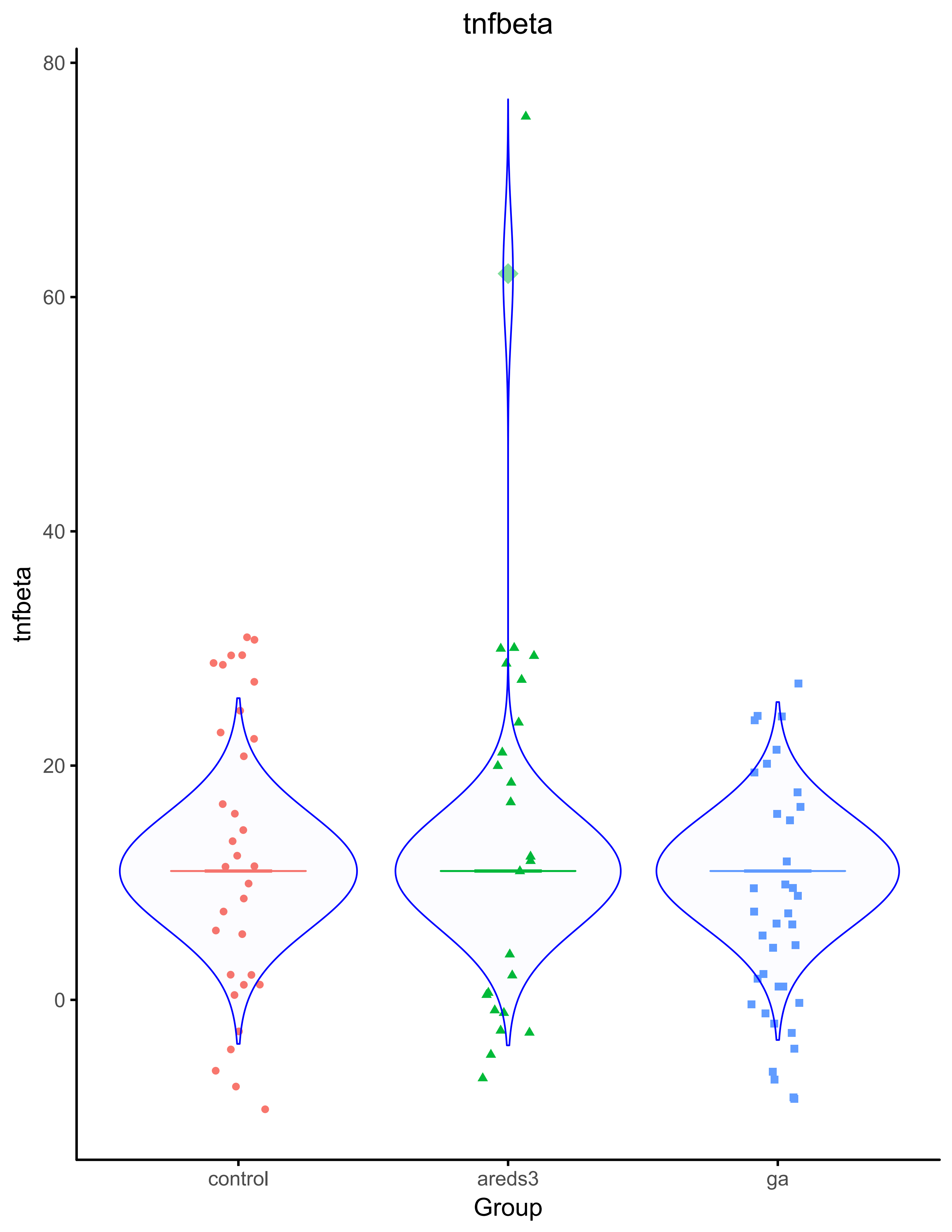

tnfbeta
80
60
40
20
0
tnfbeta
control
areds3
ga
Group

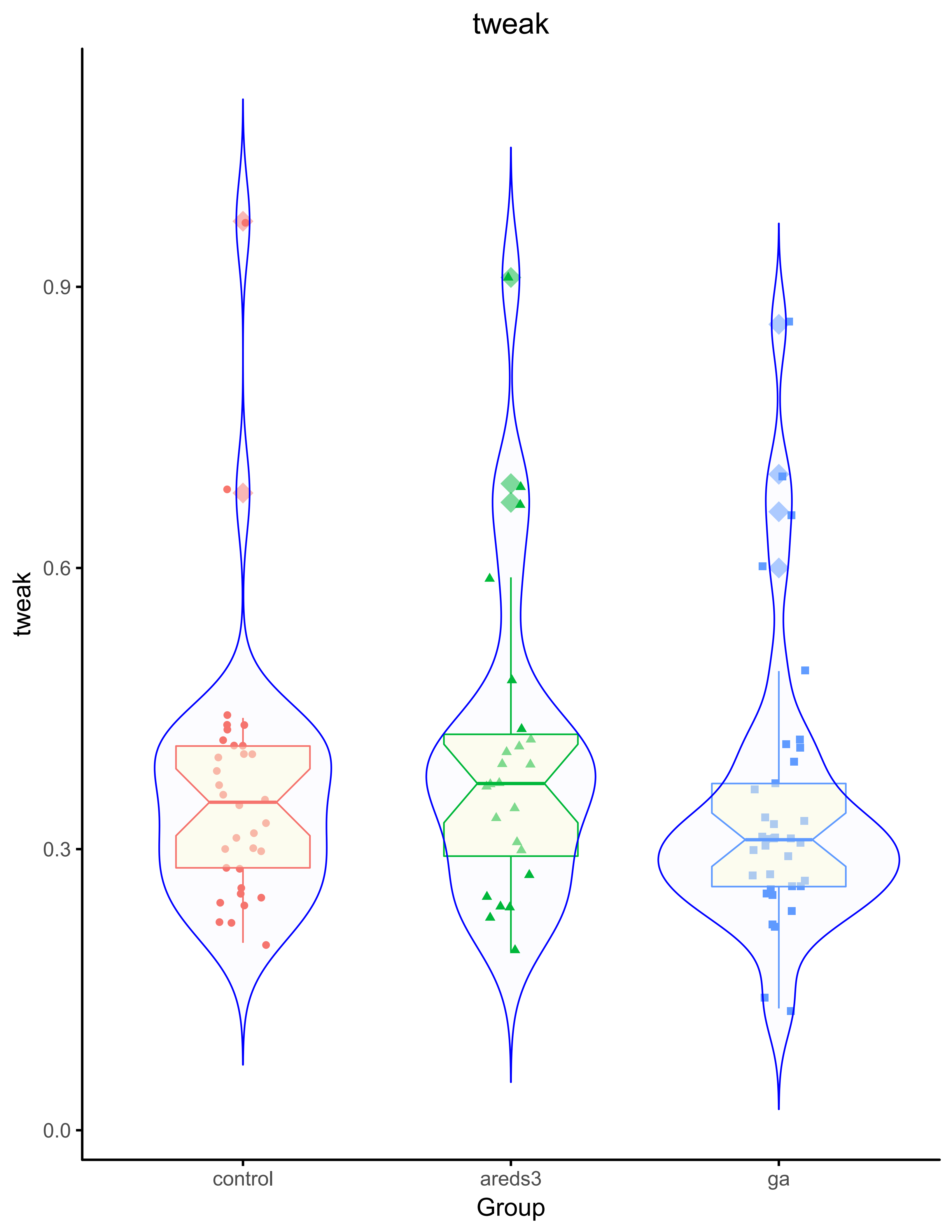

tweak
tweak
0.0
0.3
0.6
0.9
control
areds3
ga
Group

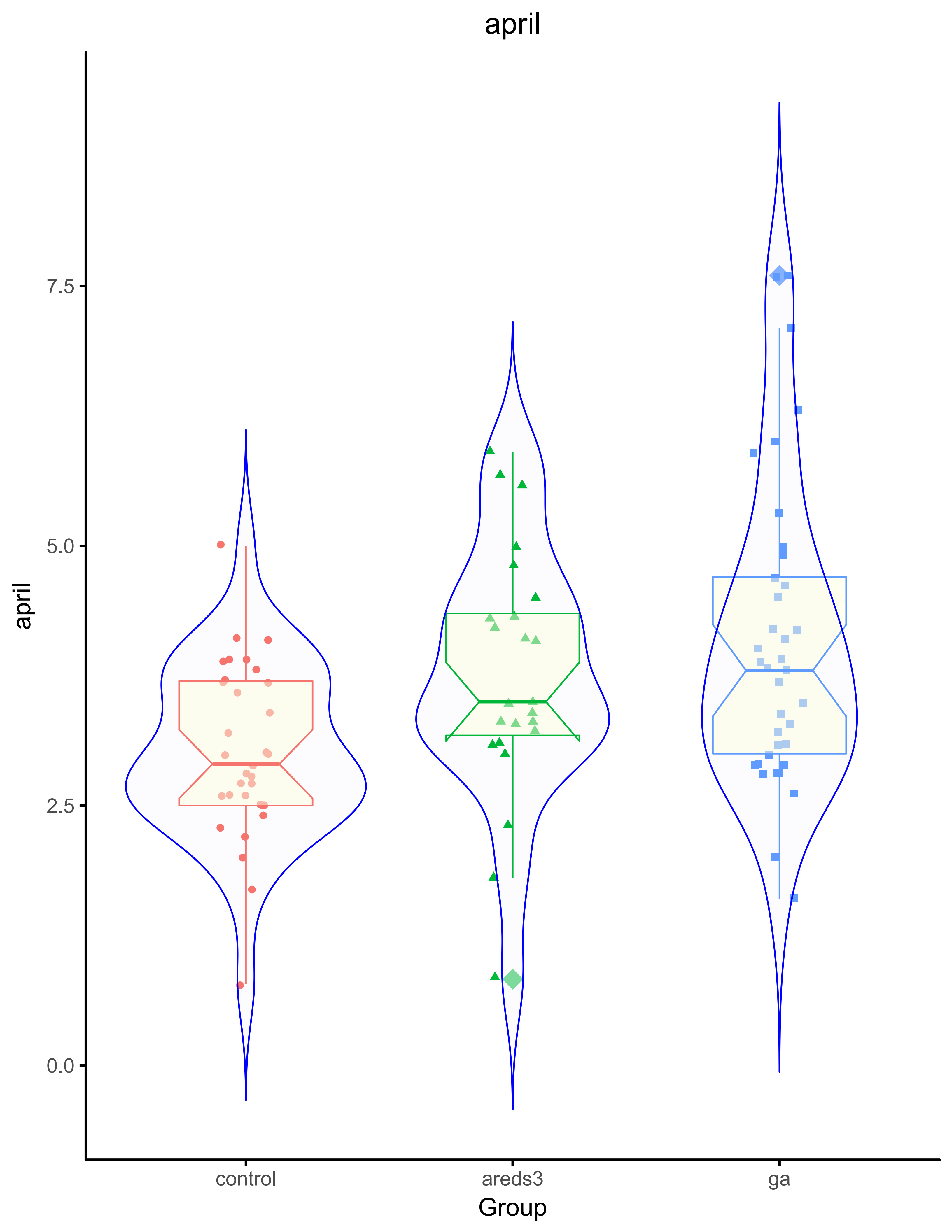

april
april
7.5
5.0
2.5
0.0
control
areds3
ga
Group

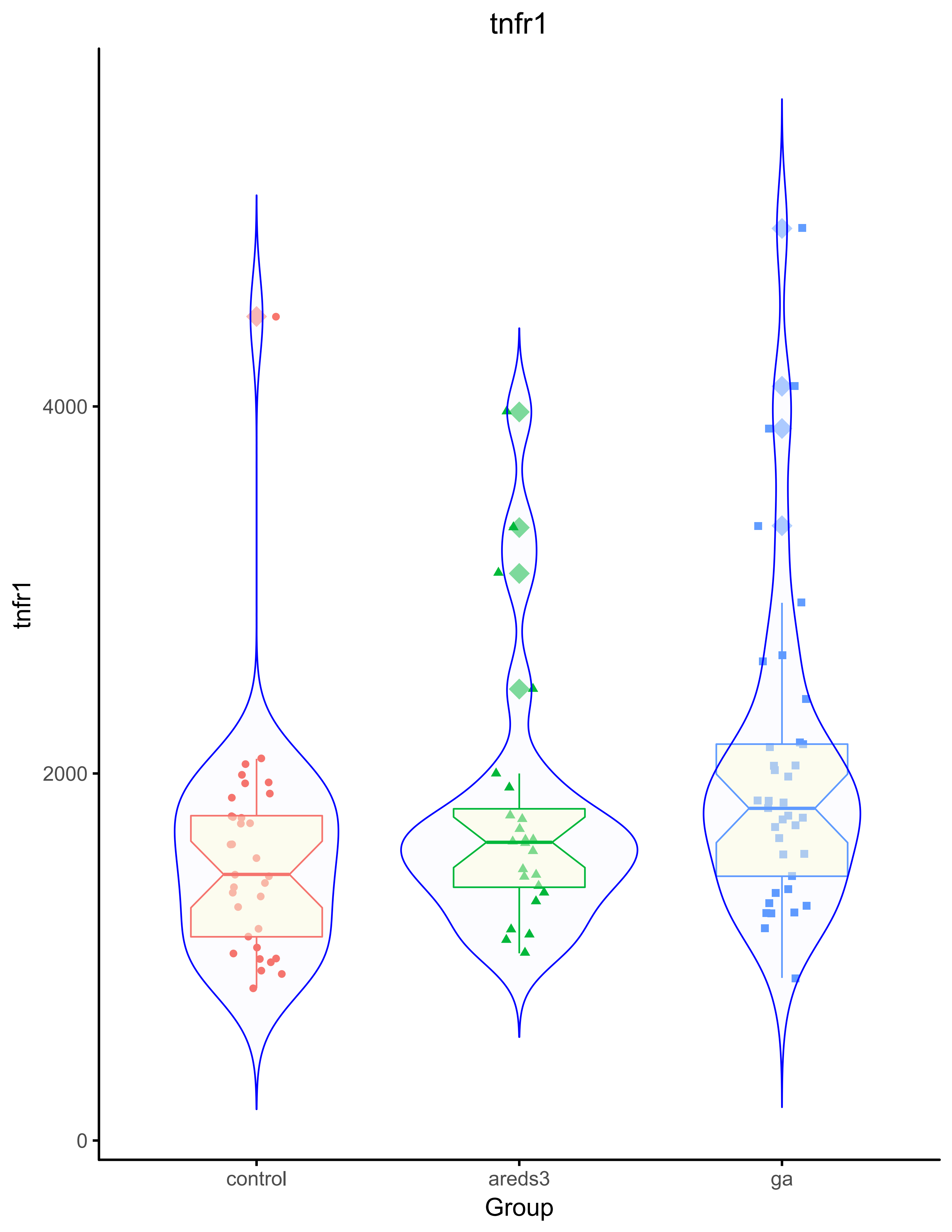

tnfr1
tnfr1
4000
2000
0
control
areds3
ga
Group

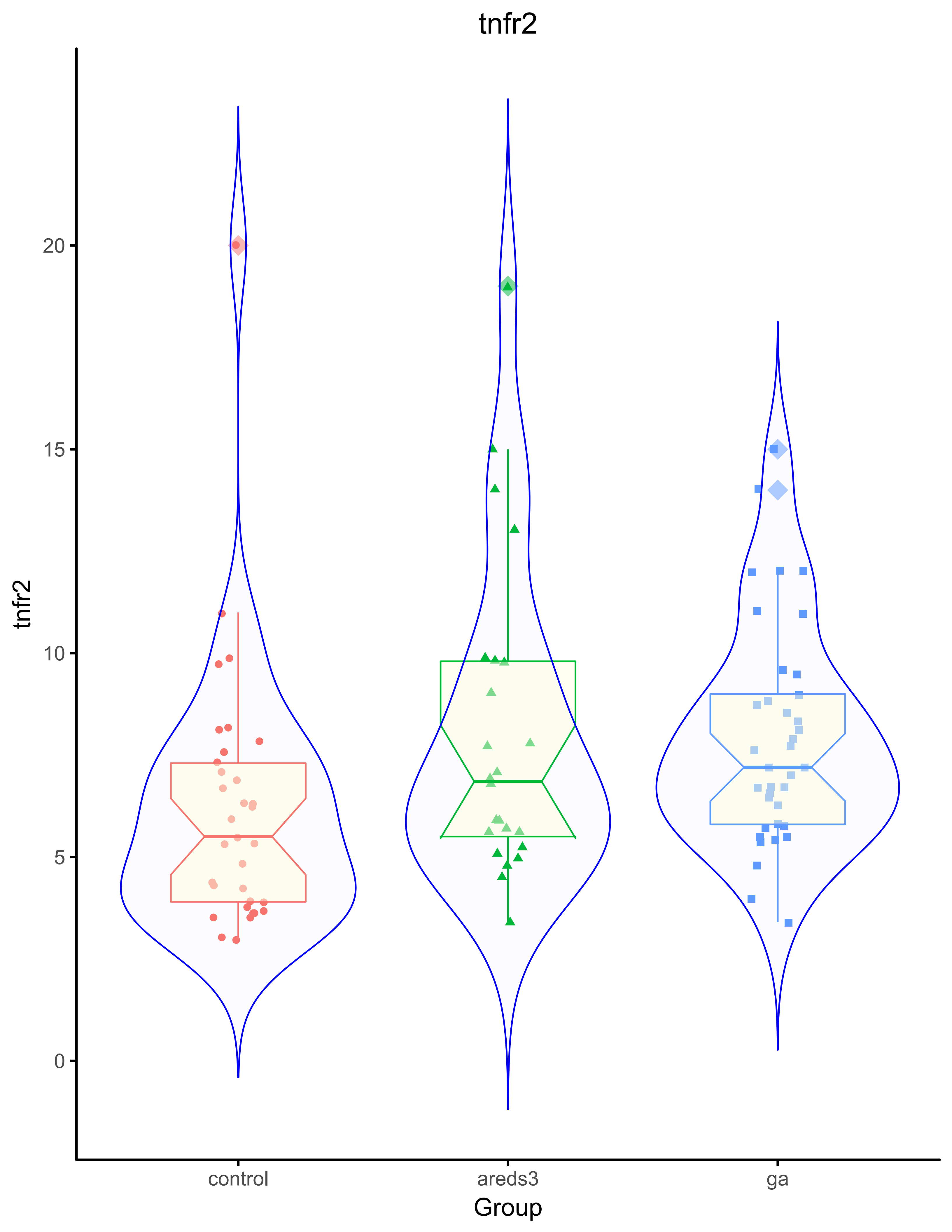
tnfr2
tnfr2
20
15
10
5
0
control
areds3
ga
Group

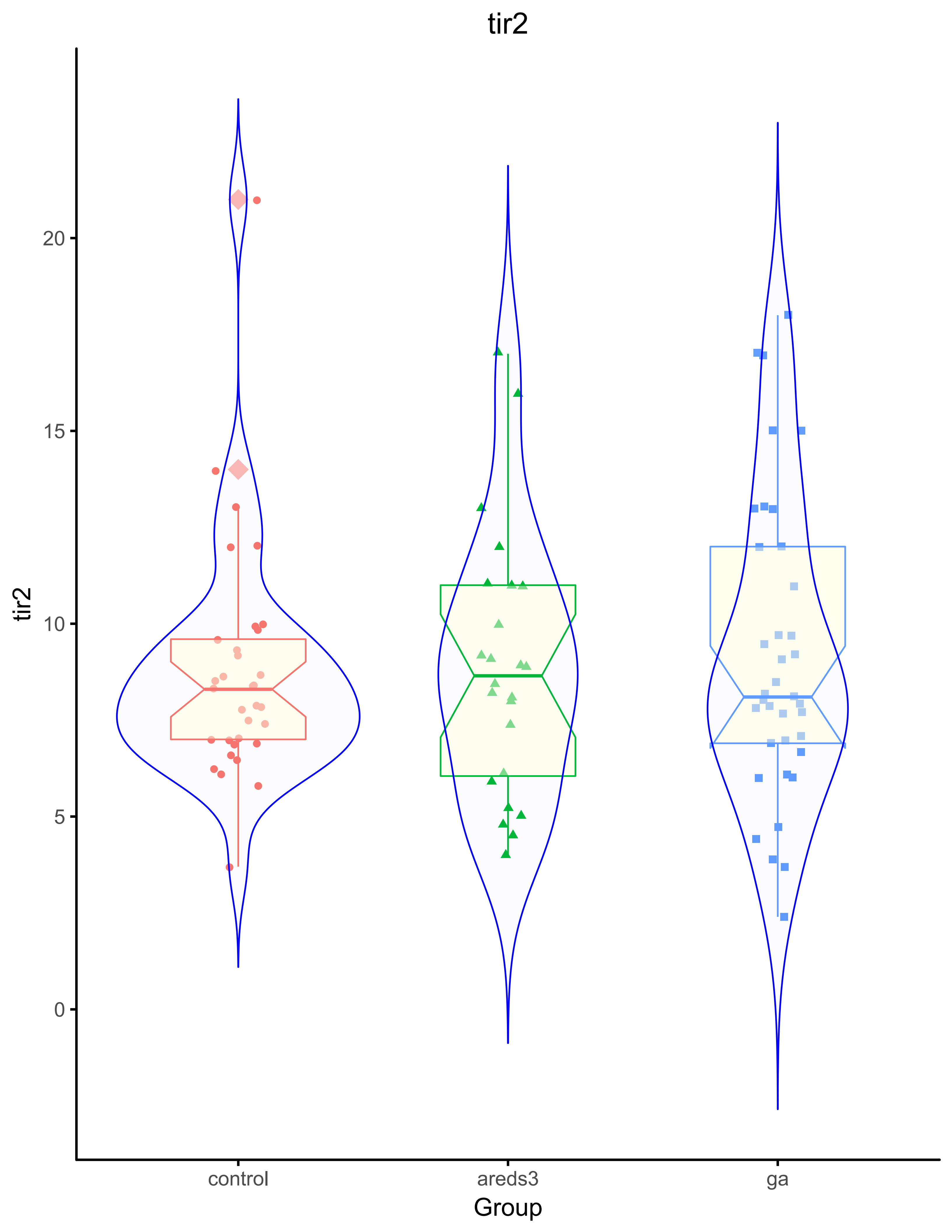
tir2
20
15
10
5
0
tir2
control
areds3
ga
Group

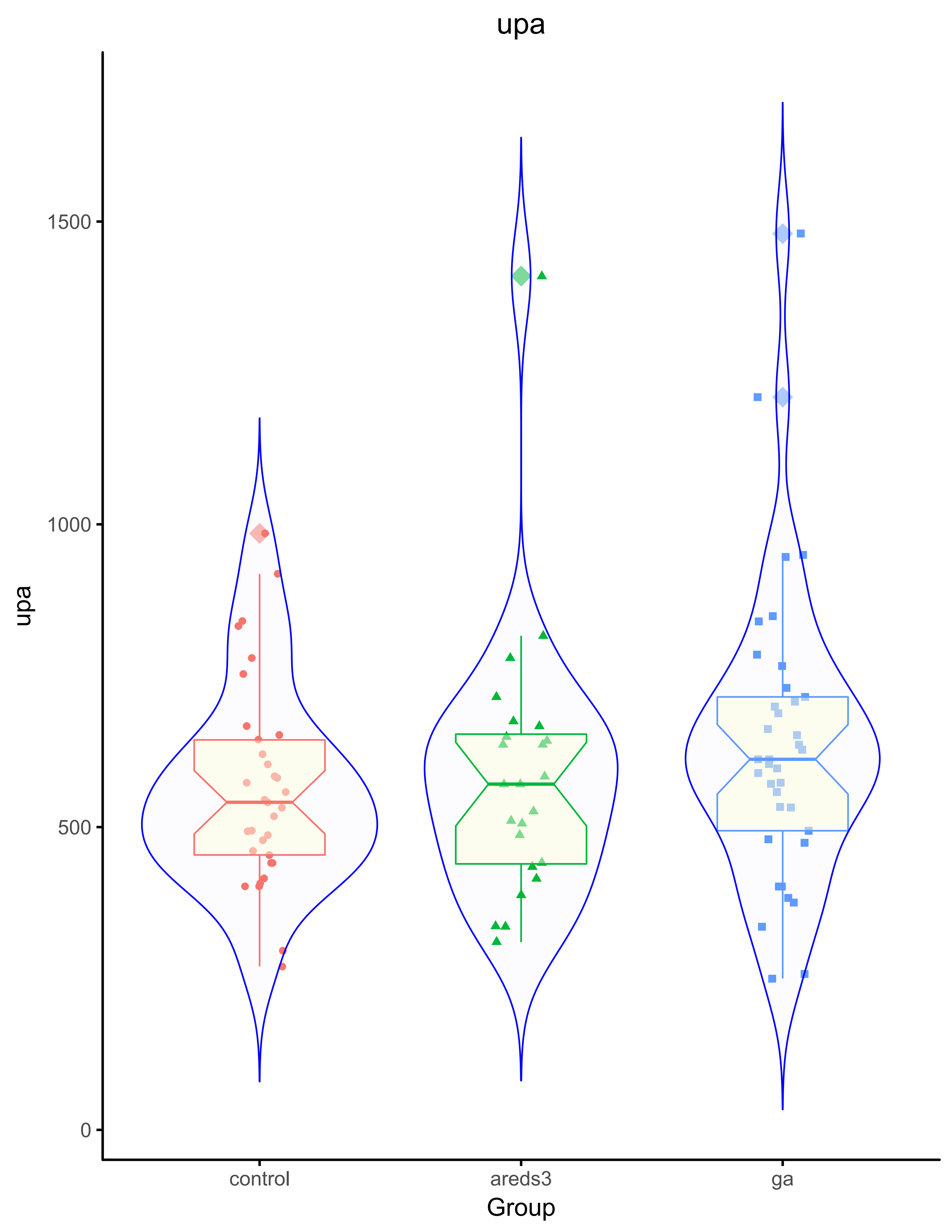
upa
upa
1500
1000
500
0
control
areds3
ga
Group

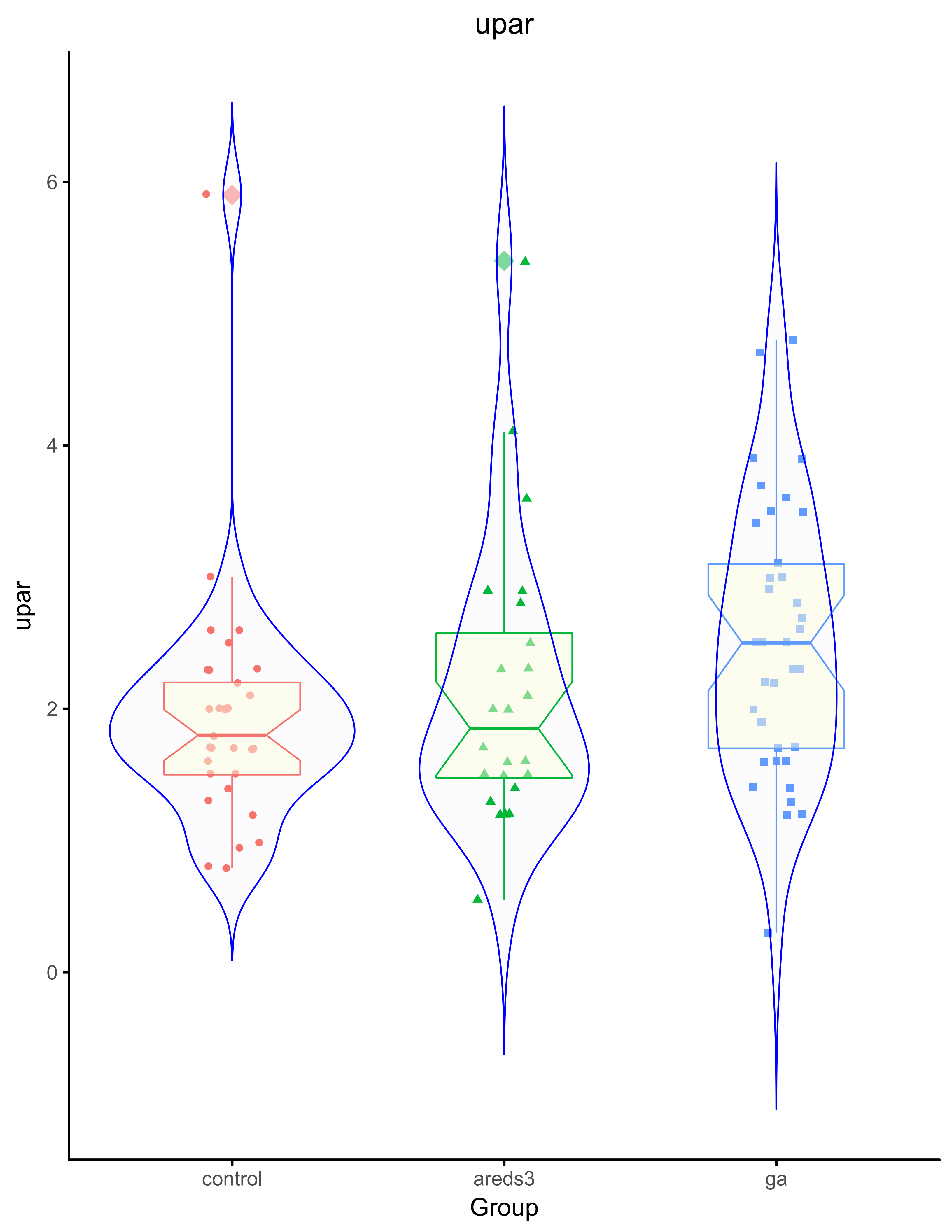

upar
6
4
2
0
upar
control
areds3
ga
Group

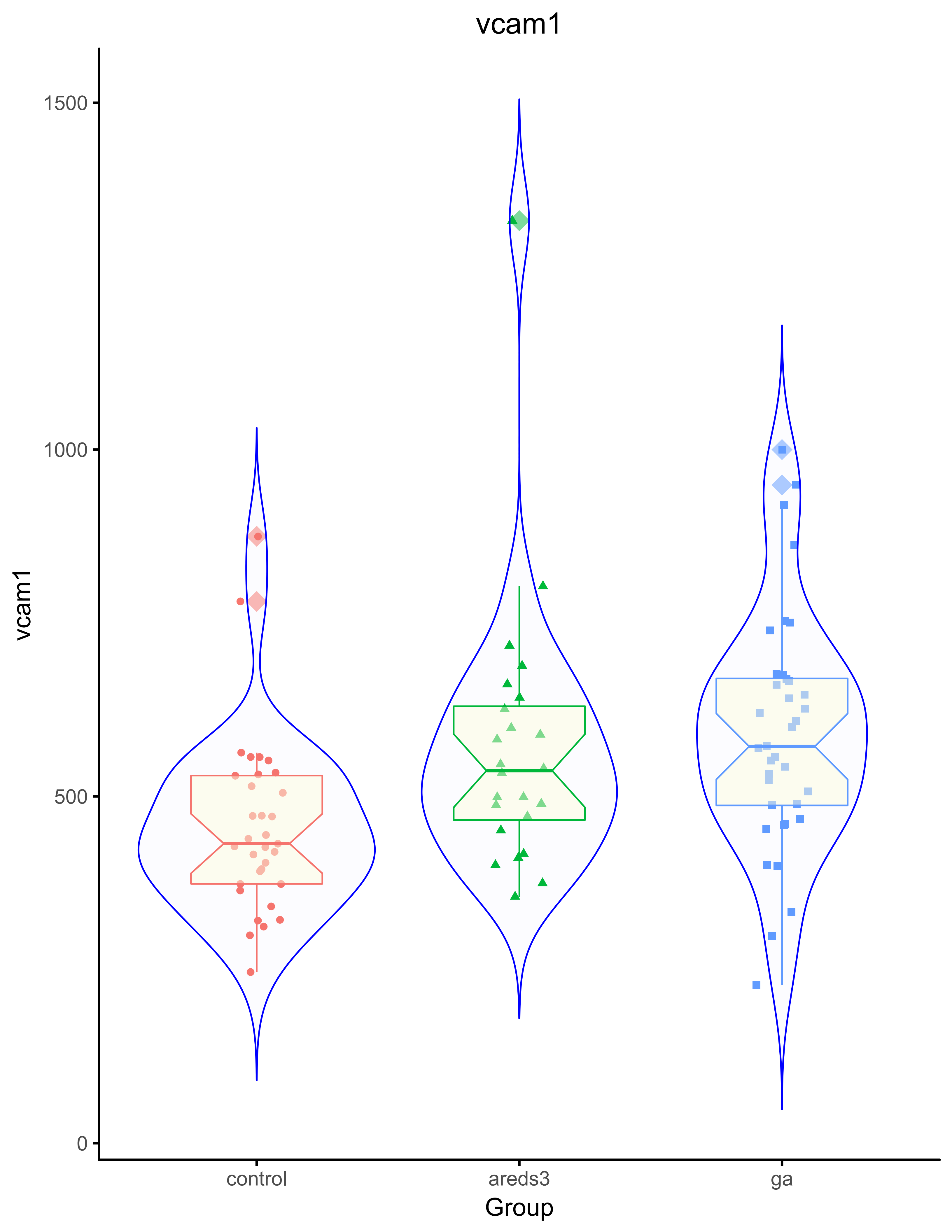

vcam1
1500
1000
500
0
vcam1
control
areds3
ga
Group

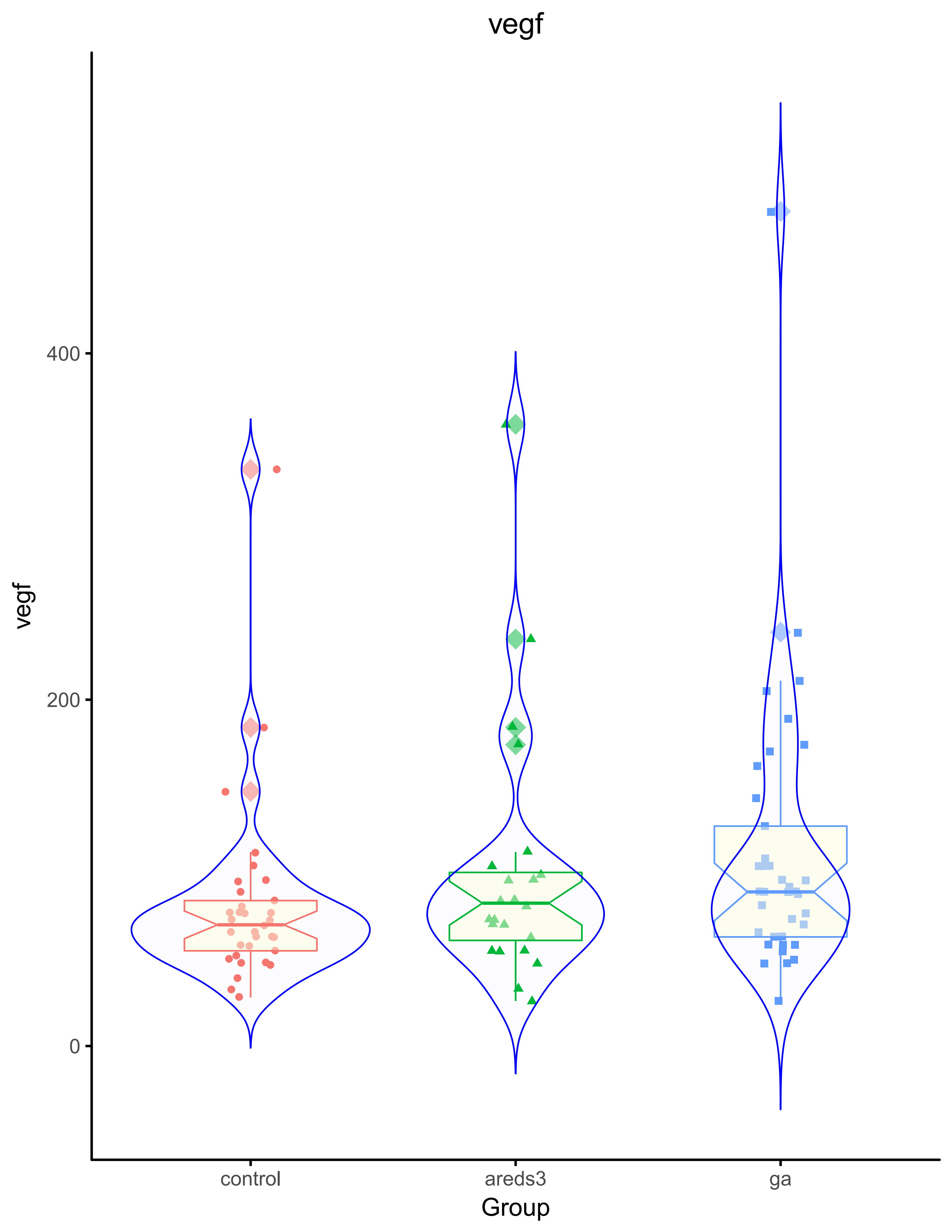

vegf
400
200
0
vegf
control
areds3
ga
Group

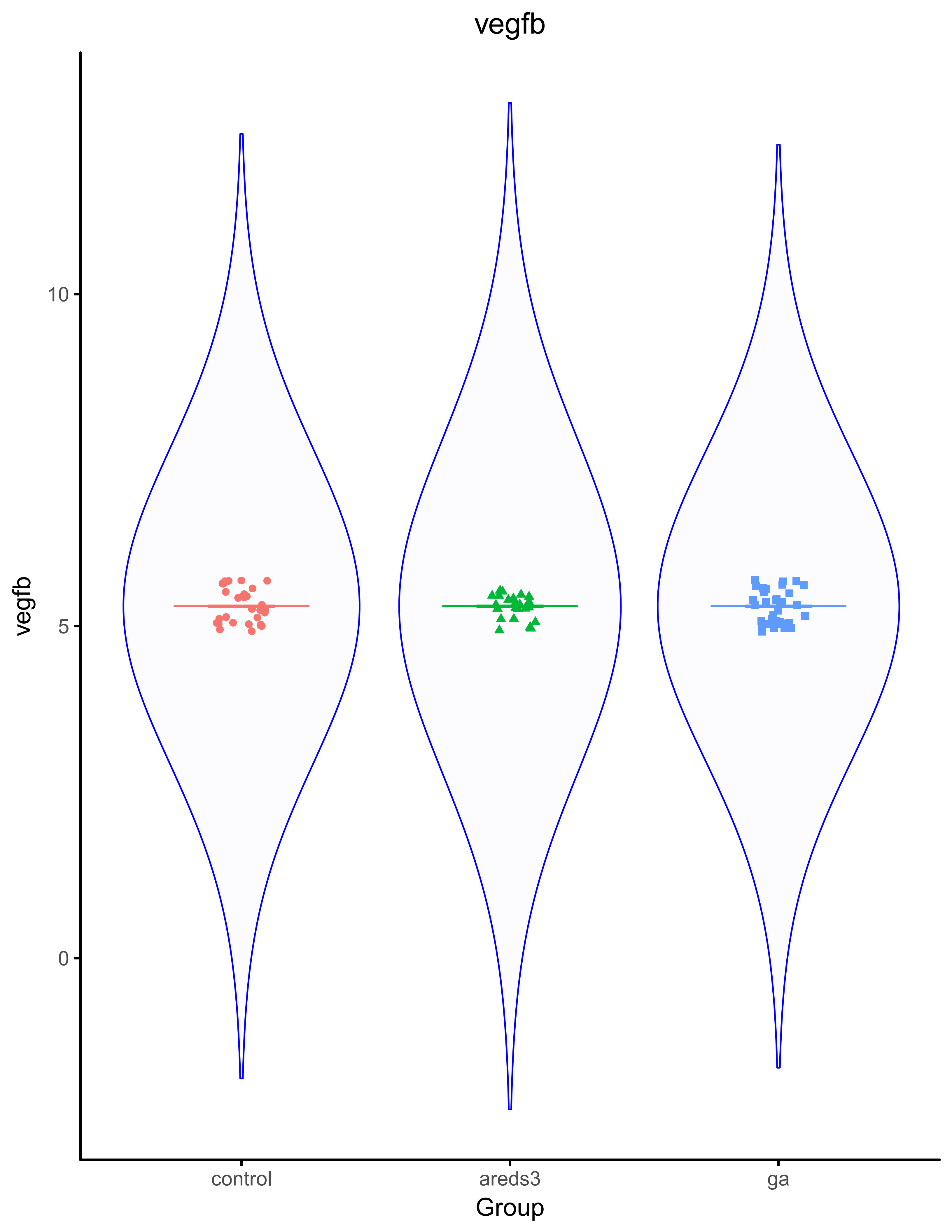
vegfb
10
5
0
vegfb
control
areds3
ga
Group

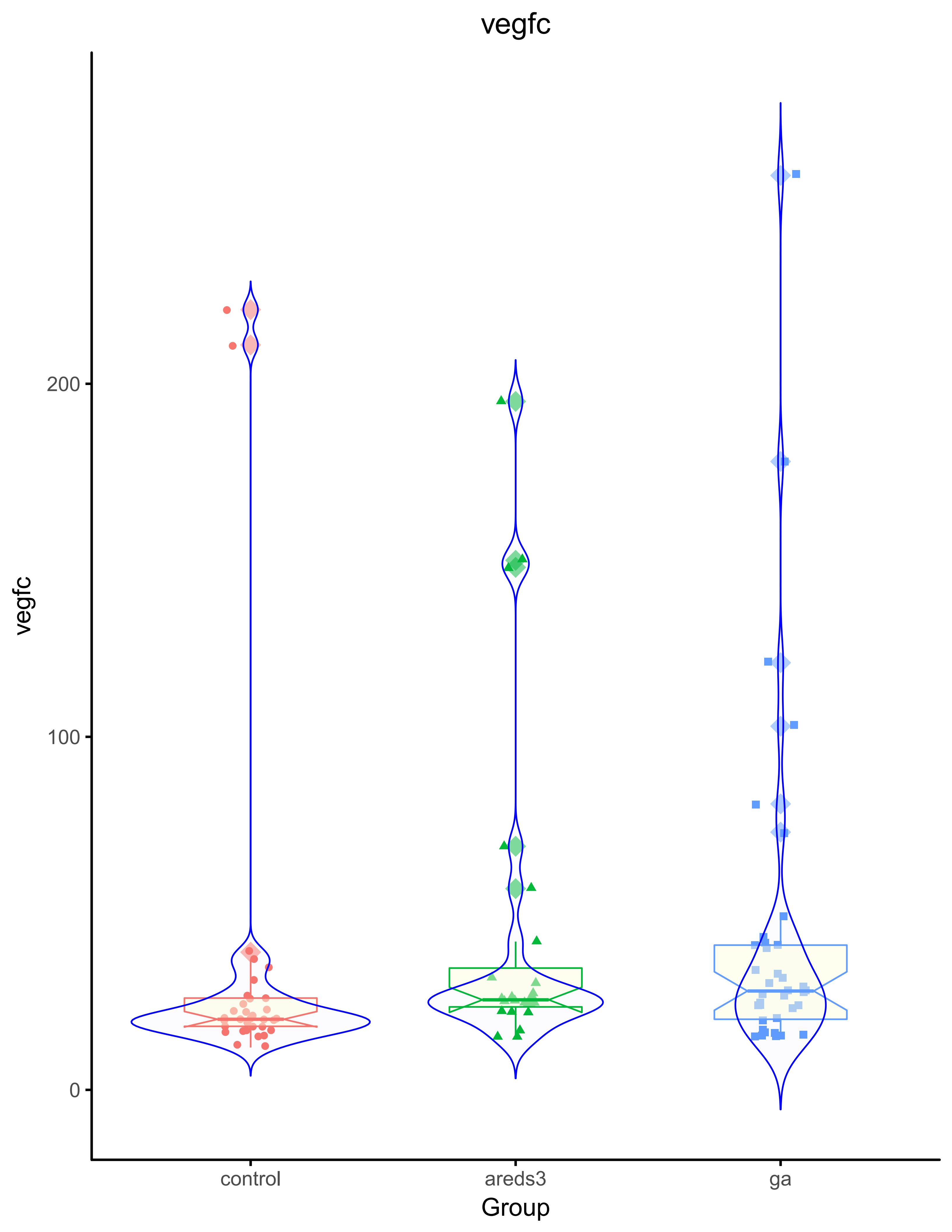

vegfc
vegfc
200
100
0
control
areds3
ga
Group

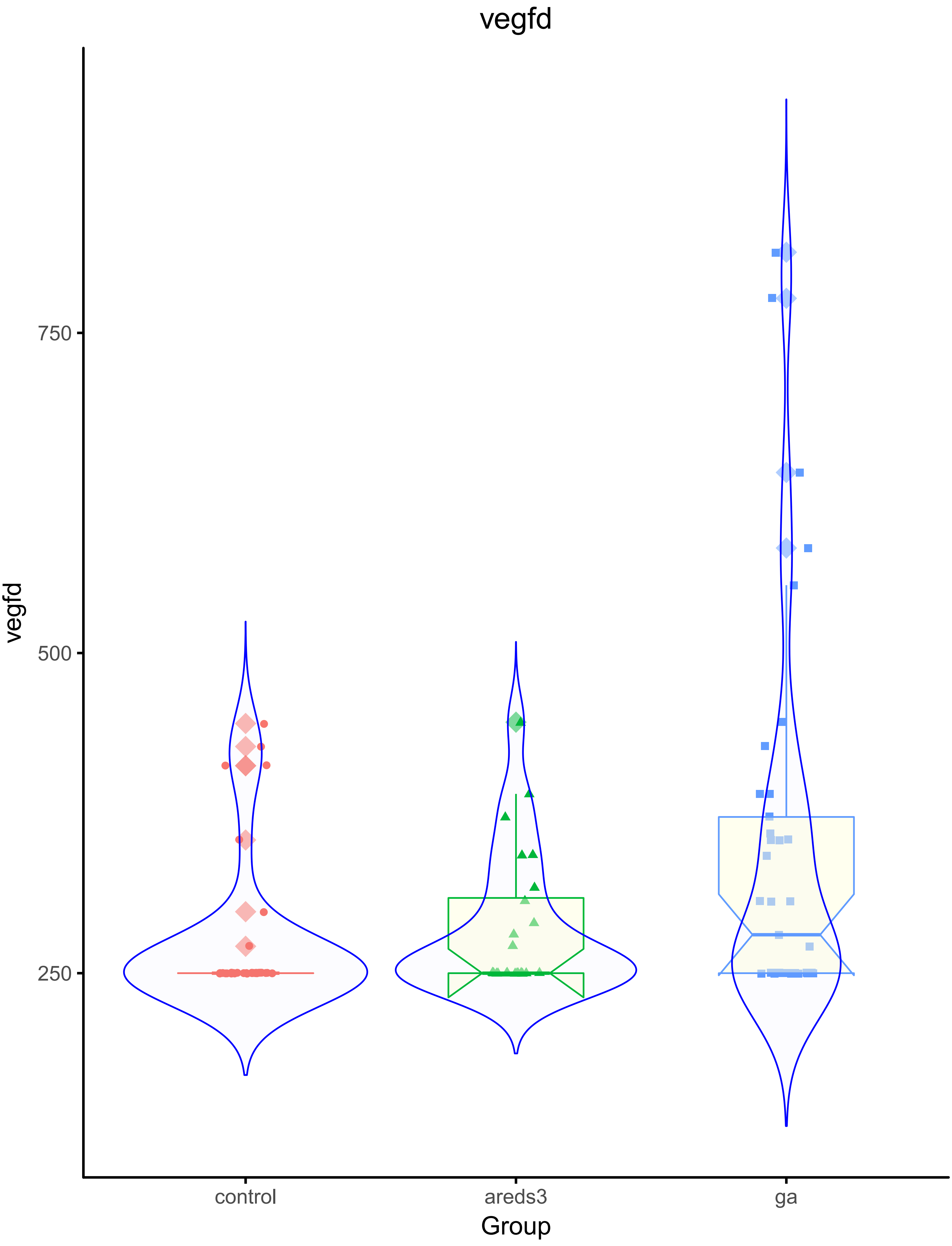

vegfd
750
500
250
vegfd
control
areds3
ga
Group

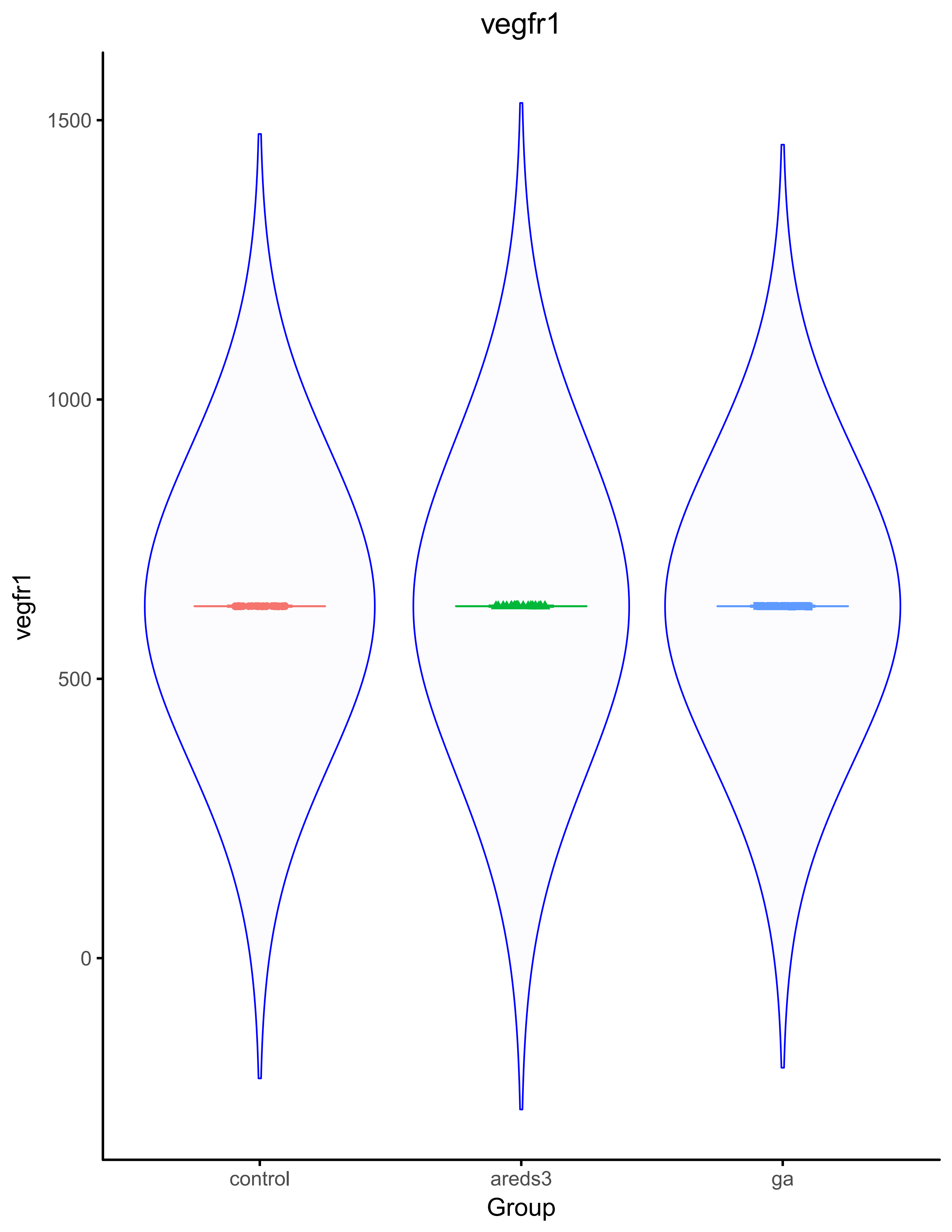
vegfr1
1500
1000
500
0
vegfr1
control
areds3
ga
Group

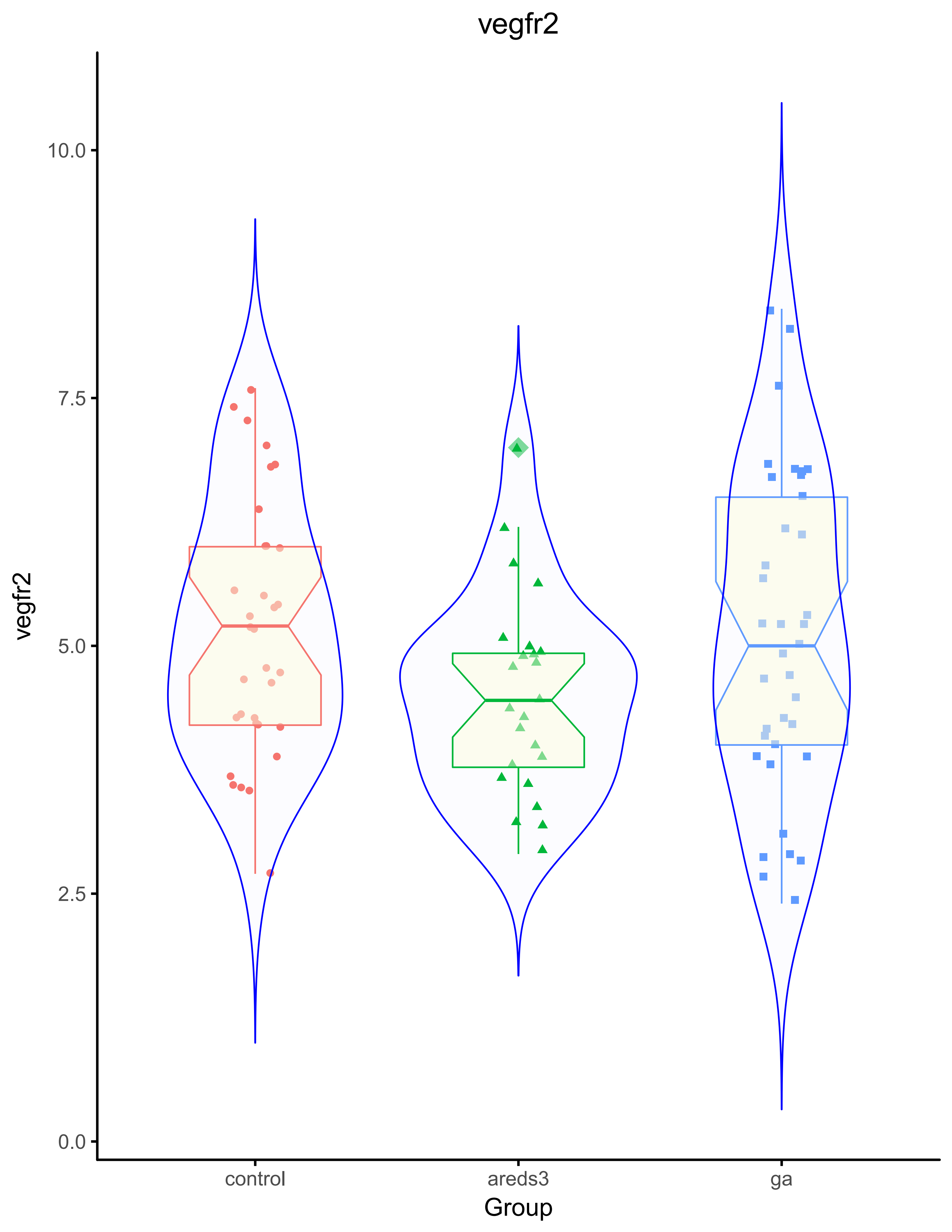
vegfr2
vegfr2
10.0
7.5
5.0
2.5
0.0
control
areds3
ga
Group

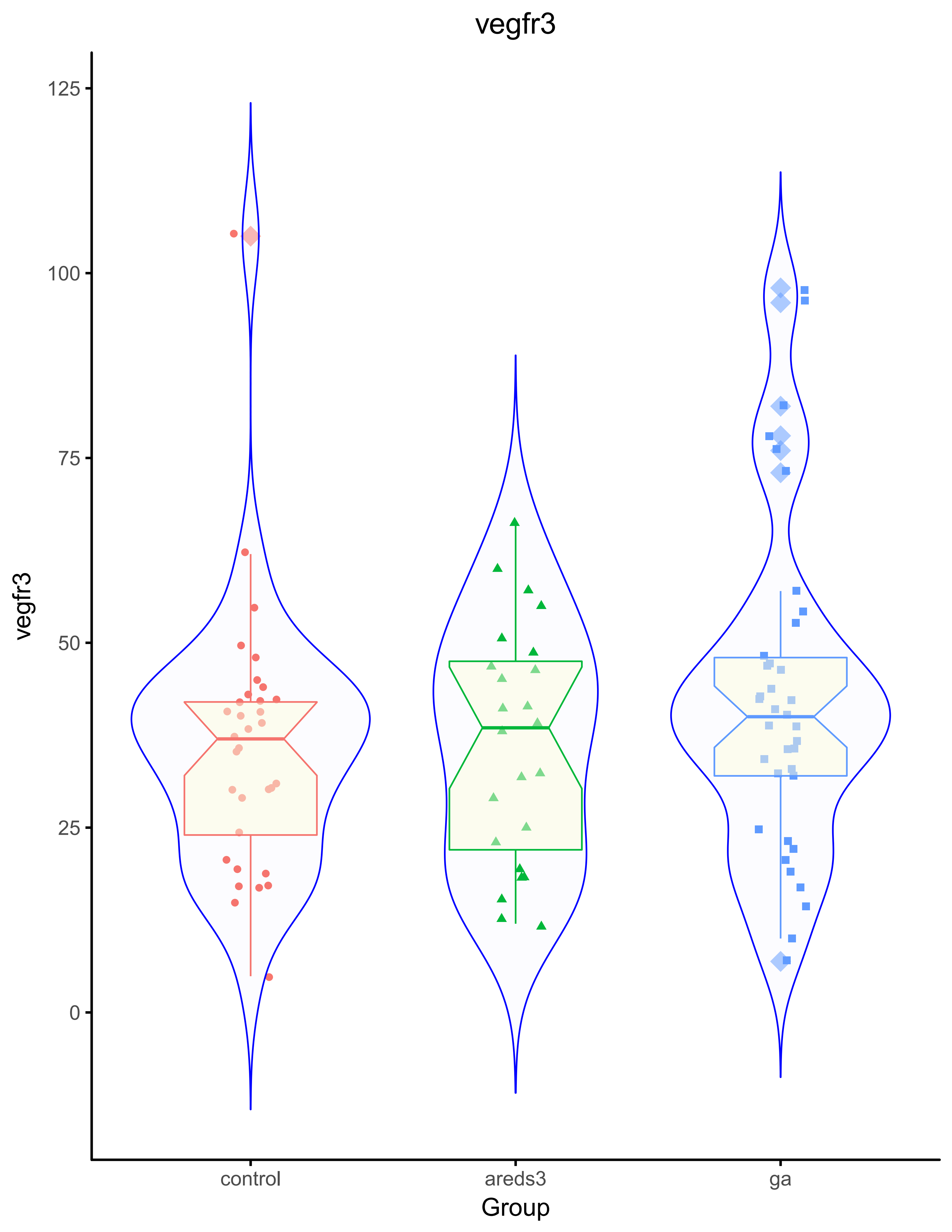
vegfr3
125
100
75
50
25
0
vegfr3
control
areds3
ga
Group

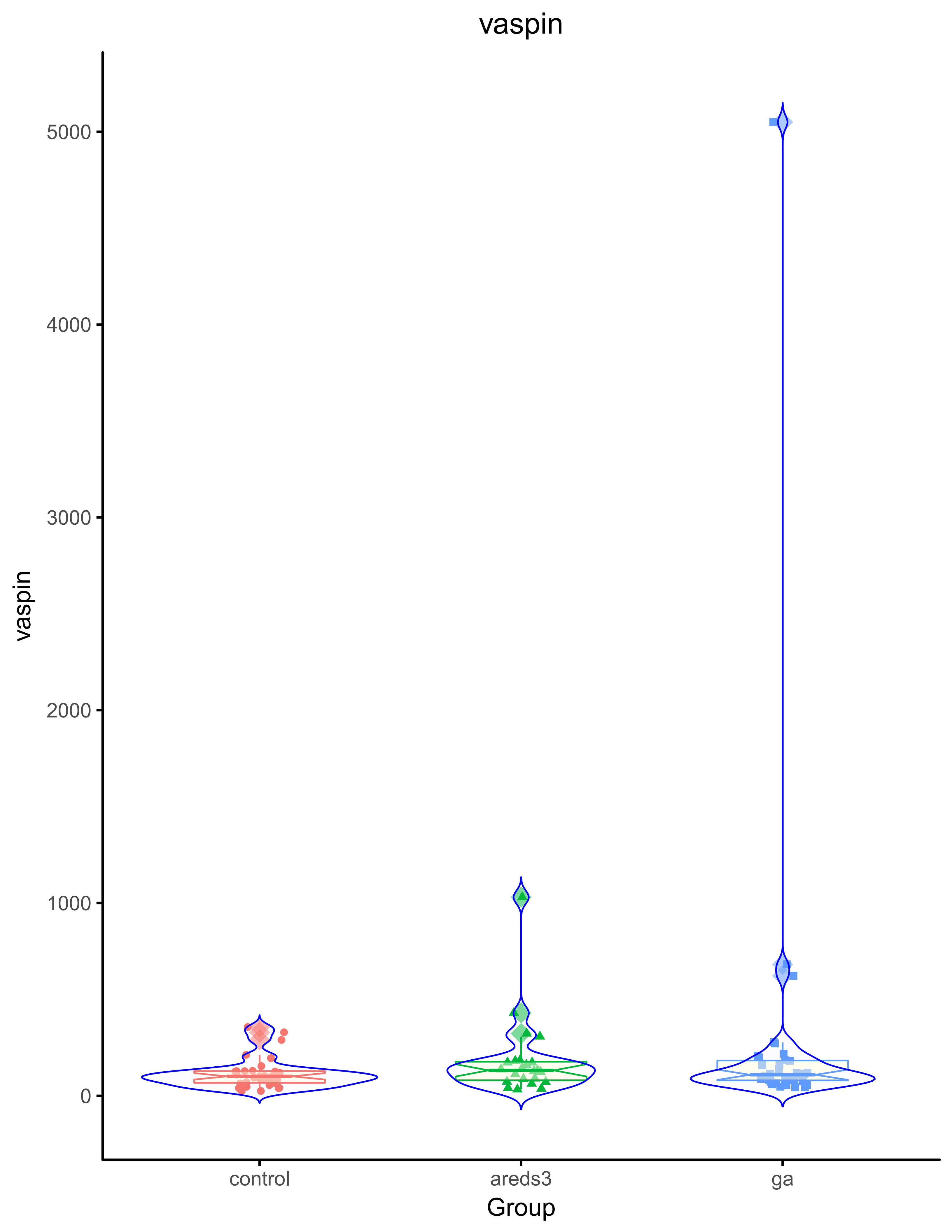

vaspin
5000
4000
3000
2000
1000
0
vaspin
control
areds3
ga
Group

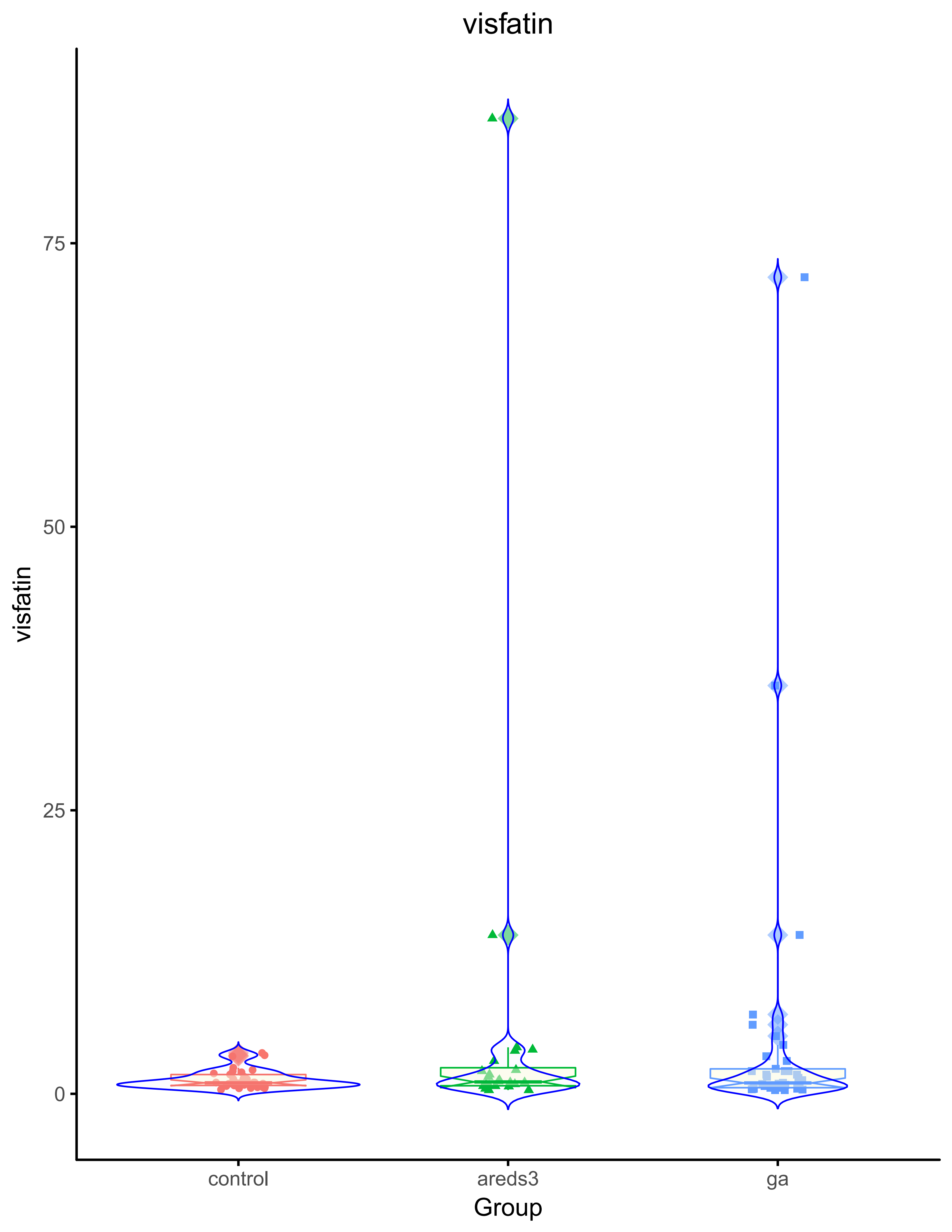
visfatin
75
50
25
0
visfatin
control
areds3
ga
Group

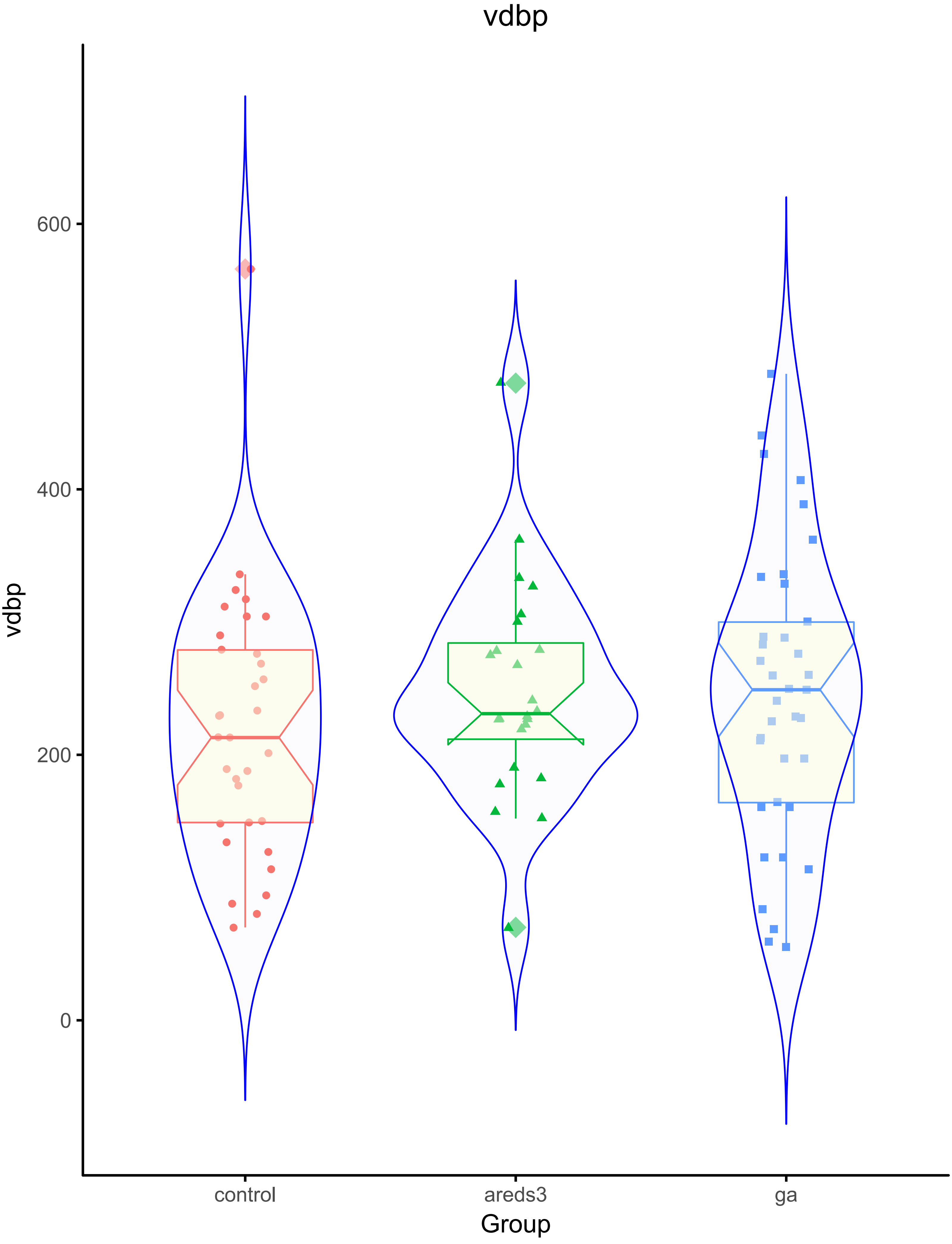
vdbp
600
400
200
0
vdbp
control
areds3
ga
Group

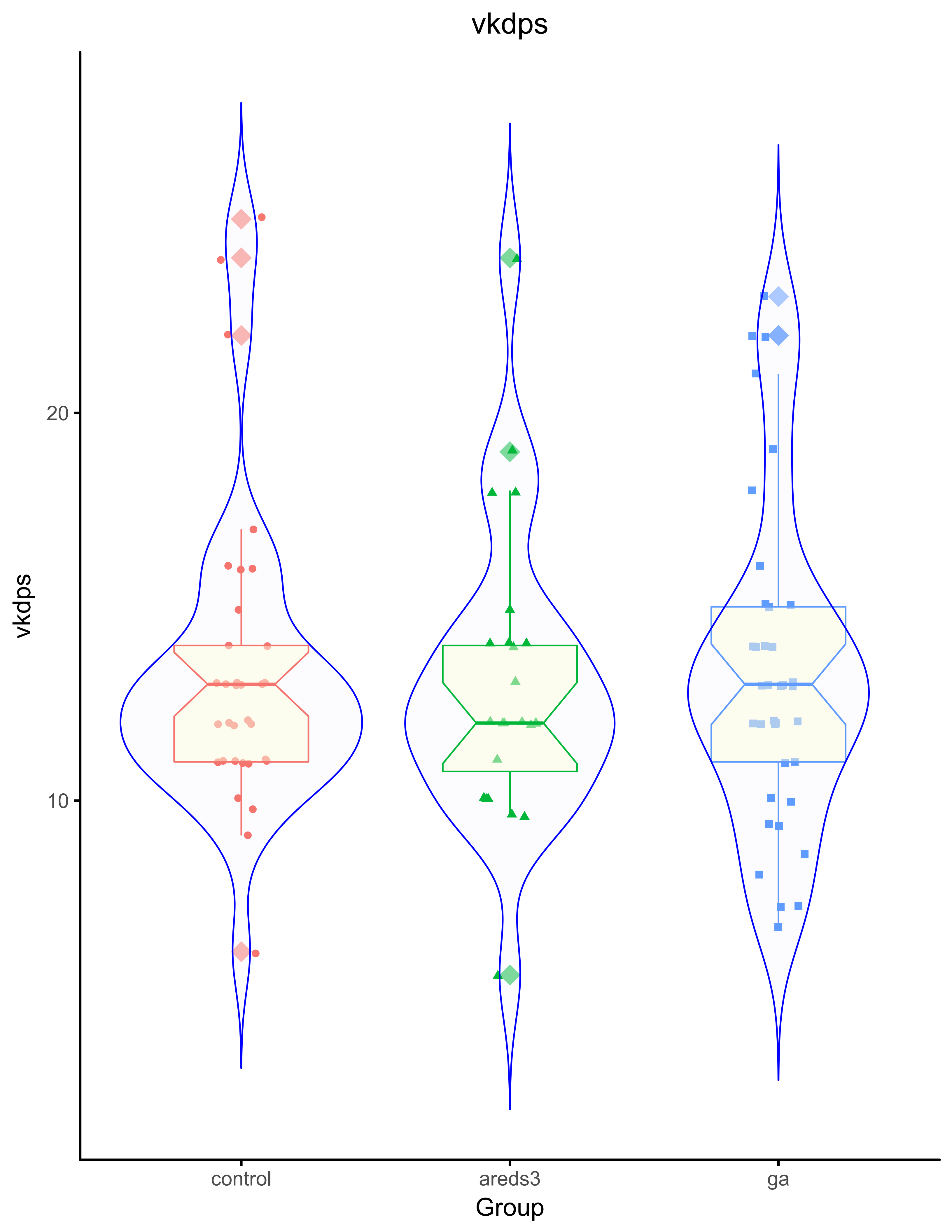

vkdps
20
10
vkdps
control
areds3
ga
Group

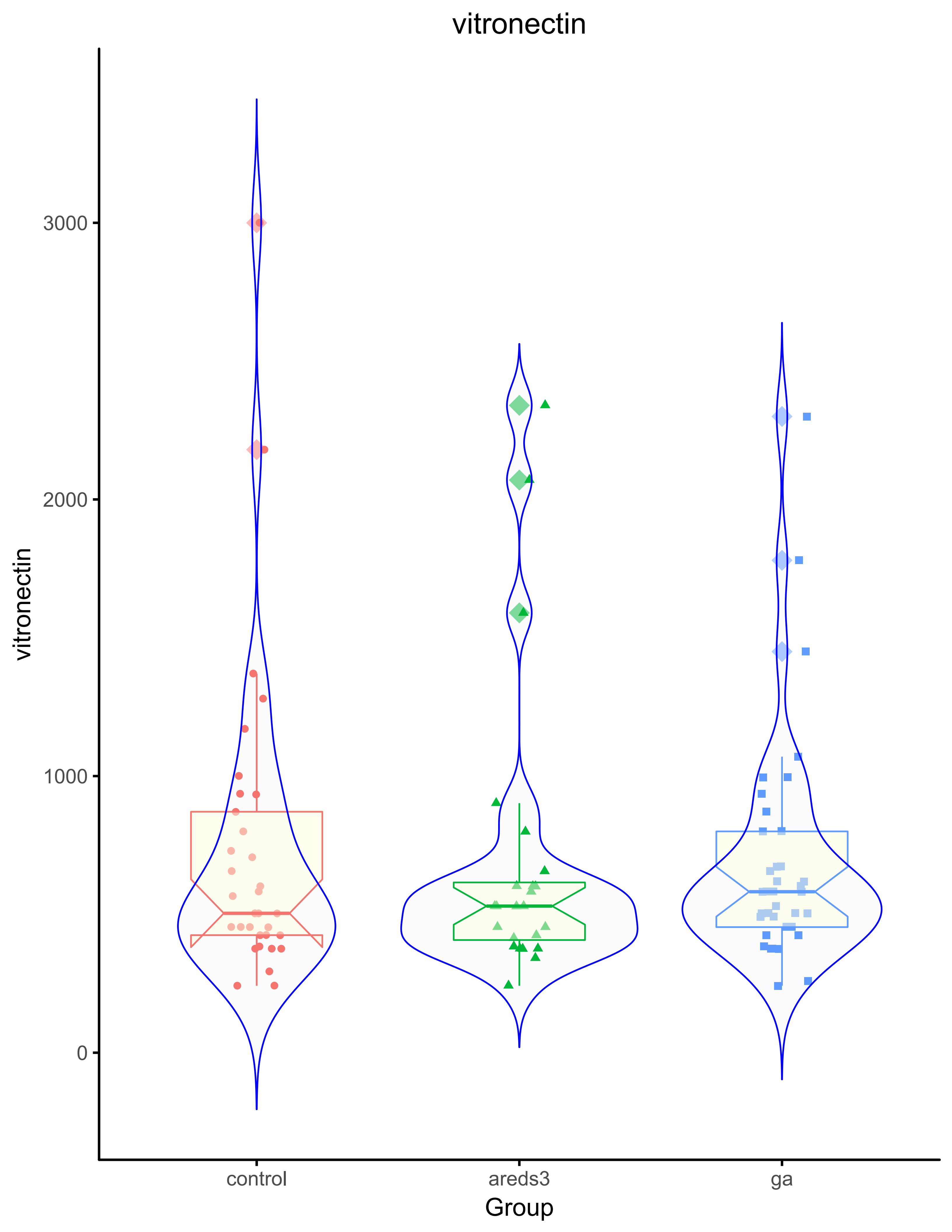
vitronectin
3000
2000
1000
0
vitronectin
control
areds3
ga
Group

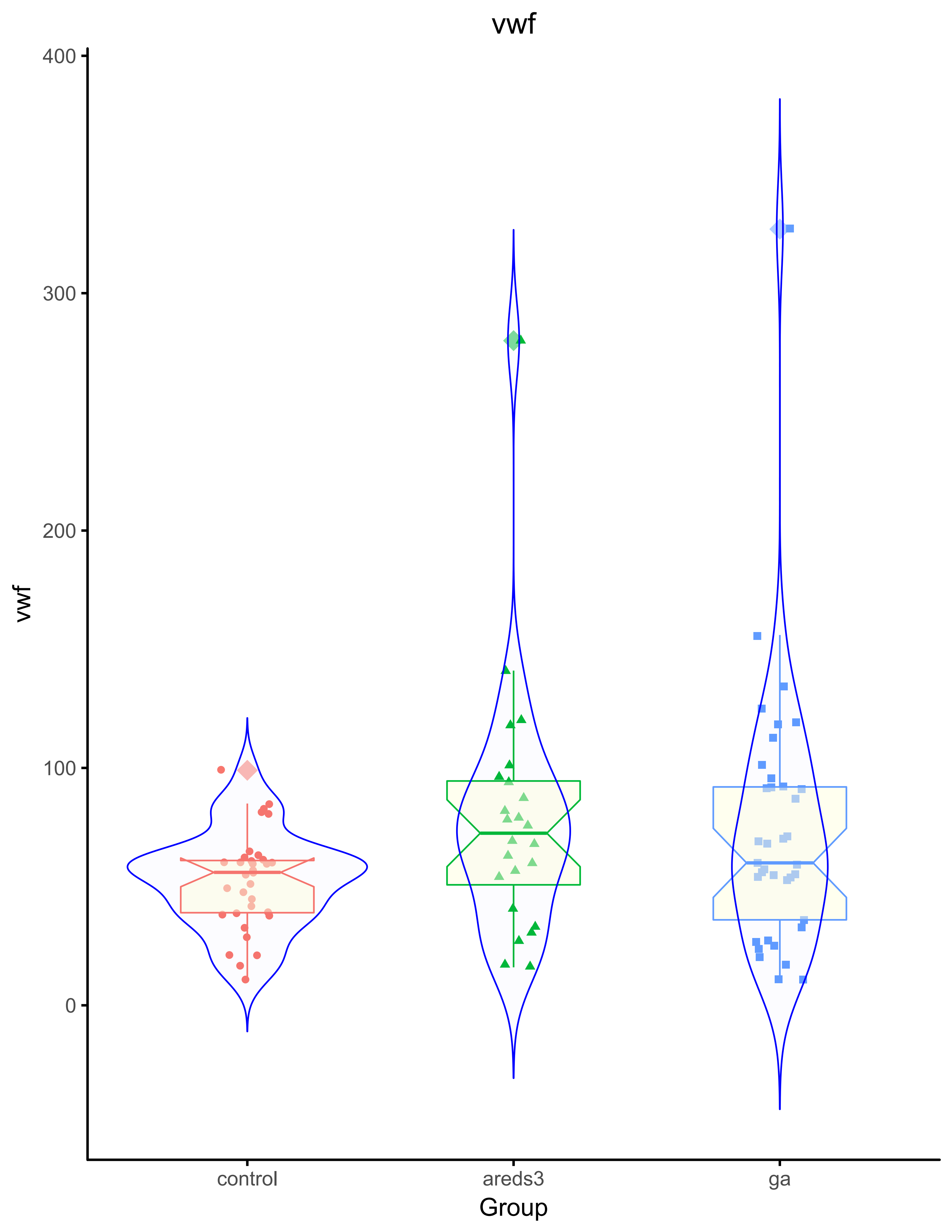
vwf
400
300
200
100
0
vwf
control
areds3
ga
Group

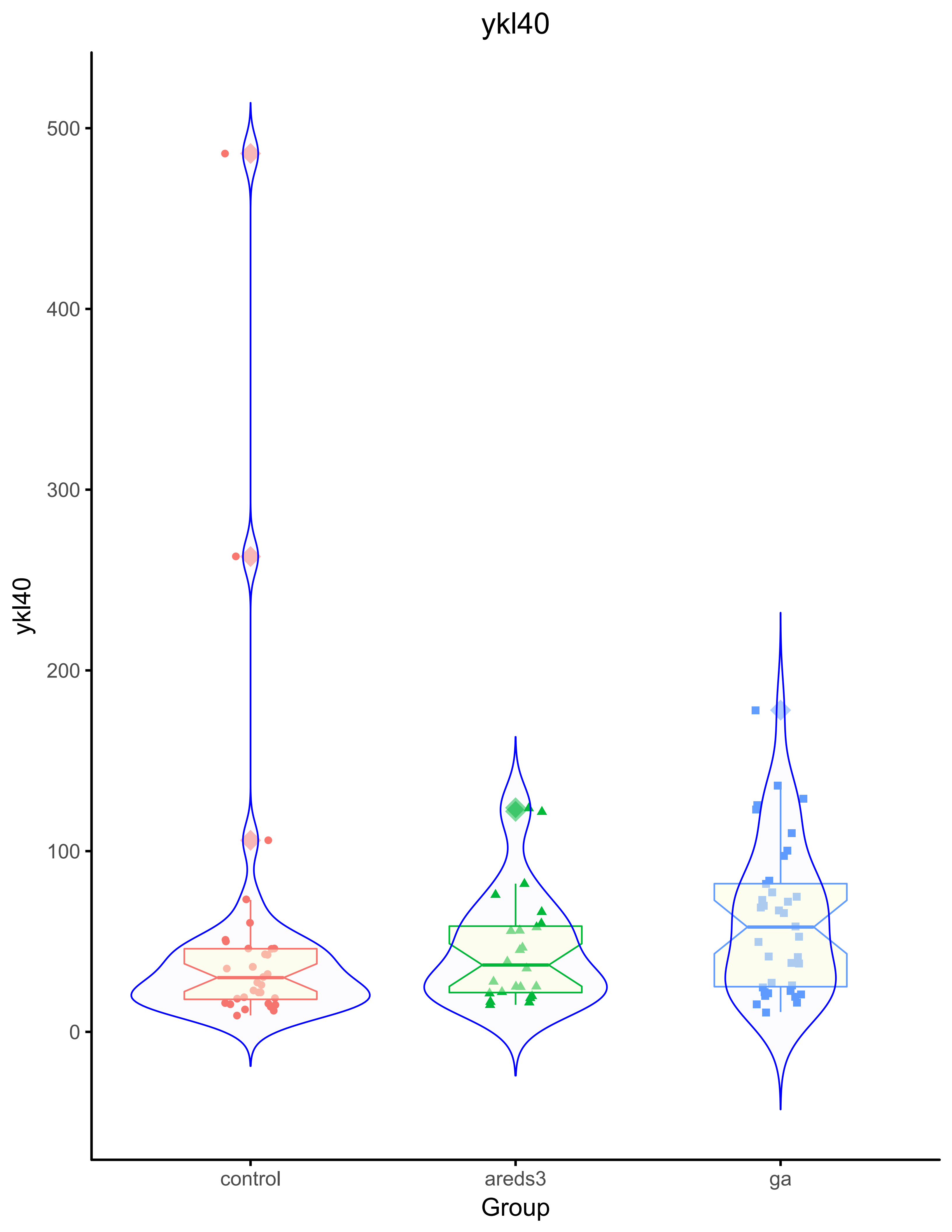
ykl40
500
400
300
200
100
0
ykl40
control
areds3
ga
Group

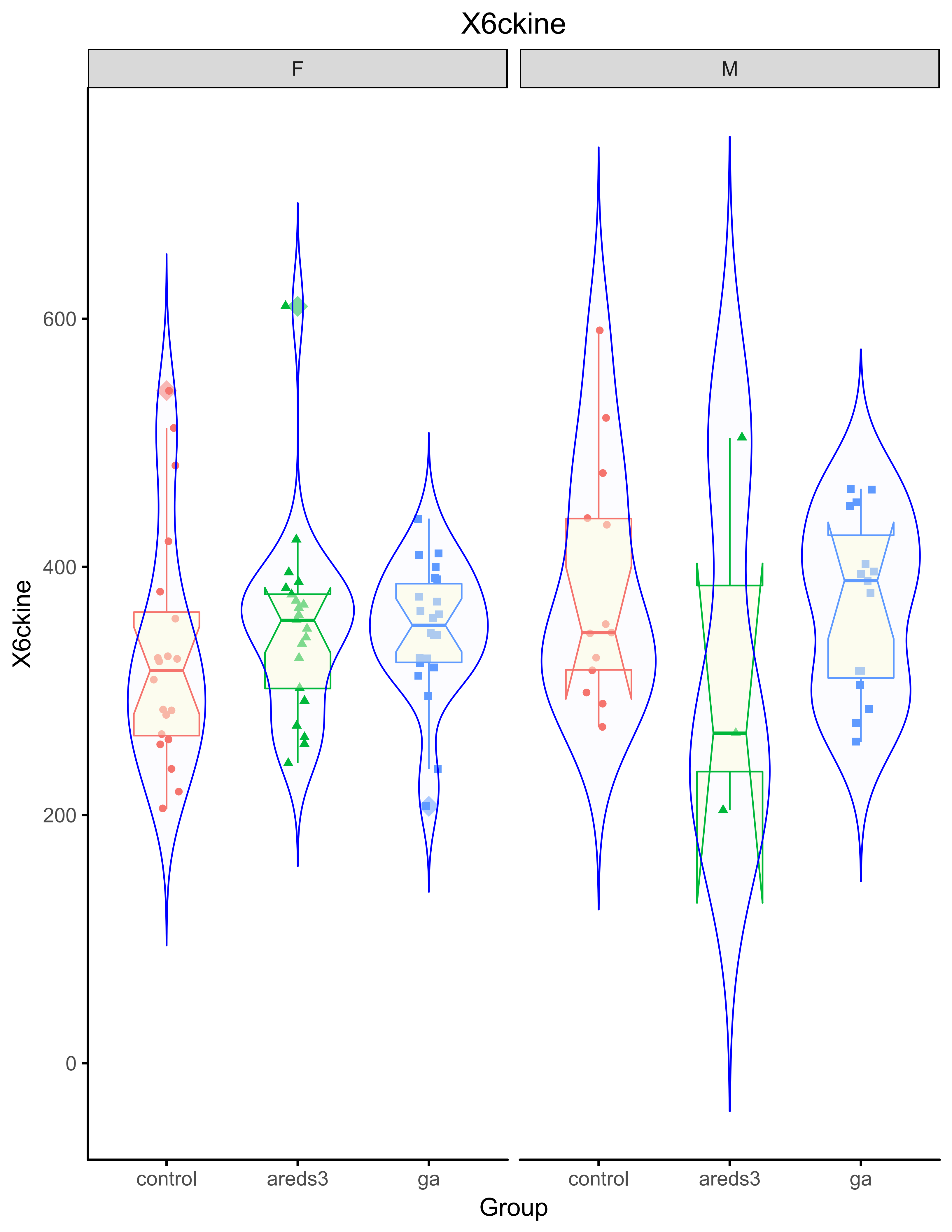

X6ckine
F
M
600
400
200
0
X6ckine
control
areds3
ga
control
areds3
ga
Group

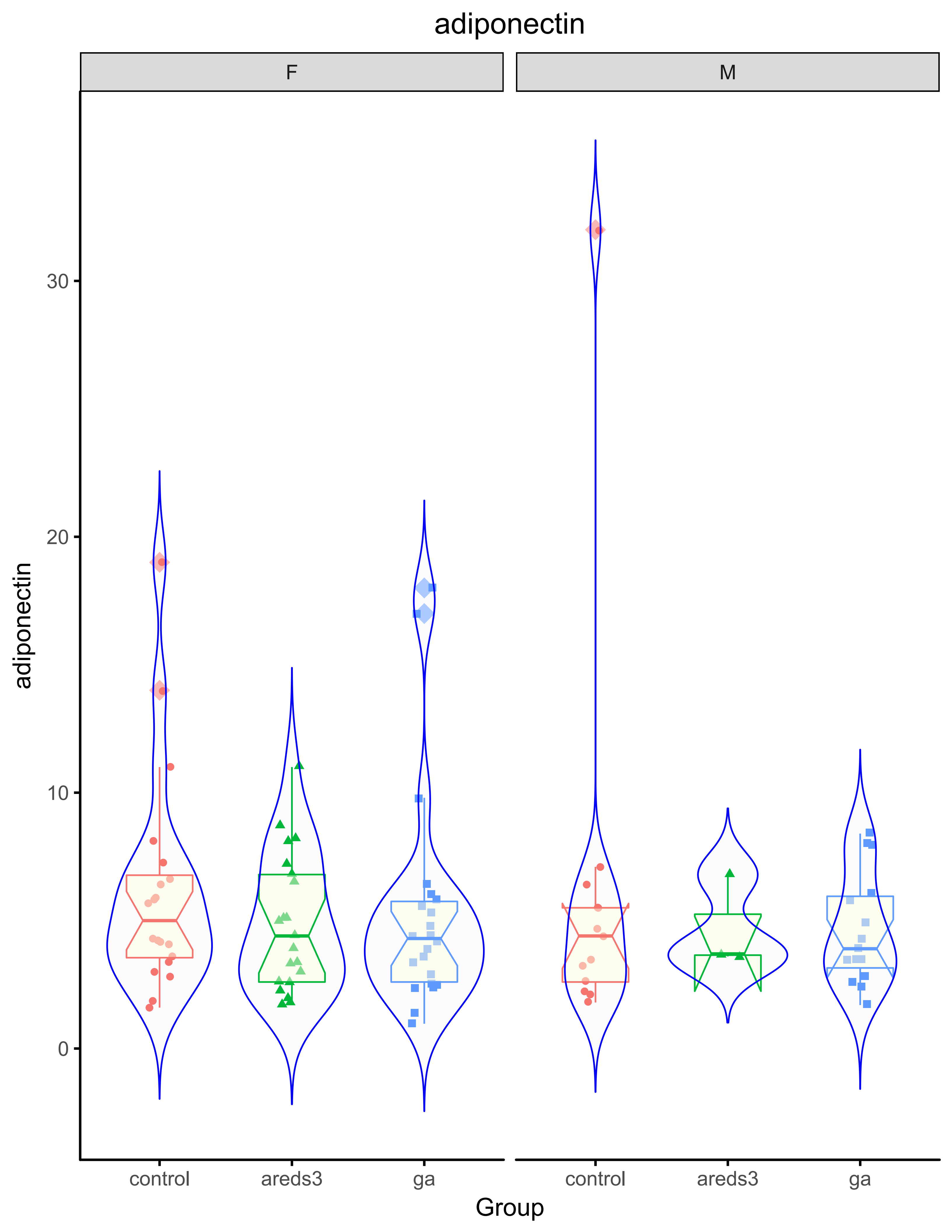
adiponectin
F
M
adiponectin
30
20
10
0
control
areds3
ga
control
areds3
ga
Group

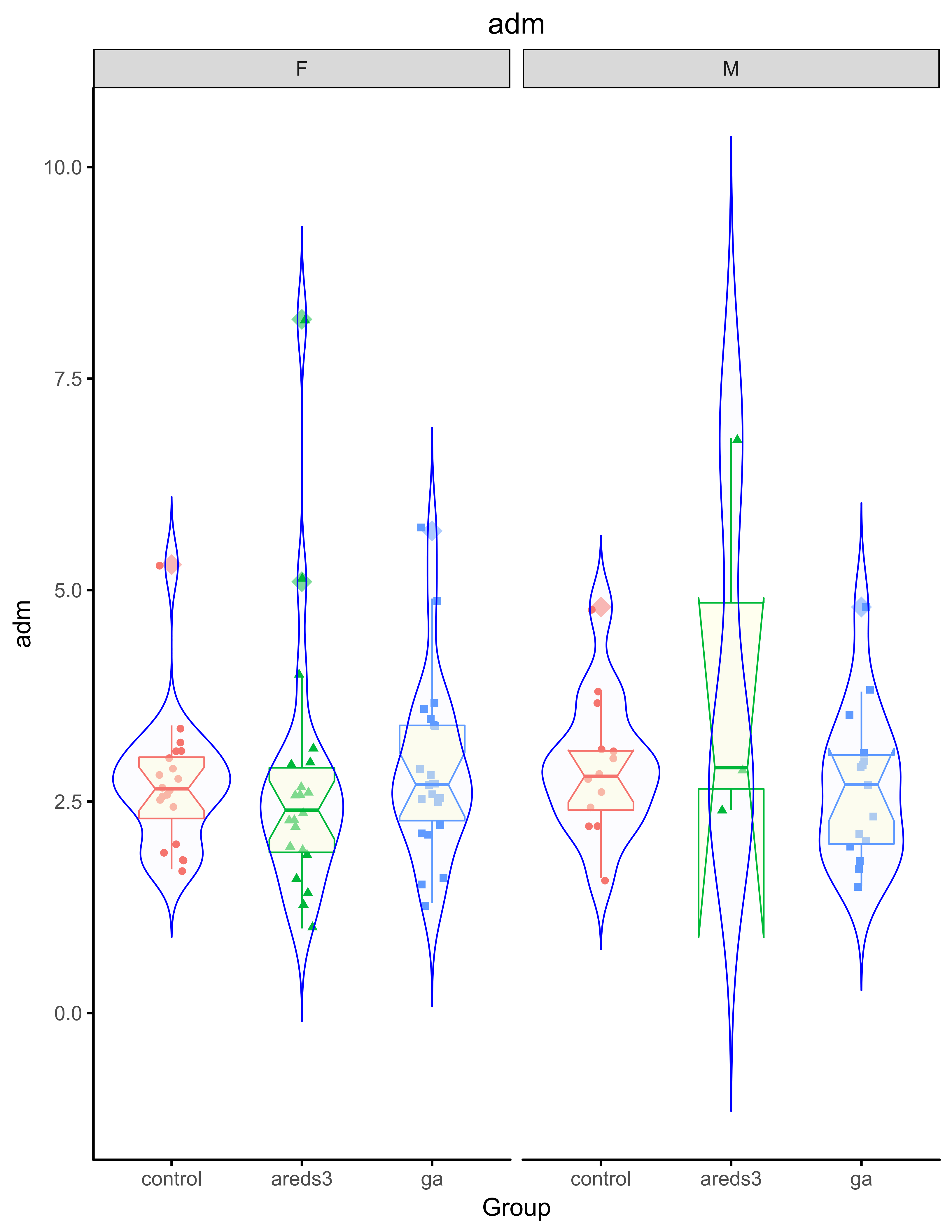

adm
F
M
10.0
7.5
5.0
2.5
0.0
adm
control
areds3
ga
control
areds3
ga
Group

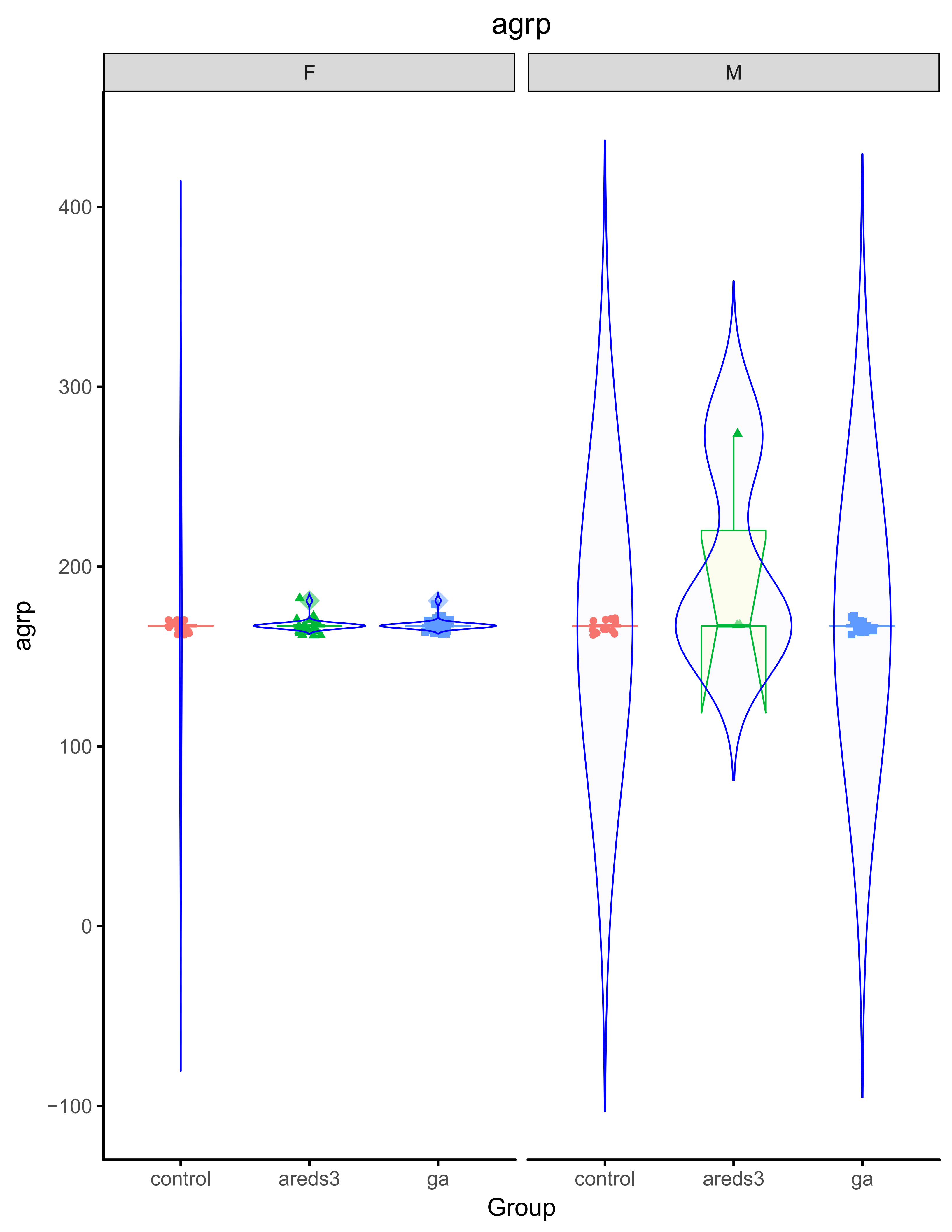

agrp
F
M
400
300
200
100
0
−100
agrp
control
areds3
ga
control
areds3
ga
Group

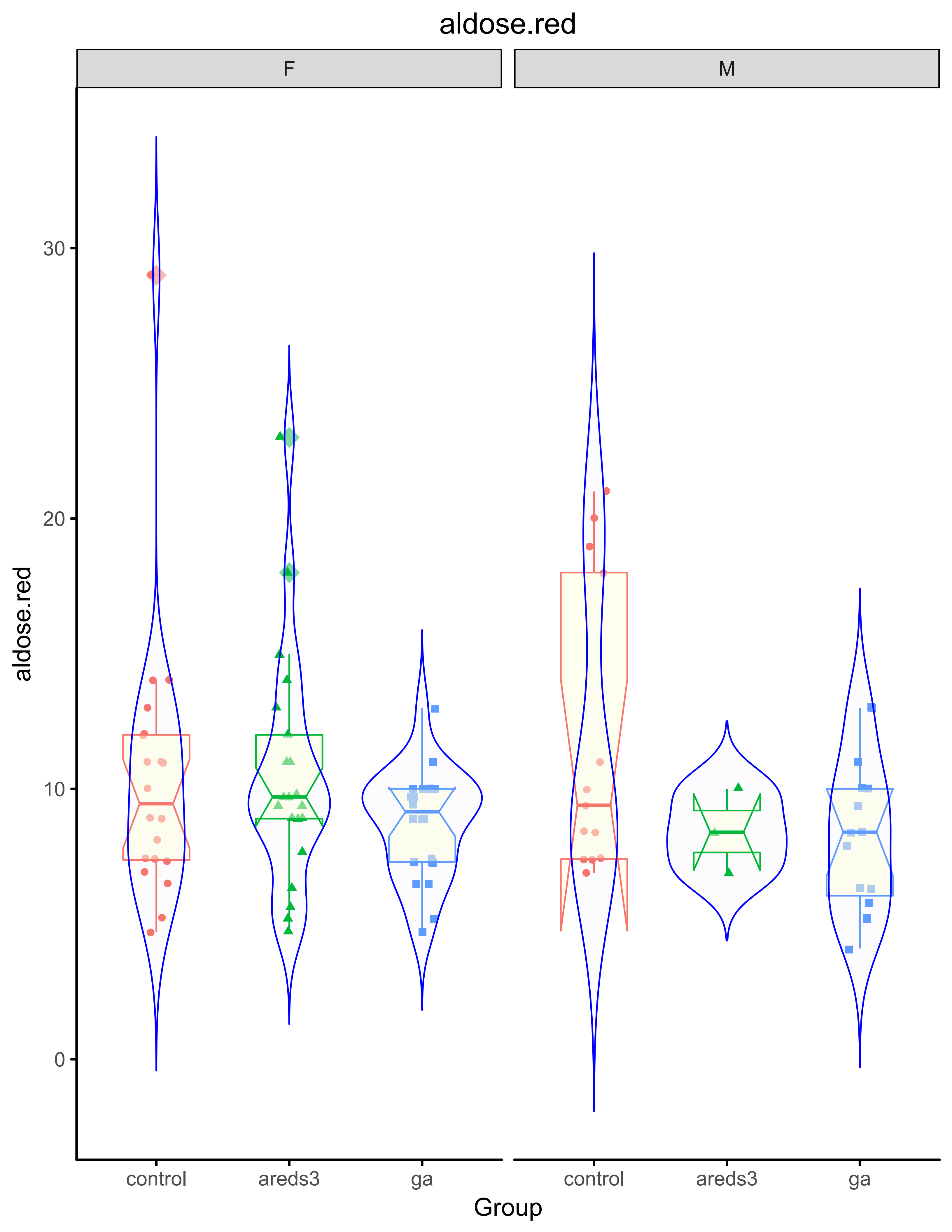
aldose.red
F
M
30
20
10
0
aldose.red
control
areds3
ga
control
areds3
ga
Group

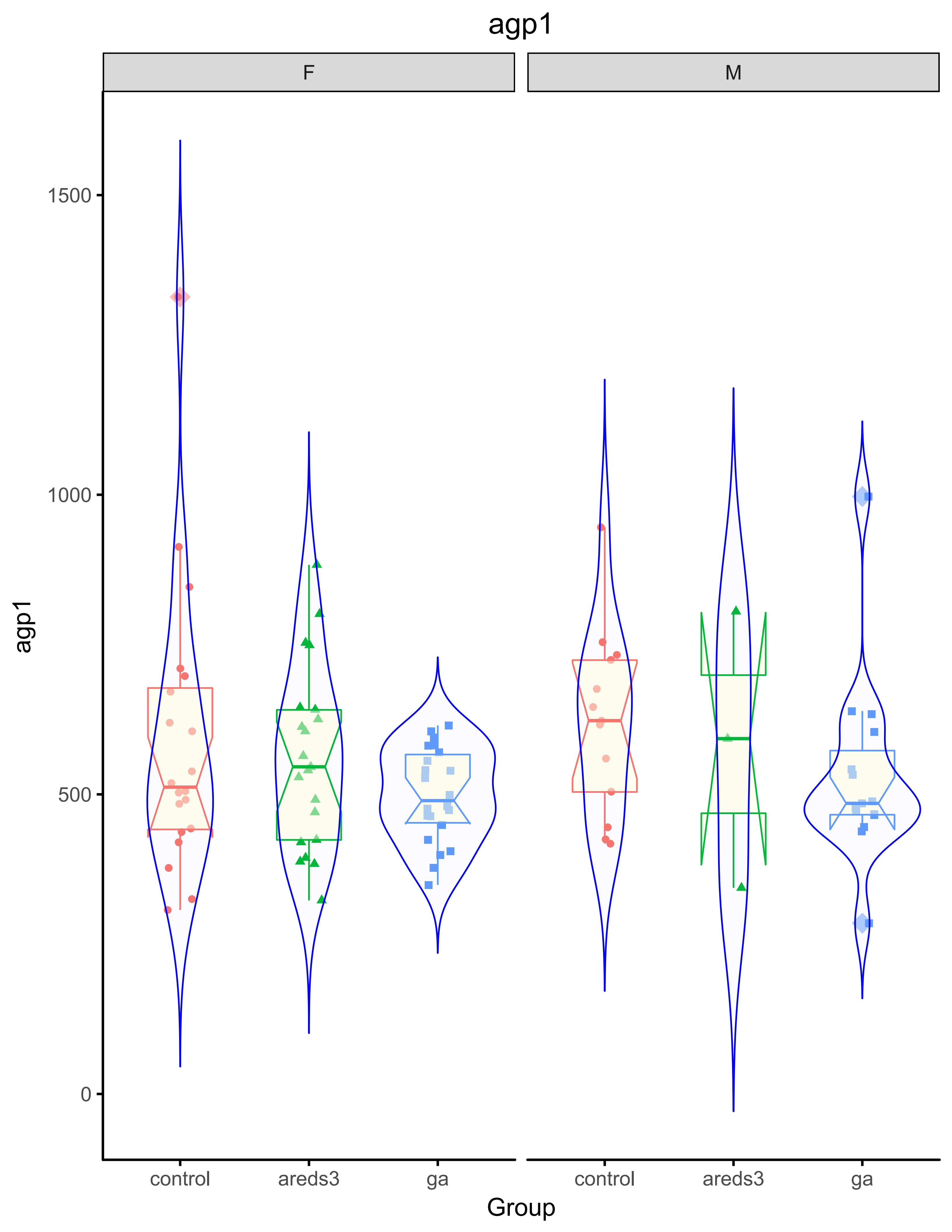

agp1
F
M
1500
1000
500
0
agp1
control
areds3
ga
control
areds3
ga
Group

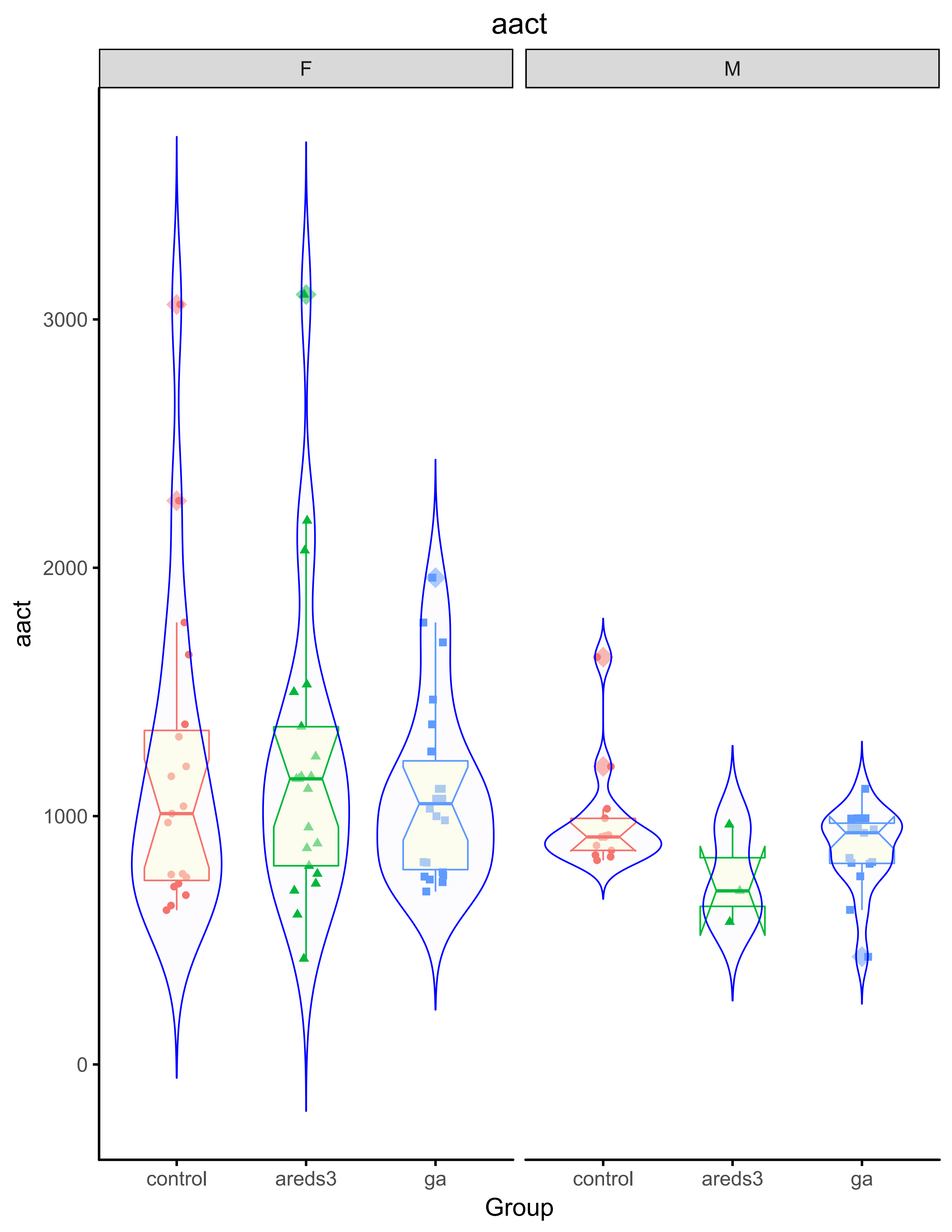

aact
F
M
3000
2000
1000
0
aact
control
areds3
ga
control
areds3
ga
Group

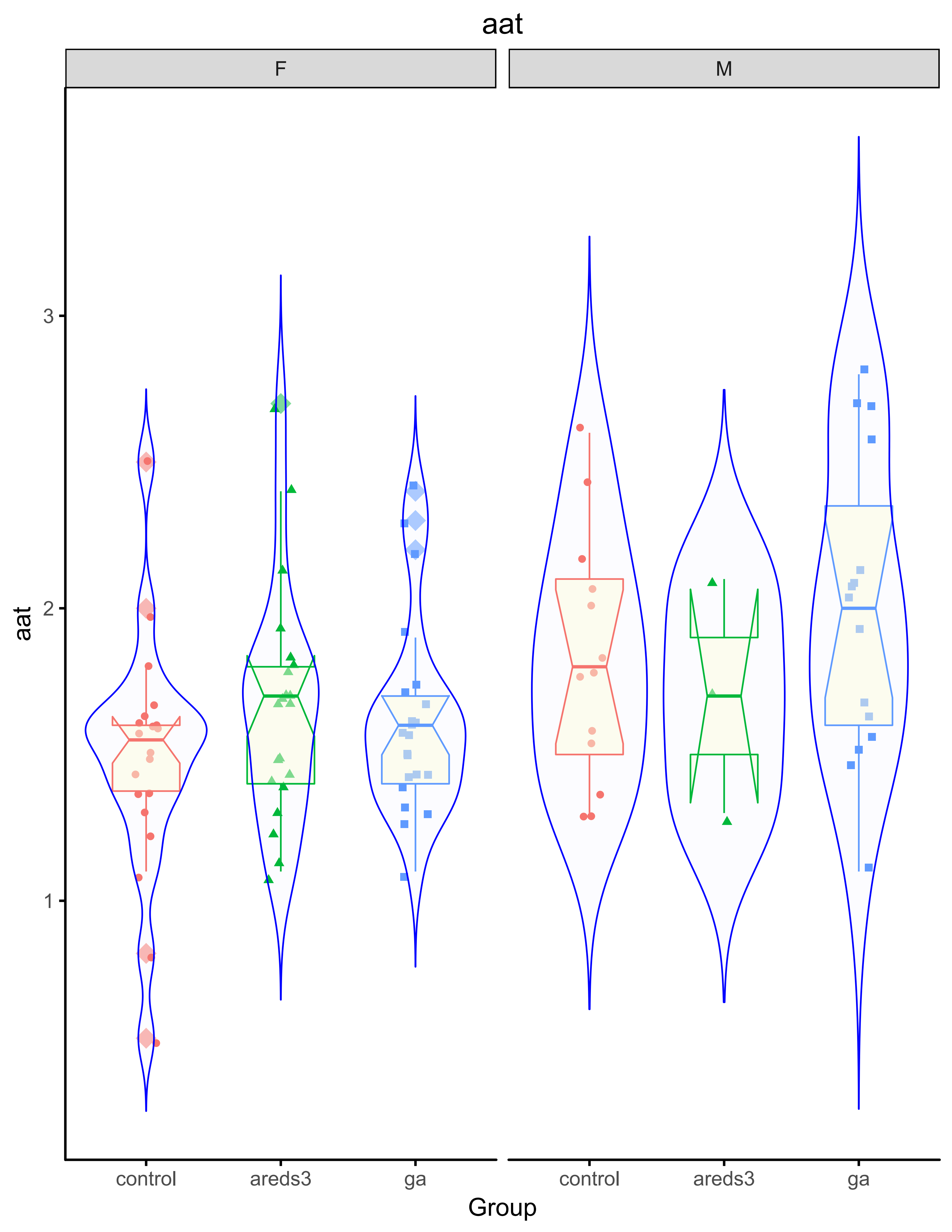
aat
F
M
3
2
1
aat
control
areds3
ga
control
areds3
ga
Group

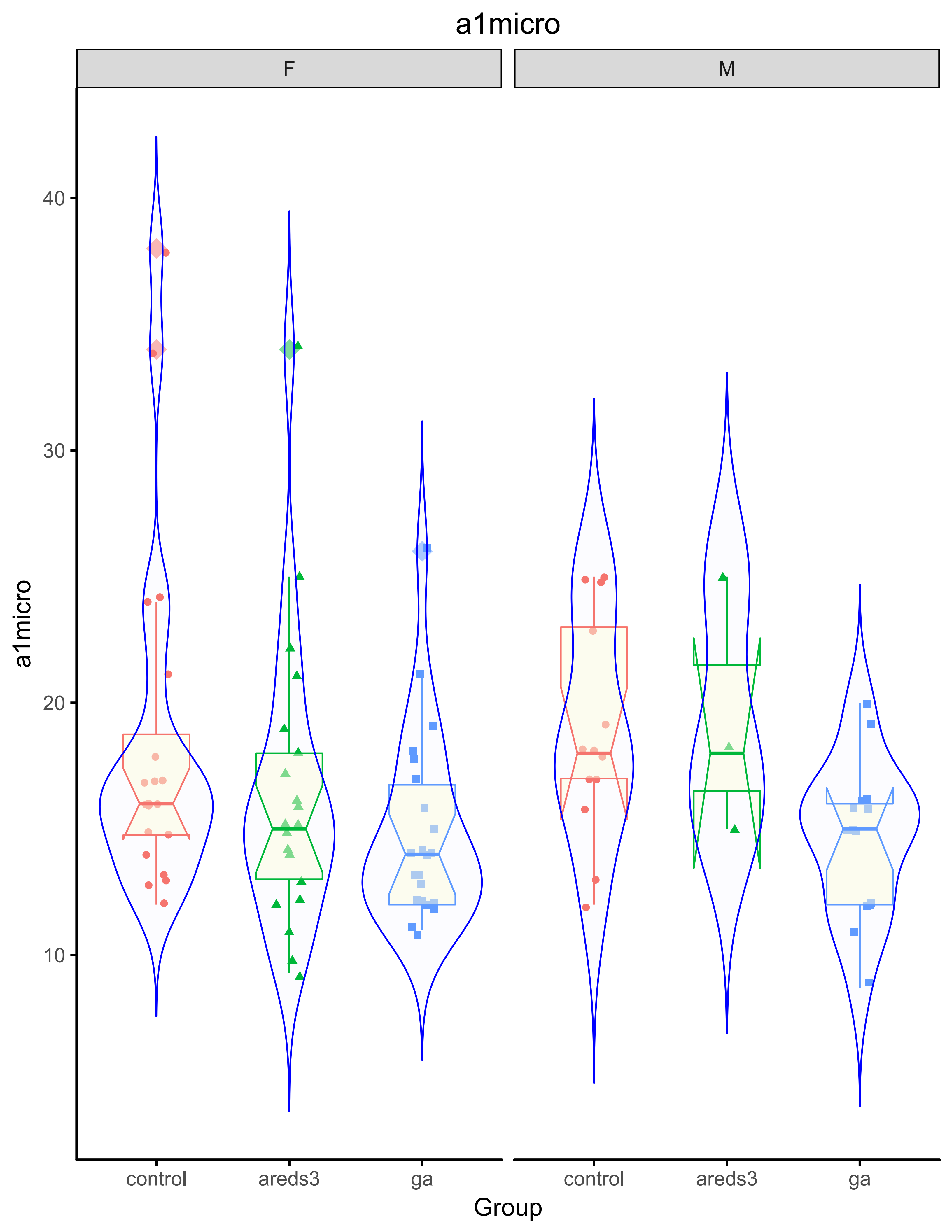
a1micro
F
M
40
30
20
10
a1micro
control
areds3
ga
control
areds3
ga
Group

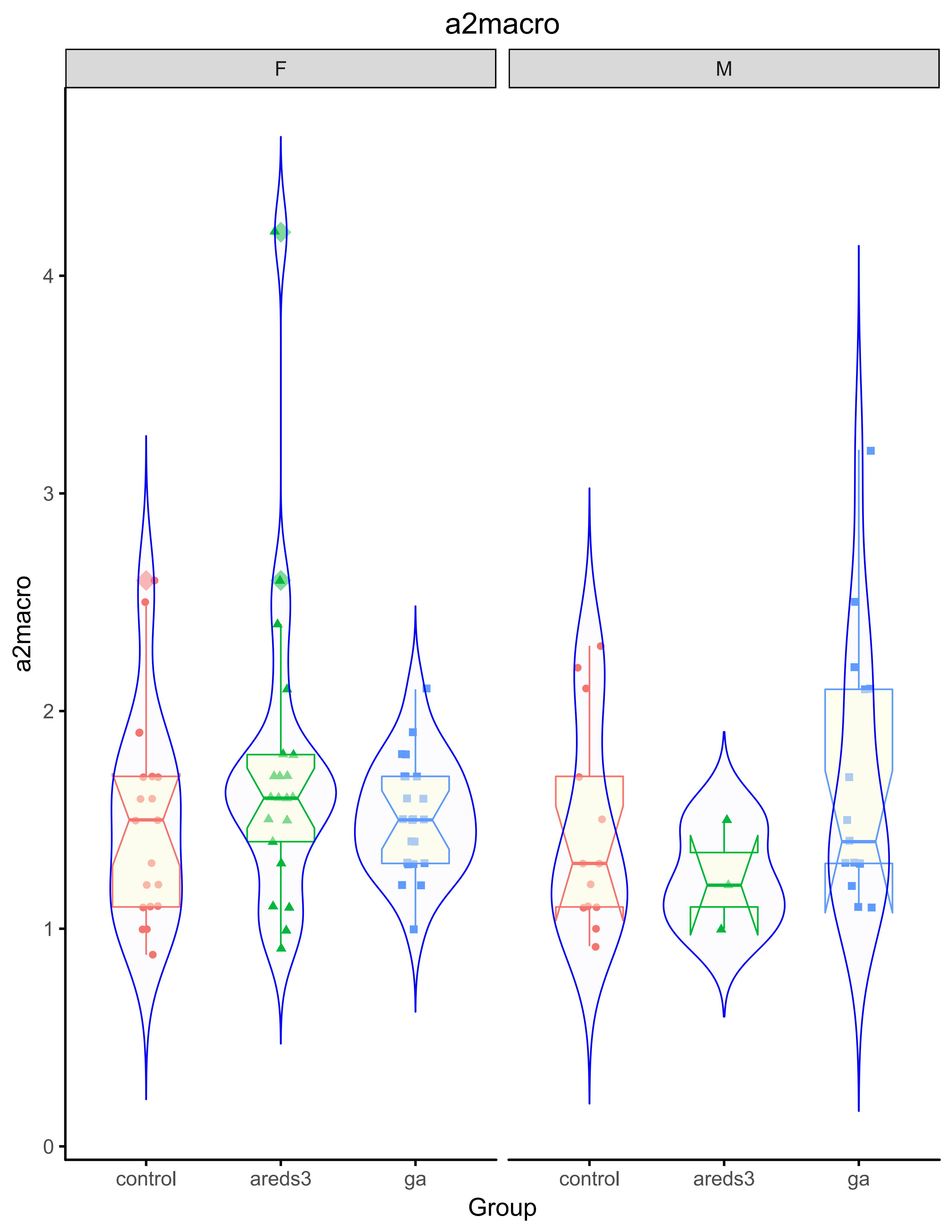
a2macro
F
M
a2macro
0
1
2
3
4
control
areds3
ga
control
areds3
ga
Group

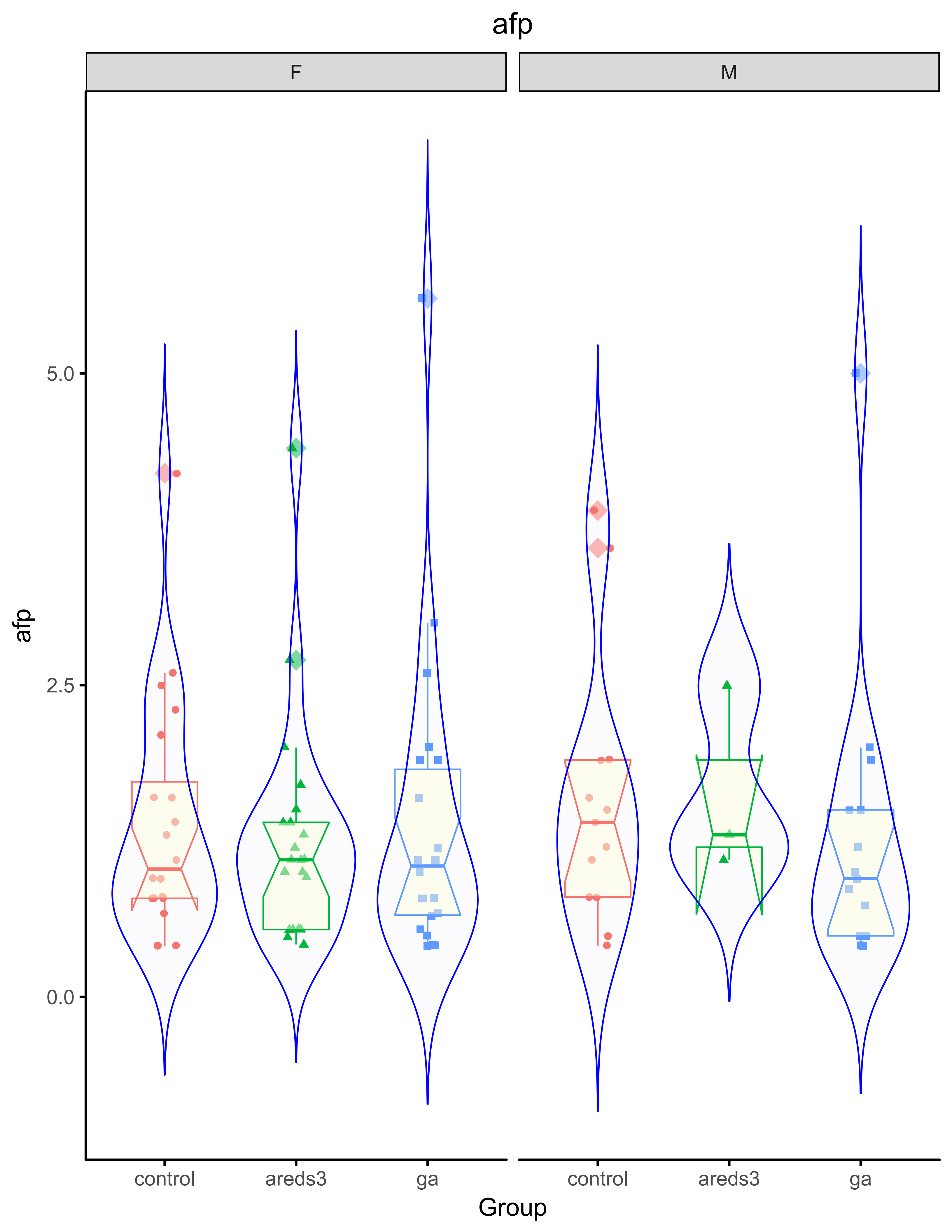
afp
F
M
5.0
2.5
0.0
afp
control
areds3
ga
control
areds3
ga
Group

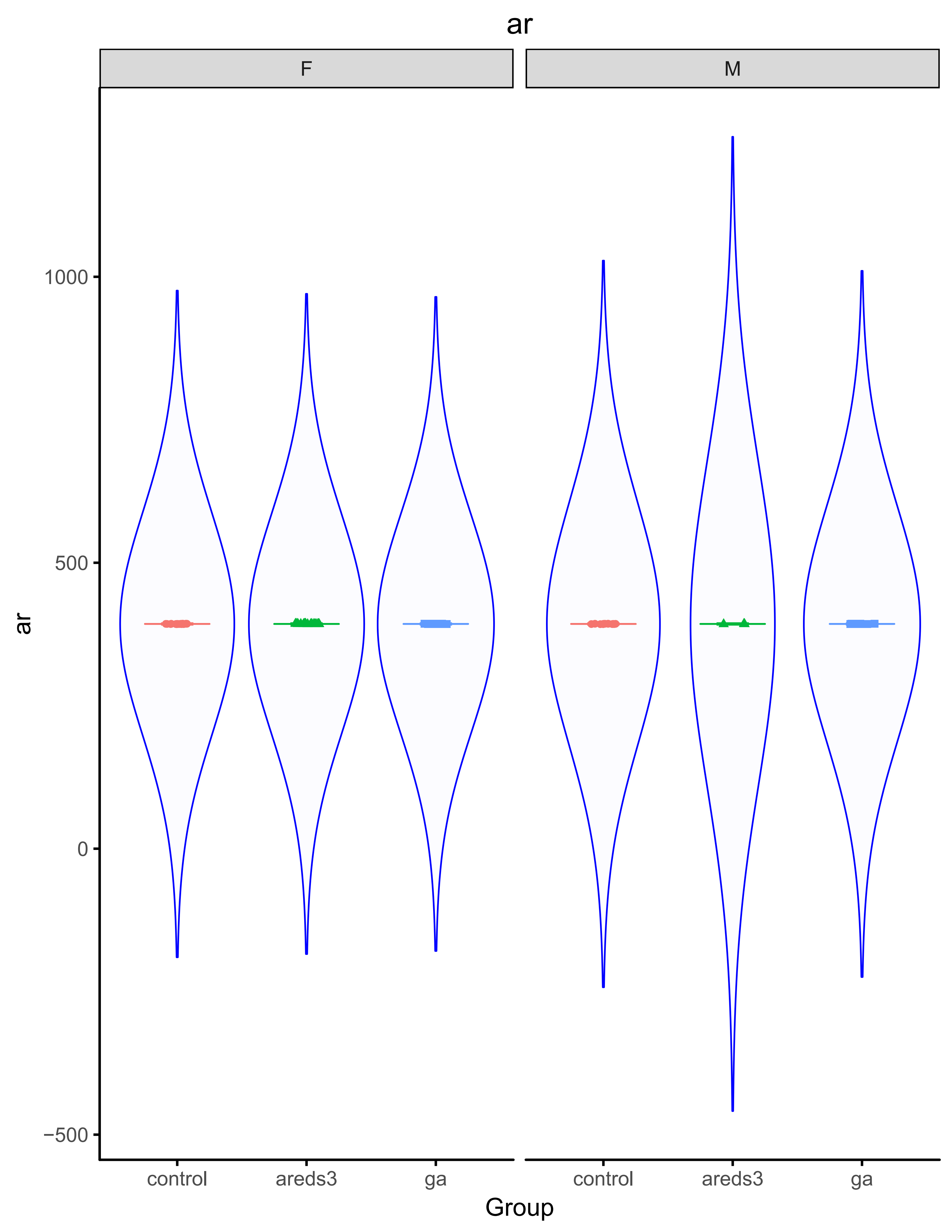
ar
F
M
1000
500
0
−500
ar
control
areds3
ga
control
areds3
ga
Group

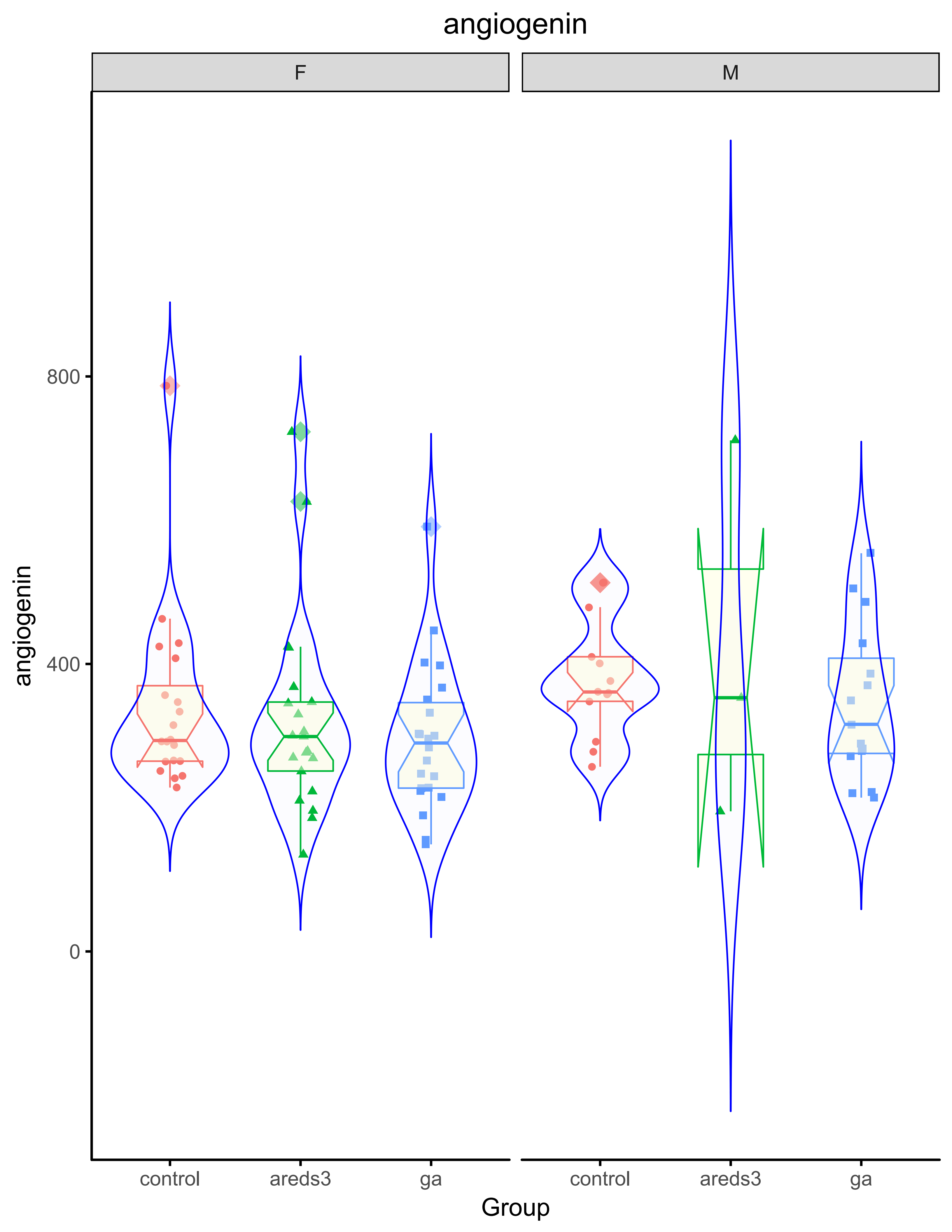

angiogenin
F
M
800
400
0
angiogenin
control
areds3
ga
control
areds3
ga
Group

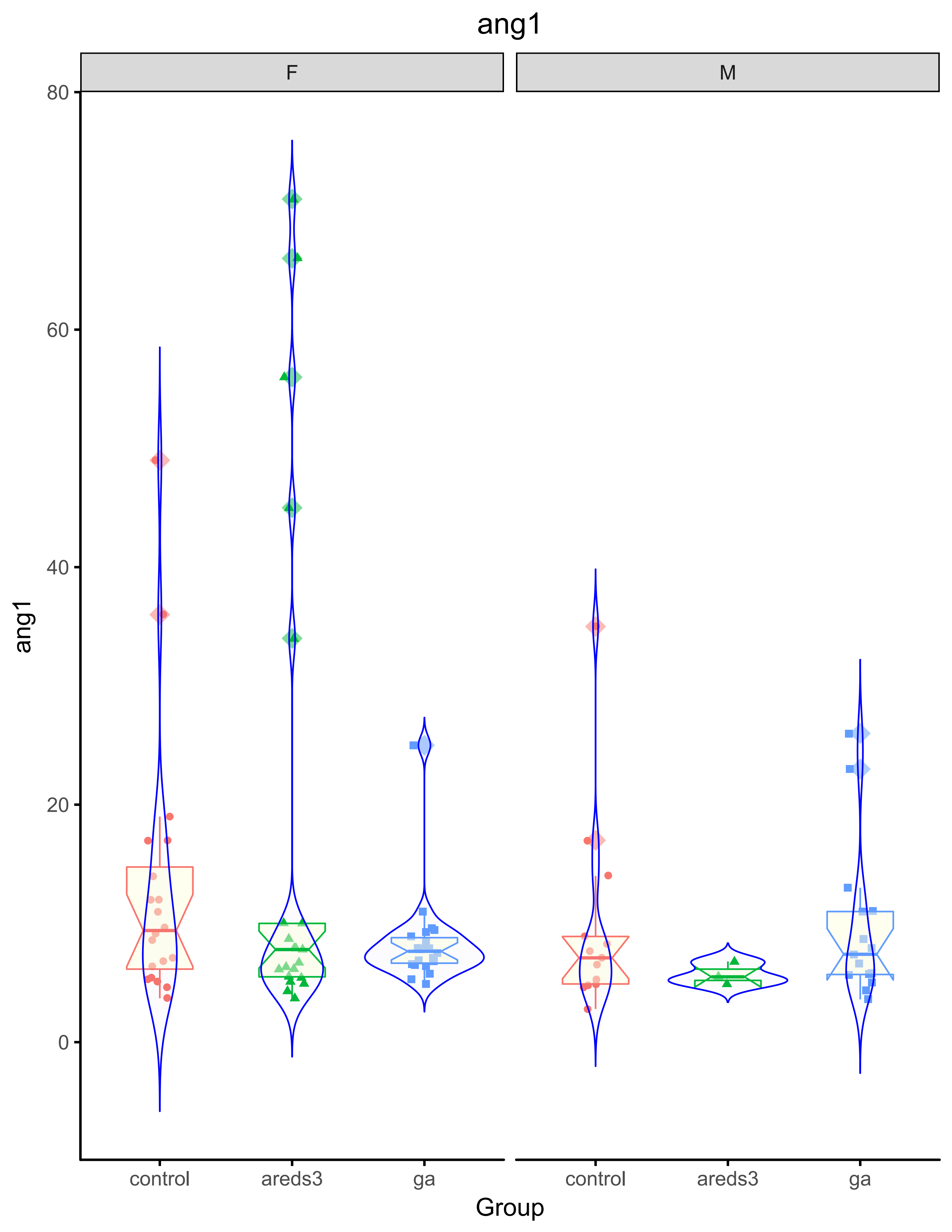
ang1
F
M
80
60
40
20
0
ang1
control
areds3
ga
control
areds3
ga
Group

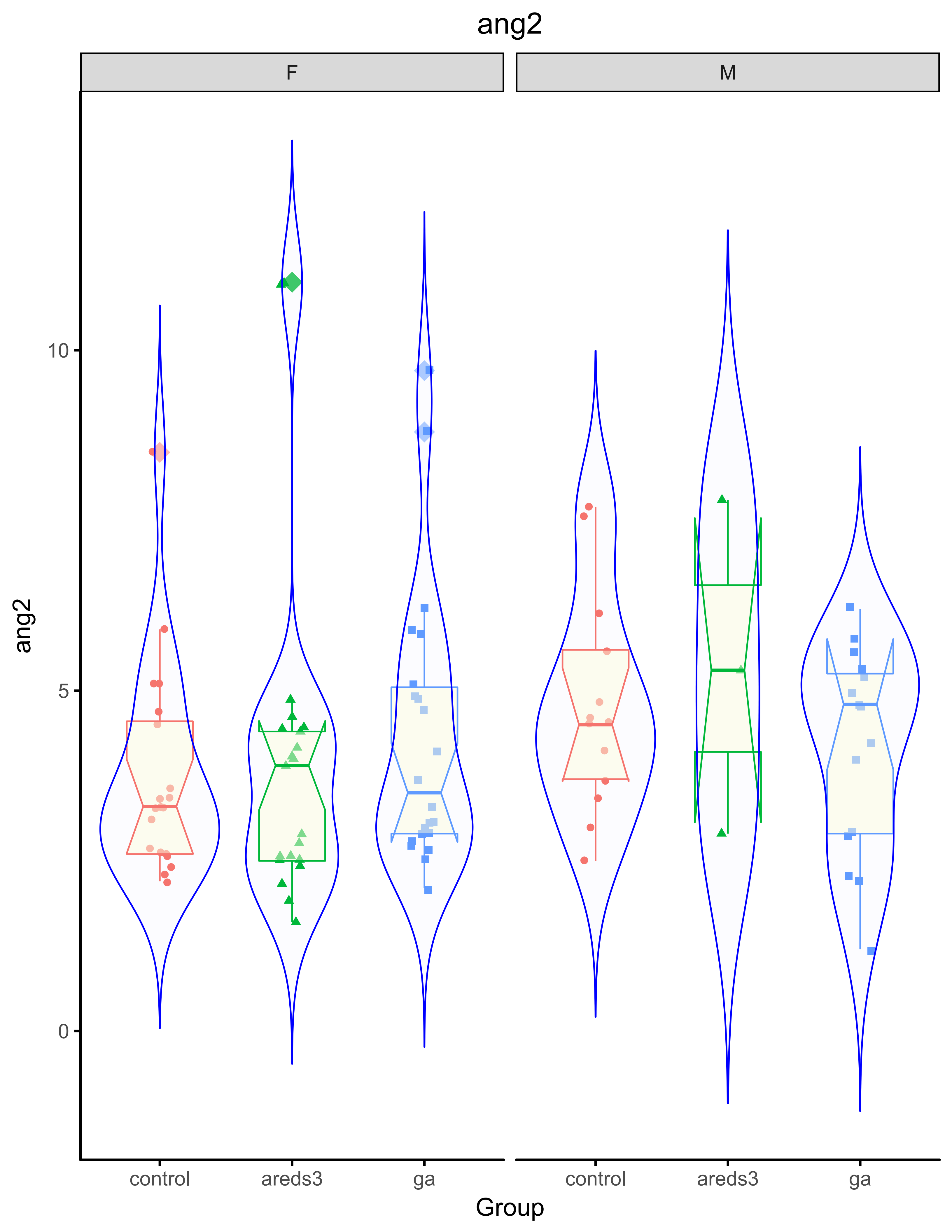
ang2
F
M
ang2
10
5
0
control
areds3
ga
control
areds3
ga
Group

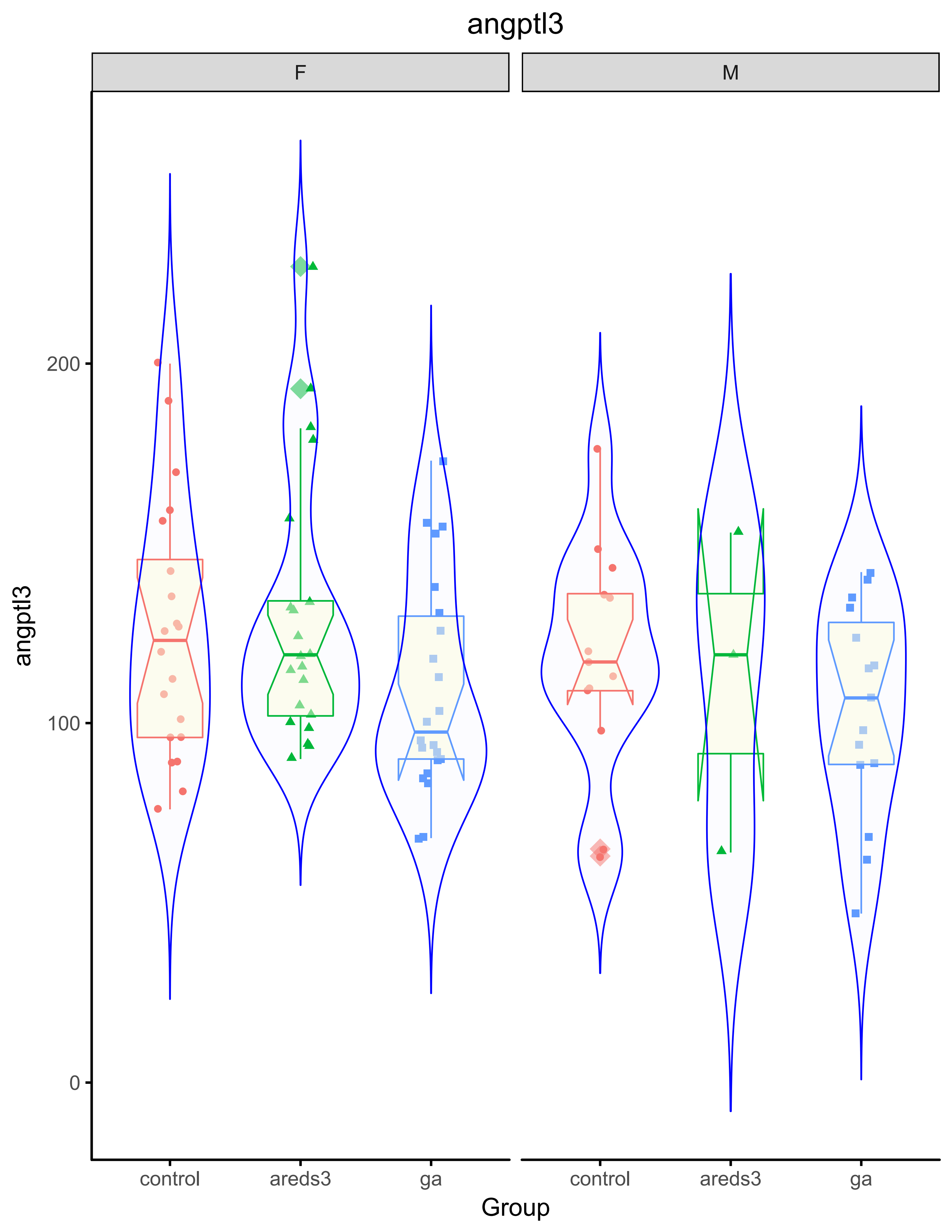

angptl3
F
M
200
100
0
angptl3
control
areds3
ga
control
areds3
ga
Group

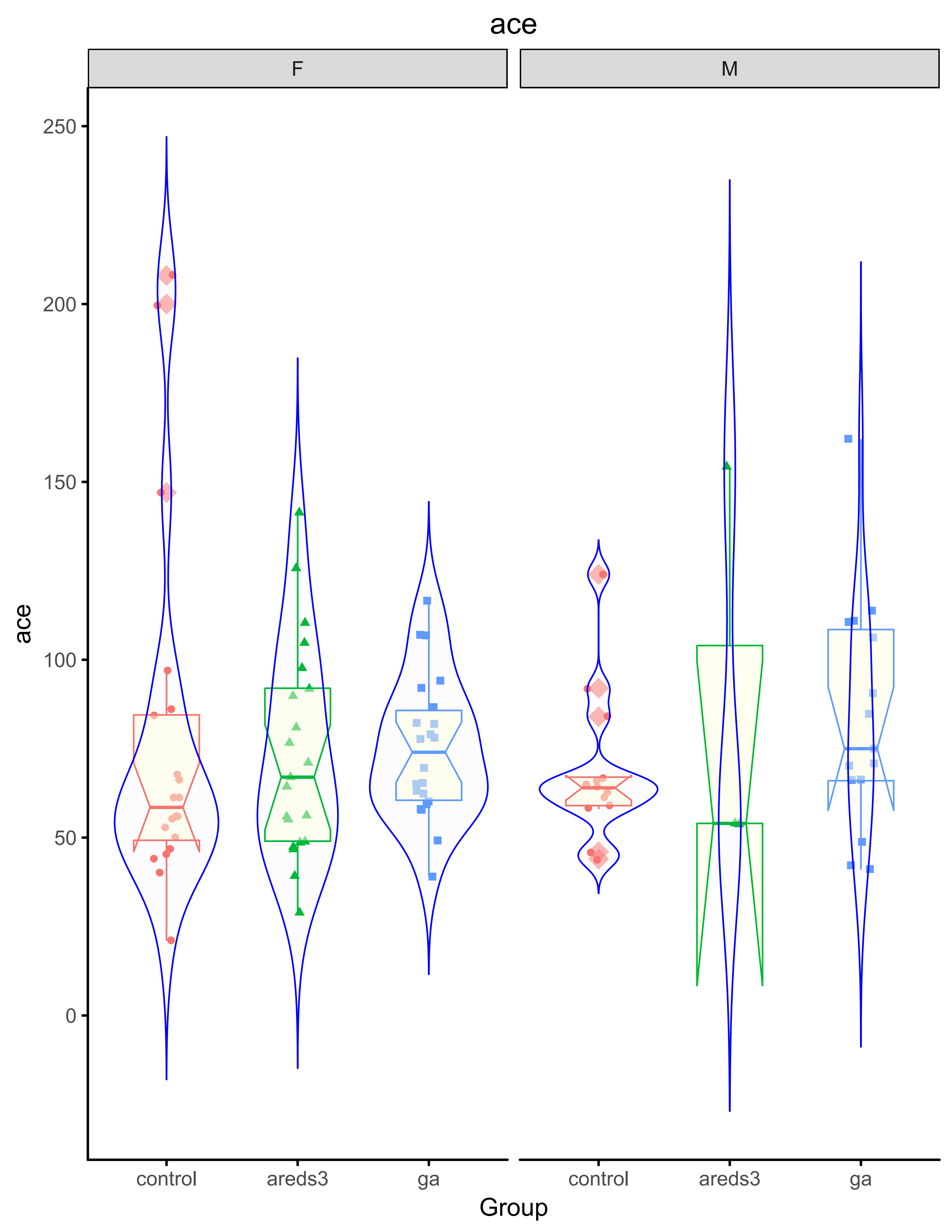

ace
F
M
250
200
150
100
50
0
ace
control
areds3
ga
control
areds3
ga
Group

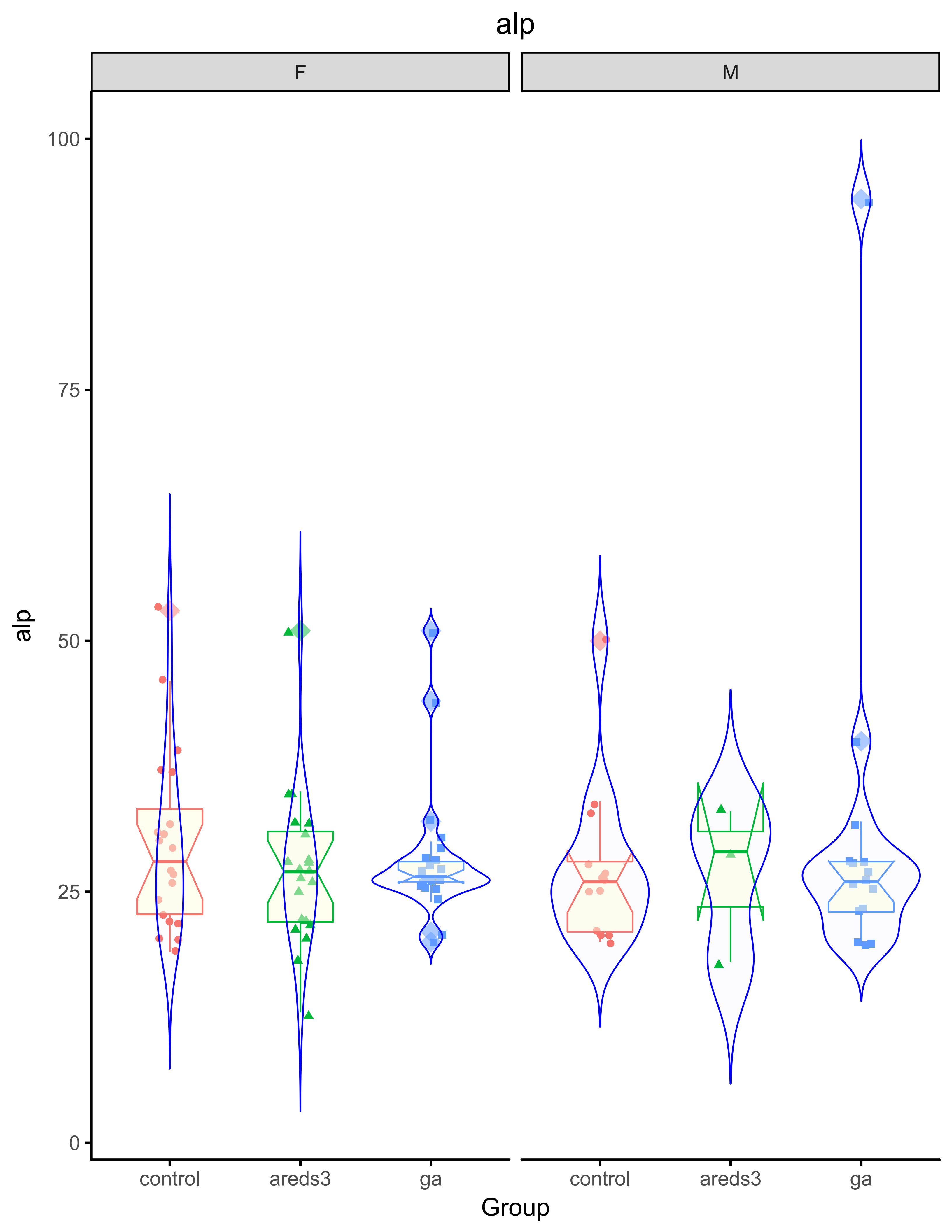

alp
F
M
100
75
50
25
0
alp
control
areds3
ga
control
areds3
ga
Group

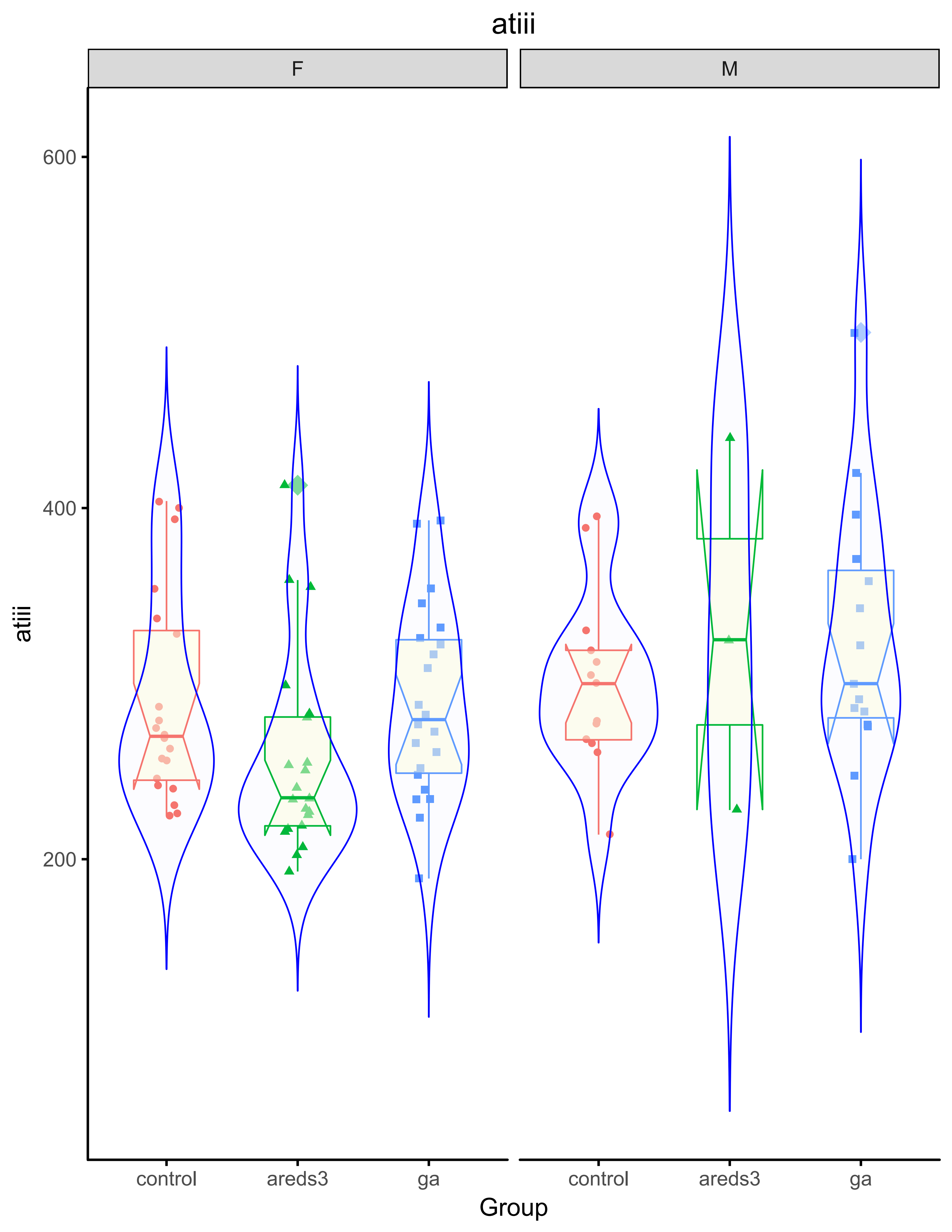

atiii
F
M
600
400
200
atiii
control
areds3
ga
control
areds3
ga
Group

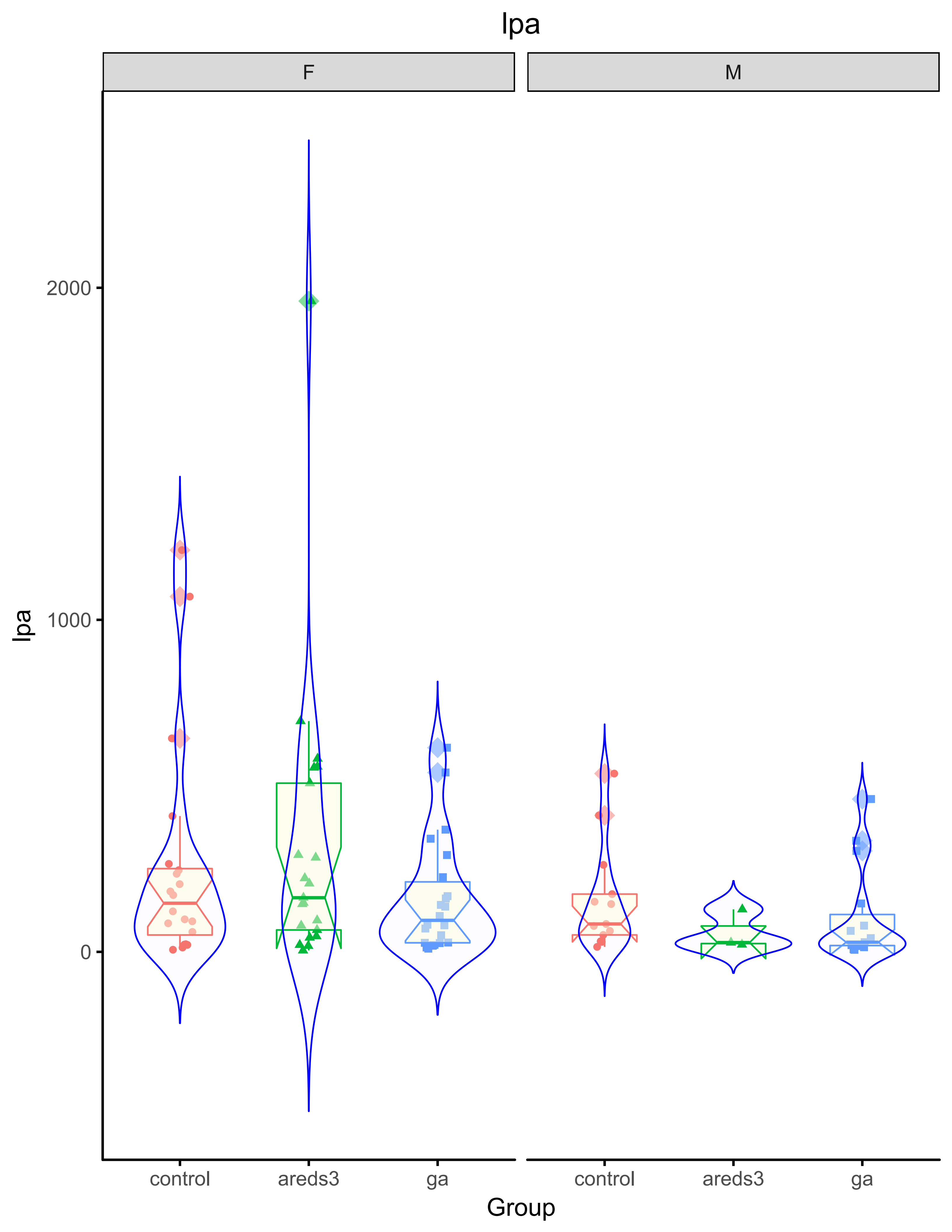

lpa
F
M
2000
1000
0
lpa
control
areds3
ga
control
areds3
ga
Group

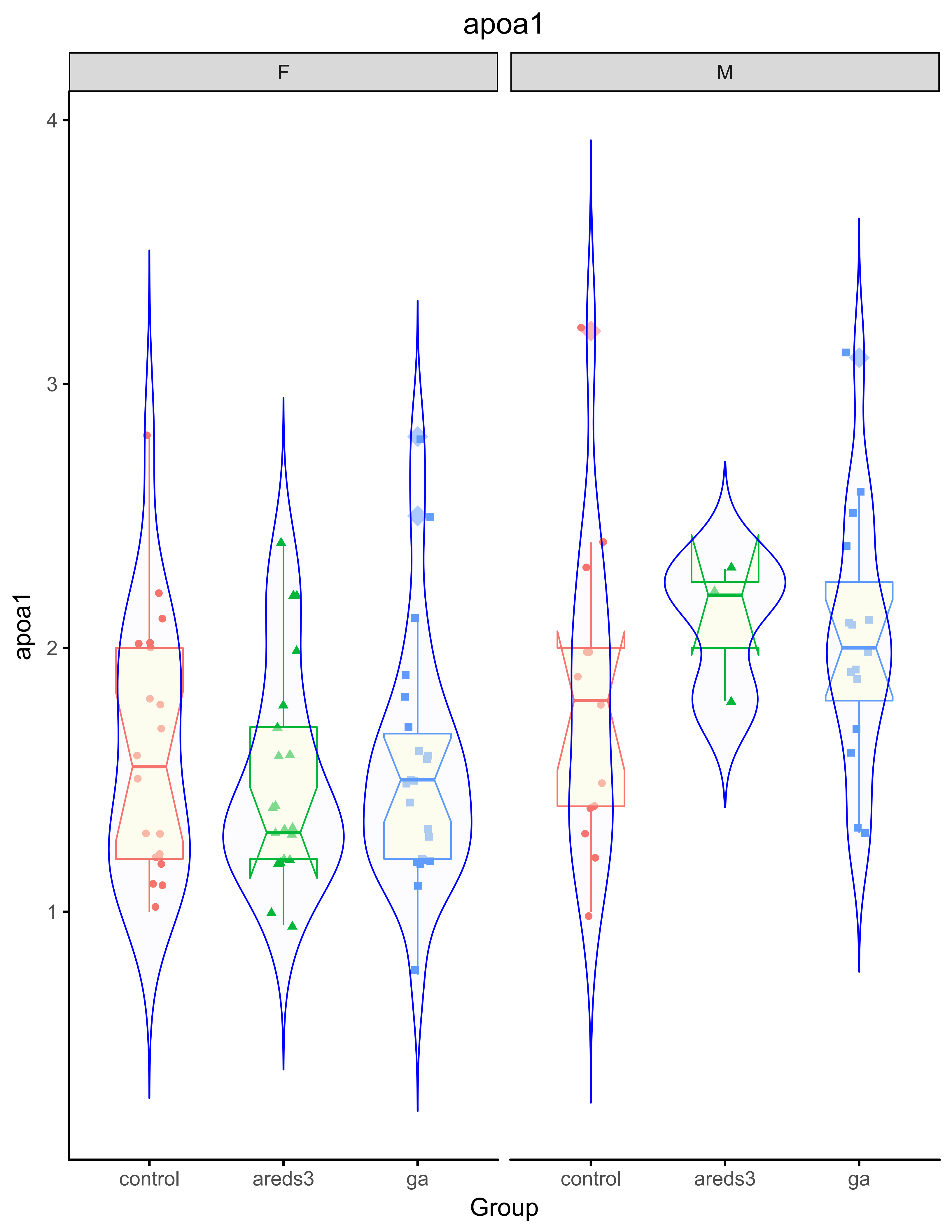
apoa1
F
M
4
3
2
1
apoa1
control
areds3
ga
control
areds3
ga
Group

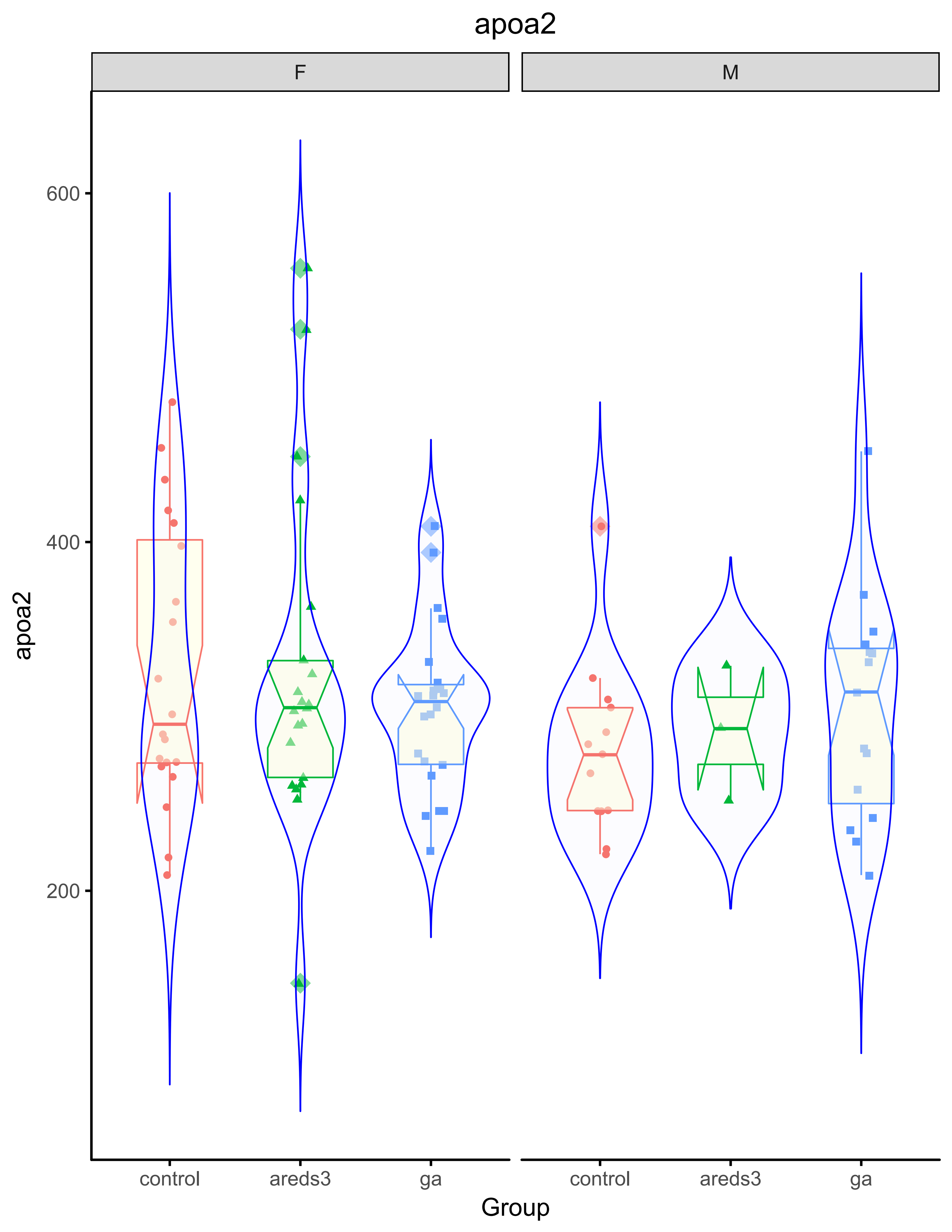

apoa2
F
M
600
400
200
apoa2
control
areds3
ga
control
areds3
ga
Group

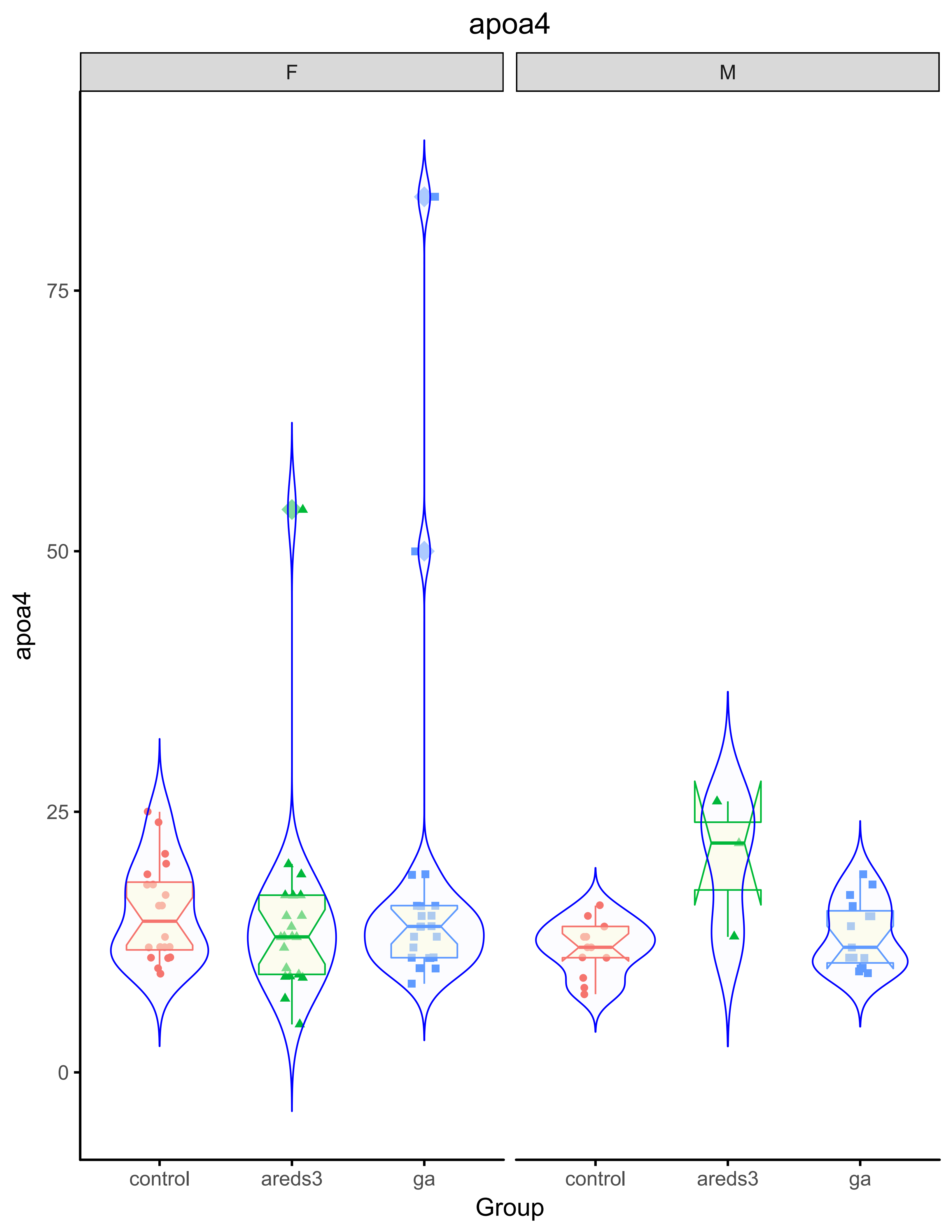
apoa4
F
M
apoa4
75
50
25
0
control
areds3
ga
control
areds3
ga
Group

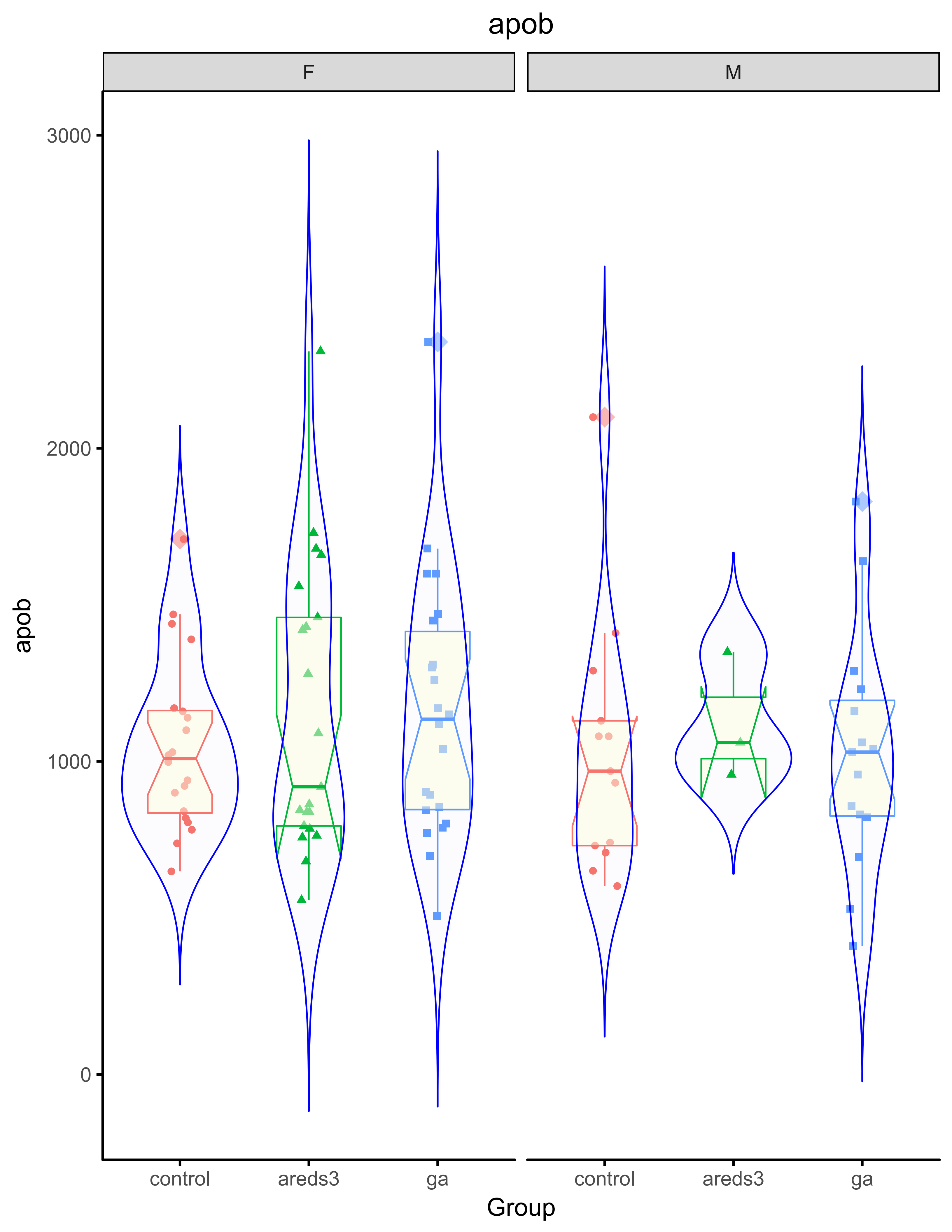

apob
F
M
3000
2000
1000
0
apob
control
areds3
ga
control
areds3
ga
Group

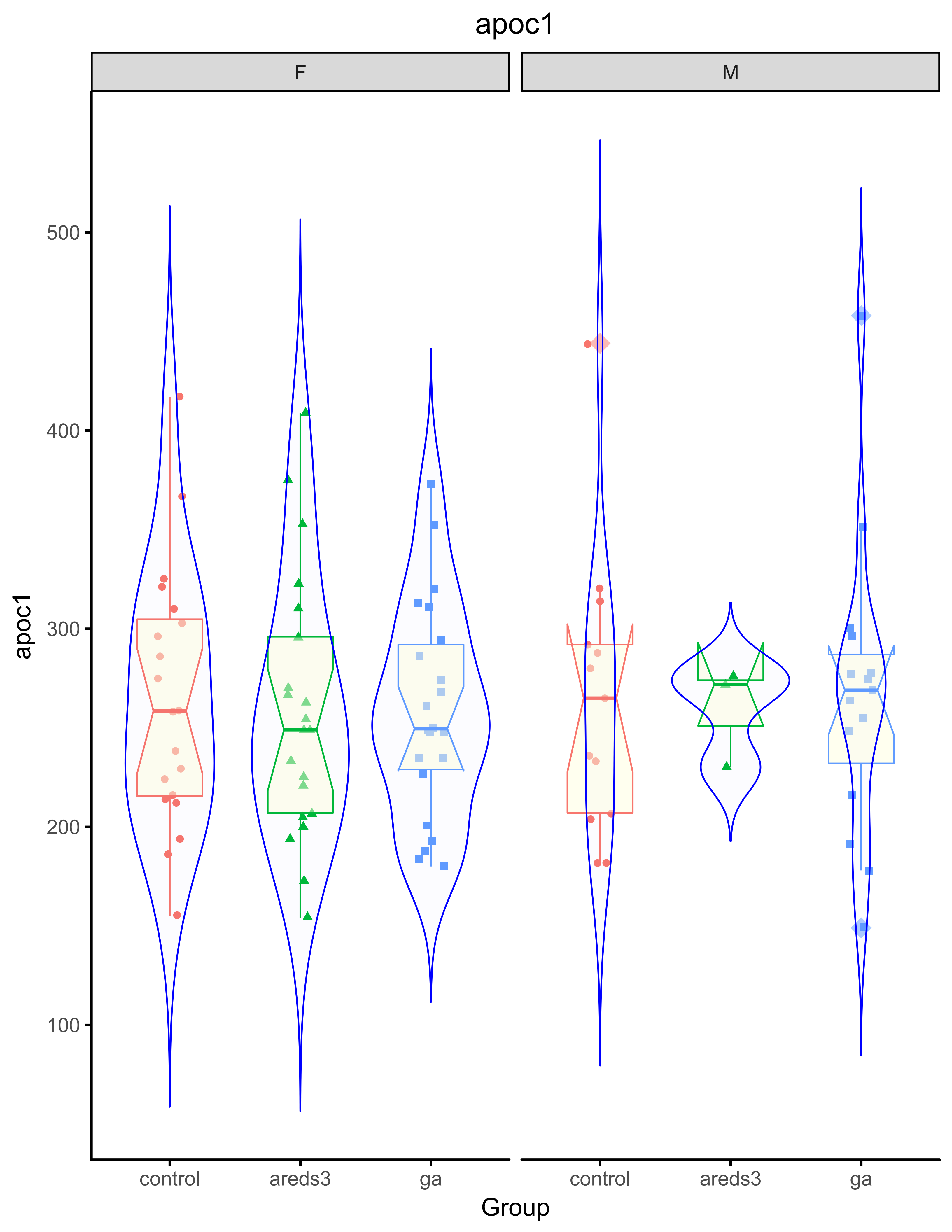

apoc1
F
M
500
400
300
200
100
apoc1
control
areds3
ga
control
areds3
ga
Group

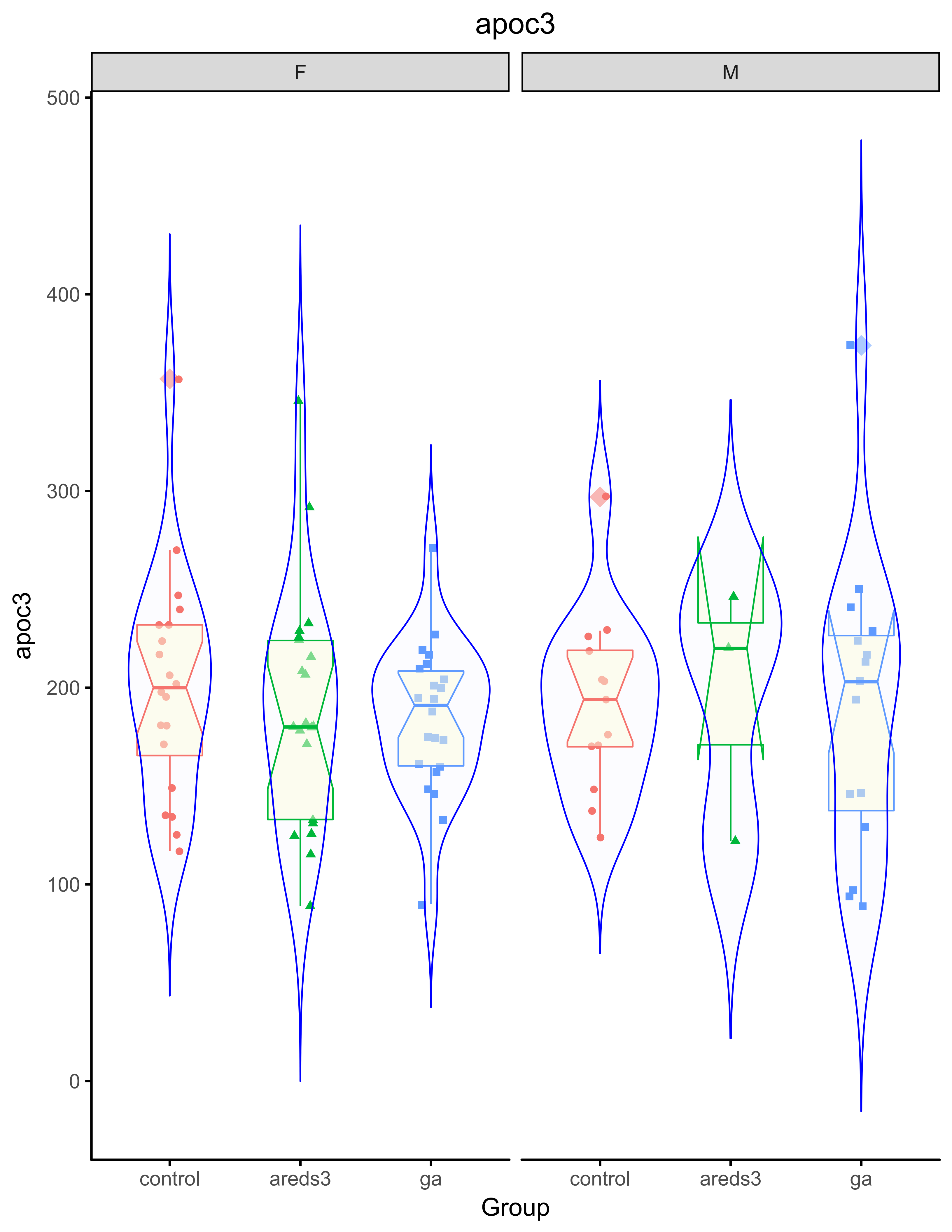
apoc3
F
M
500
400
300
200
100
0
apoc3
control
areds3
ga
control
areds3
ga
Group

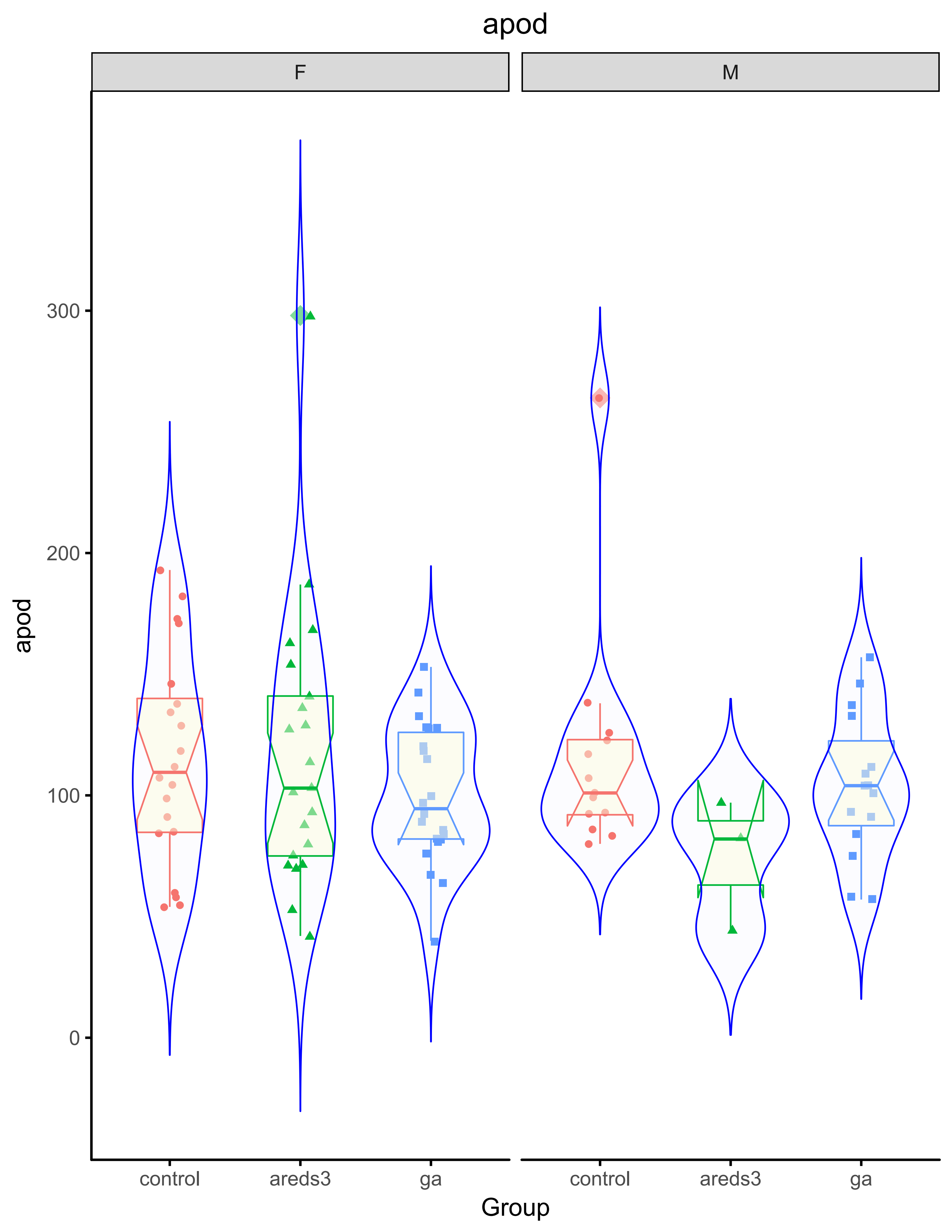
apod
F
M
300
200
100
0
apod
control
areds3
ga
control
areds3
ga
Group

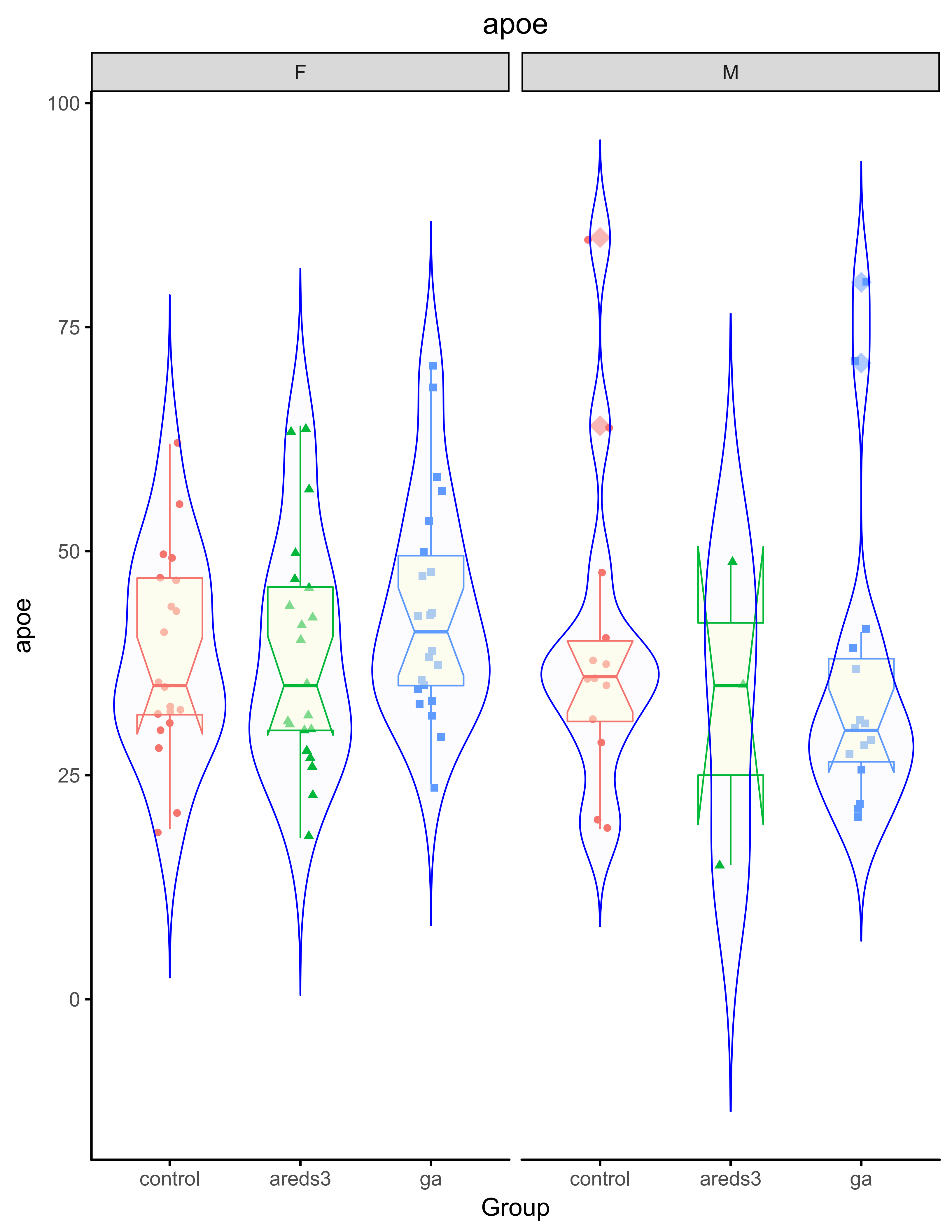
apoe
F
M
100
75
50
25
0
apoe
control
areds3
ga
control
areds3
ga
Group

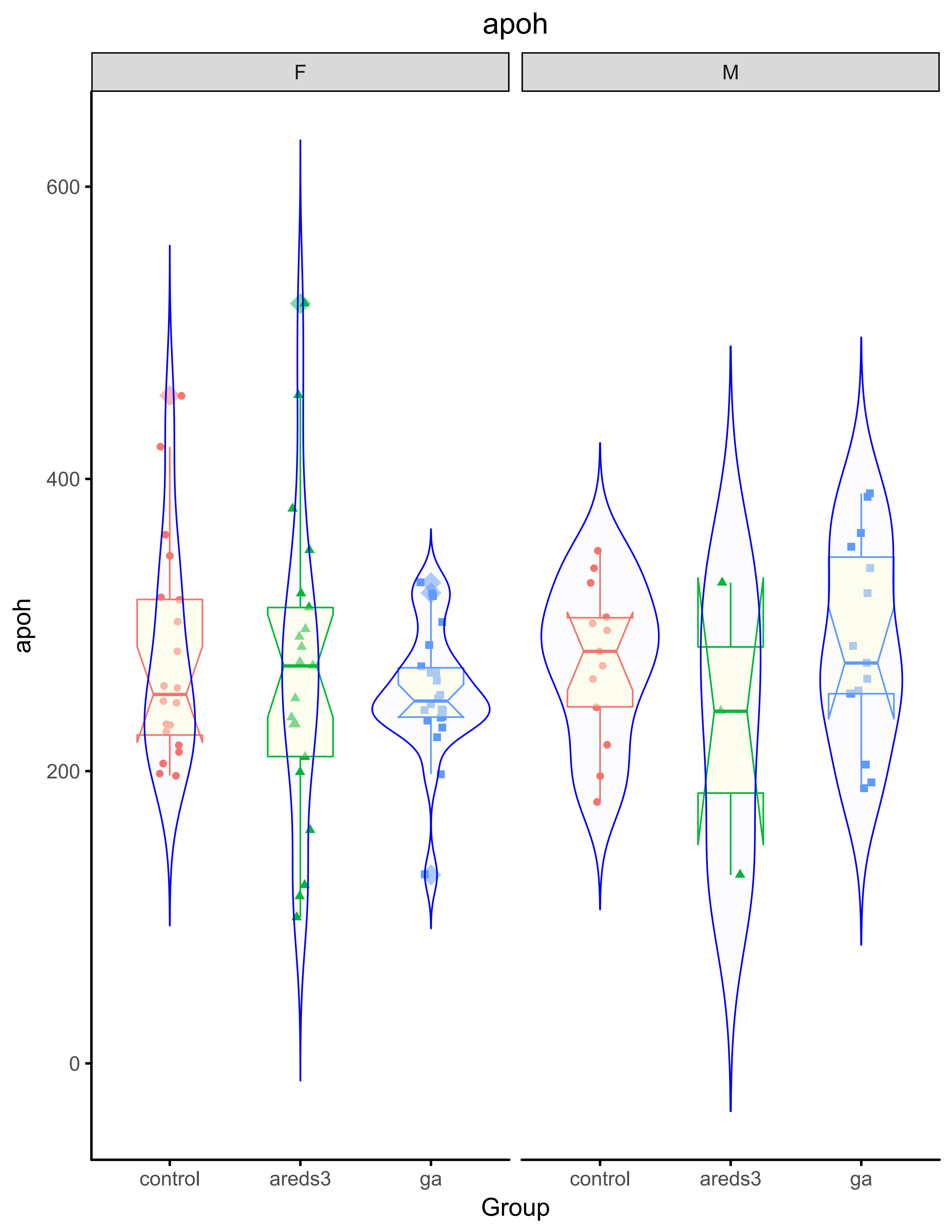

apoh
F
M
600
400
200
0
apoh
control
areds3
ga
control
areds3
ga
Group

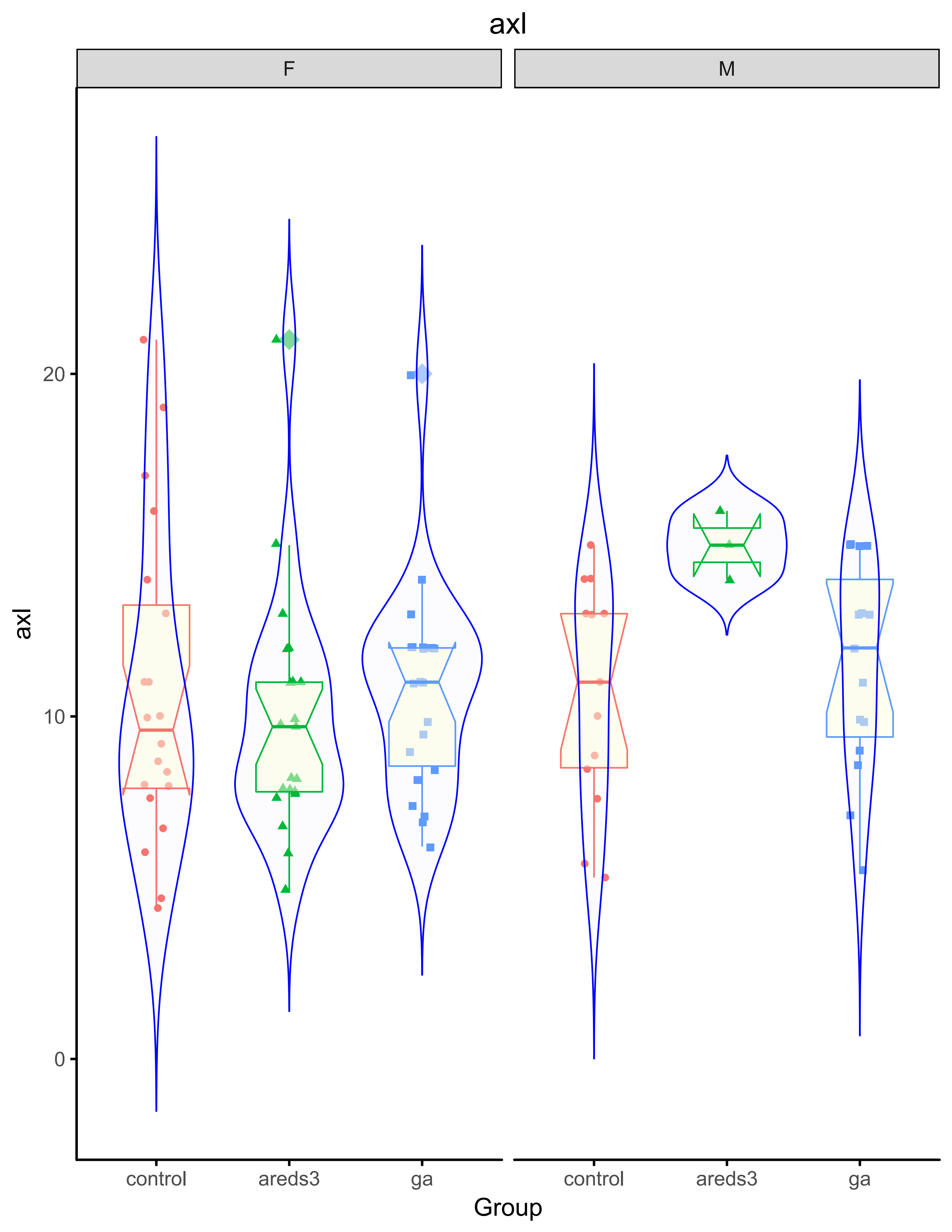

axl
F
M
axl
20
10
0
control
areds3
ga
control
areds3
ga
Group

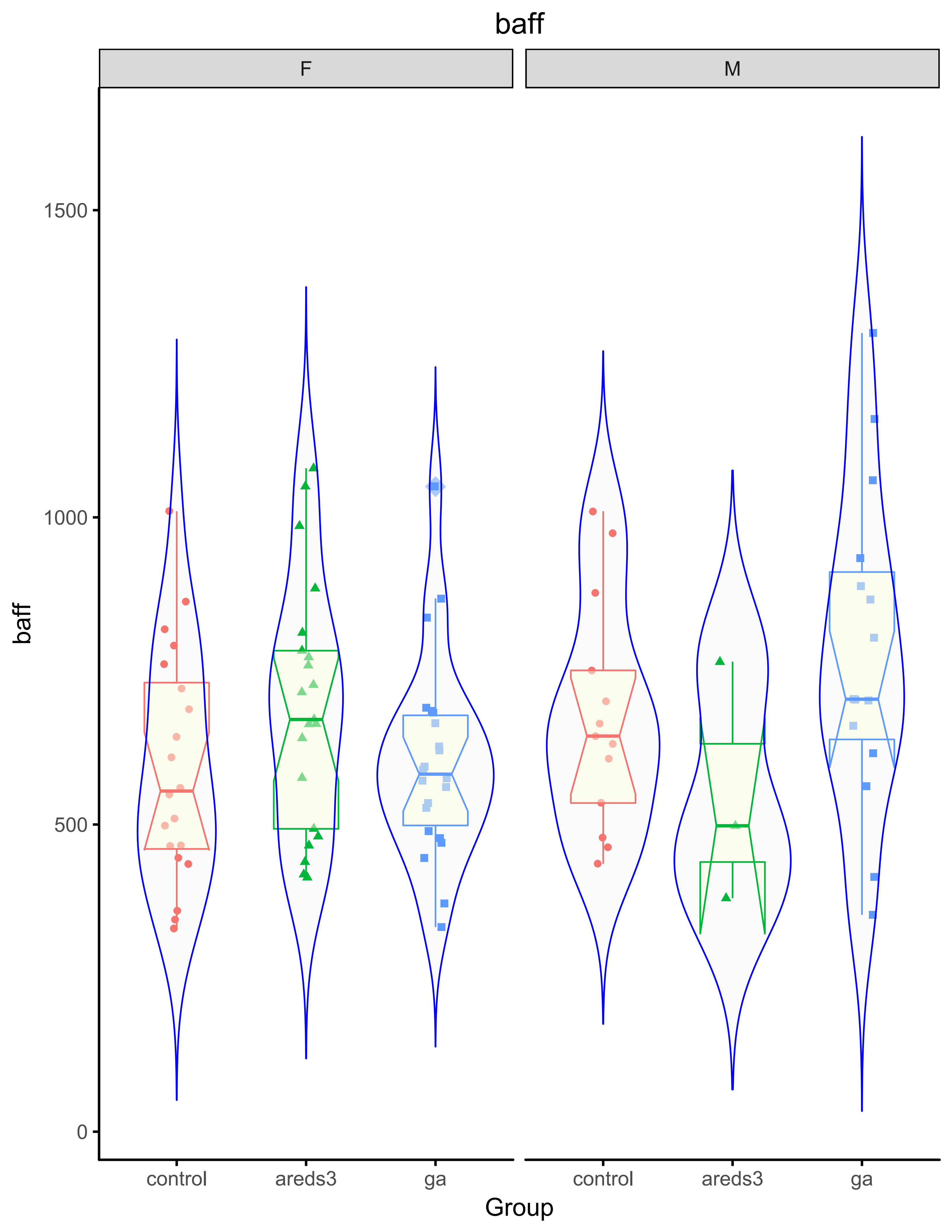

baff
F
M
1500
1000
500
0
baff
control
areds3
ga
control
areds3
ga
Group

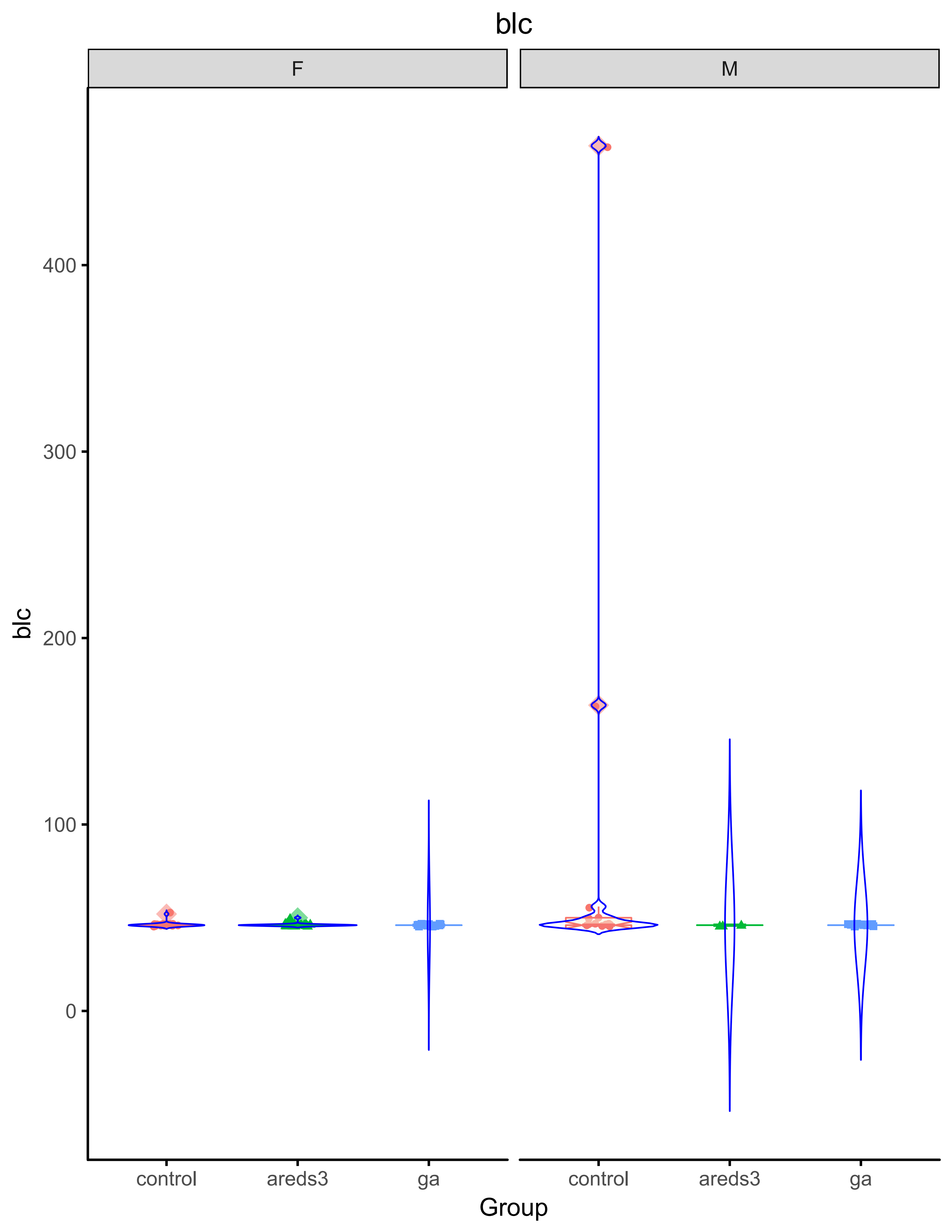

blc
F
M
400
300
200
100
0
blc
control
areds3
ga
control
areds3
ga
Group

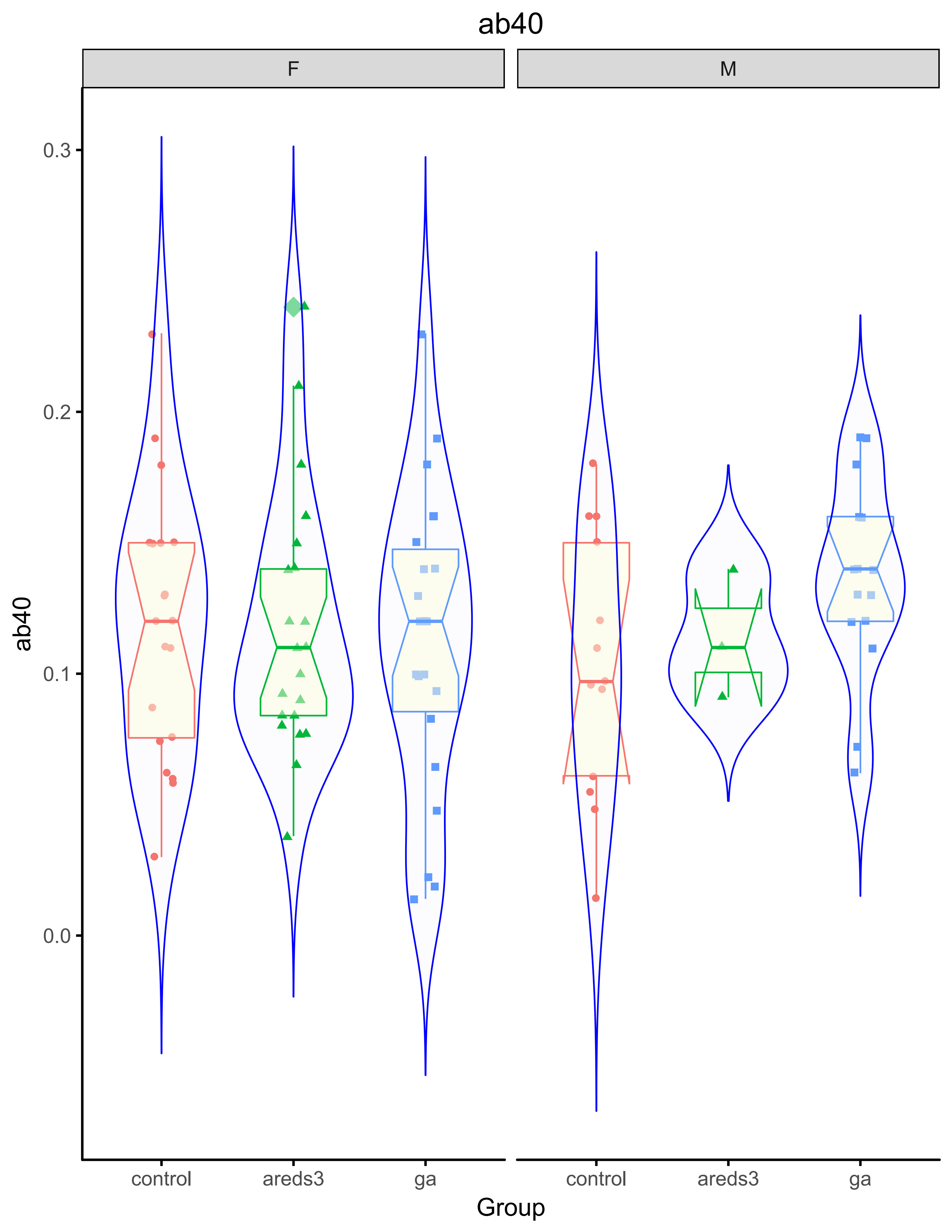
ab40
F
M
0.3
0.2
0.1
0.0
ab40
control
areds3
ga
control
areds3
ga
Group

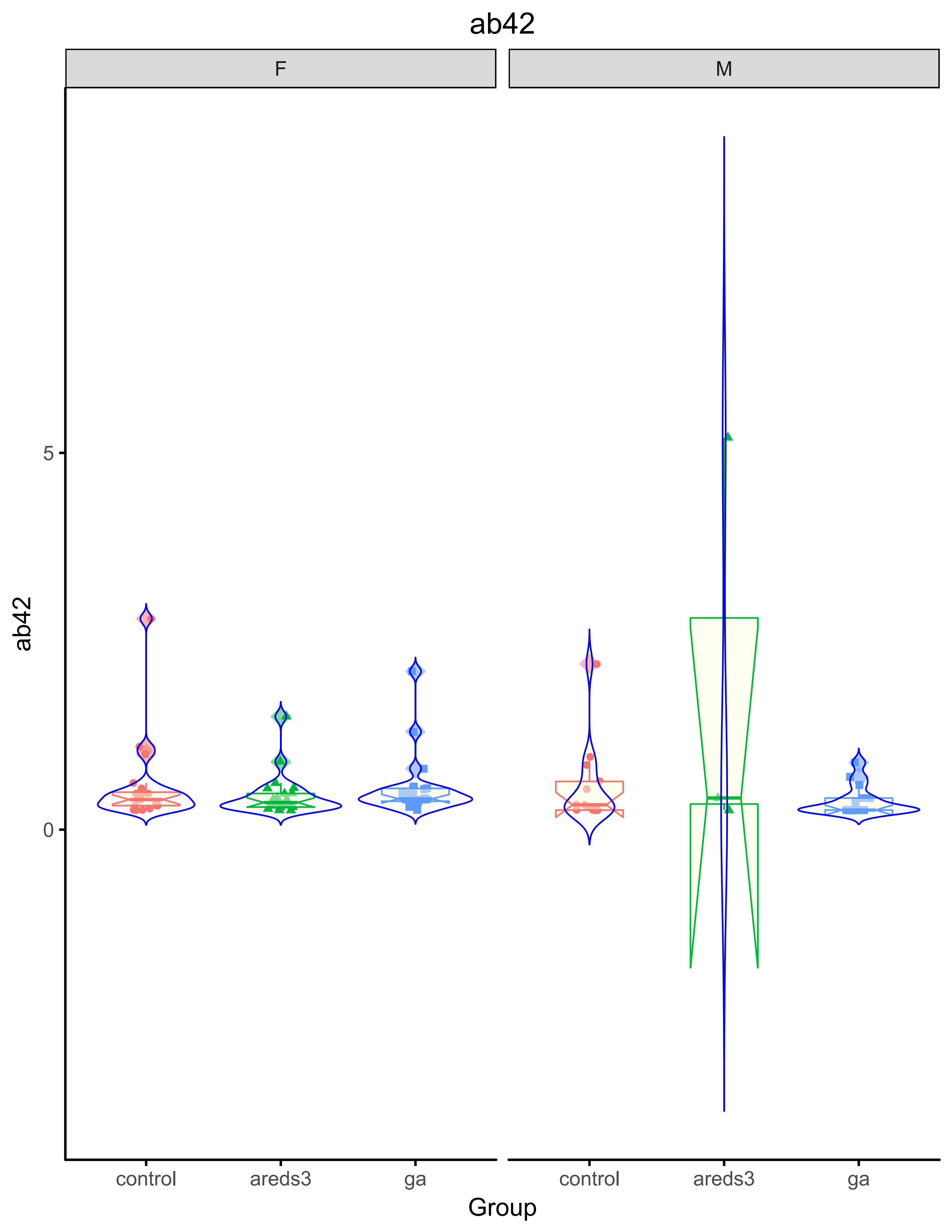

ab42
F
M
5
0
ab42
control
areds3
ga
control
areds3
ga
Group

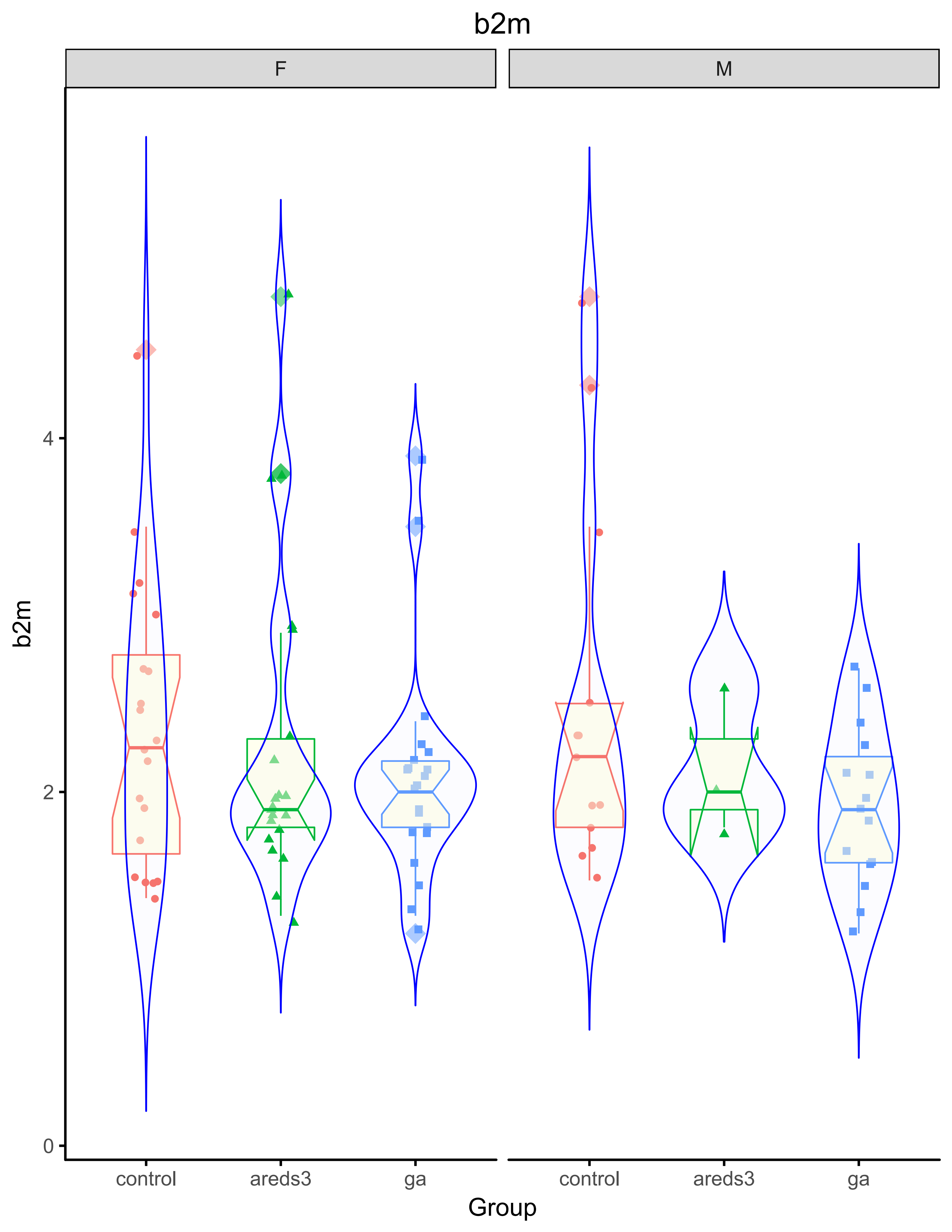

b2m
F
M
b2m
0
2
4
control
areds3
ga
control
areds3
ga
Group

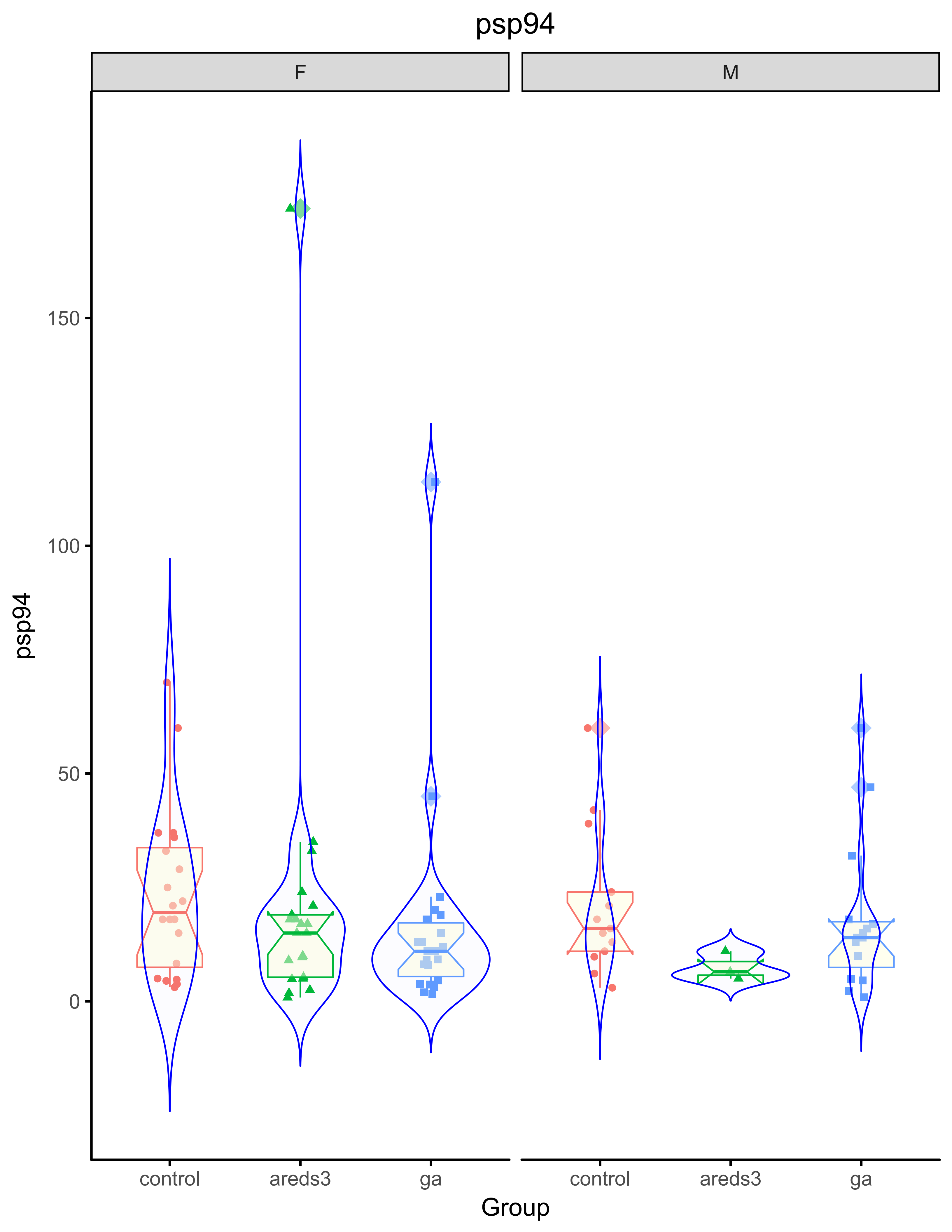

psp94
F
M
150
100
50
0
psp94
control
areds3
ga
control
areds3
ga
Group

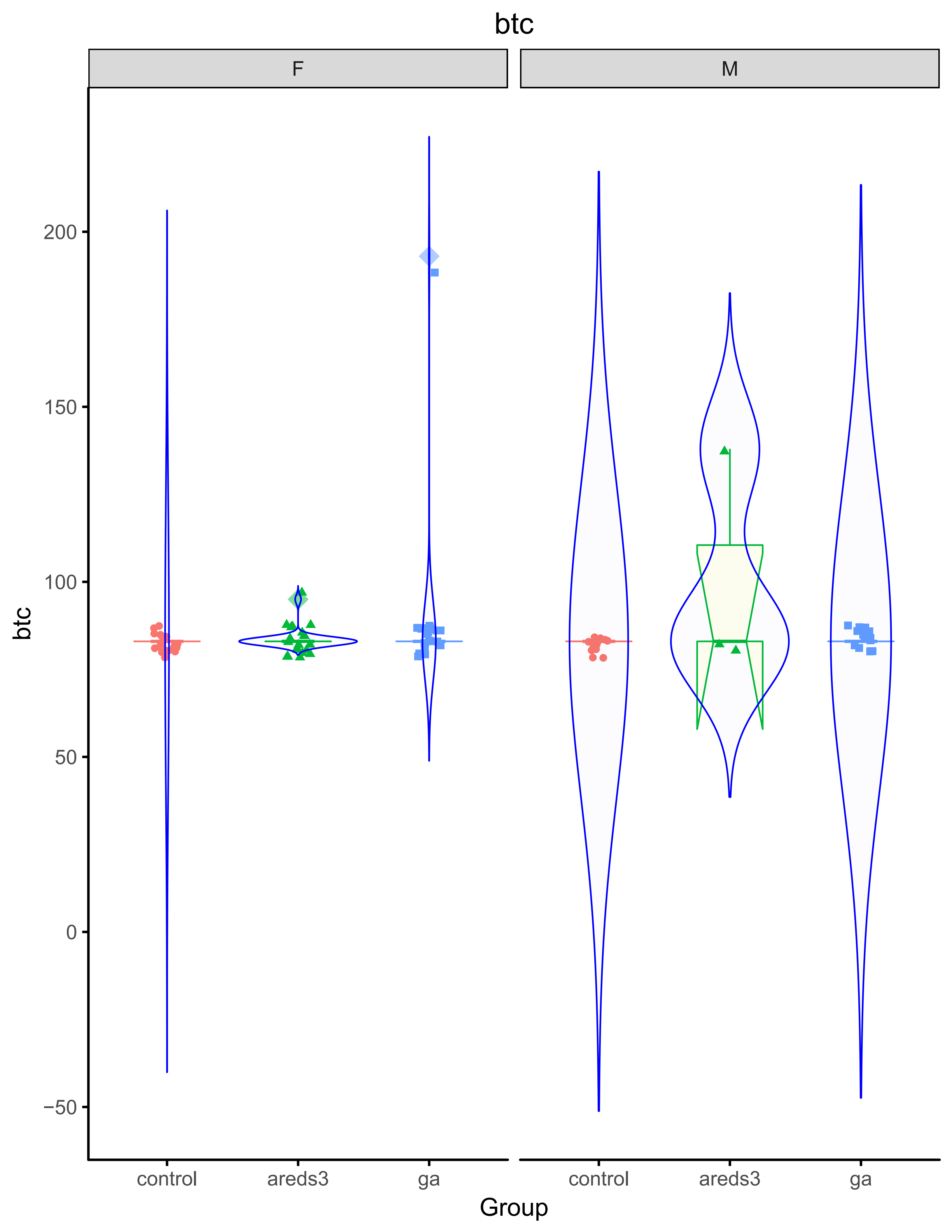
btc
F
M
200
150
100
50
0
−50
btc
control
areds3
ga
control
areds3
ga
Group

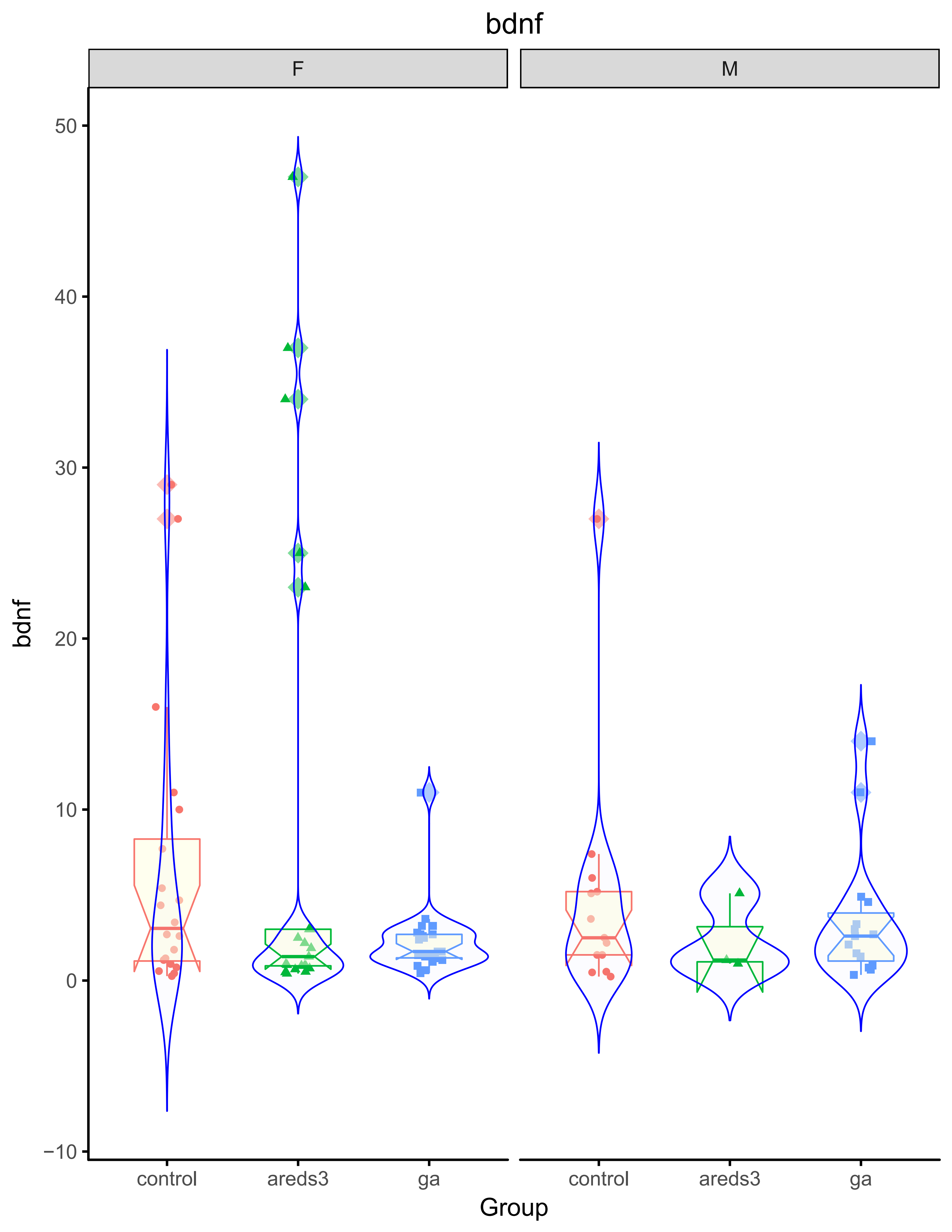

bdnf
F
M
50
40
30
20
10
0
−10
bdnf
control
areds3
ga
control
areds3
ga
Group

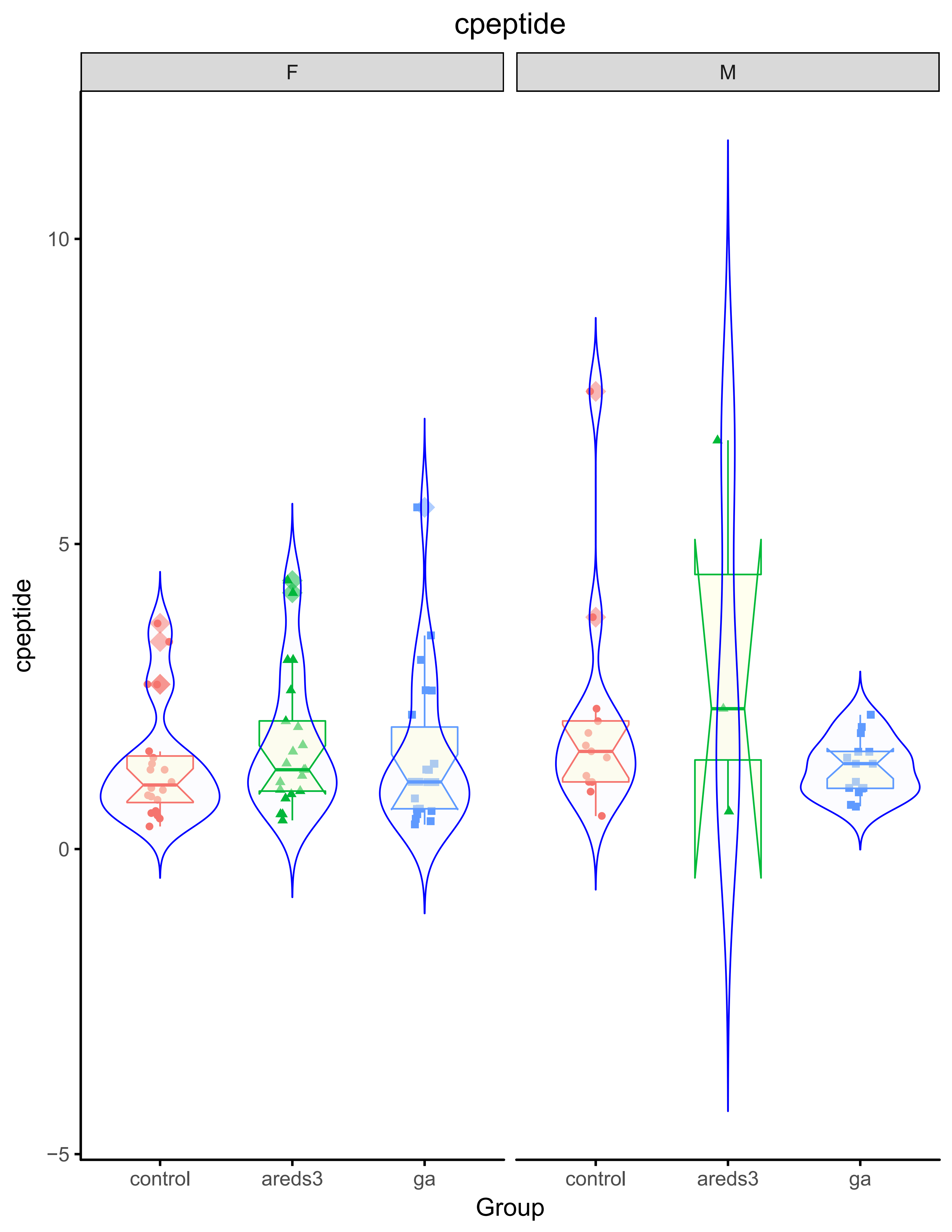
cpeptide
F
M
10
5
0
−5
cpeptide
control
areds3
ga
control
areds3
ga
Group

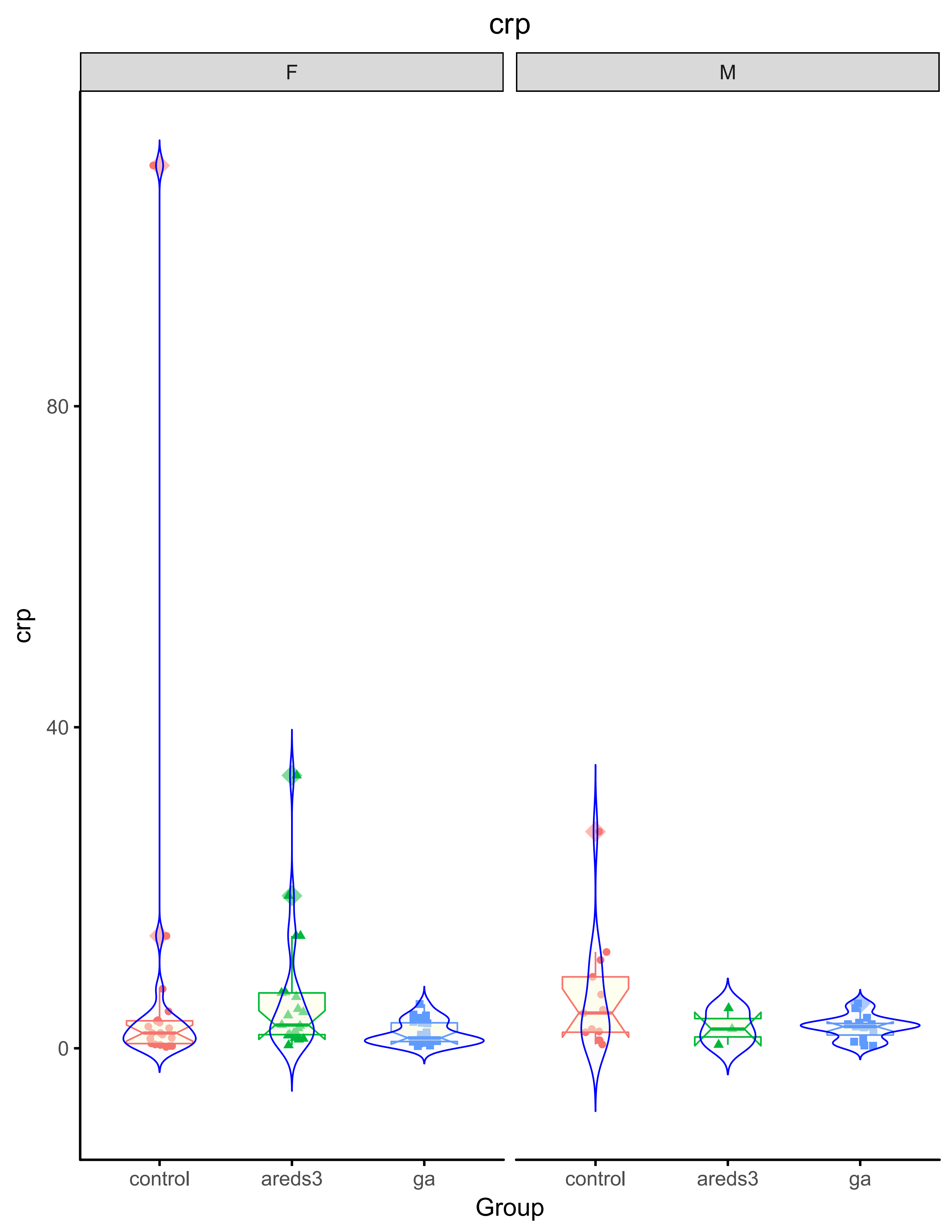

crp
F
M
80
40
0
crp
control
areds3
ga
control
areds3
ga
Group

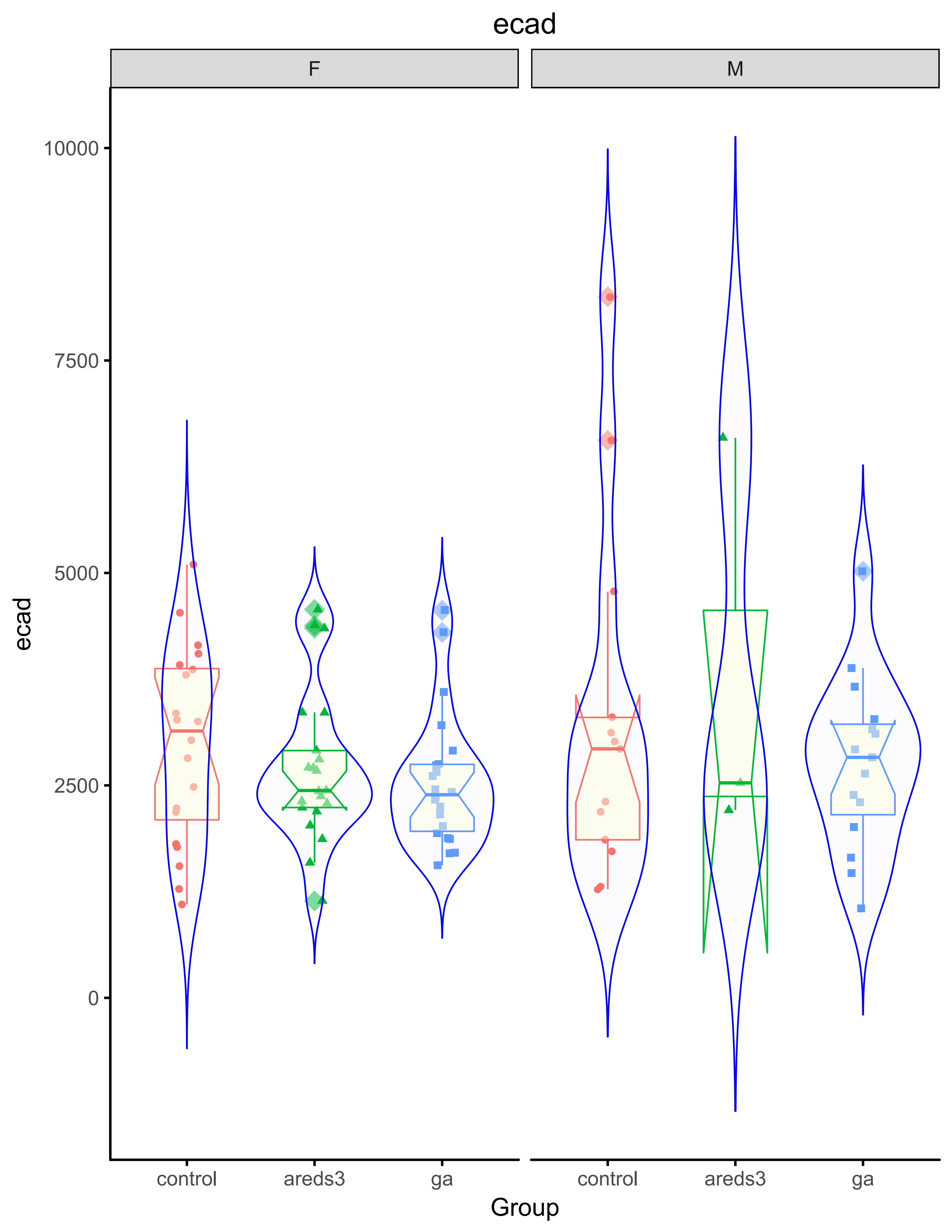
ecad
F
M
10000
7500
5000
2500
0
ecad
control
areds3
ga
control
areds3
ga
Group

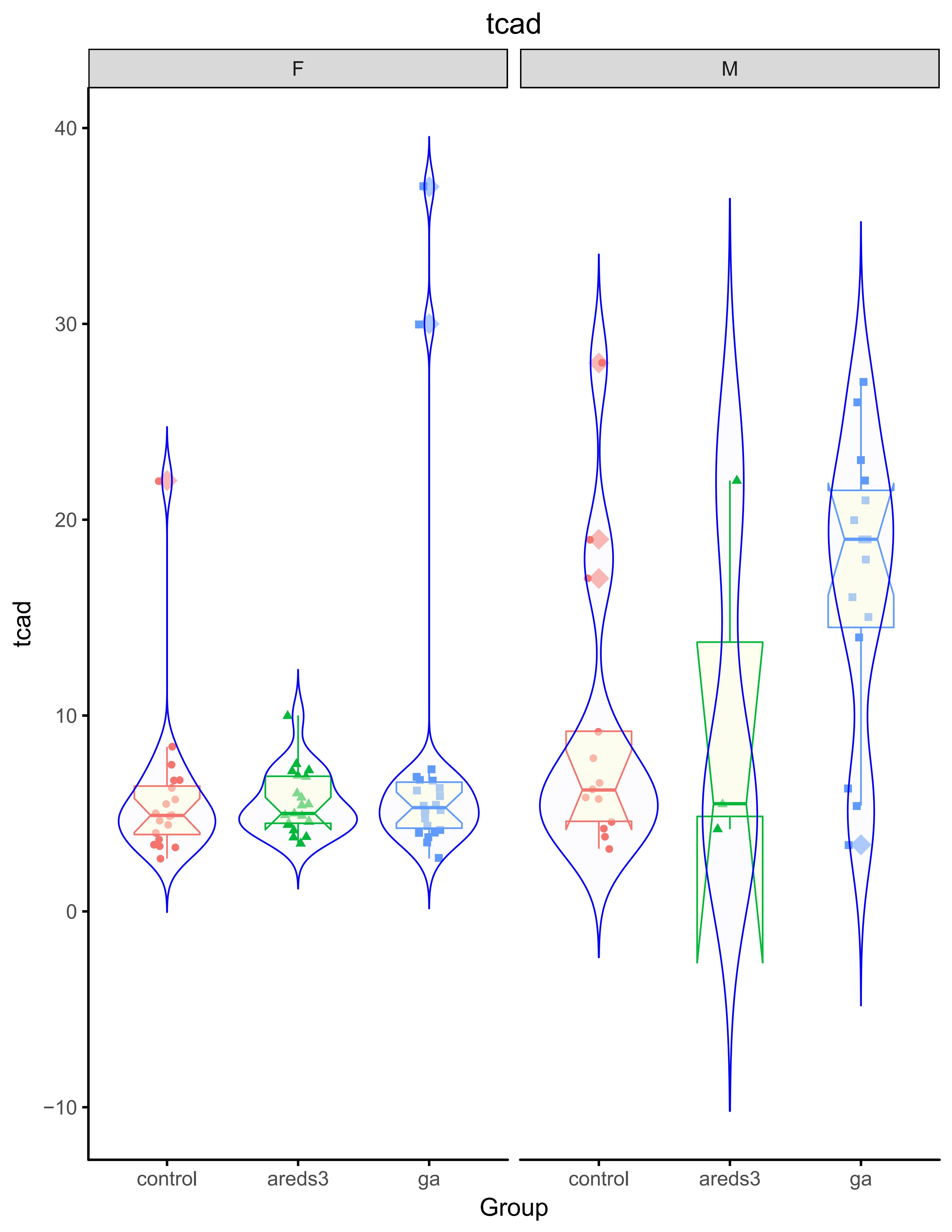

tcad
F
M
40
30
20
10
0
−10
tcad
control
areds3
ga
control
areds3
ga
Group

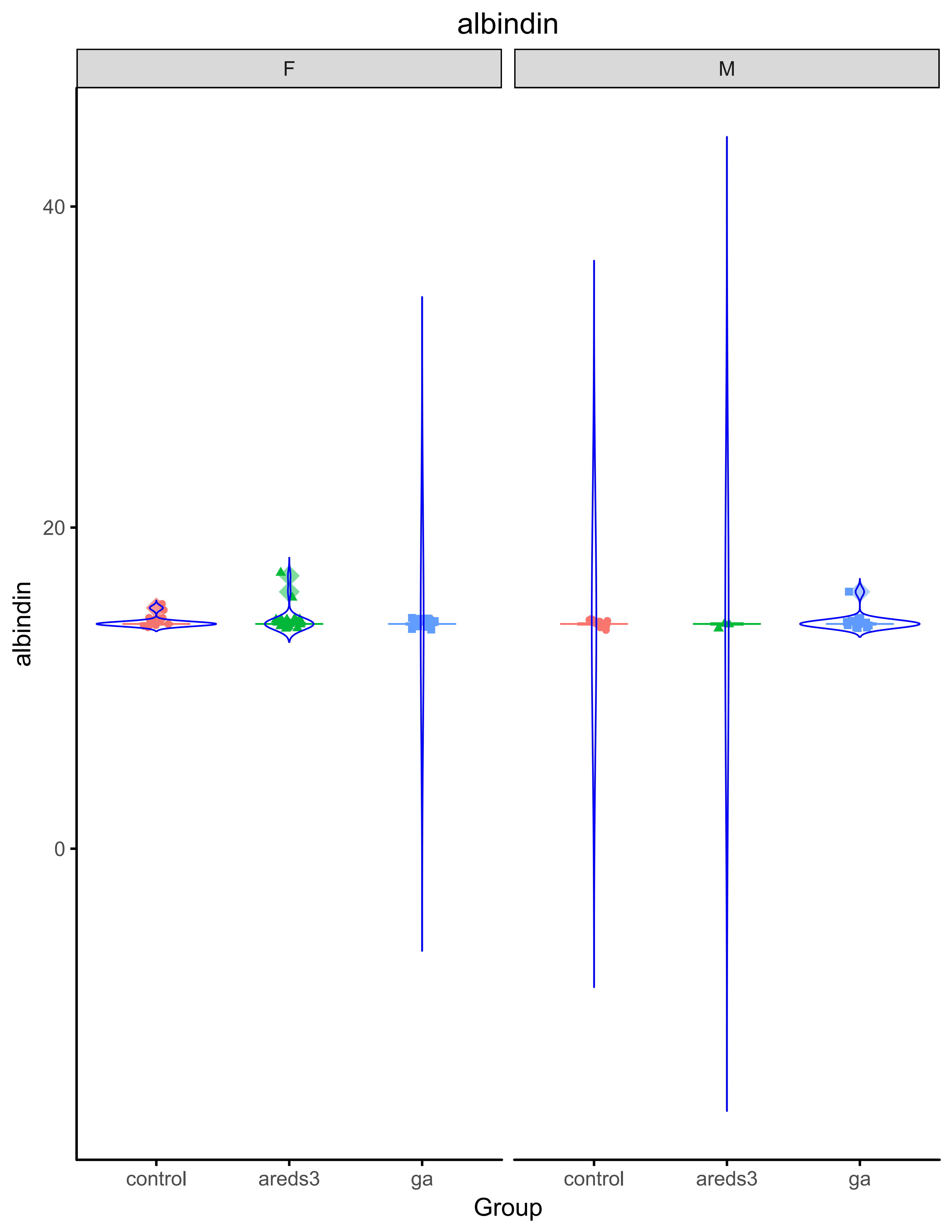

albindin
F
M
40
20
0
albindin
control
areds3
ga
control
areds3
ga
Group

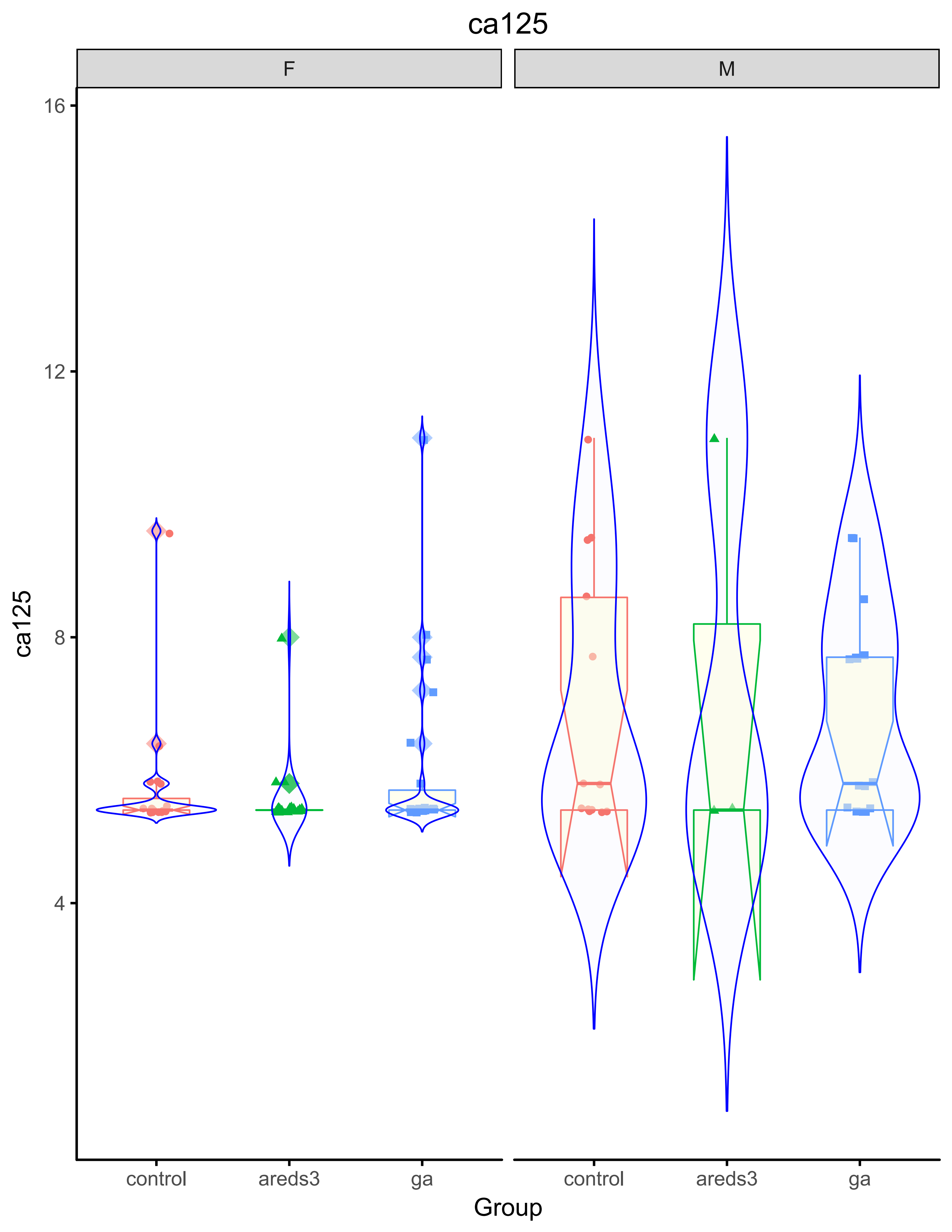

ca125
F
M
16
12
8
4
ca125
control
areds3
ga
control
areds3
ga
Group

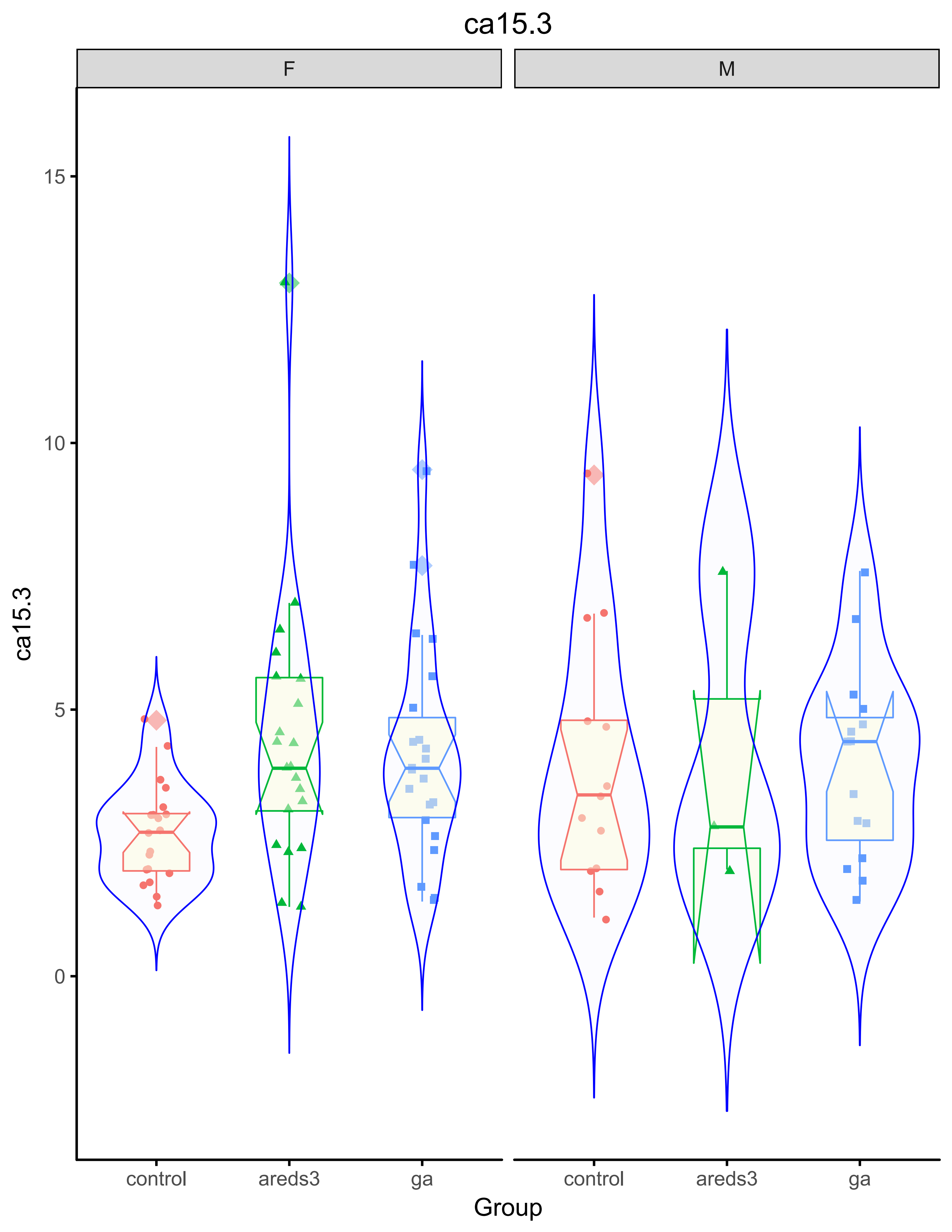

ca15.3
F
M
15
10
5
0
ca15.3
control
areds3
ga
control
areds3
ga
Group

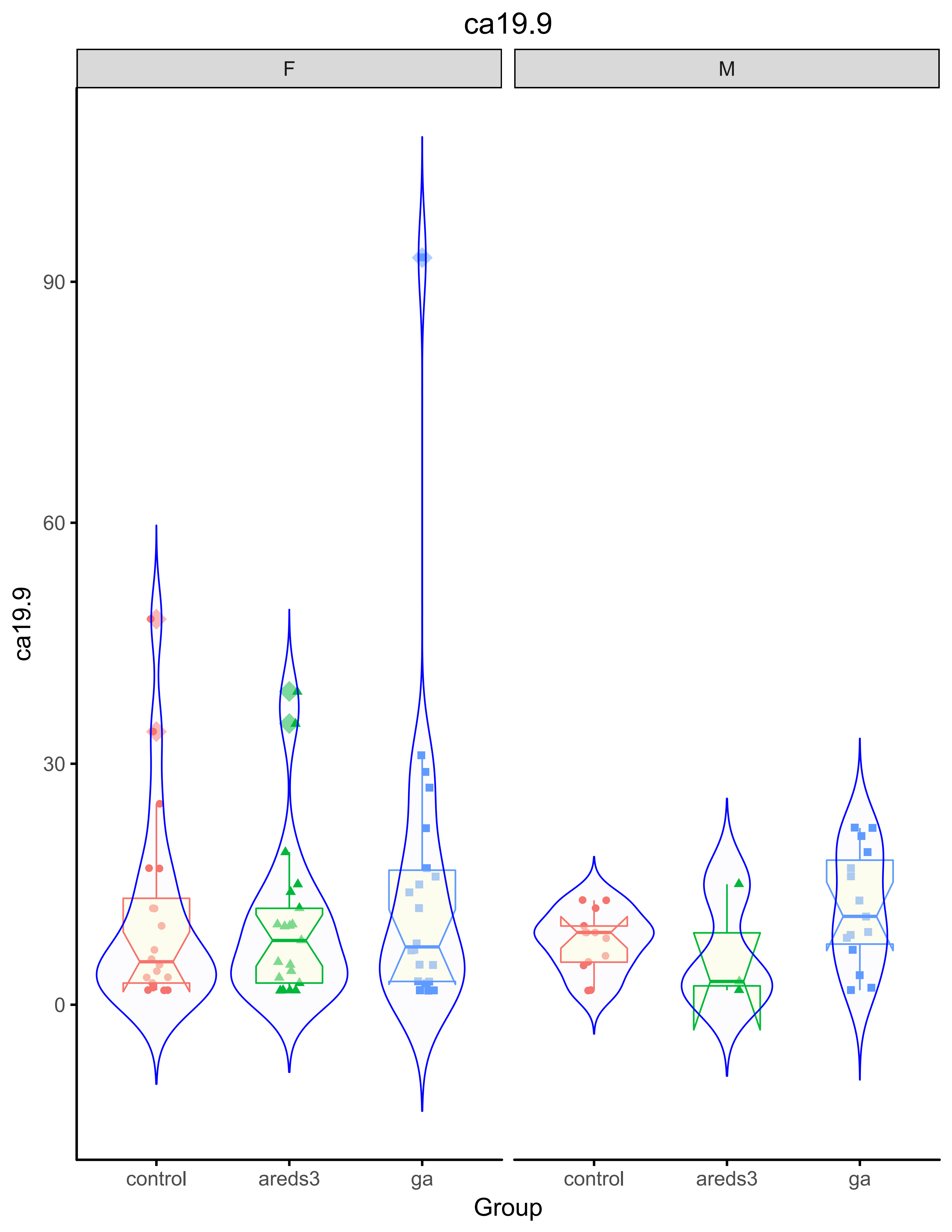
ca19.9
F
M
90
60
30
0
ca19.9
control
areds3
ga
control
areds3
ga
Group

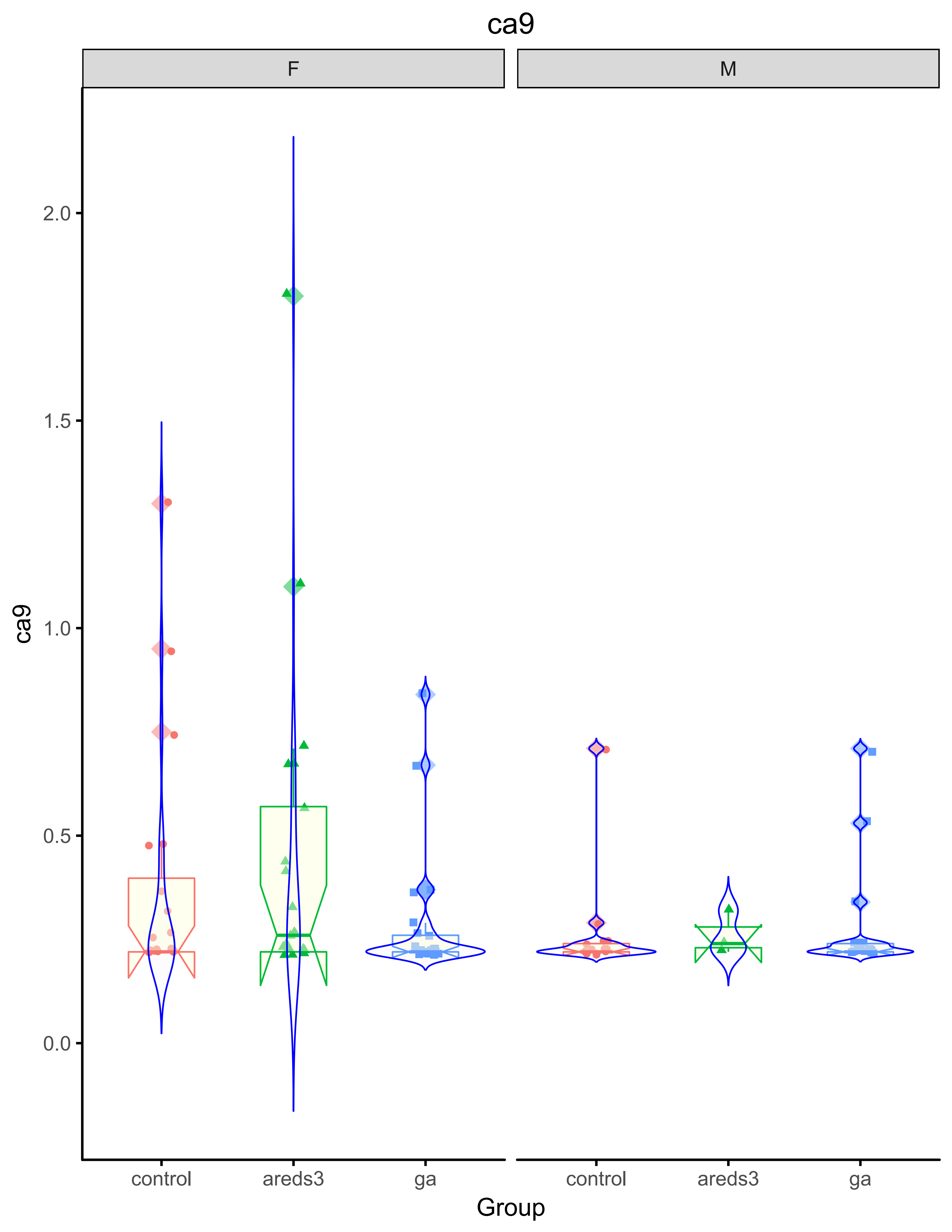

ca9
F
M
ca9
2.0
1.5
1.0
0.5
0.0
control
areds3
ga
control
areds3
ga
Group

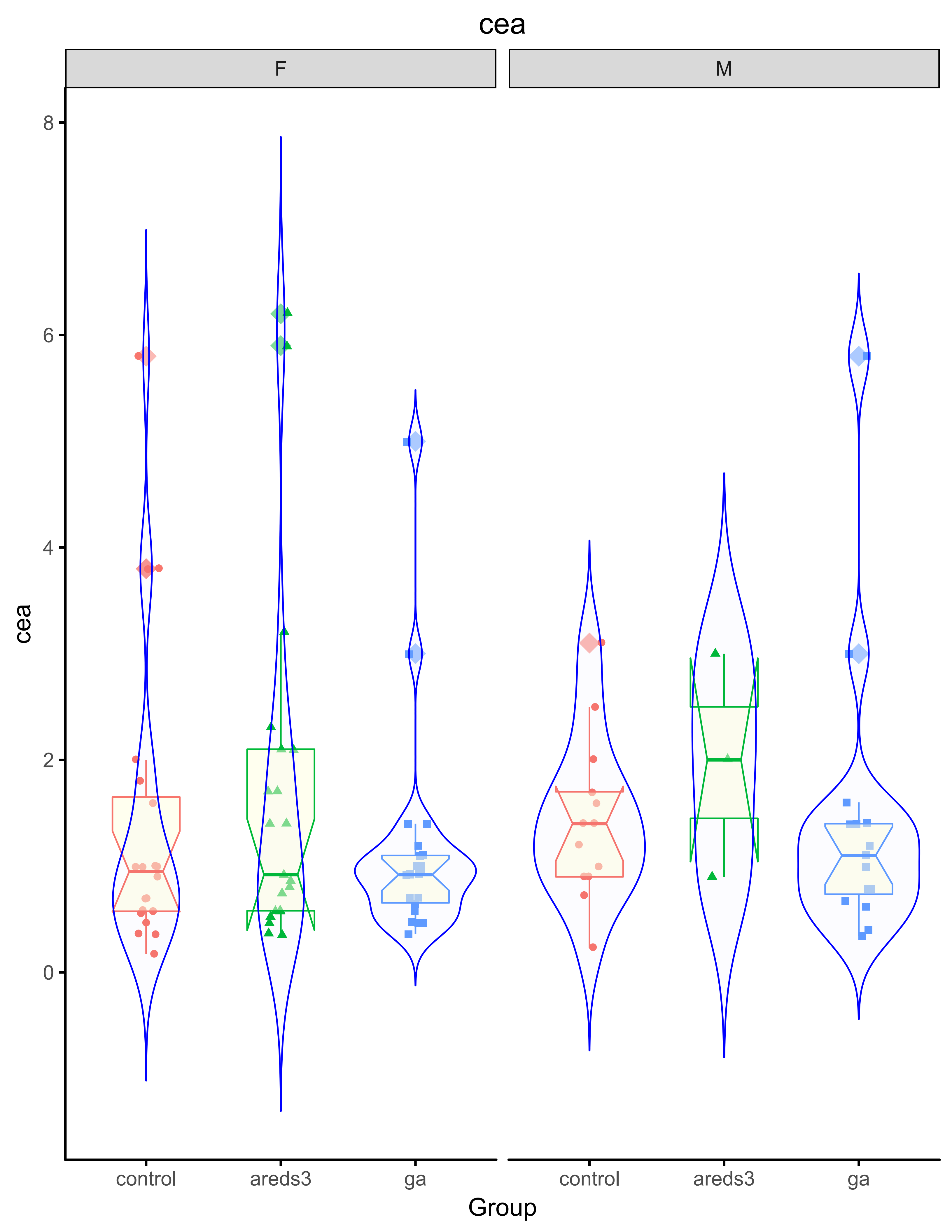
cea
F
M
cea
0
2
4
6
8
control
areds3
ga
control
areds3
ga
Group

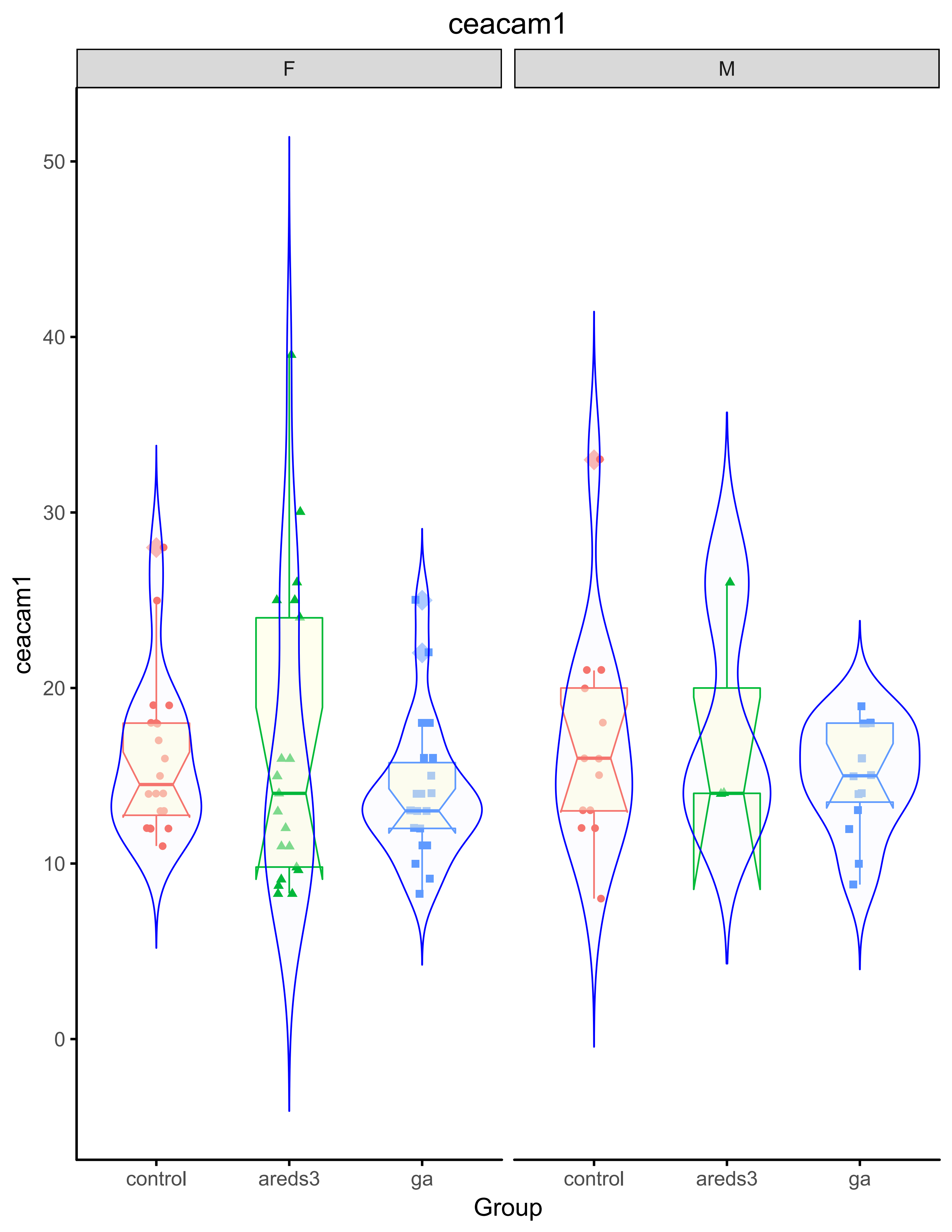

ceacam1
F
M
50
40
30
20
10
0
ceacam1
control
areds3
ga
control
areds3
ga
Group

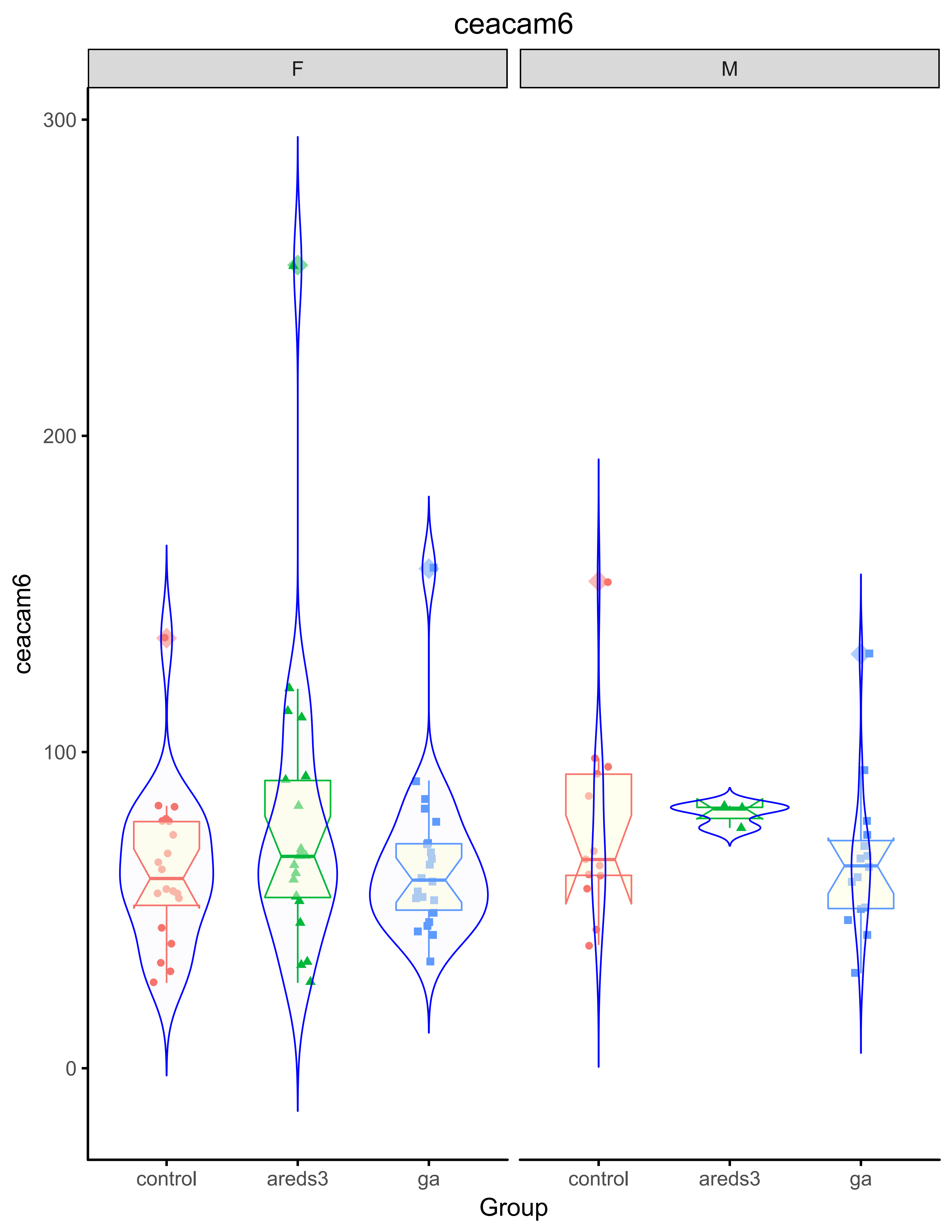
ceacam6
F
M
300
200
100
0
ceacam6
control
areds3
ga
control
areds3
ga
Group

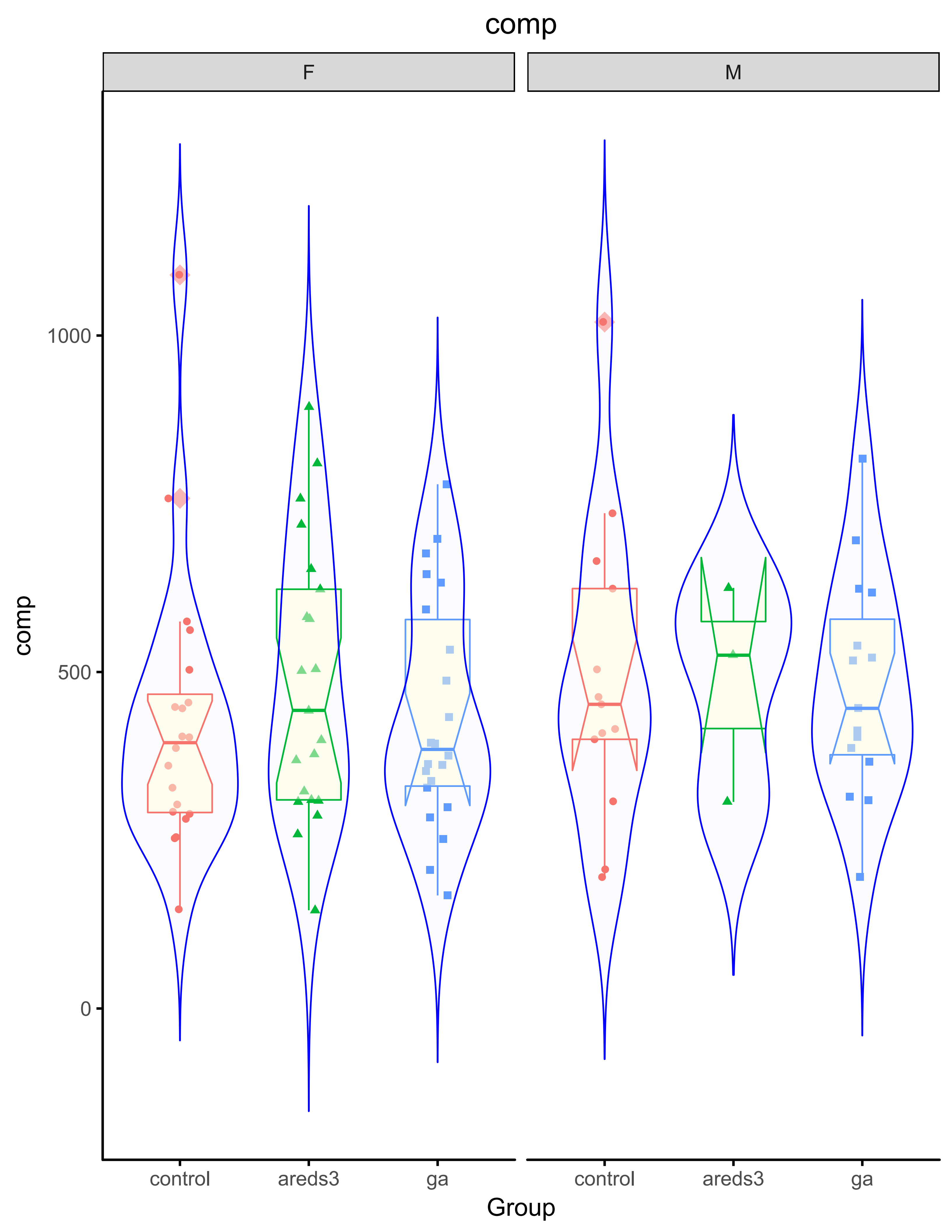

comp
F
M
1000
500
0
comp
control
areds3
ga
control
areds3
ga
Group

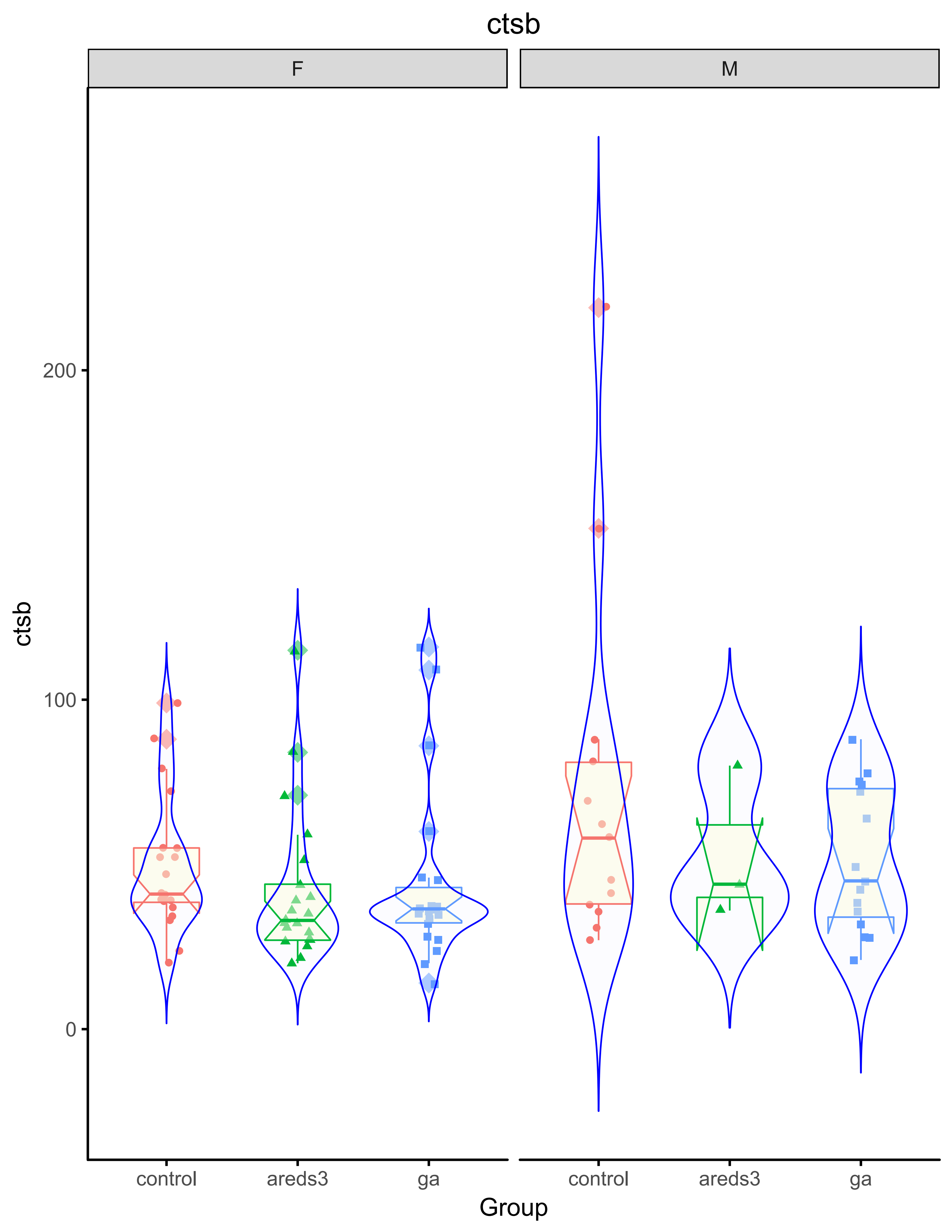

ctsb
F
M
200
100
0
ctsb
control
areds3
ga
control
areds3
ga
Group

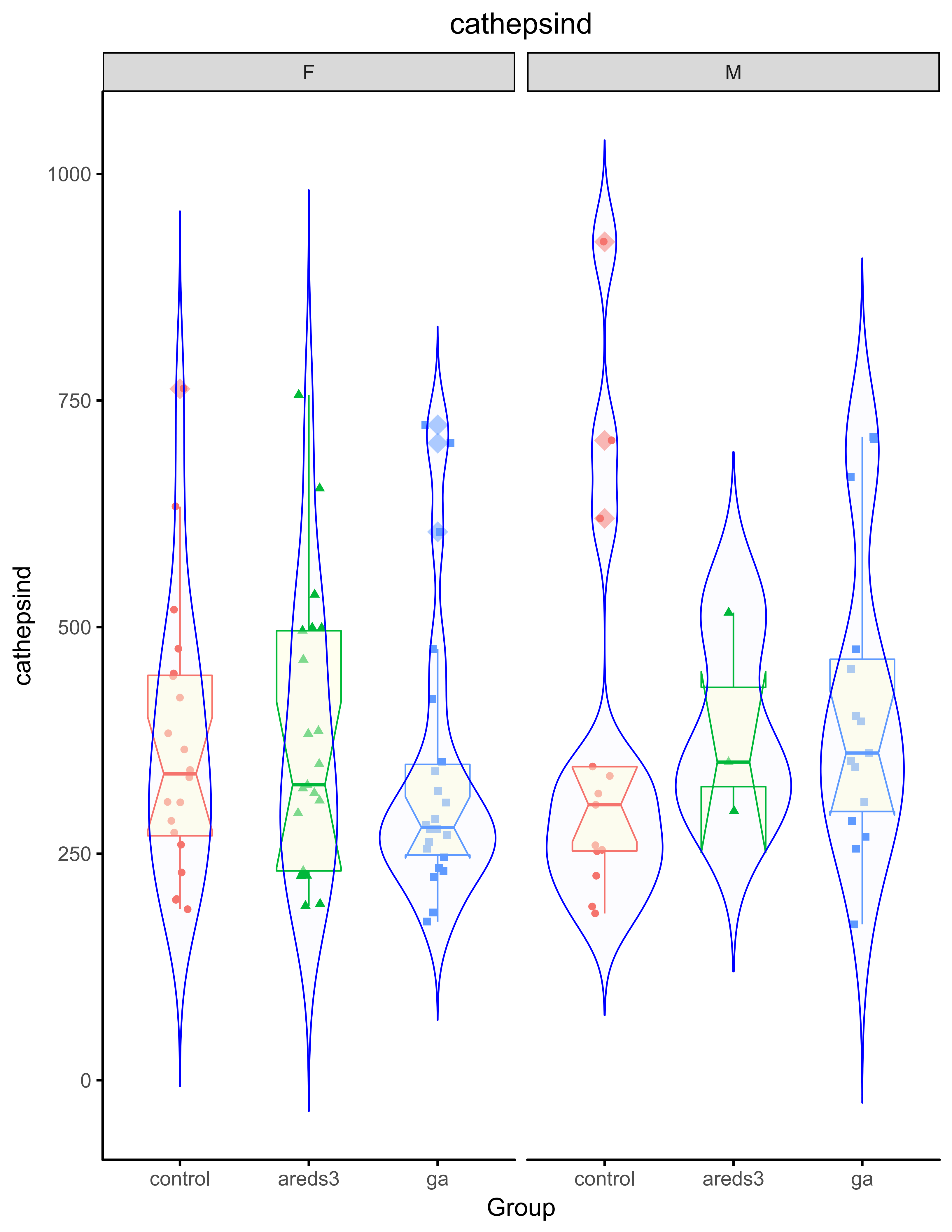
cathepsind
F
M
1000
750
500
250
0
cathepsind
control
areds3
ga
control
areds3
ga
Group

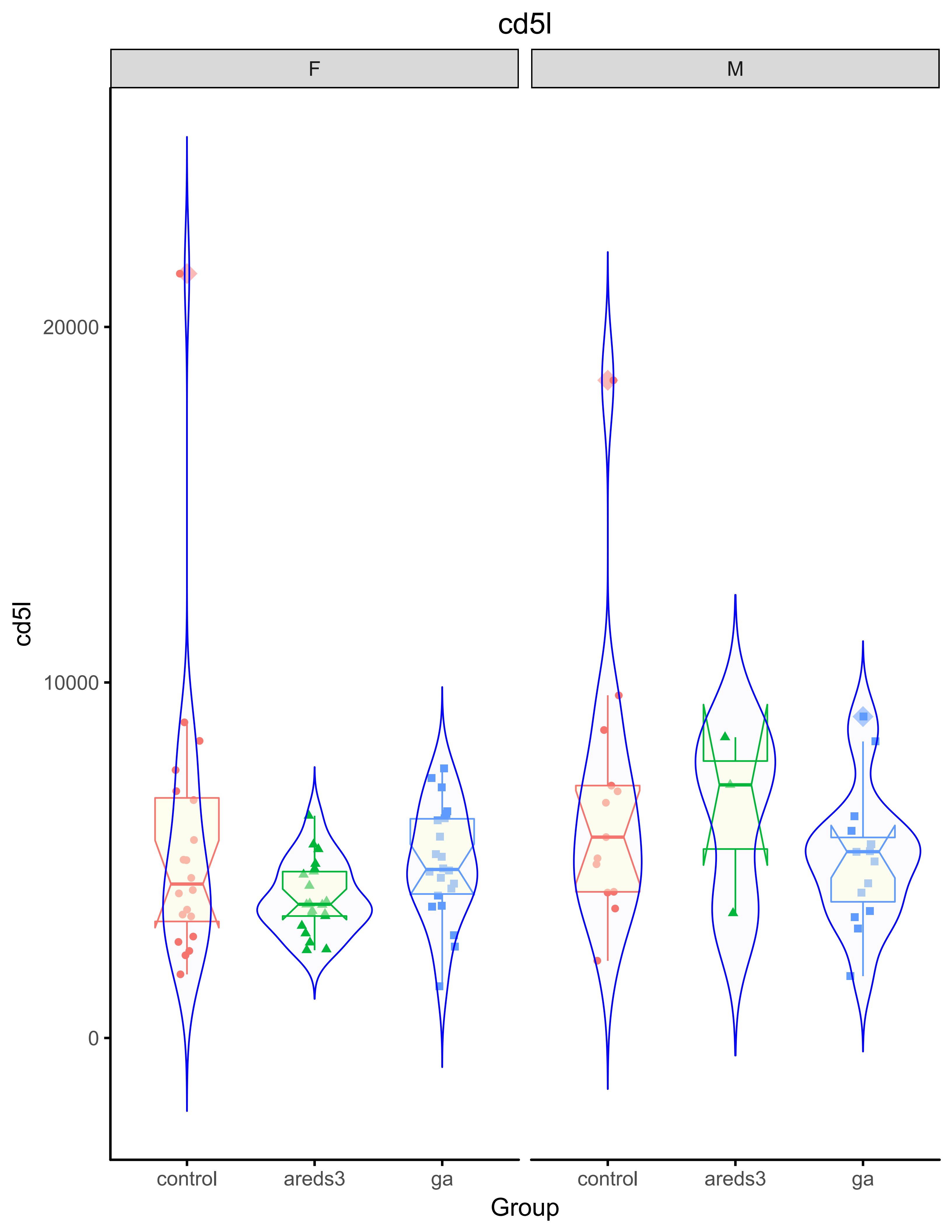

cd5l
F
M
20000
10000
0
cd5l
control
areds3
ga
control
areds3
ga
Group

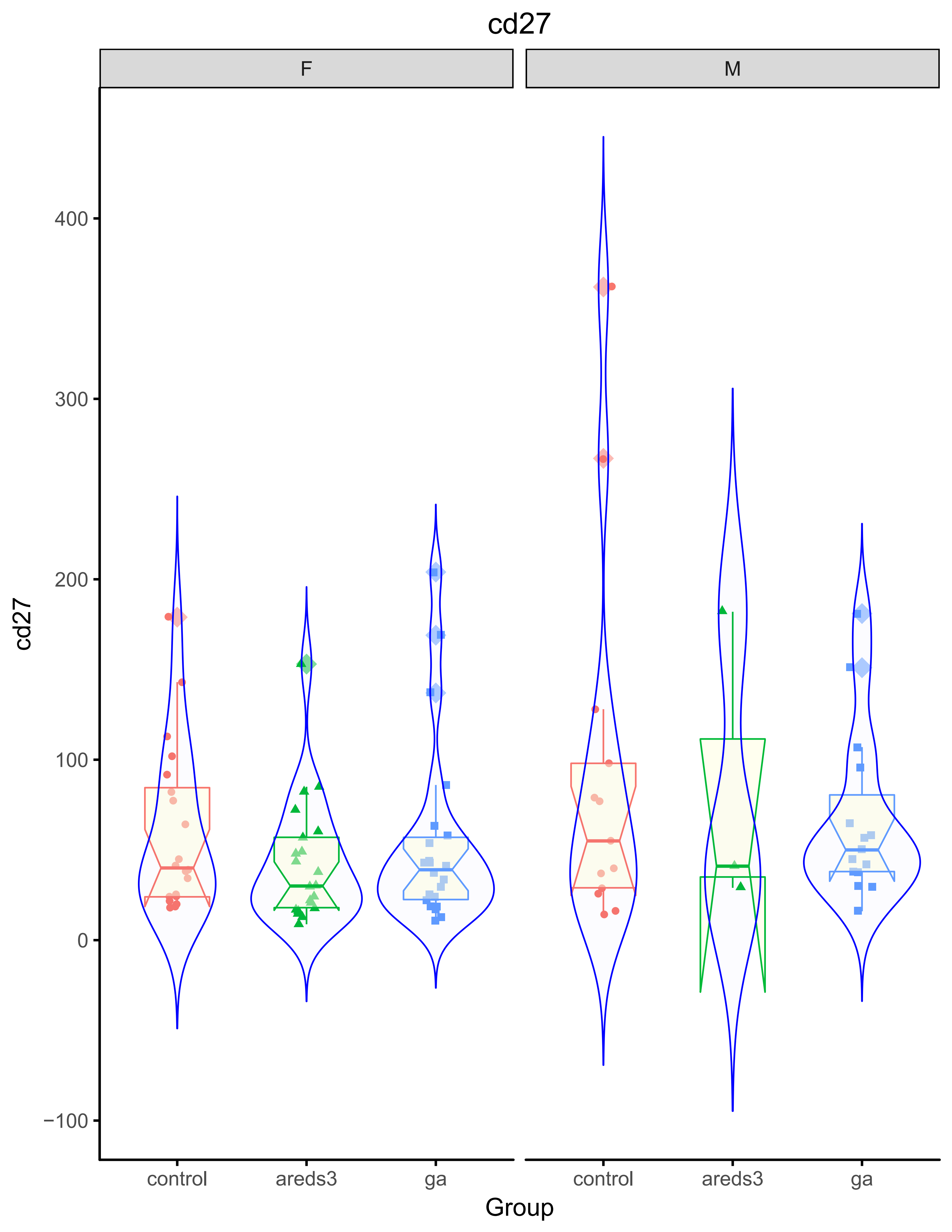

cd27
F
M
cd27
400
300
200
100
0
−100
control
areds3
ga
control
areds3
ga
Group

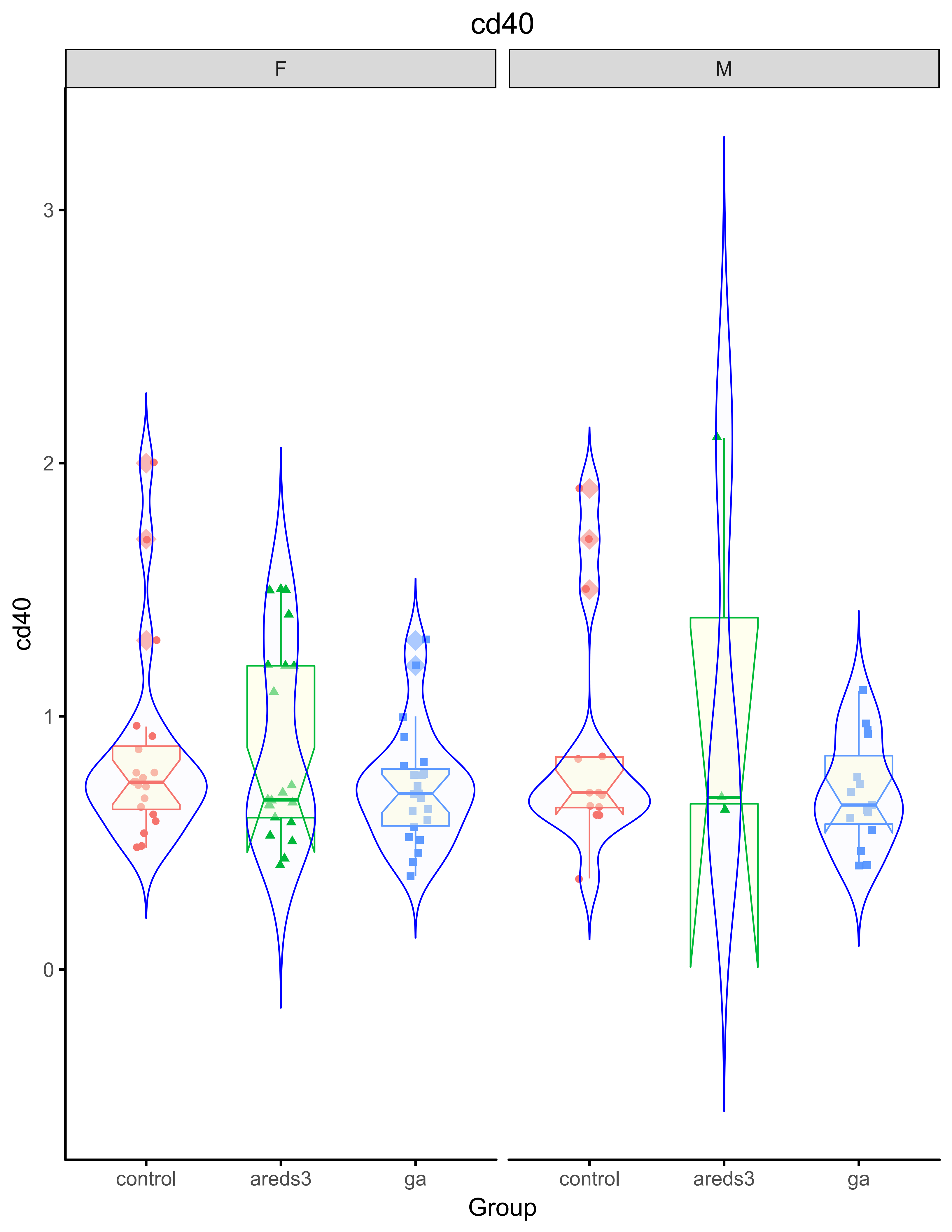

cd40
F
M
3
2
1
0
cd40
control
areds3
ga
control
areds3
ga
Group

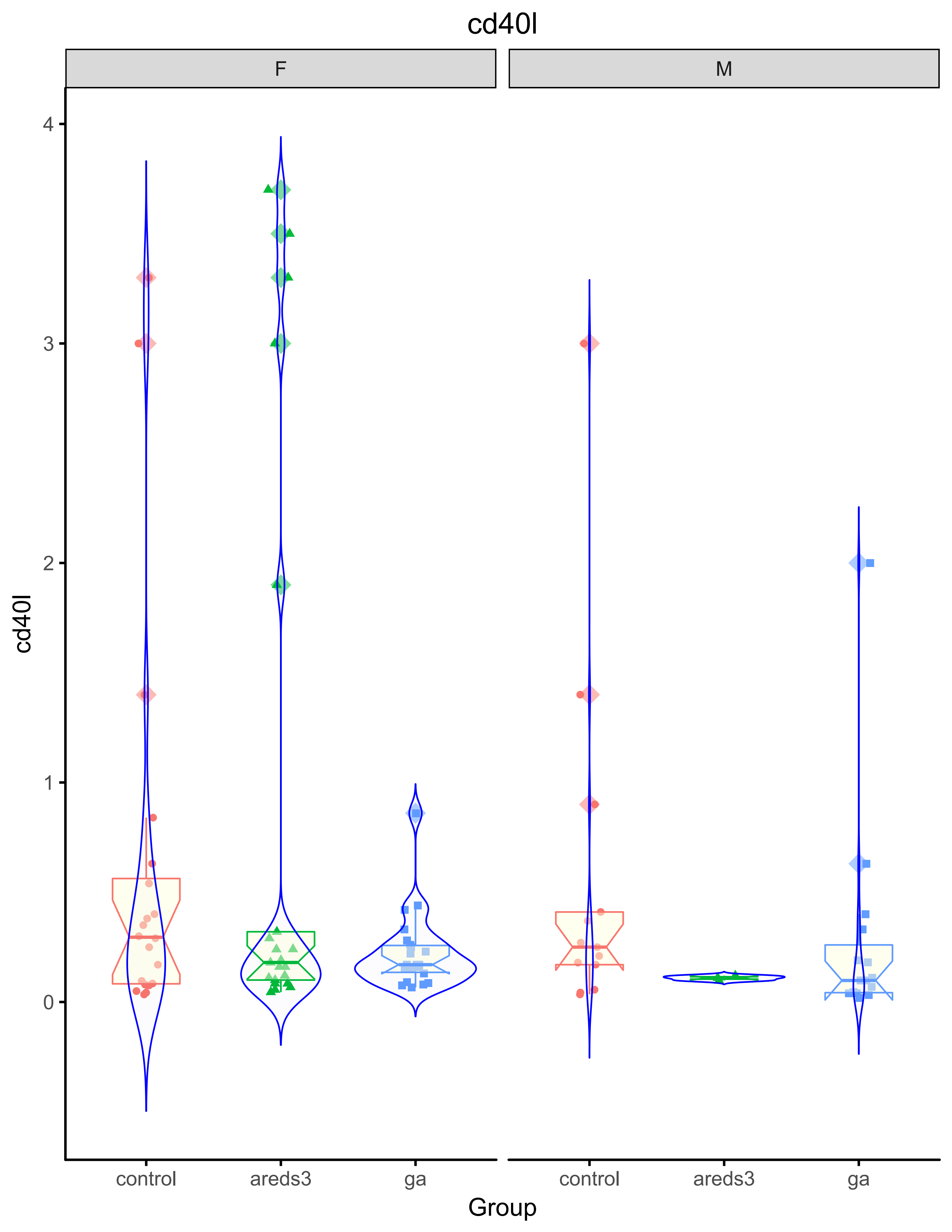

cd40l
F
M
4
3
2
1
0
cd40l
control
areds3
ga
control
areds3
ga
Group

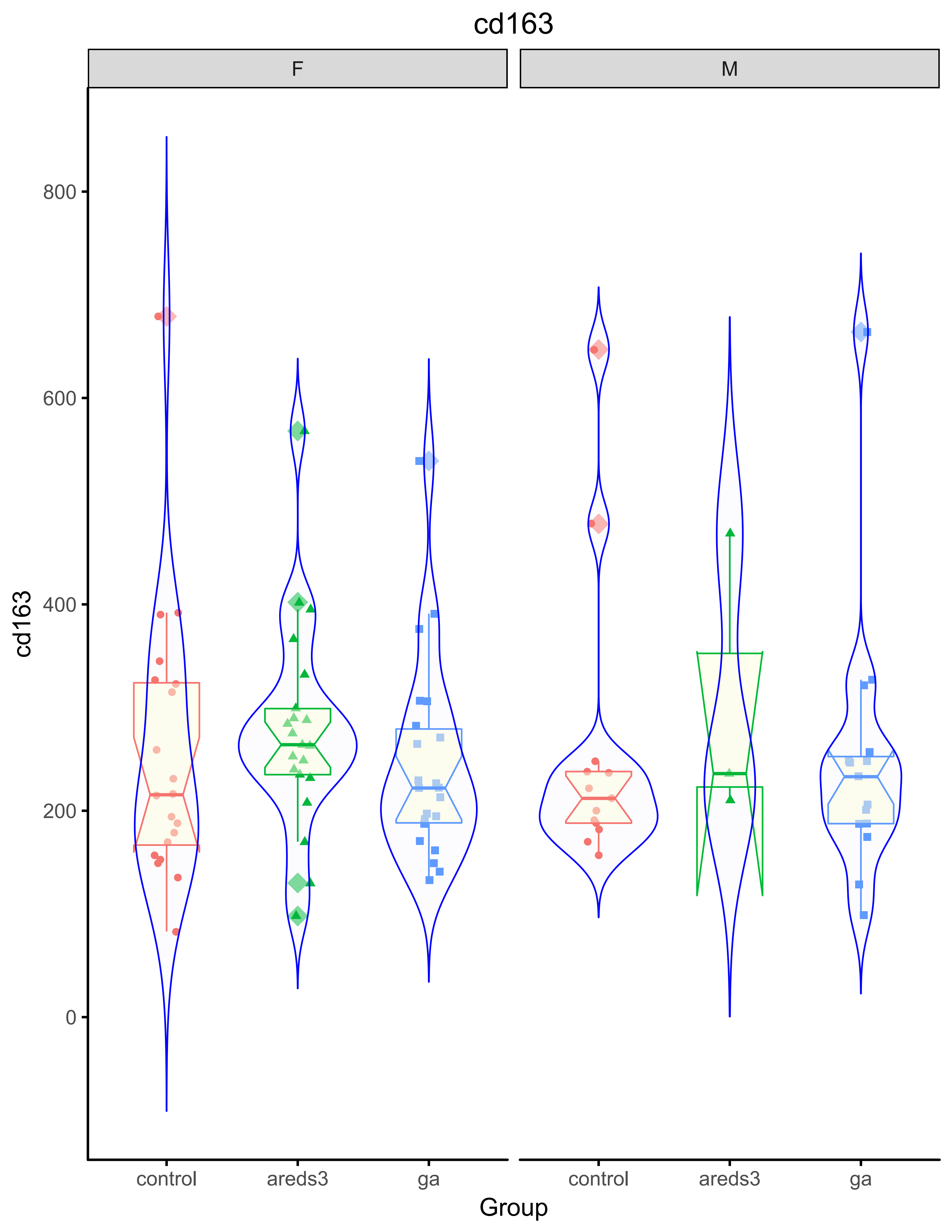

cd163
F
M
800
600
400
200
0
cd163
control
areds3
ga
control
areds3
ga
Group

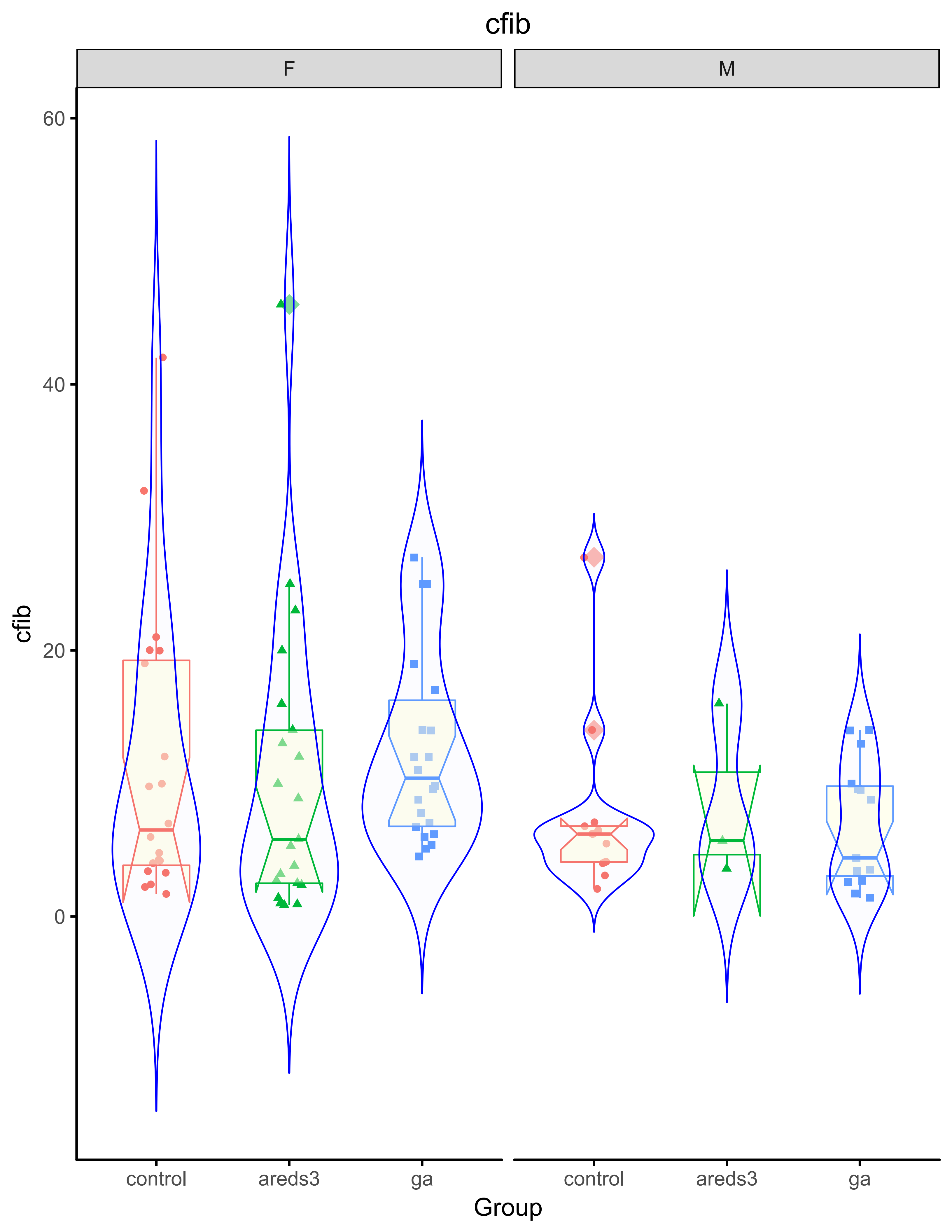
cfib
F
M
60
40
20
0
cfib
control
areds3
ga
control
areds3
ga
Group

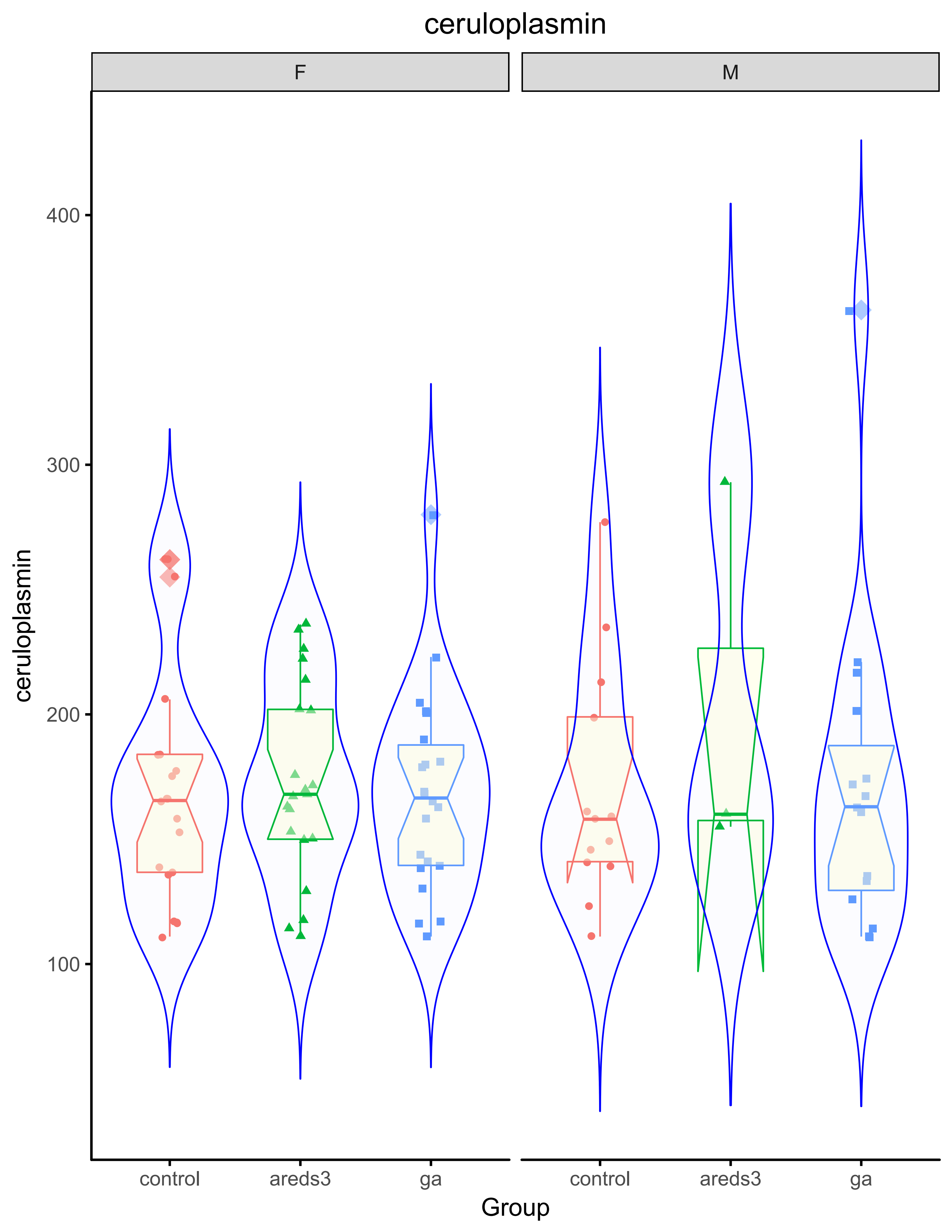

ceruloplasmin
F
M
ceruloplasmin
400
300
200
100
control
areds3
ga
control
areds3
ga
Group

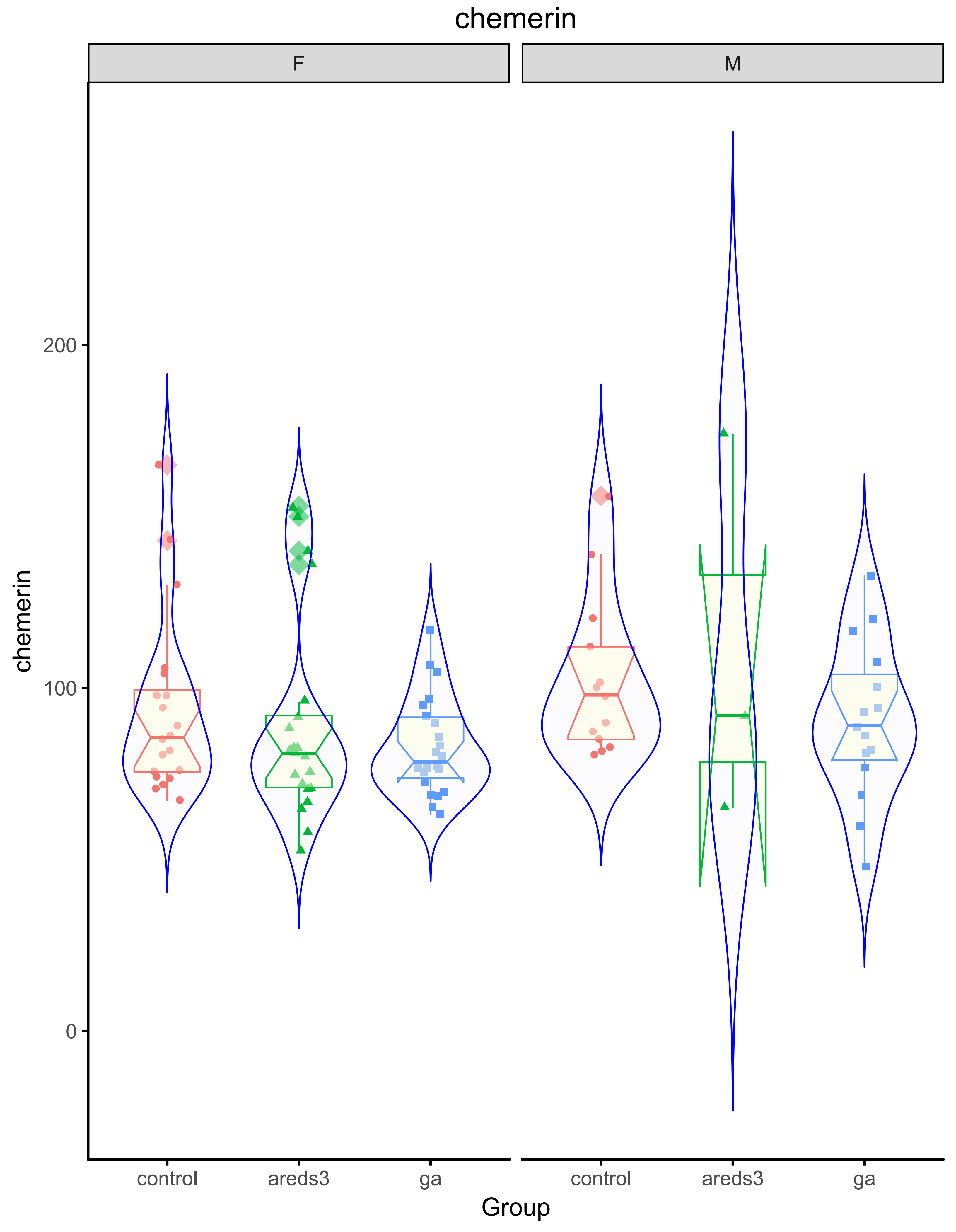

chemerin
F
M
200
100
0
chemerin
control
areds3
ga
control
areds3
ga
Group

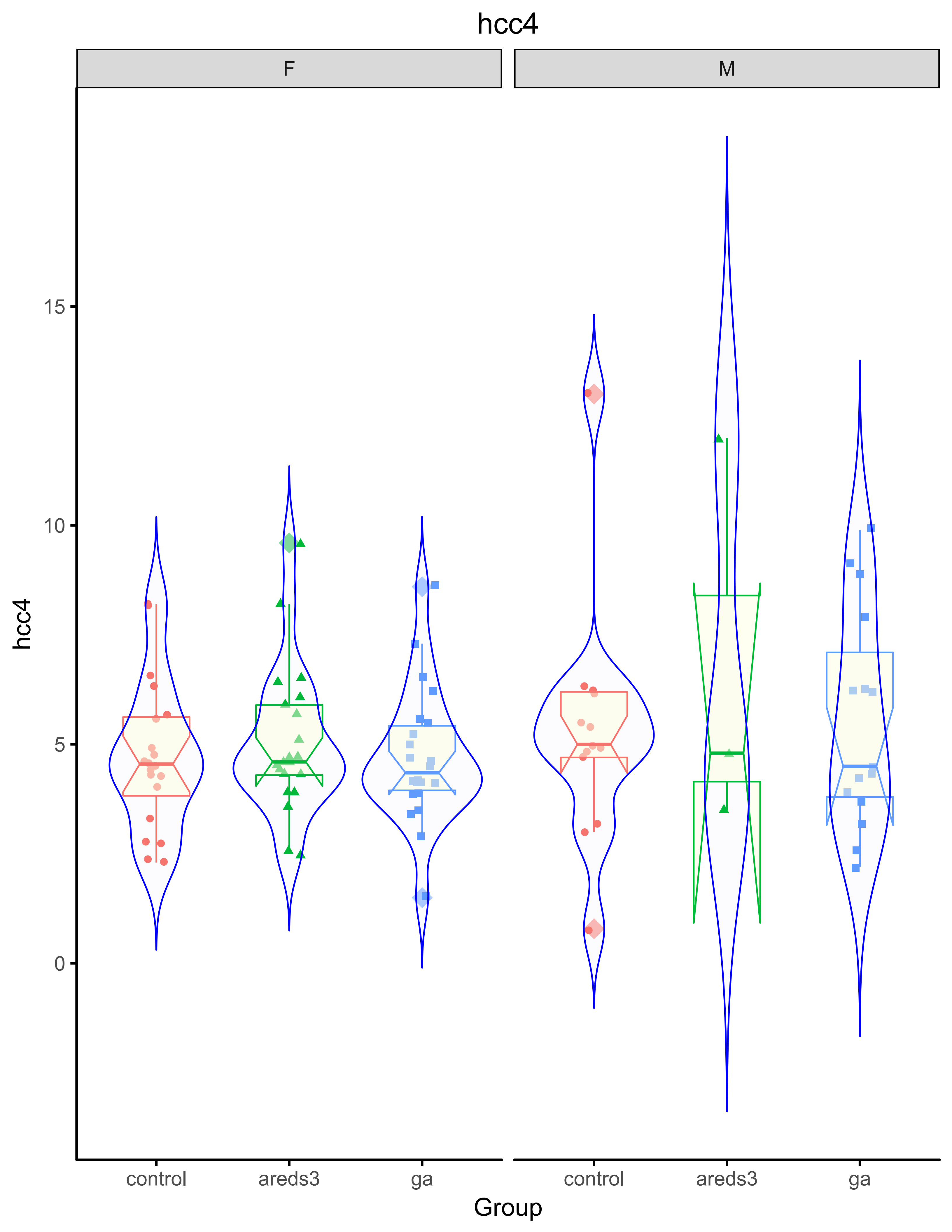

hcc4
F
M
hcc4
15
10
5
0
control
areds3
ga
control
areds3
ga
Group

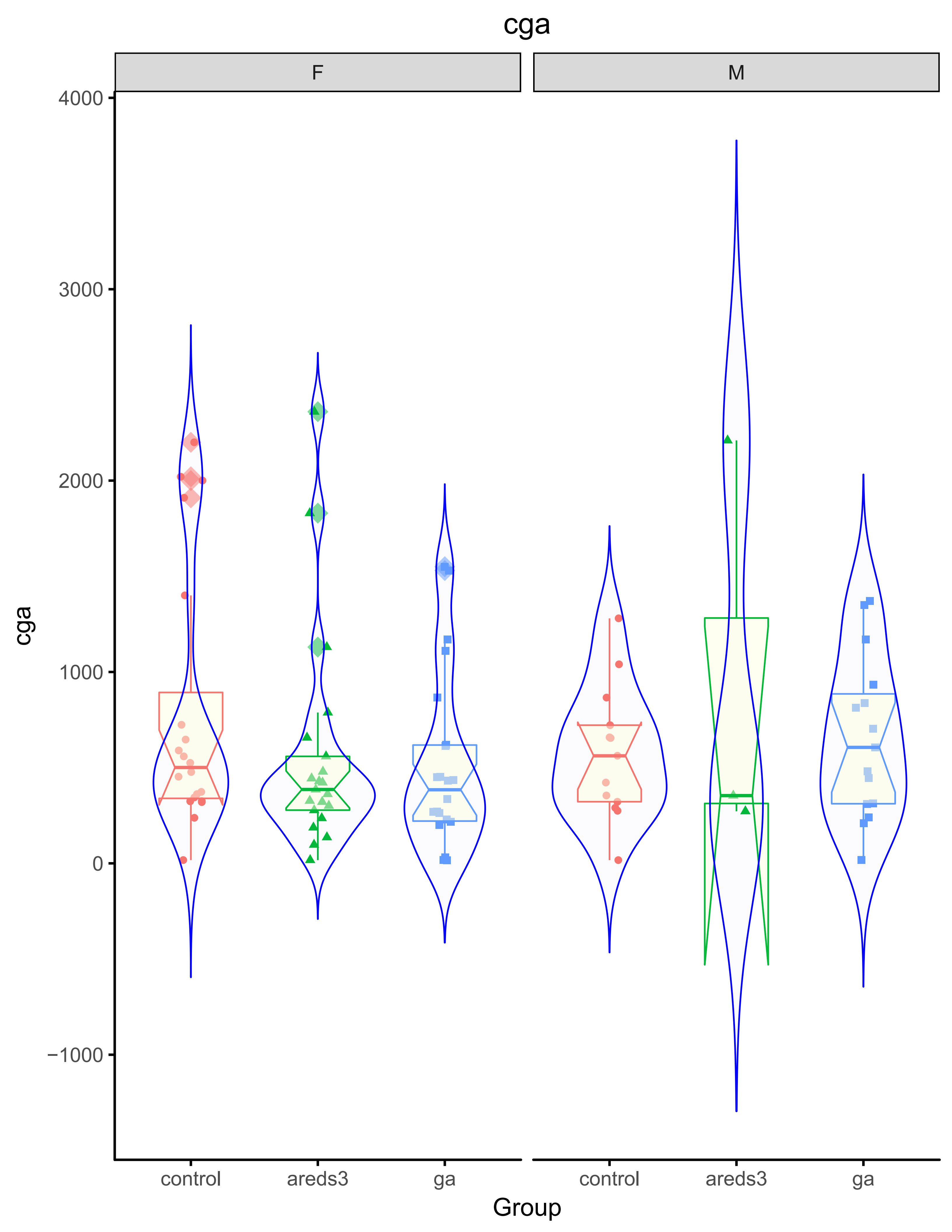

cga
F
M
4000
3000
2000
1000
0
−1000
cga
control
areds3
ga
control
areds3
ga
Group

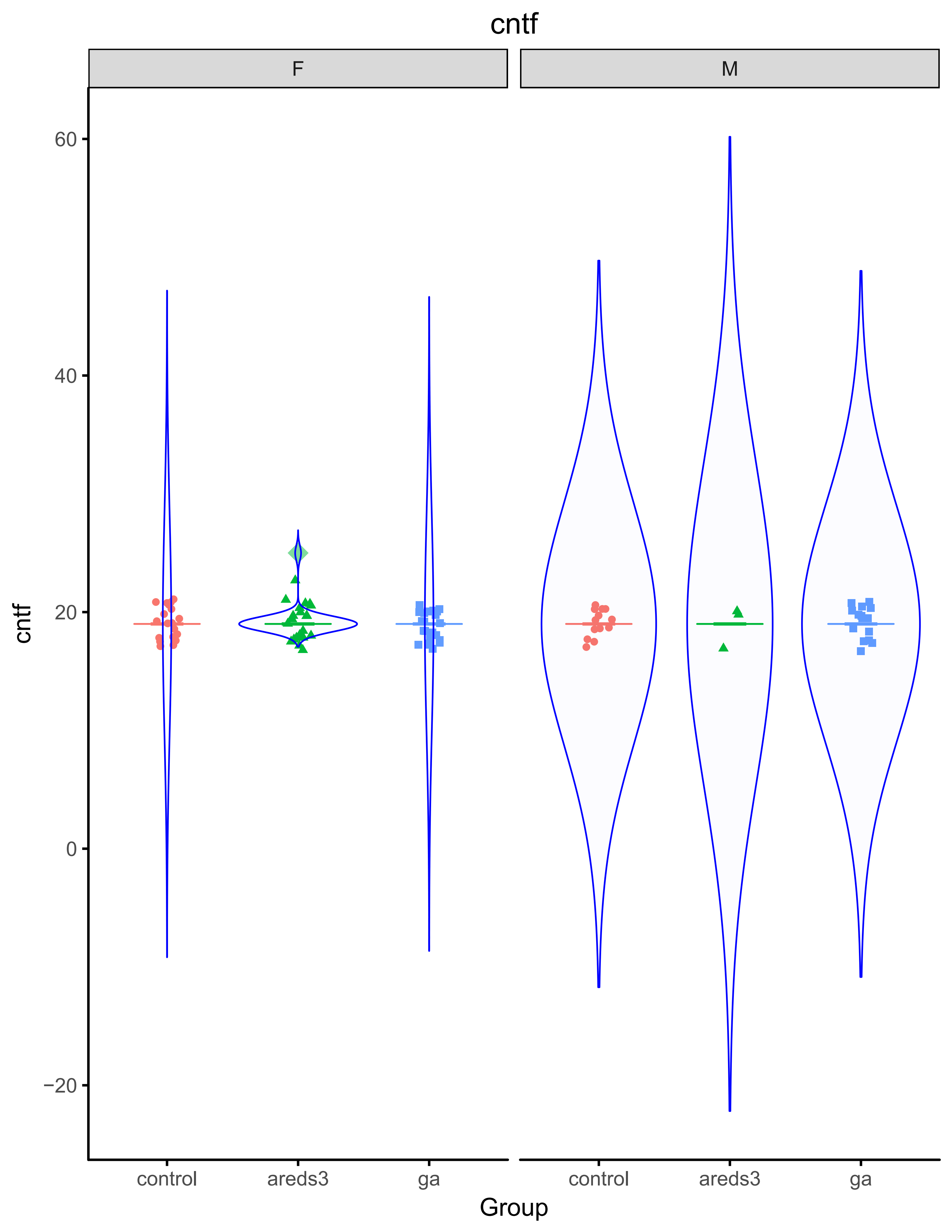
cntf
F
M
60
40
20
0
−20
cntf
control
areds3
ga
control
areds3
ga
Group

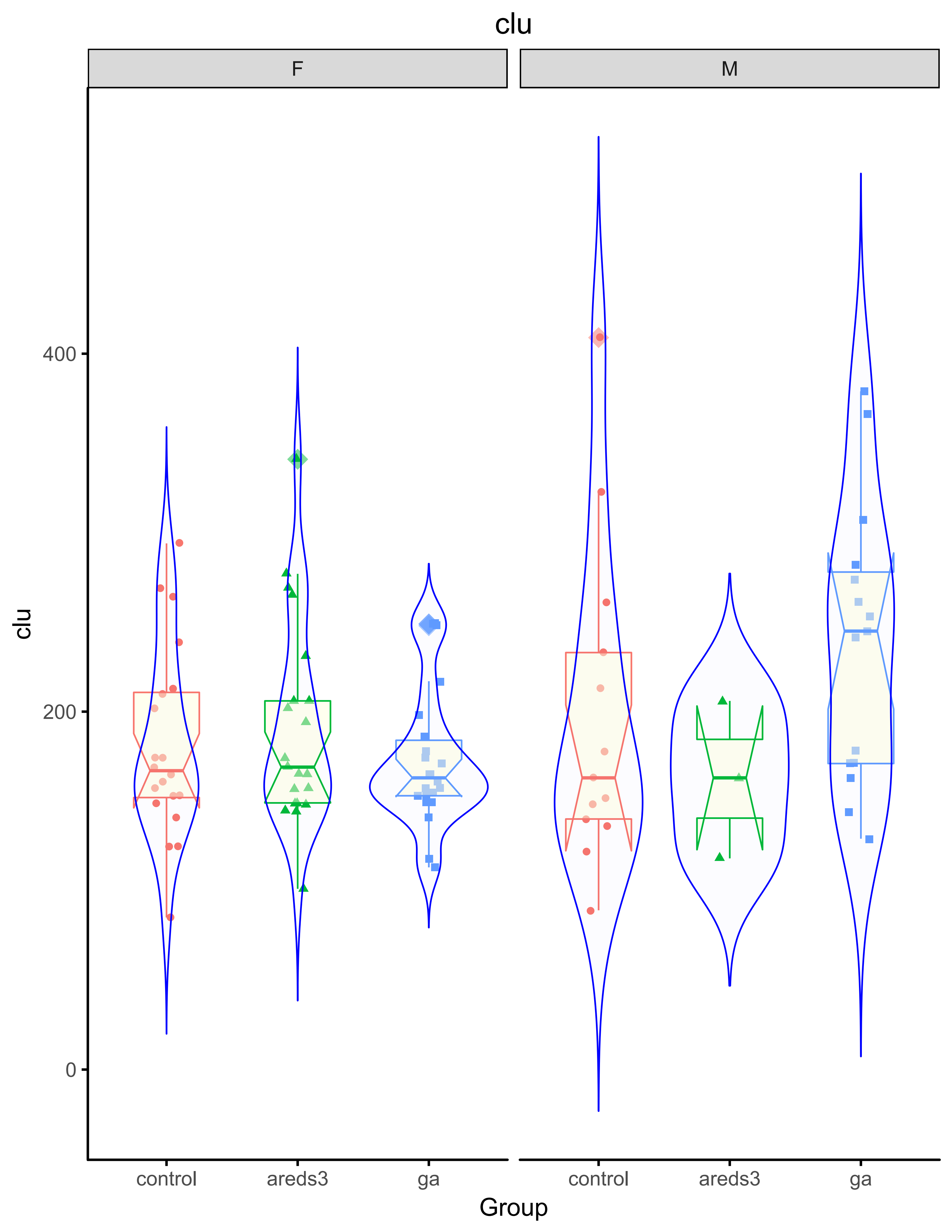
clu
F
M
400
200
0
clu
control
areds3
ga
control
areds3
ga
Group

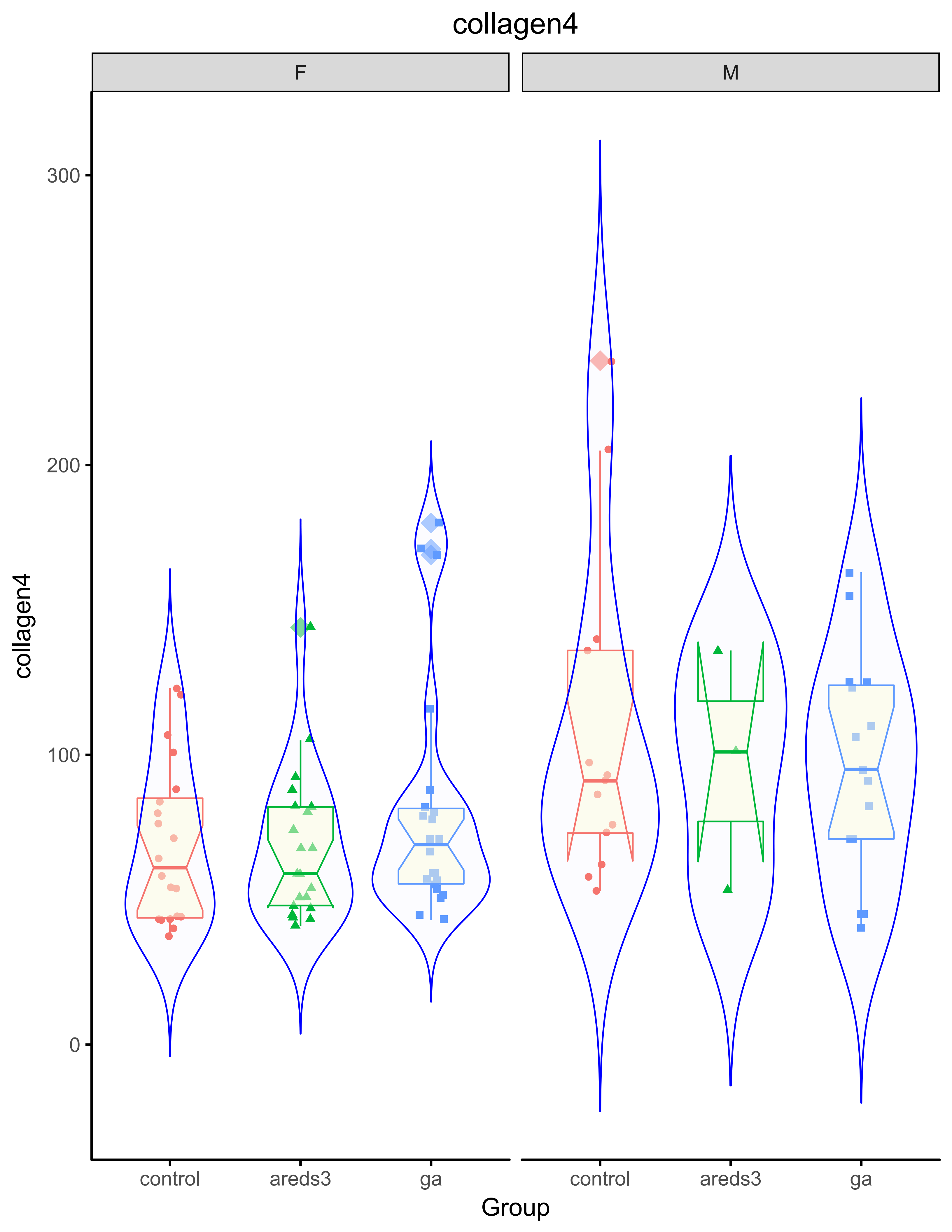
collagen4
F
M
300
200
100
0
collagen4
control
areds3
ga
control
areds3
ga
Group

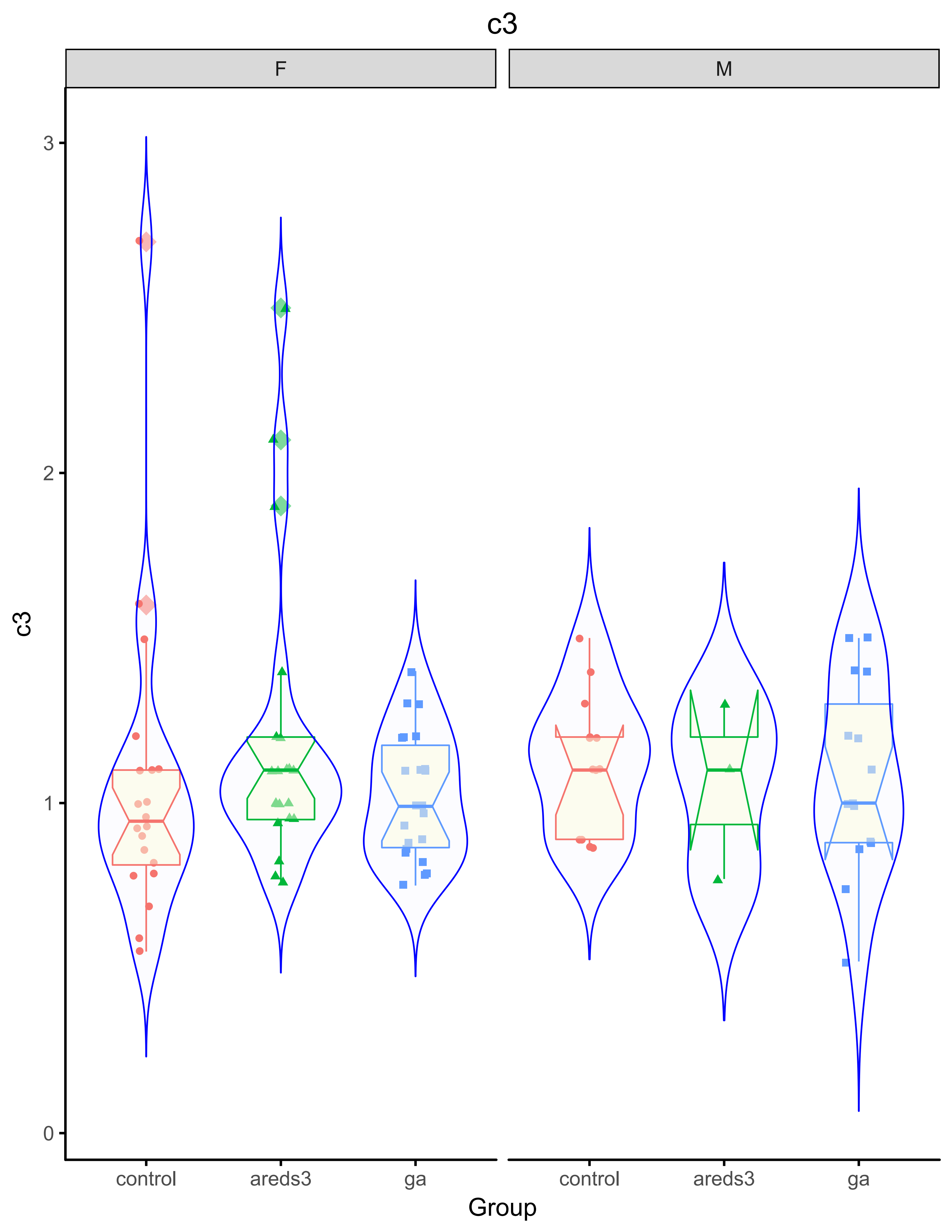
c3
F
M
3
2
1
0
c3
control
areds3
ga
control
areds3
ga
Group

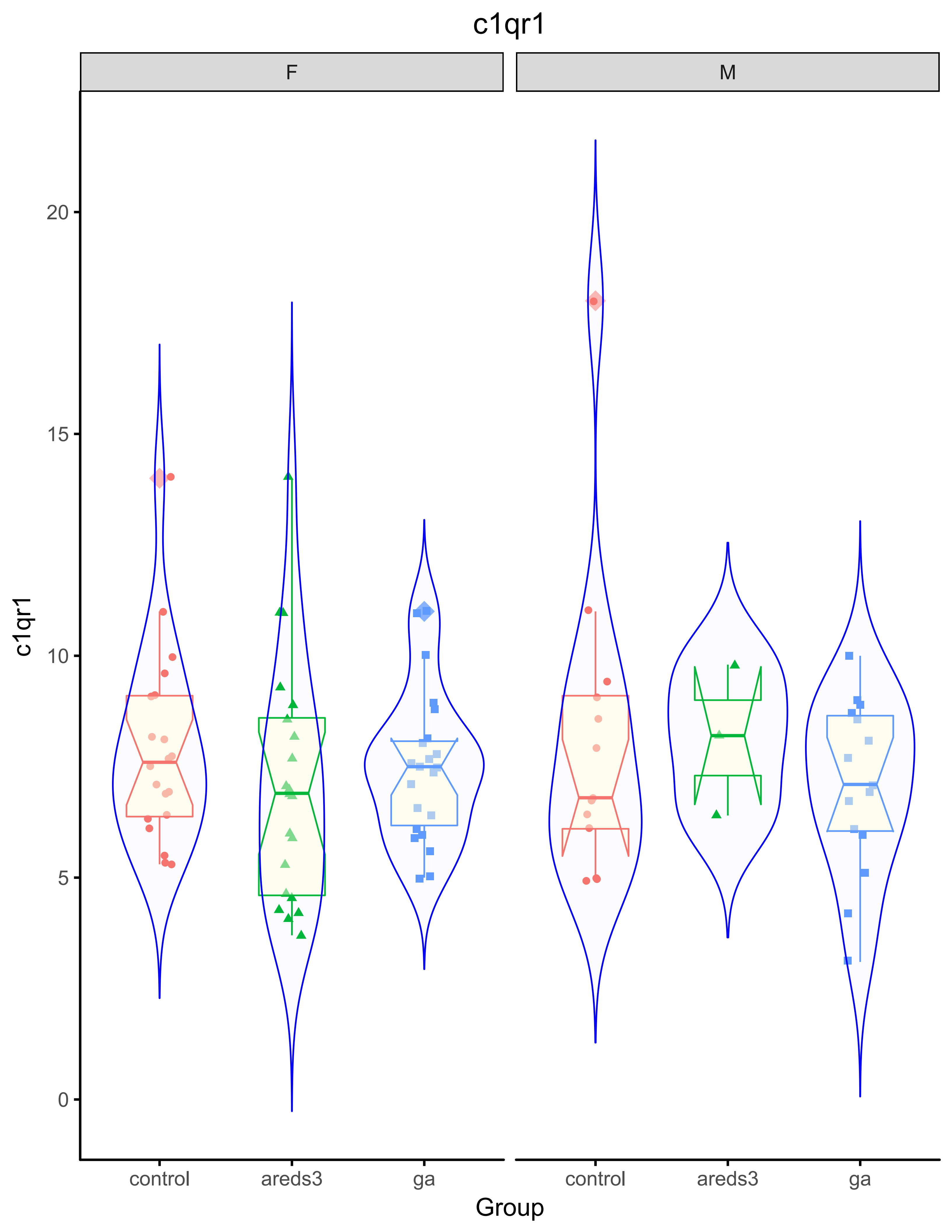
c1qr1
F
M
20
15
10
5
0
c1qr1
control
areds3
ga
control
areds3
ga
Group

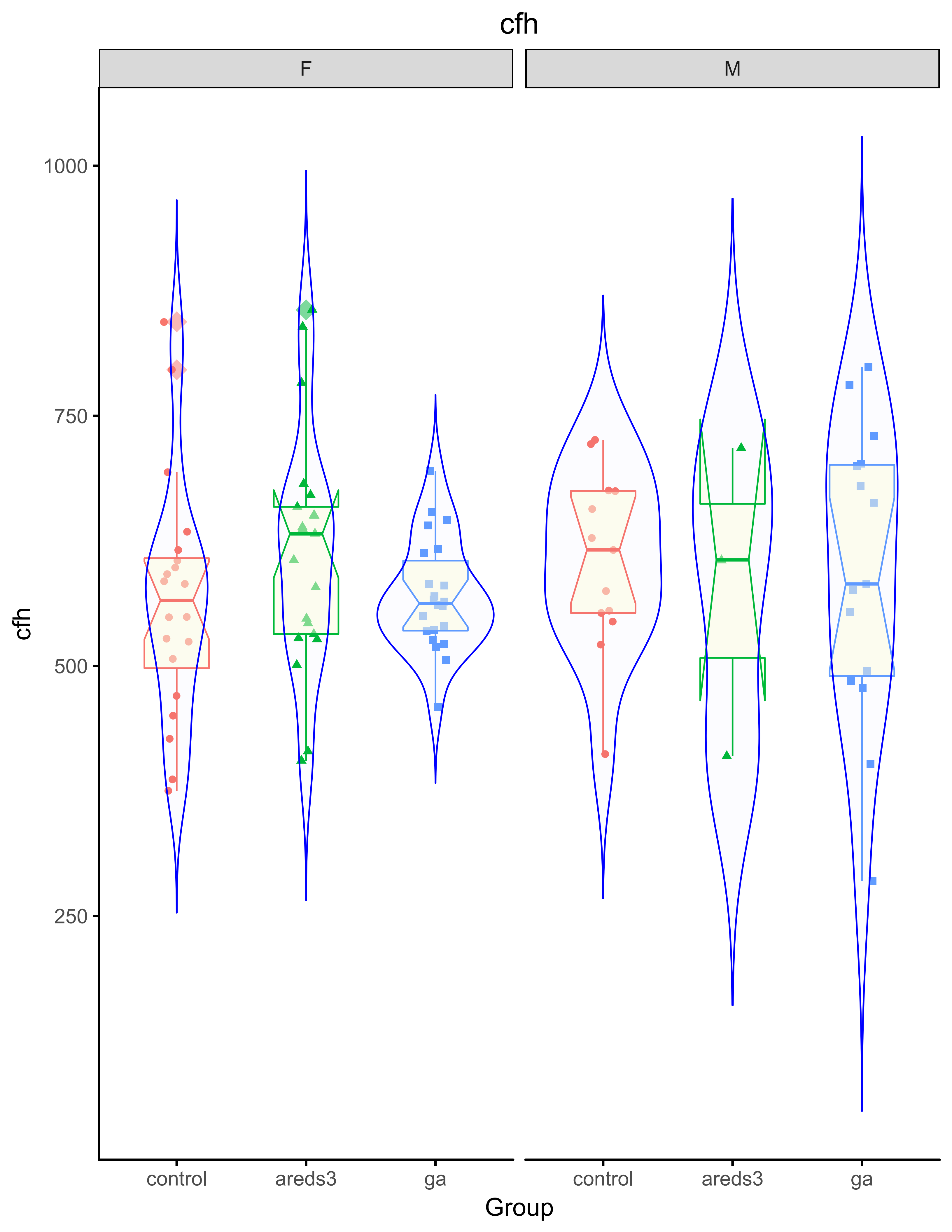

cfh
F
M
1000
750
500
250
cfh
control
areds3
ga
control
areds3
ga
Group

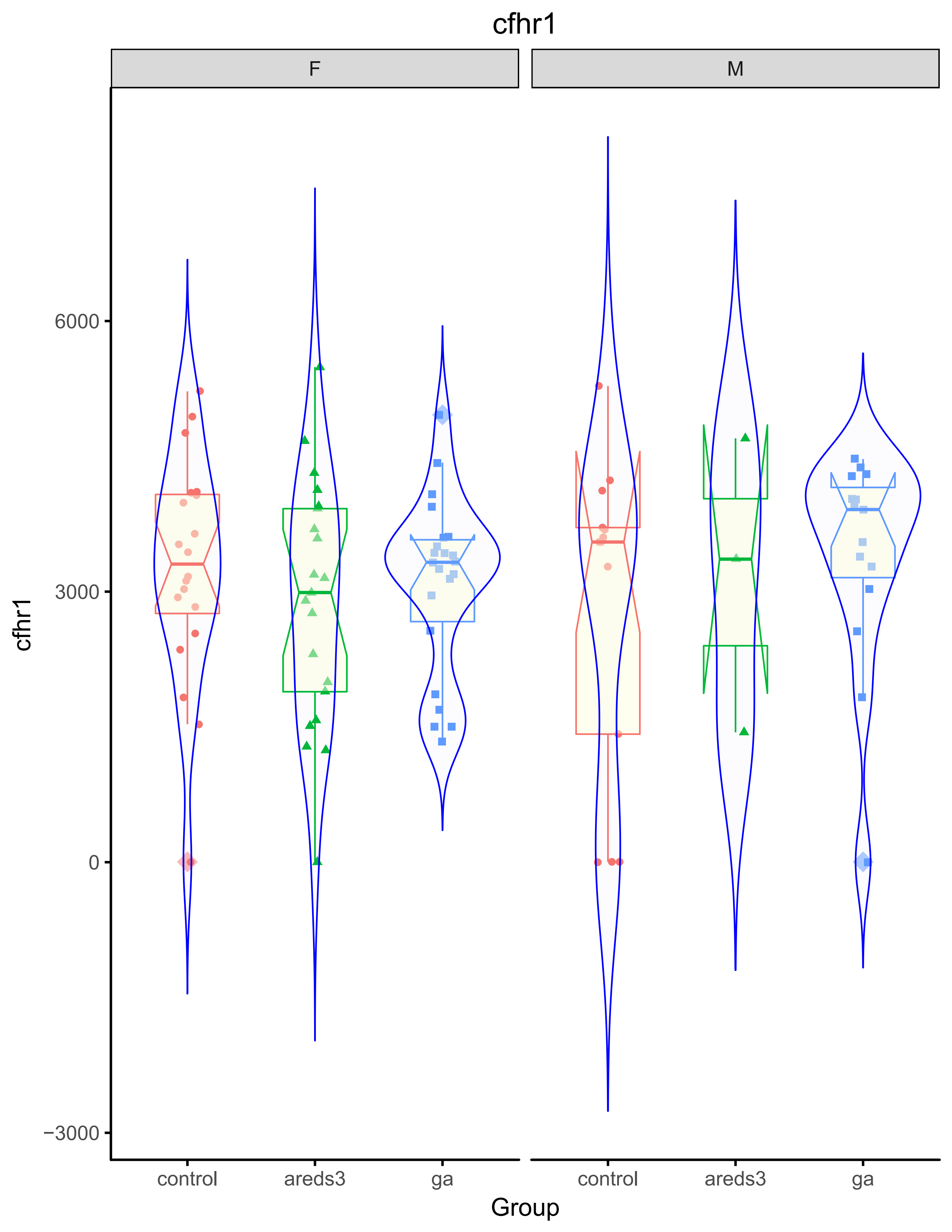

cfhr1
F
M
6000
3000
0
−3000
cfhr1
control
areds3
ga
control
areds3
ga
Group

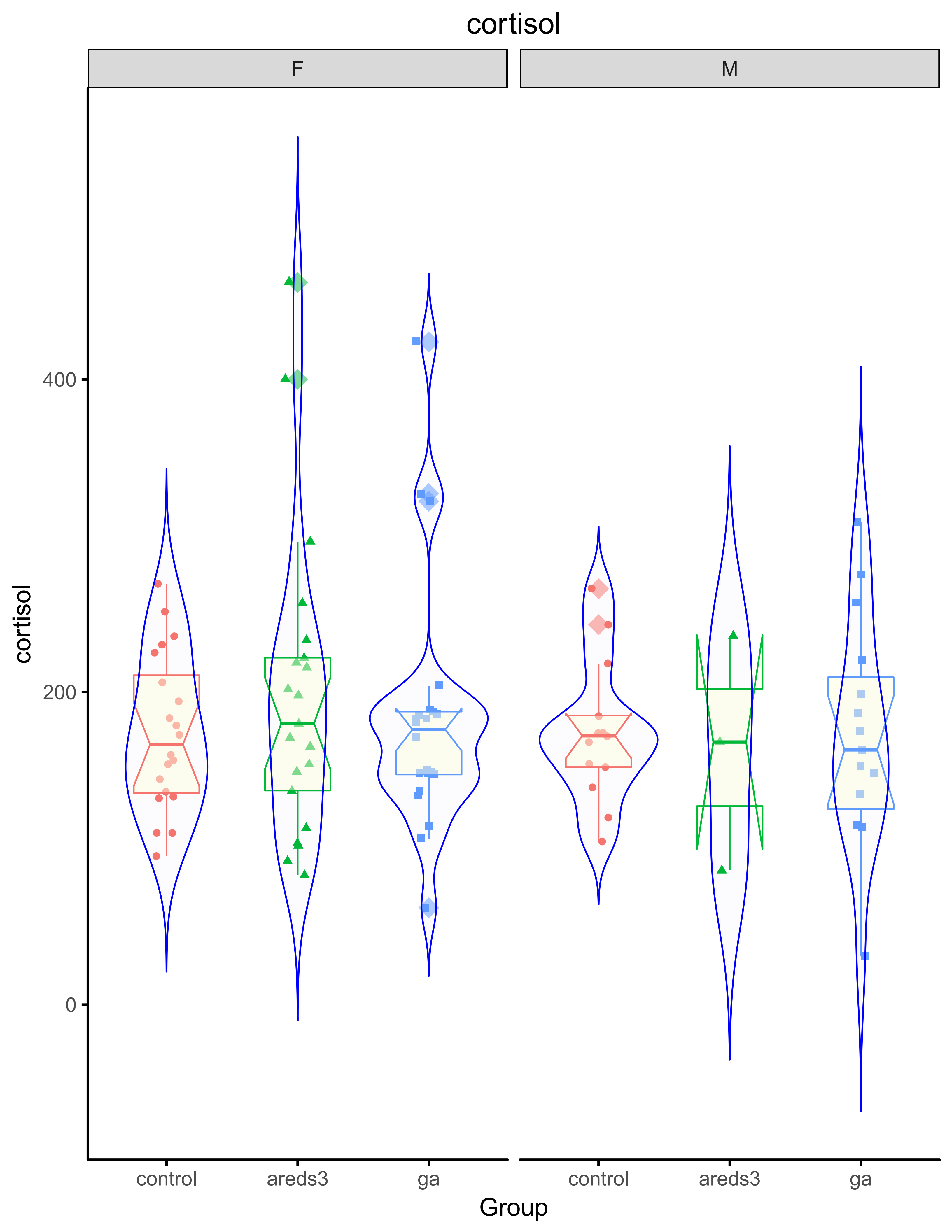
cortisol
F
M
400
200
0
cortisol
control
areds3
ga
control
areds3
ga
Group

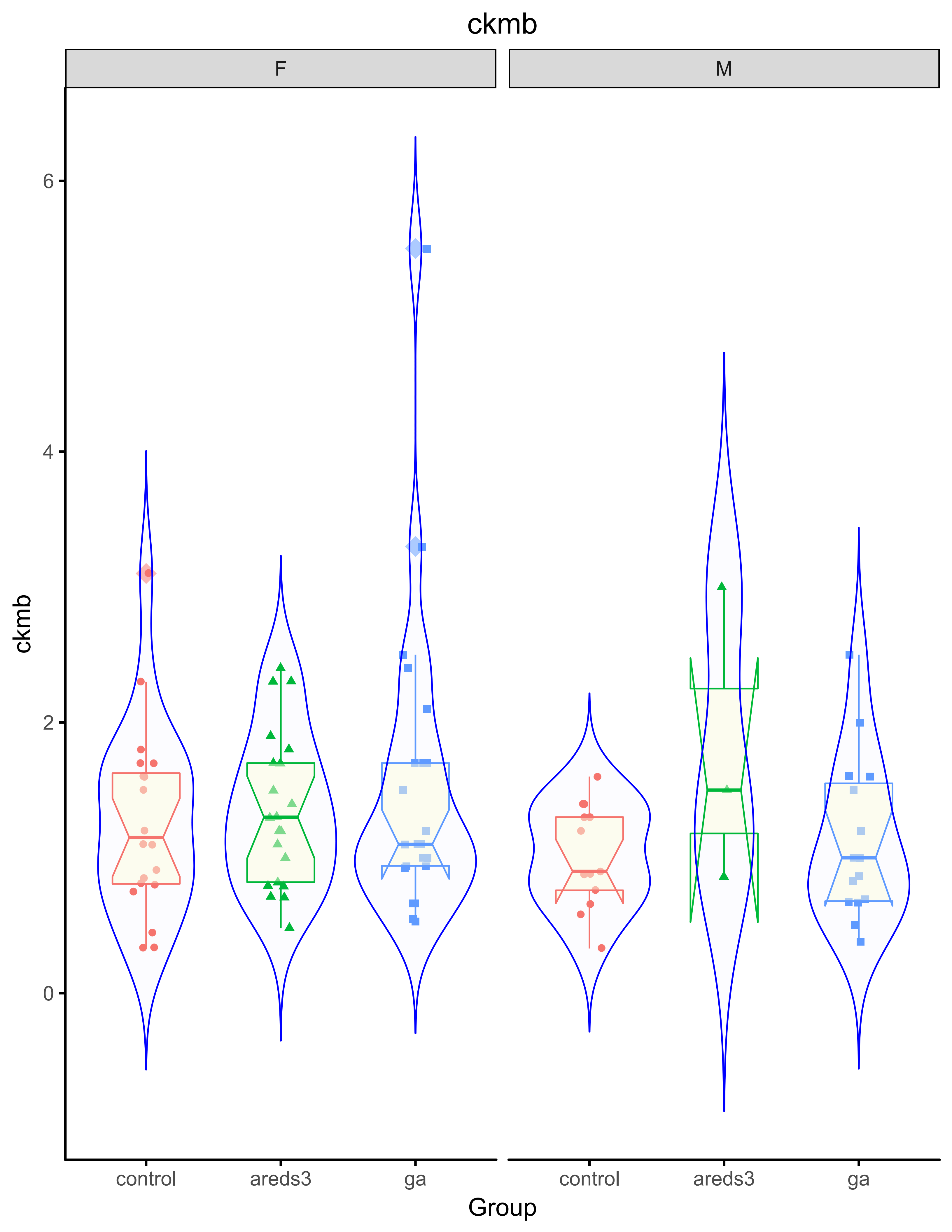
ckmb
F
M
6
4
2
0
ckmb
control
areds3
ga
control
areds3
ga
Group

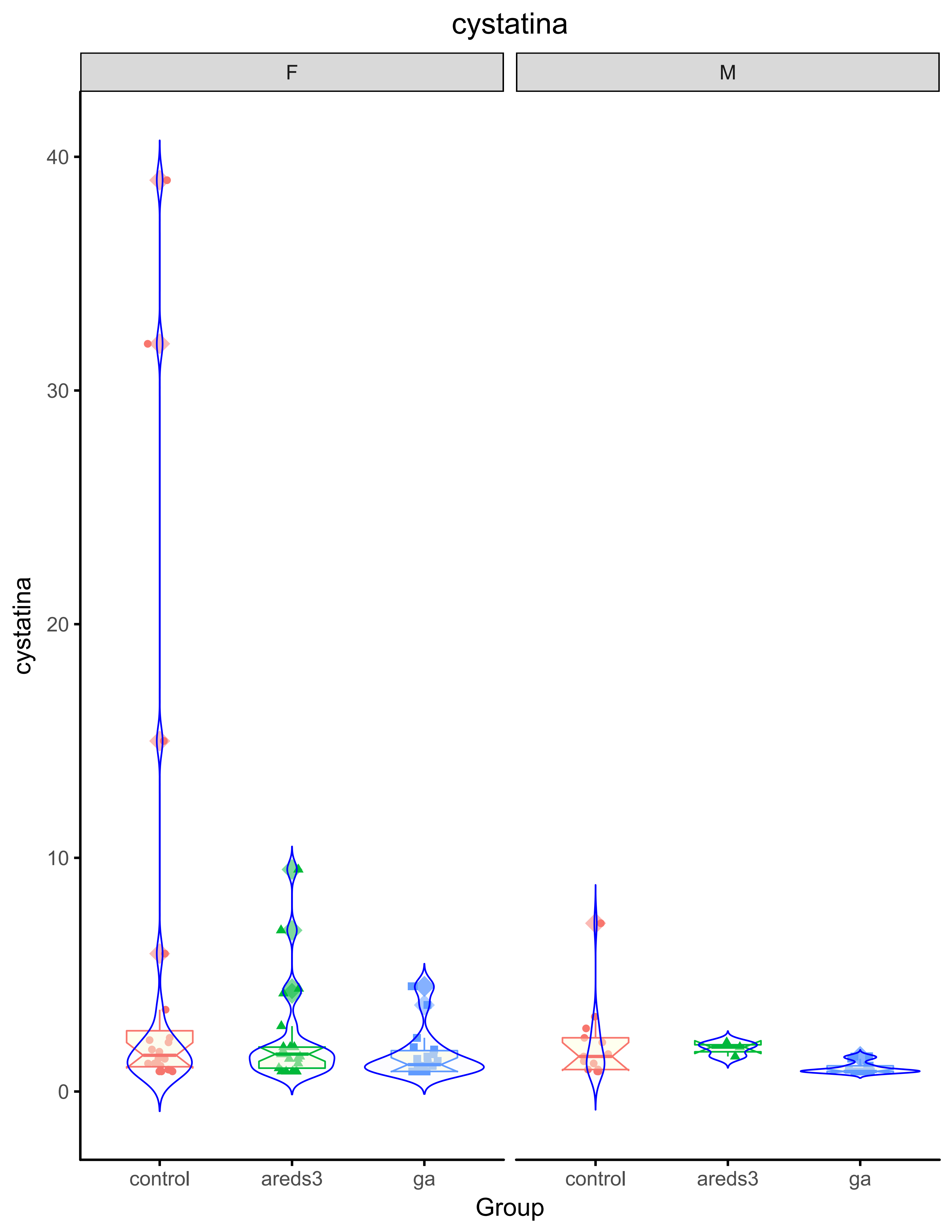
cystatina
F
M
40
30
20
10
0
cystatina
control
areds3
ga
control
areds3
ga
Group

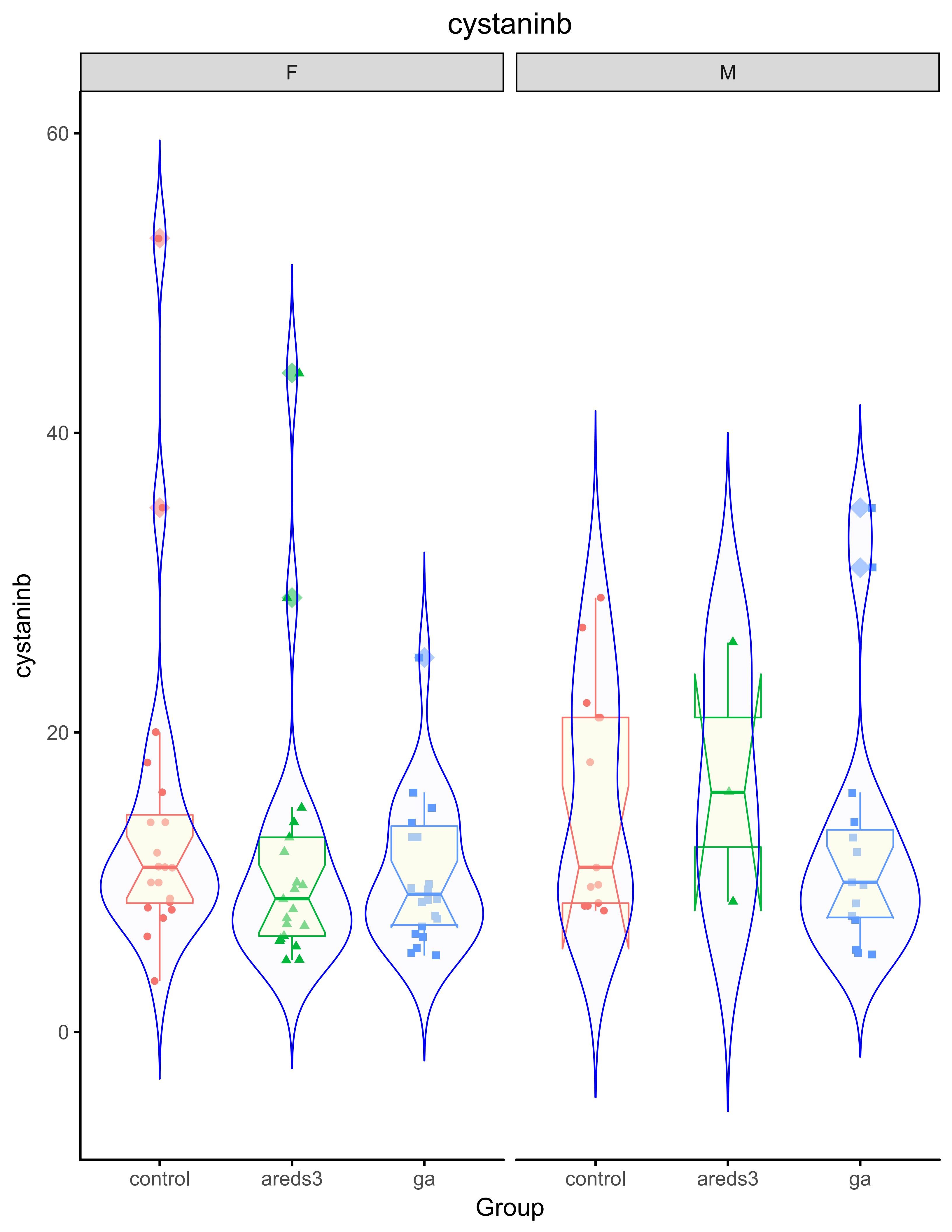

cystaninb
F
M
60
40
20
0
cystaninb
control
areds3
ga
control
areds3
ga
Group

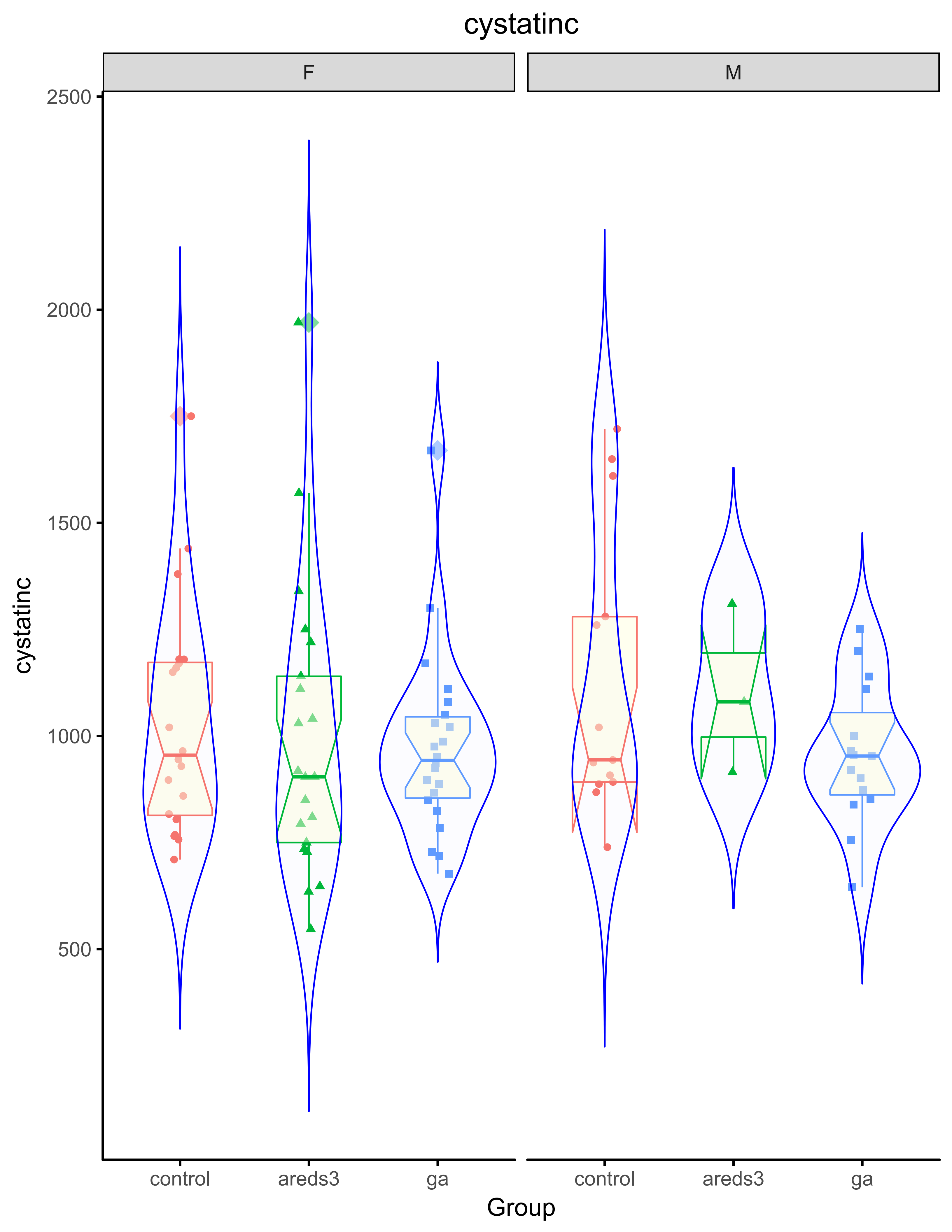

cystatinc
F
M
2500
2000
1500
1000
500
cystatinc
control
areds3
ga
control
areds3
ga
Group

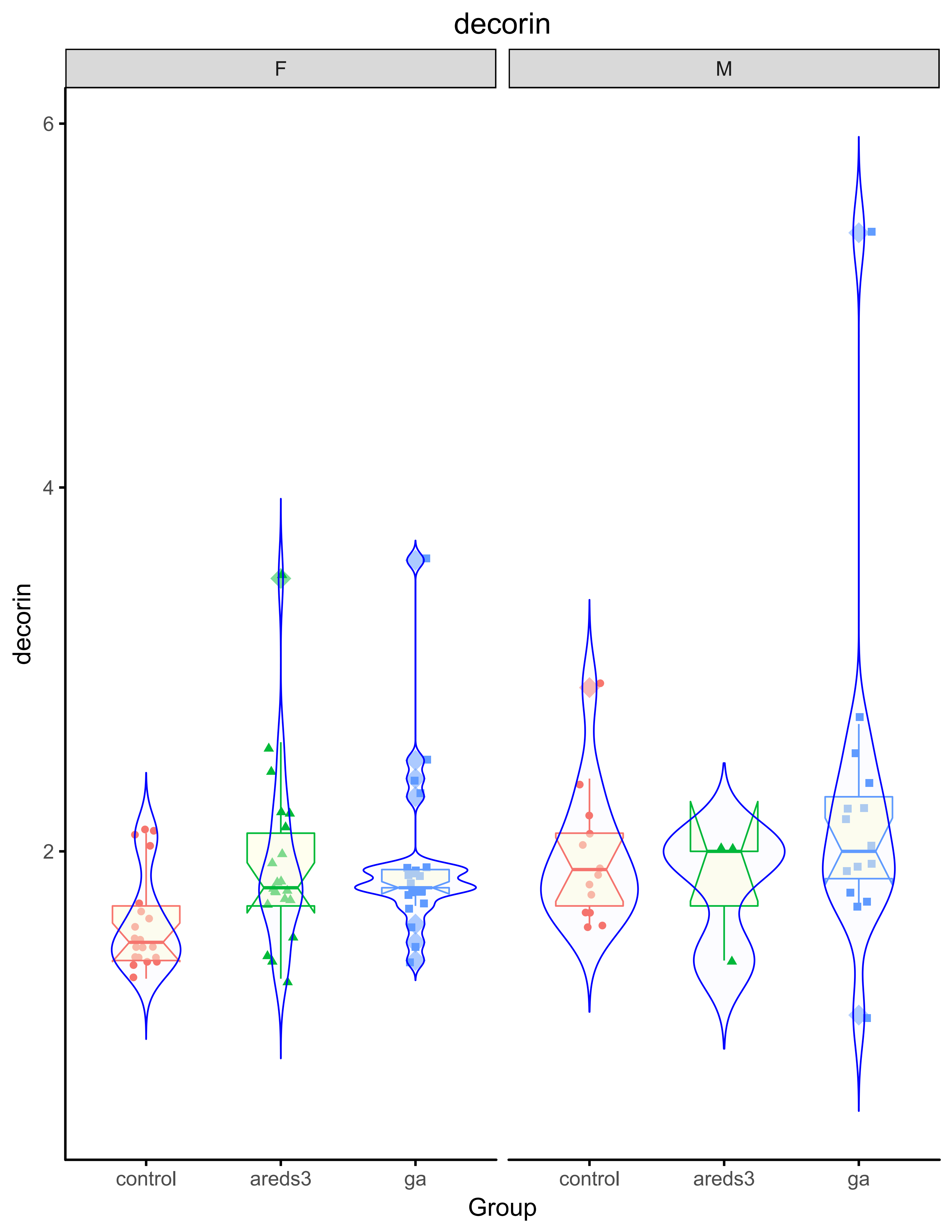

decorin
F
M
6
4
2
decorin
control
areds3
ga
control
areds3
ga
Group

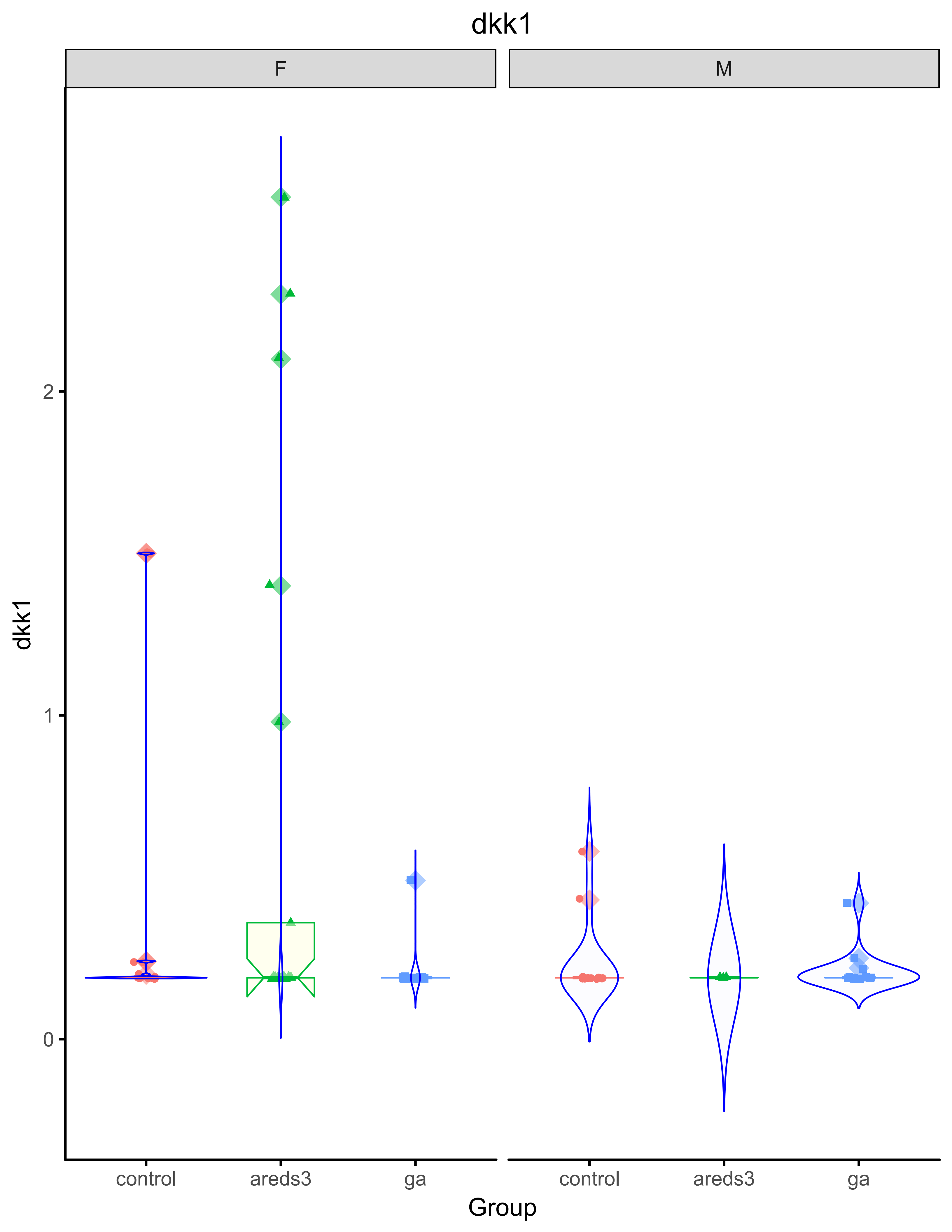

dkk1
F
M
dkk1
2
1
0
control
areds3
ga
control
areds3
ga
Group

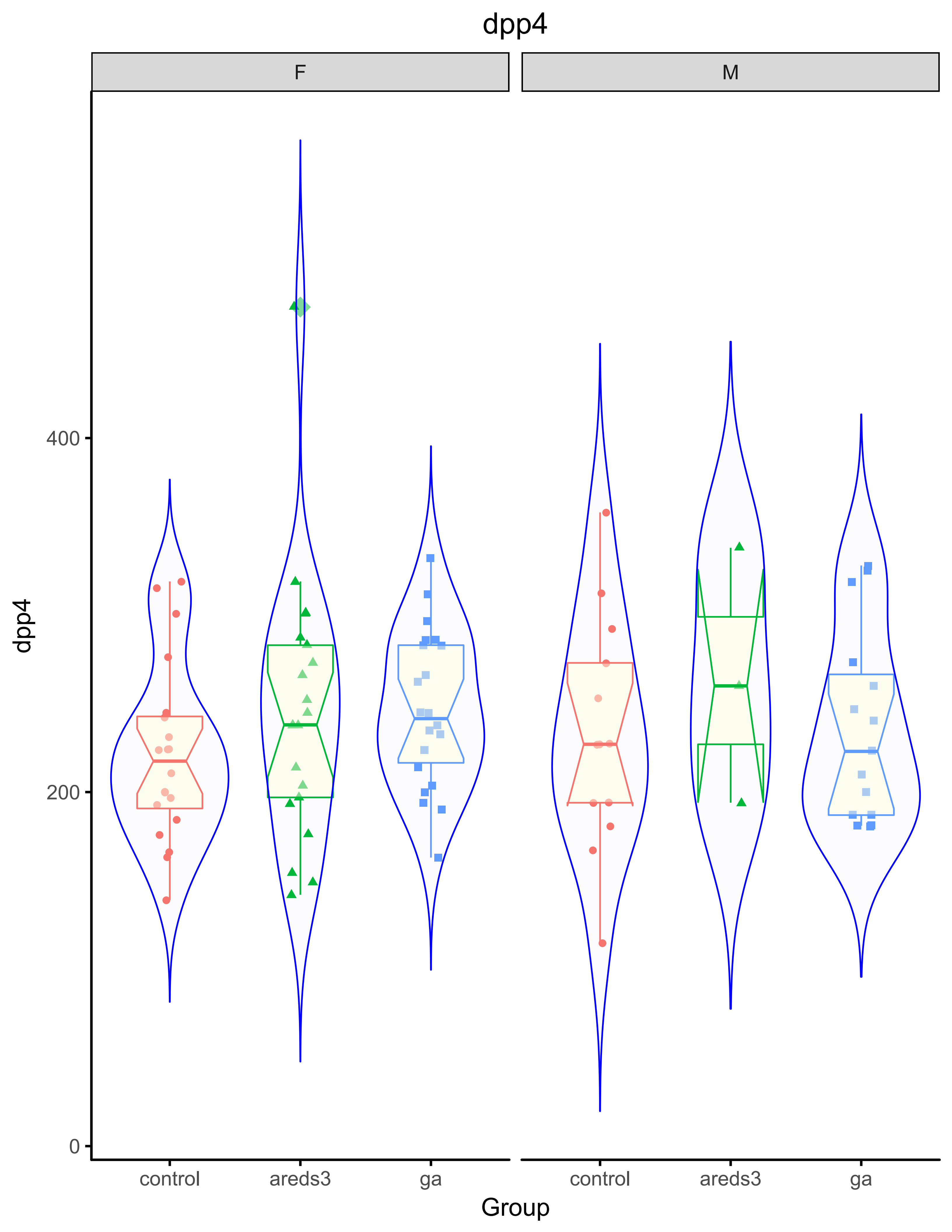
dpp4
F
M
dpp4
400
200
0
control
areds3
ga
control
areds3
ga
Group

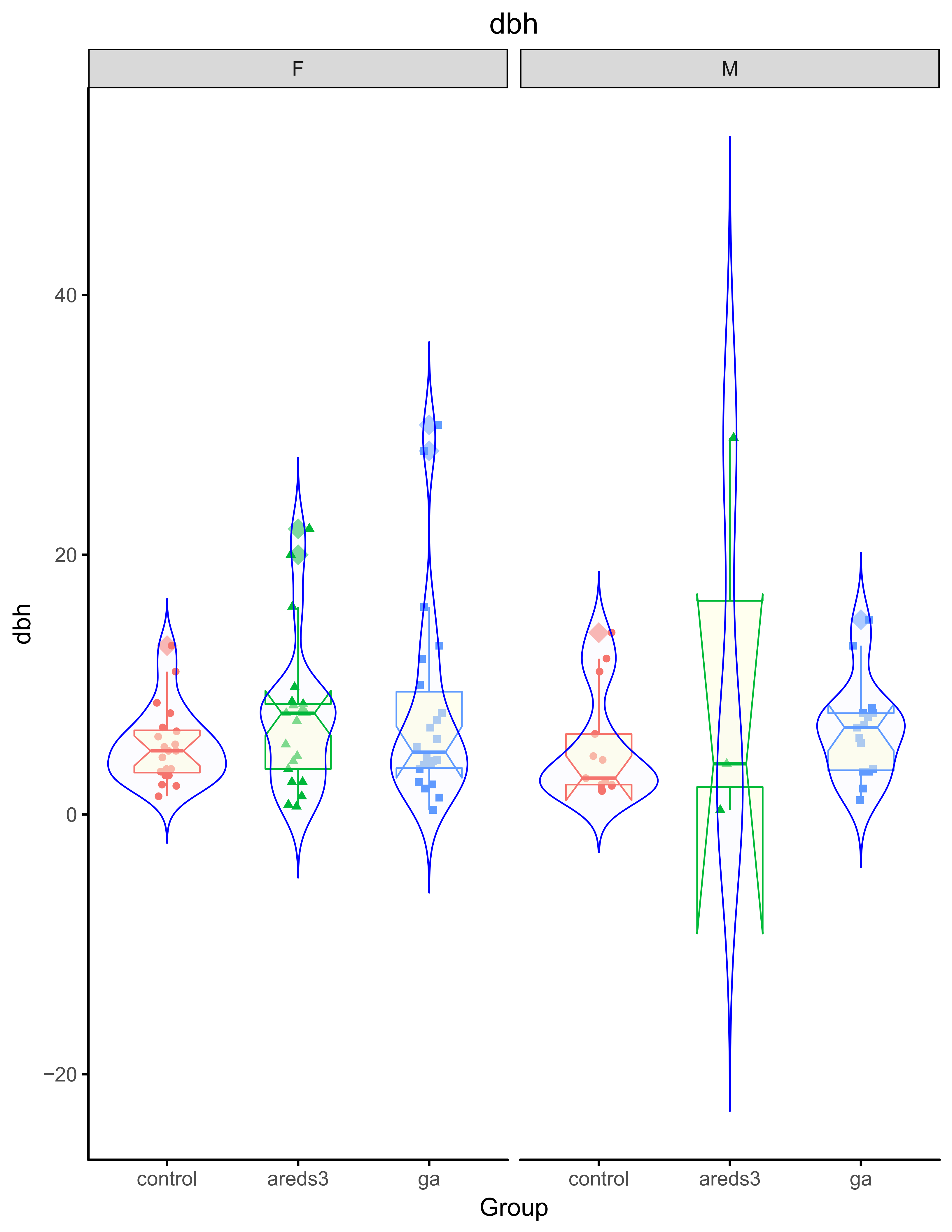

dbh
F
M
40
20
0
−20
dbh
control
areds3
ga
control
areds3
ga
Group

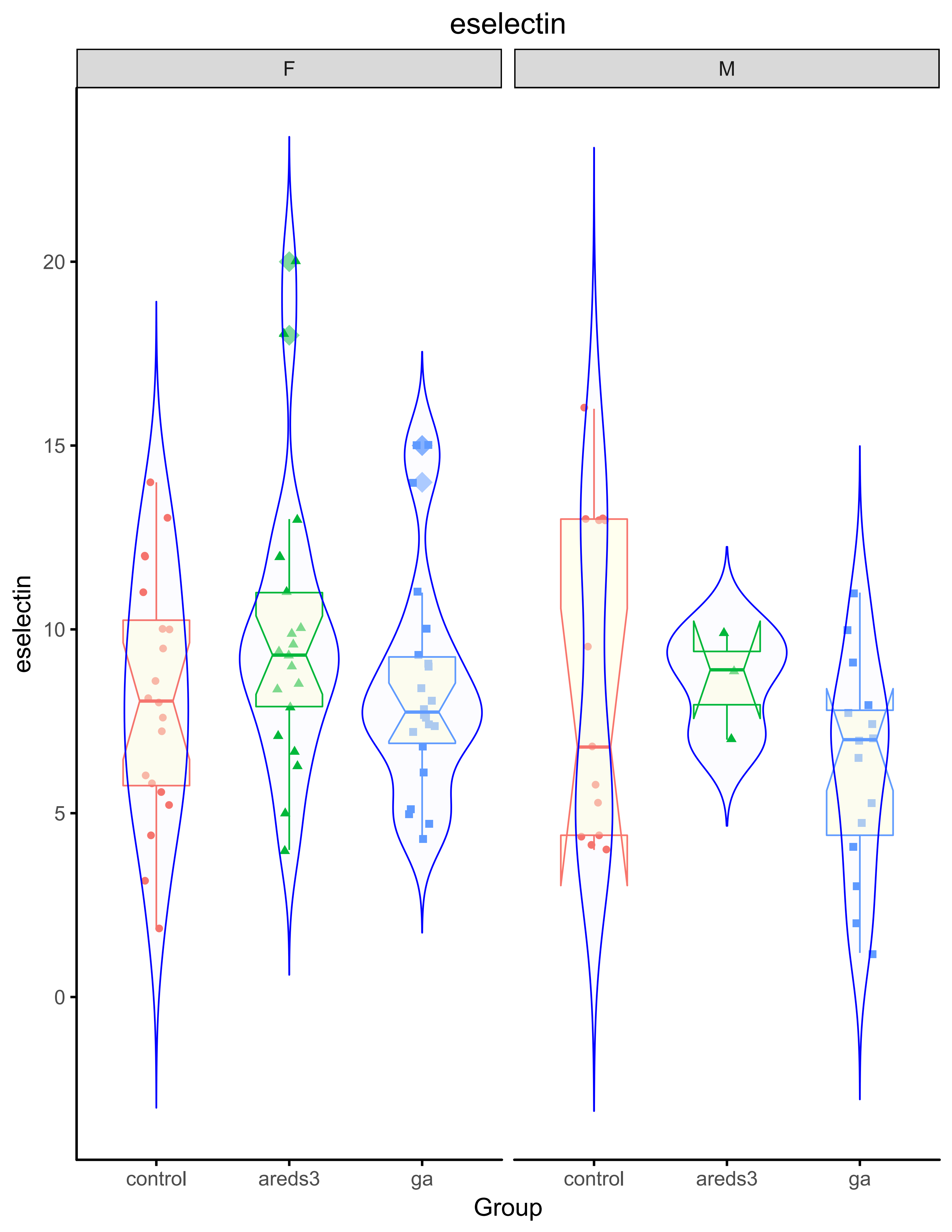
eselectin
F
M
20
15
10
5
0
eselectin
control
areds3
ga
control
areds3
ga
Group

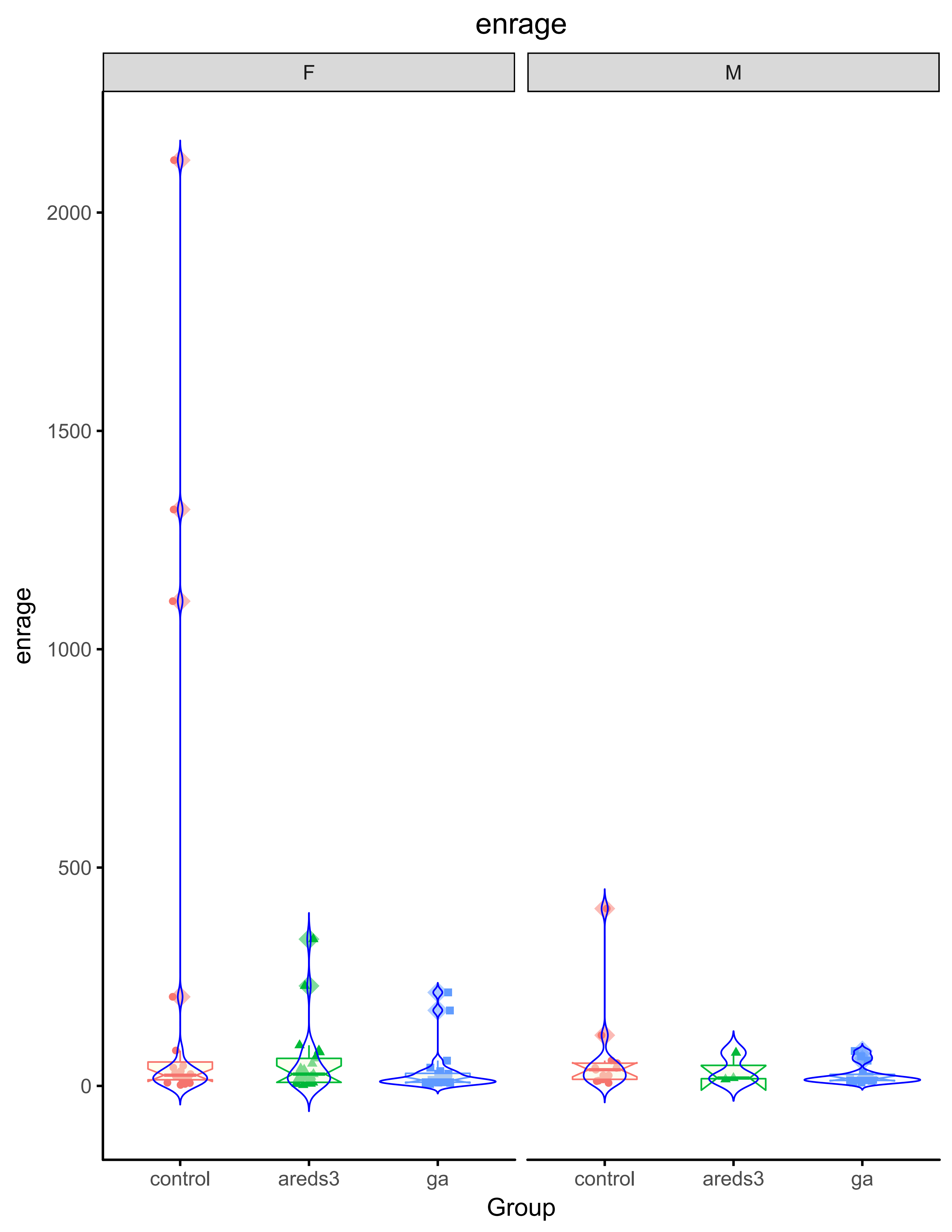

enrage
F
M
2000
1500
1000
500
0
enrage
control
areds3
ga
control
areds3
ga
Group

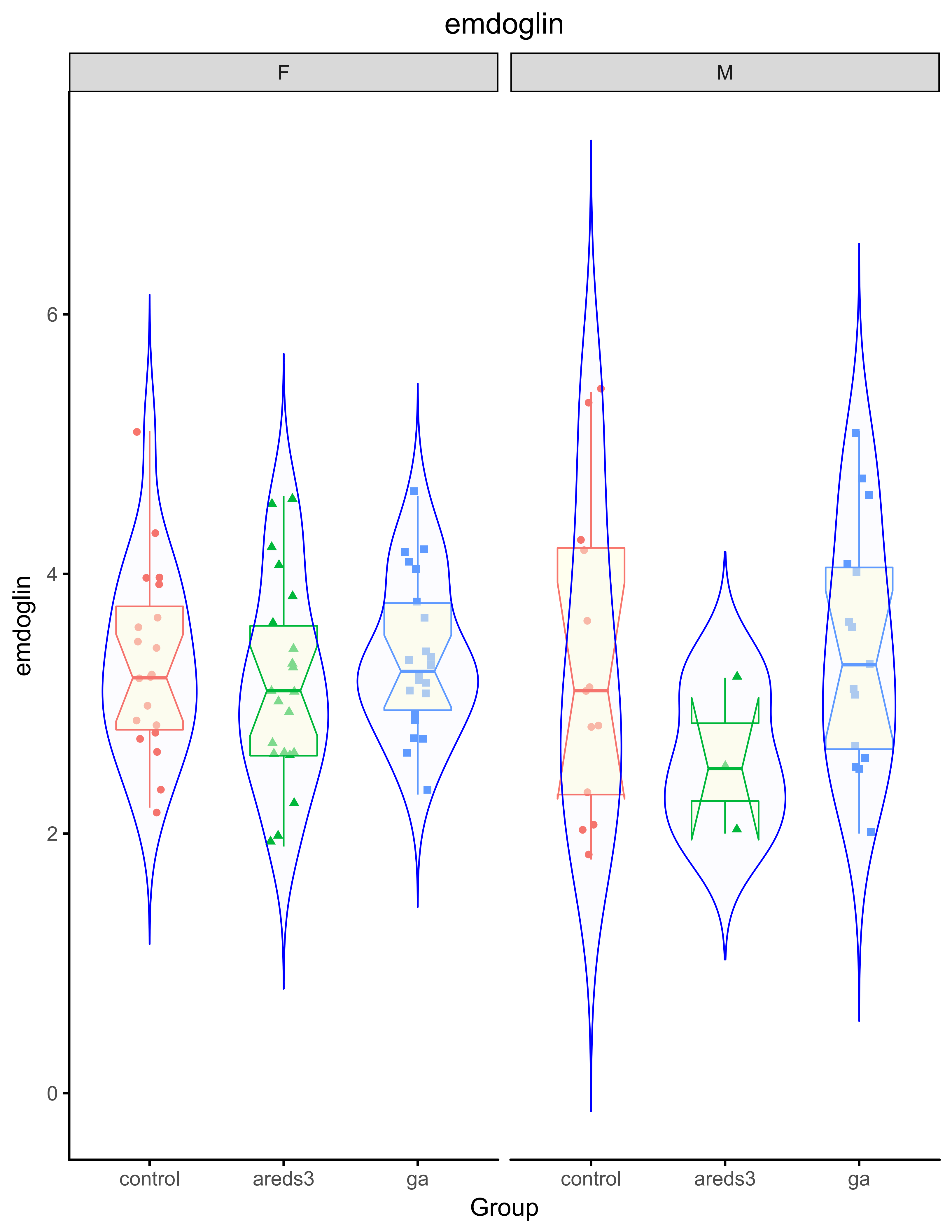
emdoglin
F
M
emdoglin
6
4
2
0
control
areds3
ga
control
areds3
ga
Group

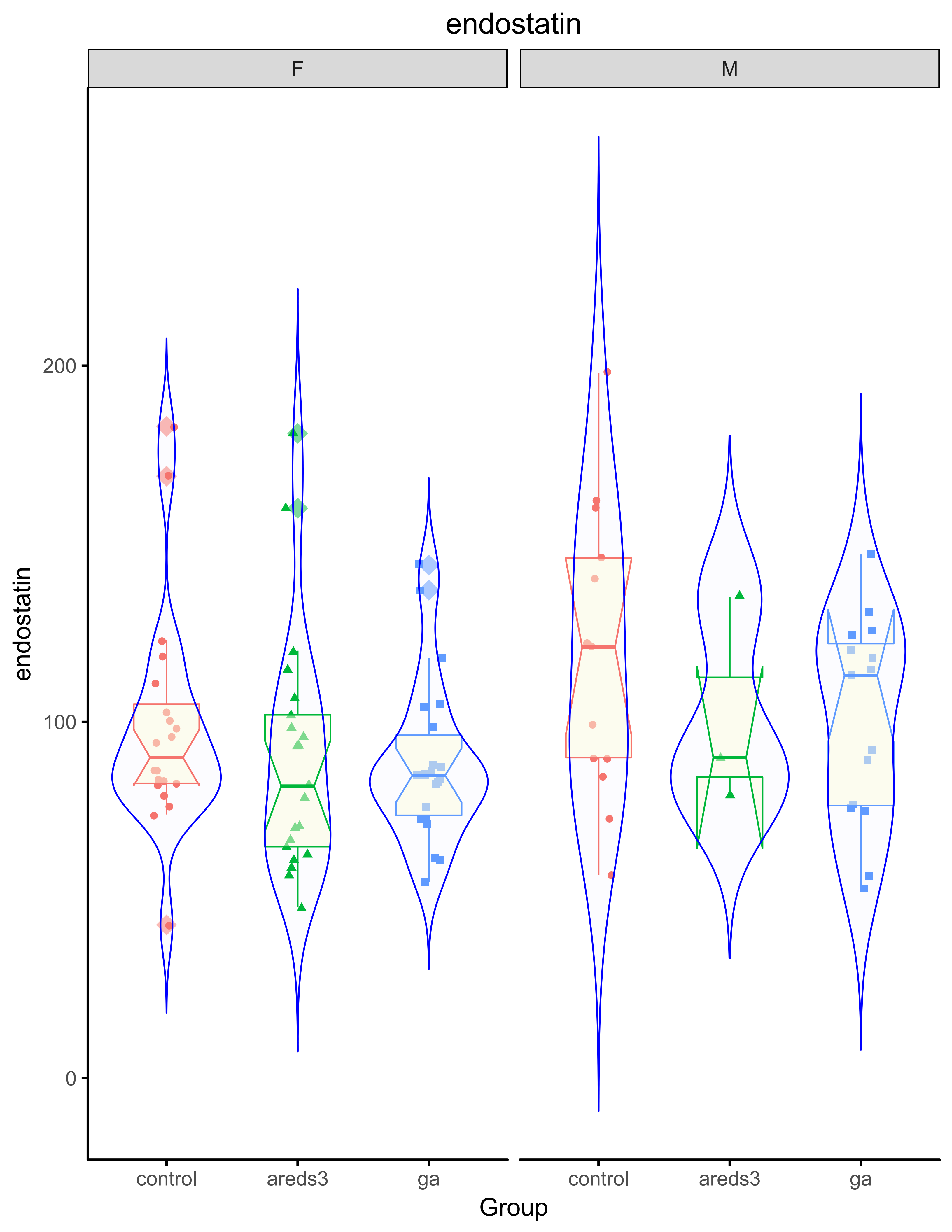

endostatin
F
M
200
100
0
endostatin
control
areds3
ga
control
areds3
ga
Group

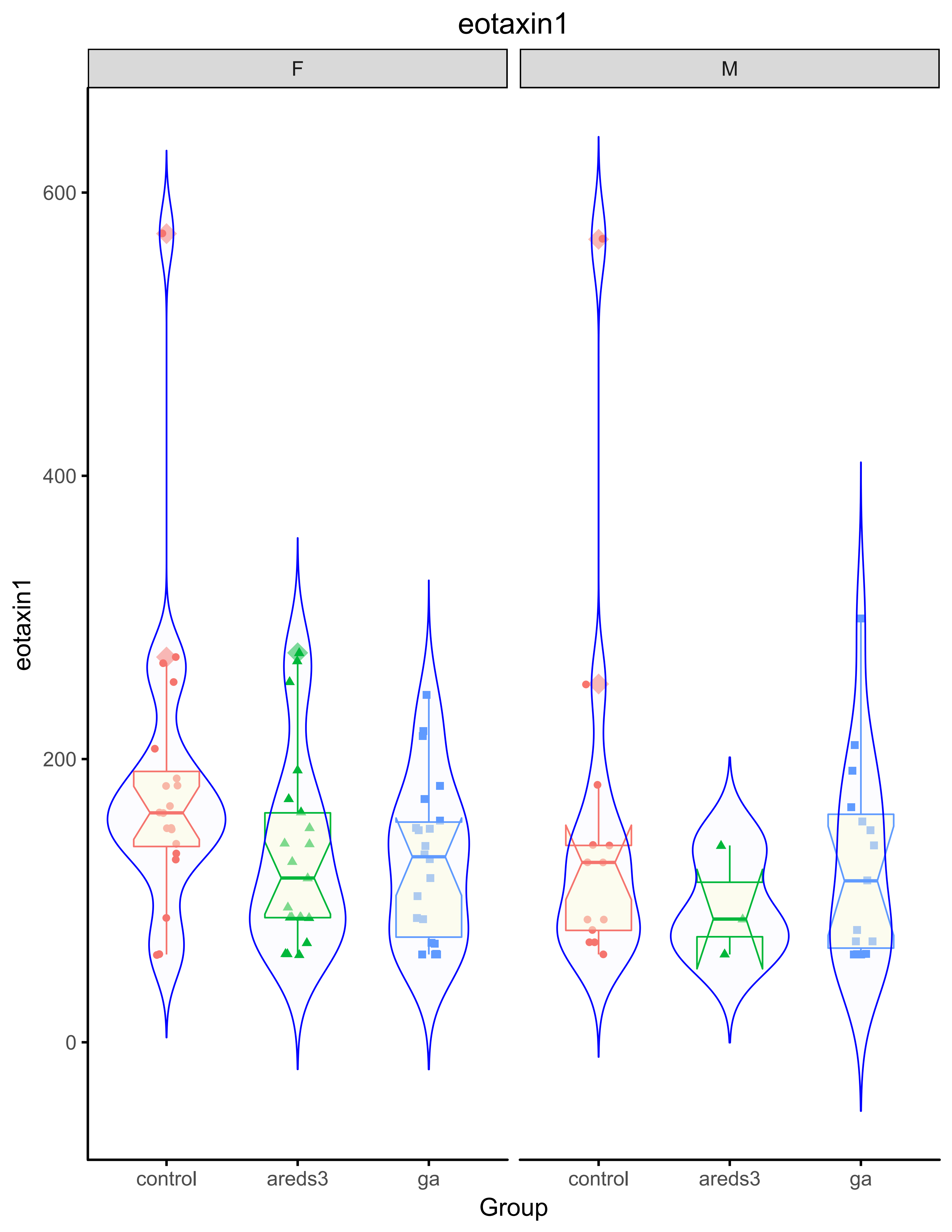

eotaxin1
F
M
600
400
200
0
eotaxin1
control
areds3
ga
control
areds3
ga
Group

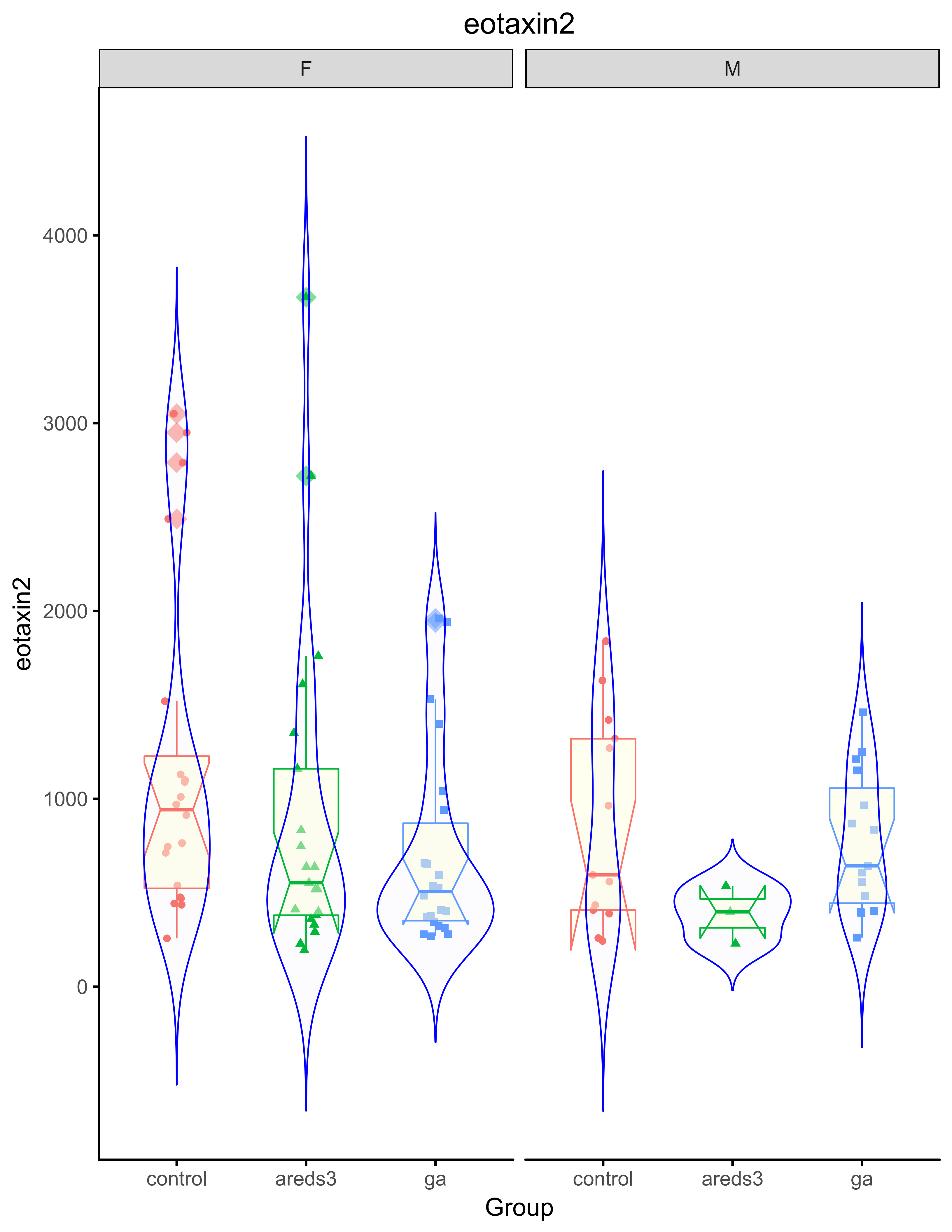

eotaxin2
F
M
4000
3000
2000
1000
0
eotaxin2
control
areds3
ga
control
areds3
ga
Group

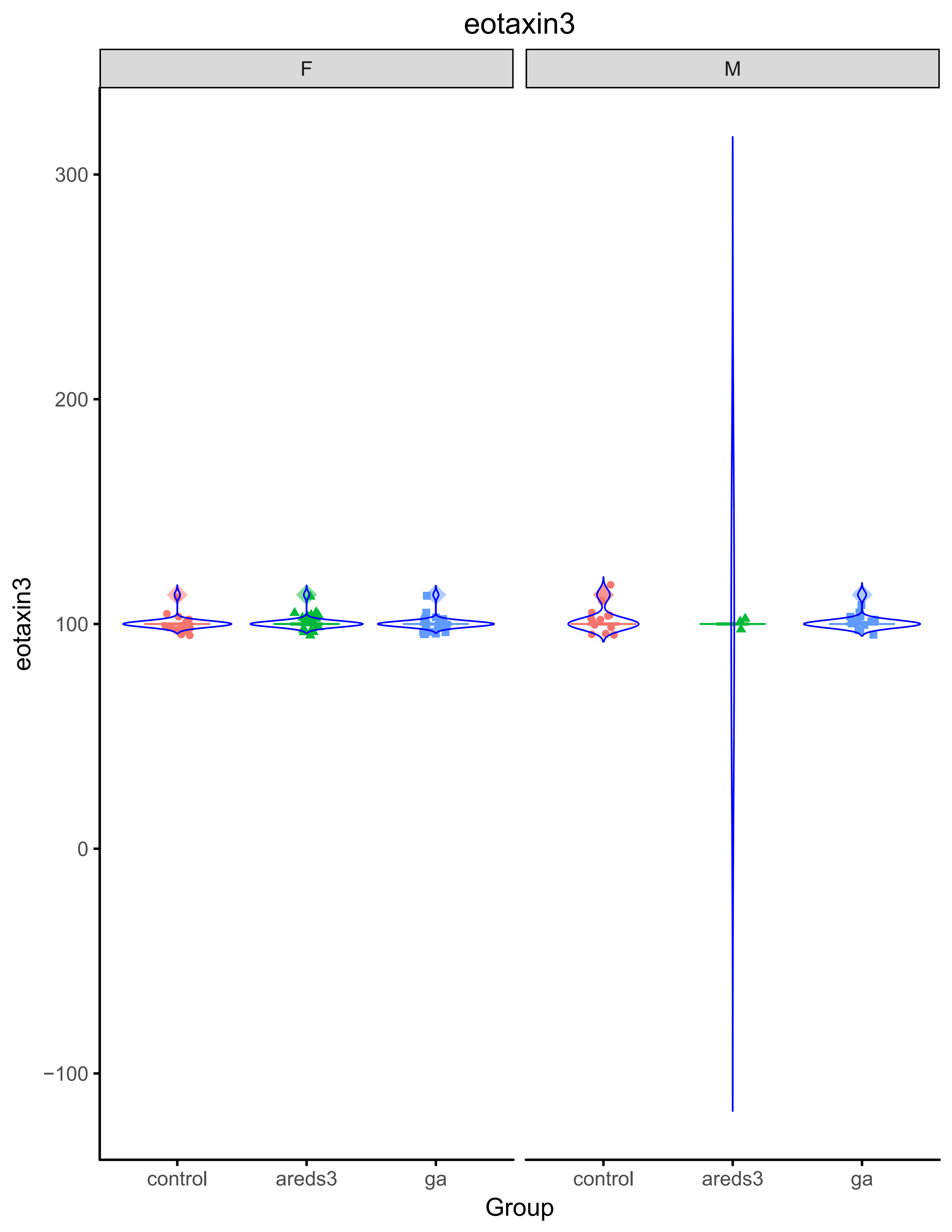
eotaxin3
F
M
300
200
100
0
−100
eotaxin3
control
areds3
ga
control
areds3
ga
Group

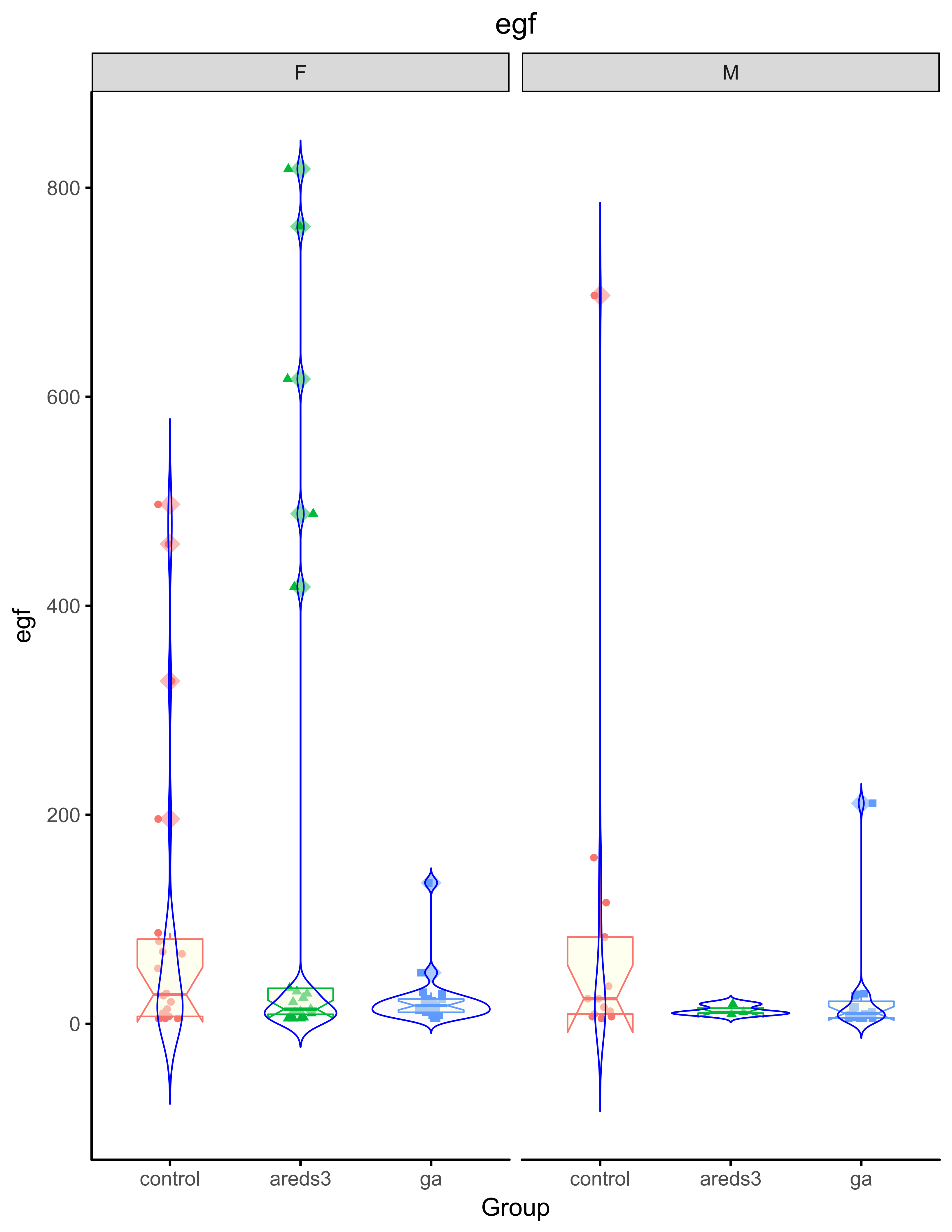

egf
F
M
800
600
400
200
0
egf
control
areds3
ga
control
areds3
ga
Group

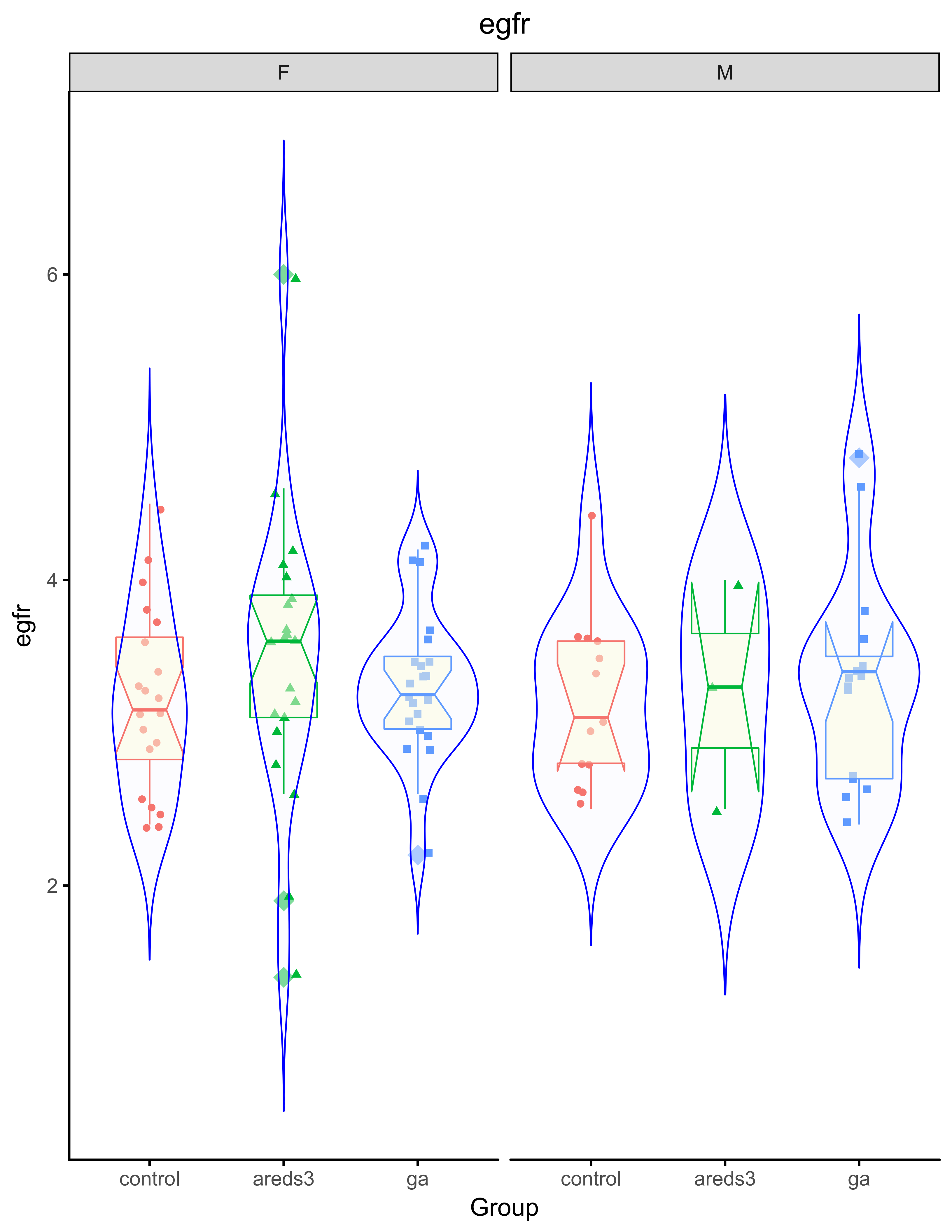

egfr
F
M
6
4
2
egfr
control
areds3
ga
control
areds3
ga
Group

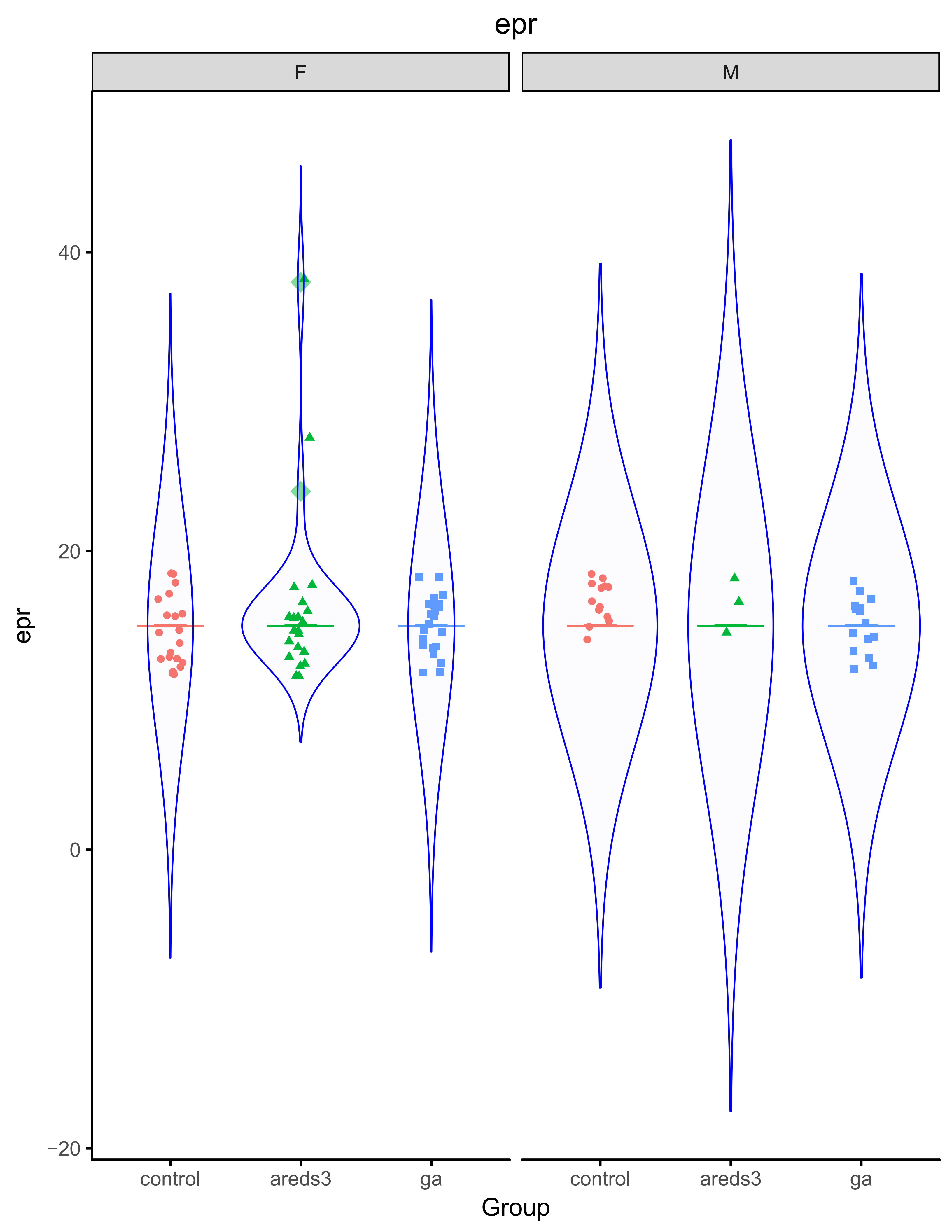

epr
F
M
40
20
0
−20
epr
control
areds3
ga
control
areds3
ga
Group

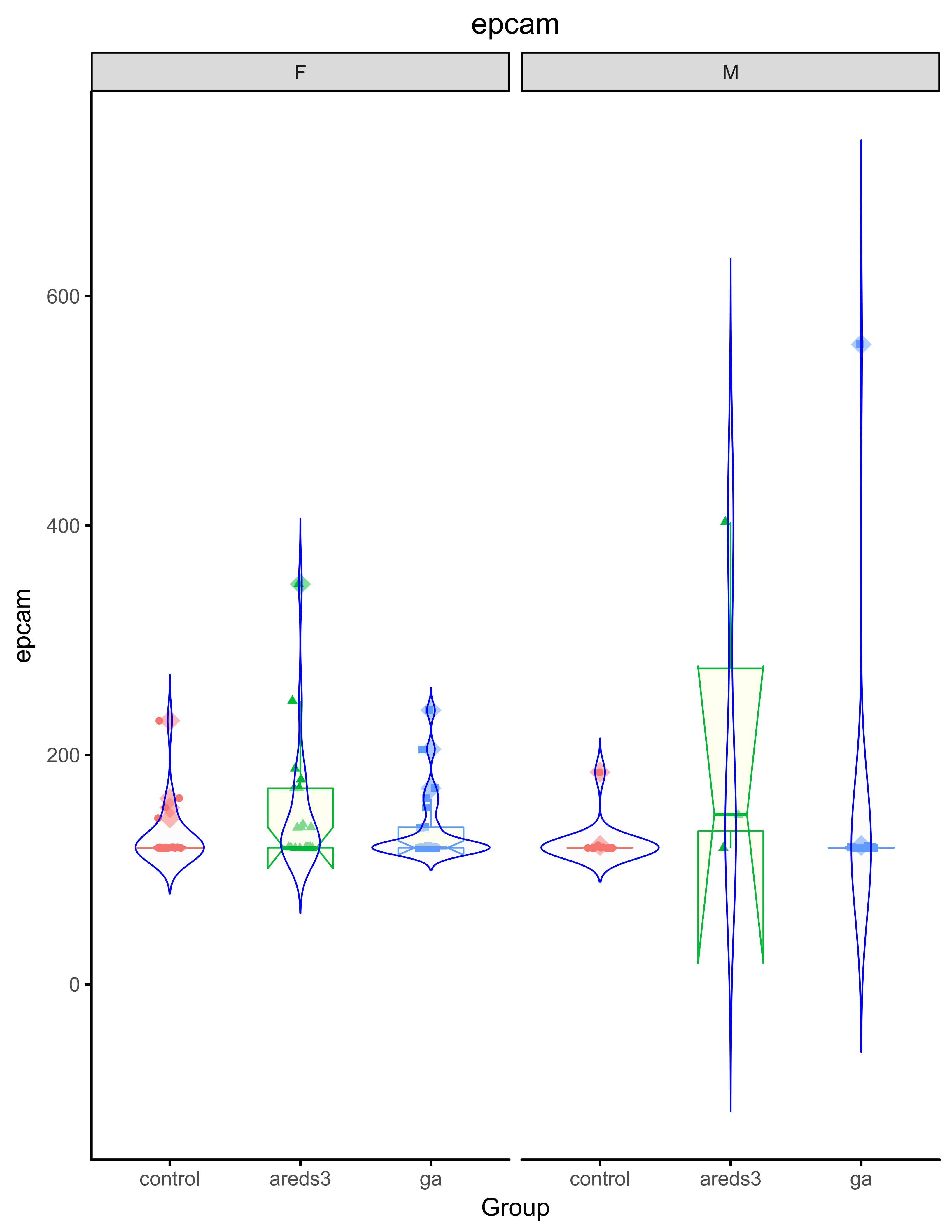

epcam
F
M
600
400
200
0
epcam
control
areds3
ga
control
areds3
ga
Group

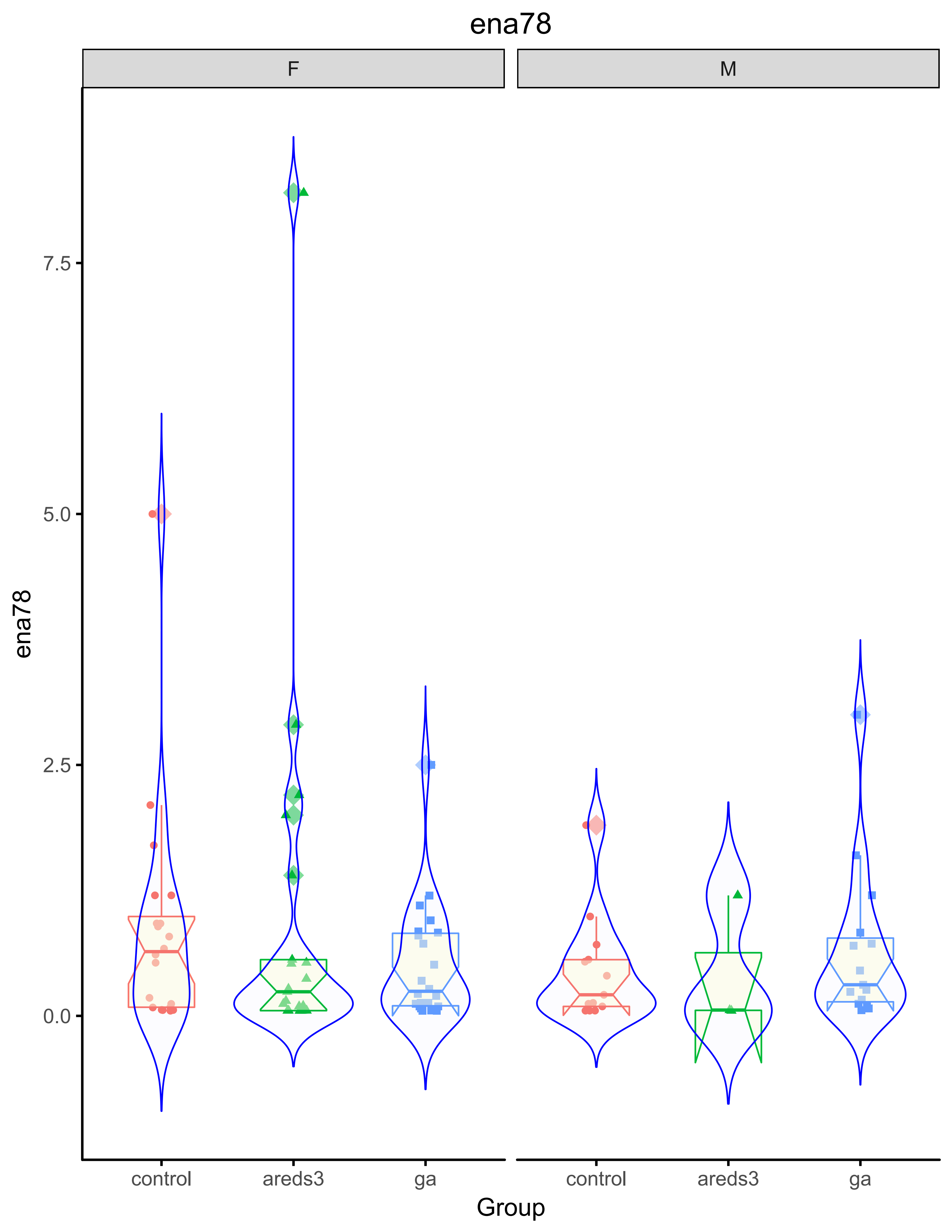
ena78
F
M
7.5
5.0
2.5
0.0
ena78
control
areds3
ga
control
areds3
ga
Group

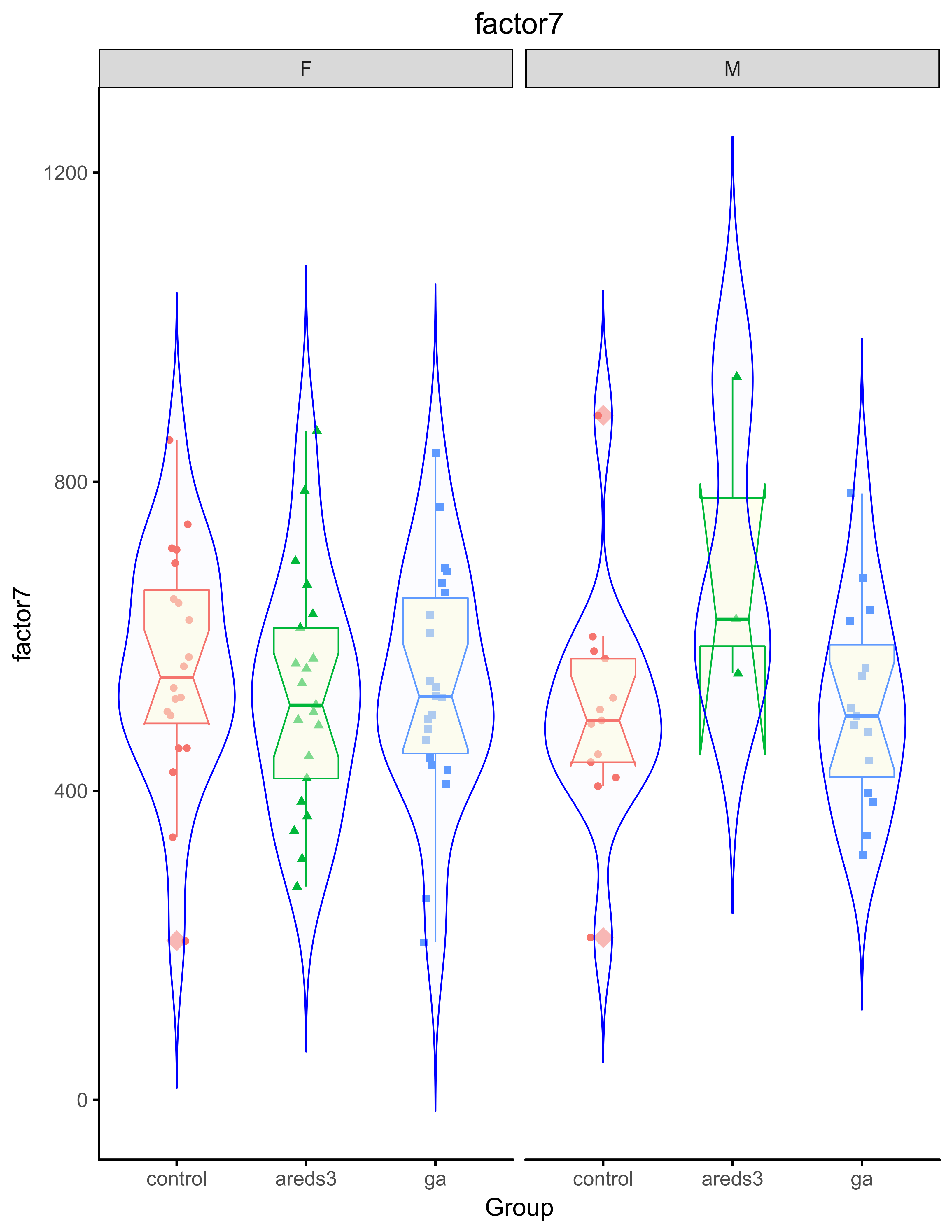
factor7
F
M
1200
800
400
0
factor7
control
areds3
ga
control
areds3
ga
Group

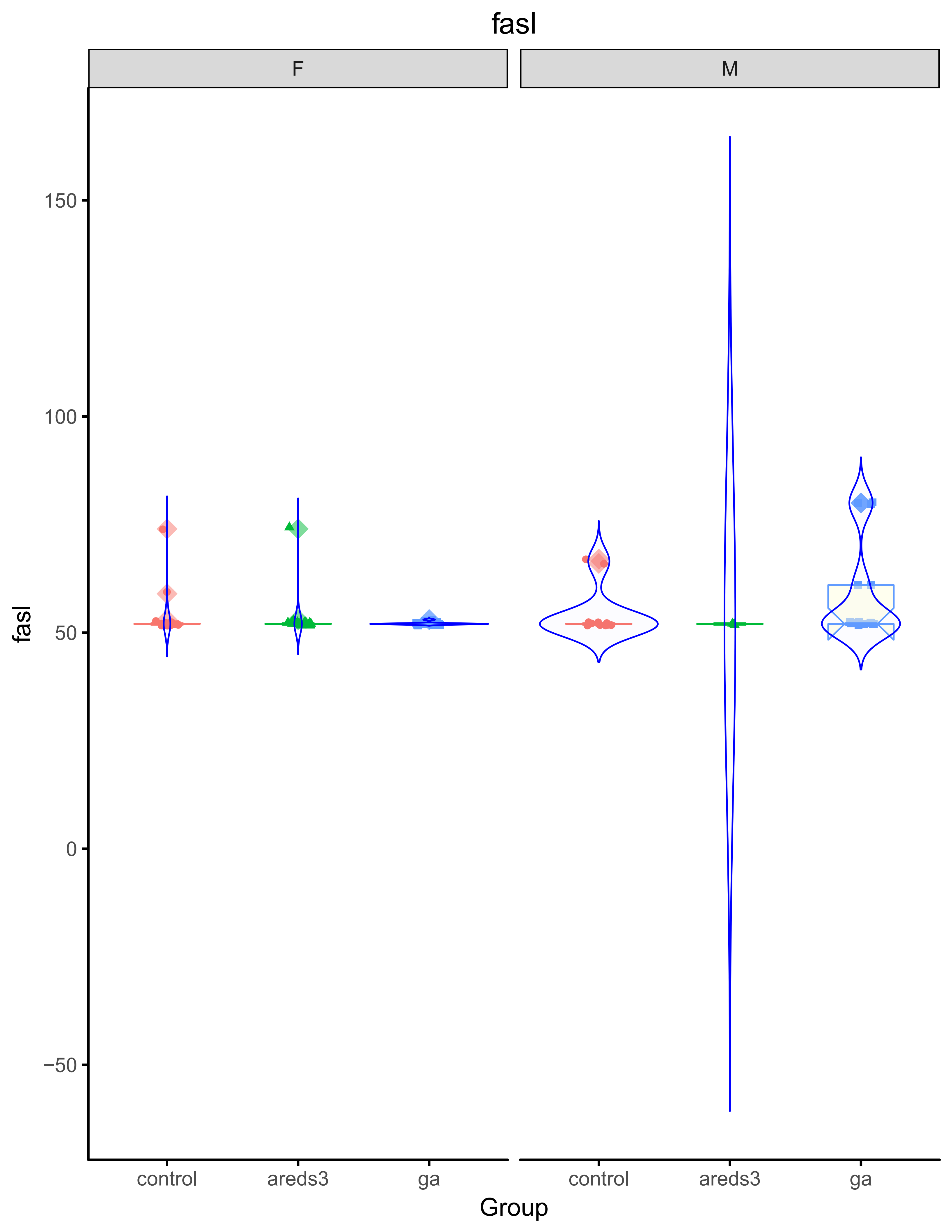
fasl
F
M
150
100
50
0
−50
fasl
control
areds3
ga
control
areds3
ga
Group

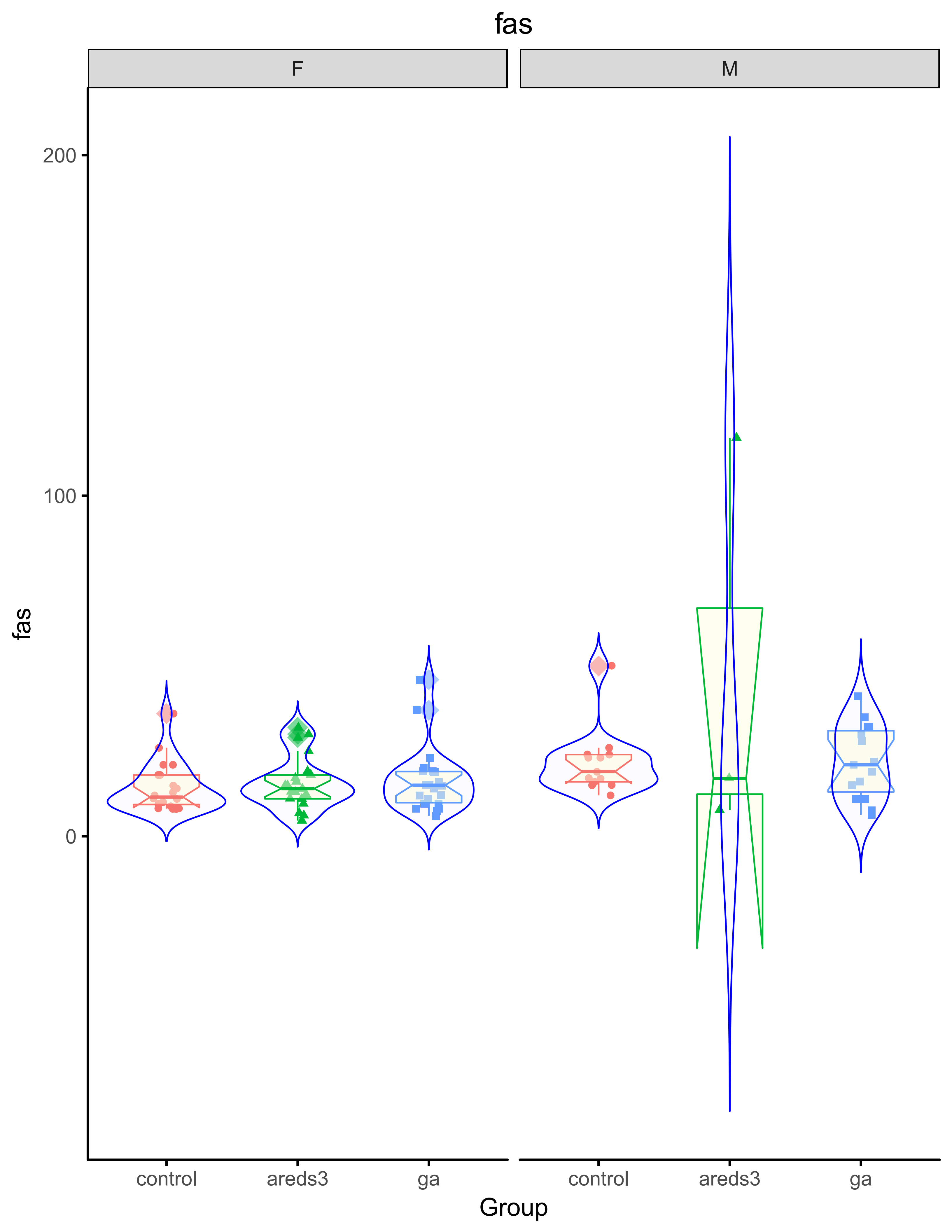
fas
F
M
200
100
0
fas
control
areds3
ga
control
areds3
ga
Group

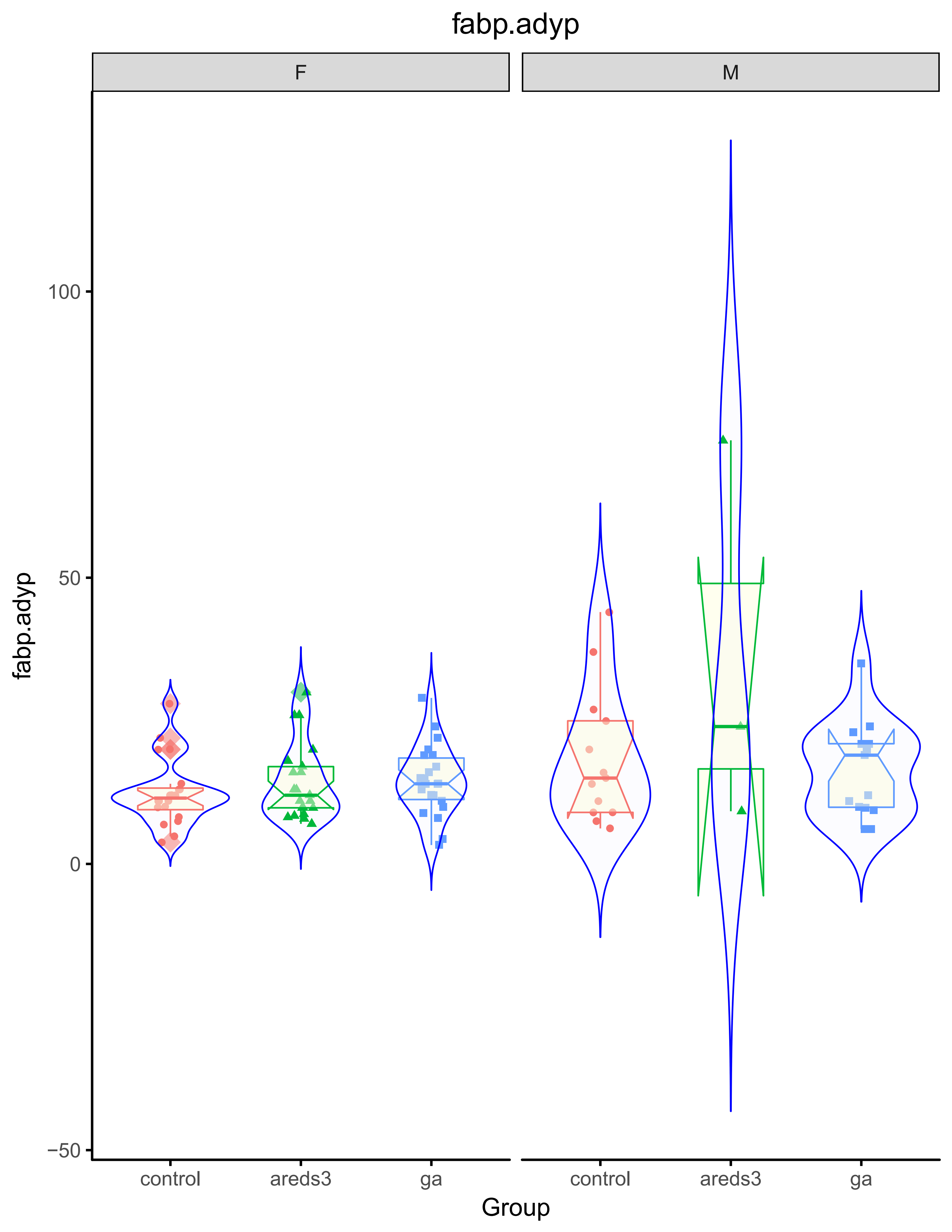
fabp.adyp
F
M
100
50
0
−50
fabp.adyp
control
areds3
ga
control
areds3
ga
Group

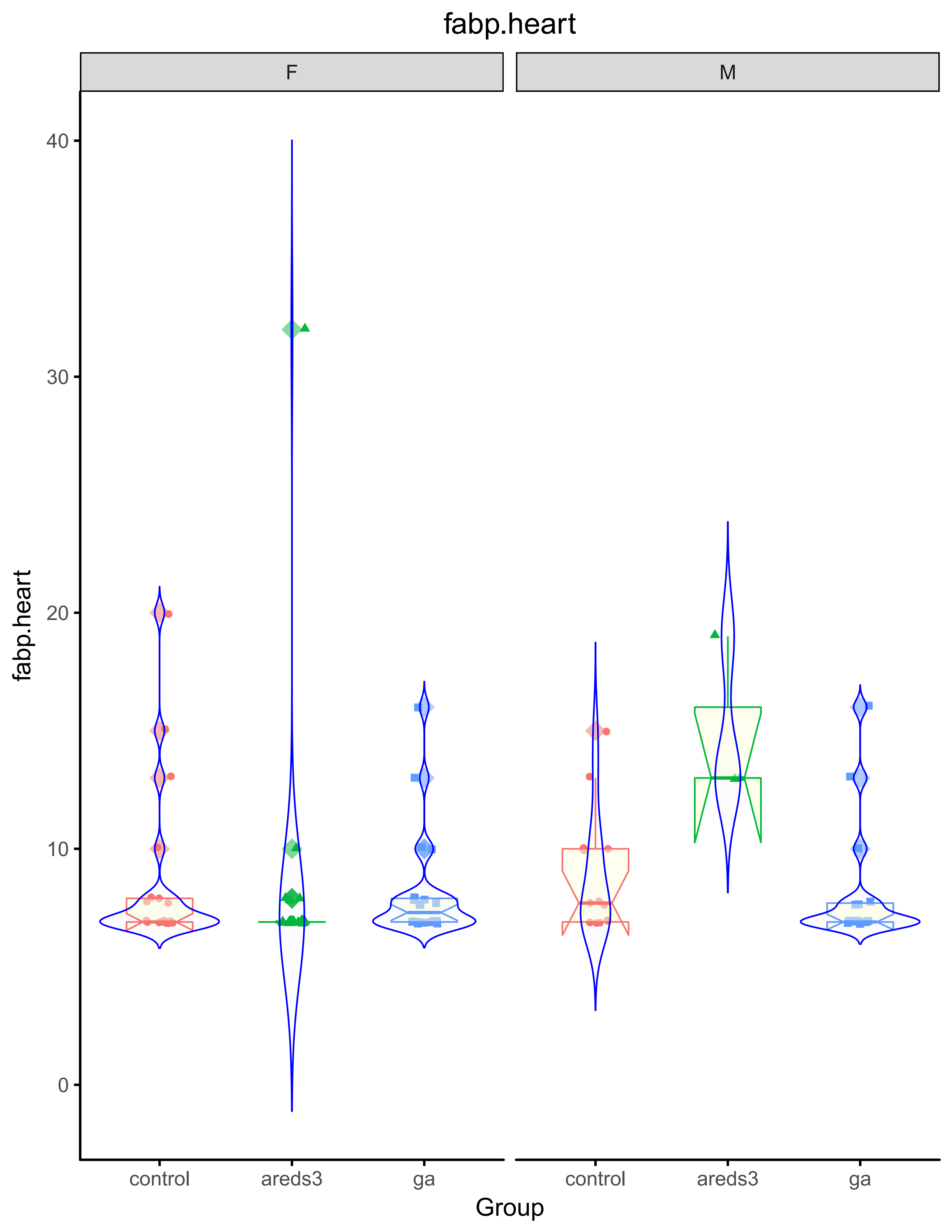
fabp.heart
F
M
fabp.heart
40
30
20
10
0
control
areds3
ga
control
areds3
ga
Group

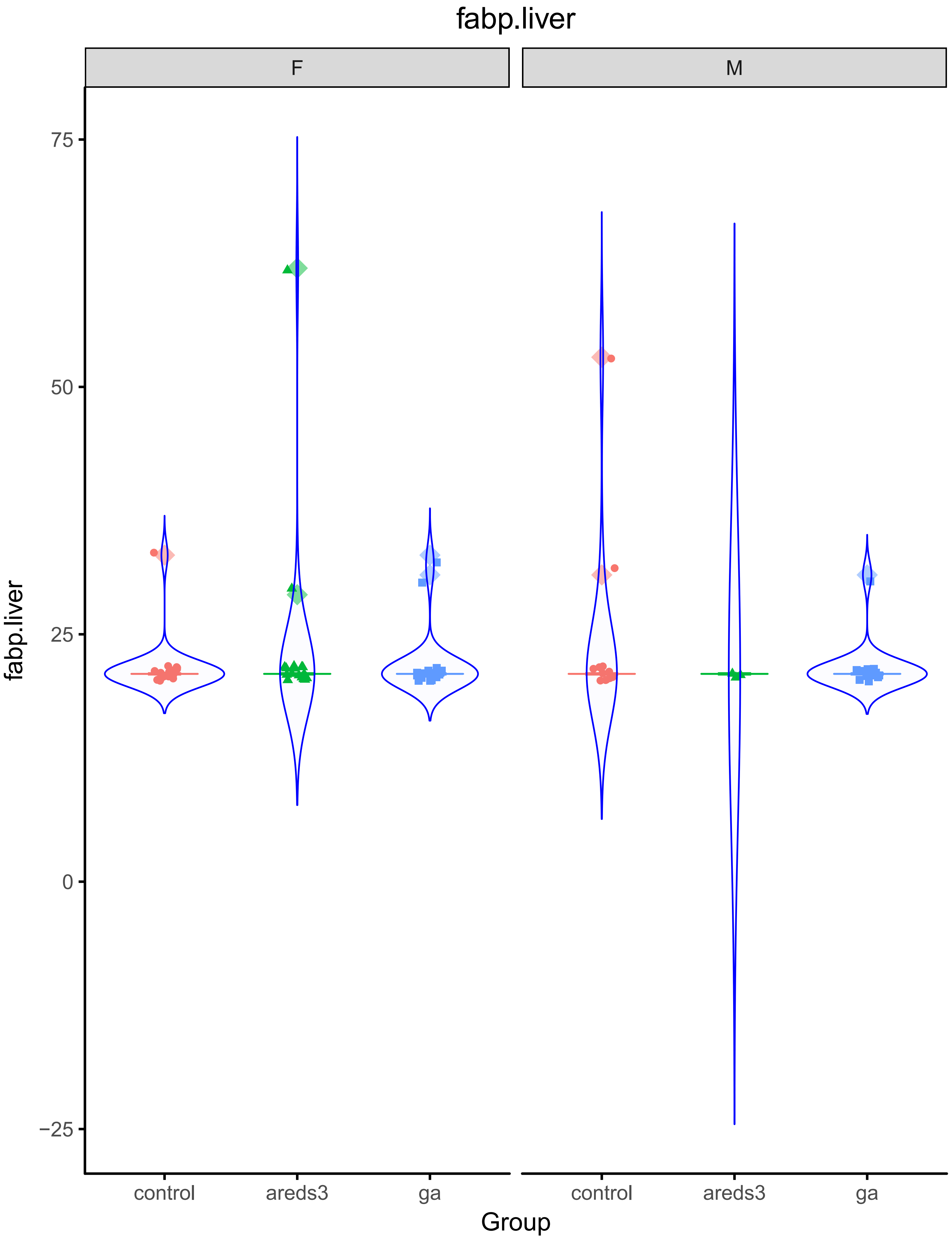
fabp.liver
F
M
75
50
25
0
−25
fabp.liver
control
areds3
ga
control
areds3
ga
Group

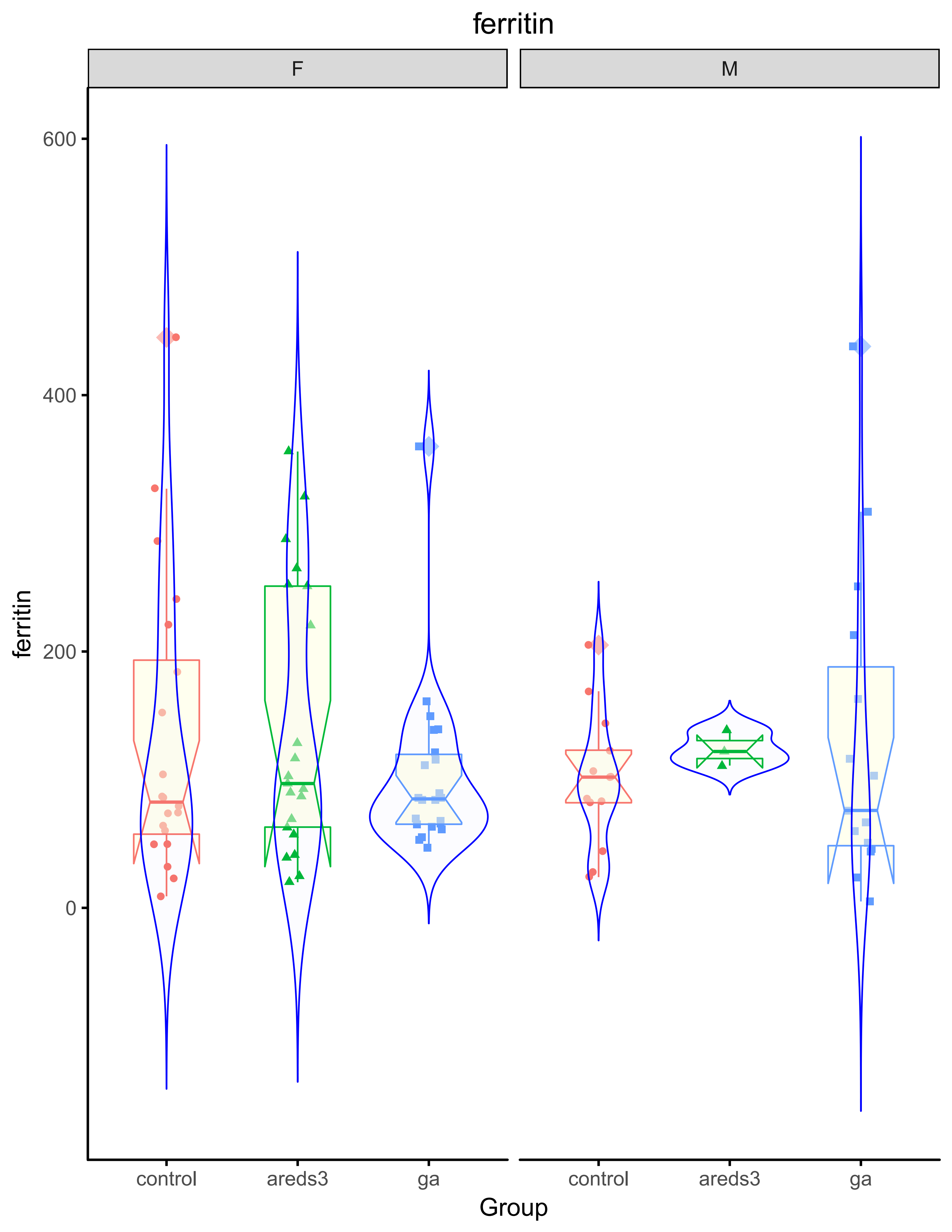

ferritin
F
M
600
400
200
0
ferritin
control
areds3
ga
control
areds3
ga
Group

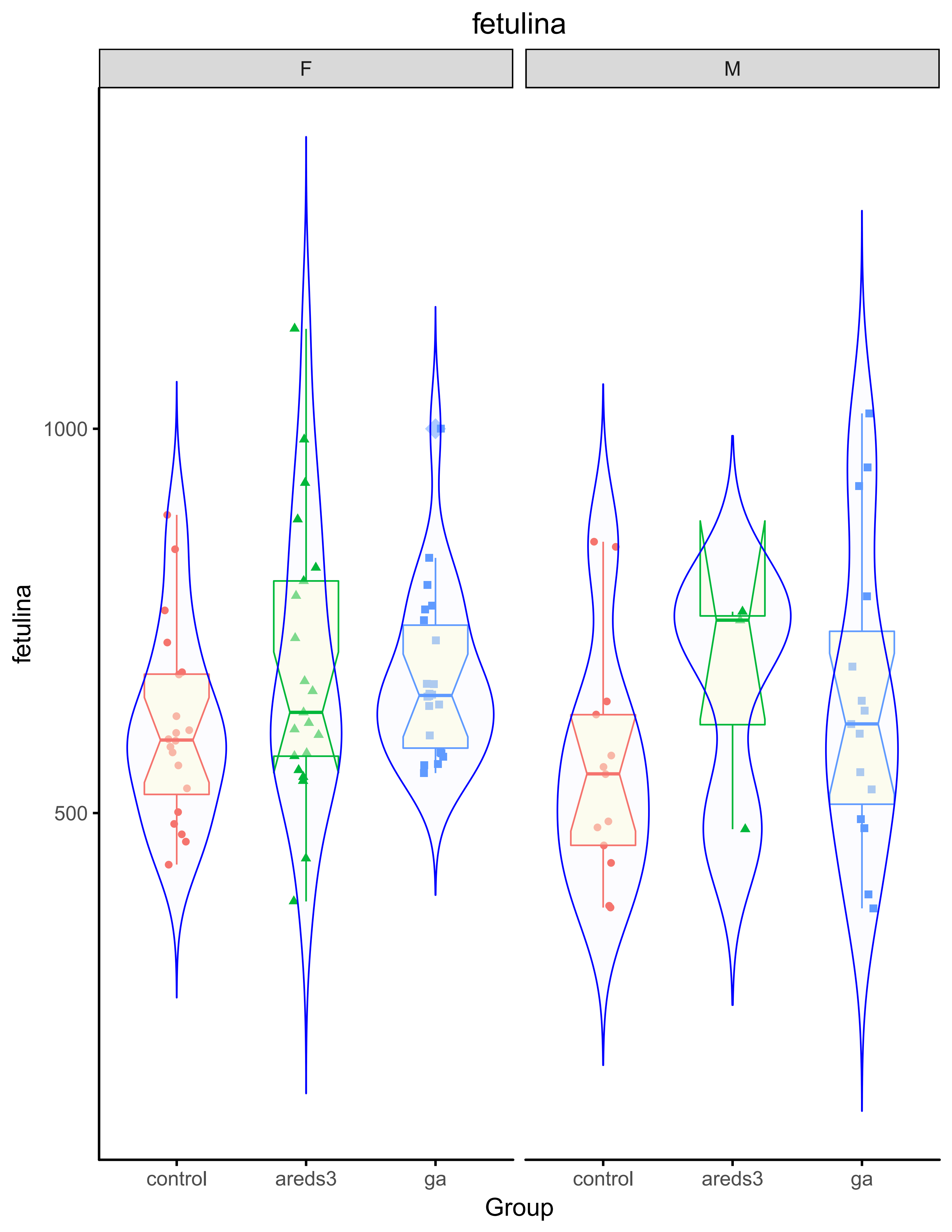
fetulina
F
M
1000
500
fetulina
control
areds3
ga
control
areds3
ga
Group

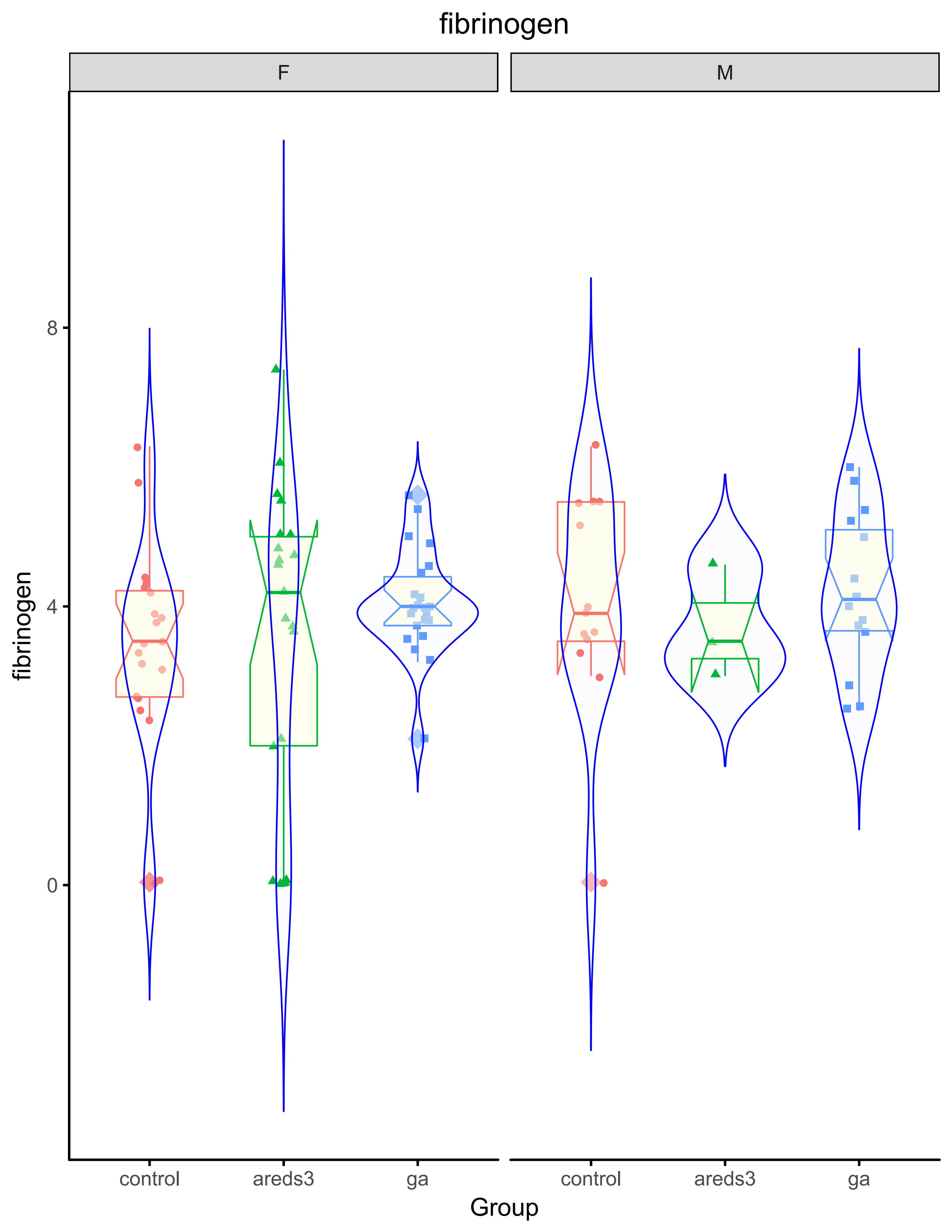
fibrinogen
F
M
8
4
0
fibrinogen
control
areds3
ga
control
areds3
ga
Group

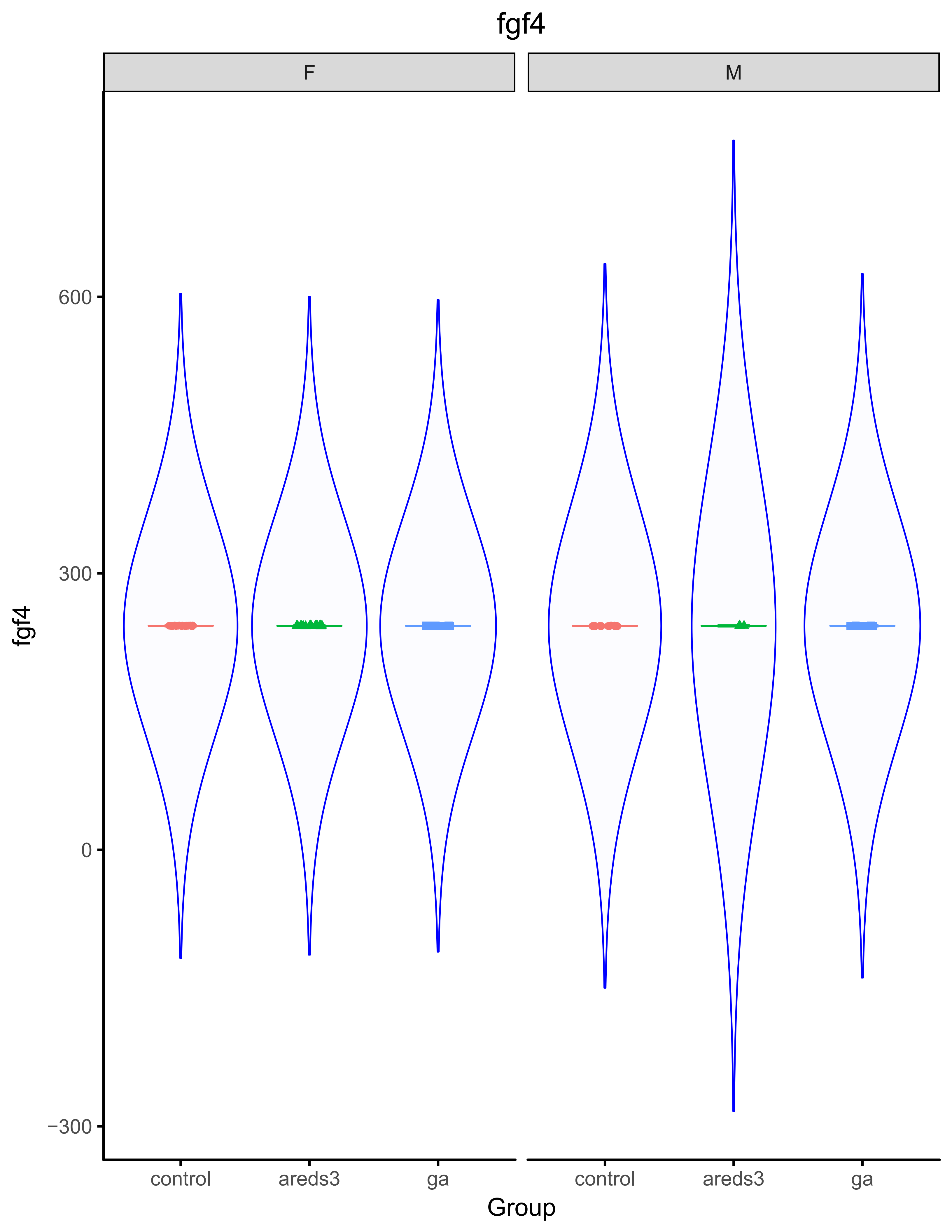
fgf4
F
M
600
300
0
−300
fgf4
control
areds3
ga
control
areds3
ga
Group

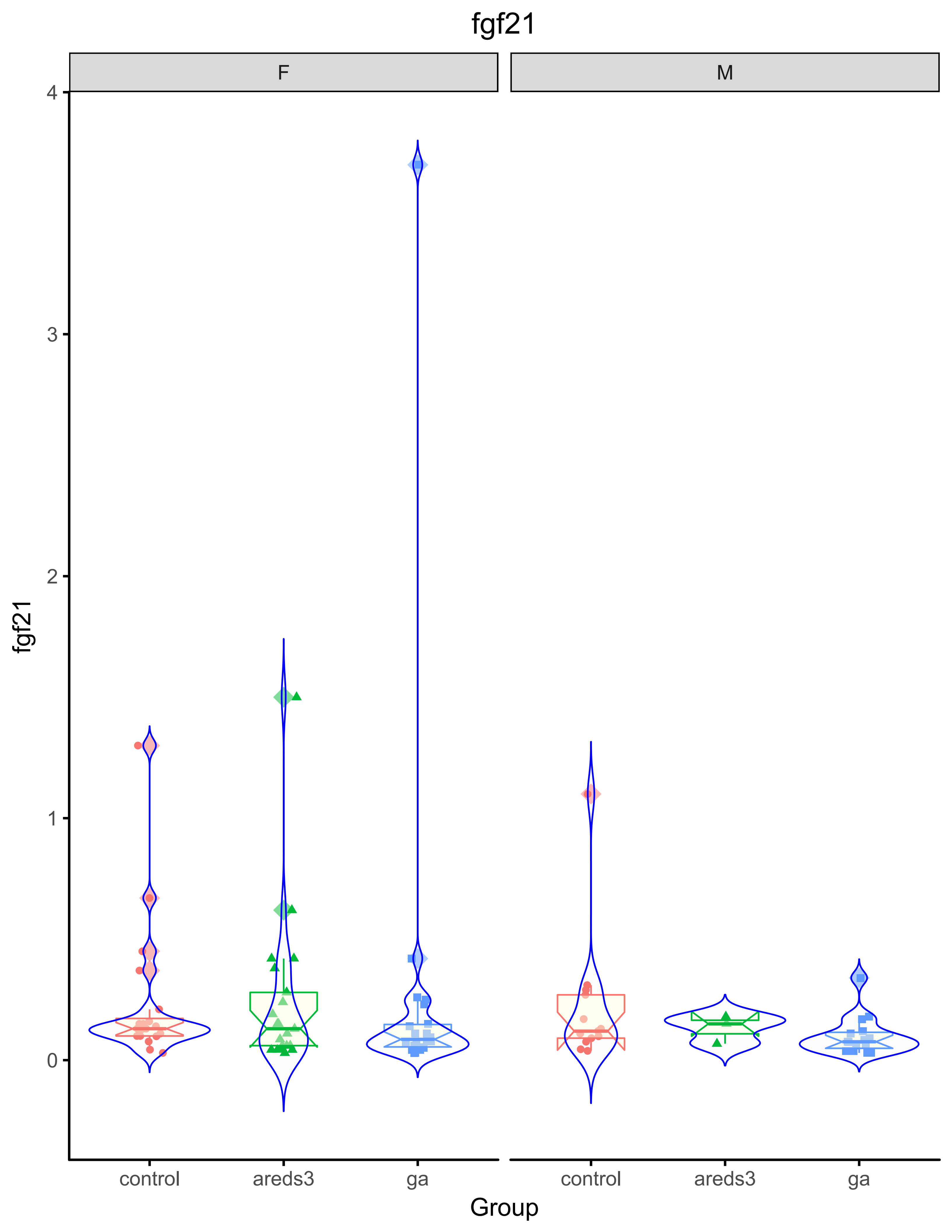
fgf21
F
M
fgf21
4
3
2
1
0
control
areds3
ga
control
areds3
ga
Group

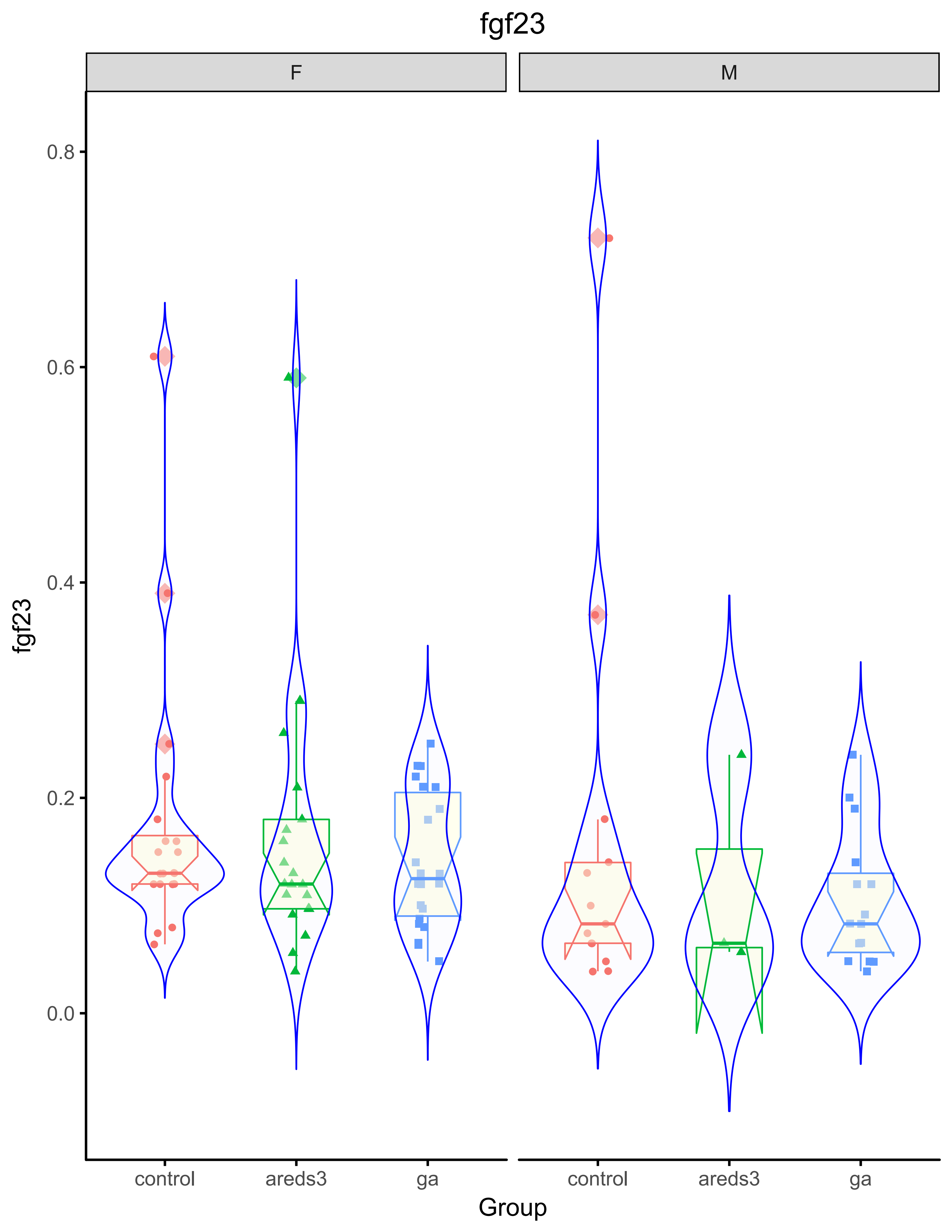
fgf23
F
M
0.8
0.6
0.4
0.2
0.0
fgf23
control
areds3
ga
control
areds3
ga
Group

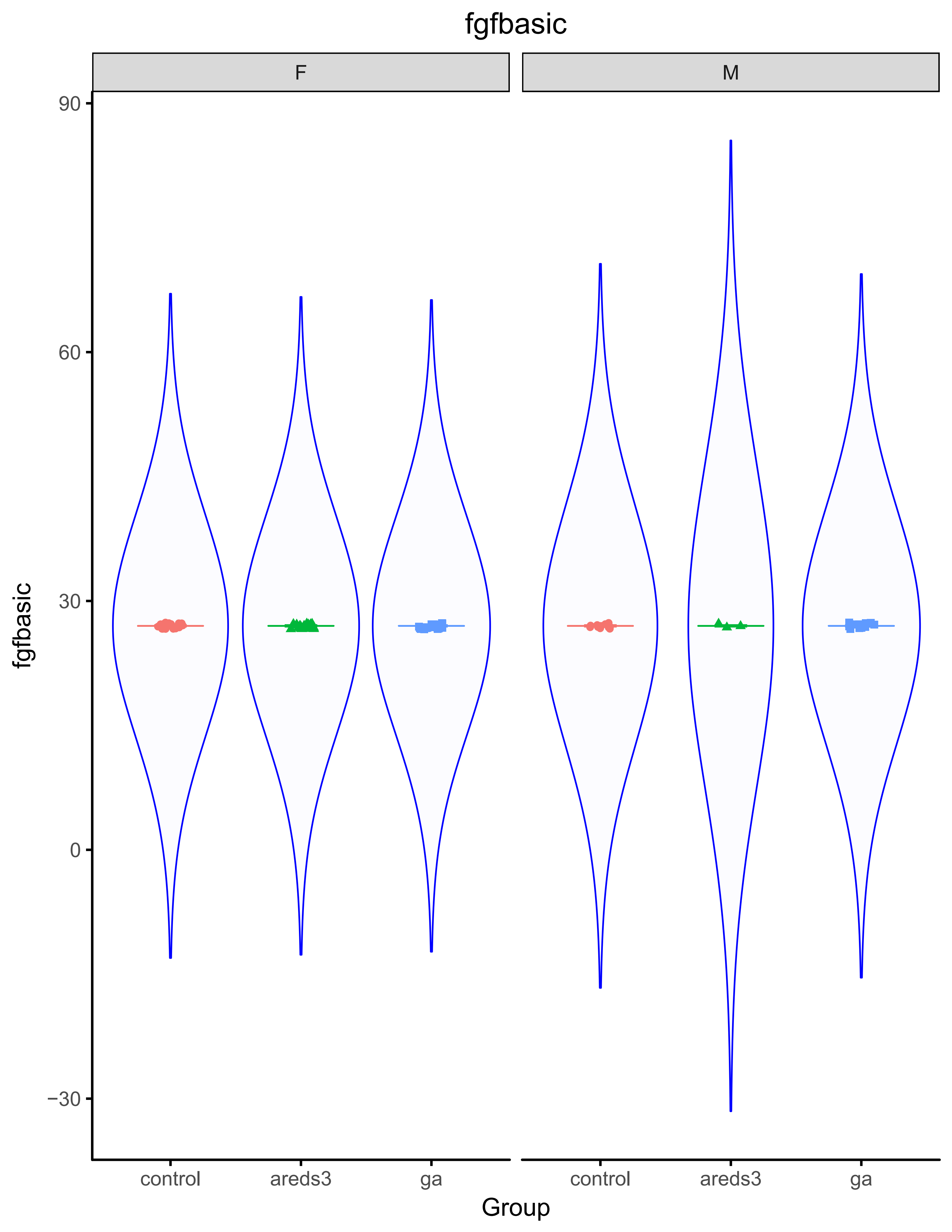

fgfbasic
F
M
90
60
30
0
−30
fgfbasic
control
areds3
ga
control
areds3
ga
Group

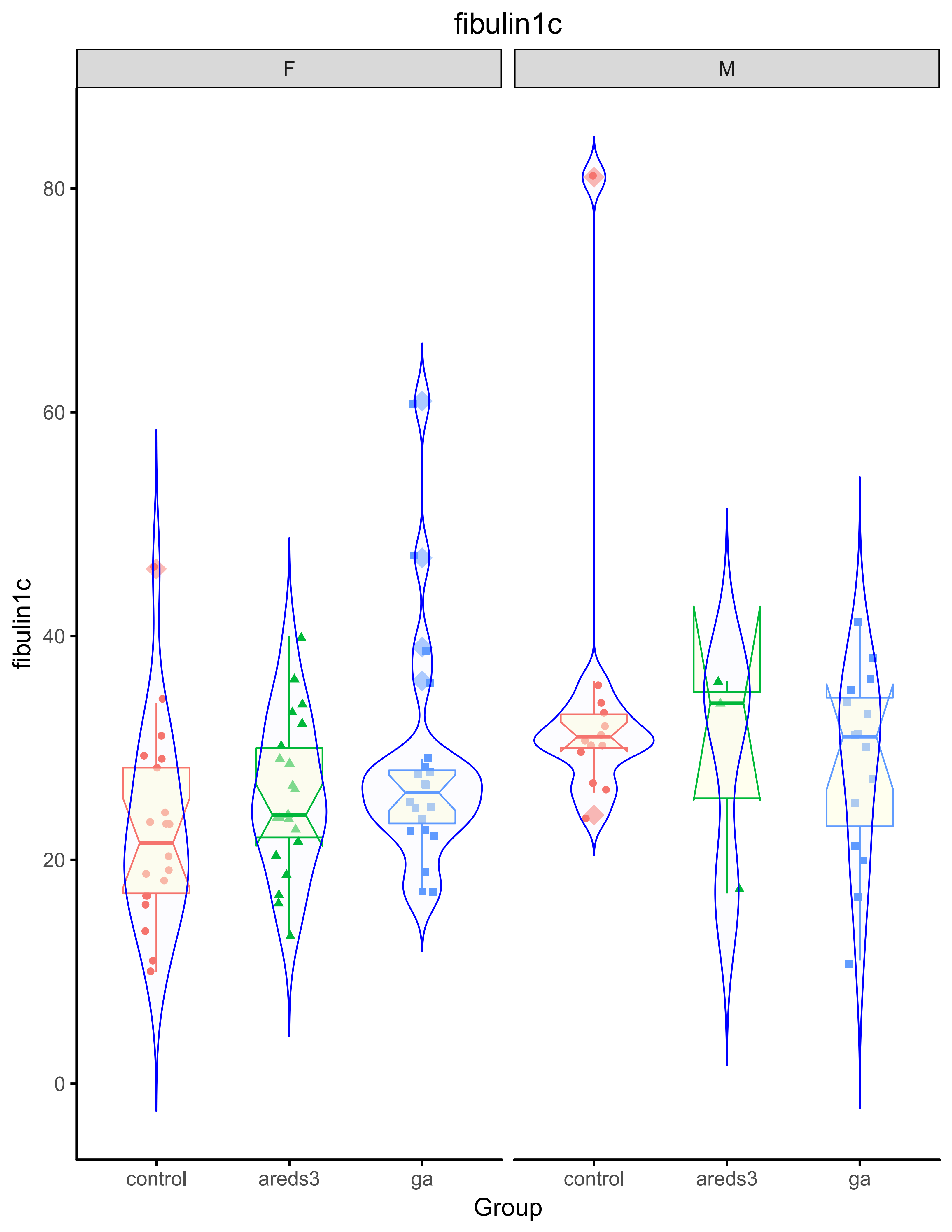
fibulin1c
F
M
80
60
40
20
0
fibulin1c
control
areds3
ga
control
areds3
ga
Group

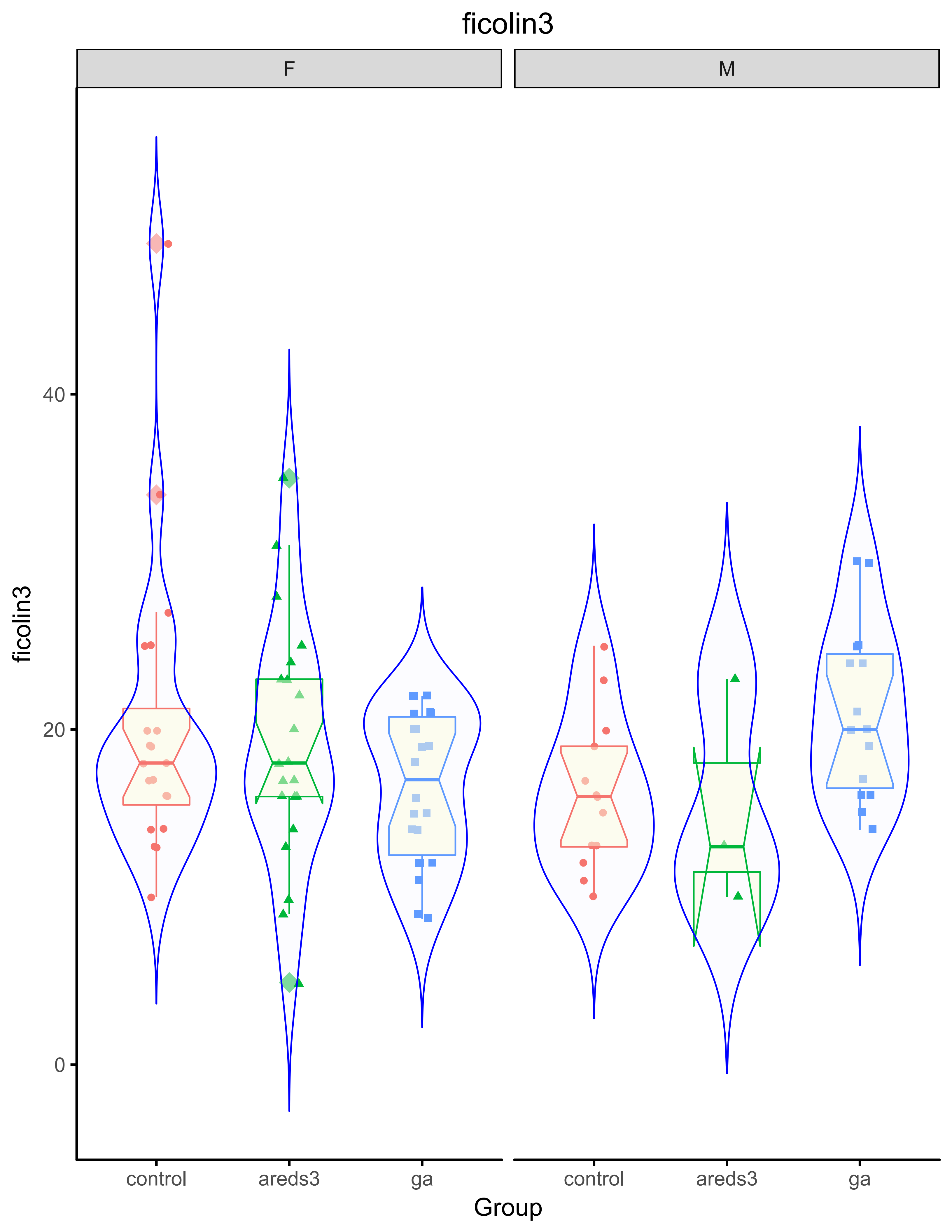
ficolin3
F
M
40
20
0
ficolin3
control
areds3
ga
control
areds3
ga
Group

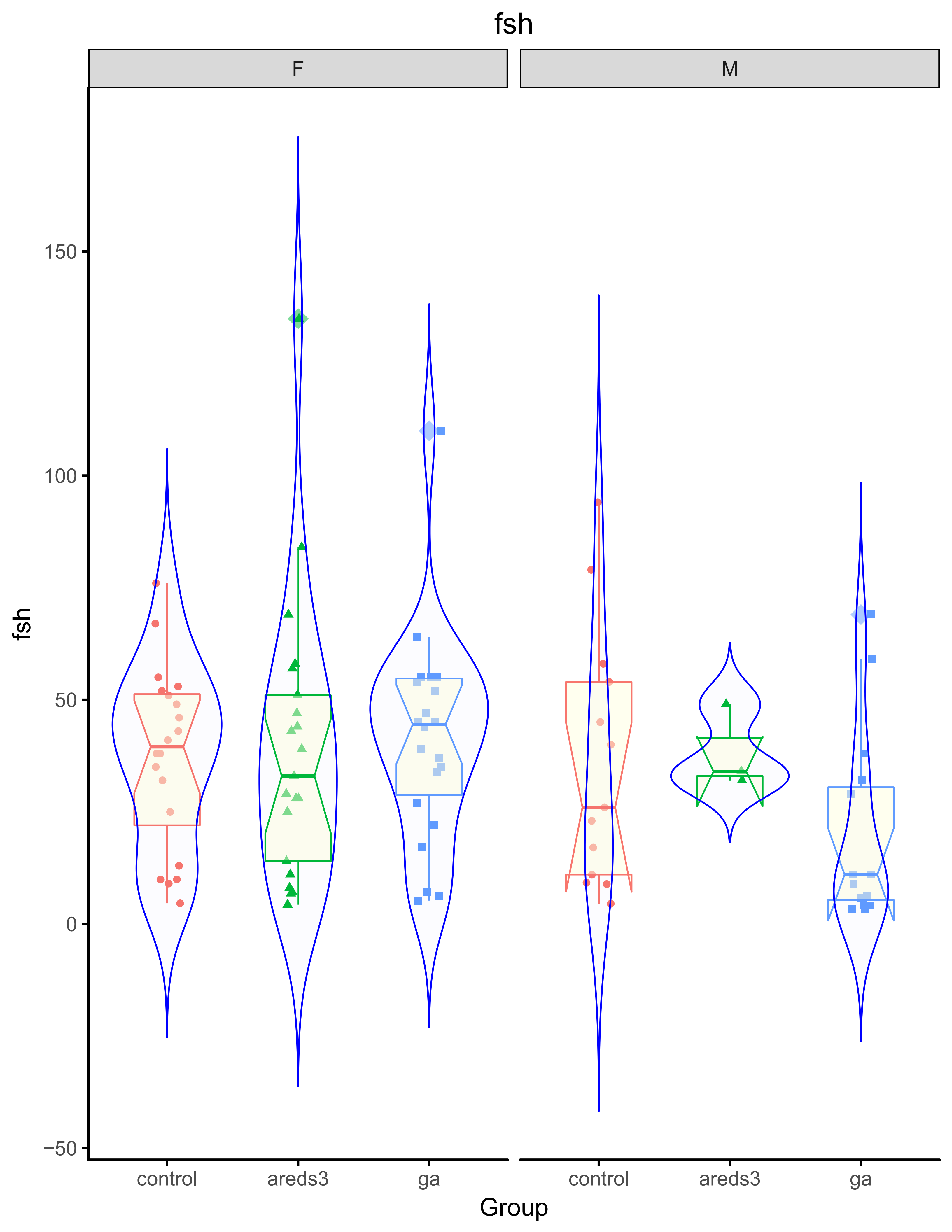
fsh
F
M
150
100
50
0
−50
fsh
control
areds3
ga
control
areds3
ga
Group

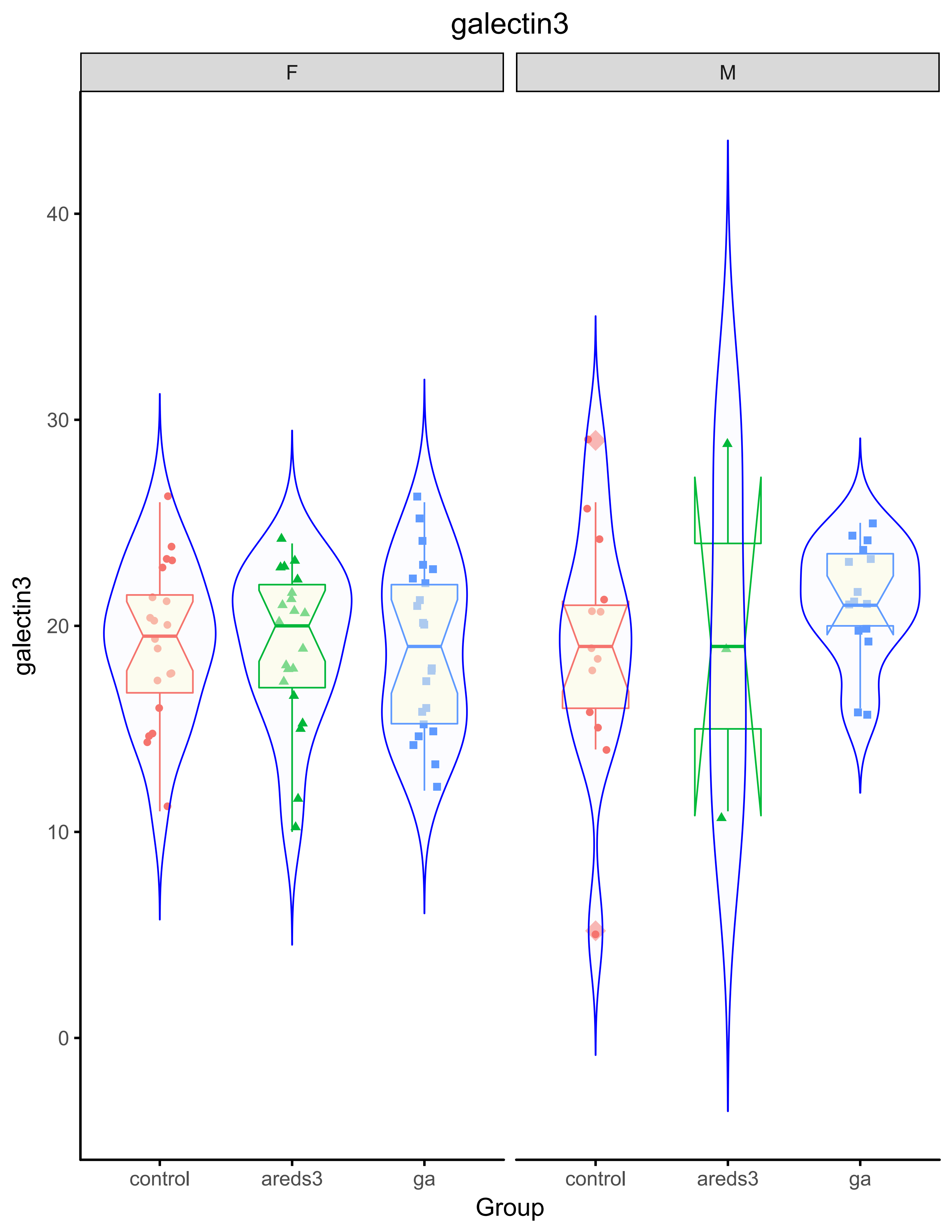

galectin3
F
M
40
30
20
10
0
galectin3
control
areds3
ga
control
areds3
ga
Group

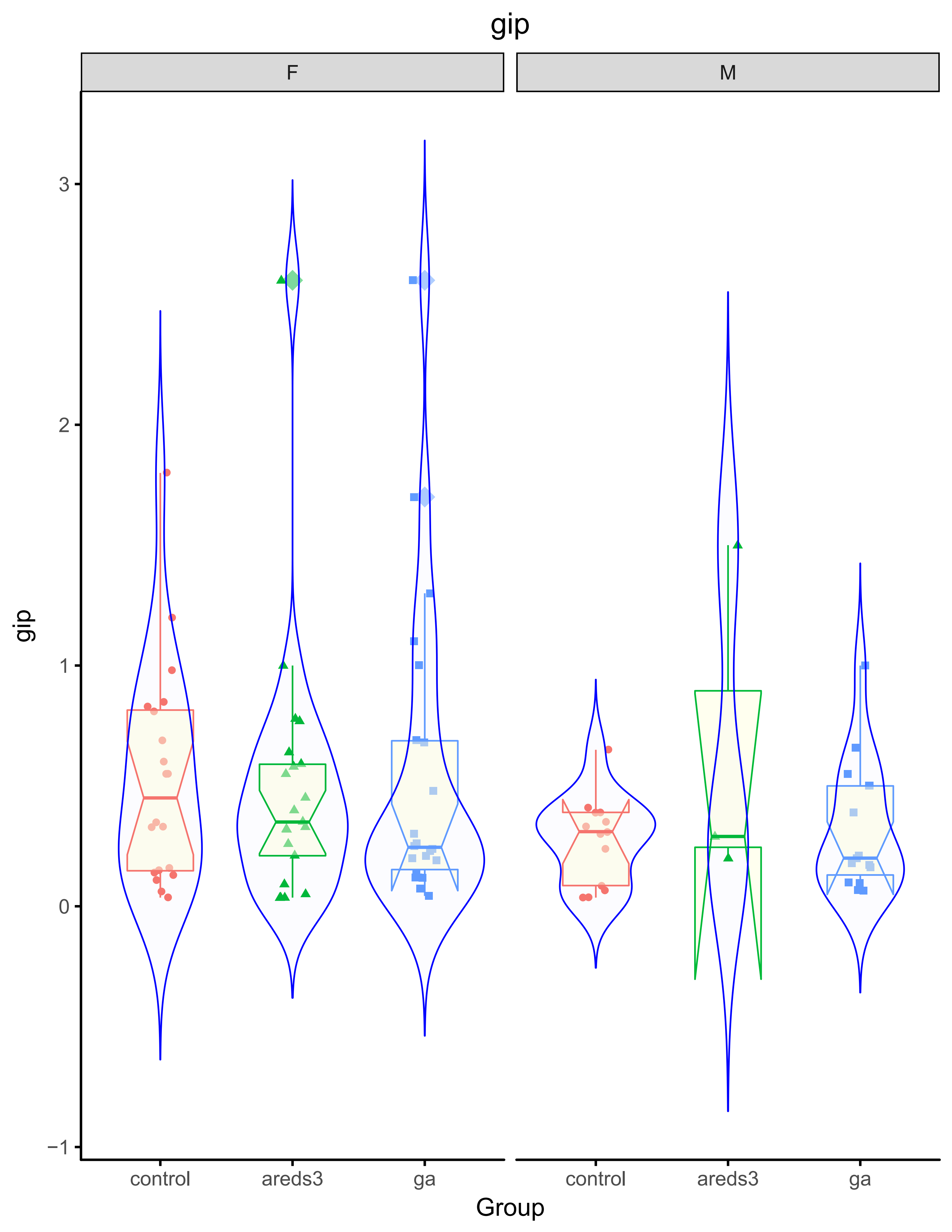

gip
F
M
3
2
1
0
−1
gip
control
areds3
ga
control
areds3
ga
Group

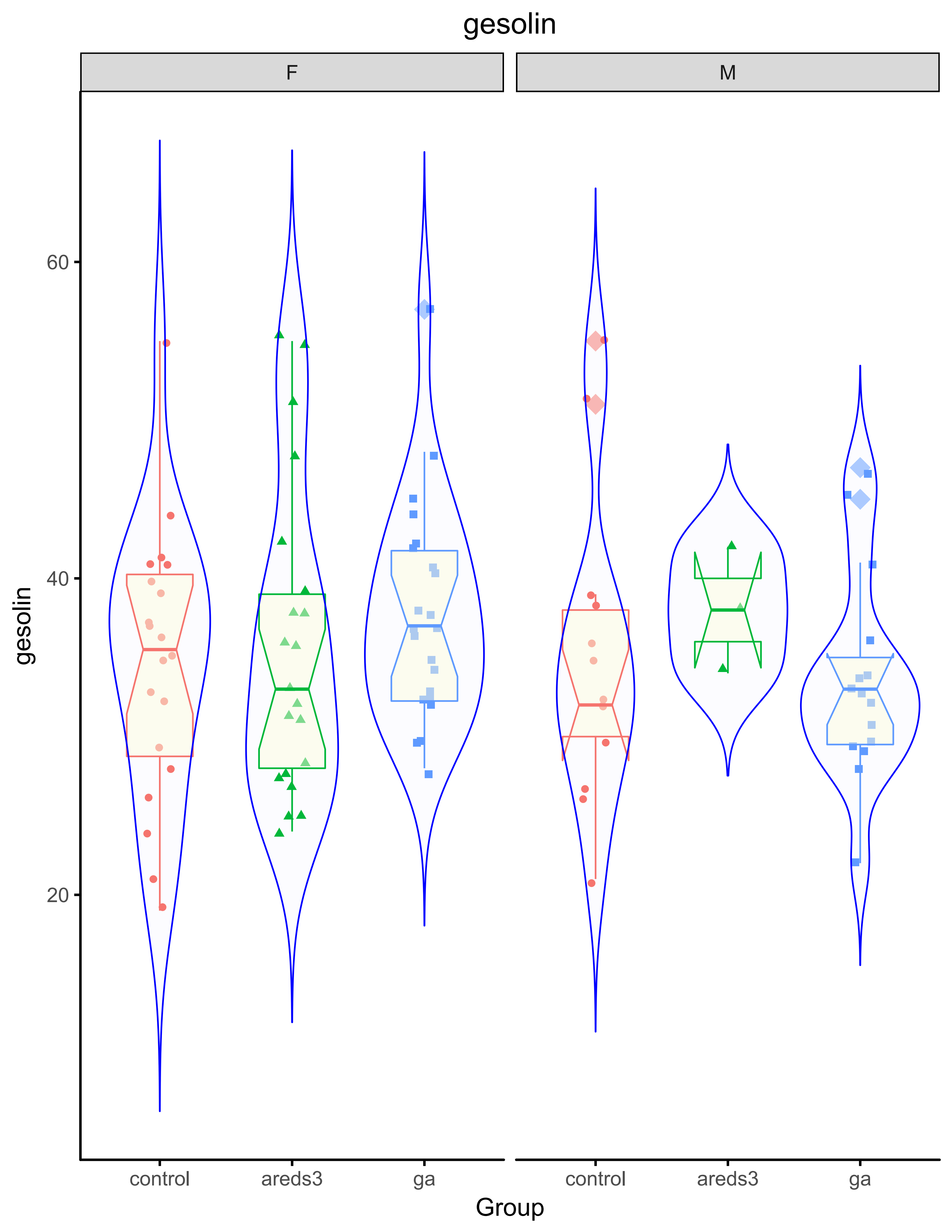

gesolin
F
M
60
40
20
gesolin
control
areds3
ga
control
areds3
ga
Group

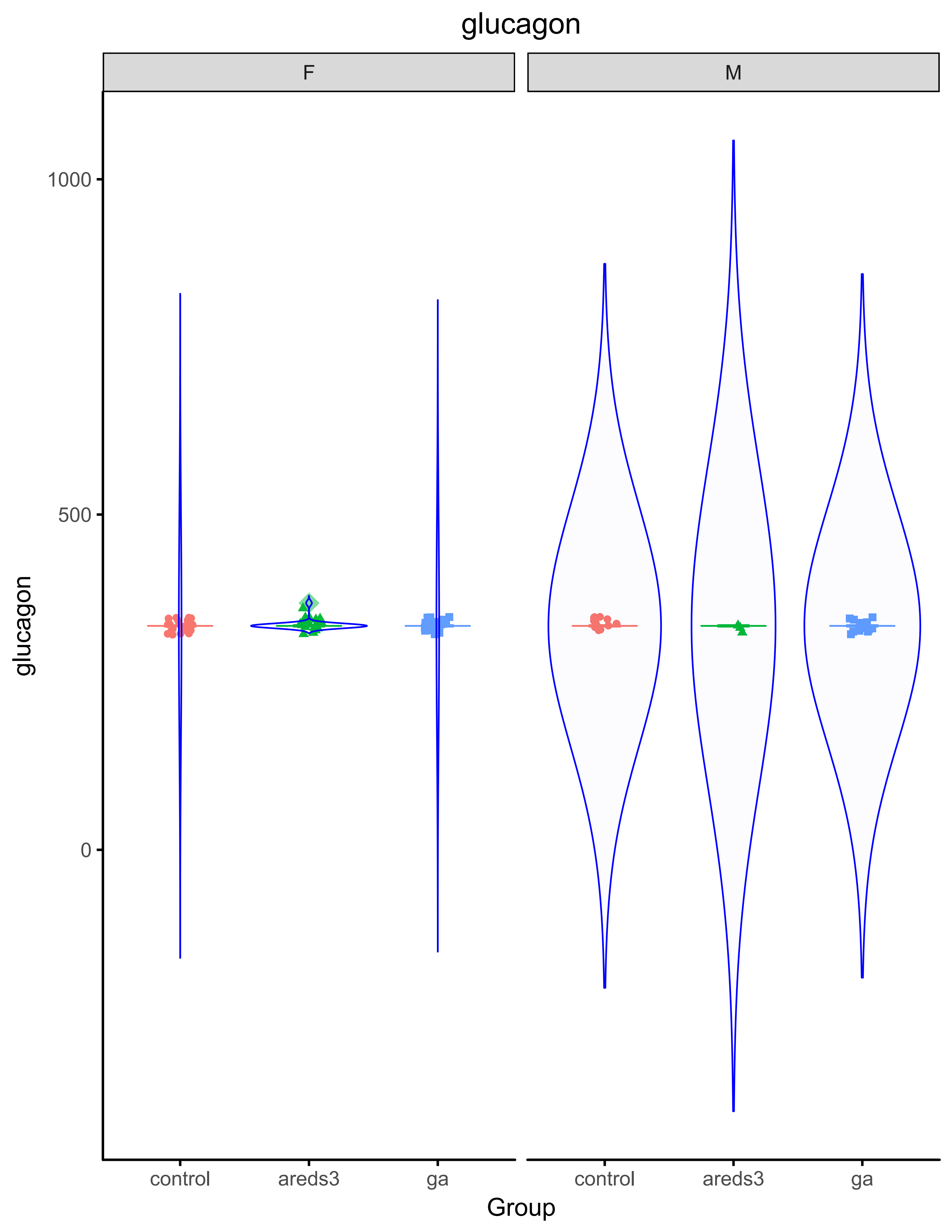
glucagon
F
M
1000
500
0
glucagon
control
areds3
ga
control
areds3
ga
Group

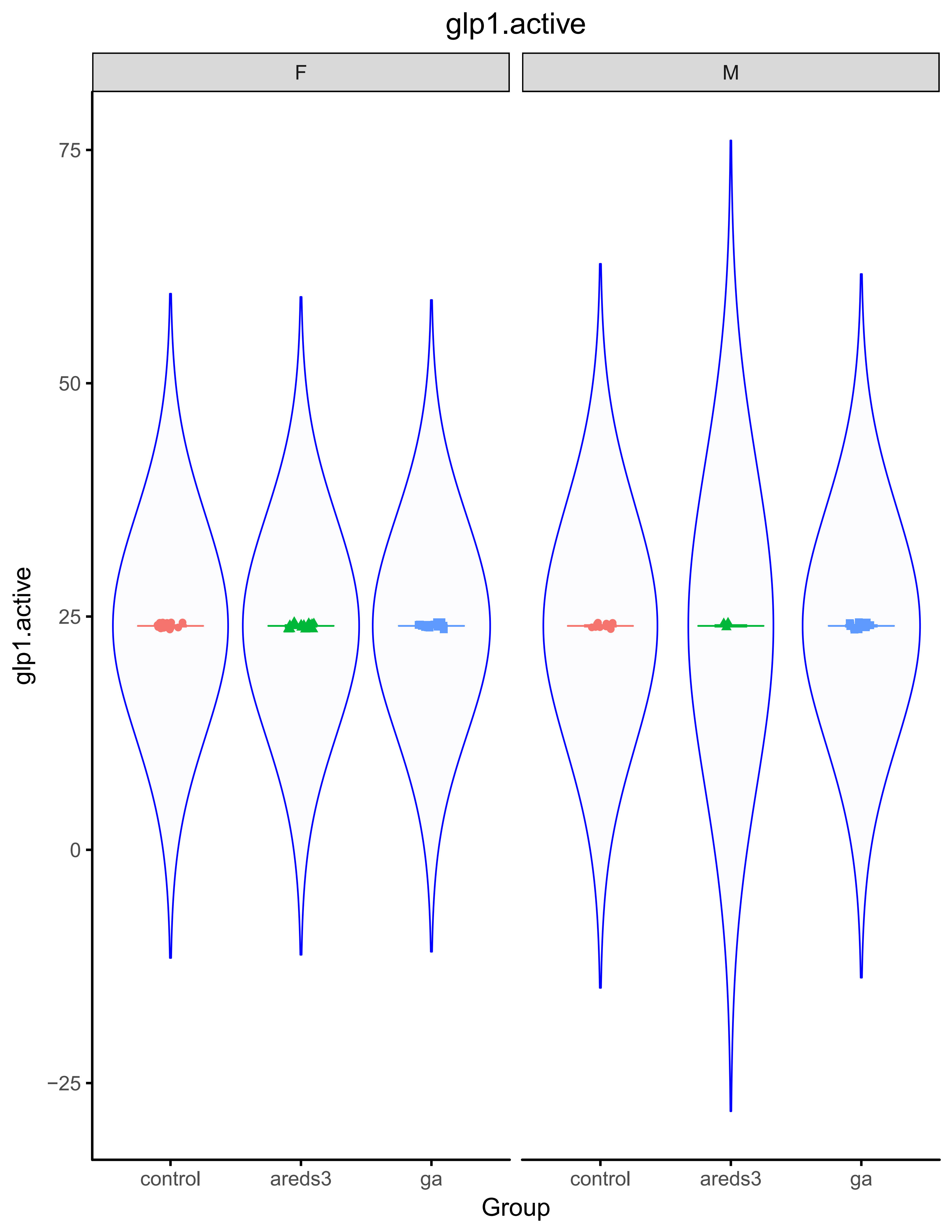
glp1.active
F
M
75
50
25
0
−25
glp1.active
control
areds3
ga
control
areds3
ga
Group

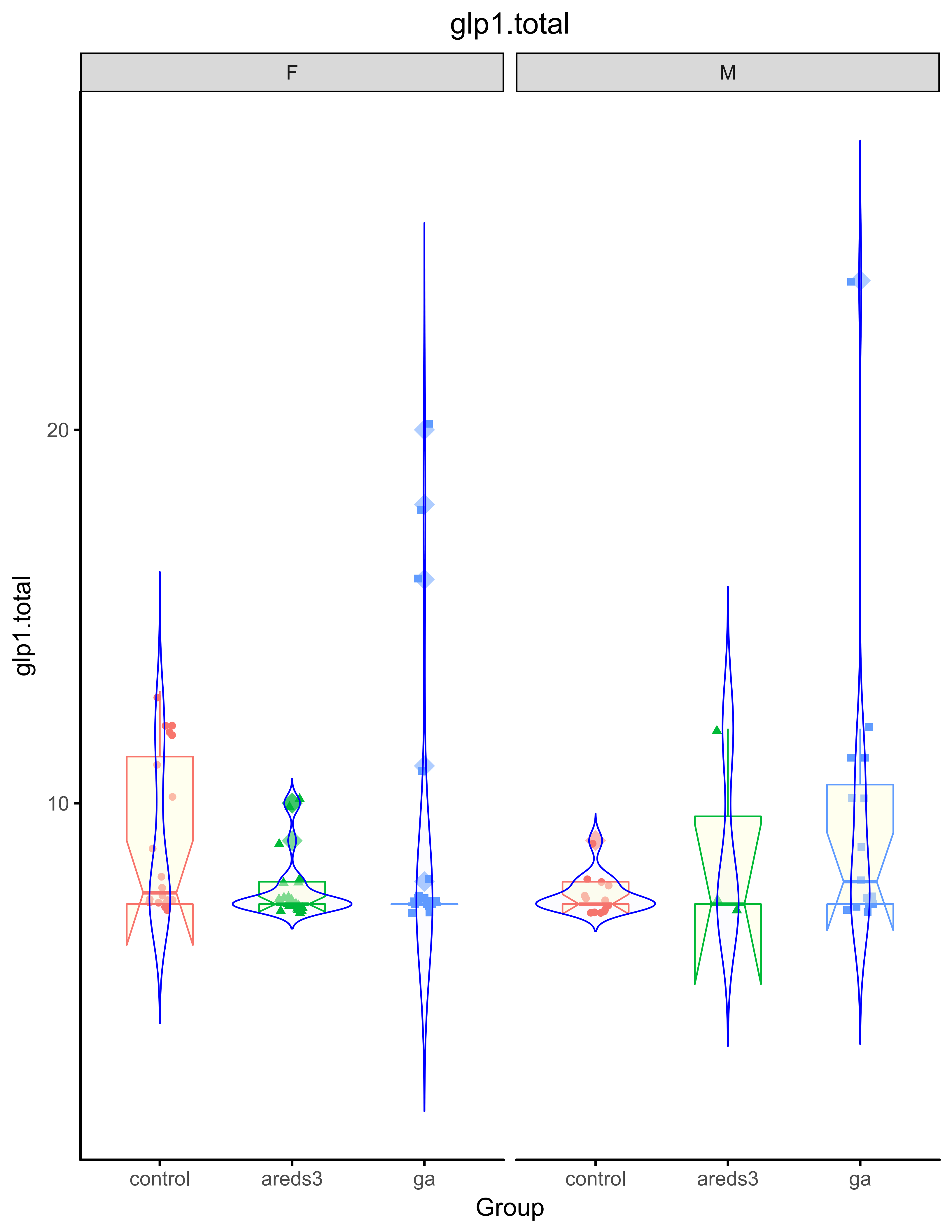

glp1.total
F
M
20
10
glp1.total
control
areds3
ga
control
areds3
ga
Group

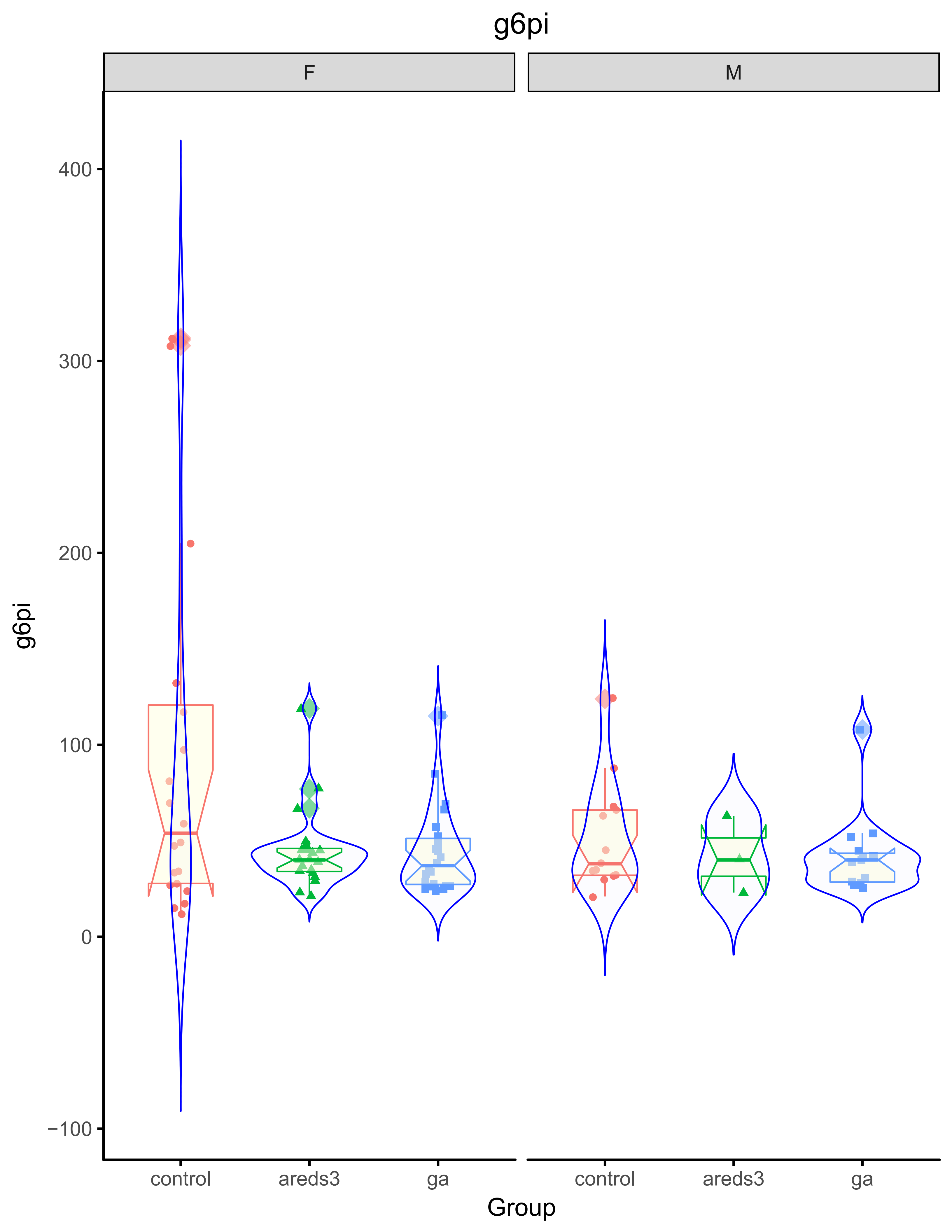

g6pi
F
M
400
300
200
100
0
−100
g6pi
control
areds3
ga
control
areds3
ga
Group

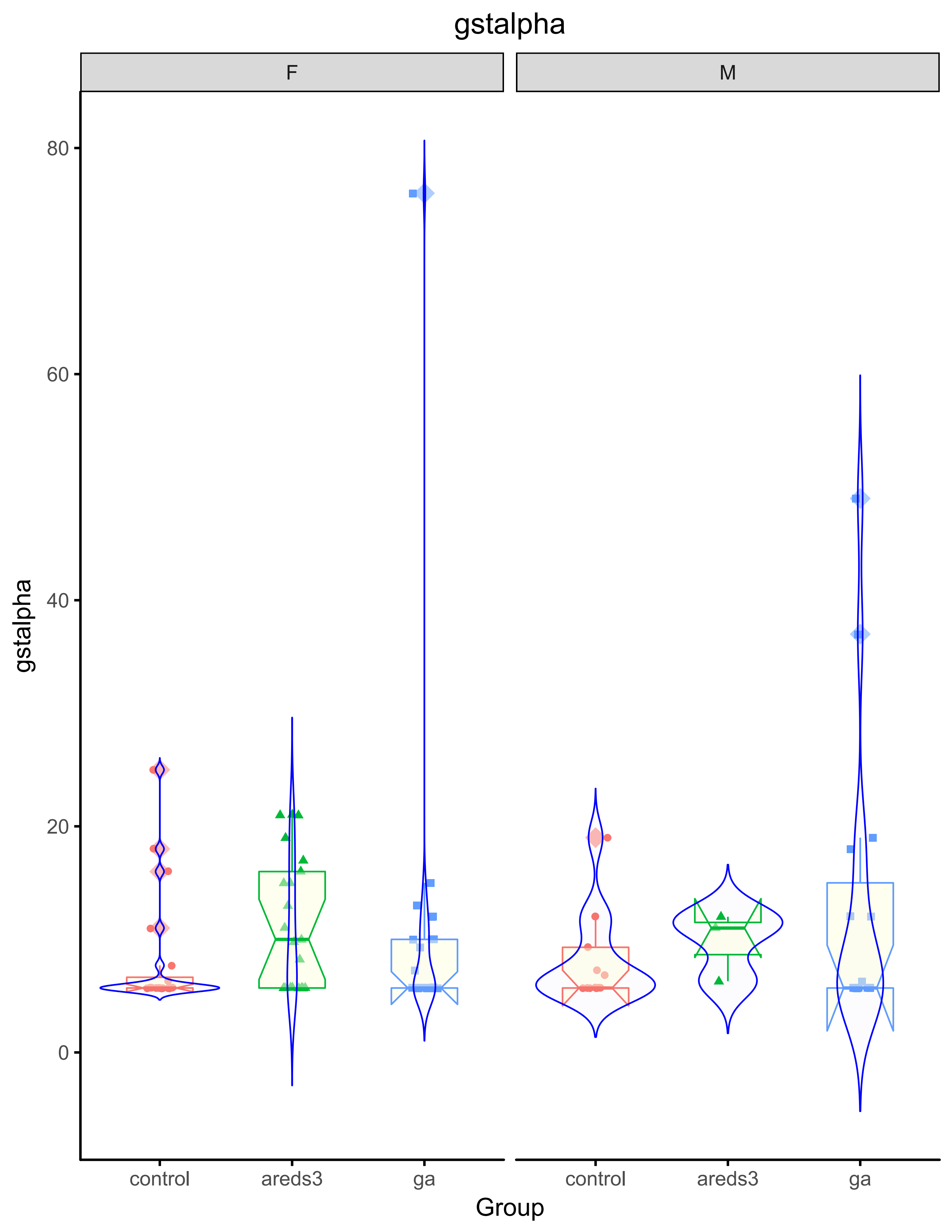

gstalpha
F
M
80
60
40
20
0
gstalpha
control
areds3
ga
control
areds3
ga
Group

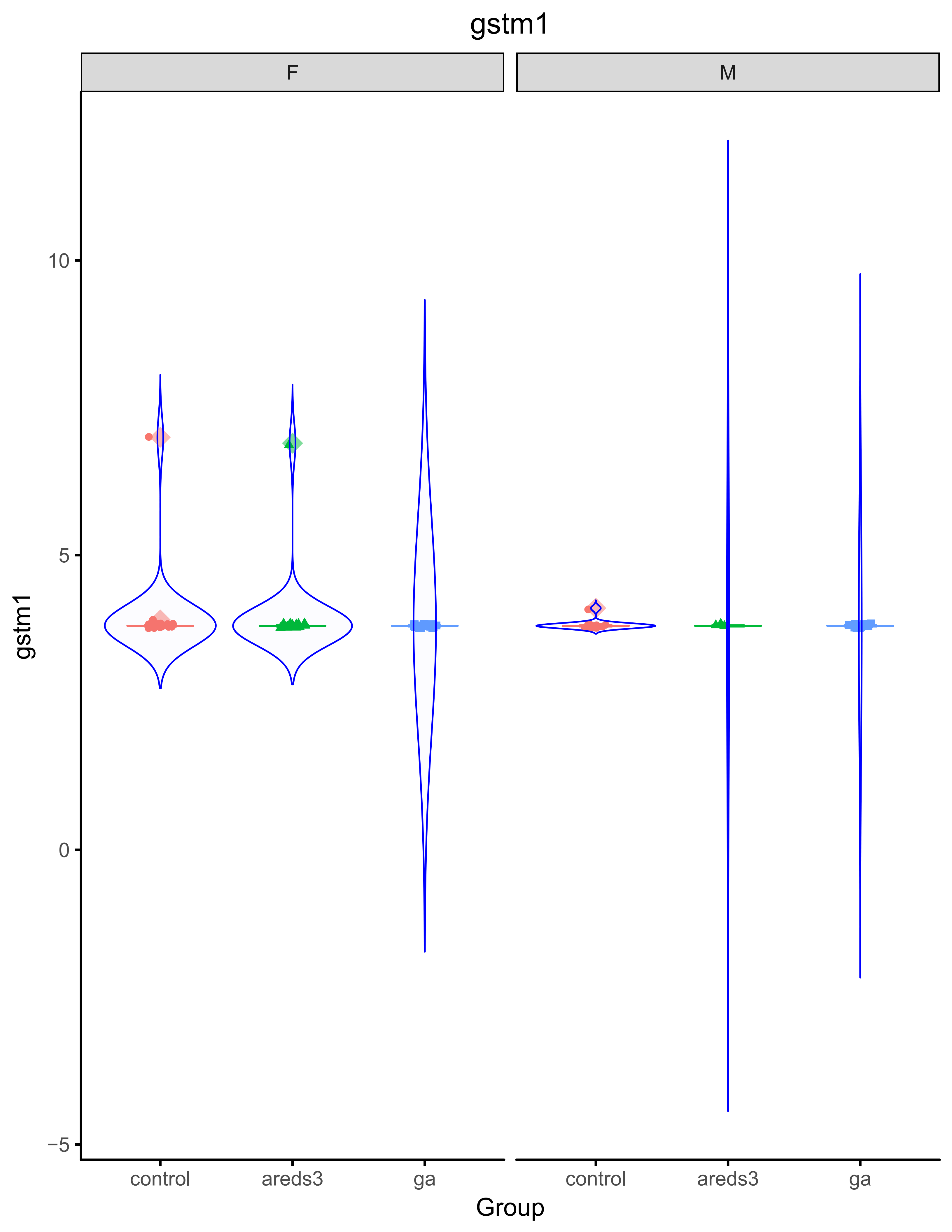
gstm1
F
M
10
5
0
−5
gstm1
control
areds3
ga
control
areds3
ga
Group

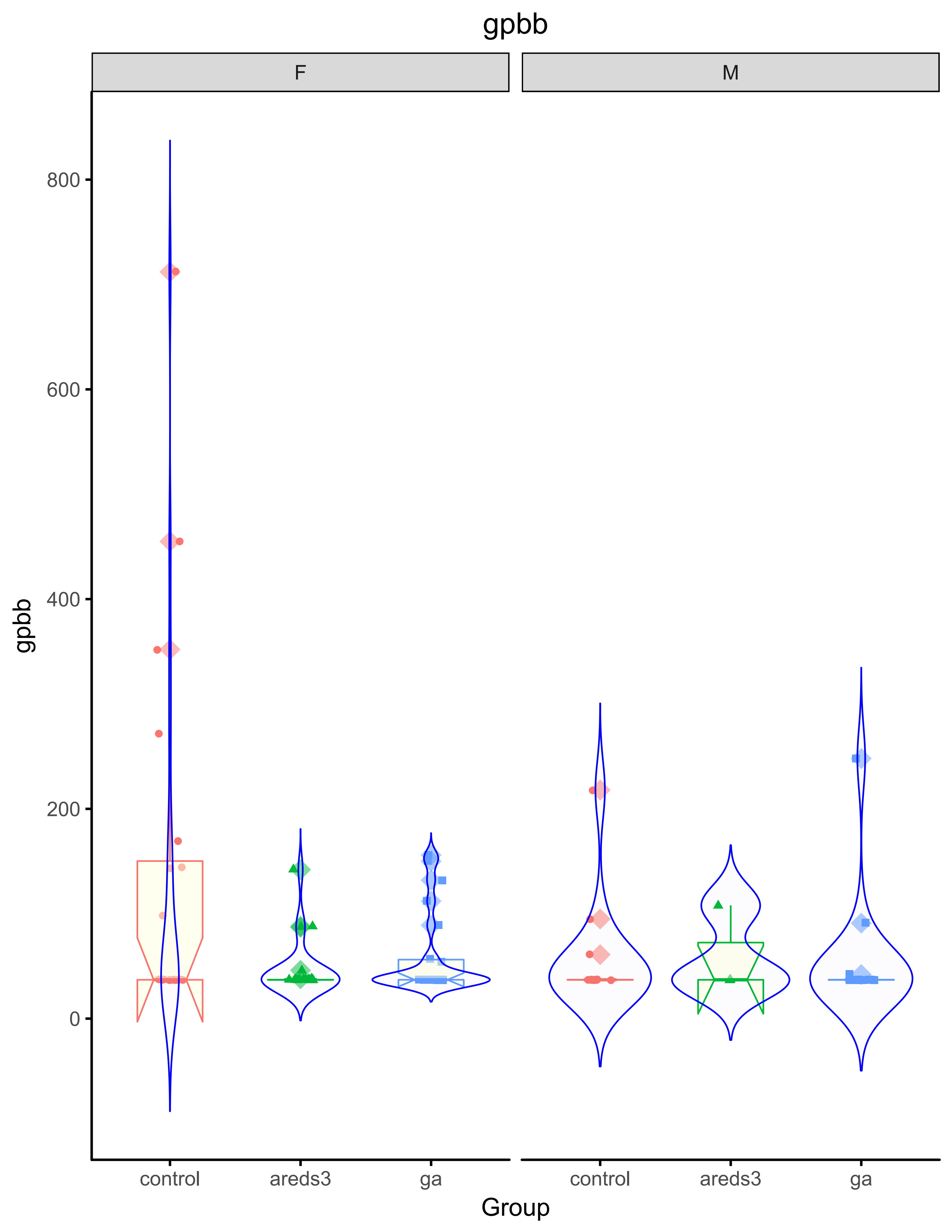
gpbb
F
M
800
600
400
200
0
gpbb
control
areds3
ga
control
areds3
ga
Group

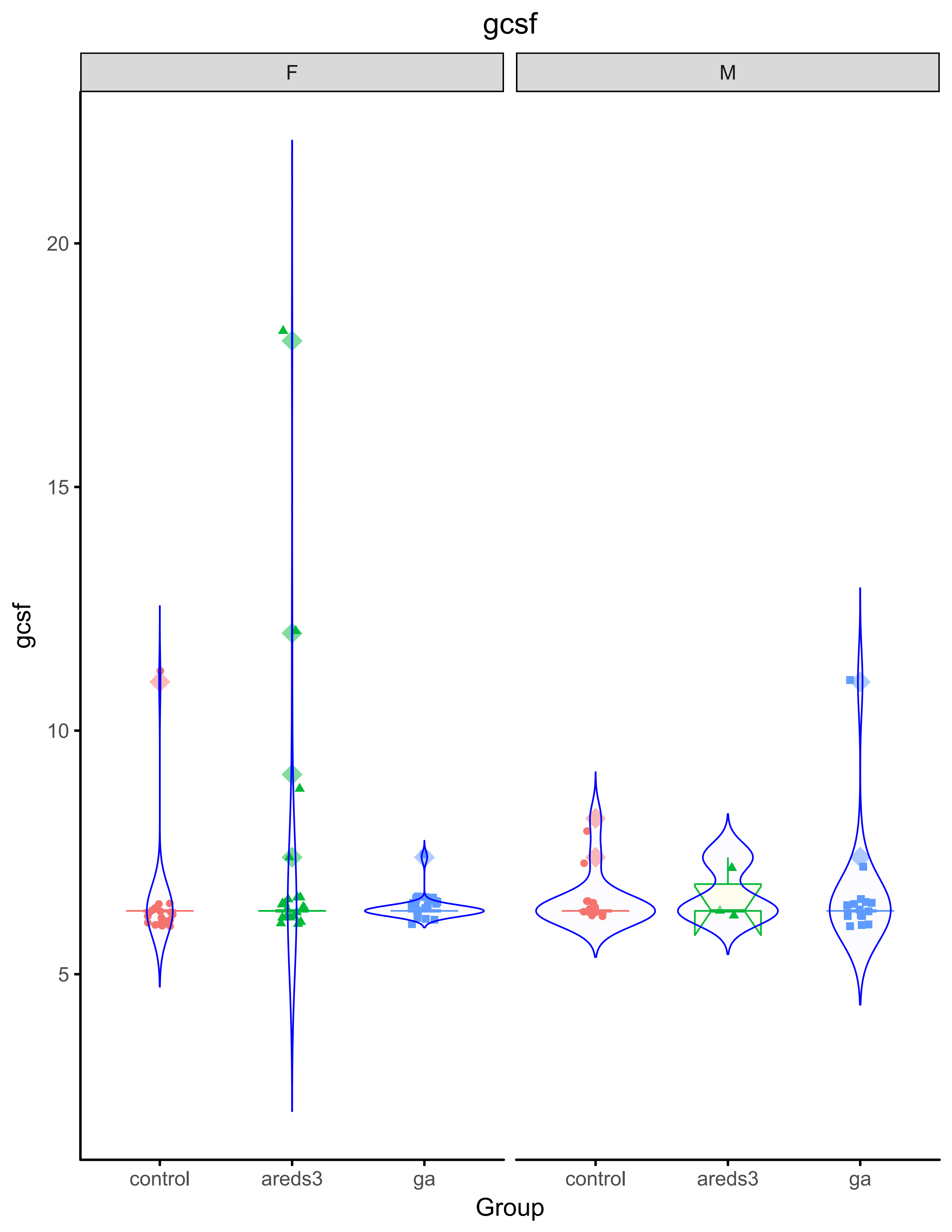
gcsf
F
M
20
15
10
5
gcsf
control
areds3
ga
control
areds3
ga
Group

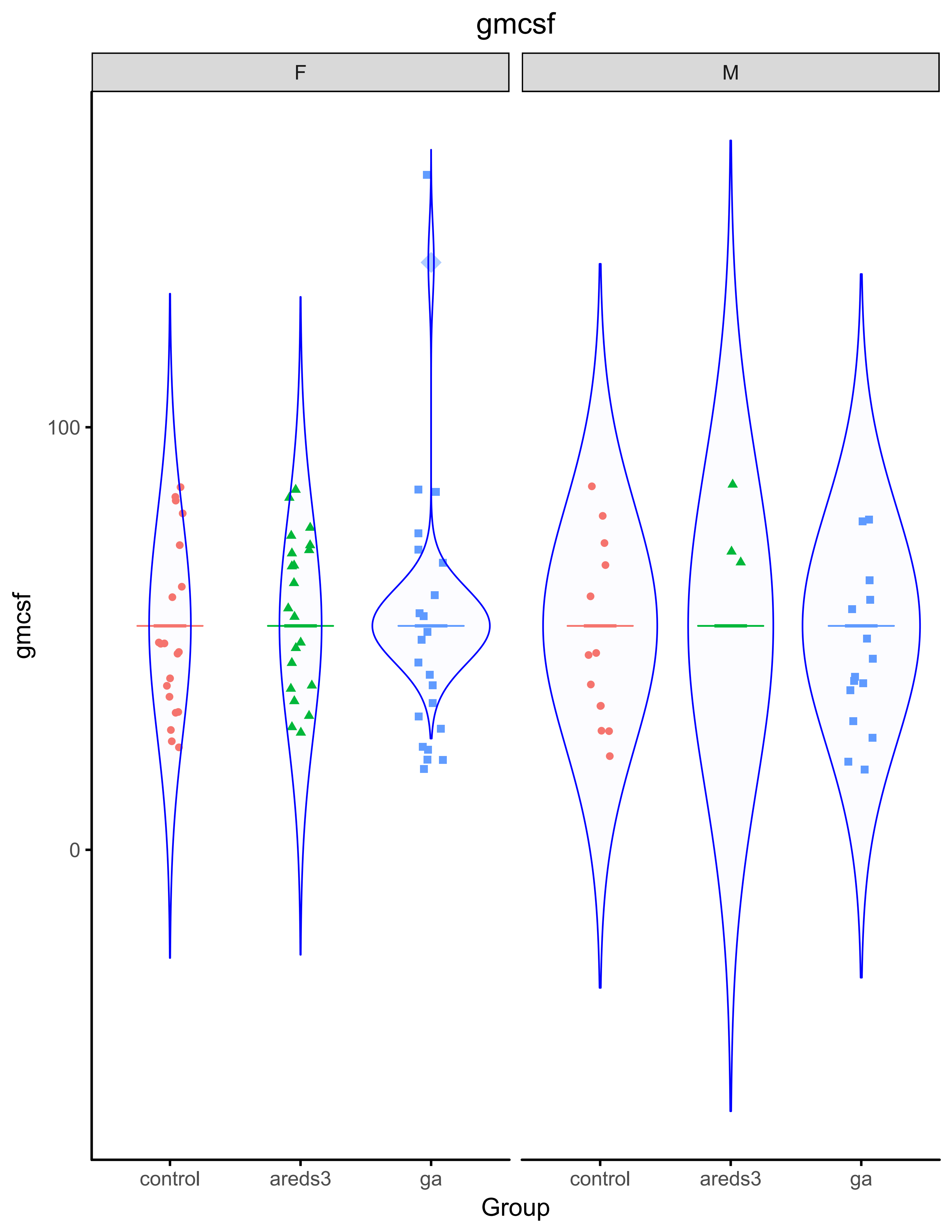
gmcsf
F
M
100
0
gmcsf
control
areds3
ga
control
areds3
ga
Group

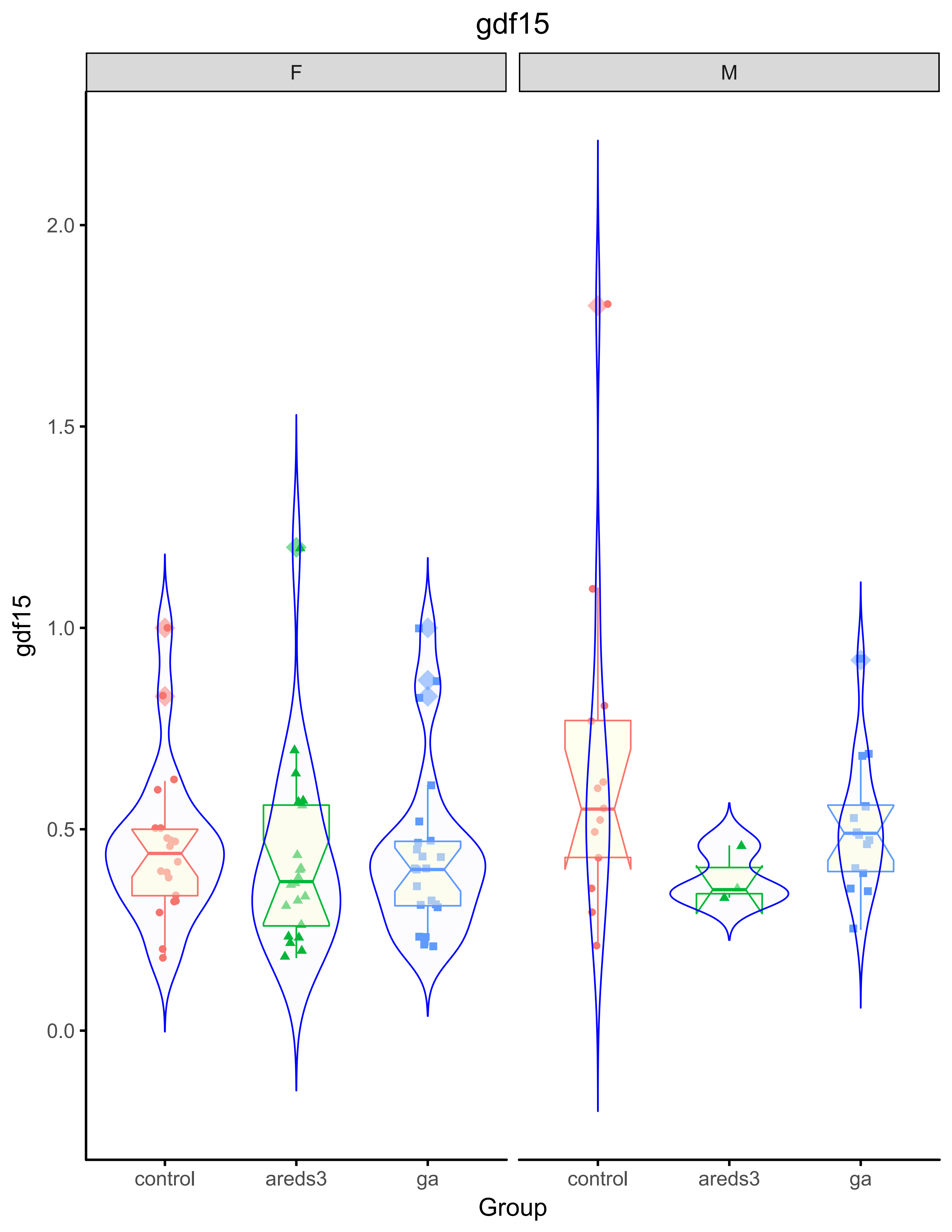

gdf15
F
M
gdf15
2.0
1.5
1.0
0.5
0.0
control
areds3
ga
control
areds3
ga
Group

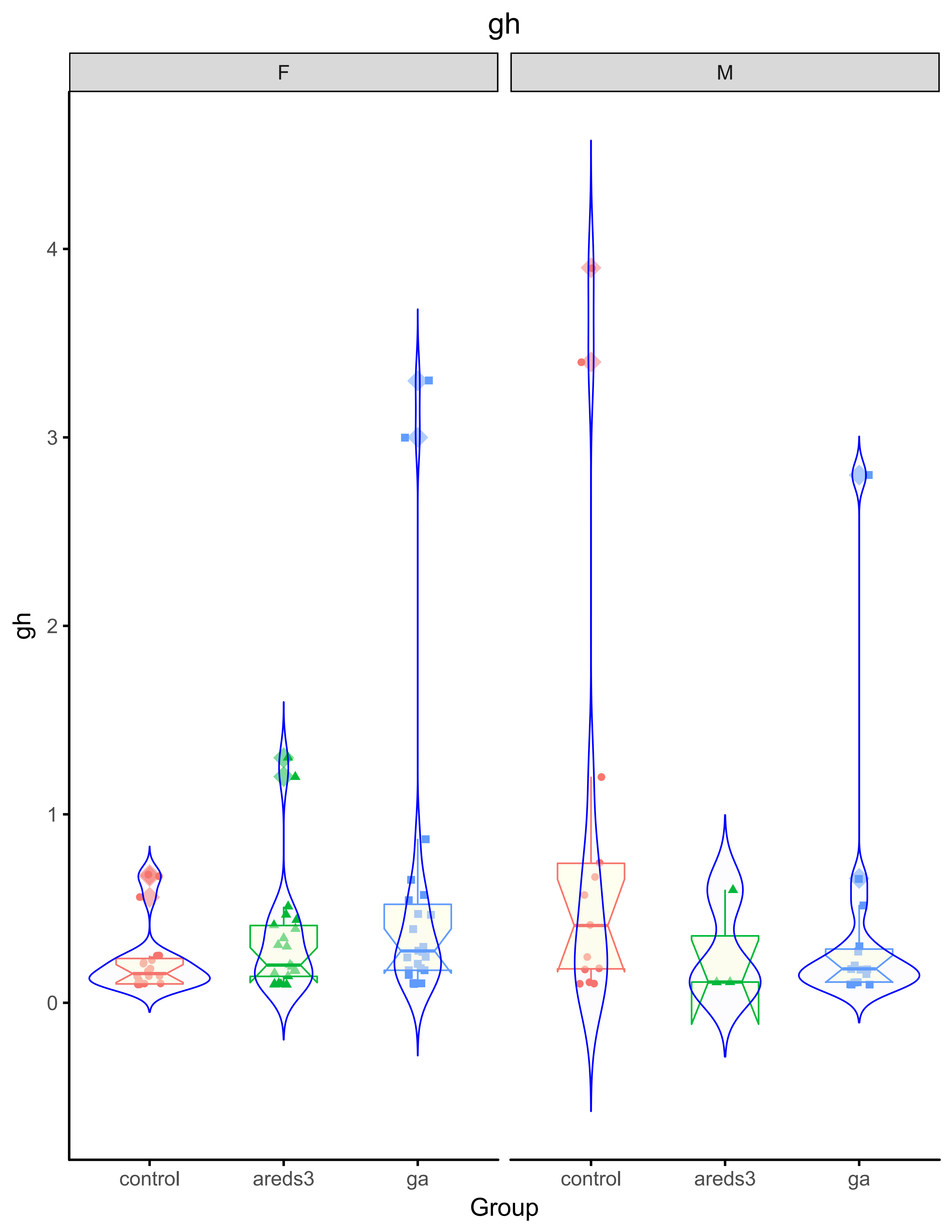
gh
F
M
4
3
2
1
0
gh
control
areds3
ga
control
areds3
ga
Group

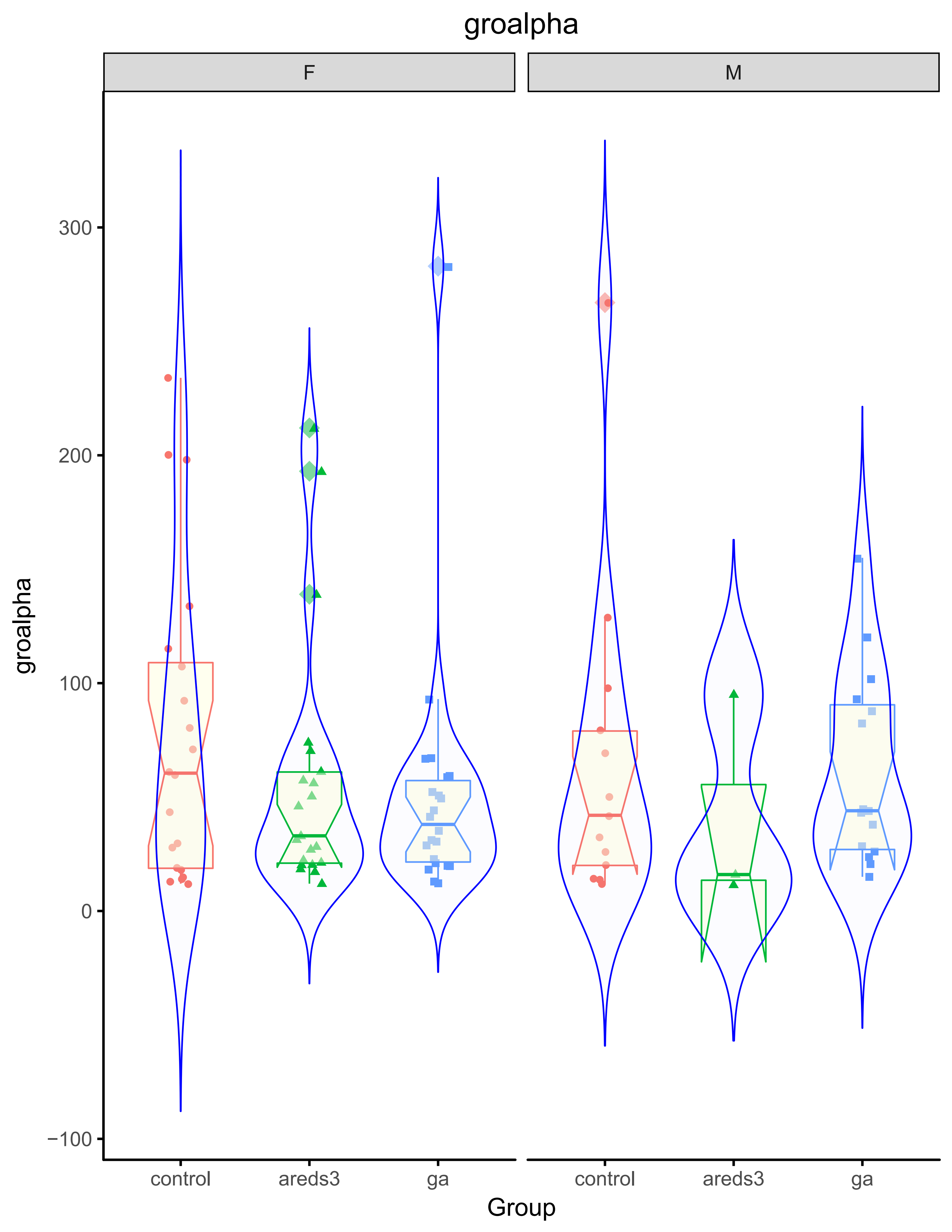
groalpha
F
M
300
200
100
0
−100
groalpha
control
areds3
ga
control
areds3
ga
Group

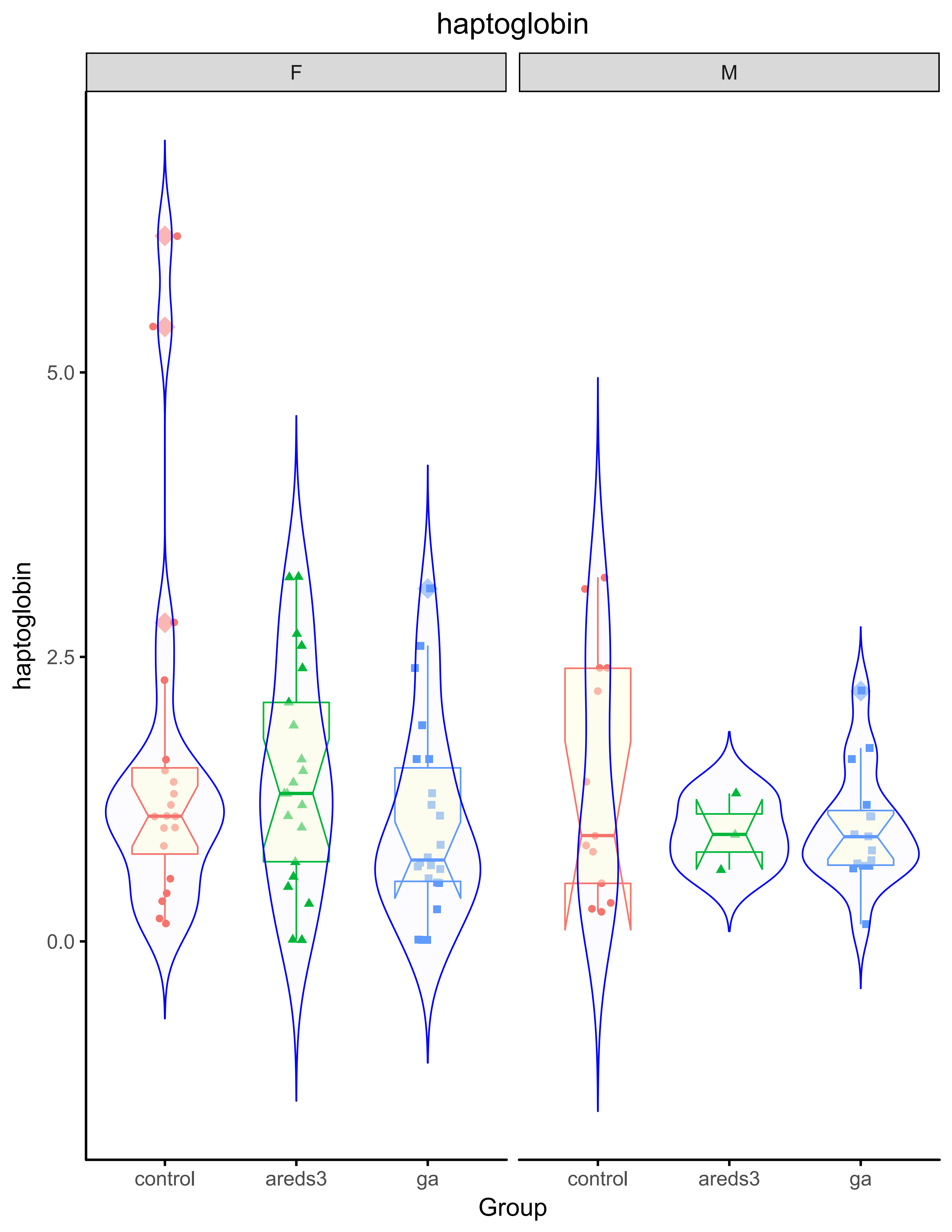

haptoglobin
F
M
5.0
2.5
0.0
haptoglobin
control
areds3
ga
control
areds3
ga
Group

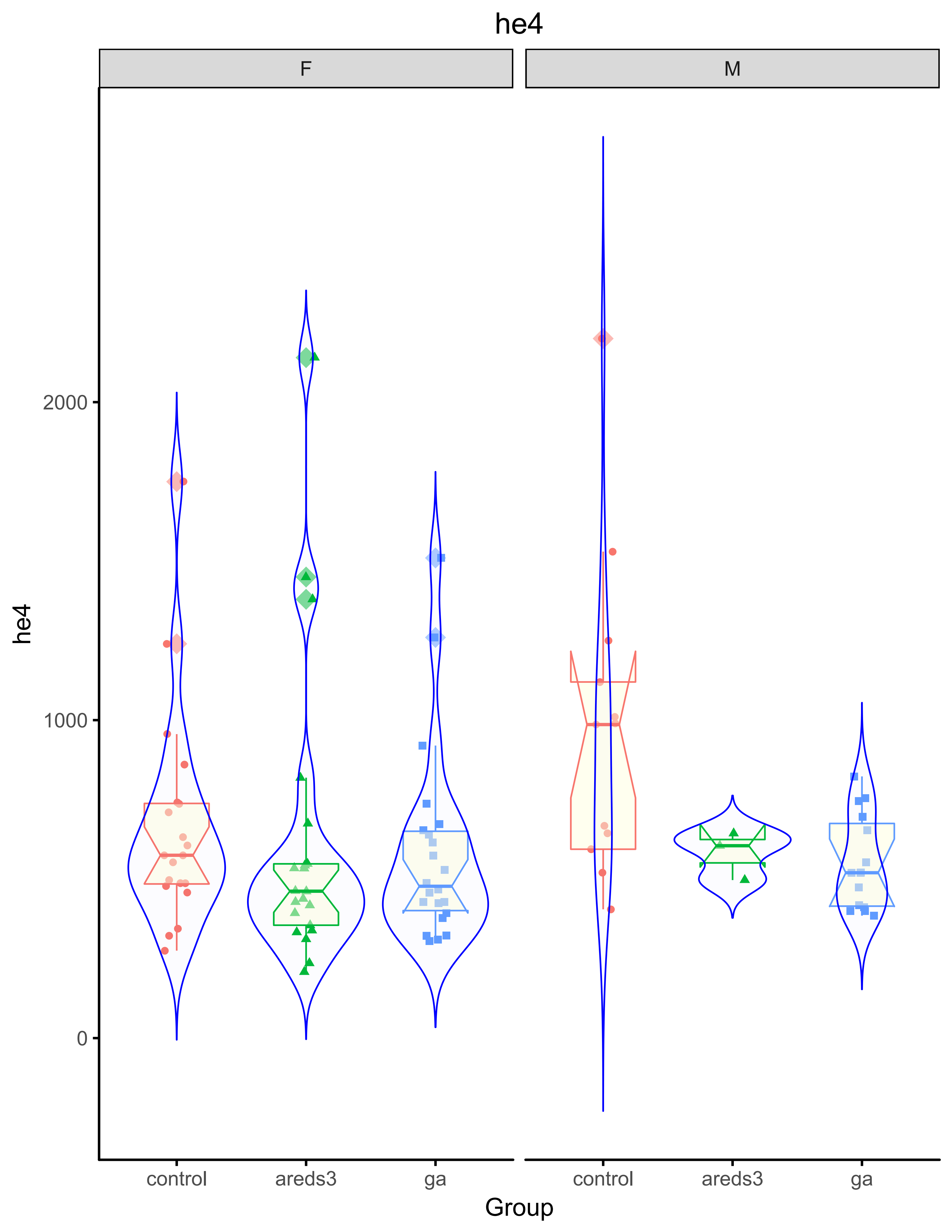
he4
F
M
2000
1000
0
he4
control
areds3
ga
control
areds3
ga
Group

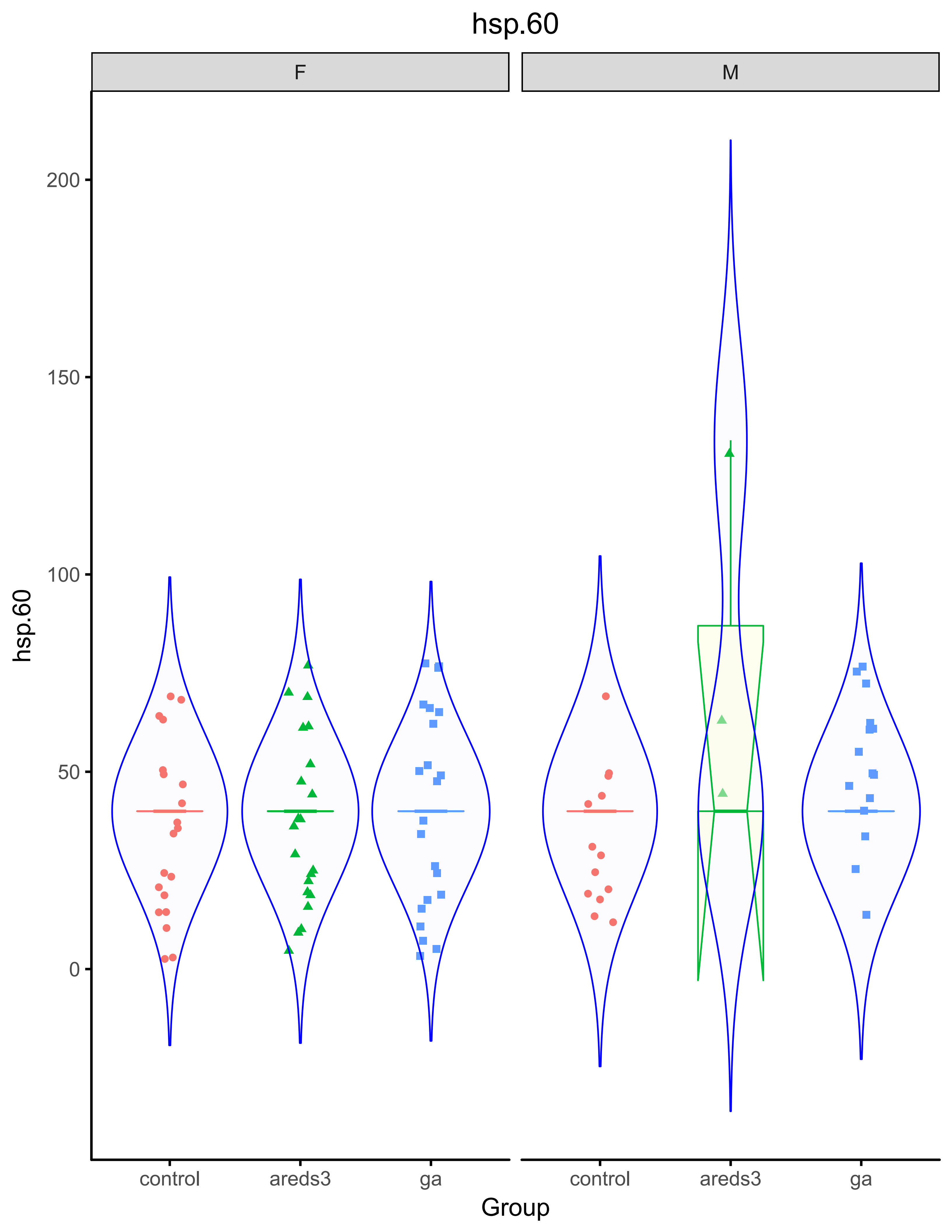

hsp.60
F
M
200
150
100
50
0
hsp.60
control
areds3
ga
control
areds3
ga
Group

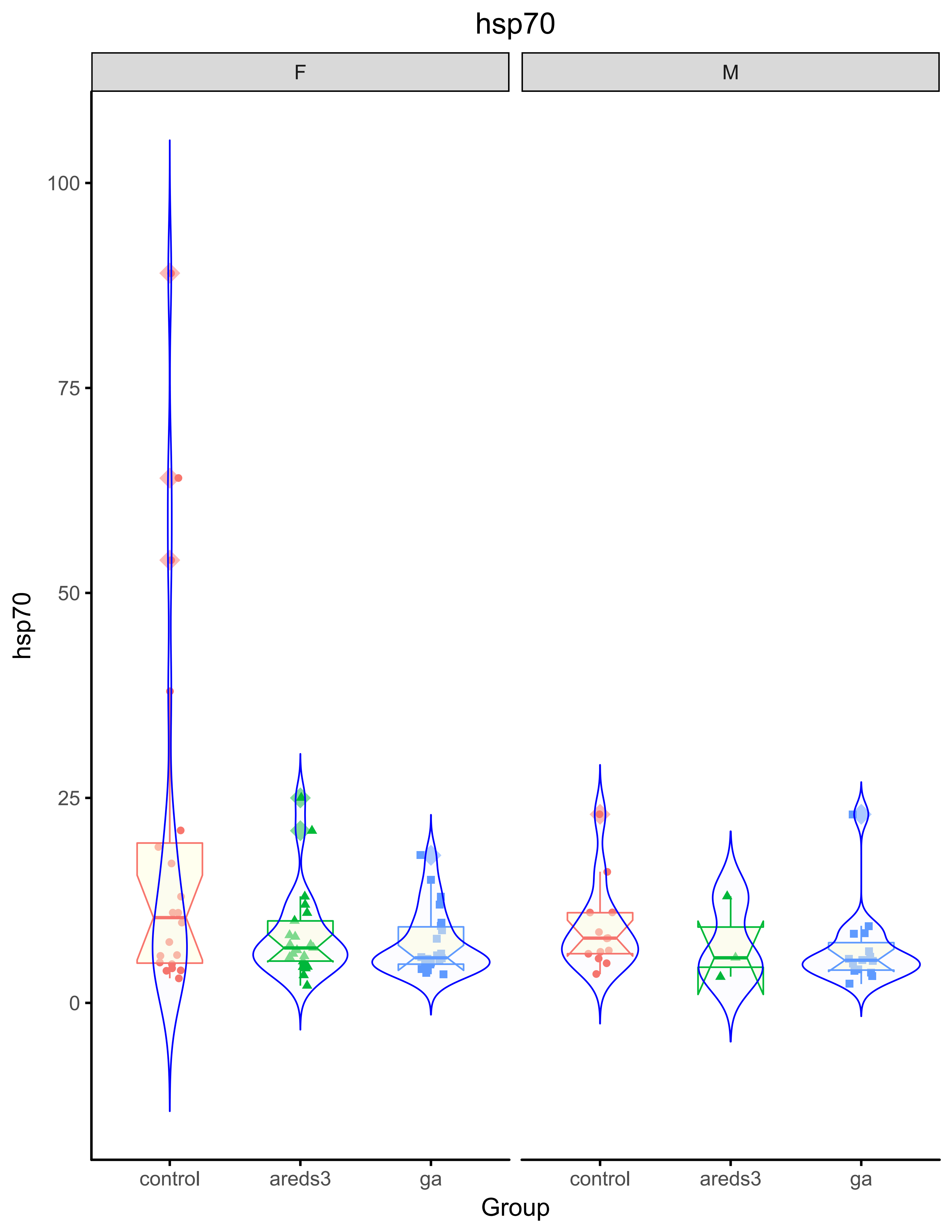

hsp70
F
M
100
75
50
25
0
hsp70
control
areds3
ga
control
areds3
ga
Group

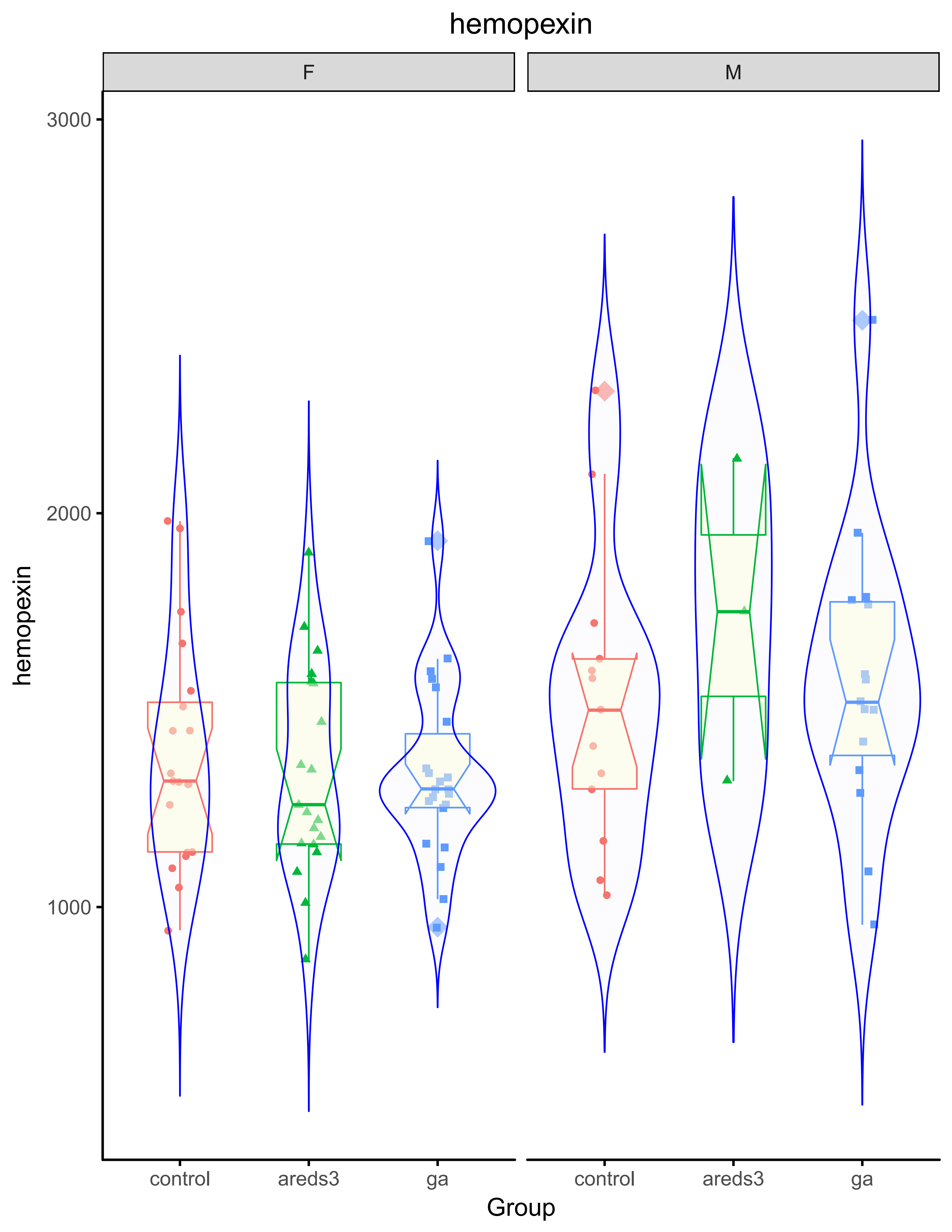
hemopexin
F
M
3000
2000
1000
hemopexin
control
areds3
ga
control
areds3
ga
Group

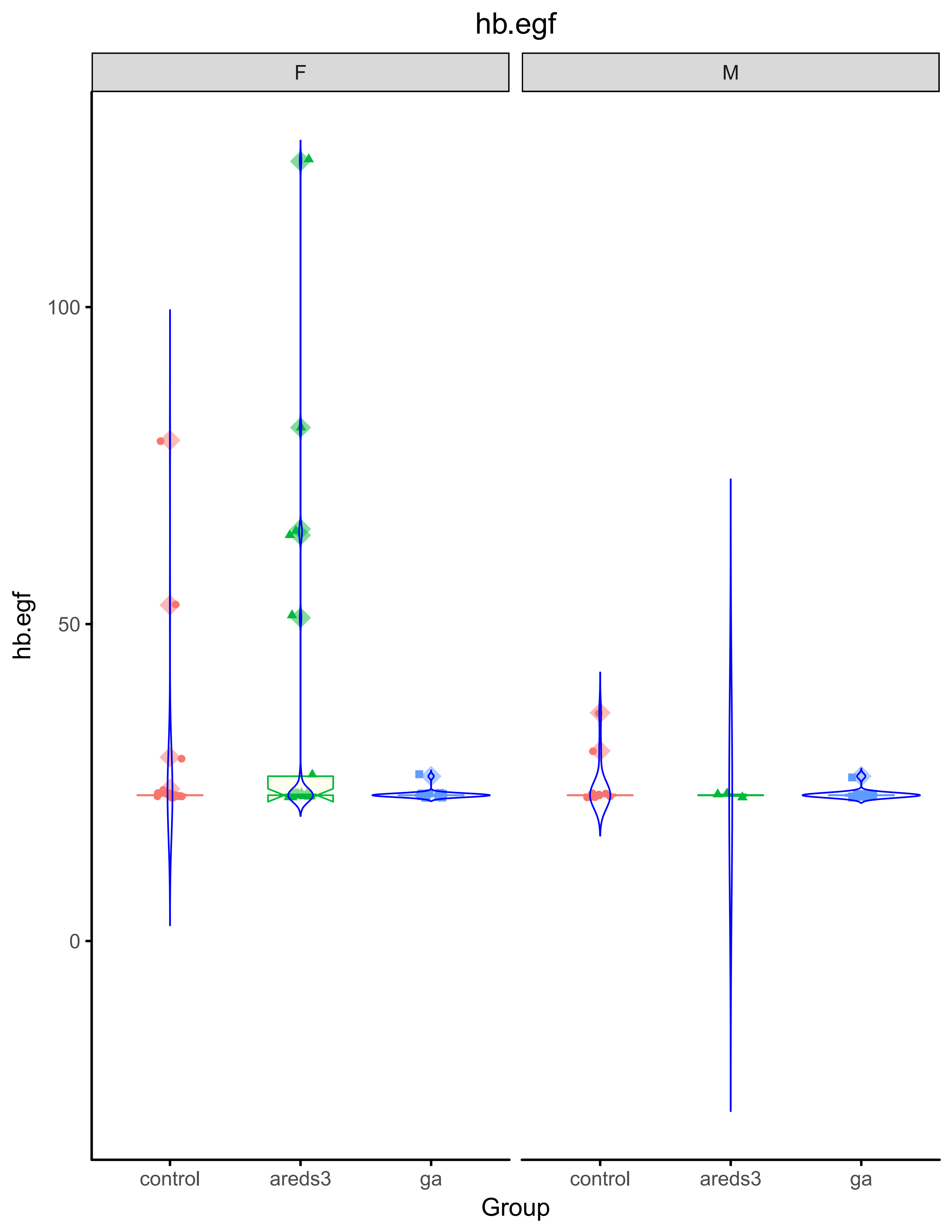

hb.egf
F
M
100
50
0
hb.egf
control
areds3
ga
control
areds3
ga
Group

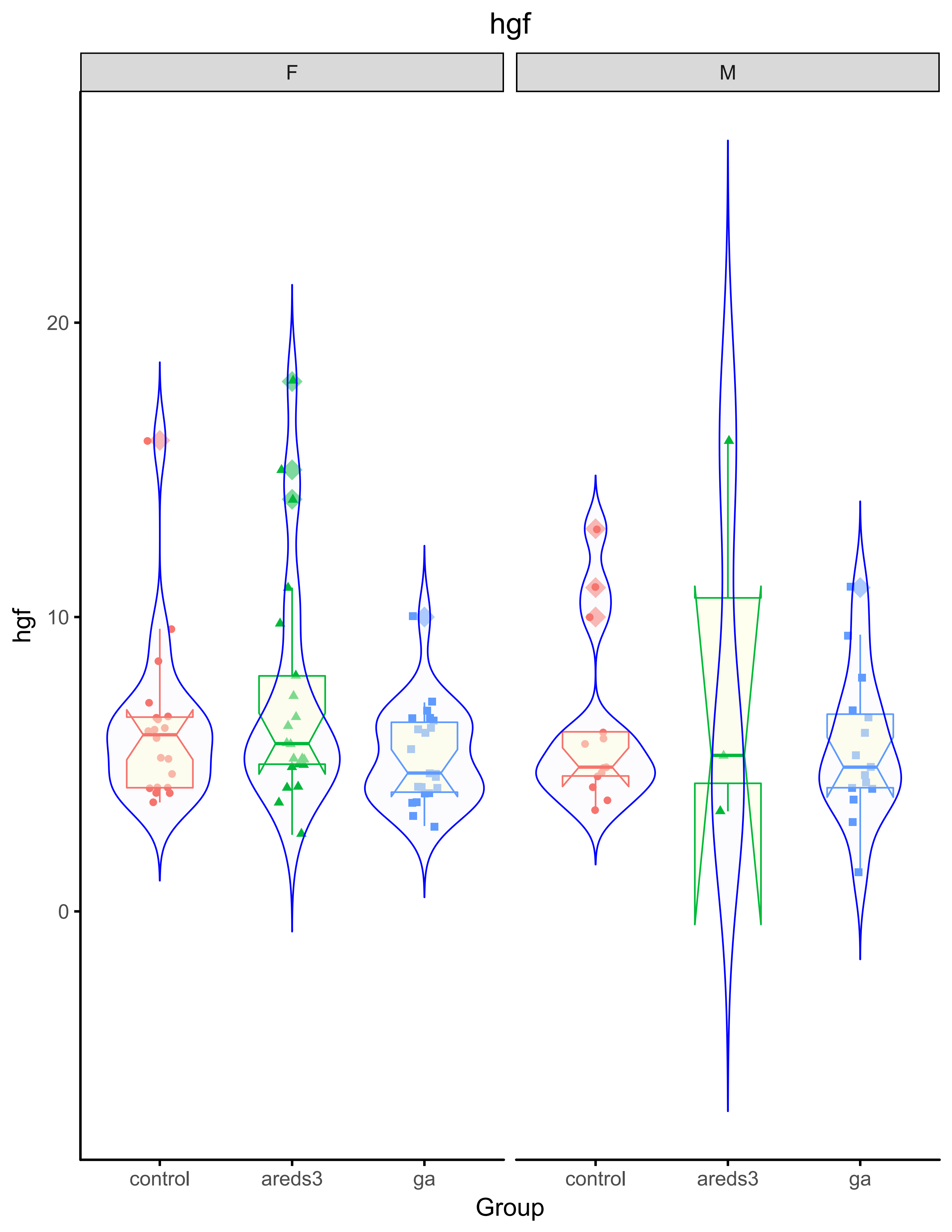
hgf
F
M
20
10
0
hgf
control
areds3
ga
control
areds3
ga
Group

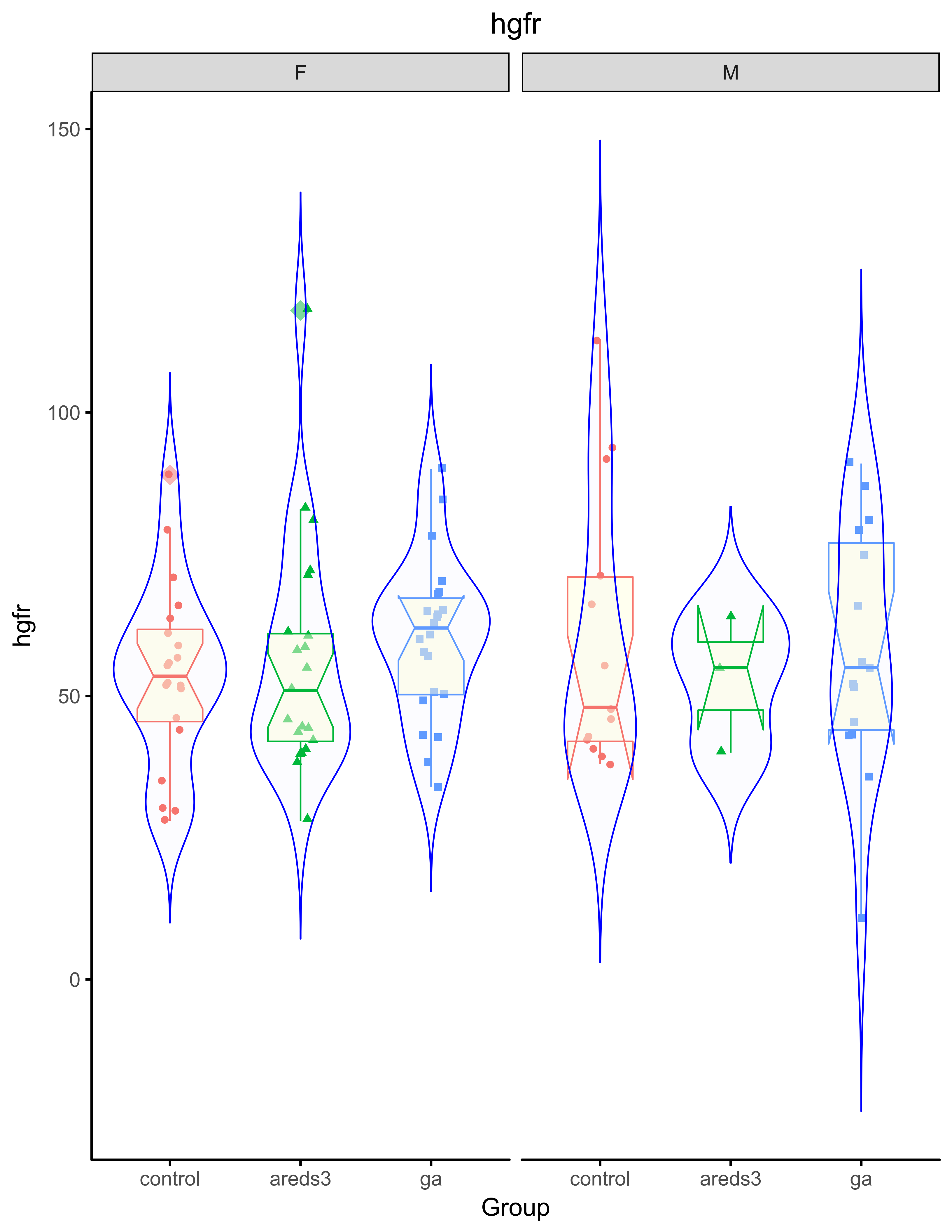

hgfr
F
M
150
100
50
0
hgfr
control
areds3
ga
control
areds3
ga
Group

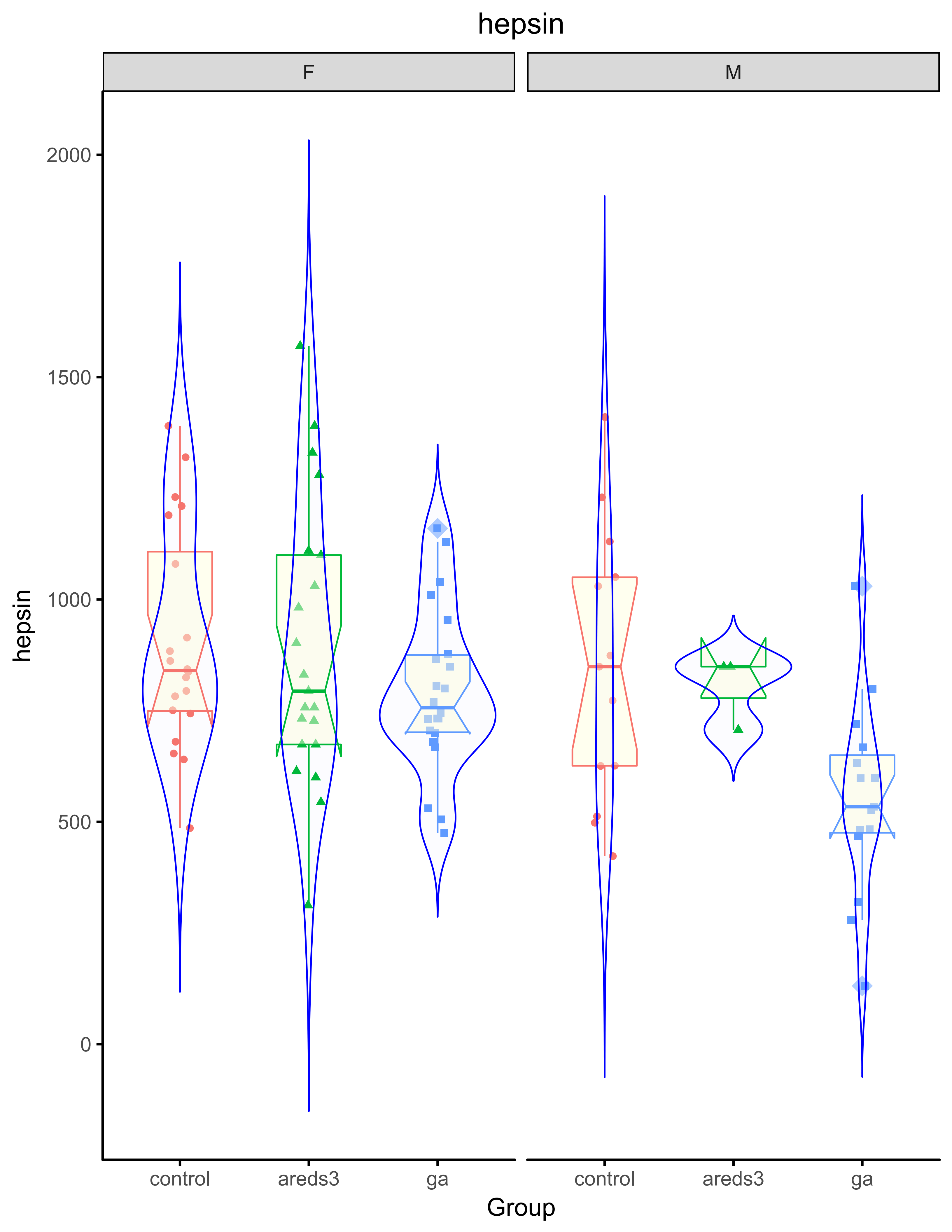
hepsin
F
M
2000
1500
1000
500
0
hepsin
control
areds3
ga
control
areds3
ga
Group

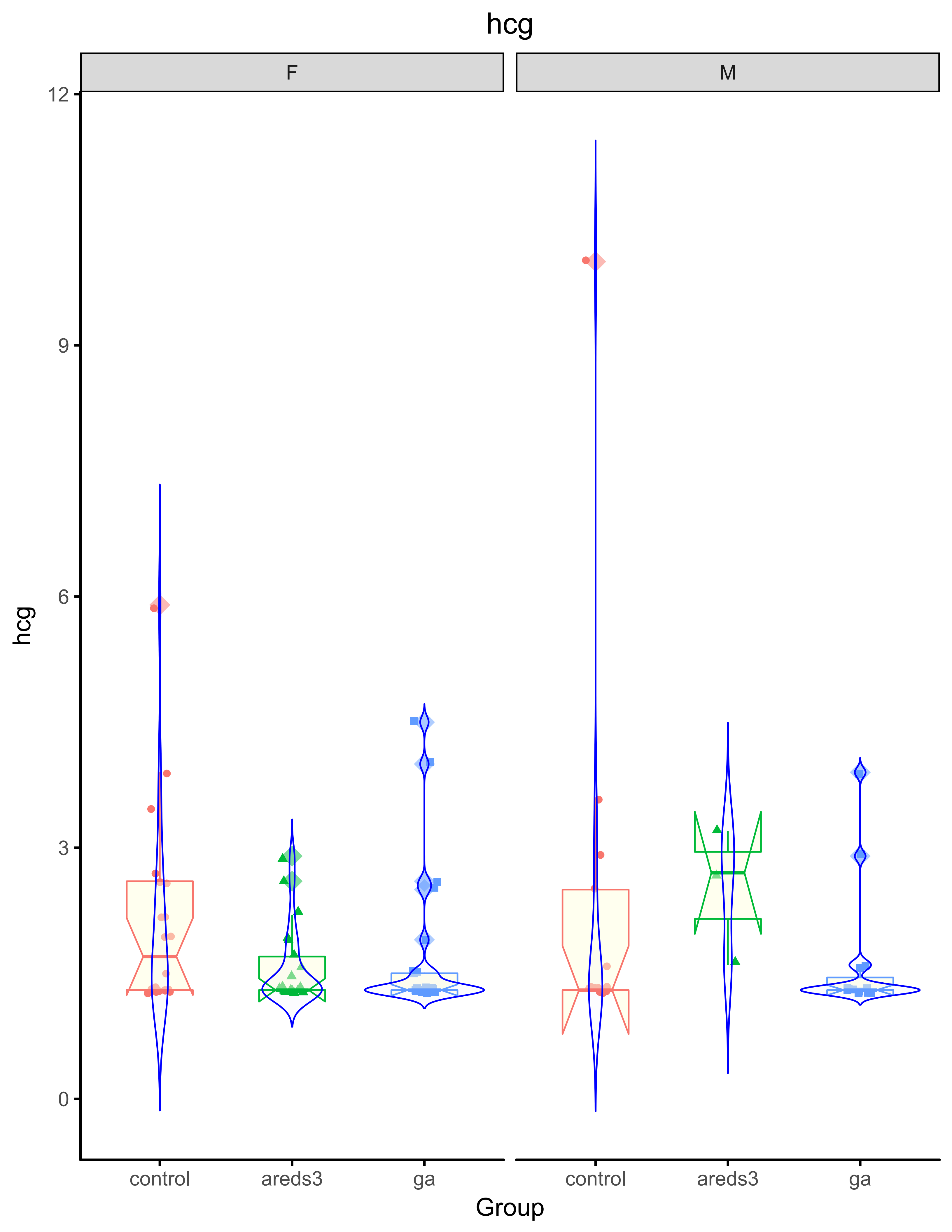

hcg
F
M
12
9
6
3
0
hcg
control
areds3
ga
control
areds3
ga
Group

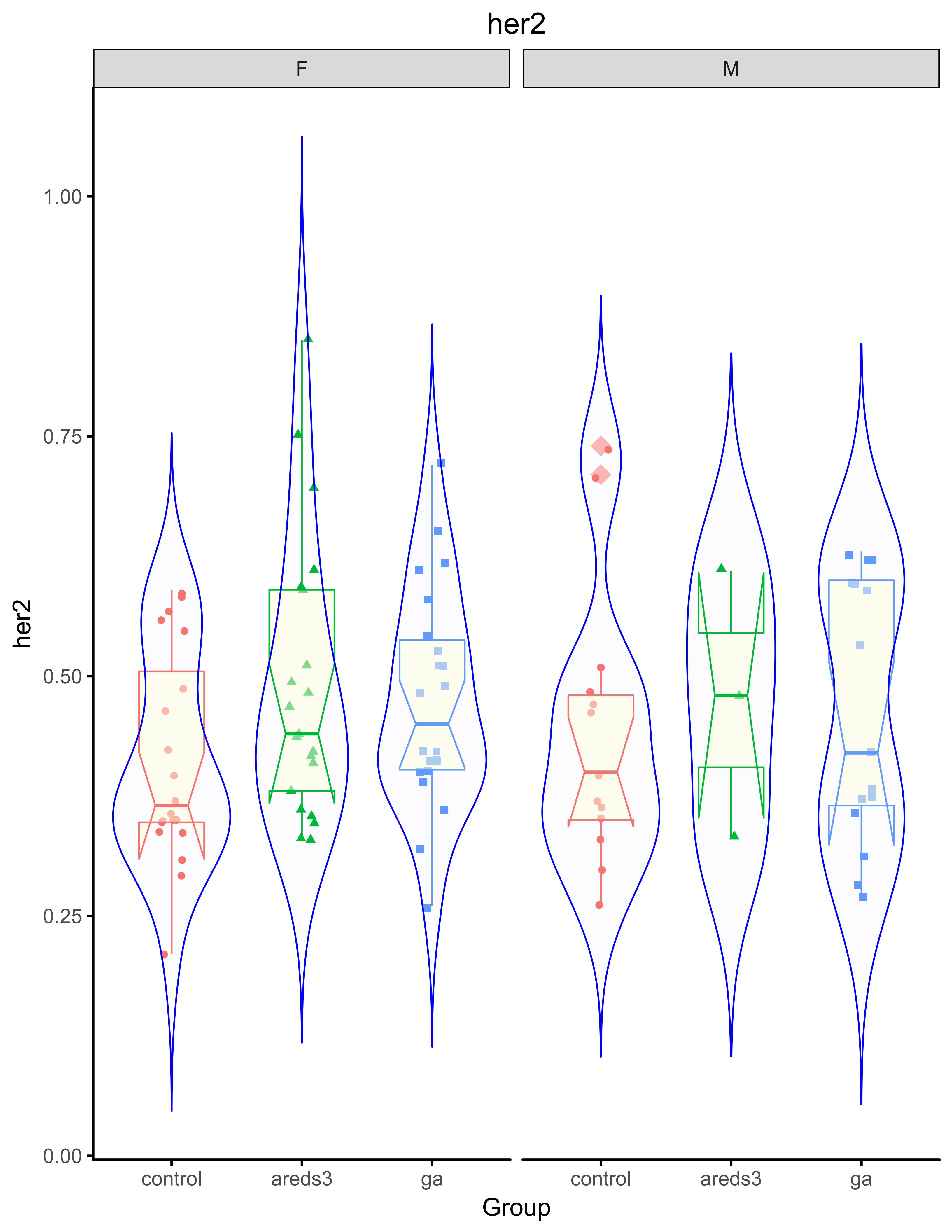

her2
F
M
1.00
0.75
0.50
0.25
0.00
her2
control
areds3
ga
control
areds3
ga
Group

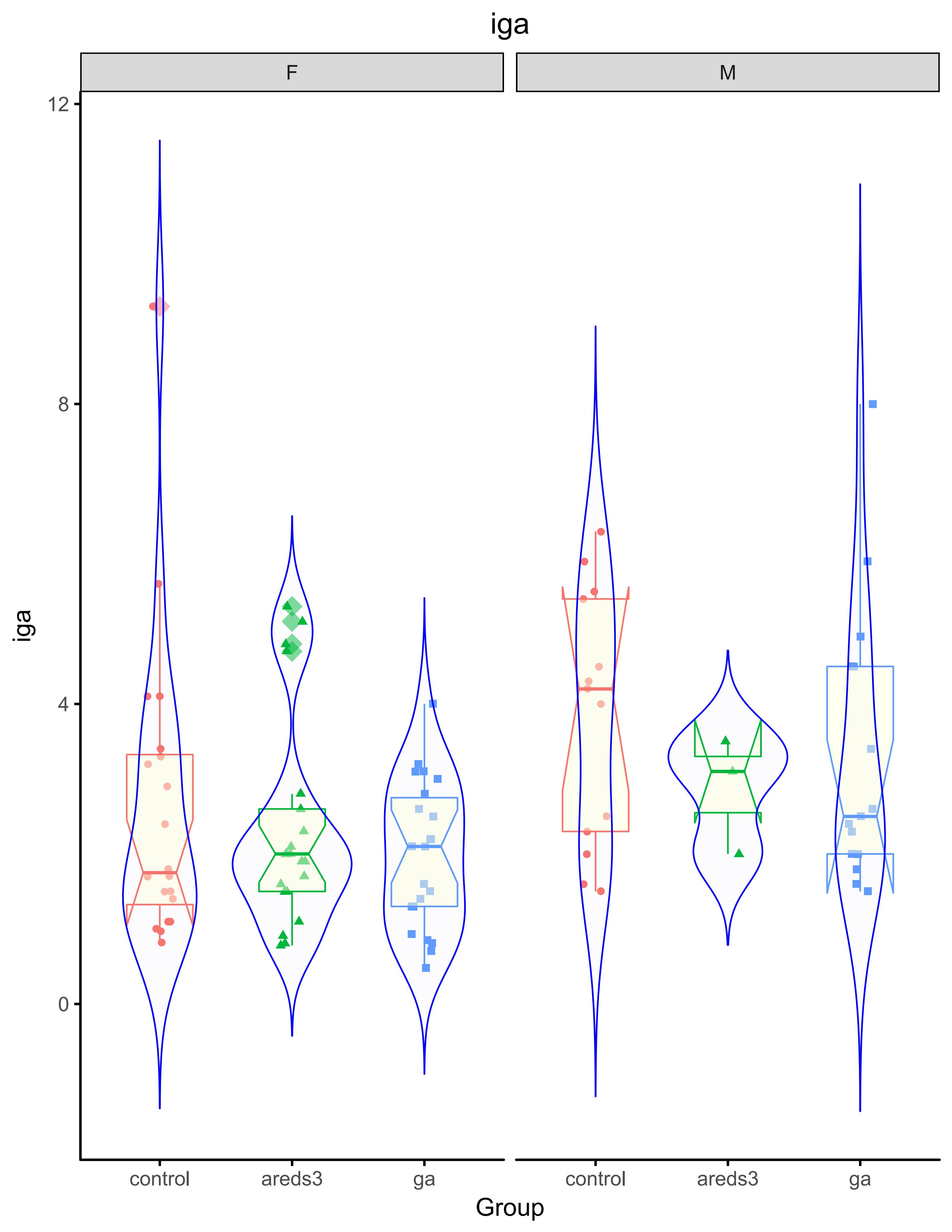
iga
F
M
12
8
4
0
iga
control
areds3
ga
control
areds3
ga
Group

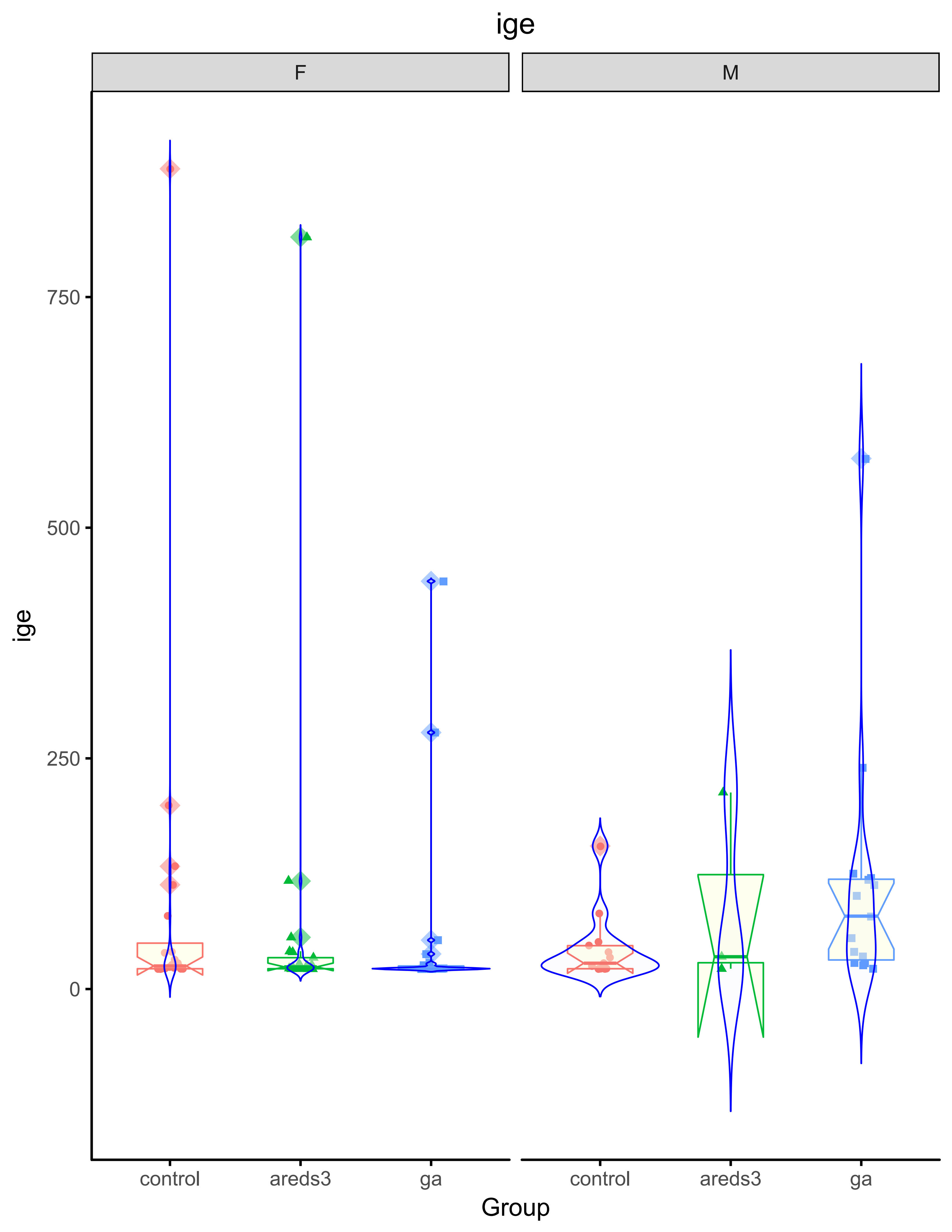
ige
F
M
750
500
250
0
ige
control
areds3
ga
control
areds3
ga
Group

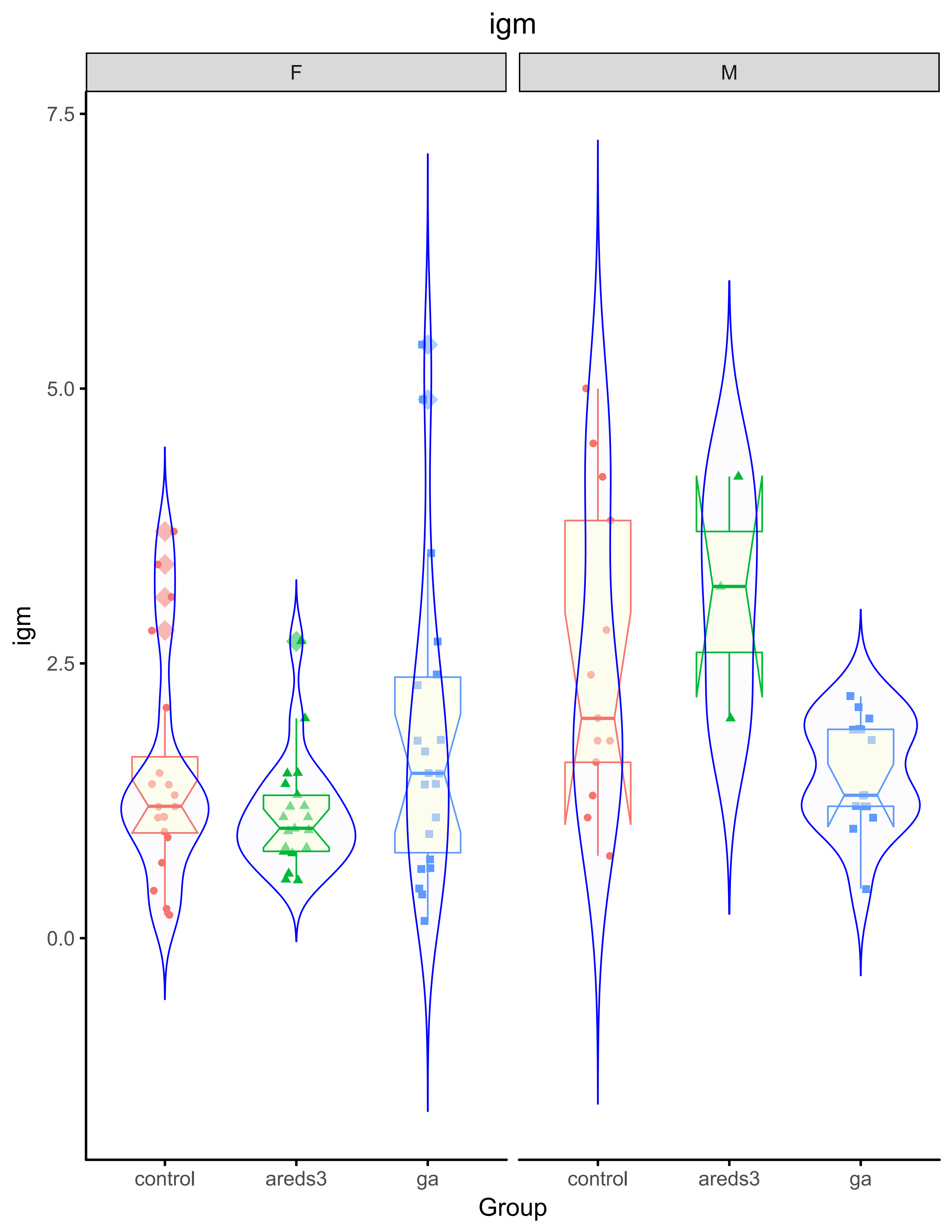

igm
F
M
7.5
5.0
2.5
0.0
igm
control
areds3
ga
control
areds3
ga
Group

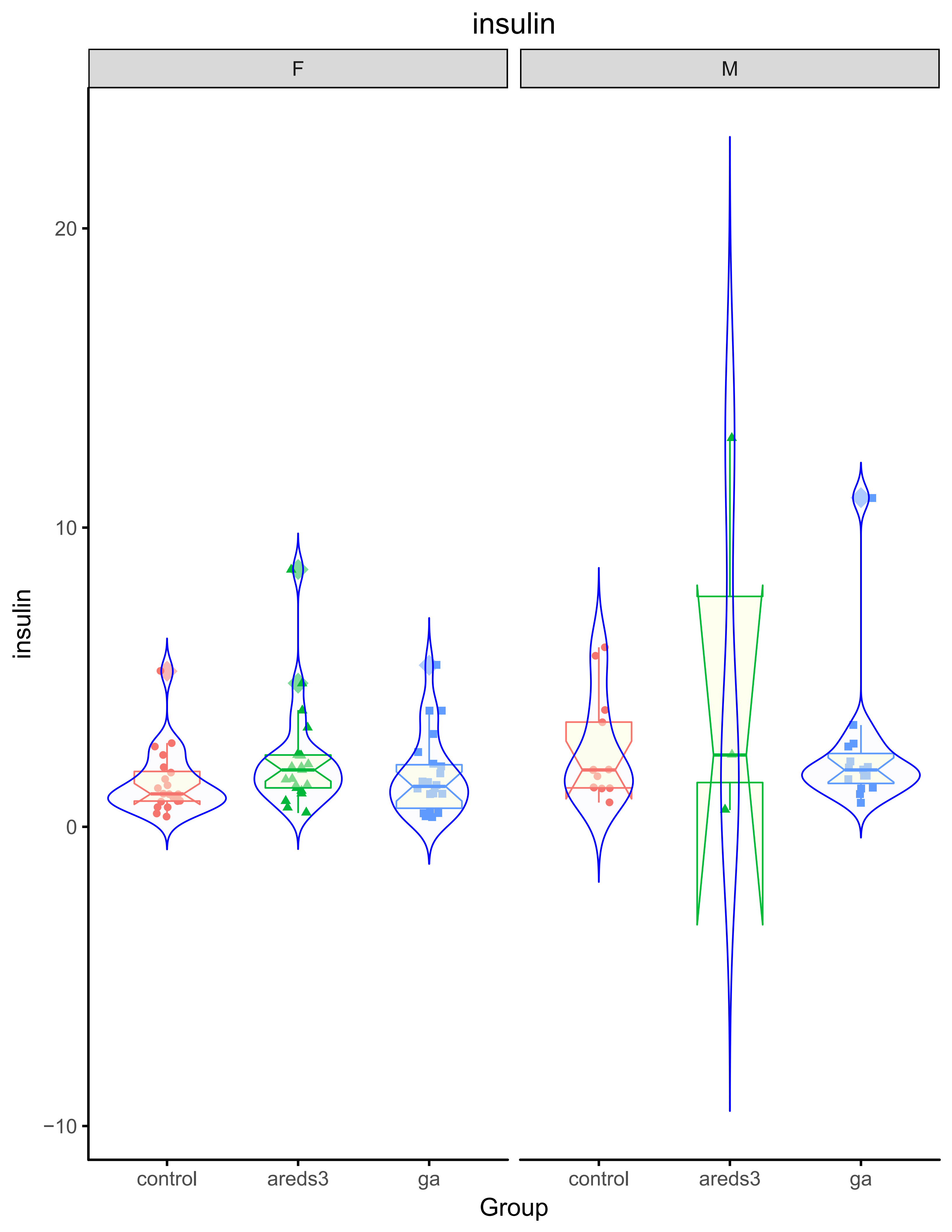
insulin
F
M
20
10
0
−10
insulin
control
areds3
ga
control
areds3
ga
Group

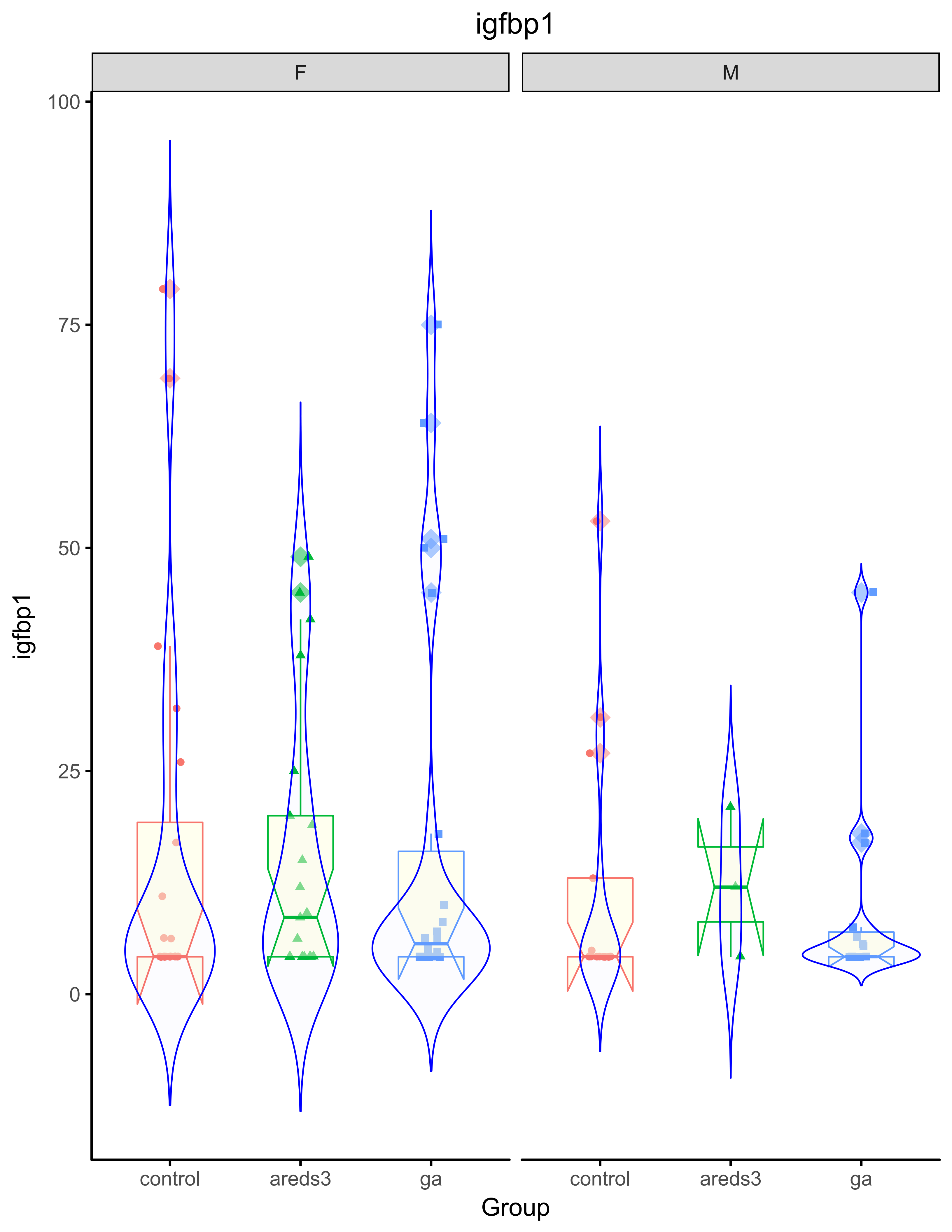

igfbp1
F
M
100
75
50
25
0
igfbp1
control
areds3
ga
control
areds3
ga
Group

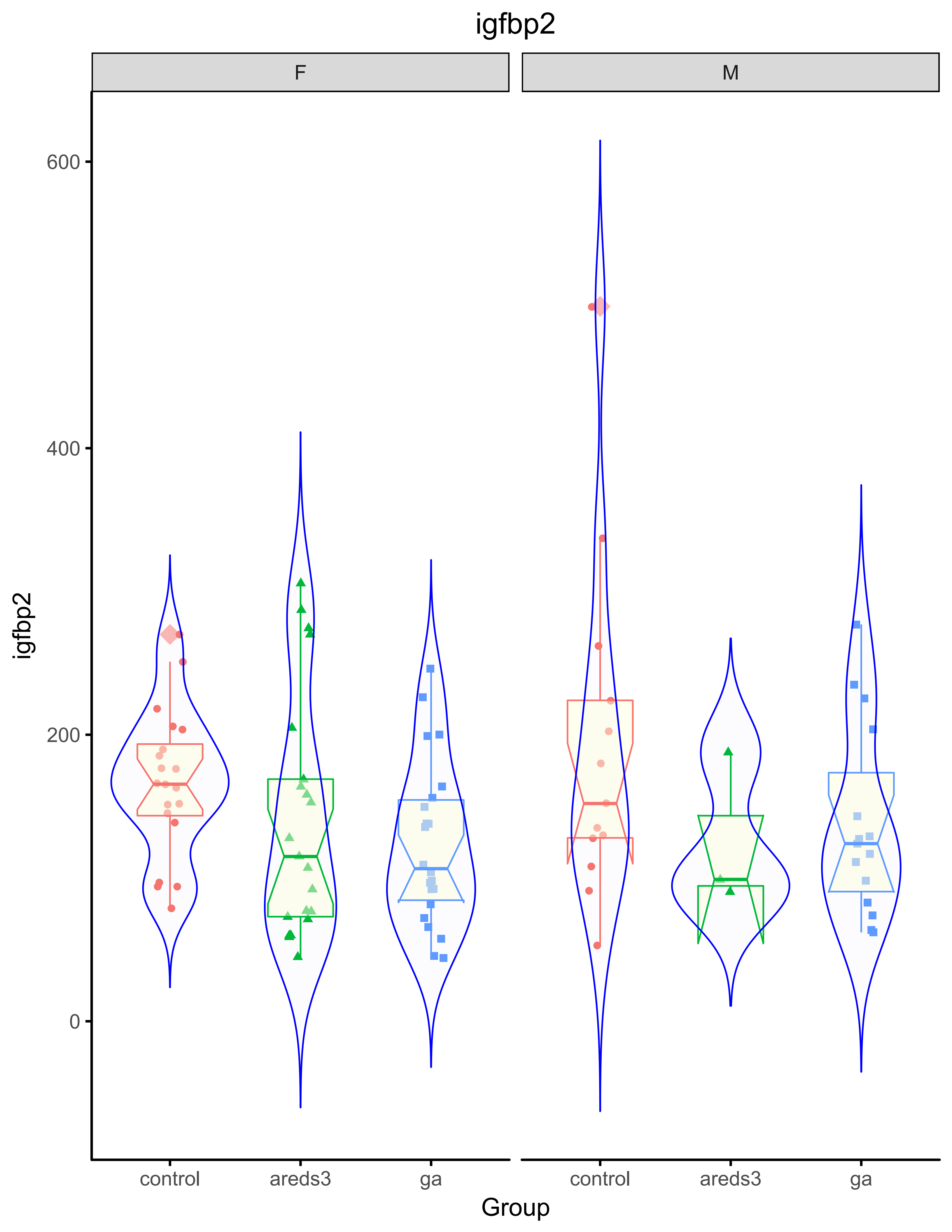

igfbp2
F
M
600
400
200
0
igfbp2
control
areds3
ga
control
areds3
ga
Group

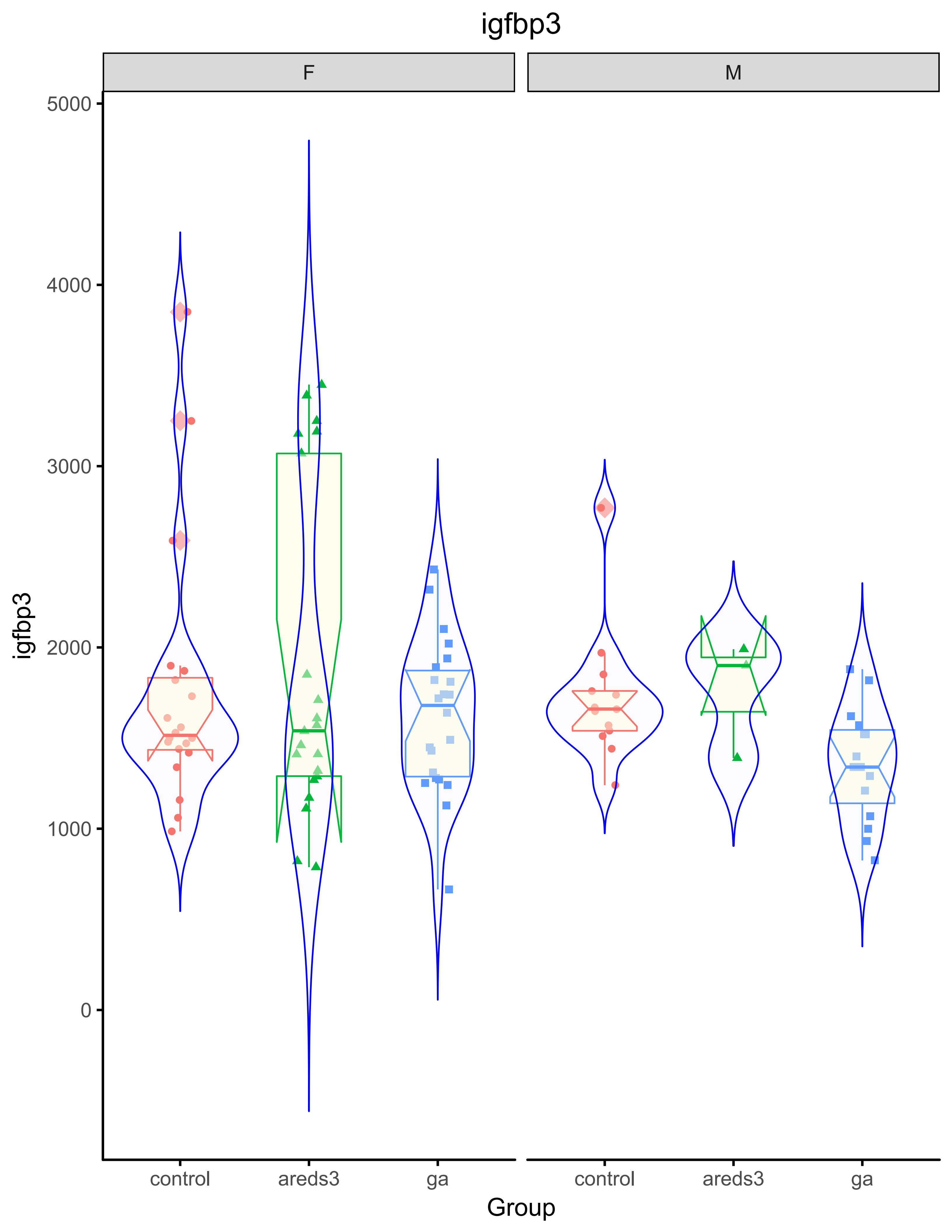
igfbp3
F
M
5000
4000
3000
2000
1000
0
igfbp3
control
areds3
ga
control
areds3
ga
Group

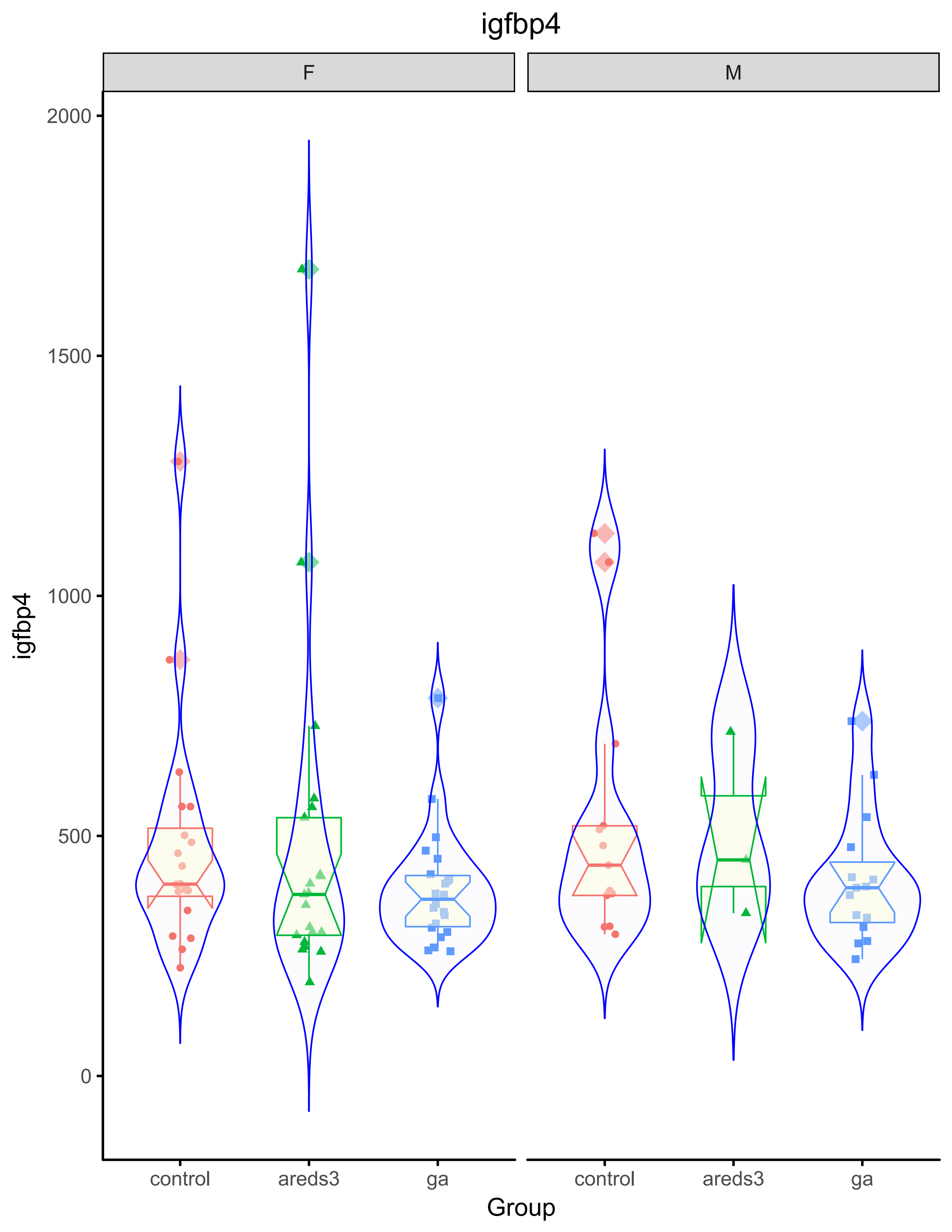
igfbp4
F
M
2000
1500
1000
500
0
igfbp4
control
areds3
ga
control
areds3
ga
Group

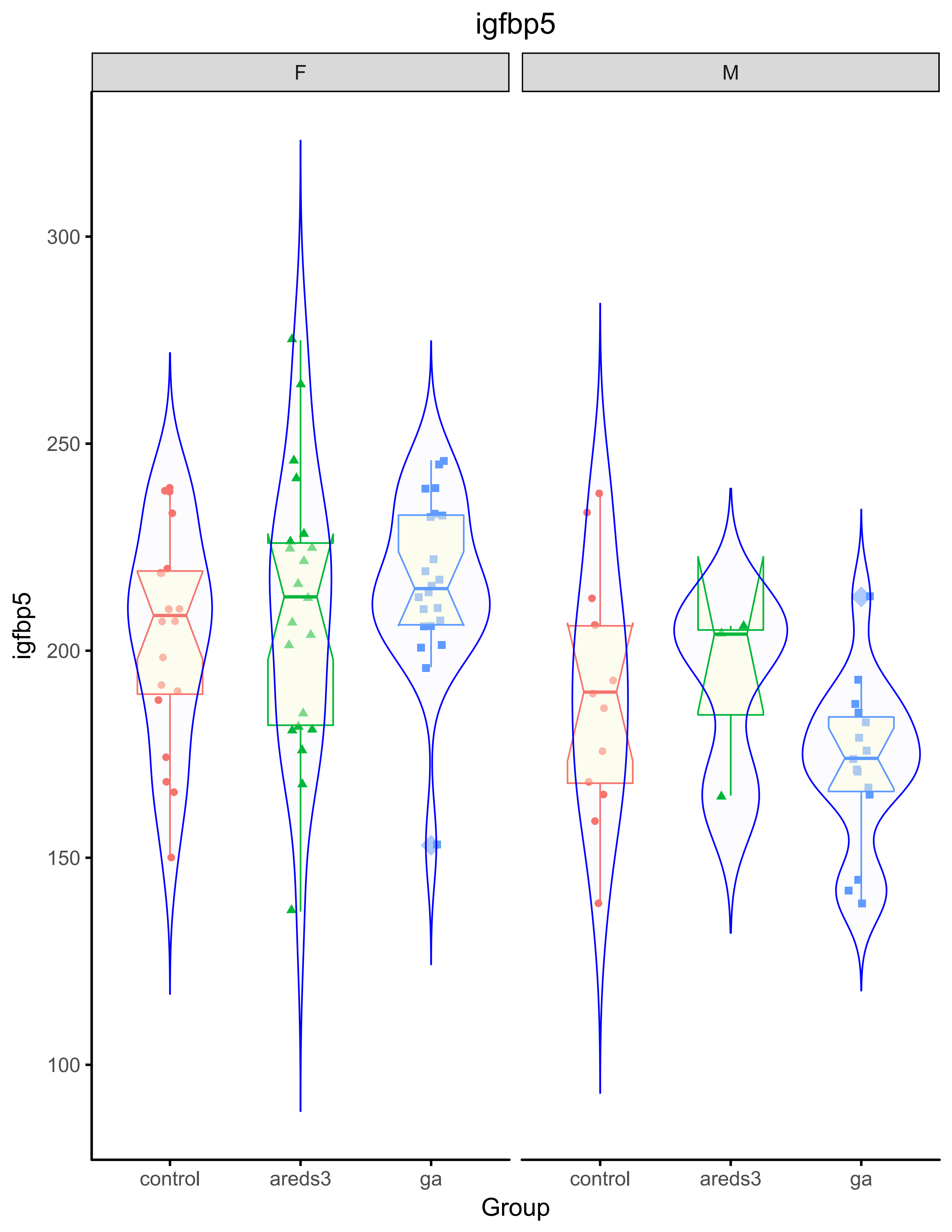

igfbp5
F
M
300
250
200
150
100
igfbp5
control
areds3
ga
control
areds3
ga
Group

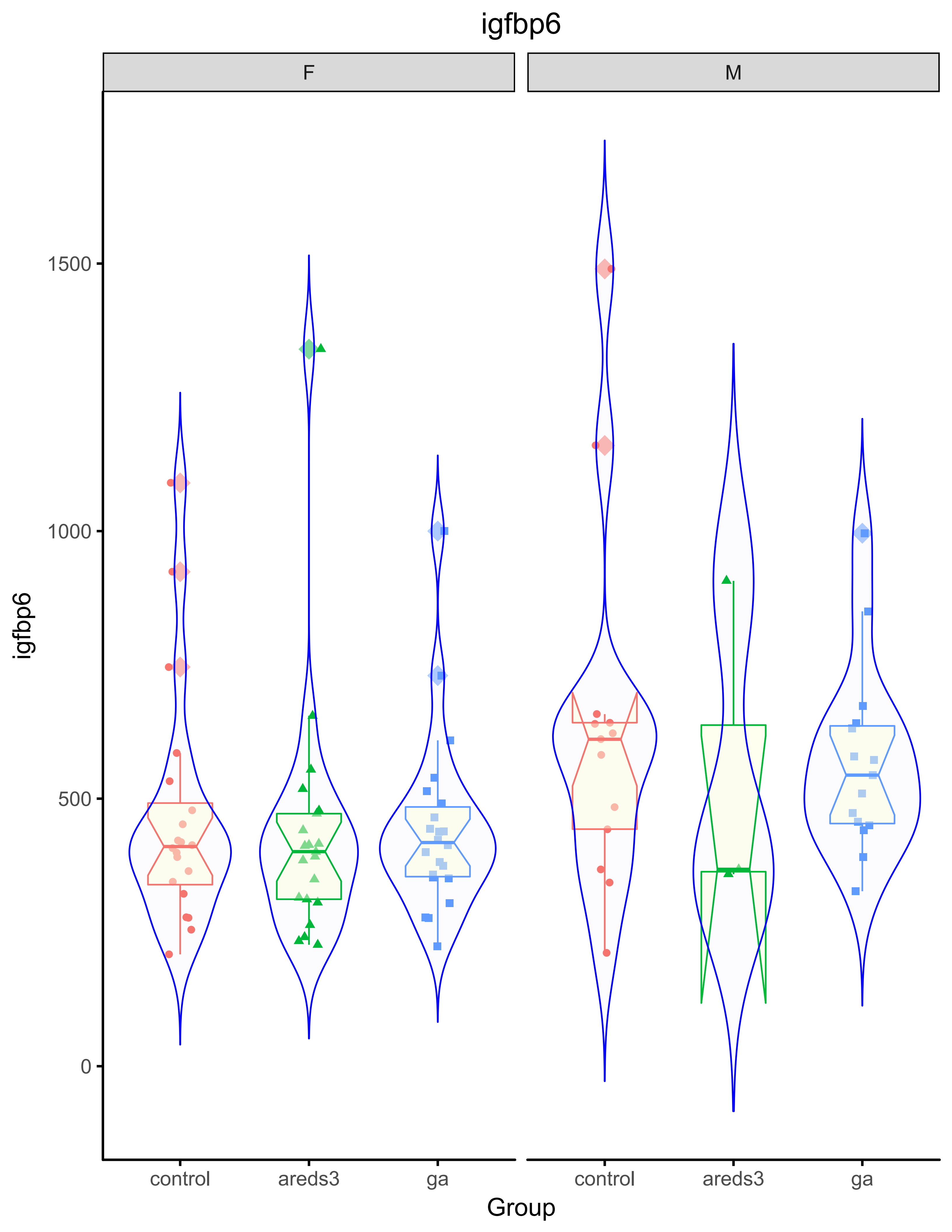

igfbp6
F
M
1500
1000
500
0
igfbp6
control
areds3
ga
control
areds3
ga
Group

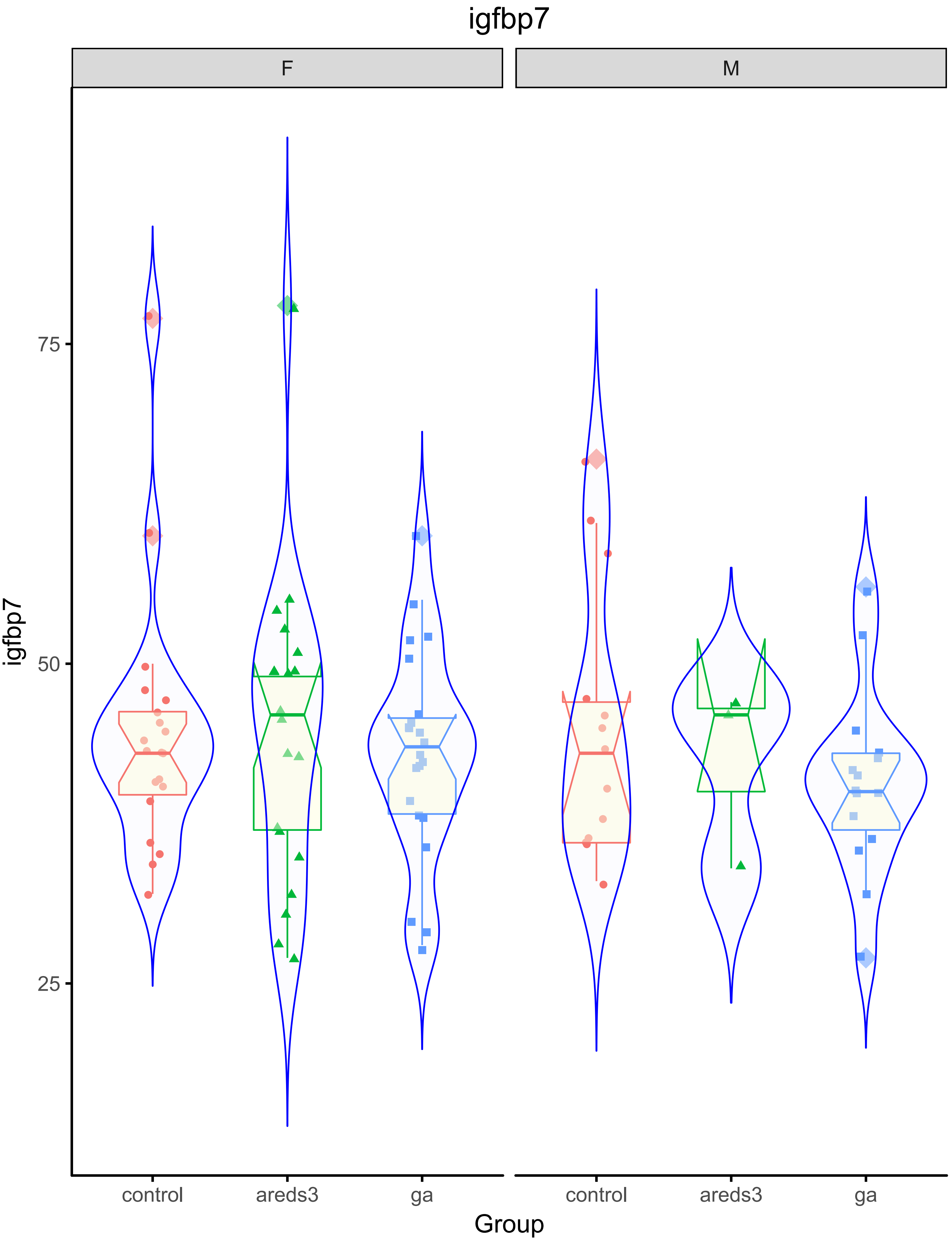

igfbp7
F
M
igfbp7
75
50
25
control
areds3
ga
control
areds3
ga
Group

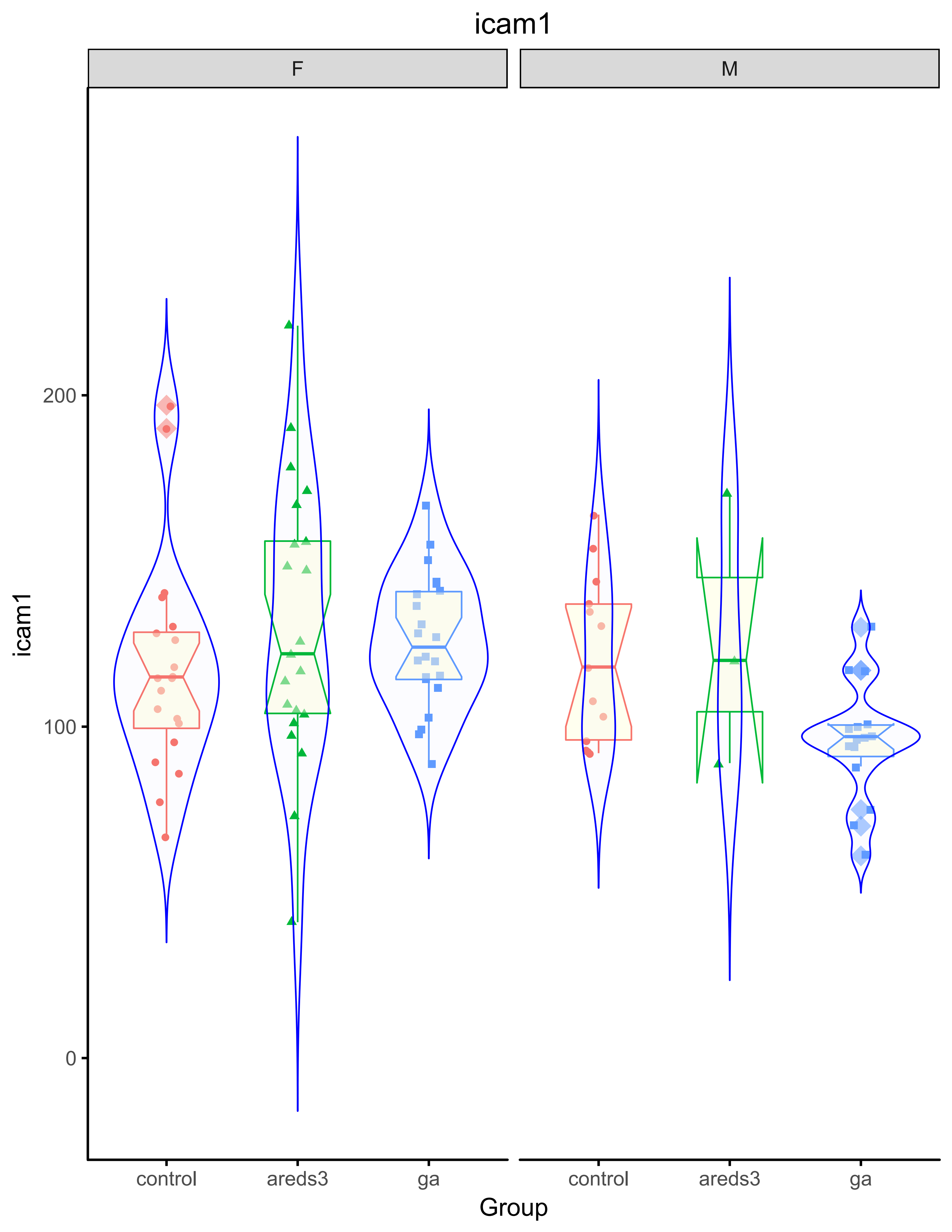

icam1
F
M
200
100
0
icam1
control
areds3
ga
control
areds3
ga
Group

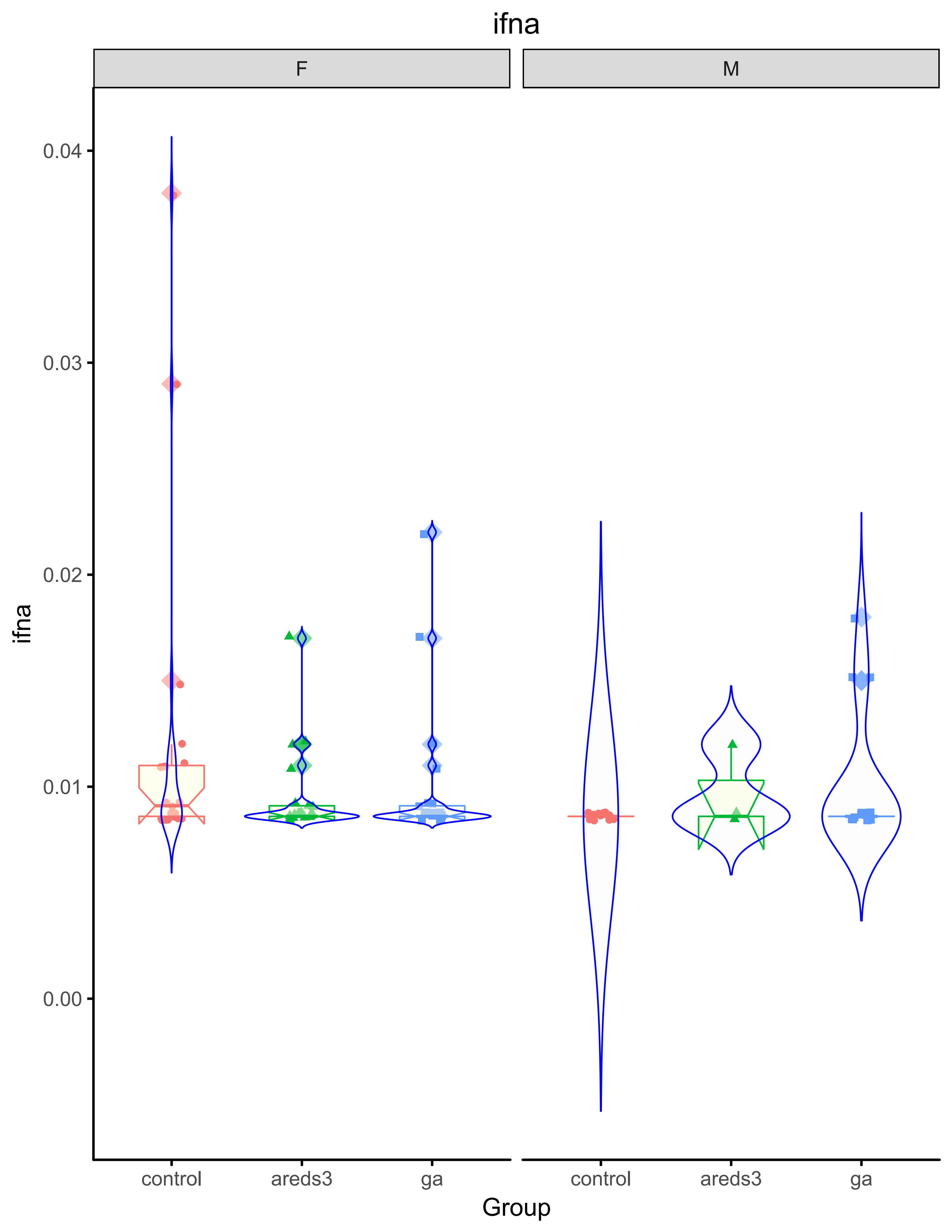

ifna
F
M
0.04
0.03
0.02
0.01
0.00
ifna
control
areds3
ga
control
areds3
ga
Group

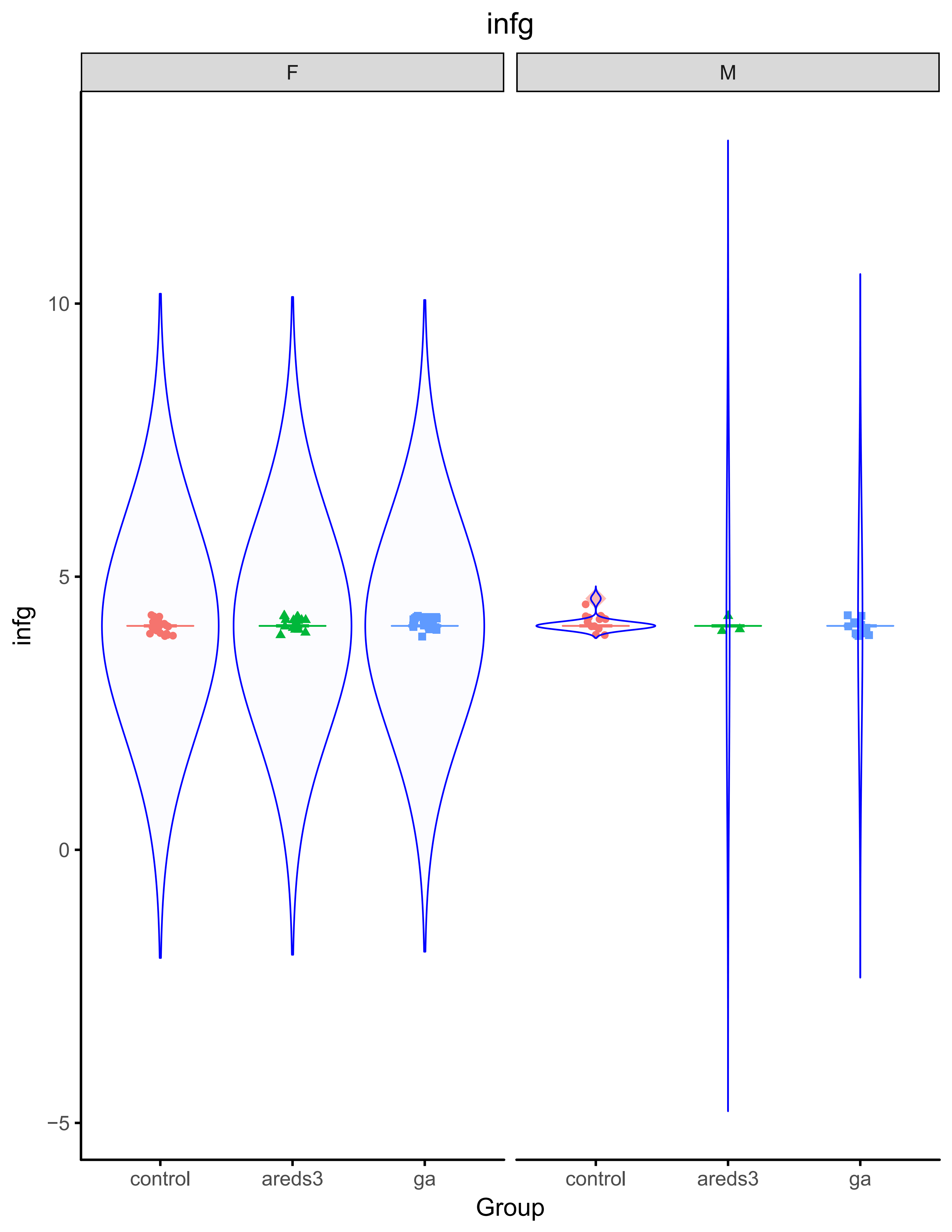

infg
F
M
10
5
0
−5
infg
control
areds3
ga
control
areds3
ga
Group

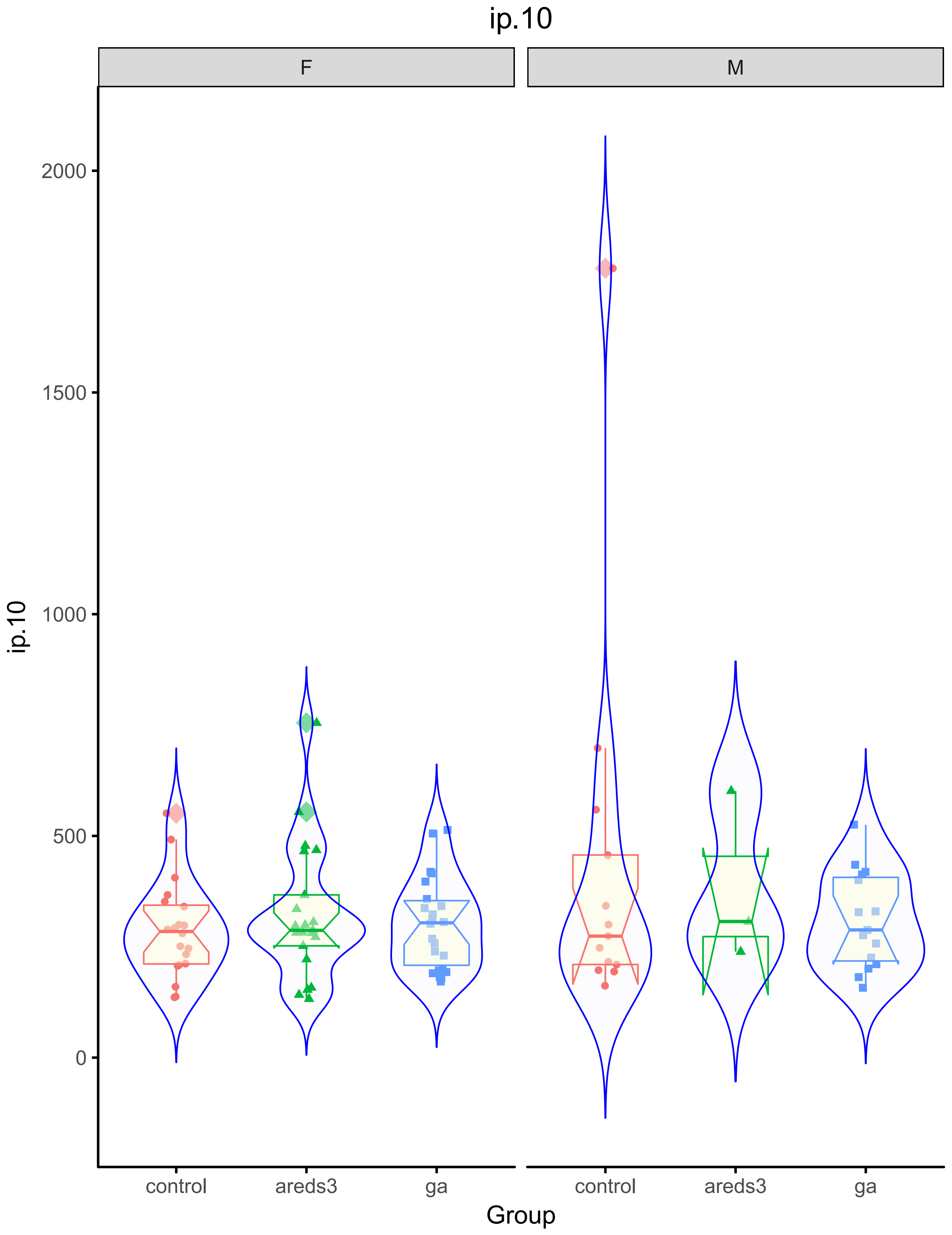

ip.10
F
M
2000
1500
1000
500
0
ip.10
control
areds3
ga
control
areds3
ga
Group

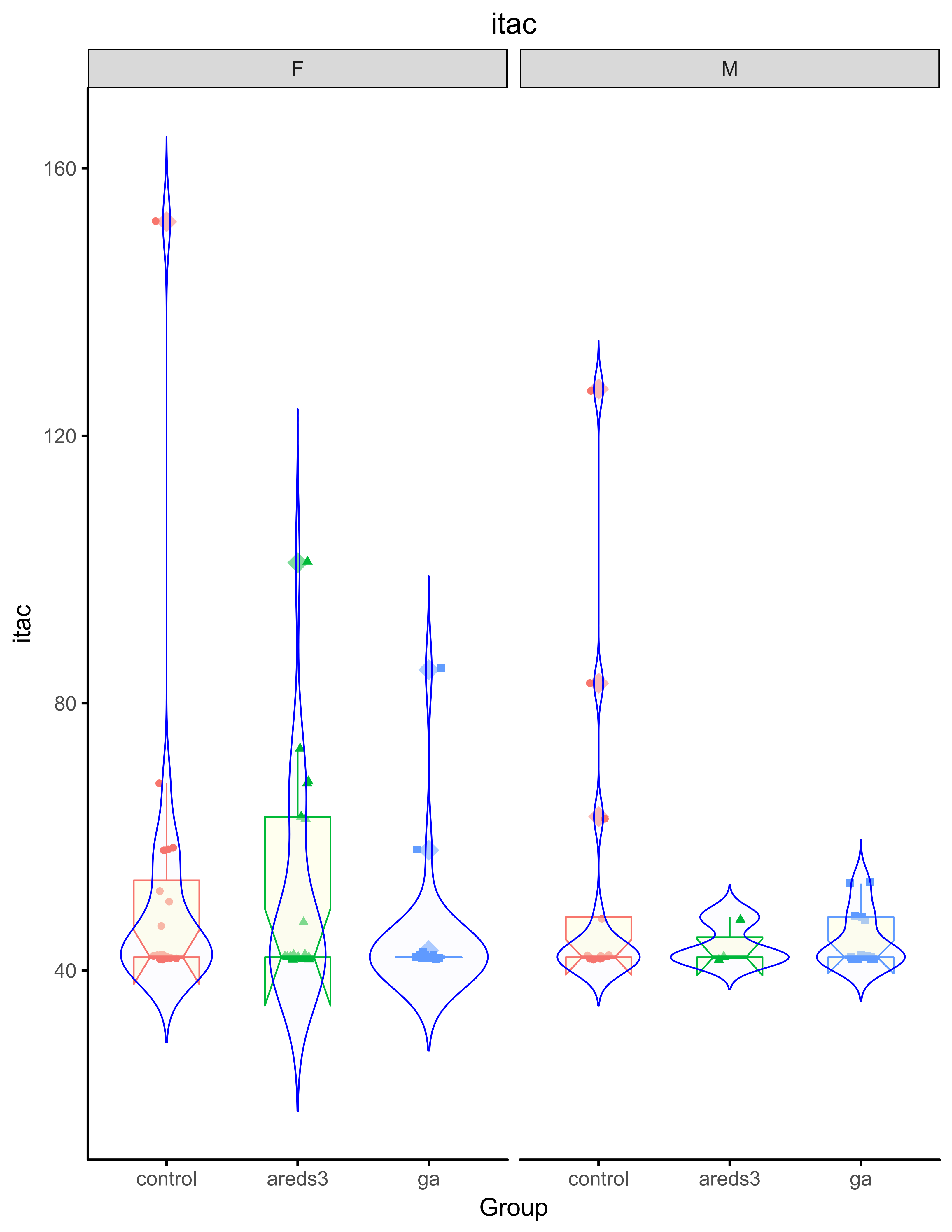

itac
F
M
160
120
80
40
itac
control
areds3
ga
control
areds3
ga
Group

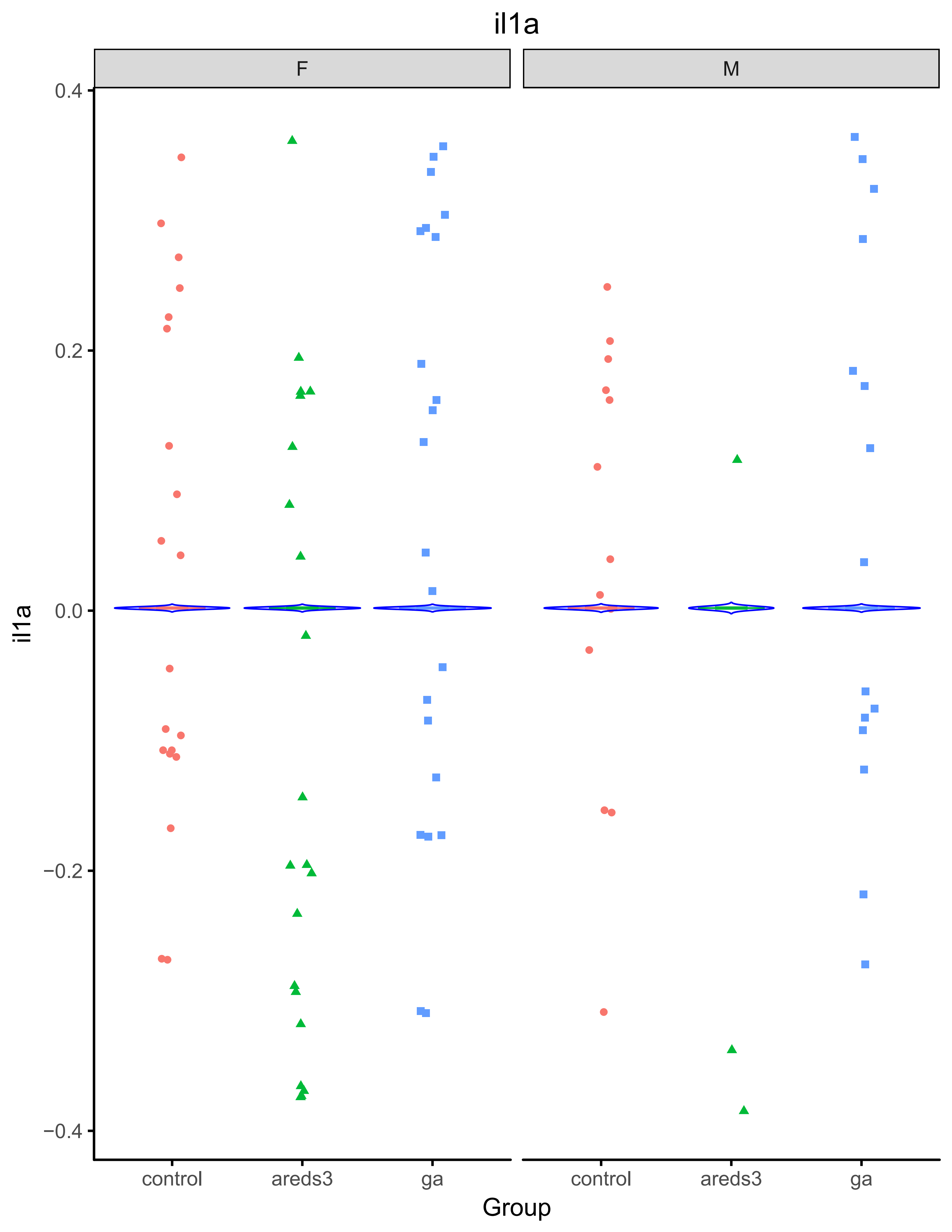

il1a
F
M
0.4
0.2
0.0
−0.2
−0.4
il1a
control
areds3
ga
control
areds3
ga
Group

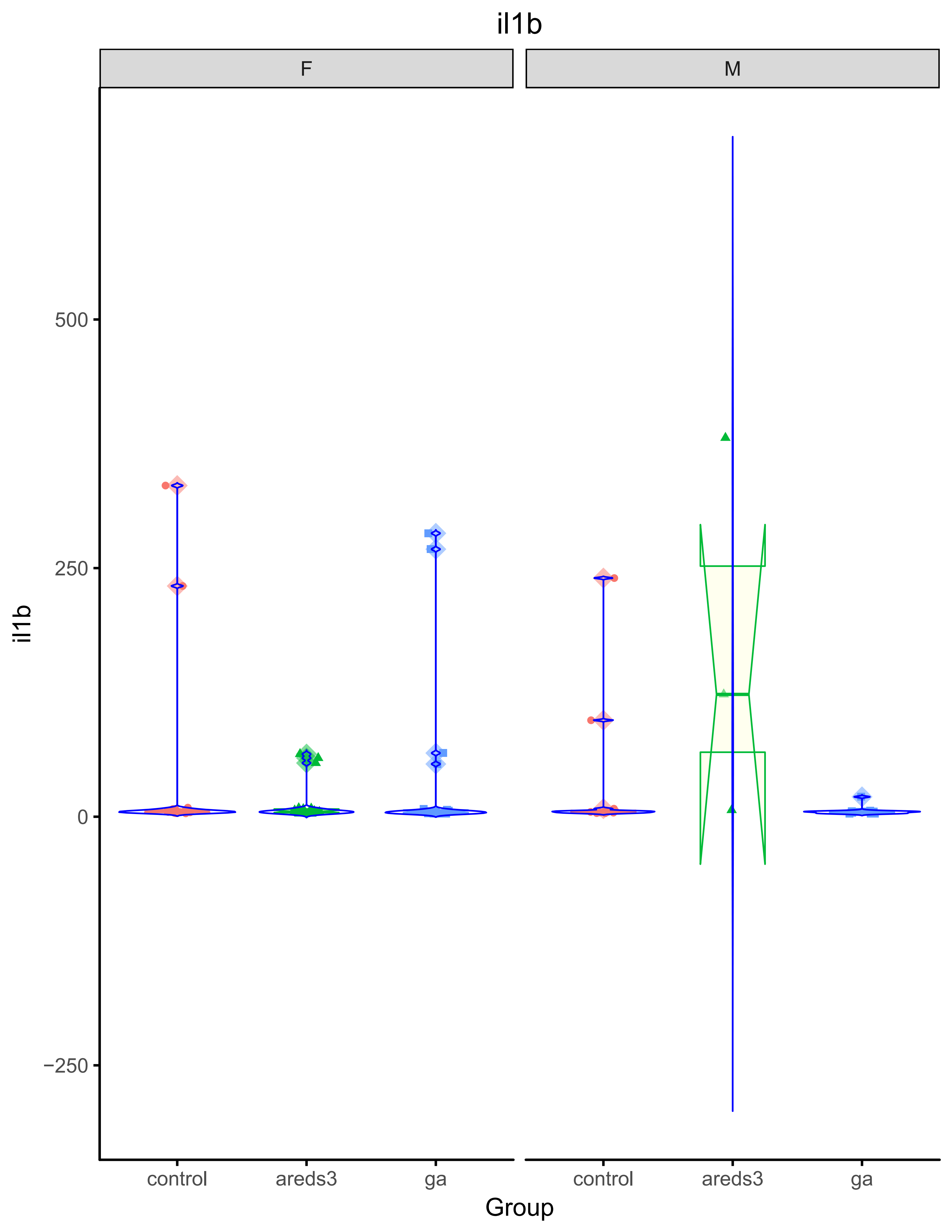

il1b
F
M
500
250
0
−250
il1b
control
areds3
ga
control
areds3
ga
Group

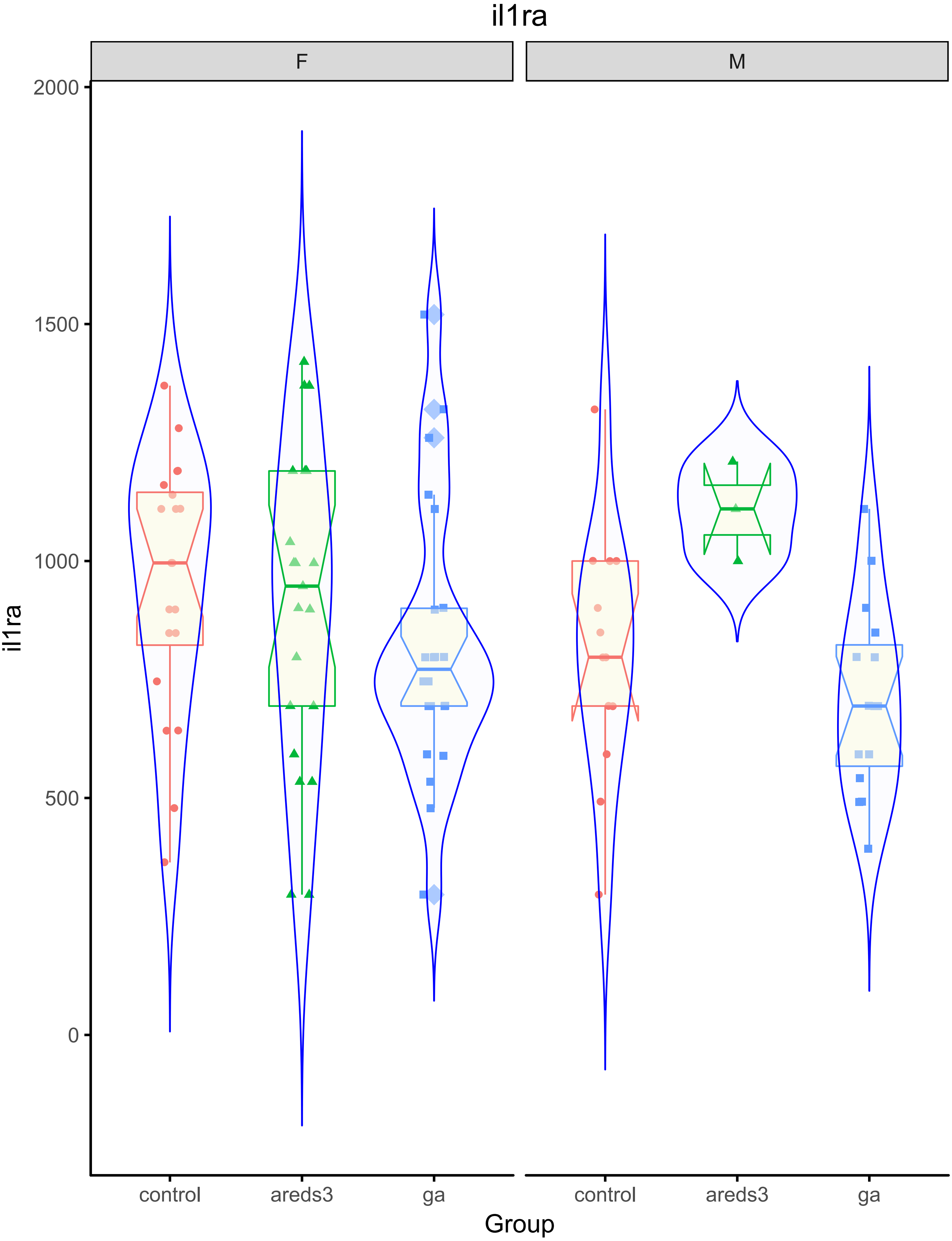

il1ra
F
M
2000
1500
1000
500
0
il1ra
control
areds3
ga
control
areds3
ga
Group

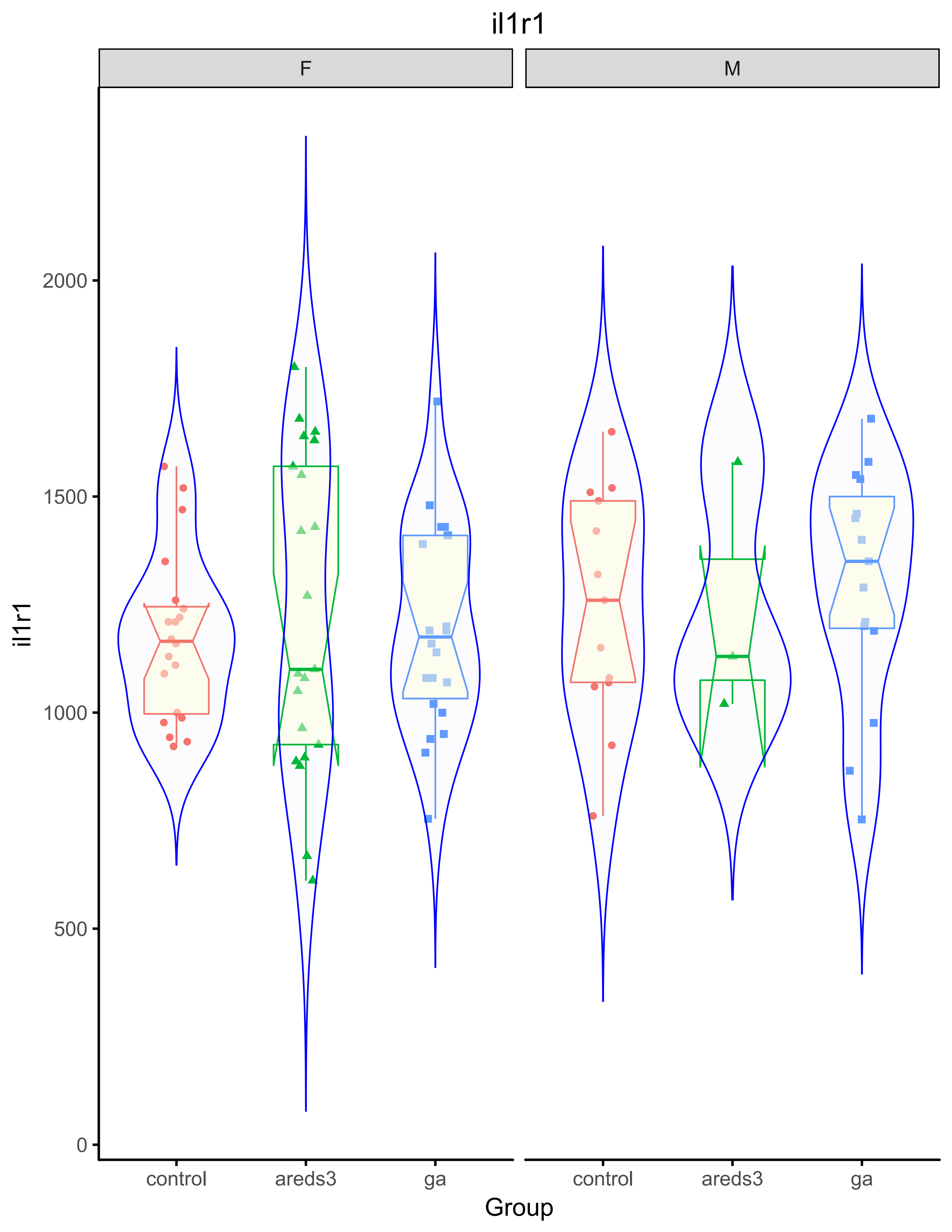

il1r1
F
M
2000
1500
1000
500
0
il1r1
control
areds3
ga
control
areds3
ga
Group

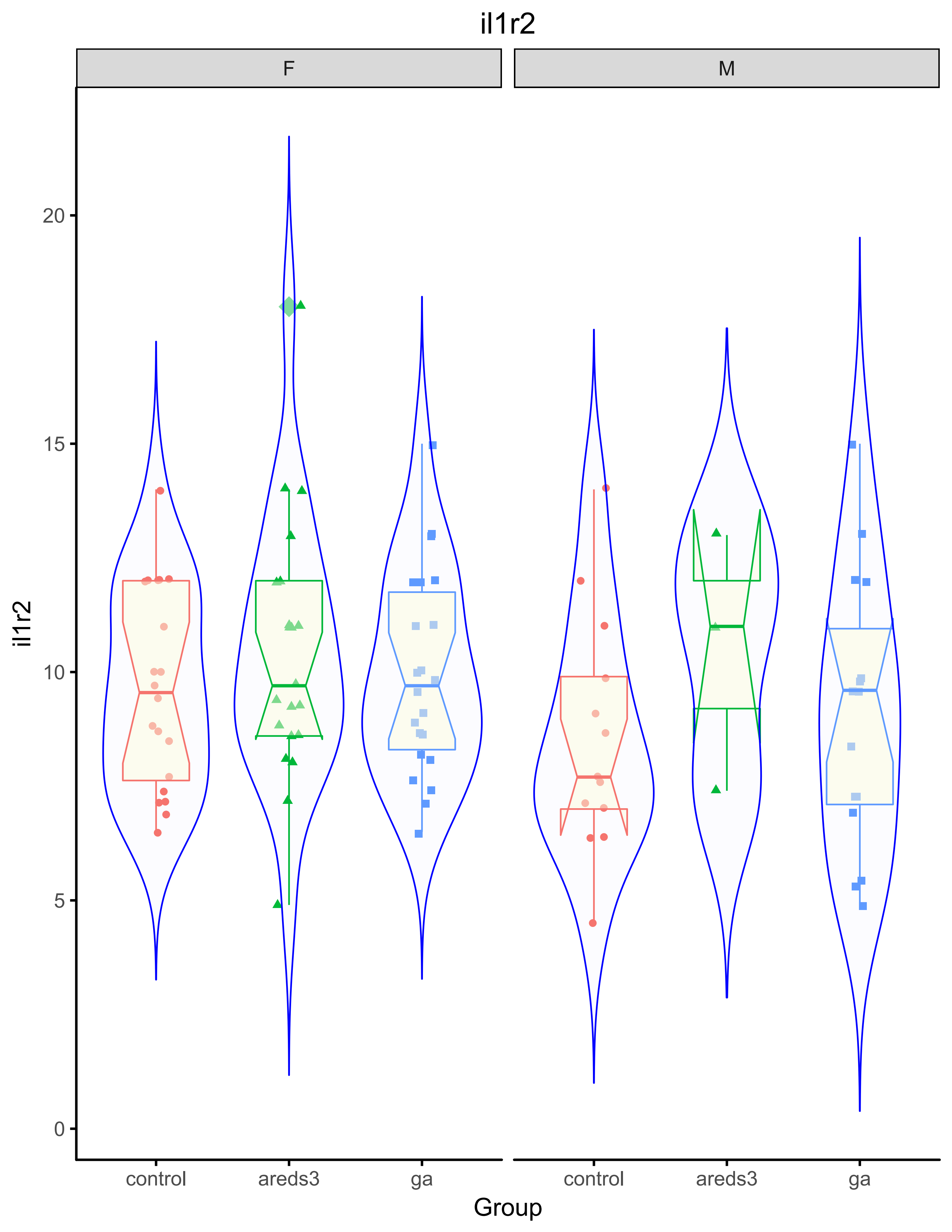

il1r2
F
M
20
15
10
5
0
il1r2
control
areds3
ga
control
areds3
ga
Group

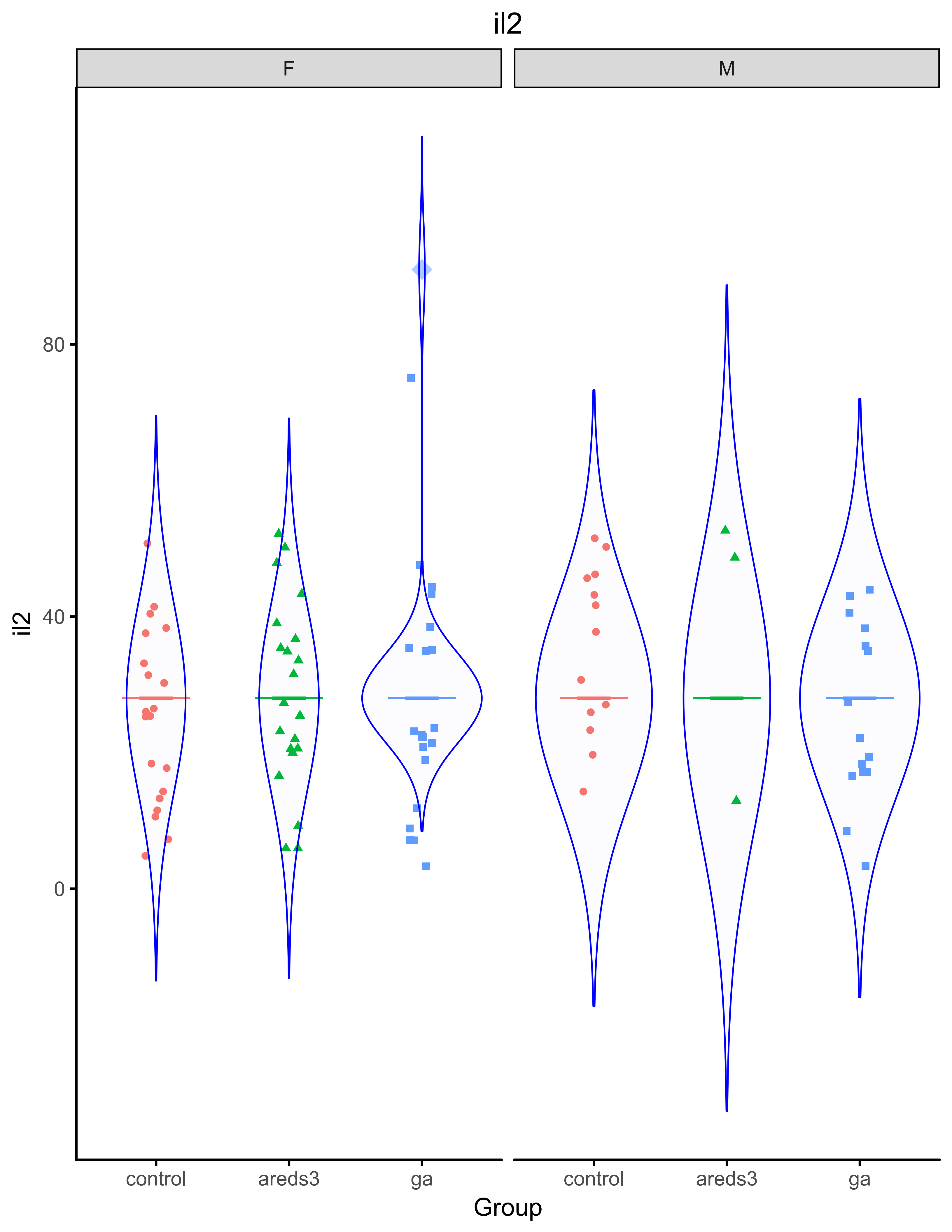

il2
F
M
80
40
0
il2
control
areds3
ga
control
areds3
ga
Group

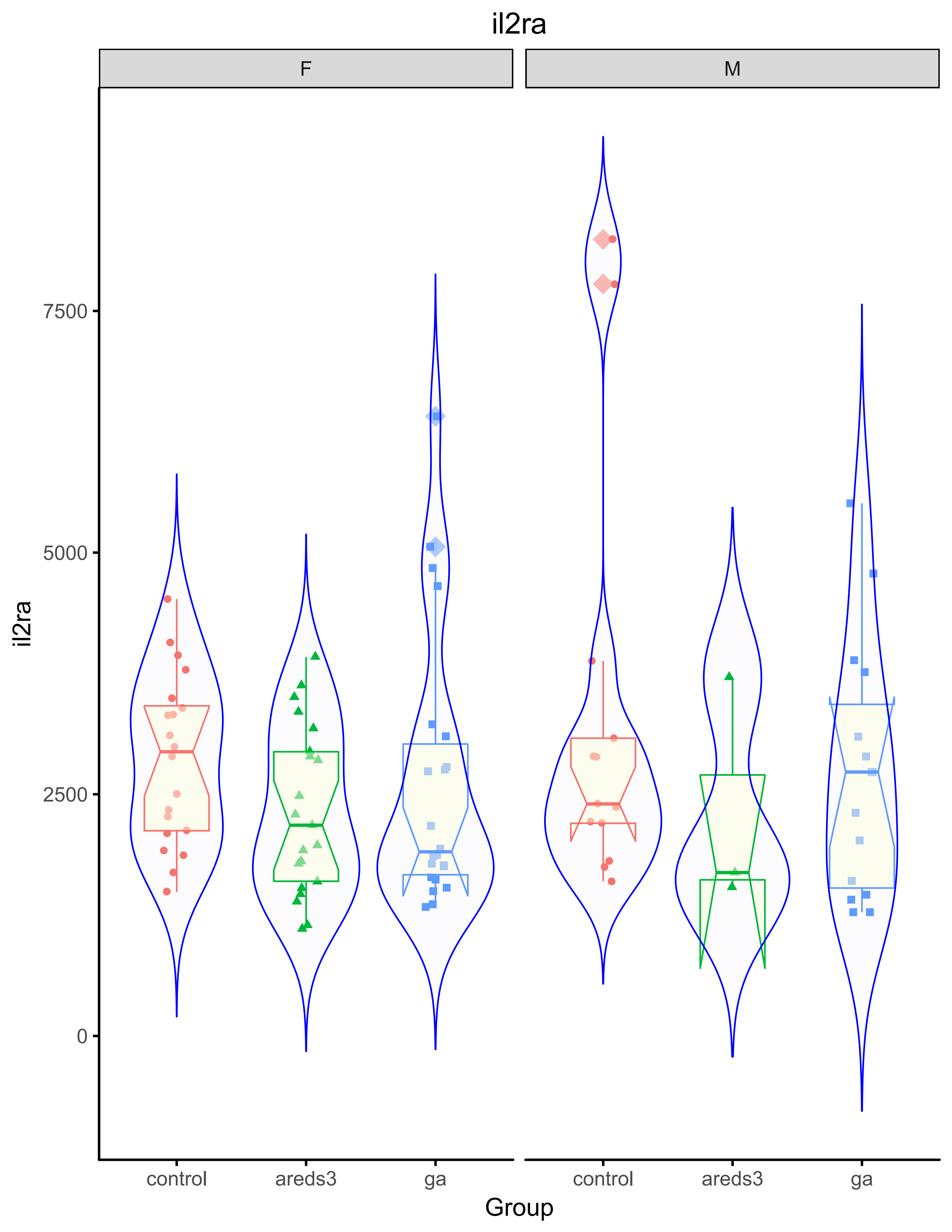
il2ra
F
M
7500
5000
2500
0
il2ra
control
areds3
ga
control
areds3
ga
Group

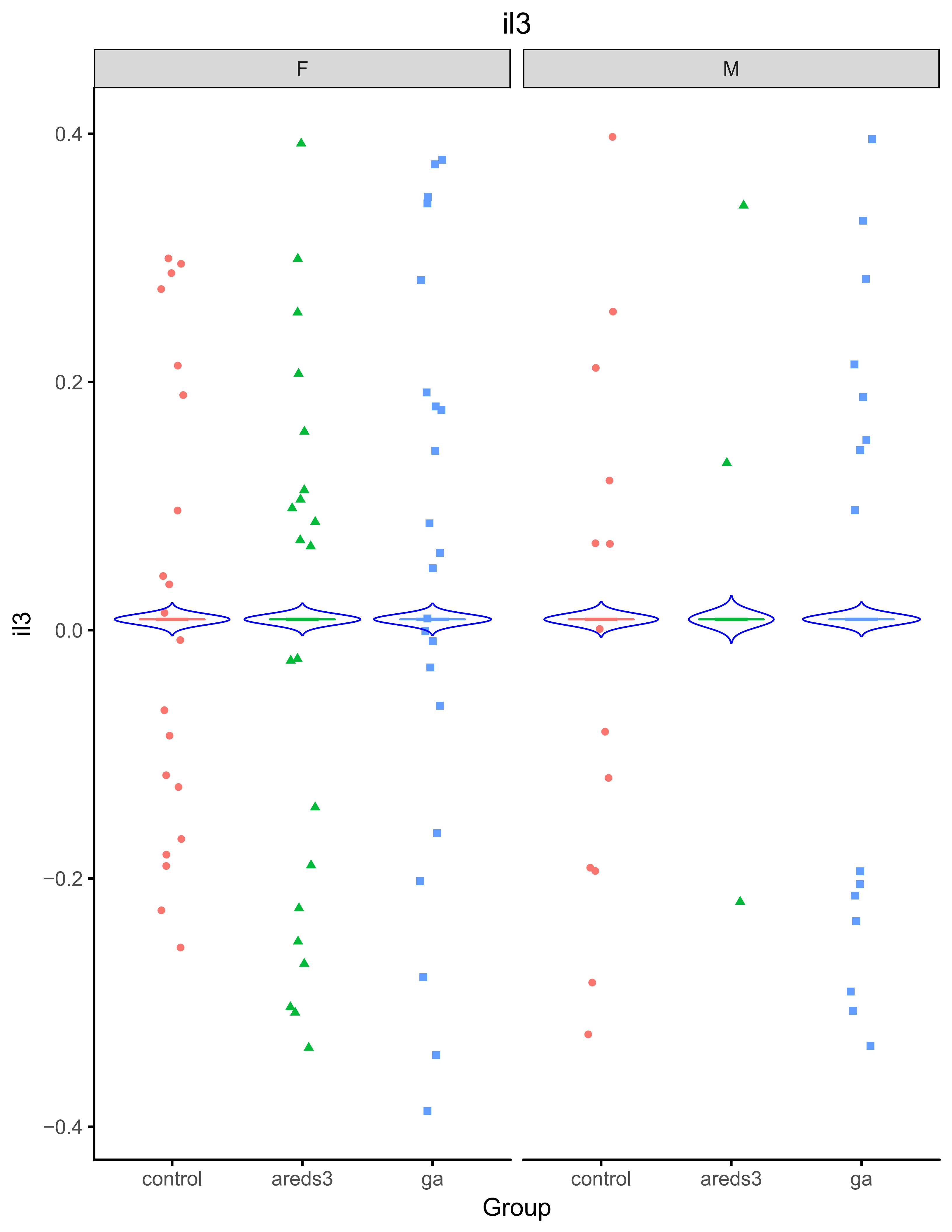

il3
F
M
0.4
0.2
0.0
−0.2
−0.4
il3
control
areds3
ga
control
areds3
ga
Group

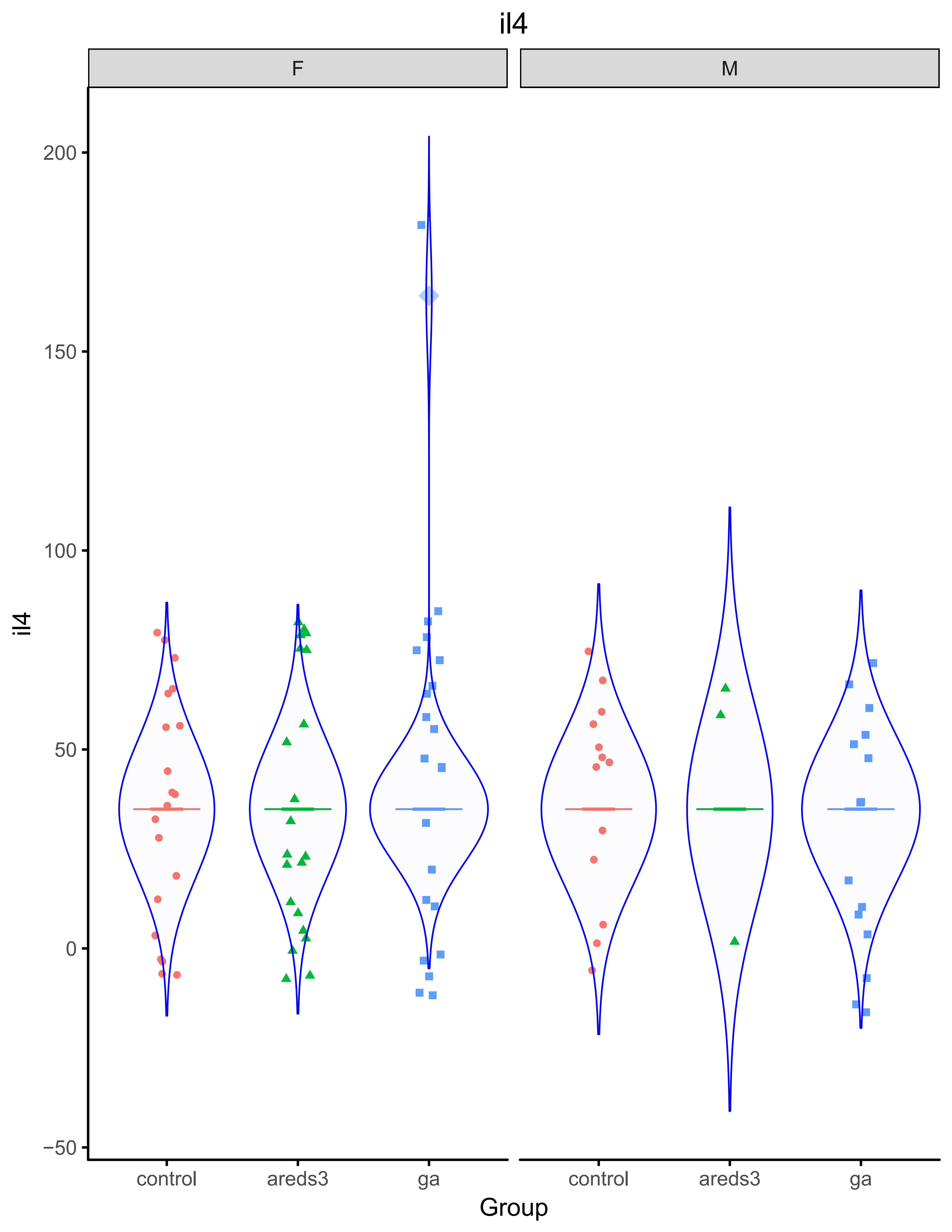

il4
F
M
il4
200
150
100
50
0
−50
control
areds3
ga
control
areds3
ga
Group

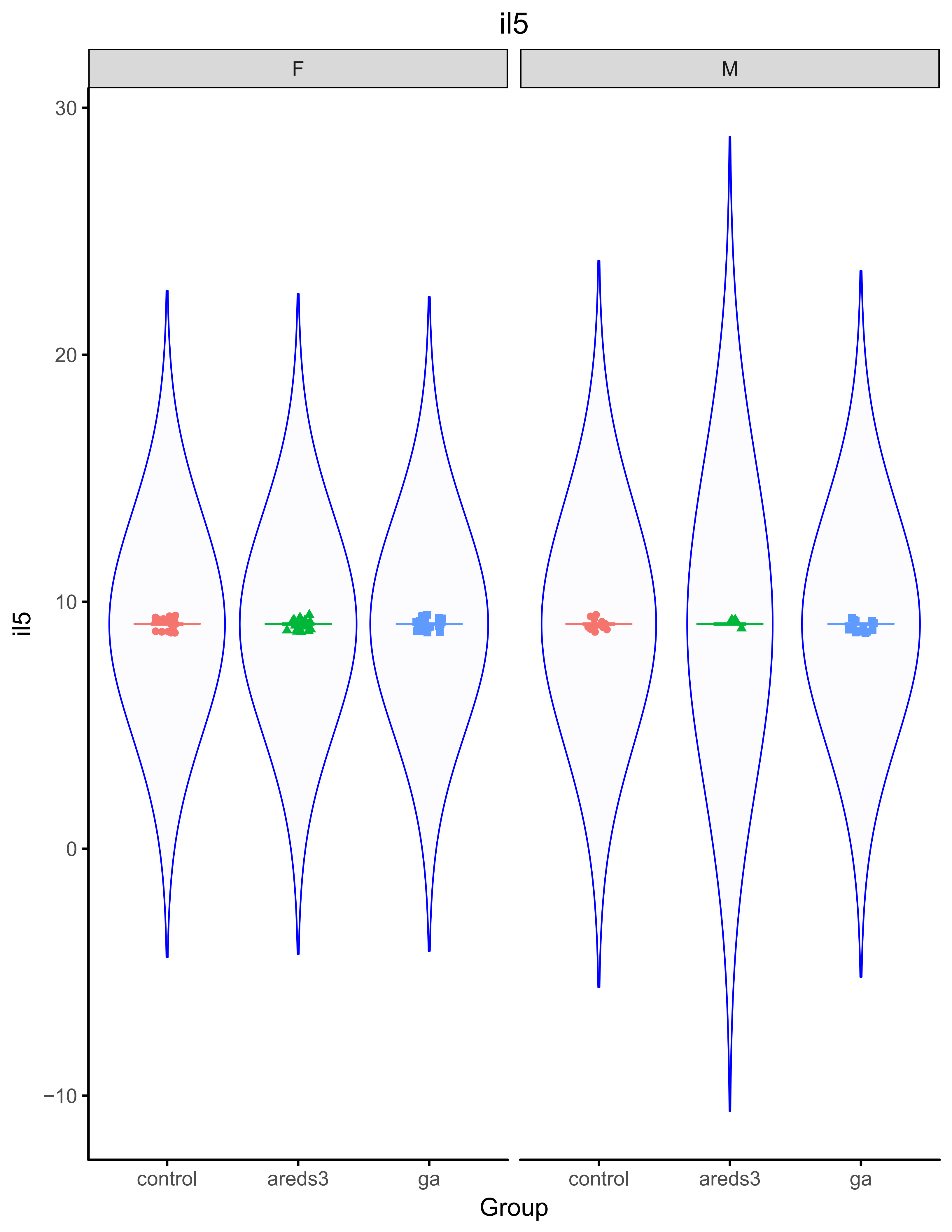

il5
F
M
30
20
10
0
−10
il5
control
areds3
ga
control
areds3
ga
Group

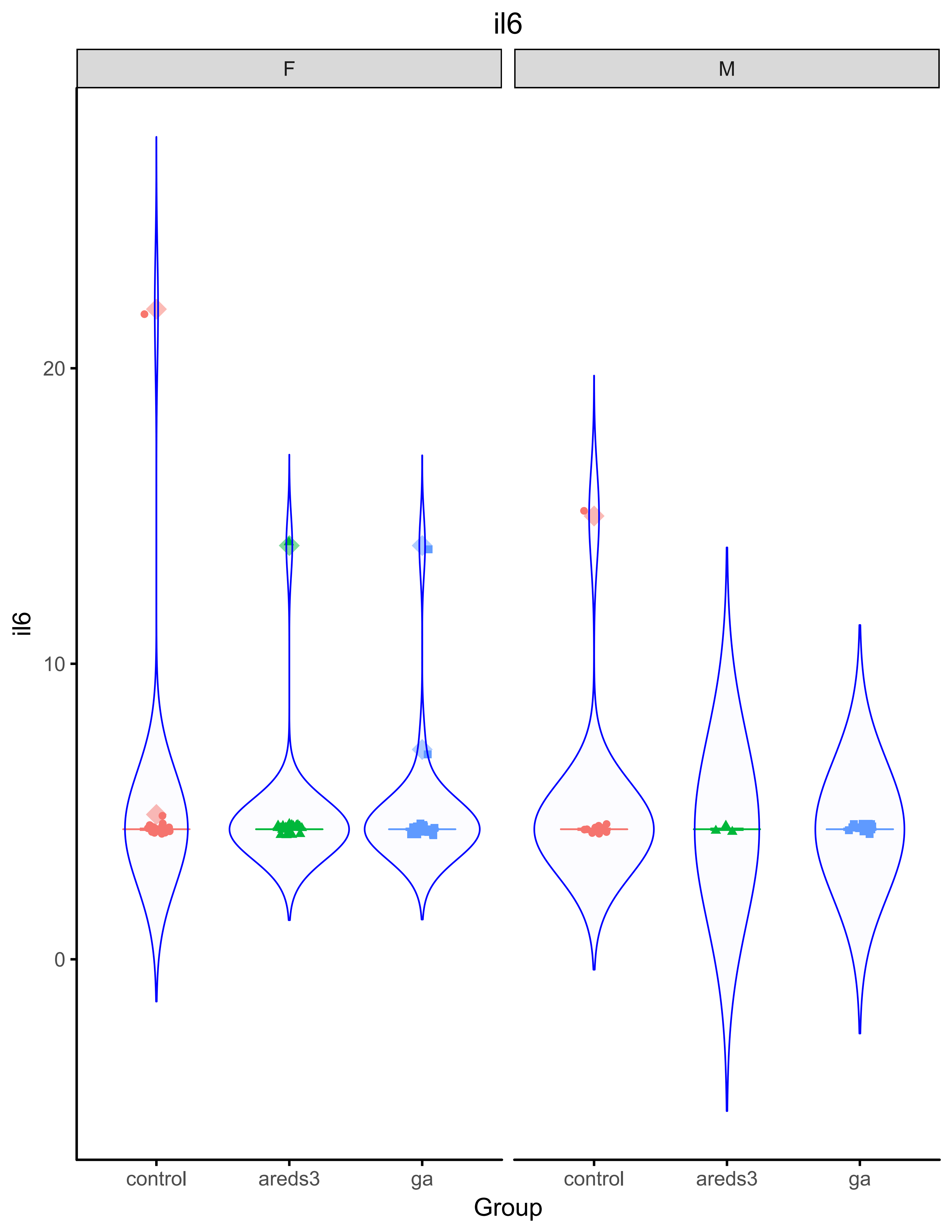
il6
F
M
il6
20
10
0
control
areds3
ga
control
areds3
ga
Group

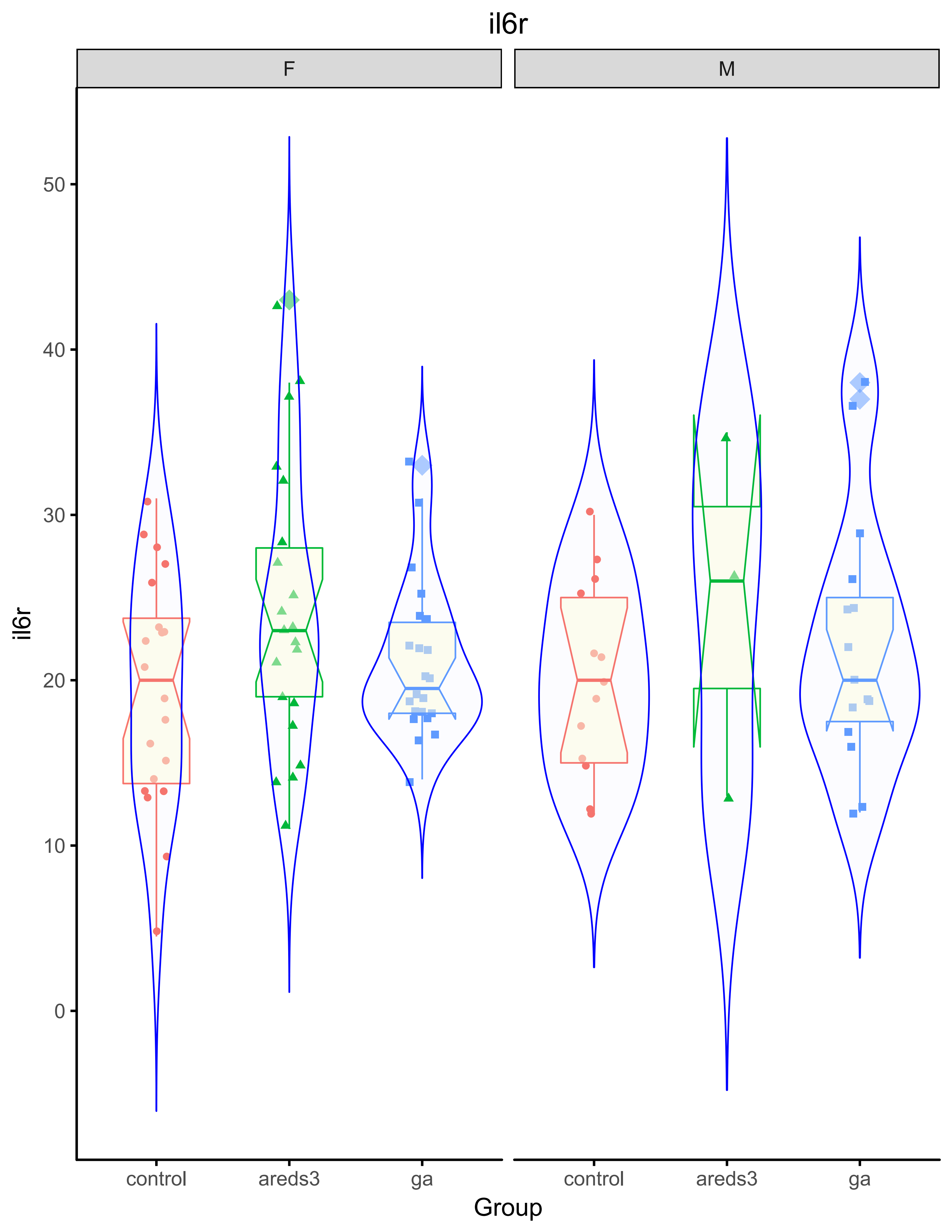

il6r
F
M
50
40
30
20
10
0
il6r
control
areds3
ga
control
areds3
ga
Group

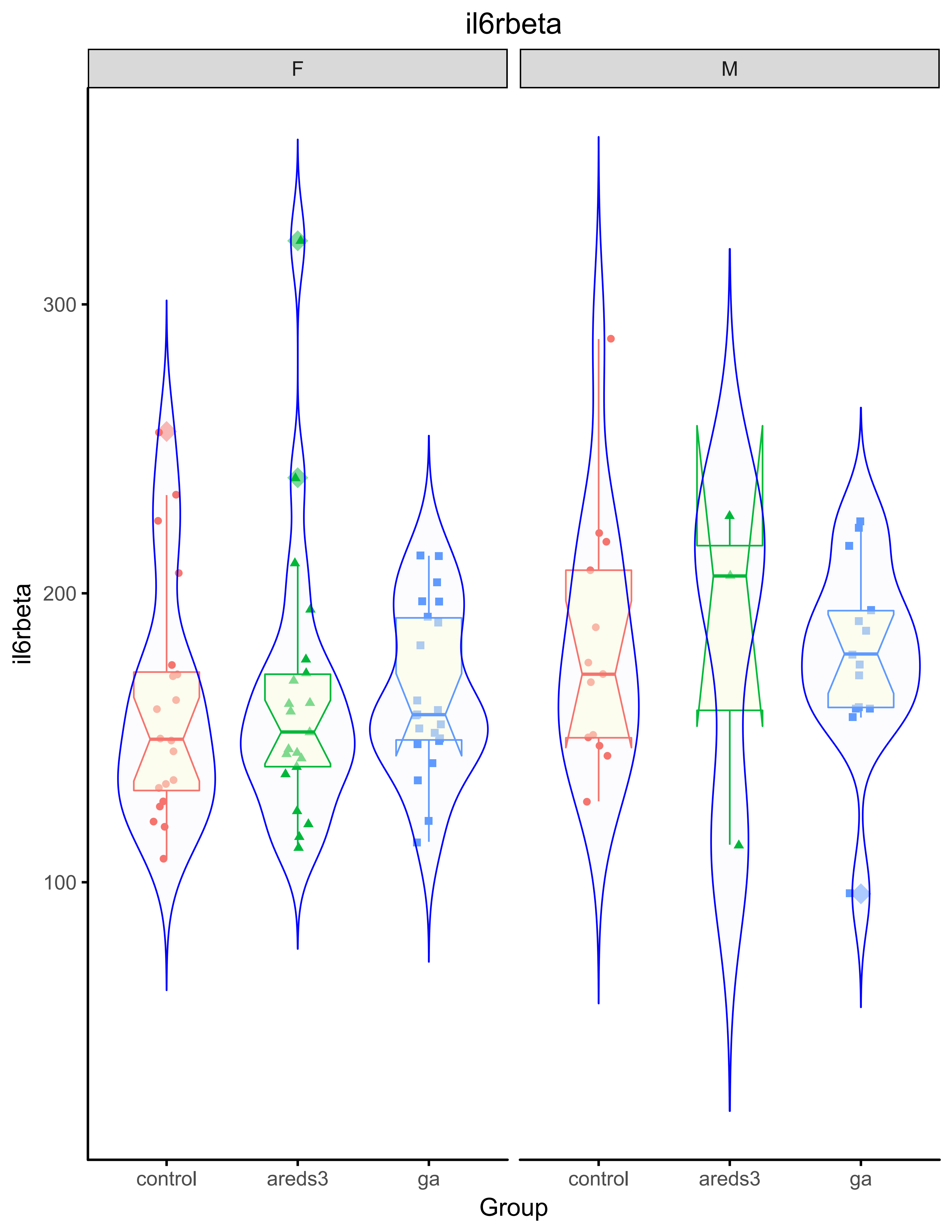
il6rbeta
F
M
300
200
100
il6rbeta
control
areds3
ga
control
areds3
ga
Group

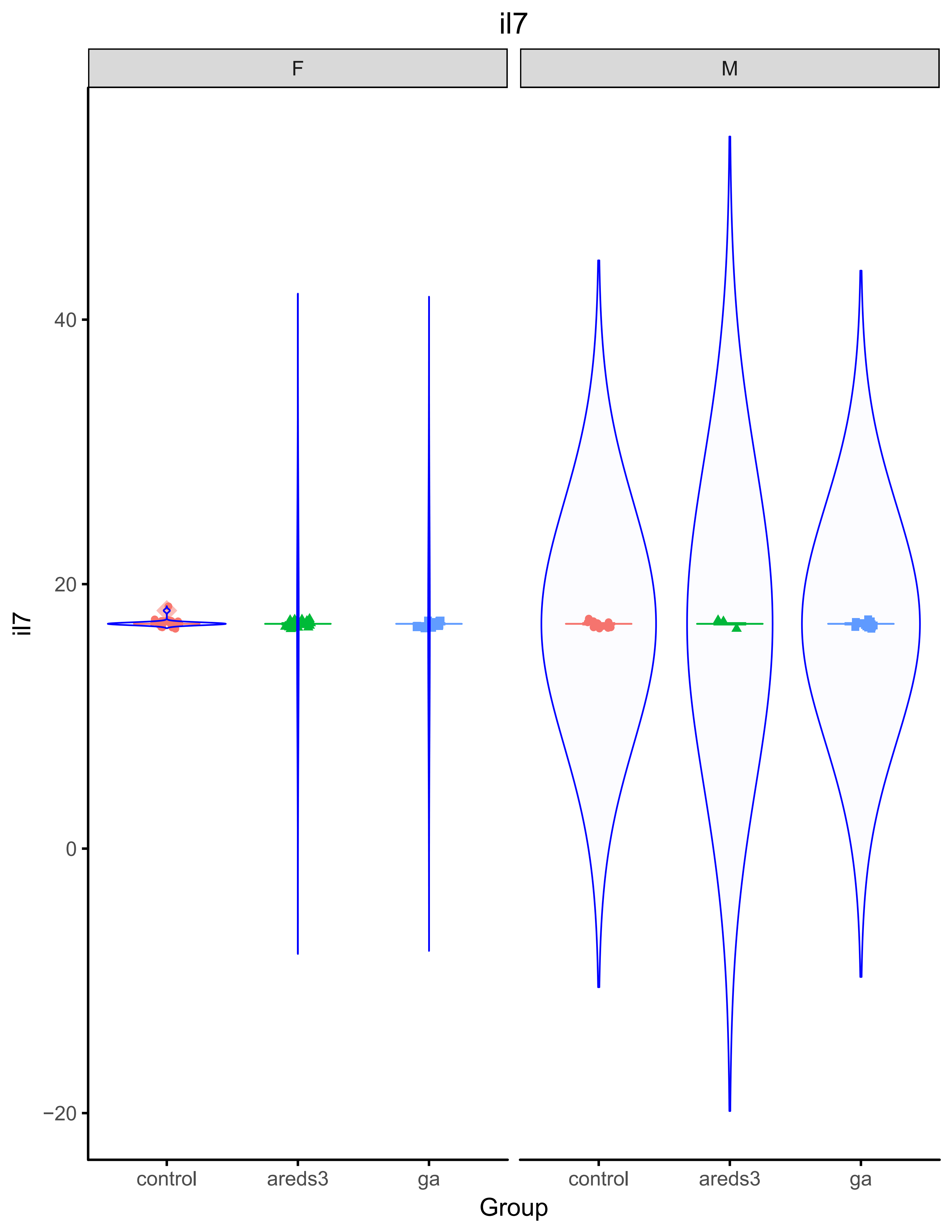
il7
F
M
40
20
0
−20
il7
control
areds3
ga
control
areds3
ga
Group

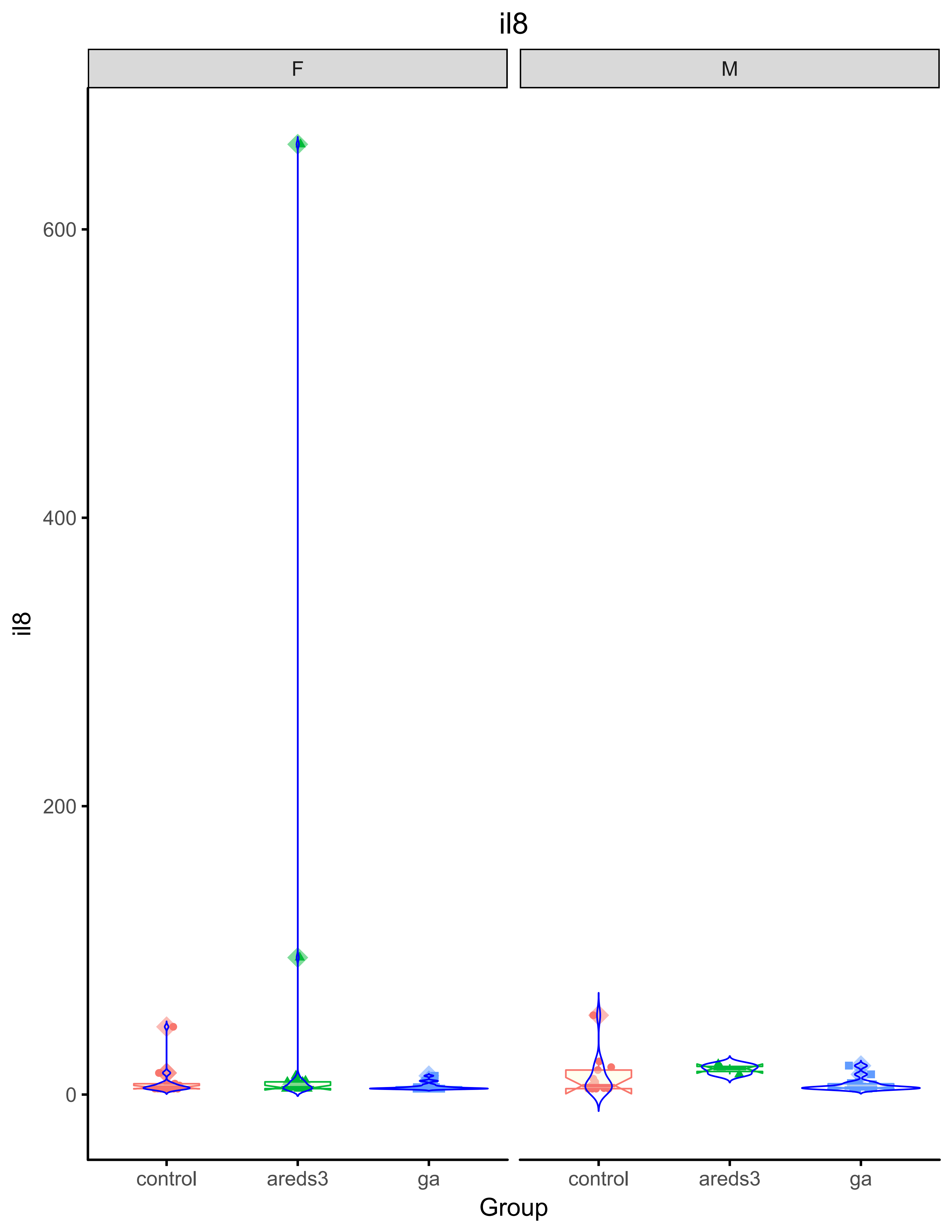

il8
F
M
600
400
200
0
il8
control
areds3
ga
control
areds3
ga
Group

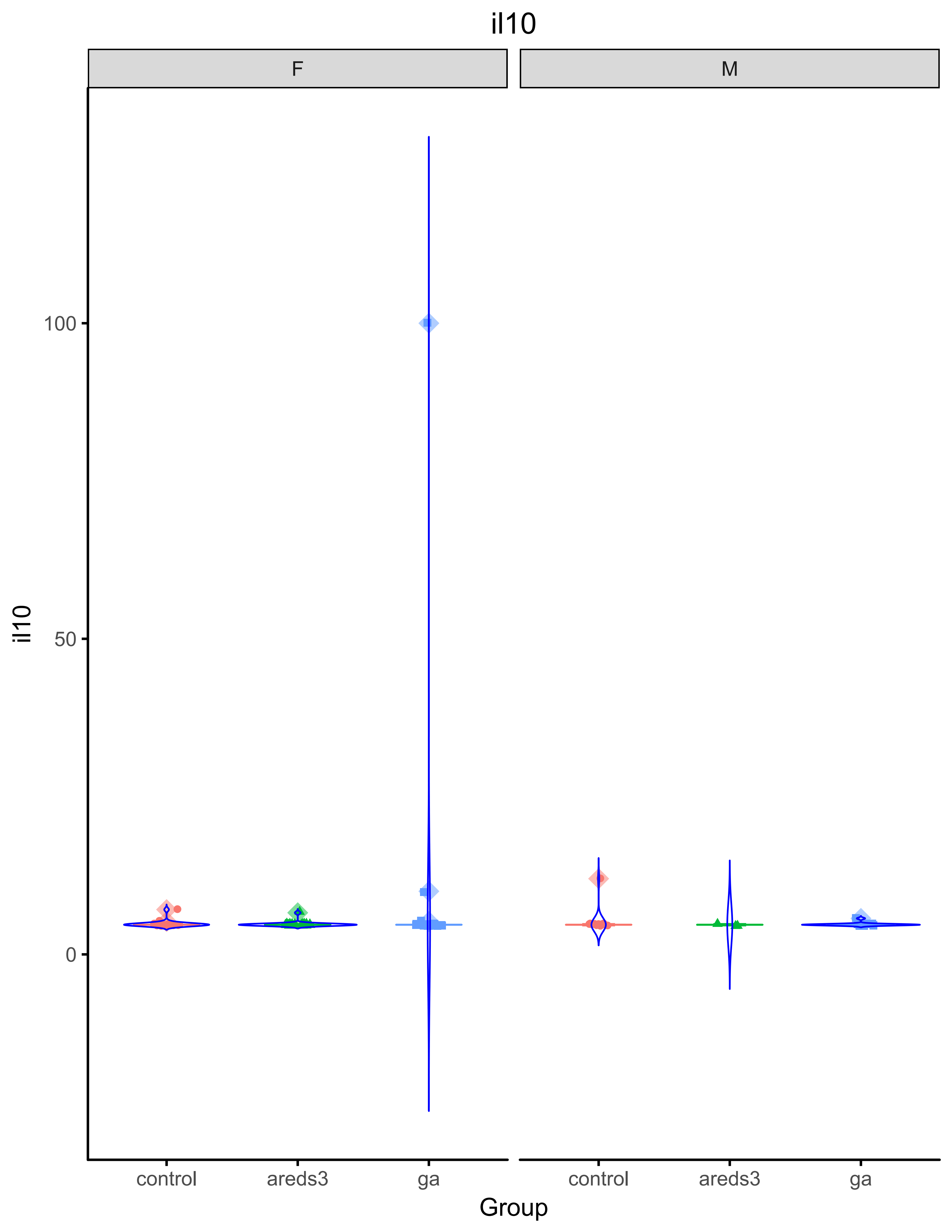

il10
F
M
100
50
0
il10
control
areds3
ga
control
areds3
ga
Group

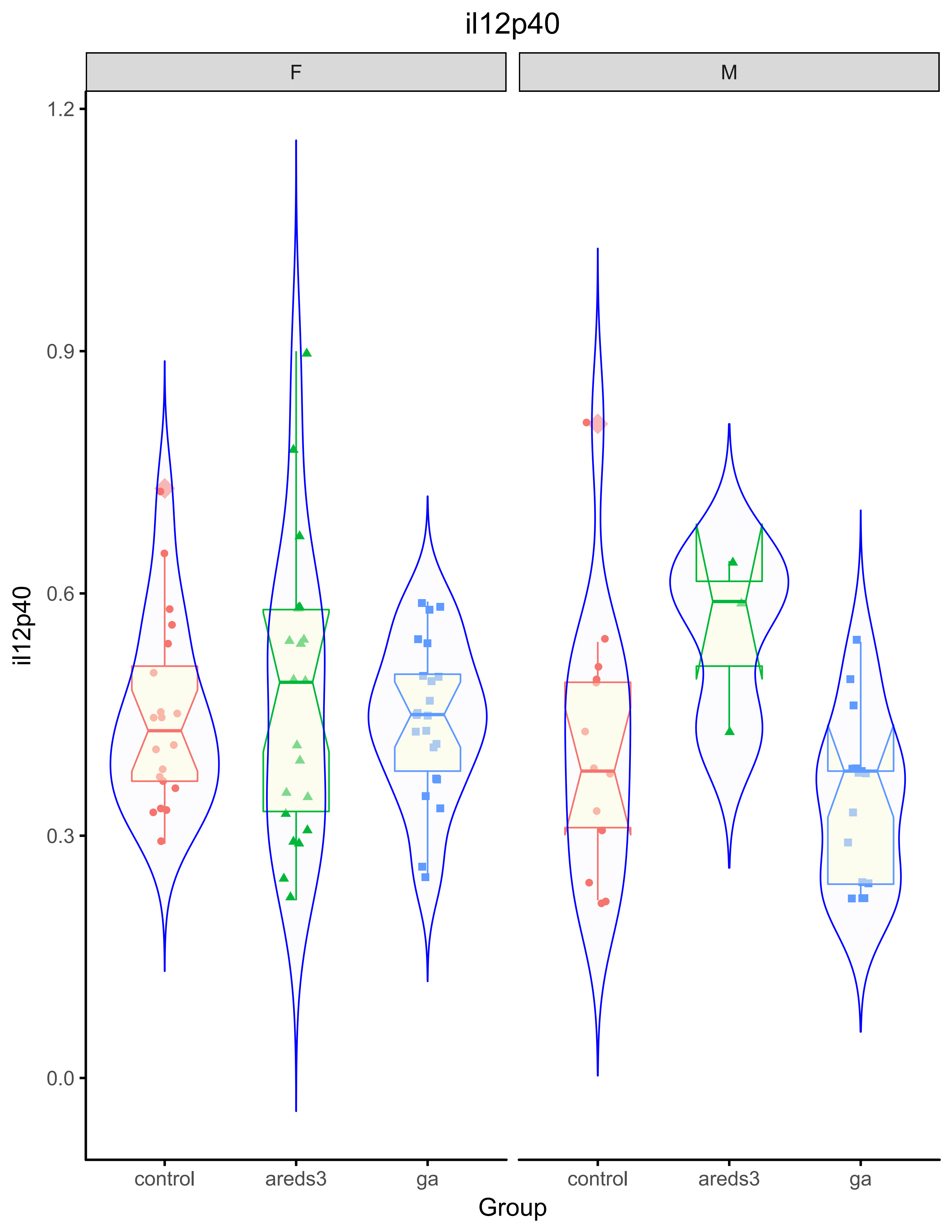
il12p40
F
M
1.2
0.9
0.6
0.3
0.0
il12p40
control
areds3
ga
control
areds3
ga
Group

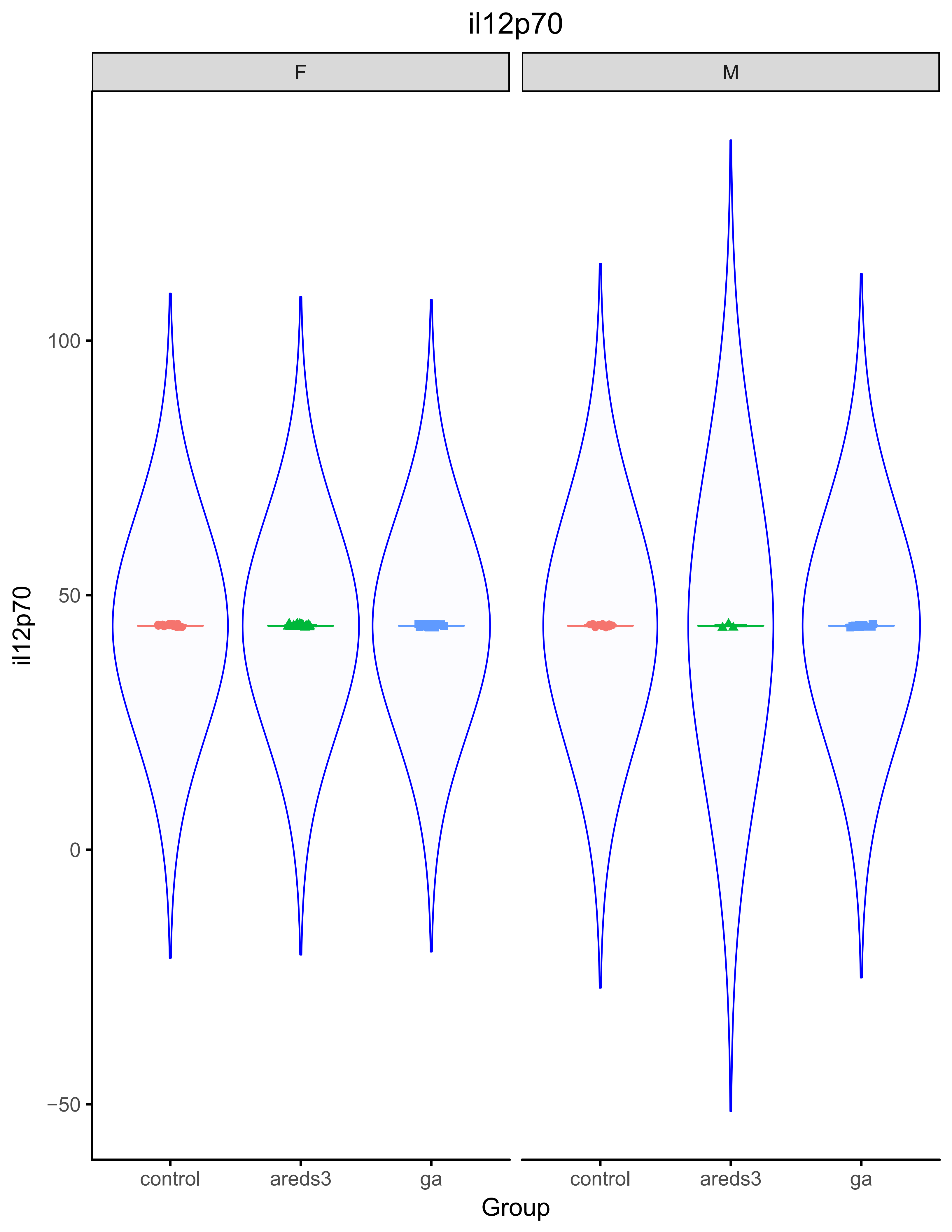
il12p70
F
M
il12p70
100
50
0
−50
control
areds3
ga
control
areds3
ga
Group

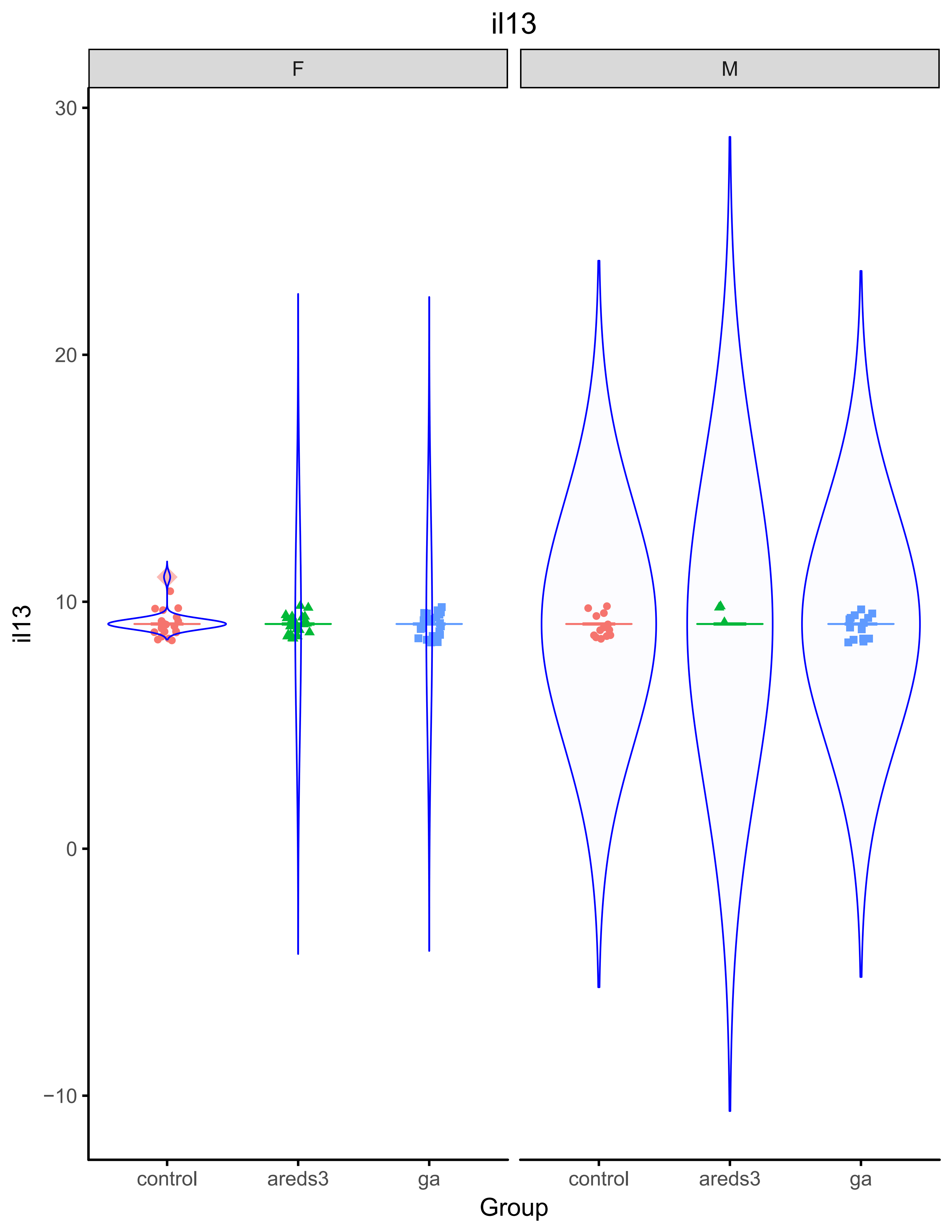
il13
F
M
30
20
10
0
−10
il13
control
areds3
ga
control
areds3
ga
Group

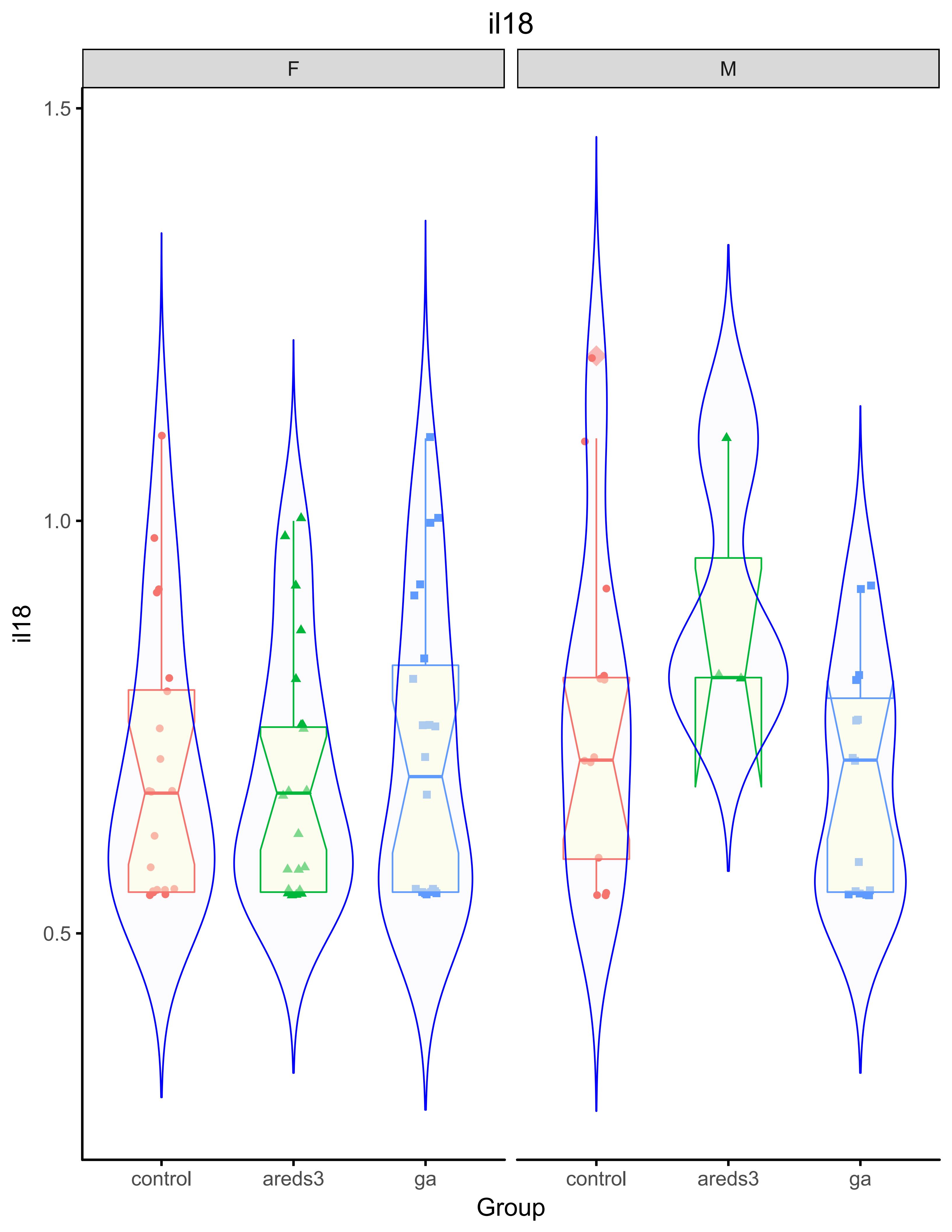

il18
F
M
1.5
1.0
0.5
il18
control
areds3
ga
control
areds3
ga
Group

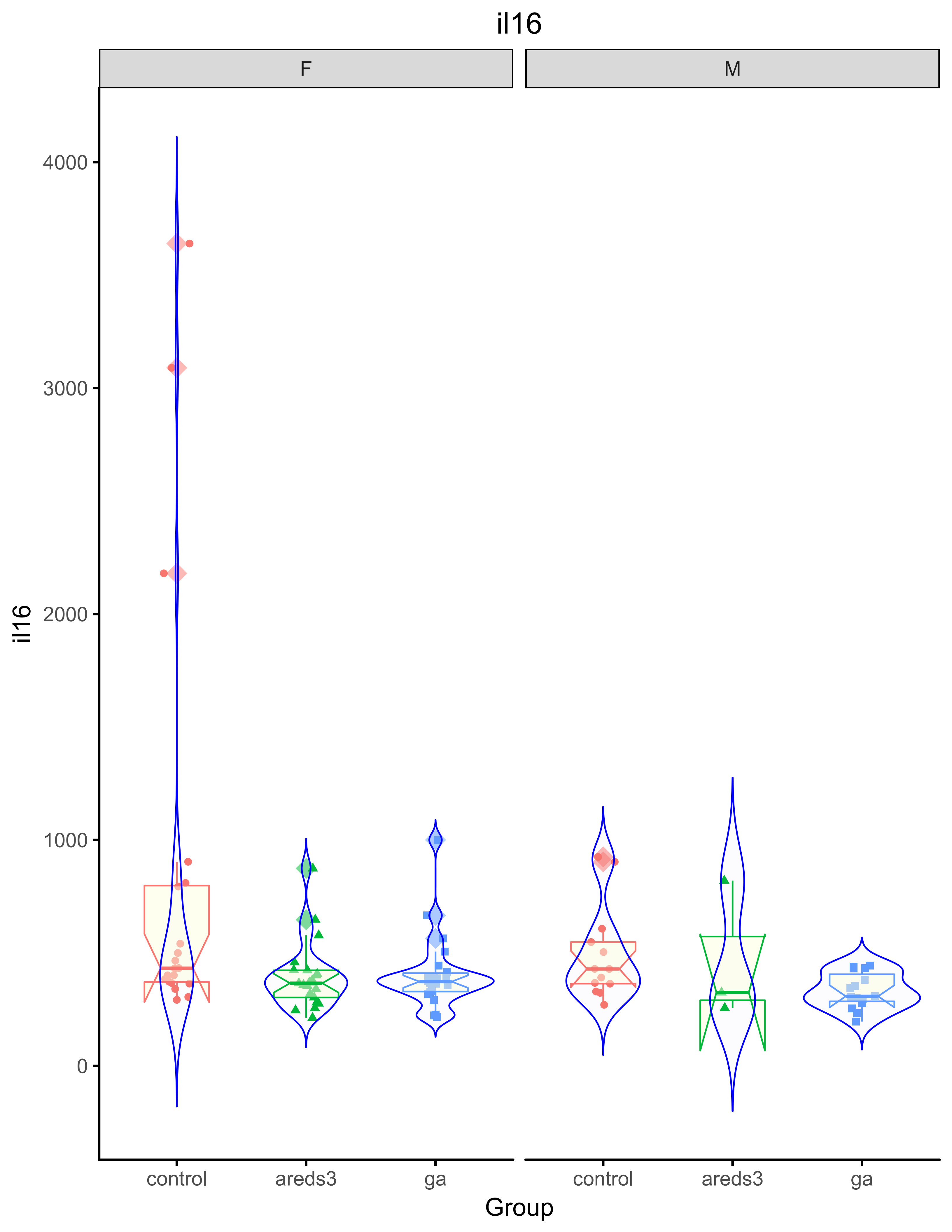

il16
F
M
4000
3000
2000
1000
0
il16
control
areds3
ga
control
areds3
ga
Group

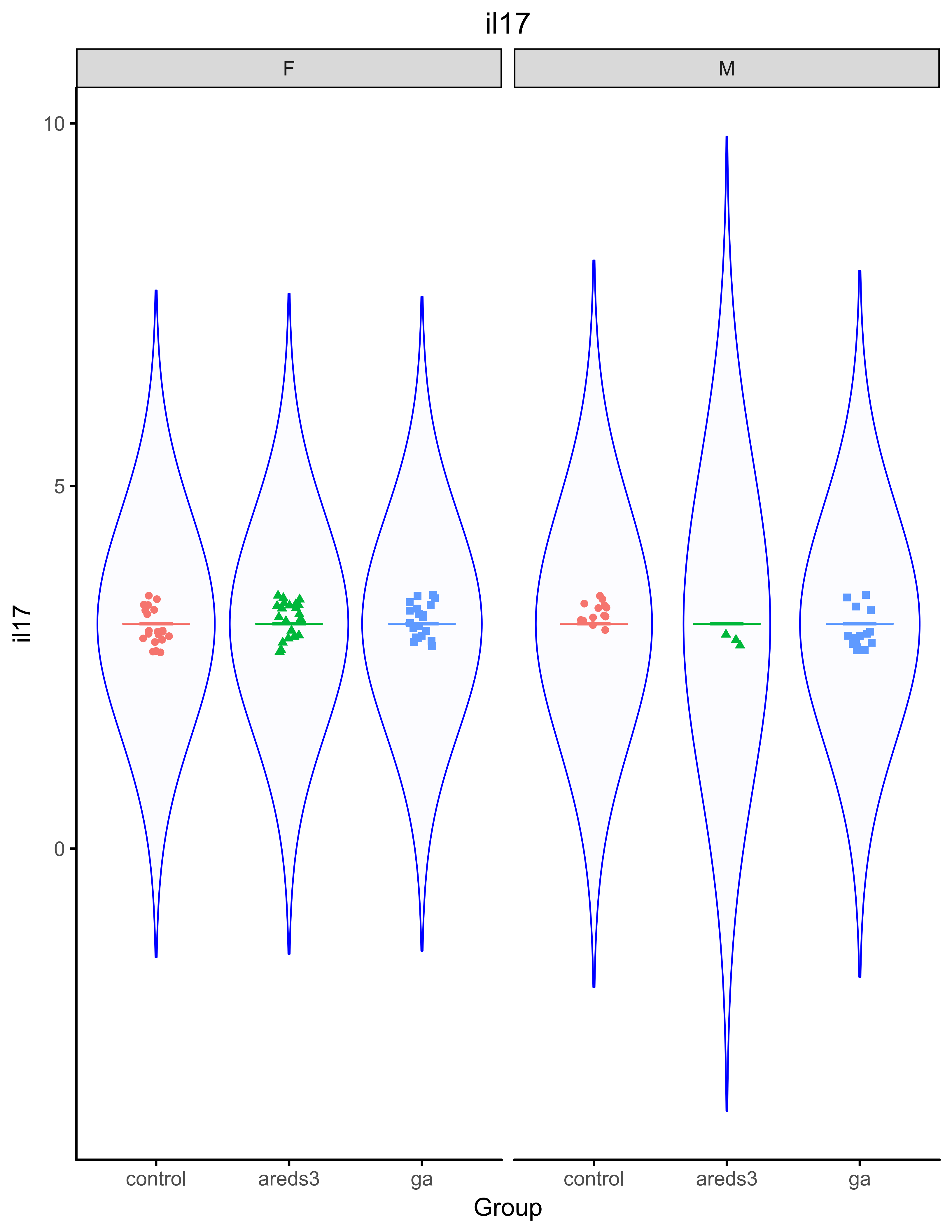
il17
F
M
10
5
0
il17
control
areds3
ga
control
areds3
ga
Group

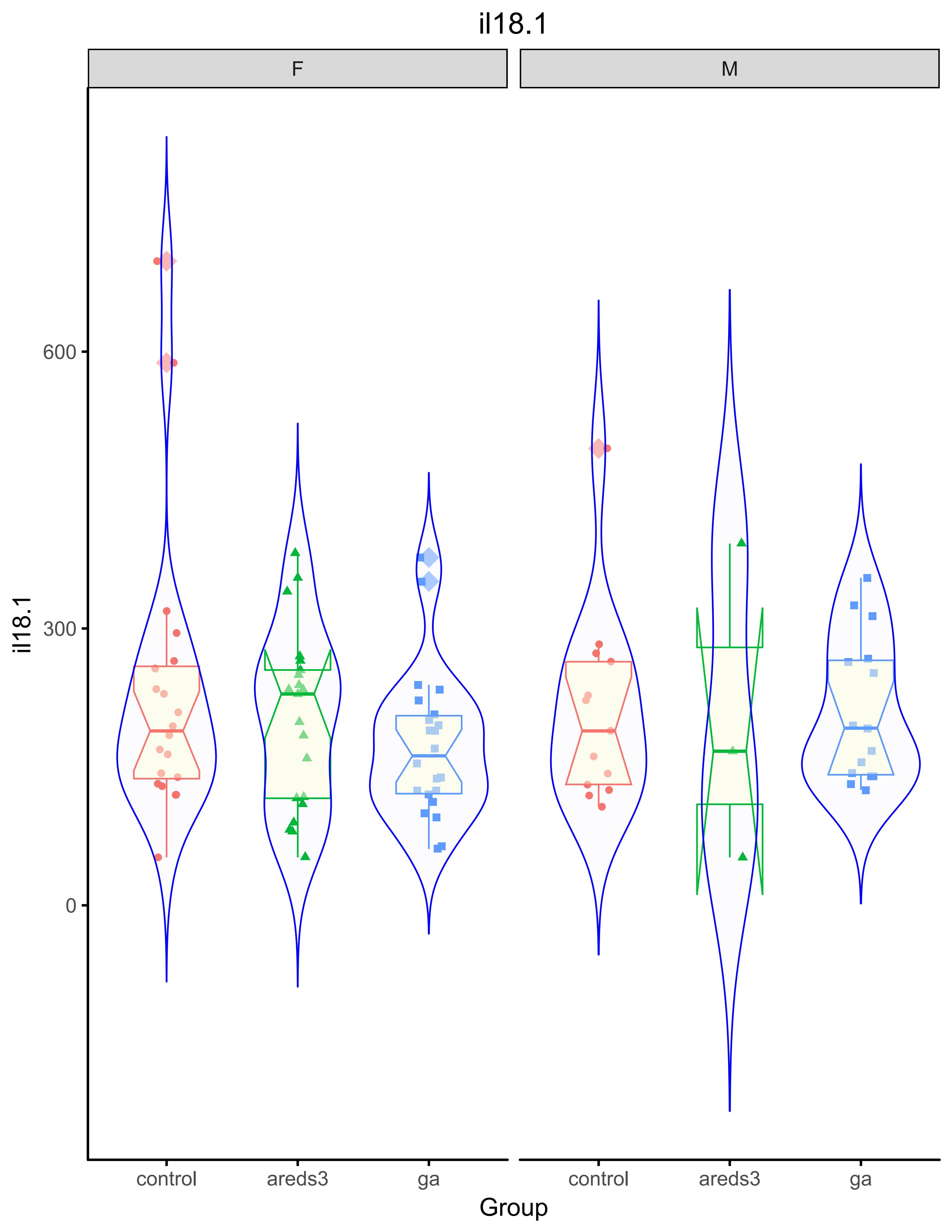
il18.1
F
M
600
300
0
il18.1
control
areds3
ga
control
areds3
ga
Group

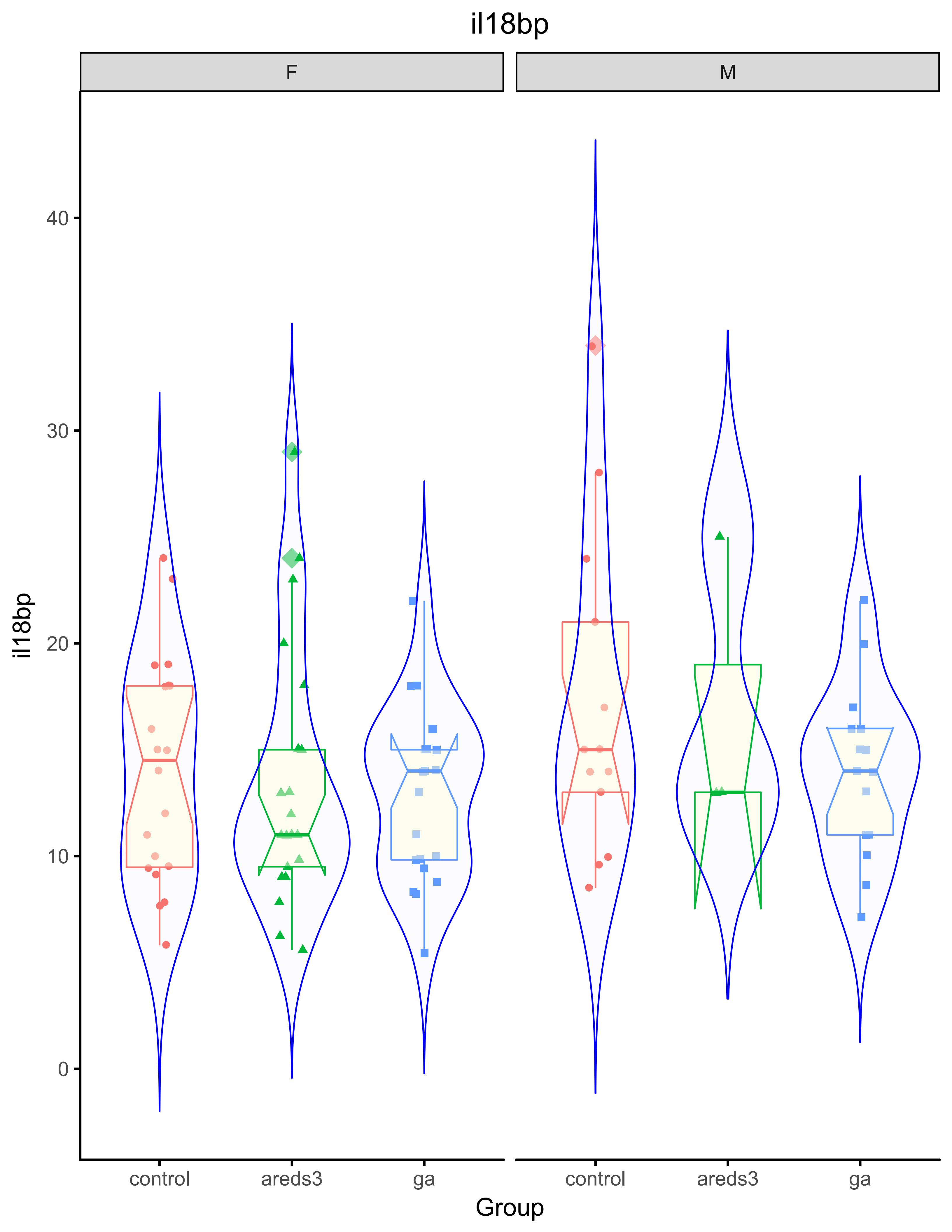

il18bp
F
M
40
30
20
10
0
il18bp
control
areds3
ga
control
areds3
ga
Group

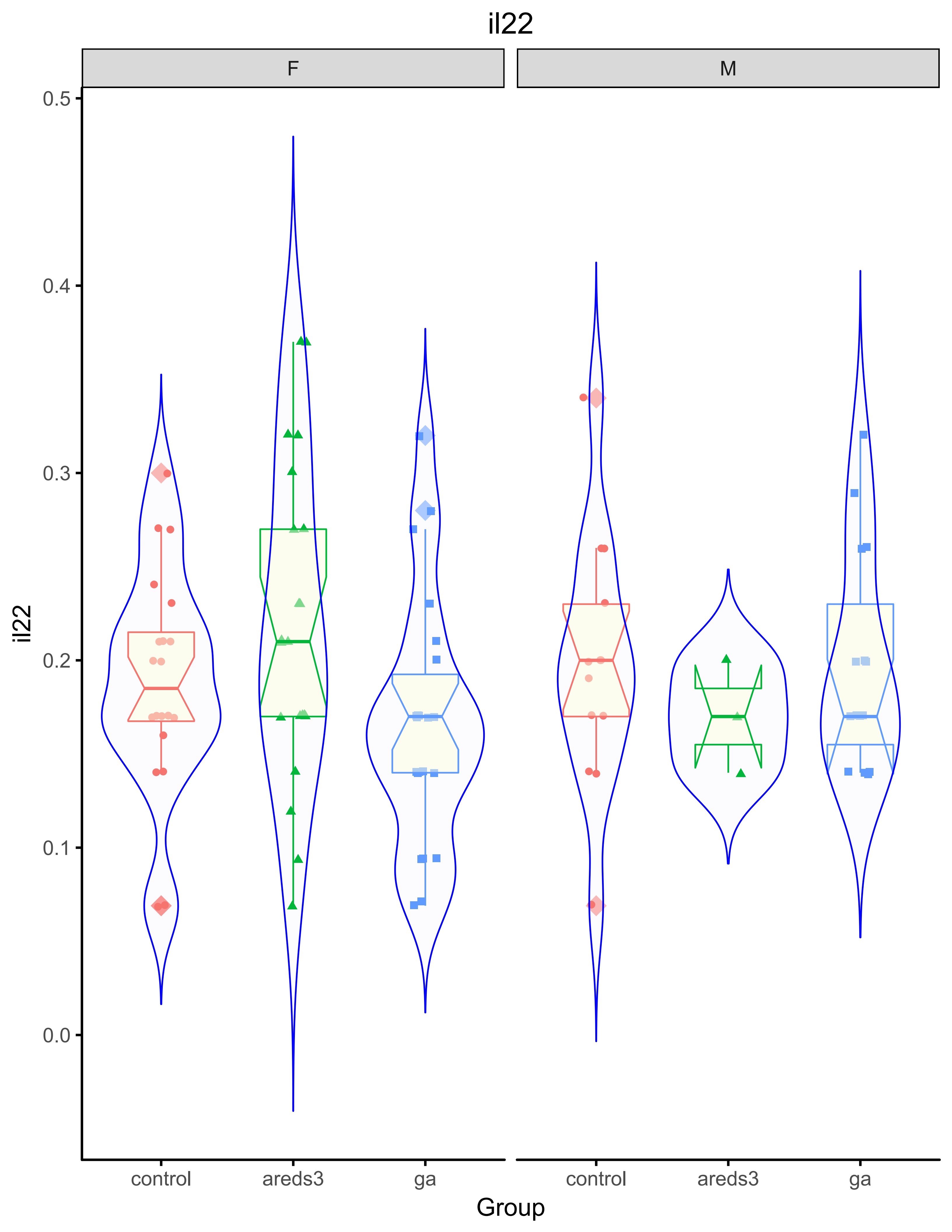
il22
F
M
0.5
0.4
0.3
0.2
0.1
0.0
il22
control
areds3
ga
control
areds3
ga
Group

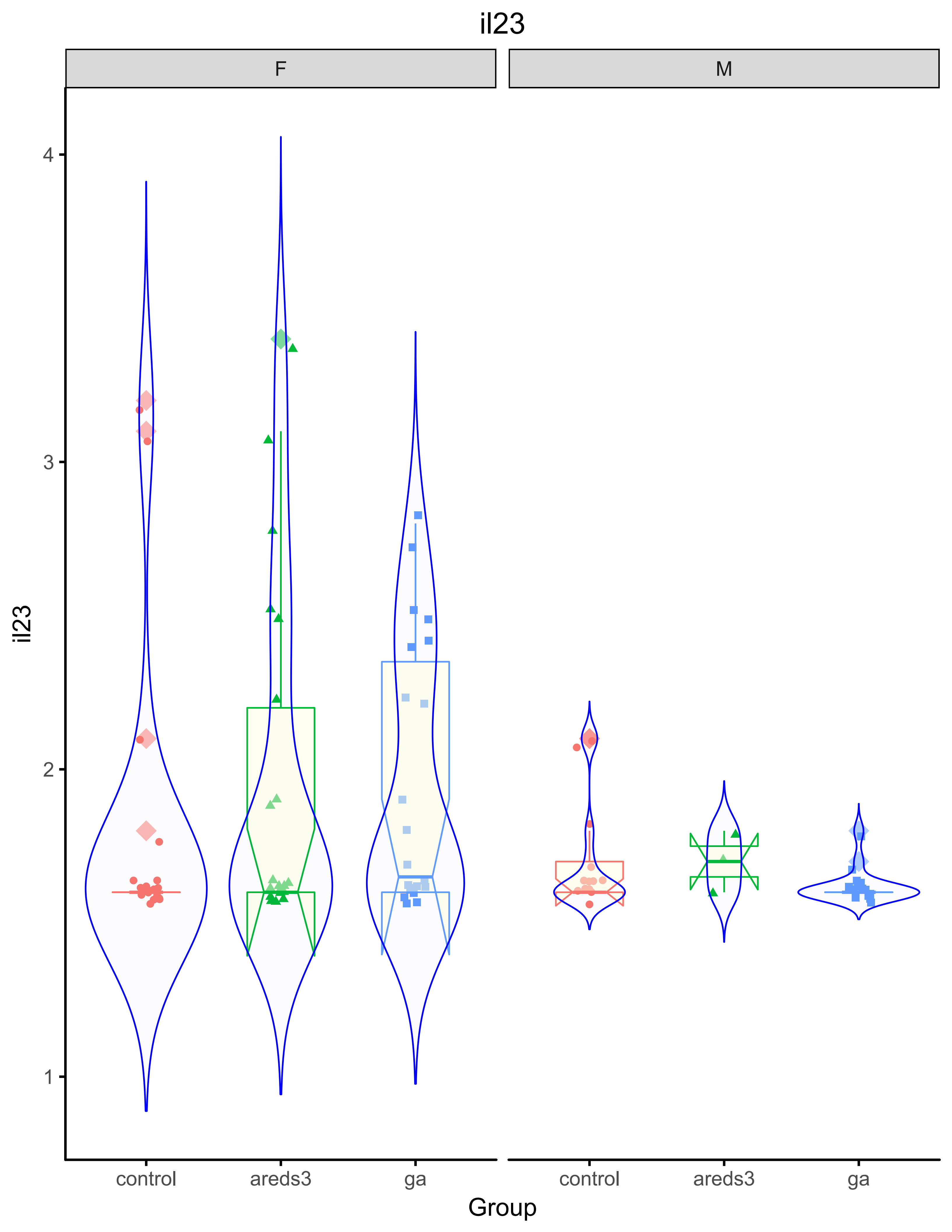
il23
F
M
4
3
2
1
il23
control
areds3
ga
control
areds3
ga
Group

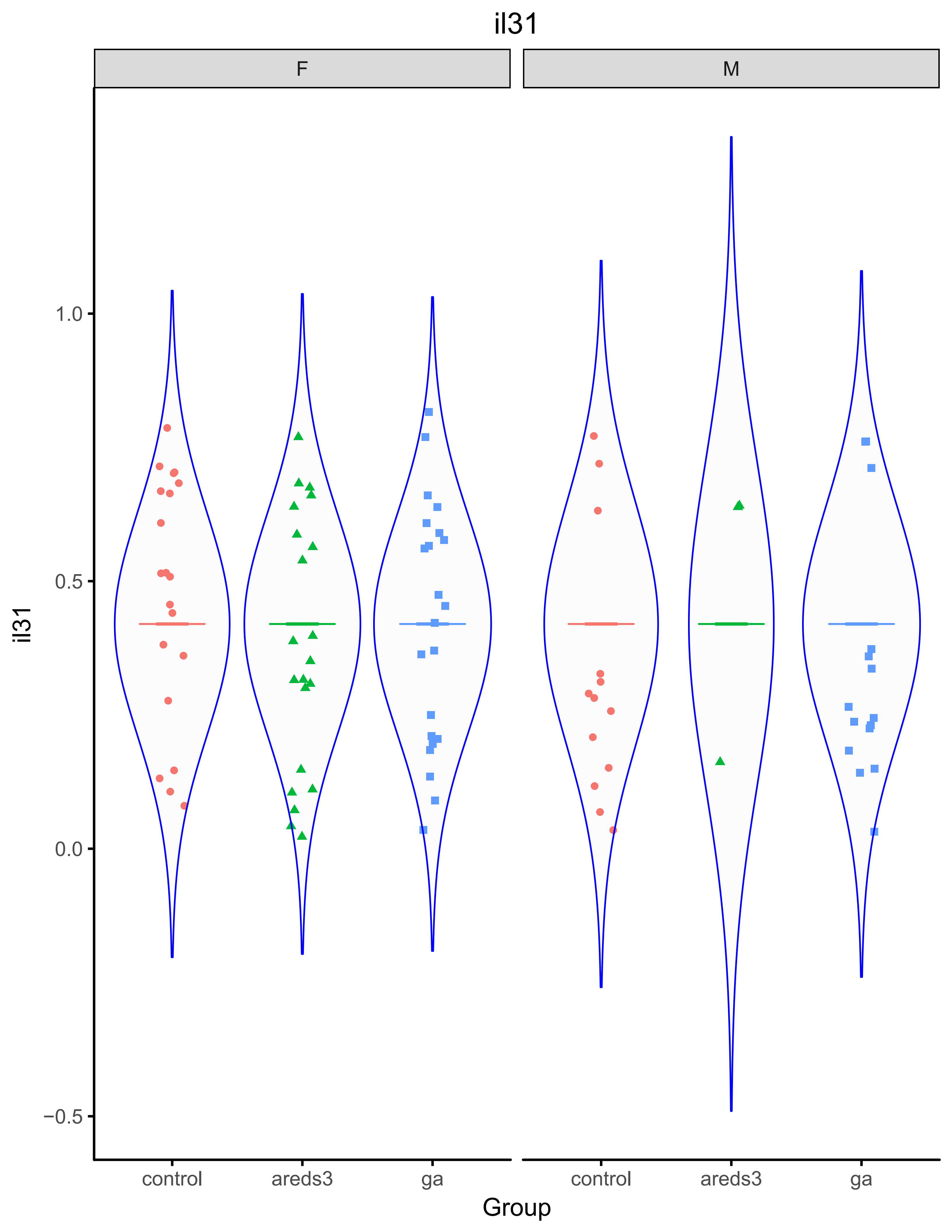

il31
F
M
1.0
0.5
0.0
−0.5
il31
control
areds3
ga
control
areds3
ga
Group

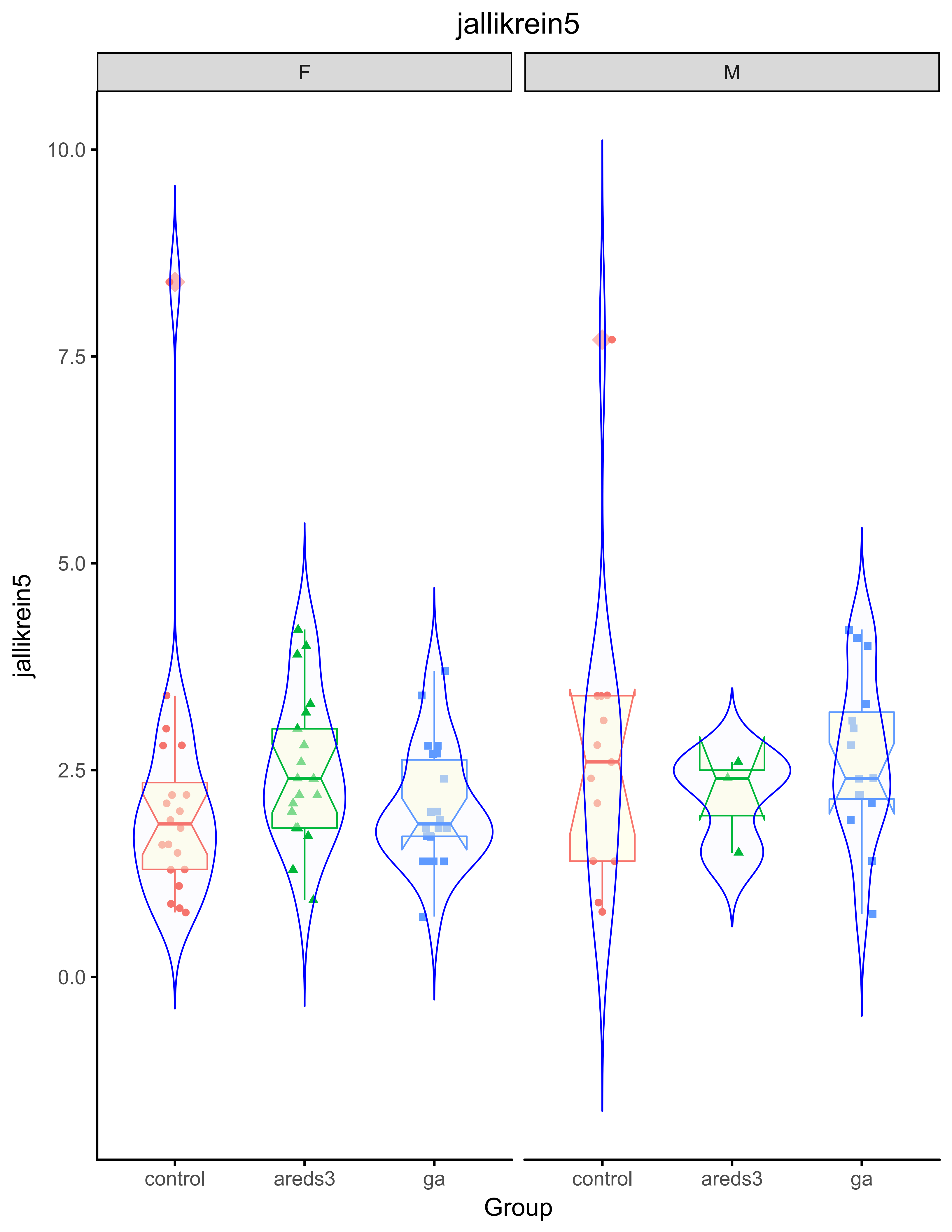

jallikrein5
F
M
10.0
7.5
5.0
2.5
0.0
jallikrein5
control
areds3
ga
control
areds3
ga
Group

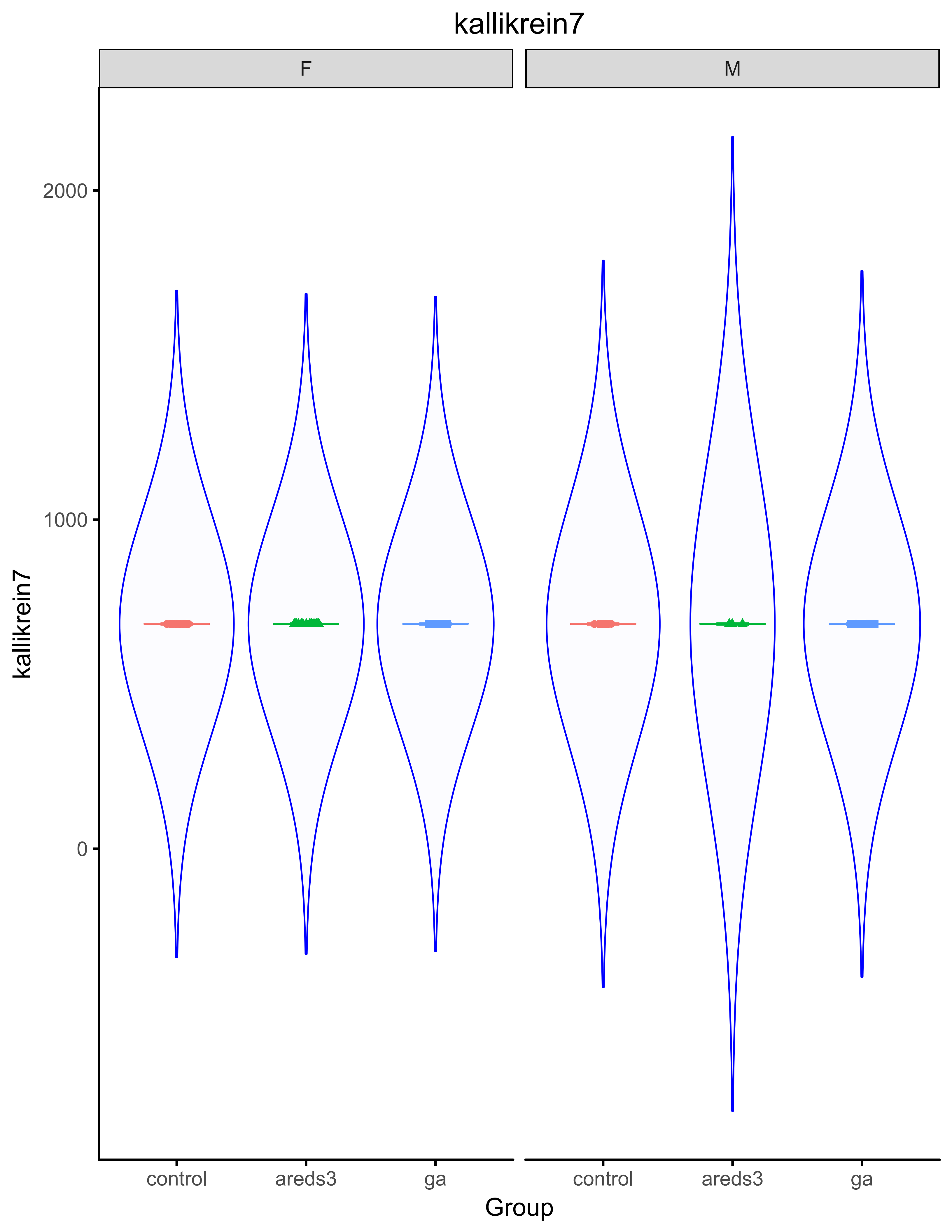
kallikrein7
F
M
2000
1000
0
kallikrein7
control
areds3
ga
control
areds3
ga
Group

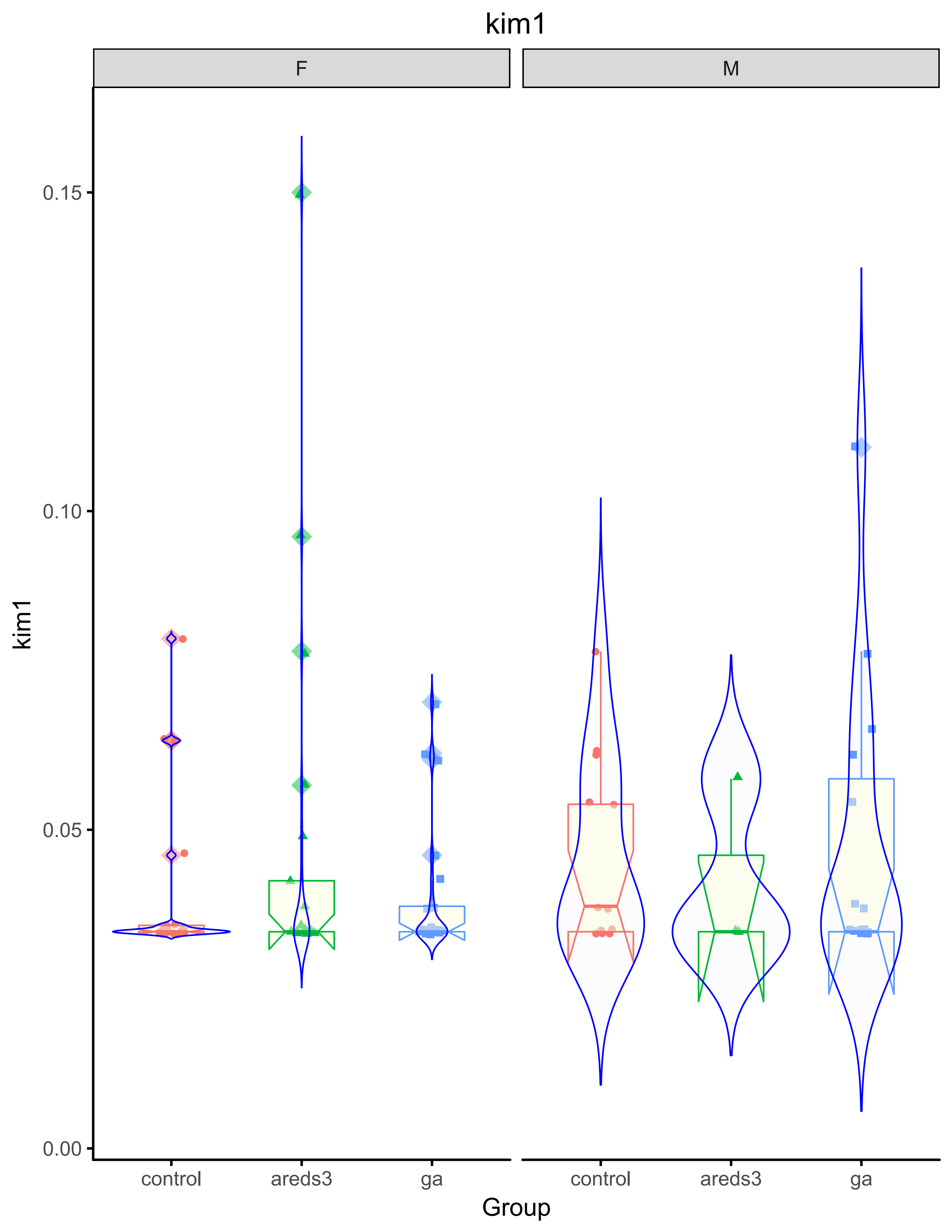
kim1
F
M
0.15
0.10
0.05
0.00
kim1
control
areds3
ga
control
areds3
ga
Group

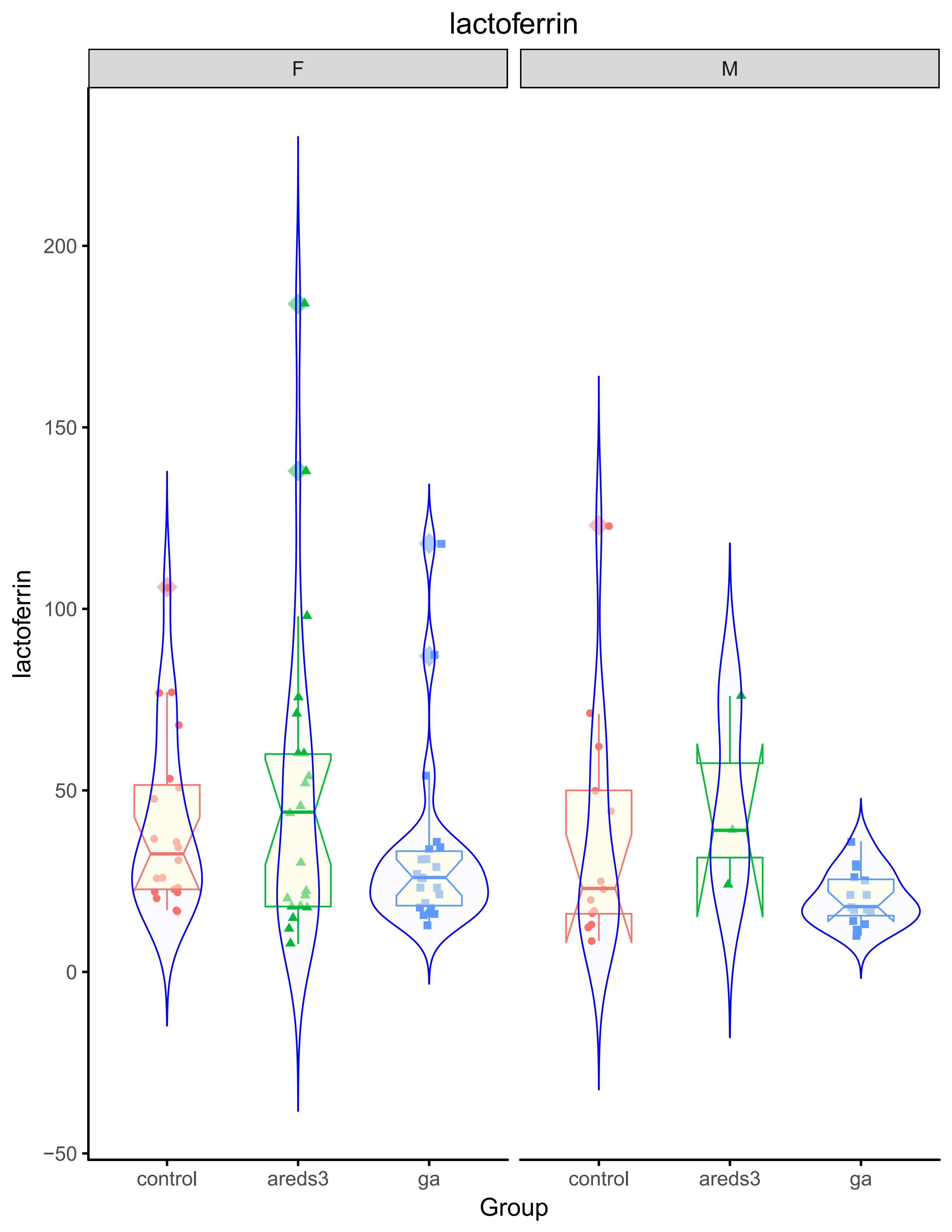
lactoferrin
F
M
200
150
100
50
0
−50
lactoferrin
control
areds3
ga
control
areds3
ga
Group

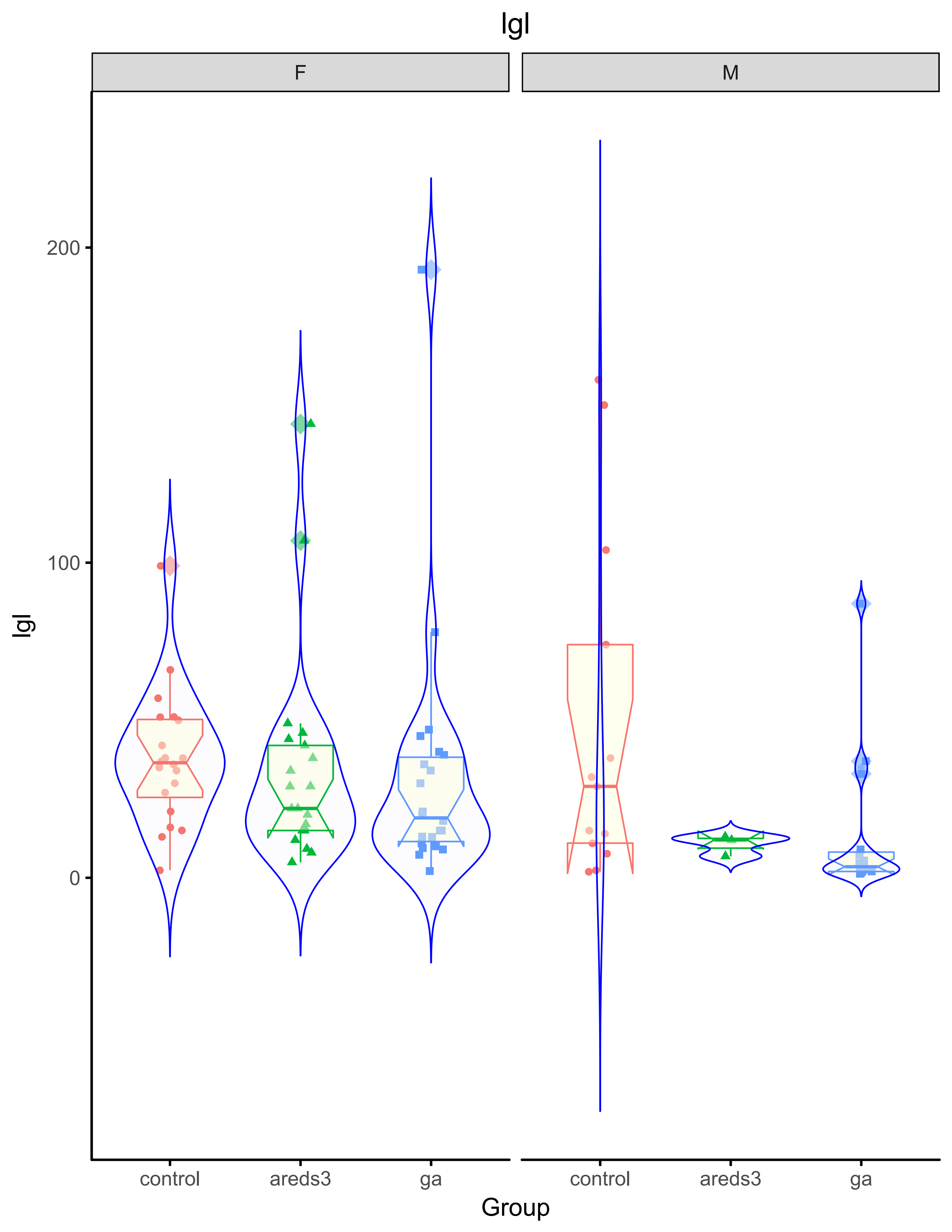

lgl
F
M
200
100
0
lgl
control
areds3
ga
control
areds3
ga
Group

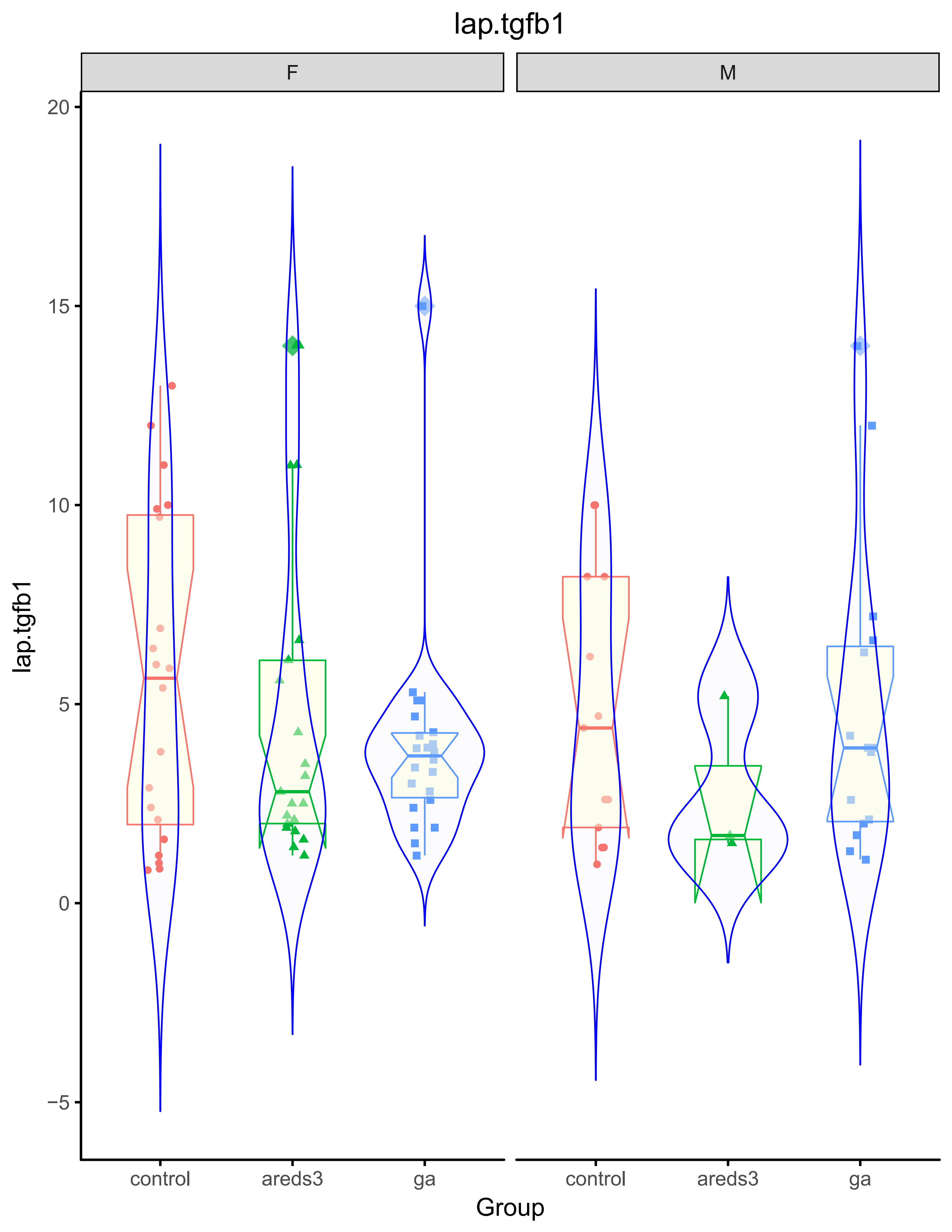
lap.tgfb1
F
M
20
15
10
5
0
−5
lap.tgfb1
control
areds3
ga
control
areds3
ga
Group

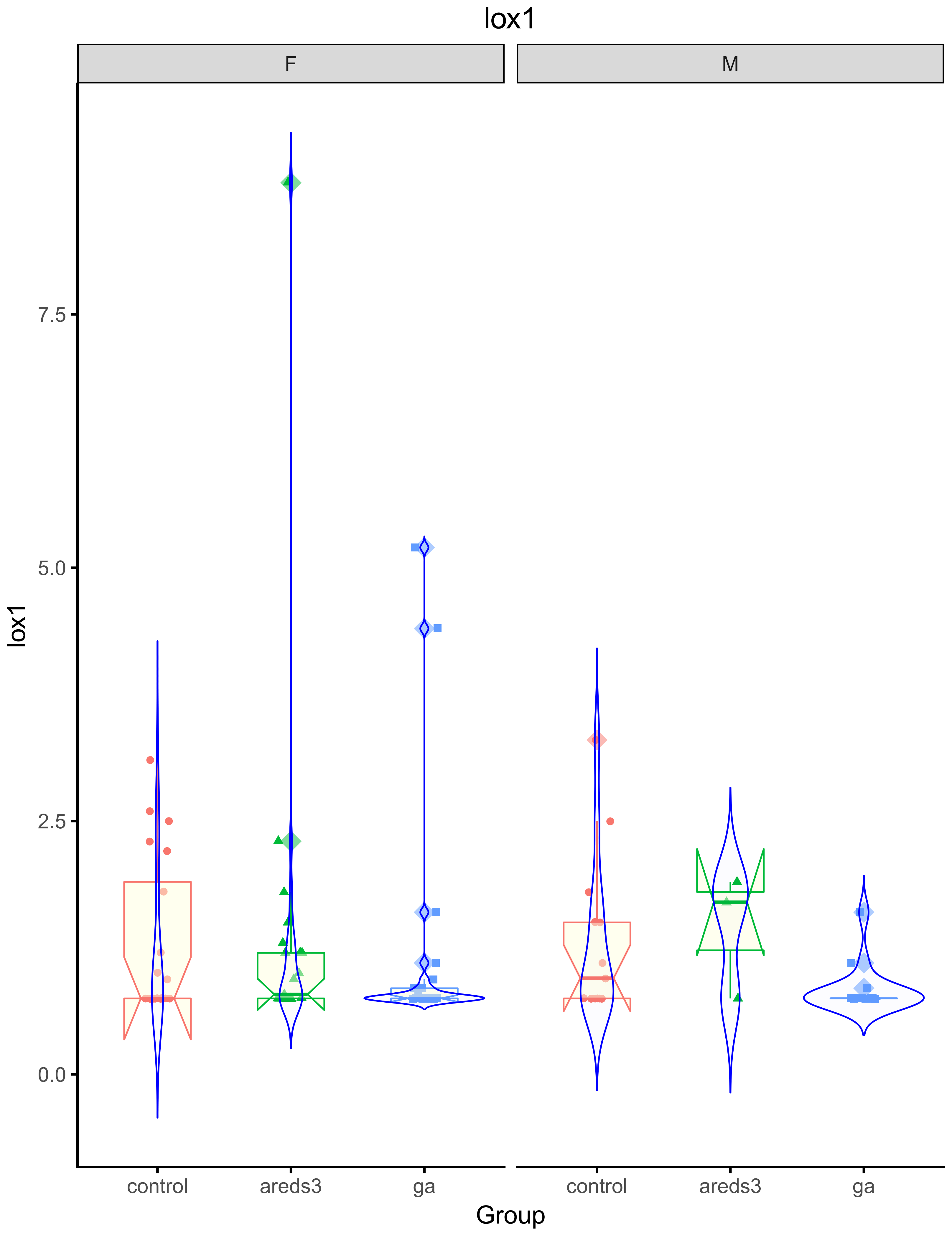
lox1
F
M
7.5
5.0
2.5
0.0
lox1
control
areds3
ga
control
areds3
ga
Group

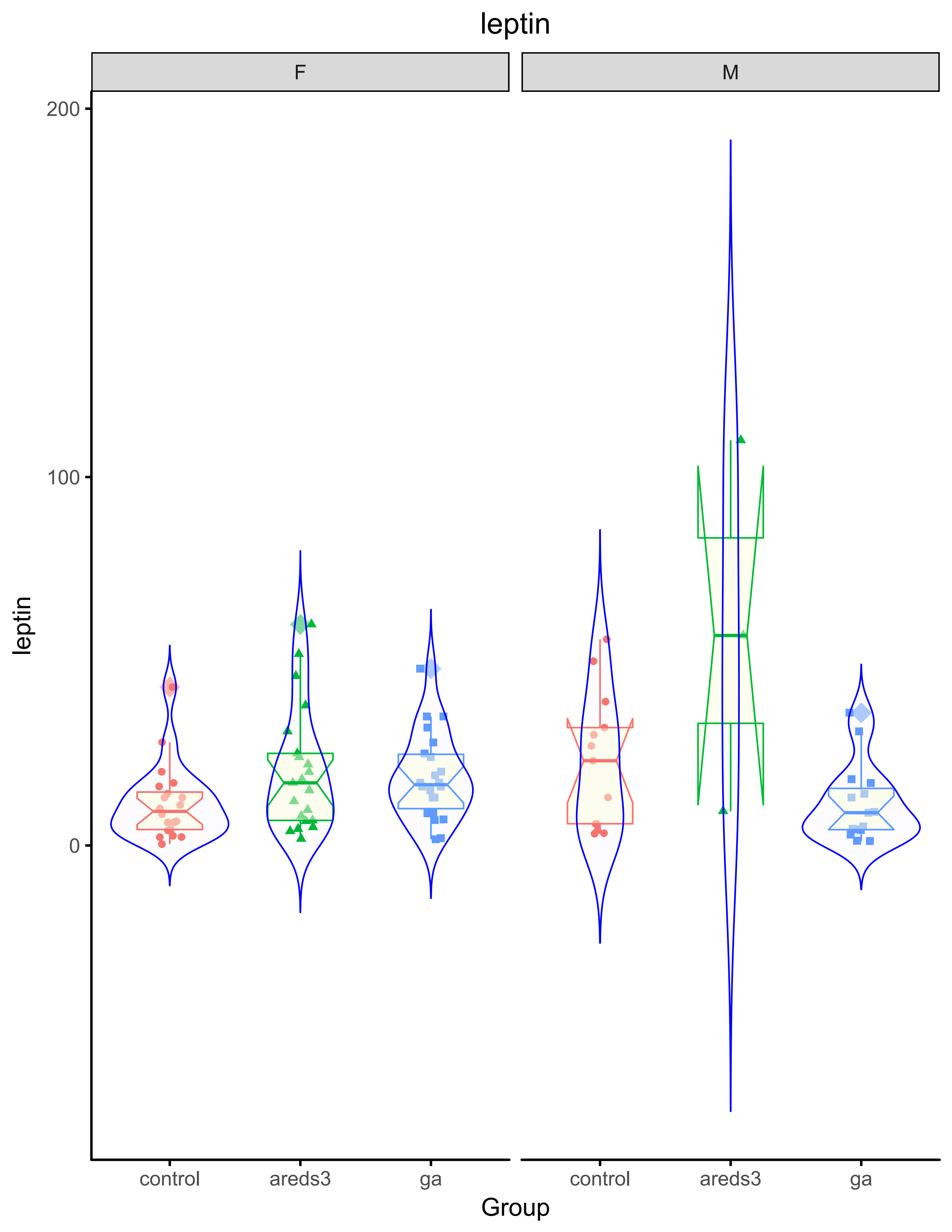
leptin
F
M
200
100
0
leptin
control
areds3
ga
control
areds3
ga
Group

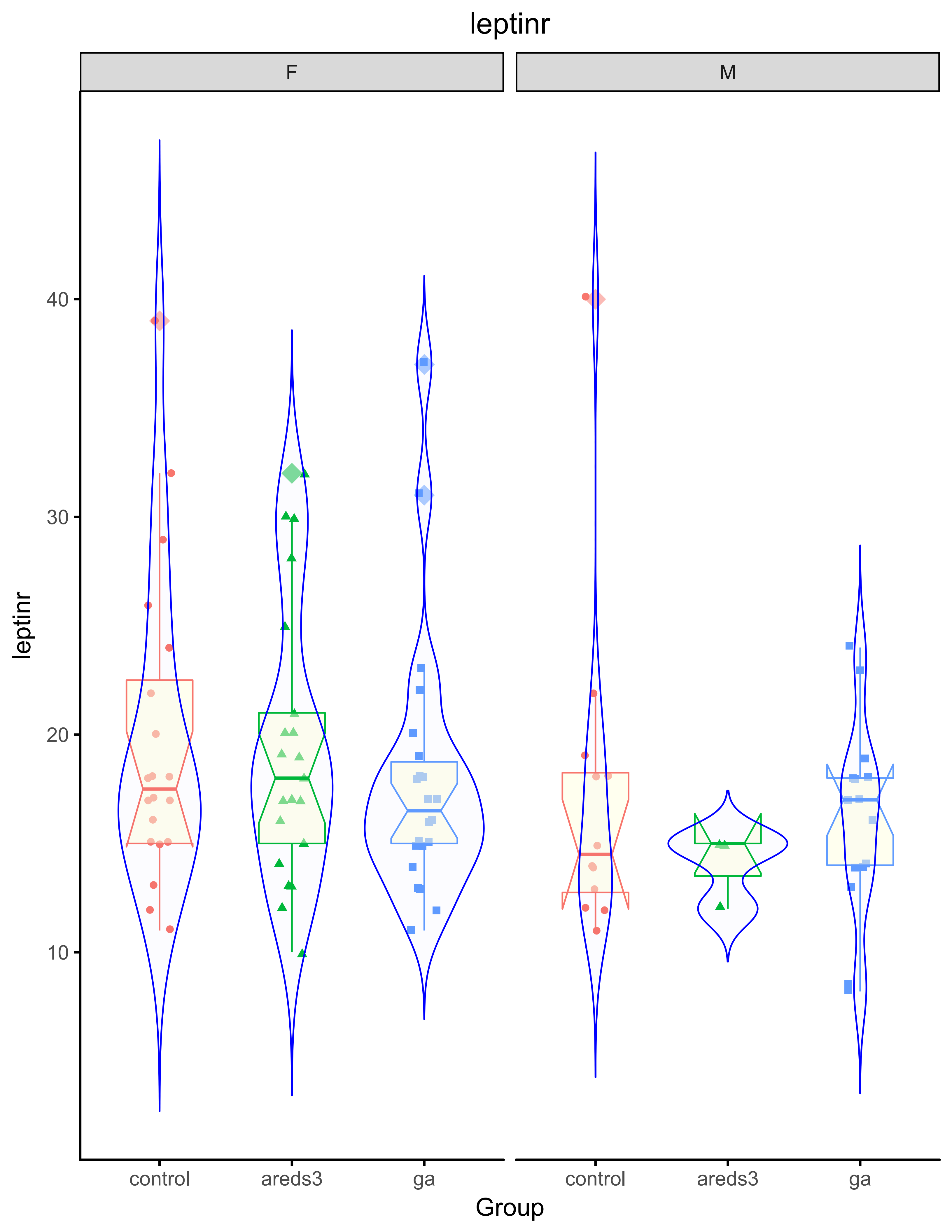
leptinr
F
M
leptinr
40
30
20
10
control
areds3
ga
control
areds3
ga
Group

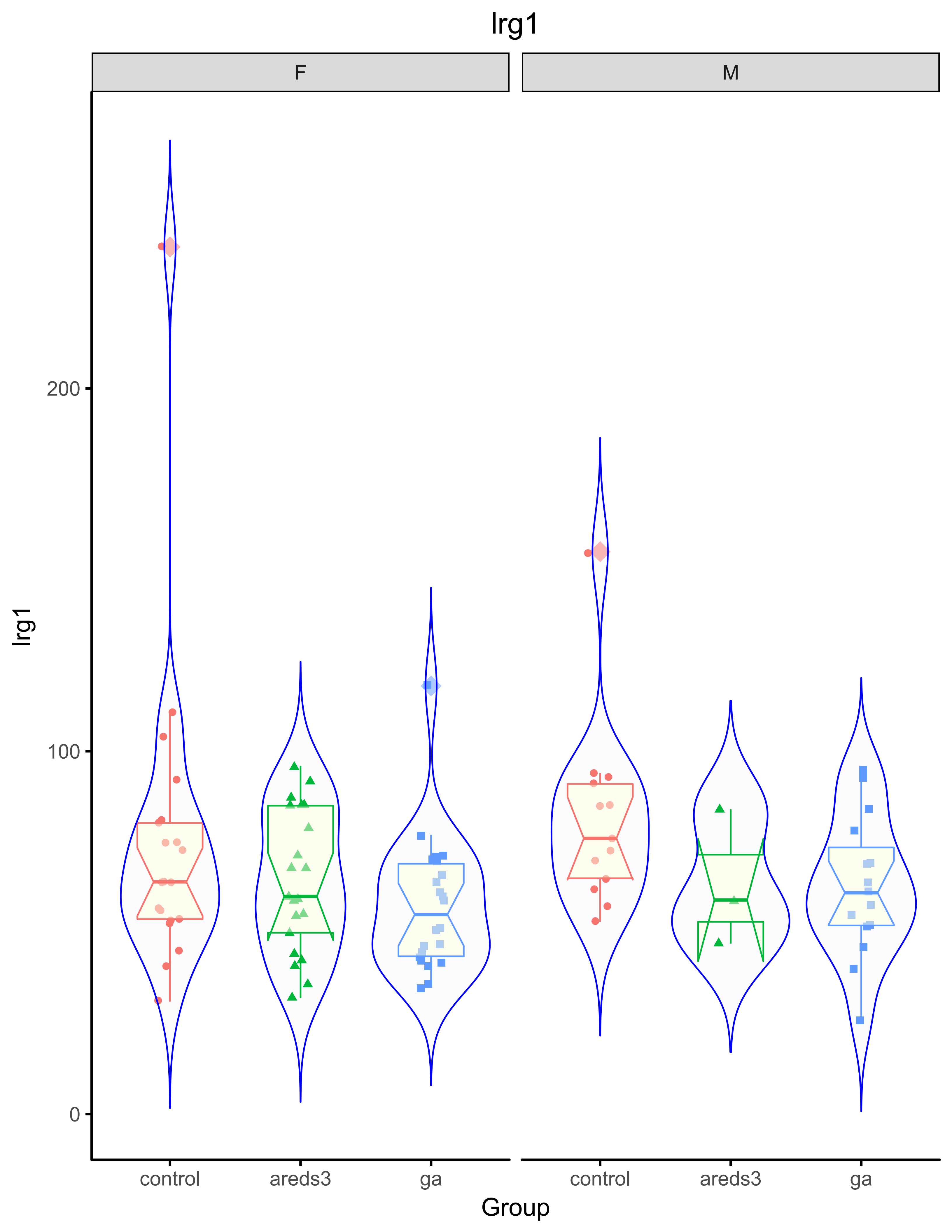

Irg1
F
M
200
100
0
Irg1
control
areds3
ga
control
areds3
ga
Group

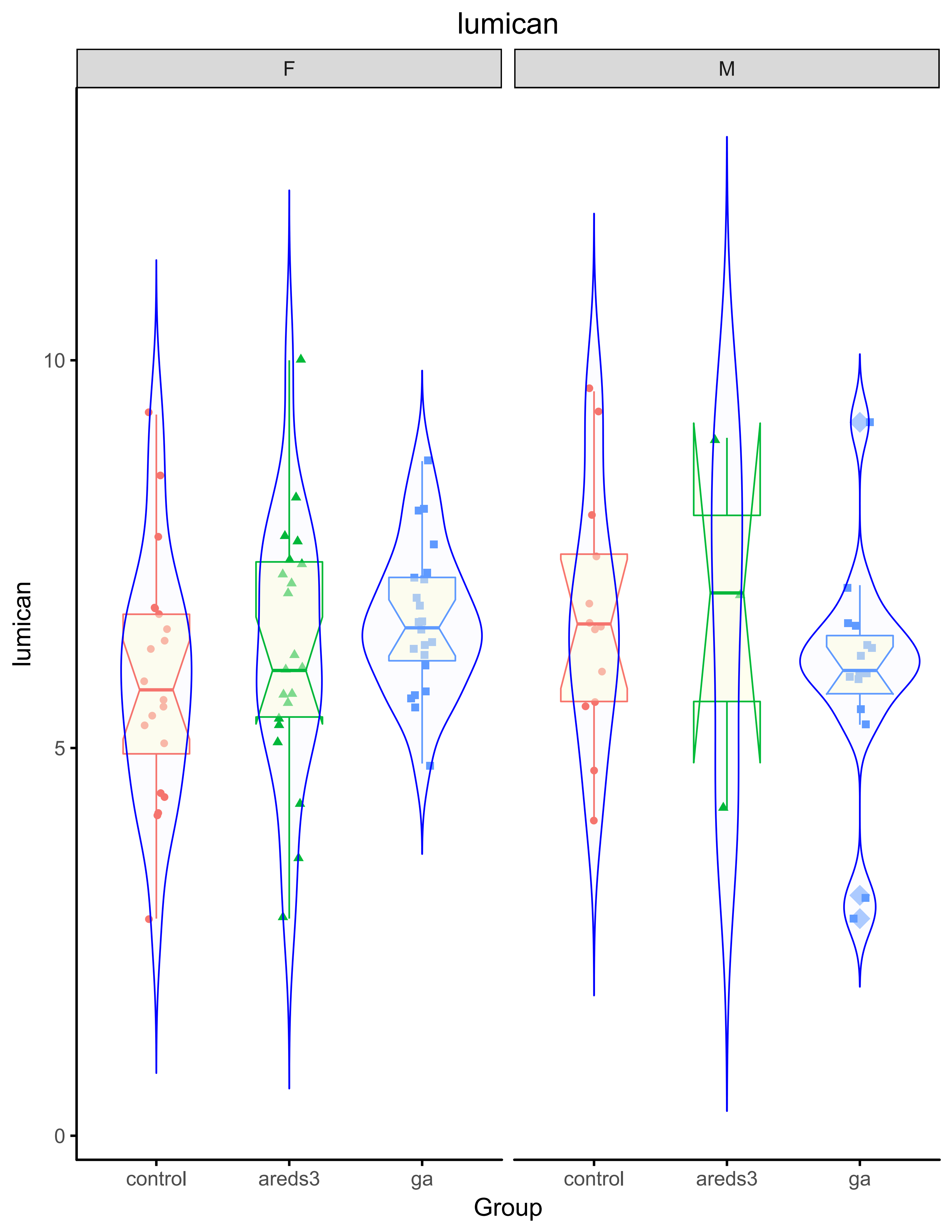

lumican
F
M
lumican
10
5
0
control
areds3
ga
control
areds3
ga
Group

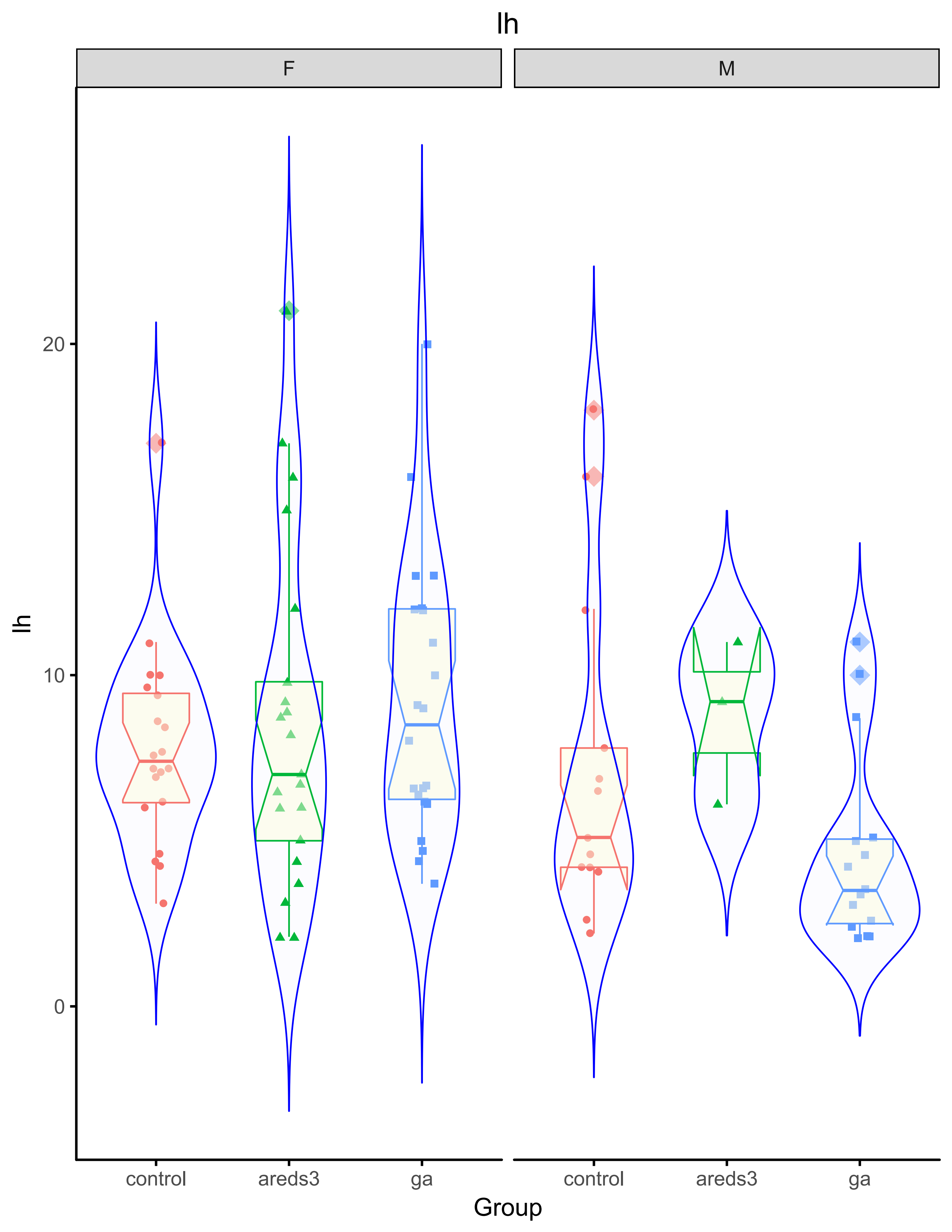
lh
F
M
20
10
0
lh
control
areds3
ga
control
areds3
ga
Group

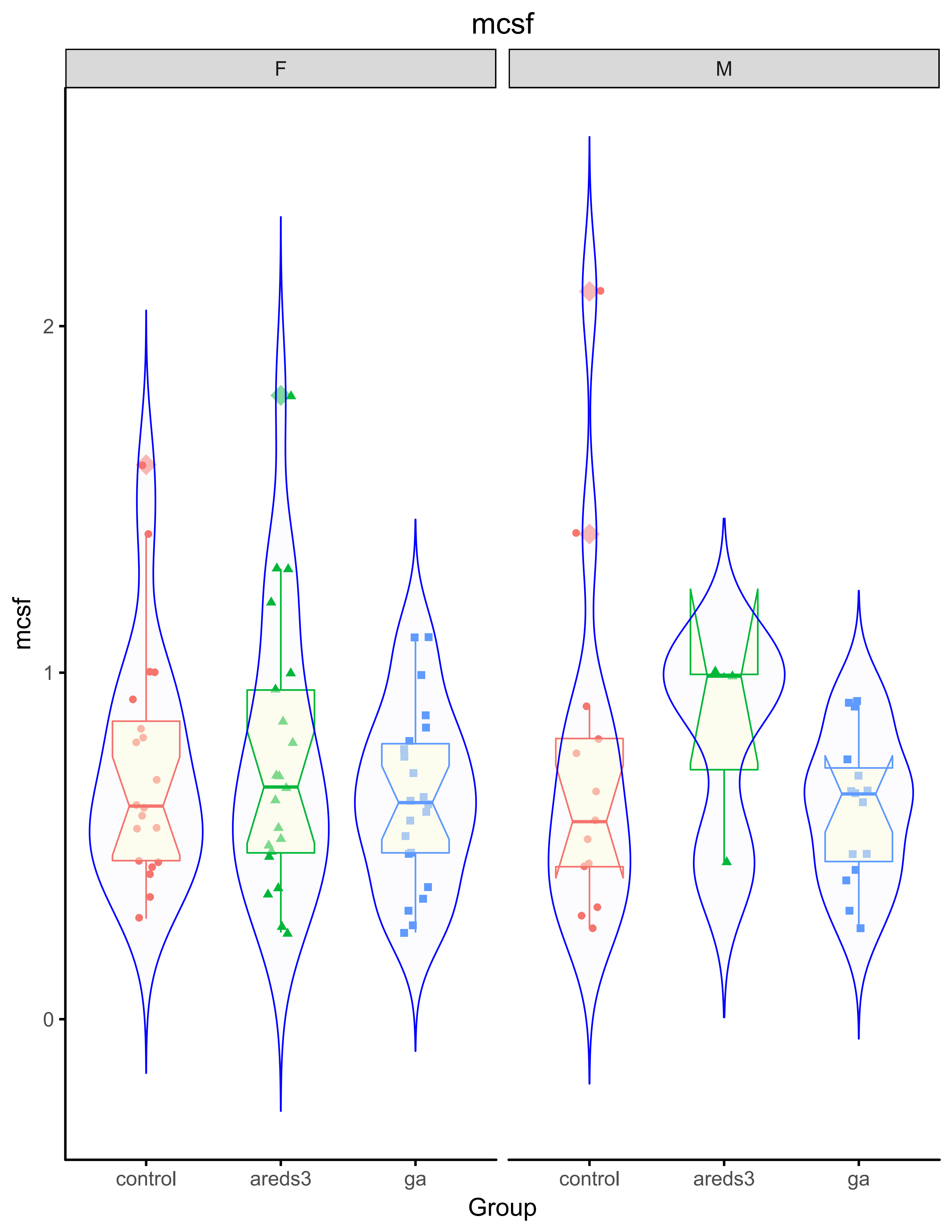

mcsf
F
M
mcsf
2
1
0
control
areds3
ga
control
areds3
ga
Group

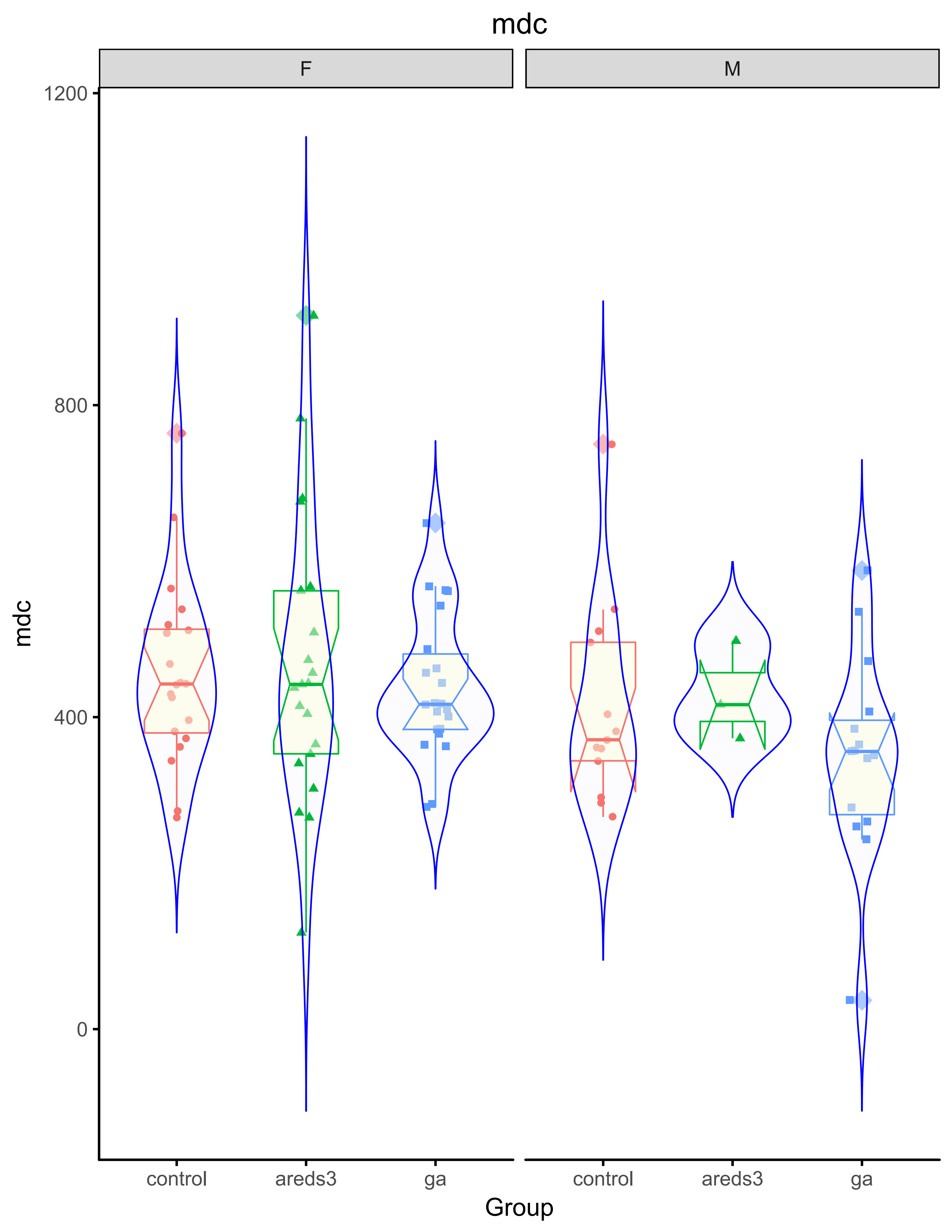
mdc
F
M
1200
800
400
0
mdc
control
areds3
ga
control
areds3
ga
Group

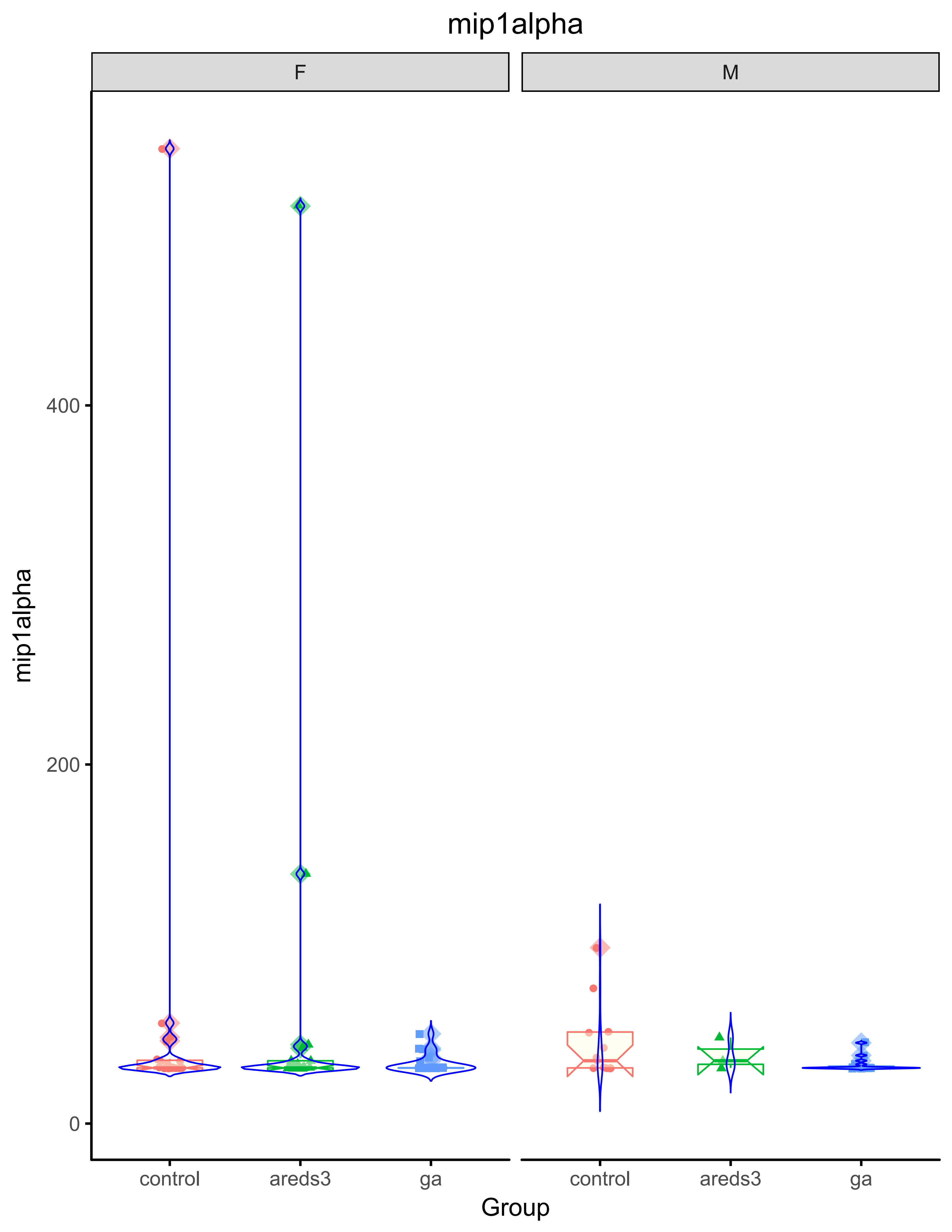

mip1alpha
F
M
400
200
0
mip1alpha
control
areds3
ga
control
areds3
ga
Group

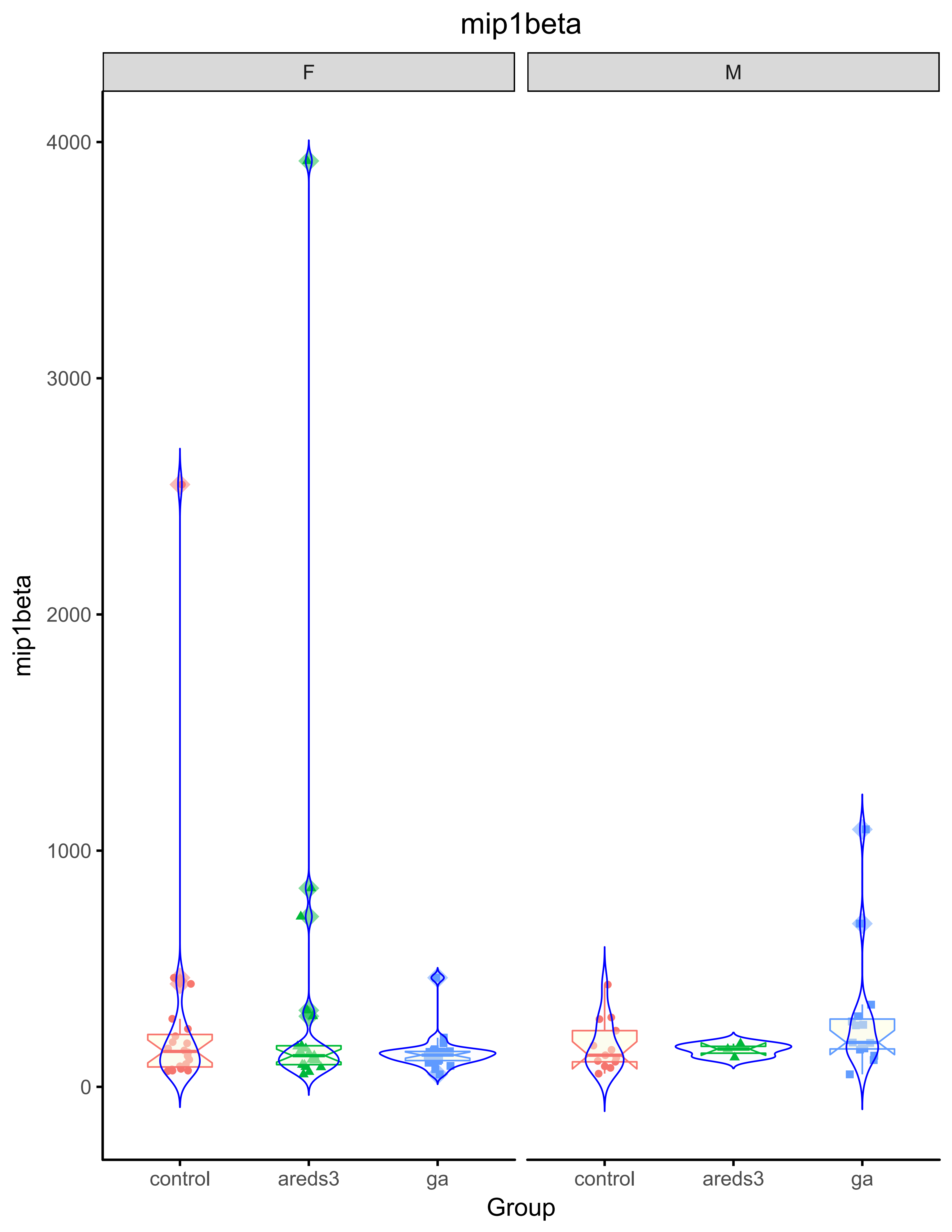

mip1beta
F
M
4000
3000
2000
1000
0
mip1beta
control
areds3
ga
control
areds3
ga
Group

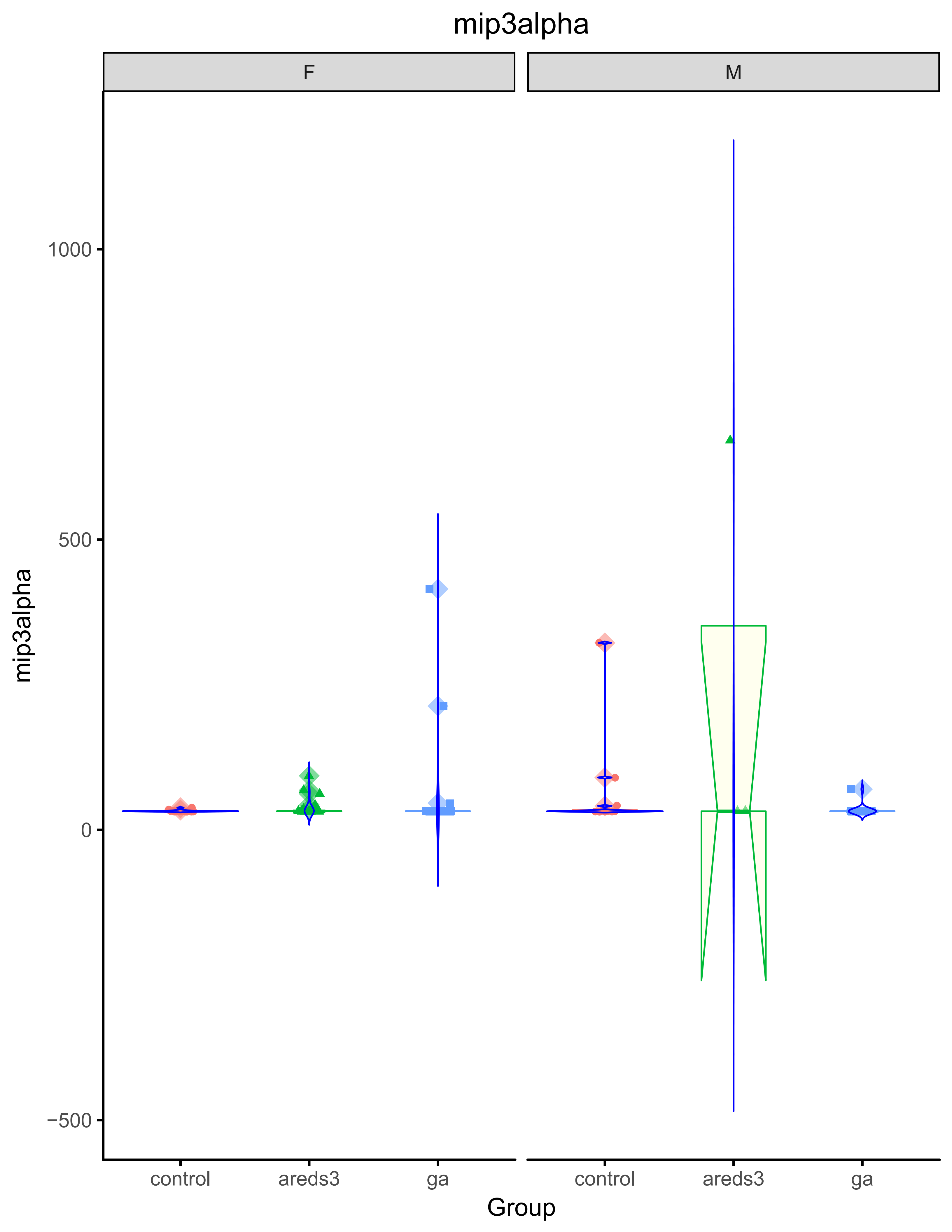
mip3alpha
F
M
1000
500
0
−500
mip3alpha
control
areds3
ga
control
areds3
ga
Group

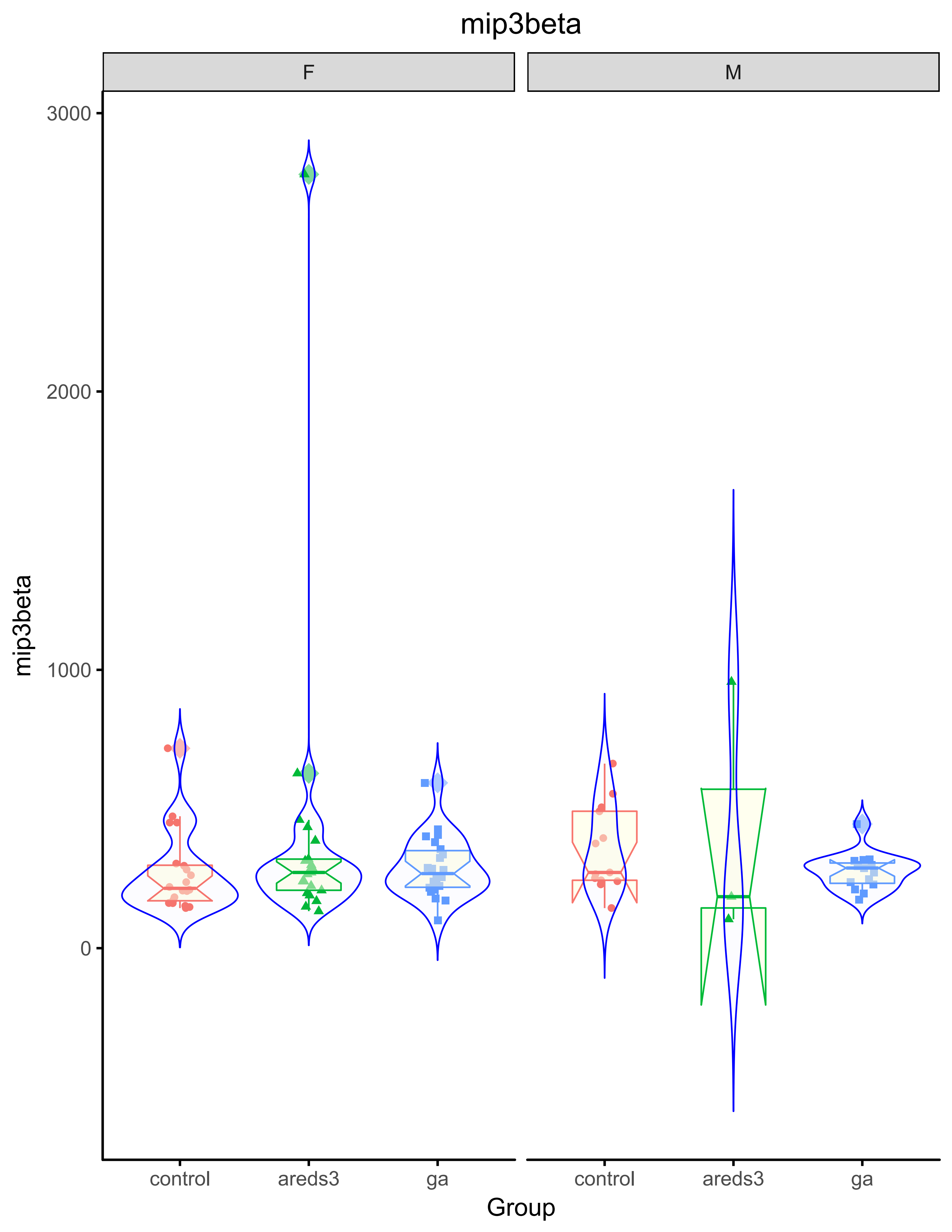
mip3beta
F
M
3000
2000
1000
0
mip3beta
control
areds3
ga
control
areds3
ga
Group

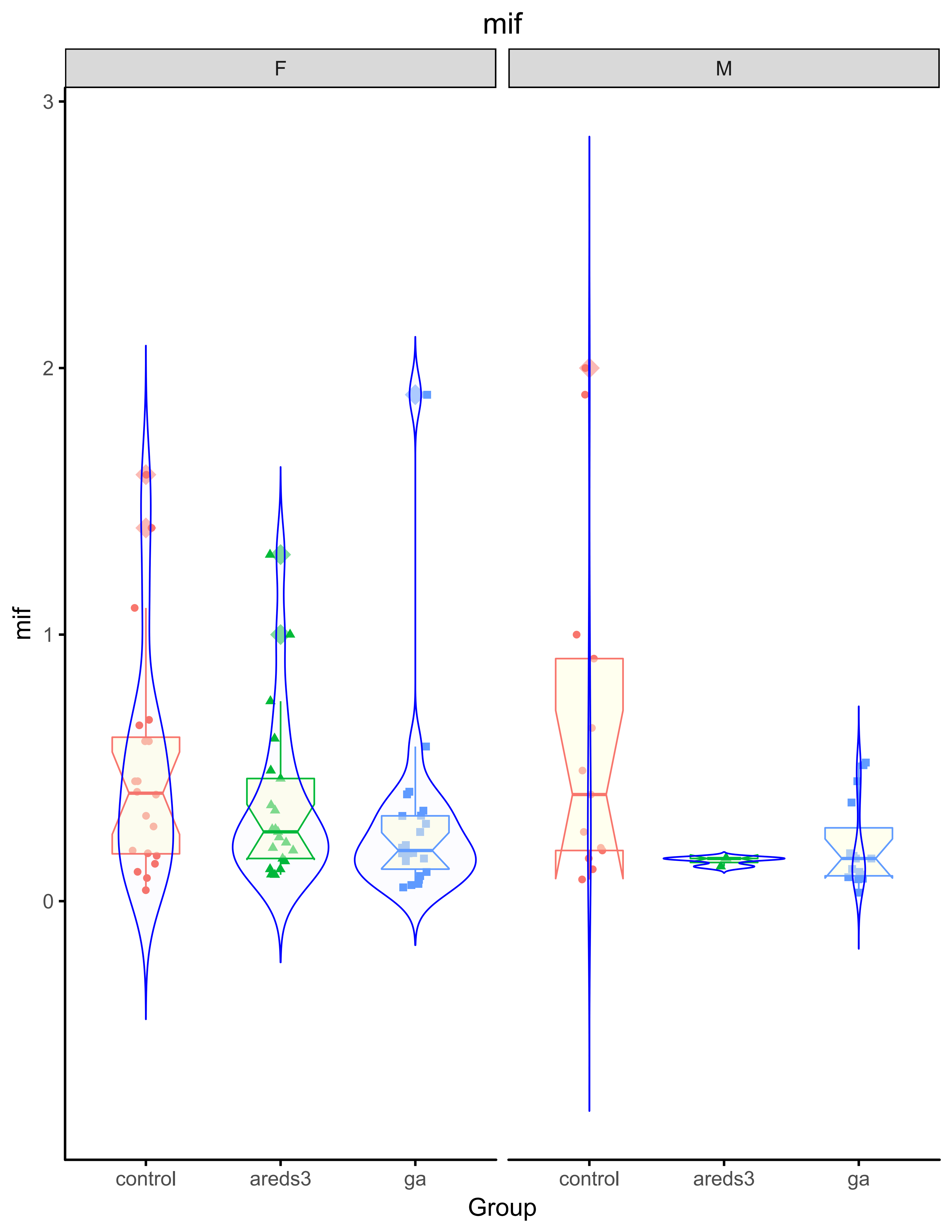
mif
F
M
3
2
1
0
mif
control
areds3
ga
control
areds3
ga
Group

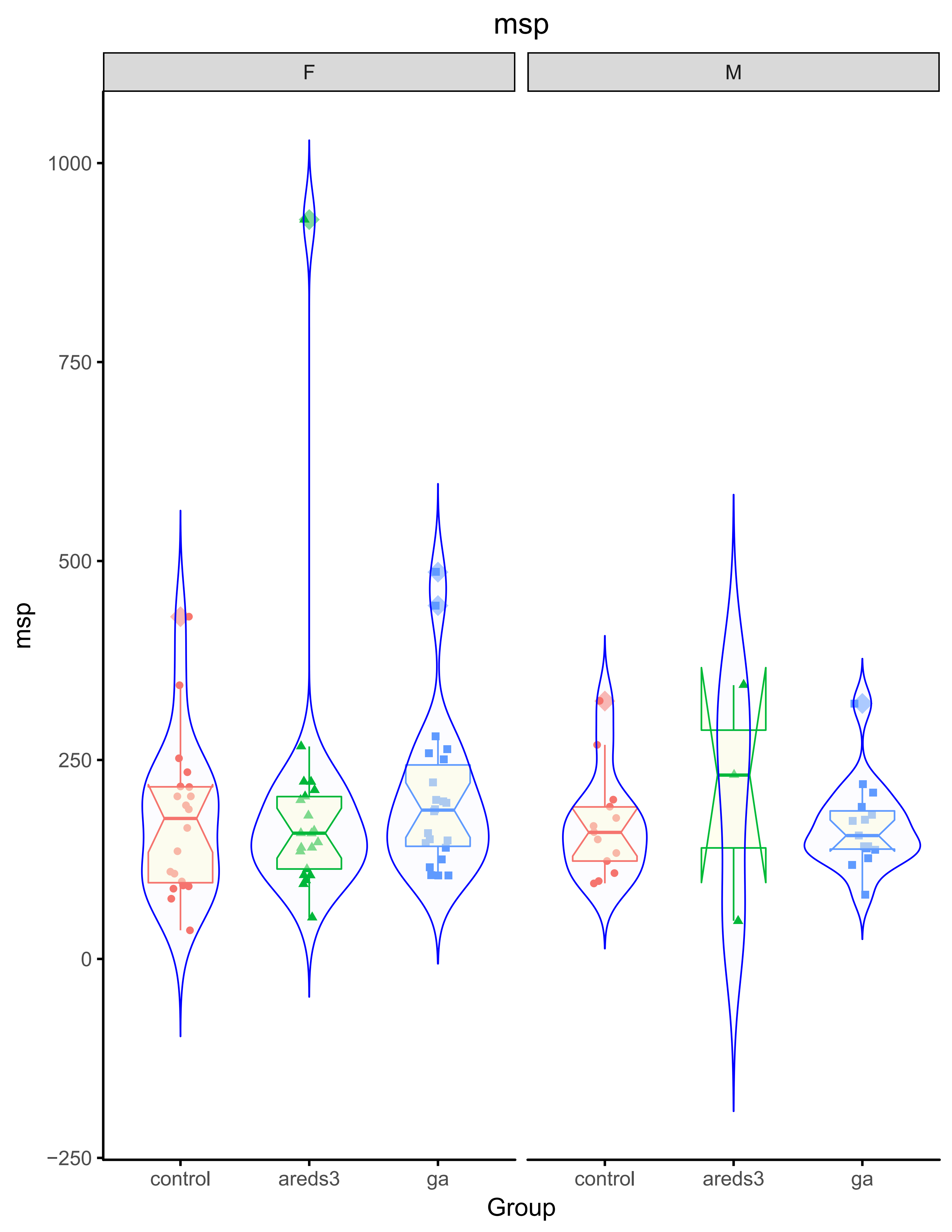

msp
F
M
1000
750
500
250
0
−250
msp
control
areds3
ga
control
areds3
ga
Group

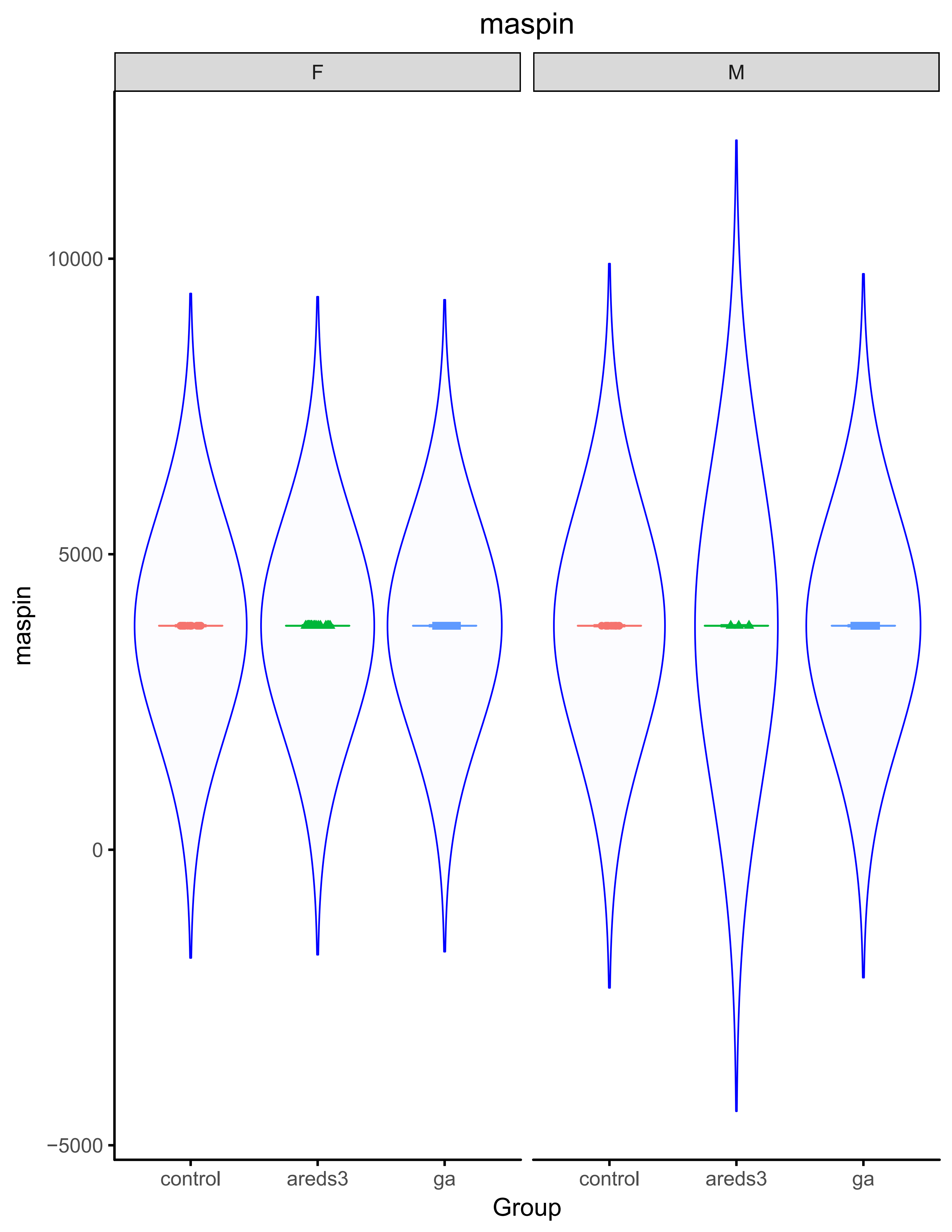
maspin
F
M
10000
5000
0
−5000
maspin
control
areds3
ga
control
areds3
ga
Group

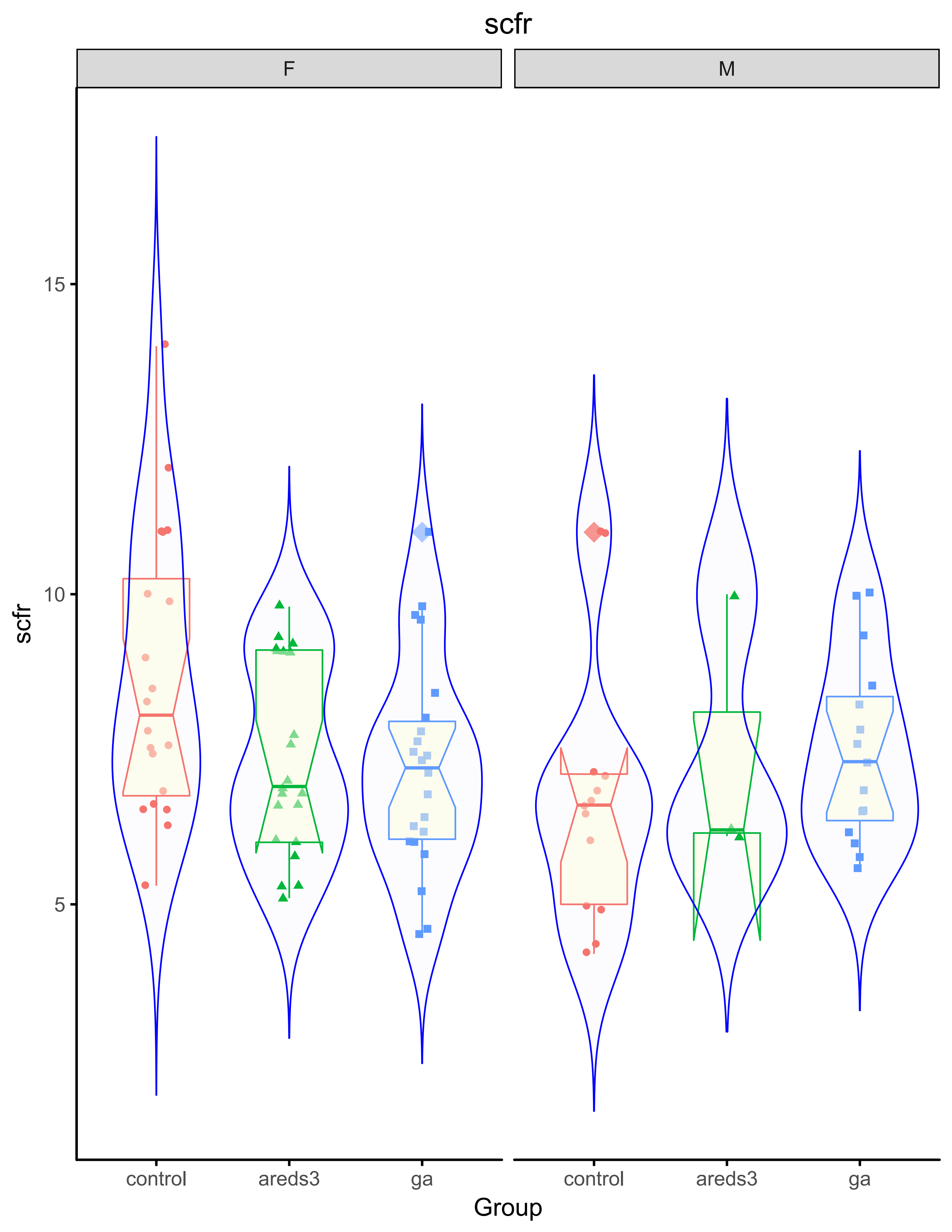
scfr
F
M
15
10
5
scfr
control
areds3
ga
control
areds3
ga
Group

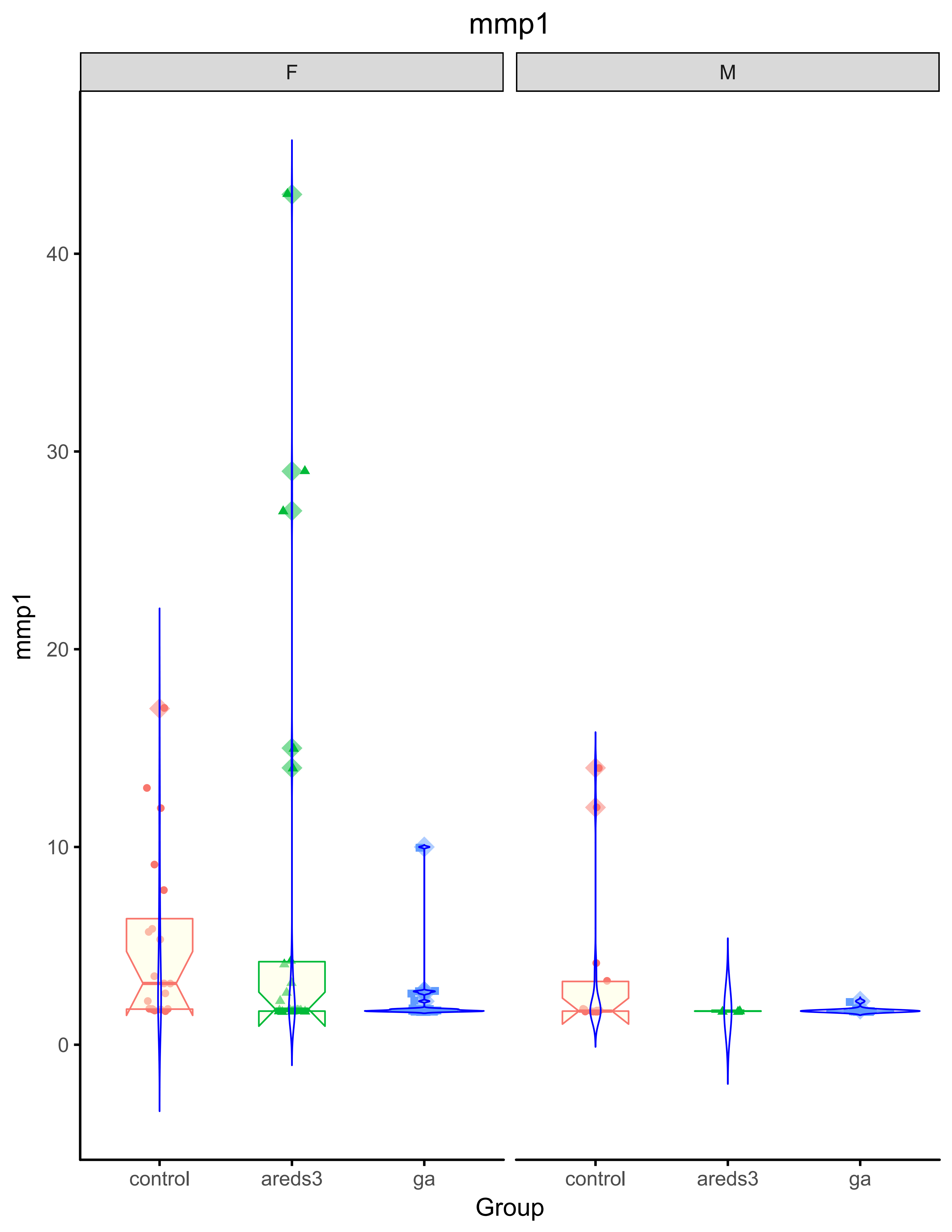

mmp1
F
M
mmp1
40
30
20
10
0
control
areds3
ga
control
areds3
ga
Group

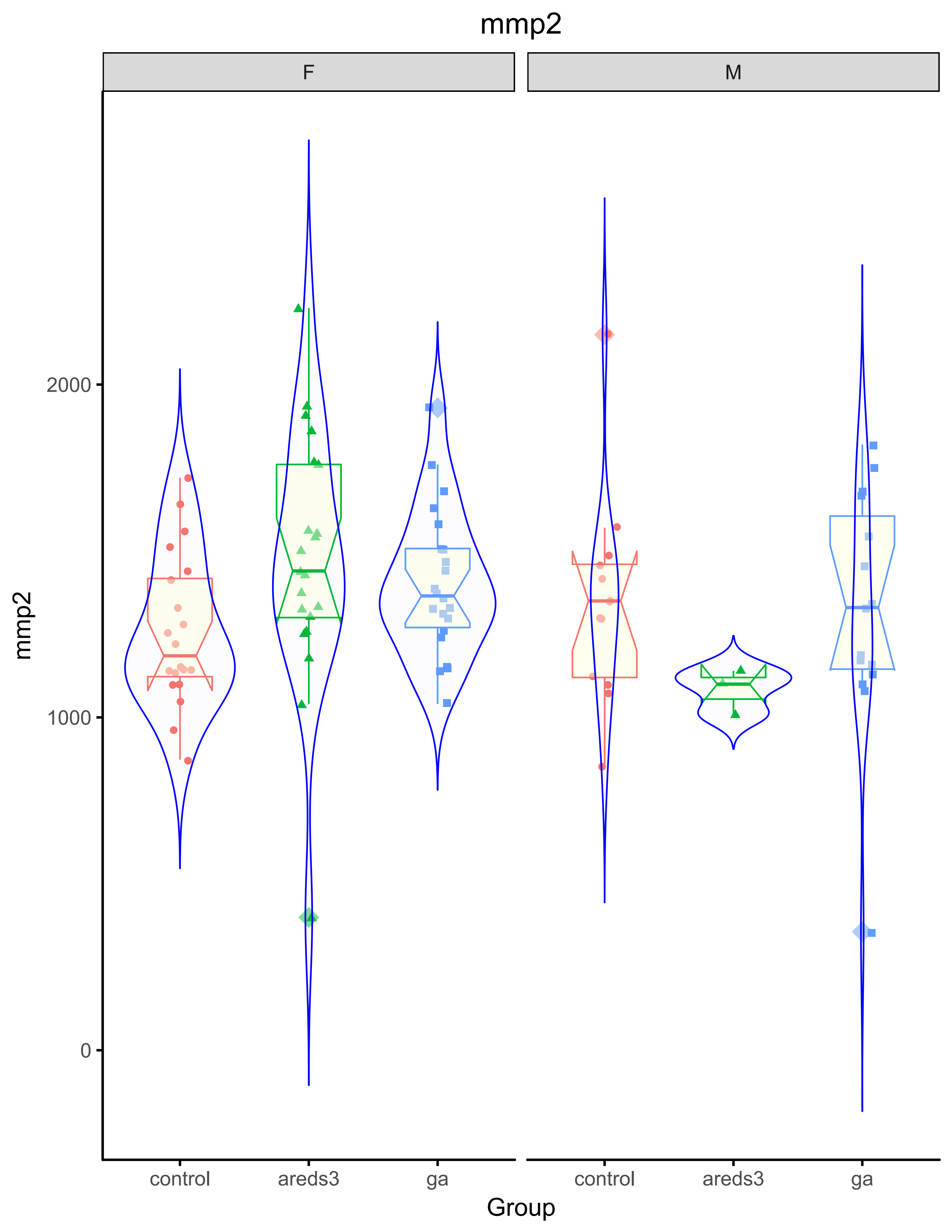

mmp2
F
M
2000
1000
0
mmp2
control
areds3
ga
control
areds3
ga
Group

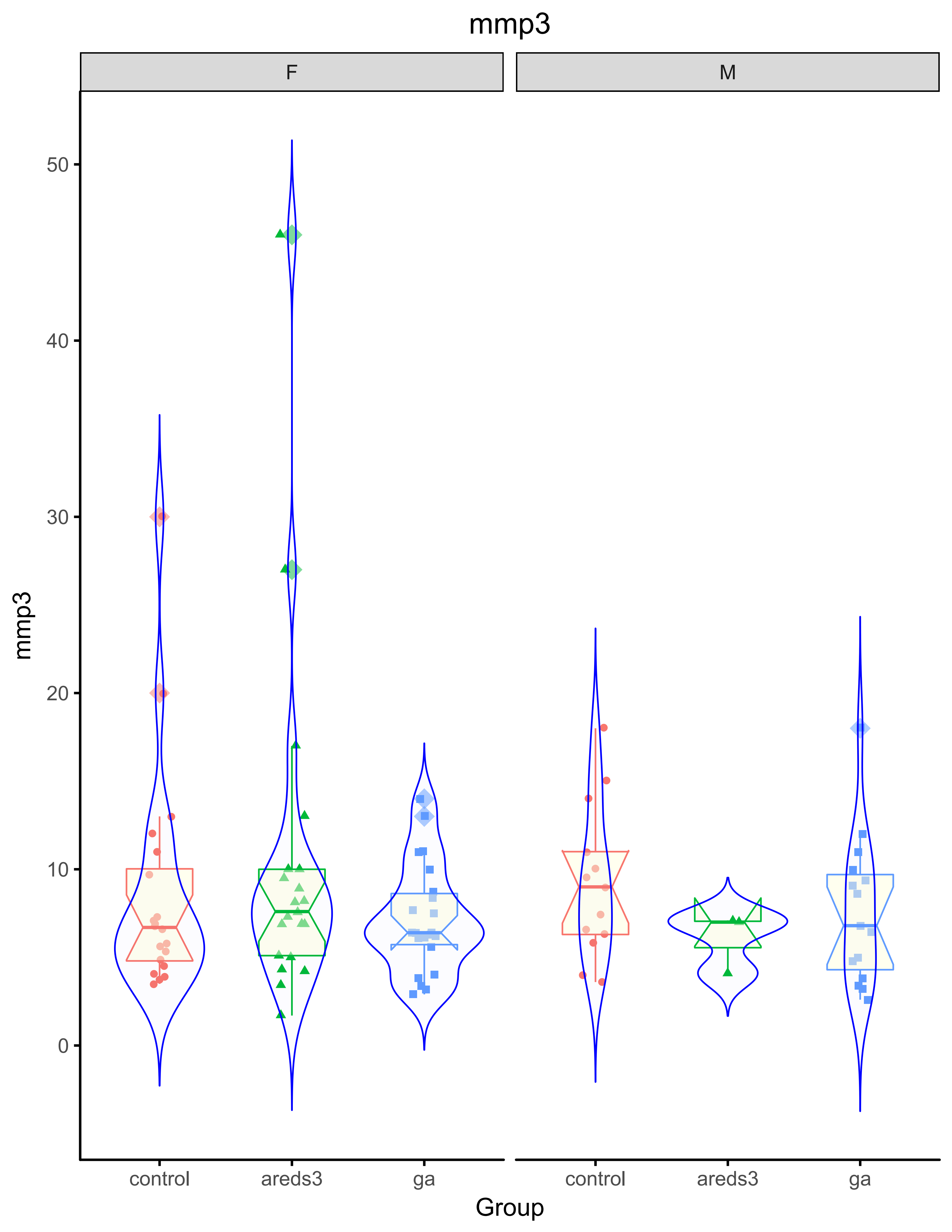

mmp3
F
M
mmp3
0
10
20
30
40
50
control
areds3
ga
control
areds3
ga
Group

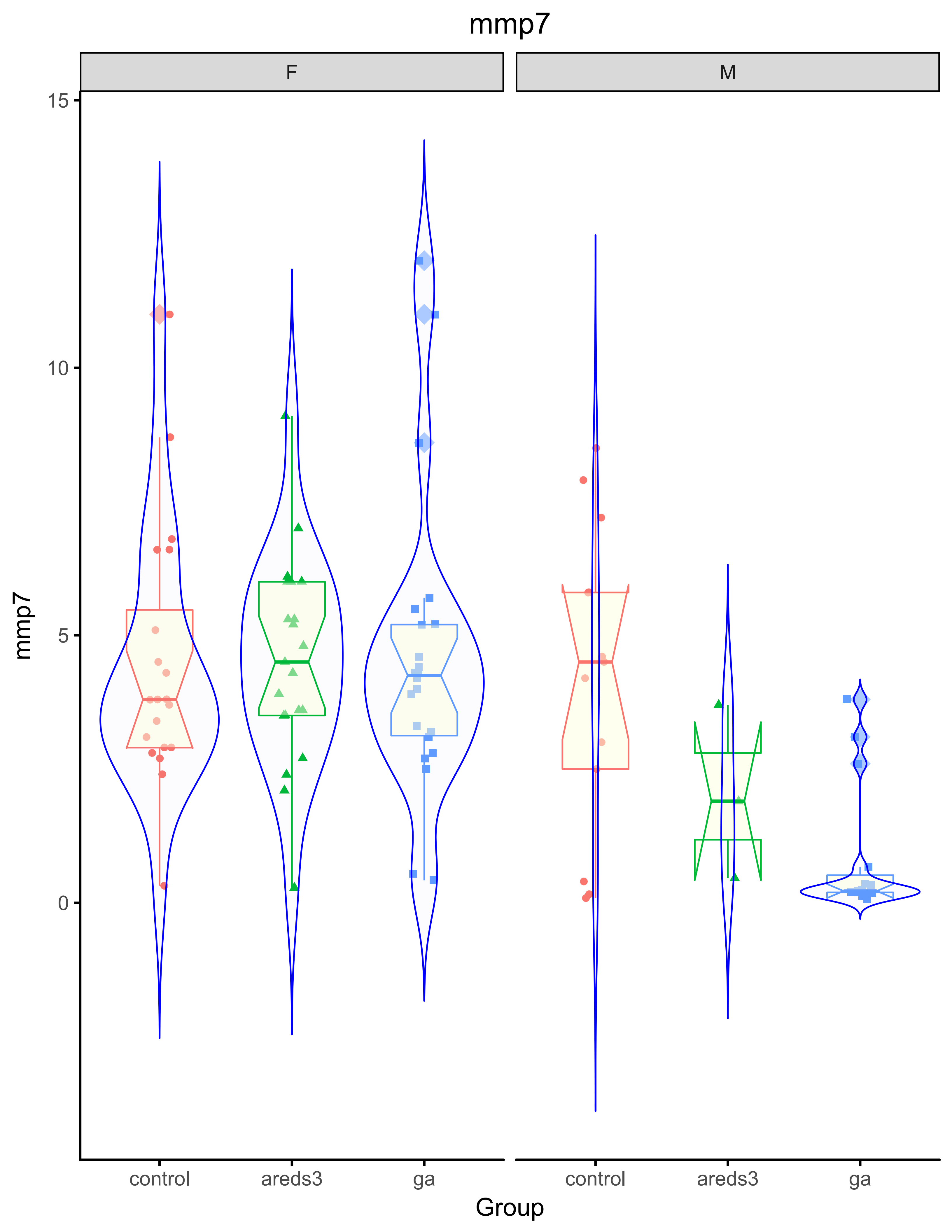
mmp7
F
M
15
10
5
0
mmp7
control
areds3
ga
control
areds3
ga
Group

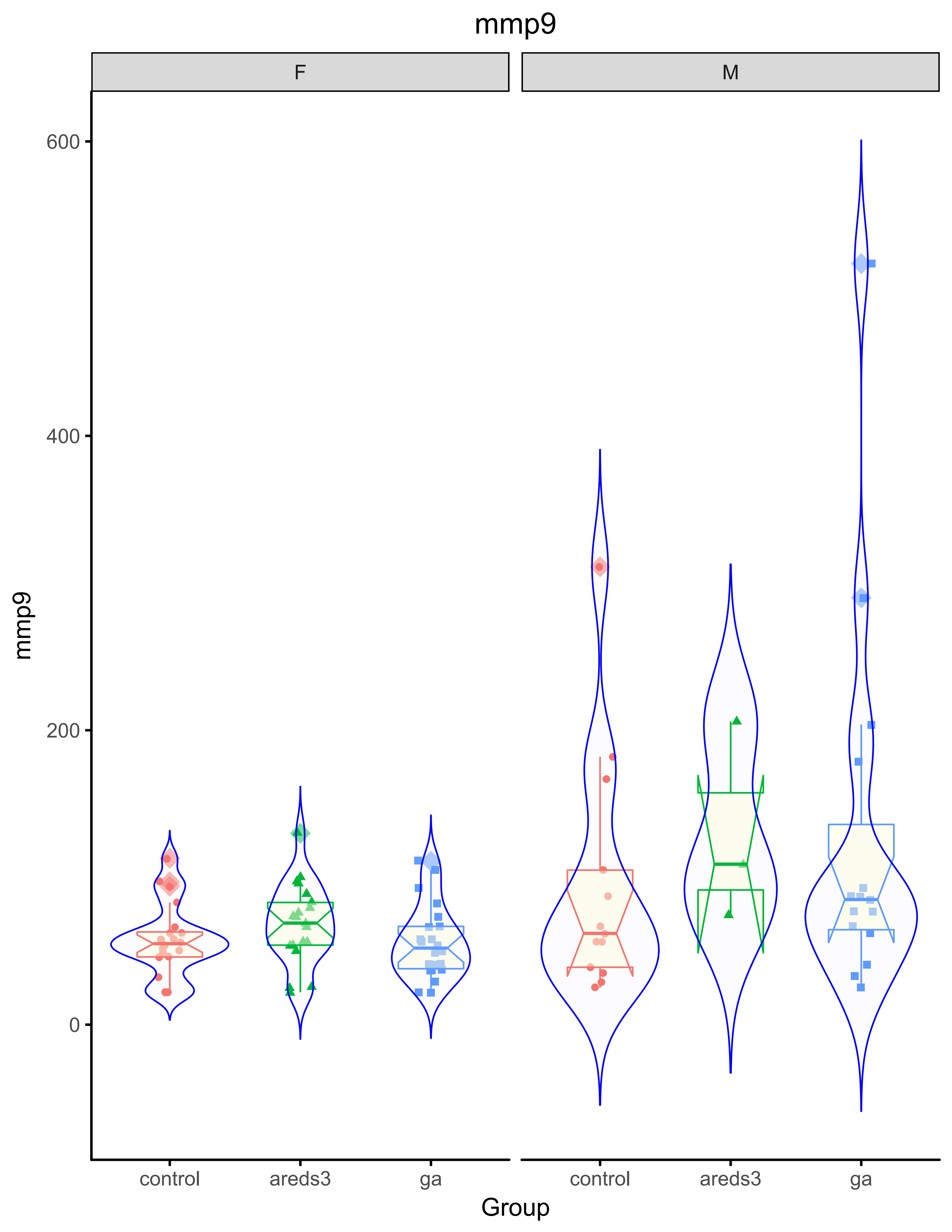
mmp9
F
M
600
400
200
0
mmp9
control
areds3
ga
control
areds3
ga
Group

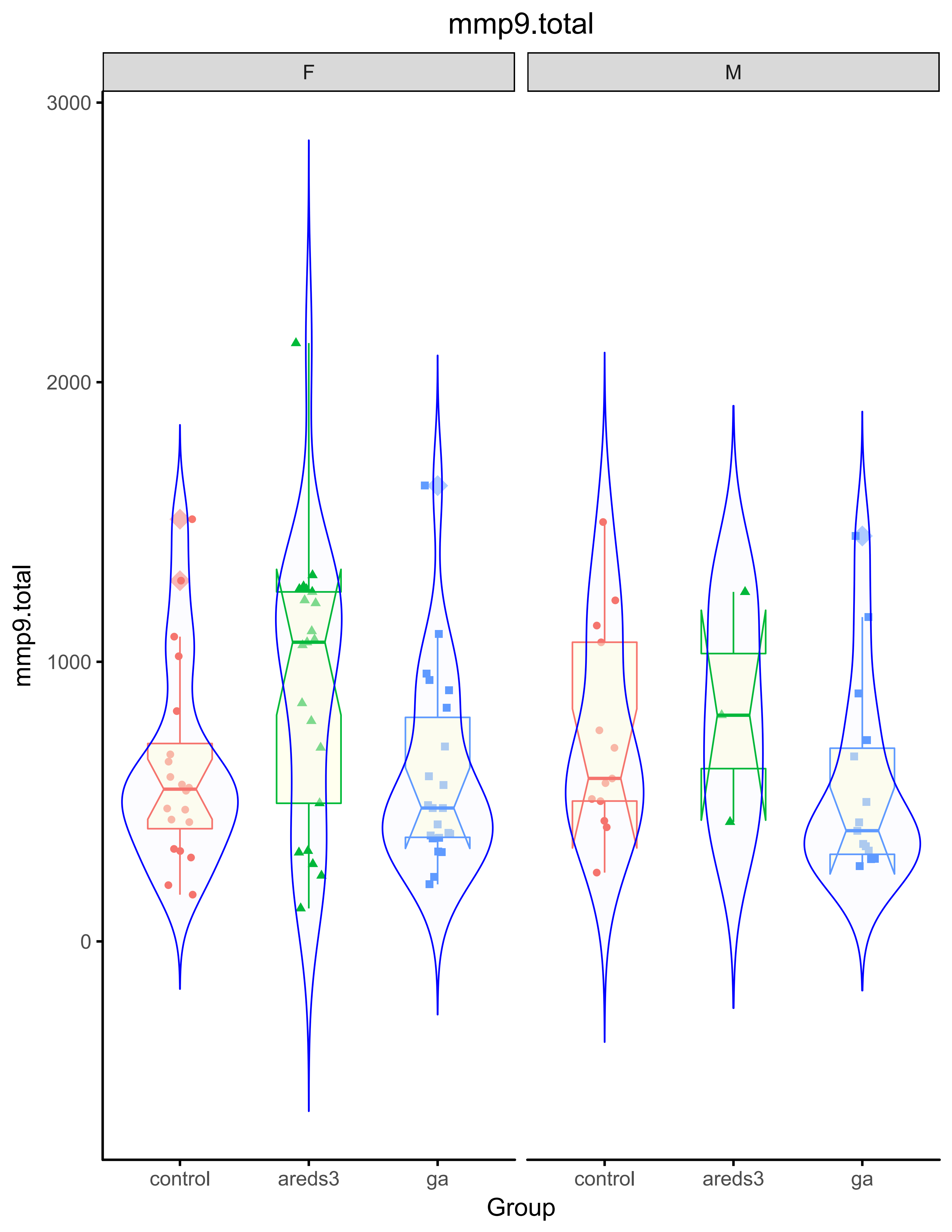
mmp9.total
F
M
3000
2000
1000
0
mmp9.total
control
areds3
ga
control
areds3
ga
Group

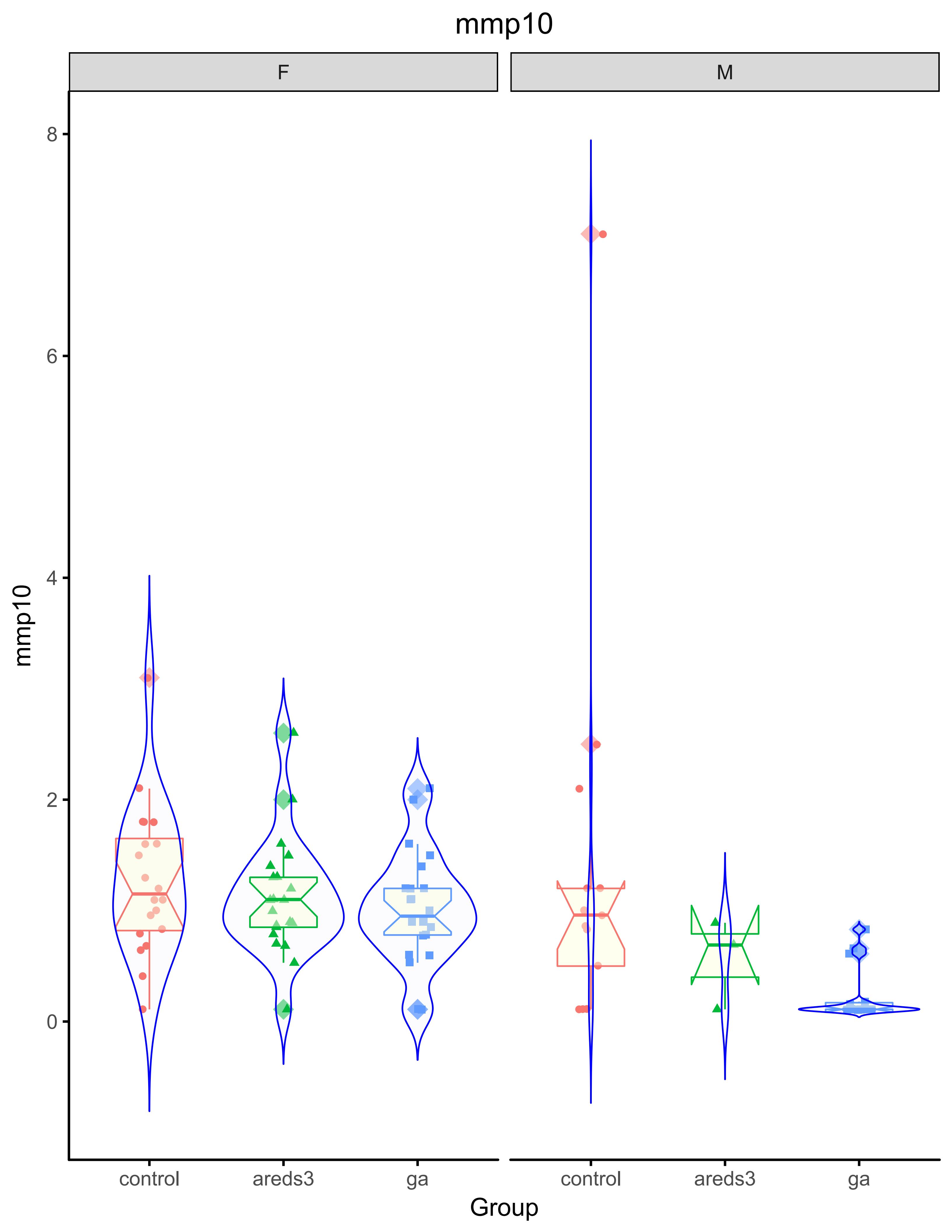

mmp10
F
M
8
6
4
2
0
mmp10
control
areds3
ga
control
areds3
ga
Group

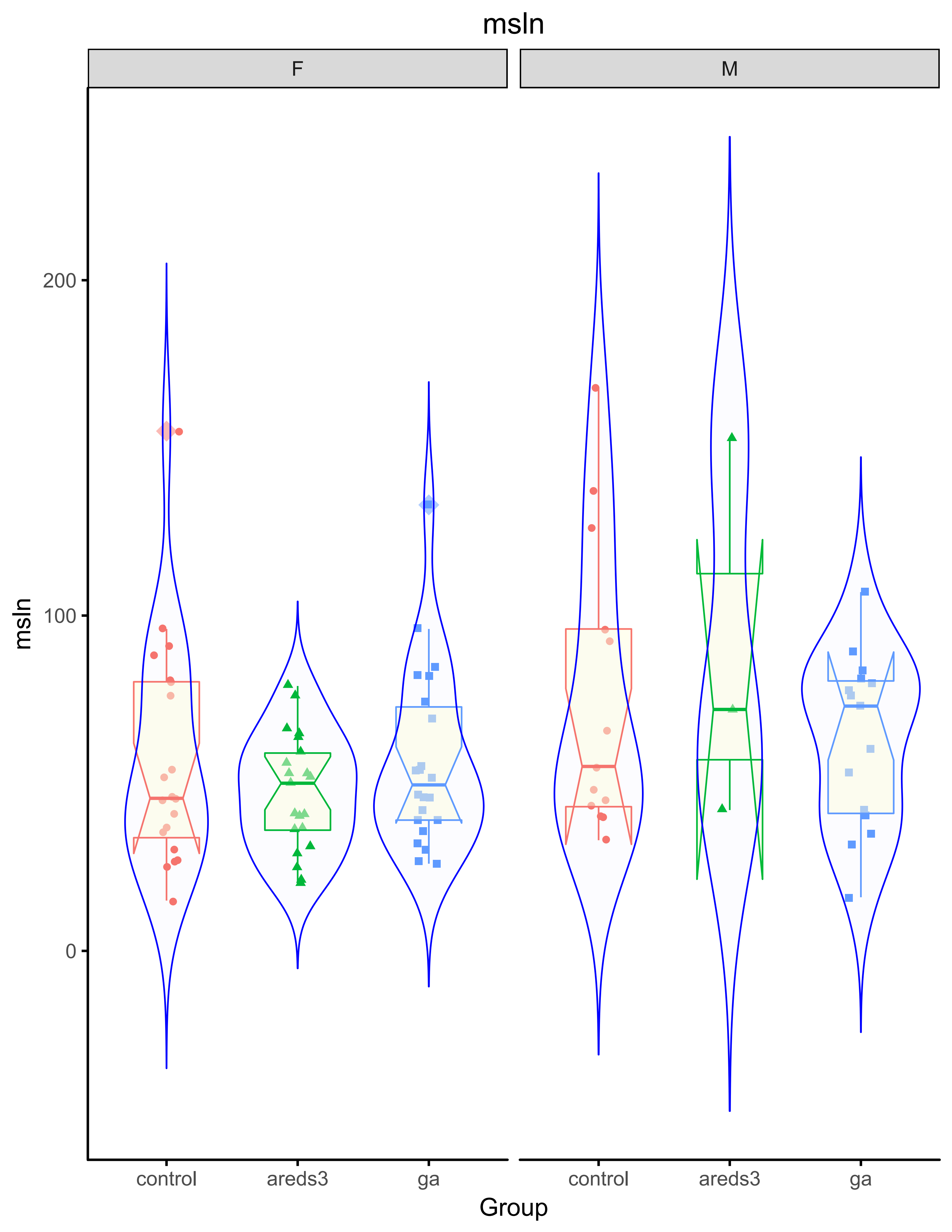
msln
F
M
200
100
0
msln
control
areds3
ga
control
areds3
ga
Group

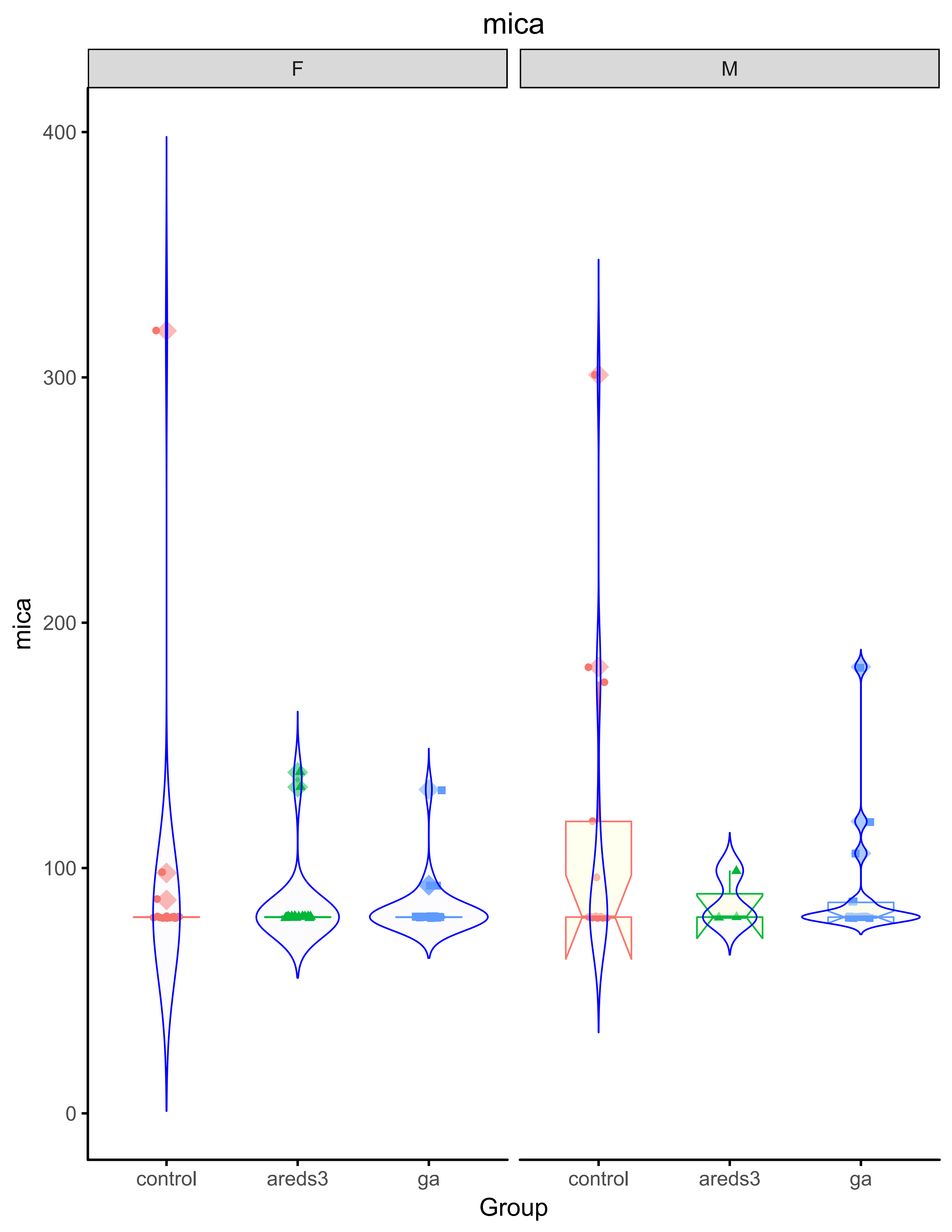

mica
F
M
400
300
200
100
0
mica
control
areds3
ga
control
areds3
ga
Group

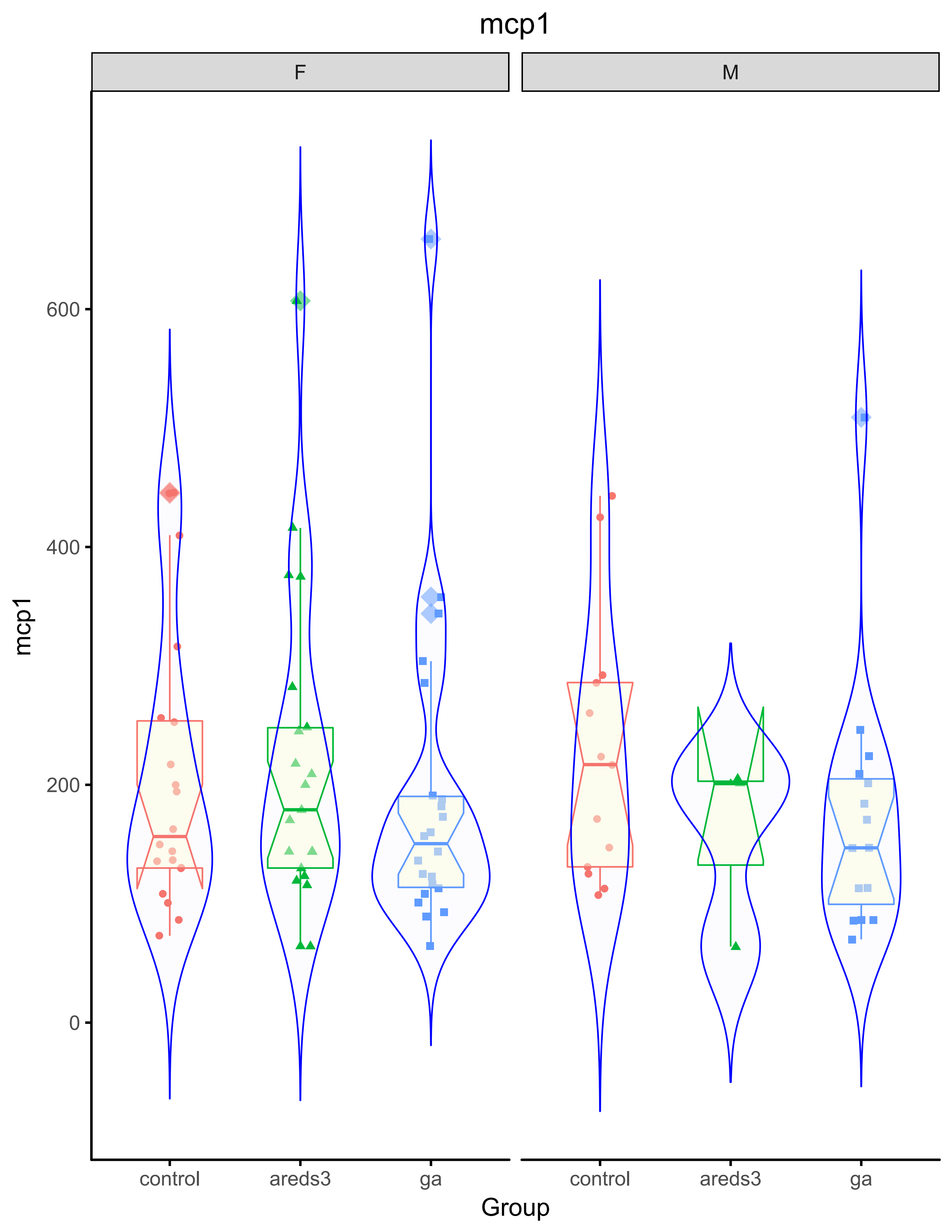
mcp1
F
M
600
400
200
0
mcp1
control
areds3
ga
control
areds3
ga
Group

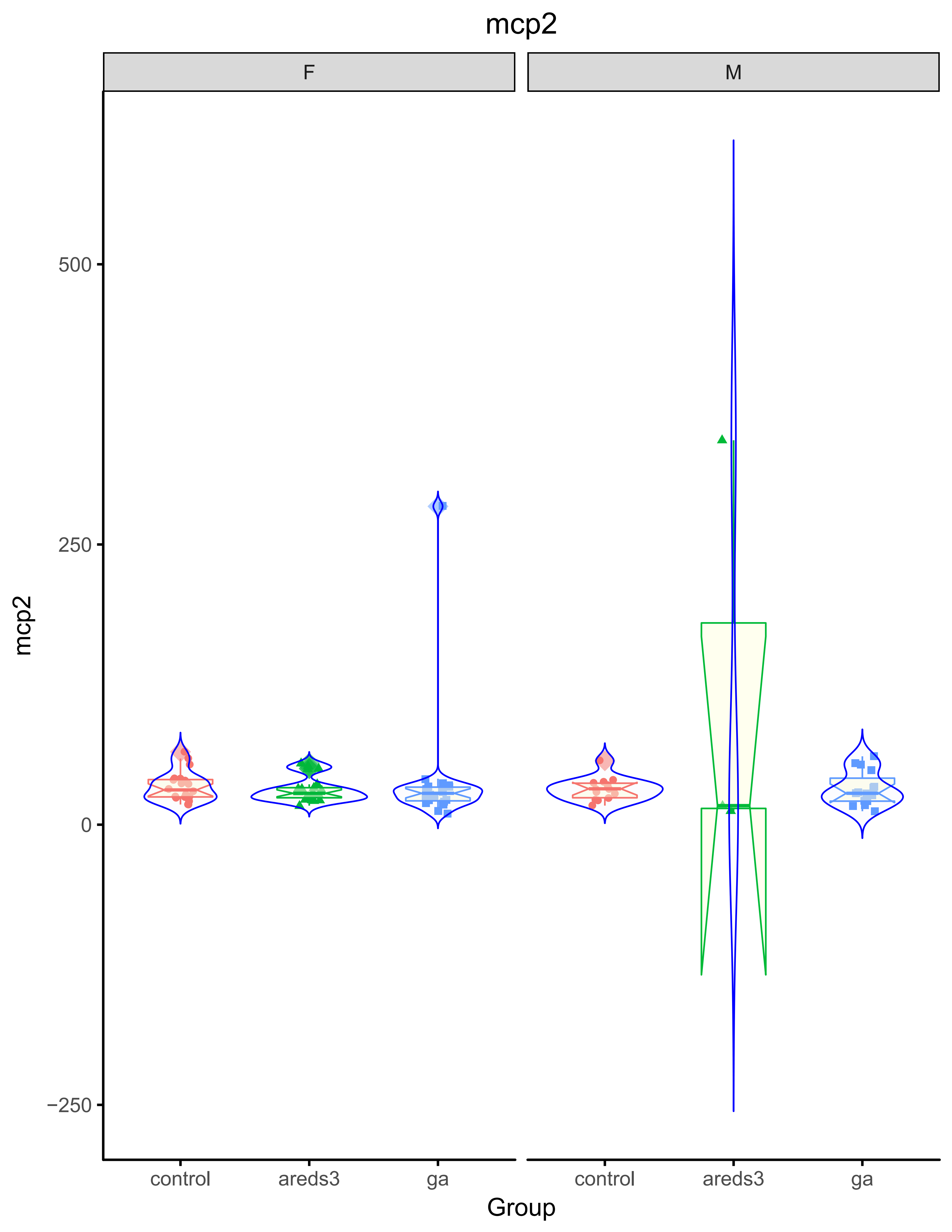
mcp2
F
M
500
250
0
−250
mcp2
control
areds3
ga
control
areds3
ga
Group

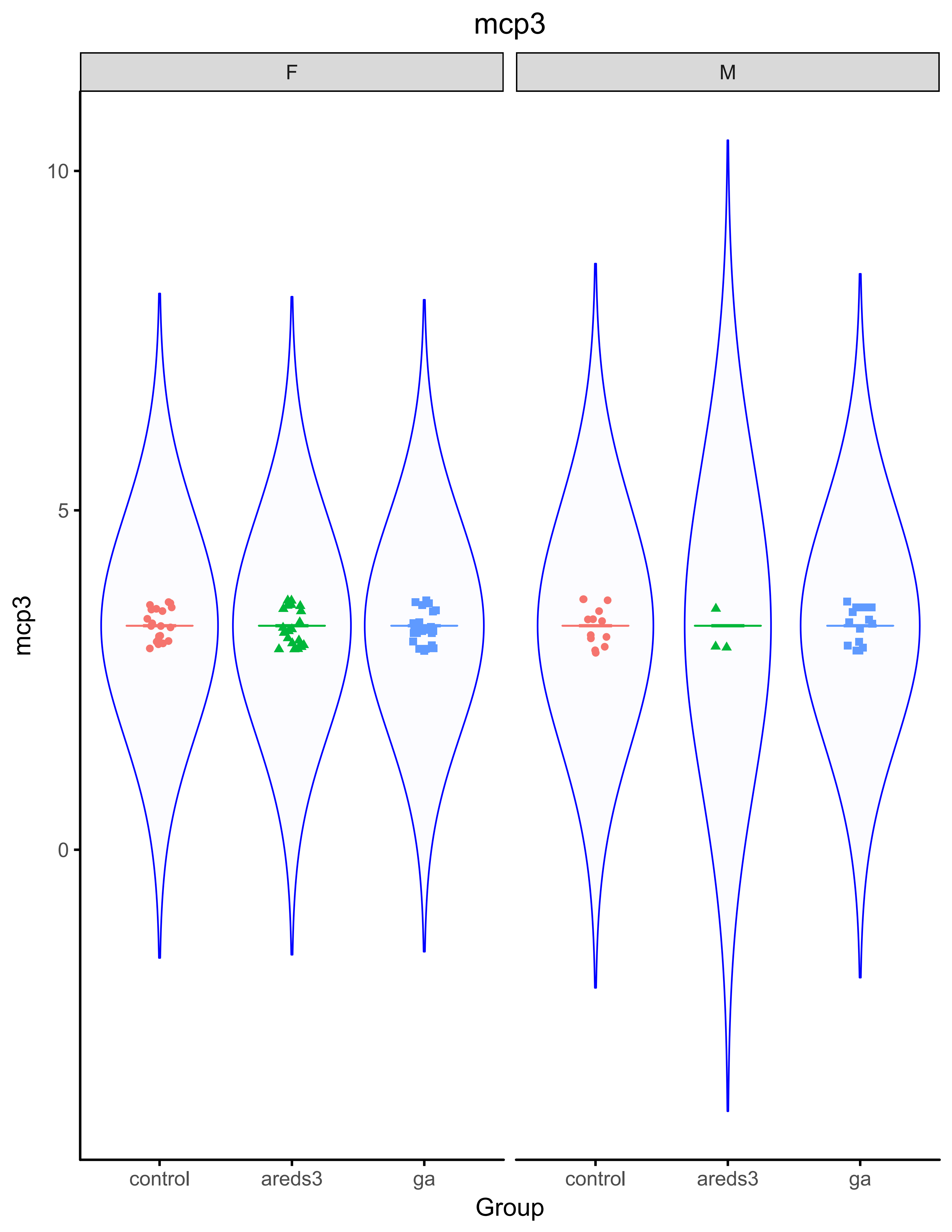
mcp3
F
M
10
5
0
mcp3
control
areds3
ga
control
areds3
ga
Group

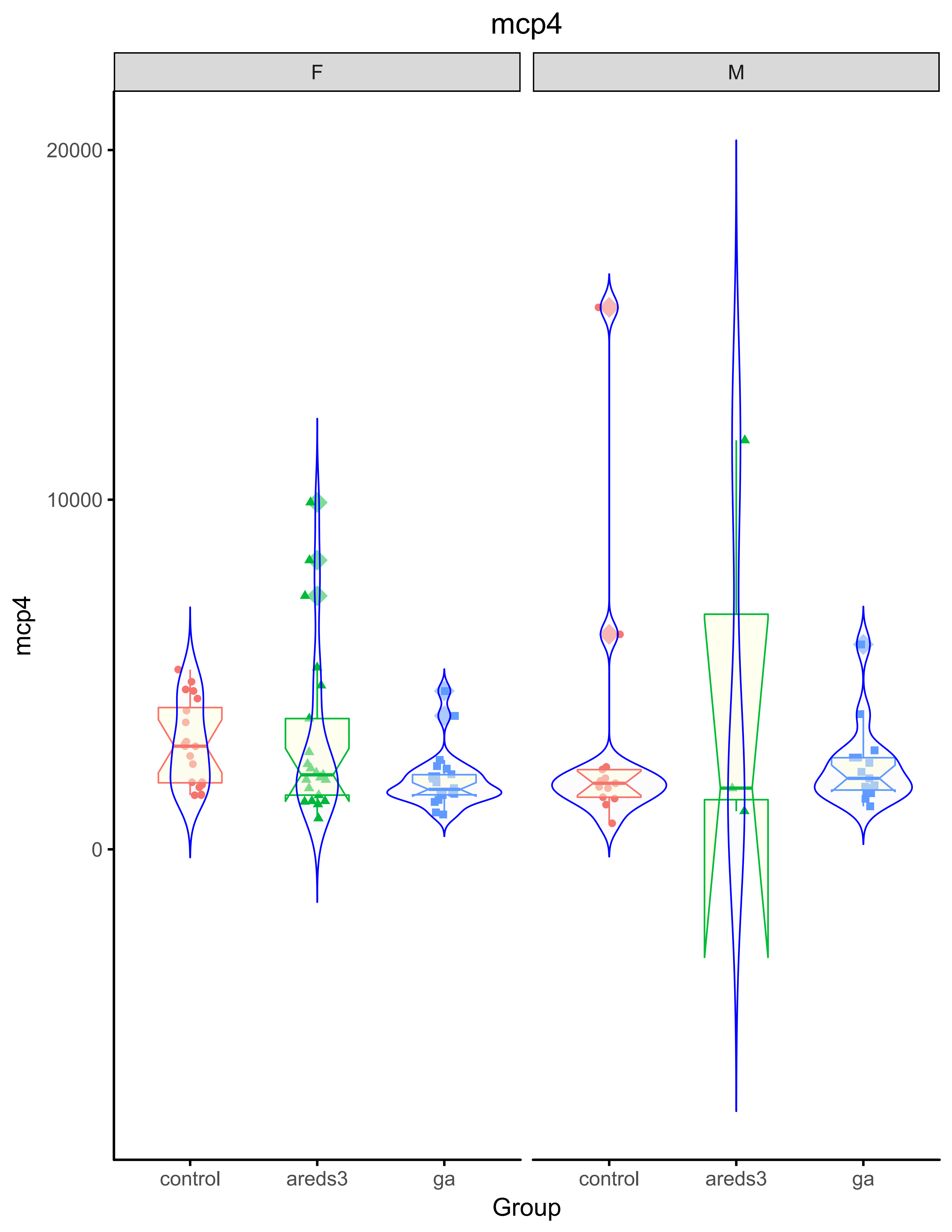
mcp4
F
M
20000
10000
0
mcp4
control
areds3
ga
control
areds3
ga
Group

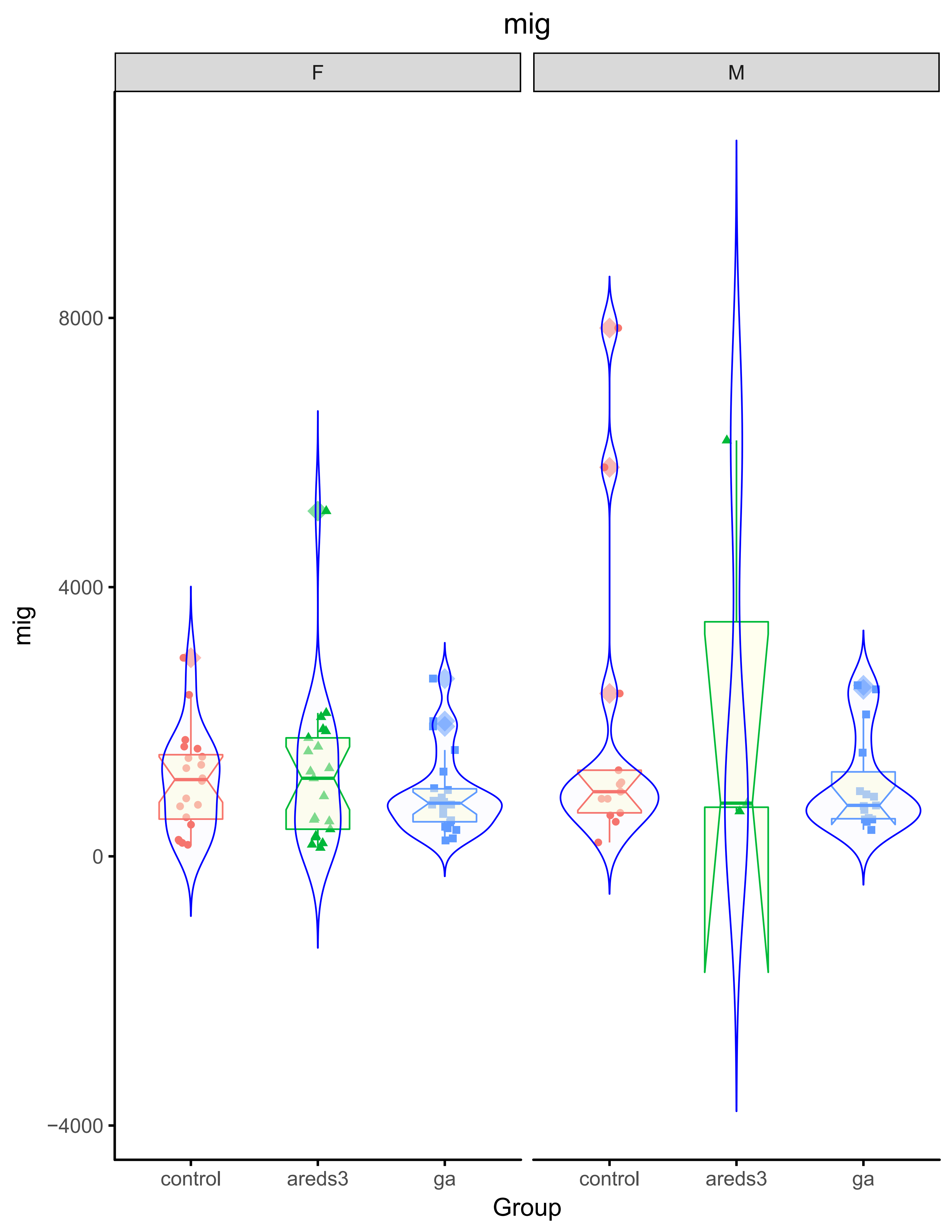

mig
F
M
8000
4000
0
−4000
mig
control
areds3
ga
control
areds3
ga
Group

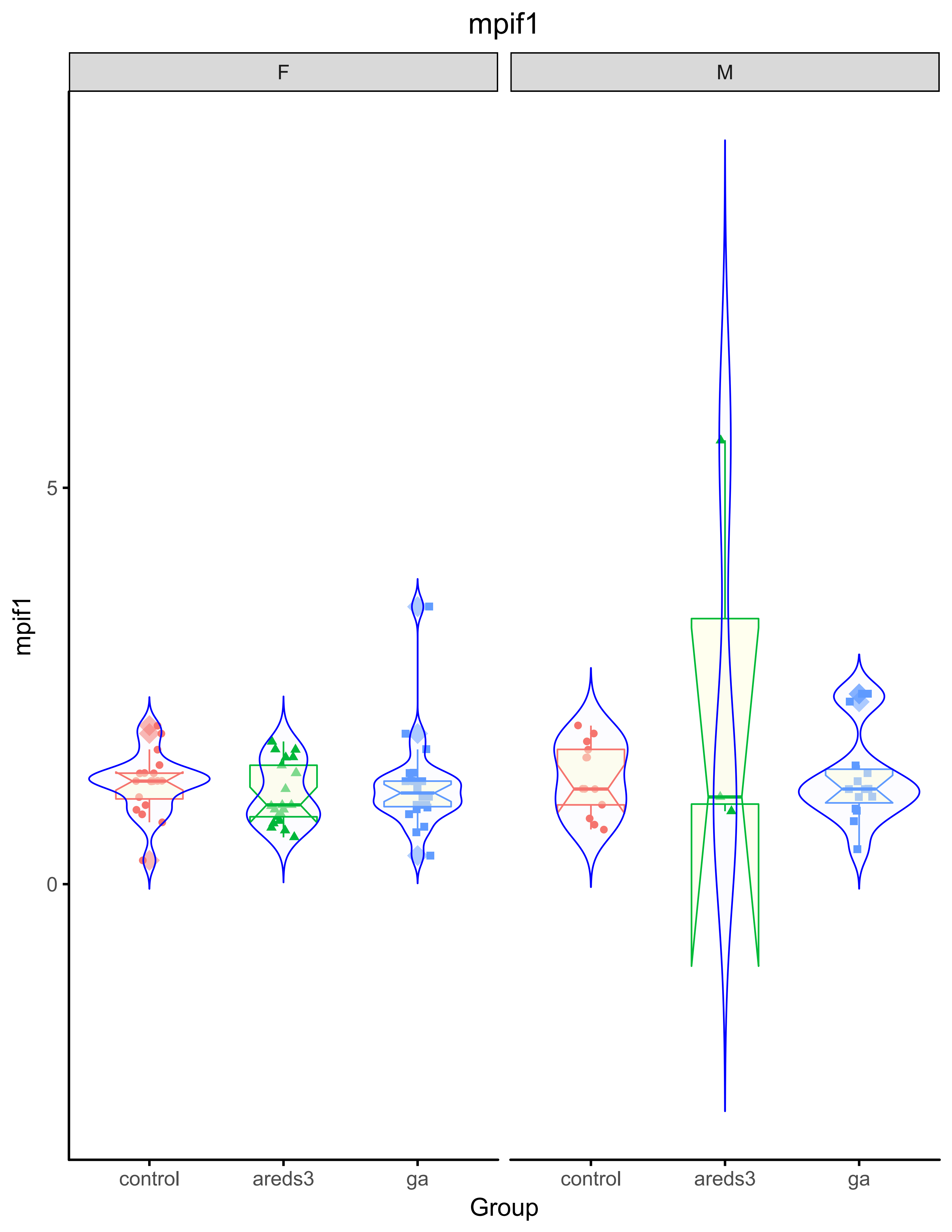
mpif1
F
M
mpif1
5
0
control
areds3
ga
control
areds3
ga
Group

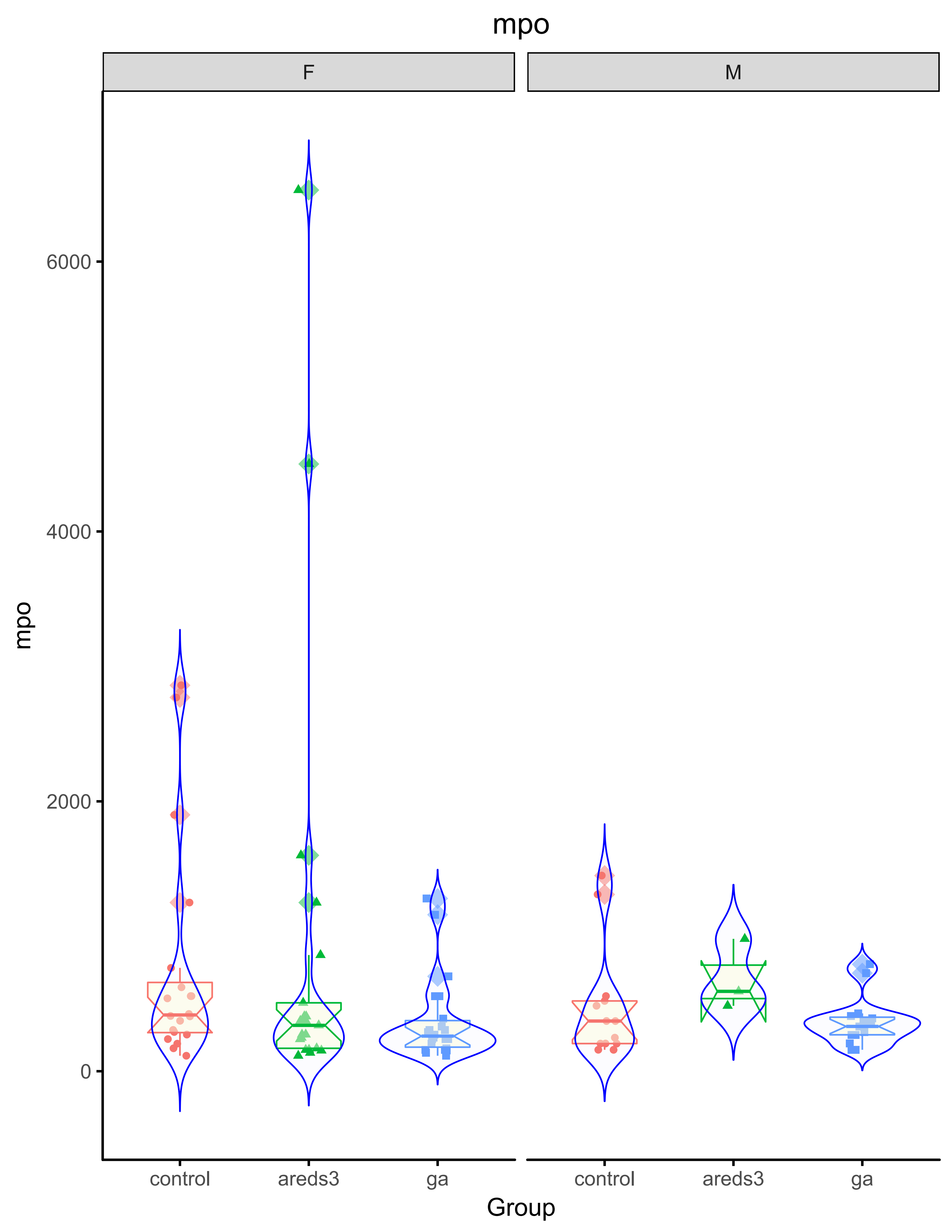
mpo
F
M
6000
4000
2000
0
mpo
control
areds3
ga
control
areds3
ga
Group

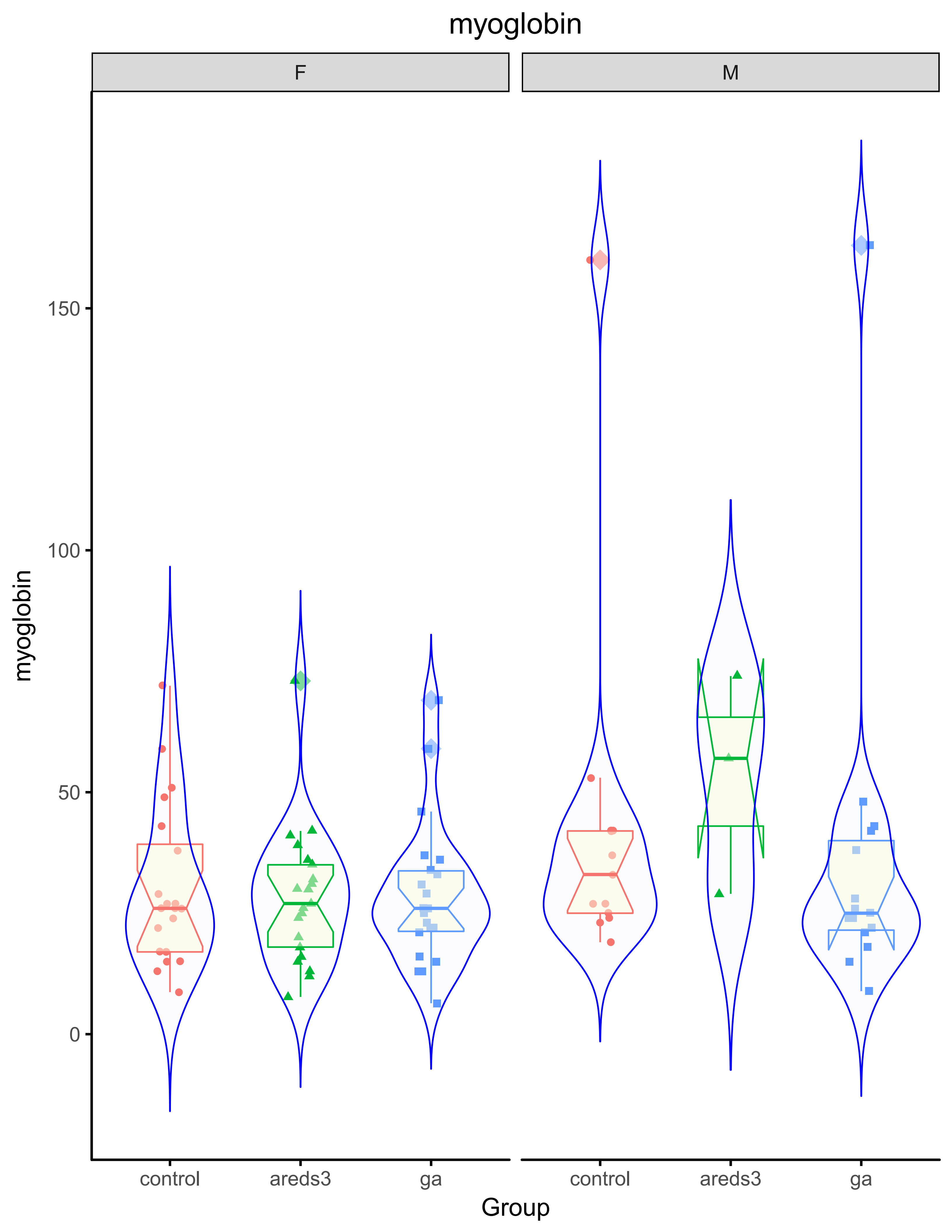
myoglobin
F
M
150
100
50
0
myoglobin
control
areds3
ga
control
areds3
ga
Group

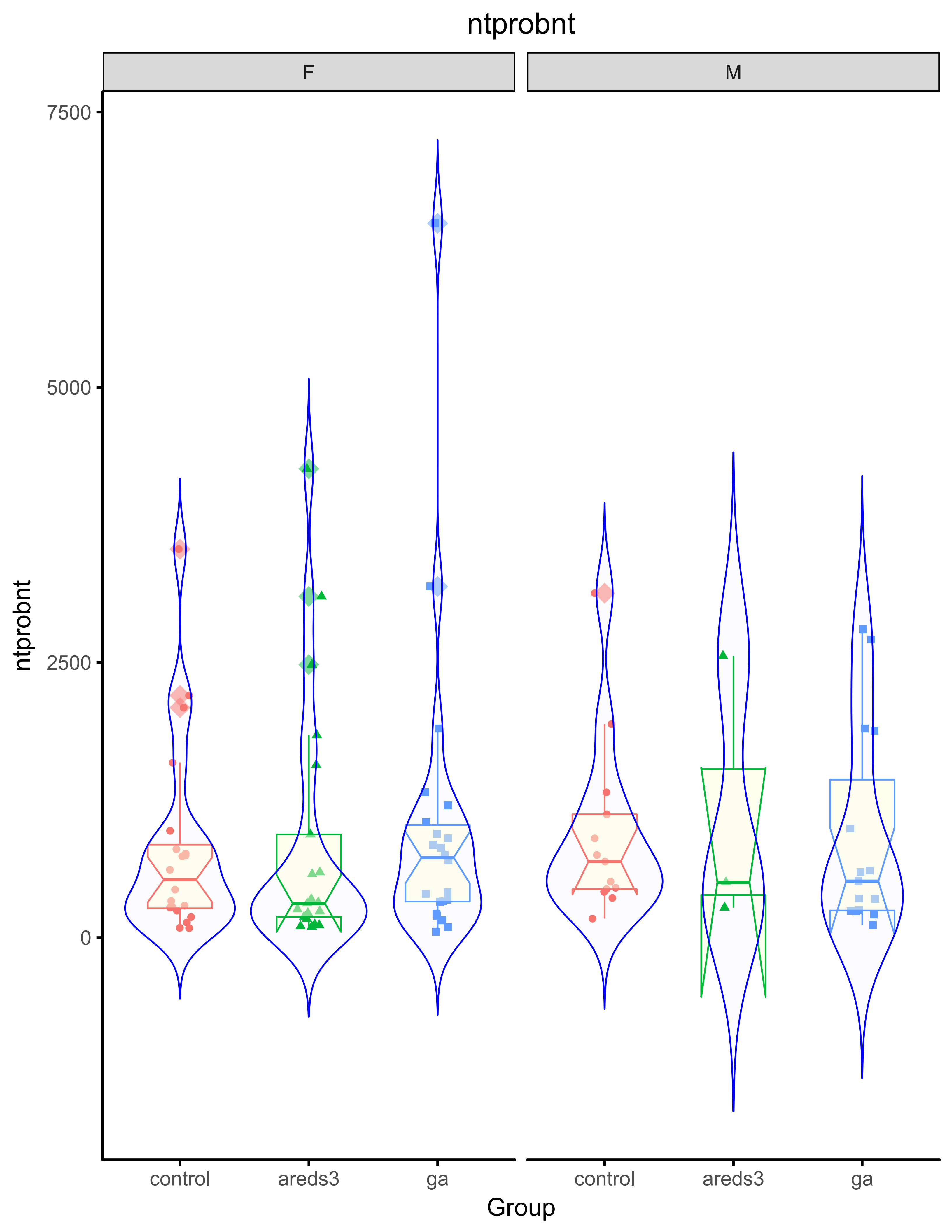

ntprobnt
F
M
7500
5000
2500
0
ntprobnt
control
areds3
ga
control
areds3
ga
Group

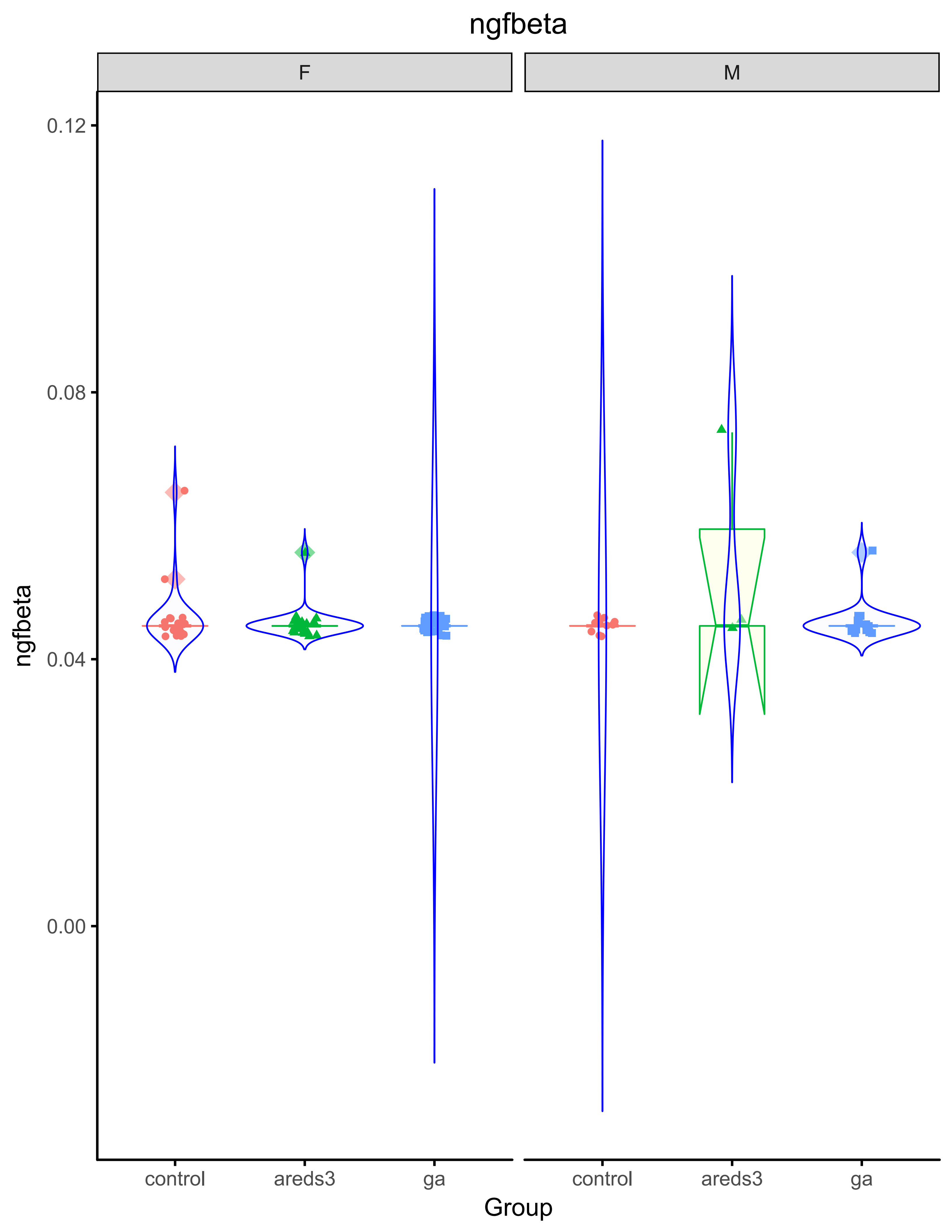

ngfbeta
F
M
0.12
0.08
0.04
0.00
ngfbeta
control
areds3
ga
control
areds3
ga
Group

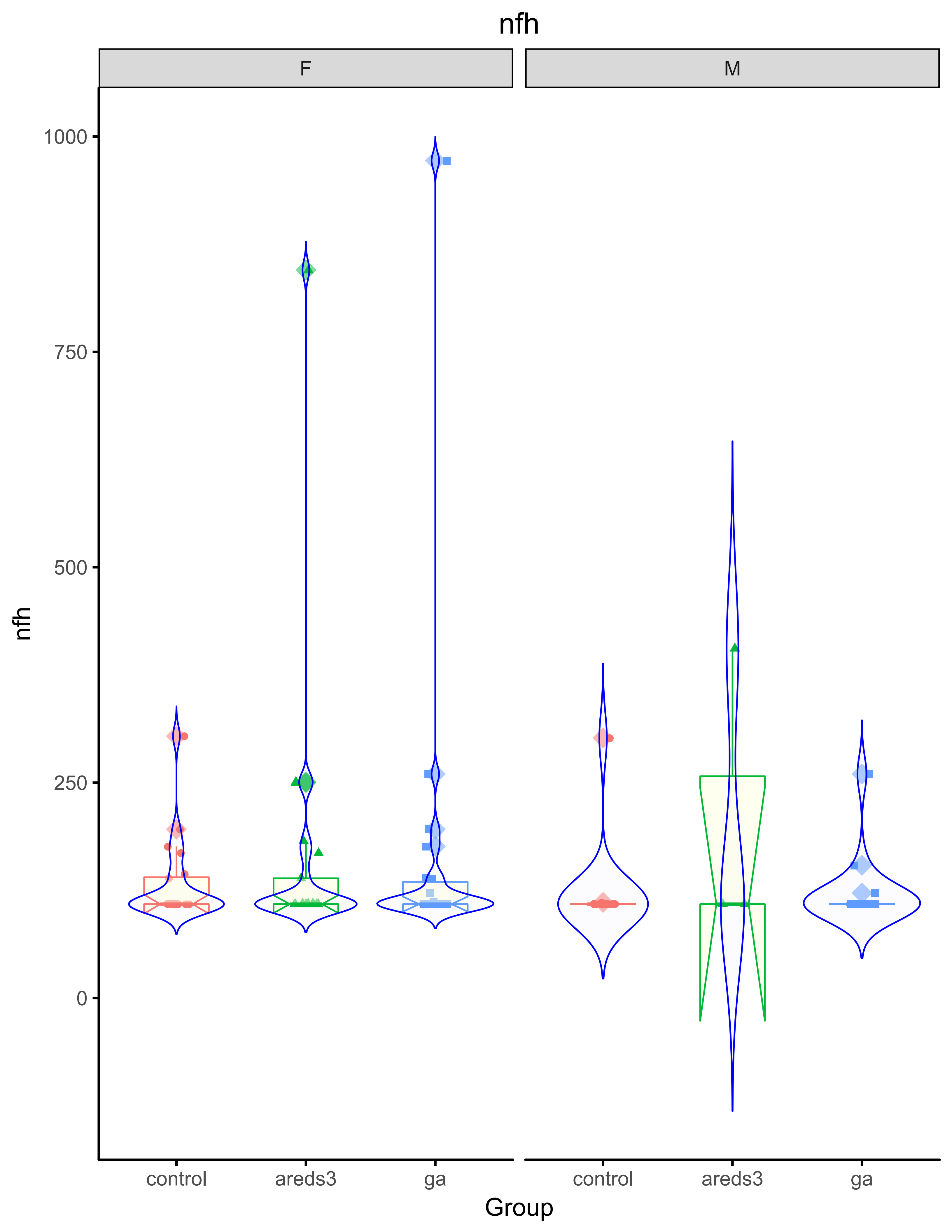

nfh
F
M
1000
750
500
250
0
nfh
control
areds3
ga
control
areds3
ga
Group

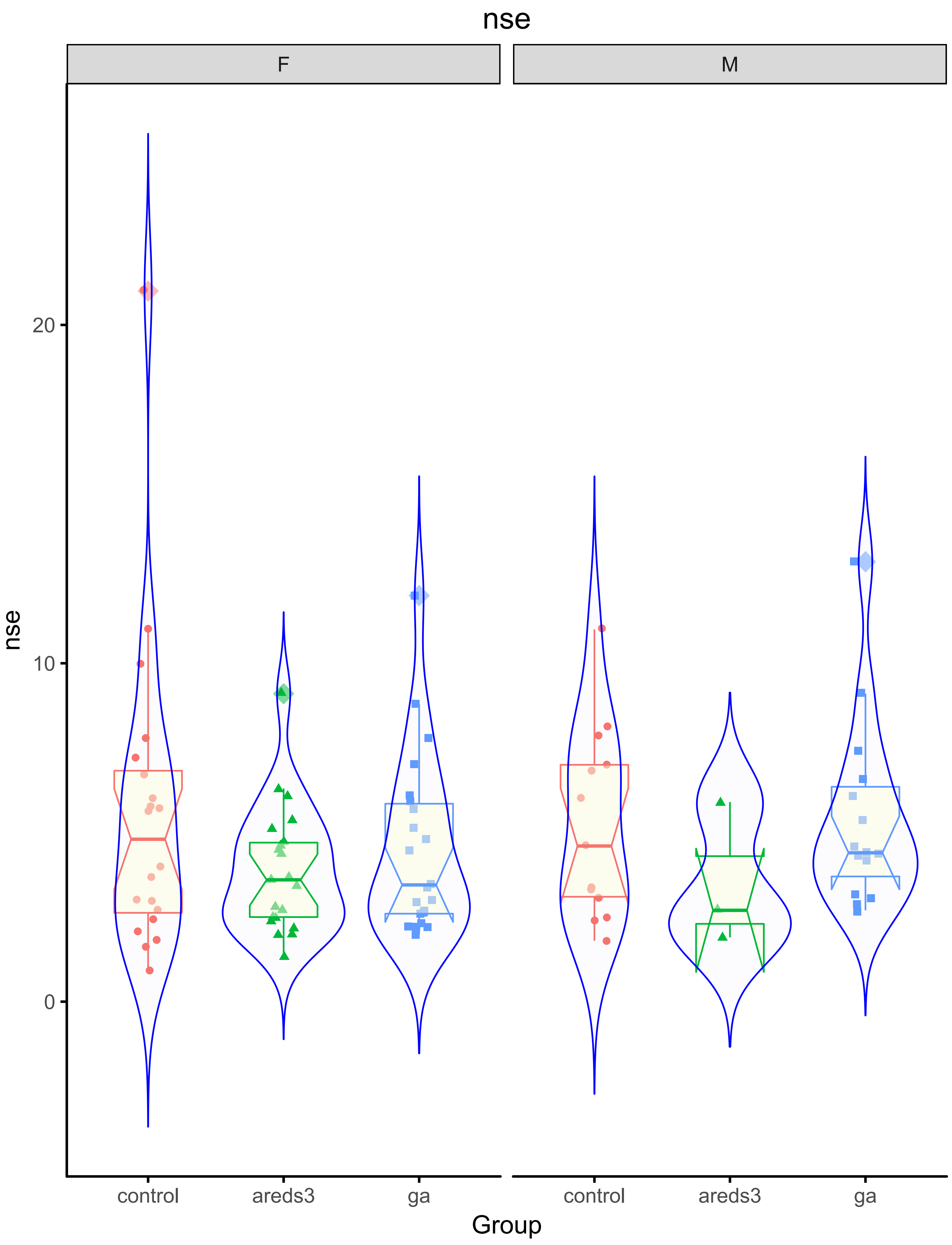
nse
F
M
20
10
0
nse
control
areds3
ga
control
areds3
ga
Group

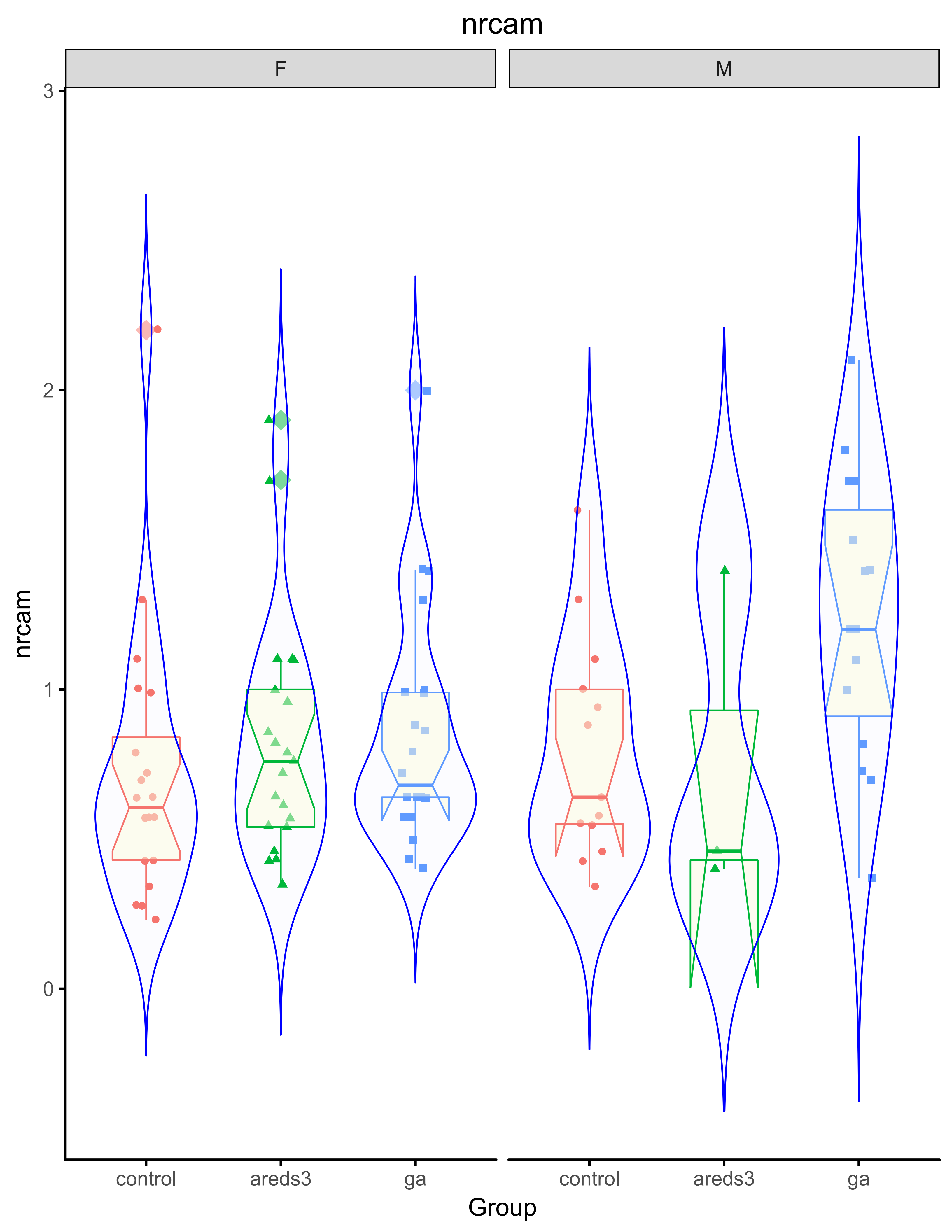

nrcam
F
M
3
2
1
0
nrcam
control
areds3
ga
control
areds3
ga
Group

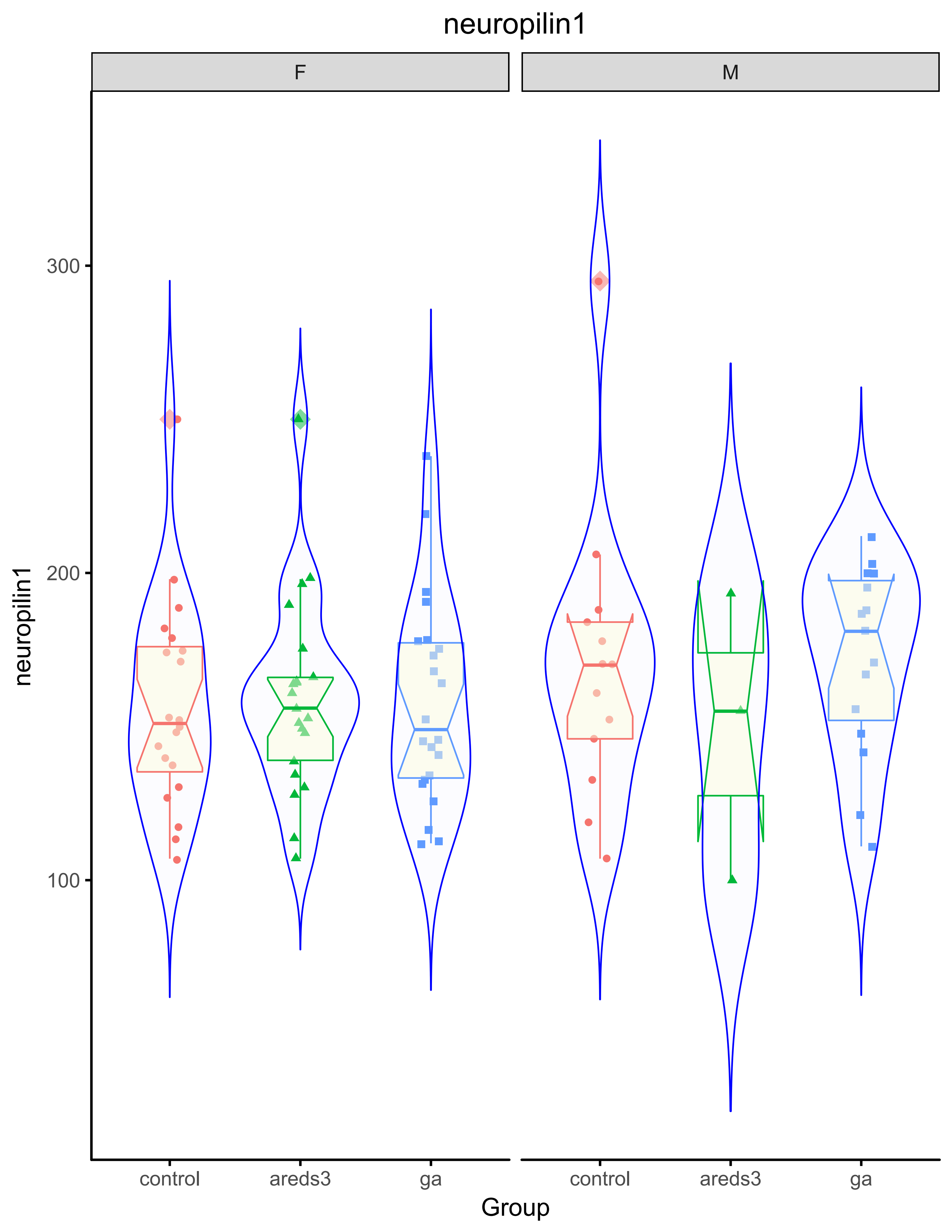

neuropilin1
F
M
300
200
100
neuropilin1
control
areds3
ga
control
areds3
ga
Group

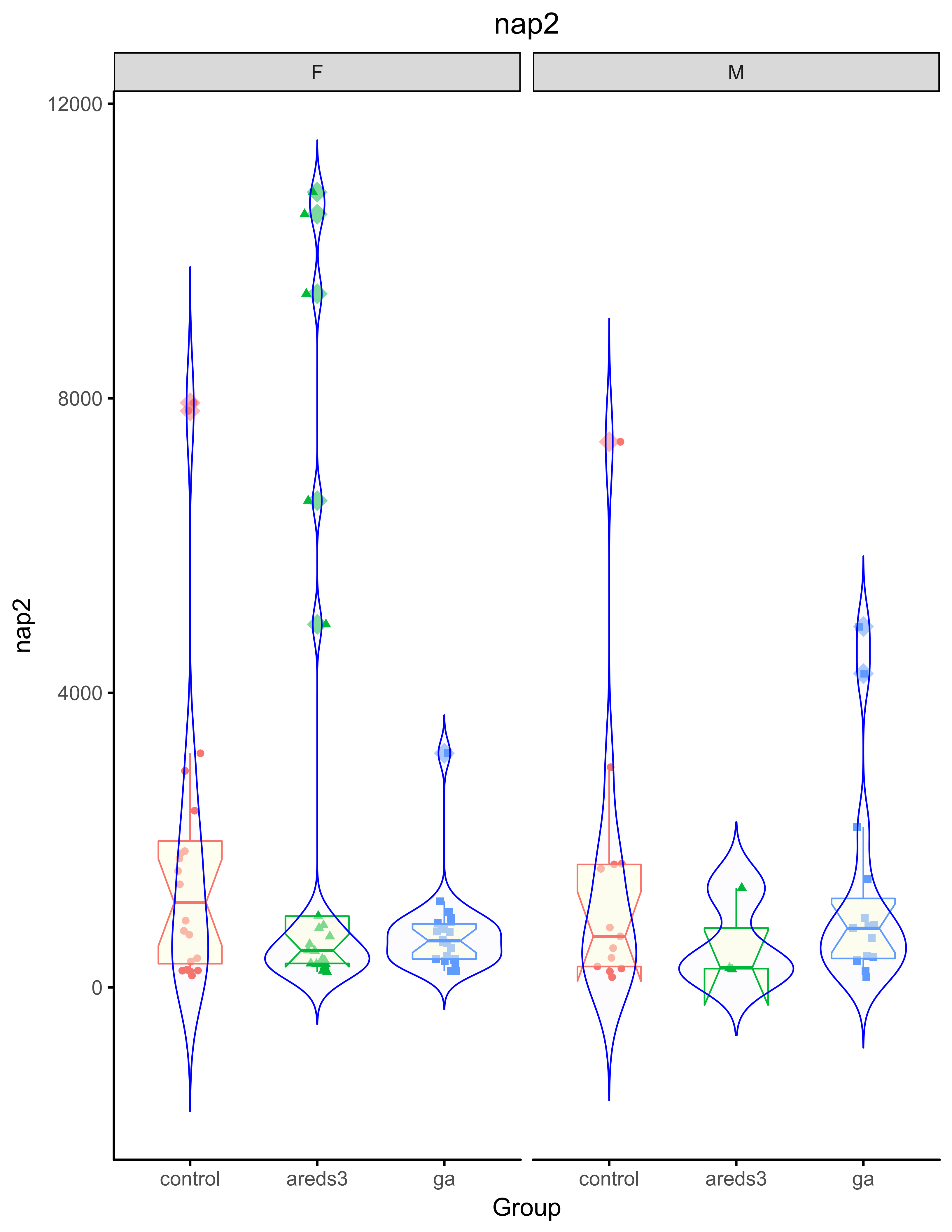
nap2
F
M
12000
8000
4000
0
nap2
control
areds3
ga
control
areds3
ga
Group

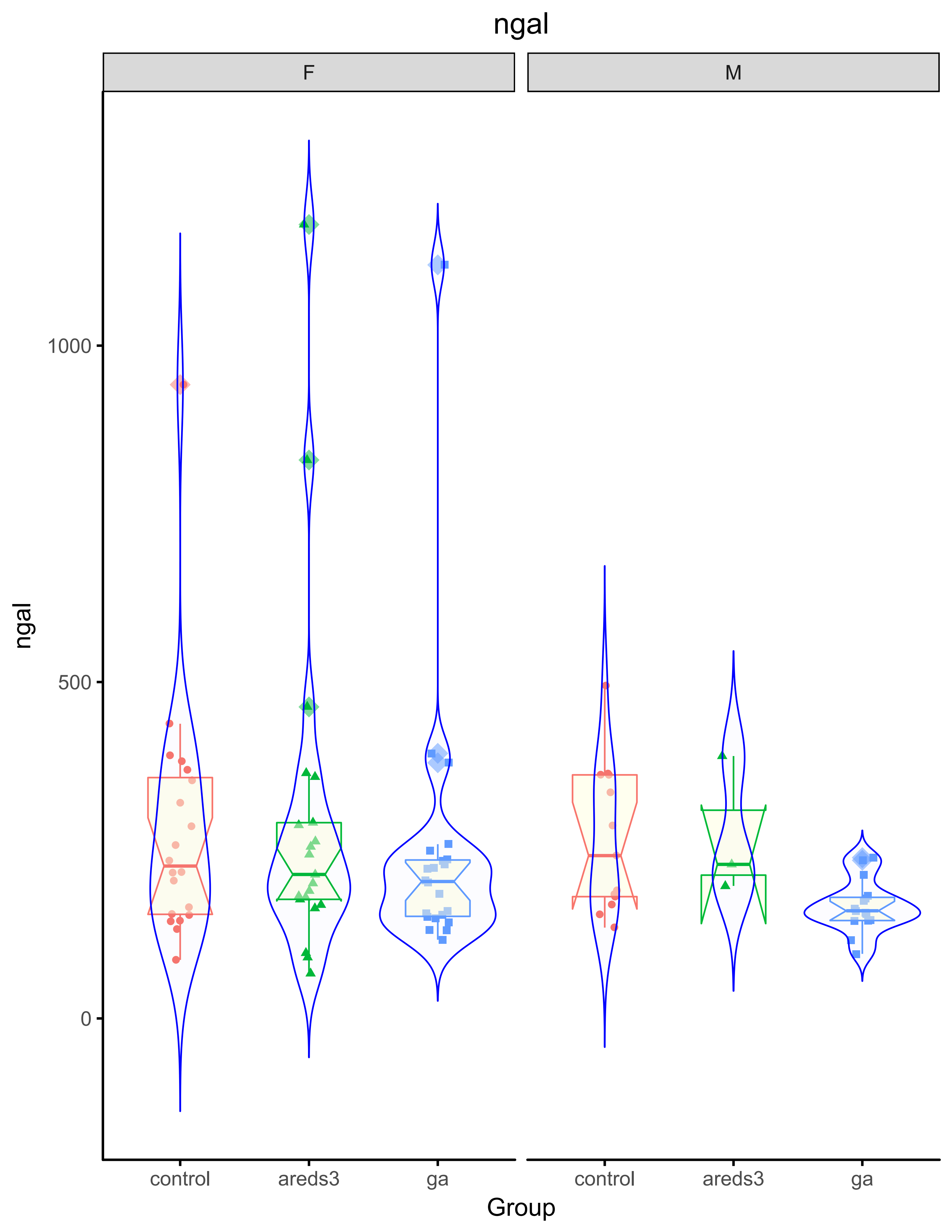
ngal
F
M
1000
500
0
ngal
control
areds3
ga
control
areds3
ga
Group

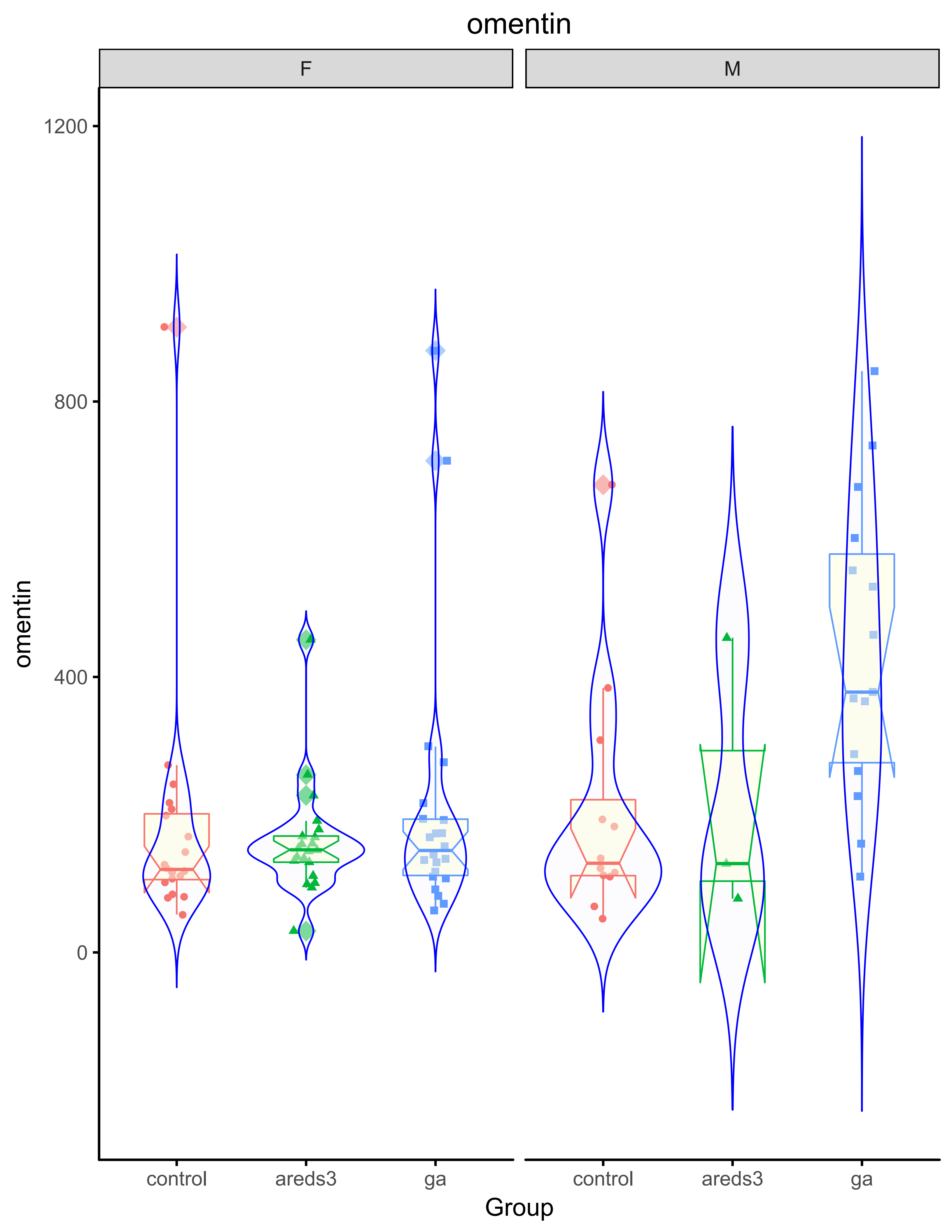
omentin
F
M
1200
800
400
0
omentin
control
areds3
ga
control
areds3
ga
Group

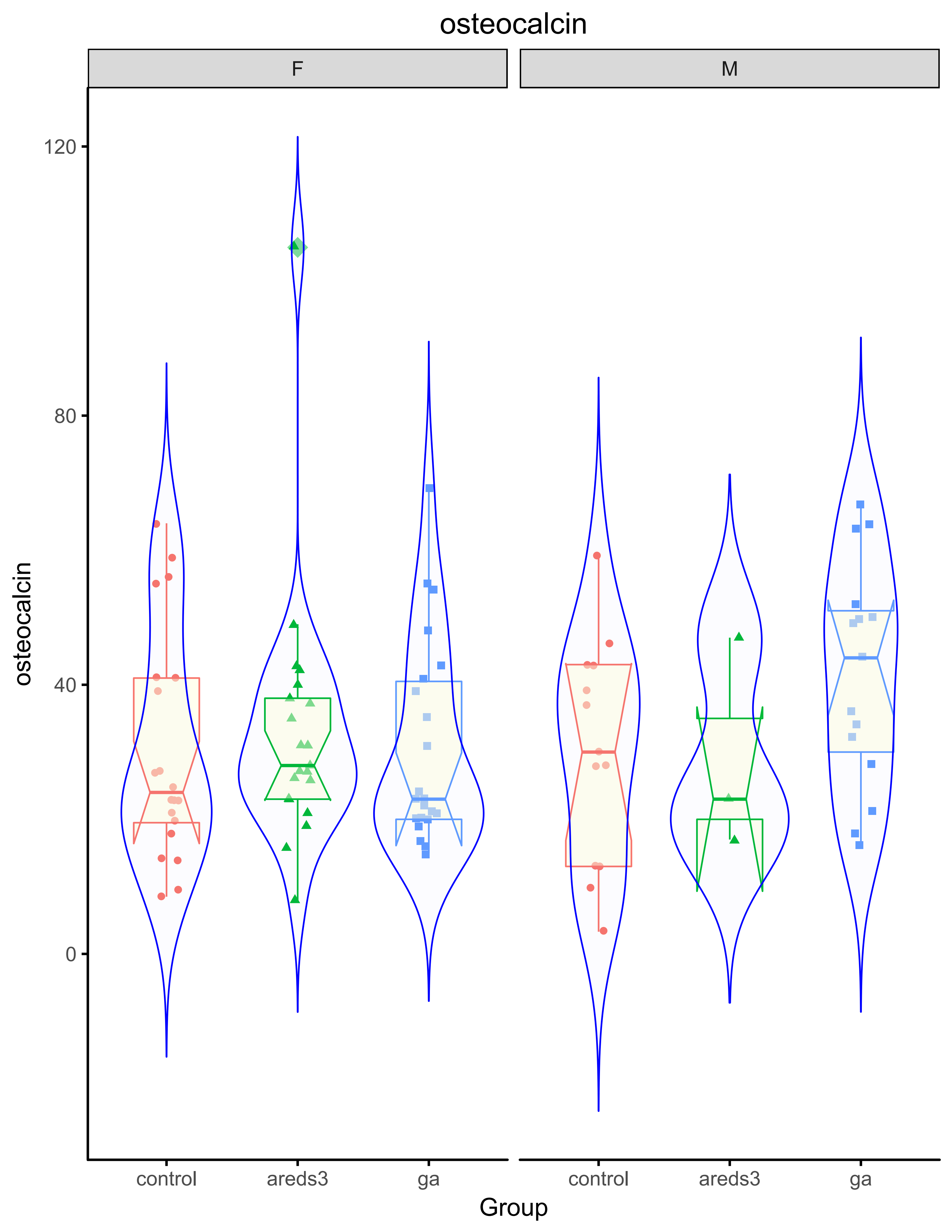

osteocalcin
F
M
120
80
40
0
osteocalcin
control
areds3
ga
control
areds3
ga
Group

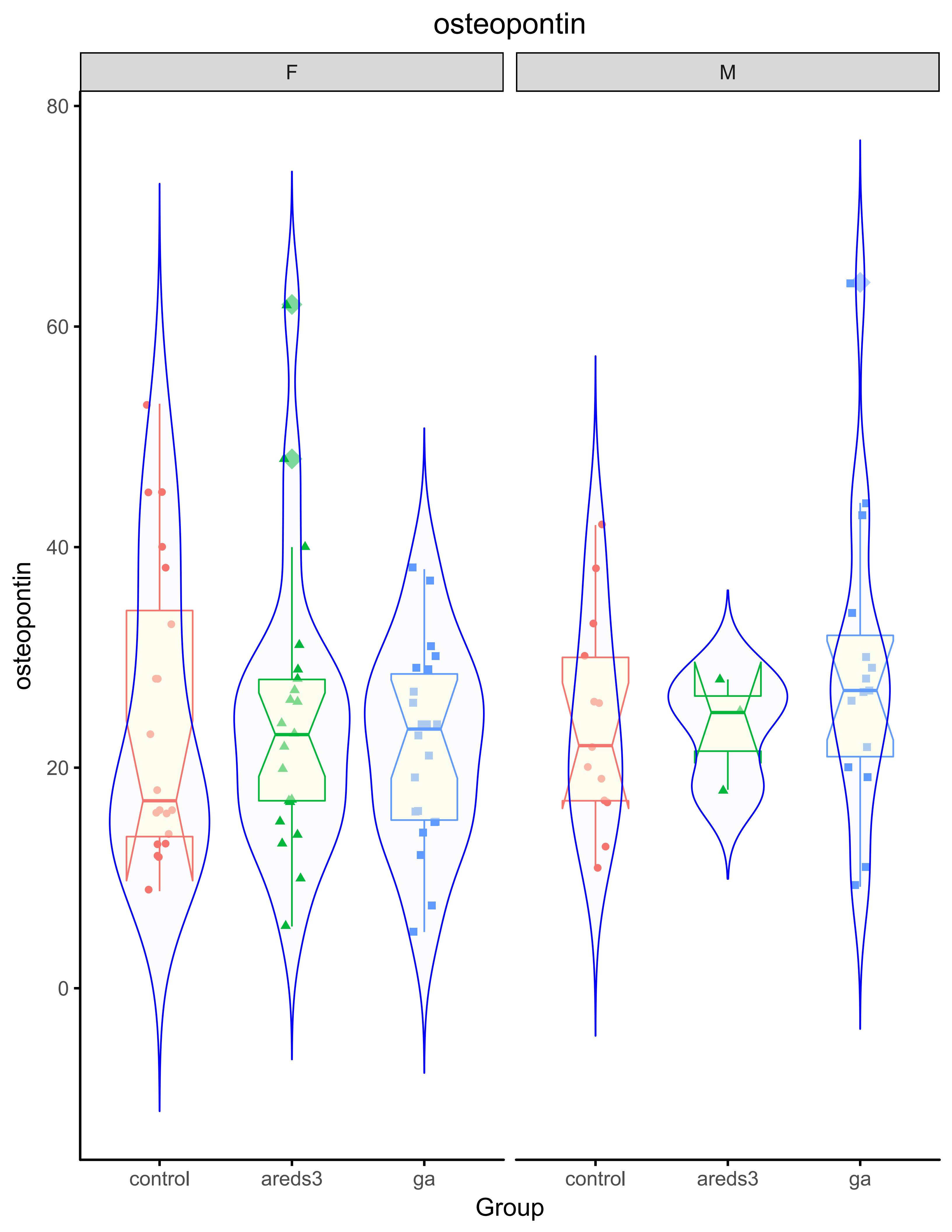

osteopontin
F
M
80
60
40
20
0
osteopontin
control
areds3
ga
control
areds3
ga
Group

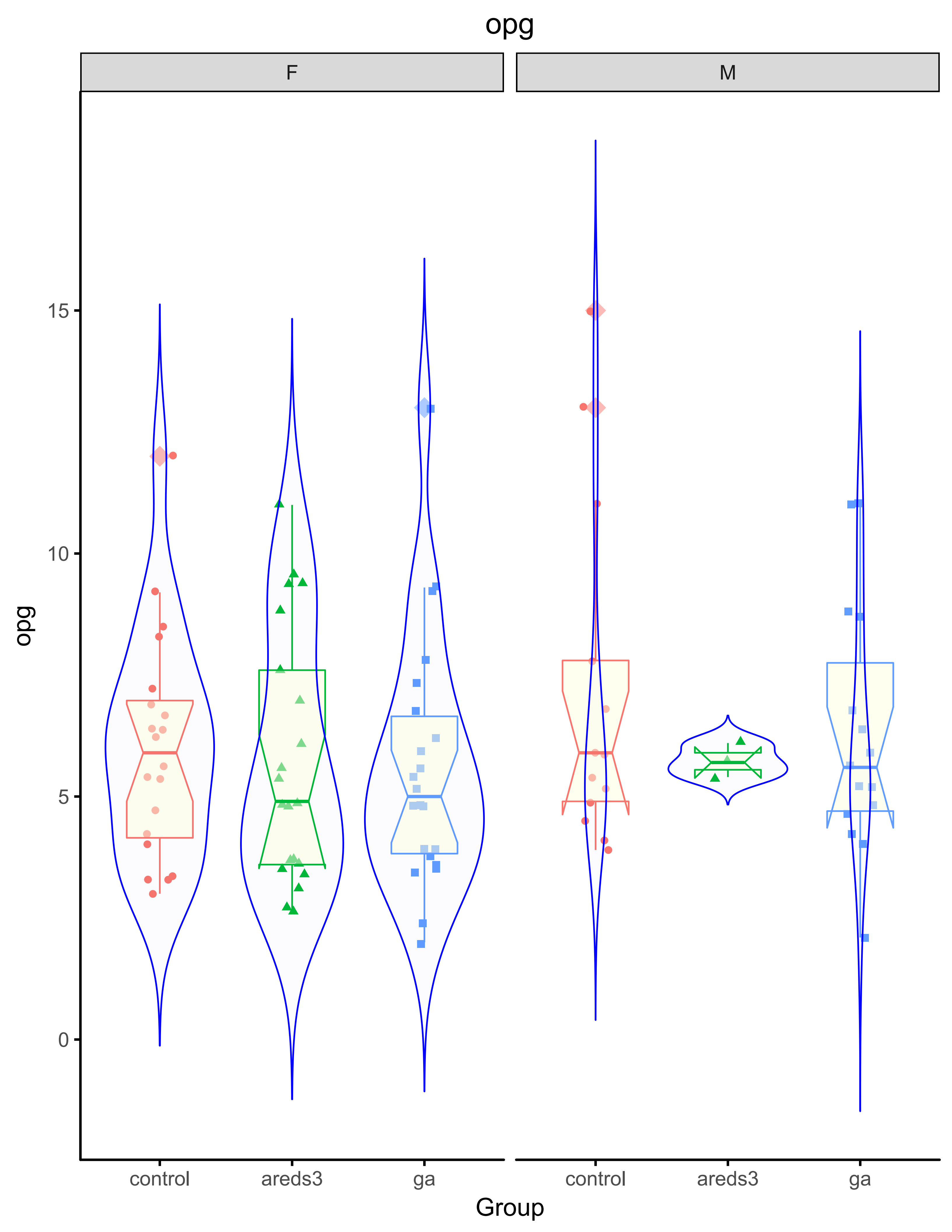

opg
F
M
15
10
5
0
opg
control
areds3
ga
control
areds3
ga
Group

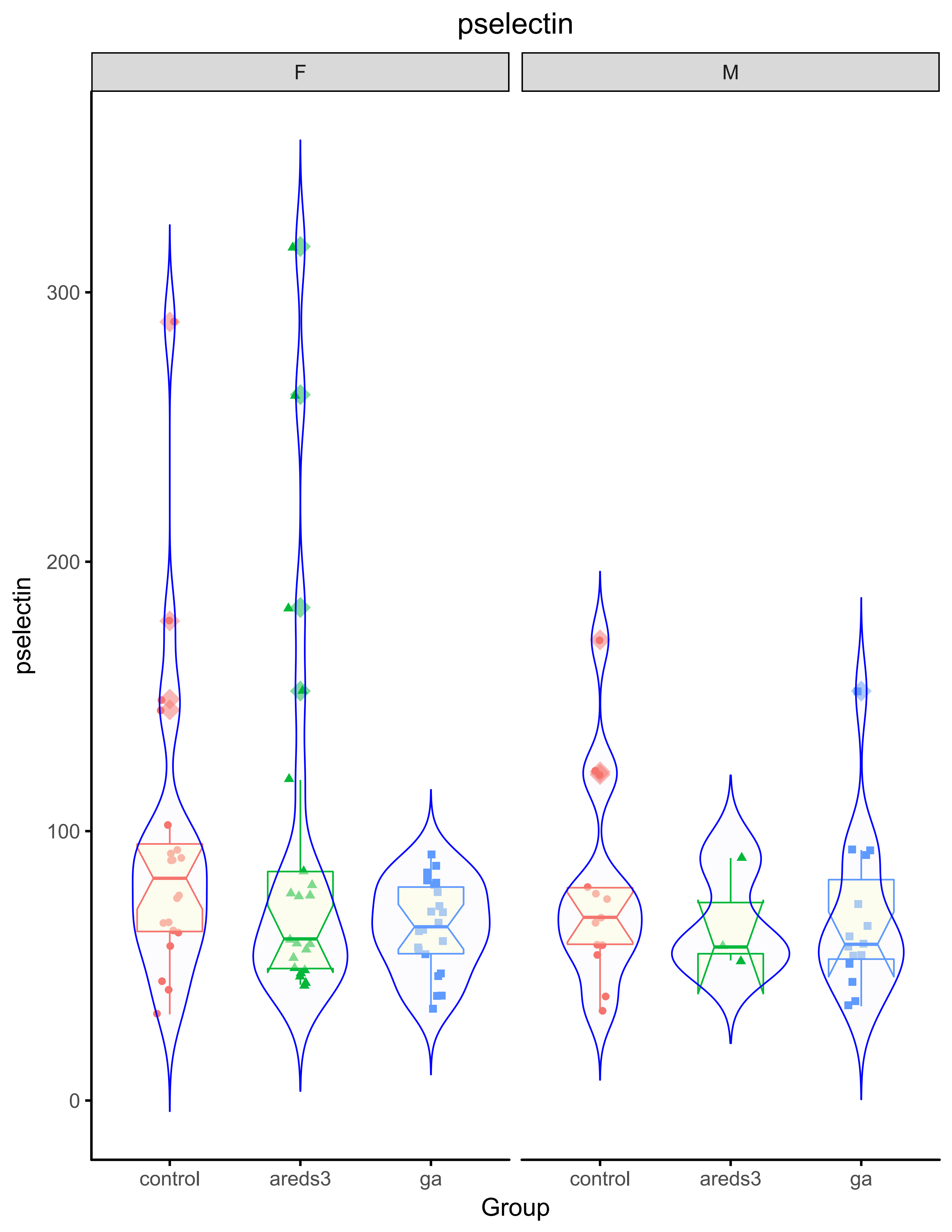
pselectin
F
M
pselectin
300
200
100
0
control
areds3
ga
control
areds3
ga
Group

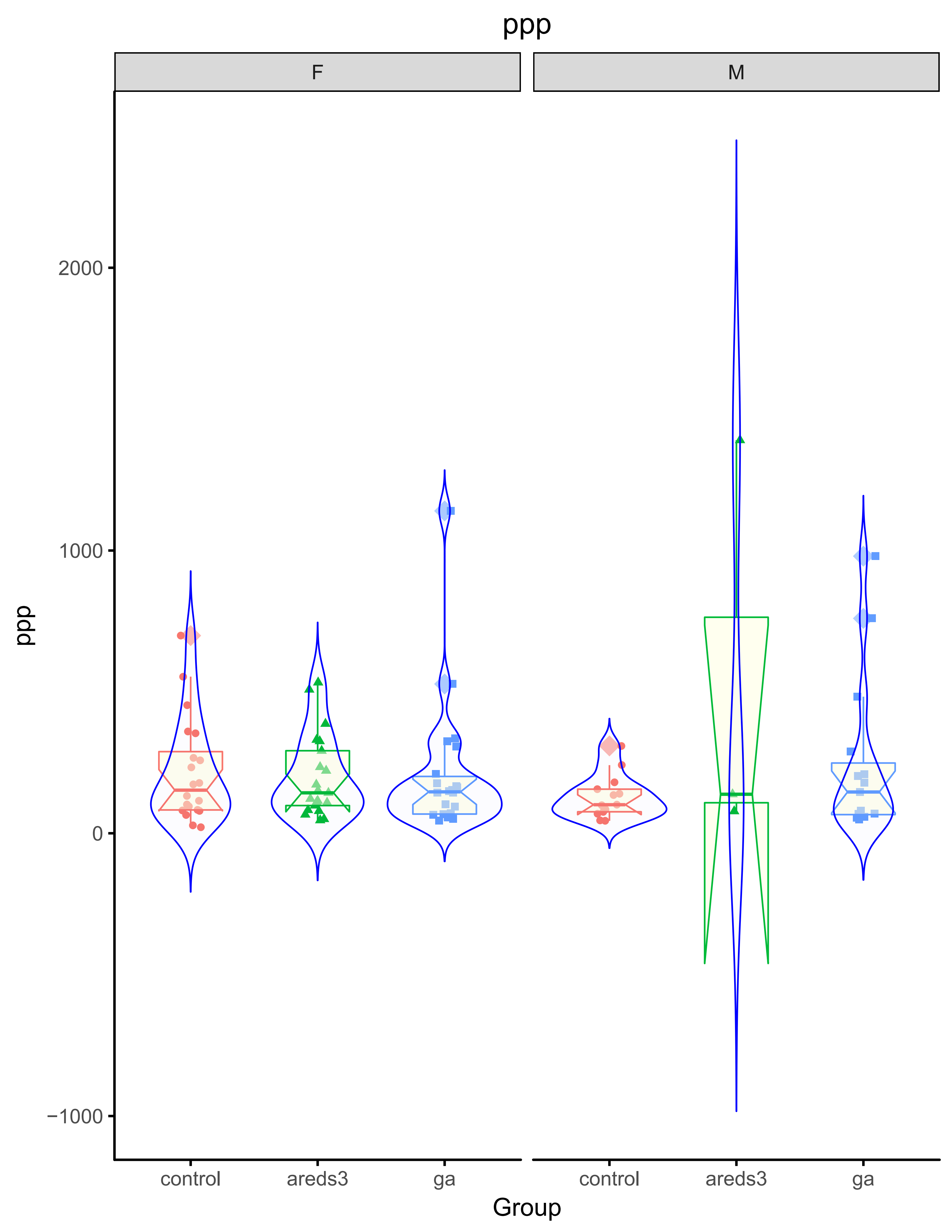

ppp
F
M
2000
1000
0
−1000
ppp
control
areds3
ga
control
areds3
ga
Group

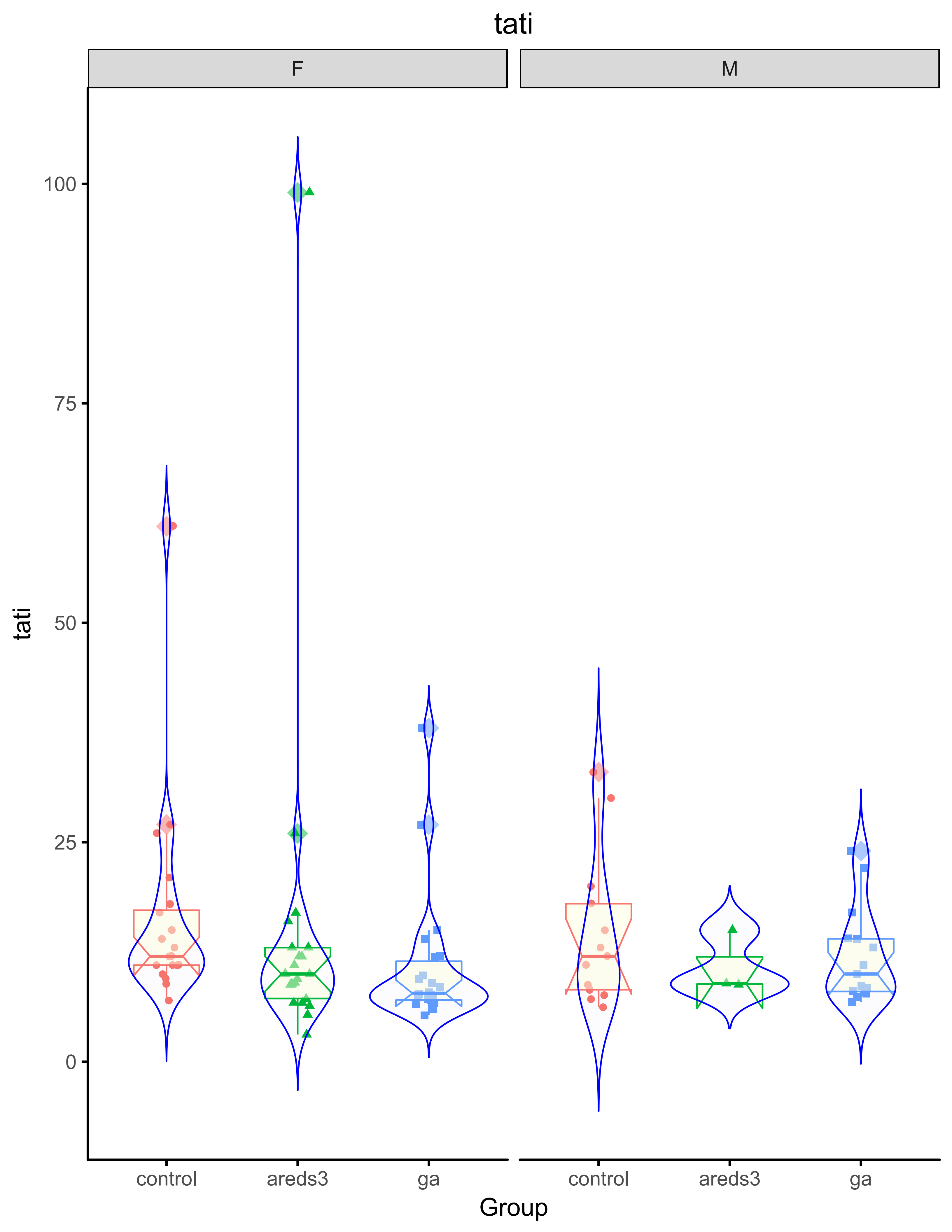

tati
F
M
100
75
50
25
0
tati
control
areds3
ga
control
areds3
ga
Group

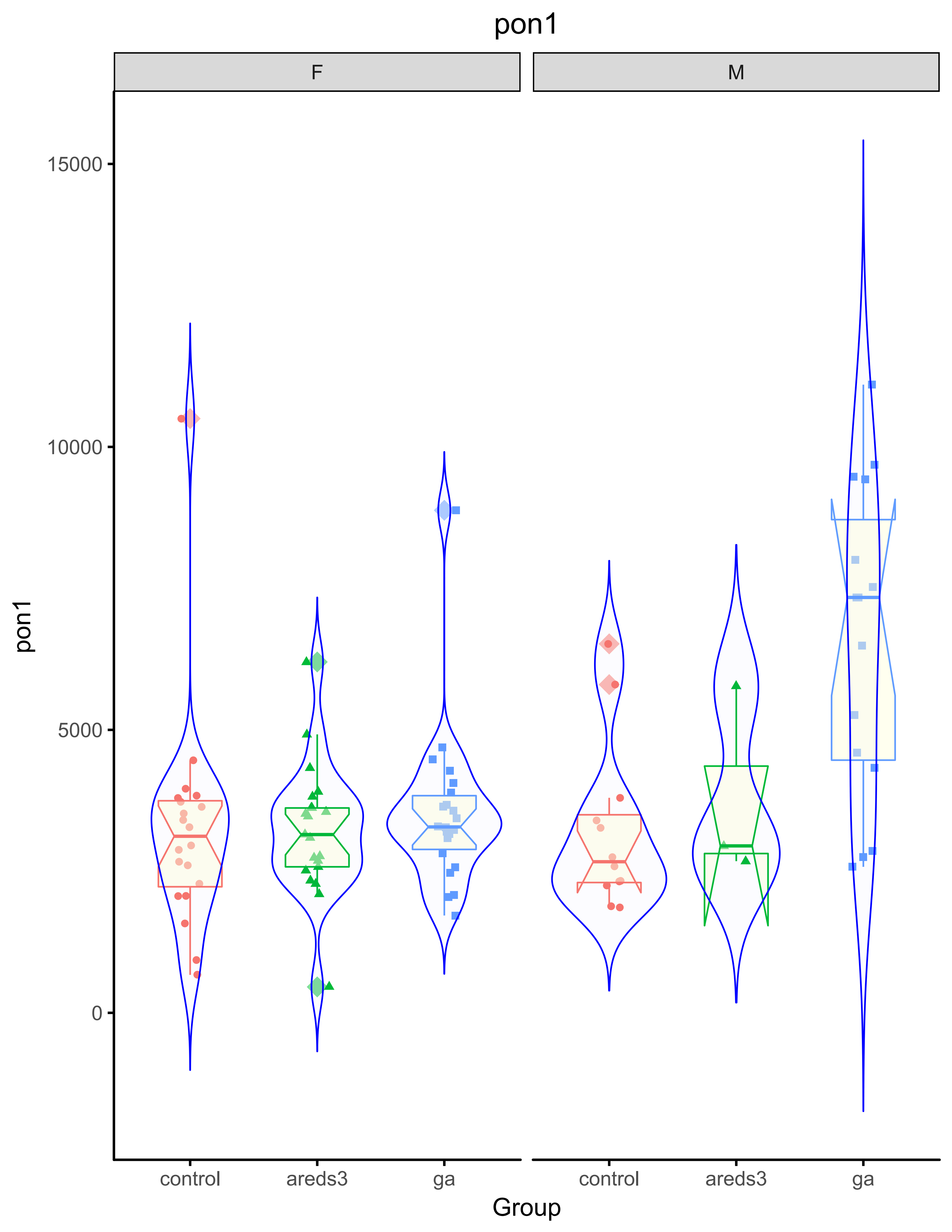

pon1
F
M
15000
10000
5000
0
pon1
control
areds3
ga
control
areds3
ga
Group

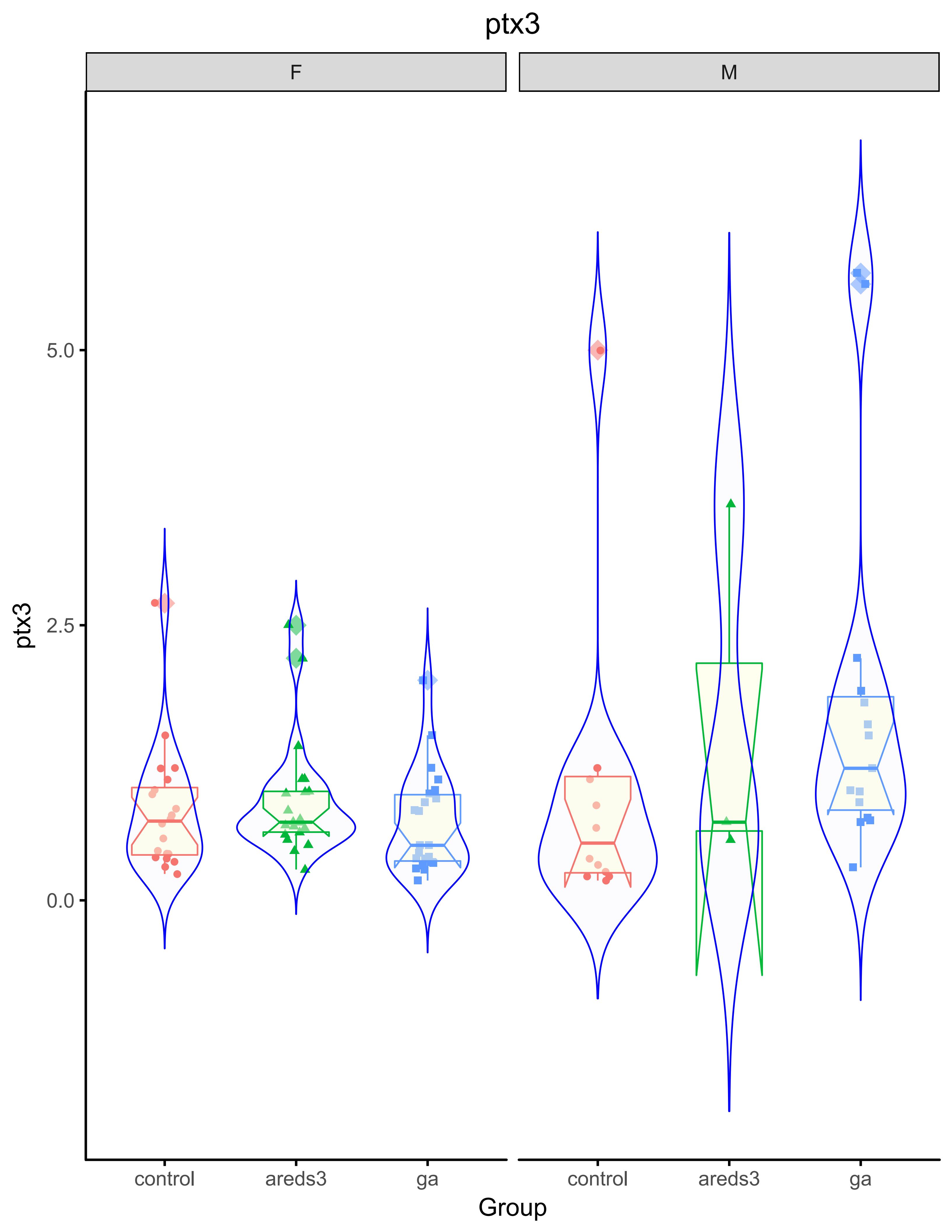
ptx3
F
M
5.0
2.5
0.0
ptx3
control
areds3
ga
control
areds3
ga
Group

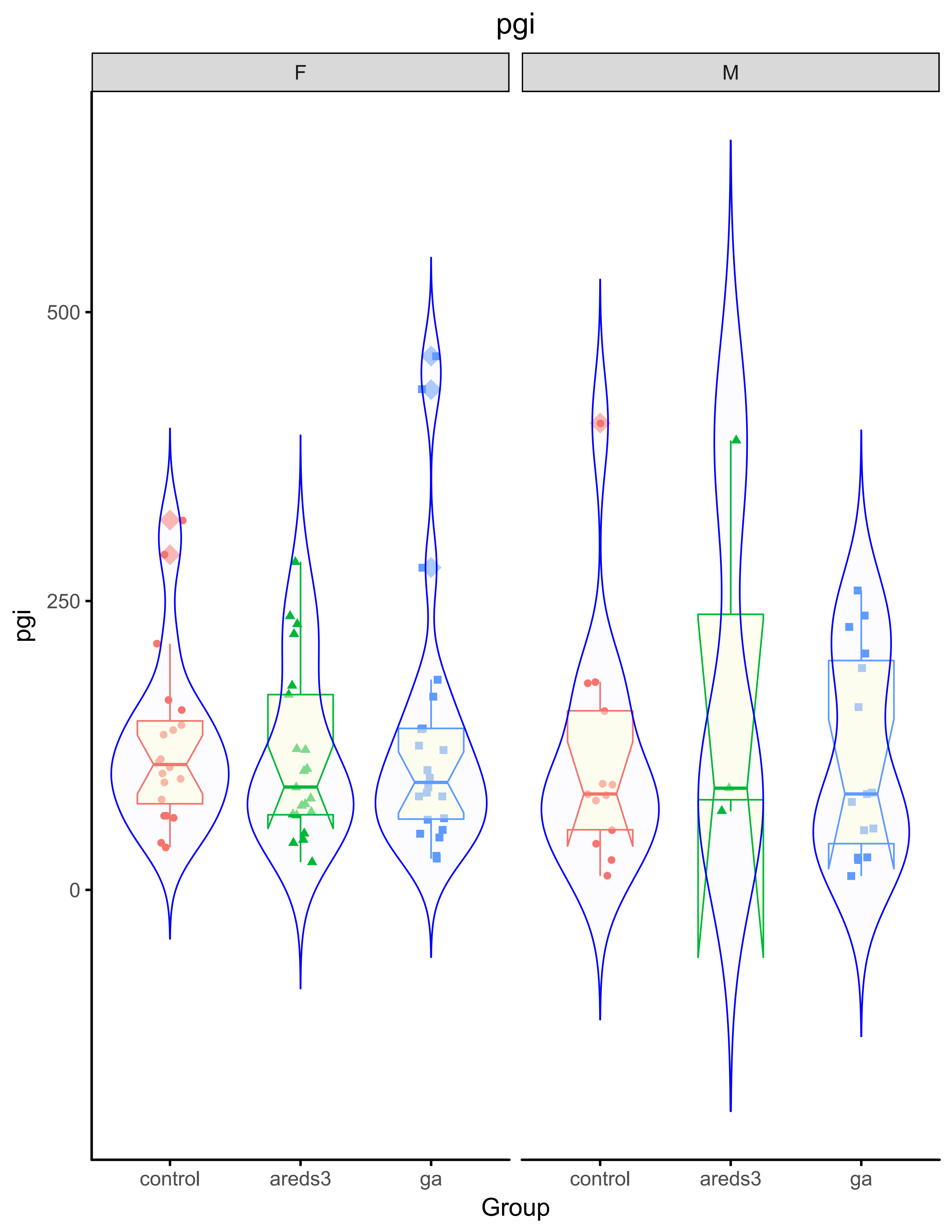
pgi
F
M
500
250
0
pgi
control
areds3
ga
control
areds3
ga
Group

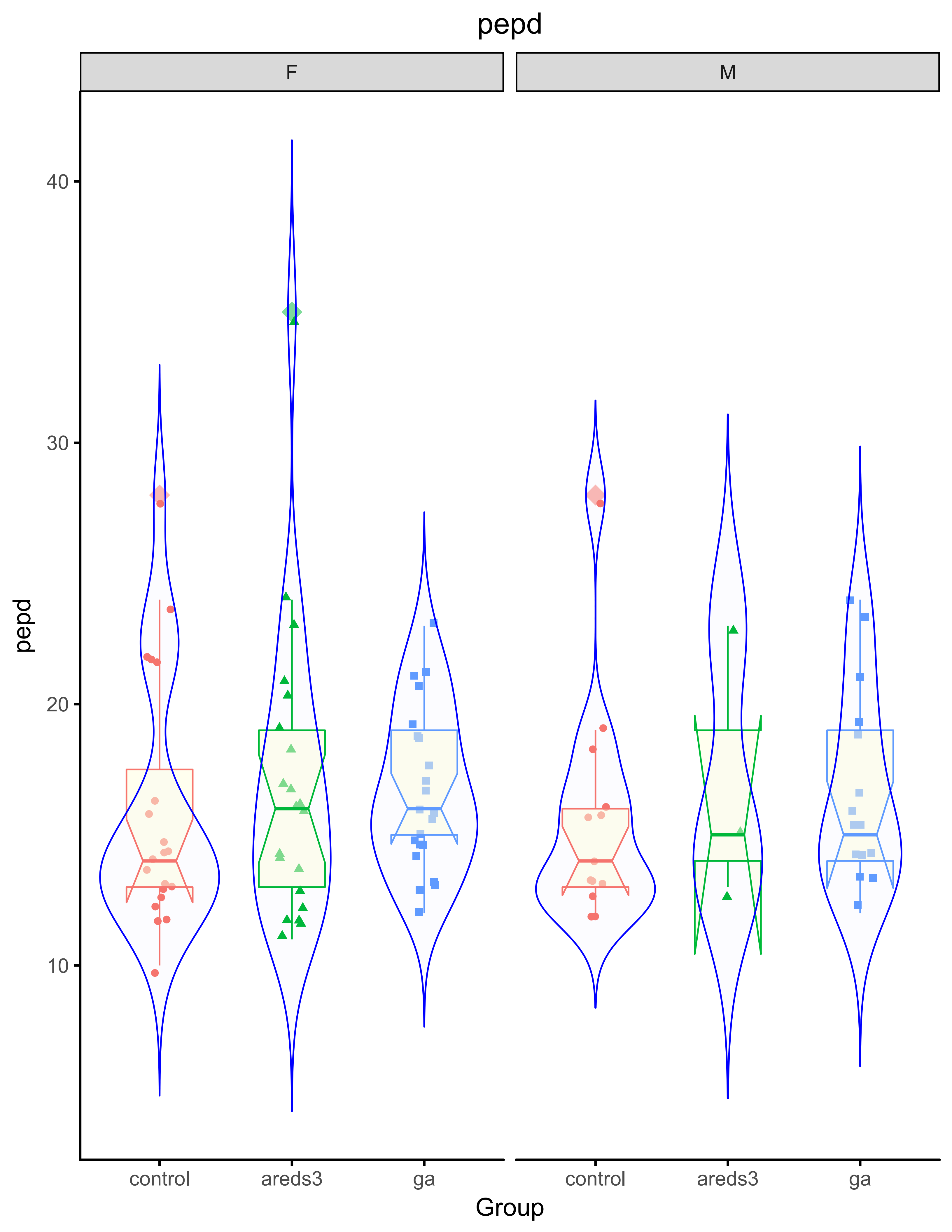
pepd
F
M
40
30
20
10
pepd
control
areds3
ga
control
areds3
ga
Group

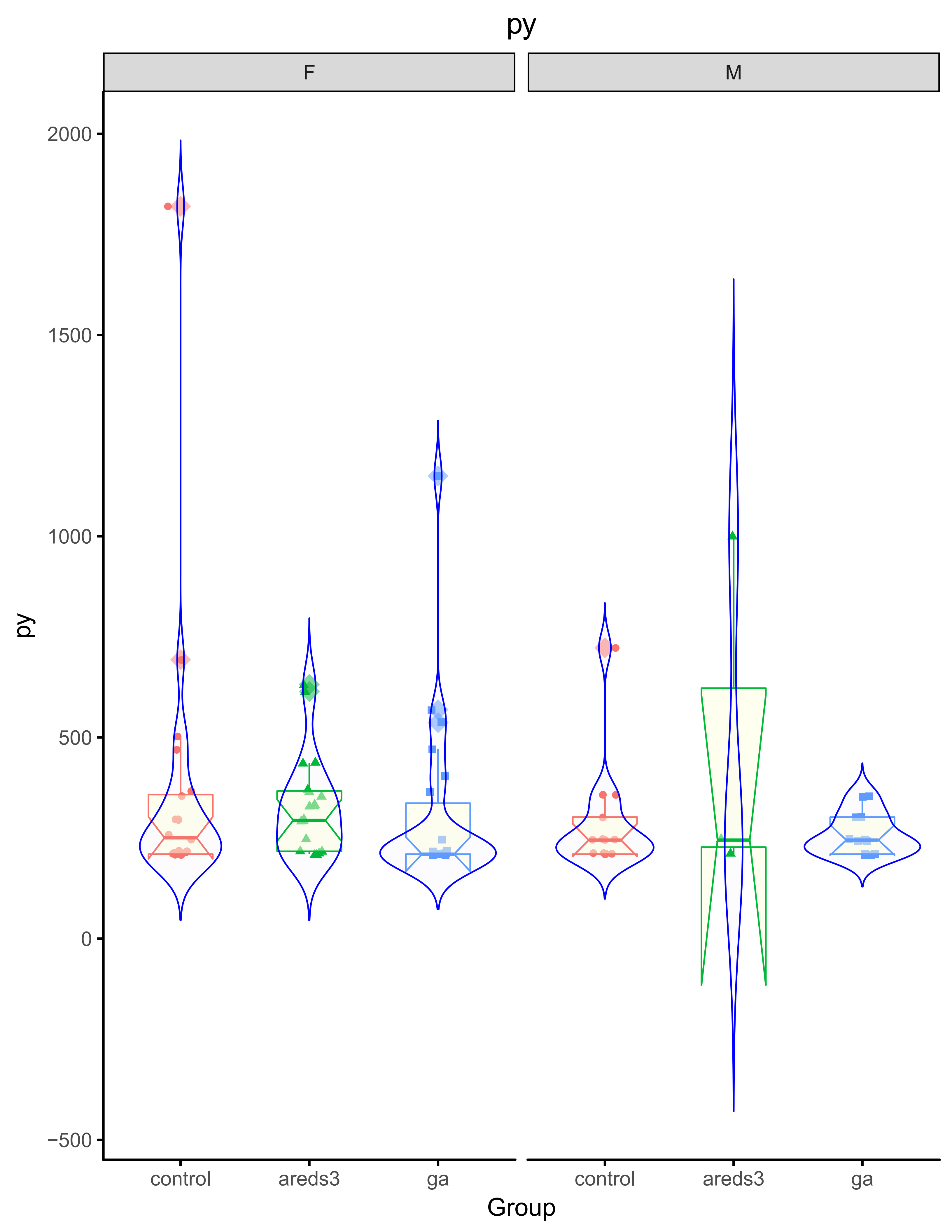

py
F
M
2000
1500
1000
500
0
−500
py
control
areds3
ga
control
areds3
ga
Group

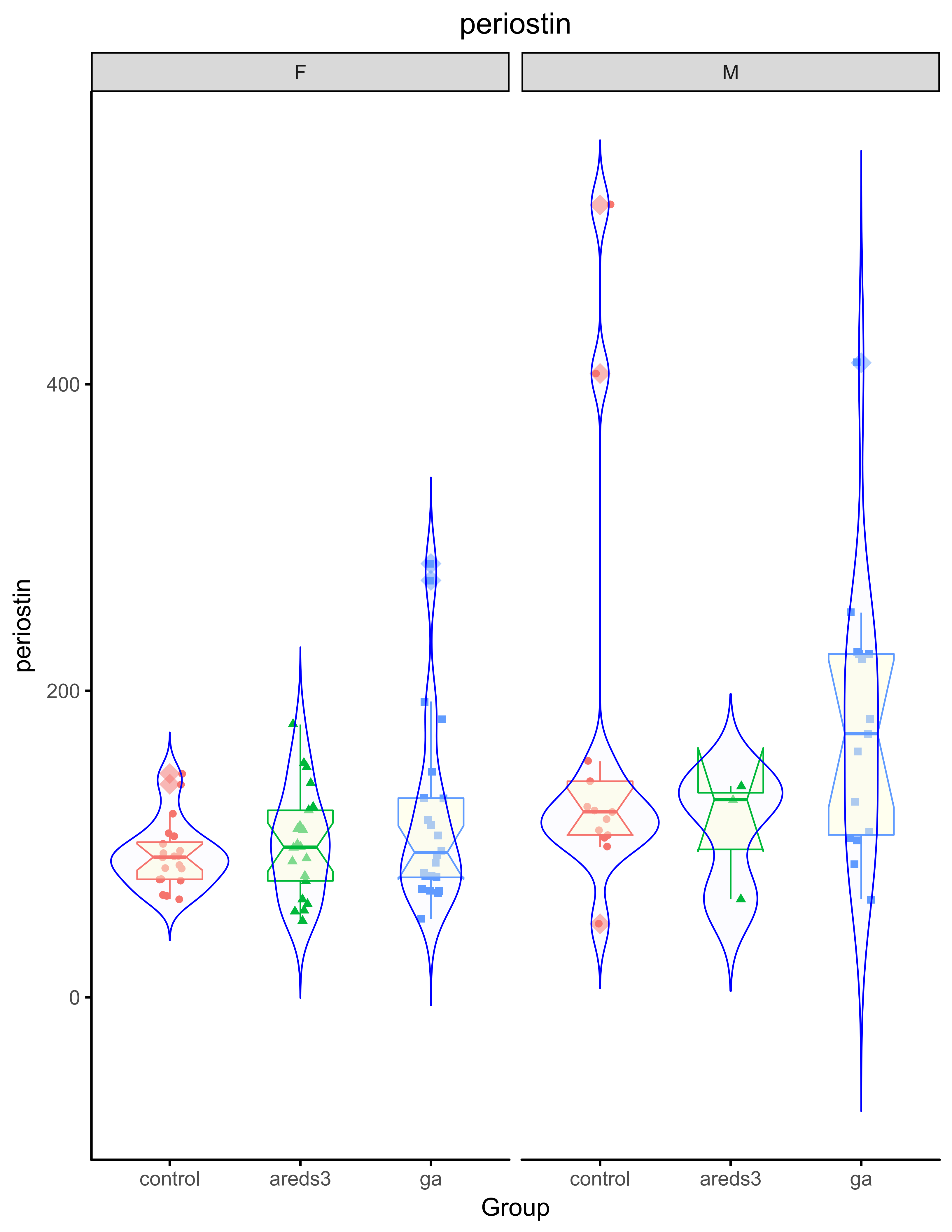

periostin
F
M
400
200
0
periostin
control
areds3
ga
control
areds3
ga
Group

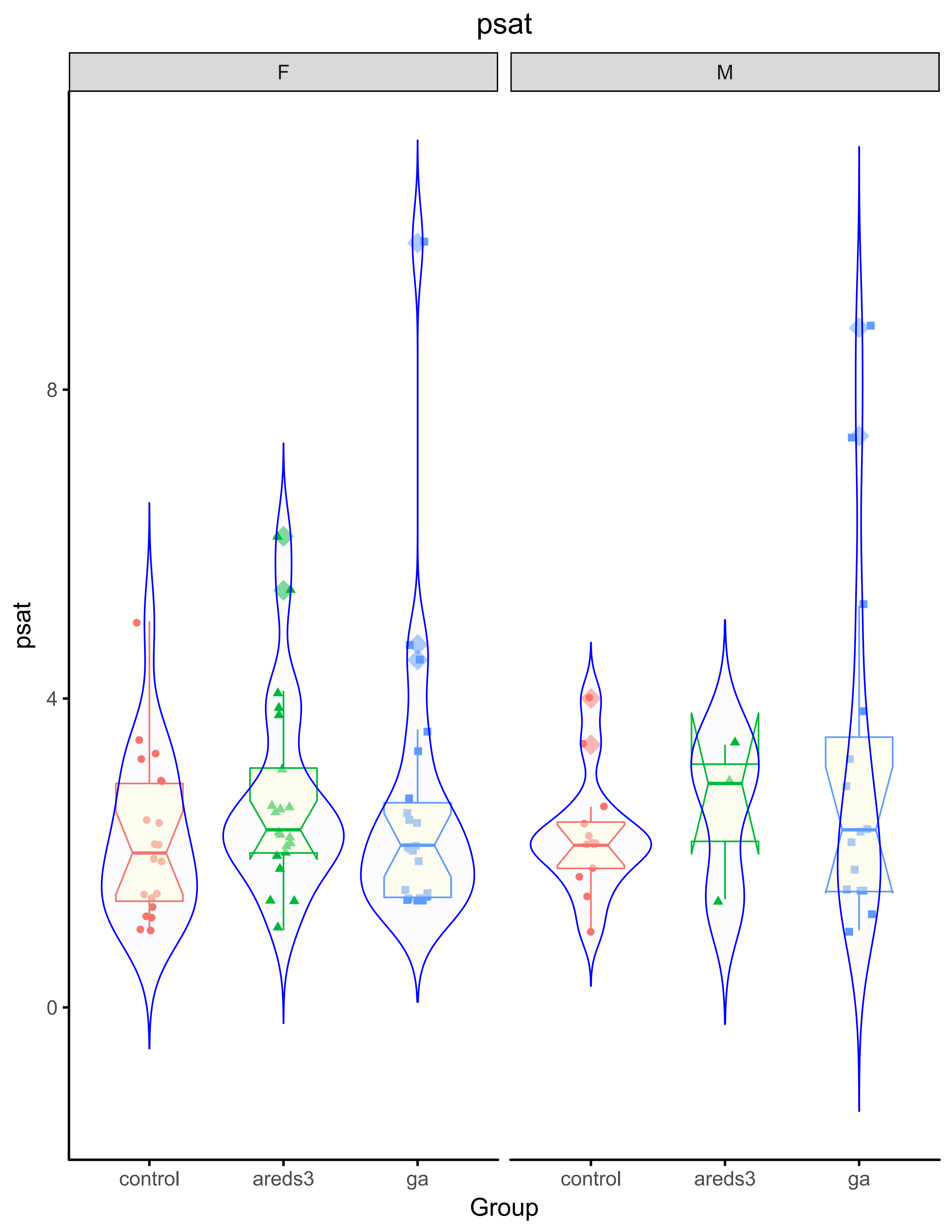
psat
F
M
8
4
0
psat
control
areds3
ga
control
areds3
ga
Group

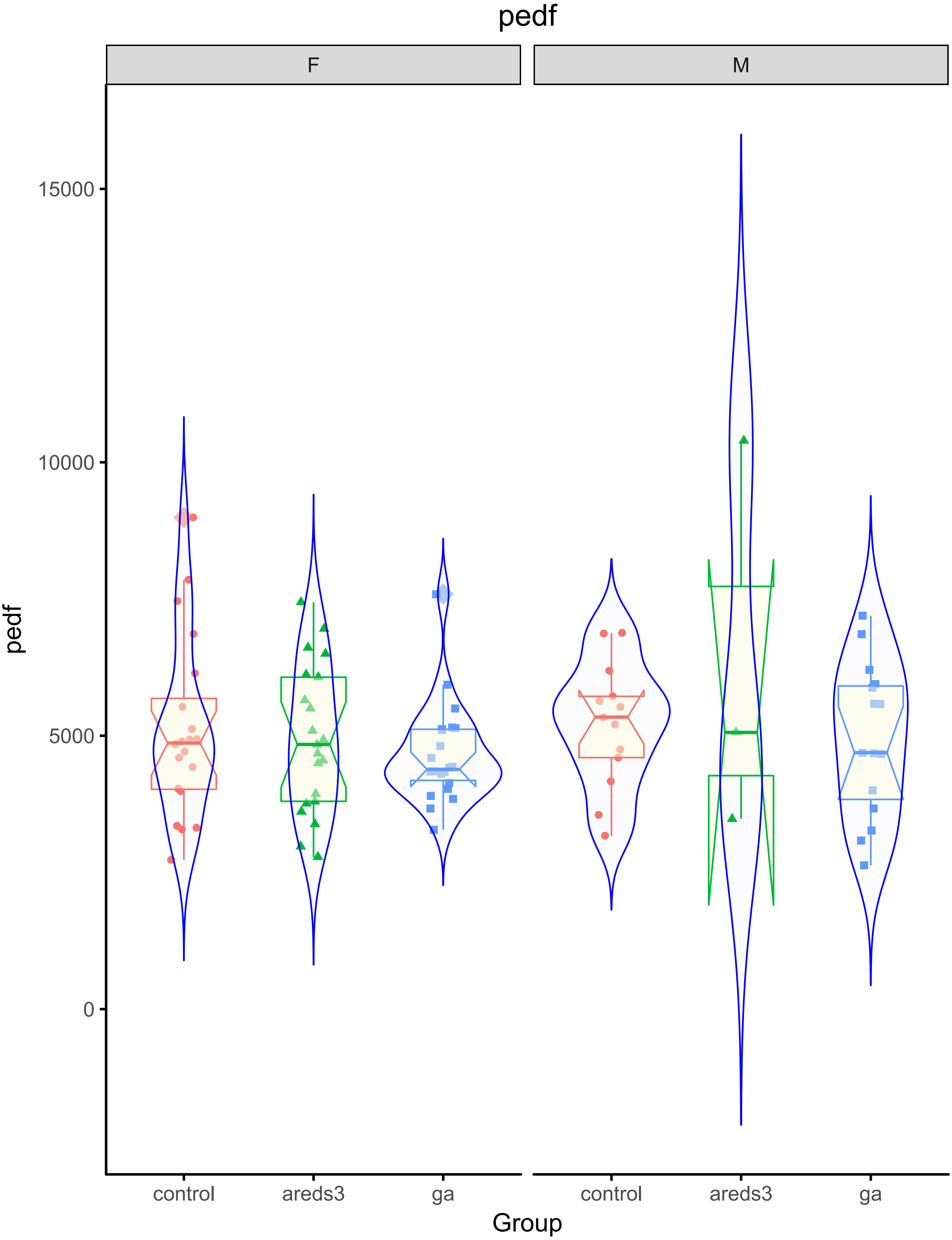
pedf
F
M
15000
10000
5000
0
pedf
control
areds3
ga
control
areds3
ga
Group

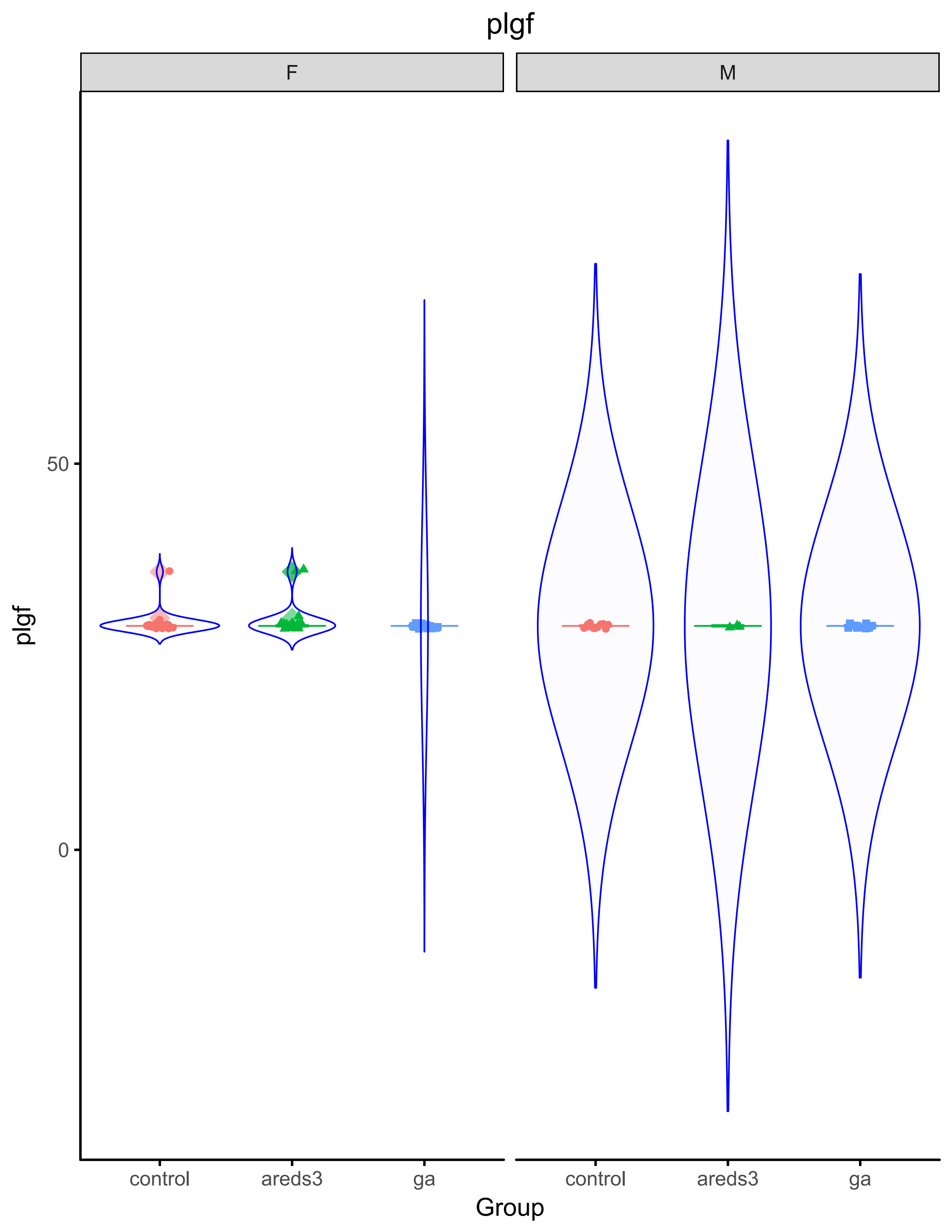

plgf
F
M
50
0
plgf
control
areds3
ga
control
areds3
ga
Group

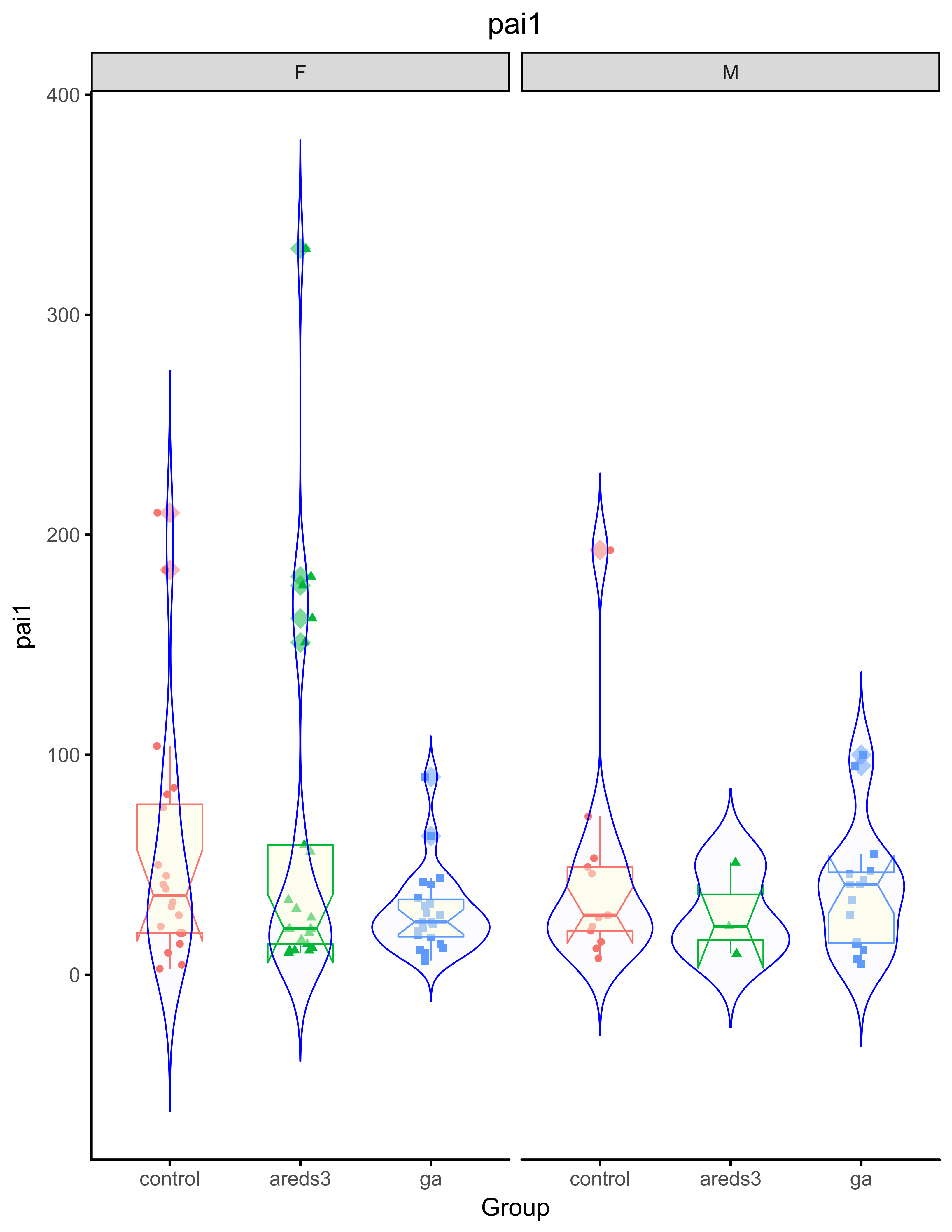
pai1
F
M
400
300
200
100
0
pai1
control
areds3
ga
control
areds3
ga
Group

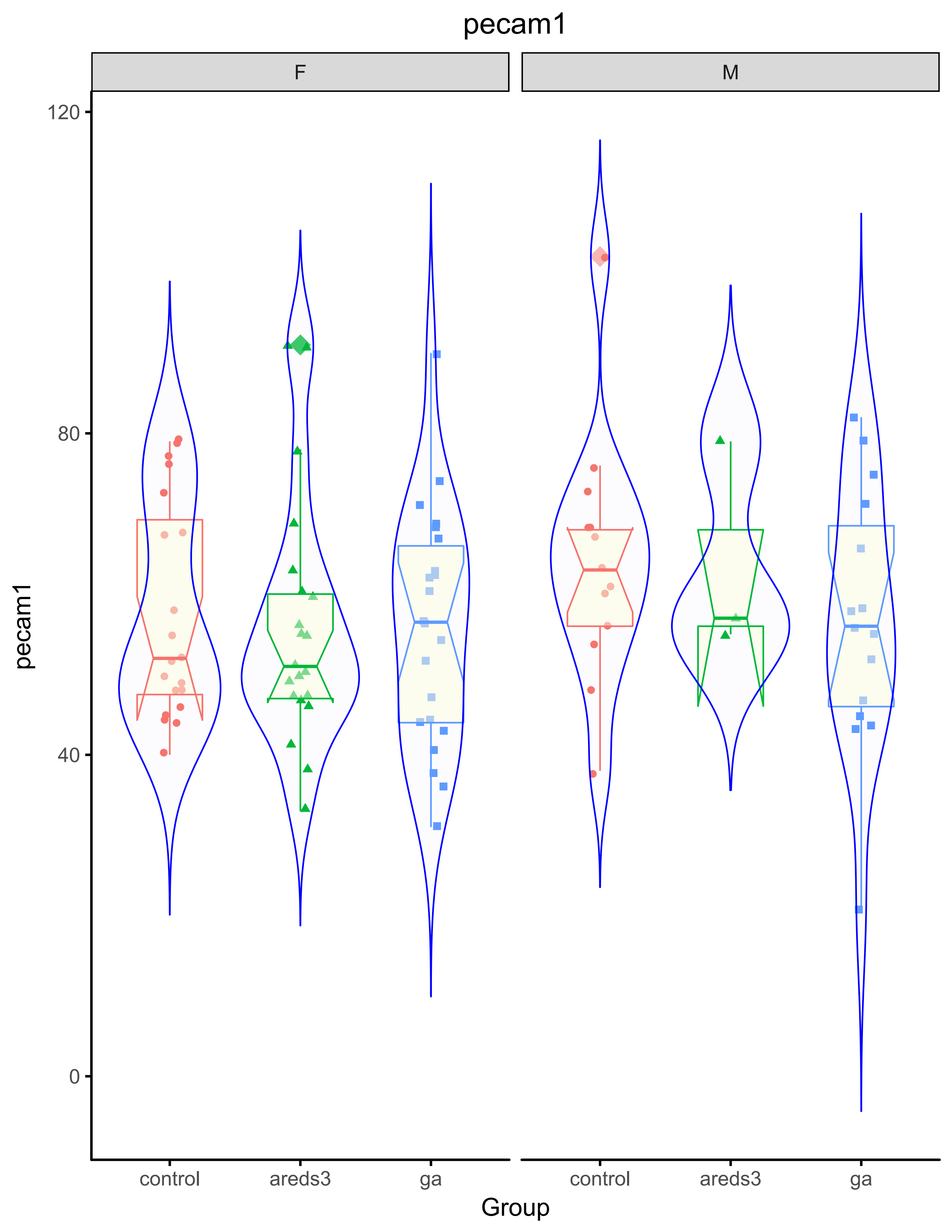

pecam1
F
M
120
80
40
0
pecam1
control
areds3
ga
control
areds3
ga
Group

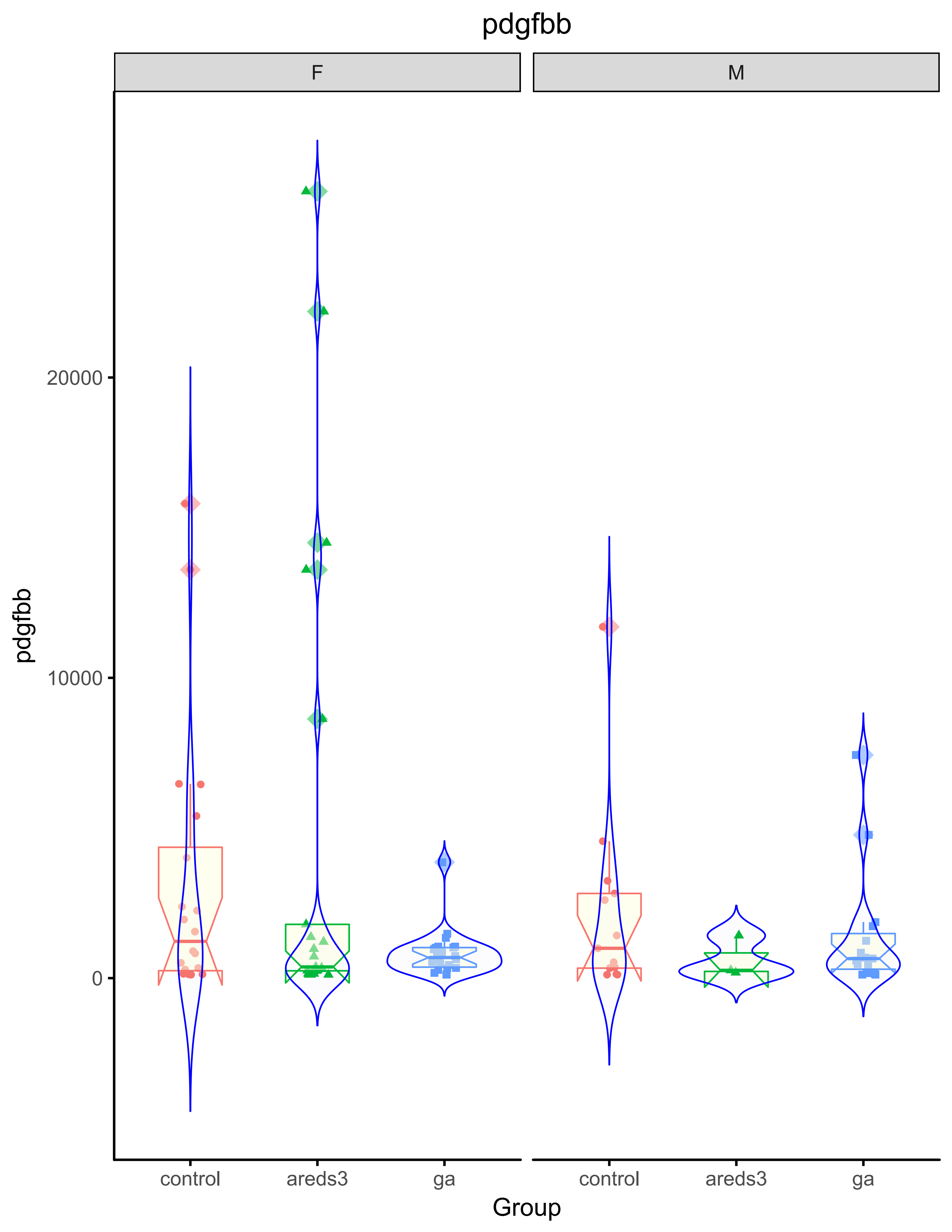

pdgfbb
F
M
pdgfbb
20000
10000
0
control
areds3
ga
control
areds3
ga
Group

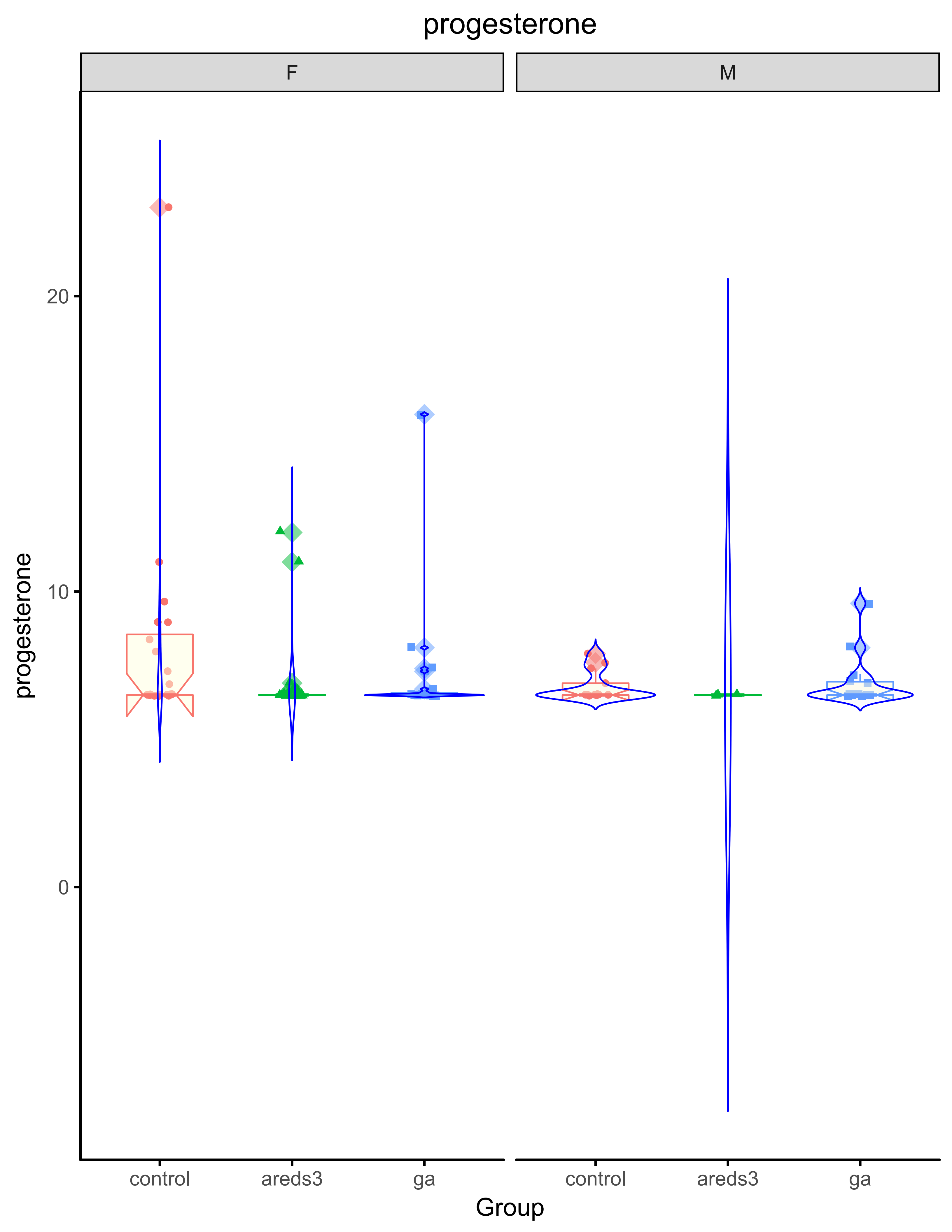
progesterone
F
M
20
10
0
progesterone
control
areds3
ga
control
areds3
ga
Group

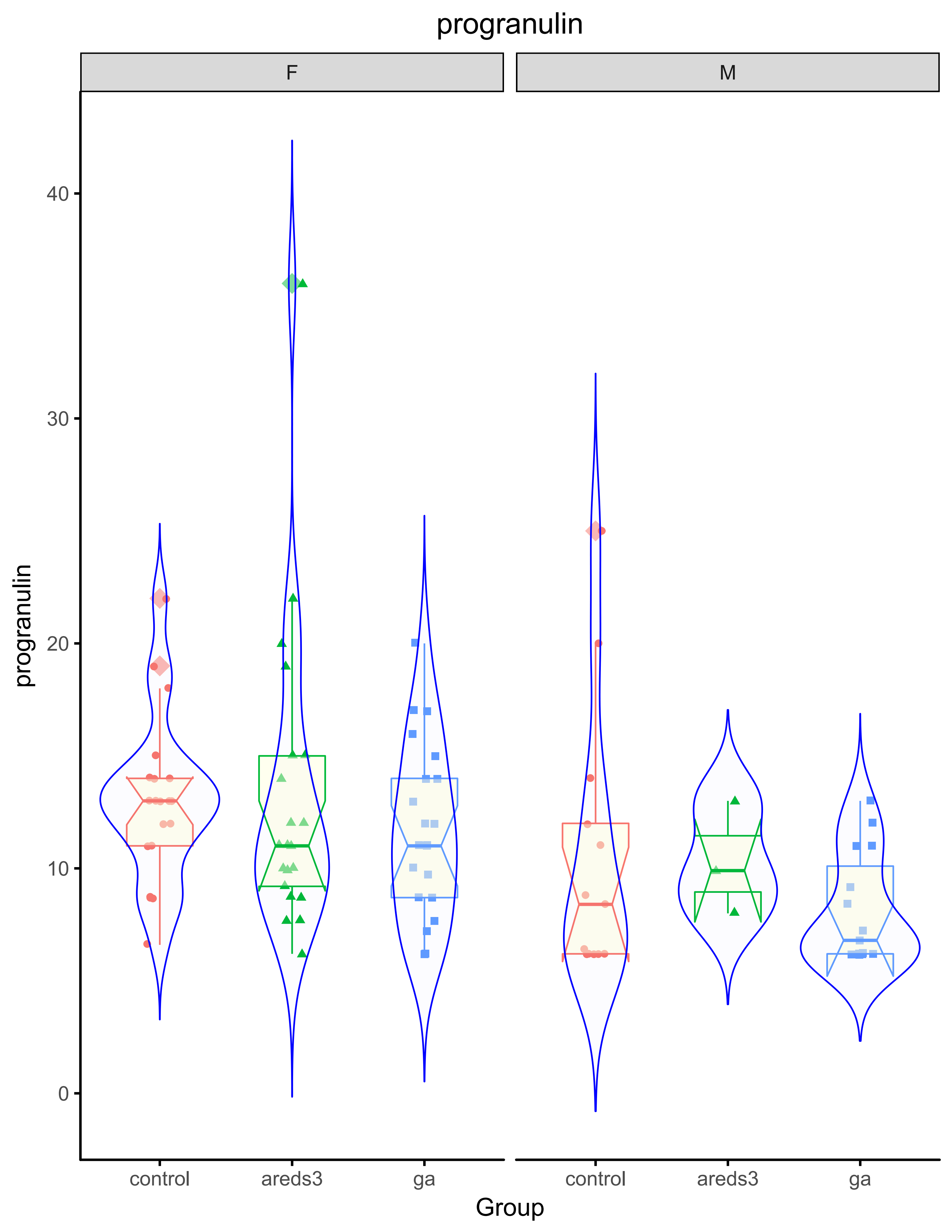

progranulin
F
M
40
30
20
10
0
progranulin
control
areds3
ga
control
areds3
ga
Group

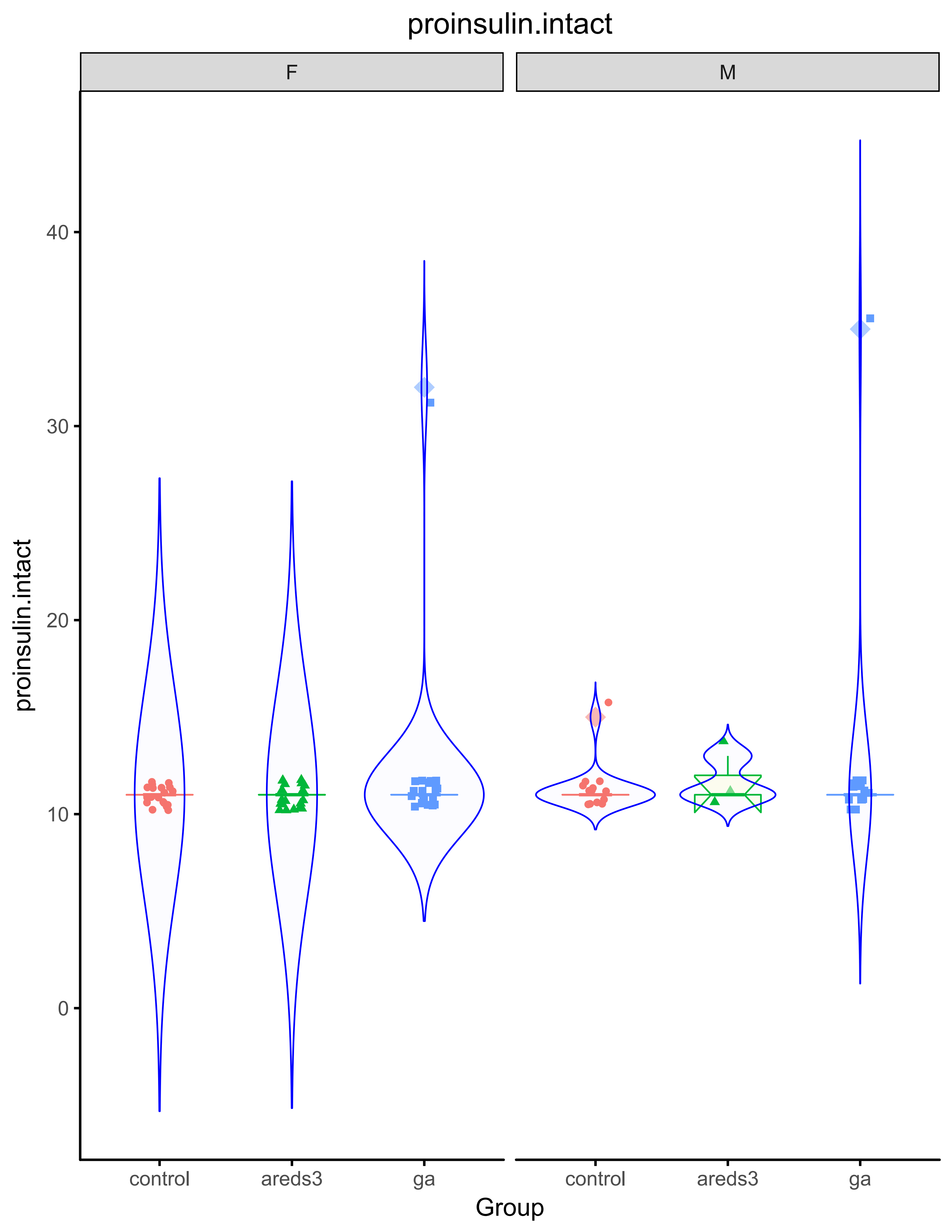

proinsulin.intact
F
M
40
30
20
10
0
proinsulin.intact
control
areds3
ga
control
areds3
ga
Group

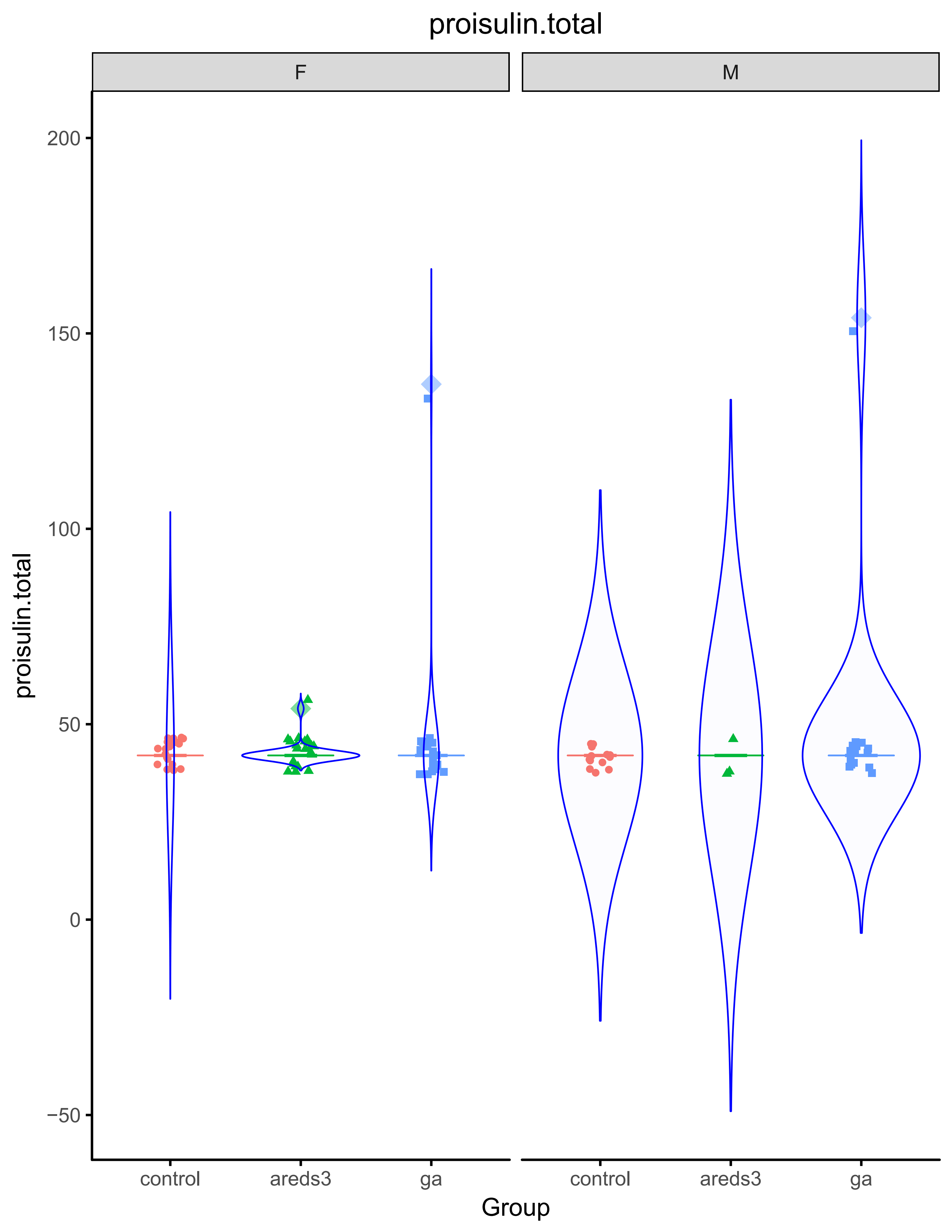

proisulin.total
F
M
200
150
100
50
0
−50
proisulin.total
control
areds3
ga
control
areds3
ga
Group

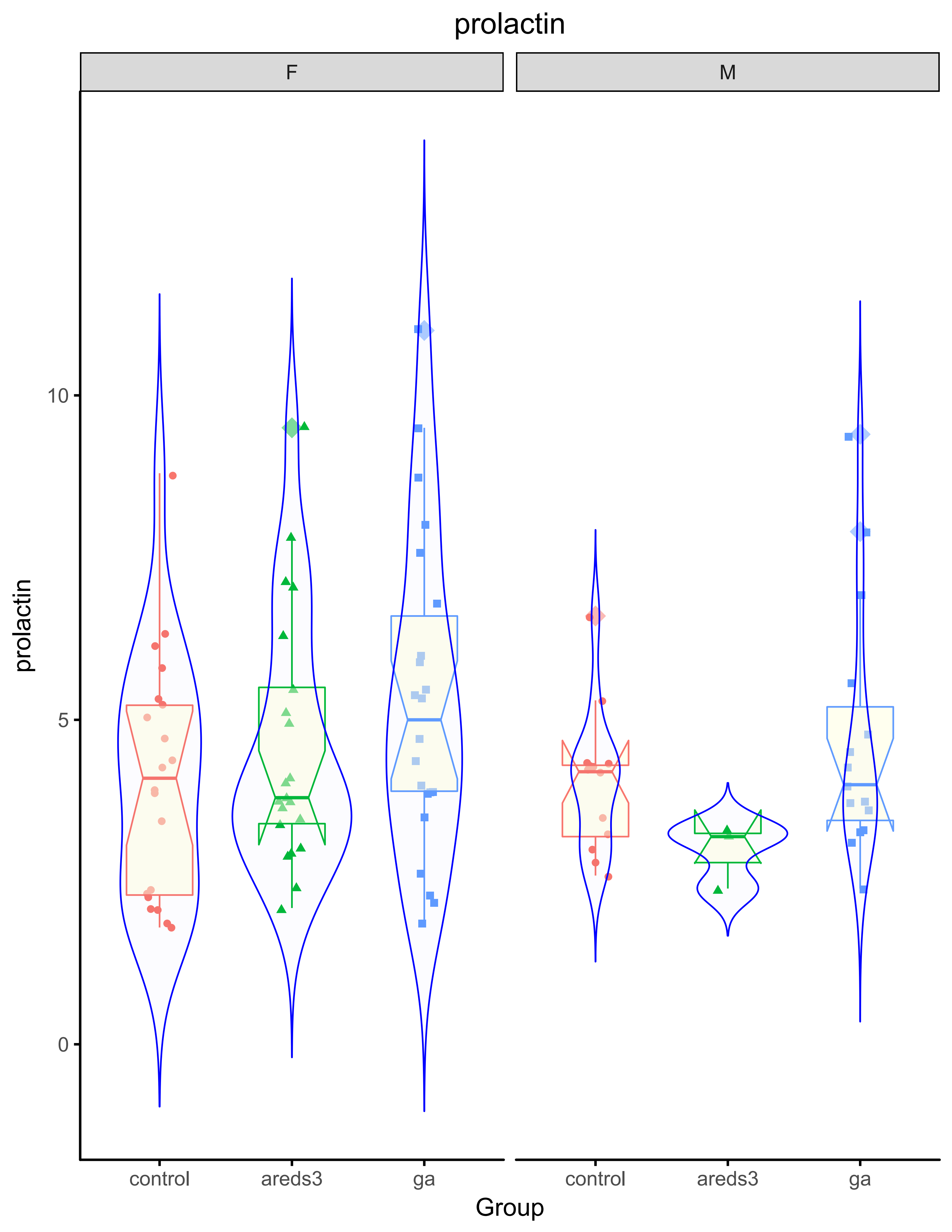

prolactin
F
M
10
5
0
prolactin
control
areds3
ga
control
areds3
ga
Group

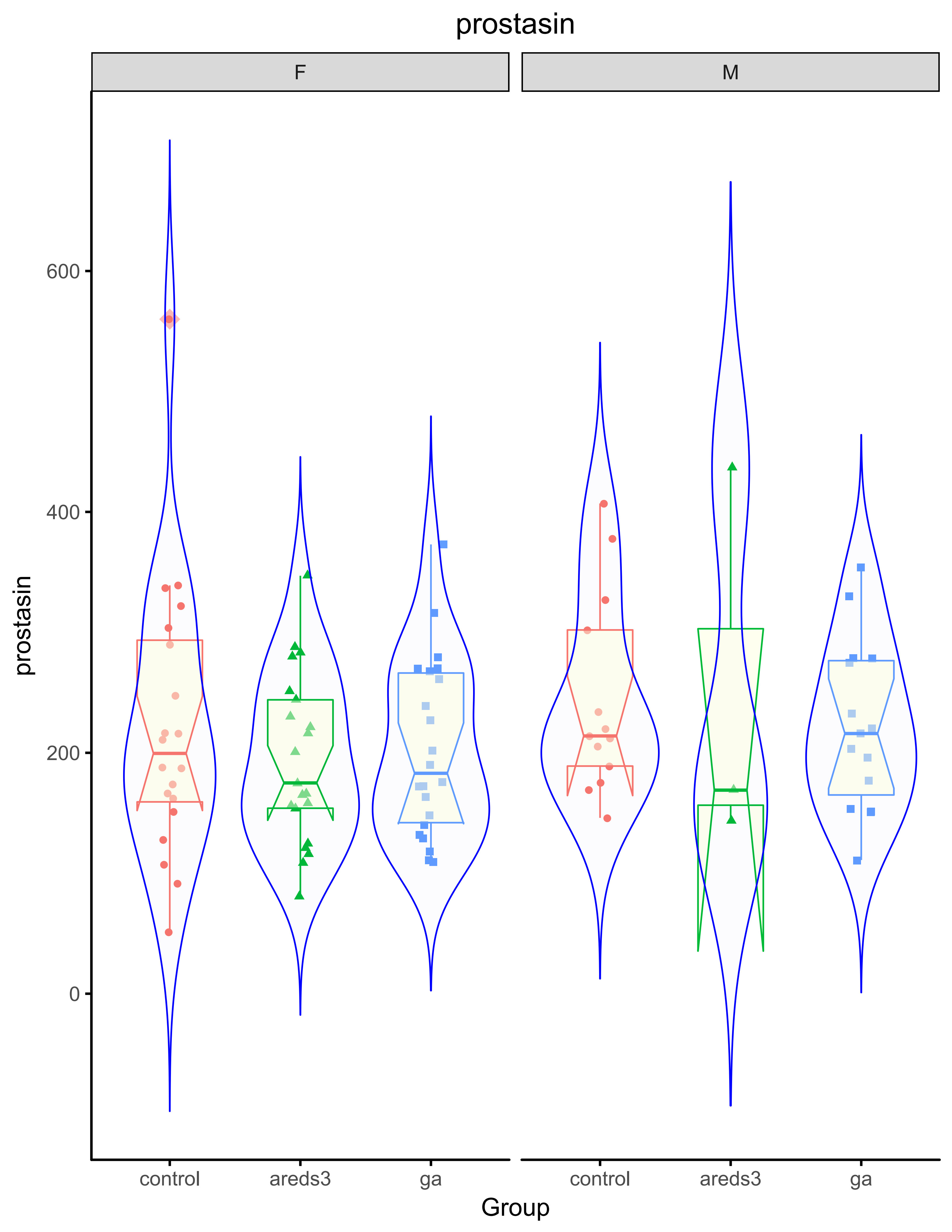

prostasin
F
M
600
400
200
0
prostasin
control
areds3
ga
control
areds3
ga
Group

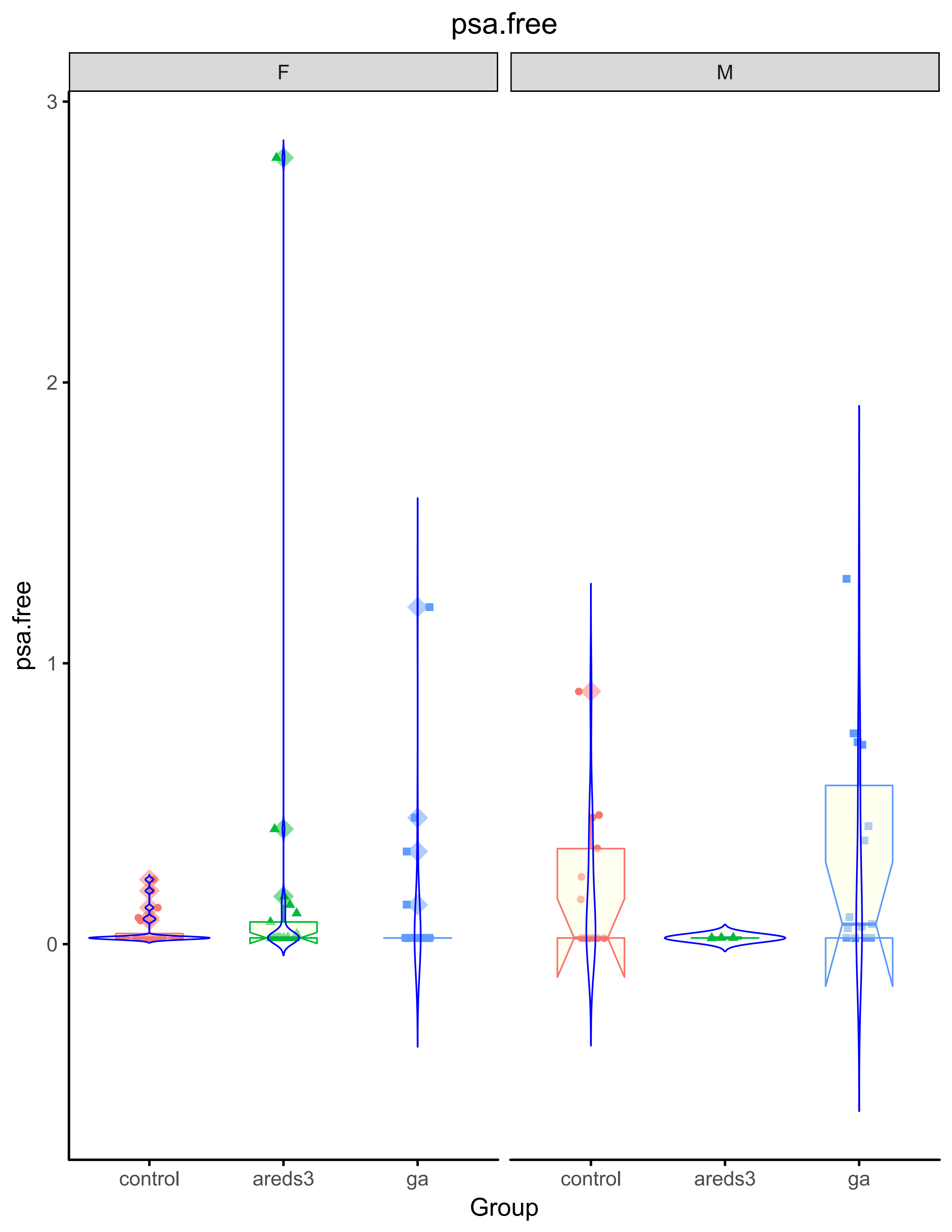

psa.free
F
M
3
2
1
0
psa.free
control
areds3
ga
control
areds3
ga
Group

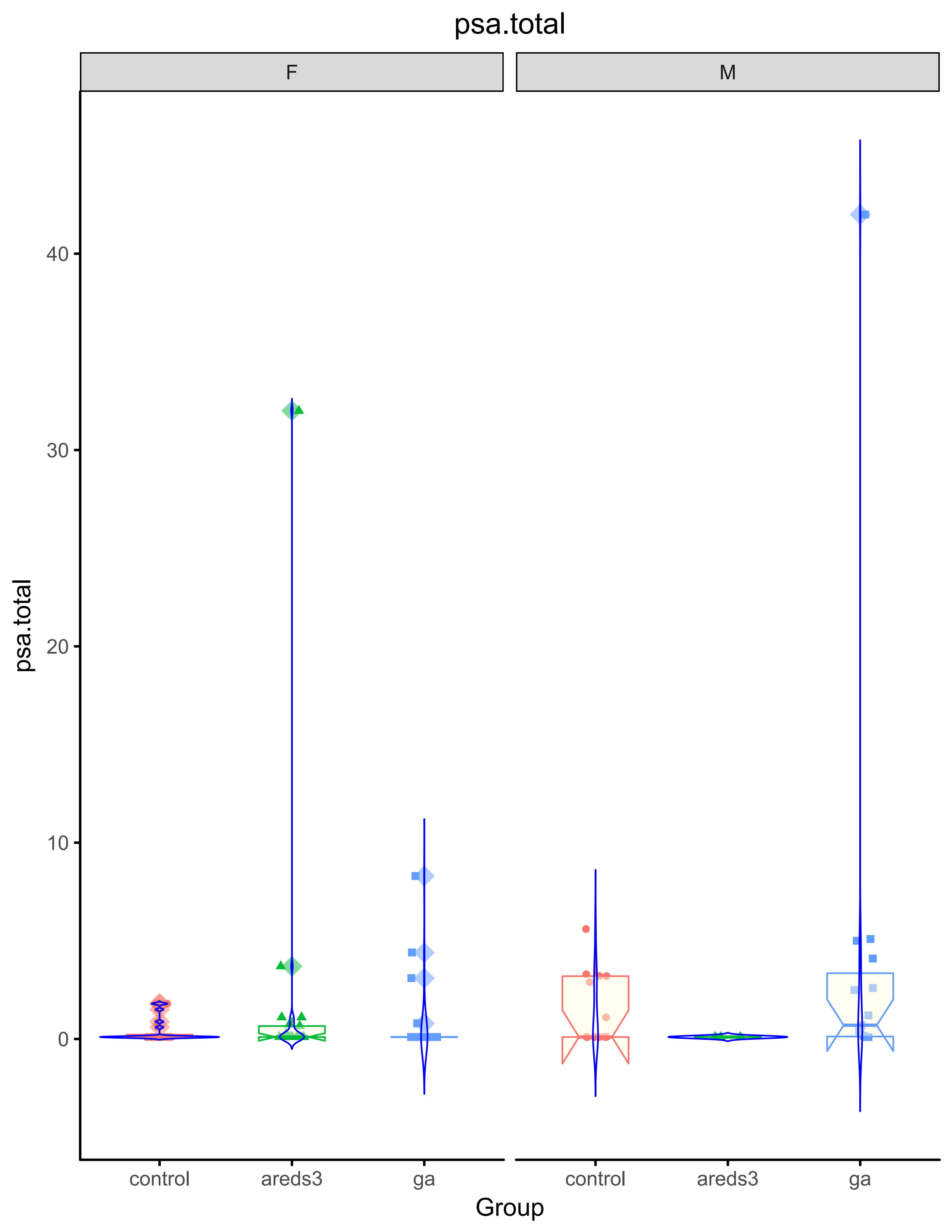
psa.total
F
M
psa.total
40
30
20
10
0
control
areds3
ga
control
areds3
ga
Group

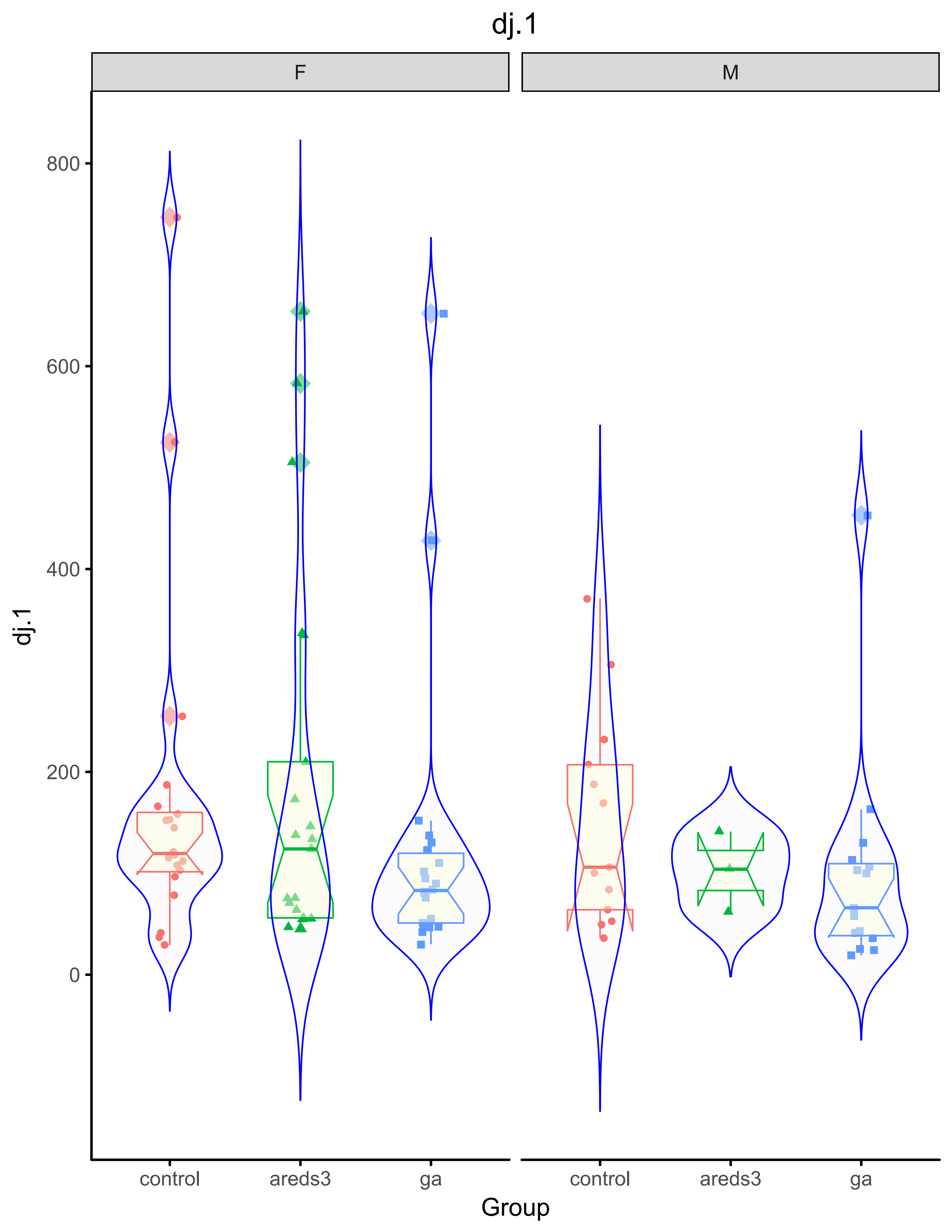
dj.1
F
M
800
600
400
200
0
dj.1
control
areds3
ga
control
areds3
ga
Group

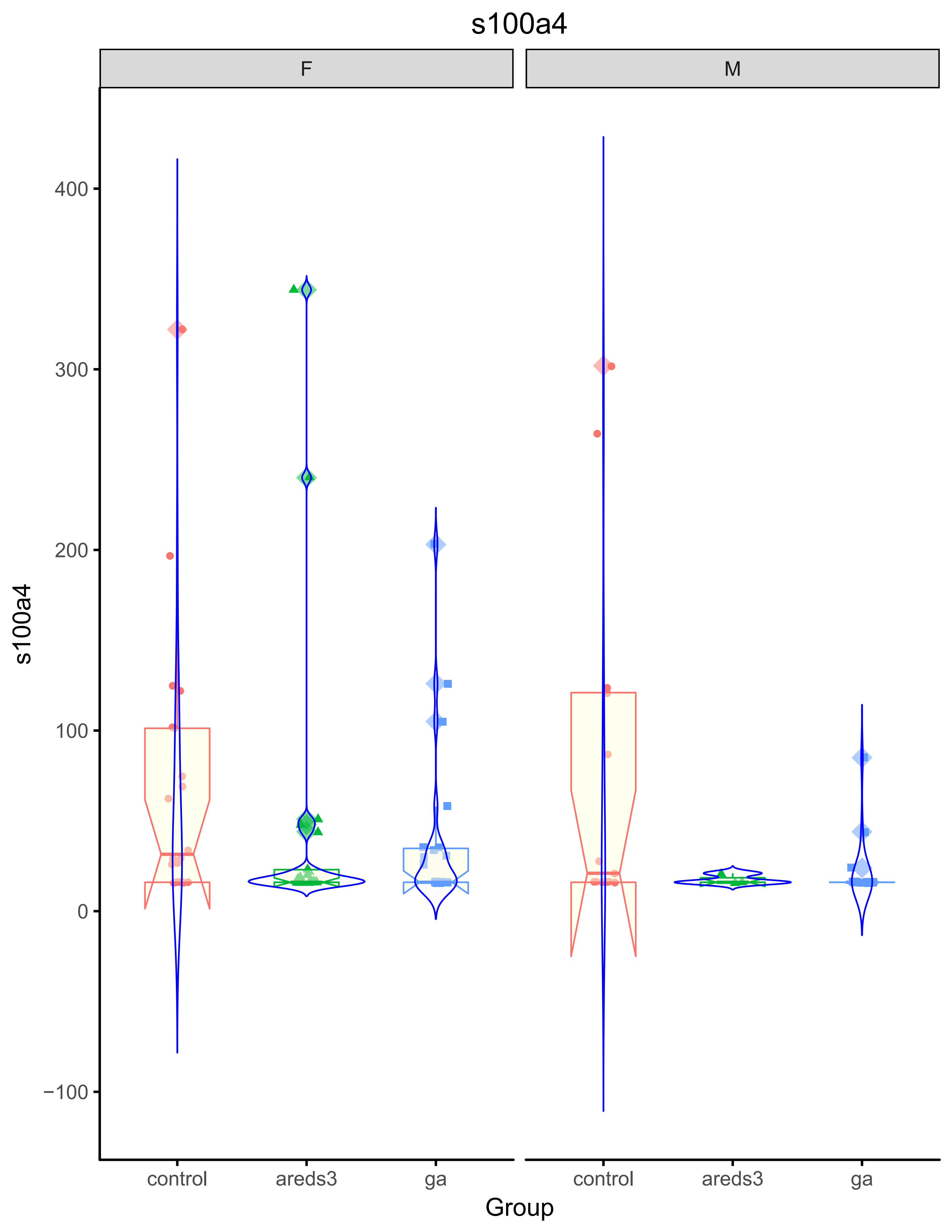
s100a4
F
M
400
300
200
100
0
−100
s100a4
control
areds3
ga
control
areds3
ga
Group

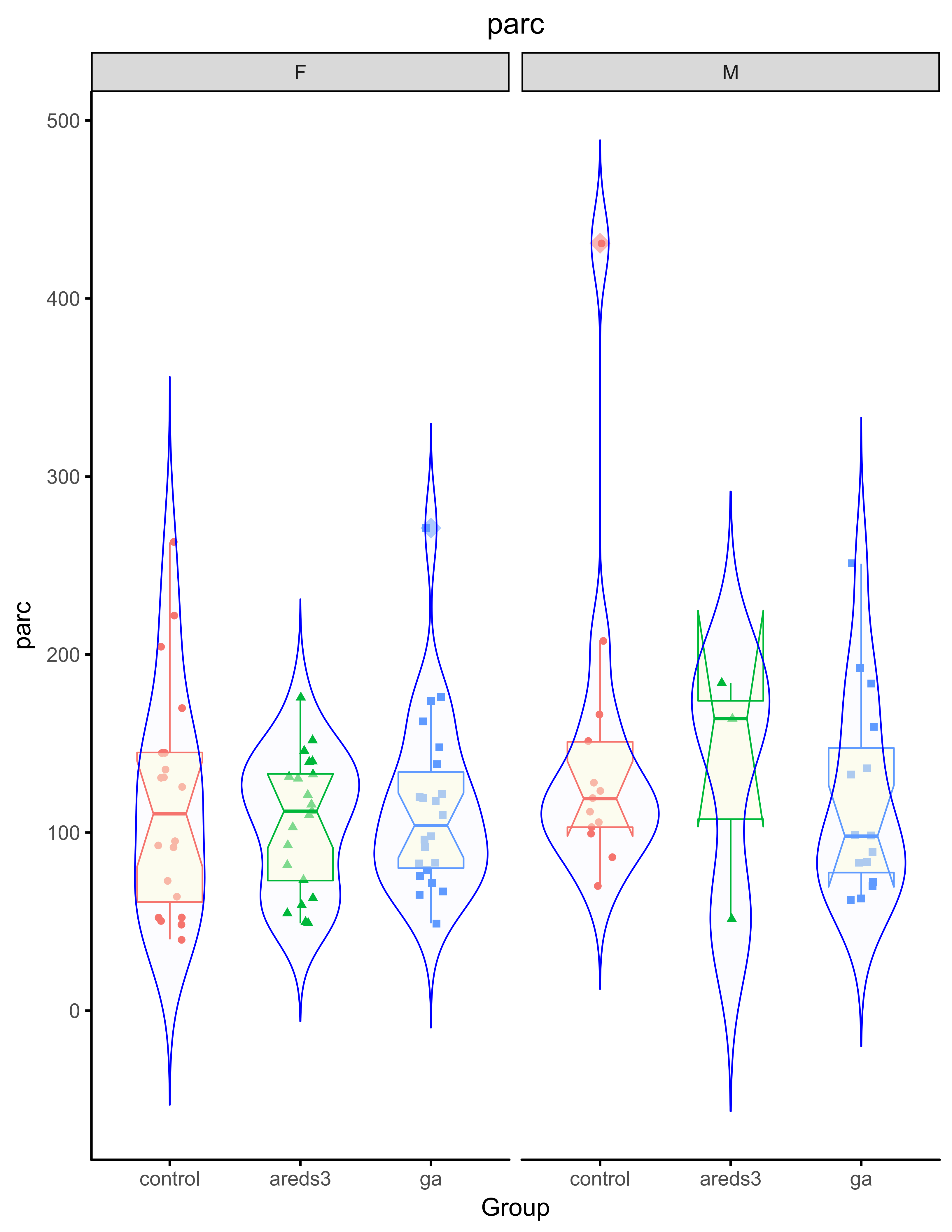

parc
F
M
500
400
300
200
100
0
parc
control
areds3
ga
control
areds3
ga
Group

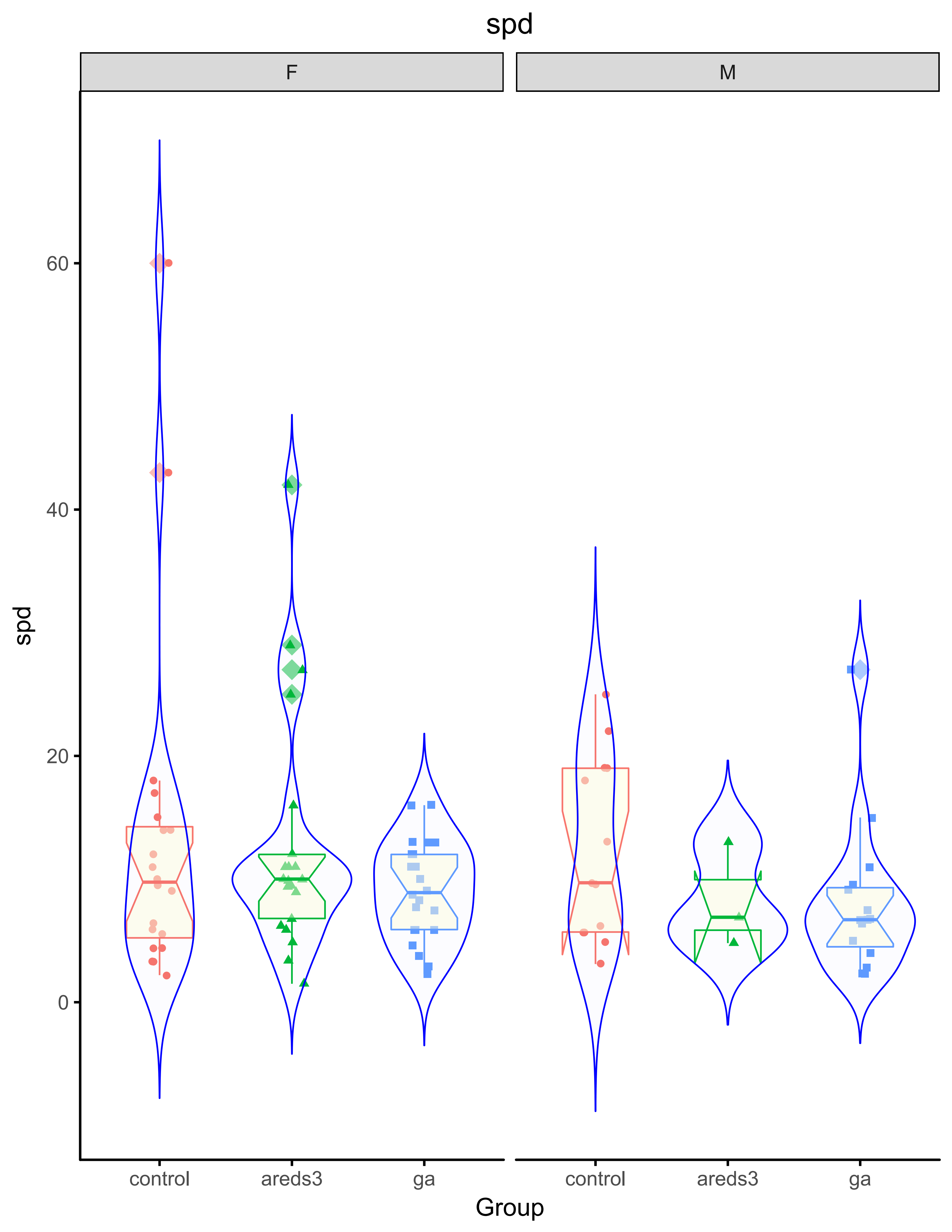

spd
F
M
60
40
20
0
spd
control
areds3
ga
control
areds3
ga
Group

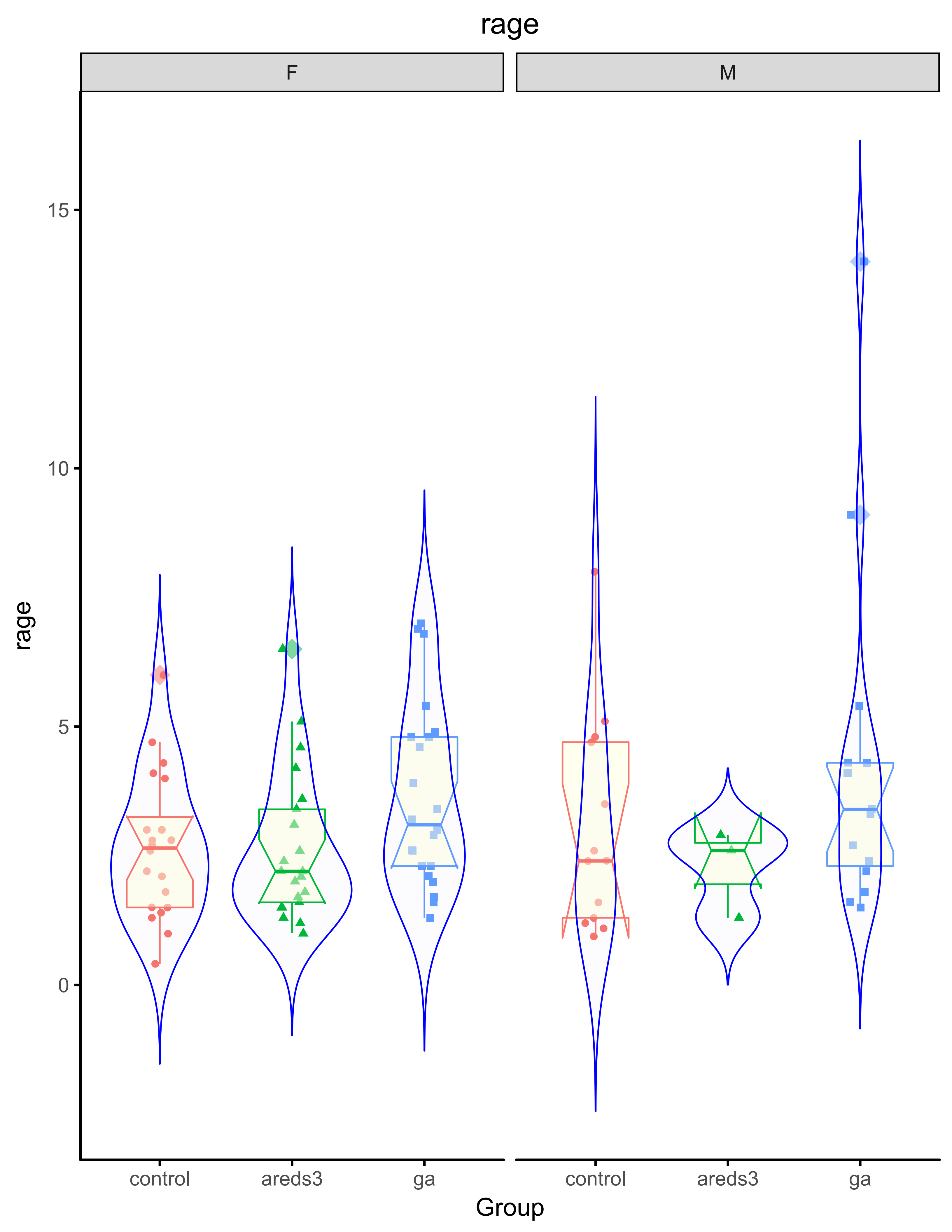

rage
F
M
15
10
5
0
rage
control
areds3
ga
control
areds3
ga
Group

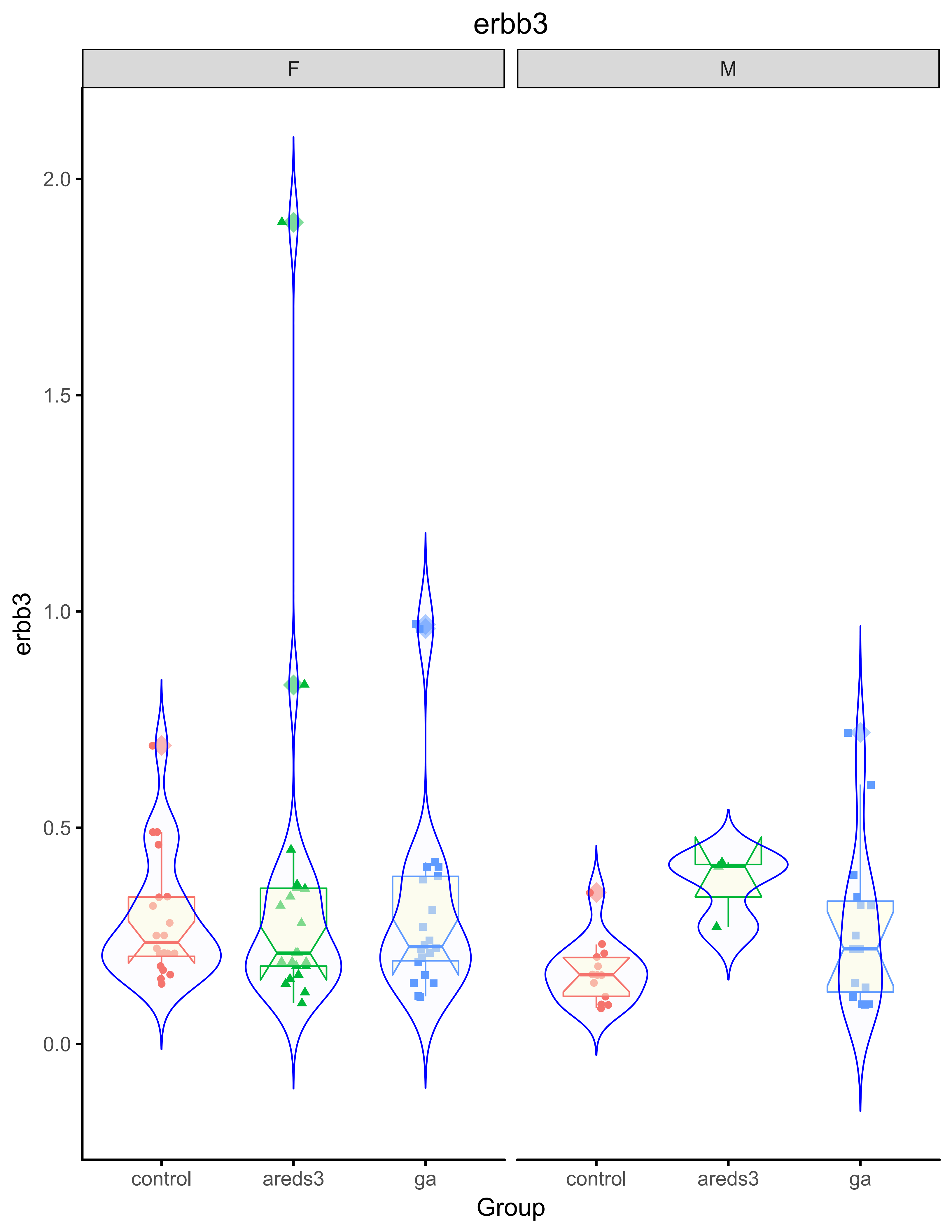

erbb3
F
M
2.0
1.5
1.0
0.5
0.0
erbb3
control
areds3
ga
control
areds3
ga
Group

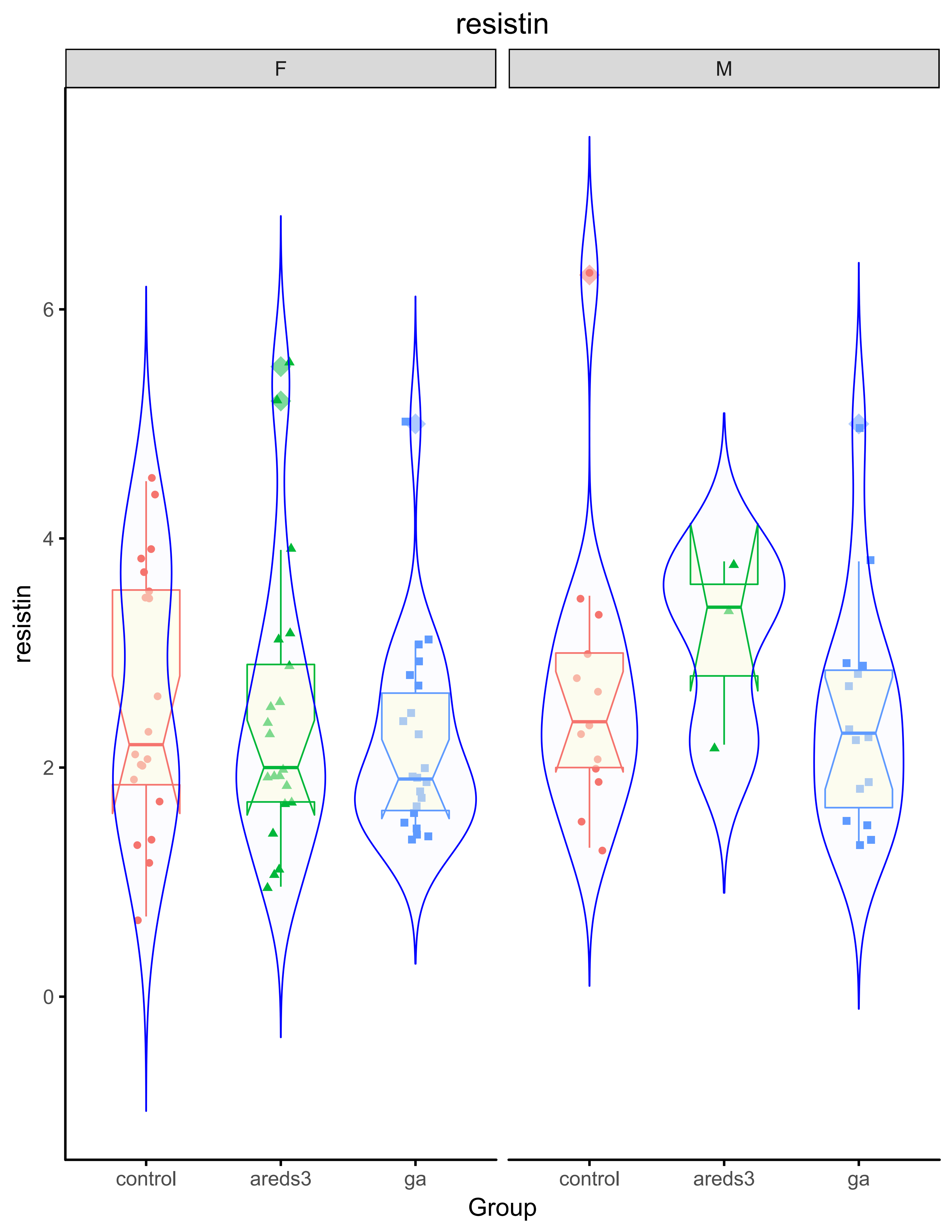

resistin
F
M
6
4
2
0
resistin
control
areds3
ga
control
areds3
ga
Group

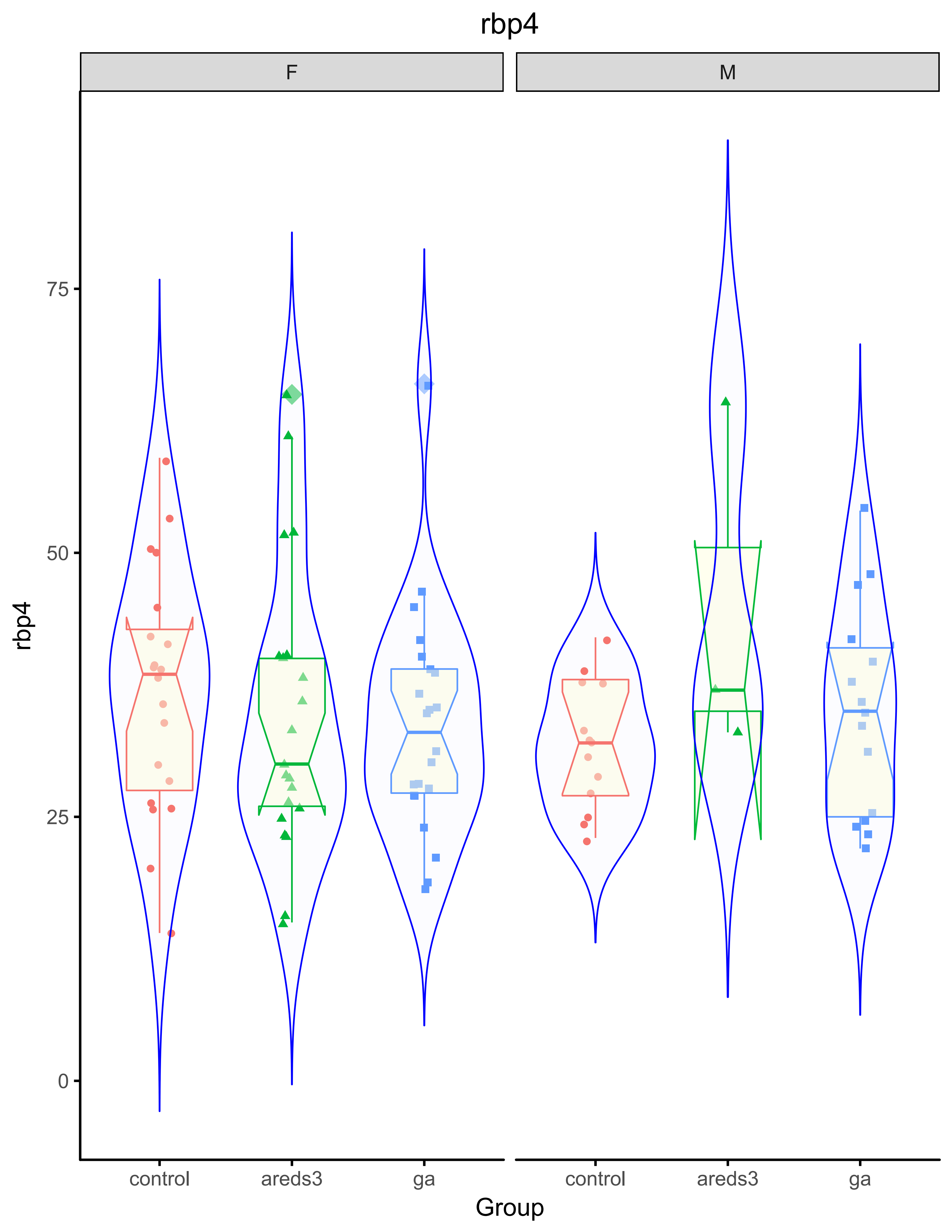

rbp4
F
M
75
50
25
0
rbp4
control
areds3
ga
control
areds3
ga
Group

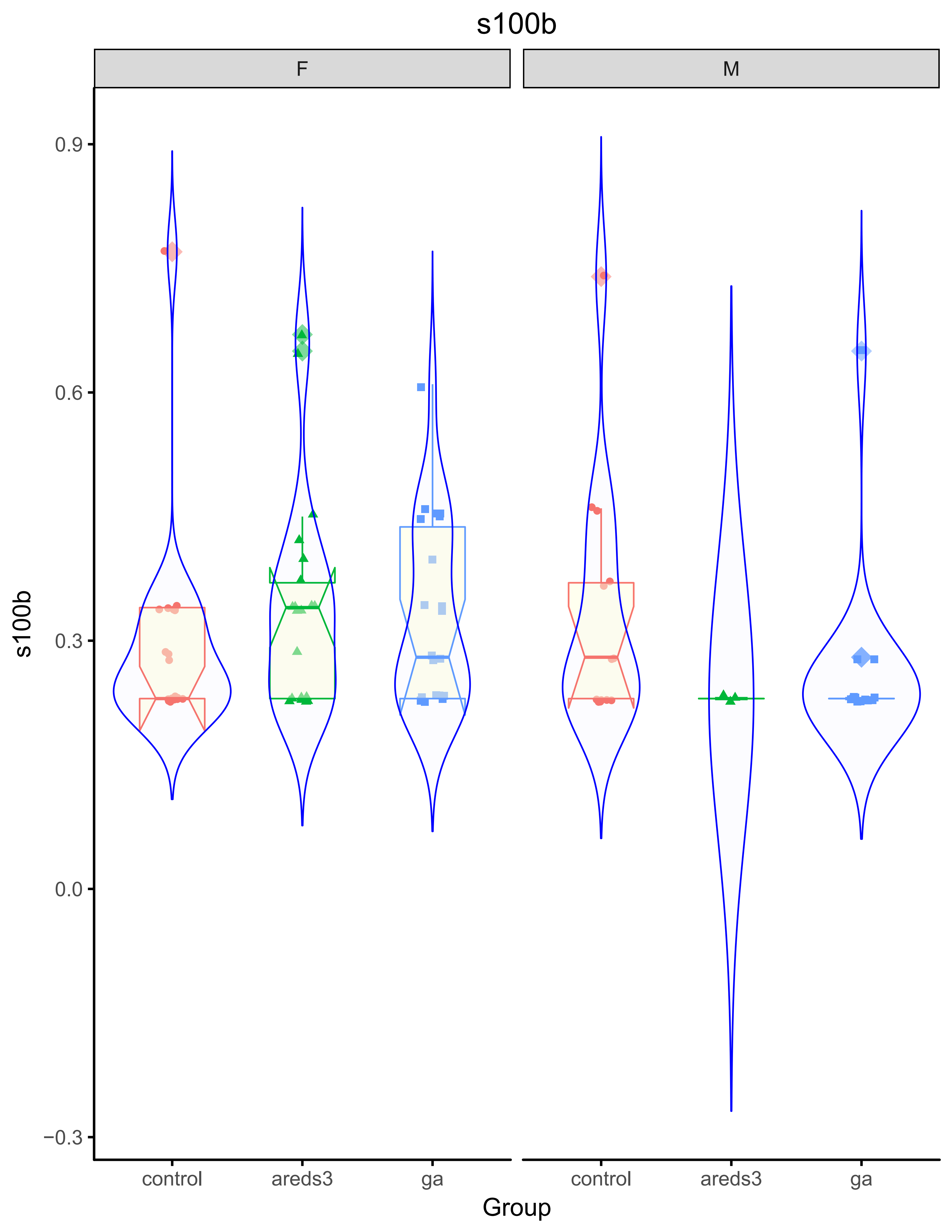
s100b
F
M
0.9
0.6
0.3
0.0
−0.3
s100b
control
areds3
ga
control
areds3
ga
Group

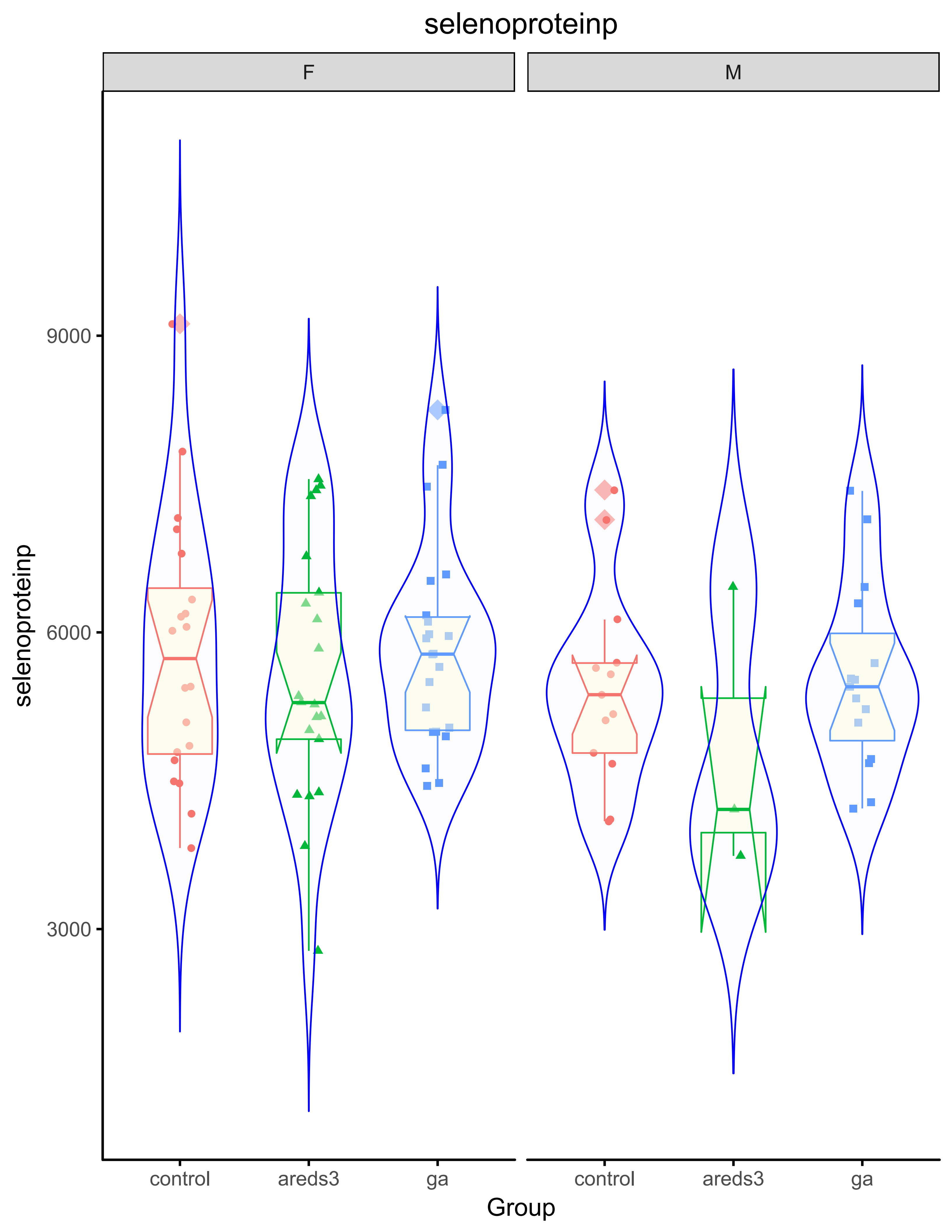

selenoproteinp
F
M
9000
6000
3000
selenoproteinp
control
areds3
ga
control
areds3
ga
Group

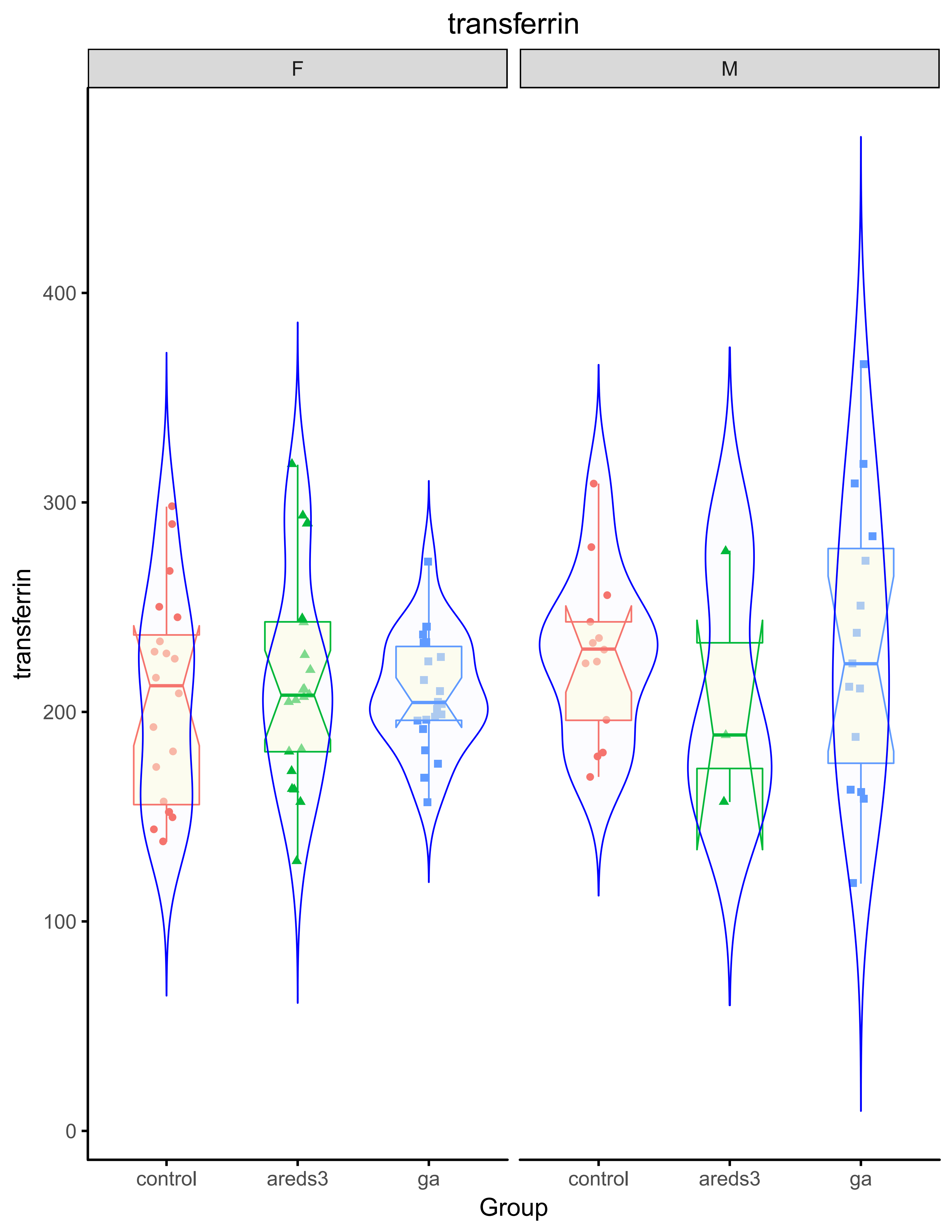

transferrin
F
M
400
300
200
100
0
transferrin
control
areds3
ga
control
areds3
ga
Group

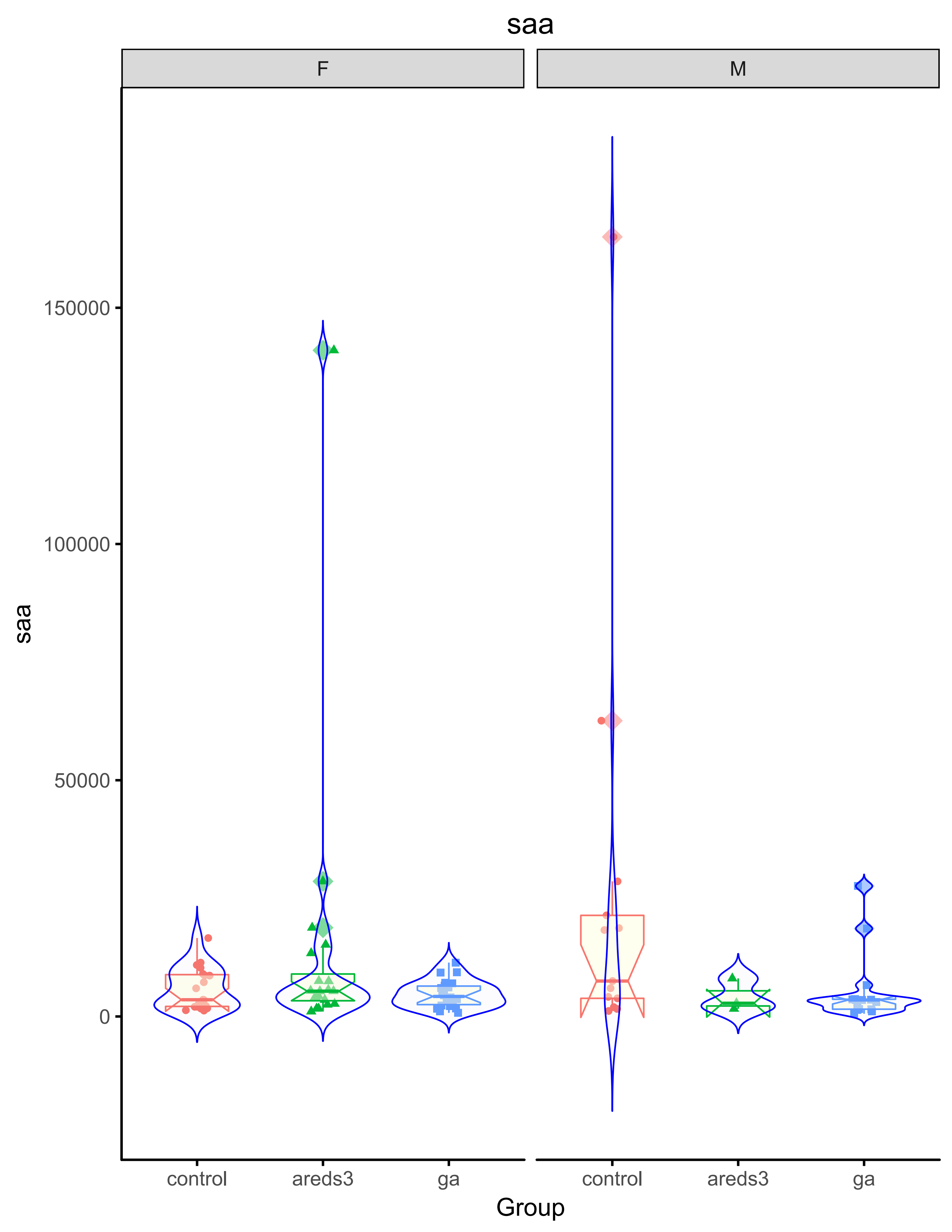

saa
F
M
150000
100000
50000
0
saa
control
areds3
ga
control
areds3
ga
Group

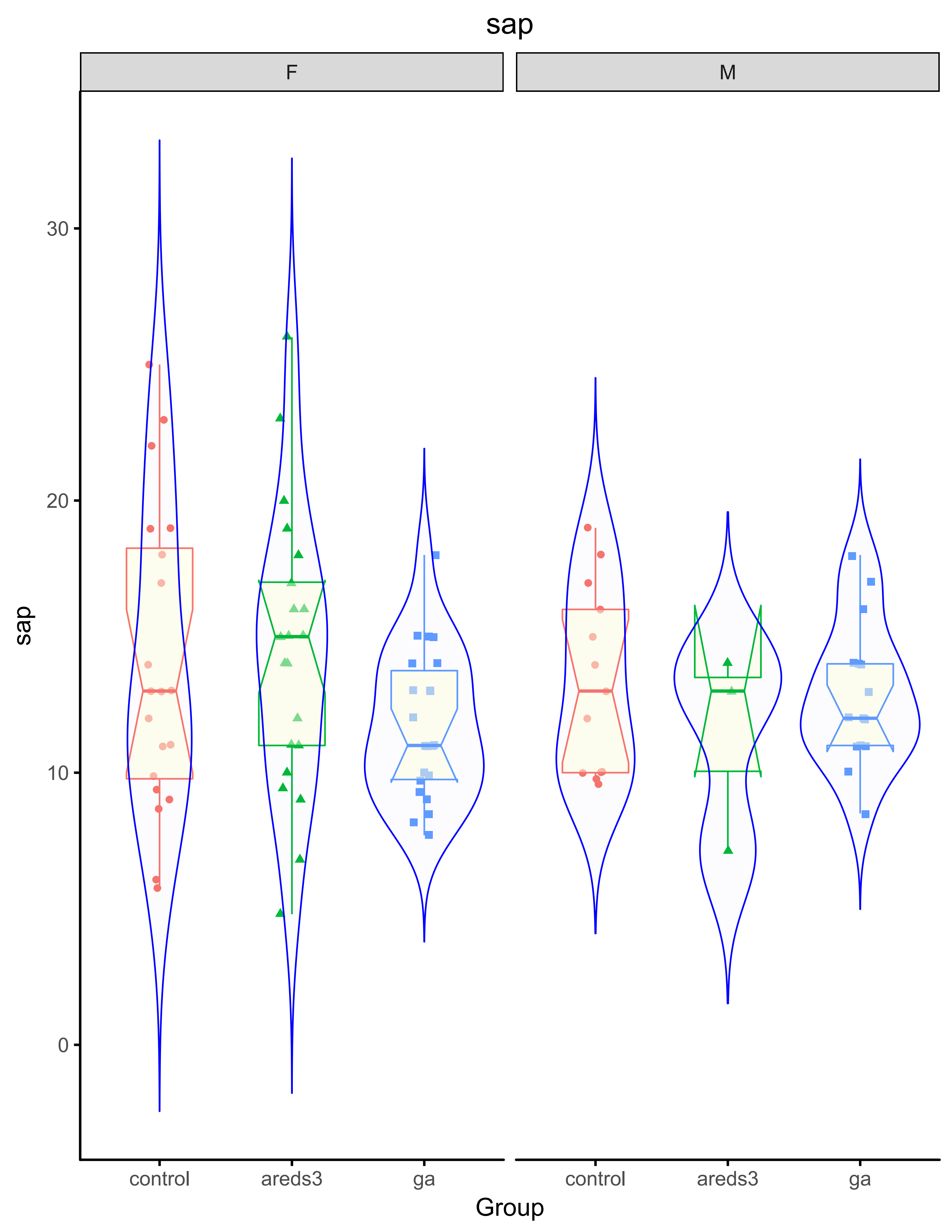
sap
F
M
30
20
10
0
sap
control
areds3
ga
control
areds3
ga
Group

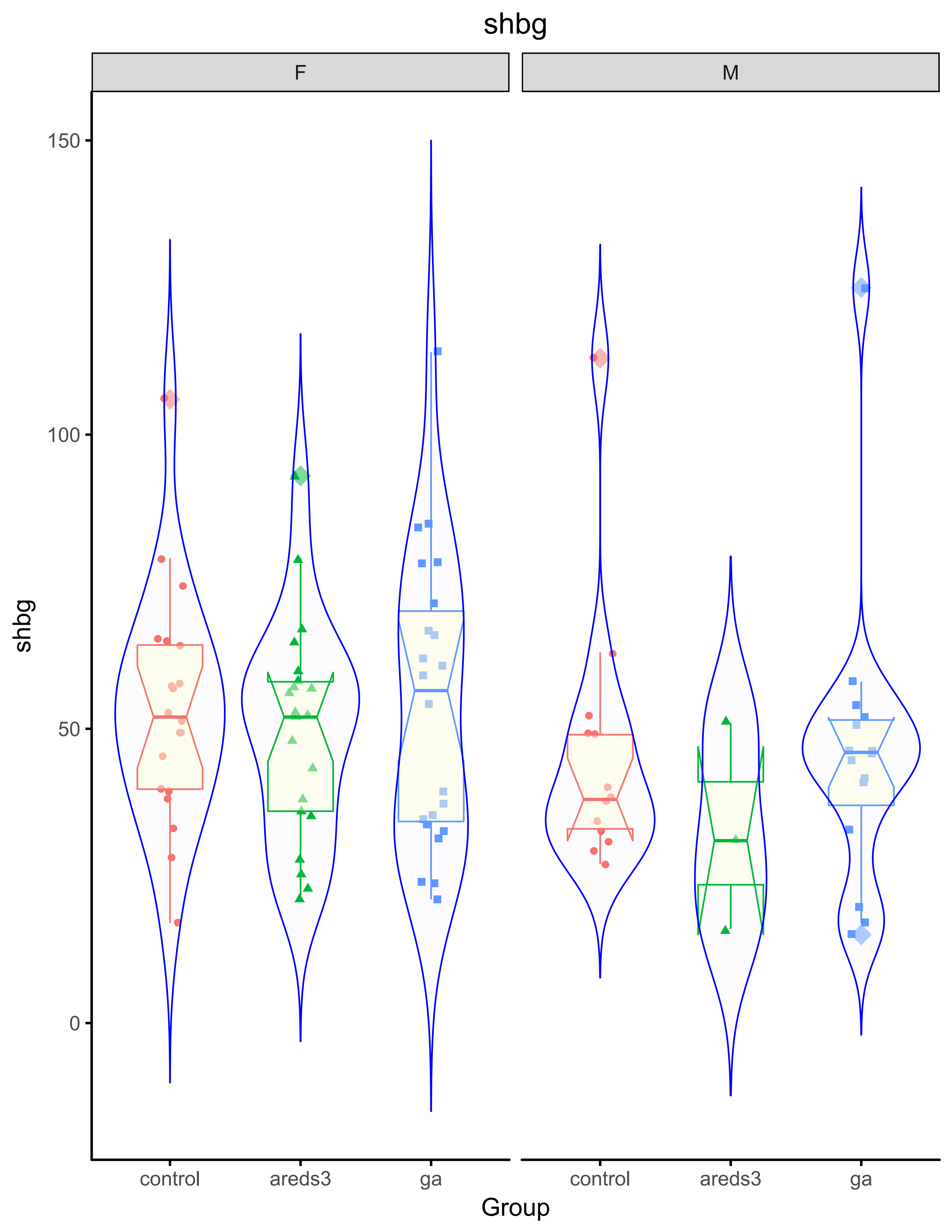

shbg
F
M
150
100
50
0
shbg
control
areds3
ga
control
areds3
ga
Group

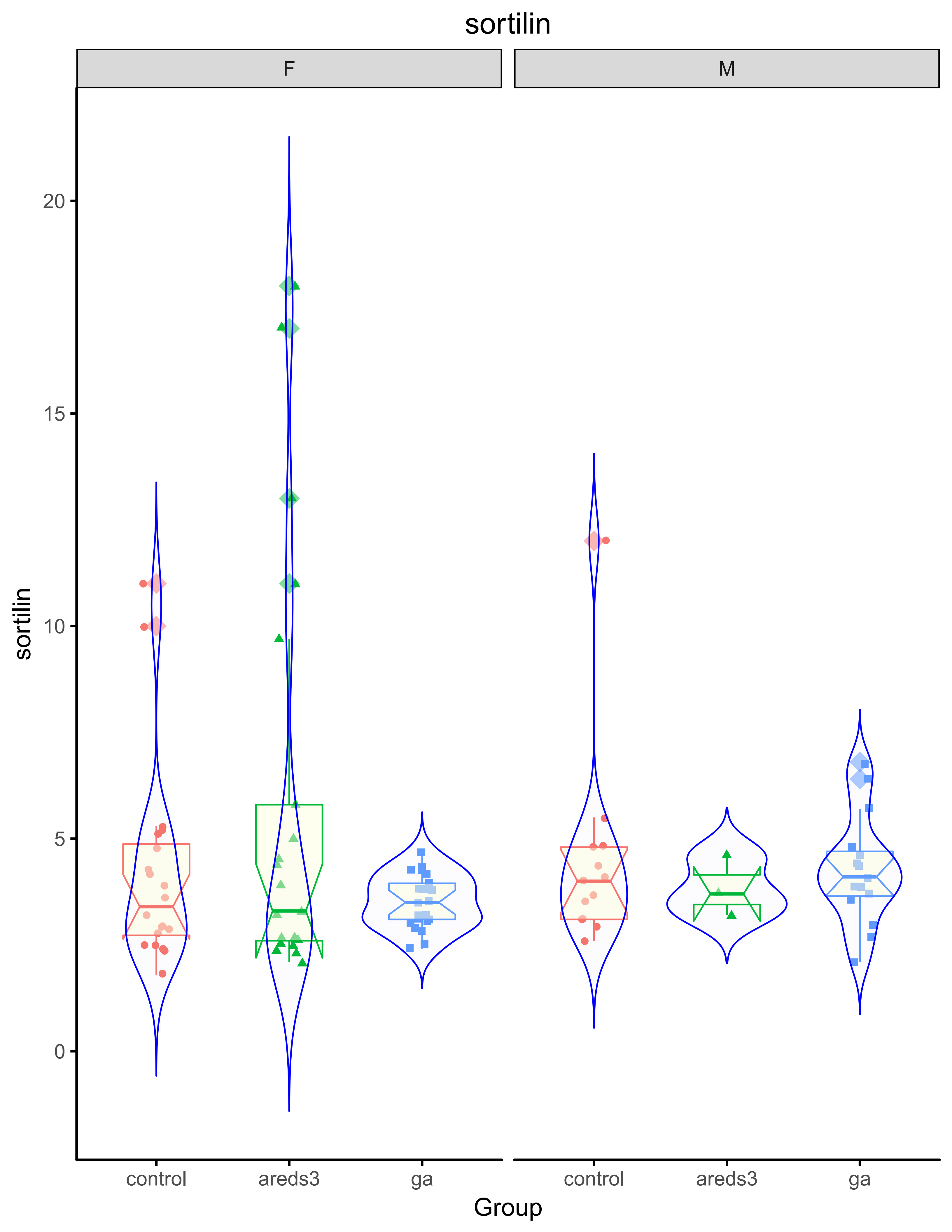
sortilin
F
M
20
15
10
5
0
sortilin
control
areds3
ga
control
areds3
ga
Group

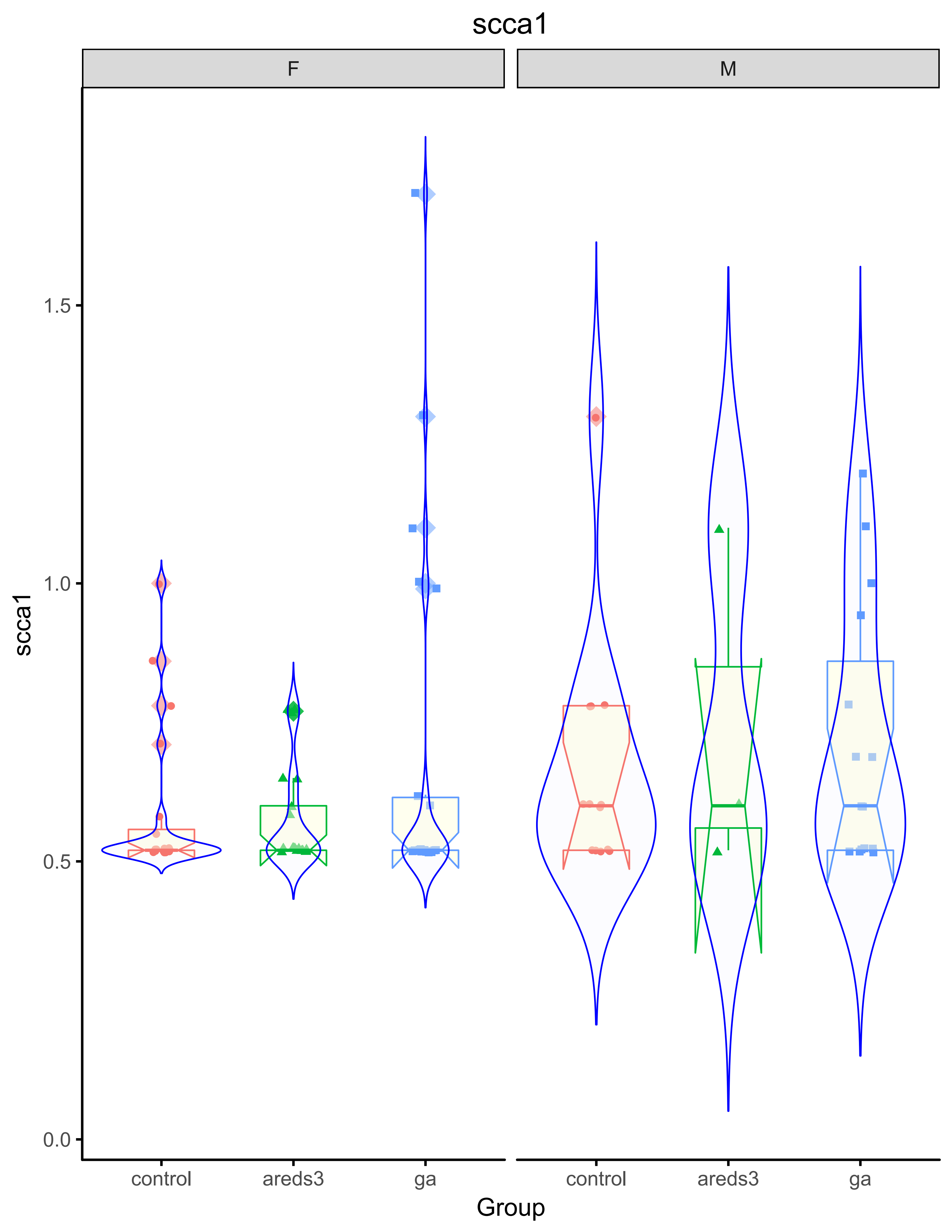
scca1
F
M
scca1
1.5
1.0
0.5
0.0
control
areds3
ga
control
areds3
ga
Group

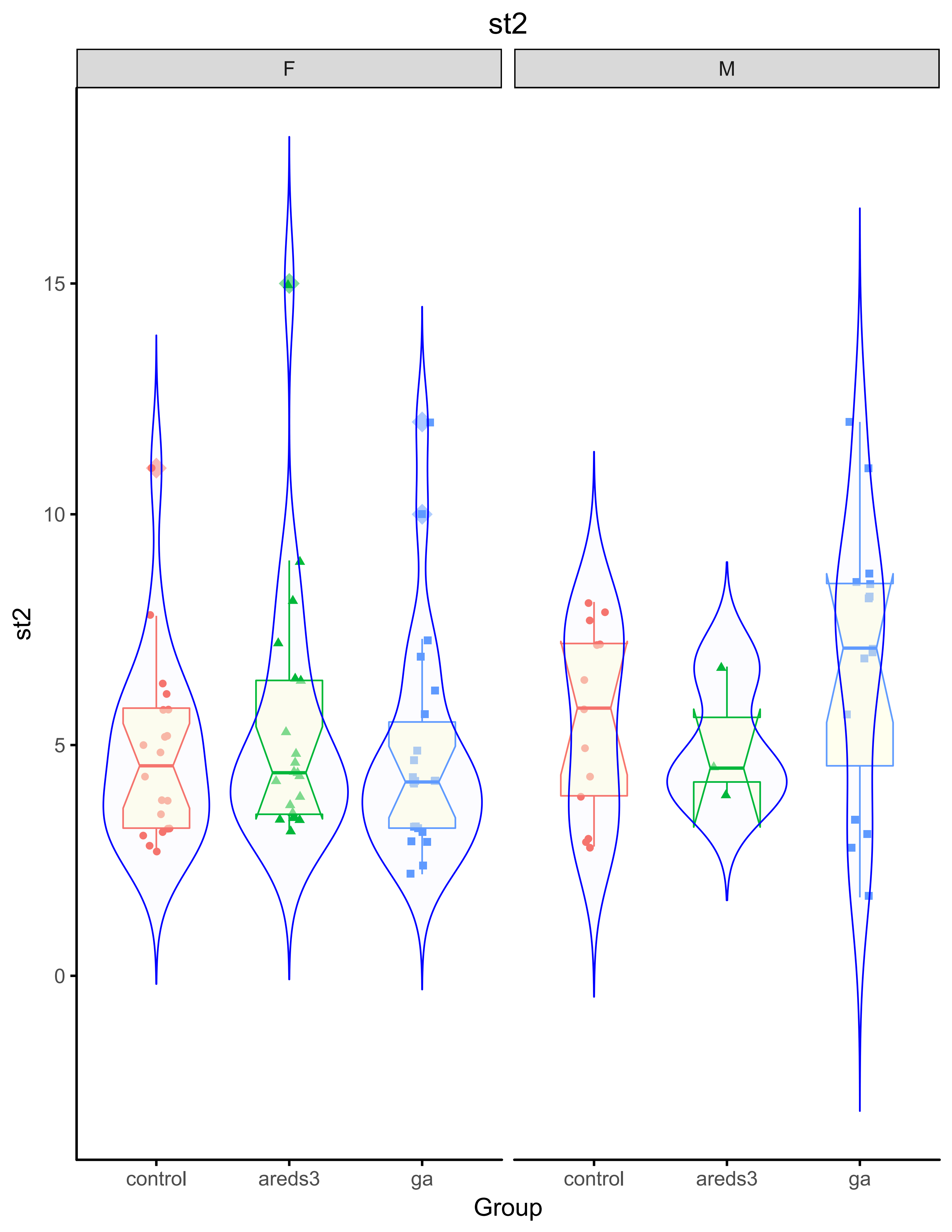

st2
F
M
st2
15
10
5
0
control
areds3
ga
control
areds3
ga
Group

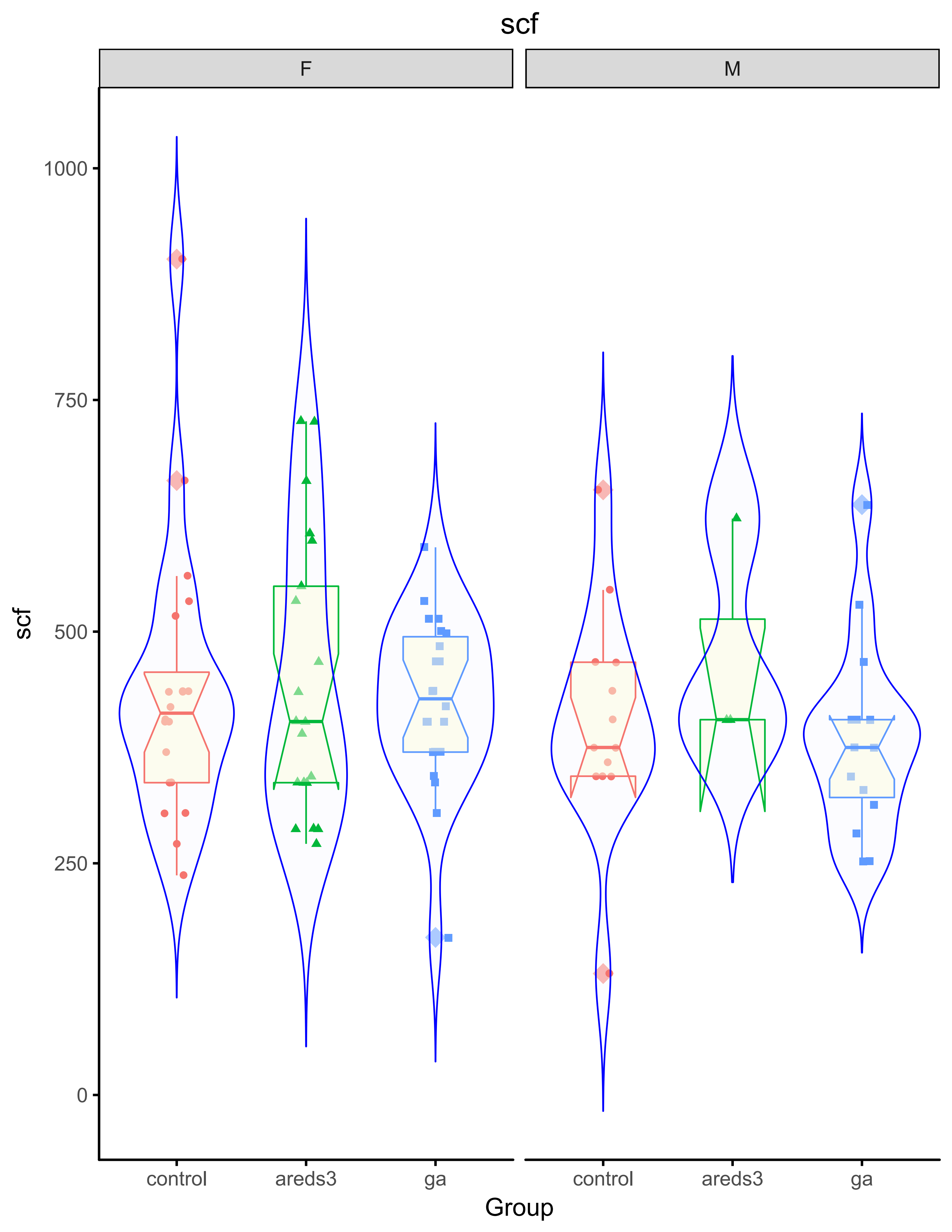

scf
F
M
1000
750
500
250
0
scf
control
areds3
ga
control
areds3
ga
Group

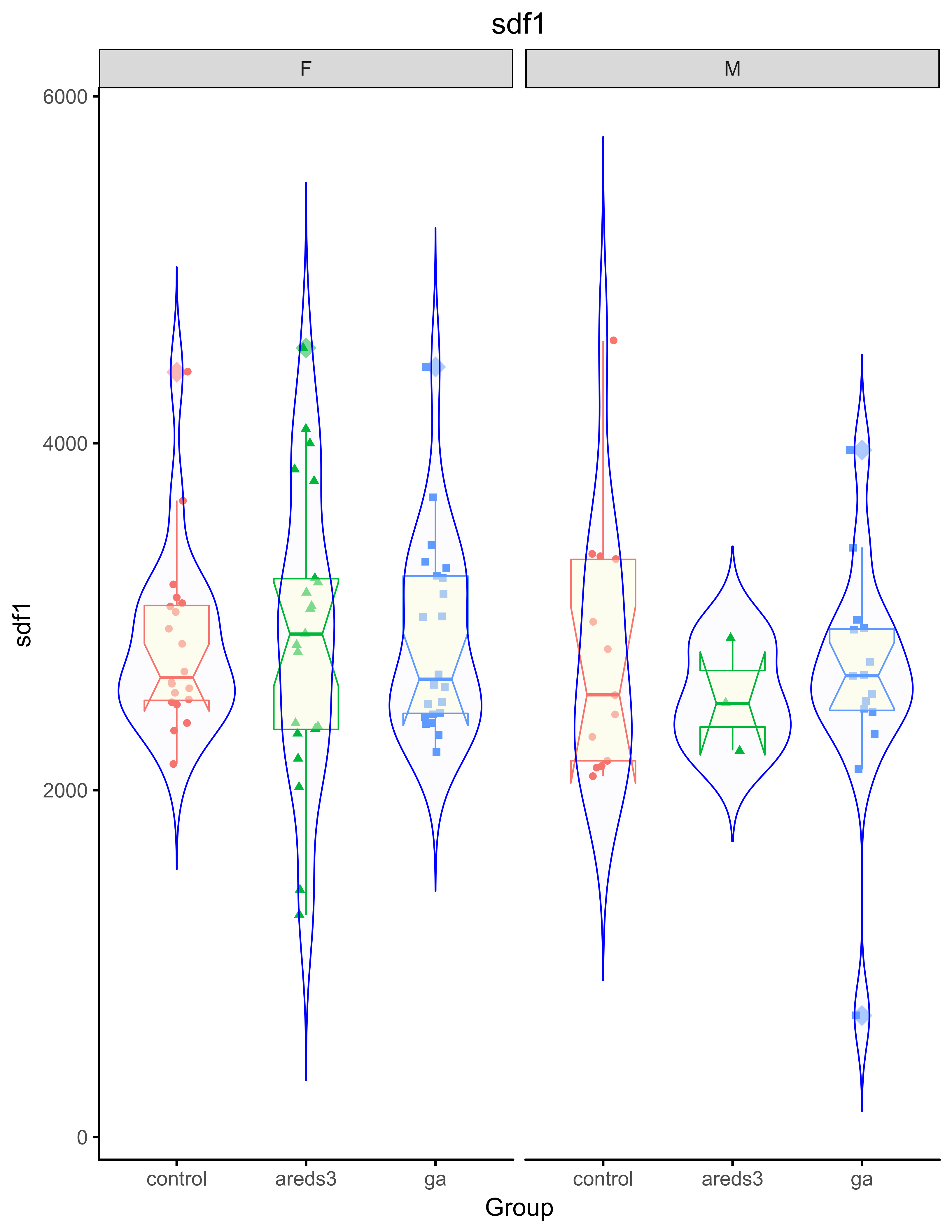

sdf1
F
M
6000
4000
2000
0
sdf1
control
areds3
ga
control
areds3
ga
Group

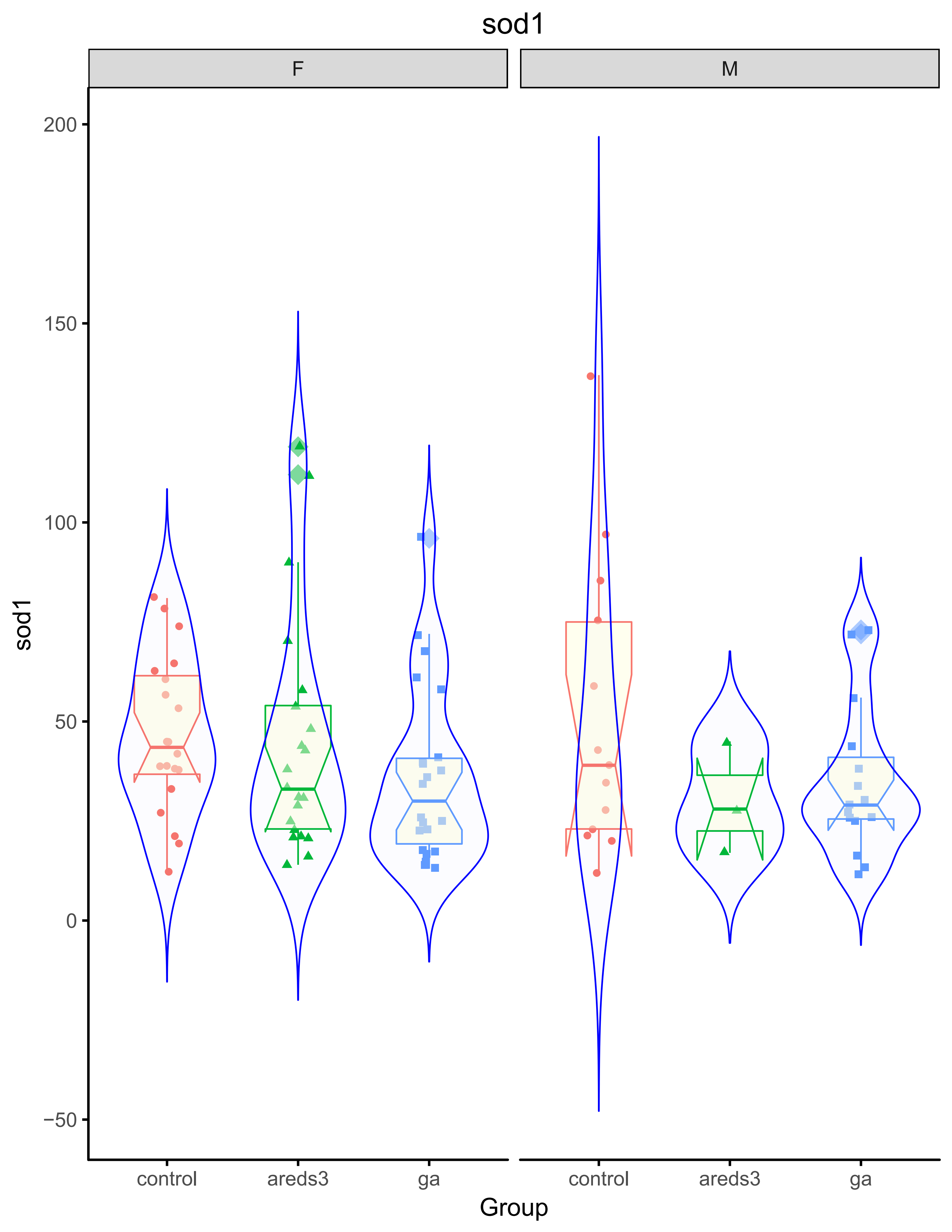

sod1
F
M
200
150
100
50
0
−50
sod1
control
areds3
ga
control
areds3
ga
Group

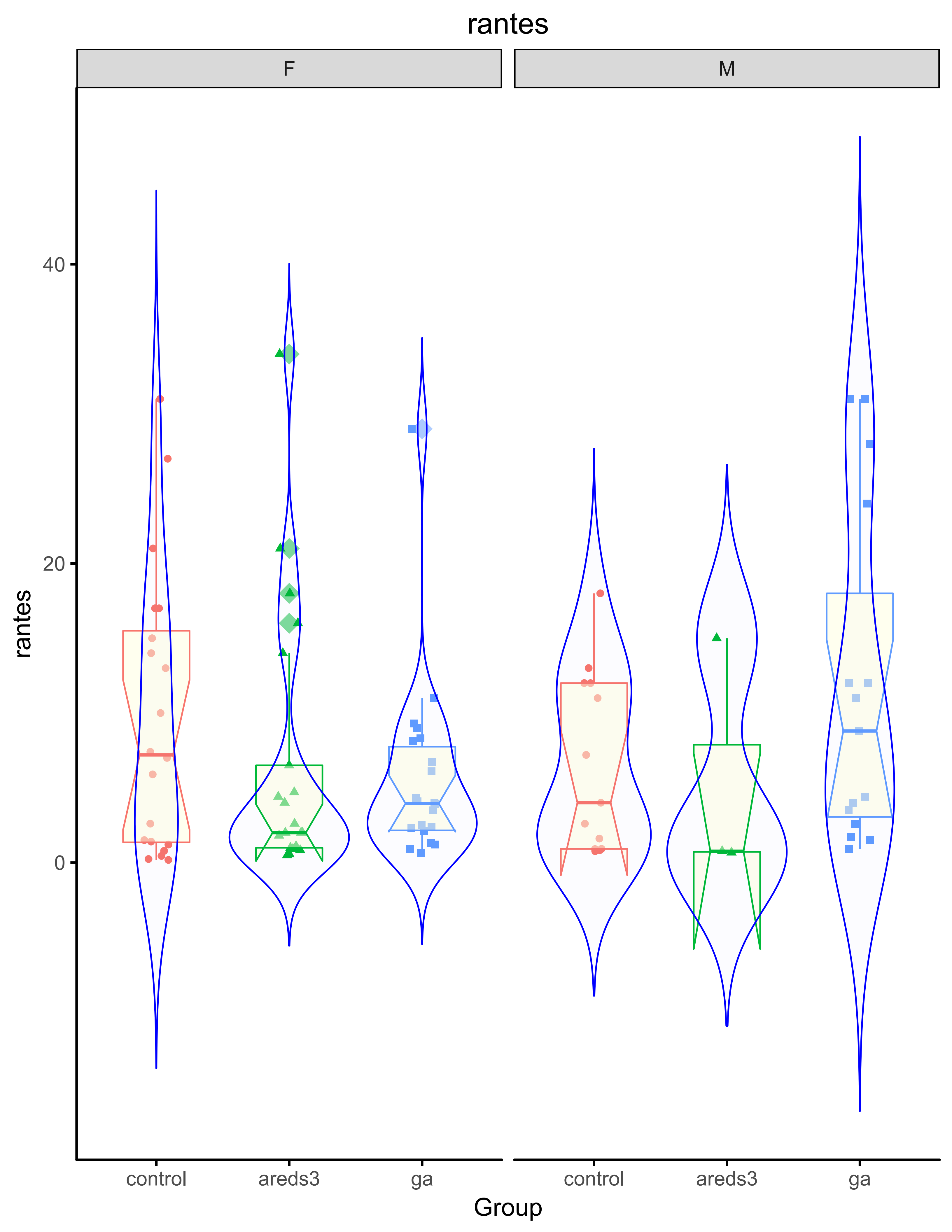
rantes
F
M
40
20
0
rantes
control
areds3
ga
control
areds3
ga
Group

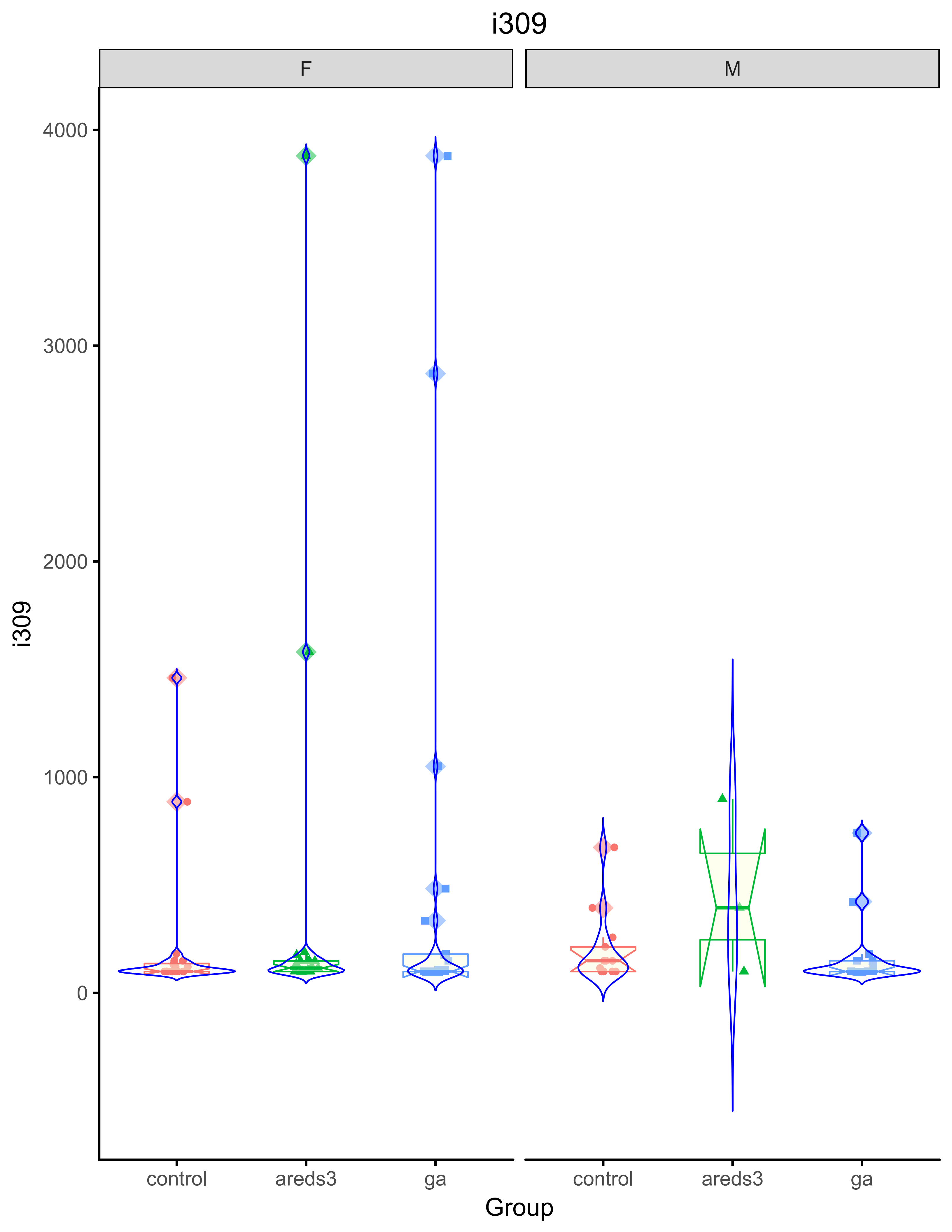
i309
F
M
4000
3000
2000
1000
0
i309
control
areds3
ga
control
areds3
ga
Group

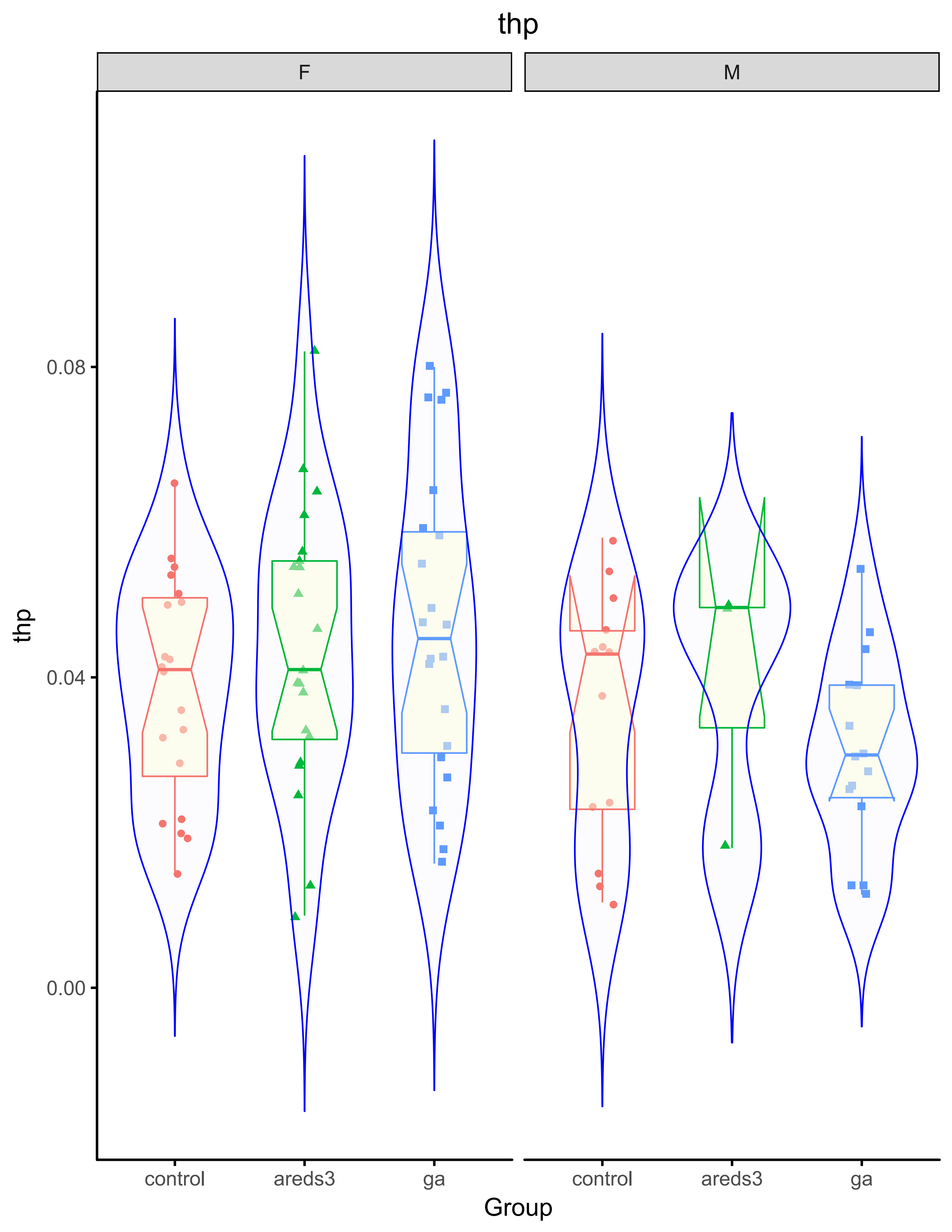

thp
F
M
0.08
0.04
0.00
thp
control
areds3
ga
control
areds3
ga
Group

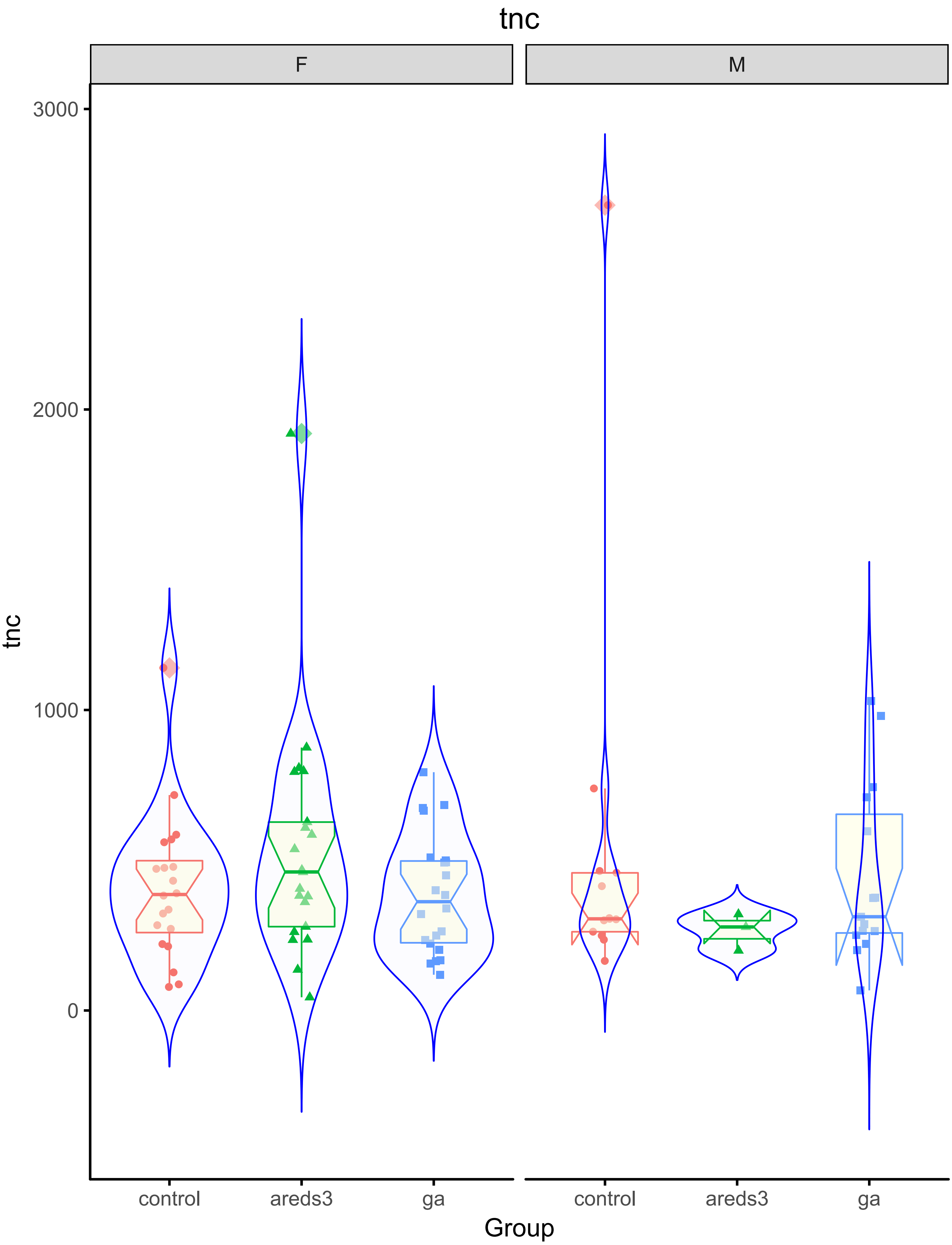

tnc
F
M
3000
2000
1000
0
tnc
control
areds3
ga
control
areds3
ga
Group

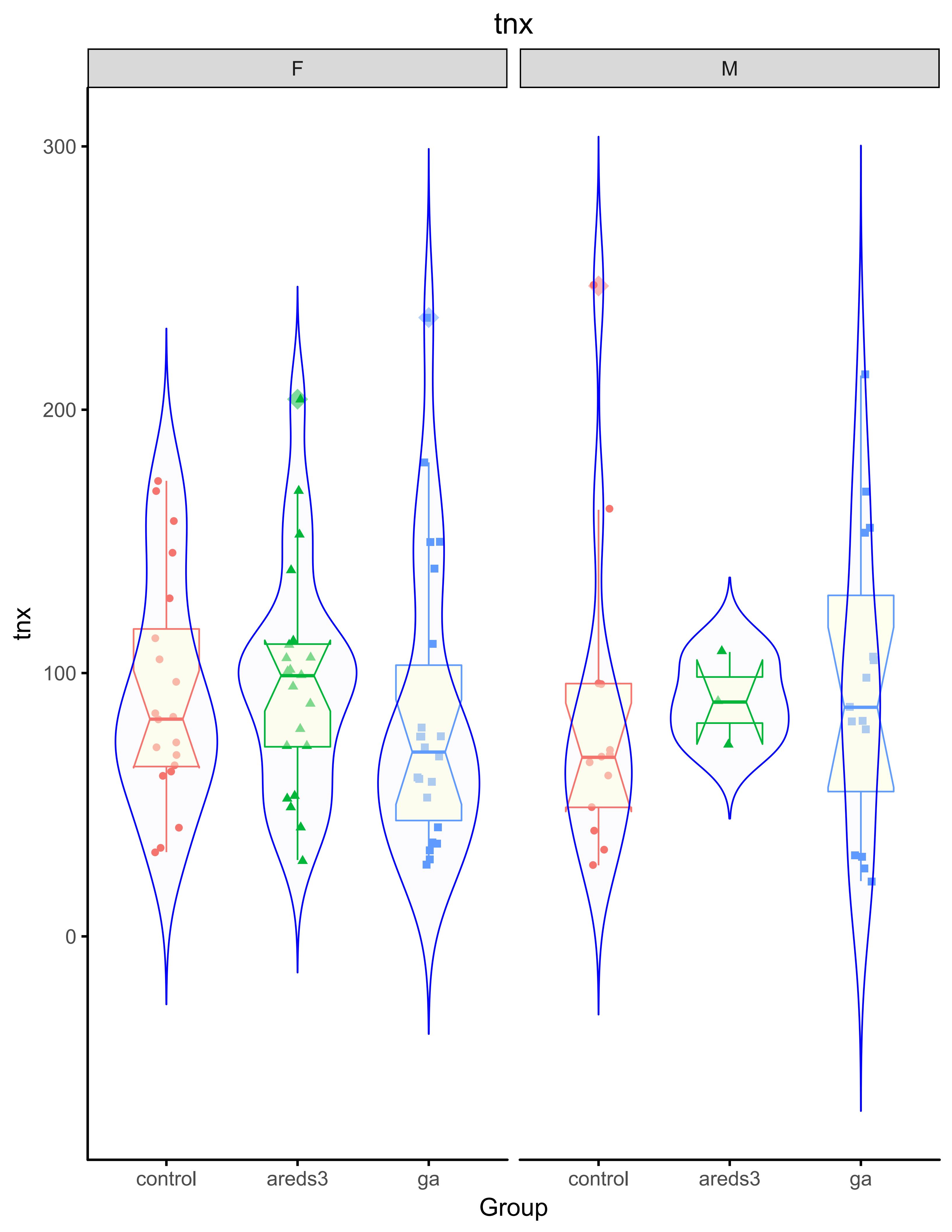

tnx
F
M
300
200
100
0
tnx
control
areds3
ga
control
areds3
ga
Group

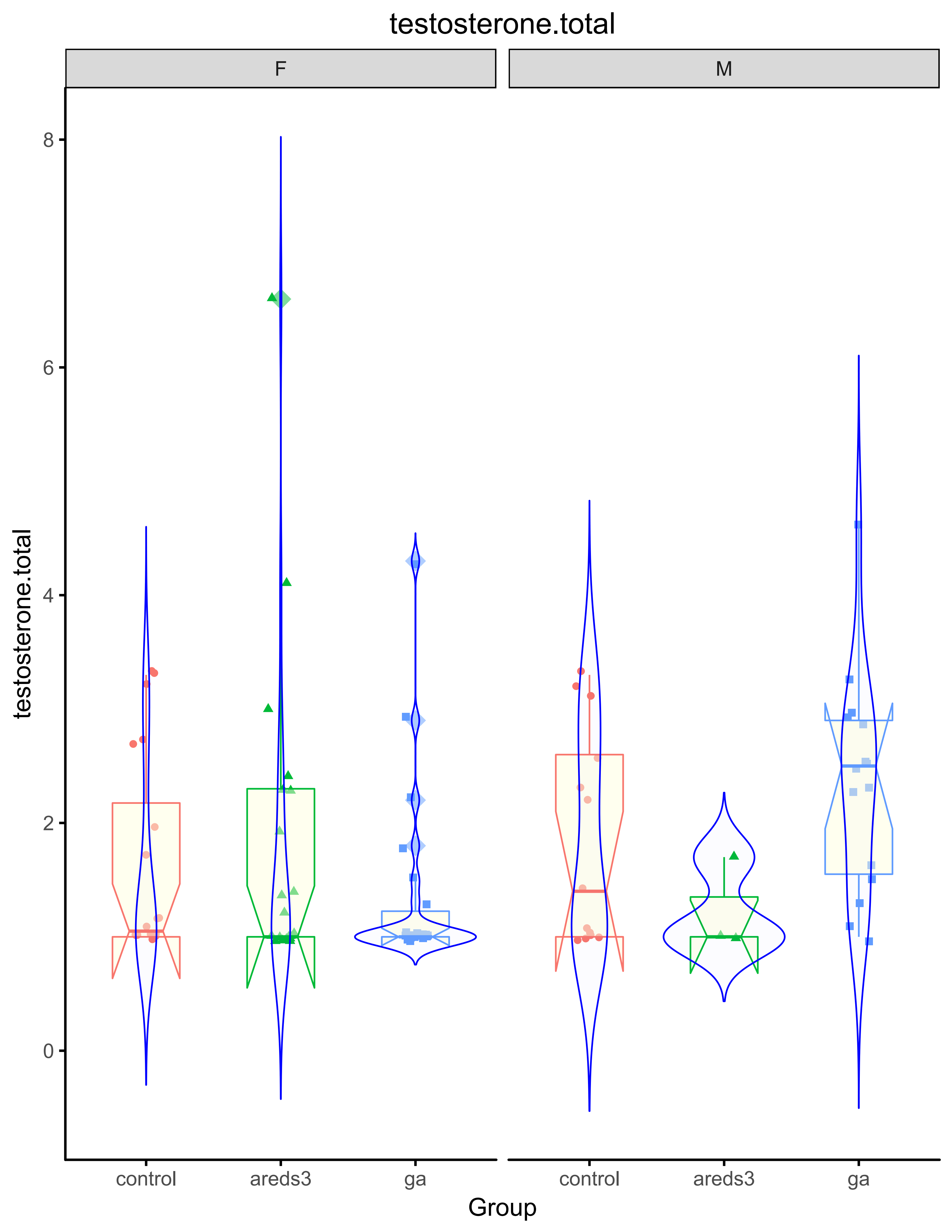
testosterone.total
F
M
8
6
4
2
0
testosterone.total
control
areds3
ga
control
areds3
ga
Group

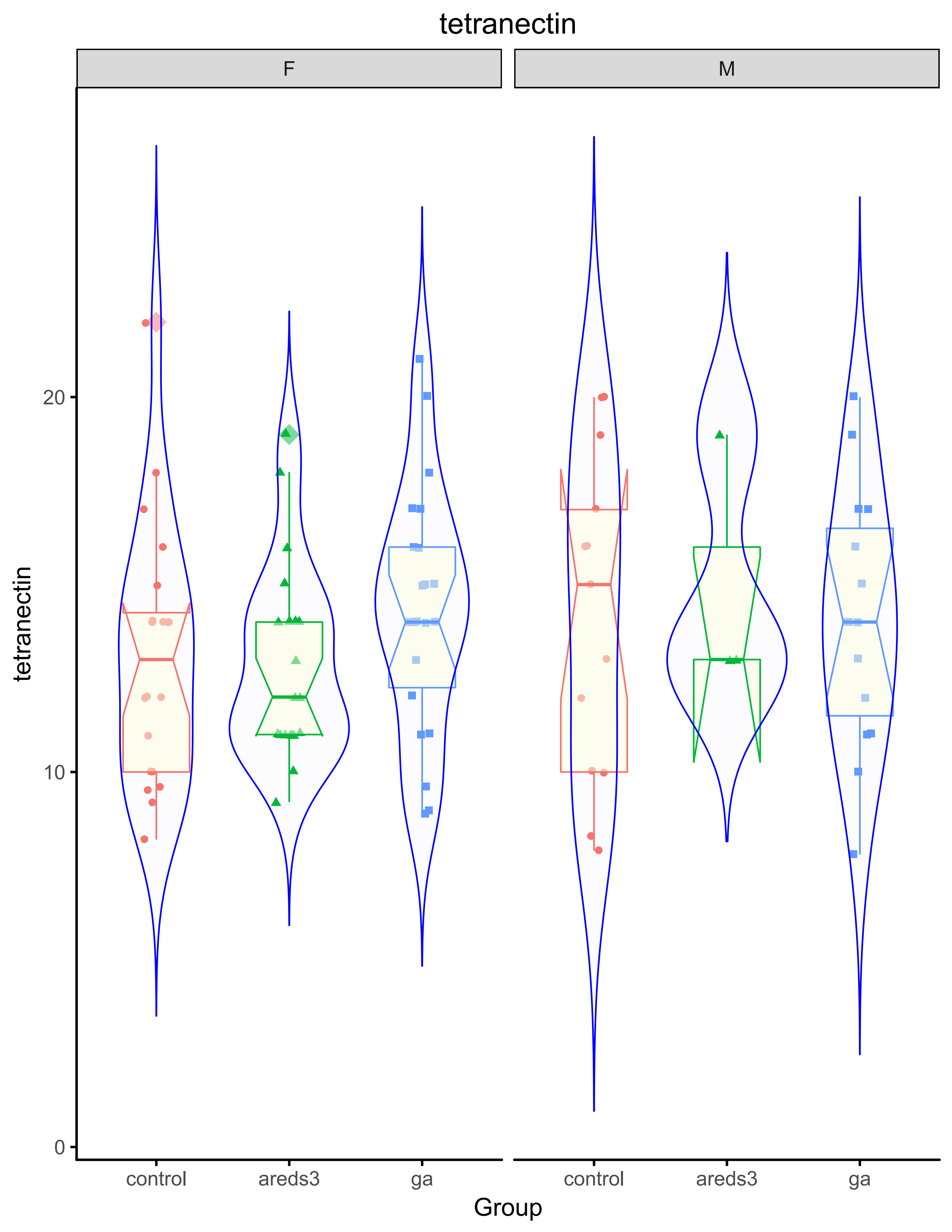
tetranectin
F
M
tetranectin
20
10
0
control
areds3
ga
control
areds3
ga
Group

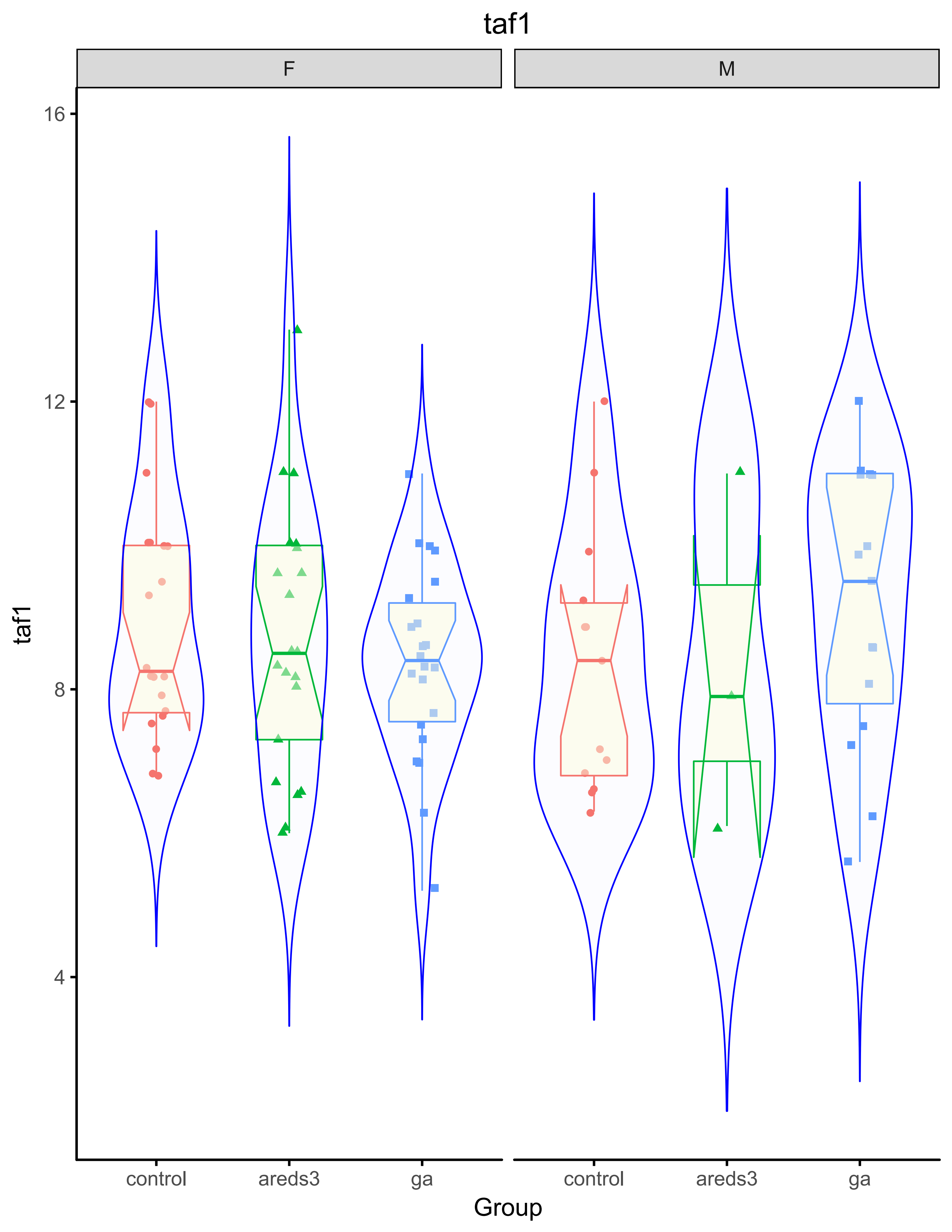
taf1
F
M
16
12
8
4
taf1
control
areds3
ga
control
areds3
ga
Group

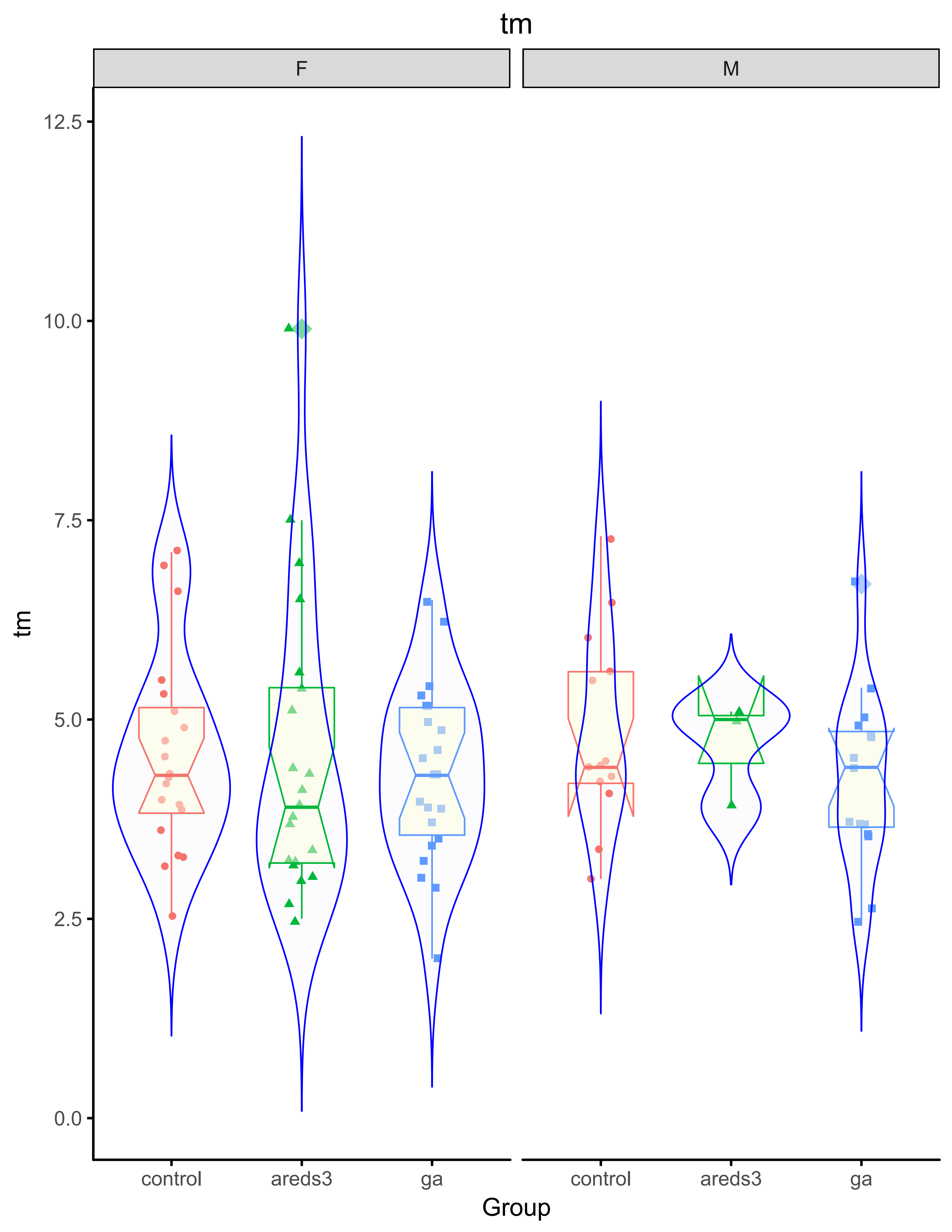

tm
F
M
12.5
10.0
7.5
5.0
2.5
0.0
tm
control
areds3
ga
control
areds3
ga
Group

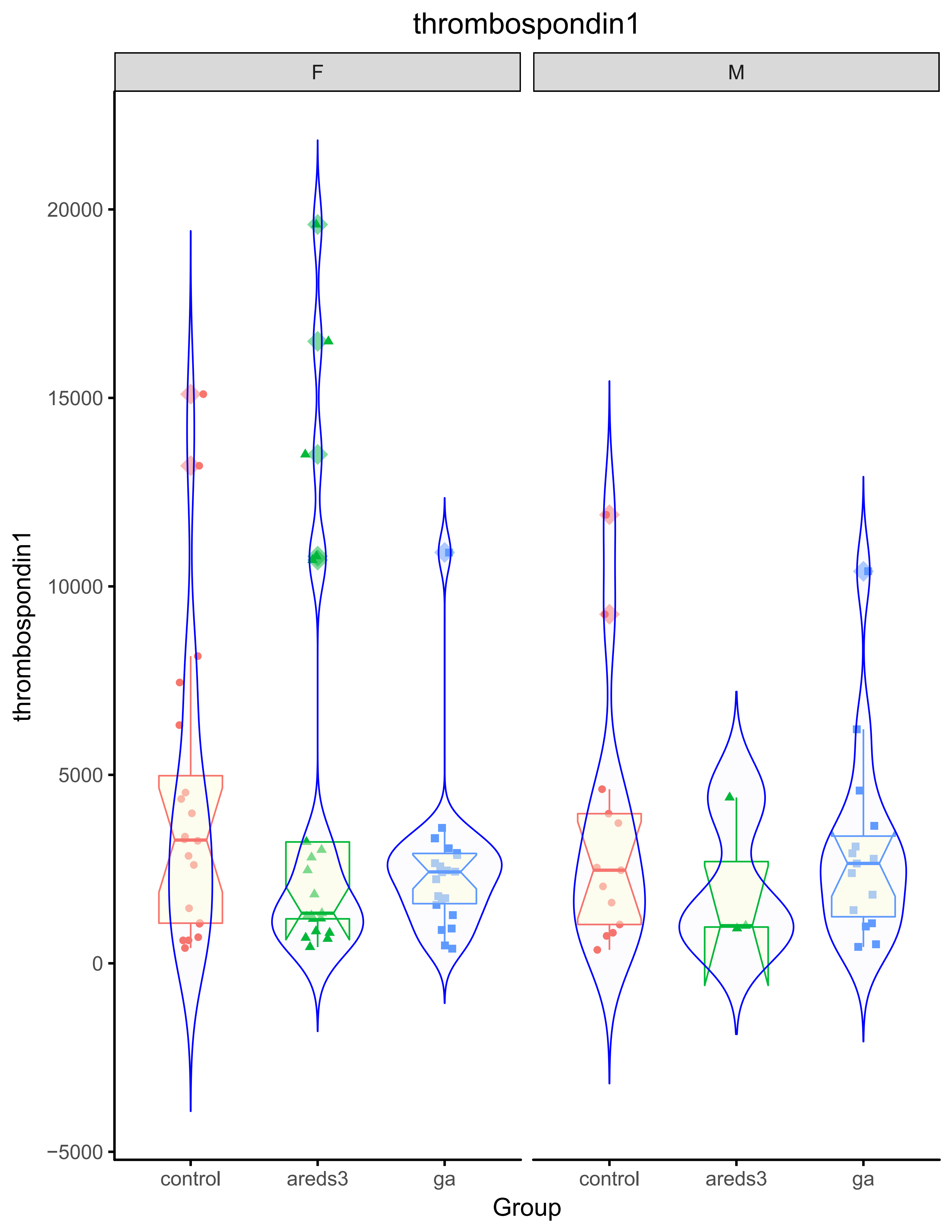
thrombospondin1
F
M
thrombospondin1
20000
15000
10000
5000
0
−5000
control
areds3
ga
control
areds3
ga
Group

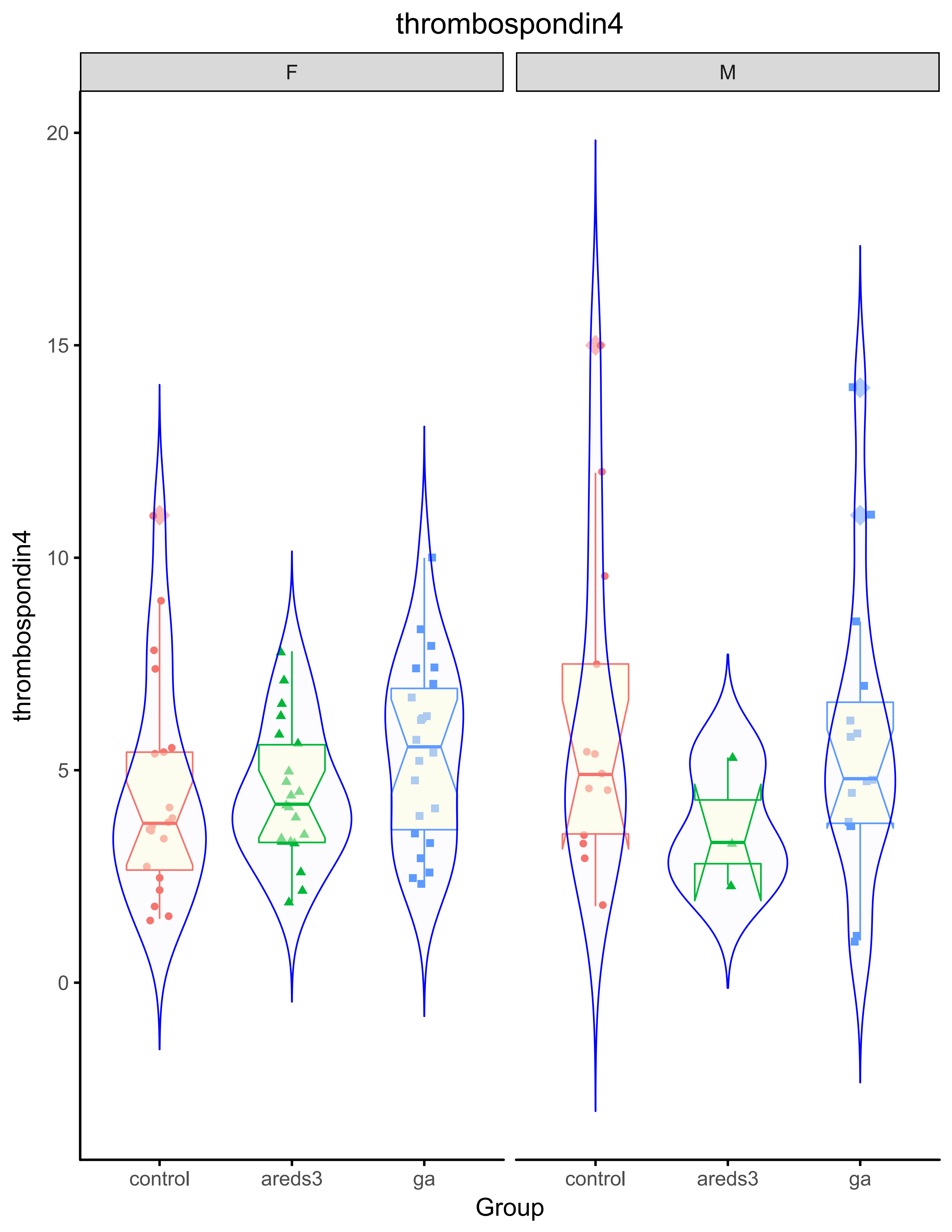

thrombospondin4
F
M
20
15
10
5
0
thrombospondin4
control
areds3
ga
control
areds3
ga
Group

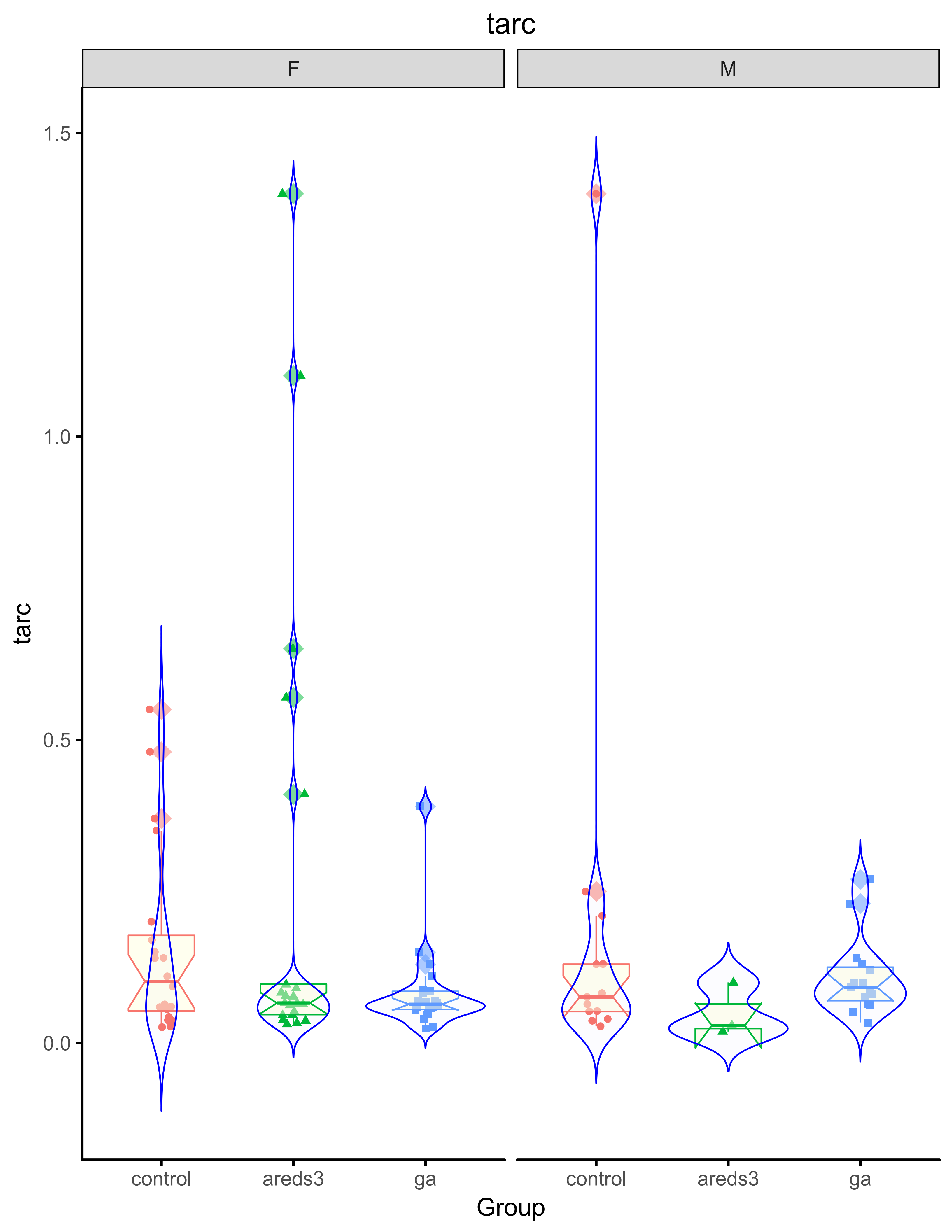

tarc
F
M
1.5
1.0
0.5
0.0
tarc
control
areds3
ga
control
areds3
ga
Group

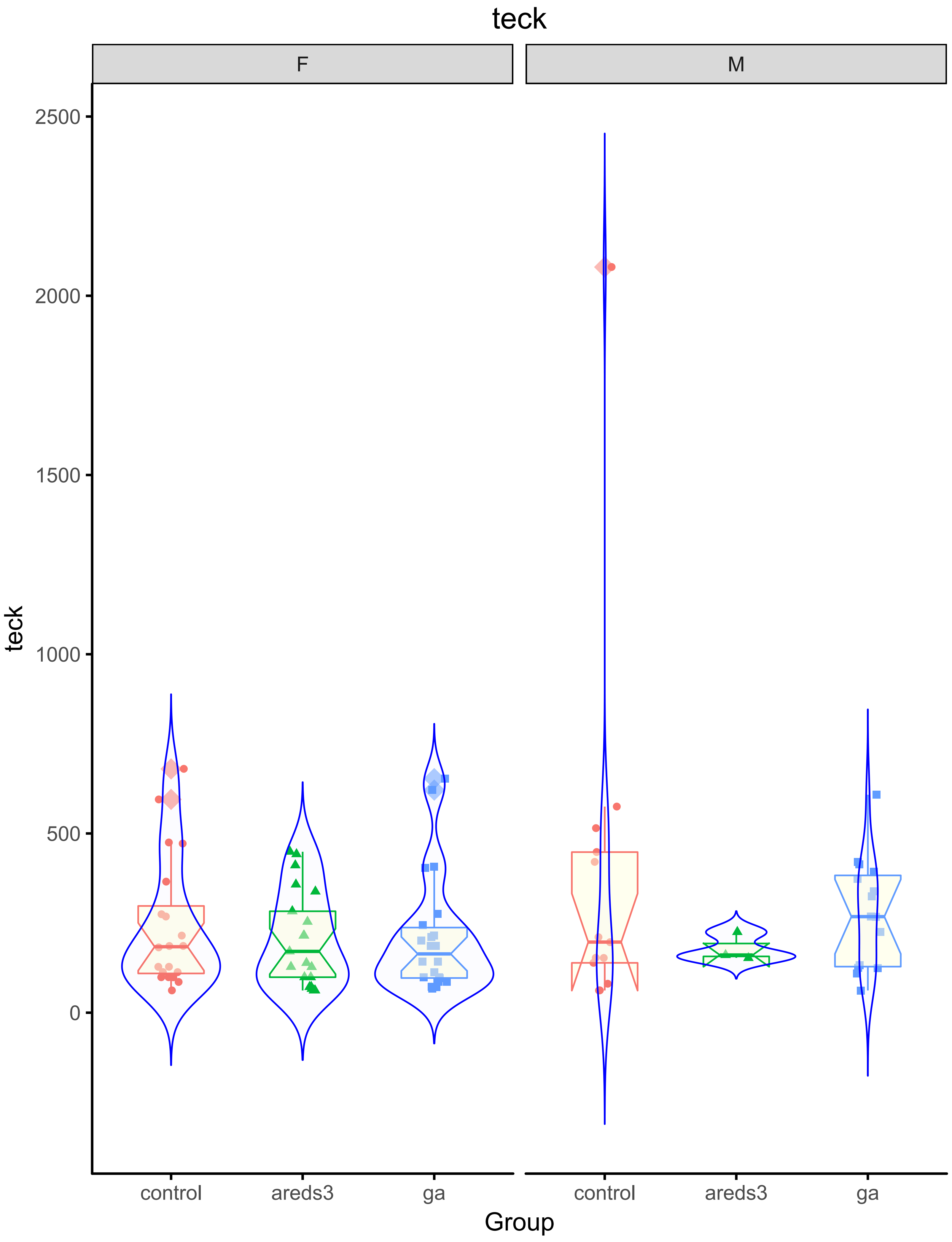

teck
F
M
2500
2000
1500
1000
500
0
teck
control
areds3
ga
control
areds3
ga
Group

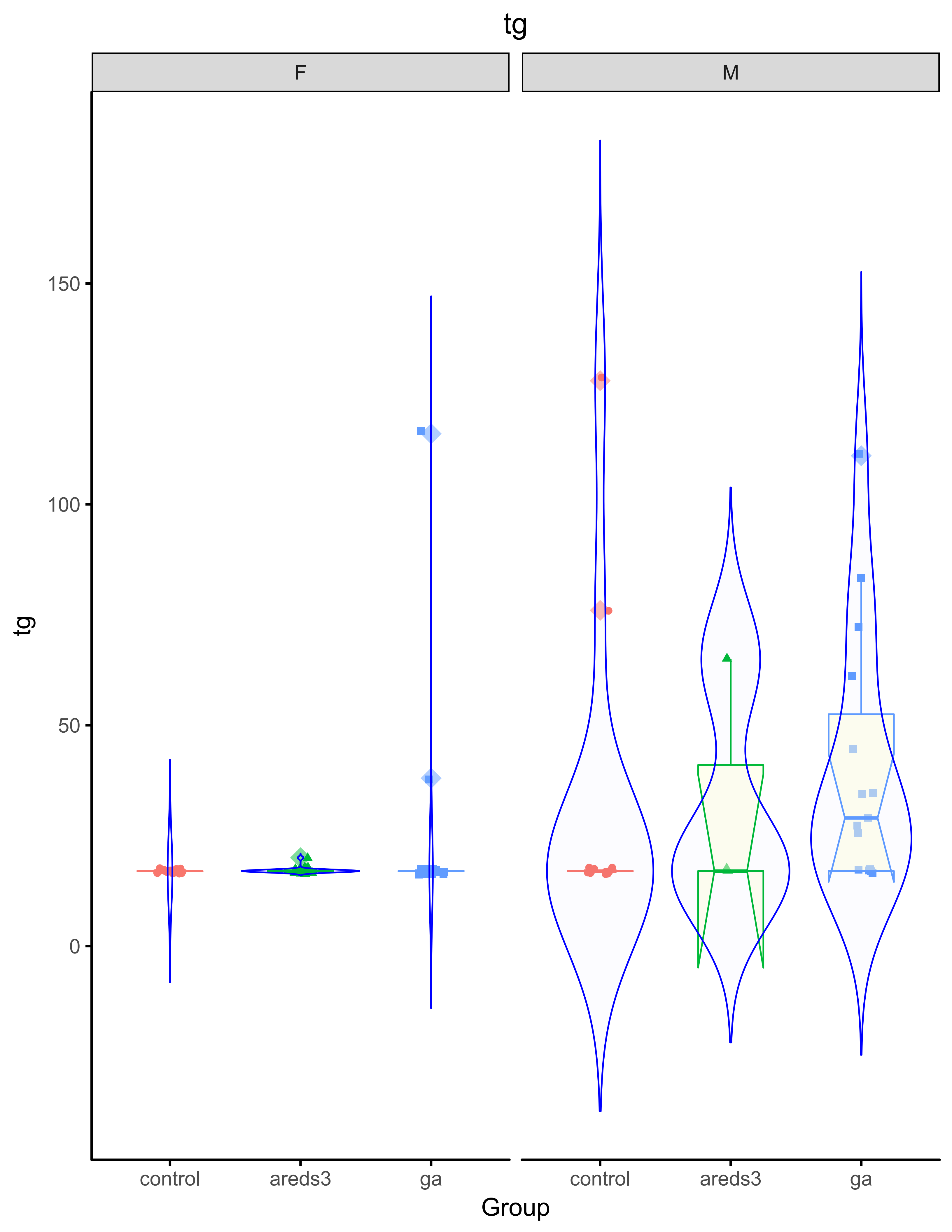

tg
F
M
150
100
50
0
tg
control
areds3
ga
control
areds3
ga
Group

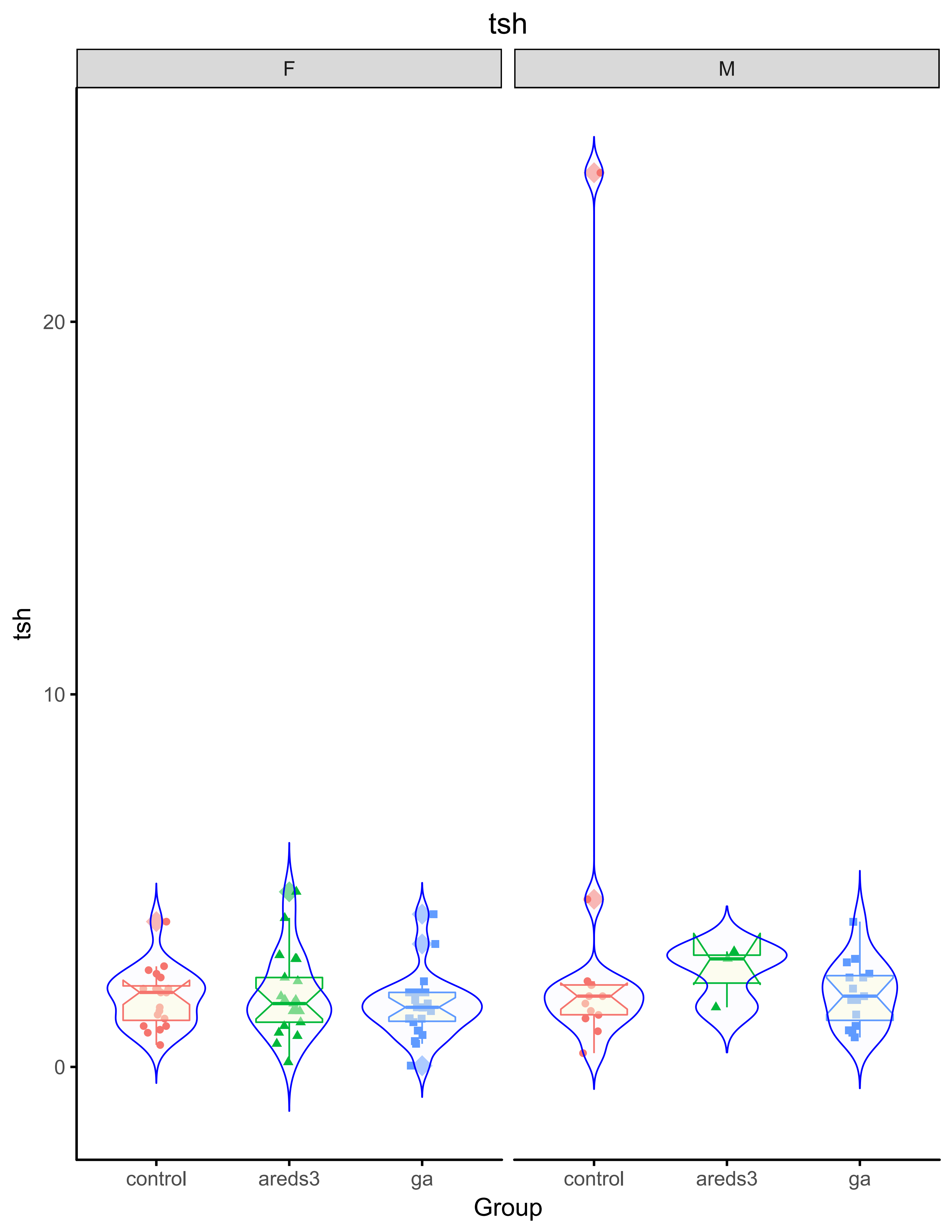

tsh
F
M
20
10
0
tsh
control
areds3
ga
control
areds3
ga
Group

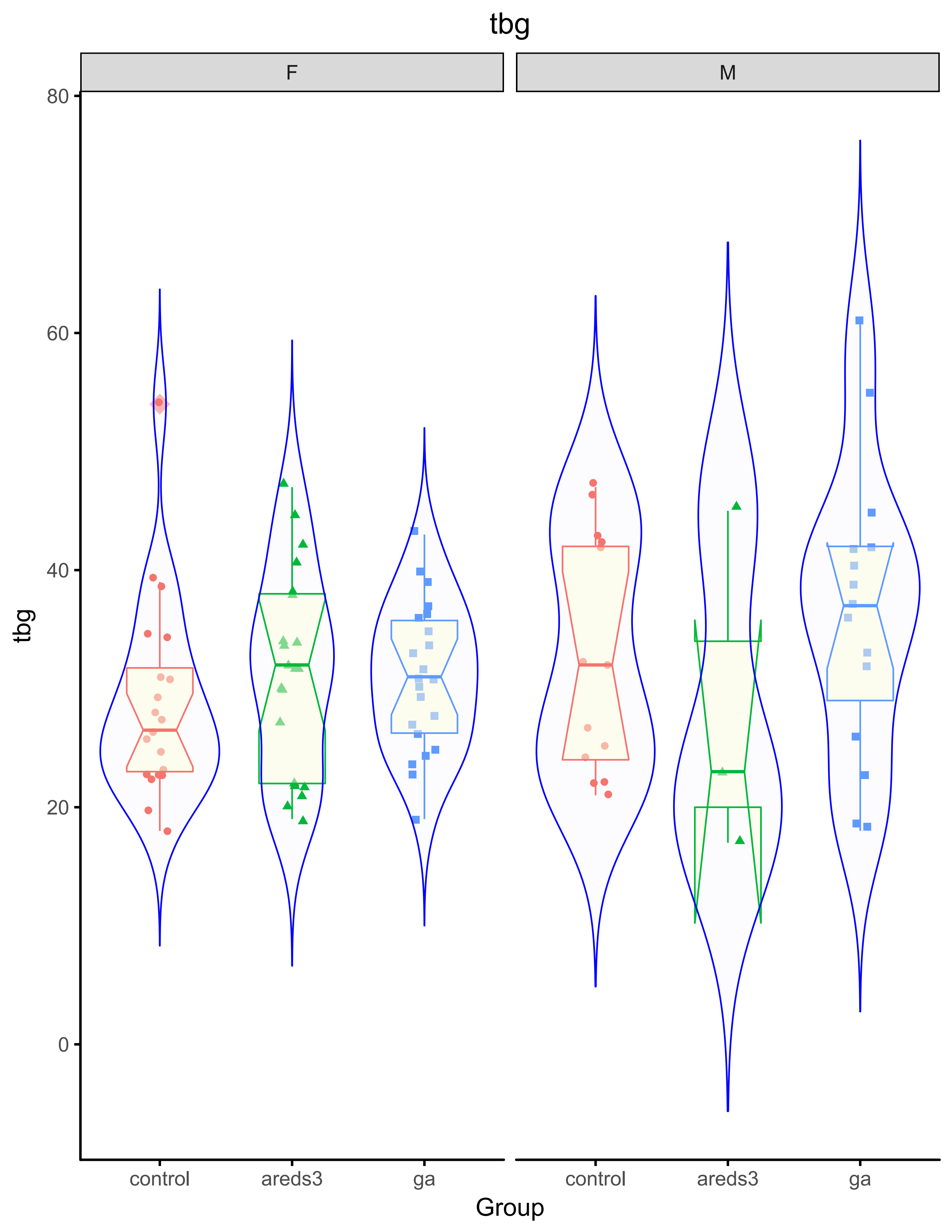
tbg
F
M
80
60
40
20
0
tbg
control
areds3
ga
control
areds3
ga
Group

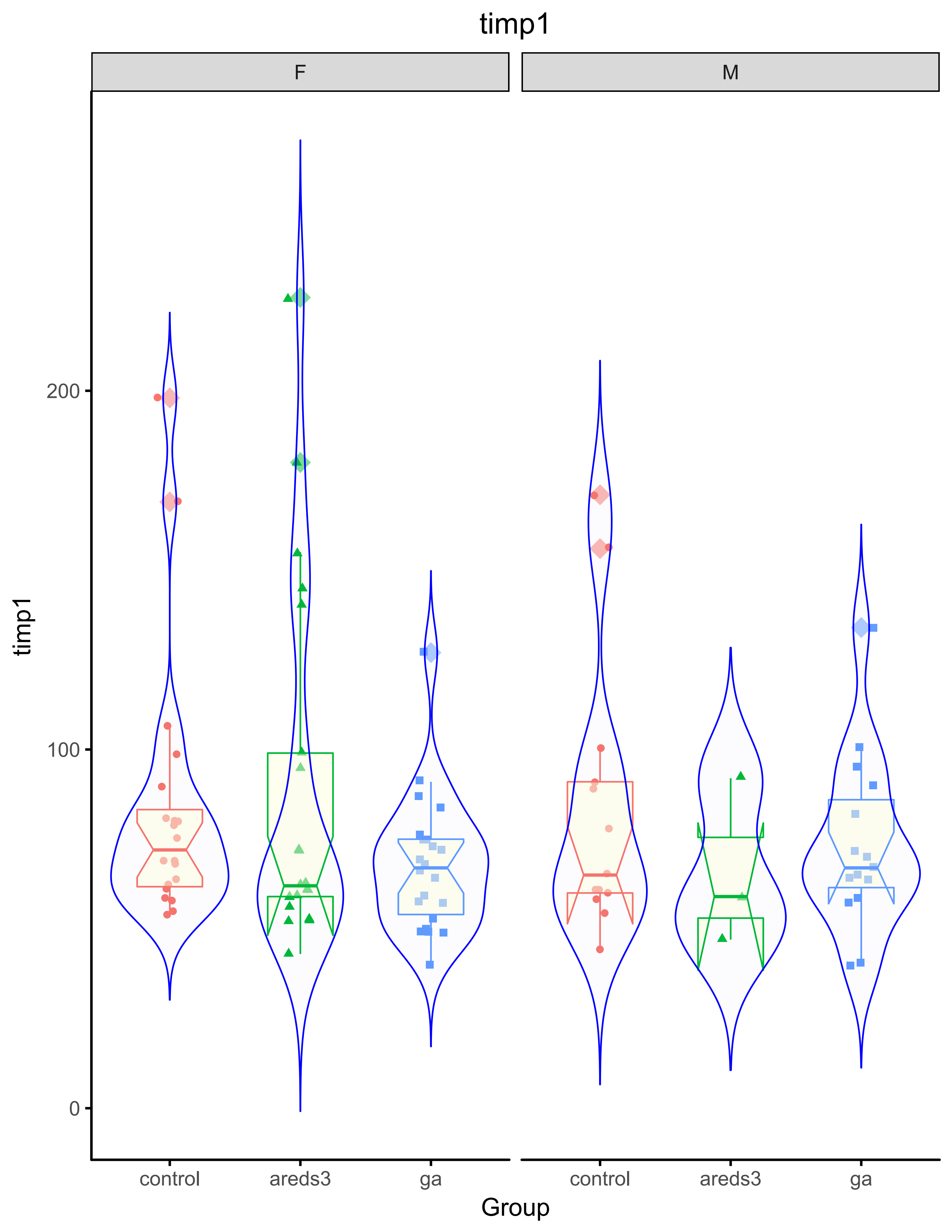
timp1
F
M
200
100
0
timp1
control
areds3
ga
control
areds3
ga
Group

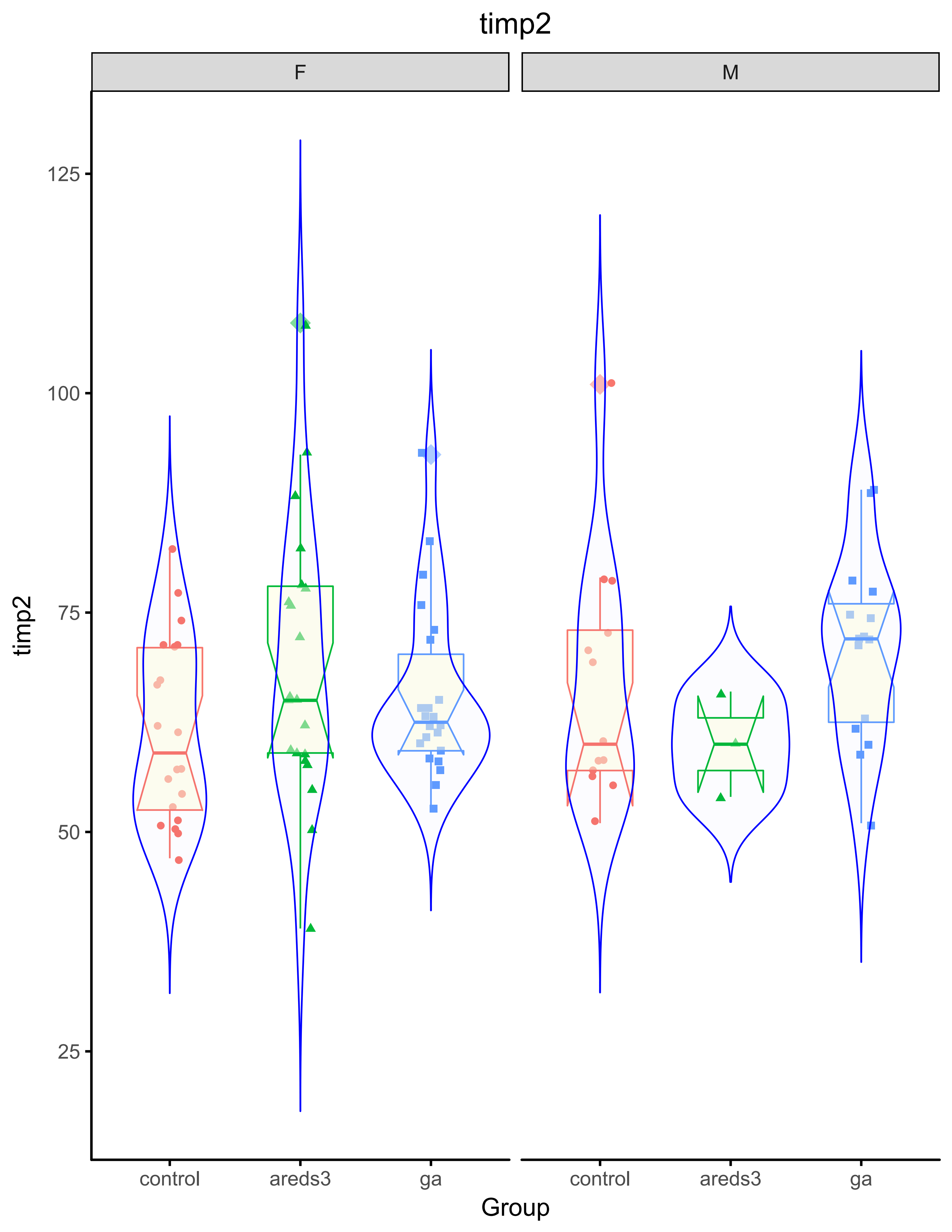
timp2
F
M
125
100
75
50
25
timp2
control
areds3
ga
control
areds3
ga
Group

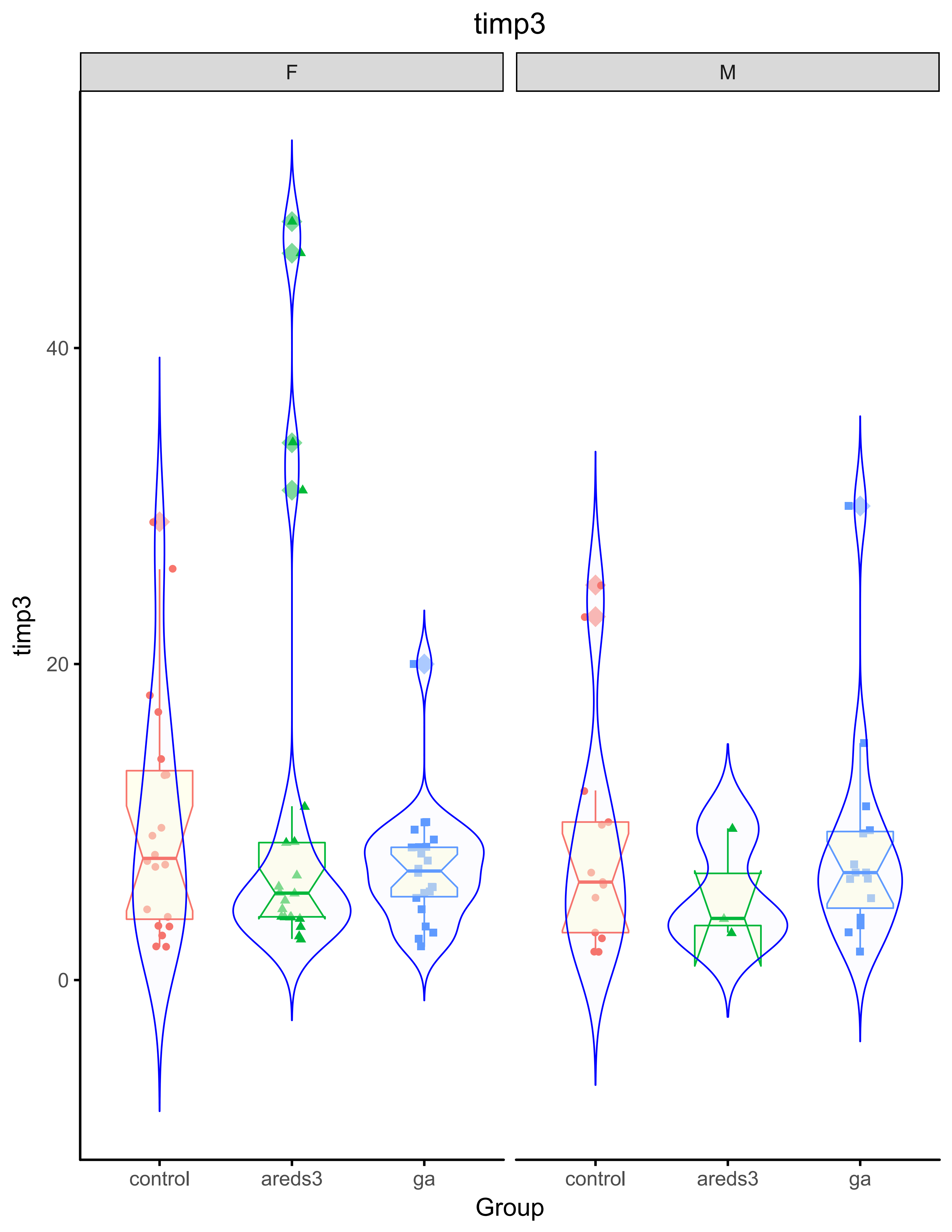
timp3
F
M
timp3
40
20
0
control
areds3
ga
control
areds3
ga
Group

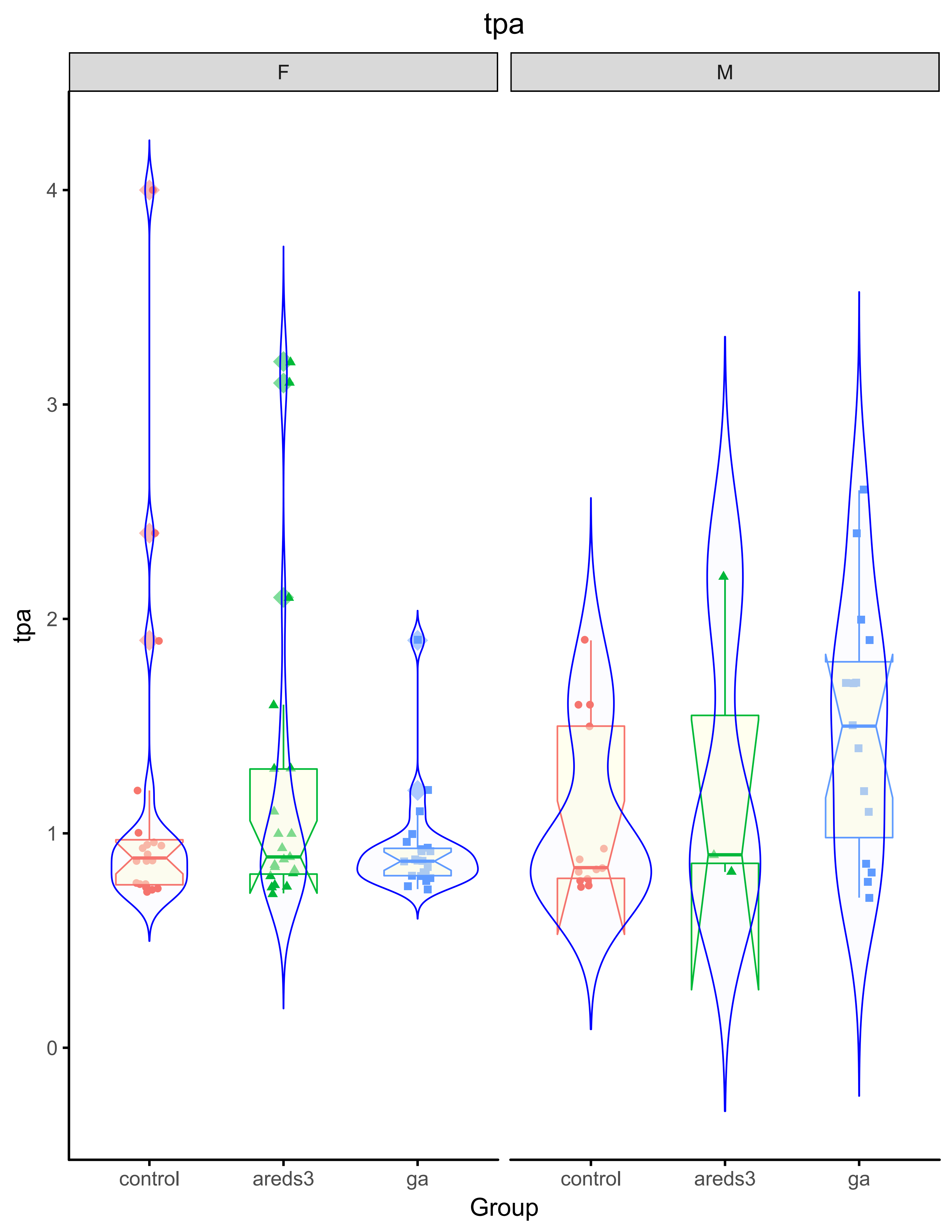
tpa
F
M
4
3
2
1
0
tpa
control
areds3
ga
control
areds3
ga
Group

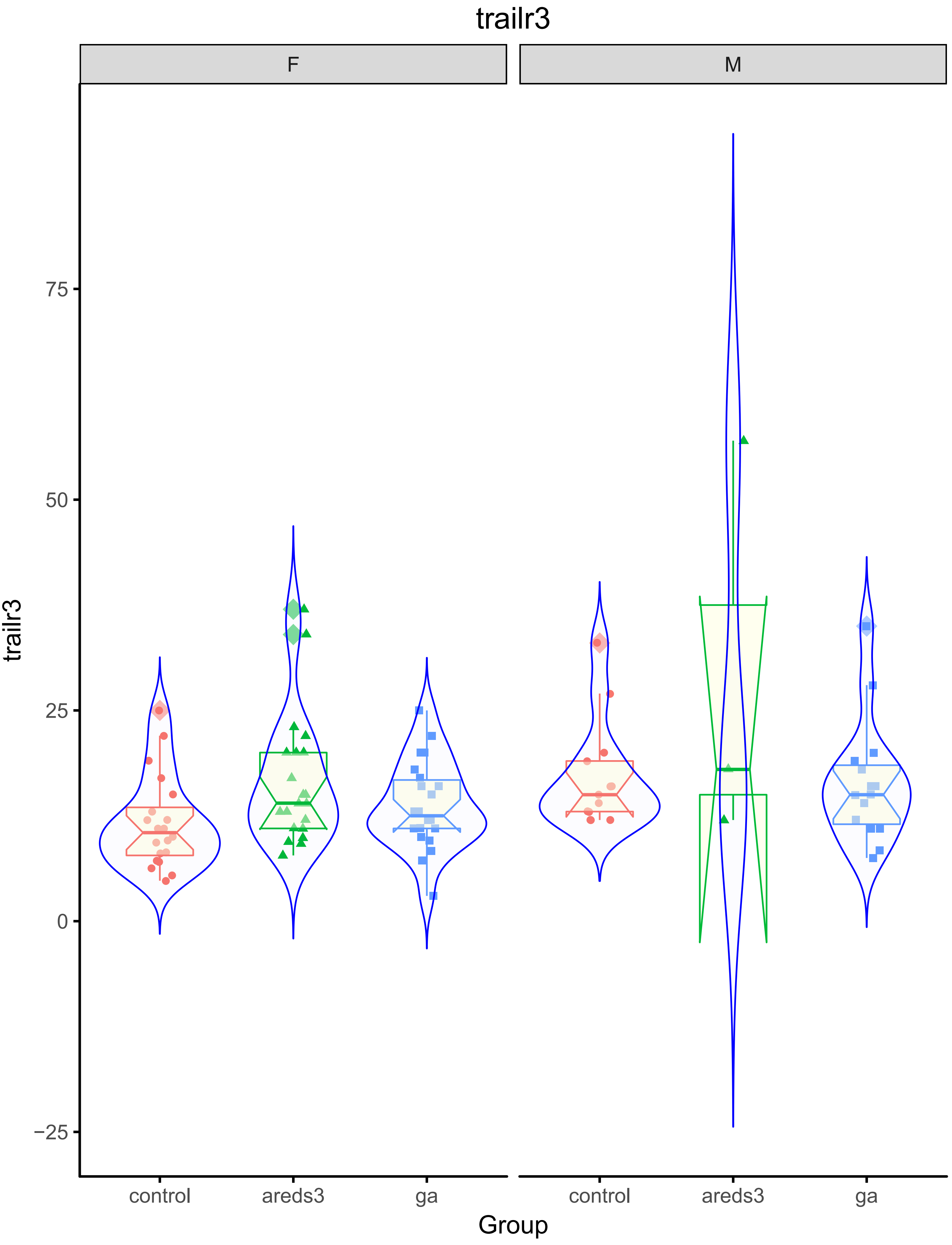
trailr3
F
M
75
50
25
0
−25
trailr3
control
areds3
ga
control
areds3
ga
Group

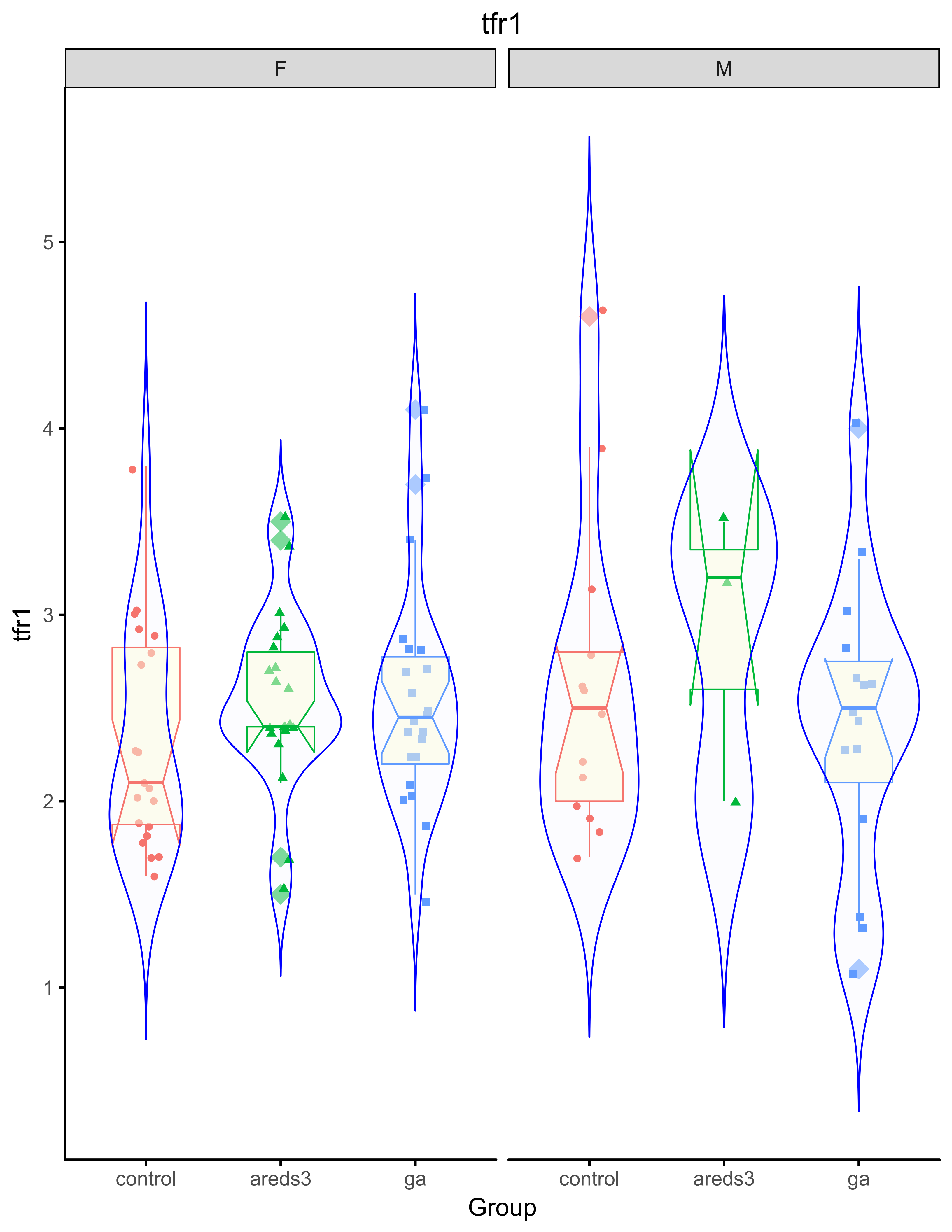
tfr1
F
M
5
4
3
2
1
tfr1
control
areds3
ga
control
areds3
ga
Group

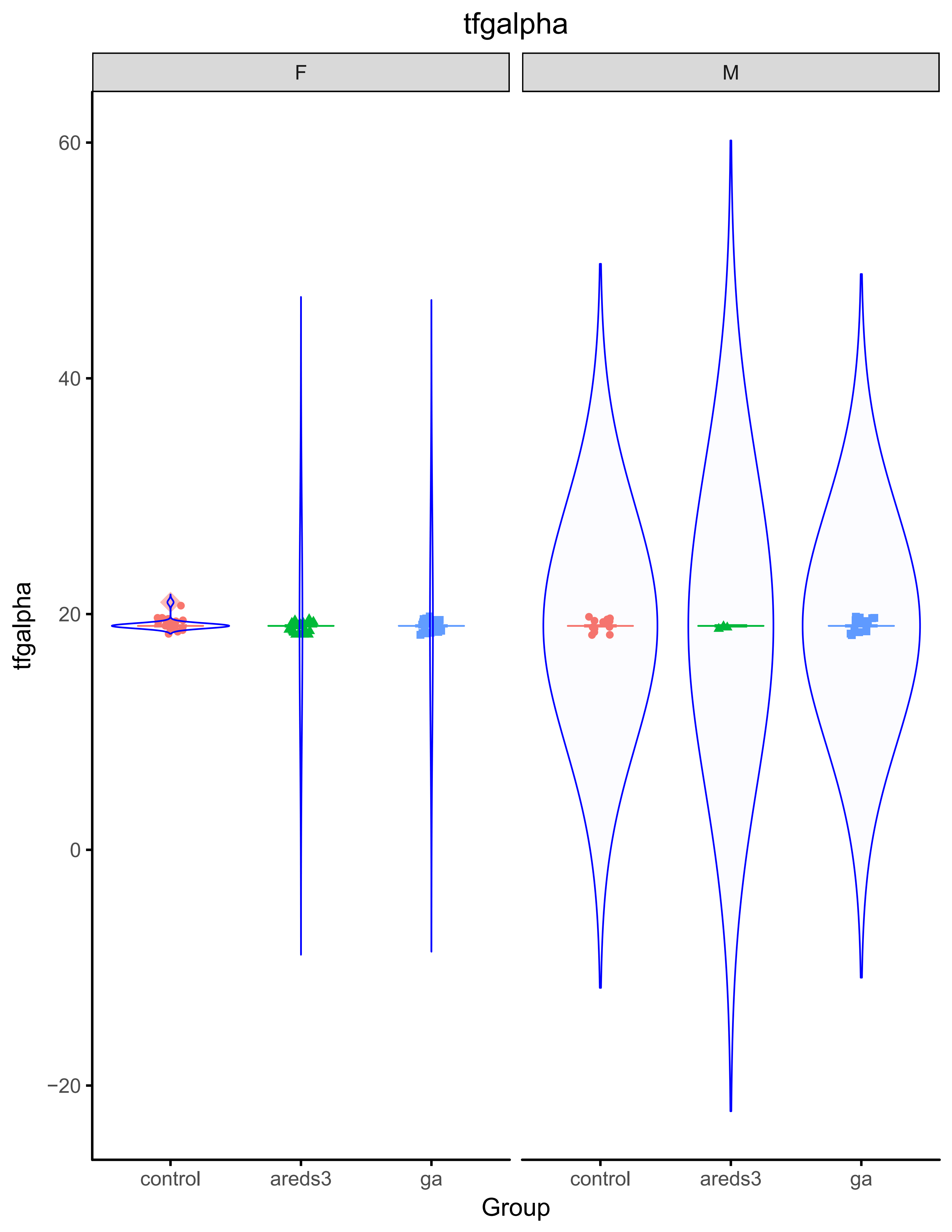

tfgalpha
F
M
60
40
20
0
−20
tfgalpha
control
areds3
ga
control
areds3
ga
Group

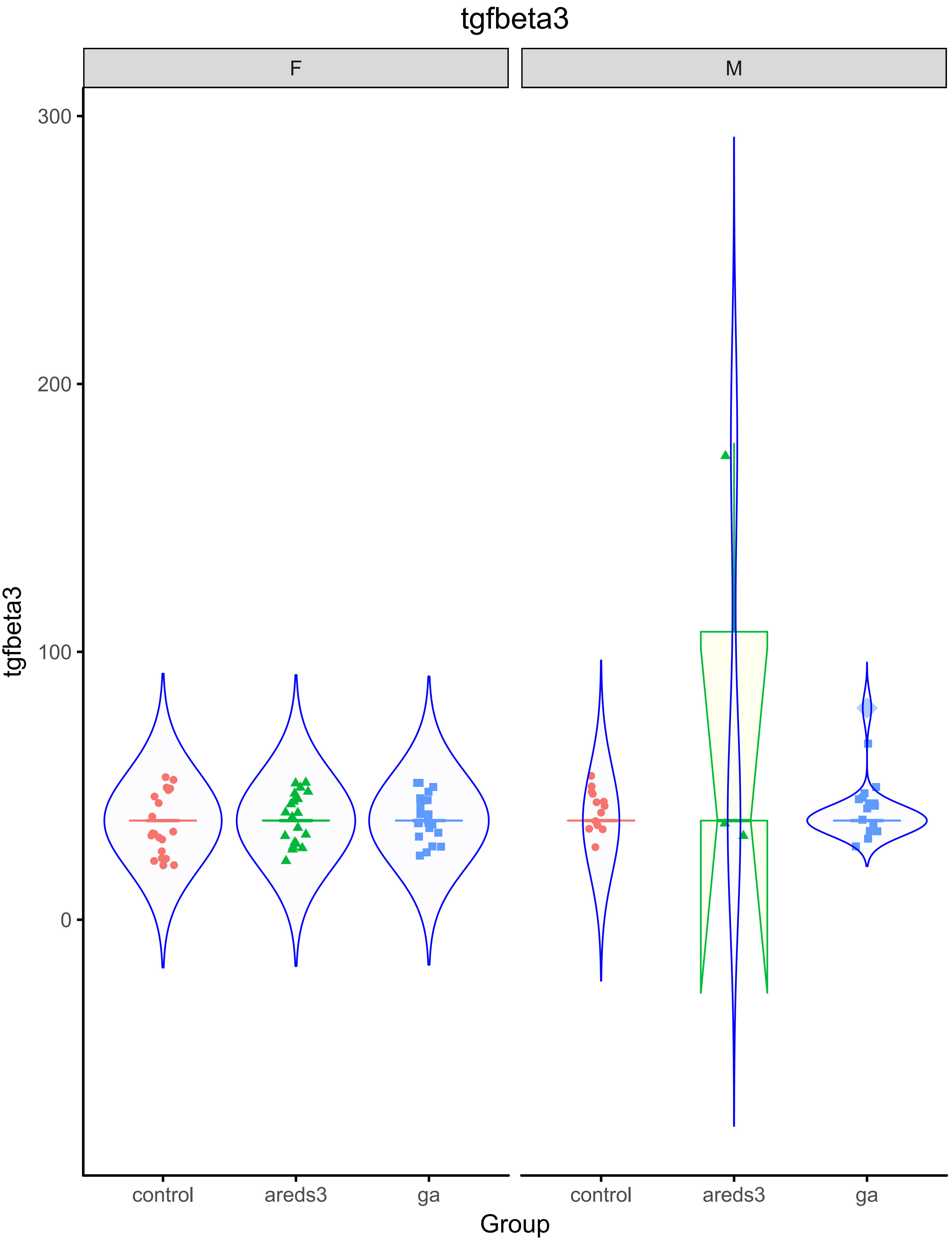

tgfbeta3
F
M
300
200
100
0
tgfbeta3
control
areds3
ga
control
areds3
ga
Group

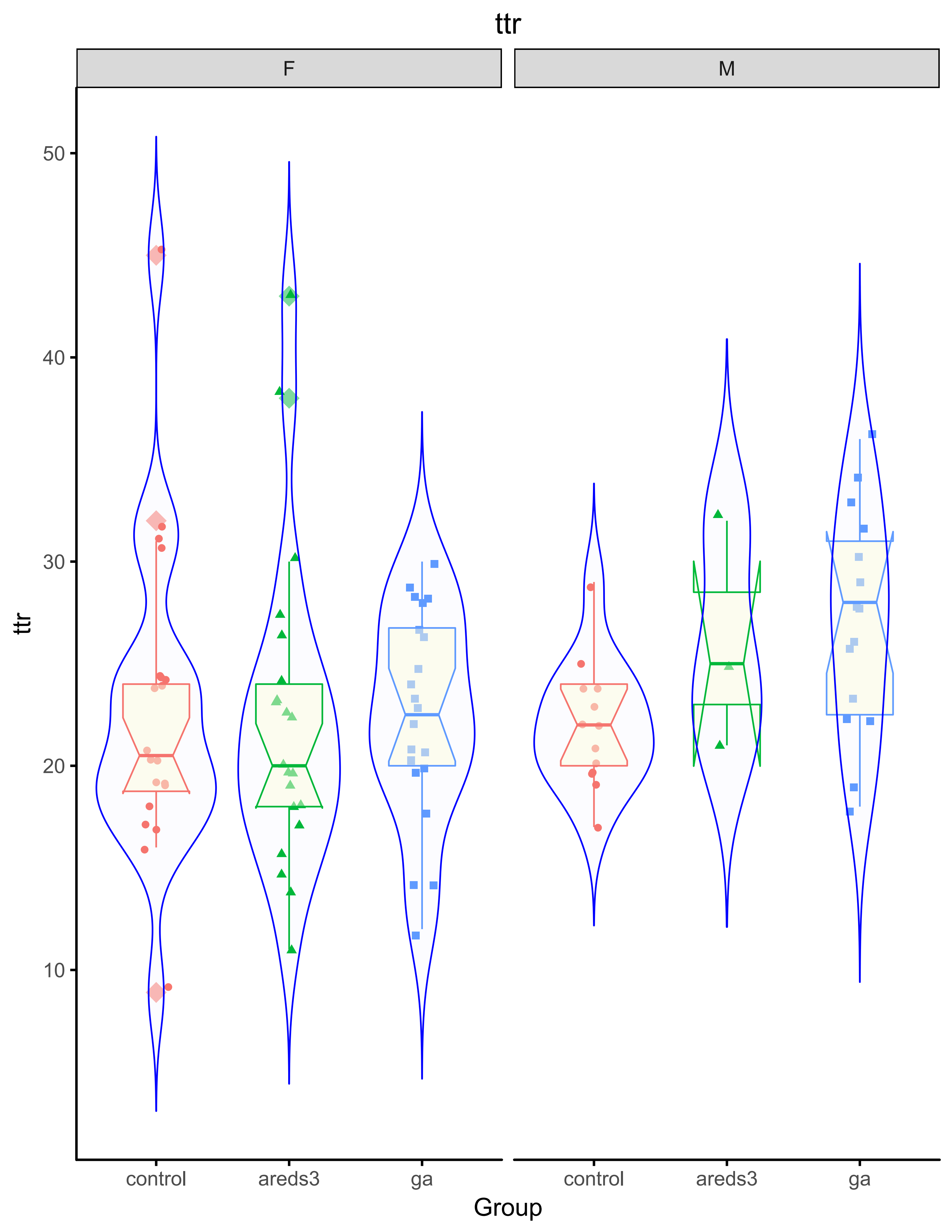
ttr
F
M
50
40
30
20
10
ttr
control
areds3
ga
control
areds3
ga
Group

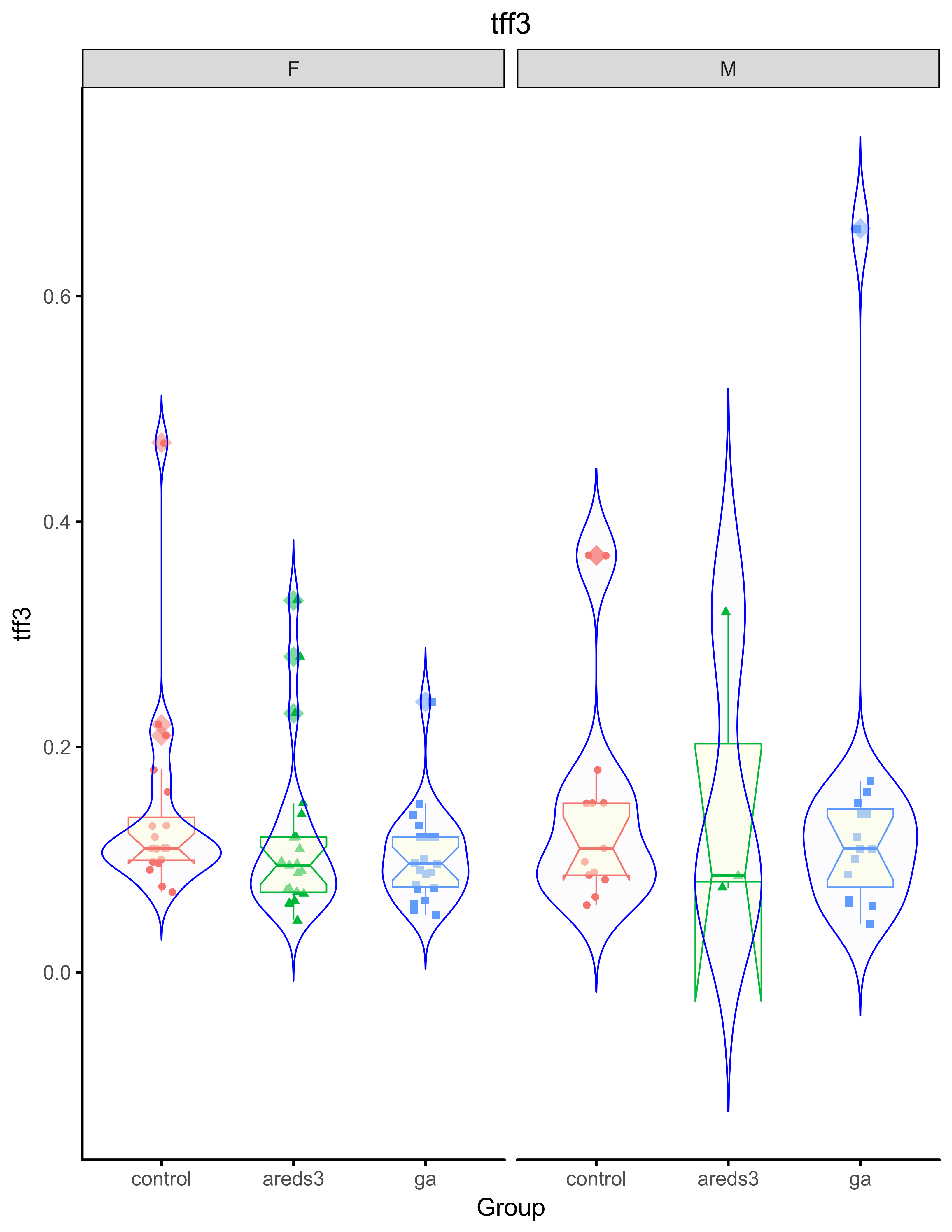
tff3
F
M
0.6
0.4
0.2
0.0
tff3
control
areds3
ga
control
areds3
ga
Group

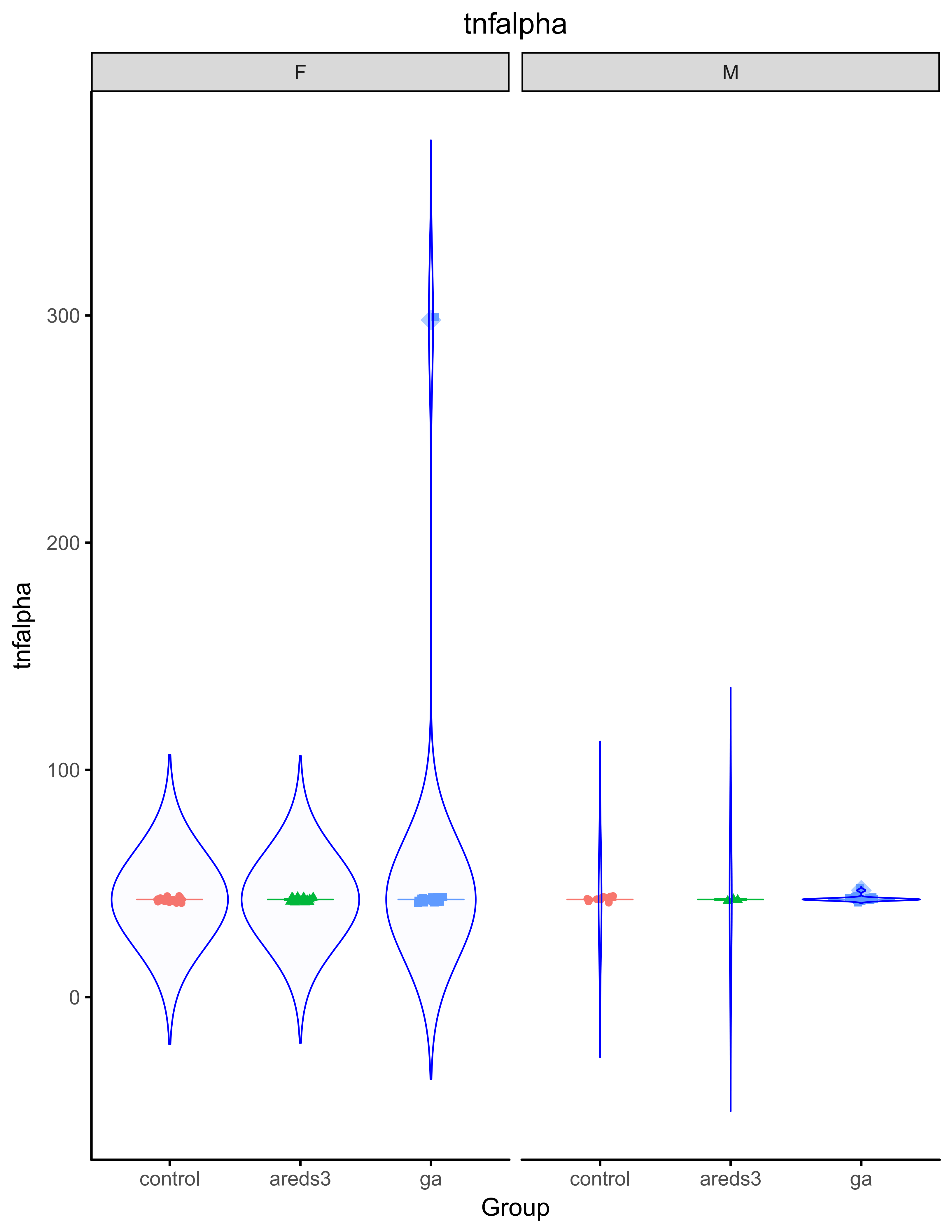

tnfalpha
F
M
300
200
100
0
tnfalpha
control
areds3
ga
control
areds3
ga
Group

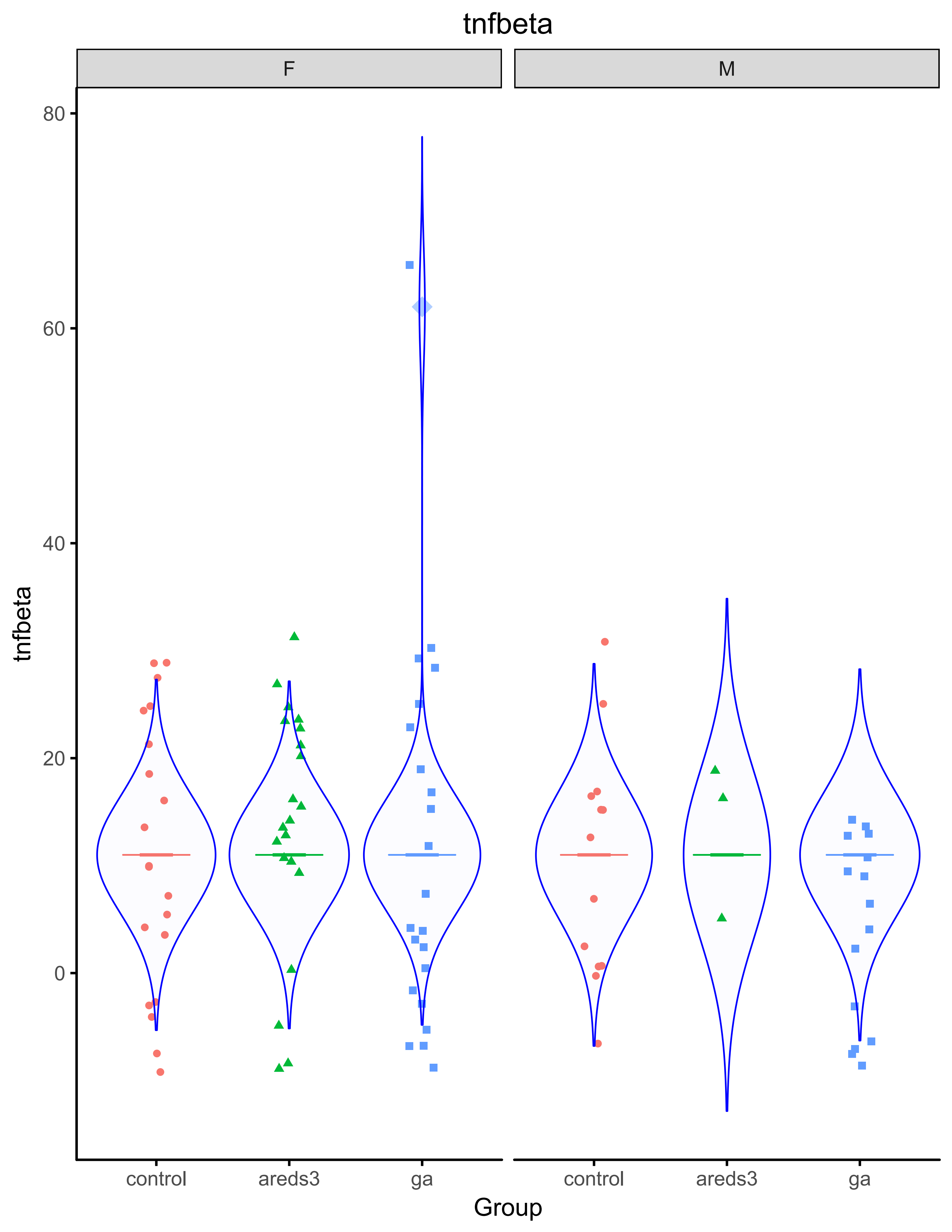

tnfbeta
F
M
80
60
40
20
0
tnfbeta
control
areds3
ga
control
areds3
ga
Group

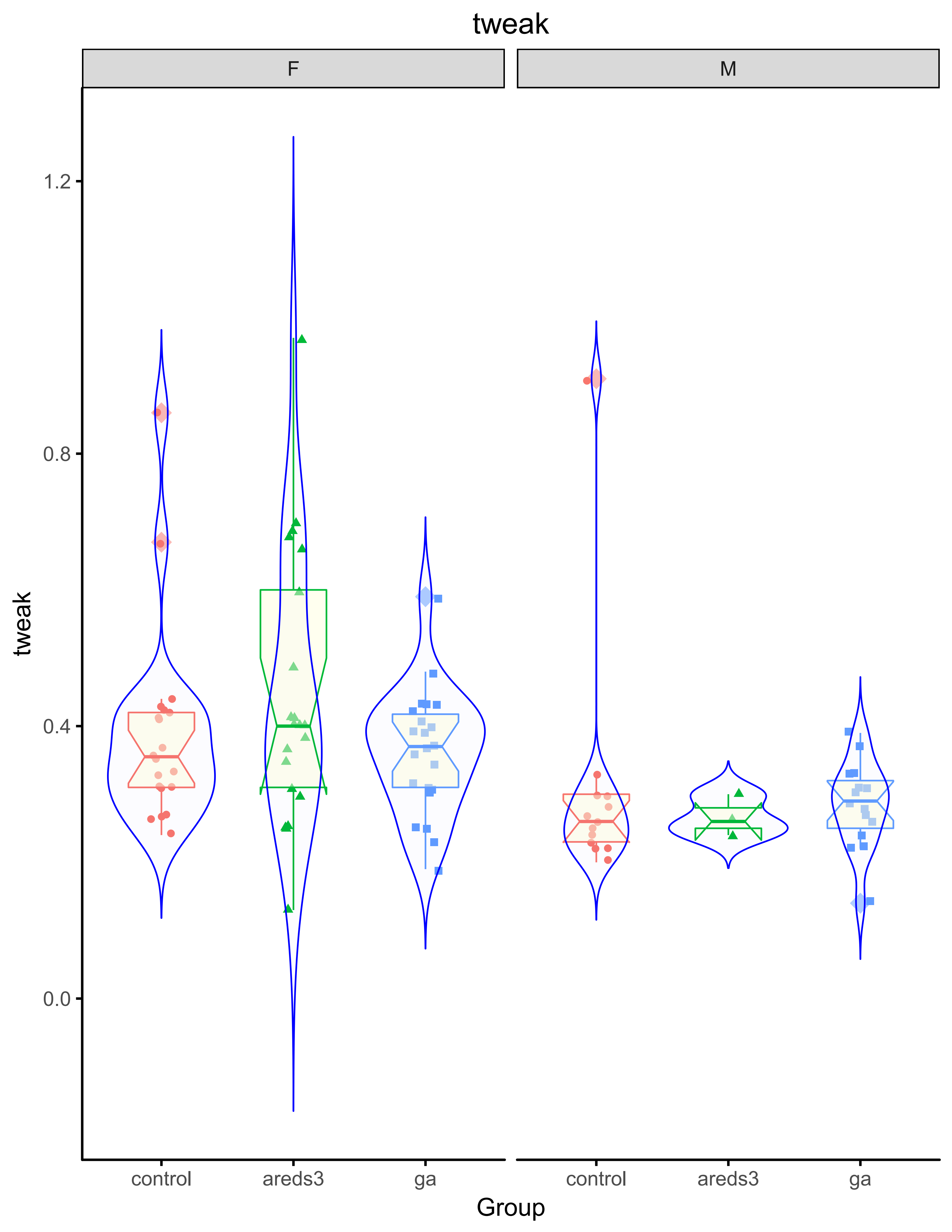

tweak
F
M
1.2
0.8
0.4
0.0
tweak
control
areds3
ga
control
areds3
ga
Group

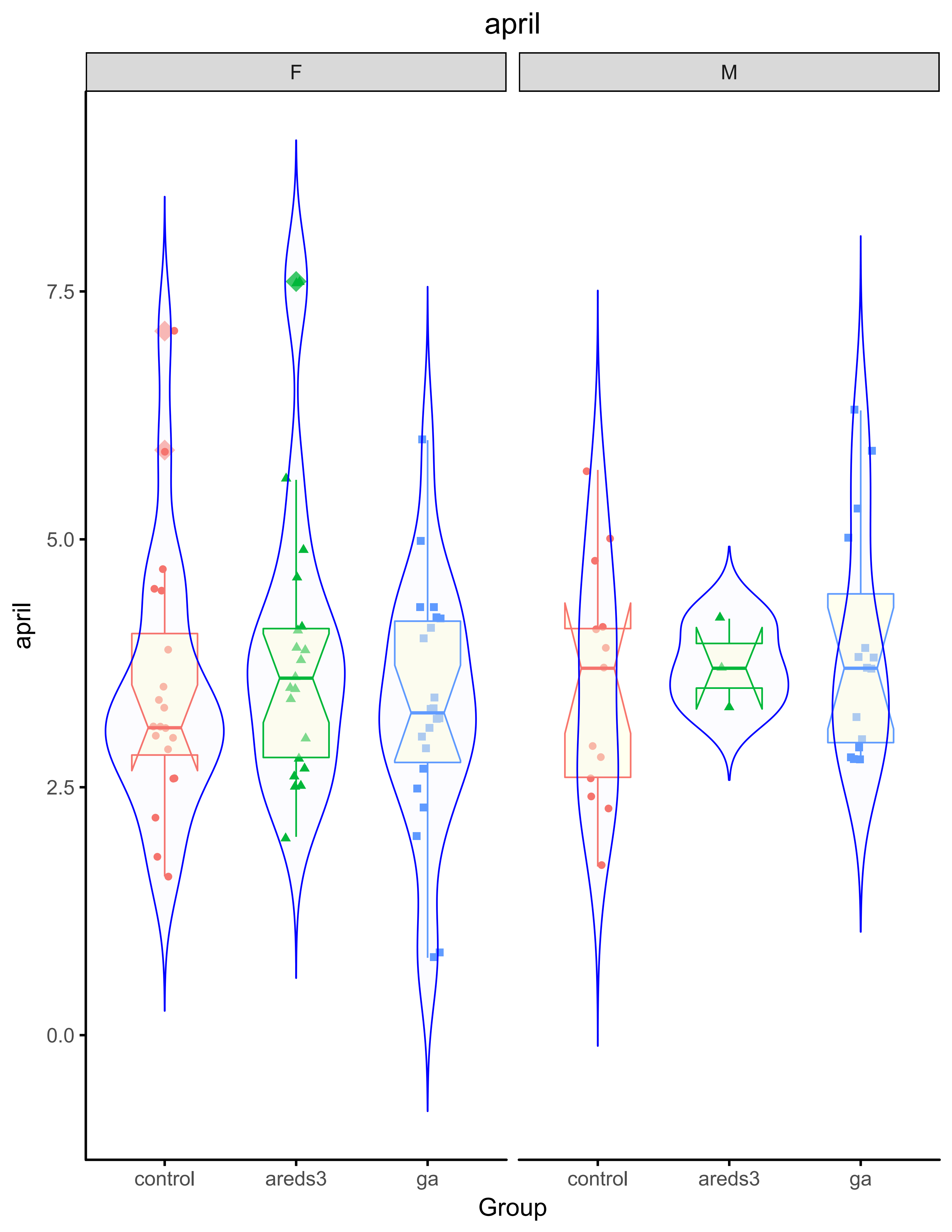
april
F
M
7.5
5.0
2.5
0.0
april
control
areds3
ga
control
areds3
ga
Group

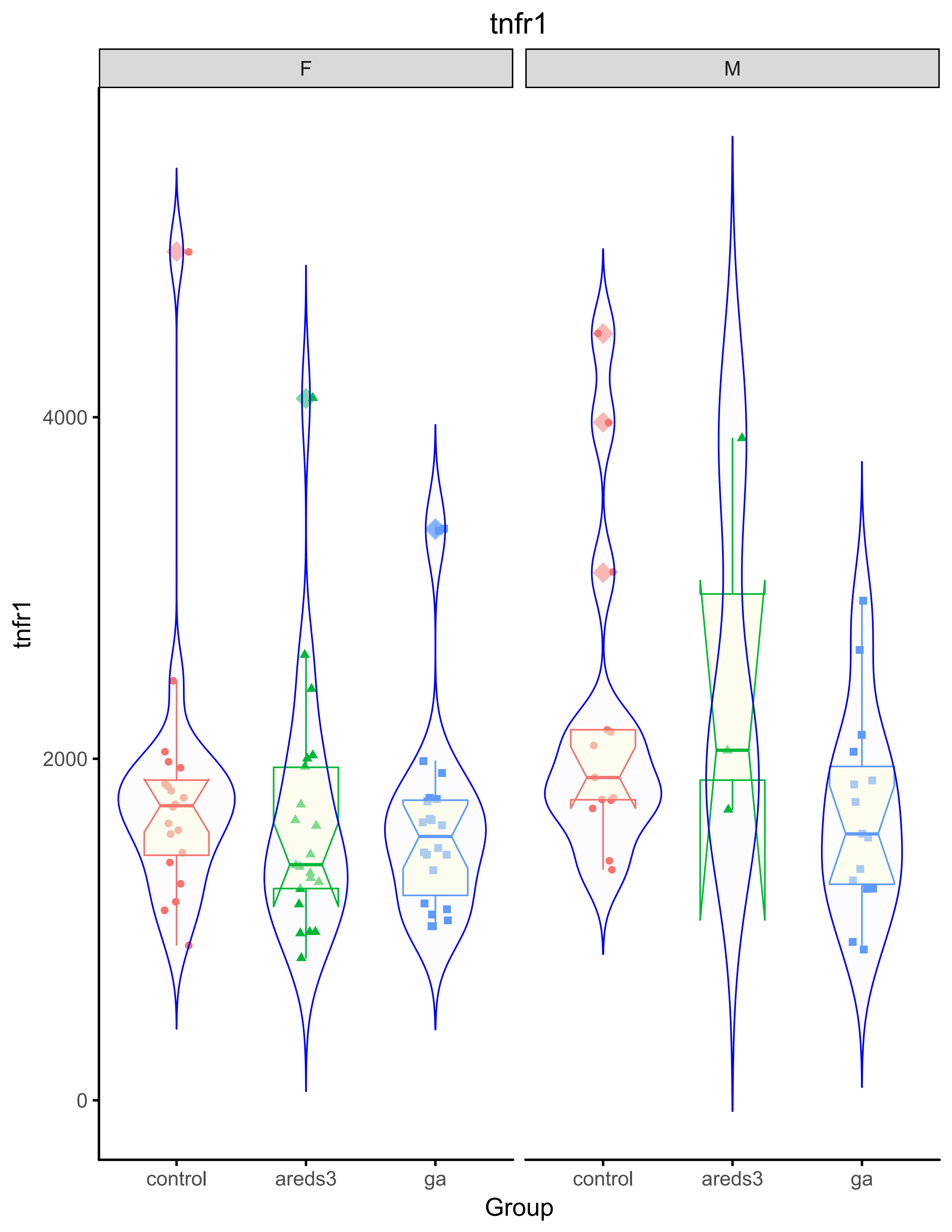

tnfr1
F
M
4000
2000
0
tnfr1
control
areds3
ga
control
areds3
ga
Group

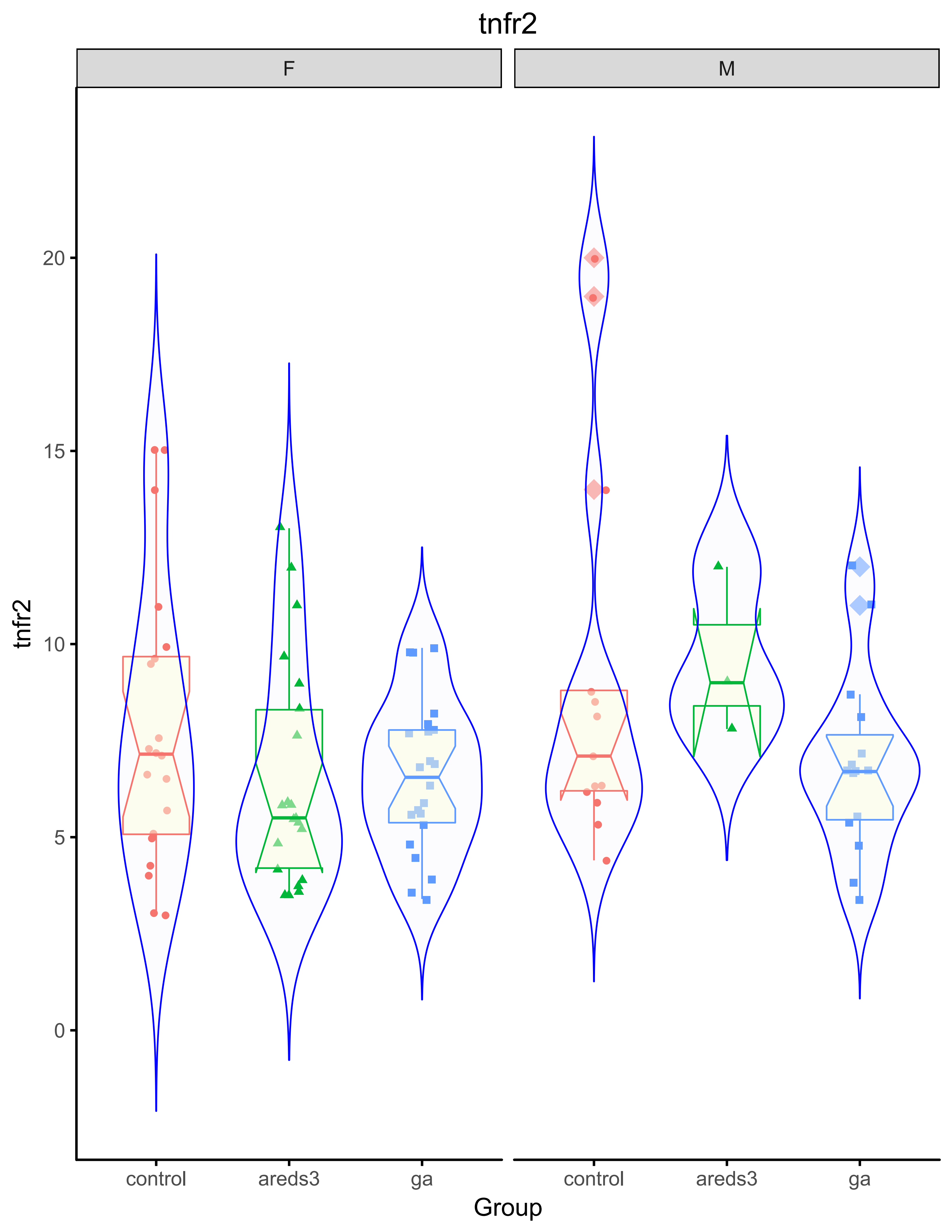

tnfr2
F
M
20
15
10
5
0
tnfr2
control
areds3
ga
control
areds3
ga
Group

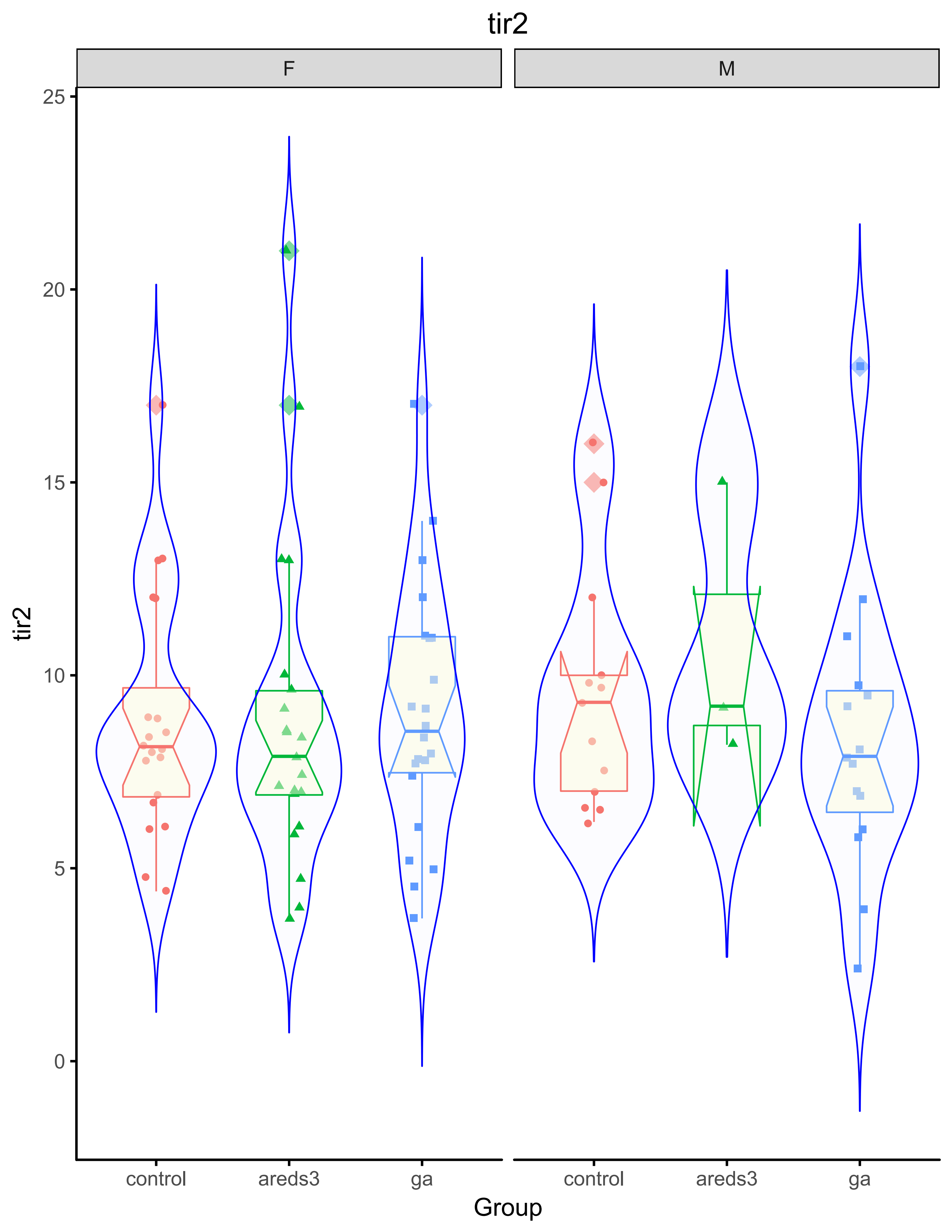

tir2
F
M
25
20
15
10
5
0
tir2
control
areds3
ga
control
areds3
ga
Group

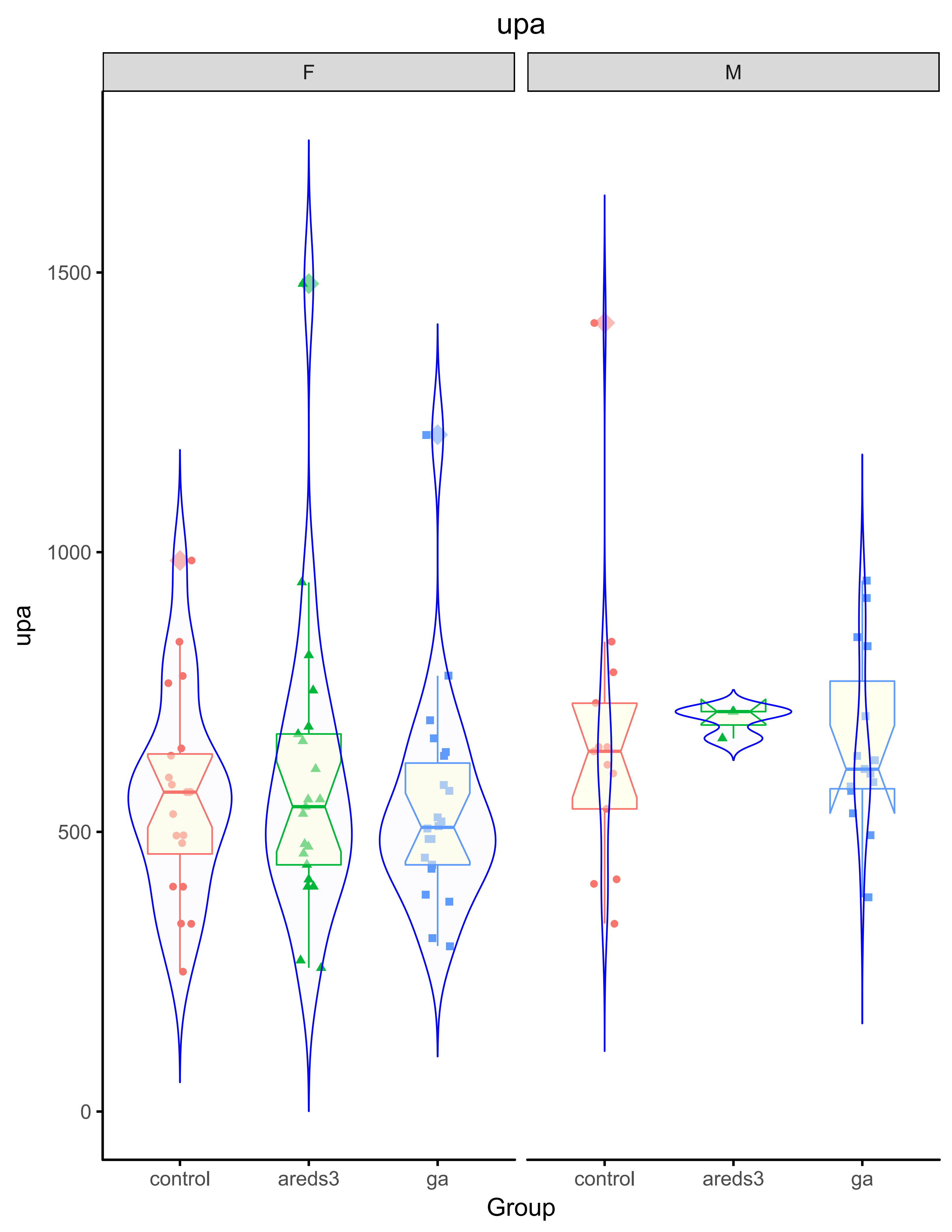

upa
F
M
1500
1000
500
0
upa
control
areds3
ga
control
areds3
ga
Group

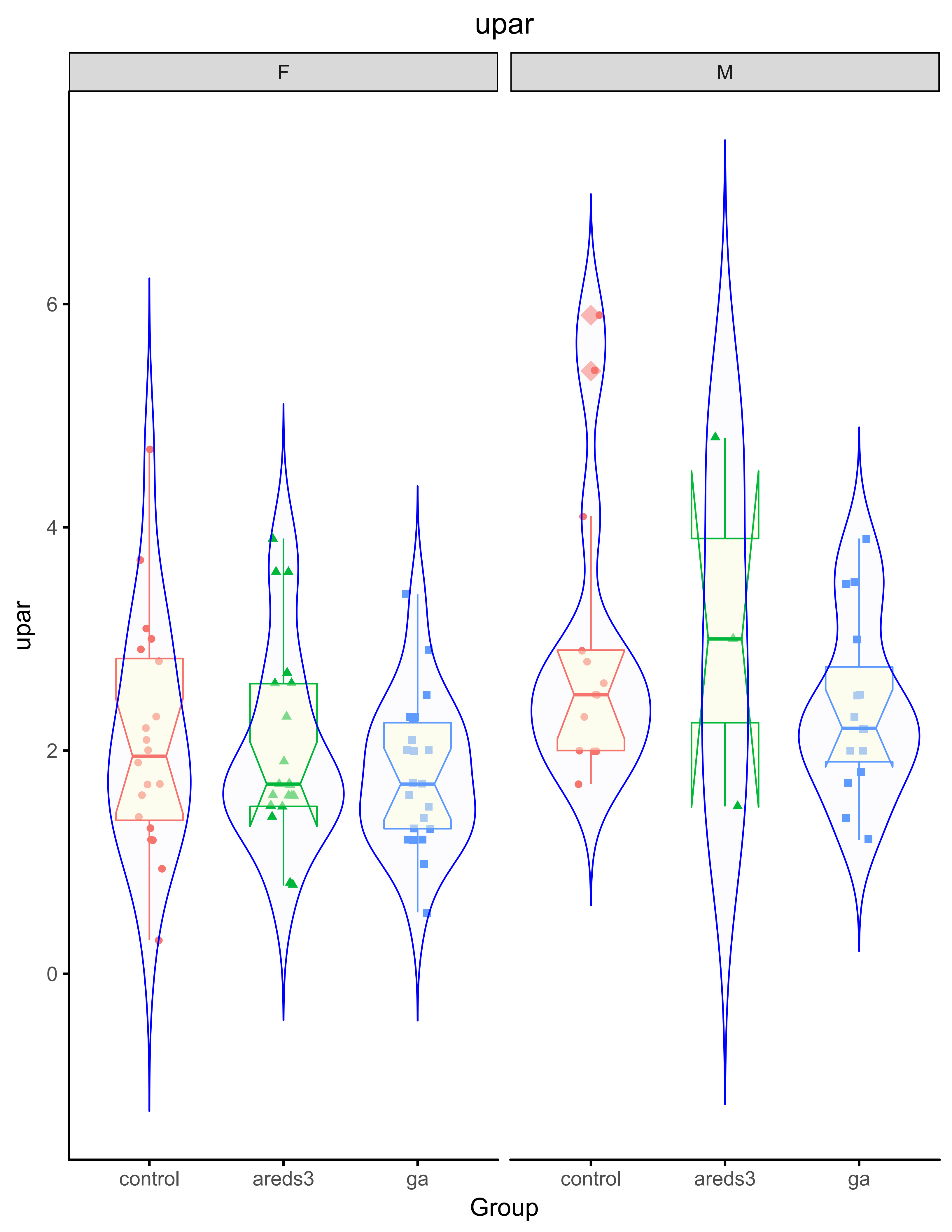

upar
F
M
6
4
2
0
upar
control
areds3
ga
control
areds3
ga
Group

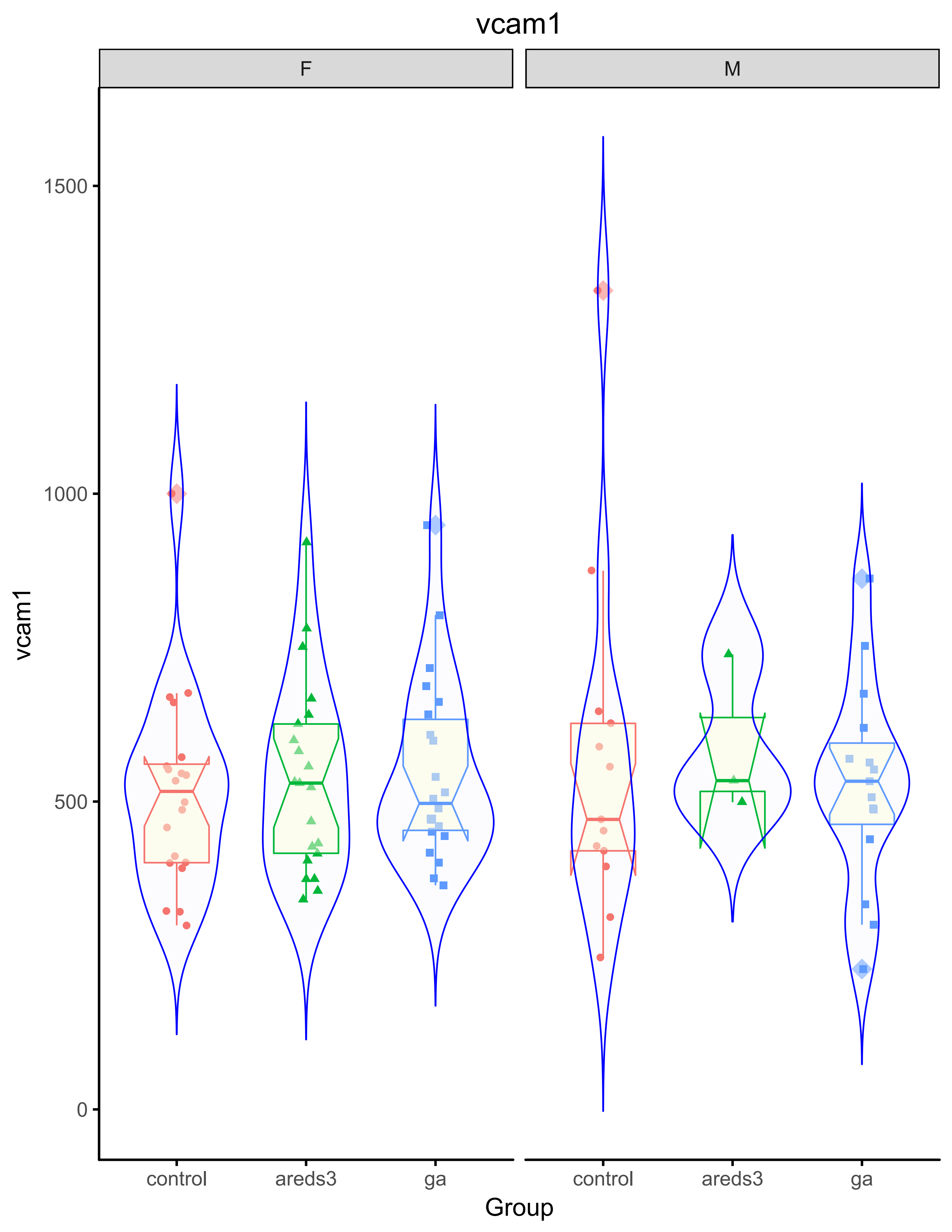

vcam1
F
M
1500
1000
500
0
vcam1
control
areds3
ga
control
areds3
ga
Group

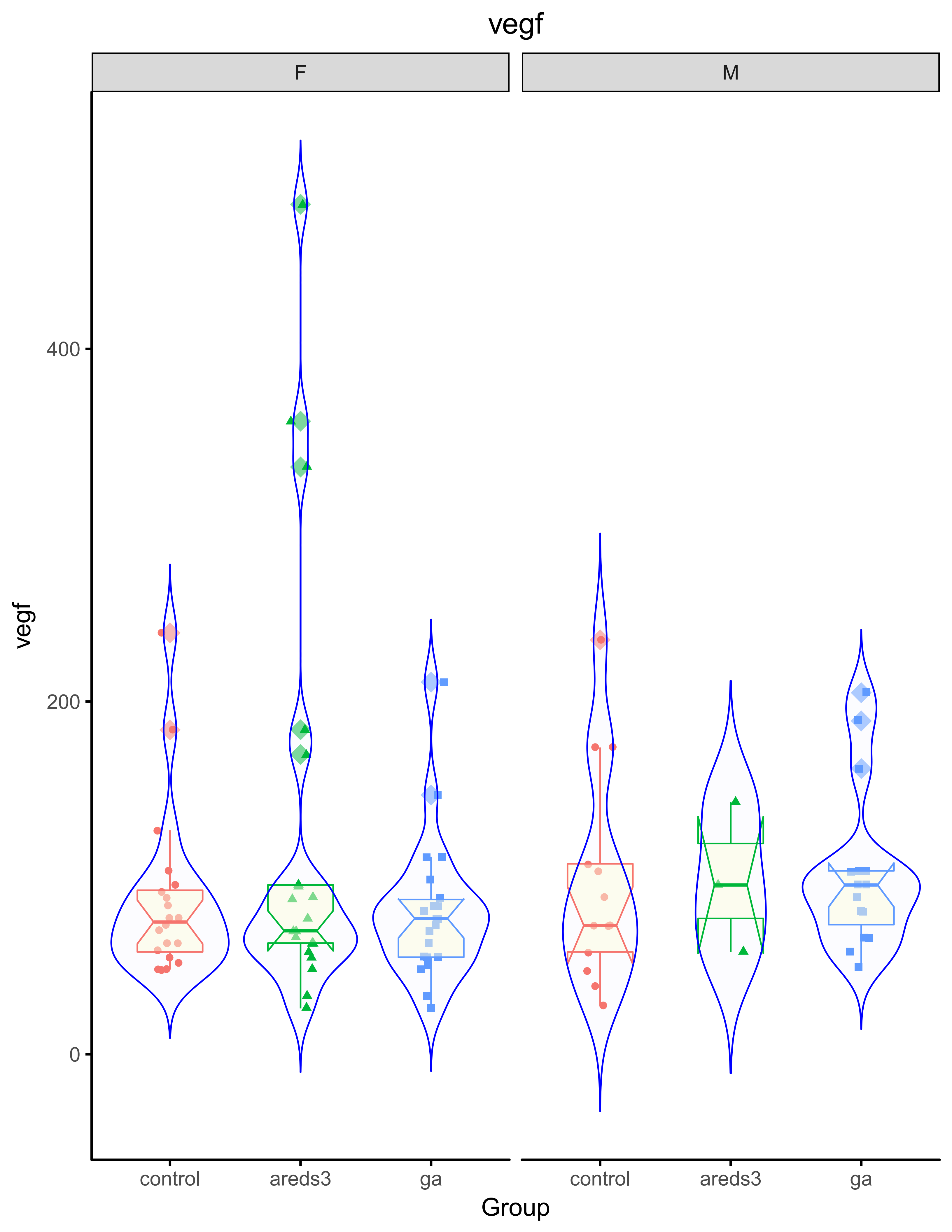
vegf
F
M
400
200
0
vegf
control
areds3
ga
control
areds3
ga
Group

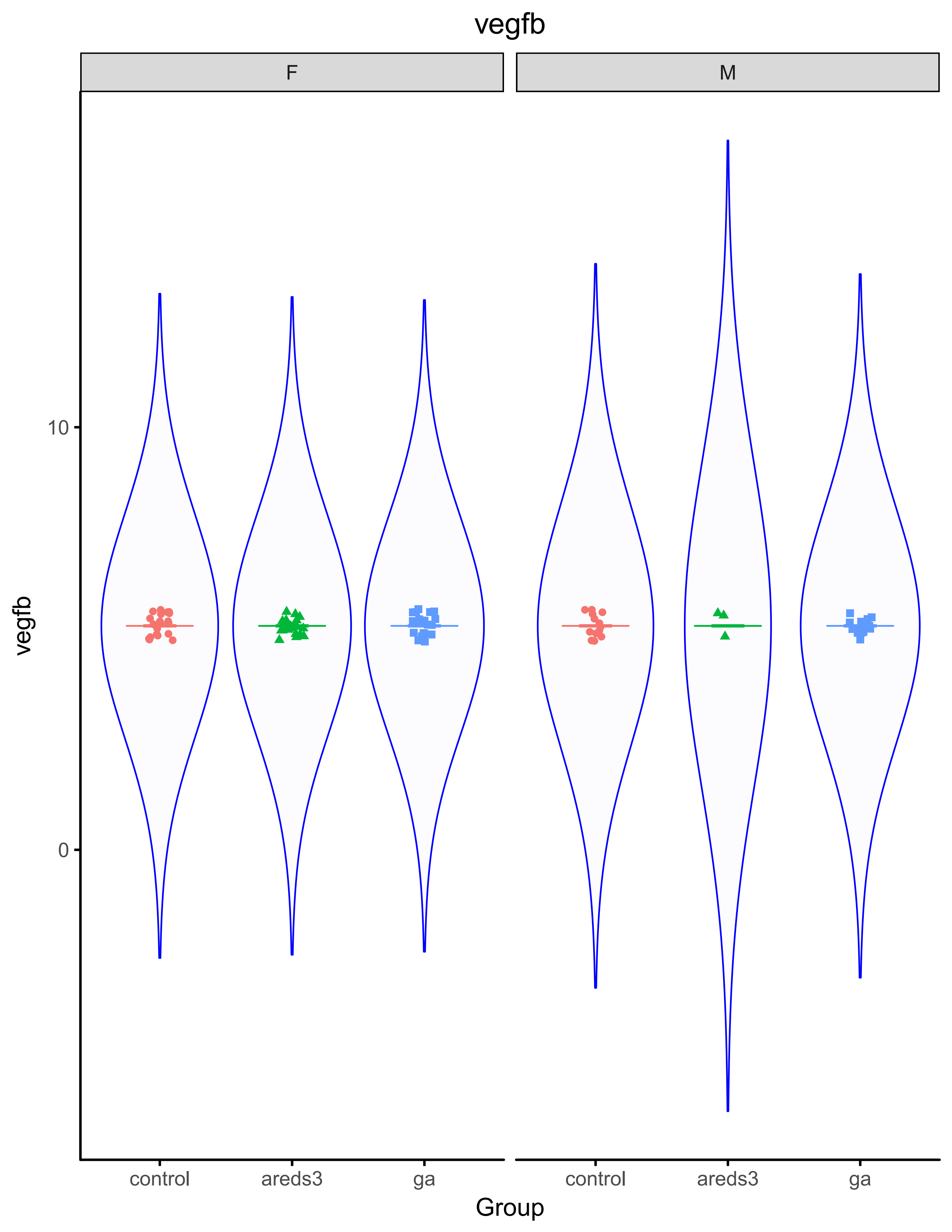

vegfb
F
M
10
0
vegfb
control
areds3
ga
control
areds3
ga
Group

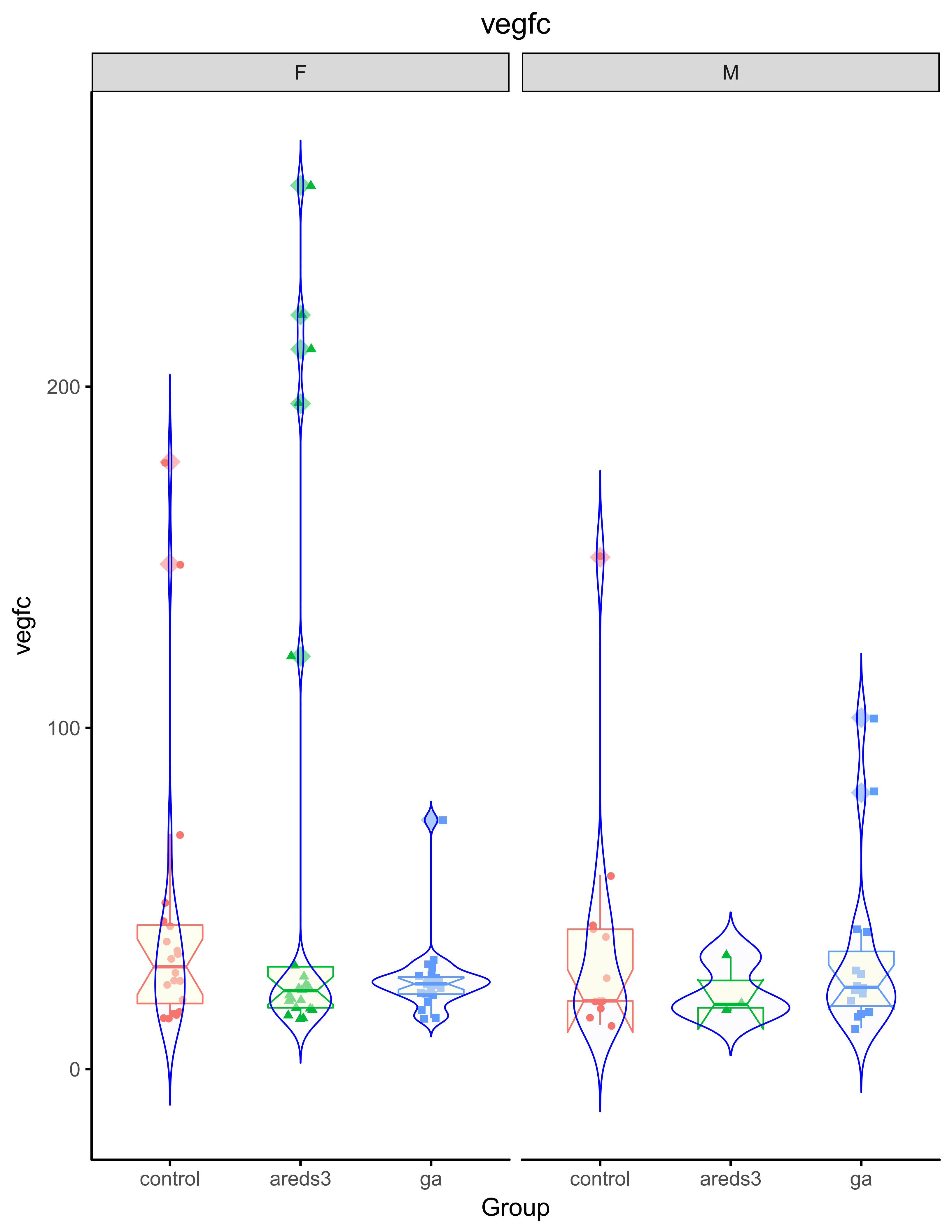
vegfc
F
M
vegfc
200
100
0
control
areds3
ga
control
areds3
ga
Group

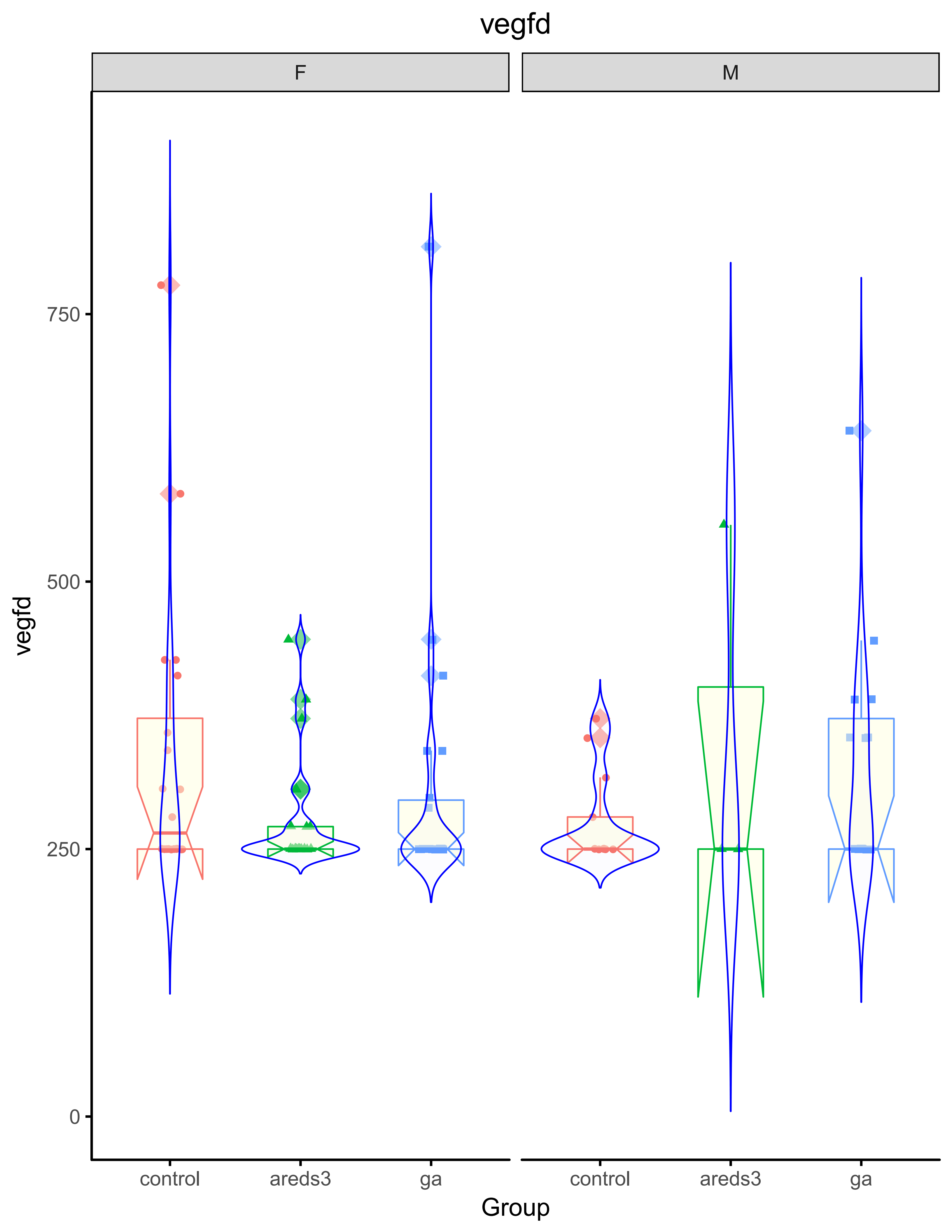

vegfd
F
M
750
500
250
0
vegfd
control
areds3
ga
control
areds3
ga
Group

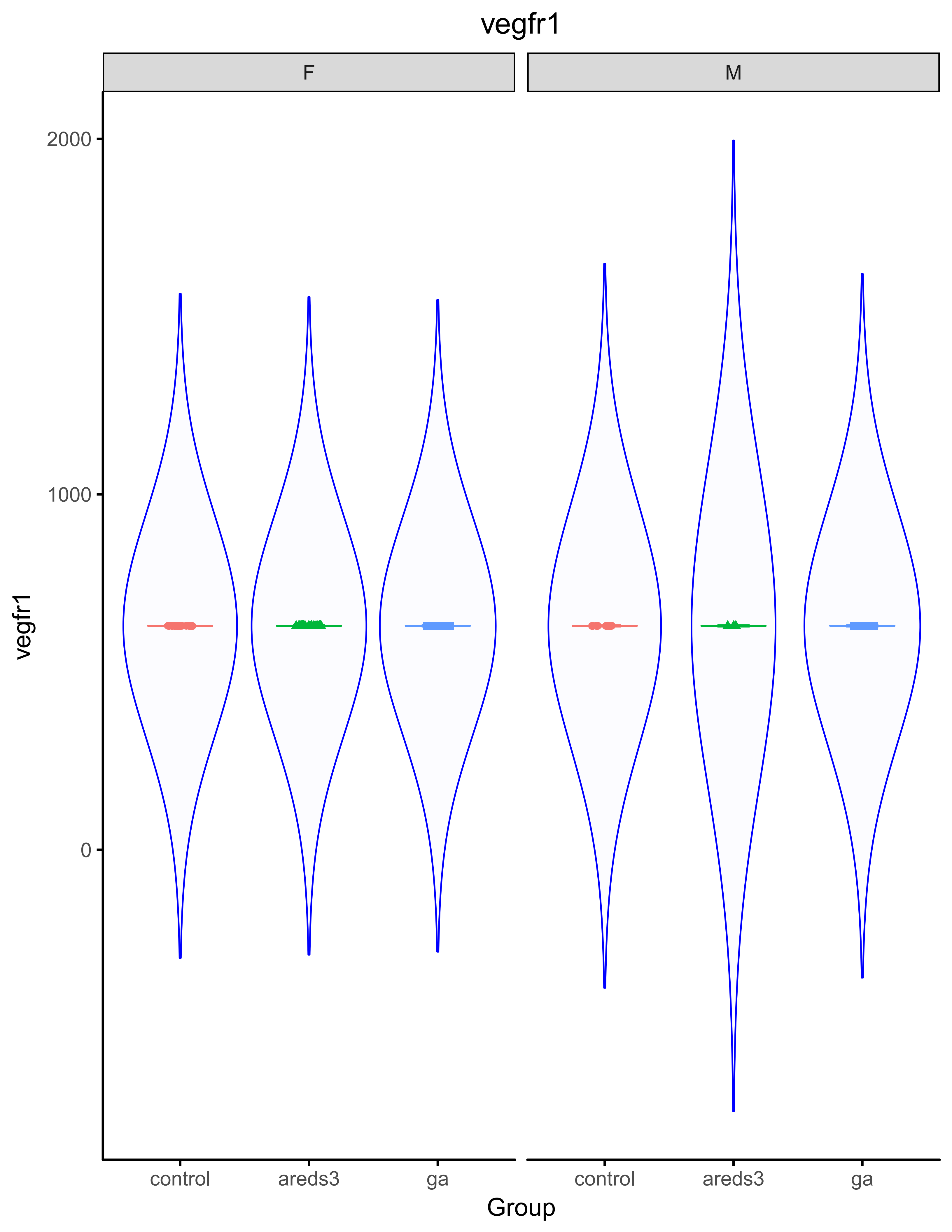
vegfr1
F
M
2000
1000
0
vegfr1
control
areds3
ga
control
areds3
ga
Group

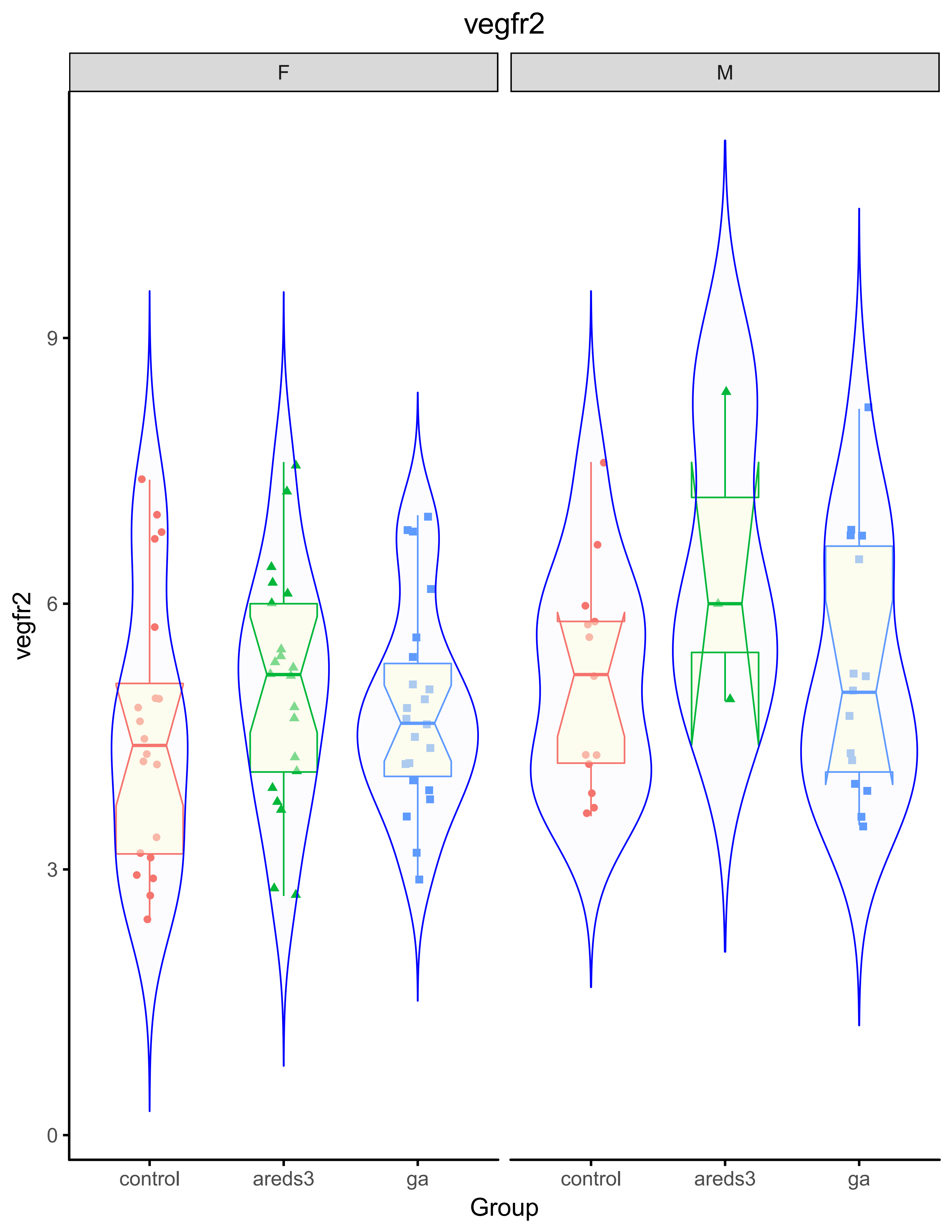

vegfr2
F
M
vegfr2
9
6
3
0
control
areds3
ga
control
areds3
ga
Group

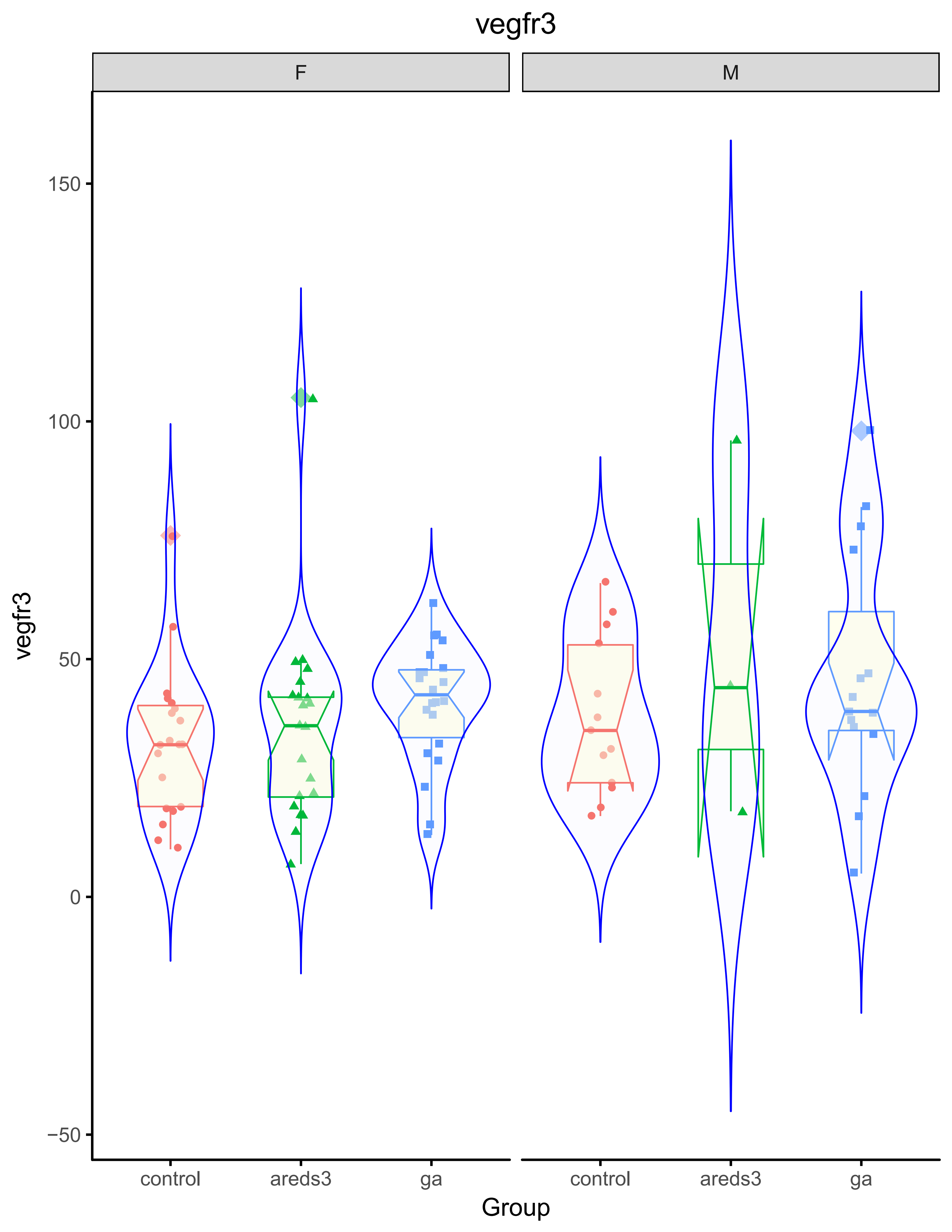
vegfr3
F
M
150
100
50
0
−50
vegfr3
control
areds3
ga
control
areds3
ga
Group

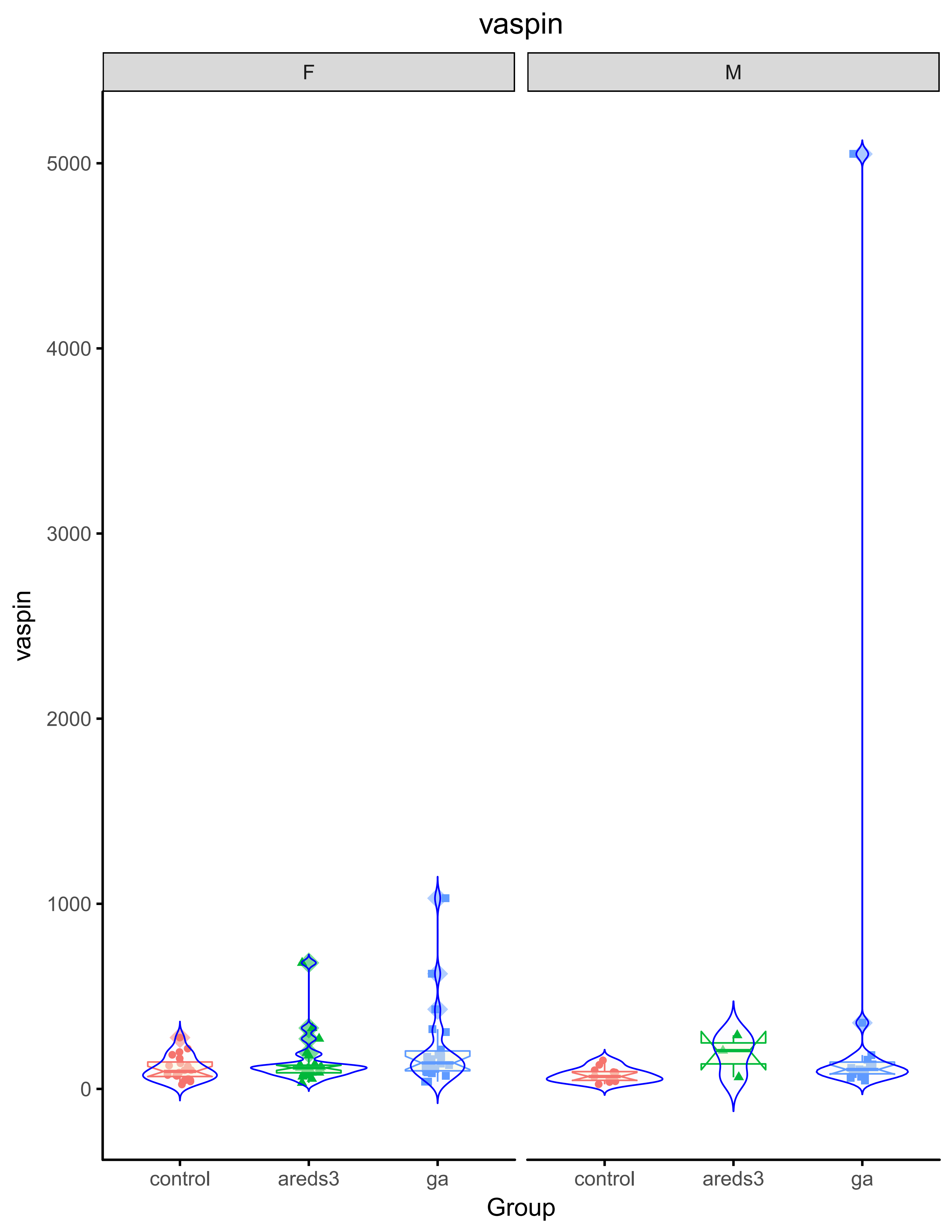

vaspin
F
M
5000
4000
3000
2000
1000
0
vaspin
control
areds3
ga
control
areds3
ga
Group

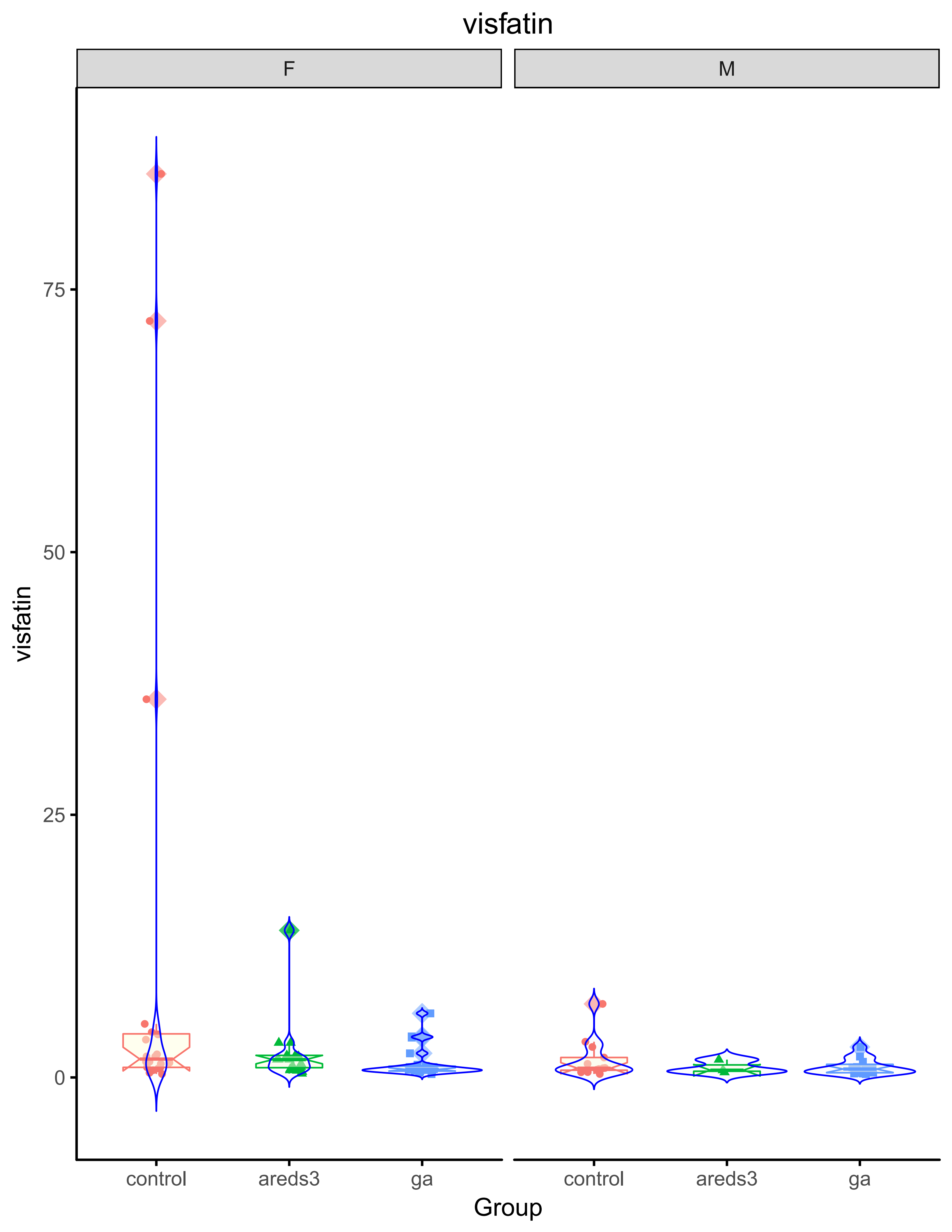

visfatin
F
M
75
50
25
0
visfatin
control
areds3
ga
control
areds3
ga
Group

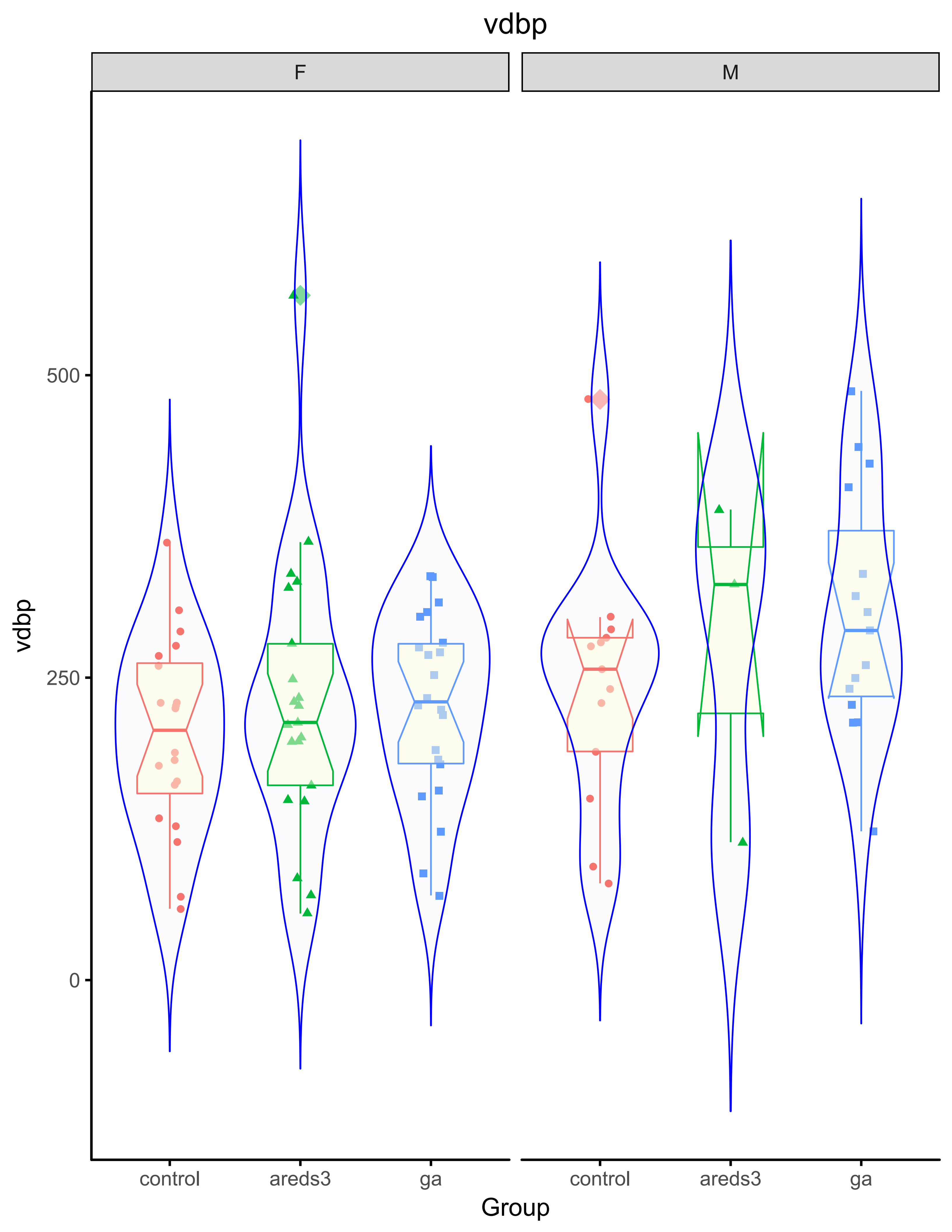
vdbp
F
M
500
250
0
vdbp
control
areds3
ga
control
areds3
ga
Group

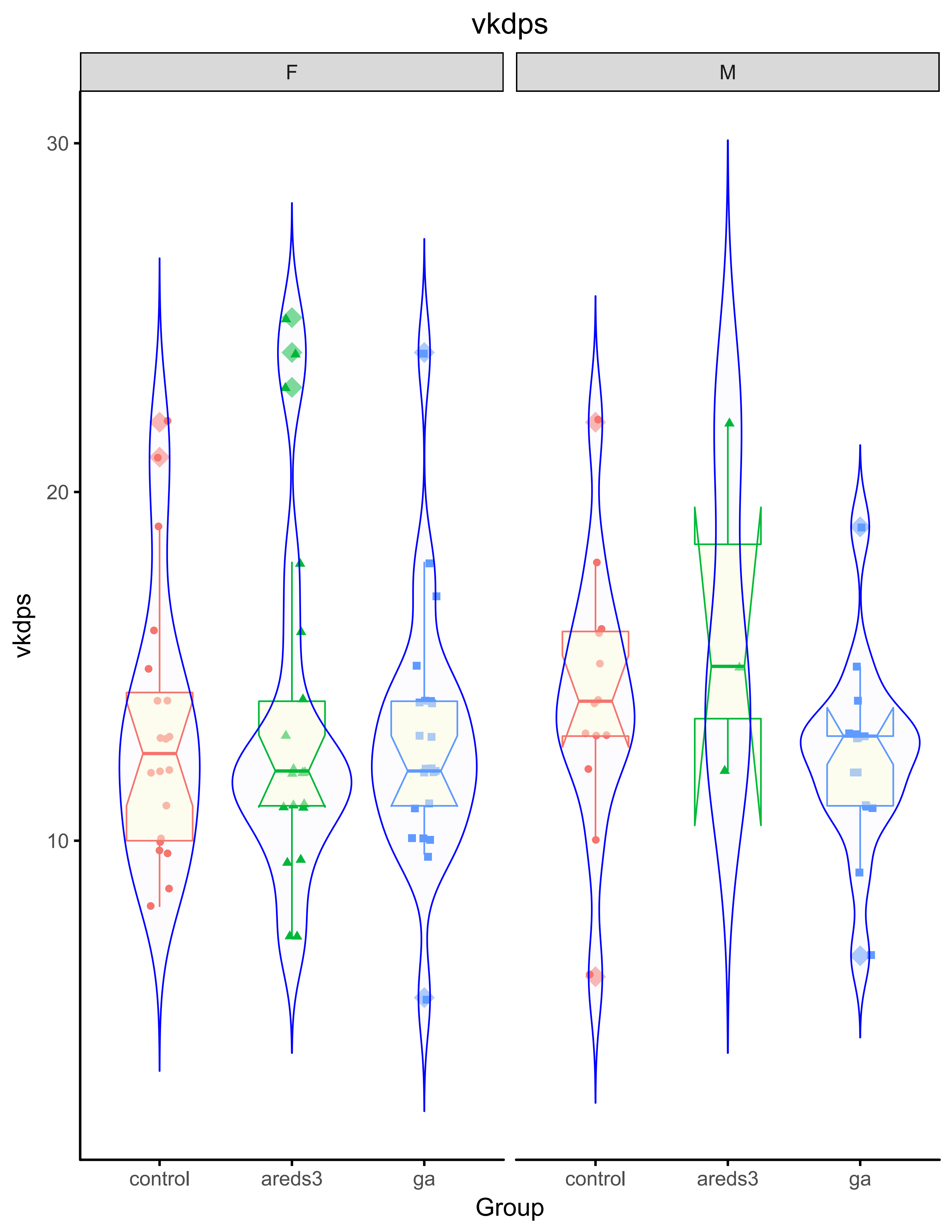

vkdps
F
M
30
20
10
vkdps
control
areds3
ga
control
areds3
ga
Group

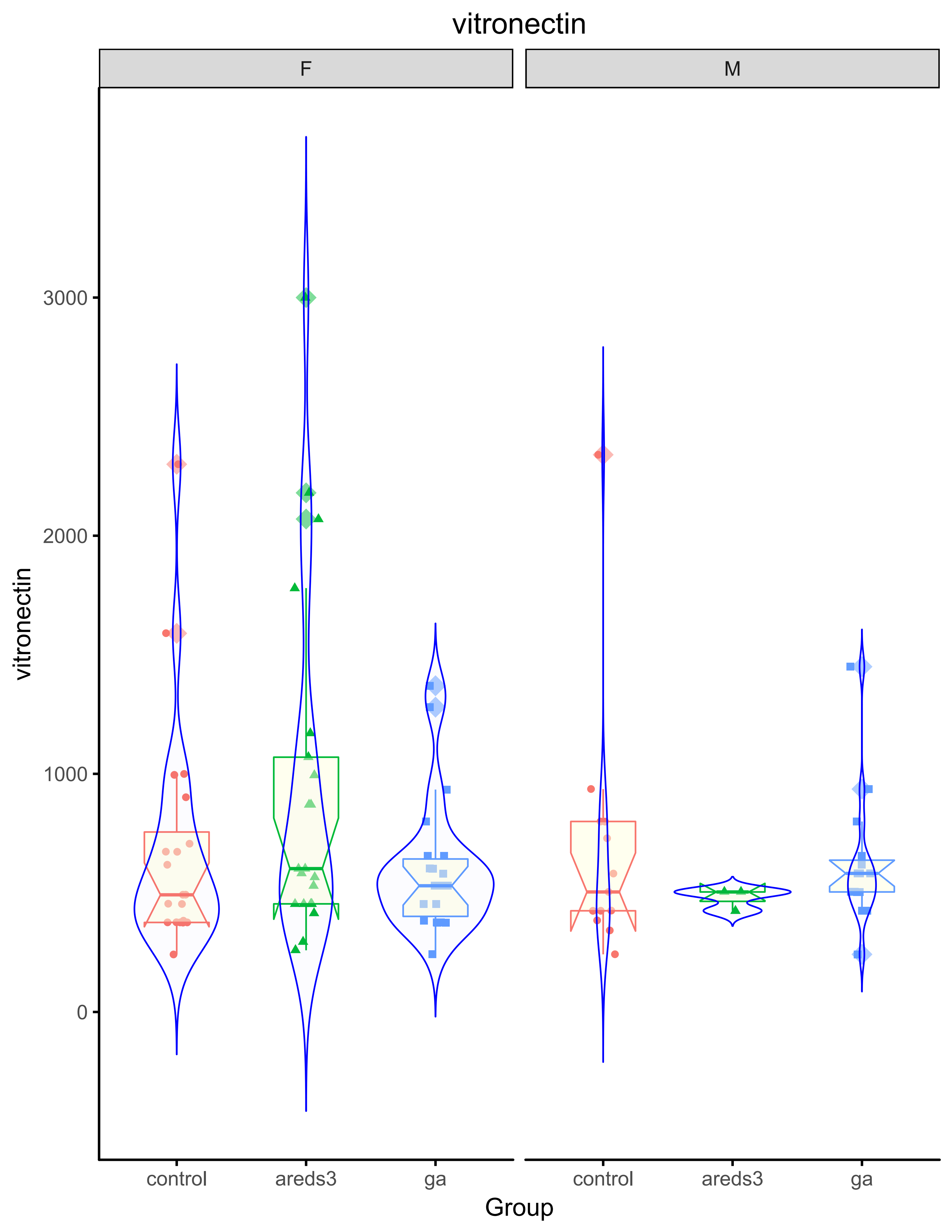

vitronectin
F
M
3000
2000
1000
0
vitronectin
control
areds3
ga
control
areds3
ga
Group

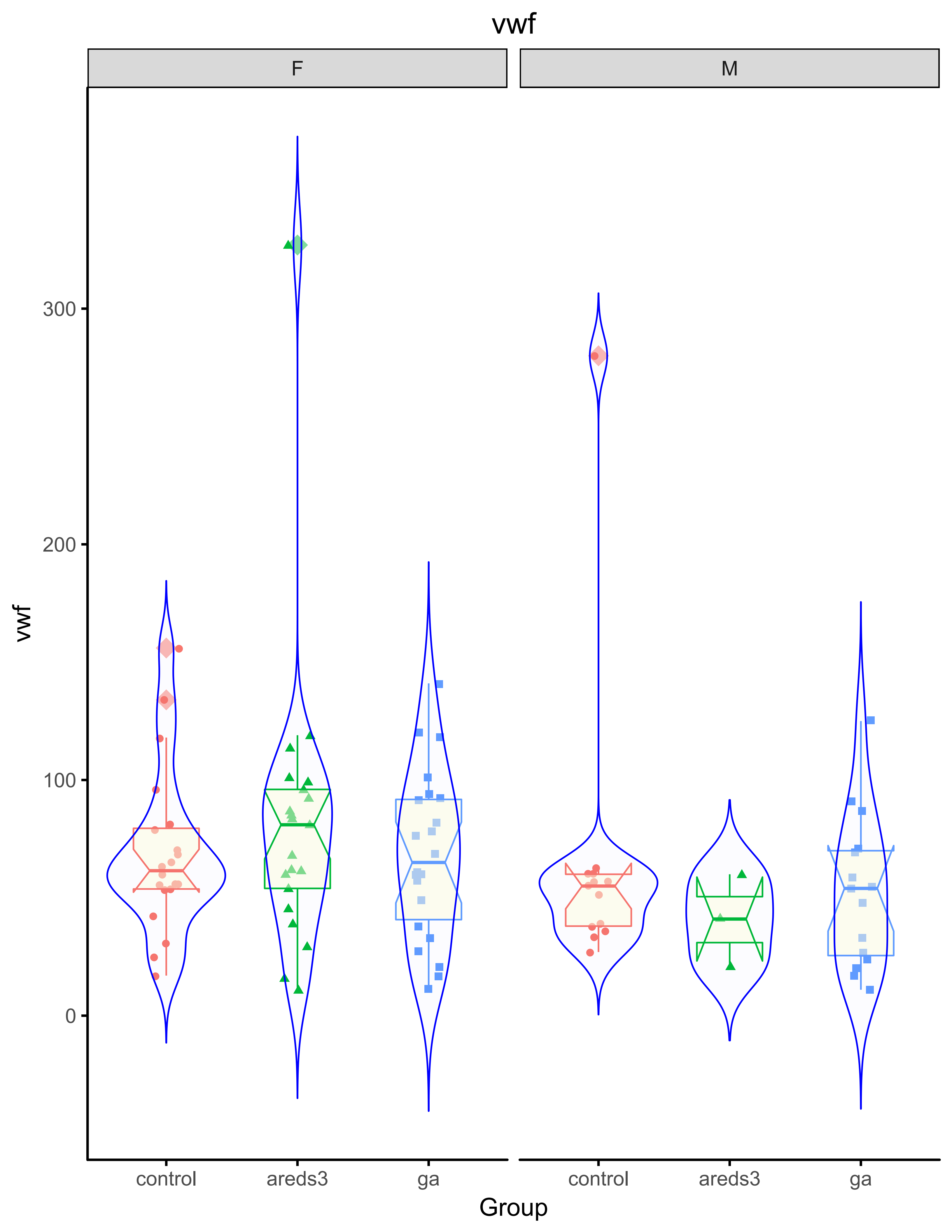

vwf
F
M
300
200
100
0
vwf
control
areds3
ga
control
areds3
ga
Group

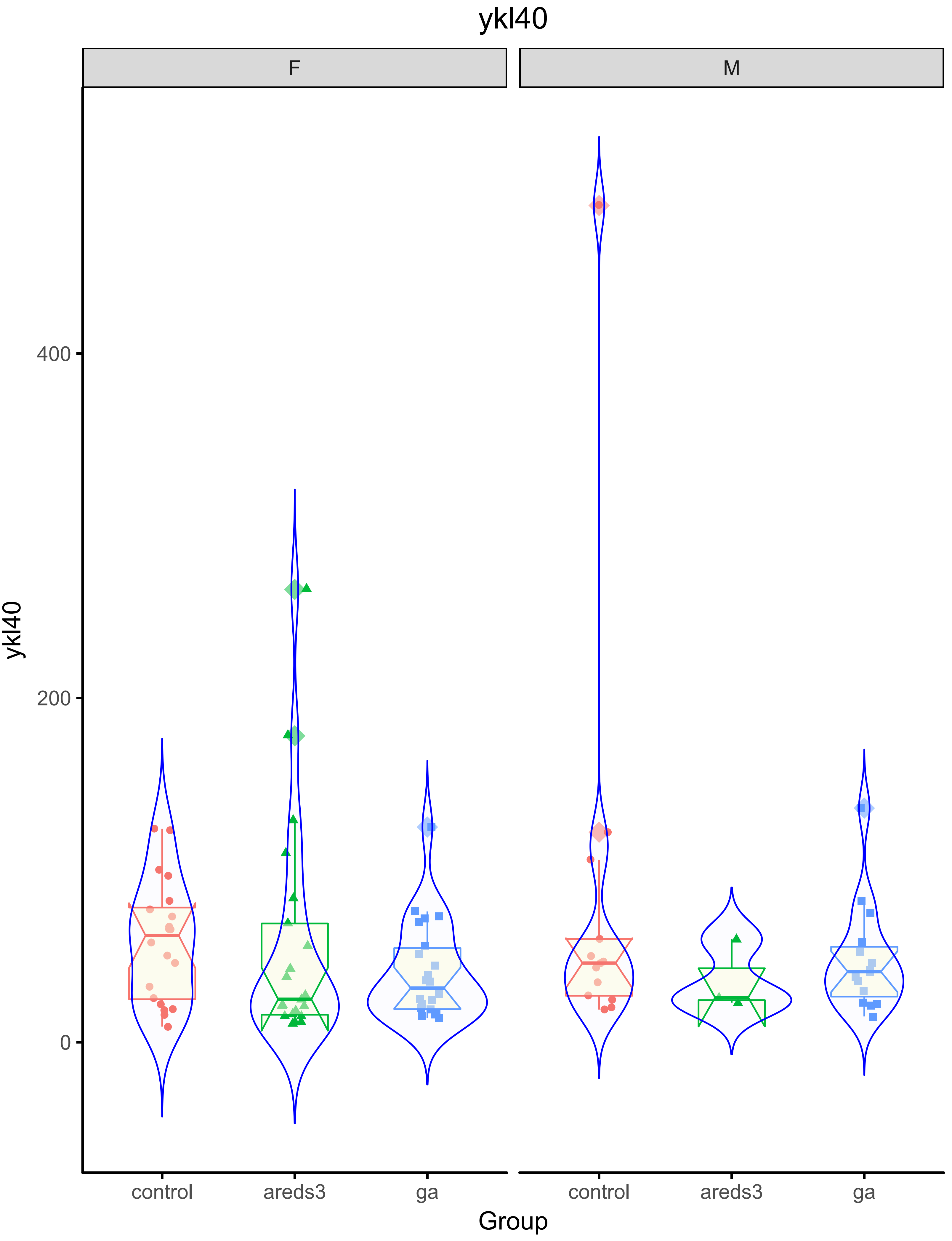

ykl40
F
M
400
200
0
ykl40
control
areds3
ga
control
areds3
ga
Group

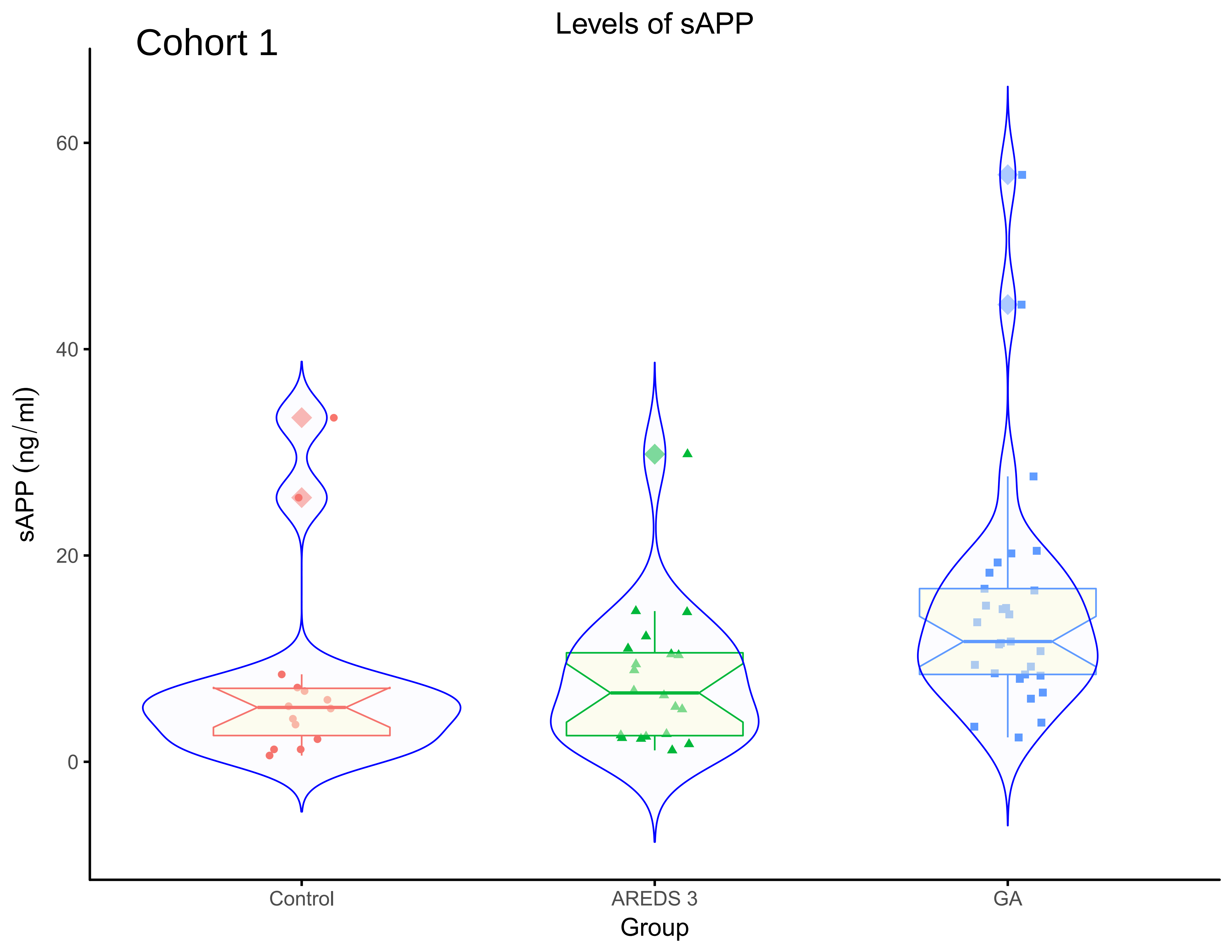

Cohort 1
Levels of sAPP
sAPP (ng/ml)
60
40
20
0
Control
AREDS 3
GA
Group

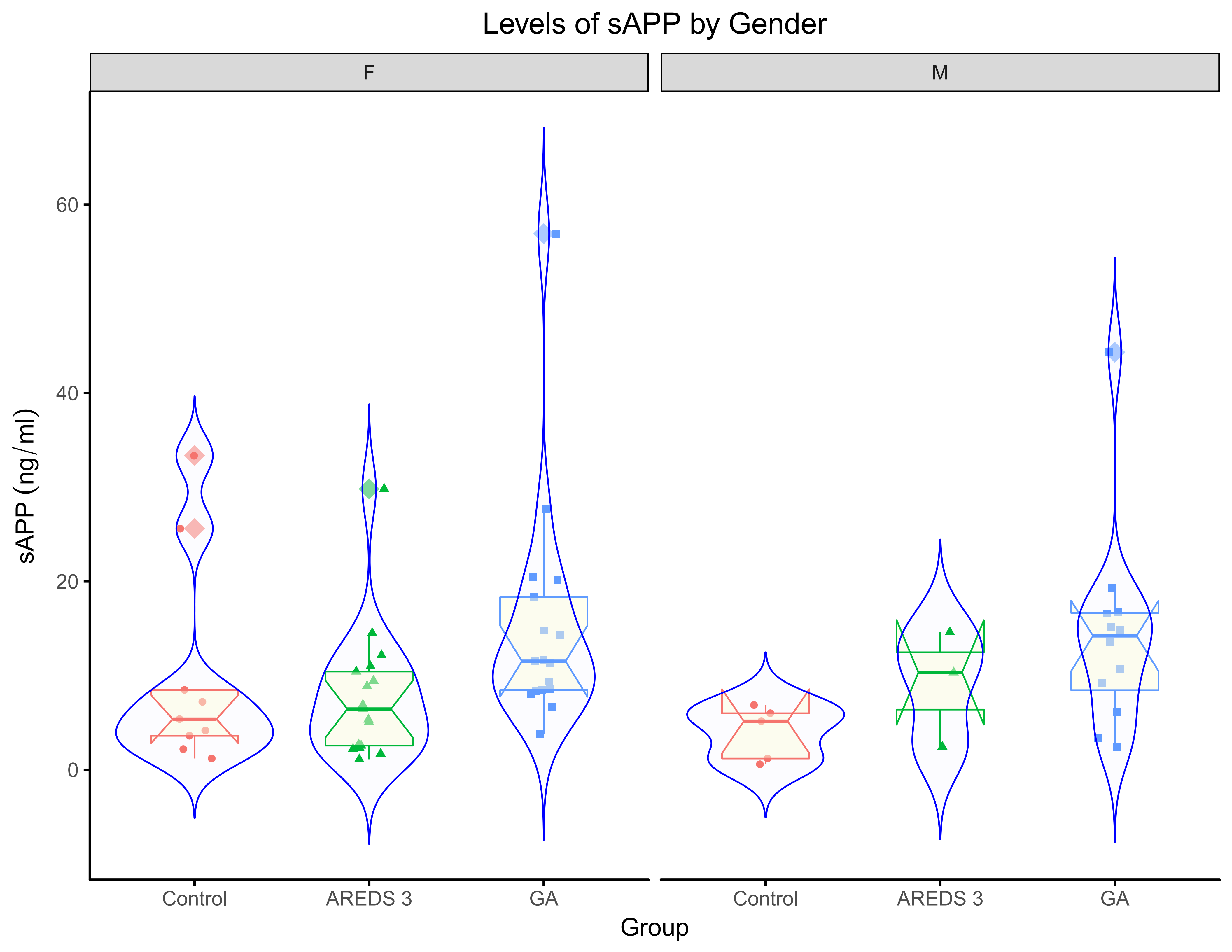

Levels of sAPP by Gender
F
M
sAPP (ng/ml)
60
40
20
0
Control
AREDS 3
GA
Control
AREDS 3
GA
Group

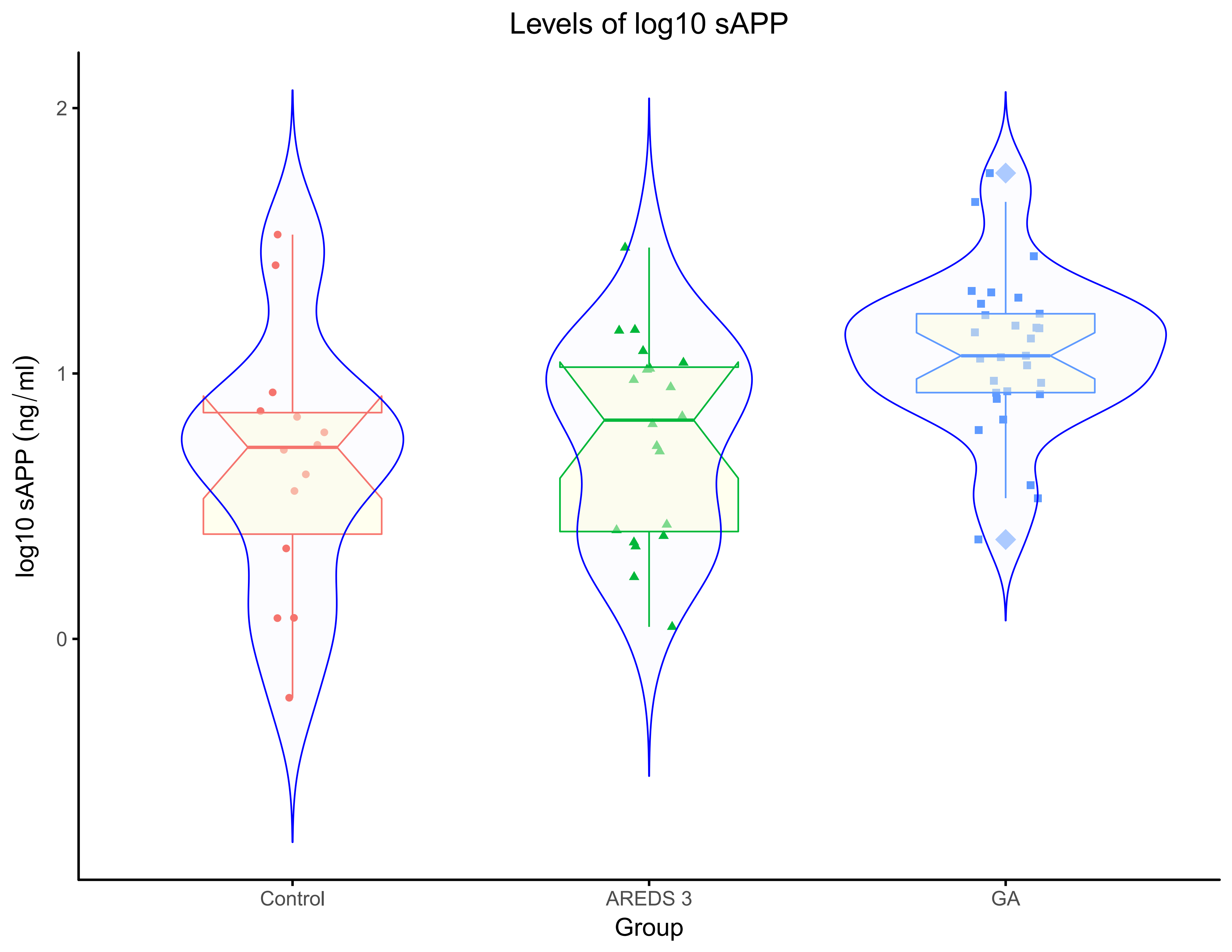
Levels of log10 sAPP
log10 sAPP (ng/ml)
2
1
0
Control
AREDS 3
GA
Group

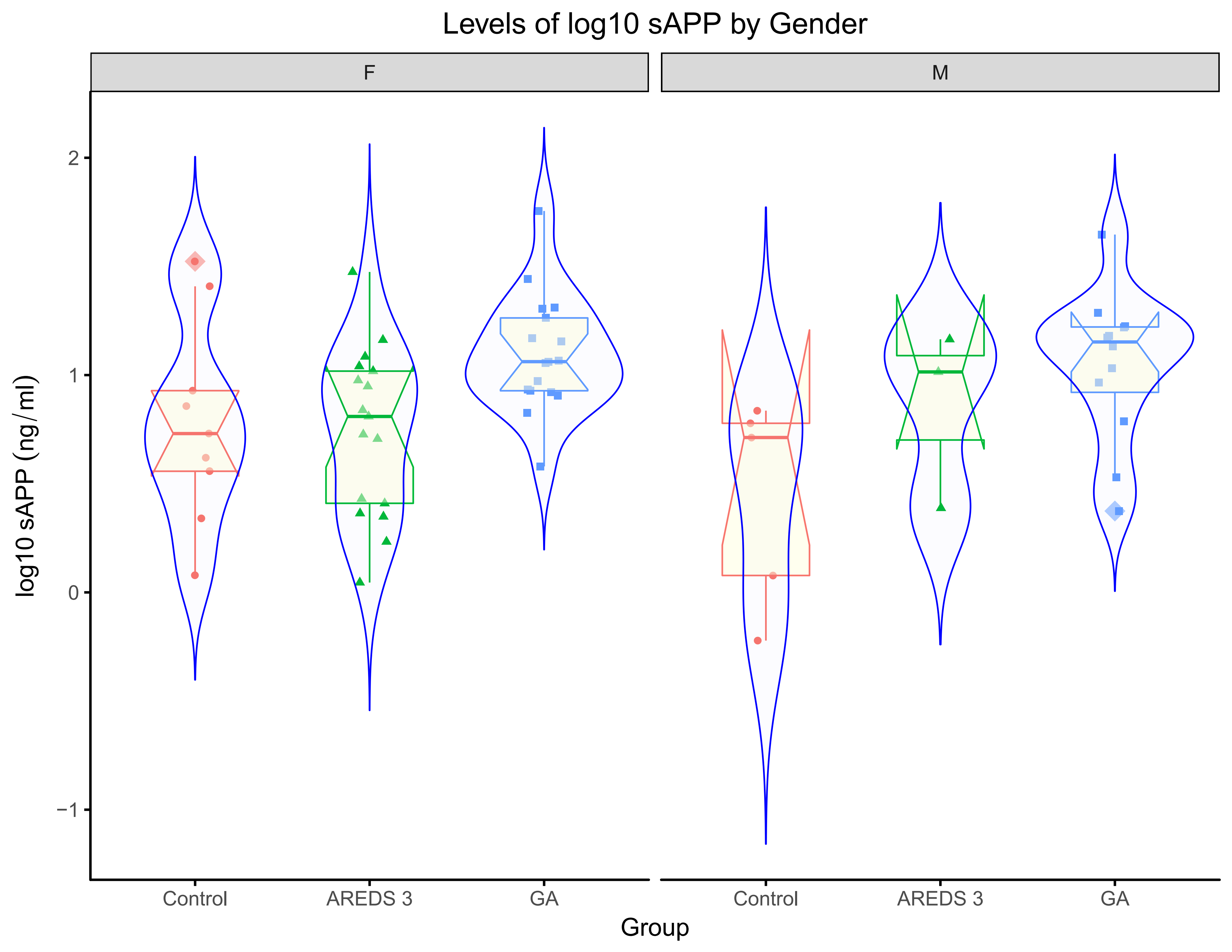

Levels of log10 sAPP by Gender
F
M
2
1
0
−1
log10 sAPP (ng/ml)
Control
AREDS 3
GA
Control
AREDS 3
GA
Group

COHORT 2 BOX PLOTS

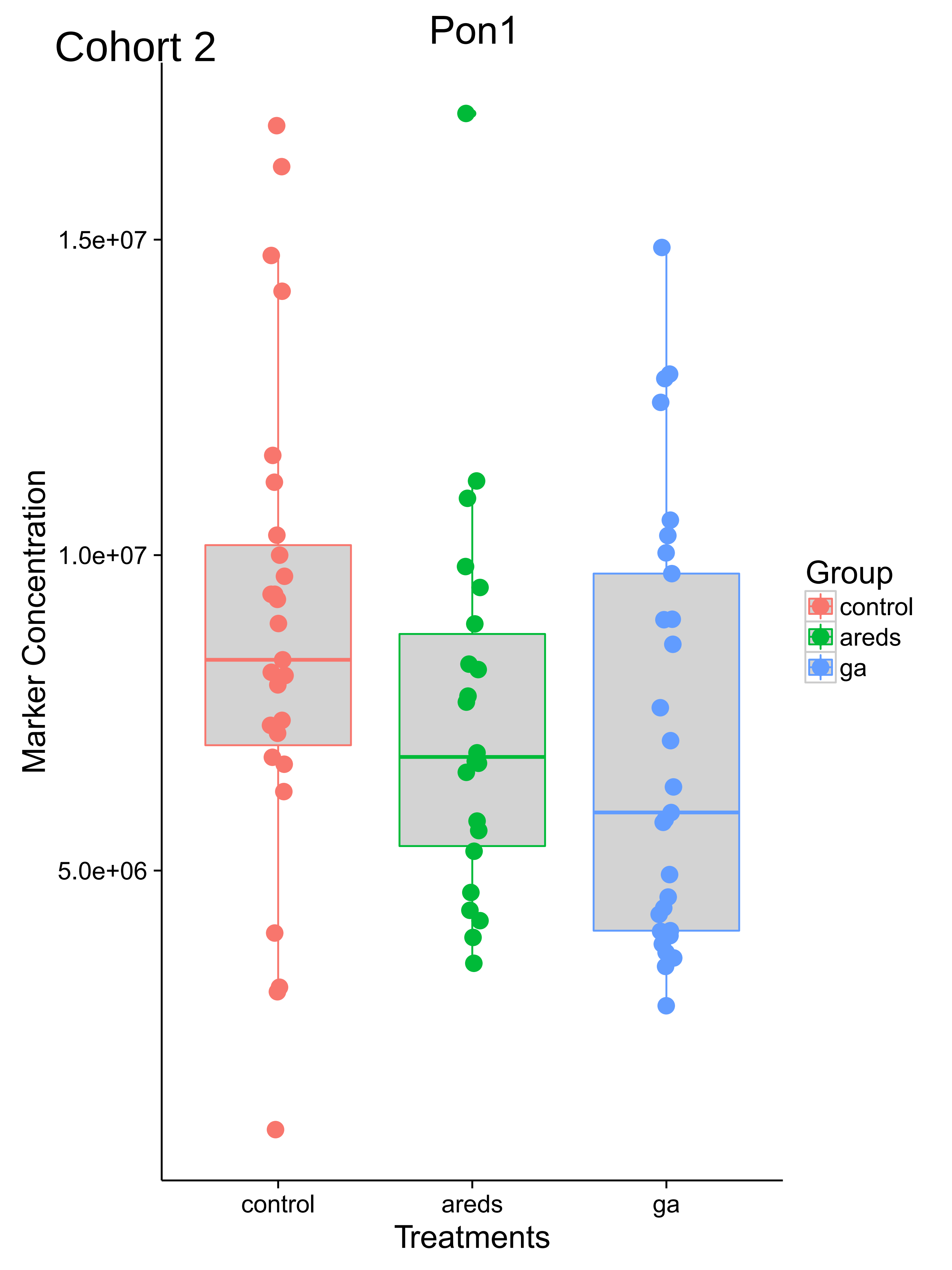

Cohort 2
Pon1
Marker Concentration
1.5e+07
1.0e+07
5.0e+06
control
areds
ga
Treatments
Group
control
areds
ga

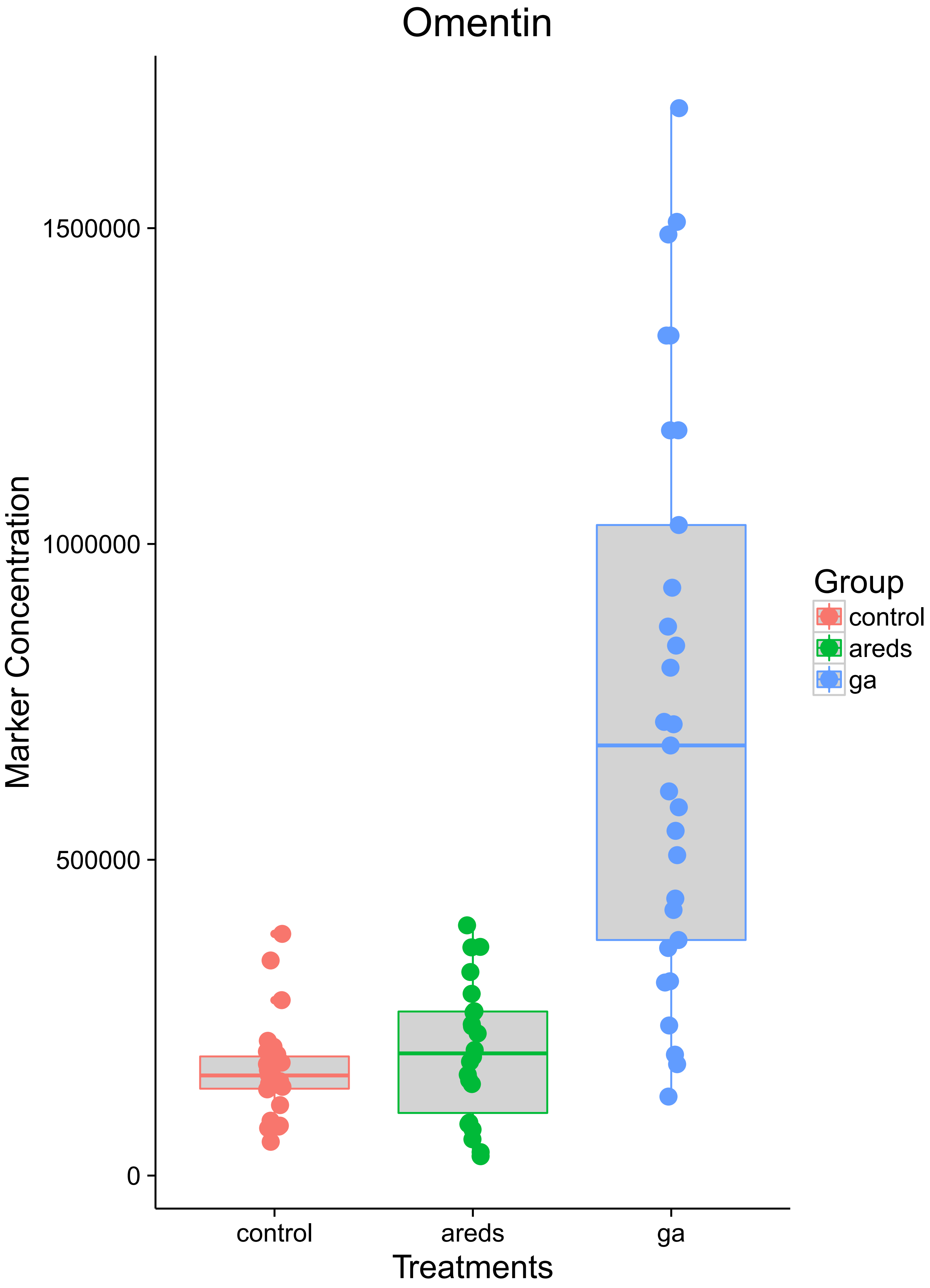
Omentin
Marker Concentration
1500000
1000000
500000
0
control
areds
ga
Treatments
Group
control
areds
ga

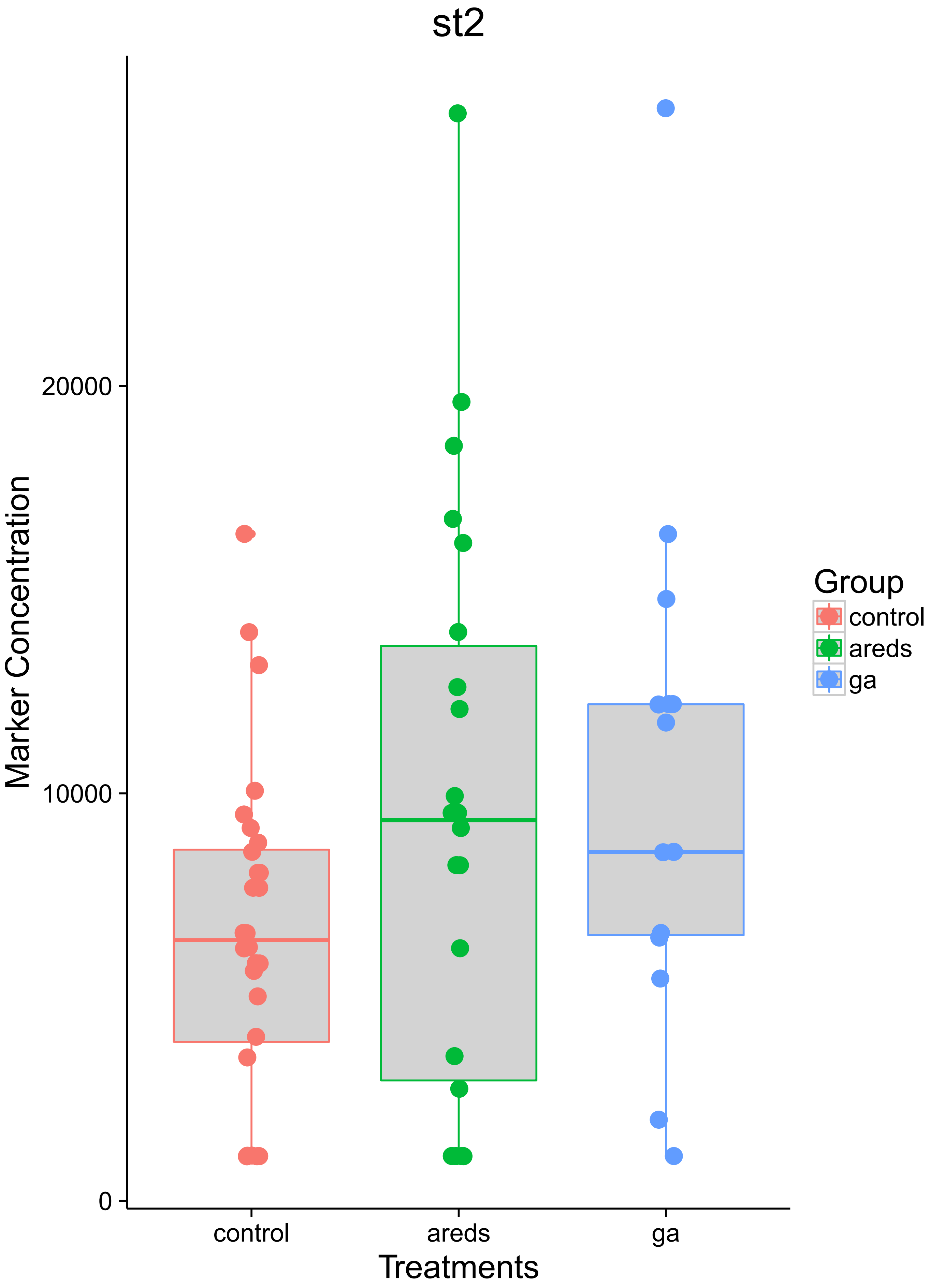

st2
Marker Concentration
20000
10000
0
control
areds
ga
Treatments
Group
control
areds
ga

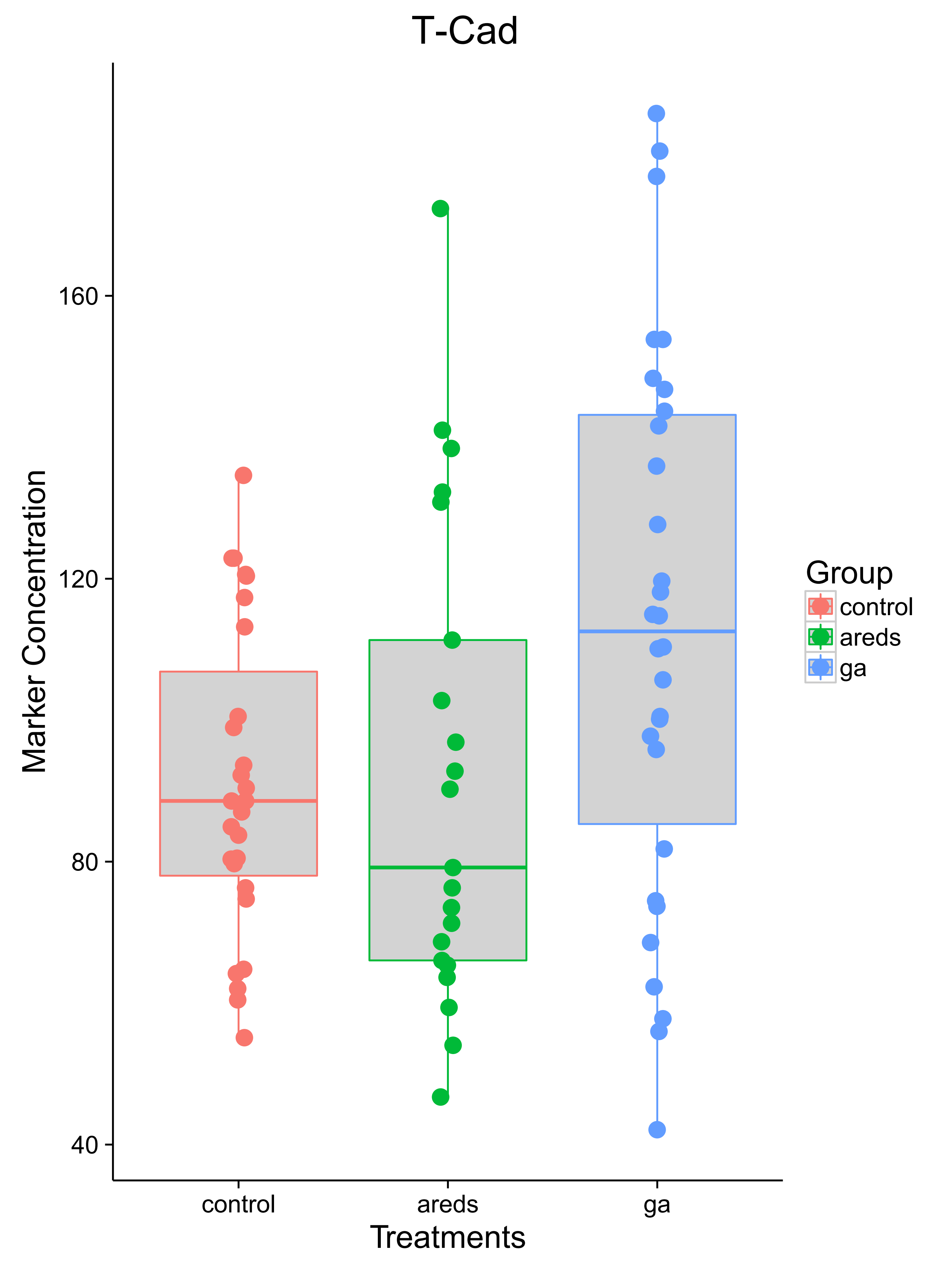
T-Cad
Marker Concentration
160
120
80
40
control
areds
ga
Treatments
Group
control
areds
ga

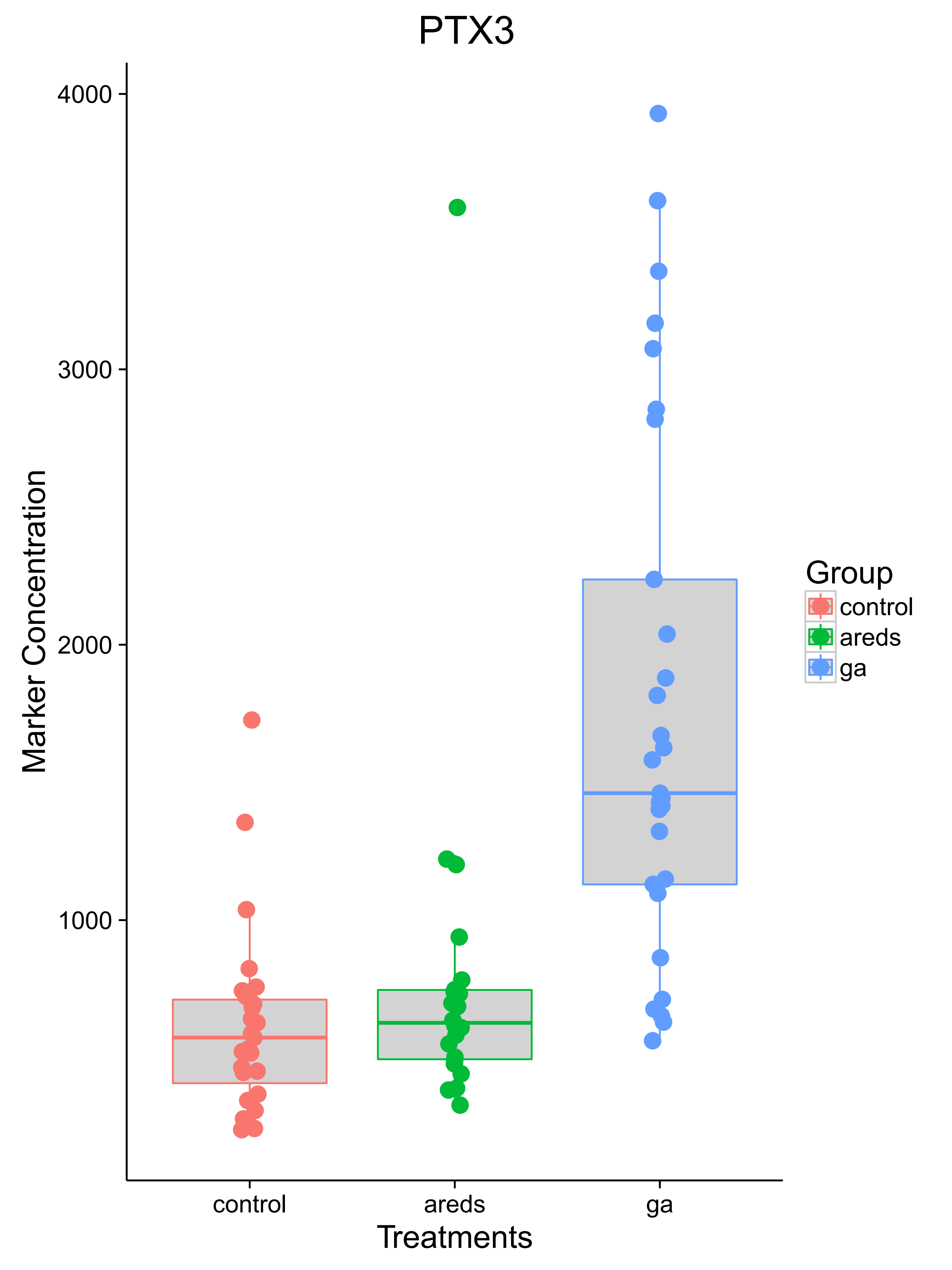
PTX3
Marker Concentration
4000
3000
2000
1000
control
areds
ga
Treatments
Group
control
areds
ga

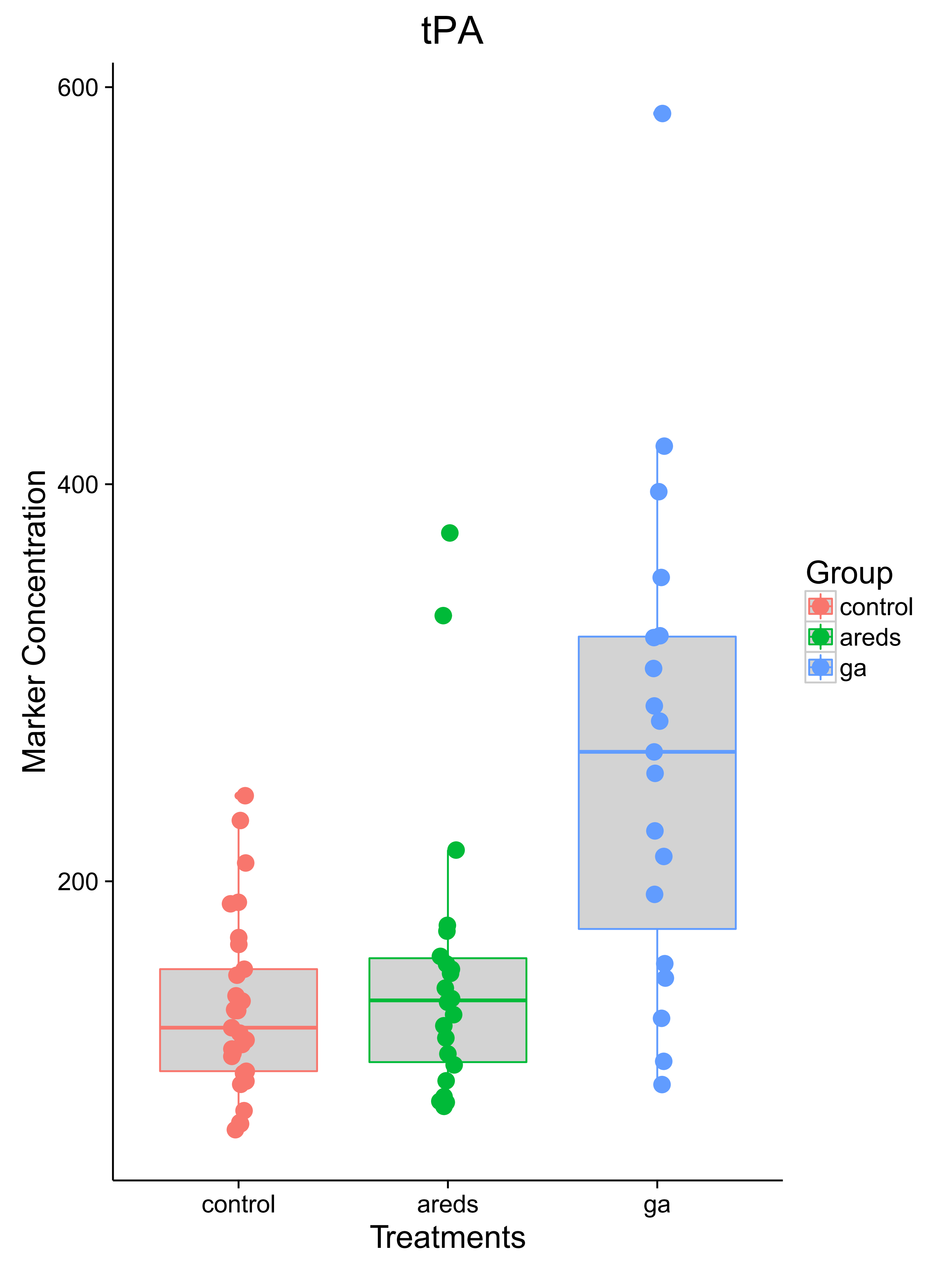
tPA
600
400
200
Marker Concentration
control
areds
ga
Treatments
Group
control
areds
ga

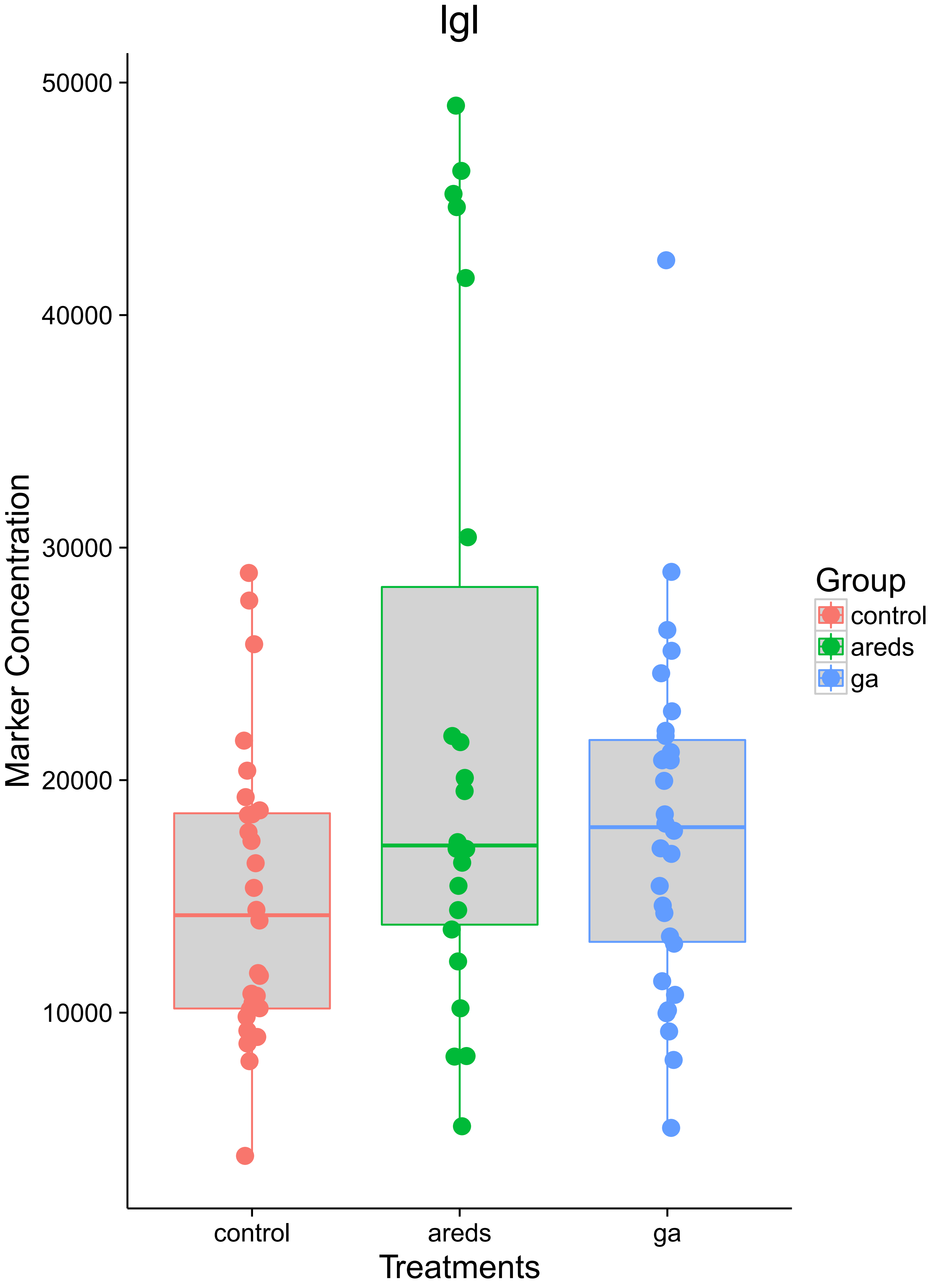

Igl
Marker Concentration
50000
40000
30000
20000
10000
control
areds
ga
Treatments
Group
control
areds
ga

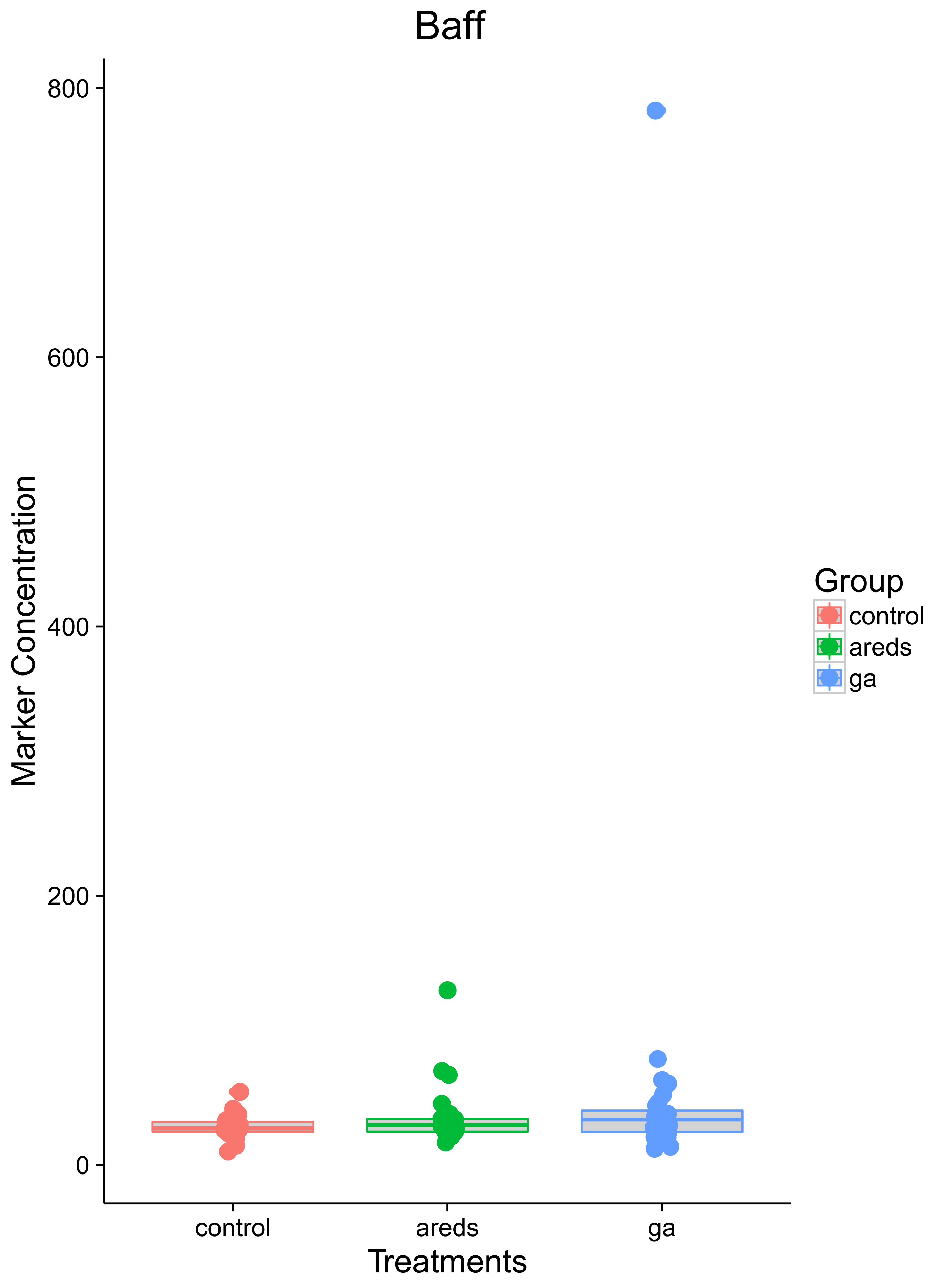
Baff
Marker Concentration
800
600
400
200
0
control
areds
ga
Treatments
Group
control
areds
ga

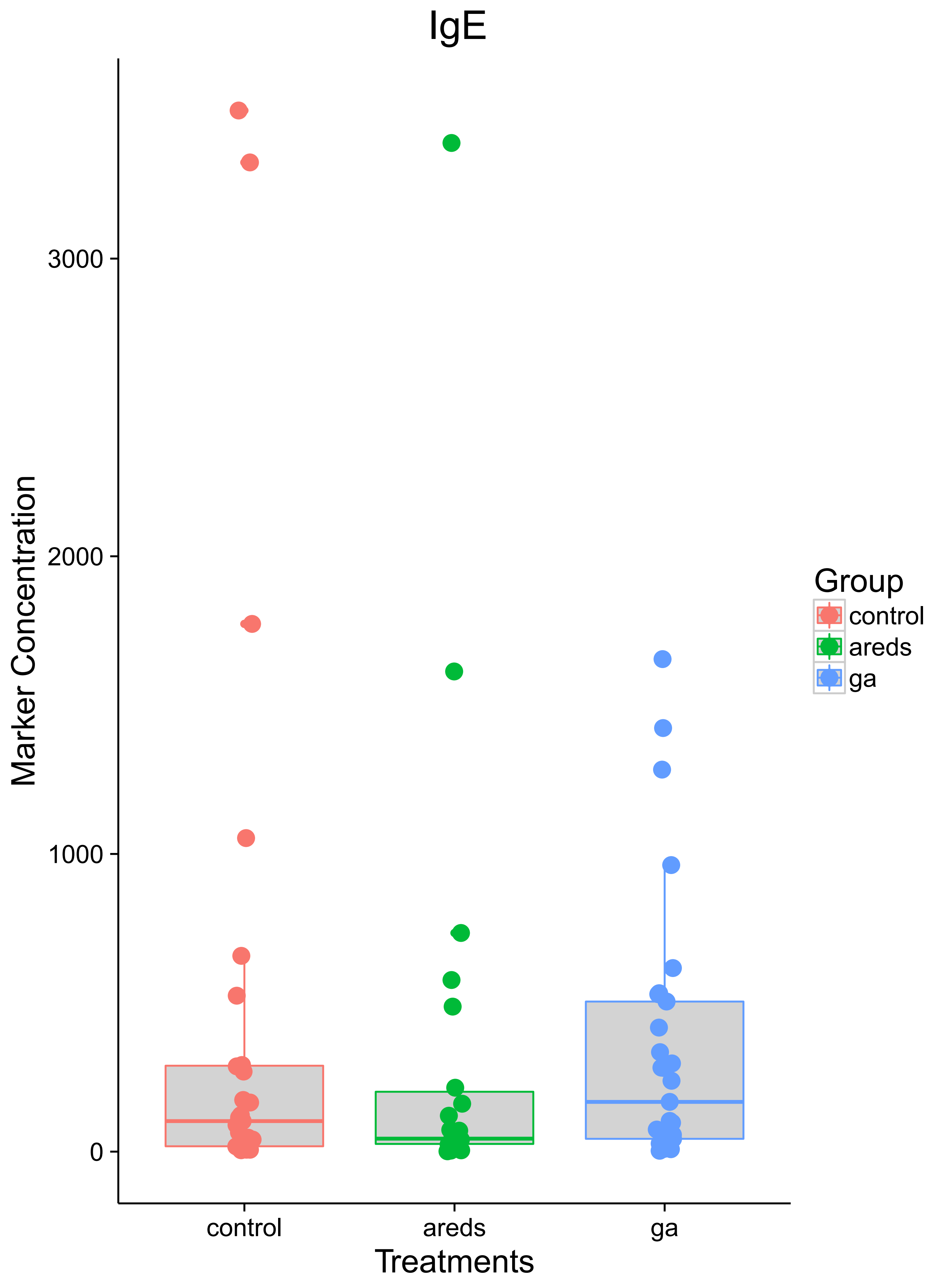

IgE
Marker Concentration
3000
2000
1000
0
control
areds
ga
Treatments
Group
control
areds
ga

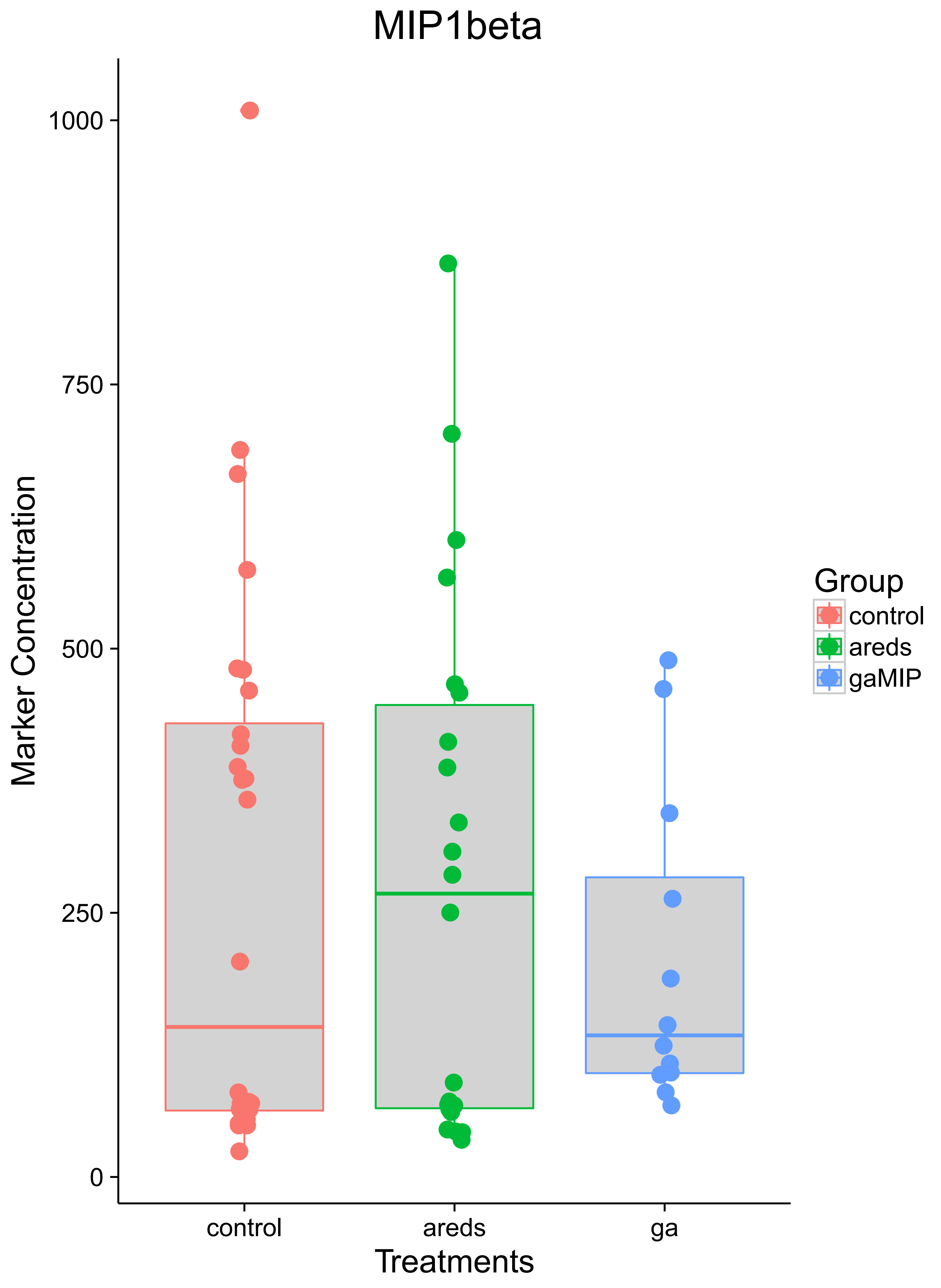

MIP1beta
Marker Concentration
1000
750
500
250
0
control
areds
ga
Treatments
Group
control
areds
gaMIP

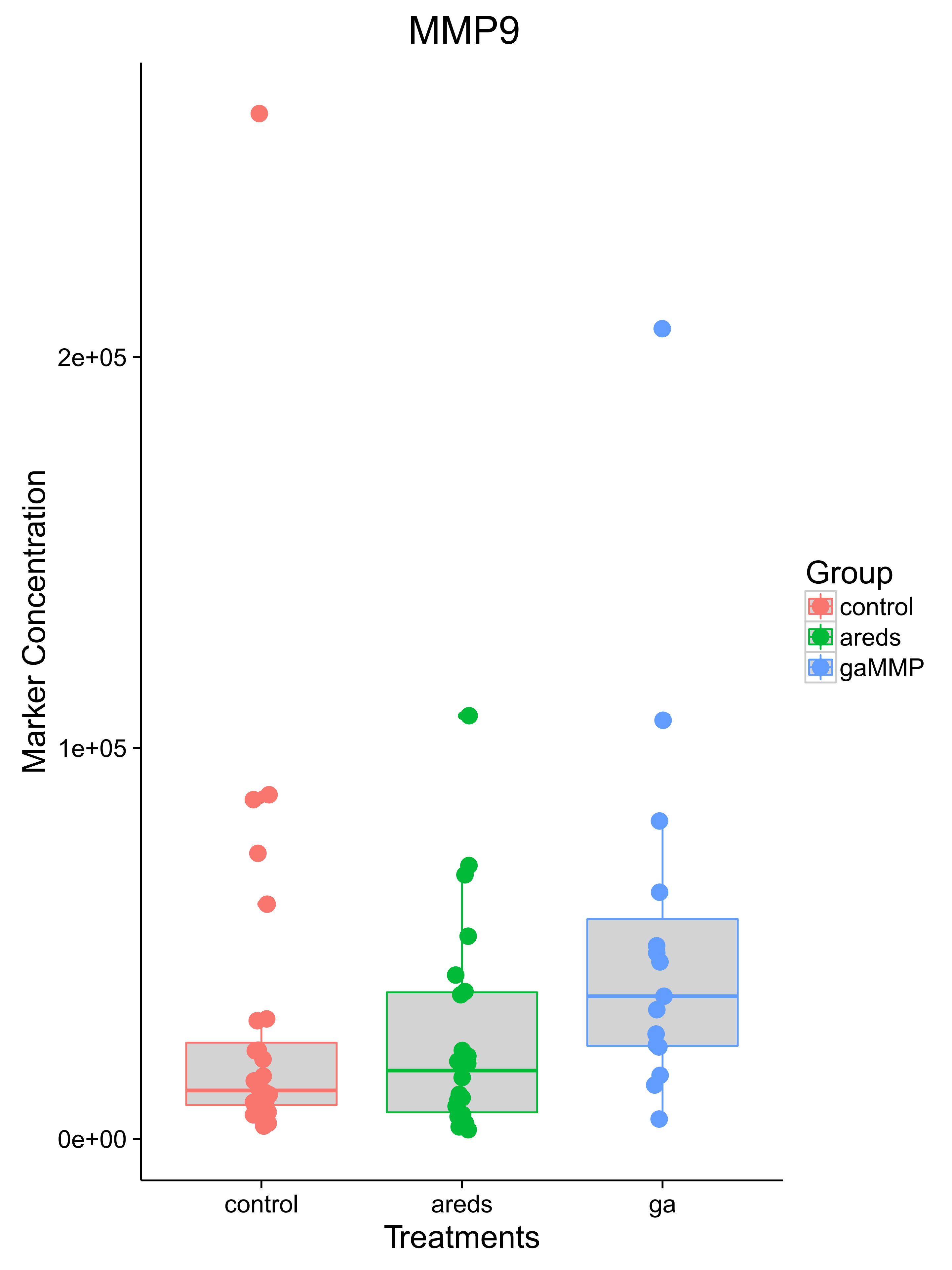

MMP9
Marker Concentration
2e+05
1e+05
0e+00
control
areds
ga
Treatments
Group
control
areds
gaMMP

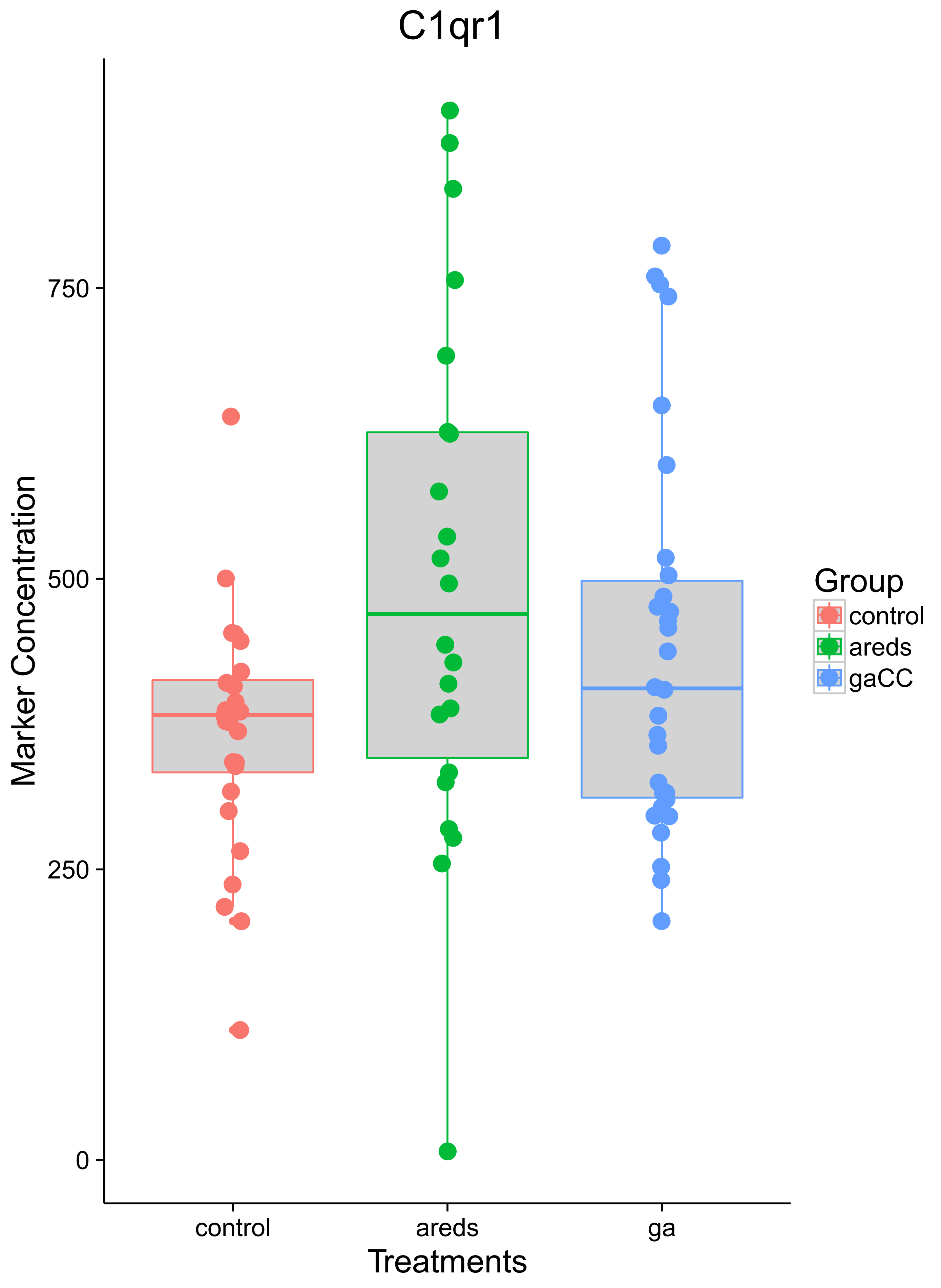

C1qr1
Marker Concentration
0
250
500
750
control
areds
ga
Treatments
Group
control
areds
gaCC

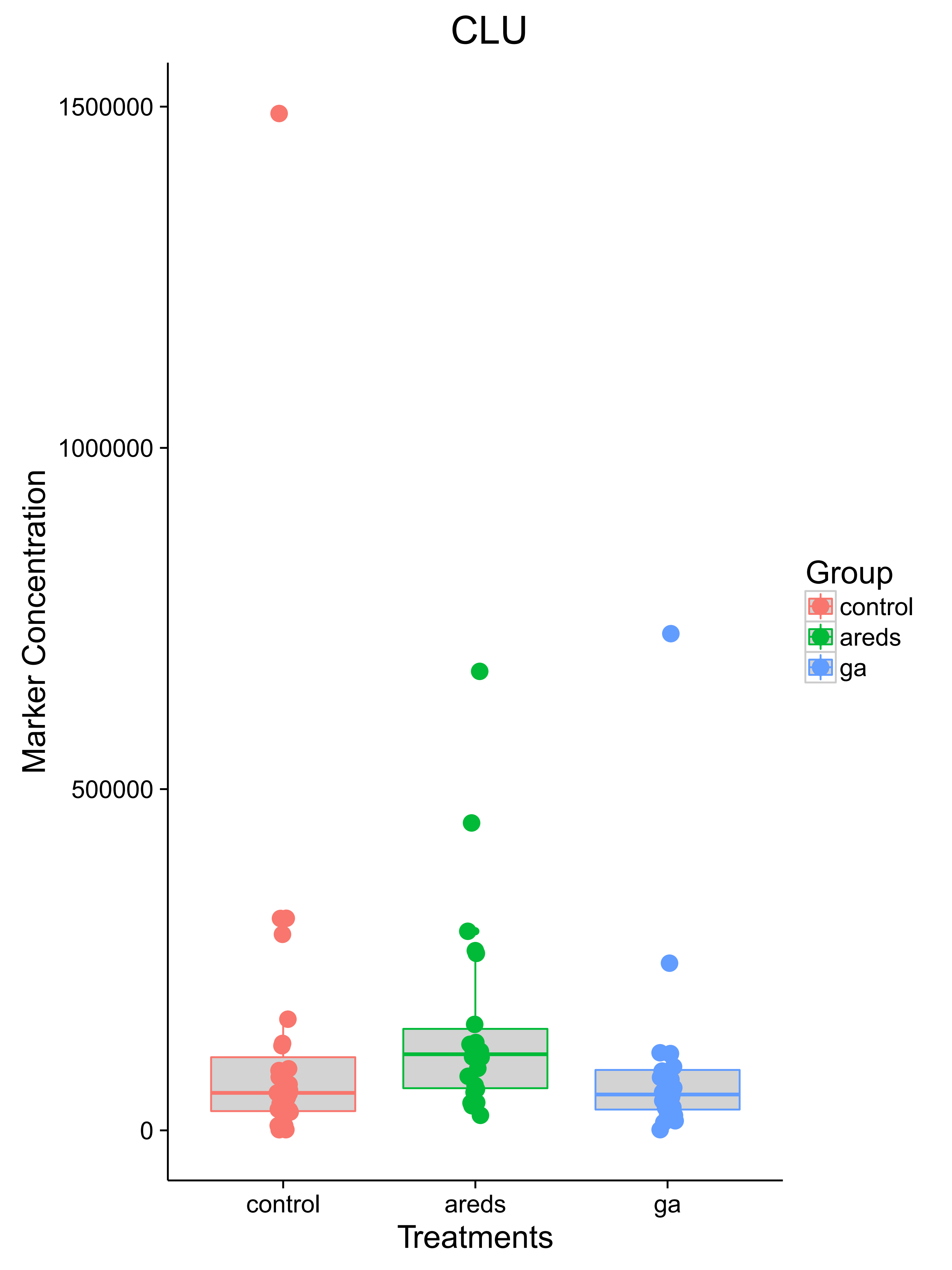

CLU
1500000
1000000
500000
0
Marker Concentration
control
areds
ga
Treatments
Group
control
areds
ga

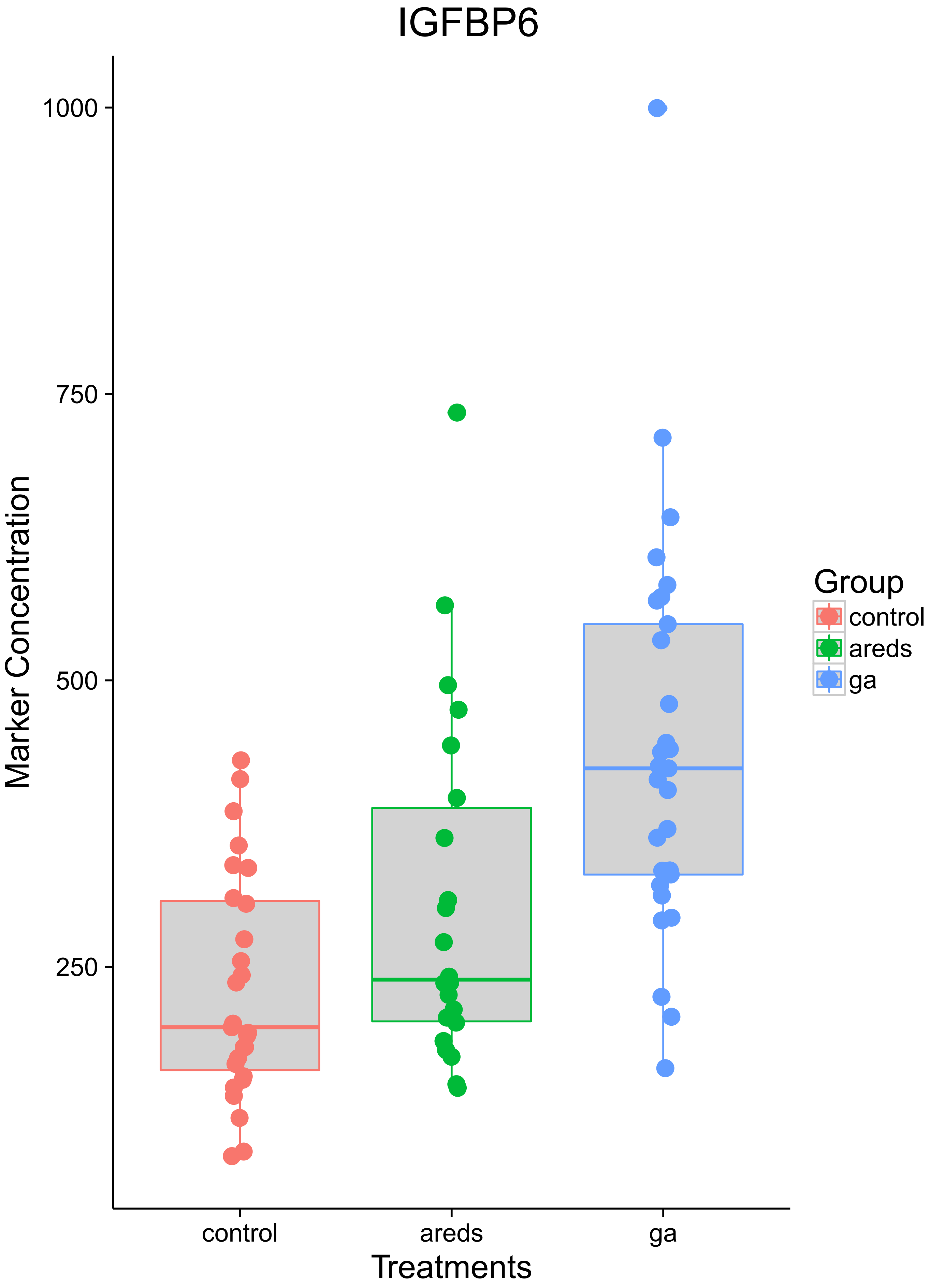
IGFBP6
1000
750
500
250
Marker Concentration
control
areds
ga
Treatments
Group
control
areds
ga

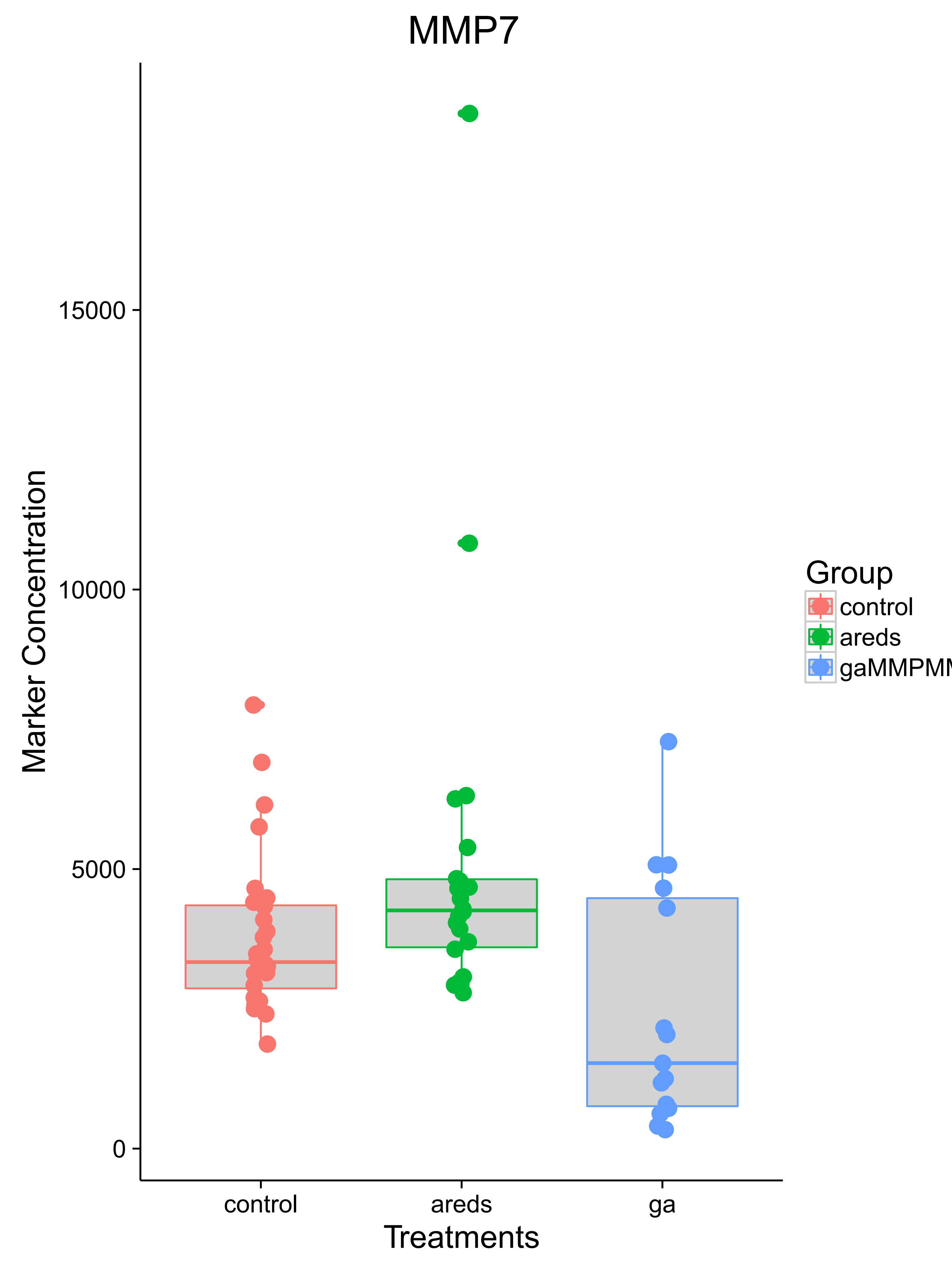
MMP7
Marker Concentration
15000
10000
5000
0
control
areds
ga
Treatments
Group
control
areds
gaMMPMM

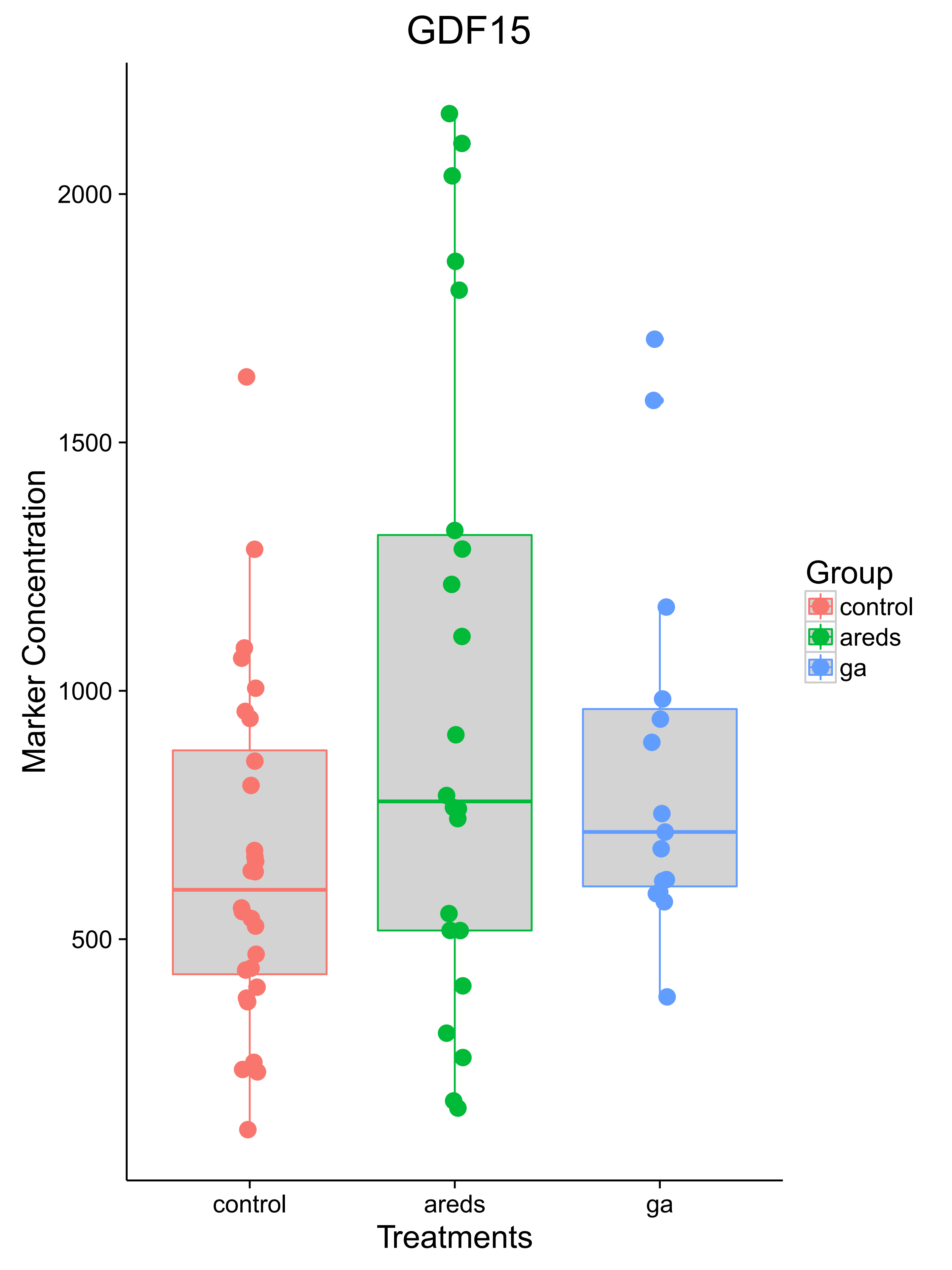
GDF15
Marker Concentration
2000
1500
1000
500
control
areds
ga
Treatments
Group
control
areds
ga

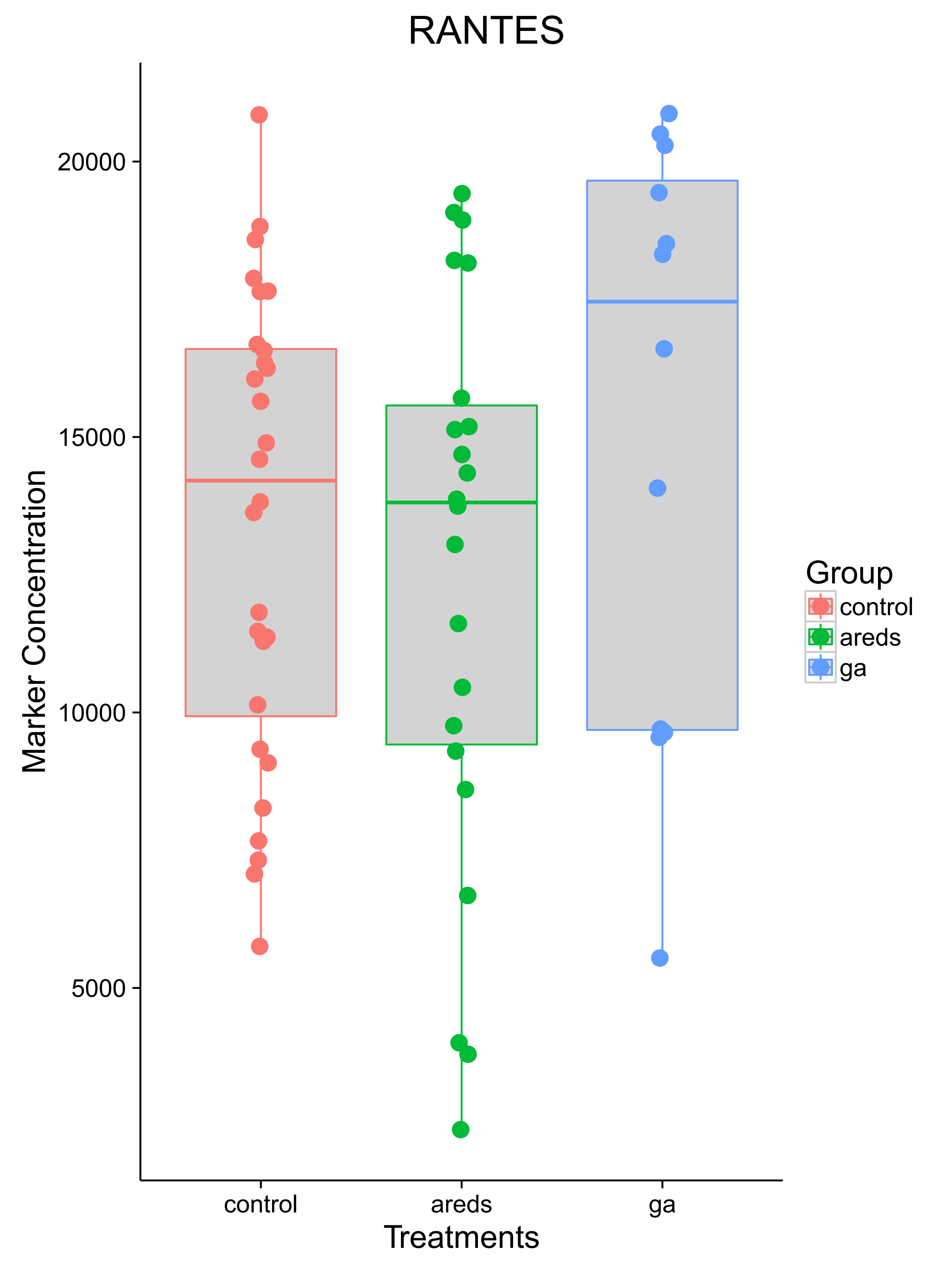
RANTES
Marker Concentration
20000
15000
10000
5000
control
areds
ga
Treatments
Group
control
areds
ga

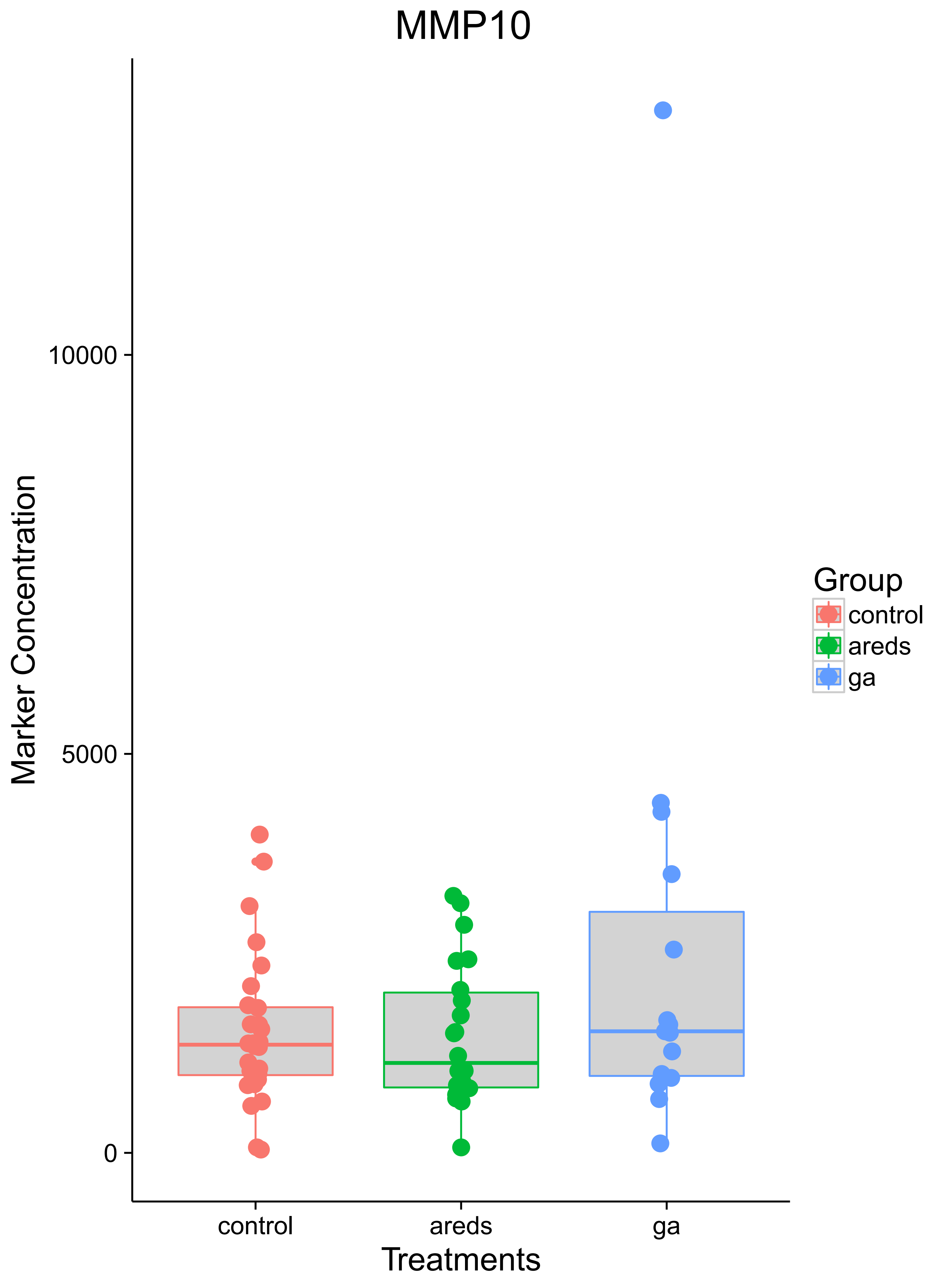
MMP10
Marker Concentration
10000
5000
0
control
areds
ga
Treatments
Group
control
areds
ga

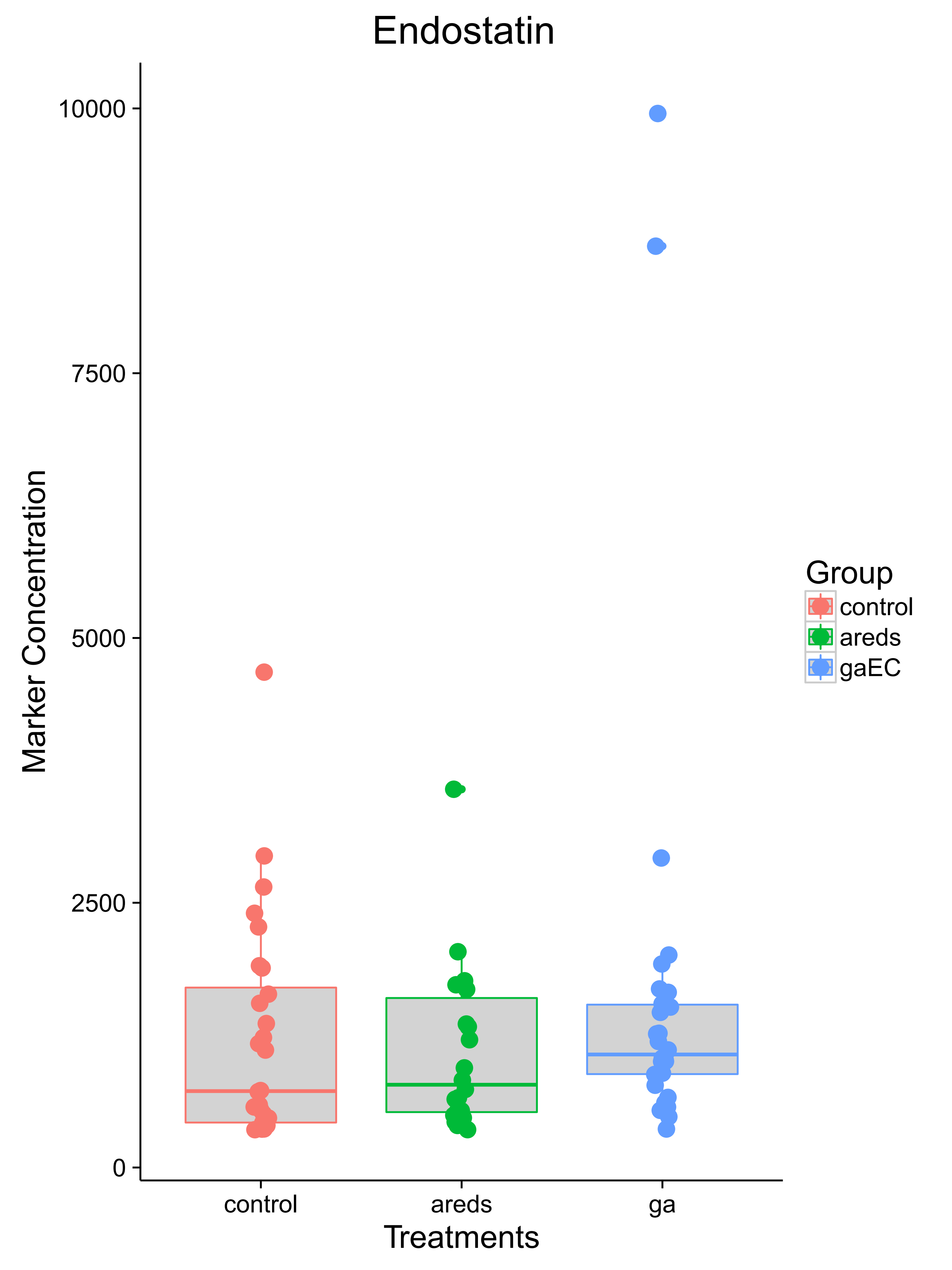

Endostatin
Marker Concentration
10000
7500
5000
2500
0
control
areds
ga
Treatments
Group
control
areds
gaEC

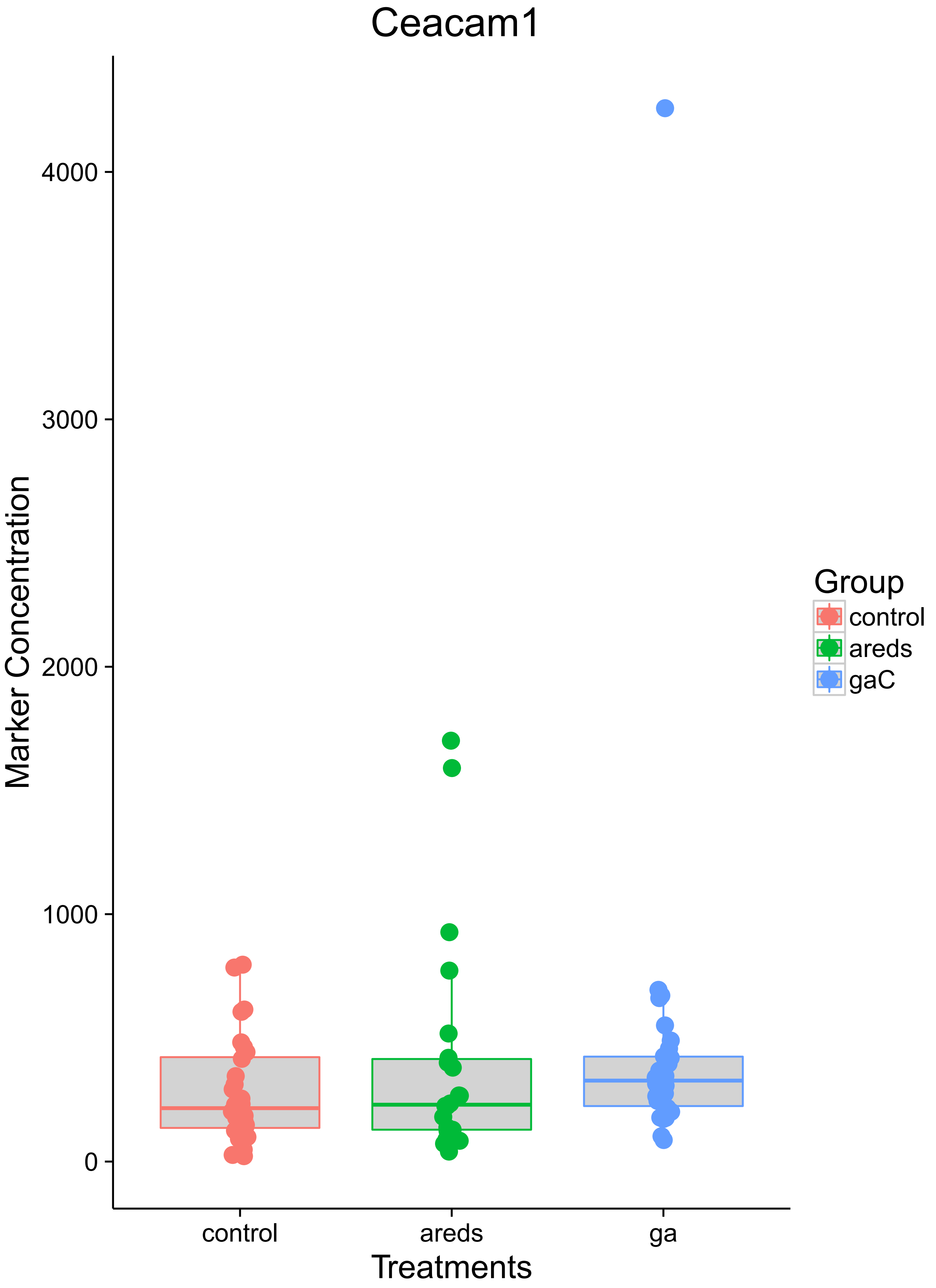

Ceacam1
Marker Concentration
4000
3000
2000
1000
0
control
areds
ga
Treatments
Group
control
areds
gaC

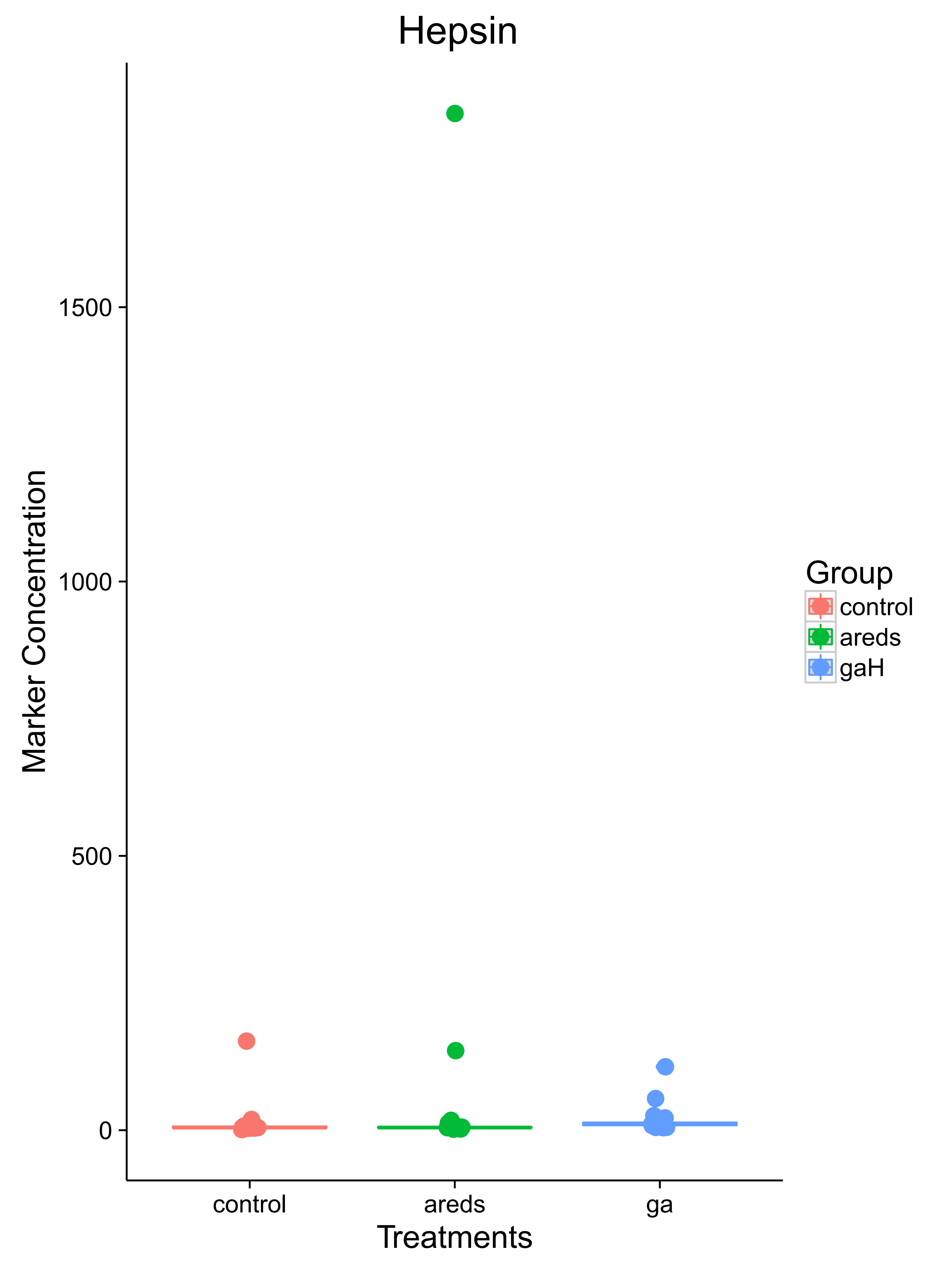
Hepsin
Marker Concentration
1500
1000
500
0
control
areds
ga
Treatments
Group
control
areds
gaH

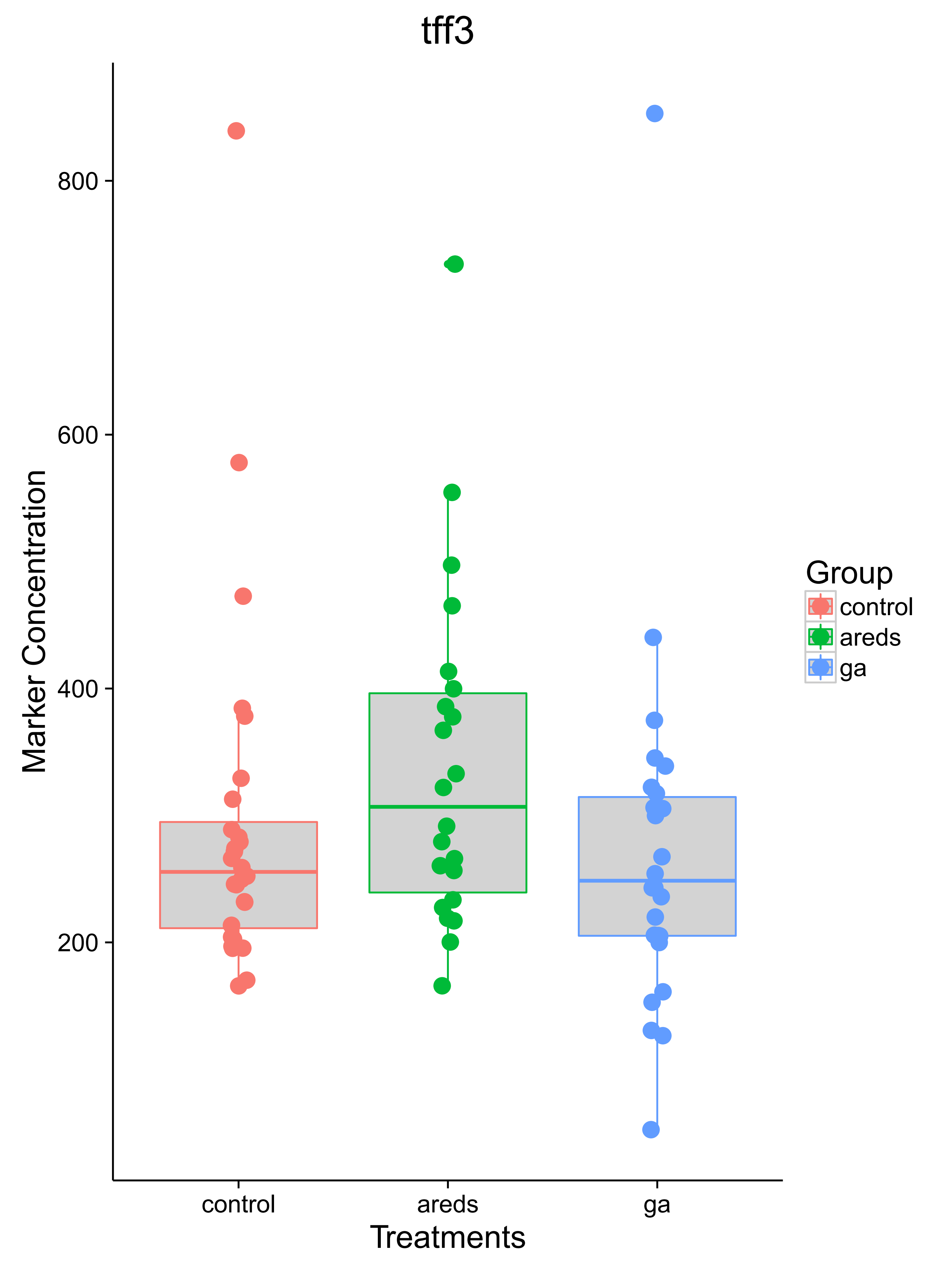

tff3
800
600
400
200
Marker Concentration
control
areds
ga
Treatments
Group
control
areds
ga

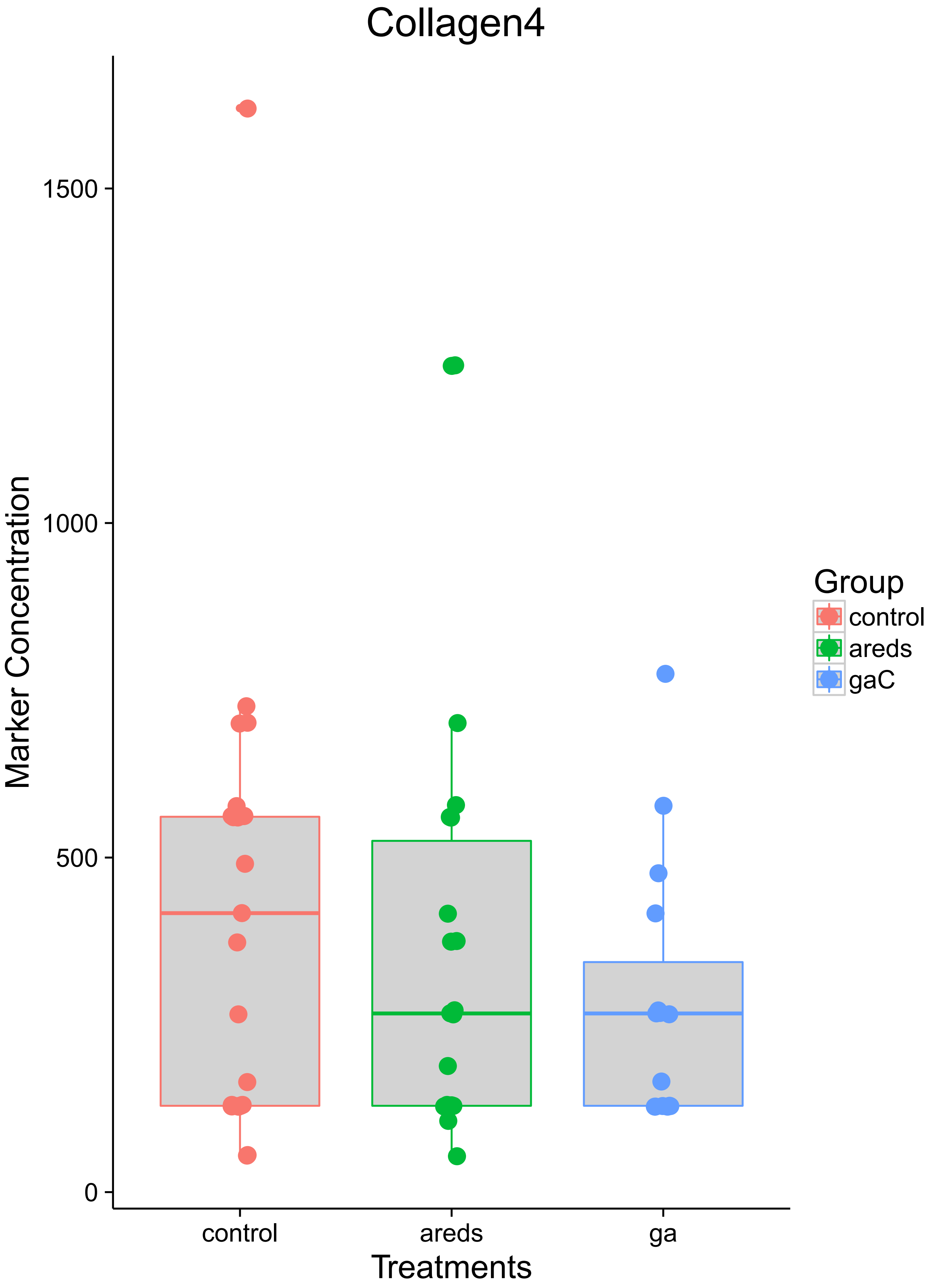
Collagen4
Marker Concentration
1500
1000
500
0
control
areds
ga
Treatments
Group
control
areds
gaC

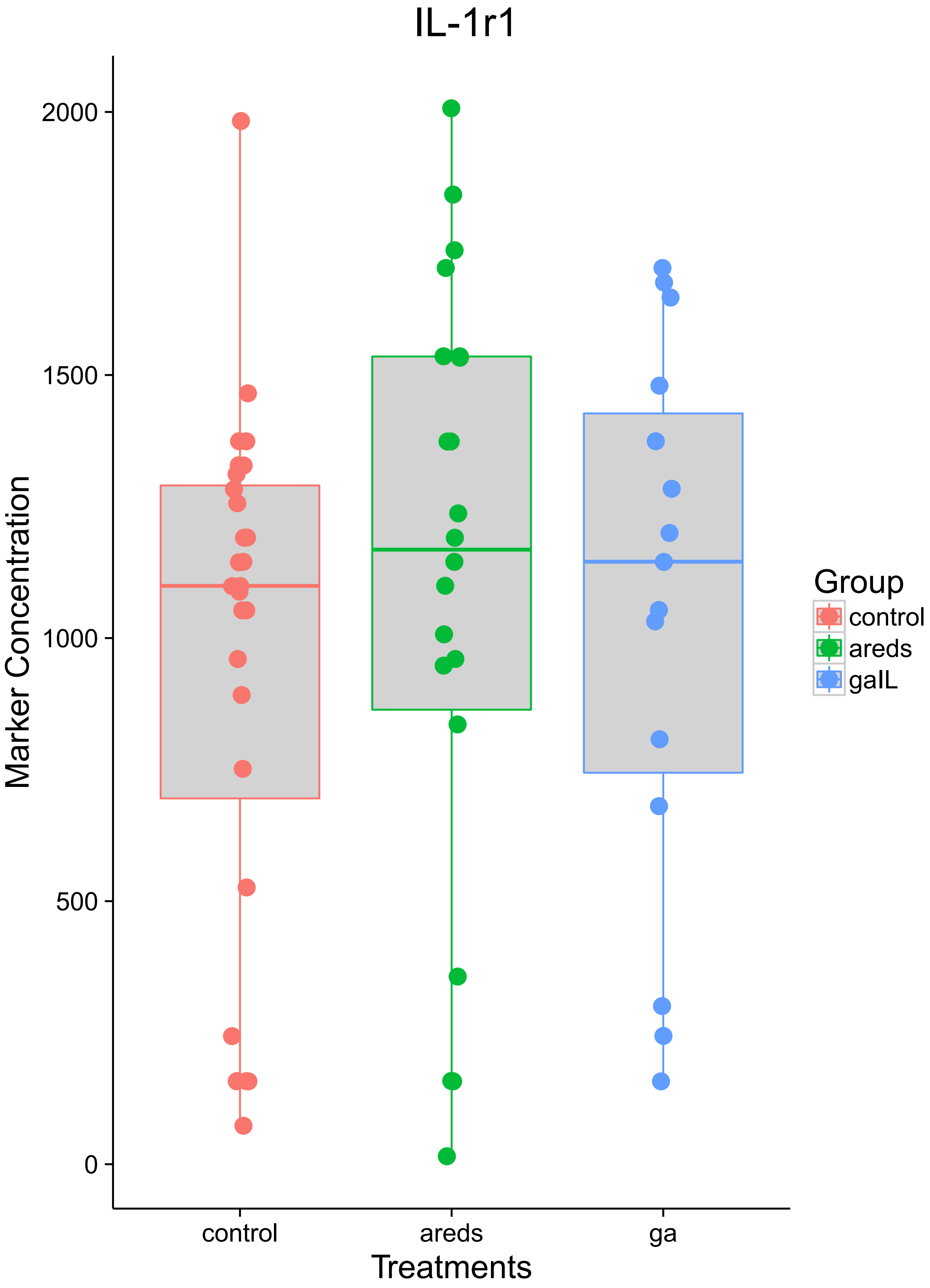
IL-1r1
Marker Concentration
2000
1500
1000
500
0
control
areds
ga
Treatments
Group
control
areds
galL

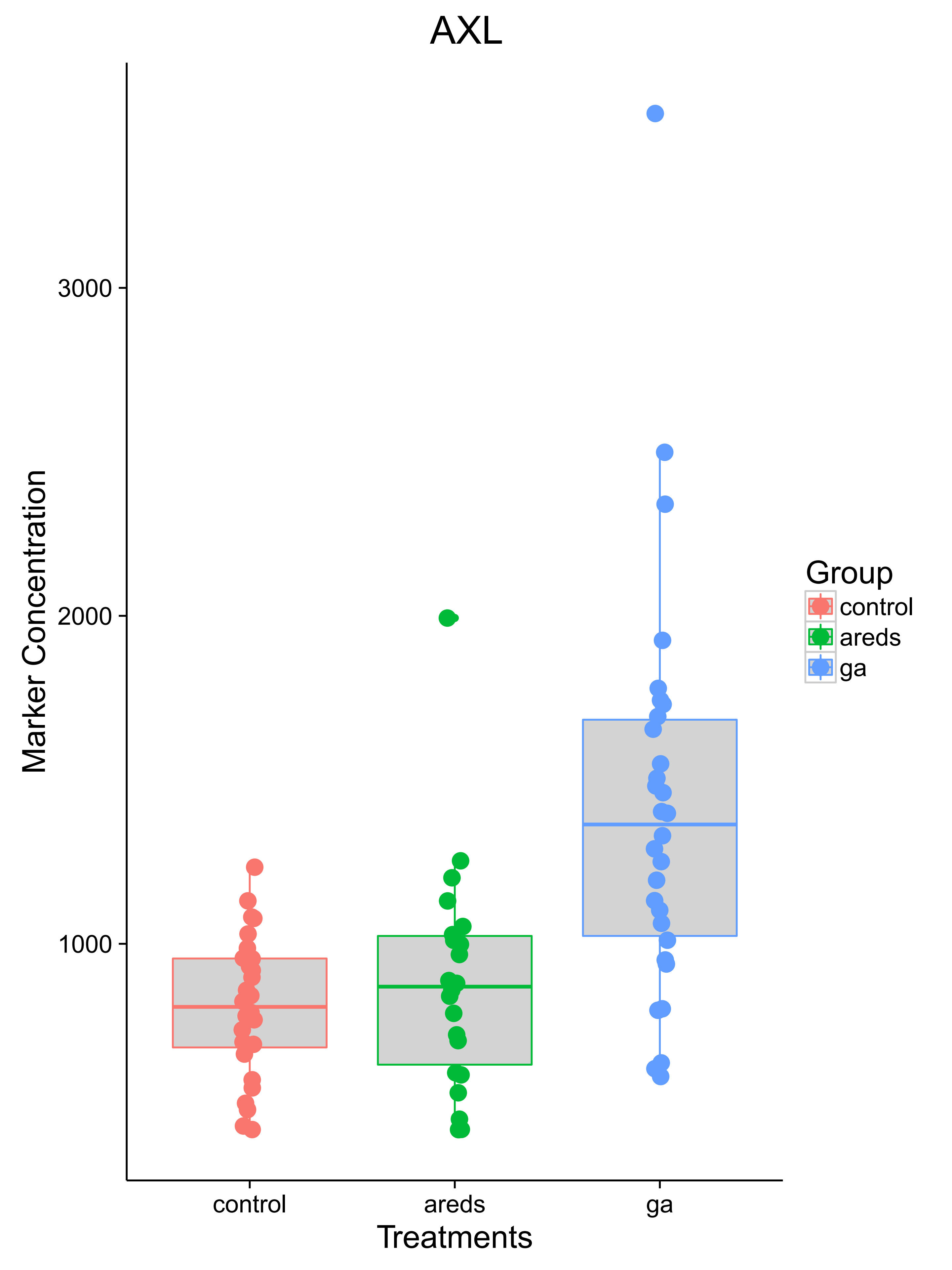
AXL
3000
2000
1000
Marker Concentration
control
areds
ga
Treatments
Group
control
areds
ga

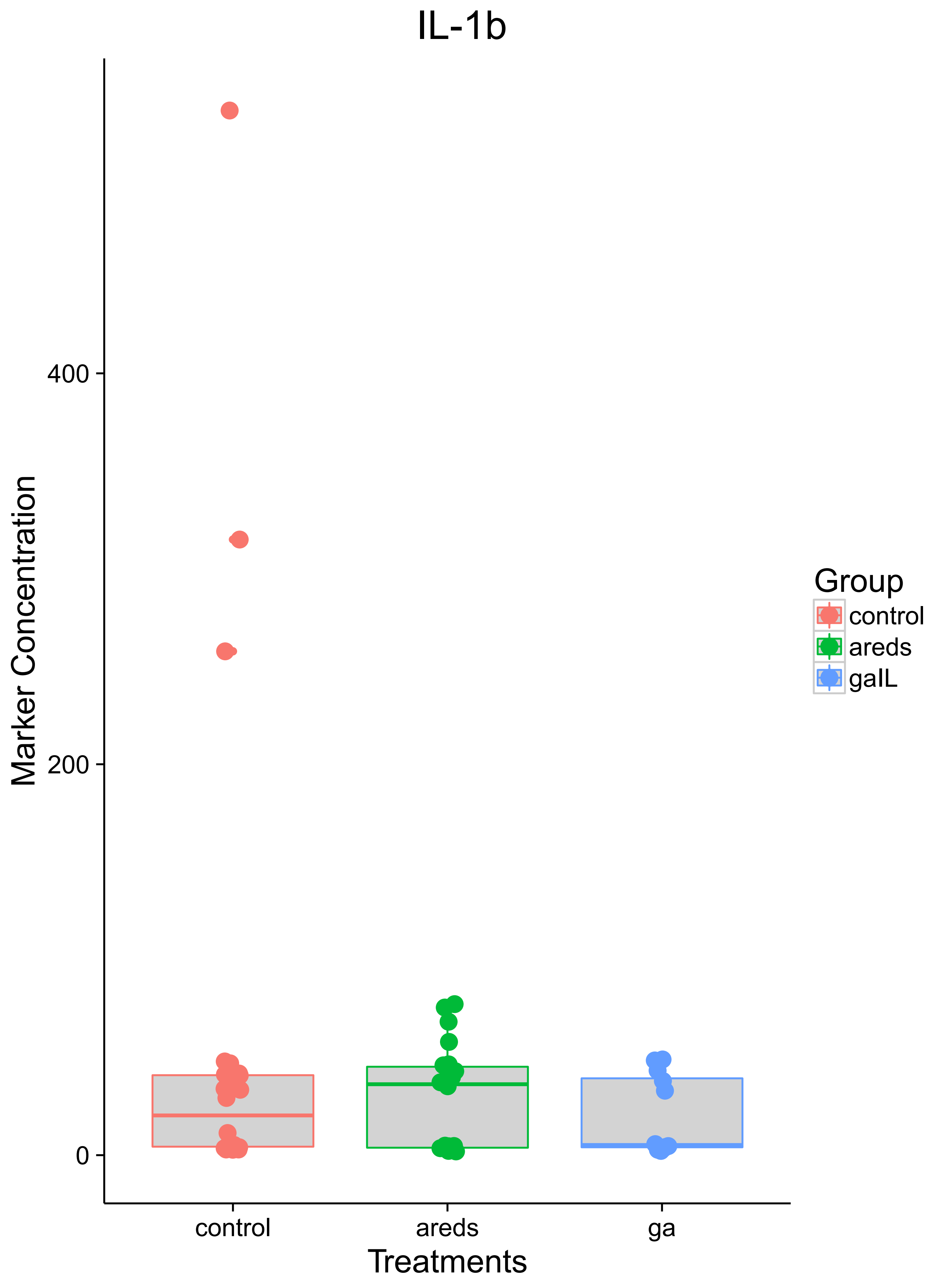
IL-1b
Marker Concentration
400
200
0
control
areds
ga
Treatments
Group
control
areds
gaIL

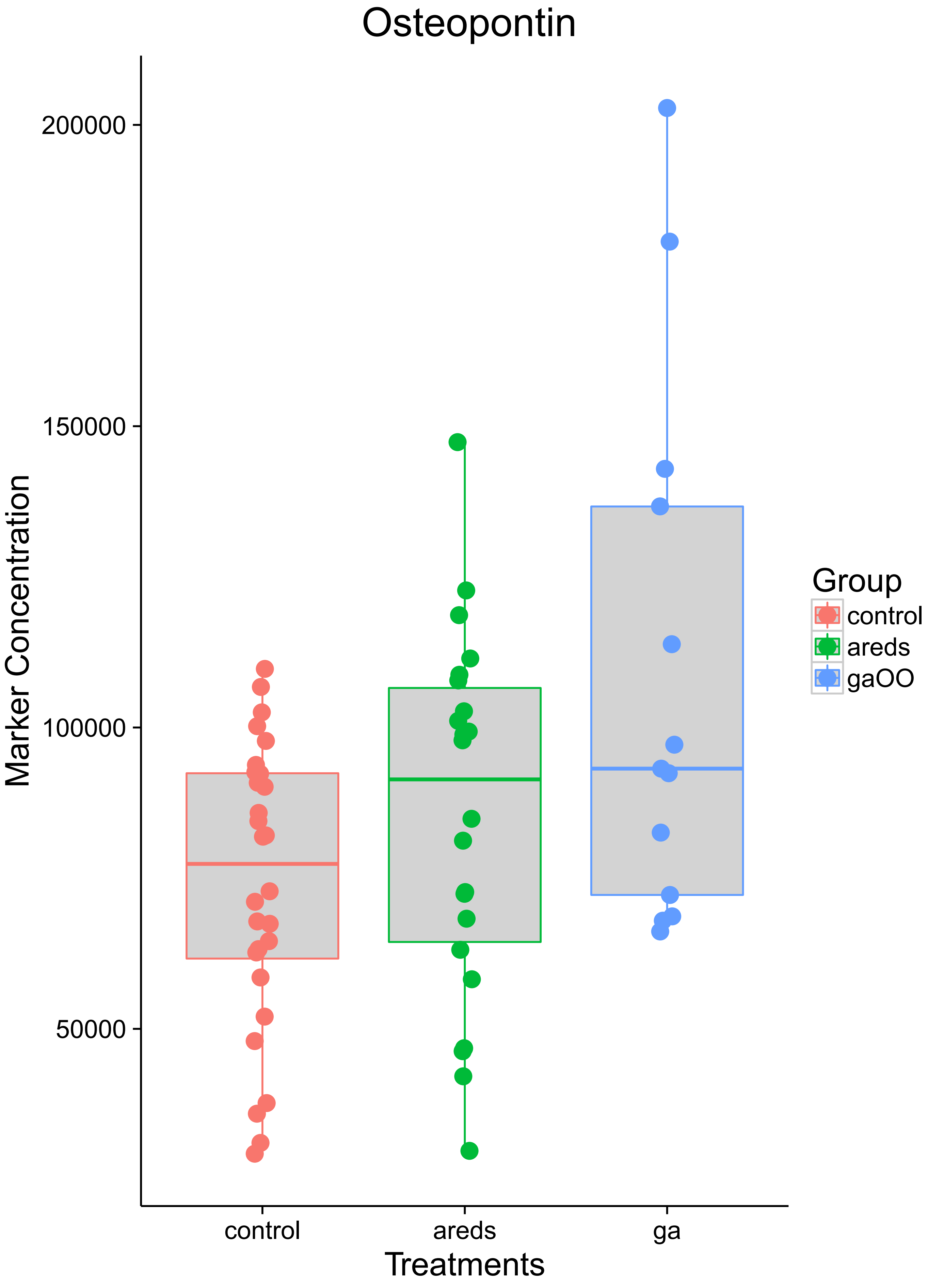

Osteopontin
Marker Concentration
200000
150000
100000
50000
control
areds
ga
Treatments
Group
control
areds
gaOO

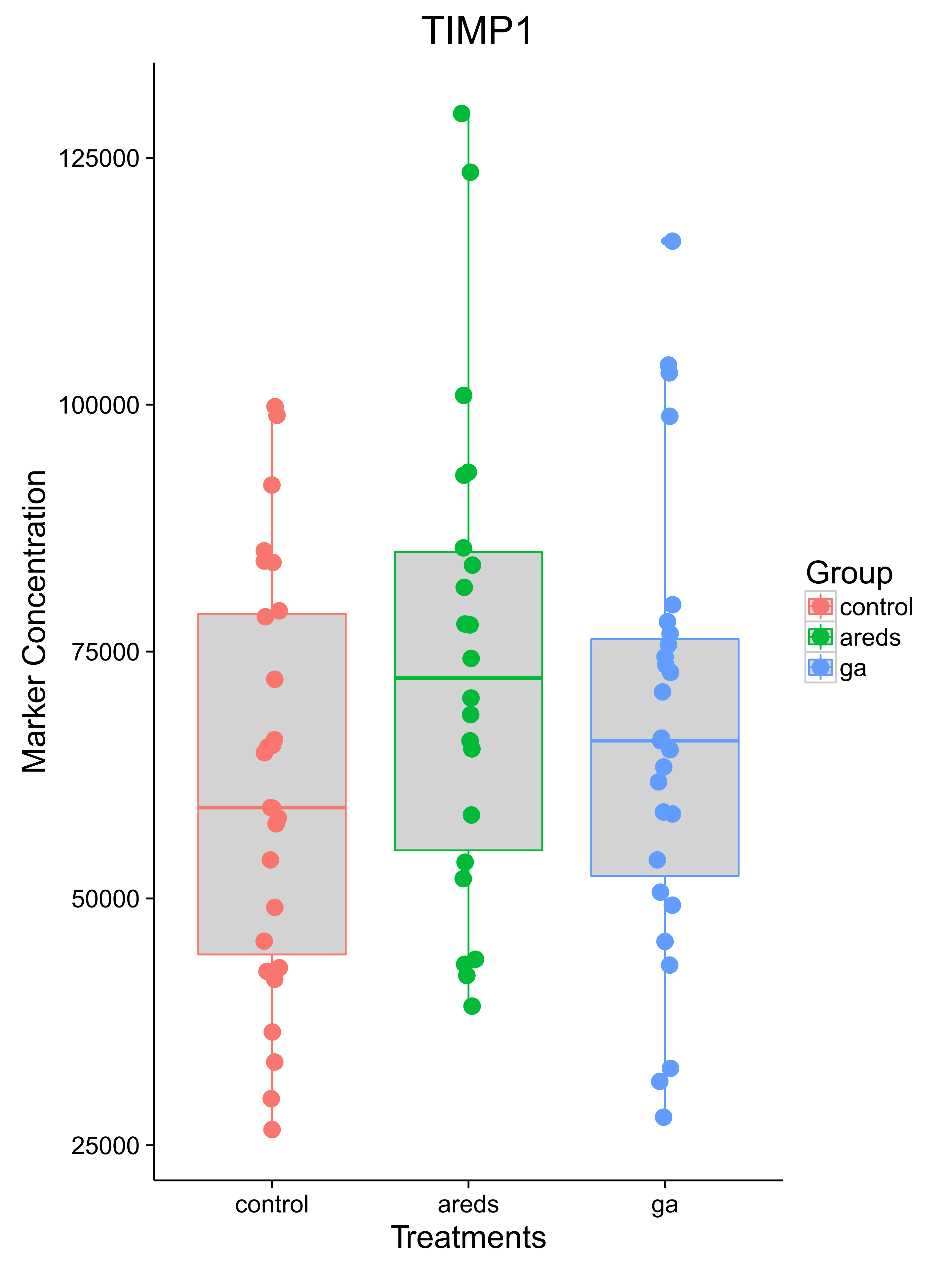
TIMP1
Marker Concentration
125000
100000
75000
50000
25000
control
areds
ga
Treatments
Group
control
areds
ga

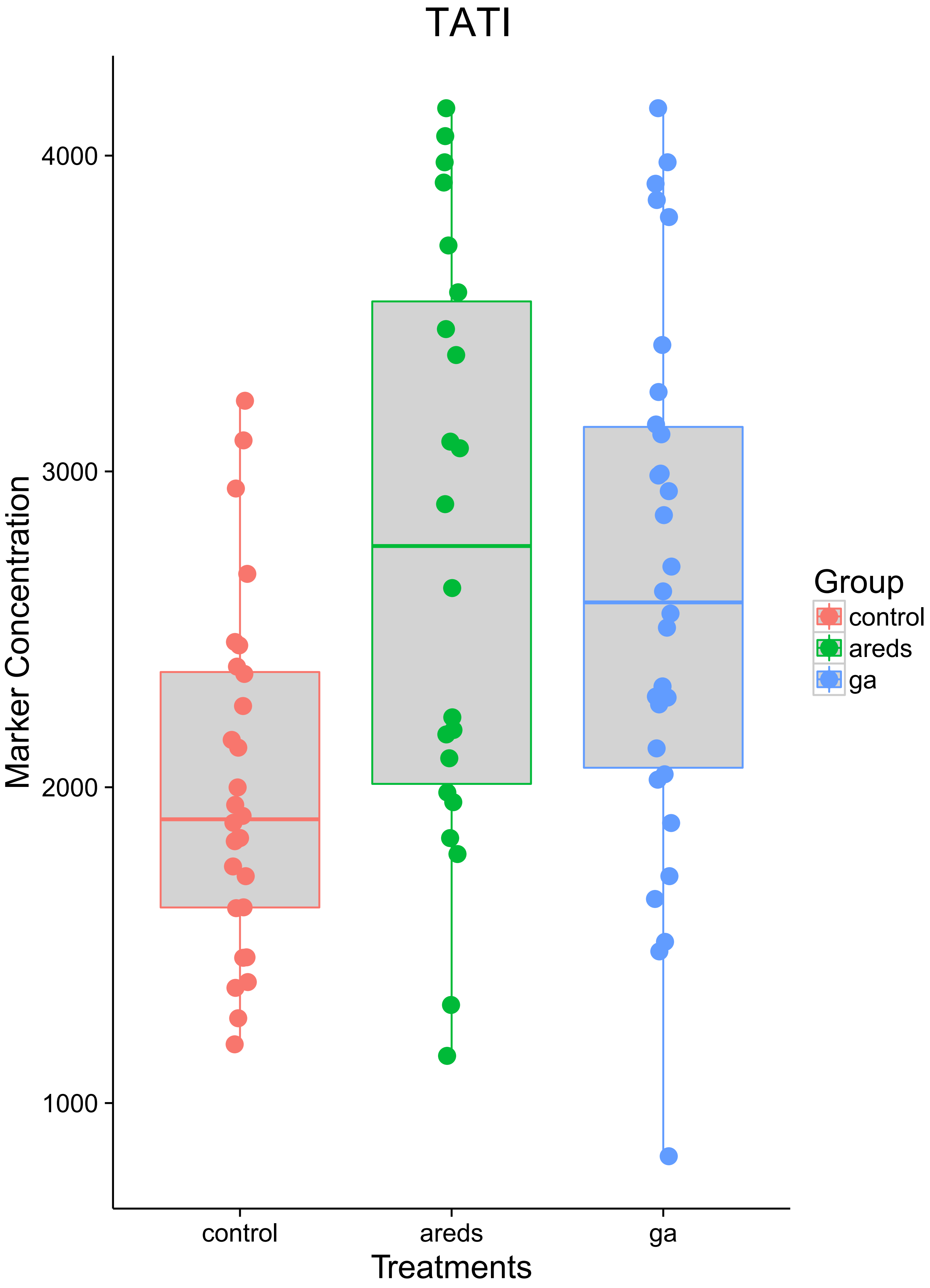

TATI
Marker Concentration
4000
3000
2000
1000
control
areds
ga
Treatments
Group
control
areds
ga

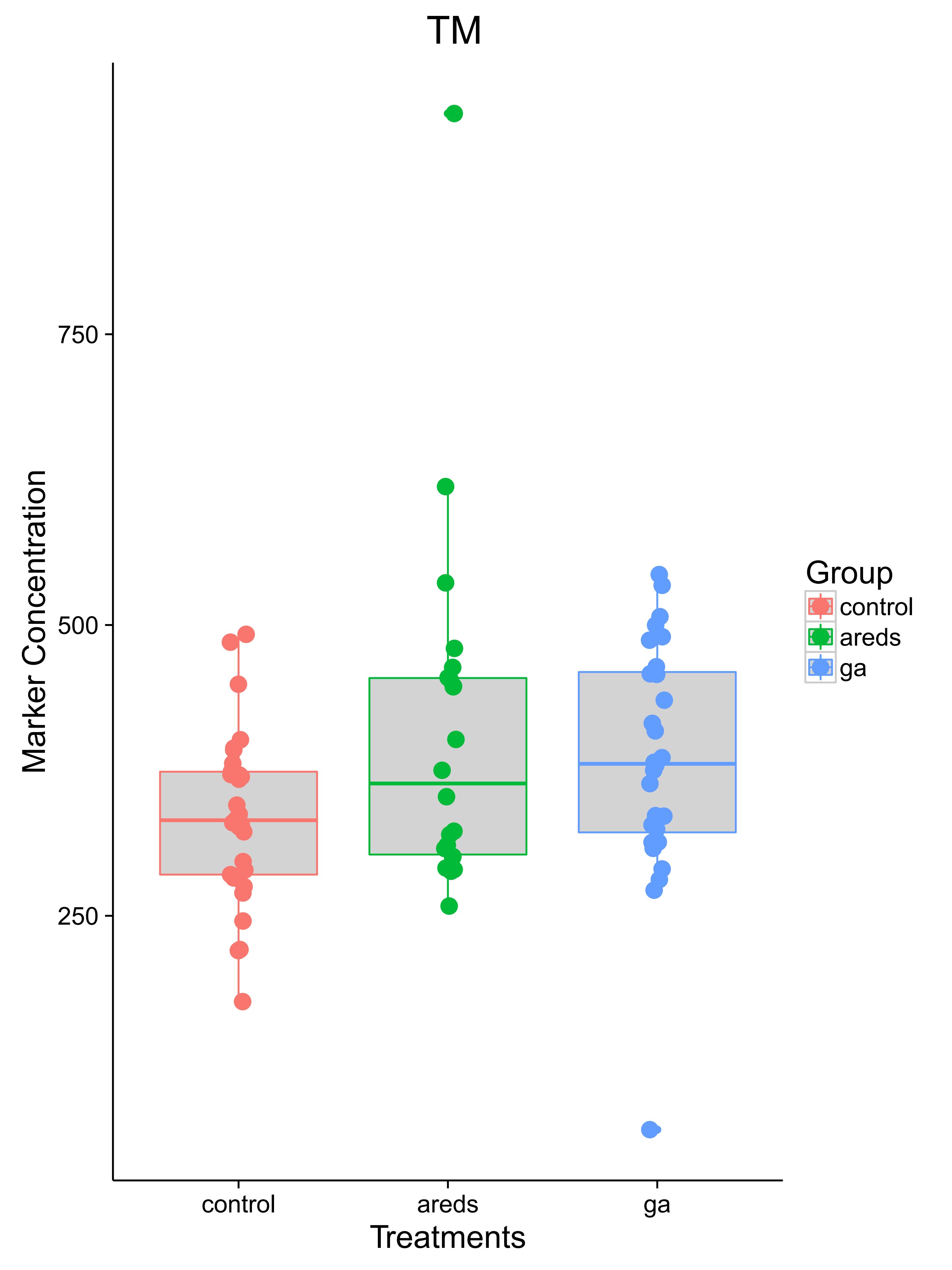

TM
Marker Concentration
750
500
250
control
areds
ga
Treatments
Group
control
areds
ga

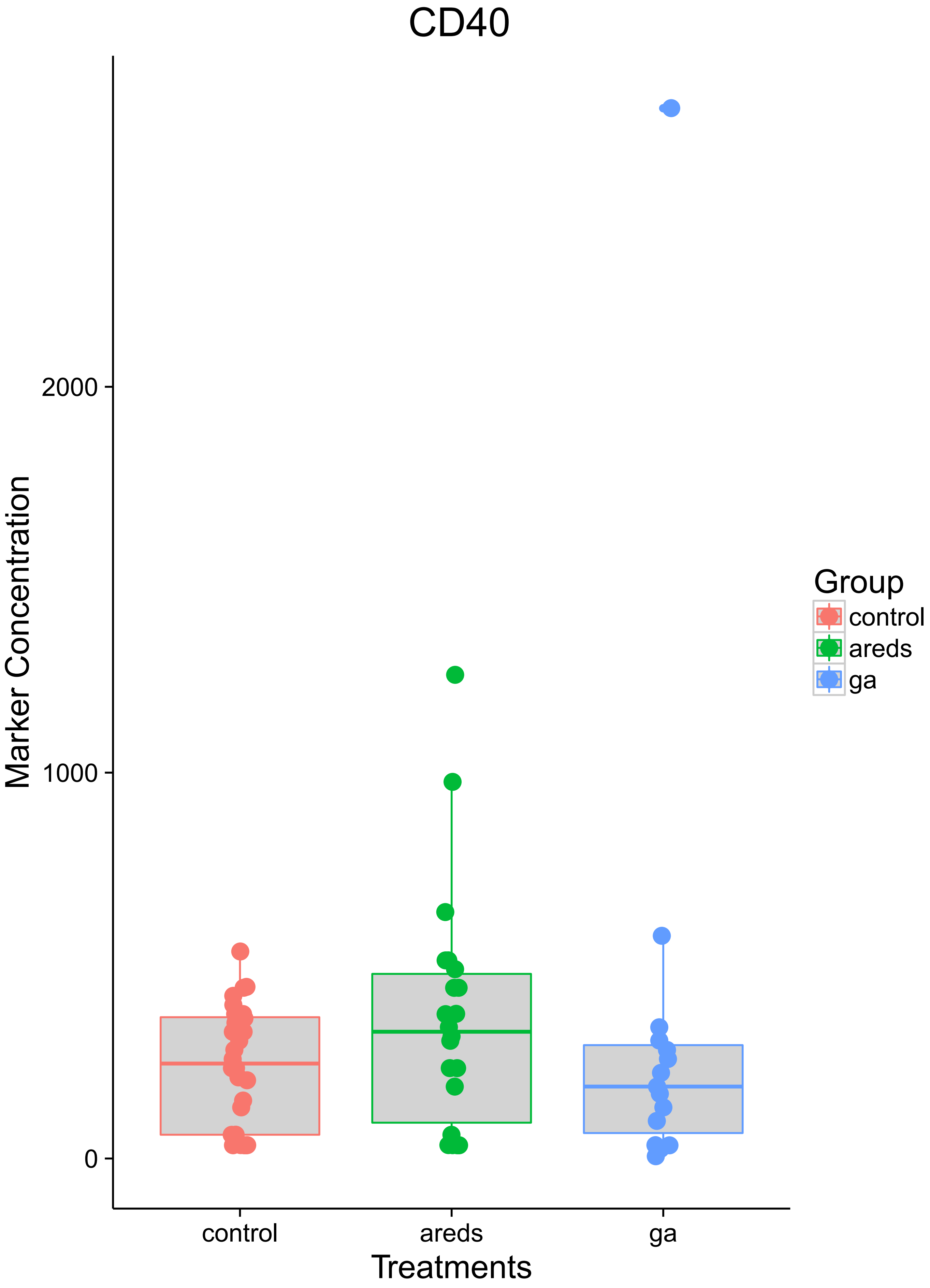

CD40
Marker Concentration
2000
1000
0
control
areds
ga
Treatments
Group
control
areds
ga

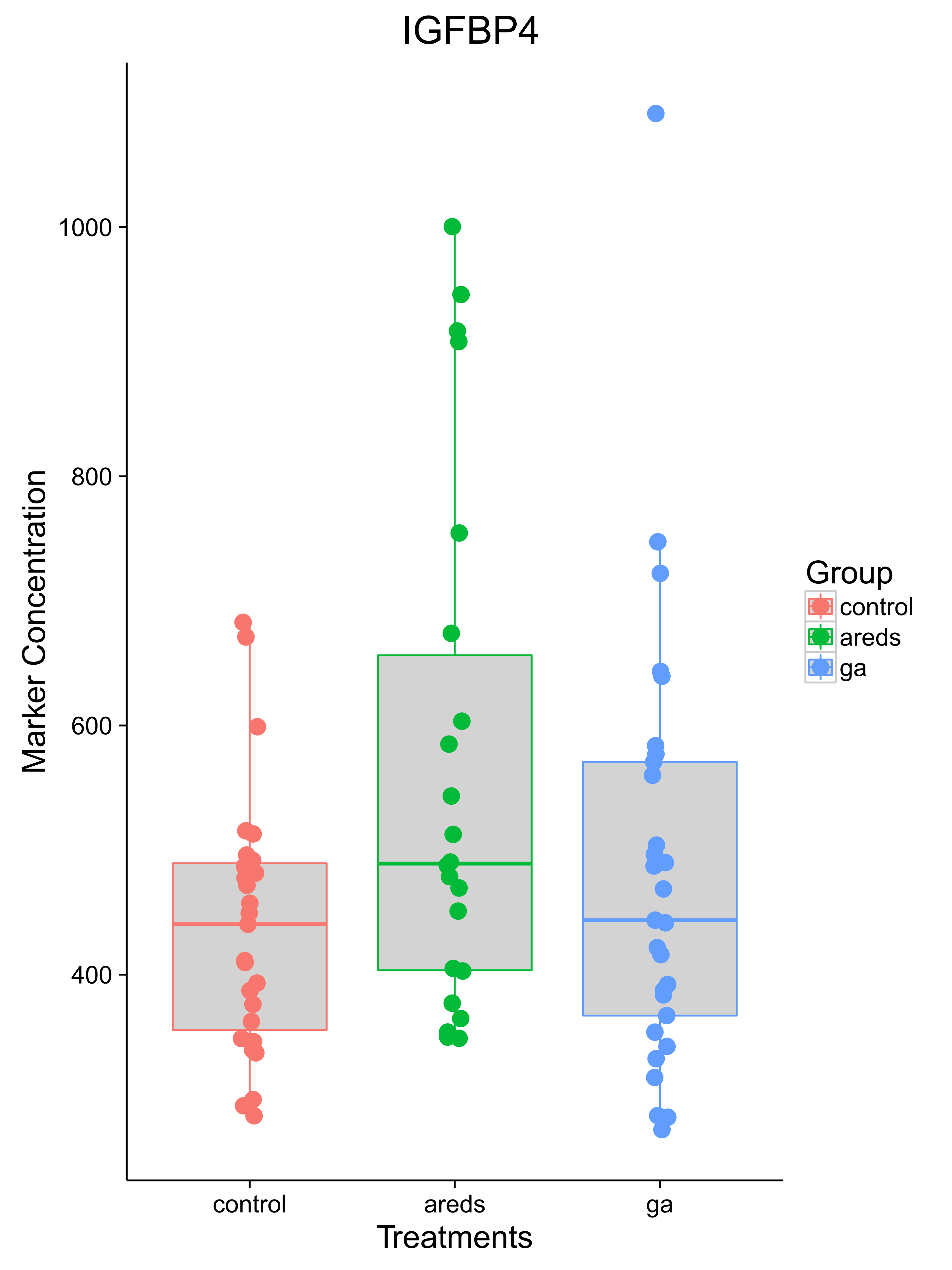
IGFBP4
1000
800
600
400
Marker Concentration
control
areds
ga
Treatments
Group
control
areds
ga

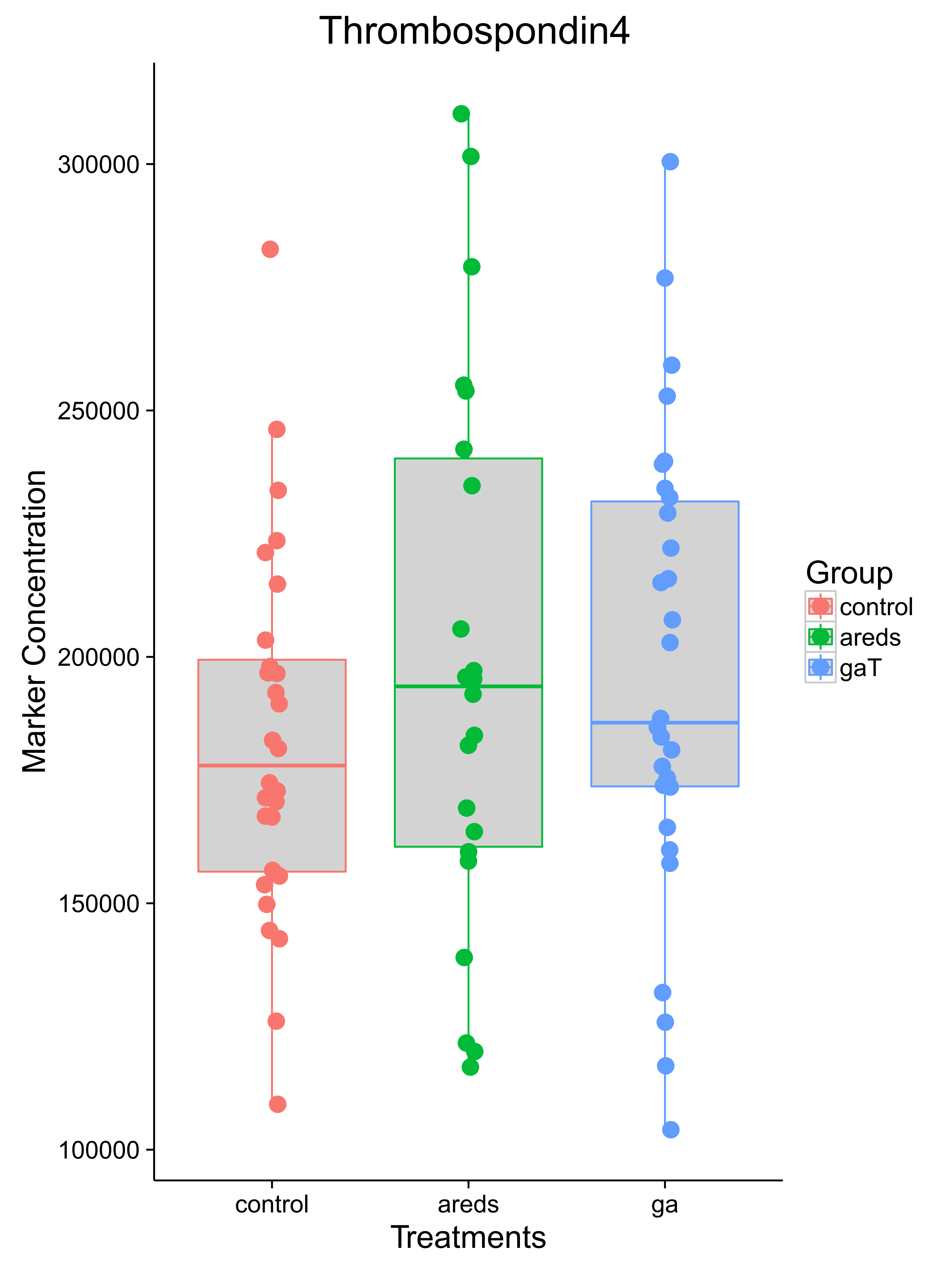

Thrombospondin4
Marker Concentration
300000
250000
200000
150000
100000
control
areds
ga
Treatments
Group
control
areds
gaT

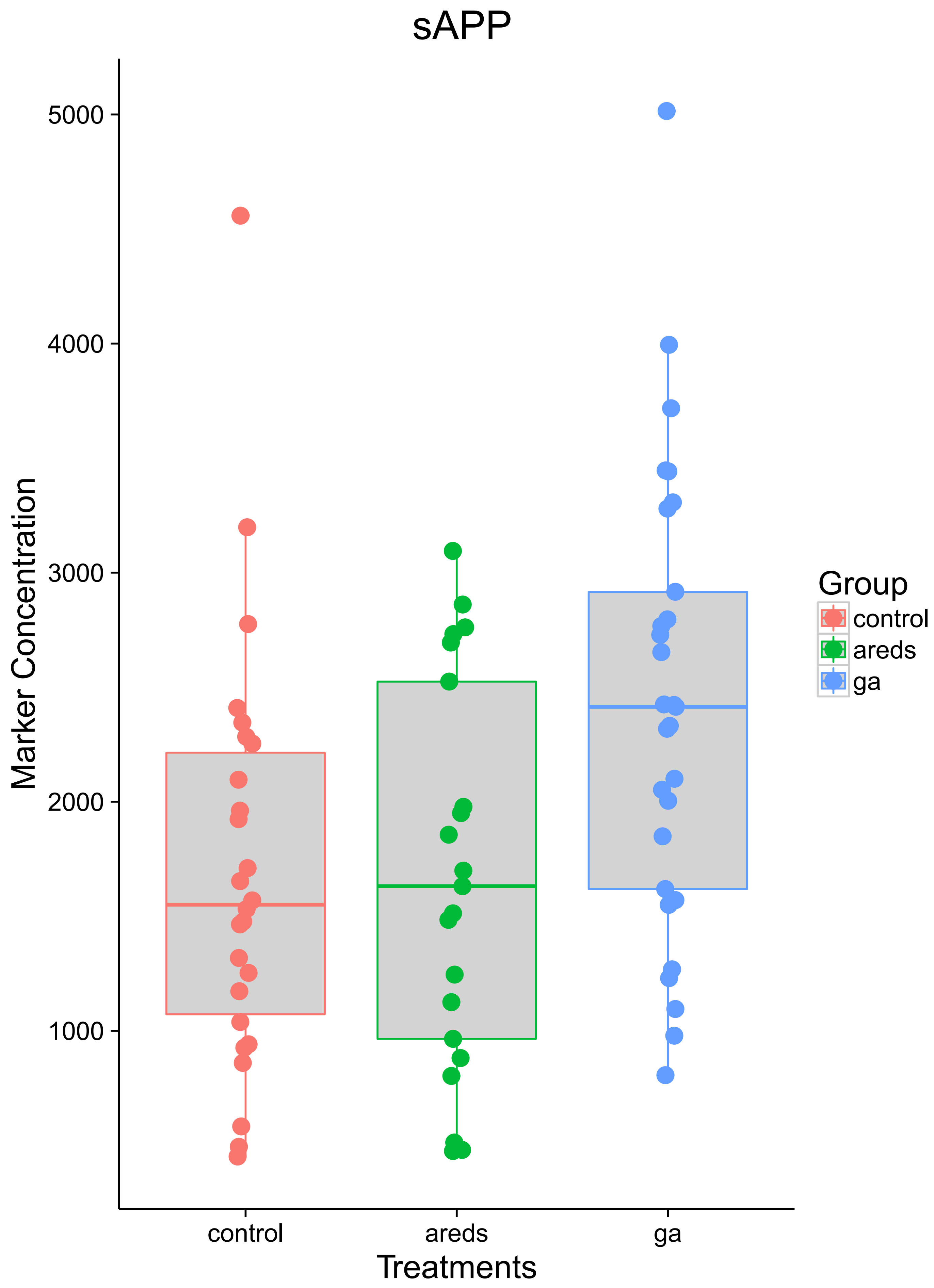
sAPP
5000
4000
3000
2000
1000
Marker Concentration
control
areds
ga
Treatments
Group
control
areds
ga

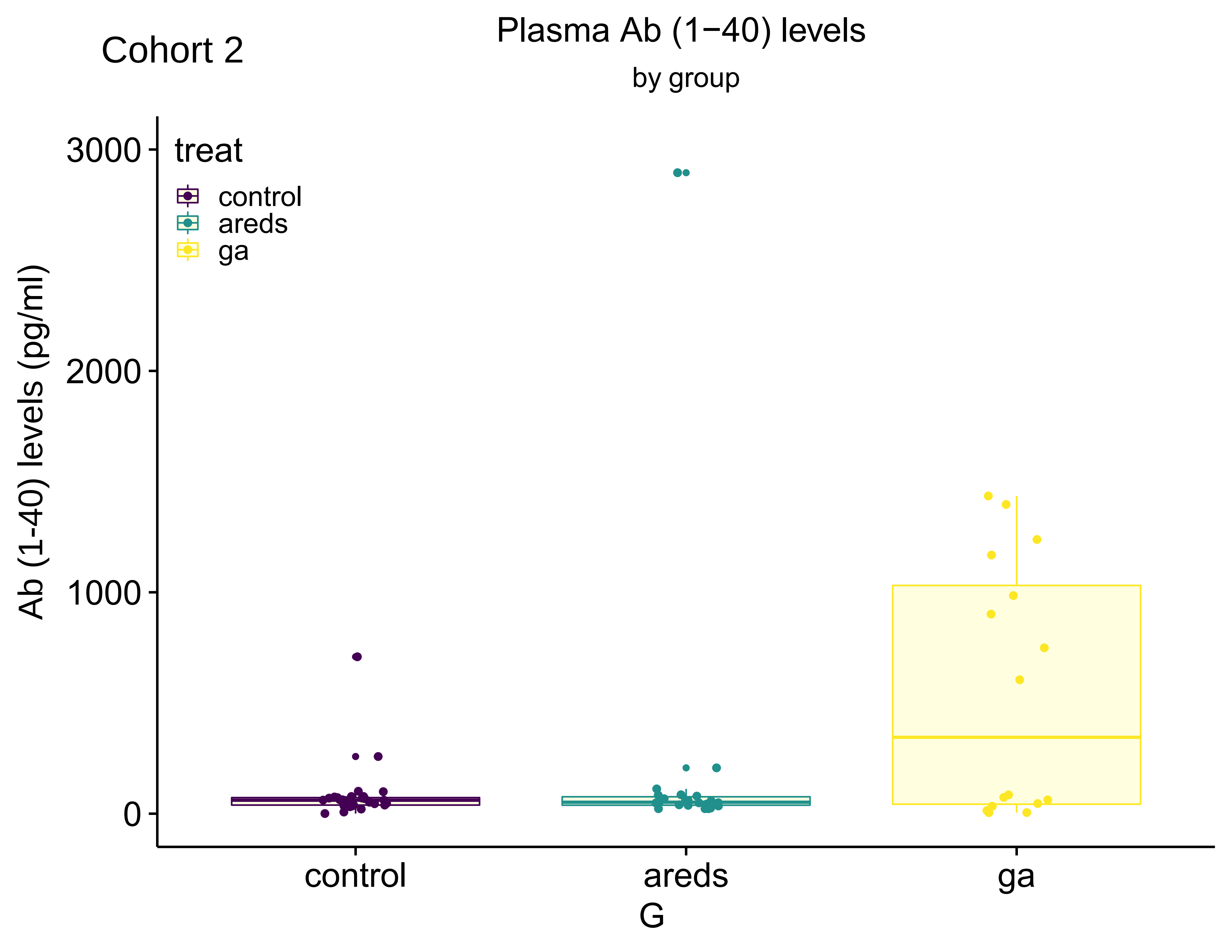
Cohort 2
Plasma Ab (1−40) levels
by group
Ab (1-40) levels (pg/ml)
3000
2000
1000
0
treat
control
areds
ga
control
areds
ga
G

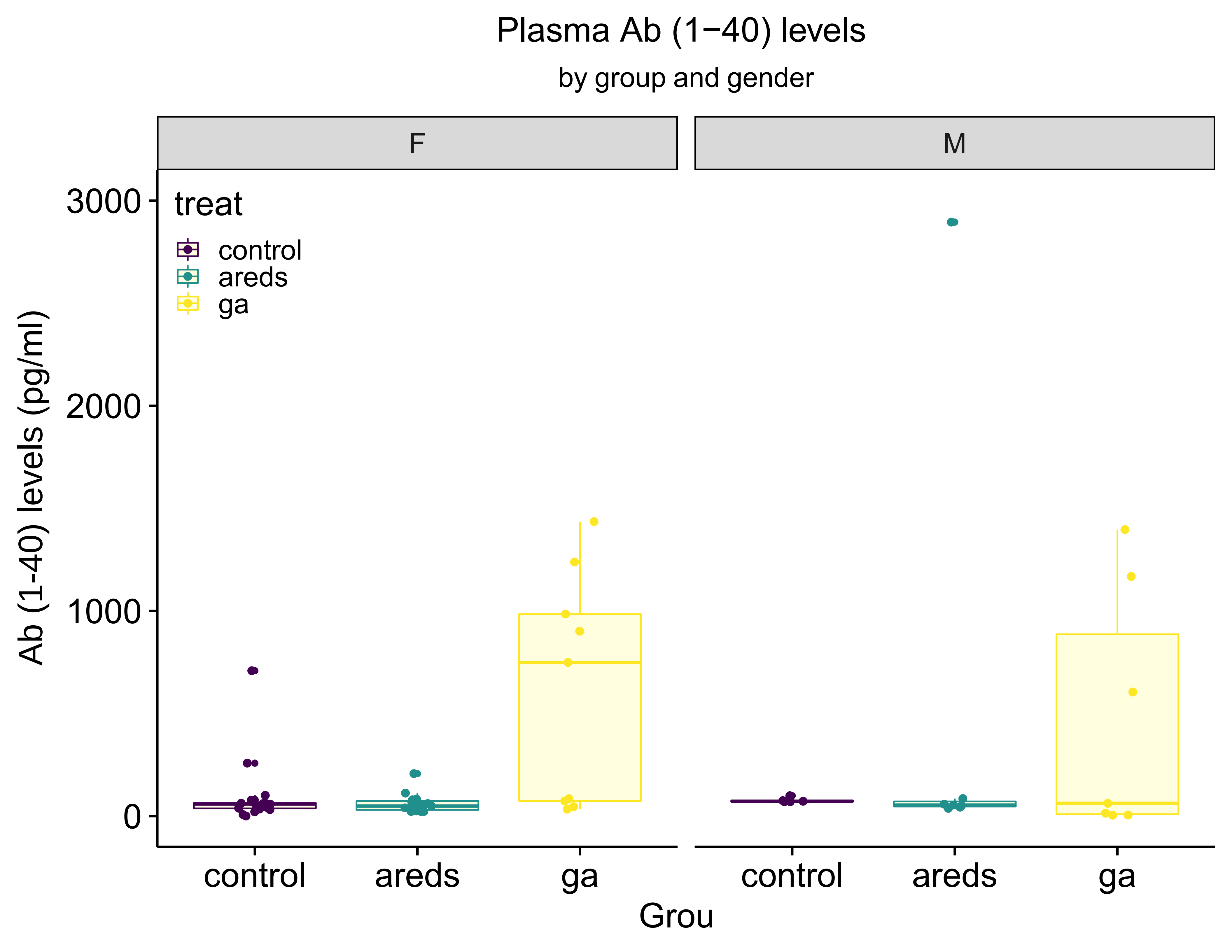

Plasma Ab (1−40) levels
by group and gender
F
M
treat
control
areds
ga
3000
2000
1000
0
Ab (1-40) levels (pg/ml)
control
areds
ga
control
areds
ga
Grou

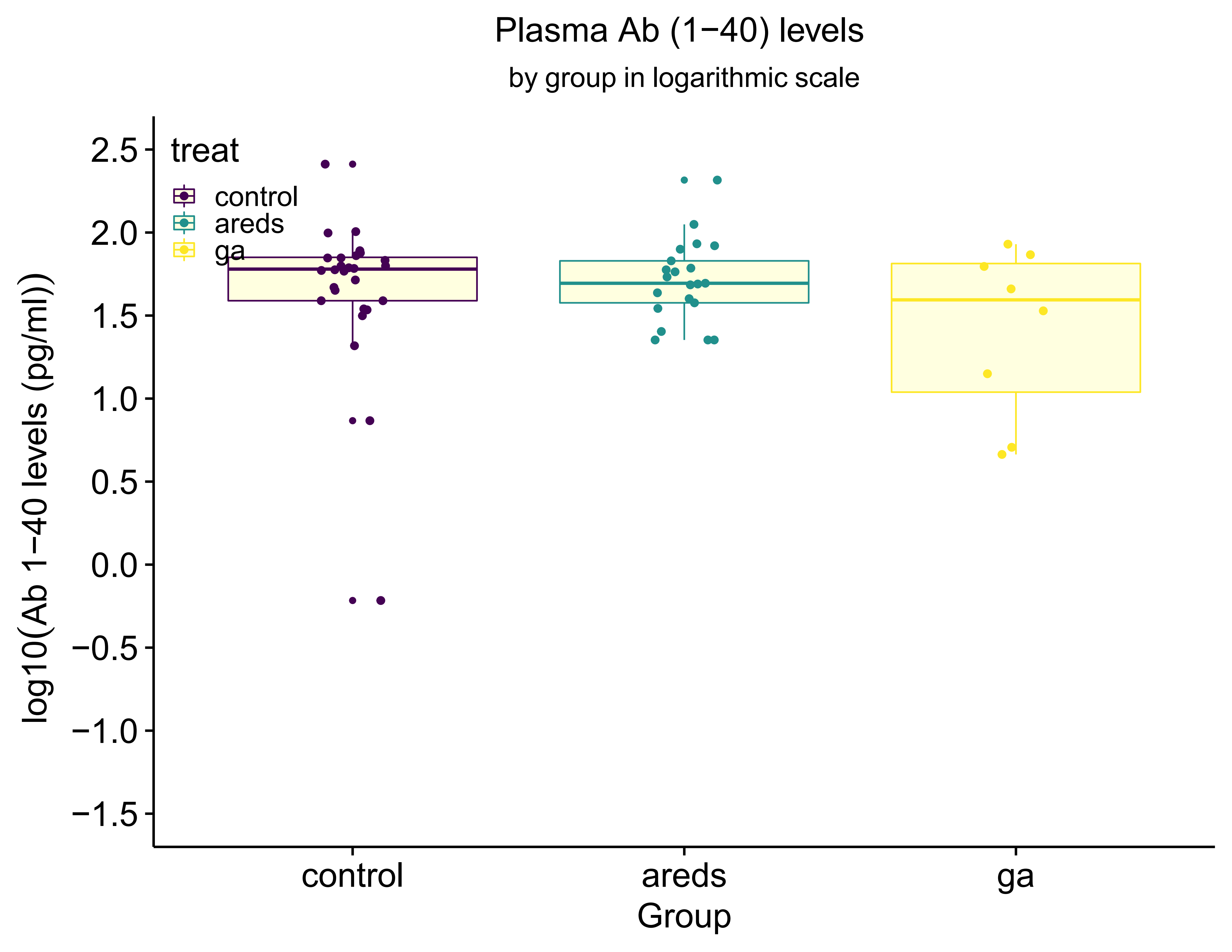
Plasma Ab (1−40) levels
by group in logarithmic scale
treat
control
areds
ga
log10(Ab 1−40 levels (pg/ml))
2.5
2.0
1.5
1.0
0.5
0.0
−0.5
−1.0
−1.5
control
areds
ga
Group

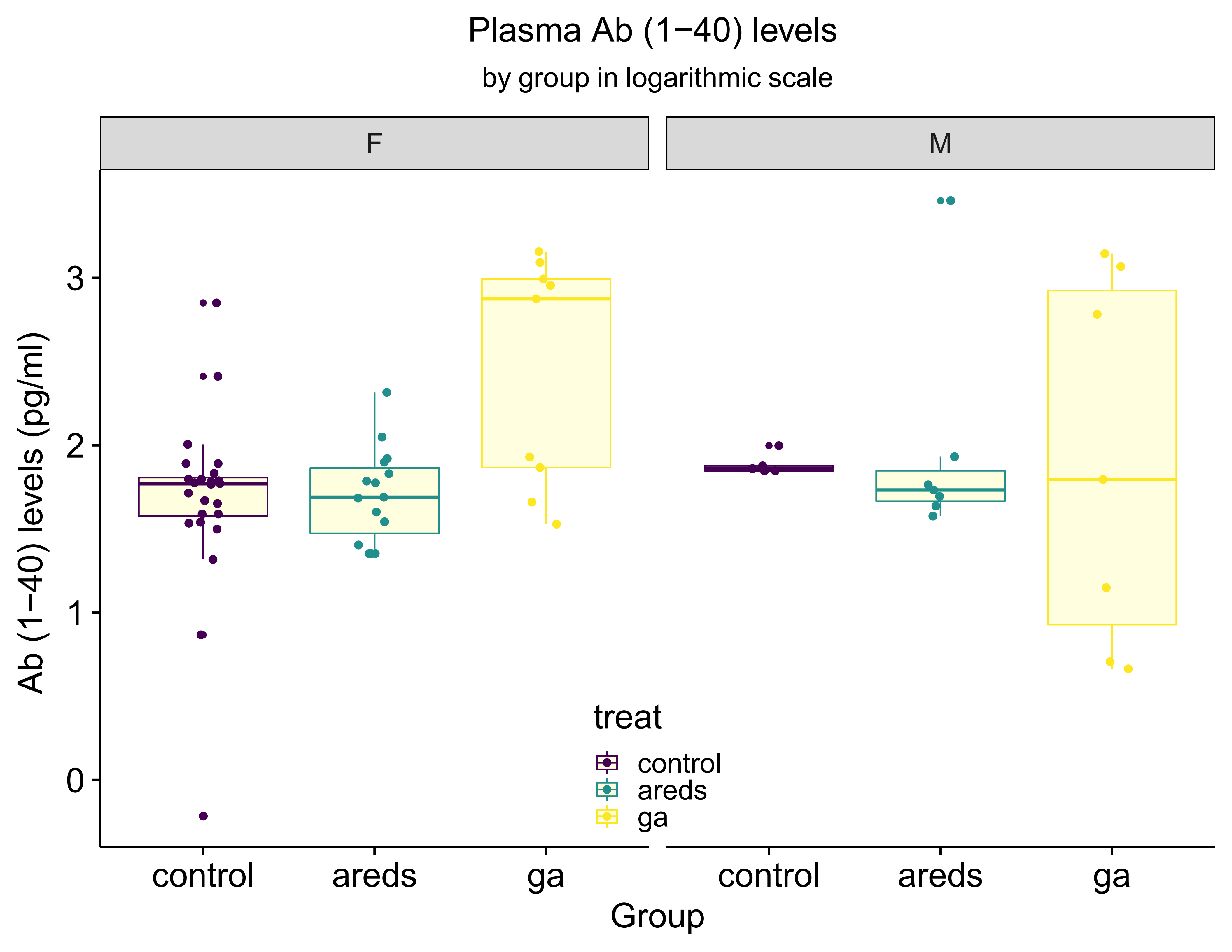

Plasma Ab (1−40) levels
by group in logarithmic scale
F
M
Ab (1−40) levels (pg/ml)
3
2
1
0
treat
control
areds
ga
control
areds
ga
control
areds
ga
Group

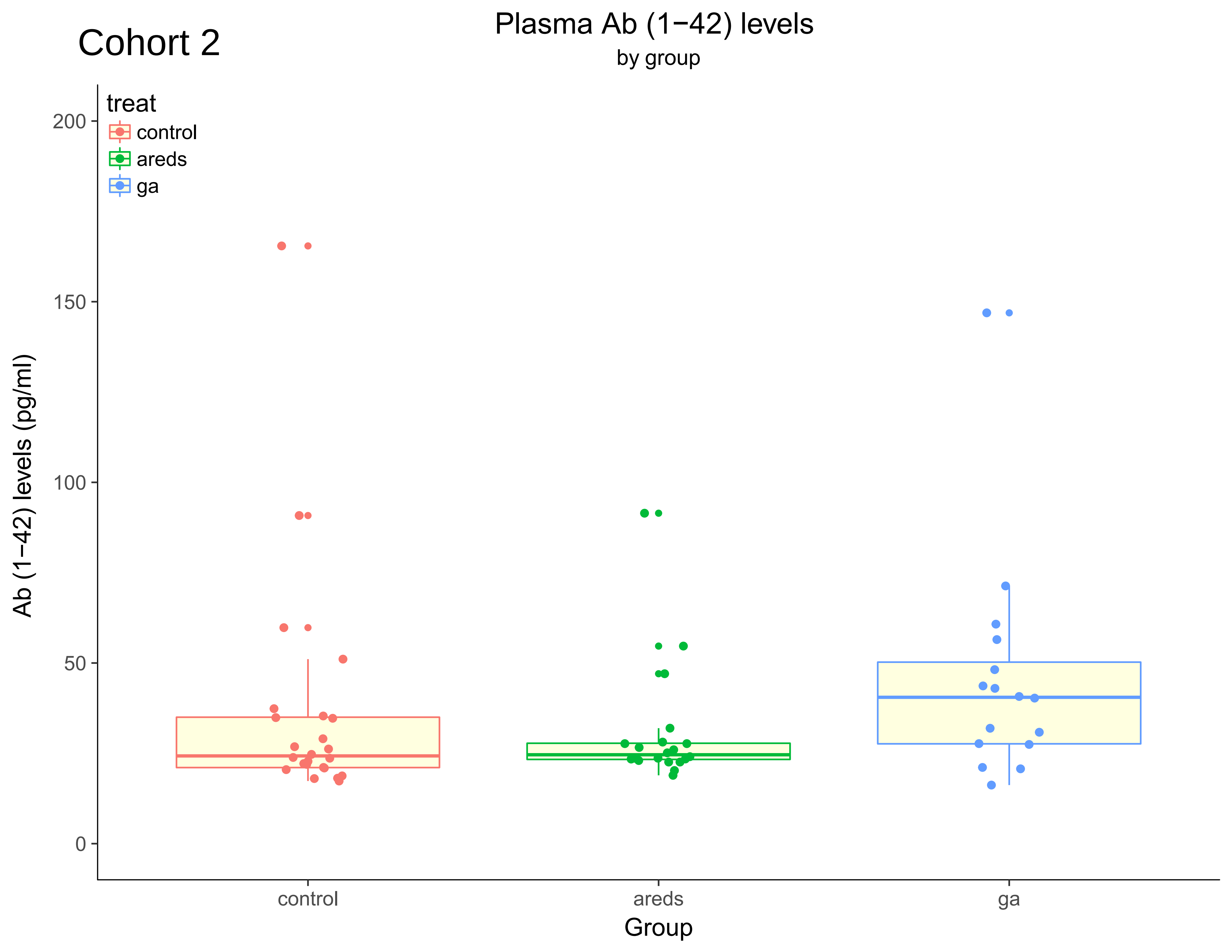

Cohort 2
Plasma Ab (1−42) levels
by group
treat
control
areds
ga
200
150
100
50
0
Ab (1−42) levels (pg/ml)
control
areds
ga
Group

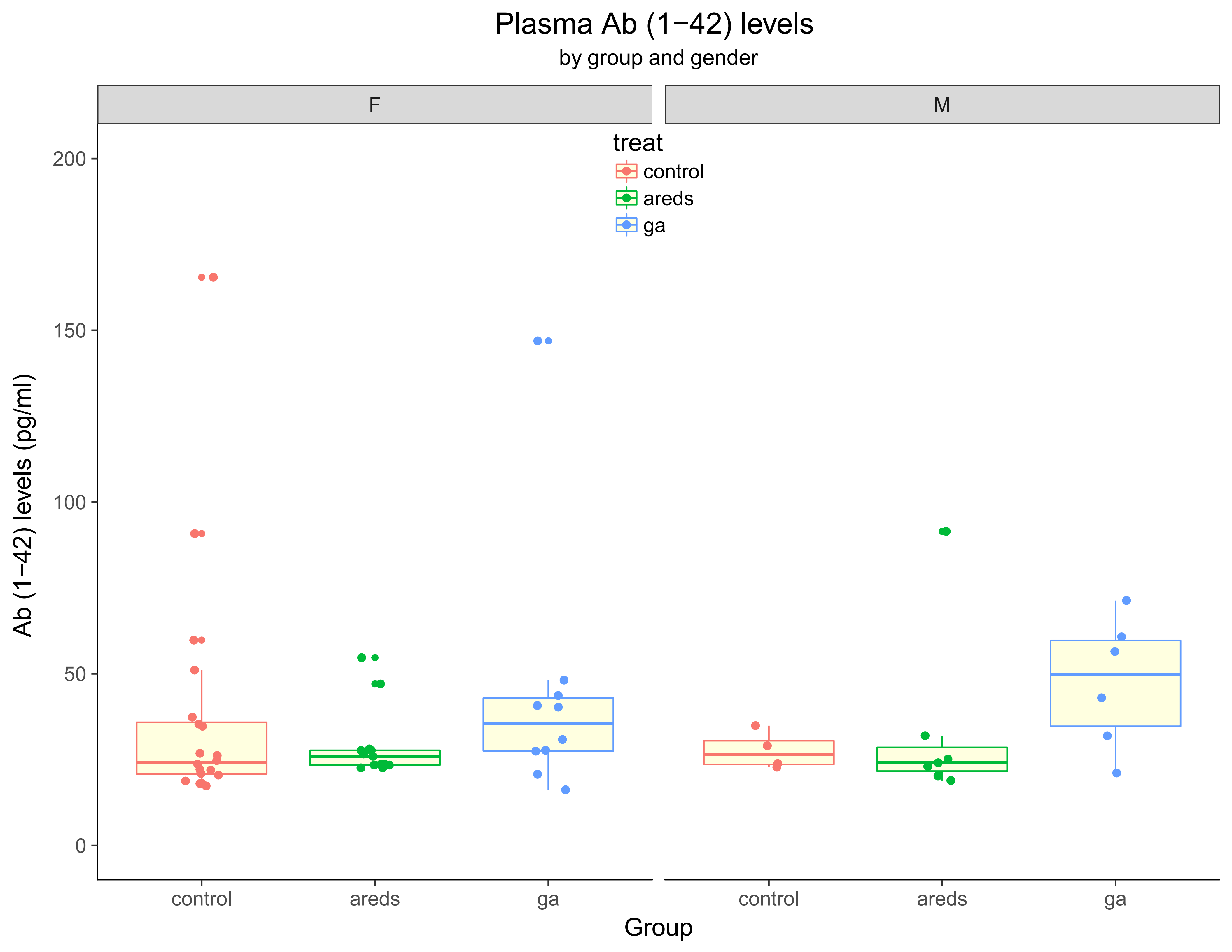

Plasma Ab (1−42) levels
by group and gender
F
M
treat
control
areds
ga
0
50
100
150
200
Ab (1−42) levels (pg/ml)
control
areds
ga
control
areds
ga
Group

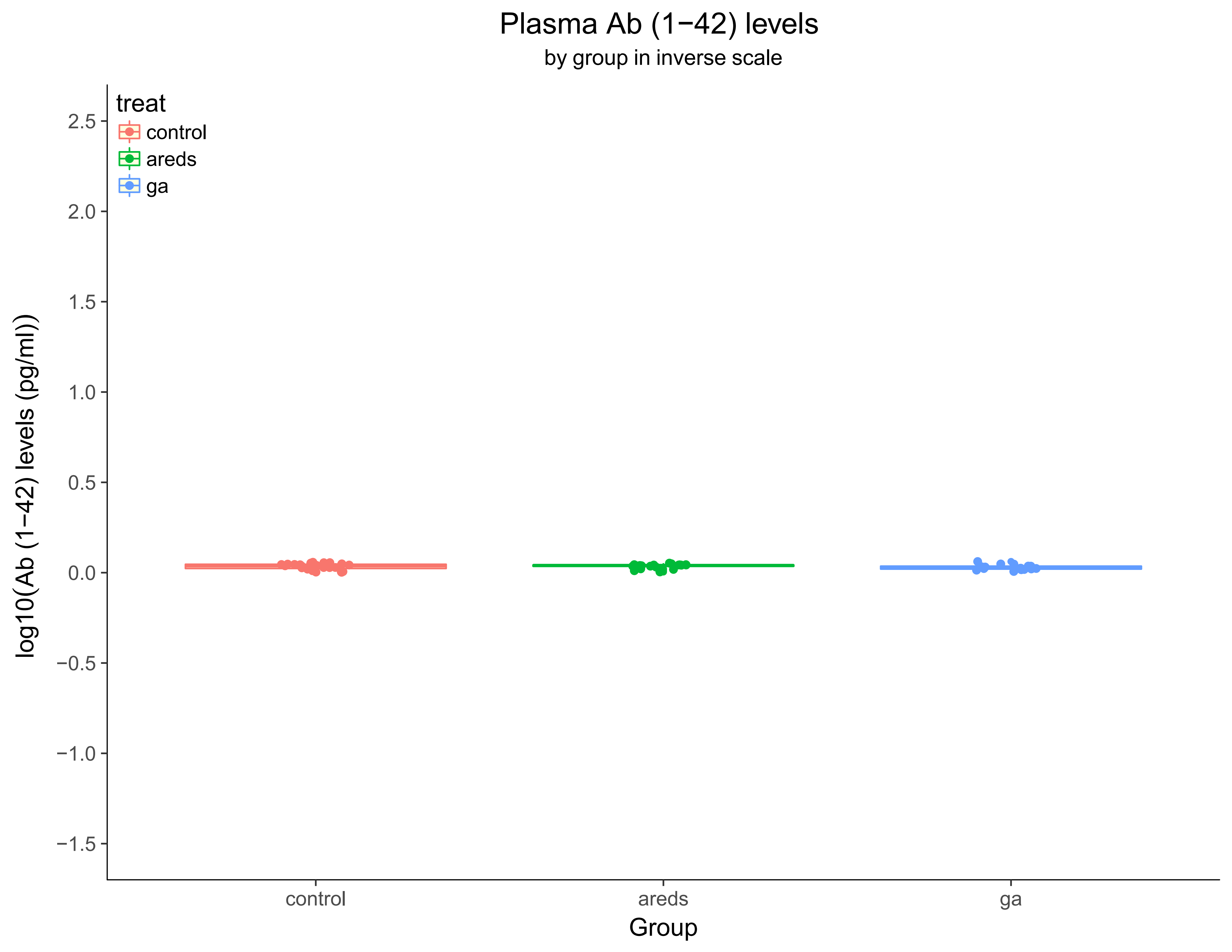
Plasma Ab (1−42) levels
by group in inverse scale
treat
control
areds
ga
2.5
2.0
1.5
1.0
0.5
0.0
−0.5
−1.0
−1.5
log10(Ab (1−42) levels (pg/ml))
control
areds
ga
Group

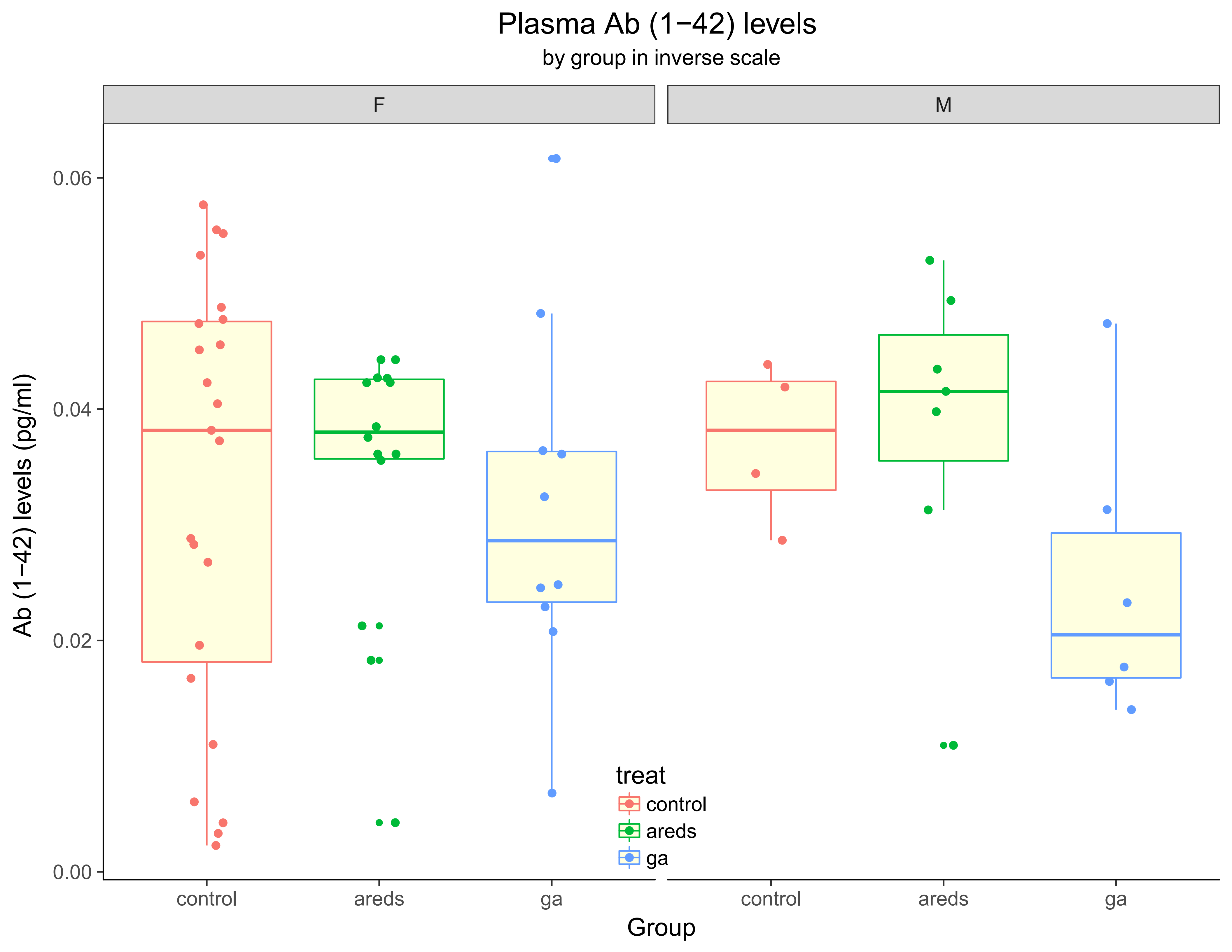

Plasma Ab (1−42) levels
by group in inverse scale
F
M
Ab (1−42) levels (pg/ml)
0.06
0.04
0.02
0.00
control
areds
ga
control
areds
ga
Group
treat
control
areds
ga

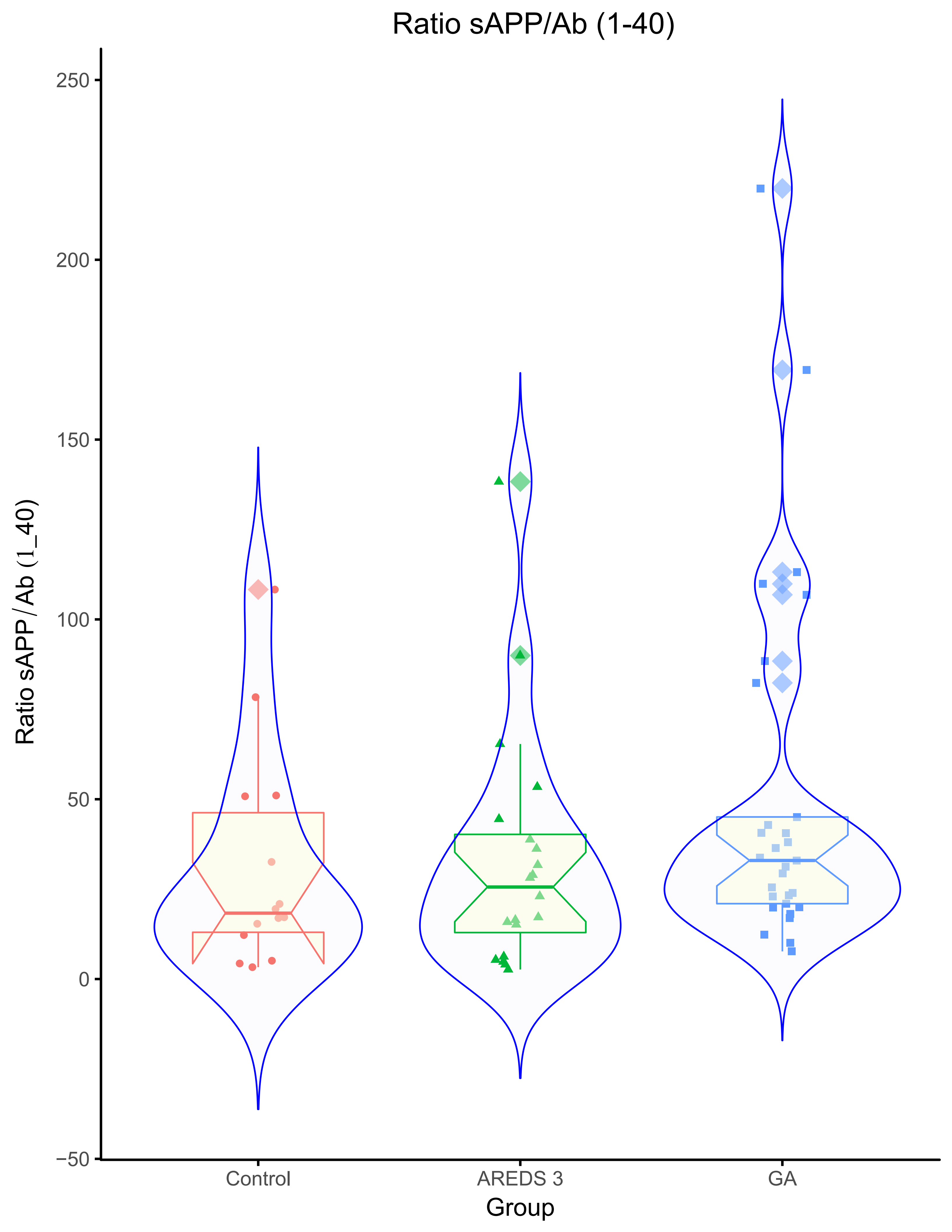

Ratio sAPP/Ab (1-40)
Ratio sAPP/Ab (1_40)
250
200
150
100
50
0
−50
Control
AREDS 3
GA
Group

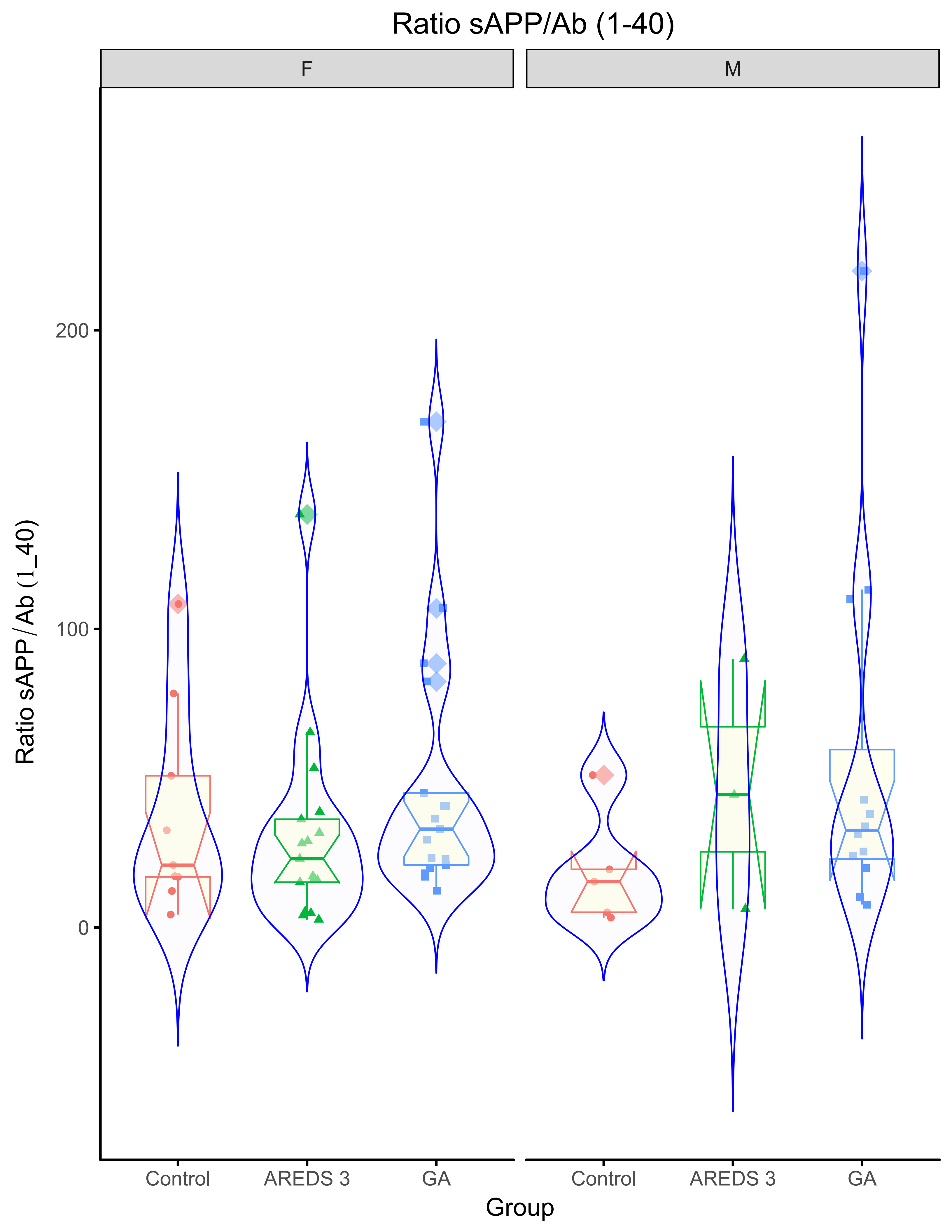

Ratio sAPP/Ab (1-40)
F
M
Ratio sAPP/Ab (1_40)
200
100
0
Control
AREDS 3
GA
Control
AREDS 3
GA
Group

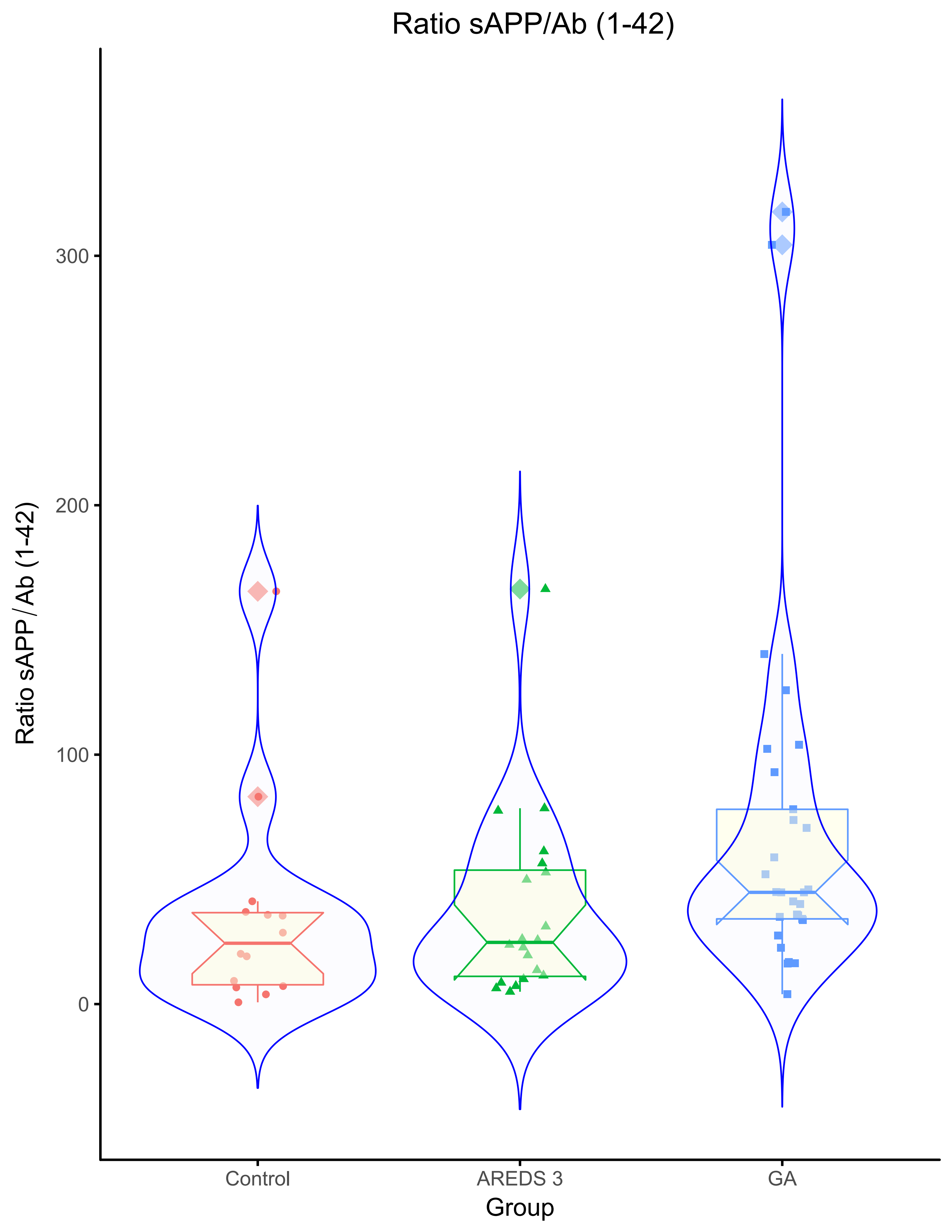

Ratio sAPP/Ab (1-42)
Ratio sAPP/Ab (1-42)
300
200
100
0
Control
AREDS 3
GA
Group

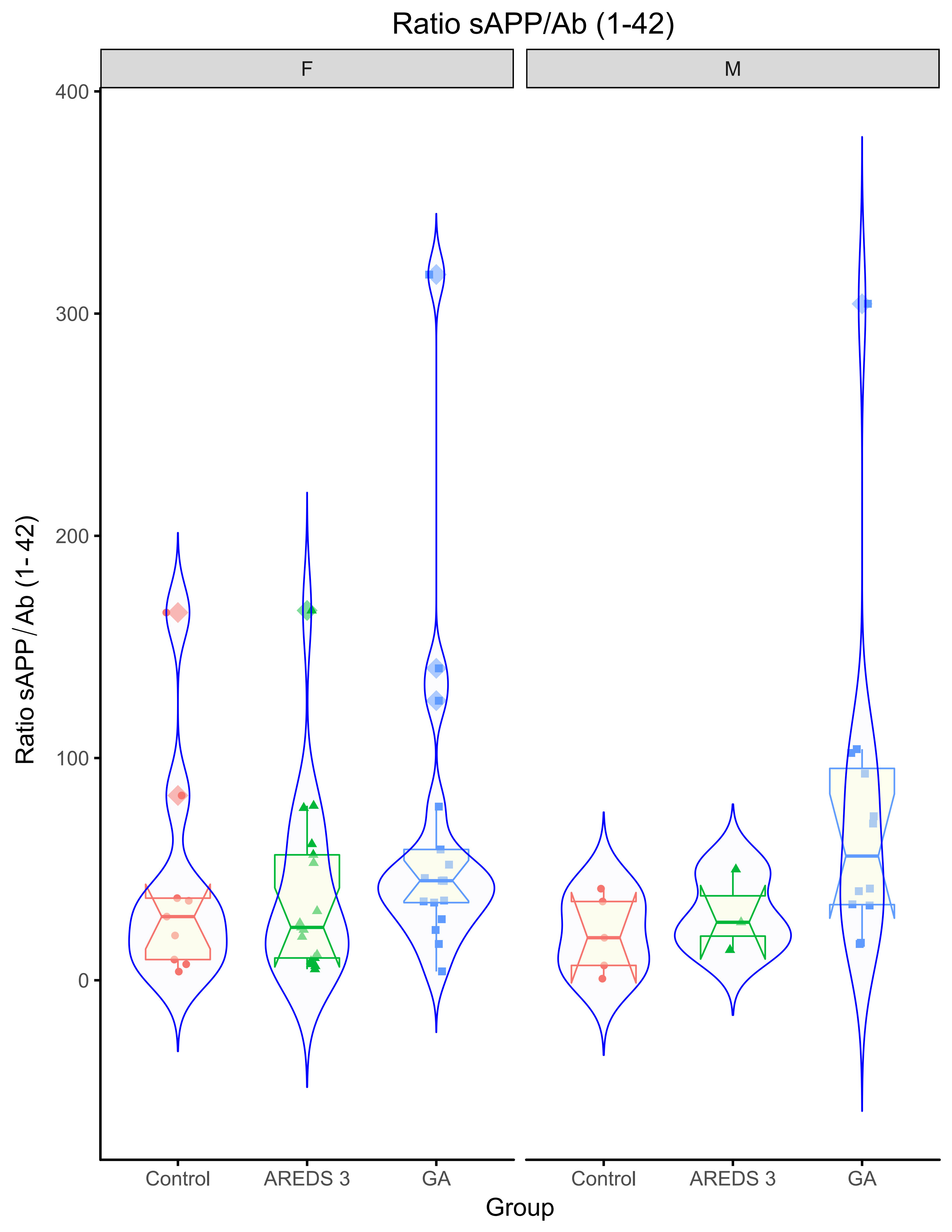
Ratio sAPP/Ab (1-42)
F
M
400
300
200
100
0
Ratio sAPP/Ab (1- 42)
Control
AREDS 3
GA
Control
AREDS 3
GA
Group

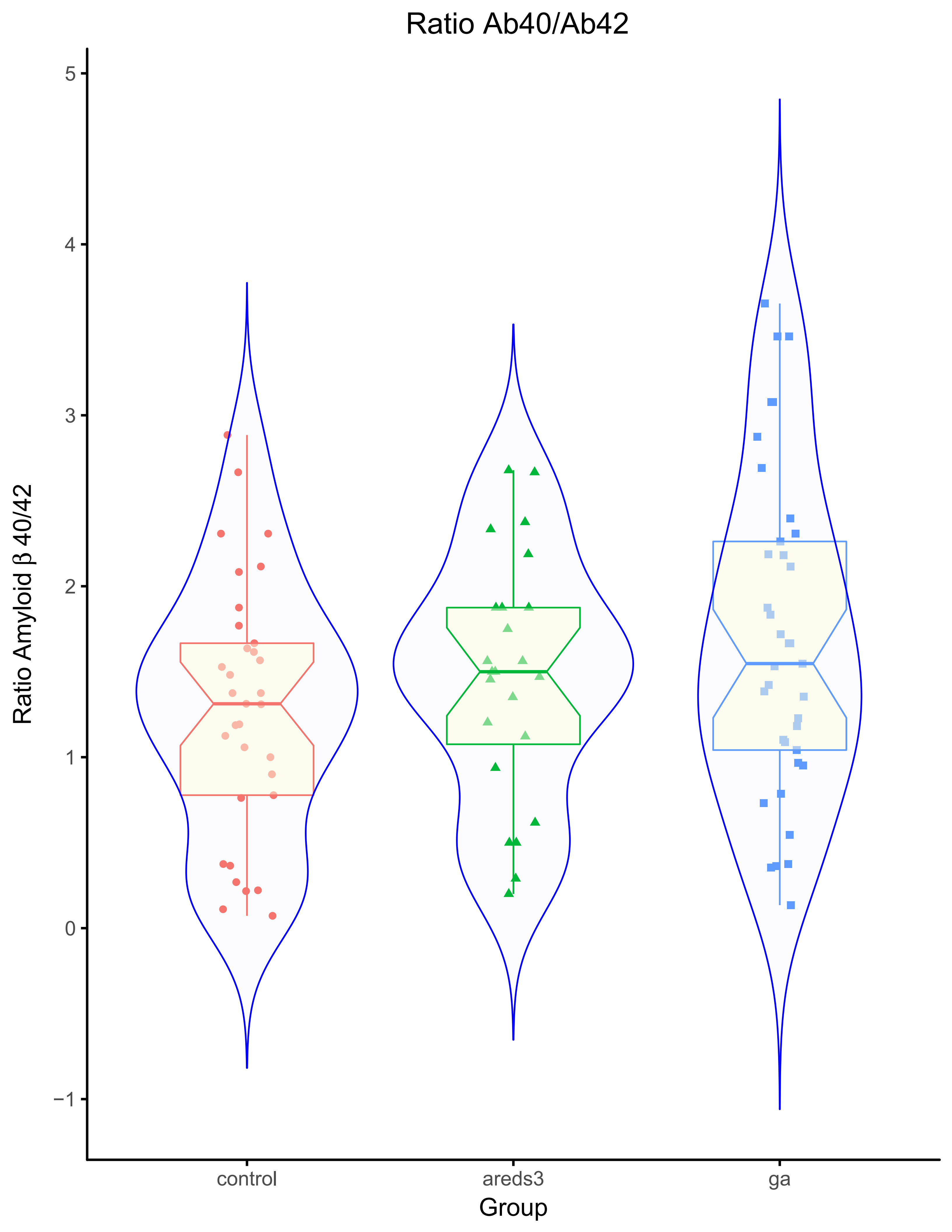

Ratio Ab40/Ab42
Ratio Amyloid β 40/42
5
4
3
2
1
0
−1
control
areds3
ga
Group

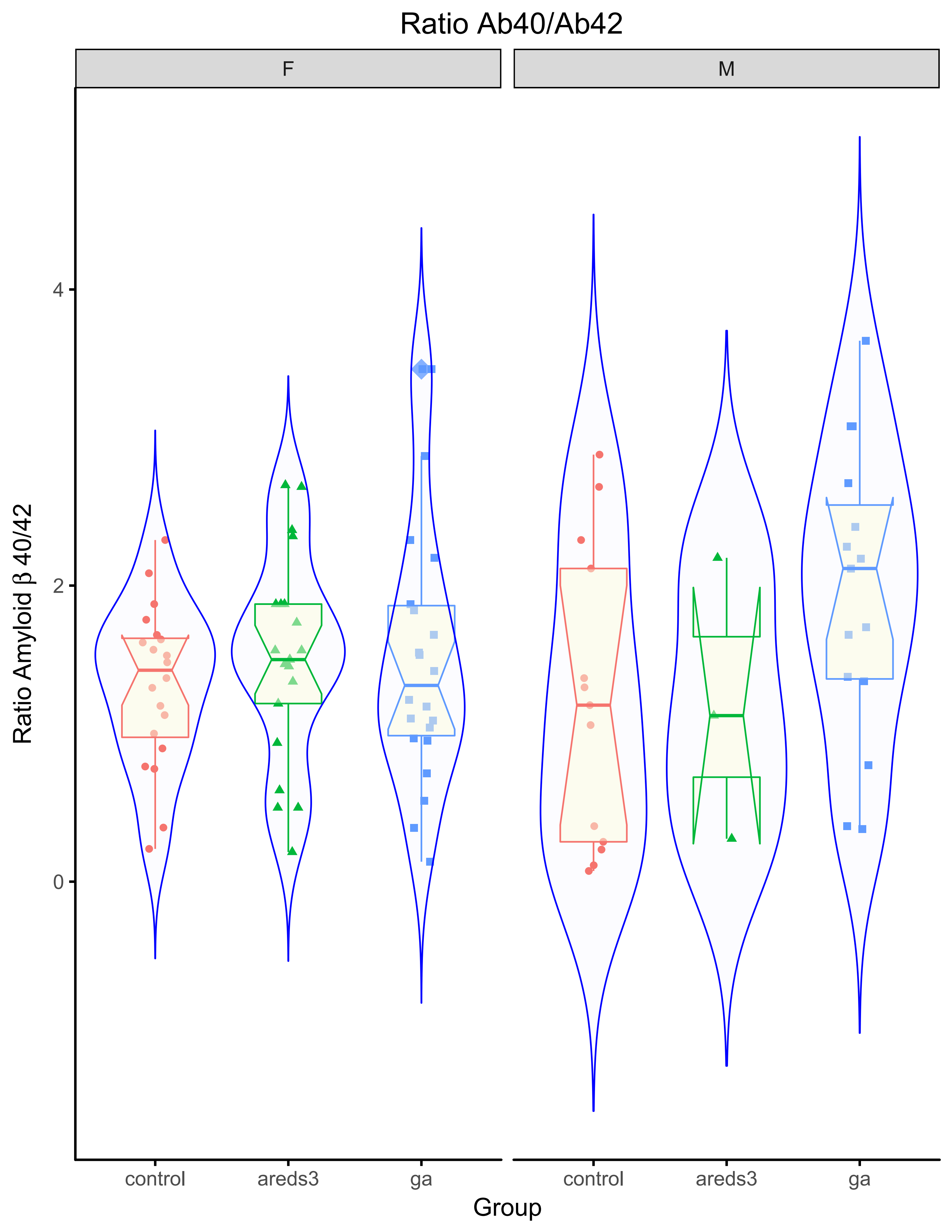

Ratio Ab40/Ab42
F
M
Ratio Amyloid β 40/42
4
2
0
control
areds3
ga
control
areds3
ga
Group
