# Supplementary material for: Plasma biomarkers of the amyloid pathway are associated with geographic atrophy secondary to age-related macular degeneration
Source: PLoS One. 2020 Aug 7;15(8):e0236283. doi: 10.1371/journal.pone.0236283 (PMC7413518; doi:10.1371/journal.pone.0236283)
Supplement: Attachment — Submitted filename: Response to Reviewers.docx [file pone.0236283.s009.docx]

RESPONSE TO REVIEWER #1

Please use the space provided to explain your answers to the questions above. You may also include additional comments for the author, including concerns about dual publication, research ethics, or publication ethics. (Please upload your review as an attachment if it exceeds 20,000 characters)

On behalf of the coauthors, we would like to thank both Reviewers for their detailed analysis, for their thoughtful suggestions and helpful comments. We have followed their instructions and feel that this has improved the quality of this manuscript. We have included our responses to all your questions in BLUE text.

**Reviewer #1:**

This a relatively small scale study searching for plasma biomarkers which may indicate the presence of GA as a consequence of AMD.

We appreciated Reviewer #1’s comments and detailed review of the manuscript. This was an exploratory study. To this end we recruited a total of 180 subjects into 2 cohorts: 94 subjects in Cohort 1 and 86 subjects in Cohort 2. This study was designed to recruit enough subjects in each cohort to produce meaningful statistical results. The results from this study were going to provide information for the development program of GSK’s anti-amyloid beta antibody for geographic atrophy (GA) and the identified markers were going to be evaluated during the late phase clinical studies for the program.

The comment by Reviewer #1 that we were “searching for plasma biomarkers which may indicate presence of GA” is somewhat misleading. The rationale for the study was to investigate whether there were any biomarkers associated specifically with GA, which could provide some input into the pathophysiology of the disease. In addition, these markers were going to be used to as potential surrogate biomarkers for assessing the effectiveness of the anti-amyloid beta antibody in patients with GA. We have added this information to the introductory statement to clarify this issue.

The rationale for the study is somewhat poorly explained. There is no real need for a predictive biomarker of late-stage disease, since it is readily detectable both from symptomatology and retinal imaging.

The rationale for this study is stated in the last paragraph of the Introduction. The purpose of the study was NOT to use biomarker data as a diagnostic or predictive modality for GA. We are well aware that there are established imaging modalities for proper diagnosis of GA besides OCT: fundus autofluorescence imaging, near infra-red (NIR) imaging and fundus photography. There are numerous biomarkers that have been associated with AMD in the literature. However, to our knowledge, there are no non-genetic plasma/tissue biomarkers that are specific to GA.

We agree that GA is a late onset consequence of AMD. Having said that, not all eyes with intermediate dry AMD progress to GA. Furthermore, GA lesions expand at different rates. For these reasons, we embarked on identifying whether there were any biomarkers that were specific to GA (versus intermediate dry AMD) to explain its pathophysiology. As mentioned earlier, this study was part of GSK’s efforts to identify candidate surrogate biomarkers for therapeutic responses in GA therapy using their anti-amyloid monoclonal antibody. Measurements of GA progression based on imaging studies is slow and tedious. Identification of a surrogate biomarkers would hasten identification of therapeutic response to treatment.

I take the point that such studies could inform on the underlying biology of disease development, but this manuscript makes no apparent effort to confirm e.g. that the identified circulating biomarkers are indeed also altered in their expression at the back of the eye, and so, while interesting, the study as it stands lacks conviction as to either the mechanistic value of the observed findings, or indeed the value of these proteins as biomarkers of disease progression, rather than just presence.

We politely disagree with the reviewer. Some of the biomarkers that we have identified in this study have been indeed identified as components of drusen and basal linear deposits (BLinD) in pathological eyes with AMD. There are numerous publications that document deposition of amyloid moieties and other inflammatory proteins in AMD within the photoreceptor-RPE complex and especially within drusen deposits. Several other factors including MMPs, tPA, pentraxin3, serine proteases, among others that we have identified in this study have been implicated in anatomical changes observed in early-late AMD and localized within the photoreceptor-RPE-choriocapillaris complex. As Reviewer #2 has indicated in their review of our study, these inflammatory proteins are detected in part due to changes in the extracellular matrix and tissue remodeling and general loss of homeostasis observed in GA, especially within the transition zone.

Since this study was not designed to prospectively collect more than 1 specimen from each study subject, we are not able to address whether these biomarkers are associated with disease progression (indeed, this was the goal of the next phase of these studies), but we do identify their associations with GA. However, again, to our knowledge this is the first attempt to dissect disease biomarkers specific to GA using wide-spectrum analysis. We plan to follow with other more specific prospective trials to answer the Reviewer’s concern.

That said, there is some interesting information in here regarding potential biomarkers. However, I have concerns about the analysis of the two chosen cohorts, especially with respect of the ‘validation’ data presented towards the end of the manuscript, which would need to be addressed before this manuscript could be suitable for publication in PLoS One.

We have revamped significantly the description of the analysis and we thank the Reviewer for pointing out that it was difficult to follow with the schematic alone. We have now included a more comprehensive description of the approach to the analysis in the statistics section and we refer to the new Fig. 1A schematic. Our statistical approach is explained in more detail in this revision (lines 293-323). We have also deposited all raw data, box plots and bar graphs into:

[dx.doi.org/10.17504/protocols.io.bhwuj7ew](https://dx.doi.org/10.17504/protocols.io.bhwuj7ew)

We hope that these modifications will satisfy the Reviewer. Given that the final analysis employed univariate ANCOVAs using age as a covariate and utilized transformations, we consider that the best representation of the data is the LSmeans or geometric means that are derived from the models with the corresponding 95% confidence limits.

We are providing you all box plots as requested in a separate file (*Boxplots for Reviewer…pdf*):

Cohort 1 (320 analytes): pages 1 - 640

Cohort 1 (sAPP): pages 641 – 645

Cohort 2 (40 analytes + sAPP + Aβ(1-42)): pages 646 – 687

sAPP/Aβ(1-40), sAPP/Abβ(1-42) and Aβ(1-40)/Aβ(1-42) ratios

**Major comments.**
The demographic details for the patient cohorts should be presented in a table.

We thank the Reviewer for this suggestion. We have included a demographic table for the 2 cohorts. This is shown as new Table 1.

The current presentation, of just age in Figure 4, is both limited and hard to discern detail. Do the authors have further clinical data? Given that their major finding is an increase in proteins commonly linked to Alzheimer’s disease, and that AMD and Alzheimer’s disease share many risk factors (including age, smoking status, BMI), it is important to present these fully, where possible, to rule out that any biomarkers are associated with these factors, rather than AMD per se.

We have provided a demographic table (Table 1) as requested by the Reviewer. We thank the Reviewer for their helpful comments. Our study subjects underwent a strict screening process for recruitment. Subjects recruited as part of the GSK clinical trial had undergone a detailed evaluation including a baseline brain MRI to rule out CNS diseases including Alzheimer’s disease (AD). Other subjects had a rigorous review of their medical records; any subjects suspected of cognitive changes, CNS disease or AD were excluded from the study. Since there are some commonalities between AMD and AD, we are not surprised by our data that identifies amyloid as an altered marker in GA. We have not included BMI as this information was not available in all subjects. We have also removed Fig. 4 and moved it to Supplementary Section as S1 Fig.

Detailed inclusion and exclusion criteria have been published: Rosenfeld, P.J., et al. (2018) A Phase 2 Study with an Anti-Amyloid Beta Monoclonal Antibody in Geographic Atrophy Secondary to Age Related Macular Degeneration. Ophthalmology Retina  <https://doi.org/10.1016/j.oret.2018.03.001>. This reference is now included in the manuscript and cited in the reference list.

Here is the detail of exclusion data:

Non-Ocular Exclusion Criteria included the following:

1.     Known risk or history of:

a.       Central nervous system (CNS) disorders:

i.         History and/or evidence (CT or MRI scan performed within the past 12 months or during screening period) of cerebral hemorrhage OR a known risk of cerebral hemorrhage, including uncontrolled hypertension, cerebrovascular malformation, coagulopathy, CNS vasculitis, degenerative or inflammatory/demyelinating CNS conditions or any other condition that the Investigator and/or the medical monitor considers as a relevant risk factor for cerebral hemorrhage

ii.      Transient ischemic attack (TIA)/cerebrovascular accident (CVA) in the last year, or other uncontrolled risk factors for stroke

b.       History of seizures (except febrile seizures in childhood) or recent unprovoked seizure

c.       Type 1 or uncontrolled type 2 diabetes mellitus, active cardiovascular disease (e.g., moderate-severe angina, unstable angina, myocardial infarction (MI) within the last 2 years, symptomatic congestive heart failure, clinically significant arrhythmia)

d.       Other severe/progressive medical or neurological conditions that limit the subject’s ability to complete or safely participate in the trial

e.       Current blood clotting or bleeding disorder or conditions that predispose to these (e.g. cancer)

f.        Current clinically significant systemic illness or significant infection within 30 days (e.g. chronic persistent or acute infection) that is likely to result in deterioration of the subject’s condition or affect the subject’s safety during the study

g.       Hepatobiliary abnormalities or disease (with the exception of Gilbert's syndrome or asymptomatic gallstones)

h.       Diagnosis of currently active, or, in remission but chronic relapsing, systemic autoimmune disease or condition (e.g. multiple sclerosis, lupus erythematosus, etc.) that the Investigator and/or the medical monitor considers as a relevant risk factor for concomitant administration of a therapeutic monoclonal antibody

i.        Any other condition that the Investigator and/or the medical monitor considers as a relevant risk factor or other clinically significant abnormality on physical, laboratory, ECG examination (e.g. atrial fibrillation) that would be detrimental to the subject

2.     Subject should have ability to comply with procedures for magnetic resonance imaging (MRI).  Subjects must be excluded if:

a.     Contraindications for MRI are known including cardiac pacemaker, CNS neurostimulator, aneurysm clips, artificial heart valves, other metallic foreign body, claustrophobia

b.     A Screening/Baseline brain MRI indicates any of the following conditions:

i.               evidence of other CNS conditions listed in exclusion criterion 8.a.i

ii.             Screening/Baseline MRI shows five or more micro-hemorrhage related events (ARIA-H), formerly characterized as micro-hemorrhage/microbleeds (longest diameter <5mm)

iii.           Screening/baseline MRI shows more than moderate white matter changes (Table 2 Scale for assessment of age-related white matter changes)

The table of ages provided via [protocols.io](http://protocols.io) doesn’t state whether each case was in cohort 1 or cohort 2, and has cases in groups labelled wetactive and wetinactive, which are not mentioned in the manuscript?

With apologies, it appears that the Reviewer may have mistakenly looked up the raw data from a previously published study in PLoS One (Lashkari K, Teague G, Chen H, Lin YQ, Kumar S, McLaughlin MM, et al. A monoclonal antibody targeting amyloid beta (Abeta) restores complement factor I bioactivity: Potential implications in age-related macular degeneration and Alzheimer's disease. PLoS One. 2018;13(5):e0195751. doi: 10.1371/journal.pone.0195751. PubMed PMID: 29782502).

We again apologize for this confusion. We have deposited all raw data, box-plots and bar graphs pertaining to this study into:

[dx.doi.org/10.17504/protocols.io.bhwuj7ew](https://dx.doi.org/10.17504/protocols.io.bhwuj7ew)

As we mentioned earlier, we consider that the best representation of the data is the LSmeans or geometric means that are derived from the models with the corresponding 95% confidence limits.

One would have thought a multiple-testing correction (Benjamini-Hochberg) should be applied to the data from cohort 1? If this is inappropriate, the authors should state why.

The initial approach to marker identification included a combination of Kruskal-Wallis tests combined with PCA and PLS. In order to include as many markers as possible in the subsequent analysis, the Kruskal-Wallis tests were not corrected for multiplicity. For the PCA/PLS we used the leave-one-out jack-knife statistical procedure, which we have now described in the materials and methods. In the subsequent analysis of the 102 identified markers, and following the MANOVAs, we included corrections for multiplicity in the analysis process. We used the Benjamini-Yekuteli correction in the process of analysis during identification of the set of markers that changed. We chose a P value of <0.1 after multiplicity correction to include as many markers as possible in the initial testing. We have added a reference pertaining to this approach and added it to the reference list.

For the final analysis of the markers, we used univariate ANCOVAs with multiple comparisons against the control group and corrected for multiplicity using the Dunnett’s correction. We have clarified our statistical approach in the statistical section in the Materials and Methods. We thank the Reviewer for pointing out that omission in the original manuscript.

Text on line 296 onwards is confusing. ‘Of 56 changing, 42 reached statistical significance’, in other words 14 of the 56 changing weren’t actually changing?

Definitions key here, I think. How the authors arrived at this list of 56 is hugely unclear, aside from a list of multiple statistical tests. How were these applied?

Once again, thank the Reviewer for pointing out this shortcoming. We have clarified and corrected this description and have modified Figures (Fig 1A) and the text to reflect these clarifications. In the original screening of 320 analytes in Cohort 1 and removal of quality failures, 273 analytes remained. These analytes were then subjected to Kruskal-Wallis, PCA, PLS rendering 56 markers that were identified in the MANOVA analysis after multiplicity correction. When these 56 markers were further analyzed in univariate ANCOVAs, 40 remained significant. At this point, we analyzed sAPP separately due to its connections in the amyloid pathway and have made the notation of “40 biomarkers + sAPP.” Since we discovered that Aβ(1-42) was surprisingly not statistically significant (which is in contrast to findings in Alzheimer’s disease in which it is the principal amyloid moiety), we included Aβ(1-42) in our analysis as well.

We then selected these 40 markers + sAPP + Aβ(1-42) for analysis in Cohort 2. Analysis of Cohort 2 resulted in 11 biomarkers that repeated. Again, Aβ(1-42) in GA was not statistically different from control. We presented Aβ(1-42) as “11 biomarkers + Aβ(1-42).” We refer you to Fig 1A.

Now we hope this clarifies the confusion.

A table spreadsheet with summary data (not raw data, as requested in the guidance – “For example, in addition to summary statistics, the data points behind means, medians and variance measures should be available.”) from all 273 measured proteins, is available in supplementary table 3, but it’s unclear how proteins were filtered and there is no statistical test result.

Summary statistics tables have now been included in the Supplemental Section per request of the Reviewer. These include:

S2 Table: Summary statistics for Cohort 1

S3 Table: Summary List and glossary of 40 biomarkers identified in Cohort 1 + sAPP + Aβ(1-42).

S2 Figures: Bar graphs of 40 biomarkers from Cohort 1 + sAPP + Aβ(1-42).

S4 Table: Summary statistics for Cohort 2

As mentioned previously, all primary data, box plots and bar graphs have been deposited as per Reviewer’s request and journal instructions. We are including here a series of boxplots for the Reviewer’s perusal. Nevertheless, we consider that the best representation of the data is the LSmeans or geometric means that are derived from the models with the corresponding 95% confidence limits.

The authors then add 2 more molecules, sAPP and the statistically insignificant Ab(1-42), bringing the number of statistically significant biomarkers to 43? I’m unclear how this works.

We thank the Reviewer for their comment. We have clarified our approaches in the manuscript to avoid confusion. In the original Myriad Luminex analysis of 94 subjects in Cohort 1, we analyzed the samples for presence of 320 analytes. In Cohort 1, after performing a series of statistical analyses as we mentioned in the previous section, we identified 56 biomarkers which had reached statistical significance in the MANOVA analysis with multiplicity correction. We then removed 2 markers that were significant for AREDS 3 vs. Control but not significant for GA vs. Control and removed another 14 markers had more than 30% of the data below the limit of quantitation.

Further analysis with univariate ANCOVAs yielded 40 biomarkers with statistically significant changes. As mentioned earlier, we discovered that Aβ(1-40) but not Aβ(1-42) was significantly altered in GA vs. AREDS 3 vs control. We then wanted to know whether the upstream of Aβ(1-40), amyloid precursor protein (APP) was also affected in GA. We analyzed sAPP using a separate ELISA.

This methodology allowed us to determine that Aβ(1-40) was not independently elevated in GA, rather APP, the upstream molecule was also elevated in GA. Furthermore, since Aβ (1-42) was not significantly affected, there was a suggestion for preferential splicing of APP to Aβ(1-40) rather to Aβ (1-42).

In summary, in Cohort 1 we identified a total of 40 factors from the original multiplexing and sAPP in the separate ELISA. We have noted this as “40 factors + sAPP” in Fig. 1A, since sAPP was analyzed separately. Since Aβ(1-42) was conspicuously not significant,

We purposefully included Aβ(1-42) (as the 42^rd^ marker) to make a point that this particular amyloid was not altered in GA. In Fig 1A, we now show this as “40 factors + sAPP and Aβ (1-42).” We hope that this further clarifies the point.

The bioinformatics network analysis is useful, although somewhat cherry-picked. For example, there seem to be more proteins directly linked to CD40 than to AP. The Ingenuity software produces significance scores as to how over-represented these pathways are in the network – these scores should be reported to support the authors’ conclusions from this analysis.

The network analysis performed in Ingenuity used an algorithm to connect the maximum number of proteins from the starting list together, based on functional relationships from the Ingenuity database. The network shown was the resulting network identified. *It was not cherry picked*. Since the approach used was not a pathway enrichment approach, no significance scores were obtained. In the text we accurately describe the network as having several hub nodes, one of which is indeed CD40; “This network includes several strongly connected gene hubs, including MMP9, CD40, IL1B, SPP1 and APP.” We then show the number of proteins connected to APP (Fig. 2). It is true that several of the other hub nodes are similarly or more highly connected than APP.

These 43 were re-evaluated by a different ELISA in a second cohort (although the heading on line 334 states 42), but the manuscript (line 303) then only discusses 12 of them, 11 of which were significant. Does this mean that 30/42 (71%) failed to replicate? Again, far more detail is required, as above, to allow myself, and a reader, to evaluate these data fully.

We apologize for this confusion and have made changes to the manuscript to better explain this. In Cohort 1, we identified a total of 40 markers, identified from the 320- analyte Myriad Luminex assay and 1 additional marker, sAPP was identified using a separate ELISA (as the 41^st^). As mentioned in the previous pages, this allowed us to observe that both sAPP and Aβ(1-40) but not Aβ(1-42) were altered in GA. Since Aβ(1-42) is the key offending amyloid moiety in Alzheimer’s disease, we made a point of including it in our further analysis as a point of comparison with Aβ(1-40).

Therefore, we included Aβ(1-42) as the 42nd marker to make the point that it was not significantly altered unlike its sister splice variant, Aβ(1-40). This is reflected in the manuscript and in Fig. 1A, where you will see the notation “40 biomarkers + sAPP and Aβ(1-42)”.

The Reviewer is correct that only 11 factors (including sAPP) replicated, probably due to different methodologies, sensitivities of detection plates and the different participants in the analysis. These data highlight the need for replication and use of various methologies to allow for identification of markers that are changing in a robust manner. Interestingly, sAPP and Aβ(1-40), but not Aβ(1-42) were again significantly elevated in Cohort 2. Aβ(1-42) replicated as well in the sense that it did not change in either cohort and could be considered at the 12^th^ marker that replicated in Cohort 2.

Box plots, such as described here (<https://blog.bioturing.com/2018/05/22/how-to-compare-box-plots/>), should be used to illustrate these data, including individual data points as shown for each patient to allow the reader to interpret these data more fully. Units should also be adjusted such that they’re easier to read (e.g. in Supp table 4, Pon1 could be 8.77ug/mL, instead of 8,771,921 pg/mL).

We have made the unit corrections as requested. This can now be seen in S2 and S4 Tables. We have included the box plots for all analytes for Cohort 1 and Cohort 2 for the Reviewer. However, we feel that they are too extensive to be included in the actual manuscript. We have enclosed this data in this response file and have also deposited them into:

[dx.doi.org/10.17504/protocols.io.bhwuj7ew](https://dx.doi.org/10.17504/protocols.io.bhwuj7ew)

As mentioned earlier we consider that the best representation of the data is the LSmeans or geometric means that are derived from the models with the corresponding 95% confidence limits.

The authors go on to study amyloid beta peptide levels in samples in both cohorts, claiming an increase in the 1-40 form (but not the 1-42 form) in GA. These data are presented in Figure 6. The authors do not address the apparently huge disparity in levels in the control samples between the 2 cohorts (for 1-40 they are ~280pg/ml in cohort 1, and ~50pg/mL in cohort 2). This is critical and impact on the reliability of the data. In my view, y-axes should equalised to make this disparity more apparent.
A similar observation can be made for the non-amyloid biomarkers presented in Figure 7 and 8. What is so different between the cohorts such that, e.g. AXL RTK is 10x lower in controls from cohort 2 than in cohort 1, while other proteins e.g. pentraxin-3 and omentin are (broadly) similar? This is a critical question for this study and the lack of reproducibility between the 2 cohorts, both in terms of identified biomarkers and raw concentrations measured, currently places major doubt on the validity of these findings.

We agree with the Reviewer that there are disparities in analyte concentrations between Cohorts 1 and 2. We noticed these disparities as well. To this end, carefully examined these findings and rechecked all methodologies and even repeated some ELISAs. Our best understanding is that there is attrition of signal even with short-term storage (at -86° C or liquid N2 temperatures) with some of these factors [See: *Enroth et al. Effects of Long-Term Storage Time and Original Sampling Month on Biobank Plasma Protein Concentrations.* [*EBioMedicine*](https://www.ncbi.nlm.nih.gov/pmc/articles/PMC5078583/)*. 2016; 12: 309–314. PMCID: PMC5078583 PMID:* [*27596149*](https://www.ncbi.nlm.nih.gov/pubmed/27596149)]. In addition, the methodologies used, as mentioned earlier, differ by design and sensitivities in the two Cohorts. These methodologies are not uniform and vary, e.g. Luminex vs. ELISA vs. multiplexing using various plates from different manufacturers. We established that our methods were sound. Nevertheless, despite signal differences, we clearly show that 11 factors replicated in Cohort 2, including sAPP and Aβ(1-40) (with Aβ(1-42) as the 12^th^ factor that replicated as non-significant). Therefore, it is plausible that different standards used in the two cohorts may account for these discrepancies. The same issues also likely to contribute to the high rate of markers that did not repeat in the second cohort. In the case of amyloids, oligomerization can occur with storage, which can result in artifactually diminished levels as time of storage increases. These ELISAs do not recognize oligomers and polymers of amyloid [*See Stine et al. Preparing synthetic Aβ in different aggregation states. Methods. Mol Biol. 2011; 670: 13-32*].

Although initially we constructed the graphics in the same scale for the Y axis, it became obvious that equalizing the “Y” axis would diminish the resolution of some of the bars in these graphs. Therefore, we opted for the current representation of the figures. To account for this and take into consideration the reviewer recommendation, we have placed a note to the reader at the end of each figure legend, alerting them of the Y axis has a different scale: “Note: The Y axes in these graphs have different scales.”

We hope that this satisfied the Reviewer’s concerns.

**Minor comments:**
The abstract should state the methodology used for the initial cohort 1 study i.e. Luminex.

We thank the Reviewer for this suggestion. We have made the correction and have included a description of the procedure.

There are numerous typographical errors which need to be corrected.

We apologize for the typographical errors and have made the corrections. We have also detected an error in subject allocation and have made the corrections. This was purely typographical and did not affect any of our statistical analyses.

There are probably too many figures. Figure 1 is redundant – a simple reference will do as this figure is widely used elsewhere.

We thank the Reviewer for this suggestion. We had included Fig. 1 for the general readers of PLoS One. We have now removed this figure.

Figures 2 and 3 could be merged into a single figure.

We thank the Reviewer for this suggestion. We have merged Figs. 2 and 3 as the new Fig. 1.

Figure 4 should be a table (see above) etc..

We thank the Reviewer for this suggestion. We have removed Fig. 4 and replaced it with a demographic table. We have moved Fig. 4 to the Supplemental section as the S1 Fig.

Line 305 – the location of where the informatics evaluation took place is irrelevant.

We have removed the location of informatics evaluation.

The y axes on figure 4 should be the same, to allow comparison.

Per Reviewer’s suggestion, we have removed Fig. 4 and replaced it with a demographic table.

Are the p-values on the arrows on Figs 6-8 the significance of the trend, as suggested in the legend? If so, what test was used?

The p values for the trend come from the univariate ANCOVA analysis. In these models, the fixed effects are coded as an ordinal factor. This allows for the determination of a potential linear trend for the fixed effect. This P value for the linear trend obtained in the ANCOVA is provided in the figures. This explanation has now been added to the description of the analysis in the methods section.

**Reviewer #2:**

Lashkari et al. used a two-stage approach to identify proteins in plasma that were significantly differentially present in patients with early or late AMD compared to healthy controls They identify a set of 11 proteins which are either elevated or reduced in patients. Their approach identified that sAPP and Aß(1-40), but not Aß(1-42) to be elevated in patients with GA, pointing towards and involvement of amyloid pathways in GA pathogenesis. Additional proteins were identified to be dysregulated, particularly those involved in ECM remodelling/homeostasis. I have some comments:

Introduction:

Line 104: These include variants of complement factor (CF) H, CFI, CFB and C3 [13-17]. There are also recent reports on a role of C9 and C4A/B in AMD risk and those studies should be cited here.

We thank the Reviewer for their comment. We have included these variants in the same discussion and have updated the references.

Methods:

Line 168: Subjects were staged as non-AMD control (AREDS stage 0), intermediate stage dry AMD - AREDS stage 3, and advanced stage dry AMD - GA (AREDS 4/5 [31]).
The cited source has two grading systems, one 9-step and one 6-step grading. While I am no expert on grading, if I remember correctly late stage GA was defined as AREDS grade 9 and 10 (peripheral and central GA, respectively). Did the authors use earlier stages in their assessment which are lacking geographic atrophy? Their fundus images suggest otherwise, with pronounced central GA in Figure 2D and F?

We apologize for this confusion. We have used the 6-step grading system and have provided a more accurate reference. GA is graded as AREDS stage 4. We have made the appropriate corrections and added the correct reference.

Line 221: Evaluation of biomarkers by ELISA and Multiplexing in Cohort 2
The full list of analytes which were analysed in cohort 2 does not need to be written as such but should rather be in a Table in the supplements and then just cited here.

We thank the reviewer and have made the changes. These can now be found in S1 Table.

Line 257: CFI Bioactivity Assay Evaluating Oligomerized Aβ(1-40) and Aβ(1-42)
A little bit more detail on the methodology would be good here so the reader does not have to read another manuscript to understand the approach.

We thank the reviewer for noticing this error. We have removed this paragraph from the manuscript as to avoid any confusion.


Results:

I would like to have a little bite more information on the patient characteristics. A Table summarizing age and other AMD risk factors for all cohorts, stratified by disease status would be good in the beginning.

We have removed Figure 4 and replaced it with a Demographic Table (Table 1 in the manuscript).

Table 1:
a) How were the P-values corrected for multiple testing? The table legend mentions multiplicity but no explanation on how that was done.

We used the Benjamini-Yekuteli correction for multiplicity in the initial MANOVA. In addition, after univariate ANCOVAs, multiple comparisons against the control were corrected for multiplicity using the Dunnett’s correction. This has now been added to the description in the statistics section in the Materials & Methods.

b) How are the effect sizes (i.e. higher or lower levels in patients compared to controls), standard errors or 95% confidence intervals? The table can be quite large for 56 markers but this should be shown. The legend can be shorter because the proteins are mentioned in Table 2, which can also be moved to the supplement.

Data are presented as the estimated means or geometric means and the errors represent the 95% confidence limits, since for some of the biomarkers we used the Box-Cox family of transformations. This has now been clarified in the legends to the figures and described in more details in the statistics section in the Materials and Methods. We have included the following tables:

S2 Table: Summary Statistics for Cohort 1. We have highlighted the 56 statistically significant analytes in YELLOW. We have highlighted Aβ(1-42) in BLUE. We have added sAPP (which was analyzed in a separate ELISA) to the bottom of the table and highlighted it in GREEN.

S3 Table: Summary List and glossary of 40 biomarkers identified in Cohort 1 + sAPP + Aβ(1-42).

S2 Figures: Bar graphs of 40 biomarkers from Cohort 1 + sAPP + Aβ(1-42).

S4 Table: Summary Statistics for Cohort 2

Table 3: see comments for Table 2.
Please see our previous response.

Line 289ff: Why did the authors not perform logistic or multinomial regression for their analyses adjusted for age and/or batches/plates for their initial screen in 273 markers? The current approach has two steps and thus more statistical tests, which should be appropriately accounted for.

We thank the Reviewer for this question. We used univariate ANCOVA in the final analysis since the dependent variable is continuous for all the markers. We accounted for multiplicity in the initial marker identification as mentioned earlier (Benjamini-Yekuteli). Thus, these multiple tests are accounted for in the analysis. This has now been clarified in the legends to the figures and described in more details in the statistics section of the Materials & Methods.

Line 301ff: The authors need to be clearer about their approach and their statistics. 43 Markers were investigated in cohort 2, but the authors then only talk about 12 markers of which 11 were stat. significant?

Once again, thank the Reviewer for pointing out this issue. We have clarified and corrected this description and have modified Figures (Fig 1A) and the text to reflect these clarifications. In the original screening of 320 analytes in Cohort 1 and removal of quality failures, 273 analytes remained. These analytes were then subjected to Kruskal-Wallis, PCA, PLS rendering 56 markers that were identified in the MANOVA analysis after multiplicity correction. When these 56 markers were further analyzed in univariate ANCOVAs, 40 remained significant. At this point, we analyzed sAPP separately due to its connections in the amyloid pathway and have made the notation of “40 biomarkers + sAPP.” Since we discovered that Aβ(1-42) was surprisingly not statistically significant (which is in contrast to findings in Alzheimer’s disease in which it is the principal amyloid moiety), we included Aβ(1-42) in our analysis as well.

In Cohort 2, these we analyzed “40 markers + sAPP and Aβ(1-42)” using different methodologies including ELISA and multiplexing (Bio-Rad or R&D plates).

The Reviewer is correct that only 11 factors replicated. However, sAPP and Aβ(1-40) results replicated in both cohorts. Interestingly, Aβ(1-42) was again not statistically significant and can be considered as the 12^th^ marker that replicated.

The reason for the low rate of replication is probably due to different methodologies, different sensitivities of detection plate, effect of storage and the different participants in the analysis. These data highlight the need for replication and use of various methodologies to allow for identification of markers that are changing in a robust manner.

Line 308-319: this information can be put into a table, as suggested above and placed before the previous paragraph, since the description of the patient cohort should come first. Otherwise the results are cut in half by this paragraph and the read flow is disrupted.

We thank the Reviewer for this suggestion. We have made the appropriate correction.

Line 334ff: See my comments above for lines 301ff

Please see our answer to Line 301ff

Figure 6D: Why was the inverse of the data used in this analysis?

We are not sure exactly what the Reviewer is alluding to in Fig 6. If the Reviewer is referencing the Figs. in the bottom insert (now Fig. 3), we are referencing the biomarker literature for Alzheimer’s disease (AD), in which the ratios of amyloid moieties are altered in the CSF of patients with AD. We hereby show that a similar observation for amyloid ratios can be made in GA.

On the other hand, if the Reviewer is referencing our statistical methods, as described in the statistical analysis (Materials and Methods) a series of Box-Cox transformations were used to analyze the data. A few markers were analyzed in the inverse of the data as the inverse performed better statistically than the raw data.

Discussion:

Line 418ff: This paragraph should rather be in the introduction and not necessarily in the discussion. It does not fit here.

We thank the Reviewer for their helpful comments and have made the appropriate changes.

Line 426 to 434: this should also be in the introduction and only briefly summarized here I it is relevant for the results in this study

We thank the Reviewer for their helpful comments and have made the appropriate changes.

Line 468: I don’t know if CRISPR/Cas9 was available in 1977.

We apologize for the typographic error in the date. It should have read 2017. We have made the correction.

Line 478: While I appreciate the in-depth discussion of all the markers not involved in amyloid pathways that were identified as significant, this section should be shortened and can be expressed more generally. Clearly the results suggest that remodelling and homeostasis of the ECM seem to be a major theme as well as immune activation and the proteins should be put into this context.

We thank the Reviewer for their helpful comments. We have made the appropriate changes and added a discussion for remodeling and homeostasis, with new references.

6. PLOS authors have the option to publish the peer review history of their article ([what does this mean?](https://nam12.safelinks.protection.outlook.com/?url=https%3A%2F%2Fjournals.plos.org%2Fplosone%2Fs%2Feditorial-and-peer-review-process%23loc-peer-review-history&data=02%7C01%7Cklashkari%40umassd.edu%7C23883204632a4789a14b08d7e5f9d060%7C328d6c0d0f2f4b7693109762ba1c3e2d%7C0%7C1%7C637230733427245789&sdata=YJFufFj%2FXkFm7JCjjjRqI%2BgeDR0UKt5X%2B00cW3wtyPI%3D&reserved=0)). If published, this will include your full peer review and any attached files.


If you choose “no”, your identity will remain anonymous but your review may still be made public.


**Do you want your identity to be public for this peer review?** For information about this choice, including consent withdrawal, please see our [Privacy Policy](https://nam12.safelinks.protection.outlook.com/?url=https%3A%2F%2Fwww.plos.org%2Fprivacy-policy&data=02%7C01%7Cklashkari%40umassd.edu%7C23883204632a4789a14b08d7e5f9d060%7C328d6c0d0f2f4b7693109762ba1c3e2d%7C0%7C0%7C637230733427245789&sdata=L9EzZSwljU6AiFKNmrAgdbXpQ37xxhpFdfDTeXaXQkg%3D&reserved=0).

Reviewer #1: No

Reviewer #2: No


[NOTE: If reviewer comments were submitted as an attachment file, they will be attached to this email and accessible via the submission site. Please log into your account, locate the manuscript record, and check for the action link "View Attachments". If this link does not appear, there are no attachment files to be viewed.]


While revising your submission, please upload your figure files to the Preflight Analysis and Conversion Engine (PACE) digital diagnostic tool, [https://pacev2.apexcovantage.com/](https://nam12.safelinks.protection.outlook.com/?url=https%3A%2F%2Fpacev2.apexcovantage.com%2F&data=02%7C01%7Cklashkari%40umassd.edu%7C23883204632a4789a14b08d7e5f9d060%7C328d6c0d0f2f4b7693109762ba1c3e2d%7C0%7C1%7C637230733427255748&sdata=JiUftGVllqJkk32o%2FR8CUcYQoP72VQEfD48ZJCYlDzc%3D&reserved=0). PACE helps ensure that figures meet PLOS requirements. To use PACE, you must first register as a user. Registration is free. Then, login and navigate to the UPLOAD tab, where you will find detailed instructions on how to use the tool. If you encounter any issues or have any questions when using PACE, please email us at [figures@plos.org](mailto:figures@plos.org). Please note that Supporting Information files do not need this step.
